# Supplementary material for: Enhanced sensitivity of TAPscan v4 enables comprehensive analysis of streptophyte transcription factor evolution
Source: Plant J. 2024 Dec 12;121(1):e17184. doi: 10.1111/tpj.17184 (PMC11712027; doi:10.1111/tpj.17184)
Supplement: Supplementary file 7 — File S1. TAPscan profile HMMs. [file TPJ-121-0-s003.pdf]

```
HMMER3/f [3.3 | Nov 2019]
NAME  Homeobox_KN
ACC   PF05920.15
DESC  Homeobox KN domain
LENG  40
ALPH  amino
RF    no
MM    no
CONS  yes
CS    no
MAP   yes
DATE  Thu Jun  1 12:21:36 2023
NSEQ  59
EFFN  4.973083
CKSUM 2977741935
GA    24 24;
TC    24 24;
NC    23.9 23.9;
BM    hmmbuild HMM.ann SEED.ann
SM    hmmsearch -E 1000 -Z 75585367 --cpu 4 HMM pfamseq
STATS LOCAL MSV       -7.7159  0.71948
STATS LOCAL VITERBI   -8.0148  0.71948
STATS LOCAL FORWARD   -4.3148  0.71948
HMM          A        C        D        E        F        G        H
I        K        L        M        N        P        Q        R        S
T        V        W        Y
            m->m     m->i     m->d     i->m     i->i     d->m     d->d
  COMPO   2.70660  4.73352  3.13316  2.77242  3.22918  3.60790  3.59261
3.08660  2.67742  2.52877  3.97408  2.65620  2.85923  2.88228  2.42319
2.88711  2.86169  3.17775  3.06871  3.27191
          2.68618  4.42225  2.77519  2.73123  3.46354  2.40513  3.72494
3.29354  2.67741  2.69355  4.24690  2.90347  2.73739  3.18146  2.89801
2.37887  2.77519  2.98518  4.58477  3.61503
          0.03632  5.34200  3.47763  0.61958  0.77255  0.00000        *
      1   6.14180  6.89418  6.41490  6.45751  5.15987  5.58747  6.34124
6.57881  6.41652  5.68335  7.08014  6.55476  6.19931  6.69732  6.26107
6.38269  6.51942  6.43356  0.04308  5.13113      1 W - - -
          2.68618  4.42225  2.77519  2.73123  3.46354  2.40513  3.72494
3.29354  2.67741  2.69355  4.24690  2.90347  2.73739  3.18146  2.89801
2.37887  2.77519  2.98518  4.58477  3.61503
          0.00735  5.31303  6.03538  0.61958  0.77255  0.42403  1.06248
      2   4.25160  5.49744  6.17583  5.59319  1.42691  5.52203  5.62017
2.71482  5.41972  0.73888  3.19060  5.65645  5.63460  5.29431  5.33838
4.88365  4.45451  3.39118  2.84618  4.51668      2 l - - -
          2.68618  4.42225  2.77519  2.73123  3.46354  2.40513  3.72494
3.29354  2.67741  2.69355  4.24690  2.90347  2.73739  3.18146  2.89801
2.37887  2.77519  2.98518  4.58477  3.61503
          0.01574  5.34200  4.52566  0.61958  0.77255  0.48576  0.95510
      3   2.84311  5.07564  3.40298  2.64654  3.03917  3.91902  3.20986
3.17273  2.47394  2.11098  4.15953  2.64071  4.30595  3.14477  2.71449
2.89262  3.27659  2.85656  4.51972  2.25660      3 l - - -
          2.68618  4.42225  2.77519  2.73123  3.46354  2.40513  3.72494
3.29354  2.67741  2.69355  4.24690  2.90347  2.73739  3.18146  2.89801
2.37887  2.77519  2.98518  4.58477  3.61503
          0.00720  5.33346  6.05581  0.61958  0.77255  0.51896  0.90421
      4   2.38757  5.54101  2.07742  1.75446  4.88686  3.80131  3.74270
4.37340  2.46197  3.42175  4.58151  2.42644  4.19507  2.27120  2.68029
2.89047  3.13260  3.71610  5.97316  4.56168      4 e - - -
```

```
          2.68618  4.42225  2.77519  2.73123  3.46354  2.40513  3.72494
3.29354  2.67741  2.69355  4.24690  2.90347  2.73739  3.18146  2.89801
2.37887  2.77519  2.98518  4.58477  3.61503
          0.00720  5.33346  6.05581  0.61958  0.77255  0.46555  0.98830
      5   3.68073  5.89824  3.39769  3.28905  5.21293  4.12736  1.02070
5.05897  3.53485  4.56564  5.43468  1.08817  4.75997  3.92359  3.92110
3.67748  3.19965  4.60318  6.49406  5.03087       5 h - - -
          2.68618  4.42225  2.77519  2.73123  3.46354  2.40513  3.72494
3.29354  2.67741  2.69355  4.24690  2.90347  2.73739  3.18146  2.89801
2.37887  2.77519  2.98518  4.58477  3.61503
          0.00714  5.34200  6.06435  0.61958  0.77255  0.48576  0.95510
      6   3.08897  3.81241  4.83186  3.89729  2.69656  4.23228  4.55351
2.58762  4.06156  1.49904  3.24434  3.89265  4.59829  2.91551  2.20779
3.30062  3.13206  2.58059  3.21679  2.71868       6 l - - -
          2.68618  4.42225  2.77519  2.73123  3.46354  2.40513  3.72494
3.29354  2.67741  2.69355  4.24690  2.90347  2.73739  3.18146  2.89801
2.37887  2.77519  2.98518  4.58477  3.61503
          0.00714  5.34200  6.06435  0.61958  0.77255  0.48576  0.95510
      7   2.77739  5.53869  2.28132  2.36799  4.23830  3.21797  2.45298
4.36713  2.23559  3.35609  3.69062  2.52911  4.19943  2.81099  2.90038
2.45314  2.77364  3.66346  5.97229  3.45559       7 k - - -
          2.68618  4.42225  2.77519  2.73123  3.46354  2.40513  3.72494
3.29354  2.67741  2.69355  4.24690  2.90347  2.73739  3.18146  2.89801
2.37887  2.77519  2.98518  4.58477  3.61503
          0.00714  5.34200  6.06435  0.61958  0.77255  0.48576  0.95510
      8   2.93839  4.14957  3.09094  2.92747  3.69927  3.89232  2.30604
3.81892  2.68524  3.43978  4.26469  1.36233  4.28126  3.26448  3.37399
2.95069  3.27896  3.51210  3.33729  2.92787       8 n - - -
          2.68619  4.42226  2.77520  2.73124  3.46355  2.40514  3.72495
3.29355  2.67734  2.69356  4.24691  2.90347  2.73740  3.18147  2.89802
2.37888  2.77520  2.98519  4.58429  3.61504
          0.01574  4.32077  6.06435  0.63785  0.75168  0.48576  0.95510
      9   2.46068  5.37982  5.08781  5.09838  6.01510  4.13091  6.03382
5.52933  5.25889  5.21971  6.07166  4.87667  0.23572  5.45349  5.35189
3.69795  4.06965  4.73146  7.25116  6.25755      11 P - - -
          2.68618  4.42225  2.77519  2.73123  3.46354  2.40513  3.72494
3.29354  2.67741  2.69355  4.24690  2.90347  2.73739  3.18146  2.89801
2.37887  2.77519  2.98518  4.58477  3.61503
          0.00714  5.34200  6.06435  0.61958  0.77255  0.48576  0.95510
     10   5.57711  6.38344  6.24115  6.28288  2.21064  5.89675  4.44874
5.09164  6.04766  4.14832  5.58225  5.39867  6.14672  5.60452  5.77864
5.34255  5.76397  5.07523  4.51235  0.22315      12 Y - - -
          2.68618  4.42225  2.77519  2.73123  3.46354  2.40513  3.72494
3.29354  2.67741  2.69355  4.24690  2.90347  2.73739  3.18146  2.89801
2.37887  2.77519  2.98518  4.58477  3.61503
          0.00714  5.34200  6.06435  0.61958  0.77255  0.48576  0.95510
     11   5.40084  6.69673  6.06063  6.12054  6.80091  5.32150  6.88271
6.93855  6.31745  6.24614  7.44297  6.29370  0.03783  6.62726  6.24864
5.64379  5.91789  6.40337  7.41456  6.98271      13 P - - -
          2.68618  4.42225  2.77519  2.73123  3.46354  2.40513  3.72494
3.29354  2.67741  2.69355  4.24690  2.90347  2.73739  3.18146  2.89801
2.37887  2.77519  2.98518  4.58477  3.61503
          0.00714  5.34200  6.06435  0.61958  0.77255  0.48576  0.95510
     12   2.81433  5.58395  2.45538  2.79400  4.94782  3.84889  4.09834
4.42674  2.47906  3.92113  4.67368  2.90753  4.27791  3.20641  3.35806
1.36288  1.66358  3.99629  6.06547  4.65839      14 s - - -
```

```
        2.68618  4.42225  2.77519  2.73123  3.46354  2.40513  3.72494
3.29354  2.67741  2.69355  4.24690  2.90347  2.73739  3.18146  2.89801
2.37887  2.77519  2.98518  4.58477  3.61503
        0.00714  5.34200  6.06435  0.61958  0.77255  0.48576  0.95510
     13  2.32444  5.56371  2.26090  1.55806  4.90874  3.81292  4.00697
4.39583  2.06236  3.86887  4.60514  3.07205  2.56945  3.10418  2.77065
2.72327  3.28138  3.95448  5.99570  4.58280     15 e - - -
        2.68618  4.42225  2.77519  2.73123  3.46354  2.40513  3.72494
3.29354  2.67741  2.69355  4.24690  2.90347  2.73739  3.18146  2.89801
2.37887  2.77519  2.98518  4.58477  3.61503
        0.00714  5.34200  6.06435  0.61958  0.77255  0.48576  0.95510
     14  2.36371  5.53271  2.27614  1.70044  4.25184  3.44569  3.60868
3.77246  2.73107  3.83622  4.57447  3.04280  4.20061  1.87183  3.08437
2.89720  2.90006  3.40815  5.96789  4.17973     16 e - - -
        2.68618  4.42225  2.77519  2.73123  3.46354  2.40513  3.72494
3.29354  2.67741  2.69355  4.24690  2.90347  2.73739  3.18146  2.89801
2.37887  2.77519  2.98518  4.58477  3.61503
        0.00714  5.34200  6.06435  0.61958  0.77255  0.48576  0.95510
     15  3.03361  5.52525  2.11666  1.40102  4.86230  3.29857  3.99797
3.68357  2.64036  3.82708  3.36276  3.28012  4.20325  2.13077  3.09531
2.99949  2.96835  3.37138  5.96269  4.55653     17 e - - -
        2.68618  4.42225  2.77519  2.73123  3.46354  2.40513  3.72494
3.29354  2.67741  2.69355  4.24690  2.90347  2.73739  3.18146  2.89801
2.37887  2.77519  2.98518  4.58477  3.61503
        0.00714  5.34200  6.06435  0.61958  0.77255  0.48576  0.95510
     16  3.67656  5.78795  4.21410  3.48721  5.18402  4.37831  4.32761
4.50064  0.56988  2.94324  4.86691  3.90257  4.72663  3.46317  2.39373
3.69066  3.34880  3.30637  6.11999  4.93099     18 K - - -
        2.68618  4.42250  2.77544  2.73122  3.46379  2.40519  3.72519
3.29334  2.67741  2.69331  4.24714  2.90341  2.73739  3.18131  2.89795
2.37894  2.77518  2.98477  4.58502  3.61528
        0.03632  3.40092  6.06435  2.15749  0.12286  0.48576  0.95510
     17  2.05386  5.50635  2.45798  1.99682  4.24344  3.81228  4.00098
3.14491  2.60631  3.11848  4.04498  2.79238  4.20538  2.50425  2.49617
2.82379  2.85765  3.15504  5.94828  4.54672     34 e - - -
        2.68618  4.42225  2.77519  2.73123  3.46354  2.40513  3.72494
3.29354  2.67741  2.69355  4.24690  2.90347  2.73739  3.18146  2.89801
2.37887  2.77519  2.98518  4.58477  3.61503
        0.00714  5.34200  6.06435  0.61958  0.77255  0.48576  0.95510
     18  2.73847  5.48616  3.15074  1.73833  3.80780  3.81632  4.00599
3.79000  2.58265  3.41058  3.19725  3.29343  3.53407  2.21281  2.33487
2.39720  2.82252  3.51686  5.93319  3.78199     35 e - - -
        2.68618  4.42225  2.77519  2.73123  3.46354  2.40513  3.72494
3.29354  2.67741  2.69355  4.24690  2.90347  2.73739  3.18146  2.89801
2.37887  2.77519  2.98518  4.58477  3.61503
        0.00714  5.34200  6.06435  0.61958  0.77255  0.48576  0.95510
     19  4.75815  5.89040  6.74232  6.14776  2.64856  6.23377  6.43400
1.93881  6.03361  0.44040  2.77657  6.42990  6.05267  5.68178  5.86465
5.67430  4.93332  3.47641  6.18164  5.32281     36 L - - -
        2.68618  4.42225  2.77519  2.73123  3.46354  2.40513  3.72494
3.29354  2.67741  2.69355  4.24690  2.90347  2.73739  3.18146  2.89801
2.37887  2.77519  2.98518  4.58477  3.61503
        0.00714  5.34200  6.06435  0.61958  0.77255  0.48576  0.95510
     20  1.31659  3.32635  4.61006  4.02013  3.64770  4.19764  4.50501
2.51749  3.01870  2.06217  2.94803  4.20288  4.56643  3.69794  4.05039
2.51523  3.04727  2.44930  5.15929  3.96999     37 a - - -
```

```
         2.68618  4.42225  2.77519  2.73123  3.46354  2.40513  3.72494
3.29354  2.67741  2.69355  4.24690  2.90347  2.73739  3.18146  2.89801
2.37887  2.77519  2.98518  4.58477  3.61503
         0.00714  5.34200  6.06435  0.61958  0.77255  0.48576  0.95510
     21  2.35846  5.54277  2.62252  2.05925  4.88808  3.56476  3.41254
4.01647  1.89114  3.84815  4.08599  3.10471  4.19874  2.10837  2.54513
2.87206  2.95685  3.11819  5.97530  4.56422     38 k - - -
         2.68618  4.42225  2.77519  2.73123  3.46354  2.40513  3.72494
3.29354  2.67741  2.69355  4.24690  2.90347  2.73739  3.18146  2.89801
2.37887  2.77519  2.98518  4.58477  3.61503
         0.00714  5.34200  6.06435  0.61958  0.77255  0.48576  0.95510
     22  2.33886  5.52591  3.28805  1.88384  4.86389  3.59989  3.53365
3.43912  2.09855  3.22816  3.80973  3.27914  4.20217  2.05559  2.37512
2.65137  2.95200  3.91274  5.96279  4.55633     39 e - - -
         2.68618  4.42225  2.77519  2.73123  3.46354  2.40513  3.72494
3.29354  2.67741  2.69355  4.24690  2.90347  2.73739  3.18146  2.89801
2.37887  2.77519  2.98518  4.58477  3.61503
         0.00714  5.34200  6.06435  0.61958  0.77255  0.48576  0.95510
     23  2.26776  2.59782  4.61070  4.11262  4.38950  3.44679  4.88023
3.75828  4.03778  3.48399  4.36967  4.24743  4.59168  4.29057  4.29566
2.50758  0.77592  3.44952  5.84671  3.46087     40 t - - -
         2.68618  4.42225  2.77519  2.73123  3.46354  2.40513  3.72494
3.29354  2.67741  2.69355  4.24690  2.90347  2.73739  3.18146  2.89801
2.37887  2.77519  2.98518  4.58477  3.61503
         0.00714  5.34200  6.06435  0.61958  0.77255  0.48576  0.95510
     24  3.05568  5.54020  2.91580  2.74696  4.87446  1.23525  3.40780
4.35254  2.54056  3.32139  4.58501  2.22499  4.22074  2.96465  2.96820
2.91061  3.02779  3.92529  5.97817  4.57391     41 g - - -
         2.68618  4.42225  2.77519  2.73123  3.46354  2.40513  3.72494
3.29354  2.67741  2.69355  4.24690  2.90347  2.73739  3.18146  2.89801
2.37887  2.77519  2.98518  4.58477  3.61503
         0.00714  5.34200  6.06435  0.61958  0.77255  0.48576  0.95510
     25  2.65327  4.48510  4.99786  4.38723  2.71733  4.26346  4.59364
2.33246  4.18694  1.01579  2.85841  4.40383  4.62680  3.60004  4.21852
2.96020  2.93864  2.49346  5.10582  3.92916     42 l - - -
         2.68618  4.42225  2.77519  2.73123  3.46354  2.40513  3.72494
3.29354  2.67741  2.69355  4.24690  2.90347  2.73739  3.18146  2.89801
2.37887  2.77519  2.98518  4.58477  3.61503
         0.00714  5.34200  6.06435  0.61958  0.77255  0.48576  0.95510
     26  3.03231  4.12161  2.56956  2.73220  4.86261  3.59924  3.61492
4.34331  2.50252  3.82711  3.95961  2.78331  3.71150  2.75661  2.95957
1.62054  1.80958  3.91164  5.96221  4.55584     43 s - - -
         2.68618  4.42225  2.77519  2.73123  3.46354  2.40513  3.72494
3.29354  2.67741  2.69355  4.24690  2.90347  2.73739  3.18146  2.89801
2.37887  2.77519  2.98518  4.58477  3.61503
         0.00714  5.34200  6.06435  0.61958  0.77255  0.48576  0.95510
     27  2.92124  4.16864  3.44732  2.48865  4.47288  3.87558  4.07990
2.79047  2.52668  2.65702  3.58181  3.01675  2.91059  2.43812  2.05176
2.95410  2.81148  2.51872  5.74073  4.40710     44 r - - -
         2.68618  4.42225  2.77519  2.73123  3.46354  2.40513  3.72494
3.29354  2.67741  2.69355  4.24690  2.90347  2.73739  3.18146  2.89801
2.37887  2.77519  2.98518  4.58477  3.61503
         0.00714  5.34200  6.06435  0.61958  0.77255  0.48576  0.95510
     28  2.81559  3.90429  3.08862  2.53342  4.86428  3.80859  3.99632
4.34533  1.62654  3.06475  4.56867  2.85484  4.20189  2.55153  2.51190
2.23852  2.36187  3.36420  5.96301  4.55635     45 k - - -
```

```
          2.68591  4.42239  2.77511  2.73137  3.46368  2.40511  3.72450
3.29368  2.67755  2.69327  4.24704  2.90361  2.73732  3.18126  2.89815
2.37901  2.77533  2.98504  4.58491  3.61517
          0.03184  3.54007  6.06435  1.74589  0.19175  0.48576  0.95510
     29   3.32349  5.76720  2.26389  2.91534  5.11511  3.97734  4.21423
4.58171  2.90777  4.06479  4.84473  3.45667  4.42284  0.81315  2.57473
3.28046  3.55865  3.38549  6.19220  4.80924     56 q - - -
          2.68631  4.42238  2.77532  2.73136  3.46366  2.40525  3.72507
3.29367  2.67733  2.69325  4.24702  2.90359  2.73752  3.18125  2.89762
2.37899  2.77487  2.98531  4.58490  3.61463
          0.03208  3.53202  6.06435  1.66250  0.21031  0.48576  0.95510
     30   4.01758  5.21753  6.13458  5.64407  4.44524  5.60186  6.23994
1.02706  5.56737  1.67636  3.67993  5.76927  5.80554  5.74707  5.67263
5.01769  3.73122  1.10941  6.51500  5.34637     66 i - - -
          2.68618  4.42225  2.77519  2.73123  3.46354  2.40513  3.72494
3.29354  2.67741  2.69355  4.24690  2.90347  2.73739  3.18146  2.89801
2.37887  2.77519  2.98518  4.58477  3.61503
          0.00714  5.34200  6.06435  0.61958  0.77255  0.48576  0.95510
     31   2.82739  3.75555  2.32339  2.73726  4.84626  3.55343  3.57044
4.32360  2.52605  3.28359  4.55753  1.87955  4.20431  2.65779  3.22990
1.75196  2.94626  3.42535  5.95375  4.55037     67 s - - -
          2.68618  4.42225  2.77519  2.73123  3.46354  2.40513  3.72494
3.29354  2.67741  2.69355  4.24690  2.90347  2.73739  3.18146  2.89801
2.37887  2.77519  2.98518  4.58477  3.61503
          0.00714  5.34200  6.06435  0.61958  0.77255  0.48576  0.95510
     32   2.85775  5.27614  3.02755  2.86894  4.52539  3.86745  3.56912
3.52404  2.86570  2.87678  4.34695  1.24085  4.25966  3.21646  3.33322
2.80028  2.06780  3.34093  5.77510  4.43263     68 n - - -
          2.68618  4.42225  2.77519  2.73123  3.46354  2.40513  3.72494
3.29354  2.67741  2.69355  4.24690  2.90347  2.73739  3.18146  2.89801
2.37887  2.77519  2.98518  4.58477  3.61503
          0.00714  5.34200  6.06435  0.61958  0.77255  0.48576  0.95510
     33   5.05371  6.21500  3.50930  5.33566  2.98122  5.50265  4.52374
4.92743  5.34221  4.14271  5.45363  5.13497  5.86975  5.28867  5.36545
5.00314  5.28637  4.82986  0.32675  2.30688     69 W - - -
          2.68618  4.42225  2.77519  2.73123  3.46354  2.40513  3.72494
3.29354  2.67741  2.69355  4.24690  2.90347  2.73739  3.18146  2.89801
2.37887  2.77519  2.98518  4.58477  3.61503
          0.00714  5.34200  6.06435  0.61958  0.77255  0.48576  0.95510
     34   4.76725  5.90346  6.52331  6.04895  0.46387  6.03723  5.63794
3.26424  5.88870  1.66068  3.00312  6.04060  6.00193  5.60101  5.74416
5.46299  4.95423  3.60457  5.59904  4.16289     70 F - - -
          2.68618  4.42225  2.77519  2.73123  3.46354  2.40513  3.72494
3.29354  2.67741  2.69355  4.24690  2.90347  2.73739  3.18146  2.89801
2.37887  2.77519  2.98518  4.58477  3.61503
          0.00714  5.34200  6.06435  0.61958  0.77255  0.48576  0.95510
     35   1.87110  4.77748  5.21428  4.67404  4.05427  3.81590  5.07813
0.85594  4.52590  2.99764  4.00988  4.71028  4.92592  4.71300  4.62561
3.83550  2.56330  2.08036  5.65620  4.46927     71 i - - -
          2.68618  4.42225  2.77519  2.73123  3.46354  2.40513  3.72494
3.29354  2.67741  2.69355  4.24690  2.90347  2.73739  3.18146  2.89801
2.37887  2.77519  2.98518  4.58477  3.61503
          0.00714  5.34200  6.06435  0.61958  0.77255  0.48576  0.95510
     36   3.19890  6.37478  2.31644  3.05466  6.02034  3.15913  4.84029
5.59812  4.00825  5.06925  5.95018  0.43812  4.79239  4.03012  4.68428
3.78354  4.25440  5.04800  7.21744  5.65141     72 N - - -
```

```
          2.68618  4.42225  2.77519  2.73123  3.46354  2.40513  3.72494
3.29354  2.67741  2.69355  4.24690  2.90347  2.73739  3.18146  2.89801
2.37887  2.77519  2.98518  4.58477  3.61503
          0.00714  5.34200  6.06435  0.61958  0.77255  0.48576  0.95510
     37   0.95754  5.19239  3.50336  2.95212  4.42606  3.90120  4.12151
3.83227  2.75614  3.45512  4.28157  3.21525  4.29701  2.49013  3.38367
2.93031  2.99589  3.10229  5.71853  3.60819     73 a - - -
          2.68618  4.42225  2.77519  2.73123  3.46354  2.40513  3.72494
3.29354  2.67741  2.69355  4.24690  2.90347  2.73739  3.18146  2.89801
2.37887  2.77519  2.98518  4.58477  3.61503
          0.00714  5.34200  6.06435  0.61958  0.77255  0.48576  0.95510
     38   5.39605  6.75076  5.98289  5.44857  6.53330  5.33498  5.88716
6.40085  4.16327  5.64706  6.72914  5.67199  5.88373  5.17534  0.07017
5.51485  5.64904  6.07243  7.11161  6.37835     74 R - - -
          2.68618  4.42225  2.77519  2.73123  3.46354  2.40513  3.72494
3.29354  2.67741  2.69355  4.24690  2.90347  2.73739  3.18146  2.89801
2.37887  2.77519  2.98518  4.58477  3.61503
          0.00714  5.34200  6.06435  0.61958  0.77255  0.48576  0.95510
     39   2.72178  5.31357  3.42905  2.86833  4.57223  3.47409  4.06886
3.63493  2.07838  3.57911  3.58298  3.00335  4.26478  3.20665  1.23184
3.07732  2.94482  2.72990  5.80082  4.45637     75 r - - -
          2.68618  4.42225  2.77519  2.73123  3.46354  2.40513  3.72494
3.29354  2.67741  2.69355  4.24690  2.90347  2.73739  3.18146  2.89801
2.37887  2.77519  2.98518  4.58477  3.61503
          0.00714  5.34200  6.06435  0.61958  0.77255  0.48576  0.95510
     40   4.03832  5.93987  4.98416  4.34153  5.88840  4.56025  4.88535
5.38098  3.04705  4.75564  5.67318  4.52737  5.11962  4.05625  0.25837
3.13852  4.36241  4.92660  6.62406  5.55553     76 R - - -
          2.68618  4.42225  2.77519  2.73123  3.46354  2.40513  3.72494
3.29354  2.67741  2.69355  4.24690  2.90347  2.73739  3.18146  2.89801
2.37887  2.77519  2.98518  4.58477  3.61503
          0.00481  5.33968        *  0.61958  0.77255  0.00000        *
//
HMMER3/f [3.3 | Nov 2019]
NAME  Acetyltransf_1
ACC   PF00583.26
DESC  Acetyltransferase (GNAT) family
LENG  117
ALPH  amino
RF    no
MM    no
CONS  yes
CS    yes
MAP   yes
DATE  Wed Apr 22 22:44:57 2020
NSEQ  223
EFFN  19.153854
CKSUM 632546623
GA    20.70 20.70
TC    20.70 20.70
NC    20.60 20.60
STATS LOCAL MSV       -9.4542  0.71401
STATS LOCAL VITERBI  -10.6440  0.71401
STATS LOCAL FORWARD   -4.0738  0.71401
HMM          A        C        D        E        F        G        H
I        K        L        M        N        P        Q        R        S
T        V        W        Y
            m->m     m->i     m->d     i->m     i->i     d->m     d->d
```

```
  COMPO   2.51073  4.27043  3.03718  2.57457  3.13914  2.58602  3.63191
2.83311  2.79933  2.39916  3.88179  3.18220  3.29449  3.24183  2.80528
2.80092  3.01869  2.72431  4.34377  3.26743
          2.68618  4.42225  2.77519  2.73123  3.46354  2.40513  3.72494
3.29354  2.67741  2.69355  4.24690  2.90347  2.73739  3.18146  2.89801
2.37887  2.77519  2.98518  4.58477  3.61503
          0.01501  6.57790  4.30471  0.61958  0.77255  0.00000        *
      1   2.25236  3.26419  4.88825  4.09282  2.82125  3.64456  4.52900
1.85680  3.18003  1.64751  3.49349  3.79958  4.48896  4.67514  3.31209
3.34469  3.35734  2.62117  2.74880  3.53830      1 l - - H
          2.68618  4.42225  2.77519  2.73123  3.46354  2.40513  3.72494
3.29354  2.67741  2.69355  4.24690  2.90347  2.73739  3.18146  2.89801
2.37887  2.77519  2.98518  4.58477  3.61503
          0.00210  6.56499  7.28734  0.61958  0.77255  0.64726  0.74124
      2   2.70783  5.63403  2.55161  2.31028  4.01472  3.90540  3.70193
3.76211  2.80158  2.14471  4.41053  3.14478  2.91217  2.94106  2.11675
2.92023  3.13786  2.90008  4.93194  3.07250      2 r - - H
          2.68618  4.42225  2.77519  2.73123  3.46354  2.40513  3.72494
3.29354  2.67741  2.69355  4.24690  2.90347  2.73739  3.18146  2.89801
2.37887  2.77519  2.98518  4.58477  3.61503
          0.00210  6.56499  7.28734  0.61958  0.77255  0.52309  0.89818
      3   2.36063  5.40805  2.00236  2.28224  3.85077  3.23183  4.25446
4.02793  2.77216  3.06325  3.57104  2.41386  3.34127  2.93205  2.59621
2.48854  2.90499  3.64646  6.76600  4.12168      3 d - - H
          2.68618  4.42225  2.77519  2.73123  3.46354  2.40513  3.72494
3.29354  2.67741  2.69355  4.24690  2.90347  2.73739  3.18146  2.89801
2.37887  2.77519  2.98518  4.58477  3.61503
          0.00208  6.57100  7.29335  0.61958  0.77255  0.43346  1.04487
      4   2.12264  4.36732  5.94898  4.63877  2.75238  4.06331  4.68458
2.05857  4.79212  1.38435  2.90687  4.27031  4.14178  4.21725  4.47339
4.26293  3.11955  1.76146  4.02485  3.90636      4 l - - H
          2.68618  4.42225  2.77519  2.73123  3.46354  2.40513  3.72494
3.29354  2.67741  2.69355  4.24690  2.90347  2.73739  3.18146  2.89801
2.37887  2.77519  2.98518  4.58477  3.61503
          0.08165  6.57790  2.56373  0.61958  0.77255  0.48576  0.95510
      5   2.92878  4.46938  3.36388  2.83010  2.85459  3.86155  3.35153
2.98836  3.13260  2.37119  3.54351  3.26379  4.35957  3.30044  2.31836
2.85262  2.93893  2.19526  4.37670  2.39985      5 v - - H
          2.68618  4.42225  2.77519  2.73123  3.46354  2.40513  3.72494
3.29354  2.67741  2.69355  4.24690  2.90347  2.73739  3.18146  2.89801
2.37887  2.77519  2.98518  4.58477  3.61503
          0.00224  6.49849  7.22084  0.61958  0.77255  0.20868  1.66948
      6   3.04663  5.59326  2.13513  1.60526  4.75446  3.74494  3.66424
4.42498  2.61726  3.83678  4.83810  2.49560  3.74266  2.66033  2.31512
2.65566  2.69306  3.90873  4.62669  4.21574      6 e - - H
          2.68618  4.42225  2.77519  2.73123  3.46354  2.40513  3.72494
3.29354  2.67741  2.69355  4.24690  2.90347  2.73739  3.18146  2.89801
2.37887  2.77519  2.98518  4.58477  3.61503
          0.00207  6.57790  7.30025  0.61958  0.77255  0.48576  0.95510
      7   2.72272  5.21262  2.88288  2.72524  3.14315  4.08735  3.76188
1.90128  3.73987  1.72687  3.78156  4.26831  5.29256  4.21517  3.04082
2.25076  3.47431  2.96807  2.78904  3.93038      7 l - - H
          2.68618  4.42225  2.77519  2.73123  3.46354  2.40513  3.72494
3.29354  2.67741  2.69355  4.24690  2.90347  2.73739  3.18146  2.89801
2.37887  2.77519  2.98518  4.58477  3.61503
          0.00207  6.57790  7.30025  0.61958  0.77255  0.48576  0.95510
```

8 2.53068 5.34891 3.32706 2.23258 2.98050 4.35886 3.74417
2.95403 3.22327 1.70597 3.99931 3.56631 3.65394 2.22186 3.12611
3.27566 3.32762 3.02096 4.05104 3.05496 8 l - - H
2.68618 4.42225 2.77519 2.73123 3.46354 2.40513 3.72494
3.29354 2.67741 2.69355 4.24690 2.90347 2.73739 3.18146 2.89801
2.37887 2.77519 2.98518 4.58477 3.61503
0.00207 6.57790 7.30025 0.61958 0.77255 0.48576 0.95510
9 2.82523 4.52595 3.21256 2.61859 3.54887 2.31151 4.17166
3.49612 2.43563 2.79345 3.64833 3.05204 3.79406 3.14007 2.53862
2.69299 2.96056 2.17222 3.58297 4.19198 9 v - - H
2.68618 4.42225 2.77519 2.73123 3.46354 2.40513 3.72494
3.29354 2.67741 2.69355 4.24690 2.90347 2.73739 3.18146 2.89801
2.37887 2.77519 2.98518 4.58477 3.61503
0.08165 6.57790 2.56373 0.61958 0.77255 0.48576 0.95510
10 2.49817 4.35183 2.26962 2.13799 4.03525 2.73945 3.93075
3.75819 2.71181 2.27676 4.43099 3.63906 2.72356 3.34569 2.41984
2.72750 2.99268 3.34781 5.17826 5.29093 10 e - - H
2.68618 4.42225 2.77519 2.73123 3.46354 2.40513 3.72494
3.29354 2.67741 2.69355 4.24690 2.90347 2.73739 3.18146 2.89801
2.37887 2.77519 2.98518 4.58477 3.61503
0.00829 6.49849 4.99812 0.61958 0.77255 1.20014 0.35832
11 2.59809 2.96621 2.67849 2.32924 3.36871 3.25365 4.55363
2.82129 2.85526 2.96713 4.19522 3.42784 2.67253 3.08263 3.22510
2.12956 2.75448 3.55120 5.11736 3.30966 11 s - - H
2.68618 4.42225 2.77519 2.73123 3.46354 2.40513 3.72494
3.29354 2.67741 2.69355 4.24690 2.90347 2.73739 3.18146 2.89801
2.37887 2.77519 2.98518 4.58477 3.61503
0.13619 6.49245 2.07302 0.61958 0.77255 1.23665 0.34299
12 2.61434 4.55526 3.44633 2.72848 2.15276 3.53912 3.59972
3.44904 2.76802 2.81563 3.62825 3.35983 2.82617 2.73037 3.07610
2.94509 2.88360 3.64116 3.39080 2.38794 12 f - - T
2.68618 4.42225 2.77519 2.73123 3.46354 2.40513 3.72494
3.29354 2.67741 2.69355 4.24690 2.90347 2.73739 3.18146 2.89801
2.37887 2.77519 2.98518 4.58477 3.61503
0.03278 6.35884 3.48961 0.61958 0.77255 0.20325 1.69321
13 3.26360 4.10441 3.54014 2.22538 1.93397 3.14524 3.42216
3.35292 3.24018 2.81225 4.35520 3.26802 2.35477 3.29673 2.73392
2.85586 2.75881 2.98459 6.62879 3.40607 13 f - - C
2.68777 4.42317 2.77561 2.72958 3.46375 2.40175 3.72507
3.29388 2.67939 2.69234 4.24331 2.90269 2.73887 3.18269 2.89608
2.38022 2.77709 2.98601 4.58031 3.61518
0.37609 1.25759 3.53633 1.68924 0.20415 0.27135 1.43696
14 2.58730 5.23726 3.07595 2.20365 3.10223 2.91469 4.01492
2.29630 2.64421 3.53153 4.35682 3.29259 2.20340 3.45593 3.04583
2.72596 3.35989 2.93482 3.79562 3.52674 24 p - - T
2.68618 4.42225 2.77519 2.73123 3.46354 2.40513 3.72494
3.29354 2.67741 2.69355 4.24690 2.90347 2.73739 3.18146 2.89801
2.37887 2.77519 2.98518 4.58477 3.61503
0.11794 6.55040 2.20890 0.61958 0.77255 0.70310 0.68330
15 2.62163 4.69699 2.98972 2.33348 3.88238 3.09852 3.08844
3.22211 3.14009 2.91593 4.25069 3.03197 1.87754 3.20502 3.04604
2.71871 2.88112 2.65096 5.38242 3.83590 25 p - - H
2.68618 4.42225 2.77519 2.73123 3.46354 2.40513 3.72494
3.29354 2.67741 2.69355 4.24690 2.90347 2.73739 3.18146 2.89801
2.37887 2.77519 2.98518 4.58477 3.61503
0.00237 6.44018 7.16253 0.61958 0.77255 0.44276 1.02795

16 3.05784 4.74966 2.80434 2.57453 2.23915 3.26500 3.38991
3.97754 2.70876 3.00899 5.30788 2.19177 4.34908 3.29864 2.62618
2.98542 3.71832 3.10464 2.53841 2.86528 26 n - - H
2.68618 4.42225 2.77519 2.73123 3.46354 2.40513 3.72494
3.29354 2.67741 2.69355 4.24690 2.90347 2.73739 3.18146 2.89801
2.37887 2.77519 2.98518 4.58477 3.61503
0.00216 6.53286 7.25520 0.61958 0.77255 0.76482 0.62627
17 3.09460 3.85658 3.16151 2.00357 3.44907 2.22959 3.42651
3.98192 2.44011 2.78410 4.08093 2.97869 2.43634 2.82028 3.27988
2.58465 2.97024 3.79740 6.74127 4.25859 27 e - - H
2.68618 4.42225 2.77519 2.73123 3.46354 2.40513 3.72494
3.29354 2.67741 2.69355 4.24690 2.90347 2.73739 3.18146 2.89801
2.37887 2.77519 2.98518 4.58477 3.61503
0.00214 6.54278 7.26513 0.61958 0.77255 0.41828 1.07346
18 2.81912 5.56237 1.95499 2.25335 4.04022 3.71912 3.19777
3.34618 2.73455 3.44605 4.18915 3.75507 3.23514 2.98873 2.09605
2.61745 2.56409 2.79866 5.77406 4.60602 28 d - - H
2.68618 4.42225 2.77519 2.73123 3.46354 2.40513 3.72494
3.29354 2.67741 2.69355 4.24690 2.90347 2.73739 3.18146 2.89801
2.37887 2.77519 2.98518 4.58477 3.61503
0.00209 6.56921 7.29156 0.61958 0.77255 0.46575 0.98795
19 2.02620 3.82926 2.66888 2.19592 4.58607 2.93962 3.81209
3.80959 2.67938 2.58321 5.07126 3.61343 2.89605 3.31035 2.62598
2.67007 2.41649 3.56475 5.83505 3.60001 29 a - - H
2.68618 4.42225 2.77519 2.73123 3.46354 2.40513 3.72494
3.29354 2.67741 2.69355 4.24690 2.90347 2.73739 3.18146 2.89801
2.37887 2.77519 2.98518 4.58477 3.61503
0.00207 6.57559 7.29794 0.61958 0.77255 0.51686 0.90731
20 2.76988 4.33422 2.81832 2.11162 3.10834 3.08795 3.65736
4.07926 2.98460 2.79856 3.52385 3.46728 2.44574 3.06737 3.03337
1.97728 2.89451 3.66915 4.25922 3.74765 30 s - - H
2.68618 4.42225 2.77519 2.73123 3.46354 2.40513 3.72494
3.29354 2.67741 2.69355 4.24690 2.90347 2.73739 3.18146 2.89801
2.37887 2.77519 2.98518 4.58477 3.61503
0.00207 6.57559 7.29794 0.61958 0.77255 0.51686 0.90731
21 2.82589 5.64012 2.99421 1.89014 3.12138 3.28668 3.73538
3.57203 3.00587 1.96482 3.61841 3.53565 3.36499 2.94592 2.98351
2.96180 3.46579 2.63709 5.11930 2.83478 31 e - - H
2.68618 4.42225 2.77519 2.73123 3.46354 2.40513 3.72494
3.29354 2.67741 2.69355 4.24690 2.90347 2.73739 3.18146 2.89801
2.37887 2.77519 2.98518 4.58477 3.61503
0.00207 6.57559 7.29794 0.61958 0.77255 0.51686 0.90731
22 2.19405 6.31504 2.28980 1.84018 3.45535 3.35425 3.68246
3.07117 2.88348 2.71094 4.99496 3.56878 3.13003 3.27128 2.88975
2.99087 3.08273 3.30334 3.54314 3.43544 32 e - - H
2.68618 4.42225 2.77519 2.73123 3.46354 2.40513 3.72494
3.29354 2.67741 2.69355 4.24690 2.90347 2.73739 3.18146 2.89801
2.37887 2.77519 2.98518 4.58477 3.61503
0.00207 6.57559 7.29794 0.61958 0.77255 0.51686 0.90731
23 2.28558 4.84203 2.73918 2.28043 4.48839 4.30748 4.06338
2.36304 2.91190 2.50942 3.69427 3.50823 3.73252 3.27851 2.33938
2.76250 2.92955 2.42280 6.69137 3.42579 33 e - - H
2.68624 4.42332 2.77508 2.72544 3.46559 2.40700 3.72478
3.29064 2.67895 2.69059 4.24587 2.90427 2.73857 3.18210 2.89741
2.37989 2.77640 2.98559 4.58716 3.61600
0.77060 0.84763 2.21790 1.02917 0.44209 0.46680 0.98620

24 3.24724 6.00336 2.99586 2.83106 2.00913 3.98373 3.51965
2.96911 2.95745 2.35821 3.62691 3.28169 4.21458 2.74247 2.37057
3.20445 3.10162 2.66067 3.84875 2.88350 45 f - - H
2.68618 4.42225 2.77519 2.73123 3.46354 2.40513 3.72494
3.29354 2.67741 2.69355 4.24690 2.90347 2.73739 3.18146 2.89801
2.37887 2.77519 2.98518 4.58477 3.61503
0.00232 6.46372 7.18606 0.61958 0.77255 1.33270 0.30621
25 2.46838 3.92747 3.16042 2.26647 2.29964 3.18547 3.61847
3.09938 2.76034 2.40545 4.79719 3.31500 3.93720 2.91418 2.34693
2.82935 3.27382 4.01102 5.06520 3.05531 46 e - - H
2.68618 4.42225 2.77519 2.73123 3.46354 2.40513 3.72494
3.29354 2.67741 2.69355 4.24690 2.90347 2.73739 3.18146 2.89801
2.37887 2.77519 2.98518 4.58477 3.61503
0.00231 6.46678 7.18912 0.61958 0.77255 0.38499 1.14086
26 2.55858 4.92359 2.13668 2.06375 3.42659 3.13523 3.61224
2.63922 2.25090 3.12041 4.46667 3.08683 2.93508 2.91930 3.28544
3.05148 3.46148 3.84349 3.47502 4.28718 47 e - - H
2.68618 4.42225 2.77519 2.73123 3.46354 2.40513 3.72494
3.29354 2.67741 2.69355 4.24690 2.90347 2.73739 3.18146 2.89801
2.37887 2.77519 2.98518 4.58477 3.61503
0.00213 6.54865 7.27099 0.61958 0.77255 0.81787 0.58227
27 2.84549 4.21825 3.13931 2.99674 2.22933 3.40294 3.83245
2.54755 3.16965 2.72906 3.89537 3.43366 3.51392 3.33215 2.82455
2.11566 3.42031 3.93814 2.76910 2.61854 48 s - - H
2.68606 4.41918 2.77424 2.73089 3.46305 2.40626 3.72399
3.29252 2.67708 2.69332 4.24735 2.90358 2.73792 3.18123 2.89743
2.37986 2.77542 2.98550 4.58483 3.61537
0.79255 1.68078 1.01864 1.34588 0.30152 0.81787 0.58227
28 2.50457 4.29640 2.96475 3.09920 1.56920 3.79279 3.47593
2.98843 3.49992 2.00691 3.75785 3.55945 4.58361 3.62336 3.20964
3.30666 3.10207 2.70920 3.49055 3.74536 53 f - - H
2.68618 4.42225 2.77519 2.73123 3.46354 2.40513 3.72494
3.29354 2.67741 2.69355 4.24690 2.90347 2.73739 3.18146 2.89801
2.37887 2.77519 2.98518 4.58477 3.61503
0.00333 6.10175 6.82410 0.61958 0.77255 2.30632 0.10495
29 2.91751 4.91417 3.57530 2.43042 3.09631 3.12218 3.36264
2.65874 2.88300 2.46789 3.99257 3.53915 4.14947 3.57044 1.96068
1.90452 3.28873 2.90739 6.22471 4.34788 54 s - - H
2.68618 4.42225 2.77519 2.73123 3.46354 2.40513 3.72494
3.29354 2.67741 2.69355 4.24690 2.90347 2.73739 3.18146 2.89801
2.37887 2.77519 2.98518 4.58477 3.61503
0.00333 6.10175 6.82410 0.61958 0.77255 2.30632 0.10495
30 2.60704 5.96480 2.16115 2.53999 4.47717 3.54020 3.90930
2.44368 2.42948 2.13861 4.56529 3.07455 3.99763 2.84281 2.47571
3.10399 3.23036 2.93679 6.40475 3.91169 55 l - - S
2.68602 4.42245 2.77484 2.73143 3.46374 2.40517 3.72408
3.29374 2.67761 2.69323 4.24604 2.90311 2.73759 3.18166 2.89820
2.37907 2.77539 2.98538 4.58497 3.61445
0.07614 3.16144 3.47554 1.25034 0.33744 0.17798 1.81374
31 2.84131 4.63373 2.63840 2.24044 3.92690 3.06928 2.48661
2.68132 2.95195 3.56728 3.96136 3.06853 2.33672 3.01571 3.07305
2.86529 2.37988 3.62625 4.88485 4.22777 60 e - - T
2.68618 4.42225 2.77519 2.73123 3.46354 2.40513 3.72494
3.29354 2.67741 2.69355 4.24690 2.90347 2.73739 3.18146 2.89801
2.37887 2.77519 2.98518 4.58477 3.61503
0.00221 6.51168 7.23402 0.61958 0.77255 1.11460 0.39756

```
     32   2.05360  5.55975  2.47196  2.36891  3.46570  3.19462  3.38778
4.58703  2.90576  3.88764  5.33047  2.07474  2.33160  3.27506  2.96105
2.80560  2.39057  4.13271  5.74453  4.60159     61 a - - T
          2.68618  4.42225  2.77519  2.73123  3.46354  2.40513  3.72494
3.29354  2.67741  2.69355  4.24690  2.90347  2.73739  3.18146  2.89801
2.37887  2.77519  2.98518  4.58477  3.61503
          0.00472  6.51168  5.73804  0.61958  0.77255  1.11460  0.39756
     33   2.92086  2.60725  2.82224  1.86236  4.48914  2.29067  3.87057
4.57124  2.78962  3.20548  4.41286  2.63429  2.53167  3.34696  3.09678
2.47872  3.62357  3.24751  6.71686  4.04147     62 e - - E
          2.68560  4.42165  2.77509  2.72865  3.46431  2.40558  3.72368
3.29352  2.67826  2.69360  4.24750  2.90439  2.73596  3.18227  2.89849
2.37930  2.77585  2.98508  4.58465  3.61595
          0.47699  1.06876  3.32657  0.37479  1.16294  0.22530  1.60084
     34   2.74083  2.77314  3.65110  2.05386  3.15116  2.03633  3.15121
3.53830  3.01401  3.00491  4.20546  3.86027  3.59545  3.32039  2.74995
2.66781  2.70680  3.36974  6.67252  3.22656     69 g - - E
          2.68618  4.42225  2.77519  2.73123  3.46354  2.40513  3.72494
3.29354  2.67741  2.69355  4.24690  2.90347  2.73739  3.18146  2.89801
2.37887  2.77519  2.98518  4.58477  3.61503
          0.00214  6.54450  7.26684  0.61958  0.77255  0.61868  0.77361
     35   2.81768  3.25771  4.49011  3.80670  2.44945  2.52615  3.74777
2.78984  4.13429  1.55522  3.78770  3.60853  3.34332  3.86073  3.25921
2.83840  2.89216  2.94588  3.87997  2.84455     70 l - - E
          2.68618  4.42225  2.77519  2.73123  3.46354  2.40513  3.72494
3.29354  2.67741  2.69355  4.24690  2.90347  2.73739  3.18146  2.89801
2.37887  2.77519  2.98518  4.58477  3.61503
          0.00211  6.55692  7.27927  0.61958  0.77255  0.73546  0.65255
     36   3.00697  2.49633  5.17352  5.31743  1.96433  4.41106  4.00281
2.40307  4.40811  1.85004  3.74423  4.88051  4.03345  4.66128  4.53353
3.89517  3.61864  1.95380  2.40442  2.54174     71 l - - E
          2.68618  4.42225  2.77519  2.73123  3.46354  2.40513  3.72494
3.29354  2.67741  2.69355  4.24690  2.90347  2.73739  3.18146  2.89801
2.37887  2.77519  2.98518  4.58477  3.61503
          0.00211  6.55692  7.27927  0.61958  0.77255  0.73546  0.65255
     37   2.43791  3.43463  4.56437  3.24823  2.68088  3.16353  4.71866
2.26912  4.99193  2.20165  3.29499  4.30871  4.56533  4.07503  4.75836
2.36104  2.96990  1.45946  4.81105  4.01698     72 v - - E
          2.68541  4.42262  2.77628  2.73159  3.45982  2.40550  3.72526
3.29106  2.67855  2.69233  4.24756  2.90463  2.73846  3.18266  2.89840
2.37987  2.77459  2.98239  4.58617  3.61447
          0.57098  1.00982  2.64867  0.68136  0.70508  0.35632  1.20480
     38   1.44168  3.79995  3.85862  3.55310  3.73811  2.16229  4.47693
2.06588  3.82015  2.45079  3.85301  4.42961  5.02552  4.95473  3.53729
3.99215  3.41140  2.21657  3.32715  3.37837     75 a - - E
          2.68618  4.42225  2.77519  2.73123  3.46354  2.40513  3.72494
3.29354  2.67741  2.69355  4.24690  2.90347  2.73739  3.18146  2.89801
2.37887  2.77519  2.98518  4.58477  3.61503
          0.00222  6.50684  7.22918  0.61958  0.77255  1.14699  0.38212
     39   2.79436  4.05530  3.03295  1.78763  3.38688  3.71870  4.76369
3.69466  2.28032  2.03241  4.15928  3.55827  3.36194  3.05241  2.78214
2.57587  3.59535  3.14338  4.69647  3.28295     76 e - - E
          2.68618  4.42225  2.77519  2.73123  3.46354  2.40513  3.72494
3.29354  2.67741  2.69355  4.24690  2.90347  2.73739  3.18146  2.89801
2.37887  2.77519  2.98518  4.58477  3.61503
          0.00222  6.50684  7.22918  0.61958  0.77255  0.28973  1.38016
```

40 2.93410 5.69014 2.59837 1.78921 2.52162 4.16045 3.60975
3.01801 2.45698 2.64460 4.16478 3.41649 4.41090 3.28313 2.89993
2.85411 3.32622 3.17810 4.47142 2.60819 77 e - - E
2.68618 4.42225 2.77519 2.73123 3.46354 2.40513 3.72494
3.29354 2.67741 2.69355 4.24690 2.90347 2.73739 3.18146 2.89801
2.37887 2.77519 2.98518 4.58477 3.61503
0.00208 6.57037 7.29272 0.61958 0.77255 0.58334 0.81651
41 2.59104 6.33568 1.57386 1.87357 5.68181 2.44273 4.18517
2.64742 2.94251 4.21409 4.51649 2.19608 4.31339 3.23249 3.71130
2.85886 3.14333 4.52917 6.76773 5.00681 78 d - - T
2.68802 4.42427 2.77588 2.72600 3.46556 2.40525 3.72582
3.29146 2.67224 2.69557 4.24892 2.90455 2.73847 3.18113 2.89726
2.38015 2.77699 2.98419 4.58593 3.61705
0.29996 1.62500 2.77675 1.62677 0.21886 0.58334 0.81651
42 3.29936 6.28875 2.20128 1.66897 5.63512 1.29036 4.57907
5.12207 3.01131 4.59458 5.32909 2.43531 3.02885 3.15906 3.69473
3.20349 3.30233 4.36832 6.72062 4.77680 89 g - - T
2.68618 4.42225 2.77519 2.73123 3.46354 2.40513 3.72494
3.29354 2.67741 2.69355 4.24690 2.90347 2.73739 3.18146 2.89801
2.37887 2.77519 2.98518 4.58477 3.61503
0.00735 6.50684 5.14515 0.61958 0.77255 1.14699 0.38213
43 3.07004 4.26334 3.13772 1.72388 5.62959 4.06036 3.79586
4.61545 1.84770 4.58911 5.32367 3.10440 4.93671 2.70969 1.93592
3.24053 2.06226 3.12927 6.71529 4.03024 90 e - - E
2.68670 4.42286 2.77462 2.73114 3.46399 2.40544 3.72499
3.29166 2.67716 2.69396 4.24751 2.90400 2.73726 3.17921 2.89795
2.37914 2.77554 2.98541 4.58538 3.61564
0.12872 2.11983 7.22407 1.00704 0.45460 0.21342 1.64930
44 3.03558 4.34888 5.95459 5.33828 3.39538 5.14825 5.04862
1.49164 4.49991 1.19081 3.98948 4.82407 2.36050 4.94038 4.18444
4.46010 3.81693 1.92287 5.09627 4.09668 94 l - - E
2.68618 4.42225 2.77519 2.73123 3.46354 2.40513 3.72494
3.29354 2.67741 2.69355 4.24690 2.90347 2.73739 3.18146 2.89801
2.37887 2.77519 2.98518 4.58477 3.61503
0.00207 6.57790 7.30025 0.61958 0.77255 0.48576 0.95510
45 2.19497 4.54200 5.95829 5.34213 3.95524 5.15185 5.48645
1.10575 4.80842 2.16254 3.78840 5.31881 5.51528 4.89244 4.73968
3.77739 3.81196 1.25940 5.97609 4.80039 95 i - - E
2.68618 4.42225 2.77519 2.73123 3.46354 2.40513 3.72494
3.29354 2.67741 2.69355 4.24690 2.90347 2.73739 3.18146 2.89801
2.37887 2.77519 2.98518 4.58477 3.61503
0.00207 6.57790 7.30025 0.61958 0.77255 0.48576 0.95510
46 2.32265 6.42625 5.48559 4.35970 7.25223 0.18621 6.86907
6.78952 6.05924 6.42228 7.18175 5.60590 5.95183 6.18955 4.71750
4.00155 5.04024 5.83811 8.53087 7.40231 96 G - - E
2.68618 4.42225 2.77519 2.73123 3.46354 2.40513 3.72494
3.29354 2.67741 2.69355 4.24690 2.90347 2.73739 3.18146 2.89801
2.37887 2.77519 2.98518 4.58477 3.61503
0.00207 6.57790 7.30025 0.61958 0.77255 0.48576 0.95510
47 2.84678 3.43470 5.91445 4.92238 1.61616 3.47260 2.44149
3.35609 4.31149 2.42793 2.44484 5.29691 5.50685 4.21038 4.23320
2.95907 2.50539 2.78068 3.84169 2.25950 97 f - - E
2.68618 4.42225 2.77519 2.73123 3.46354 2.40513 3.72494
3.29354 2.67741 2.69355 4.24690 2.90347 2.73739 3.18146 2.89801
2.37887 2.77519 2.98518 4.58477 3.61503
0.00207 6.57790 7.30025 0.61958 0.77255 0.48576 0.95510

```
      48   1.52471  3.57162  5.95203  5.33590  3.76688  2.75546  3.94635
 1.49148  4.61318  2.06891  3.49776  4.97462  5.51152  5.24739  4.36931
 3.85905  3.44153  1.92610  5.02450  4.20333     98 i - - E
          2.68898  4.42590  2.77689  2.73249  3.45555  2.40607  3.72860
 3.29299  2.67074  2.68736  4.25055  2.90712  2.73916  3.18160  2.89883
 2.37831  2.77885  2.98528  4.58842  3.61202
          0.12725  2.39264  3.57192  2.93738  0.05446  0.48576  0.95510
      49   2.59599  3.80266  4.03250  2.94799  4.88164  2.21747  4.31196
 3.04025  3.41479  2.67620  2.41885  3.47683  4.62813  3.19584  2.16811
 2.00824  2.62687  3.12413  5.12490  4.41814    125 s - - E
          2.68618  4.42225  2.77519  2.73123  3.46354  2.40513  3.72494
 3.29354  2.67741  2.69355  4.24690  2.90347  2.73739  3.18146  2.89801
 2.37887  2.77519  2.98518  4.58477  3.61503
          0.00213  6.55009  7.27244  0.61958  0.77255  0.70275  0.68363
      50   2.25547  3.50004  5.93656  5.01399  2.66367  1.93810  4.98443
 2.24023  5.10180  1.63787  2.62061  5.29653  5.49303  5.23028  5.10489
 2.97393  3.42834  2.57153  5.43496  2.65014    126 l - - E
          2.68618  4.42225  2.77519  2.73123  3.46354  2.40513  3.72494
 3.29354  2.67741  2.69355  4.24690  2.90347  2.73739  3.18146  2.89801
 2.37887  2.77519  2.98518  4.58477  3.61503
          0.00212  6.55502  7.27736  0.61958  0.77255  0.41389  1.08198
      51   2.84500  3.70405  3.35806  3.31591  2.89133  3.31623  3.44731
 3.39098  2.65076  2.16355  4.49180  2.99057  4.35728  3.59800  2.41121
 2.47881  2.26339  3.14661  3.35649  3.07318    127 l - - E
          2.68618  4.42225  2.77519  2.73123  3.46354  2.40513  3.72494
 3.29354  2.67741  2.69355  4.24690  2.90347  2.73739  3.18146  2.89801
 2.37887  2.77519  2.98518  4.58477  3.61503
          0.00208  6.57358  7.29593  0.61958  0.77255  0.54302  0.86986
      52   3.01352  6.19868  3.01850  2.89580  3.38796  3.76576  4.14061
 2.01190  1.89025  2.47091  3.58912  3.52618  2.53564  3.00677  3.24586
 3.09696  2.96807  3.10095  4.08899  3.93147    128 k - - E
          2.68618  4.42225  2.77519  2.73123  3.46354  2.40513  3.72494
 3.29354  2.67741  2.69355  4.24690  2.90347  2.73739  3.18146  2.89801
 2.37887  2.77519  2.98518  4.58477  3.61503
          0.00208  6.57358  7.29593  0.61958  0.77255  0.54302  0.86986
      53   3.03420  4.61044  2.90841  2.92993  3.55105  3.15993  4.03245
 1.92086  2.62425  2.72360  4.00463  2.60111  3.30331  3.04376  2.34960
 3.20635  3.30967  2.67093  4.47359  3.48557    129 i - - E
          2.68618  4.42225  2.77519  2.73123  3.46354  2.40513  3.72494
 3.29354  2.67741  2.69355  4.24690  2.90347  2.73739  3.18146  2.89801
 2.37887  2.77519  2.98518  4.58477  3.61503
          0.01203  6.57358  4.55028  0.61958  0.77255  0.54302  0.86986
      54   3.04756  6.30832  2.32005  2.84645  3.47841  3.01114  3.92319
 4.03814  3.23451  3.64021  4.41406  2.71102  1.98644  3.01259  2.99687
 2.70995  2.83499  3.50599  3.23885  2.28368    130 p - - E
          2.68618  4.42225  2.77519  2.73123  3.46354  2.40513  3.72494
 3.29354  2.67741  2.69355  4.24690  2.90347  2.73739  3.18146  2.89801
 2.37887  2.77519  2.98518  4.58477  3.61503
          0.00210  6.56365  7.28599  0.61958  0.77255  0.55217  0.85731
      55   3.18225  4.59661  2.15729  2.31322  3.07708  2.86570  2.26590
 4.63126  2.87613  2.65818  4.50812  3.07054  2.88803  2.95517  3.15490
 2.78514  3.31613  2.96915  4.04015  4.12712    131 d - - T
          2.68724  4.41231  2.77311  2.72947  3.46623  2.40798  3.71623
 3.29473  2.67687  2.69357  4.25078  2.90418  2.73477  3.18296  2.89751
 2.38245  2.77304  2.98700  4.57618  3.61482
          1.66420  0.73558  1.10434  1.83374  0.17413  0.60051  0.79525
```

56 2.75253 6.02025 3.06005 1.90031 5.35778 3.26479 3.67565
4.83847 3.15552 2.27392 3.64760 2.89443 1.96475 2.97424 3.32274
2.86446 2.46420 3.23569 4.47934 4.25285 157 e - - T
2.68618 4.42225 2.77519 2.73123 3.46354 2.40513 3.72494
3.29354 2.67741 2.69355 4.24690 2.90347 2.73739 3.18146 2.89801
2.37887 2.77519 2.98518 4.58477 3.61503
0.15738 6.16737 1.94126 0.61958 0.77255 1.27850 0.32636
57 2.50581 4.80482 2.48208 2.46346 3.91151 2.80894 3.04494
4.37224 1.68300 4.11469 4.26314 3.12379 3.16753 3.36429 2.76141
2.34932 3.13379 3.75886 6.44343 3.29712 158 k - - S
2.68618 4.42225 2.77519 2.73123 3.46354 2.40513 3.72494
3.29354 2.67741 2.69355 4.24690 2.90347 2.73739 3.18146 2.89801
2.37887 2.77519 2.98518 4.58477 3.61503
0.00324 6.12829 6.85064 0.61958 0.77255 1.20451 0.35645
58 2.88539 6.09859 3.34922 2.18402 4.27501 1.99128 3.89464
4.11481 2.01149 3.56805 5.13920 3.10658 2.52971 3.13110 2.76206
2.79024 2.64246 3.15527 6.53115 3.46406 159 g - - T
2.68618 4.42225 2.77519 2.73123 3.46354 2.40513 3.72494
3.29354 2.67741 2.69355 4.24690 2.90347 2.73739 3.18146 2.89801
2.37887 2.77519 2.98518 4.58477 3.61503
0.00286 6.25407 6.97642 0.61958 0.77255 0.79813 0.59814
59 3.12085 5.60815 2.68128 2.67051 3.88640 2.03057 3.27329
3.89477 2.04904 3.36033 4.97007 2.26470 2.67340 3.03919 2.73902
3.15014 3.04055 3.37435 4.69538 4.22897 160 g - - T
2.68618 4.42225 2.77519 2.73123 3.46354 2.40513 3.72494
3.29354 2.67741 2.69355 4.24690 2.90347 2.73739 3.18146 2.89801
2.37887 2.77519 2.98518 4.58477 3.61503
0.00248 6.39856 7.12091 0.61958 0.77255 0.97472 0.47369
60 3.06401 3.48475 3.34185 2.68261 3.75005 3.01420 2.65447
3.50097 2.24025 2.60269 4.44593 3.01277 4.68881 3.49996 2.69300
3.47541 2.54207 1.91943 4.66729 3.45544 161 v - - E
2.68618 4.42225 2.77519 2.73123 3.46354 2.40513 3.72494
3.29354 2.67741 2.69355 4.24690 2.90347 2.73739 3.18146 2.89801
2.37887 2.77519 2.98518 4.58477 3.61503
0.00235 6.45047 7.17281 0.61958 0.77255 0.35368 1.21100
61 1.54377 3.98970 4.36326 4.73447 4.08409 2.06620 4.03377
2.57660 4.28353 2.49886 3.60795 4.47504 4.11120 5.01772 3.56687
2.70687 2.67740 2.63782 3.56201 2.42990 162 a - - E
2.68618 4.42225 2.77519 2.73123 3.46354 2.40513 3.72494
3.29354 2.67741 2.69355 4.24690 2.90347 2.73739 3.18146 2.89801
2.37887 2.77519 2.98518 4.58477 3.61503
0.00213 6.54818 7.27053 0.61958 0.77255 0.61476 0.77820
62 2.50744 4.98605 4.31191 1.98298 2.91413 3.29346 2.77264
2.41542 3.75482 2.99438 4.19995 4.45812 4.74410 3.47243 3.89474
3.34215 3.45661 2.03566 4.00119 1.95551 163 y - - E
2.68618 4.42225 2.77519 2.73123 3.46354 2.40513 3.72494
3.29354 2.67741 2.69355 4.24690 2.90347 2.73739 3.18146 2.89801
2.37887 2.77519 2.98518 4.58477 3.61503
0.00211 6.55882 7.28117 0.61958 0.77255 0.50565 0.92411
63 3.69568 4.77020 4.40217 2.86070 3.85671 3.73827 5.47314
1.12675 5.10375 1.56881 3.63815 4.70696 5.50326 5.23484 5.11169
4.45086 2.54338 1.94202 4.74055 4.10723 164 i - - E
2.68618 4.42225 2.77519 2.73123 3.46354 2.40513 3.72494
3.29354 2.67741 2.69355 4.24690 2.90347 2.73739 3.18146 2.89801
2.37887 2.77519 2.98518 4.58477 3.61503
0.03002 6.56948 3.56936 0.61958 0.77255 0.59424 0.80292

64 2.81582 3.83886 2.82682 2.02125 3.65126 2.96521 2.80786
2.21522 3.41117 2.74158 4.26054 2.64787 5.04932 3.66042 2.78553
3.06474 3.23962 3.01662 3.84386 3.04654 165 e - - E
2.68880 4.42487 2.77782 2.72918 3.46616 2.40425 3.71498
3.28774 2.67781 2.69392 4.23890 2.90310 2.74002 3.18041 2.90063
2.37656 2.77782 2.98479 4.58739 3.61765
0.09994 2.39806 5.46863 2.64776 0.07344 0.29943 1.35187
65 2.57186 4.97689 2.24469 2.47583 2.53414 2.09216 4.16705
4.67591 3.15712 3.67905 4.11019 3.49231 3.71576 3.13440 2.79036
1.89137 3.07443 3.41544 5.20647 3.92329 183 s - - E
2.68618 4.42225 2.77519 2.73123 3.46354 2.40513 3.72494
3.29354 2.67741 2.69355 4.24690 2.90347 2.73739 3.18146 2.89801
2.37887 2.77519 2.98518 4.58477 3.61503
0.00208 6.57451 7.29685 0.61958 0.77255 0.53105 0.88670
66 3.74195 4.12909 5.95544 5.33899 2.48143 5.14702 5.48142
1.59246 5.12029 1.29044 3.16585 5.31460 5.51049 5.24844 5.12283
3.73846 3.88778 1.57148 4.20056 3.09476 184 l - - E
2.68618 4.42225 2.77519 2.73123 3.46354 2.40513 3.72494
3.29354 2.67741 2.69355 4.24690 2.90347 2.73739 3.18146 2.89801
2.37887 2.77519 2.98518 4.58477 3.61503
0.00208 6.57451 7.29685 0.61958 0.77255 0.53105 0.88670
67 1.59057 4.40368 5.00131 3.54989 2.70490 2.93876 3.13091
2.99573 5.02899 2.57784 3.59364 4.82006 5.49330 5.17991 3.11333
3.24840 3.80462 1.80130 3.71181 2.18791 185 a - - E
2.68618 4.42225 2.77519 2.73123 3.46354 2.40513 3.72494
3.29354 2.67741 2.69355 4.24690 2.90347 2.73739 3.18146 2.89801
2.37887 2.77519 2.98518 4.58477 3.61503
0.00469 6.57451 5.71968 0.61958 0.77255 0.53105 0.88670
68 3.27291 4.62166 5.96128 5.34550 3.80132 4.56642 5.49038
1.90652 4.48391 2.65120 4.07896 5.32219 5.51808 5.25642 5.13101
4.46725 3.03732 0.56629 5.22249 4.80376 186 V - - E
2.68583 4.42299 2.77475 2.73197 3.46428 2.40505 3.72569
3.29428 2.67815 2.69429 4.24764 2.90421 2.73814 3.18220 2.89740
2.37801 2.77594 2.98152 4.58551 3.61302
0.04024 3.25040 7.29425 2.15093 0.12372 0.56436 0.84100
69 2.21650 5.59911 1.87442 2.98682 4.66036 3.47626 2.79369
3.73775 2.19200 2.87385 4.00019 3.16201 4.59989 3.49396 2.57151
1.83624 3.69908 4.01685 6.76569 4.70770 197 s - - S
2.68618 4.42225 2.77519 2.73123 3.46354 2.40513 3.72494
3.29354 2.67741 2.69355 4.24690 2.90347 2.73739 3.18146 2.89801
2.37887 2.77519 2.98518 4.58477 3.61503
0.00208 6.57190 7.29425 0.61958 0.77255 0.50835 0.92001
70 3.06604 6.33909 3.39984 2.34242 5.68523 4.18290 4.43452
5.17194 2.60567 4.44897 5.06113 3.94985 0.80219 3.27686 2.68153
2.66681 3.49739 4.22122 6.77112 5.35954 198 p - - G
2.68618 4.42225 2.77519 2.73123 3.46354 2.40513 3.72494
3.29354 2.67741 2.69355 4.24690 2.90347 2.73739 3.18146 2.89801
2.37887 2.77519 2.98518 4.58477 3.61503
0.00208 6.57451 7.29685 0.61958 0.77255 0.53105 0.88670
71 2.36103 6.33929 2.33082 1.67833 3.91731 2.97111 3.84007
4.94505 2.34181 2.78474 5.05112 2.96343 3.50707 3.11458 3.02108
2.25927 2.87193 4.38801 5.79581 5.35952 199 e - - G
2.68618 4.42225 2.77519 2.73123 3.46354 2.40513 3.72494
3.29354 2.67741 2.69355 4.24690 2.90347 2.73739 3.18146 2.89801
2.37887 2.77519 2.98518 4.58477 3.61503
0.00208 6.57451 7.29685 0.61958 0.77255 0.53105 0.88670

```
      72   2.52609  4.09140  5.10741  3.52422  2.21078  4.67772  2.08252
3.43261  3.17022  2.89304  4.09877  4.84676  4.71031  3.72444  2.56215
3.63093  4.00515  3.42484  4.27605  1.31426    200 y - - G
           2.68627  4.42234  2.77529  2.73132  3.46126  2.40522  3.72503
3.29363  2.67750  2.69351  4.24699  2.90356  2.73749  3.18155  2.89794
2.37896  2.77529  2.98527  4.58486  3.61512
           0.05223  3.04820  5.67145  0.11699  2.20359  0.45845  1.00039
      73   3.32298  6.29480  4.11643  3.22642  5.10930  4.43938  3.76019
4.80586  3.07122  3.65851  5.34174  3.91760  5.01734  1.41960  0.89572
3.81674  3.49610  4.30985  3.41963  5.34522    202 r - - T
           2.68618  4.42225  2.77519  2.73123  3.46354  2.40513  3.72494
3.29354  2.67741  2.69355  4.24690  2.90347  2.73739  3.18146  2.89801
2.37887  2.77519  2.98518  4.58477  3.61503
           0.00207  6.57514  7.29749  0.61958  0.77255  0.52272  0.89871
      74   3.58996  4.63623  3.72061  3.52189  5.68338  1.15761  3.14297
5.16894  2.08630  4.38969  5.37908  2.26738  4.99573  3.27430  1.83614
3.07280  4.05972  3.71802  6.77078  5.36040    203 g - - T
           2.68618  4.42225  2.77519  2.73123  3.46354  2.40513  3.72494
3.29354  2.67741  2.69355  4.24690  2.90347  2.73739  3.18146  2.89801
2.37887  2.77519  2.98518  4.58477  3.61503
           0.00207  6.57514  7.29749  0.61958  0.77255  0.46333  0.99206
      75   3.58742  4.72572  4.08737  3.35390  4.72721  4.01456  3.42030
4.56491  1.35700  2.95136  3.33681  3.11608  5.00058  1.86720  1.83985
2.46731  3.57208  4.47228  6.75758  5.03376    204 k - - S
           2.68618  4.42225  2.77519  2.73123  3.46354  2.40513  3.72494
3.29354  2.67741  2.69355  4.24690  2.90347  2.73739  3.18146  2.89801
2.37887  2.77519  2.98518  4.58477  3.61503
           0.00735  6.57790  5.12741  0.61958  0.77255  0.48576  0.95510
      76   3.85602  6.82432  4.41372  3.99735  6.25171  0.37145  4.48315
5.69576  3.09152  5.13994  5.92706  2.49821  5.47361  3.68526  3.59746
3.62148  4.36271  5.27696  7.22690  5.88701    205 G - - S
           2.68618  4.42225  2.77519  2.73123  3.46354  2.40513  3.72494
3.29354  2.67741  2.69355  4.24690  2.90347  2.73739  3.18146  2.89801
2.37887  2.77519  2.98518  4.58477  3.61503
           0.00579  6.57263  5.43241  0.61958  0.77255  0.44472  1.02445
      77   2.33512  5.34750  5.95134  5.33505  3.37542  4.81396  4.85551
1.33965  4.59402  1.31674  4.46652  5.31195  5.50884  4.91550  4.45211
4.45710  4.20677  2.12727  5.49630  2.19240    206 l - - H
           2.68618  4.42225  2.77519  2.73123  3.46354  2.40513  3.72494
3.29354  2.67741  2.69355  4.24690  2.90347  2.73739  3.18146  2.89801
2.37887  2.77519  2.98518  4.58477  3.61503
           0.00208  6.57422  7.29657  0.61958  0.77255  0.45631  1.00408
      78   1.35805  5.05648  6.57416  6.28718  6.17326  0.46395  6.73545
4.63783  6.13426  5.30171  4.21915  4.99772  5.84601  6.25969  6.17802
3.64518  4.10574  4.47361  7.62649  6.50840    207 G - - H
           2.68618  4.42225  2.77519  2.73123  3.46354  2.40513  3.72494
3.29354  2.67741  2.69355  4.24690  2.90347  2.73739  3.18146  2.89801
2.37887  2.77519  2.98518  4.58477  3.61503
           0.00207  6.57790  7.30025  0.61958  0.77255  0.48576  0.95510
      79   2.78328  6.33401  3.97225  3.15352  3.39753  4.02779  3.81315
5.16101  2.04895  4.23703  5.37529  3.89017  4.57133  3.03511  2.16381
1.50465  1.61148  4.00227  5.72564  4.52279    208 s - - H
           2.68618  4.42225  2.77519  2.73123  3.46354  2.40513  3.72494
3.29354  2.67741  2.69355  4.24690  2.90347  2.73739  3.18146  2.89801
2.37887  2.77519  2.98518  4.58477  3.61503
           0.00207  6.57790  7.30025  0.61958  0.77255  0.48576  0.95510
```

```
     80   1.79234  6.20746  4.03245  2.37363  4.51346  3.99713  3.49545
3.37516  2.26452  2.02089  3.50491  3.57886  5.03000  2.87200  2.25754
3.32159  2.83606  3.44926  4.58741  3.58054    209 a - - H
          2.68618  4.42225  2.77519  2.73123  3.46354  2.40513  3.72494
3.29354  2.67741  2.69355  4.24690  2.90347  2.73739  3.18146  2.89801
2.37887  2.77519  2.98518  4.58477  3.61503
          0.00207  6.57790  7.30025  0.61958  0.77255  0.48576  0.95510
     81   2.64312  4.83986  5.98026  5.36496  4.46151  4.56916  5.51221
3.04841  5.14787  0.39650  3.31127  5.34317  5.53808  5.27573  5.15169
4.08497  4.23540  2.82379  5.99965  4.37098    210 L - - H
          2.68632  4.42239  2.77534  2.73138  3.46368  2.40487  3.72509
3.29270  2.67755  2.69369  4.24704  2.90361  2.73754  3.18161  2.89815
2.37862  2.77475  2.98533  4.58491  3.61518
          0.02435  3.92682  5.43768  1.21273  0.35295  0.48576  0.95510
     82   2.92106  4.57225  5.95392  4.89582  3.84087  5.14563  5.04498
2.18610  4.68258  1.03531  1.93485  5.31314  5.50918  5.24699  5.12139
4.45757  4.10639  1.74424  4.42417  3.33054    215 l - - H
          2.68618  4.42225  2.77519  2.73123  3.46354  2.40513  3.72494
3.29354  2.67741  2.69355  4.24690  2.90347  2.73739  3.18146  2.89801
2.37887  2.77519  2.98518  4.58477  3.61503
          0.00531  6.57422  5.54748  0.61958  0.77255  0.53474  0.88146
     83   2.54963  6.33658  2.79503  1.65228  5.13184  3.75342  3.82678
4.52841  2.22553  3.45901  4.65182  2.60065  4.98981  2.43760  2.44943
2.84354  2.24028  4.72789  4.38304  4.15209    216 e - - H
          2.68618  4.42225  2.77519  2.73123  3.46354  2.40513  3.72494
3.29354  2.67741  2.69355  4.24690  2.90347  2.73739  3.18146  2.89801
2.37887  2.77519  2.98518  4.58477  3.61503
          0.00445  6.57100  5.79679  0.61958  0.77255  0.49719  0.93710
     84   1.92246  5.79693  2.49311  2.27900  3.54045  4.11757  2.78606
4.43424  1.97661  3.20521  3.96507  3.50490  4.33653  2.91421  3.00084
2.91546  2.78417  3.83392  4.89271  2.87174    217 a - - H
          2.68618  4.42225  2.77519  2.73123  3.46354  2.40513  3.72494
3.29354  2.67741  2.69355  4.24690  2.90347  2.73739  3.18146  2.89801
2.37887  2.77519  2.98518  4.58477  3.61503
          0.00208  6.57234  7.29469  0.61958  0.77255  0.50815  0.92031
     85   1.58191  3.63832  5.95270  5.33634  3.98489  3.80587  5.48000
2.11512  5.11798  1.33449  3.02075  5.31276  5.50931  5.24650  4.14064
3.65188  3.86147  2.18014  5.96969  2.47306    218 l - - H
          2.68618  4.42225  2.77519  2.73123  3.46354  2.40513  3.72494
3.29354  2.67741  2.69355  4.24690  2.90347  2.73739  3.18146  2.89801
2.37887  2.77519  2.98518  4.58477  3.61503
          0.00207  6.57469  7.29704  0.61958  0.77255  0.52865  0.89014
     86   2.31612  4.33944  4.46386  1.74086  3.69392  5.06879  5.37553
1.76246  3.27304  1.90015  3.61307  5.07180  5.43756  3.88308  4.07441
3.87520  3.23873  1.90511  6.03217  3.90156    219 e - - H
          2.68633  4.42106  2.77486  2.73109  3.46369  2.40520  3.72510
3.29369  2.67746  2.69370  4.24553  2.90347  2.73755  3.18162  2.89798
2.37902  2.77492  2.98534  4.58492  3.61489
          0.04904  3.21260  4.87888  0.68124  0.70520  0.45985  0.99799
     87   2.66480  6.33684  2.06495  1.56588  4.94942  3.62844  3.52630
4.55096  2.25037  4.64271  5.02049  3.36767  4.46699  1.91185  2.27901
3.29883  3.22363  4.23235  6.76870  4.69604    222 e - - H
          2.68618  4.42225  2.77519  2.73123  3.46354  2.40513  3.72494
3.29354  2.67741  2.69355  4.24690  2.90347  2.73739  3.18146  2.89801
2.37887  2.77519  2.98518  4.58477  3.61503
          0.08224  6.57097  2.55680  0.61958  0.77255  0.47559  0.97158
```

88 2.76565 5.78228 3.54287 2.49237 2.91633 4.71065 3.25518
2.44552 2.78651 2.44100 4.38198 4.31808 5.09627 3.19963 2.00892
3.57689 3.32103 2.85338 2.47573 2.62337 223 r - - H
2.68618 4.42225 2.77519 2.73123 3.46354 2.40513 3.72494
3.29354 2.67741 2.69355 4.24690 2.90347 2.73739 3.18146 2.89801
2.37887 2.77519 2.98518 4.58477 3.61503
0.00224 6.49616 7.21850 0.61958 0.77255 0.22298 1.61011
89 1.13491 3.41119 5.05794 4.89007 2.94943 3.02345 4.55490
3.52946 2.60015 1.90153 3.10821 5.23921 5.08650 5.16045 5.06071
3.00539 3.62576 2.29847 5.98784 3.91645 224 a - - H
2.68618 4.42225 2.77519 2.73123 3.46354 2.40513 3.72494
3.29354 2.67741 2.69355 4.24690 2.90347 2.73739 3.18146 2.89801
2.37887 2.77519 2.98518 4.58477 3.61503
0.00207 6.57575 7.29809 0.61958 0.77255 0.46805 0.98409
90 2.75860 6.23067 3.72517 2.68026 2.73827 3.70824 4.46645
3.48313 1.84189 2.05638 4.06698 3.82961 3.54907 3.24294 1.67965
3.23836 3.60649 2.91241 5.88584 4.88268 225 r - - H
2.68618 4.42225 2.77519 2.73123 3.46354 2.40513 3.72494
3.29354 2.67741 2.69355 4.24690 2.90347 2.73739 3.18146 2.89801
2.37887 2.77519 2.98518 4.58477 3.61503
0.00480 6.57790 5.68352 0.61958 0.77255 0.48576 0.95510
91 2.57080 4.89624 2.67251 1.65670 5.07646 2.83004 3.14811
4.89384 2.54117 3.85515 5.38038 2.79157 4.35871 2.48795 2.44427
1.96042 3.36952 4.51568 6.77194 5.36016 226 e - - H
2.68618 4.42225 2.77519 2.73123 3.46354 2.40513 3.72494
3.29354 2.67741 2.69355 4.24690 2.90347 2.73739 3.18146 2.89801
2.37887 2.77519 2.98518 4.58477 3.61503
0.00207 6.57517 7.29752 0.61958 0.77255 0.52233 0.89928
92 2.86185 4.49389 3.12462 2.11584 3.96997 2.72831 3.46902
3.38442 2.62238 2.53334 4.15123 2.22683 3.52610 2.87359 2.21587
3.24125 3.29624 3.39235 4.07720 3.88282 227 e - - T
2.68669 4.42301 2.77571 2.73169 3.46298 2.40543 3.72159
3.29397 2.67795 2.69316 4.24618 2.90392 2.73715 3.17832 2.89799
2.37896 2.77595 2.98561 4.58454 3.61484
0.51207 1.19253 2.33002 0.24559 1.52438 0.46356 0.99167
93 2.81279 5.57315 3.09408 2.18722 5.61054 0.85201 3.98176
5.09734 3.08106 4.39947 4.44822 2.79533 4.46856 3.11418 2.94694
3.15714 3.65320 4.65557 6.69628 5.28462 230 g - - T
2.68618 4.42225 2.77519 2.73123 3.46354 2.40513 3.72494
3.29354 2.67741 2.69355 4.24690 2.90347 2.73739 3.18146 2.89801
2.37887 2.77519 2.98518 4.58477 3.61503
0.00229 6.47659 7.19893 0.61958 0.77255 1.27016 0.32960
94 2.21655 2.22677 4.25005 3.45091 2.95001 2.27443 4.64036
1.92991 3.39407 2.10455 3.89992 4.07324 5.34934 4.37218 4.54128
2.85991 3.58311 2.51807 5.96478 3.26675 231 i - - C
2.68635 4.42273 2.77526 2.73077 3.46384 2.40380 3.72522
3.29313 2.67747 2.69357 4.24683 2.90386 2.73777 3.18176 2.89826
2.37909 2.77519 2.98541 4.58525 3.61551
0.43693 1.31554 2.45746 0.02633 3.65001 0.89676 0.52406
95 3.23976 5.44237 2.43577 2.13796 5.56710 3.55047 3.21983
4.11914 1.87589 3.91043 4.91374 2.21383 3.29020 3.09563 2.25675
2.72204 2.68038 4.21215 6.65259 3.35591 233 k - - S
2.68618 4.42225 2.77519 2.73123 3.46354 2.40513 3.72494
3.29354 2.67741 2.69355 4.24690 2.90347 2.73739 3.18146 2.89801
2.37887 2.77519 2.98518 4.58477 3.61503
0.00243 6.41815 7.14049 0.61958 0.77255 1.54943 0.23872

96 2.49118 4.93108 4.00489 2.21207 4.23919 3.64101 3.95164
3.43099 1.60201 3.34004 4.56312 3.54102 3.72252 3.80737 1.74789
2.73168 2.93354 3.16971 6.58627 3.45865 234 k - - E
2.68608 4.42229 2.77524 2.73127 3.46339 2.40517 3.72471
3.29358 2.67745 2.69359 4.24694 2.90351 2.73744 3.18151 2.89805
2.37868 2.77524 2.98523 4.58481 3.61507
0.28623 3.60366 1.50651 0.20276 1.69541 0.32156 1.29106
97 3.19588 4.75144 5.75757 5.14124 3.31919 4.94974 5.28427
1.02496 4.92273 1.61311 3.14052 5.11717 5.31312 5.05100 4.92550
3.38025 3.67793 1.75053 5.29800 3.82183 236 i - - E
2.68516 4.42264 2.77558 2.73094 3.46292 2.40520 3.72429
3.29359 2.67779 2.69344 4.24682 2.90385 2.73745 3.18162 2.89830
2.37912 2.77520 2.98481 4.58516 3.61542
0.31049 1.38055 4.16860 0.03238 3.44625 0.13306 2.08275
98 2.89258 5.91612 3.23679 2.19585 2.62055 3.74970 3.99740
3.10927 3.18481 3.07089 2.99757 3.74050 5.10790 3.38159 3.25308
2.90920 3.02174 2.02555 3.24662 2.13828 238 v - - E
2.68618 4.42225 2.77519 2.73123 3.46354 2.40513 3.72494
3.29354 2.67741 2.69355 4.24690 2.90347 2.73739 3.18146 2.89801
2.37887 2.77519 2.98518 4.58477 3.61503
0.00211 6.55787 7.28021 0.61958 0.77255 0.60535 0.78940
99 2.27966 2.73291 5.94175 4.82587 3.08848 4.82985 5.47055
2.26395 4.78011 0.92167 3.87154 4.48515 5.49997 4.22291 5.11134
3.78796 3.20213 2.47073 3.19466 4.78507 239 l - - E
2.68630 4.42278 2.77545 2.73087 3.46407 2.40483 3.72478
3.28910 2.67779 2.69384 4.24690 2.90381 2.73793 3.18200 2.89802
2.37923 2.77557 2.98461 4.58530 3.61556
0.50624 1.75580 1.49401 0.46731 0.98533 0.45072 1.01382
100 1.73535 4.51655 2.49135 2.13872 3.83138 2.82848 2.96324
3.81460 2.87587 3.65723 5.14993 3.43997 4.28906 3.21085 3.42294
3.21126 2.75587 3.14270 4.20255 2.41941 244 a - - E
2.68618 4.42225 2.77519 2.73123 3.46354 2.40513 3.72494
3.29354 2.67741 2.69355 4.24690 2.90347 2.73739 3.18146 2.89801
2.37887 2.77519 2.98518 4.58477 3.61503
0.00267 6.32412 7.04647 0.61958 0.77255 1.15282 0.37942
101 3.08492 3.27439 4.39611 3.75609 3.98930 3.93828 2.54356
2.76038 3.71705 3.00314 4.49586 4.32650 3.81004 4.31021 3.51750
3.51344 1.82299 1.09369 5.98330 4.76958 245 v - - E
2.68618 4.42225 2.77519 2.73123 3.46354 2.40513 3.72494
3.29354 2.67741 2.69355 4.24690 2.90347 2.73739 3.18146 2.89801
2.37887 2.77519 2.98518 4.58477 3.61503
0.09990 6.38518 2.37101 0.61958 0.77255 1.61983 0.22056
102 2.67914 5.41681 2.61229 2.05140 3.20470 3.25444 3.49429
3.42934 2.99879 2.73247 3.92766 3.56742 4.32793 2.62444 2.79094
2.18764 2.51771 2.96358 5.47677 3.70666 246 e - - E
2.68618 4.42225 2.77519 2.73123 3.46354 2.40513 3.72494
3.29354 2.67741 2.69355 4.24690 2.90347 2.73739 3.18146 2.89801
2.37887 2.77519 2.98518 4.58477 3.61503
0.00275 6.29357 7.01592 0.61958 0.77255 0.60623 0.78835
103 2.42585 5.26069 2.62460 1.93748 3.77509 3.76377 4.08789
3.76679 2.67951 2.82186 3.70227 2.81156 2.70437 3.17246 3.37955
2.47845 3.15146 2.25457 6.65893 4.18460 247 e - - T
2.68618 4.42225 2.77519 2.73123 3.46354 2.40513 3.72494
3.29354 2.67741 2.69355 4.24690 2.90347 2.73739 3.18146 2.89801
2.37887 2.77519 2.98518 4.58477 3.61503
0.01160 6.45278 4.60994 0.61958 0.77255 0.80863 0.58963

```
     104   2.64156  4.43197  1.68196  2.73061  4.38157  3.31801  3.60312
4.63547  3.08802  3.46793  5.30495  2.57957  2.05271  3.24448  3.68047
2.21911  2.89994  3.64246  3.70497  4.17704    248 d - - T
           2.68583  4.42109  2.77533  2.73129  3.46306  2.40519  3.72470
3.29384  2.67763  2.69369  4.24720  2.90367  2.73747  3.18177  2.89786
2.37889  2.77533  2.98520  4.58311  3.61444
           0.20740  1.78297  3.95453  0.04075  3.22056  0.25114  1.50469
     105   3.52509  6.30423  2.76579  3.10882  4.90817  2.79883  3.59170
5.12273  3.51313  2.52863  5.00059  0.78120  4.98100  3.07458  3.37943
3.20815  3.68701  3.76607  6.74135  4.79683    250 n - - E
           2.68618  4.42225  2.77519  2.73123  3.46354  2.40513  3.72494
3.29354  2.67741  2.69355  4.24690  2.90347  2.73739  3.18146  2.89801
2.37887  2.77519  2.98518  4.58477  3.61503
           0.09554  6.55363  2.41131  0.61958  0.77255  0.76918  0.62249
     106   2.69435  5.56009  2.21457  2.51163  3.94738  3.75764  2.92783
3.56258  2.60886  2.36310  3.86800  2.95255  3.38783  2.42931  2.89192
3.29607  2.74195  2.56557  6.67087  4.14219    251 d - - H
           2.68618  4.42225  2.77519  2.73123  3.46354  2.40513  3.72494
3.29354  2.67741  2.69355  4.24690  2.90347  2.73739  3.18146  2.89801
2.37887  2.77519  2.98518  4.58477  3.61503
           0.00233  6.46041  7.18275  0.61958  0.77255  1.40781  0.28061
     107   1.55448  6.24778  3.20147  2.76057  4.45680  2.19945  3.62368
3.73066  2.60326  4.01645  5.01259  3.24295  2.36821  3.06351  2.25132
3.14313  3.47999  3.68788  5.55982  3.75198    252 a - - H
           2.68618  4.42225  2.77519  2.73123  3.46354  2.40513  3.72494
3.29354  2.67741  2.69355  4.24690  2.90347  2.73739  3.18146  2.89801
2.37887  2.77519  2.98518  4.58477  3.61503
           0.00233  6.46041  7.18275  0.61958  0.77255  1.40781  0.28061
     108   1.01206  5.30097  3.99912  3.51300  5.10682  2.67617  4.83400
4.25781  3.65924  3.14542  3.67750  3.19440  5.01256  3.81235  3.50896
1.72765  2.59284  3.43055  6.40960  5.09341    253 a - - H
           2.68634  4.42241  2.77536  2.73140  3.46303  2.40529  3.72511
3.29313  2.67726  2.69334  4.24706  2.90363  2.73756  3.18118  2.89817
2.37883  2.77506  2.98493  4.58493  3.61519
           0.03406  3.58720  5.14773  1.14338  0.38381  0.16609  1.87714
     109   2.89761  3.85069  3.91876  3.03269  4.54173  4.56476  3.29491
1.25759  3.57513  2.46593  3.68268  3.94293  4.94967  3.03939  2.34900
4.16053  3.10205  2.24869  4.55139  3.55774    258 i - - H
           2.68618  4.42225  2.77519  2.73123  3.46354  2.40513  3.72494
3.29354  2.67741  2.69355  4.24690  2.90347  2.73739  3.18146  2.89801
2.37887  2.77519  2.98518  4.58477  3.61503
           0.00208  6.57340  7.29575  0.61958  0.77255  0.54533  0.86667
     110   2.24522  6.33872  3.02291  2.15351  4.95905  2.24256  2.91388
4.82784  2.43133  4.21252  5.37902  2.58627  2.26165  3.25694  1.98927
2.88682  4.05597  4.73010  6.77058  4.96848    259 r - - H
           2.68618  4.42225  2.77519  2.73123  3.46354  2.40513  3.72494
3.29354  2.67741  2.69355  4.24690  2.90347  2.73739  3.18146  2.89801
2.37887  2.77519  2.98518  4.58477  3.61503
           0.00208  6.57340  7.29575  0.61958  0.77255  0.45024  1.01467
     111   3.15715  3.79372  5.37543  4.56113  1.35315  5.14831  5.48256
3.62806  5.12038  1.18492  2.95312  4.93738  5.51188  5.24895  4.73730
4.16148  3.28885  2.31278  5.04641  2.55806    260 l - - H
           2.68618  4.42225  2.77519  2.73123  3.46354  2.40513  3.72494
3.29354  2.67741  2.69355  4.24690  2.90347  2.73739  3.18146  2.89801
2.37887  2.77519  2.98518  4.58477  3.61503
           0.00207  6.57790  7.30025  0.61958  0.77255  0.48576  0.95510
```

```
    112   3.06640  4.89466  5.96170  5.34755  2.24701  5.16178  3.01059
3.58601  5.13058  2.17619  4.02236  5.32440  5.04521  5.25867  5.13532
4.47363  3.70627  3.61315  3.62018  0.69163    261 Y - - H
          2.68626  4.42233  2.77527  2.73131  3.46344  2.40520  3.72502
3.29346  2.67748  2.69354  4.24697  2.90354  2.73747  3.18154  2.89808
2.37895  2.77518  2.98511  4.58345  3.61385
          0.45957  3.19535  1.11629  0.13165  2.09269  0.48576  0.95510
    113   2.66087  6.04746  3.36807  1.18698  5.39255  3.69386  3.78823
4.87854  2.56360  3.35308  3.73895  3.53979  4.27420  2.66847  2.72208
3.35427  2.04413  3.48866  6.48014  4.00872    263 e - - H
          2.68625  4.42232  2.77526  2.73130  3.46361  2.40457  3.72501
3.29361  2.67748  2.69362  4.24697  2.90354  2.73746  3.18153  2.89808
2.37894  2.77526  2.98525  4.58344  3.61510
          0.07664  2.90542  3.96048  0.14332  2.01346  0.09716  2.37962
    114   2.68676  6.32563  3.50647  2.54500  5.67193  4.58555  3.91112
4.77733  1.14142  4.10632  4.49058  3.66769  4.97904  2.90246  1.37135
3.00199  3.72458  4.44818  6.75754  5.34593    265 k - - H
          2.68538  4.42288  2.77540  2.73154  3.46417  2.40555  3.72495
3.29417  2.67555  2.69387  4.24752  2.90387  2.73802  3.18150  2.89709
2.37831  2.77546  2.98572  4.58540  3.61566
          0.39416  1.13149  5.74451  0.02046  3.89953  0.35514  1.20756
    115   2.57145  2.59151  5.24449  4.88422  2.00862  5.12565  3.52509
3.19913  3.62540  1.44799  2.91353  4.07790  5.49065  3.26307  3.01705
3.60328  3.59565  2.17823  5.39272  3.54920    267 l - - T
          2.68618  4.42225  2.77519  2.73123  3.46354  2.40513  3.72494
3.29354  2.67741  2.69355  4.24690  2.90347  2.73739  3.18146  2.89801
2.37887  2.77519  2.98518  4.58477  3.61503
          0.00207  6.57544  7.29779  0.61958  0.77255  0.51879  0.90447
    116   3.20990  7.33934  3.38009  3.41626  6.65772  0.29410  4.14437
6.19139  3.93411  5.64941  6.45980  3.29666  5.65155  4.69823  5.11310
3.71424  4.04484  5.72974  7.78991  6.27645    268 G - - T
          2.68618  4.42225  2.77519  2.73123  3.46354  2.40513  3.72494
3.29354  2.67741  2.69355  4.24690  2.90347  2.73739  3.18146  2.89801
2.37887  2.77519  2.98518  4.58477  3.61503
          0.00207  6.57544  7.29779  0.61958  0.77255  0.51879  0.90447
    117   3.00504  5.36272  5.03993  5.31332  0.60003  4.86489  4.29012
3.54944  4.51798  3.07415  3.38830  5.30521  5.51502  4.90068  5.11787
4.46190  4.21740  3.54816  3.83229  1.92838    269 F - - E
          2.68618  4.42225  2.77519  2.73123  3.46354  2.40513  3.72494
3.29354  2.67741  2.69355  4.24690  2.90347  2.73739  3.18146  2.89801
2.37887  2.77519  2.98518  4.58477  3.61503
          0.00140  6.57477        *  0.61958  0.77255  0.00000        *
//
HMMER3/f [3.3 | Nov 2019]
NAME  ARID
ACC   PF01388.22
DESC  ARID/BRIGHT DNA binding domain
LENG  90
ALPH  amino
RF    no
MM    no
CONS  yes
CS    yes
MAP   yes
DATE  Tue Apr 21 21:07:35 2020
NSEQ  386
EFFN  5.133041
CKSUM 1470591244
```

```
GA    24.10 24.10
TC    24.10 24.10
NC    24.00 24.00
STATS LOCAL MSV       -9.4098  0.71841
STATS LOCAL VITERBI  -10.5785  0.71841
STATS LOCAL FORWARD   -3.5199  0.71841
HMM          A        C        D        E        F        G        H
I        K        L        M        N        P        Q        R        S
T        V        W        Y
            m->m     m->i     m->d     i->m     i->i     d->m     d->d
  COMPO   2.67685  4.49035  3.01642  2.62308  3.11340  2.81839  3.63350
3.01874  2.62621  2.36545  3.71369  3.03522  3.29668  2.94278  2.91794
2.70464  2.94281  2.79111  4.26208  3.18272
          2.68618  4.42225  2.77519  2.73123  3.46354  2.40513  3.72494
3.29354  2.67741  2.69355  4.24690  2.90347  2.73739  3.18146  2.89801
2.37887  2.77519  2.98518  4.58477  3.61503
          0.24962  5.36917  1.53136  0.61958  0.77255  0.00000        *
      1   2.34730  5.44047  2.70305  1.71569  4.78518  3.70459  3.75141
3.99362  2.33346  3.62393  4.18737  3.02140  3.21529  2.16132  2.60964
2.67350  3.00765  3.53637  5.87336  4.46264      1 e - - H
          2.68618  4.42225  2.77519  2.73123  3.46354  2.40513  3.72494
3.29354  2.67741  2.69355  4.24690  2.90347  2.73739  3.18146  2.89801
2.37887  2.77519  2.98518  4.58477  3.61503
          0.00884  5.12840  5.85074  0.61958  0.77255  0.68303  0.70337
      2   2.77486  5.47952  3.20903  2.18286  4.70775  3.71191  3.16904
3.49590  1.70247  3.75740  4.14489  2.80060  3.79148  2.61340  2.14426
2.56657  3.05769  3.76094  5.91393  3.52066      2 k - - H
          2.68618  4.42225  2.77519  2.73123  3.46354  2.40513  3.72494
3.29354  2.67741  2.69355  4.24690  2.90347  2.73739  3.18146  2.89801
2.37887  2.77519  2.98518  4.58477  3.61503
          0.01212  5.22657  5.00922  0.61958  0.77255  0.65727  0.73036
      3   2.67041  5.37669  2.22274  1.79287  4.47292  3.59044  3.11974
4.13802  2.25601  2.65607  4.49438  3.02535  4.16630  2.78205  2.54538
2.74401  3.15234  3.34081  5.78287  4.12795      3 e - - H
          2.68618  4.42225  2.77519  2.73123  3.46354  2.40513  3.72494
3.29354  2.67741  2.69355  4.24690  2.90347  2.73739  3.18146  2.89801
2.37887  2.77519  2.98518  4.58477  3.61503
          0.00764  5.27341  5.99575  0.61958  0.77255  0.37380  1.16513
      4   2.57789  5.28644  2.66028  1.91647  4.80537  3.49166  3.56632
3.77426  2.49762  2.78777  4.37847  2.43757  4.07730  2.36788  2.48393
2.58087  2.80532  3.82422  5.76804  4.33189      4 e - - H
          2.68618  4.42225  2.77519  2.73123  3.46354  2.40513  3.72494
3.29354  2.67741  2.69355  4.24690  2.90347  2.73739  3.18146  2.89801
2.37887  2.77519  2.98518  4.58477  3.61503
          0.00702  5.35850  6.08085  0.61958  0.77255  0.46015  0.99748
      5   3.92062  5.09384  5.49758  4.42451  0.45240  4.93638  4.41690
3.47476  4.43399  3.10624  4.36650  4.85745  5.27038  4.66253  4.74359
4.26478  4.14583  3.55390  3.94826  2.37022      5 F - - H
          2.68618  4.42225  2.77519  2.73123  3.46354  2.40513  3.72494
3.29354  2.67741  2.69355  4.24690  2.90347  2.73739  3.18146  2.89801
2.37887  2.77519  2.98518  4.58477  3.61503
          0.00694  5.36917  6.09152  0.61958  0.77255  0.48576  0.95510
      6   3.02329  4.37364  4.65935  3.61840  3.14036  4.26614  3.62371
2.30412  4.09244  1.45317  2.08140  4.12906  4.63074  3.81735  3.95900
3.43700  3.10277  2.35873  4.04884  2.43774      6 l - - H
          2.68618  4.42225  2.77519  2.73123  3.46354  2.40513  3.72494
3.29354  2.67741  2.69355  4.24690  2.90347  2.73739  3.18146  2.89801
2.37887  2.77519  2.98518  4.58477  3.61503
```

```
          0.01172  5.36917  4.96223  0.61958  0.77255  0.48576  0.95510
      7   2.48613  4.22297  2.65488  2.27511  4.26849  3.30515  3.52437
4.26037  2.22902  3.24177  3.38113  2.86644  4.21205  2.62354  2.52596
2.09759  2.87607  3.40812  5.97802  4.19918      7 s - - H
          2.68618  4.42225  2.77519  2.73123  3.46354  2.40513  3.72494
3.29354  2.67741  2.69355  4.24690  2.90347  2.73739  3.18146  2.89801
2.37887  2.77519  2.98518  4.58477  3.61503
          0.00698  5.36443  6.08677  0.61958  0.77255  0.49594  0.93904
      8   2.54160  4.55136  2.46529  2.46608  4.88196  2.84980  3.78668
4.22745  2.39364  3.18645  4.28041  2.99045  4.21293  2.34730  2.90982
2.45238  2.01932  3.26473  5.97793  4.08048      8 t - - H
          2.68618  4.42225  2.77519  2.73123  3.46354  2.40513  3.72494
3.29354  2.67741  2.69355  4.24690  2.90347  2.73739  3.18146  2.89801
2.37887  2.77519  2.98518  4.58477  3.61503
          0.00697  5.36579  6.08813  0.61958  0.77255  0.49945  0.93360
      9   3.70590  5.03083  5.65616  5.05064  3.17542  4.91802  5.18923
2.48464  4.85353  0.51590  3.50912  5.07403  5.18438  4.88619  4.83149
4.24675  3.92718  2.54637  4.41953  3.36746      9 L - - H
          2.68618  4.42225  2.77519  2.73123  3.46354  2.40513  3.72494
3.29354  2.67741  2.69355  4.24690  2.90347  2.73739  3.18146  2.89801
2.37887  2.77519  2.98518  4.58477  3.61503
          0.00697  5.36579  6.08813  0.61958  0.77255  0.49945  0.93360
     10   2.43397  5.20287  3.24595  2.44019  3.61245  3.45264  3.13061
3.55035  1.91236  2.95884  3.57900  3.08220  4.09689  2.72009  2.33606
2.74033  3.01933  3.47391  5.91828  2.99491     10 k - - H
          2.68618  4.42225  2.77519  2.73123  3.46354  2.40513  3.72494
3.29354  2.67741  2.69355  4.24690  2.90347  2.73739  3.18146  2.89801
2.37887  2.77519  2.98518  4.58477  3.61503
          0.00697  5.36579  6.08813  0.61958  0.77255  0.49945  0.93360
     11   2.62749  5.36168  2.23398  2.14679  4.90249  3.40328  3.55024
4.12223  2.14641  3.61520  4.50812  2.56964  4.21034  1.99971  2.76748
2.54508  2.97127  3.64258  5.98851  4.54332     11 q - - H
          2.68618  4.42225  2.77519  2.73123  3.46354  2.40513  3.72494
3.29354  2.67741  2.69355  4.24690  2.90347  2.73739  3.18146  2.89801
2.37887  2.77519  2.98518  4.58477  3.61503
          0.00697  5.36579  6.08813  0.61958  0.77255  0.49945  0.93360
     12   3.10448  4.41344  5.03764  4.42919  1.01496  4.29068  3.64884
2.74305  4.22493  2.24205  3.32103  4.00313  4.65409  4.36241  4.24955
3.52395  3.36157  2.34688  4.82581  2.54483     12 f - - H
          2.68618  4.42225  2.77519  2.73123  3.46354  2.40513  3.72494
3.29354  2.67741  2.69355  4.24690  2.90347  2.73739  3.18146  2.89801
2.37887  2.77519  2.98518  4.58477  3.61503
          0.00697  5.36579  6.08813  0.61958  0.77255  0.49945  0.93360
     13   2.92123  3.43450  4.88236  4.27898  3.31092  4.25139  1.53761
2.93608  3.97903  1.98013  1.96604  3.76936  4.61699  4.06068  4.08635
3.33148  3.33564  2.62447  3.79763  3.12870     13 h - - H
          2.68618  4.42225  2.77519  2.73123  3.46354  2.40513  3.72494
3.29354  2.67741  2.69355  4.24690  2.90347  2.73739  3.18146  2.89801
2.37887  2.77519  2.98518  4.58477  3.61503
          0.02281  5.36579  4.02425  0.61958  0.77255  0.49945  0.93360
     14   2.38411  5.54393  2.84147  2.04577  4.84573  3.36472  3.66072
3.34508  2.16067  3.50642  3.74476  2.86519  4.20324  2.36335  2.31260
2.67086  2.70840  3.53387  5.97721  4.47347     14 e - - H
          2.68653  4.42262  2.77513  2.73107  3.46356  2.40501  3.72466
3.29363  2.67694  2.69347  4.24754  2.90359  2.73697  3.18170  2.89770
2.37903  2.77541  2.98557  4.58516  3.61510
          0.19350  2.13243  2.85800  2.37250  0.09789  0.56015  0.84658
```

```
     15   2.65167  5.21260  2.86056  2.35123  4.80137  3.75873  3.93855
3.95968  2.16819  3.26117  4.51665  2.11717  3.77144  2.25146  2.26398
2.45238  3.00891  3.31027  5.94985  4.53928     37 n - - H
          2.68618  4.42225  2.77519  2.73123  3.46354  2.40513  3.72494
3.29354  2.67741  2.69355  4.24690  2.90347  2.73739  3.18146  2.89801
2.37887  2.77519  2.98518  4.58477  3.61503
          0.07976  5.29340  2.63618  0.61958  0.77255  0.74630  0.64267
     16   2.97500  5.25132  3.19486  2.59500  4.76196  3.38266  3.58543
4.14753  2.43289  3.27041  4.23529  2.53135  3.99193  2.32637  1.41750
2.62420  2.93028  3.79987  5.42175  4.49594     38 r - - T
          2.68618  4.42225  2.77519  2.73123  3.46354  2.40513  3.72494
3.29354  2.67741  2.69355  4.24690  2.90347  2.73739  3.18146  2.89801
2.37887  2.77519  2.98518  4.58477  3.61503
          0.00805  5.22170  5.94405  0.61958  0.77255  0.73136  0.65634
     17   2.86860  5.15435  3.26374  2.59244  4.63318  1.30013  3.91841
4.09301  2.62949  3.66009  4.41886  2.27042  4.03188  2.87777  3.01104
2.56157  3.18166  3.38427  5.86953  4.28656     39 g - - T
          2.68618  4.42225  2.77519  2.73123  3.46354  2.40513  3.72494
3.29354  2.67741  2.69355  4.24690  2.90347  2.73739  3.18146  2.89801
2.37887  2.77519  2.98518  4.58477  3.61503
          0.01777  5.25939  4.38922  0.61958  0.77255  0.83930  0.56566
     18   2.84947  4.77138  3.55190  2.92044  4.01816  3.47915  4.00713
2.62546  2.89264  2.68591  3.41721  2.89247  3.81921  3.13362  3.04487
2.33124  1.63570  2.59063  5.49991  4.15575     40 t - - S
          2.68642  4.42256  2.77536  2.73143  3.46362  2.40541  3.72475
3.29362  2.67754  2.69371  4.24702  2.90361  2.73543  3.18135  2.89816
2.37903  2.77534  2.98536  4.58462  3.61469
          0.89848  0.77032  2.04062  0.29115  1.37595  0.45332  1.00927
     19   2.93410  4.07948  4.34748  3.97235  2.88077  3.77547  4.38955
1.94289  3.59213  1.79045  2.97051  3.68852  2.11880  4.11855  3.67815
3.09228  2.92766  2.47288  4.97009  3.19920     44 l - - S
          2.68657  4.42271  2.77542  2.73159  3.46355  2.40527  3.72512
3.29186  2.67788  2.69252  4.24728  2.90351  2.73698  3.18071  2.89789
2.37922  2.77548  2.98537  4.58471  3.61538
          0.84683  0.72022  2.47000  0.99890  0.45932  0.81200  0.58693
     20   2.72949  5.42704  2.87623  2.39965  4.39076  2.49948  3.76272
3.68386  2.33205  3.30699  3.88518  2.88373  2.52179  2.71196  2.69605
2.51738  2.51866  3.43472  5.86618  3.92730     58 k - - S
          2.68618  4.42225  2.77519  2.73123  3.46354  2.40513  3.72494
3.29354  2.67741  2.69355  4.24690  2.90347  2.73739  3.18146  2.89801
2.37887  2.77519  2.98518  4.58477  3.61503
          0.23612  5.15271  1.58704  0.61958  0.77255  0.67353  0.71316
     21   2.75735  5.39132  3.08509  2.38486  3.80546  2.74387  3.34574
4.02015  1.74704  3.47163  4.28280  2.89640  3.31795  2.49714  2.65274
2.32504  2.93223  3.73545  5.82646  4.33162     59 k - - S
          2.68618  4.42225  2.77519  2.73123  3.46354  2.40513  3.72494
3.29354  2.67741  2.69355  4.24690  2.90347  2.73739  3.18146  2.89801
2.37887  2.77519  2.98518  4.58477  3.61503
          0.06835  5.03556  2.82070  0.61958  0.77255  0.93744  0.49697
     22   2.75024  4.13342  4.38642  3.03463  2.97716  3.84094  4.29395
2.06219  3.65232  2.03627  3.24762  3.72148  2.64900  3.59529  3.67307
2.22885  3.07583  2.28338  4.29267  3.38757     60 l - - -
          2.68618  4.42225  2.77519  2.73123  3.46354  2.40513  3.72494
3.29354  2.67741  2.69355  4.24690  2.90347  2.73739  3.18146  2.89801
2.37887  2.77519  2.98518  4.58477  3.61503
          0.00950  5.05747  5.77982  0.61958  0.77255  0.56777  0.83653
```

```
     23   3.14149  5.07072  4.32765  3.99619  4.61476  3.95878  4.91729
3.36815  3.78782  3.31761  4.62681  4.20766  0.46097  3.97611  4.03062
3.42103  3.65693  3.54913  6.09128  4.83959     61 P - - -
          2.68608  4.42135  2.77592  2.73124  3.46263  2.40552  3.72491
3.29381  2.67793  2.69355  4.24772  2.90375  2.73667  3.18076  2.89806
2.37893  2.77451  2.98542  4.58201  3.61571
          0.35955  1.61252  2.27666  1.99201  0.14667  0.76535  0.62581
     24   2.61770  4.75738  3.15850  2.87696  3.77070  3.50826  3.80948
3.05295  2.71099  2.00787  3.15074  3.26936  3.98807  3.00422  3.23367
2.71522  2.72189  1.90248  5.04022  3.88492     87 v - - -
          2.68673  4.42304  2.77533  2.73144  3.46354  2.40505  3.72505
3.29293  2.67827  2.69409  4.24751  2.90379  2.73629  3.18172  2.89836
2.37840  2.77499  2.98510  4.58074  3.61421
          0.25920  2.13870  2.20251  2.92612  0.05509  0.80374  0.59358
     25   2.77212  4.21675  4.90288  4.28971  3.04763  4.13256  4.46539
1.36854  3.95305  1.69711  3.27002  4.28641  4.31239  4.21695  4.02717
3.33701  2.84986  1.92934  4.77078  3.69502    118 i - - -
          2.68629  4.42236  2.77515  2.73134  3.46358  2.40521  3.72499
3.29340  2.67749  2.69343  4.24677  2.90343  2.73739  3.18157  2.89780
2.37895  2.77526  2.98478  4.58488  3.61506
          0.09568  2.42710  5.82447  0.96028  0.48254  0.34977  1.22027
     26   2.12622  4.21123  2.28937  2.41939  4.64192  1.98294  3.90421
4.01136  2.64460  3.71235  4.11562  2.44754  4.06329  2.63942  3.08111
2.77174  3.07612  3.42200  5.94675  4.32156    123 g - - S
          2.68619  4.42237  2.77526  2.73132  3.46341  2.40510  3.72491
3.29287  2.67743  2.69351  4.24702  2.90357  2.73752  3.18152  2.89781
2.37899  2.77508  2.98529  4.58489  3.61510
          0.12485  2.16287  6.04317  0.67897  0.70753  0.38957  1.13118
     27   2.89825  5.37010  3.14064  2.67880  4.54921  1.24440  3.63383
4.27952  2.43018  3.73412  4.52797  2.48313  4.11220  3.03031  2.91690
2.49408  3.06915  3.87161  5.97035  4.42376    126 g - - S
          2.68618  4.42225  2.77519  2.73123  3.46354  2.40513  3.72494
3.29354  2.67741  2.69355  4.24690  2.90347  2.73739  3.18146  2.89801
2.37887  2.77519  2.98518  4.58477  3.61503
          0.00694  5.36917  6.09152  0.61958  0.77255  0.48576  0.95510
     28   2.81040  4.71978  3.25161  2.66072  4.44243  3.82722  3.24594
3.88041  1.41393  3.66712  4.17581  3.15878  4.22025  2.63574  1.82519
2.77454  3.07605  3.45774  5.95873  4.38581    127 k - - S
          2.68618  4.42225  2.77519  2.73123  3.46354  2.40513  3.72494
3.29354  2.67741  2.69355  4.24690  2.90347  2.73739  3.18146  2.89801
2.37887  2.77519  2.98518  4.58477  3.61503
          0.00694  5.36917  6.09152  0.61958  0.77255  0.48576  0.95510
     29   2.79630  5.25733  2.26366  1.93280  3.87273  3.57711  3.64108
4.05155  2.24693  3.60520  4.54328  3.13067  2.32342  2.86629  2.65834
2.63964  2.75585  3.56255  5.62861  4.29623    128 e - - S
          2.68618  4.42225  2.77519  2.73123  3.46354  2.40513  3.72494
3.29354  2.67741  2.69355  4.24690  2.90347  2.73739  3.18146  2.89801
2.37887  2.77519  2.98518  4.58477  3.61503
          0.00694  5.36917  6.09152  0.61958  0.77255  0.48576  0.95510
     30   3.42129  4.76677  5.40259  4.79368  3.04755  4.62815  4.96446
1.51147  4.58839  1.51415  2.99711  4.79517  4.80098  4.68379  4.58558
3.94965  3.65064  1.32610  3.90836  4.24257    129 v - - -
          2.68618  4.42225  2.77519  2.73123  3.46354  2.40513  3.72494
3.29354  2.67741  2.69355  4.24690  2.90347  2.73739  3.18146  2.89801
2.37887  2.77519  2.98518  4.58477  3.61503
          0.00694  5.36917  6.09152  0.61958  0.77255  0.48576  0.95510
```

```
     31   3.17728  5.22321  1.02592  2.32697  5.04833  3.77310  3.90175
4.53944  2.86943  3.92106  4.76295  2.13852  3.26846  3.21714  3.36229
2.80892  3.42699  4.07121  6.14689  4.72156    130 d - - S
          2.68618  4.42225  2.77519  2.73123  3.46354  2.40513  3.72494
3.29354  2.67741  2.69355  4.24690  2.90347  2.73739  3.18146  2.89801
2.37887  2.77519  2.98518  4.58477  3.61503
          0.00694  5.36917  6.09152  0.61958  0.77255  0.48576  0.95510
     32   3.14558  4.00286  5.08655  4.47305  3.23295  4.31487  4.50731
2.63425  4.26362  0.70719  3.48143  4.46992  4.53795  4.06743  4.27856
3.62600  3.16632  2.34529  4.51402  3.96592    131 l - - T
          2.68628  4.42235  2.77529  2.73133  3.46363  2.40522  3.72446
3.29364  2.67710  2.69364  4.24699  2.90331  2.73728  3.18156  2.89785
2.37896  2.77529  2.98500  4.58487  3.61513
          0.03076  3.57456  6.09152  1.45692  0.26521  0.48576  0.95510
     33   2.94222  3.90007  4.88508  4.00050  1.81972  4.12642  2.79172
2.93080  4.10554  2.11501  3.44372  4.35329  4.24148  3.32293  4.14496
3.51451  3.33803  2.59210  3.74740  1.62785    139 y - - T
          2.68618  4.42225  2.77519  2.73123  3.46354  2.40513  3.72494
3.29354  2.67741  2.69355  4.24690  2.90347  2.73739  3.18146  2.89801
2.37887  2.77519  2.98518  4.58477  3.61503
          0.00694  5.36917  6.09152  0.61958  0.77255  0.48576  0.95510
     34   2.61027  5.37067  2.98571  2.60383  4.04811  3.55840  3.32613
3.88177  1.78810  2.44104  4.05918  3.02456  4.17829  2.57280  2.17439
2.91757  2.80804  3.41281  5.29927  4.07399    140 k - - H
          2.68618  4.42225  2.77519  2.73123  3.46354  2.40513  3.72494
3.29354  2.67741  2.69355  4.24690  2.90347  2.73739  3.18146  2.89801
2.37887  2.77519  2.98518  4.58477  3.61503
          0.00694  5.36917  6.09152  0.61958  0.77255  0.48576  0.95510
     35   4.40979  5.80500  6.61613  6.01989  2.72152  6.06359  6.30195
2.61199  5.89145  0.34905  2.96484  6.25152  5.97324  5.61641  5.75748
5.47712  4.72794  3.06937  6.15080  5.26518    141 L - - H
          2.68618  4.42225  2.77519  2.73123  3.46354  2.40513  3.72494
3.29354  2.67741  2.69355  4.24690  2.90347  2.73739  3.18146  2.89801
2.37887  2.77519  2.98518  4.58477  3.61503
          0.00694  5.36917  6.09152  0.61958  0.77255  0.48576  0.95510
     36   2.99042  4.76902  4.02288  3.51246  2.12523  3.97654  3.17361
3.25538  2.89506  2.97445  3.87555  3.70388  4.48548  3.65044  3.19416
3.22752  3.28126  3.03890  2.55078  1.41961    142 y - - H
          2.68618  4.42225  2.77519  2.73123  3.46354  2.40513  3.72494
3.29354  2.67741  2.69355  4.24690  2.90347  2.73739  3.18146  2.89801
2.37887  2.77519  2.98518  4.58477  3.61503
          0.00694  5.36917  6.09152  0.61958  0.77255  0.48576  0.95510
     37   2.41304  4.89263  3.12256  2.49071  4.39953  2.81945  3.38379
3.73471  2.30552  3.21931  3.71888  2.67370  4.16128  2.38080  2.09184
2.87555  2.94739  2.83004  5.96337  4.47189    143 r - - H
          2.68618  4.42225  2.77519  2.73123  3.46354  2.40513  3.72494
3.29354  2.67741  2.69355  4.24690  2.90347  2.73739  3.18146  2.89801
2.37887  2.77519  2.98518  4.58477  3.61503
          0.00694  5.36917  6.09152  0.61958  0.77255  0.48576  0.95510
     38   2.02786  3.93677  3.64949  2.39594  3.95944  3.90723  3.95931
2.71501  2.73648  2.10977  3.49775  3.47805  4.36543  2.39416  3.20285
2.84688  2.78102  2.64008  5.38917  3.93182    144 a - - H
          2.68618  4.42225  2.77519  2.73123  3.46354  2.40513  3.72494
3.29354  2.67741  2.69355  4.24690  2.90347  2.73739  3.18146  2.89801
2.37887  2.77519  2.98518  4.58477  3.61503
          0.00694  5.36917  6.09152  0.61958  0.77255  0.48576  0.95510
```

```
     39   3.32547  4.23072  5.55464  4.99677  4.14212  4.87952  5.34526
1.93200  4.84115  2.93474  3.57679  5.04290  5.21609  5.02145  4.91043
3.11393  3.75108  0.56159  5.82694  4.63229    145 V - - H
          2.68618  4.42225  2.77519  2.73123  3.46354  2.40513  3.72494
3.29354  2.67741  2.69355  4.24690  2.90347  2.73739  3.18146  2.89801
2.37887  2.77519  2.98518  4.58477  3.61503
          0.03231  5.36917  3.60676  0.61958  0.77255  0.48576  0.95510
     40   2.78890  4.05874  3.53169  2.73816  3.41423  3.76332  3.52290
2.16050  2.85227  2.60934  2.98098  3.19609  4.28553  2.43479  2.93896
2.53250  2.66470  2.60635  5.34063  3.84137    146 i - - T
          2.68611  4.42226  2.77521  2.73124  3.46355  2.40514  3.72493
3.29355  2.67740  2.69356  4.24691  2.90347  2.73741  3.18147  2.89802
2.37886  2.77516  2.98519  4.58478  3.61504
          0.20415  3.63517  1.84338  0.38796  1.13458  0.58241  0.81769
     41   2.72539  5.46133  2.79489  2.27058  4.65884  3.57017  3.71031
4.17265  1.65091  3.35539  4.29835  3.06597  3.97951  2.15502  2.44956
2.37320  3.14343  3.50378  5.89419  4.48349    148 k - - T
          2.68615  4.42227  2.77518  2.73122  3.46325  2.40515  3.72497
3.29356  2.67743  2.69357  4.24692  2.90349  2.73742  3.18149  2.89803
2.37889  2.77512  2.98521  4.58479  3.61505
          0.04997  3.51419  3.96510  0.65210  0.73595  0.35051  1.21851
     42   2.43732  4.71125  3.20872  2.42717  2.66619  3.52729  3.17633
3.63422  2.52735  2.55045  3.36628  2.69590  4.23036  2.97697  2.21849
2.56181  3.15326  3.34455  5.81488  4.16349    151 r - - T
          2.68625  4.42235  2.77530  2.73134  3.46258  2.40523  3.72505
3.29364  2.67746  2.69317  4.24695  2.90350  2.73746  3.18157  2.89779
2.37894  2.77530  2.98529  4.58487  3.61491
          0.21390  1.65964  6.04670  0.10414  2.31368  0.56543  0.83960
     43   3.90235  6.26834  3.04472  3.00489  5.79693  0.35964  4.72558
5.30512  3.62973  4.81692  5.71586  3.51038  4.81556  3.88988  4.04762
3.73184  4.24621  4.86068  6.97354  5.25845    153 G - - S
          2.68613  4.42226  2.77521  2.73124  3.46355  2.40514  3.72495
3.29355  2.67742  2.69356  4.24691  2.90348  2.73738  3.18147  2.89802
2.37881  2.77521  2.98519  4.58478  3.61504
          0.02756  3.69477  6.06057  0.39941  1.11083  0.41890  1.07227
     44   3.15560  5.25676  3.99946  4.12020  5.70593  0.31800  5.40957
5.18604  4.51472  4.45731  5.62468  3.56159  4.49209  4.68587  4.84597
3.07446  3.52898  4.24454  7.01600  5.80984    155 G - - S
          2.68618  4.42225  2.77519  2.73123  3.46354  2.40513  3.72494
3.29354  2.67741  2.69355  4.24690  2.90347  2.73739  3.18146  2.89801
2.37887  2.77519  2.98518  4.58477  3.61503
          0.01000  5.36917  5.24060  0.61958  0.77255  0.48576  0.95510
     45   2.85791  3.66373  4.45183  4.14439  1.81071  3.54735  3.83688
2.63812  4.10121  2.40218  3.37335  4.29938  3.51462  4.14982  4.17529
2.50575  3.21685  2.68173  2.76445  2.00177    156 f - - H
          2.68618  4.42225  2.77519  2.73123  3.46354  2.40513  3.72494
3.29354  2.67741  2.69355  4.24690  2.90347  2.73739  3.18146  2.89801
2.37887  2.77519  2.98518  4.58477  3.61503
          0.00697  5.36613  6.08848  0.61958  0.77255  0.47815  0.96741
     46   2.31370  5.55593  2.35243  2.31566  4.90103  3.71056  3.24815
3.79309  2.49764  3.58220  4.06002  2.54727  3.86507  2.28835  2.10132
2.70538  3.03831  3.56771  5.81025  4.28148    157 r - - H
          2.68618  4.42225  2.77519  2.73123  3.46354  2.40513  3.72494
3.29354  2.67741  2.69355  4.24690  2.90347  2.73739  3.18146  2.89801
2.37887  2.77519  2.98518  4.58477  3.61503
          0.02273  5.36917  4.02764  0.61958  0.77255  0.48576  0.95510
```

```
     47   2.43638  4.73480  3.13294  2.11012  4.82116  3.72164  3.21320
4.04274  1.62283  3.60516  3.60531  2.63617  4.13730  2.66782  2.73309
2.65198  2.88501  3.34347  5.97638  4.51390    158 k - - H
          2.68515  4.41563  2.77555  2.73166  3.46224  2.40543  3.72481
3.29417  2.67641  2.69481  4.24529  2.90360  2.73701  3.18224  2.89909
2.37977  2.77639  2.98309  4.58680  3.61319
          0.17928  2.37095  2.64882  3.41702  0.03336  0.44913  1.01661
     48   2.83202  4.18440  5.06739  4.17825  3.14136  4.15757  4.62587
1.95075  4.24570  2.03881  3.08536  4.44657  4.49307  4.38045  4.25929
3.59991  3.27357  1.01881  5.11989  3.84244    198 v - - H
          2.68625  4.42255  2.77544  2.73140  3.46334  2.40537  3.72509
3.29340  2.67723  2.69324  4.24599  2.90358  2.73735  3.18050  2.89808
2.37889  2.77535  2.98541  4.58470  3.61533
          0.28599  2.91674  1.63669  2.03148  0.14058  0.69232  0.69398
     49   2.85653  3.72140  2.83214  2.59131  4.57746  3.65763  3.53800
3.26461  2.60933  3.53452  4.10431  2.60330  3.60010  2.71331  3.15164
1.96123  1.85233  3.40805  5.75374  4.03837    212 t - - T
          2.68624  4.42243  2.77457  2.73115  3.46372  2.40522  3.72513
3.29353  2.67702  2.69369  4.24670  2.90349  2.73756  3.18153  2.89807
2.37900  2.77529  2.98509  4.58495  3.61522
          0.17283  2.00749  3.71338  1.07298  0.41853  0.26745  1.44957
     50   2.27335  4.07258  2.37654  2.40946  4.48493  3.31774  3.63406
4.16533  2.40101  3.36300  4.05524  2.30658  3.93043  2.27908  2.62595
2.60829  2.95336  3.49153  5.96705  4.38970    217 a - - T
          2.68616  4.42264  2.77513  2.73121  3.46386  2.40534  3.72499
3.29373  2.67734  2.69394  4.24729  2.90276  2.73743  3.18041  2.89791
2.37877  2.77517  2.98557  4.58516  3.61535
          0.36346  1.36144  3.02735  0.96348  0.48056  0.53477  0.88142
     51   2.78079  5.30995  2.74484  2.08198  4.80834  3.42290  3.81925
3.57689  1.96163  2.76042  4.37400  2.47980  3.73757  2.29005  2.68440
2.75264  2.81760  3.78649  5.95368  4.50345    221 k - - .
          2.68615  4.42231  2.77503  2.73113  3.46360  2.40517  3.72500
3.29360  2.67734  2.69359  4.24671  2.90347  2.73742  3.18152  2.89802
2.37888  2.77522  2.98524  4.58483  3.61509
          0.42512  2.19796  1.44698  0.16421  1.88761  0.69846  0.68786
     52   2.84393  5.38541  2.93774  2.52620  4.72245  2.67910  3.83426
4.20246  2.00792  2.44812  4.18161  2.01916  4.06445  2.68738  2.64879
2.53784  3.02891  3.77176  5.82269  4.41710    223 k - - C
          2.68618  4.42225  2.77519  2.73123  3.46354  2.40513  3.72494
3.29354  2.67741  2.69355  4.24690  2.90347  2.73739  3.18146  2.89801
2.37887  2.77519  2.98518  4.58477  3.61503
          0.03497  5.04106  3.57920  0.61958  0.77255  1.17421  0.36971
     53   2.47440  4.88290  3.40509  2.80989  3.53217  3.12313  3.89392
2.32760  2.21740  2.21415  3.27166  3.30108  3.10103  2.64903  2.97622
2.93844  3.11479  3.13573  5.25798  4.01757    224 l - - H
          2.68627  4.42234  2.77528  2.73132  3.46362  2.40521  3.72503
3.29329  2.67749  2.69345  4.24698  2.90310  2.73729  3.18155  2.89809
2.37882  2.77508  2.98527  4.58486  3.61512
          0.03981  3.32803  5.75442  1.42377  0.27550  0.23352  1.56899
     54   3.56819  5.00478  5.00331  4.46684  3.64291  4.50009  4.67743
3.31175  3.56045  3.23658  4.36601  4.54684  4.97675  4.40157  4.00708
3.52587  3.89184  3.47989  0.46132  3.71387    232 W - - H
          2.68618  4.42225  2.77519  2.73123  3.46354  2.40513  3.72494
3.29354  2.67741  2.69355  4.24690  2.90347  2.73739  3.18146  2.89801
2.37887  2.77519  2.98518  4.58477  3.61503
          0.00706  5.35272  6.07506  0.61958  0.77255  0.55020  0.85999
```

55 2.22321 4.10791 3.08478 2.08039 4.30829 3.16659 3.68031
3.97923 2.37805 3.37306 4.08927 2.80818 2.73822 2.94379 2.58840
2.40200 2.77470 3.54108 4.48975 4.16873 233 e - - H
2.68611 4.42227 2.77521 2.73125 3.46356 2.40506 3.72496
3.29356 2.67742 2.69357 4.24691 2.90348 2.73741 3.18148 2.89802
2.37888 2.77521 2.98508 4.58479 3.61505
0.04003 3.29834 6.07506 0.32693 1.27704 0.44748 1.01954
56 2.87045 5.54991 2.25289 1.98601 4.41783 3.32564 3.76855
3.69013 2.47260 3.05774 4.41917 3.10185 3.79343 2.15704 2.62033
2.50145 2.92029 2.97763 5.83039 4.42523 235 e - - H
2.68618 4.42225 2.77519 2.73123 3.46354 2.40513 3.72494
3.29354 2.67741 2.69355 4.24690 2.90347 2.73739 3.18146 2.89801
2.37887 2.77519 2.98518 4.58477 3.61503
0.00694 5.36917 6.09152 0.61958 0.77255 0.48576 0.95510
57 3.04504 4.64787 5.27283 4.66413 2.28997 4.49126 4.83333
1.44845 4.45614 2.05925 3.18250 4.65665 4.83431 4.57467 4.46161
3.80938 3.52469 1.29074 4.71602 4.05627 236 v - - H
2.68618 4.42225 2.77519 2.73123 3.46354 2.40513 3.72494
3.29354 2.67741 2.69355 4.24690 2.90347 2.73739 3.18146 2.89801
2.37887 2.77519 2.98518 4.58477 3.61503
0.01537 5.36917 4.54743 0.61958 0.77255 0.48576 0.95510
58 1.32132 3.53522 4.92898 4.32273 3.03352 2.68339 4.58182
2.55117 4.08922 1.68811 3.58202 4.37194 4.62048 4.29361 4.14154
3.09888 3.18843 2.56577 5.11823 3.56803 237 a - - H
2.68616 4.42226 2.77521 2.73122 3.46355 2.40514 3.72496
3.29355 2.67740 2.69354 4.24675 2.90345 2.73741 3.18148 2.89802
2.37888 2.77518 2.98516 4.58478 3.61504
0.02486 4.08593 4.86012 0.64343 0.74547 0.51919 0.90388
59 2.63769 5.47395 2.87068 2.19296 4.89294 2.72464 3.50423
4.28365 2.42300 2.61934 4.18459 3.06209 3.60400 2.20971 2.31251
2.39253 2.98605 3.80321 5.98103 4.34991 240 e - - H
2.68618 4.42225 2.77519 2.73123 3.46354 2.40513 3.72494
3.29354 2.67741 2.69355 4.24690 2.90347 2.73739 3.18146 2.89801
2.37887 2.77519 2.98518 4.58477 3.61503
0.00704 5.35532 6.07767 0.61958 0.77255 0.54035 0.87357
60 2.58346 5.54651 2.36651 1.99521 4.64368 3.55750 3.16687
3.78575 1.92552 3.10222 3.89064 3.09079 3.75978 2.62701 2.64401
2.75068 3.08467 3.18369 5.97981 4.51556 241 k - - H
2.68621 4.42228 2.77523 2.73122 3.46330 2.40506 3.72483
3.29357 2.67744 2.69347 4.24693 2.90347 2.73743 3.18149 2.89804
2.37890 2.77523 2.98521 4.58480 3.61506
0.04241 3.44533 4.64319 0.65761 0.73000 0.48658 0.95379
61 2.96353 4.43071 5.06192 4.44699 1.74743 4.27247 4.52907
2.26408 4.23306 1.35369 2.44880 4.07889 4.48540 4.30427 4.24302
3.45930 3.27345 2.34076 5.10042 3.55490 244 l - - H
2.68627 4.42182 2.77494 2.73140 3.46343 2.40490 3.72463
3.29341 2.67757 2.69330 4.24723 2.90331 2.73741 3.18150 2.89811
2.37905 2.77551 2.98536 4.58510 3.61473
0.27996 1.85851 2.42721 1.24671 0.33890 0.52734 0.89202
62 2.97500 4.82335 2.26400 2.62408 4.21504 1.72768 3.86221
4.01242 2.09729 3.66071 4.38753 2.70492 4.01681 2.65102 2.92901
2.72160 3.12757 3.46482 5.92512 3.93725 250 g - - T
2.68618 4.42225 2.77519 2.73123 3.46354 2.40513 3.72494
3.29354 2.67741 2.69355 4.24690 2.90347 2.73739 3.18146 2.89801
2.37887 2.77519 2.98518 4.58477 3.61503
0.06047 5.26799 2.92761 0.61958 0.77255 0.76498 0.62613

```
     63   2.94093  4.37902  4.29295  4.20423  2.21929  3.80884  3.53028
1.69088  3.88856  1.66697  3.44925  3.66968  4.07815  3.90528  4.04678
3.34125  3.20984  2.29322  5.04677  2.85932    251 l - - S
          2.68618  4.42225  2.77519  2.73123  3.46354  2.40513  3.72494
3.29354  2.67741  2.69355  4.24690  2.90347  2.73739  3.18146  2.89801
2.37887  2.77519  2.98518  4.58477  3.61503
          0.17533  5.22494  1.86148  0.61958  0.77255  0.88419  0.53281
     64   2.69246  4.43284  2.88629  2.59355  4.12664  3.39991  3.32464
3.66999  2.38450  3.25708  3.95227  2.73615  1.99653  2.94584  3.13211
2.30621  2.79371  3.48646  5.73829  3.28135    252 p - - -
          2.68618  4.42225  2.77519  2.73123  3.46354  2.40513  3.72494
3.29354  2.67741  2.69355  4.24690  2.90347  2.73739  3.18146  2.89801
2.37887  2.77519  2.98518  4.58477  3.61503
          0.25624  5.06712  1.51529  0.61958  0.77255  0.79257  0.60272
     65   2.71808  4.97626  2.76089  2.21864  4.49616  3.26645  3.80227
3.74118  2.40343  3.30855  4.33133  2.98603  2.29057  2.88104  2.20153
2.71654  2.58470  3.13793  5.76257  4.32773    253 r - - .
          2.68618  4.42225  2.77519  2.73123  3.46354  2.40513  3.72494
3.29354  2.67741  2.69355  4.24690  2.90347  2.73739  3.18146  2.89801
2.37887  2.77519  2.98518  4.58477  3.61503
          0.19681  4.94951  1.76279  0.61958  0.77255  0.92620  0.50427
     66   2.42596  5.31753  2.49356  2.14440  4.40118  2.50501  3.48676
3.87929  2.44245  3.39421  3.87980  2.90115  3.26916  2.76166  2.99344
2.45439  2.63067  3.40054  5.75789  3.92120    254 e - - .
          2.68618  4.42225  2.77519  2.73123  3.46354  2.40513  3.72494
3.29354  2.67741  2.69355  4.24690  2.90347  2.73739  3.18146  2.89801
2.37887  2.77519  2.98518  4.58477  3.61503
          0.10464  4.90518  2.38657  0.61958  0.77255  0.78526  0.60881
     67   2.61380  4.80467  2.16129  2.43273  4.65791  3.34092  3.20702
3.77794  2.50202  3.08371  4.35712  2.65429  3.17957  2.33090  2.89725
2.47101  2.76273  3.47487  5.81273  3.86753    255 d - - -
          2.68618  4.42225  2.77519  2.73123  3.46354  2.40513  3.72494
3.29354  2.67741  2.69355  4.24690  2.90347  2.73739  3.18146  2.89801
2.37887  2.77519  2.98518  4.58477  3.61503
          0.45896  5.01744  1.01766  0.61958  0.77255  0.60610  0.78850
     68   2.73073  4.95339  2.92197  2.52908  3.15564  2.90013  3.18206
3.48845  2.58526  2.90366  3.99800  2.55902  2.59079  2.79692  2.86587
2.64532  2.76786  3.19827  4.37871  3.98689    256 e - - .
          2.68625  4.42275  2.77498  2.73145  3.46381  2.40545  3.72527
3.29381  2.67742  2.69388  4.24664  2.90378  2.73571  3.18043  2.89813
2.37886  2.77526  2.98549  4.58532  3.61558
          1.25938  0.38866  3.26486  0.83068  0.57226  1.45934  0.26447
     69   2.51189  4.86627  3.12586  2.23739  4.13629  2.82065  3.73798
3.12028  2.46600  3.04788  3.37233  2.53710  3.24208  2.65328  2.95287
2.38258  2.81432  3.38762  5.60754  3.91930    263 e - - .
          2.68618  4.42225  2.77519  2.73123  3.46354  2.40513  3.72494
3.29354  2.67741  2.69355  4.24690  2.90347  2.73739  3.18146  2.89801
2.37887  2.77519  2.98518  4.58477  3.61503
          0.01209  4.81684  5.53919  0.61958  0.77255  0.62710  0.76387
     70   2.45966  2.46488  2.84062  2.75058  4.18016  2.87146  3.93677
3.33367  2.60997  2.99679  2.89048  2.92887  3.99830  2.82107  3.17974
2.50496  2.60217  2.92673  5.33848  4.26809    264 a - - .
          2.68609  4.42229  2.77523  2.73128  3.46389  2.40501  3.72530
3.29372  2.67767  2.69361  4.24683  2.90339  2.73686  3.18141  2.89799
2.37893  2.77514  2.98513  4.58512  3.61500
          0.29517  1.88188  2.27019  1.51609  0.24791  0.49012  0.94817
```

71 2.37883 5.41141 3.20355 2.61323 4.73227 3.26613 3.84765
3.95974 2.35555 3.29171 3.81955 2.93409 2.76320 2.66435 2.66785
1.89130 2.46018 3.40258 5.85886 3.91793 274 s - - .
2.68618 4.42225 2.77519 2.73123 3.46354 2.40513 3.72494
3.29354 2.67741 2.69355 4.24690 2.90347 2.73739 3.18146 2.89801
2.37887 2.77519 2.98518 4.58477 3.61503
0.39032 5.19062 1.14700 0.61958 0.77255 0.92692 0.50380
72 2.65756 5.29150 2.85996 2.00956 4.58336 2.91406 3.64183
3.87898 2.43868 3.48855 4.15529 3.02470 3.02092 2.24367 2.95997
2.53394 2.30401 3.27516 5.73131 4.32757 275 e - - .
2.68618 4.42225 2.77519 2.73123 3.46354 2.40513 3.72494
3.29354 2.67741 2.69355 4.24690 2.90347 2.73739 3.18146 2.89801
2.37887 2.77519 2.98518 4.58477 3.61503
0.01882 4.83132 4.54024 0.61958 0.77255 0.34530 1.23103
73 2.60818 5.11173 2.60626 2.26657 4.84214 3.23719 3.85124
4.18026 2.58288 3.80330 4.14072 2.10264 3.78417 2.76647 2.89189
1.82720 2.63547 3.39656 5.93238 4.30915 276 s - - .
2.68618 4.42225 2.77519 2.73123 3.46354 2.40513 3.72494
3.29354 2.67741 2.69355 4.24690 2.90347 2.73739 3.18146 2.89801
2.37887 2.77519 2.98518 4.58477 3.61503
0.07966 5.26082 2.63981 0.61958 0.77255 0.74673 0.64229
74 2.04891 4.04092 4.40584 3.96040 3.25636 2.58787 4.34835
2.26077 3.72174 1.90453 3.47036 3.64960 4.19379 3.55595 3.61426
3.18264 2.78742 1.83679 4.96958 3.85034 277 v - - .
2.68618 4.42225 2.77519 2.73123 3.46354 2.40513 3.72494
3.29354 2.67741 2.69355 4.24690 2.90347 2.73739 3.18146 2.89801
2.37887 2.77519 2.98518 4.58477 3.61503
0.02438 5.20612 3.98446 0.61958 0.77255 0.77857 0.61445
75 1.77559 5.02182 2.90855 2.49768 4.54912 2.73041 3.86903
3.73462 2.61559 3.26209 4.38567 3.07226 3.40415 3.07225 3.00068
2.17250 2.34056 3.18199 5.84296 4.37525 278 a - - H
2.68618 4.42225 2.77519 2.73123 3.46354 2.40513 3.72494
3.29354 2.67741 2.69355 4.24690 2.90347 2.73739 3.18146 2.89801
2.37887 2.77519 2.98518 4.58477 3.61503
0.01245 5.22621 4.96145 0.61958 0.77255 0.80124 0.59561
76 2.64252 5.38197 2.91764 2.55736 3.35412 3.68679 3.64056
3.90632 2.45662 3.27351 3.45392 2.58048 3.89921 2.01358 2.78998
2.62446 2.66125 3.02393 4.02380 3.46340 279 q - - H
2.68618 4.42192 2.77514 2.73122 3.46361 2.40520 3.72463
3.29339 2.67733 2.69362 4.24697 2.90345 2.73732 3.18153 2.89808
2.37894 2.77523 2.98516 4.58484 3.61510
0.03629 3.40907 5.96590 1.19502 0.36054 0.48979 0.94869
77 2.03858 4.66343 3.18958 2.31945 4.36186 3.55232 3.35537
3.95241 2.53168 2.81696 3.61294 2.88000 4.13245 2.36705 2.84052
2.40269 2.64556 2.86958 5.89983 4.28309 285 a - - H
2.68618 4.42225 2.77519 2.73123 3.46354 2.40513 3.72494
3.29354 2.67741 2.69355 4.24690 2.90347 2.73739 3.18146 2.89801
2.37887 2.77519 2.98518 4.58477 3.61503
0.00728 5.32174 6.04409 0.61958 0.77255 0.39101 1.12816
78 3.07934 3.82957 5.14883 4.53682 3.38746 4.36822 4.60239
2.30274 4.32668 0.79236 3.22156 4.52881 4.72369 4.45470 4.33726
3.61799 3.15541 2.16135 5.18862 3.97180 286 l - - H
2.68618 4.42225 2.77519 2.73123 3.46354 2.40513 3.72494
3.29354 2.67741 2.69355 4.24690 2.90347 2.73739 3.18146 2.89801
2.37887 2.77519 2.98518 4.58477 3.61503
0.00694 5.36917 6.09152 0.61958 0.77255 0.48576 0.95510

79 2.50681 4.05616 3.17220 2.64231 4.68675 3.49685 4.01290
3.97153 1.37795 3.45449 4.20683 3.15055 4.21779 2.54924 2.13252
2.51747 3.19065 3.76967 5.96770 4.47815 287 k - - H
2.68618 4.42225 2.77519 2.73123 3.46354 2.40513 3.72494
3.29354 2.67741 2.69355 4.24690 2.90347 2.73739 3.18146 2.89801
2.37887 2.77519 2.98518 4.58477 3.61503
0.00694 5.36917 6.09152 0.61958 0.77255 0.48576 0.95510
80 2.38942 5.45026 3.10677 2.41815 2.96145 3.56191 2.94703
4.07405 2.35319 3.40691 4.39746 2.43160 4.21416 2.47202 2.55611
2.17575 2.74137 3.73894 5.98159 4.50702 288 s - - H
2.68618 4.42225 2.77519 2.73123 3.46354 2.40513 3.72494
3.29354 2.67741 2.69355 4.24690 2.90347 2.73739 3.18146 2.89801
2.37887 2.77519 2.98518 4.58477 3.61503
0.00694 5.36917 6.09152 0.61958 0.77255 0.48576 0.95510
81 2.40287 2.80872 4.73213 4.34226 2.94709 4.26863 2.87800
1.85064 3.91318 2.09506 3.34875 3.94226 4.63302 4.09602 4.15448
3.14152 3.00274 2.21492 4.88202 2.31946 289 i - - H
2.68620 4.42227 2.77522 2.73126 3.46356 2.40515 3.72497
3.29356 2.67743 2.69357 4.24633 2.90349 2.73742 3.18128 2.89803
2.37889 2.77522 2.98504 4.58479 3.61505
0.02122 3.97760 6.09152 0.81504 0.58451 0.48576 0.95510
82 5.63617 6.41231 6.28752 6.33497 2.13719 5.94930 4.13383
5.15876 6.08614 4.21071 5.63995 5.41193 6.18167 5.62053 5.80495
5.37191 5.80954 5.13064 4.33419 0.23788 293 Y - - H
2.68618 4.42225 2.77519 2.73123 3.46354 2.40513 3.72494
3.29354 2.67741 2.69355 4.24690 2.90347 2.73739 3.18146 2.89801
2.37887 2.77519 2.98518 4.58477 3.61503
0.00694 5.36917 6.09152 0.61958 0.77255 0.48576 0.95510
83 2.72121 4.20780 3.00377 1.97771 3.78568 3.58545 3.46217
3.60523 2.50116 2.90016 3.56315 2.72630 4.24347 2.56392 2.83886
2.84769 2.92273 2.50068 5.57173 3.12097 294 e - - H
2.68618 4.42225 2.77519 2.73123 3.46354 2.40513 3.72494
3.29354 2.67741 2.69355 4.24690 2.90347 2.73739 3.18146 2.89801
2.37887 2.77519 2.98518 4.58477 3.61503
0.00694 5.36917 6.09152 0.61958 0.77255 0.48576 0.95510
84 2.70698 5.55831 2.84239 2.25113 4.90443 3.16474 3.07767
4.39110 1.86670 3.71868 4.04235 2.70329 4.21216 2.52907 1.89491
2.77499 2.92441 3.90560 5.99035 4.53260 295 k - - H
2.68618 4.42225 2.77519 2.73123 3.46354 2.40513 3.72494
3.29354 2.67741 2.69355 4.24690 2.90347 2.73739 3.18146 2.89801
2.37887 2.77519 2.98518 4.58477 3.61503
0.00755 5.36917 5.85630 0.61958 0.77255 0.48576 0.95510
85 3.03048 3.54368 4.84377 4.30958 2.94091 4.25717 3.30081
1.95162 3.93384 2.27378 3.56249 2.98966 4.62244 4.28743 3.44078
3.29283 3.13449 2.59127 3.52797 1.54236 296 y - - H
2.68618 4.42225 2.77520 2.73123 3.46354 2.40510 3.72495
3.29354 2.67741 2.69355 4.24690 2.90347 2.73740 3.18146 2.89801
2.37887 2.77520 2.98518 4.58477 3.61503
0.01694 4.71450 4.84939 0.55176 0.85788 0.48821 0.95121
86 4.03076 4.30538 6.02067 5.43304 3.23516 5.35094 5.69056
2.01711 5.26872 0.46805 3.59158 5.34857 5.53178 5.25118 5.24144
4.70777 4.25052 2.66823 5.89632 4.84718 298 L - - H
2.68618 4.42225 2.77519 2.73123 3.46354 2.40513 3.72494
3.29354 2.67741 2.69355 4.24690 2.90347 2.73739 3.18146 2.89801
2.37887 2.77519 2.98518 4.58477 3.61503
0.00699 5.36299 6.08533 0.61958 0.77255 0.51060 0.91662

```
     87   2.22951  4.72872  2.53945  2.67918  3.54255  3.28494  3.73473
3.41712  2.76286  1.71509  4.12630  3.45130  4.31180  2.94482  3.06525
2.72244  3.22429  3.13971  4.13975  3.10792    299 l - - H
          2.68618  4.42225  2.77519  2.73123  3.46354  2.40513  3.72494
3.29354  2.67741  2.69355  4.24690  2.90347  2.73739  3.18146  2.89801
2.37887  2.77519  2.98518  4.58477  3.61503
          0.02276  5.36299  4.02774  0.61958  0.77255  0.47436  0.97360
     88   2.06450  5.44639  2.75414  2.24107  4.56194  3.45924  3.66686
4.29082  2.56833  3.72311  4.39767  3.11635  1.58557  2.97863  2.84208
2.72492  3.08362  3.82082  5.97369  4.56515    300 p - - H
          2.68618  4.42225  2.77519  2.73123  3.46354  2.40513  3.72494
3.29354  2.67741  2.69355  4.24690  2.90347  2.73739  3.18146  2.89801
2.37887  2.77519  2.98518  4.58477  3.61503
          0.03249  5.35300  3.60332  0.61958  0.77255  0.54915  0.86143
     89   5.28010  6.20010  6.18502  6.13510  0.87696  5.79127  4.42632
4.81555  5.89924  2.97845  4.38022  5.33337  6.03546  5.50926  5.66237
5.19545  5.46901  4.77970  3.80288  0.84828    301 y - - H
          2.68618  4.42225  2.77519  2.73123  3.46354  2.40513  3.72494
3.29354  2.67741  2.69355  4.24690  2.90347  2.73739  3.18146  2.89801
2.37887  2.77519  2.98518  4.58477  3.61503
          0.11781  5.32775  2.24169  0.61958  0.77255  0.63892  0.75048
     90   3.97835  6.79772  2.04870  0.43131  6.03163  4.01889  4.33226
5.64425  3.87723  5.06274  5.97789  3.02621  4.71787  3.86896  4.60980
3.78433  4.29782  5.15022  7.20556  5.55841    302 E - - H
          2.68618  4.42225  2.77519  2.73123  3.46354  2.40513  3.72494
3.29354  2.67741  2.69355  4.24690  2.90347  2.73739  3.18146  2.89801
2.37887  2.77519  2.98518  4.58477  3.61503
          0.00545  5.21539        *  0.61958  0.77255  0.00000        *
//
HMMER3/f [3.3 | Nov 2019]
NAME  Auxin_resp
ACC   PF06507.14
DESC  Auxin response factor
LENG  83
ALPH  amino
RF    no
MM    no
CONS  yes
CS    no
MAP   yes
DATE  Fri Apr 17 15:29:11 2020
NSEQ  80
EFFN  1.811523
CKSUM 2375421058
GA    21.10 21.10
TC    21.20 21.20
NC    20.80 21.00
STATS LOCAL MSV       -9.3008  0.71849
STATS LOCAL VITERBI   -9.6186  0.71849
STATS LOCAL FORWARD   -3.9360  0.71849
HMM          A        C        D        E        F        G        H
I        K        L        M        N        P        Q        R        S
T        V        W        Y
            m->m     m->i     m->d     i->m     i->i     d->m     d->d
  COMPO   2.53419  4.41307  3.14256  2.66829  3.04145  2.95775  3.79512
2.88759  2.70900  2.70869  3.46686  3.18366  3.36170  3.12140  2.83924
2.53088  2.85645  2.53364  3.67259  3.40639
```

```
         2.68618  4.42225  2.77519  2.73123  3.46354  2.40513  3.72494
3.29354  2.67741  2.69355  4.24690  2.90347  2.73739  3.18146  2.89801
2.37887  2.77519  2.98518  4.58477  3.61503
         0.13922  4.55604  2.12481  0.61958  0.77255  0.00000        *
      1  0.86555  4.40104  4.21868  3.86151  4.16126  3.44424  4.69881
2.89913  3.78846  3.03310  4.08576  3.89339  4.15530  4.07119  4.01924
2.89395  3.08107  1.92734  5.73315  4.53365      1 a - - -
         2.68618  4.42225  2.77519  2.73123  3.46354  2.40513  3.72494
3.29354  2.67741  2.69355  4.24690  2.90347  2.73739  3.18146  2.89801
2.37887  2.77519  2.98518  4.58477  3.61503
         0.01778  4.43460  5.15695  0.61958  0.77255  0.40285  1.10387
      2  1.72689  4.35530  3.72884  3.15851  3.28158  3.71988  3.99465
2.76985  3.02541  2.51737  3.46711  3.52200  4.10076  3.38286  3.34441
2.12063  2.89399  2.18016  4.43276  3.73139      2 a - - -
         2.68618  4.42225  2.77519  2.73123  3.46354  2.40513  3.72494
3.29354  2.67741  2.69355  4.24690  2.90347  2.73739  3.18146  2.89801
2.37887  2.77519  2.98518  4.58477  3.61503
         0.01586  4.54749  5.26984  0.61958  0.77255  0.50105  0.93114
      3  2.69901  5.20912  2.85467  2.13649  4.54117  3.49467  2.20891
4.01796  2.40731  3.50776  4.25440  2.35467  3.89366  2.51354  2.73492
2.58088  2.77067  3.59300  5.64786  4.01615      3 e - - -
         2.68618  4.42225  2.77519  2.73123  3.46354  2.40513  3.72494
3.29354  2.67741  2.69355  4.24690  2.90347  2.73739  3.18146  2.89801
2.37887  2.77519  2.98518  4.58477  3.61503
         0.01586  4.54749  5.26984  0.61958  0.77255  0.50105  0.93114
      4  1.53220  4.41817  3.63784  3.06934  3.48605  3.70155  3.67465
2.91086  2.99856  2.02264  3.27601  3.46098  4.08460  3.31075  3.03217
2.82895  2.95221  2.65629  5.00532  3.77450      4 a - - -
         2.68618  4.42225  2.77519  2.73123  3.46354  2.40513  3.72494
3.29354  2.67741  2.69355  4.24690  2.90347  2.73739  3.18146  2.89801
2.37887  2.77519  2.98518  4.58477  3.61503
         0.01586  4.54749  5.26984  0.61958  0.77255  0.47632  0.97039
      5  1.71982  4.25397  4.36116  3.77993  3.14890  3.95543  4.29029
1.83916  3.65557  2.22891  3.37426  3.58902  4.31990  3.87166  3.82227
3.25349  2.99546  1.79782  4.94007  3.74767      5 a - - -
         2.68618  4.42225  2.77519  2.73123  3.46354  2.40513  3.72494
3.29354  2.67741  2.69355  4.24690  2.90347  2.73739  3.18146  2.89801
2.37887  2.77519  2.98518  4.58477  3.61503
         0.01573  4.55604  5.27839  0.61958  0.77255  0.48576  0.95510
      6  1.98308  4.96288  3.02278  2.45719  4.21596  3.28011  3.54885
3.19107  2.42188  3.22878  3.94470  2.92185  3.92706  2.84255  2.99518
1.96610  2.66445  3.24662  5.45742  4.11136      6 s - - -
         2.68618  4.42225  2.77519  2.73123  3.46354  2.40513  3.72494
3.29354  2.67741  2.69355  4.24690  2.90347  2.73739  3.18146  2.89801
2.37887  2.77519  2.98518  4.58477  3.61503
         0.01573  4.55604  5.27839  0.61958  0.77255  0.48576  0.95510
      7  2.52456  4.72409  3.08061  2.52321  4.08140  3.53335  3.60161
3.65890  2.47267  3.24211  3.64968  2.59216  3.92409  2.50831  2.81420
2.33915  1.93588  3.31765  5.46603  3.95422      7 t - - -
         2.68618  4.42225  2.77519  2.73123  3.46354  2.40513  3.72494
3.29354  2.67741  2.69355  4.24690  2.90347  2.73739  3.18146  2.89801
2.37887  2.77519  2.98518  4.58477  3.61503
         0.01573  4.55604  5.27839  0.61958  0.77255  0.48576  0.95510
      8  2.77166  5.23991  2.86017  2.44820  4.57630  1.93114  3.46336
4.04719  2.02639  3.53449  4.28834  2.59014  3.92325  2.57768  2.58450
2.66130  3.00183  3.62708  5.67041  4.27687      8 g - - -
```

2.68618 4.42225 2.77519 2.73123 3.46354 2.40513 3.72494
3.29354 2.67741 2.69355 4.24690 2.90347 2.73739 3.18146 2.89801
2.37887 2.77519 2.98518 4.58477 3.61503
0.01573 4.55604 5.27839 0.61958 0.77255 0.48576 0.95510
9 2.71351 3.61431 3.10022 2.67805 3.75146 3.58810 3.51092
3.32754 2.60798 2.70357 3.42923 3.04250 3.97504 2.68391 3.04848
1.93346 2.29158 2.97001 5.27602 3.97983 9 s - - -
2.68618 4.42225 2.77519 2.73123 3.46354 2.40513 3.72494
3.29354 2.67741 2.69355 4.24690 2.90347 2.73739 3.18146 2.89801
2.37887 2.77519 2.98518 4.58477 3.61503
0.01573 4.55604 5.27839 0.61958 0.77255 0.48576 0.95510
10 2.33431 4.40436 3.63935 3.07047 3.47917 3.70460 3.96258
2.68170 2.95754 2.27633 2.51893 3.46226 2.48021 3.10196 3.00858
2.78901 2.86681 2.49678 4.99025 3.76140 10 l - - -
2.68618 4.42225 2.77519 2.73123 3.46354 2.40513 3.72494
3.29354 2.67741 2.69355 4.24690 2.90347 2.73739 3.18146 2.89801
2.37887 2.77519 2.98518 4.58477 3.61503
0.01573 4.55604 5.27839 0.61958 0.77255 0.48576 0.95510
11 4.27385 5.48115 5.20167 5.08339 0.33412 4.70382 4.37974
3.77999 4.99190 3.00539 4.42556 4.87097 5.17668 4.97978 4.94063
4.47376 4.58981 3.81271 4.50631 2.82148 11 F - - -
2.68618 4.42225 2.77519 2.73123 3.46354 2.40513 3.72494
3.29354 2.67741 2.69355 4.24690 2.90347 2.73739 3.18146 2.89801
2.37887 2.77519 2.98518 4.58477 3.61503
0.01573 4.55604 5.27839 0.61958 0.77255 0.48576 0.95510
12 2.71456 4.30854 2.83123 2.01313 4.36362 3.51329 3.08529
3.40451 2.40973 3.18879 4.13197 2.82012 3.90529 2.70903 2.95050
2.46764 2.17788 3.14295 5.54425 4.17076 12 e - - -
2.68618 4.42225 2.77519 2.73123 3.46354 2.40513 3.72494
3.29354 2.67741 2.69355 4.24690 2.90347 2.73739 3.18146 2.89801
2.37887 2.77519 2.98518 4.58477 3.61503
0.01573 4.55604 5.27839 0.61958 0.77255 0.48576 0.95510
13 3.48651 4.71120 5.48996 5.03455 4.04849 5.02870 5.78600
1.43364 4.95805 2.48435 3.79684 5.19789 5.29080 5.22466 5.13332
4.46632 3.75575 0.68270 6.14632 4.92048 13 V - - -
2.68618 4.42225 2.77519 2.73123 3.46354 2.40513 3.72494
3.29354 2.67741 2.69355 4.24690 2.90347 2.73739 3.18146 2.89801
2.37887 2.77519 2.98518 4.58477 3.61503
0.01573 4.55604 5.27839 0.61958 0.77255 0.48576 0.95510
14 2.71704 3.50990 4.47184 3.87083 2.09482 3.86184 4.18091
2.52421 3.69936 2.17805 3.24428 3.74651 4.22786 3.86912 3.78094
3.01344 2.65711 1.81288 4.73886 2.49897 14 v - - -
2.68618 4.42225 2.77519 2.73123 3.46354 2.40513 3.72494
3.29354 2.67741 2.69355 4.24690 2.90347 2.73739 3.18146 2.89801
2.37887 2.77519 2.98518 4.58477 3.61503
0.01573 4.55604 5.27839 0.61958 0.77255 0.48576 0.95510
15 3.93588 4.26359 4.97321 4.63636 2.09948 4.69953 3.49695
3.76696 4.45888 3.15917 4.35539 4.39327 5.02092 4.44984 4.49934
4.05509 4.16177 3.63990 3.95727 0.54845 15 Y - - -
2.68618 4.42225 2.77519 2.73123 3.46354 2.40513 3.72494
3.29354 2.67741 2.69355 4.24690 2.90347 2.73739 3.18146 2.89801
2.37887 2.77519 2.98518 4.58477 3.61503
0.01573 4.55604 5.27839 0.61958 0.77255 0.48576 0.95510
16 2.78831 5.13232 3.06851 2.47726 4.42535 3.56050 3.56054
3.87155 1.92934 3.40613 4.19150 2.13451 3.95534 2.85769 2.70211
2.60928 3.01461 3.50073 5.57559 2.29869 16 k - - -

```
          2.68618  4.42225  2.77519  2.73123  3.46354  2.40513  3.72494
3.29354  2.67741  2.69355  4.24690  2.90347  2.73739  3.18146  2.89801
2.37887  2.77519  2.98518  4.58477  3.61503
          0.01573  4.55604  5.27839  0.61958  0.77255  0.48576  0.95510
     17   2.69049  4.82698  3.91727  3.82792  4.93872  3.54106  4.94209
4.38853  3.95116  4.07762  5.04724  3.97552  0.45845  4.28650  4.17954
3.12811  3.46377  3.87178  6.22142  5.08856     17 P - - -
          2.68618  4.42225  2.77519  2.73123  3.46354  2.40513  3.72494
3.29354  2.67741  2.69355  4.24690  2.90347  2.73739  3.18146  2.89801
2.37887  2.77519  2.98518  4.58477  3.61503
          0.05655  4.55604  3.11273  0.61958  0.77255  0.48576  0.95510
     18   2.77942  5.05177  3.25357  2.67280  4.34780  3.02228  3.77226
3.75557  2.29566  3.31799  4.13776  2.87512  4.02173  2.91462  1.33282
2.81329  3.07407  3.42595  4.07327  4.20832     18 r - - -
          2.68618  4.42225  2.77519  2.73123  3.46354  2.40513  3.72494
3.29354  2.67741  2.69355  4.24690  2.90347  2.73739  3.18146  2.89801
2.37887  2.77519  2.98518  4.58477  3.61503
          0.01638  4.51587  5.23821  0.61958  0.77255  0.55457  0.85406
     19   1.75814  4.78651  3.04623  2.51838  3.89399  3.56013  3.77432
3.25587  2.60832  2.93144  3.74194  3.12607  3.94914  2.95309  2.59922
2.72666  2.28458  2.99370  5.31339  4.00485     19 a - - -
          2.68618  4.42225  2.77519  2.73123  3.46354  2.40513  3.72494
3.29354  2.67741  2.69355  4.24690  2.90347  2.73739  3.18146  2.89801
2.37887  2.77519  2.98518  4.58477  3.61503
          0.06595  4.51587  2.93964  0.61958  0.77255  0.44502  1.02391
     20   2.66536  4.32810  2.97225  2.51414  4.32002  2.40901  3.74022
3.75852  2.44988  3.32332  4.11082  2.74469  3.90785  2.87020  2.74806
1.73878  2.77955  3.38802  5.52697  4.16914     20 s - - -
          2.68618  4.42225  2.77519  2.73123  3.46354  2.40513  3.72494
3.29354  2.67741  2.69355  4.24690  2.90347  2.73739  3.18146  2.89801
2.37887  2.77519  2.98518  4.58477  3.61503
          0.05409  4.50926  3.17858  0.61958  0.77255  0.56519  0.83991
     21   2.54795  4.75825  3.17799  2.61839  3.83256  3.55025  3.37434
3.36077  2.55143  3.00155  3.83957  3.11934  2.62471  2.82798  2.71801
2.27578  2.56047  2.77993  3.59036  3.98008     21 s - - -
          2.68618  4.42225  2.77520  2.73124  3.46354  2.40513  3.72495
3.29354  2.67741  2.69355  4.24690  2.90347  2.73740  3.18147  2.89801
2.37887  2.77520  2.98519  4.58477  3.61481
          0.05317  3.31320  4.17468  0.48026  0.96397  0.40940  1.09079
     22   2.31713  4.86800  3.14627  2.41243  3.94785  3.49594  3.84421
3.67044  2.65844  3.27738  4.08909  3.13421  2.83658  3.00086  3.11511
1.46891  2.84382  3.31674  5.51695  4.18801     23 s - - -
          2.68618  4.42225  2.77519  2.73123  3.46354  2.40513  3.72494
3.29354  2.67741  2.69355  4.24690  2.90347  2.73739  3.18146  2.89801
2.37887  2.77519  2.98518  4.58477  3.61503
          0.01587  4.54696  5.26930  0.61958  0.77255  0.47575  0.97133
     23   2.57402  5.37482  2.28168  1.43071  4.70325  3.40604  3.77665
4.19525  2.49427  3.67340  4.42996  2.90064  3.81699  2.39565  3.08409
2.53057  3.09036  3.75843  5.80985  4.37888     24 e - - -
          2.68618  4.42225  2.77519  2.73123  3.46354  2.40513  3.72494
3.29354  2.67741  2.69355  4.24690  2.90347  2.73739  3.18146  2.89801
2.37887  2.77519  2.98518  4.58477  3.61503
          0.01573  4.55604  5.27839  0.61958  0.77255  0.48576  0.95510
     24   4.32350  5.42537  5.26182  5.09027  0.46848  4.94762  3.86492
3.82839  4.90180  3.03089  4.39911  4.61614  5.24569  4.73015  4.81517
4.37679  4.54861  3.82312  3.96109  2.03180     25 F - - -
```

```
          2.68618  4.42225  2.77519  2.73123  3.46354  2.40513  3.72494
3.29354  2.67741  2.69355  4.24690  2.90347  2.73739  3.18146  2.89801
2.37887  2.77519  2.98518  4.58477  3.61503
          0.01573  4.55604  5.27839  0.61958  0.77255  0.48576  0.95510
     25   3.29969  2.64357  5.31067  4.80016  3.91048  4.73131  5.33903
1.24078  4.69200  2.52622  3.72837  4.90085  5.03711  4.91310  4.81579
4.11805  3.56063  1.03456  5.78299  4.57337     26 v - - -
          2.68618  4.42225  2.77519  2.73123  3.46354  2.40513  3.72494
3.29354  2.67741  2.69355  4.24690  2.90347  2.73739  3.18146  2.89801
2.37887  2.77519  2.98518  4.58477  3.61503
          0.01573  4.55604  5.27839  0.61958  0.77255  0.48576  0.95510
     26   3.49052  4.71094  5.50427  5.04832  4.05399  5.04450  5.80215
1.36109  4.97507  2.48722  3.79933  5.21197  5.30050  5.24053  5.14997
4.48236  3.75862  0.71561  6.15613  4.93094     27 v - - -
          2.68618  4.42225  2.77519  2.73123  3.46354  2.40513  3.72494
3.29354  2.67741  2.69355  4.24690  2.90347  2.73739  3.18146  2.89801
2.37887  2.77519  2.98518  4.58477  3.61503
          0.01573  4.55604  5.27839  0.61958  0.77255  0.48576  0.95510
     27   2.61825  5.17774  2.98948  2.32747  4.50008  3.29868  3.62634
3.96310  2.21070  3.47129  4.23152  2.83812  2.08818  2.81663  2.52165
2.25753  2.97696  3.55762  5.62537  4.23807     28 p - - -
          2.68618  4.42225  2.77519  2.73123  3.46354  2.40513  3.72494
3.29354  2.67741  2.69355  4.24690  2.90347  2.73739  3.18146  2.89801
2.37887  2.77519  2.98518  4.58477  3.61503
          0.01573  4.55604  5.27839  0.61958  0.77255  0.48576  0.95510
     28   2.20572  3.85956  4.20432  3.48854  2.85829  3.81889  4.12387
2.53916  3.49107  2.05383  3.29554  3.81162  4.18803  3.70634  3.00321
2.99818  2.75260  2.00424  4.79271  2.46619     29 v - - -
          2.68618  4.42225  2.77519  2.73123  3.46354  2.40513  3.72494
3.29354  2.67741  2.69355  4.24690  2.90347  2.73739  3.18146  2.89801
2.37887  2.77519  2.98518  4.58477  3.61503
          0.01573  4.55604  5.27839  0.61958  0.77255  0.48576  0.95510
     29   2.27211  4.82186  2.55492  2.19182  4.50971  3.42887  3.06111
3.98328  2.40544  3.48013  4.22799  2.37718  3.77297  2.63375  2.79395
2.42363  2.91763  3.56354  3.88472  4.22604     30 e - - -
          2.68618  4.42225  2.77519  2.73123  3.46354  2.40513  3.72494
3.29354  2.67741  2.69355  4.24690  2.90347  2.73739  3.18146  2.89801
2.37887  2.77519  2.98518  4.58477  3.61503
          0.02424  4.55604  4.30880  0.61958  0.77255  0.48576  0.95510
     30   2.49412  5.04223  2.93824  2.49171  4.32107  3.52159  3.71601
3.54349  1.59690  3.26292  4.10322  3.02378  3.81122  2.71794  2.81499
2.52803  2.67616  2.86746  5.51767  4.15481     31 k - - -
          2.68618  4.42225  2.77519  2.73123  3.46354  2.40513  3.72494
3.29354  2.67741  2.69355  4.24690  2.90347  2.73739  3.18146  2.89801
2.37887  2.77519  2.98518  4.58477  3.61503
          0.01586  4.54766  5.27000  0.61958  0.77255  0.47650  0.97009
     31   3.30586  4.65423  4.78614  4.30618  1.84361  4.32272  3.72064
3.07878  4.13845  2.69786  3.75448  4.22966  4.66144  4.21346  4.19539
3.64542  3.53373  1.55340  4.20315  1.39482     32 y - - -
          2.68618  4.42225  2.77519  2.73123  3.46354  2.40513  3.72494
3.29354  2.67741  2.69355  4.24690  2.90347  2.73739  3.18146  2.89801
2.37887  2.77519  2.98518  4.58477  3.61503
          0.01573  4.55604  5.27839  0.61958  0.77255  0.48576  0.95510
     32   2.71379  4.84683  2.61937  2.33779  4.06668  3.56101  3.49330
3.06165  2.53526  2.31488  3.68133  2.90554  3.94976  2.86842  2.86905
2.64893  2.78014  2.51170  5.36265  3.71260     33 l - - -
```

```
          2.68618  4.42225  2.77519  2.73123  3.46354  2.40513  3.72494
3.29354  2.67741  2.69355  4.24690  2.90347  2.73739  3.18146  2.89801
2.37887  2.77519  2.98518  4.58477  3.61503
          0.01573  4.55604  5.27839  0.61958  0.77255  0.48576  0.95510
     33   2.29662  4.84519  2.67837  1.98173  4.51003  3.33411  3.68491
3.98346  1.85483  3.48031  3.74415  2.85911  3.88898  2.78738  2.79212
2.58378  2.88469  3.39605  5.62612  4.22639     34 k - - -
          2.68618  4.42225  2.77519  2.73123  3.46354  2.40513  3.72494
3.29354  2.67741  2.69355  4.24690  2.90347  2.73739  3.18146  2.89801
2.37887  2.77519  2.98518  4.58477  3.61503
          0.01573  4.55604  5.27839  0.61958  0.77255  0.48576  0.95510
     34   0.97742  4.22685  3.84600  3.39439  4.46468  3.18502  4.41117
3.87815  3.35847  3.54831  4.37005  3.45872  4.01152  3.66340  3.46759
1.73229  2.77801  3.39208  5.80884  4.58815     35 a - - -
          2.68618  4.42225  2.77519  2.73123  3.46354  2.40513  3.72494
3.29354  2.67741  2.69355  4.24690  2.90347  2.73739  3.18146  2.89801
2.37887  2.77519  2.98518  4.58477  3.61503
          0.01573  4.55604  5.27839  0.61958  0.77255  0.48576  0.95510
     35   2.37632  4.14636  4.36552  3.76954  2.77259  3.84741  4.16330
2.24358  3.14074  1.91027  2.38052  3.89983  4.21403  3.80679  3.51496
3.06356  2.76408  2.01680  4.76414  3.57964     36 l - - -
          2.68618  4.42225  2.77519  2.73123  3.46354  2.40513  3.72494
3.29354  2.67741  2.69355  4.24690  2.90347  2.73739  3.18146  2.89801
2.37887  2.77519  2.98518  4.58477  3.61503
          0.01573  4.55604  5.27839  0.61958  0.77255  0.48576  0.95510
     36   2.61008  5.19300  2.84253  2.14867  4.52403  3.38462  3.68345
4.00001  2.13331  3.49206  4.11361  2.15366  3.88812  2.67079  2.68331
2.42197  2.89226  3.44186  5.63389  3.25124     37 k - - -
          2.68618  4.42225  2.77519  2.73123  3.46354  2.40513  3.72494
3.29354  2.67741  2.69355  4.24690  2.90347  2.73739  3.18146  2.89801
2.37887  2.77519  2.98518  4.58477  3.61503
          0.01573  4.55604  5.27839  0.61958  0.77255  0.48576  0.95510
     37   2.68248  4.47207  3.30490  2.74362  3.60089  3.45660  2.80007
2.90224  2.68328  2.74185  3.19939  2.52570  3.78377  2.79259  2.80750
2.69077  2.76501  2.45212  5.21272  3.71703     38 v - - -
          2.68620  4.42227  2.77521  2.73125  3.46356  2.40515  3.72490
3.29356  2.67743  2.69357  4.24664  2.90338  2.73738  3.18148  2.89798
2.37889  2.77515  2.98520  4.58479  3.61505
          0.07736  2.66869  5.27839  0.44030  1.03238  0.48576  0.95510
     38   2.65787  4.69021  2.66506  2.36604  4.09533  3.25730  3.51297
4.02103  2.08437  3.50625  4.24766  2.63889  2.96908  2.37582  2.56240
2.39377  2.94596  3.59071  5.64251  4.02381     41 k - - -
          2.68618  4.42225  2.77519  2.73123  3.46354  2.40513  3.72494
3.29354  2.67741  2.69355  4.24690  2.90347  2.73739  3.18146  2.89801
2.37887  2.77519  2.98518  4.58477  3.61503
          0.01573  4.55604  5.27839  0.61958  0.77255  0.48576  0.95510
     39   2.71962  3.83265  4.57278  3.96597  2.26263  3.87601  3.99837
2.27821  3.77428  2.19426  3.11963  4.00029  4.06614  3.92447  3.82027
3.18275  2.88713  2.05148  2.23299  3.01817     42 v - - -
          2.68618  4.42225  2.77519  2.73123  3.46354  2.40513  3.72494
3.29354  2.67741  2.69355  4.24690  2.90347  2.73739  3.18146  2.89801
2.37887  2.77519  2.98518  4.58477  3.61503
          0.01573  4.55604  5.27839  0.61958  0.77255  0.48576  0.95510
     40   2.68054  3.16861  3.32751  2.54335  3.82352  3.61354  3.84622
3.07433  2.74428  2.73838  3.73990  2.90837  3.68451  3.07501  3.16266
1.79905  2.52472  2.73939  5.20009  3.81789     43 s - - -
```

```
         2.68618  4.42225  2.77519  2.73123  3.46354  2.40513  3.72494
3.29354  2.67741  2.69355  4.24690  2.90347  2.73739  3.18146  2.89801
2.37887  2.77519  2.98518  4.58477  3.61503
         0.01573  4.55604  5.27839  0.61958  0.77255  0.48576  0.95510
     41  2.47983  4.24857  4.00214  3.42318  3.36396  3.66194  4.08157
2.10859  3.32889  2.30307  3.28235  3.69814  2.90144  3.57813  3.35917
2.77964  2.70317  1.74739  4.86058  3.65951      44 v - - -
         2.68618  4.42225  2.77519  2.73123  3.46354  2.40513  3.72494
3.29354  2.67741  2.69355  4.24690  2.90347  2.73739  3.18146  2.89801
2.37887  2.77519  2.98518  4.58477  3.61503
         0.01573  4.55604  5.27839  0.61958  0.77255  0.48576  0.95510
     42  2.99958  5.17851  2.21408  2.72145  5.07525  0.76906  4.33490
4.60374  3.41721  4.17914  5.02738  3.22198  4.15021  3.54332  3.95043
2.82729  3.42226  4.05325  6.32574  4.92412      45 g - - -
         2.68618  4.42225  2.77519  2.73123  3.46354  2.40513  3.72494
3.29354  2.67741  2.69355  4.24690  2.90347  2.73739  3.18146  2.89801
2.37887  2.77519  2.98518  4.58477  3.61503
         0.01573  4.55604  5.27839  0.61958  0.77255  0.48576  0.95510
     43  2.77605  4.31286  4.10030  3.53069  3.43707  3.83651  4.19669
2.53416  3.42249  2.37956  1.46654  3.79241  4.23251  3.68120  3.65683
2.71073  2.41689  2.29421  4.97318  3.77625      46 m - - -
         2.68618  4.42225  2.77519  2.73123  3.46354  2.40513  3.72494
3.29354  2.67741  2.69355  4.24690  2.90347  2.73739  3.18146  2.89801
2.37887  2.77519  2.98518  4.58477  3.61503
         0.01573  4.55604  5.27839  0.61958  0.77255  0.48576  0.95510
     44  3.17549  5.32167  3.61634  2.97677  4.80042  3.84232  3.87163
4.15362  2.06140  3.62515  4.47207  3.39888  3.24398  3.00520  0.92794
3.18348  3.02050  3.81366  5.70537  4.50706      47 r - - -
         2.68618  4.42225  2.77519  2.73123  3.46354  2.40513  3.72494
3.29354  2.67741  2.69355  4.24690  2.90347  2.73739  3.18146  2.89801
2.37887  2.77519  2.98518  4.58477  3.61503
         0.01573  4.55604  5.27839  0.61958  0.77255  0.48576  0.95510
     45  3.37982  4.69449  5.13549  4.60763  1.11298  4.61631  4.78546
2.14748  4.46340  2.05213  3.37891  4.68966  4.89458  4.56355  4.53618
3.97144  3.62056  1.66600  5.08235  3.70342      48 f - - -
         2.68618  4.42225  2.77519  2.73123  3.46354  2.40513  3.72494
3.29354  2.67741  2.69355  4.24690  2.90347  2.73739  3.18146  2.89801
2.37887  2.77519  2.98518  4.58477  3.61503
         0.01573  4.55604  5.27839  0.61958  0.77255  0.48576  0.95510
     46  2.89834  5.29166  3.28667  2.67955  4.67423  3.29272  3.76395
4.08826  1.32273  3.40371  4.35438  3.11656  4.06302  2.78596  1.84606
2.85487  2.88842  3.70936  5.66332  4.36601      49 k - - -
         2.68618  4.42225  2.77519  2.73123  3.46354  2.40513  3.72494
3.29354  2.67741  2.69355  4.24690  2.90347  2.73739  3.18146  2.89801
2.37887  2.77519  2.98518  4.58477  3.61503
         0.01573  4.55604  5.27839  0.61958  0.77255  0.48576  0.95510
     47  3.18238  4.79166  5.03931  4.46874  3.18264  4.65947  4.96839
2.32715  4.32355  1.62096  0.96311  4.69214  4.88767  4.44405  4.43924
3.99597  3.66574  2.51839  5.33329  4.23490      50 m - - -
         2.68618  4.42225  2.77519  2.73123  3.46354  2.40513  3.72494
3.29354  2.67741  2.69355  4.24690  2.90347  2.73739  3.18146  2.89801
2.37887  2.77519  2.98518  4.58477  3.61503
         0.06338  4.55604  2.97768  0.61958  0.77255  0.48576  0.95510
     48  2.19694  4.60123  3.08831  2.49793  4.17522  3.52755  3.59393
3.46897  2.30463  2.96583  3.43025  3.05174  3.74317  2.68708  2.11990
2.56421  2.68196  3.26532  5.42494  4.08481      51 r - - -
```

```
          2.68618  4.42225  2.77519  2.73123  3.46354  2.40513  3.72494
3.29354  2.67741  2.69355  4.24690  2.90347  2.73739  3.18146  2.89801
2.37887  2.77519  2.98518  4.58477  3.61503
          0.01649  4.50915  5.23150  0.61958  0.77255  0.43902  1.03471
     49   2.70729  4.19213  4.52382  3.92734  1.52602  3.93184  3.91108
2.48032  3.75830  2.02969  2.87822  4.01208  4.28879  3.92064  3.84063
3.23529  3.01846  2.09289  4.74434  3.15668     52 f - - -
          2.68618  4.42225  2.77519  2.73123  3.46354  2.40513  3.72494
3.29354  2.67741  2.69355  4.24690  2.90347  2.73739  3.18146  2.89801
2.37887  2.77519  2.98518  4.58477  3.61503
          0.01573  4.55604  5.27839  0.61958  0.77255  0.48576  0.95510
     50   3.21074  5.76087  2.33077  0.76961  5.09122  3.52971  4.05510
4.61083  3.03657  4.10024  4.93732  3.00267  3.12742  3.21500  3.61201
3.10546  3.49807  4.17300  6.24697  4.75713     53 e - - -
          2.68618  4.42225  2.77519  2.73123  3.46354  2.40513  3.72494
3.29354  2.67741  2.69355  4.24690  2.90347  2.73739  3.18146  2.89801
2.37887  2.77519  2.98518  4.58477  3.61503
          0.01573  4.55604  5.27839  0.61958  0.77255  0.48576  0.95510
     51   2.64364  4.28014  3.35887  2.80134  3.86097  2.18603  3.87740
3.23926  2.53604  2.86890  3.38033  3.26192  4.00334  3.10642  3.18723
2.58719  1.86760  2.82052  5.23527  3.96184     54 t - - -
          2.68618  4.42225  2.77519  2.73123  3.46354  2.40513  3.72494
3.29354  2.67741  2.69355  4.24690  2.90347  2.73739  3.18146  2.89801
2.37887  2.77519  2.98518  4.58477  3.61503
          0.01573  4.55604  5.27839  0.61958  0.77255  0.48576  0.95510
     52   2.93098  5.45409  1.90711  1.26209  4.76690  3.50848  3.56039
4.25905  2.49798  3.74541  4.52003  2.96948  3.99805  2.96024  3.18698
2.86655  3.17902  3.28896  5.89071  4.45137     55 e - - -
          2.68618  4.42225  2.77519  2.73123  3.46354  2.40513  3.72494
3.29354  2.67741  2.69355  4.24690  2.90347  2.73739  3.18146  2.89801
2.37887  2.77519  2.98518  4.58477  3.61503
          0.05155  4.55604  3.22536  0.61958  0.77255  0.48576  0.95510
     53   3.20207  5.83176  1.10805  1.43545  5.10597  3.33787  4.01538
4.64647  3.02117  4.11728  4.94980  2.94438  4.10643  3.17256  3.62646
3.07875  3.48400  4.19999  6.26338  4.15757     56 d - - -
          2.68618  4.42225  2.77519  2.73123  3.46354  2.40513  3.72494
3.29354  2.67741  2.69355  4.24690  2.90347  2.73739  3.18146  2.89801
2.37887  2.77519  2.98518  4.58477  3.61503
          0.01630  4.52079  5.24313  0.61958  0.77255  0.54654  0.86500
     54   2.18317  3.73693  3.88852  3.32436  3.06628  3.69540  4.08399
2.78443  3.24968  2.44902  3.46807  3.62389  4.11931  3.51733  3.52890
1.64591  2.79835  2.16666  4.96434  3.75500     57 s - - -
          2.68618  4.42225  2.77519  2.73123  3.46354  2.40513  3.72494
3.29354  2.67741  2.69355  4.24690  2.90347  2.73739  3.18146  2.89801
2.37887  2.77519  2.98518  4.58477  3.61503
          0.01630  4.52079  5.24313  0.61958  0.77255  0.54654  0.86500
     55   2.27884  4.83445  3.16344  2.61886  4.15315  3.07178  3.79981
3.56703  2.61447  2.98642  3.99815  2.80349  2.65032  2.90100  3.06936
1.86478  2.89831  2.87707  5.43220  4.10910     58 s - - -
          2.68618  4.42225  2.77519  2.73123  3.46354  2.40513  3.72494
3.29354  2.67741  2.69355  4.24690  2.90347  2.73739  3.18146  2.89801
2.37887  2.77519  2.98518  4.58477  3.61503
          0.01630  4.52079  5.24313  0.61958  0.77255  0.54654  0.86500
     56   2.72384  5.16032  2.92205  1.67679  4.48171  3.50192  3.21421
3.57858  2.29692  3.45141  4.20911  2.98091  3.89503  2.74519  2.17355
2.69930  2.83313  3.28239  5.60344  4.21624     59 e - - -
```

```
          2.68621  4.42228  2.77517  2.73126  3.46357  2.40516  3.72497
3.29357  2.67744  2.69358  4.24693  2.90343  2.73743  3.18120  2.89804
2.37890  2.77523  2.98497  4.58480  3.61506
          0.09515  2.62749  3.98984  0.65725  0.73039  0.44955  1.01588
     57   2.76876  4.93938  3.19467  2.56639  4.18583  3.59165  3.76376
2.67857  2.16341  3.18988  3.33599  3.12626  3.97579  2.47595  1.83966
2.80694  2.86432  3.16507  5.42544  4.11206     62 r - - -
          2.68619  4.42226  2.77520  2.73124  3.46355  2.40514  3.72495
3.29355  2.67742  2.69356  4.24690  2.90347  2.73740  3.18147  2.89801
2.37882  2.77520  2.98510  4.58478  3.61504
          0.08382  3.54618  2.96484  0.63734  0.75226  0.47175  0.97792
     58   2.67137  4.92495  3.17917  2.62285  4.25551  3.16055  3.78310
3.39320  2.46786  3.25857  4.07104  3.12164  3.95790  2.93107  1.57841
2.26408  2.60706  3.32723  5.48041  4.16132     65 r - - -
          2.68618  4.42225  2.77519  2.73123  3.46354  2.40513  3.72494
3.29354  2.67741  2.69355  4.24690  2.90347  2.73739  3.18146  2.89801
2.37887  2.77519  2.98518  4.58477  3.61503
          0.03679  4.50915  3.68422  0.61958  0.77255  0.56537  0.83967
     59   2.79887  3.07344  4.49264  3.91316  2.24982  3.92008  3.52730
2.57740  3.74544  2.20920  3.32167  3.97057  4.28140  3.89932  3.82703
2.89147  3.03007  2.46170  2.53572  2.13238     66 y - - -
          2.68623  4.42203  2.77525  2.73129  3.46239  2.40518  3.72500
3.29359  2.67746  2.69360  4.24695  2.90352  2.73745  3.18152  2.89806
2.37890  2.77525  2.98506  4.58482  3.61492
          0.28156  1.42735  5.21153  0.16935  1.85929  0.42232  1.06574
     60   2.59126  4.96054  3.03540  2.43008  4.03098  3.53609  3.73940
3.63691  2.52827  3.22591  2.67829  2.81914  3.80401  2.34777  2.99682
2.17099  2.32557  3.30054  5.45558  4.11020     68 s - - -
          2.68618  4.42225  2.77519  2.73123  3.46354  2.40513  3.72494
3.29354  2.67741  2.69355  4.24690  2.90347  2.73739  3.18146  2.89801
2.37887  2.77519  2.98518  4.58477  3.61503
          0.01573  4.55604  5.27839  0.61958  0.77255  0.48576  0.95510
     61   3.72031  5.37555  4.52375  4.52573  5.55802  0.17442  5.54597
5.37810  4.79263  4.91665  5.93355  4.69146  4.77807  5.06393  4.90345
3.91850  4.24893  4.77525  6.42132  5.69974     69 G - - -
          2.68618  4.42225  2.77519  2.73123  3.46354  2.40513  3.72494
3.29354  2.67741  2.69355  4.24690  2.90347  2.73739  3.18146  2.89801
2.37887  2.77519  2.98518  4.58477  3.61503
          0.01573  4.55604  5.27839  0.61958  0.77255  0.48576  0.95510
     62   2.71811  4.43736  3.62006  3.05397  3.59062  3.69240  3.97018
2.73889  2.99462  2.40604  3.42902  3.37981  4.08282  3.30442  3.18310
2.45936  1.52180  2.55177  5.03854  3.80569     70 t - - -
          2.68618  4.42225  2.77519  2.73123  3.46354  2.40513  3.72494
3.29354  2.67741  2.69355  4.24690  2.90347  2.73739  3.18146  2.89801
2.37887  2.77519  2.98518  4.58477  3.61503
          0.01573  4.55604  5.27839  0.61958  0.77255  0.48576  0.95510
     63   2.81151  4.62734  5.33381  4.83389  3.96752  4.85944  5.48128
0.95008  4.74565  2.38325  3.74969  4.98885  5.13476  4.98803  4.90588
4.25350  3.63602  1.21688  5.92197  4.71273     71 i - - -
          2.68618  4.42225  2.77519  2.73123  3.46354  2.40513  3.72494
3.29354  2.67741  2.69355  4.24690  2.90347  2.73739  3.18146  2.89801
2.37887  2.77519  2.98518  4.58477  3.61503
          0.01573  4.55604  5.27839  0.61958  0.77255  0.48576  0.95510
     64   2.45674  4.30333  4.00373  3.43343  3.45656  3.80583  4.14368
2.24898  3.34890  2.29389  3.44050  3.72216  4.19714  3.60877  3.60683
2.07841  2.23513  1.88940  4.96314  3.75759     72 v - - -
```

2.68618 4.42225 2.77519 2.73123 3.46354 2.40513 3.72494
3.29354 2.67741 2.69355 4.24690 2.90347 2.73739 3.18146 2.89801
2.37887 2.77519 2.98518 4.58477 3.61503
0.01573 4.55604 5.27839 0.61958 0.77255 0.48576 0.95510
65 2.39283 4.63065 3.47659 3.23225 4.77107 1.00542 4.44951
4.21851 3.11458 3.85409 4.65983 3.51142 4.04117 3.67634 3.75000
1.74571 3.09632 3.64188 6.05975 4.81167 73 g - - -
2.68618 4.42225 2.77519 2.73123 3.46354 2.40513 3.72494
3.29354 2.67741 2.69355 4.24690 2.90347 2.73739 3.18146 2.89801
2.37887 2.77519 2.98518 4.58477 3.61503
0.01573 4.55604 5.27839 0.61958 0.77255 0.48576 0.95510
66 2.48146 4.17963 4.24575 3.52616 3.28505 3.83482 3.87529
1.95411 3.52578 2.28392 2.90780 3.67933 4.20341 3.73776 3.68670
3.00448 2.62772 1.59065 4.79938 3.60977 74 v - - -
2.68618 4.42225 2.77519 2.73123 3.46354 2.40513 3.72494
3.29354 2.67741 2.69355 4.24690 2.90347 2.73739 3.18146 2.89801
2.37887 2.77519 2.98518 4.58477 3.61503
0.01573 4.55604 5.27839 0.61958 0.77255 0.48576 0.95510
67 2.65684 4.52327 2.99581 2.18936 4.34453 2.40002 3.63154
3.15914 2.47615 3.19497 4.11918 2.75266 3.90798 2.56639 2.80442
2.19797 2.89904 3.41836 5.53324 3.96056 75 e - - -
2.68618 4.42225 2.77519 2.73123 3.46354 2.40513 3.72494
3.29354 2.67741 2.69355 4.24690 2.90347 2.73739 3.18146 2.89801
2.37887 2.77519 2.98518 4.58477 3.61503
0.01573 4.55604 5.27839 0.61958 0.77255 0.48576 0.95510
68 2.40389 5.02035 1.85028 2.35424 4.28930 3.52439 3.72515
3.72411 2.50153 2.91944 3.67729 2.94722 3.66824 2.79509 2.97705
2.61278 2.84850 2.86537 5.50280 4.14322 76 d - - -
2.68618 4.42230 2.77525 2.73129 3.46359 2.40505 3.72500
3.29359 2.67746 2.69360 4.24672 2.90352 2.73735 3.18152 2.89784
2.37892 2.77496 2.98524 4.58482 3.61508
0.25418 2.52095 1.93749 0.90793 0.51644 0.48576 0.95510
69 2.26316 4.21997 3.86251 3.28548 3.33398 3.71390 3.76329
2.22726 3.15714 2.06144 2.86497 3.06251 3.65955 3.46220 3.46448
2.86288 2.76357 2.20665 4.82205 3.45711 80 l - - -
2.68618 4.42225 2.77519 2.73123 3.46354 2.40513 3.72494
3.29354 2.67741 2.69355 4.24690 2.90347 2.73739 3.18146 2.89801
2.37887 2.77519 2.98518 4.58477 3.61503
0.01829 4.40642 5.12877 0.61958 0.77255 0.40400 1.10154
70 2.70902 4.47317 1.71356 2.45538 3.53795 3.03305 3.74177
3.34449 2.50755 3.06851 3.86944 3.02430 3.69888 2.88964 3.00490
2.60267 2.94062 3.11860 5.42150 4.08383 81 d - - -
2.68618 4.42225 2.77519 2.73123 3.46354 2.40513 3.72494
3.29354 2.67741 2.69355 4.24690 2.90347 2.73739 3.18146 2.89801
2.37887 2.77519 2.98518 4.58477 3.61503
0.01600 4.53868 5.26103 0.61958 0.77255 0.46706 0.98576
71 2.42092 4.42871 3.17123 2.61707 4.11632 3.54258 3.65030
3.52611 2.54582 3.14473 3.96653 2.97335 2.08598 2.95312 2.93655
2.06711 2.72954 3.00637 5.40278 4.08206 82 s - - -
2.68618 4.42225 2.77519 2.73123 3.46354 2.40513 3.72494
3.29354 2.67741 2.69355 4.24690 2.90347 2.73739 3.18146 2.89801
2.37887 2.77519 2.98518 4.58477 3.61503
0.02169 4.55604 4.51408 0.61958 0.77255 0.48576 0.95510
72 2.52220 4.43863 3.18153 2.94169 3.49026 3.02673 3.93916
2.33196 2.96478 2.54801 3.45902 3.41807 3.21975 3.26779 3.32184
2.49039 2.84637 2.03519 5.01982 3.45182 83 v - - -

```
         2.68618  4.42225  2.77519  2.73123  3.46354  2.40513  3.72494
3.29354  2.67741  2.69355  4.24690  2.90347  2.73739  3.18146  2.89801
2.37887  2.77519  2.98518  4.58477  3.61503
         0.01582  4.55017  5.27252  0.61958  0.77255  0.47923  0.96563
     73  2.67358  4.99034  3.09445  2.53361  4.25035  3.41271  3.18634
3.57922  2.49964  3.03973  3.95008  3.00906  3.38799  2.47548  1.74613
2.74523  2.95829  2.98139  5.47456  3.85696     84 r - - -
         2.68618  4.42225  2.77519  2.73123  3.46354  2.40513  3.72494
3.29354  2.67741  2.69355  4.24690  2.90347  2.73739  3.18146  2.89801
2.37887  2.77519  2.98518  4.58477  3.61503
         0.01573  4.55604  5.27839  0.61958  0.77255  0.48576  0.95510
     74  3.73542  5.26134  4.60162  4.32580  3.58713  4.21505  4.65289
4.16219  4.07218  3.63317  4.75954  4.48704  2.90592  4.49255  4.22161
3.93956  4.09239  3.95410  0.42561  3.59109     85 W - - -
         2.68618  4.42225  2.77519  2.73123  3.46354  2.40513  3.72494
3.29354  2.67741  2.69355  4.24690  2.90347  2.73739  3.18146  2.89801
2.37887  2.77519  2.98518  4.58477  3.61503
         0.01573  4.55604  5.27839  0.61958  0.77255  0.48576  0.95510
     75  2.49379  5.00038  3.08668  2.52948  4.27805  3.11361  3.73761
3.65201  2.24830  3.23037  3.95320  3.00399  1.93582  2.87119  2.58255
2.64728  2.86925  3.23671  5.49449  4.14485     86 p - - -
         2.68618  4.42225  2.77519  2.73123  3.46354  2.40513  3.72494
3.29354  2.67741  2.69355  4.24690  2.90347  2.73739  3.18146  2.89801
2.37887  2.77519  2.98518  4.58477  3.61503
         0.01573  4.55604  5.27839  0.61958  0.77255  0.48576  0.95510
     76  2.49994  4.58967  2.48320  2.39505  4.48760  2.19218  3.59351
3.95481  2.40082  3.35663  4.22036  2.19487  3.89936  2.77777  2.78973
2.67106  2.95949  3.54624  5.62051  4.22580     87 g - - -
         2.68618  4.42225  2.77519  2.73123  3.46354  2.40513  3.72494
3.29354  2.67741  2.69355  4.24690  2.90347  2.73739  3.18146  2.89801
2.37887  2.77519  2.98518  4.58477  3.61503
         0.01573  4.55604  5.27839  0.61958  0.77255  0.48576  0.95510
     77  2.56452  4.50342  3.93963  3.74422  4.84973  3.26561  4.78298
4.27566  3.79969  3.99514  4.82419  3.77135  4.06639  4.08487  4.05235
0.57746  2.51386  3.63635  6.19525  4.99997     88 S - - -
         2.68618  4.42225  2.77519  2.73123  3.46354  2.40513  3.72494
3.29354  2.67741  2.69355  4.24690  2.90347  2.73739  3.18146  2.89801
2.37887  2.77519  2.98518  4.58477  3.61503
         0.01573  4.55604  5.27839  0.61958  0.77255  0.48576  0.95510
     78  2.72623  5.20709  2.83173  2.05506  4.54147  3.49806  3.45262
4.01817  1.91266  3.19846  4.25150  2.88929  2.51254  2.66528  2.79816
2.69328  2.68154  3.59217  5.64446  4.24250     89 k - - -
         2.68618  4.42225  2.77519  2.73123  3.46354  2.40513  3.72494
3.29354  2.67741  2.69355  4.24690  2.90347  2.73739  3.18146  2.89801
2.37887  2.77519  2.98518  4.58477  3.61503
         0.01573  4.55604  5.27839  0.61958  0.77255  0.48576  0.95510
     79  4.67920  5.76259  5.25116  5.15351  3.68382  4.60978  4.98055
4.81483  4.91804  4.06787  5.40128  5.19925  5.18686  5.26073  4.90695
4.87914  5.02407  4.71528  0.19240  3.65967     90 W - - -
         2.68618  4.42225  2.77519  2.73123  3.46354  2.40513  3.72494
3.29354  2.67741  2.69355  4.24690  2.90347  2.73739  3.18146  2.89801
2.37887  2.77519  2.98518  4.58477  3.61503
         0.01573  4.55604  5.27839  0.61958  0.77255  0.48576  0.95510
     80  3.35209  5.52301  3.71028  2.85353  5.01697  3.54429  3.64807
4.34319  1.84042  3.74318  4.59895  3.44811  4.30947  2.98633  0.87046
3.32571  3.50366  4.01063  5.75983  4.59953     91 r - - -
```

```
          2.68618  4.42225  2.77519  2.73123  3.46354  2.40513  3.72494
3.29354  2.67741  2.69355  4.24690  2.90347  2.73739  3.18146  2.89801
2.37887  2.77519  2.98518  4.58477  3.61503
          0.02496  4.55604  4.25844  0.61958  0.77255  0.48576  0.95510
     81   2.70890  2.61292  3.95689  3.37855  3.09412  3.77168  4.06008
2.64220  3.28966  2.14685  2.73751  3.04552  4.14543  3.41080  3.53842
2.03713  2.84912  2.47884  4.85894  3.65573     92 s - - -
          2.68618  4.42225  2.77519  2.73123  3.46354  2.40513  3.72494
3.29354  2.67741  2.69355  4.24690  2.90347  2.73739  3.18146  2.89801
2.37887  2.77519  2.98518  4.58477  3.61503
          0.01587  4.54696  5.26930  0.61958  0.77255  0.50199  0.92969
     82   3.90269  5.14791  5.59508  5.06340  3.08108  5.20236  5.40791
2.43311  4.89436  0.46591  3.07982  5.29180  5.26916  4.85714  4.91193
4.60946  4.11852  2.65456  5.45203  4.33316     93 L - - -
          2.68618  4.42225  2.77519  2.73123  3.46354  2.40513  3.72494
3.29354  2.67741  2.69355  4.24690  2.90347  2.73739  3.18146  2.89801
2.37887  2.77519  2.98518  4.58477  3.61503
          0.19760  4.54696  1.77963  0.61958  0.77255  0.47575  0.97133
     83   2.96588  5.34093  3.07741  2.21881  4.73699  3.61809  3.72595
4.15556  1.58893  3.60131  4.40159  3.09272  4.02468  1.66551  2.49092
2.92488  3.17192  3.76681  5.68250  4.38034     94 k - - -
          2.68618  4.42225  2.77519  2.73123  3.46354  2.40513  3.72494
3.29354  2.67741  2.69355  4.24690  2.90347  2.73739  3.18146  2.89801
2.37887  2.77519  2.98518  4.58477  3.61503
          0.01269  4.37311        *  0.61958  0.77255  0.00000        *
//
HMMER3/f [3.3 | Nov 2019]
NAME  AUX_IAA
ACC   PF02309.17
DESC  AUX/IAA family
LENG  240
ALPH  amino
RF    no
MM    no
CONS  yes
CS    yes
MAP   yes
DATE  Tue Apr 21 16:44:17 2020
NSEQ  100
EFFN  1.321411
CKSUM 1336242258
GA    22.70 22.70
TC    22.70 22.70
NC    22.60 22.60
STATS LOCAL MSV      -10.8426  0.70332
STATS LOCAL VITERBI  -11.9629  0.70332
STATS LOCAL FORWARD   -4.7160  0.70332
HMM          A        C        D        E        F        G        H
I        K        L        M        N        P        Q        R        S
T        V        W        Y
            m->m     m->i     m->d     i->m     i->i     d->m     d->d
  COMPO   2.57741  4.50744  2.83843  2.60304  3.47233  2.80346  3.79554
3.09717  2.53267  2.54720  3.63252  3.04115  3.14288  3.04933  2.83574
2.55294  2.85574  2.73810  4.43066  3.57629
          2.68618  4.42225  2.77519  2.73123  3.46354  2.40513  3.72494
3.29354  2.67741  2.69355  4.24690  2.90347  2.73739  3.18146  2.89801
2.37887  2.77519  2.98518  4.58477  3.61503
          0.16718  4.35126  1.95856  0.61958  0.77255  0.00000        *
```

```
      1   2.23795  5.12471  2.64994  1.65083  4.43426  3.22604  3.65427
3.89621  2.30941  3.41673  4.19394  2.80922  3.83648  2.77580  2.78397
2.67269  2.88730  3.49971  5.58472  4.18929      1 e - - X
          2.68618  4.42225  2.77519  2.73123  3.46354  2.40513  3.72494
3.29354  2.67741  2.69355  4.24690  2.90347  2.73739  3.18146  2.89801
2.37887  2.77519  2.98518  4.58477  3.61503
          0.02238  4.20646  4.92881  0.61958  0.77255  0.59140  0.80644
      2   2.72554  4.46019  3.82366  3.38871  3.70588  3.58385  4.28303
2.73018  3.23595  2.03483  3.67538  3.66438  4.14422  3.62012  3.51238
2.98208  1.23112  2.55547  5.31133  4.05806      3 t - - -
          2.68618  4.42225  2.77519  2.73123  3.46354  2.40513  3.72494
3.29354  2.67741  2.69355  4.24690  2.90347  2.73739  3.18146  2.89801
2.37887  2.77519  2.98518  4.58477  3.61503
          0.02156  4.24364  4.96599  0.61958  0.77255  0.48685  0.95337
      3   3.13873  5.59520  2.36265  0.81766  4.89526  3.42610  3.96153
4.41537  2.89951  3.93391  4.81564  2.92051  4.04588  3.02407  3.40826
3.04224  3.43073  4.01518  6.06883  4.61262      4 e - - -
          2.68618  4.42226  2.77520  2.73124  3.46354  2.40511  3.72495
3.29355  2.67741  2.69350  4.24690  2.90347  2.73740  3.18147  2.89798
2.37887  2.77520  2.98519  4.58477  3.61504
          0.02866  3.82903  5.03301  0.72850  0.65900  0.48130  0.96229
      4   3.72569  5.06372  4.93825  4.60425  3.38661  4.57009  5.11931
2.63634  4.37087  0.45932  3.30548  4.83767  4.94046  4.62200  4.46063
4.23581  4.00646  2.78986  5.40745  4.23057      9 L - - -
          2.68618  4.42225  2.77519  2.73123  3.46354  2.40513  3.72494
3.29354  2.67741  2.69355  4.24690  2.90347  2.73739  3.18146  2.89801
2.37887  2.77519  2.98518  4.58477  3.61503
          0.01960  4.33784  5.06019  0.61958  0.77255  0.50522  0.92477
      5   2.84591  4.32062  3.22182  2.45785  4.41893  3.60068  3.69705
3.81948  2.09530  3.34666  4.16087  3.11209  3.97997  2.69963  1.40038
2.74693  2.95001  3.47652  5.49893  4.21228     10 r - - -
          2.68618  4.42225  2.77519  2.73123  3.46354  2.40513  3.72494
3.29354  2.67741  2.69355  4.24690  2.90347  2.73739  3.18146  2.89801
2.37887  2.77519  2.98518  4.58477  3.61503
          0.01960  4.33784  5.06019  0.61958  0.77255  0.47379  0.97455
      6   3.65015  4.97015  5.14265  4.67010  3.22313  4.81529  5.15402
2.29312  4.41869  0.56191  3.06590  4.90843  5.02399  4.58525  4.51033
4.25773  3.89538  2.53411  5.36420  4.21648     11 L - - S
          2.68618  4.42225  2.77519  2.73123  3.46354  2.40513  3.72494
3.29354  2.67741  2.69355  4.24690  2.90347  2.73739  3.18146  2.89801
2.37887  2.77519  2.98518  4.58477  3.61503
          0.01934  4.35126  5.07360  0.61958  0.77255  0.48576  0.95510
      7   2.73613  4.63626  3.63576  3.58179  4.84142  0.51949  4.76206
4.44087  3.83886  4.11648  5.00240  3.74556  4.10974  4.11037  4.08554
2.80227  3.26496  3.81134  6.08078  4.93753     12 G - - S
          2.68618  4.42225  2.77519  2.73123  3.46354  2.40513  3.72494
3.29354  2.67741  2.69355  4.24690  2.90347  2.73739  3.18146  2.89801
2.37887  2.77519  2.98518  4.58477  3.61503
          0.01934  4.35126  5.07360  0.61958  0.77255  0.48576  0.95510
      8   3.02710  4.64880  4.05709  3.64400  3.52785  3.89611  4.44945
2.80040  3.49481  0.94539  3.48891  3.93634  2.26351  3.84663  3.73198
3.32361  3.33409  2.75550  5.25423  4.03557     13 l - - -
          2.68618  4.42225  2.77519  2.73123  3.46354  2.40513  3.72494
3.29354  2.67741  2.69355  4.24690  2.90347  2.73739  3.18146  2.89801
2.37887  2.77519  2.98518  4.58477  3.61503
          0.03684  4.35126  3.76028  0.61958  0.77255  0.48576  0.95510
```

```
      9   2.48386  3.91314  3.89387  3.61760  4.43814  3.19732  4.55622
3.84621  3.53443  3.59254  4.48475  3.66663  0.79392  3.88260  3.77509
2.56374  2.98771  3.34555  5.81773  4.59966     14 p - - T
          2.68394  4.42300  2.77691  2.73054  3.46394  2.40546  3.72444
3.29433  2.67731  2.69535  4.24442  2.90242  2.73639  3.18014  2.89915
2.37871  2.77679  2.98405  4.58366  3.61654
          0.26988  1.99295  2.30025  3.94745  0.01949  0.47892  0.96615
     10   2.79349  4.92542  2.71384  2.50749  4.55882  1.13531  3.97802
4.00558  2.61623  3.60199  4.45505  2.88730  3.95451  3.17289  3.20828
2.80271  3.14833  3.58746  5.79245  4.45783    135 g - - T
          2.68618  4.42225  2.77519  2.73123  3.46354  2.40513  3.72494
3.29354  2.67741  2.69355  4.24690  2.90347  2.73739  3.18146  2.89801
2.37887  2.77519  2.98518  4.58477  3.61503
          0.27506  4.25047  1.48628  0.61958  0.77255  0.53762  0.87741
     11   2.45561  4.55938  3.10413  2.48297  3.73220  2.44123  3.70856
3.19371  2.57178  2.64205  3.71388  2.89070  3.83469  2.91356  2.99639
2.65935  2.41120  2.82561  5.16407  3.87511    136 t - - .
          2.68618  4.42225  2.77519  2.73123  3.46354  2.40513  3.72494
3.29354  2.67741  2.69355  4.24690  2.90347  2.73739  3.18146  2.89801
2.37887  2.77519  2.98518  4.58477  3.61503
          0.03148  4.05120  4.29867  0.61958  0.77255  0.80935  0.58905
     12   2.57594  4.93282  2.57019  2.17984  4.21375  2.42774  3.55096
3.54960  2.41053  3.21807  4.01305  2.81990  3.79005  2.68044  2.89213
2.44762  2.82666  3.09703  5.42538  4.05708    137 e - - .
          2.68618  4.42225  2.77519  2.73123  3.46354  2.40513  3.72494
3.29354  2.67741  2.69355  4.24690  2.90347  2.73739  3.18146  2.89801
2.37887  2.77519  2.98518  4.58477  3.61503
          0.02633  4.04605  4.76839  0.61958  0.77255  0.81340  0.58582
     13   2.52557  4.93544  2.80151  2.09041  4.21763  2.99188  3.55063
3.66060  2.25473  3.11305  3.99512  2.50277  3.77867  2.64824  2.77610
2.57007  2.80341  3.29228  4.45550  4.03765    138 e - - .
          2.68618  4.42225  2.77519  2.73123  3.46354  2.40513  3.72494
3.29354  2.67741  2.69355  4.24690  2.90347  2.73739  3.18146  2.89801
2.37887  2.77519  2.98518  4.58477  3.61503
          0.02633  4.04605  4.76839  0.61958  0.77255  0.81340  0.58582
     14   2.48380  4.84325  2.73032  2.23339  4.10727  2.46223  3.60968
3.53401  2.39994  3.08870  3.92264  2.87957  3.79205  2.75095  2.77587
2.43955  2.79559  3.16054  4.78292  3.99610    139 e - - .
          2.68618  4.42225  2.77519  2.73123  3.46354  2.40513  3.72494
3.29354  2.67741  2.69355  4.24690  2.90347  2.73739  3.18146  2.89801
2.37887  2.77519  2.98518  4.58477  3.61503
          0.02633  4.04605  4.76839  0.61958  0.77255  0.80433  0.59310
     15   2.27961  4.78824  2.86428  2.17874  4.05660  2.70205  3.63379
3.47408  2.43105  2.89279  3.89132  2.93320  3.68628  2.74695  2.90482
2.49718  2.76115  3.05080  5.31902  3.98200    140 e - - .
          2.68618  4.42225  2.77519  2.73123  3.46354  2.40513  3.72494
3.29354  2.67741  2.69355  4.24690  2.90347  2.73739  3.18146  2.89801
2.37887  2.77519  2.98518  4.58477  3.61503
          0.02619  4.05120  4.77354  0.61958  0.77255  0.80935  0.58905
     16   2.16532  4.87277  2.84745  2.26807  4.14997  3.10892  3.57175
3.58168  2.37376  3.15732  3.95552  2.58398  3.61000  2.72034  2.77924
2.48932  2.73130  3.12839  5.37374  4.01882    141 a - - .
          2.68618  4.42225  2.77519  2.73123  3.46354  2.40513  3.72494
3.29354  2.67741  2.69355  4.24690  2.90347  2.73739  3.18146  2.89801
2.37887  2.77519  2.98518  4.58477  3.61503
          0.02619  4.05120  4.77354  0.61958  0.77255  0.80935  0.58905
```

```
     17   2.29007  4.23337  2.93619  2.24848  3.97365  3.10086  3.64069
3.38337  2.43555  3.00166  3.78128  2.94834  3.74413  2.80311  2.88675
2.25742  2.69881  2.94693  5.25936  3.93665    142 e - - .
          2.68618  4.42225  2.77519  2.73123  3.46354  2.40513  3.72494
3.29354  2.67741  2.69355  4.24690  2.90347  2.73739  3.18146  2.89801
2.37887  2.77519  2.98518  4.58477  3.61503
          0.04629  4.05120  3.58150  0.61958  0.77255  0.80935  0.58905
     18   2.23943  4.74697  2.92053  2.29992  4.03811  3.32058  3.64501
3.45174  2.44841  3.03807  3.87931  2.94968  3.69225  2.74021  2.79457
2.08367  2.73272  3.03907  5.30636  3.97870    143 s - - .
          2.68618  4.42225  2.77519  2.73123  3.46354  2.40513  3.72494
3.29354  2.67741  2.69355  4.24690  2.90347  2.73739  3.18146  2.89801
2.37887  2.77519  2.98518  4.58477  3.61503
          0.04920  4.03162  3.49791  0.61958  0.77255  0.82455  0.57702
     19   2.37917  4.52628  2.90315  2.40587  3.86279  3.03739  3.65009
3.04677  2.48920  2.81223  3.70234  2.99620  3.81009  2.83199  2.92002
2.20742  2.71760  2.91847  5.18215  3.87607    144 s - - .
          2.68618  4.42225  2.77519  2.73123  3.46354  2.40513  3.72494
3.29354  2.67741  2.69355  4.24690  2.90347  2.73739  3.18146  2.89801
2.37887  2.77519  2.98518  4.58477  3.61503
          0.04707  4.00974  3.58129  0.61958  0.77255  0.84093  0.56442
     20   2.39859  4.83352  2.92029  2.38405  4.12652  3.20687  3.60231
3.54992  2.16562  3.08668  3.93380  2.50088  3.78839  2.74300  2.63707
2.37817  2.79365  3.09890  5.34498  4.00585    145 k - - .
          2.68618  4.42225  2.77519  2.73123  3.46354  2.40513  3.72494
3.29354  2.67741  2.69355  4.24690  2.90347  2.73739  3.18146  2.89801
2.37887  2.77519  2.98518  4.58477  3.61503
          0.03161  3.99053  4.37185  0.61958  0.77255  0.83156  0.57159
     21   2.43307  4.90296  2.78043  2.35044  4.19683  3.22238  3.58465
3.63461  2.18788  3.13214  3.98183  2.76071  3.00850  2.71310  2.54356
2.47727  2.77743  3.27076  5.39085  4.03085    146 k - - .
          2.68630  4.42201  2.77526  2.73117  3.46369  2.40503  3.72494
3.29361  2.67746  2.69367  4.24705  2.90341  2.73714  3.18158  2.89800
2.37888  2.77531  2.98485  4.58492  3.61514
          0.53690  0.94931  3.56019  0.92828  0.50291  0.44022  1.03252
     22   2.46595  3.98844  3.25082  2.59544  3.70550  3.37268  3.75725
3.03293  2.53305  2.66259  3.57972  3.08114  3.91131  2.99312  2.99562
2.03329  2.76873  2.44292  5.08816  3.77516    150 s - - X
          2.68618  4.42225  2.77519  2.73123  3.46354  2.40513  3.72494
3.29354  2.67741  2.69355  4.24690  2.90347  2.73739  3.18146  2.89801
2.37887  2.77519  2.98518  4.58477  3.61503
          0.10217  4.24637  2.49119  0.61958  0.77255  0.62270  0.76893
     23   2.34452  4.97165  2.81239  2.06650  4.25983  3.10551  3.53497
3.41160  2.36376  3.16672  4.02912  2.88495  3.80049  2.45667  2.64903
2.50266  2.84406  3.23808  5.44002  4.06739    151 e - - X
          2.68618  4.42225  2.77519  2.73123  3.46354  2.40513  3.72494
3.29354  2.67741  2.69355  4.24690  2.90347  2.73739  3.18146  2.89801
2.37887  2.77519  2.98518  4.58477  3.61503
          0.04434  4.16748  3.57995  0.61958  0.77255  0.65418  0.73369
     24   2.62635  4.92149  2.91942  2.42170  4.22012  3.20590  3.40825
3.65081  2.35665  3.21984  4.02019  2.92665  2.77917  2.66958  2.73285
1.91940  2.88787  3.29691  5.42685  4.07859    152 s - - X
          2.68618  4.42225  2.77519  2.73123  3.46354  2.40513  3.72494
3.29354  2.67741  2.69355  4.24690  2.90347  2.73739  3.18146  2.89801
2.37887  2.77519  2.98518  4.58477  3.61503
          0.02312  4.17423  4.89658  0.61958  0.77255  0.65803  0.72955
```

```
     25   2.27420  5.01552  2.81919  2.29955  4.31255  3.41737  3.57752
3.75999  2.24357  3.29726  4.07580  2.90941  2.54730  2.49119  2.66030
2.62463  2.80701  3.38143  5.47695  4.10518    153 k - - X
          2.68618  4.42225  2.77519  2.73123  3.46354  2.40513  3.72494
3.29354  2.67741  2.69355  4.24690  2.90347  2.73739  3.18146  2.89801
2.37887  2.77519  2.98518  4.58477  3.61503
          0.02703  4.19546  4.45678  0.61958  0.77255  0.67857  0.70794
     26   2.59960  5.14872  2.36898  1.91021  4.46910  3.22207  3.62272
3.94589  2.31984  3.44361  4.20216  2.73508  2.93284  2.52102  2.89429
2.63328  2.90929  3.31033  5.59308  4.18286    154 e - - X
          2.68618  4.42225  2.77519  2.73123  3.46354  2.40513  3.72494
3.29354  2.67741  2.69355  4.24690  2.90347  2.73739  3.18146  2.89801
2.37887  2.77519  2.98518  4.58477  3.61503
          0.06690  4.19116  3.00407  0.61958  0.77255  0.68301  0.70339
     27   2.66557  5.00648  2.88184  2.25876  4.31185  3.42538  3.61746
3.75075  2.32522  3.18344  4.07469  2.83801  3.53876  2.63431  2.04026
2.19529  2.89503  3.37937  5.46335  4.10754    155 r - - X
          2.68618  4.42225  2.77519  2.73123  3.46354  2.40513  3.72494
3.29354  2.67741  2.69355  4.24690  2.90347  2.73739  3.18146  2.89801
2.37887  2.77519  2.98518  4.58477  3.61503
          0.02743  4.14801  4.48614  0.61958  0.77255  0.64294  0.74601
     28   2.49615  5.04888  2.67338  2.24198  4.29492  3.30777  3.53684
3.78275  2.23069  3.34040  4.09854  2.79487  3.66116  2.31258  2.66782
2.36910  2.75752  3.17988  5.50037  4.10944    156 k - - X
          2.68622  4.42209  2.77539  2.73130  3.46331  2.40509  3.72506
3.29369  2.67760  2.69332  4.24711  2.90336  2.73713  3.18132  2.89811
2.37874  2.77524  2.98539  4.58498  3.61513
          0.62038  1.11967  1.99604  1.20501  0.35623  0.68655  0.69978
     29   2.59329  4.86630  2.92136  2.38668  4.15305  3.14306  3.40297
3.57967  2.02277  3.05292  3.95643  2.92066  3.17367  2.73708  2.80962
2.25865  2.72106  3.23225  5.36685  4.02328    161 k - - .
          2.68618  4.42225  2.77519  2.73123  3.46354  2.40513  3.72494
3.29354  2.67741  2.69355  4.24690  2.90347  2.73739  3.18146  2.89801
2.37887  2.77519  2.98518  4.58477  3.61503
          0.04719  4.05017  3.55186  0.61958  0.77255  0.81016  0.58841
     30   2.19967  4.02898  2.88452  2.40819  3.71104  3.45597  3.68308
2.93032  2.54668  2.71825  3.61901  3.06621  3.84362  2.90034  2.99132
2.55982  2.77580  2.71392  5.07646  3.36800    162 a - - .
          2.68618  4.42225  2.77519  2.73123  3.46354  2.40513  3.72494
3.29354  2.67741  2.69355  4.24690  2.90347  2.73739  3.18146  2.89801
2.37887  2.77519  2.98518  4.58477  3.61503
          0.03345  4.02975  4.19195  0.61958  0.77255  0.81722  0.58278
     31   2.51473  4.25545  3.47371  2.91290  3.12971  3.25664  3.81833
2.67875  2.86050  2.05519  3.38485  3.30025  3.46377  3.16207  3.20340
2.40714  2.65152  2.50417  4.86756  3.63601    163 l - - .
          2.68618  4.42225  2.77519  2.73123  3.46354  2.40513  3.72494
3.29354  2.67741  2.69355  4.24690  2.90347  2.73739  3.18146  2.89801
2.37887  2.77519  2.98518  4.58477  3.61503
          0.02681  4.02833  4.75067  0.61958  0.77255  0.80023  0.59643
     32   2.08274  3.71628  3.31401  2.76186  3.58262  3.31325  3.78409
2.95967  2.66446  2.66644  3.57360  3.14456  3.26134  3.04850  3.11737
2.25774  2.69945  2.63862  5.03977  3.78559    164 a - - .
          2.68618  4.42225  2.77519  2.73123  3.46354  2.40513  3.72494
3.29354  2.67741  2.69355  4.24690  2.90347  2.73739  3.18146  2.89801
2.37887  2.77519  2.98518  4.58477  3.61503
          0.04664  4.04388  3.57419  0.61958  0.77255  0.81509  0.58447
```

```
     33   2.53585  4.27003  3.03073  2.66346  3.69012  3.42194  3.73380
3.01124  2.63876  2.30168  3.61021  3.11609  3.29409  2.97012  3.04664
2.38984  2.33049  2.78432  5.07232  3.80223    165 l - - .
          2.68592  4.42199  2.77532  2.73136  3.46345  2.40521  3.72507
3.29363  2.67738  2.69340  4.24668  2.90353  2.73724  3.18136  2.89799
2.37891  2.77528  2.98529  4.58489  3.61515
          0.56099  0.86589  4.74651  0.67897  0.70753  0.83020  0.57263
     34   2.43326  4.30181  3.04786  2.60891  3.77255  2.91393  3.71193
2.84233  2.59099  2.82284  3.52776  3.07317  2.85342  2.92507  3.01404
2.28725  2.78655  2.86843  5.12949  3.84717    168 s - - .
          2.68618  4.42225  2.77519  2.73123  3.46354  2.40513  3.72494
3.29354  2.67741  2.69355  4.24690  2.90347  2.73739  3.18146  2.89801
2.37887  2.77519  2.98518  4.58477  3.61503
          0.02692  4.02416  4.74651  0.61958  0.77255  0.83020  0.57263
     35   2.49635  3.63744  3.91150  3.32815  2.85866  3.54752  3.92509
2.34053  2.97500  1.92299  3.16228  3.57291  4.00125  3.45729  3.34301
2.83940  2.68180  2.30326  4.05676  3.31399    169 l - - .
          2.68618  4.42225  2.77519  2.73123  3.46354  2.40513  3.72494
3.29354  2.67741  2.69355  4.24690  2.90347  2.73739  3.18146  2.89801
2.37887  2.77519  2.98518  4.58477  3.61503
          0.02692  4.02416  4.74651  0.61958  0.77255  0.83020  0.57263
     36   2.52277  4.60375  2.92437  2.49776  3.44616  3.31347  3.37225
3.19375  2.41380  2.80561  3.00750  3.01801  3.76388  2.75761  2.90958
2.59420  2.47272  2.91232  5.13684  3.83903    170 k - - .
          2.68618  4.42225  2.77519  2.73123  3.46354  2.40513  3.72494
3.29354  2.67741  2.69355  4.24690  2.90347  2.73739  3.18146  2.89801
2.37887  2.77519  2.98518  4.58477  3.61503
          0.02692  4.02416  4.74651  0.61958  0.77255  0.79638  0.59958
     37   2.48188  4.49765  3.18518  2.63118  3.50284  3.46684  3.36638
3.01009  2.53042  2.44773  3.59944  3.10013  2.64461  2.93590  3.00151
2.49133  2.80671  2.78159  5.05254  3.77554    171 l - - .
          2.68618  4.42225  2.77519  2.73123  3.46354  2.40513  3.72494
3.29354  2.67741  2.69355  4.24690  2.90347  2.73739  3.18146  2.89801
2.37887  2.77519  2.98518  4.58477  3.61503
          0.02639  4.04388  4.76623  0.61958  0.77255  0.72595  0.66138
     38   2.57006  5.02231  2.28842  2.26350  4.32223  3.37862  3.53591
3.77802  2.29121  3.00259  4.07920  2.82532  3.78848  2.53604  2.70096
2.27887  2.82678  3.38929  5.48022  4.09612    172 e - - .
          2.68618  4.42225  2.77519  2.73123  3.46354  2.40513  3.72494
3.29354  2.67741  2.69355  4.24690  2.90347  2.73739  3.18146  2.89801
2.37887  2.77519  2.98518  4.58477  3.61503
          0.04168  4.09545  3.72257  0.61958  0.77255  0.74804  0.64111
     39   2.62631  5.06186  2.67419  2.08681  4.37208  2.99381  3.09370
3.83753  2.29324  3.35179  4.11623  2.71980  3.61559  2.60263  2.81711
2.29668  2.70874  3.43451  5.51384  4.11795    173 e - - .
          2.68618  4.42225  2.77519  2.73123  3.46354  2.40513  3.72494
3.29354  2.67741  2.69355  4.24690  2.90347  2.73739  3.18146  2.89801
2.37887  2.77519  2.98518  4.58477  3.61503
          0.03988  4.09296  3.79845  0.61958  0.77255  0.76202  0.62872
     40   2.56538  4.77198  2.95546  2.44040  4.00721  3.39289  3.42294
3.42063  2.21218  2.90122  3.10288  2.81330  3.54895  2.67700  2.78746
2.42871  2.69102  3.00534  5.27614  3.94659    174 k - - .
          2.68618  4.42225  2.77519  2.73123  3.46354  2.40513  3.72494
3.29354  2.67741  2.69355  4.24690  2.90347  2.73739  3.18146  2.89801
2.37887  2.77519  2.98518  4.58477  3.61503
          0.03379  4.08573  4.10947  0.61958  0.77255  0.74890  0.64034
```

41 2.47998 4.87929 2.90251 2.34833 4.14352 3.02807 3.60226
3.57483 2.27605 3.07024 3.88084 2.64841 3.45399 2.53883 2.75480
2.58587 2.76662 3.22596 5.36303 3.18505 175 k - - X
2.68618 4.42225 2.77519 2.73123 3.46354 2.40513 3.72494
3.29354 2.67741 2.69355 4.24690 2.90347 2.73739 3.18146 2.89801
2.37887 2.77519 2.98518 4.58477 3.61503
0.07669 4.09530 2.86173 0.61958 0.77255 0.68901 0.69730
42 2.50645 4.72767 2.93742 2.46532 3.75561 3.30913 3.64052
3.31179 2.40962 2.75035 3.80838 2.88783 2.76778 2.62047 2.91126
2.45839 2.70039 3.00367 5.24602 3.92459 176 k - - X
2.68599 4.42222 2.77540 2.73149 3.46332 2.40528 3.72493
3.29384 2.67770 2.69336 4.24687 2.90339 2.73742 3.18138 2.89806
2.37833 2.77549 2.98520 4.58345 3.61524
0.68595 0.83288 2.78722 1.34270 0.30265 0.75915 0.63123
43 2.35067 4.44654 3.04233 2.50443 4.01753 3.14317 3.67261
3.42611 2.13655 2.92630 3.86487 2.99334 2.84857 2.83704 2.90962
2.30522 2.78311 3.09196 5.29478 3.98004 183 k - - X
2.68618 4.42225 2.77519 2.73123 3.46354 2.40513 3.72494
3.29354 2.67741 2.69355 4.24690 2.90347 2.73739 3.18146 2.89801
2.37887 2.77519 2.98518 4.58477 3.61503
0.08844 4.04738 2.70042 0.61958 0.77255 0.80471 0.59279
44 2.35550 4.65044 2.75609 2.39917 4.05971 3.33816 3.61265
3.48018 2.40970 2.86166 3.88685 2.81729 3.68908 2.41582 2.87127
2.28193 2.75547 3.14415 5.31225 3.97312 184 s - - X
2.68618 4.42225 2.77519 2.73123 3.46354 2.40513 3.72494
3.29354 2.67741 2.69355 4.24690 2.90347 2.73739 3.18146 2.89801
2.37887 2.77519 2.98518 4.58477 3.61503
0.02783 3.99150 4.71384 0.61958 0.77255 0.85411 0.55453
45 2.43365 4.42838 3.24706 2.68830 3.60082 3.43086 3.71516
2.97497 2.36936 2.24371 3.53517 3.03840 3.86704 2.96987 2.99270
2.41068 2.72113 2.72573 4.24209 3.73297 185 l - - X
2.68618 4.42225 2.77519 2.73123 3.46354 2.40513 3.72494
3.29354 2.67741 2.69355 4.24690 2.90347 2.73739 3.18146 2.89801
2.37887 2.77519 2.98518 4.58477 3.61503
0.02783 3.99150 4.71384 0.61958 0.77255 0.79405 0.60150
46 2.53993 4.83274 2.59969 2.35209 4.12985 3.36004 3.62303
3.31525 2.42484 3.14331 3.94703 2.73906 2.86240 2.76615 2.89667
2.29910 2.58245 3.20811 5.36807 4.01737 186 s - - X
2.68577 4.42173 2.77530 2.73135 3.46362 2.40512 3.72498
3.29366 2.67749 2.69350 4.24702 2.90354 2.73748 3.18159 2.89813
2.37872 2.77515 2.98520 4.58489 3.61501
0.56043 0.86658 4.75020 0.66427 0.72288 0.82741 0.57480
47 2.57326 4.70448 2.90152 2.36839 3.92484 3.23103 3.36829
3.33152 2.44676 2.93469 3.61302 2.80679 3.21096 2.79459 2.89939
2.39522 2.75430 3.02712 3.52136 3.90142 189 e - - X
2.68618 4.42225 2.77519 2.73123 3.46354 2.40513 3.72494
3.29354 2.67741 2.69355 4.24690 2.90347 2.73739 3.18146 2.89801
2.37887 2.77519 2.98518 4.58477 3.61503
0.05774 4.02786 3.26247 0.61958 0.77255 0.81518 0.58440
48 2.48568 4.44354 3.22653 2.62852 3.55916 3.47857 3.46533
2.89506 2.61365 2.33902 3.53804 3.02541 3.86226 2.95418 2.59333
2.58469 2.72326 2.61604 4.99922 3.47057 190 l - - X
2.68616 4.42192 2.77536 2.73132 3.46368 2.40510 3.72456
3.29370 2.67748 2.69337 4.24590 2.90363 2.73713 3.18151 2.89811
2.37881 2.77527 2.98534 4.58493 3.61514
0.56866 0.91680 3.38366 0.94933 0.48939 0.63687 0.75279

```
     49   2.46125  4.89659  2.71021  2.15584  3.92173  2.93754  3.60177
  3.50454  2.35693  3.02482  3.96038  2.83011  3.68838  2.73156  2.79786
  2.44523  2.63845  3.17997  5.37970  4.02017    194 e - - .
          2.68637  4.42200  2.77561  2.73219  3.46277  2.40545  3.72573
  3.29435  2.67733  2.69405  4.24471  2.90259  2.73709  3.18087  2.89872
  2.37721  2.77560  2.98504  4.58585  3.61600
          0.68374  0.79122  3.17045  2.51755  0.08410  0.65387  0.73403
     50   2.42098  4.20900  3.03766  2.59593  3.84824  2.68739  3.71935
  3.23826  2.45950  2.81067  3.73827  3.07240  3.84530  2.91799  3.01229
  2.21749  2.56682  2.67341  5.18827  3.89636    230 s - - X
          2.68618  4.42225  2.77519  2.73123  3.46354  2.40513  3.72494
  3.29354  2.67741  2.69355  4.24690  2.90347  2.73739  3.18146  2.89801
  2.37887  2.77519  2.98518  4.58477  3.61503
          0.11861  4.13547  2.34495  0.61958  0.77255  0.73736  0.65081
     51   2.55822  4.51230  2.97622  2.66821  3.86611  1.88971  3.81989
  3.26675  2.70455  2.30274  3.82576  2.98711  3.84833  3.05789  3.04878
  2.64684  2.86577  2.96224  5.23497  3.94124    231 g - - X
          2.68620  4.42227  2.77521  2.73120  3.46356  2.40511  3.72496
  3.29356  2.67743  2.69352  4.24692  2.90348  2.73739  3.18141  2.89803
  2.37885  2.77516  2.98520  4.58479  3.61505
          0.05641  3.07192  4.76561  0.88300  0.53365  0.32390  1.28491
     52   2.06946  4.85194  2.98530  2.30531  4.25279  2.92502  3.59067
  3.69067  2.35057  3.17435  4.04019  2.67298  3.85579  2.79061  2.77301
  2.54219  2.39642  3.32979  5.45639  4.09473    237 a - - X
          2.68618  4.42225  2.77519  2.73123  3.46354  2.40513  3.72494
  3.29354  2.67741  2.69355  4.24690  2.90347  2.73739  3.18146  2.89801
  2.37887  2.77519  2.98518  4.58477  3.61503
          0.01934  4.35126  5.07360  0.61958  0.77255  0.48576  0.95510
     53   2.95357  5.13261  3.20417  2.75481  4.66511  3.47311  3.82896
  4.07132  1.01740  3.57481  4.41069  3.20855  4.06243  2.97189  2.55531
  2.41429  3.20928  3.69010  5.67324  4.41561    238 k - - X
          2.68618  4.42225  2.77519  2.73123  3.46354  2.40513  3.72494
  3.29354  2.67741  2.69355  4.24690  2.90347  2.73739  3.18146  2.89801
  2.37887  2.77519  2.98518  4.58477  3.61503
          0.01934  4.35126  5.07360  0.61958  0.77255  0.48576  0.95510
     54   3.19335  5.34732  3.59276  2.91522  4.75900  3.82558  2.51736
  4.14355  1.90667  3.57282  4.42833  3.23851  4.18107  2.88058  1.10283
  3.17896  3.35363  3.81731  5.59816  4.40852    239 r - - X
          2.68618  4.42225  2.77519  2.73123  3.46354  2.40513  3.72494
  3.29354  2.67741  2.69355  4.24690  2.90347  2.73739  3.18146  2.89801
  2.37887  2.77519  2.98518  4.58477  3.61503
          0.01934  4.35126  5.07360  0.61958  0.77255  0.48576  0.95510
     55   2.19803  4.53540  3.34346  2.89772  4.25219  1.56240  4.04086
  3.66034  2.92880  3.30999  4.13372  2.57997  3.89281  3.24598  3.33800
  2.34036  2.78613  2.67937  5.56887  4.29447    240 g - - X
          2.68618  4.42225  2.77519  2.73123  3.46354  2.40513  3.72494
  3.29354  2.67741  2.69355  4.24690  2.90347  2.73739  3.18146  2.89801
  2.37887  2.77519  2.98518  4.58477  3.61503
          0.01934  4.35126  5.07360  0.61958  0.77255  0.48576  0.95510
     56   2.49744  4.01062  3.80066  3.22726  1.76144  3.60116  3.97348
  2.62587  2.63111  2.43695  3.35101  3.54693  4.06752  3.41573  3.41431
  2.92613  2.82002  2.40428  4.83570  3.51835    241 f - - X
          2.68618  4.42225  2.77519  2.73123  3.46354  2.40513  3.72494
  3.29354  2.67741  2.69355  4.24690  2.90347  2.73739  3.18146  2.89801
  2.37887  2.77519  2.98518  4.58477  3.61503
          0.01934  4.35126  5.07360  0.61958  0.77255  0.48576  0.95510
```

```
     57   2.31181  4.85575  2.84539  2.13811  4.02926  3.38717  3.65791
3.63227  2.40769  3.06404  4.03746  2.96233  3.59879  2.63315  2.90595
1.98734  2.88649  3.23123  5.45458  4.09072    242 s - - X
          2.68618  4.42225  2.77519  2.73123  3.46354  2.40513  3.72494
3.29354  2.67741  2.69355  4.24690  2.90347  2.73739  3.18146  2.89801
2.37887  2.77519  2.98518  4.58477  3.61503
          0.02283  4.35126  4.63720  0.61958  0.77255  0.48576  0.95510
     58   2.77353  5.45389  1.35334  1.83128  4.76372  3.40941  3.80813
4.26170  2.69643  3.75205  4.54428  2.87138  3.53464  2.82731  3.24220
2.65331  3.17920  3.69830  5.90660  4.44770    243 d - - X
          2.68618  4.42225  2.77519  2.73123  3.46354  2.40513  3.72494
3.29354  2.67741  2.69355  4.24690  2.90347  2.73739  3.18146  2.89801
2.37887  2.77519  2.98518  4.58477  3.61503
          0.02263  4.34783  4.66304  0.61958  0.77255  0.49080  0.94710
     59   1.82075  4.55976  3.30199  2.61658  3.88871  3.46152  3.86019
3.09076  2.75058  2.93953  3.79614  3.21117  3.84165  3.08326  3.16221
2.41728  1.96942  2.85400  5.25347  3.97965    244 a - - X
          2.68601  4.42239  2.77506  2.73118  3.46367  2.40510  3.72505
3.29368  2.67745  2.69365  4.24703  2.90356  2.73735  3.18160  2.89814
2.37884  2.77490  2.98518  4.58491  3.61517
          0.19976  2.54260  2.27878  1.72692  0.19581  0.48789  0.95171
     60   2.48582  4.11971  4.21949  3.63389  3.23061  3.81009  4.13133
1.91500  3.50518  2.12369  2.82347  3.81892  4.17238  3.71780  3.66723
3.03710  2.43900  1.80202  4.58317  3.58816    257 v - - X
          2.68474  4.42209  2.77555  2.73054  3.46426  2.40468  3.72636
3.29538  2.67648  2.69289  4.24735  2.90411  2.73794  3.18081  2.89930
2.37832  2.77604  2.98465  4.58463  3.61692
          0.89520  0.76557  2.06827  3.31222  0.03712  0.62184  0.76993
     61   2.22194  3.83680  2.96746  2.47970  3.78527  3.25352  3.43425
3.19861  2.49649  2.81212  3.64962  2.93717  3.78993  2.76236  2.92592
2.48250  2.76582  2.71008  5.16772  3.86724    391 a - - X
          2.68618  4.42225  2.77519  2.73123  3.46354  2.40513  3.72494
3.29354  2.67741  2.69355  4.24690  2.90347  2.73739  3.18146  2.89801
2.37887  2.77519  2.98518  4.58477  3.61503
          0.15754  4.11990  2.04396  0.61958  0.77255  0.75153  0.63799
     62   2.41863  4.77620  2.79413  2.38293  3.92552  3.22419  3.52973
3.44105  2.35411  2.93659  3.77063  2.91937  3.00753  2.67287  2.86281
2.44146  2.63471  3.11433  5.28235  3.94513    392 k - - X
          2.68618  4.42225  2.77519  2.73123  3.46354  2.40513  3.72494
3.29354  2.67741  2.69355  4.24690  2.90347  2.73739  3.18146  2.89801
2.37887  2.77519  2.98518  4.58477  3.61503
          0.02786  3.99022  4.71256  0.61958  0.77255  0.80124  0.59560
     63   2.38212  4.83495  2.90970  2.31897  4.09280  3.28849  3.47887
3.40562  2.27447  3.03956  3.90414  2.84159  3.15742  2.56141  2.84019
2.45536  2.69281  3.11495  5.32671  3.97959    393 k - - X
          2.68618  4.42225  2.77519  2.73123  3.46354  2.40513  3.72494
3.29354  2.67741  2.69355  4.24690  2.90347  2.73739  3.18146  2.89801
2.37887  2.77519  2.98518  4.58477  3.61503
          0.03251  4.02277  4.26263  0.61958  0.77255  0.74334  0.64535
     64   2.41043  4.86012  2.89990  2.32492  4.12462  3.22343  3.42132
3.50320  2.23550  3.11100  3.89151  2.83324  2.99061  2.73737  2.82325
2.35163  2.78522  3.08443  5.35079  3.99952    394 k - - X
          2.68618  4.42225  2.77519  2.73123  3.46354  2.40513  3.72494
3.29354  2.67741  2.69355  4.24690  2.90347  2.73739  3.18146  2.89801
2.37887  2.77519  2.98518  4.58477  3.61503
          0.05460  4.06954  3.32284  0.61958  0.77255  0.76093  0.62967
```

```
     65   2.31813  4.45585  2.86480  2.37054  3.97398  3.38579  3.59076
3.34931  2.43355  2.91958  3.67739  2.95865  2.84600  2.73704  2.74930
2.51236  2.75891  3.01262  5.25452  3.92826    395 a - - X
          2.68618  4.42225  2.77519  2.73123  3.46354  2.40513  3.72494
3.29354  2.67741  2.69355  4.24690  2.90347  2.73739  3.18146  2.89801
2.37887  2.77519  2.98518  4.58477  3.61503
          0.02594  4.06070  4.78305  0.61958  0.77255  0.76219  0.62856
     66   2.33291  4.92822  2.85693  2.26972  4.21117  3.25577  3.59069
3.65247  2.20534  3.17032  3.90840  2.79695  3.14811  2.66905  2.73314
2.36795  2.76900  3.24398  5.40393  4.03800    396 k - - X
          2.68618  4.42225  2.77519  2.73123  3.46354  2.40513  3.72494
3.29354  2.67741  2.69355  4.24690  2.90347  2.73739  3.18146  2.89801
2.37887  2.77519  2.98518  4.58477  3.61503
          0.02536  4.08299  4.80533  0.61958  0.77255  0.78350  0.61029
     67   2.38332  4.66669  2.81576  2.27891  4.11514  3.32835  3.50691
3.45576  2.35080  3.08835  3.87799  2.79697  3.22620  2.67776  2.82110
2.43131  2.63600  3.01447  5.34493  3.99352    397 e - - X
          2.68618  4.42225  2.77519  2.73123  3.46354  2.40513  3.72494
3.29354  2.67741  2.69355  4.24690  2.90347  2.73739  3.18146  2.89801
2.37887  2.77519  2.98518  4.58477  3.61503
          0.03680  4.08299  3.94896  0.61958  0.77255  0.77733  0.61551
     68   2.35597  4.82528  2.85600  2.29280  4.07587  3.17026  3.25334
3.46838  2.35088  2.95584  3.84402  2.89793  3.58556  2.74906  2.71710
2.47735  2.75024  2.86936  5.31991  3.97579    398 e - - X
          2.68618  4.42225  2.77519  2.73123  3.46354  2.40513  3.72494
3.29354  2.67741  2.69355  4.24690  2.90347  2.73739  3.18146  2.89801
2.37887  2.77519  2.98518  4.58477  3.61503
          0.08705  4.07525  2.71233  0.61958  0.77255  0.78254  0.61109
     69   2.33096  4.76305  2.94366  2.37939  4.00418  2.83619  3.61029
3.38848  2.24357  3.00545  3.55252  2.88021  3.70232  2.73142  2.73781
2.52710  2.79445  2.98055  5.26967  3.93929    399 k - - X
          2.68618  4.42225  2.77519  2.73123  3.46354  2.40513  3.72494
3.29354  2.67741  2.69355  4.24690  2.90347  2.73739  3.18146  2.89801
2.37887  2.77519  2.98518  4.58477  3.61503
          0.02704  4.01968  4.74203  0.61958  0.77255  0.69380  0.69249
     70   2.35291  4.64281  2.94364  2.31555  4.09545  3.14824  3.37112
3.49321  2.28894  3.03294  3.84792  2.82001  3.46874  2.62632  2.86456
2.40369  2.74775  3.04892  5.33416  3.82194    400 k - - X
          2.68618  4.42225  2.77519  2.73123  3.46354  2.40513  3.72494
3.29354  2.67741  2.69355  4.24690  2.90347  2.73739  3.18146  2.89801
2.37887  2.77519  2.98518  4.58477  3.61503
          0.02485  4.10331  4.82566  0.61958  0.77255  0.75135  0.63815
     71   2.45694  5.06575  2.51010  2.07472  4.38294  3.26574  3.40037
3.85171  2.21580  3.33444  4.11636  2.83529  3.31105  2.63027  2.74959
2.46970  2.70706  3.44182  5.51397  4.11637    401 e - - X
          2.68615  4.42229  2.77521  2.73124  3.46358  2.40514  3.72499
3.29358  2.67745  2.69356  4.24627  2.90350  2.73732  3.18137  2.89805
2.37889  2.77518  2.98520  4.58481  3.61507
          0.33953  1.33631  3.68578  0.20882  1.66888  0.75920  0.63118
     72   2.19971  4.91404  2.88650  2.31164  4.19795  3.37350  3.60352
3.63136  1.97474  3.17340  3.94319  2.84724  3.65561  2.73305  2.67680
2.54406  2.72537  3.21784  5.39410  4.04011    403 k - - X
          2.68618  4.42225  2.77519  2.73123  3.46354  2.40513  3.72494
3.29354  2.67741  2.69355  4.24690  2.90347  2.73739  3.18146  2.89801
2.37887  2.77519  2.98518  4.58477  3.61503
          0.05095  4.09400  3.41125  0.61958  0.77255  0.74909  0.64017
```

```
     73   2.37660  4.98175  2.81379  2.22874  4.28046  3.27564  3.53435
3.69479  2.13689  3.26555  4.03710  2.81936  3.39622  2.58729  2.56781
2.54881  2.66340  3.25243  5.44227  4.06520    404 k - - X
          2.68618  4.42225  2.77519  2.73123  3.46354  2.40513  3.72494
3.29354  2.67741  2.69355  4.24690  2.90347  2.73739  3.18146  2.89801
2.37887  2.77519  2.98518  4.58477  3.61503
          0.03932  4.08276  3.83062  0.61958  0.77255  0.76869  0.62291
     74   2.36433  4.99752  2.72124  2.23876  4.21423  3.31456  3.46633
3.75019  2.25134  3.26074  4.05450  2.58313  3.40904  2.60052  2.81831
2.28015  2.83842  3.33944  5.45987  4.07688    405 e - - X
          2.68618  4.42225  2.77519  2.73123  3.46354  2.40513  3.72494
3.29354  2.67741  2.69354  4.24686  2.90347  2.73740  3.18146  2.89801
2.37887  2.77519  2.98518  4.58477  3.61503
          0.03872  3.70673  4.31068  0.58459  0.81494  0.78808  0.60645
     75   2.35205  4.88468  2.85098  2.26388  4.15177  3.25119  3.51334
3.58569  2.28713  3.10763  3.94818  2.87549  3.24444  2.53551  2.84423
2.47733  2.70258  3.11130  5.36746  3.68851    407 e - - X
          2.68618  4.42225  2.77519  2.73123  3.46354  2.40513  3.72494
3.29354  2.67741  2.69355  4.24690  2.90347  2.73739  3.18146  2.89801
2.37887  2.77519  2.98518  4.58477  3.61503
          0.02953  4.07228  4.41795  0.61958  0.77255  0.76183  0.62888
     76   2.36331  4.75825  2.97179  2.38405  3.90157  3.25725  3.59575
3.26335  2.28389  2.97398  3.83274  2.91087  3.51820  2.75587  2.75863
2.42280  2.60051  2.87661  5.26697  3.93855    408 k - - X
          2.68591  4.42218  2.77530  2.73133  3.46384  2.40519  3.72498
3.29362  2.67739  2.69370  4.24681  2.90329  2.73746  3.18138  2.89816
2.37868  2.77518  2.98488  4.58510  3.61536
          0.70099  0.83354  2.66795  1.45198  0.26671  0.72392  0.66329
     77   2.22327  4.21630  3.08269  2.50952  3.79773  3.24614  3.54315
3.24058  2.46655  2.85760  3.72562  2.68161  3.47238  2.85578  2.95395
2.45058  2.65650  2.78075  5.17257  3.87186    415 a - - .
          2.68614  4.42214  2.77521  2.73125  3.46356  2.40515  3.72496
3.29356  2.67743  2.69349  4.24692  2.90349  2.73734  3.18145  2.89801
2.37886  2.77521  2.98518  4.58479  3.61505
          0.11443  2.65042  3.28331  0.71237  0.67429  0.73897  0.64934
     78   2.22455  4.12135  3.01841  2.46213  3.88502  3.28375  3.64939
3.26226  2.46835  2.80185  3.75425  2.83531  3.66426  2.70956  2.76901
2.48026  2.60975  2.92929  5.19735  3.88775    419 a - - .
          2.68618  4.42225  2.77519  2.73123  3.46354  2.40513  3.72494
3.29354  2.67741  2.69355  4.24690  2.90347  2.73739  3.18146  2.89801
2.37887  2.77519  2.98518  4.58477  3.61503
          0.08211  4.06549  2.78584  0.61958  0.77255  0.77125  0.62070
     79   2.22089  4.43103  3.03985  2.50152  3.97040  3.36062  3.66793
3.37525  2.38796  2.94502  3.82695  2.43988  3.23747  2.83773  2.92896
2.43238  2.70307  2.94580  5.26276  3.94992    420 a - - .
          2.68618  4.42225  2.77519  2.73123  3.46354  2.40513  3.72494
3.29354  2.67741  2.69355  4.24690  2.90347  2.73739  3.18146  2.89801
2.37887  2.77519  2.98518  4.58477  3.61503
          0.02686  4.02640  4.74875  0.61958  0.77255  0.78128  0.61215
     80   2.59596  5.08333  2.09297  2.20527  4.21791  3.10681  3.32565
3.79138  2.33972  3.37151  4.14199  2.49989  3.71849  2.63900  2.88127
2.53041  2.88154  3.45353  5.53696  4.13468    421 d - - .
          2.68618  4.42225  2.77519  2.73123  3.46354  2.40513  3.72494
3.29354  2.67741  2.69355  4.24690  2.90347  2.73739  3.18146  2.89801
2.37887  2.77519  2.98518  4.58477  3.61503
          0.02612  4.05395  4.77630  0.61958  0.77255  0.55924  0.84780
```

```
     81   2.40130  4.61905  2.95803  2.18365  4.17428  3.39696  3.30673
3.60695  2.38644  3.13492  3.97242  2.72916  2.65698  2.75852  2.79719
2.52157  2.79461  3.25595  5.39219  3.74231    422 e - - X
          2.68606  4.42227  2.77521  2.73125  3.46356  2.40515  3.72496
3.29356  2.67743  2.69357  4.24671  2.90349  2.73741  3.18148  2.89803
2.37880  2.77521  2.98520  4.58479  3.61505
          0.15206  2.08733  4.07191  0.31658  1.30429  0.48623  0.95435
     82   2.21313  4.92466  2.95472  2.06792  4.04310  2.97503  3.65169
3.59281  2.43105  3.15404  3.99243  2.88539  3.84187  2.65393  2.83537
2.34883  2.87180  2.92766  5.41333  4.05792    424 e - - X
          2.68618  4.42225  2.77519  2.73123  3.46354  2.40513  3.72494
3.29354  2.67741  2.69355  4.24690  2.90347  2.73739  3.18146  2.89801
2.37887  2.77519  2.98518  4.58477  3.61503
          0.02690  4.28682  4.35913  0.61958  0.77255  0.50887  0.91923
     83   2.22859  4.97642  2.97349  2.42043  4.26503  2.96918  3.64794
3.66884  2.10543  3.26118  4.00345  2.89140  3.62407  2.52873  2.81111
2.25452  2.84846  3.11907  5.45763  4.09319    425 k - - .
          2.68618  4.42225  2.77519  2.73123  3.46354  2.40513  3.72494
3.29354  2.67741  2.69355  4.24690  2.90347  2.73739  3.18146  2.89801
2.37887  2.77519  2.98518  4.58477  3.61503
          0.06730  4.31027  2.96325  0.61958  0.77255  0.50445  0.92593
     84   2.10649  4.66935  3.14889  2.60605  3.92536  3.47130  3.48775
3.32019  2.44248  2.96590  3.80658  3.09239  2.58420  2.93410  3.02027
2.42948  2.76211  2.58325  5.25143  3.95307    426 a - - .
          2.68618  4.42225  2.77519  2.73123  3.46354  2.40513  3.72494
3.29354  2.67741  2.69355  4.24690  2.90347  2.73739  3.18146  2.89801
2.37887  2.77519  2.98518  4.58477  3.61503
          0.02074  4.28196  5.00430  0.61958  0.77255  0.44624  1.02173
     85   2.83324  5.06746  3.06393  2.52117  4.43682  3.53890  3.75204
3.85491  2.36205  3.35800  4.21343  2.81115  1.79829  2.89228  1.95099
2.80608  3.07101  3.49842  5.55883  4.25138    427 p - - X
          2.68618  4.42225  2.77519  2.73123  3.46354  2.40513  3.72494
3.29354  2.67741  2.69355  4.24690  2.90347  2.73739  3.18146  2.89801
2.37887  2.77519  2.98518  4.58477  3.61503
          0.01948  4.34396  5.06630  0.61958  0.77255  0.49643  0.93828
     86   1.72798  4.69757  3.15583  2.55532  3.94058  3.18034  3.61306
3.33773  2.59666  2.95905  3.82059  3.10163  3.82629  2.88397  3.03453
2.29199  2.60433  3.01089  4.93974  3.96481    428 a - - X
          2.68618  4.42225  2.77519  2.73123  3.46354  2.40513  3.72494
3.29354  2.67741  2.69355  4.24690  2.90347  2.73739  3.18146  2.89801
2.37887  2.77519  2.98518  4.58477  3.61503
          0.07948  4.34396  2.75807  0.61958  0.77255  0.48768  0.95204
     87   1.51102  4.56514  3.25952  2.77804  4.15187  3.31760  3.86120
3.56224  2.77515  3.20084  4.02737  3.12208  2.62496  3.11504  3.05002
2.25545  2.63290  3.18155  5.46115  4.17678    429 a - - X
          2.68618  4.42225  2.77519  2.73123  3.46354  2.40513  3.72494
3.29354  2.67741  2.69355  4.24690  2.90347  2.73739  3.18146  2.89801
2.37887  2.77519  2.98518  4.58477  3.61503
          0.04421  4.28906  3.52222  0.61958  0.77255  0.44356  1.02652
     88   2.49003  4.90361  3.05598  2.50106  4.20007  3.39197  3.68882
3.50695  1.68630  3.20248  4.00650  3.01548  3.68102  2.82873  2.76985
2.21015  2.90558  2.97080  5.41979  4.08397    430 k - - X
          2.68624  4.42218  2.77526  2.73122  3.46360  2.40519  3.72481
3.29338  2.67738  2.69356  4.24696  2.90344  2.73736  3.18137  2.89798
2.37881  2.77526  2.98521  4.58483  3.61509
          0.05193  3.11935  5.04818  1.45049  0.26717  0.52209  0.89962
```

```
     89   1.60769  4.59023  3.24309  2.68965  3.57124  3.08771  3.81102
3.25846  2.69146  2.87784  3.77567  3.16669  3.00099  2.89030  3.11225
2.66152  2.80715  2.91022  5.22858  3.94721    441 a - - X
          2.68618  4.42225  2.77519  2.73123  3.46354  2.40513  3.72494
3.29354  2.67741  2.69355  4.24690  2.90347  2.73739  3.18146  2.89801
2.37887  2.77519  2.98518  4.58477  3.61503
          0.01984  4.32584  5.04818  0.61958  0.77255  0.51349  0.91231
     90   2.71586  4.95503  3.01101  2.52752  4.31676  3.24183  3.73642
3.74644  2.45692  3.28175  4.11950  3.03555  2.34656  1.73842  2.78806
2.69356  2.96883  3.24944  5.50946  4.17449    442 q - - X
          2.68618  4.42225  2.77519  2.73123  3.46354  2.40513  3.72494
3.29354  2.67741  2.69355  4.24690  2.90347  2.73739  3.18146  2.89801
2.37887  2.77519  2.98518  4.58477  3.61503
          0.01977  4.32953  5.05188  0.61958  0.77255  0.51695  0.90717
     91   2.62570  4.47334  4.68982  4.26823  3.88084  4.12848  5.02524
1.96448  4.15057  2.49827  3.71655  4.39486  4.64029  4.45424  4.35521
3.55412  3.35006  0.77087  5.72329  4.49769    443 v - - X
          2.68618  4.42225  2.77519  2.73123  3.46354  2.40513  3.72494
3.29354  2.67741  2.69355  4.24690  2.90347  2.73739  3.18146  2.89801
2.37887  2.77519  2.98518  4.58477  3.61503
          0.01977  4.32953  5.05188  0.61958  0.77255  0.46674  0.98629
     92   3.20021  4.58785  4.90427  4.51209  3.83844  4.38627  5.23584
1.91888  4.36248  2.39476  3.69784  4.64448  4.84772  4.68635  4.54667
3.84394  3.52442  0.66593  5.79122  4.52040    444 V - - X
          2.68618  4.42225  2.77519  2.73123  3.46354  2.40513  3.72494
3.29354  2.67741  2.69355  4.24690  2.90347  2.73739  3.18146  2.89801
2.37887  2.77519  2.98518  4.58477  3.61503
          0.01934  4.35126  5.07360  0.61958  0.77255  0.48576  0.95510
     93   3.36987  5.07041  4.13839  4.10947  5.19133  0.26594  5.17194
4.93531  4.35370  4.51632  5.51465  4.30529  4.47298  4.64076  4.51115
3.55933  3.88673  4.37049  6.13257  5.31844    445 G - - X
          2.68618  4.42225  2.77519  2.73123  3.46354  2.40513  3.72494
3.29354  2.67741  2.69355  4.24690  2.90347  2.73739  3.18146  2.89801
2.37887  2.77519  2.98518  4.58477  3.61503
          0.01934  4.35126  5.07360  0.61958  0.77255  0.48576  0.95510
     94   4.24880  5.42805  4.89379  4.73576  3.32330  4.33122  4.61806
4.33045  4.47147  3.62991  4.93519  4.80088  4.89186  4.83078  4.50533
4.44805  4.57923  4.23115  0.30145  3.30497    446 W - - X
          2.68618  4.42225  2.77519  2.73123  3.46354  2.40513  3.72494
3.29354  2.67741  2.69355  4.24690  2.90347  2.73739  3.18146  2.89801
2.37887  2.77519  2.98518  4.58477  3.61503
          0.01934  4.35126  5.07360  0.61958  0.77255  0.48576  0.95510
     95   3.51551  5.16293  4.17656  4.10153  5.01421  3.82493  5.11378
4.71707  4.20668  4.27537  5.36211  4.34900  0.28080  4.57809  4.37322
3.70338  3.99760  4.30069  6.03770  5.14909    447 P - - X
          2.68618  4.42225  2.77519  2.73123  3.46354  2.40513  3.72494
3.29354  2.67741  2.69355  4.24690  2.90347  2.73739  3.18146  2.89801
2.37887  2.77519  2.98518  4.58477  3.61503
          0.01934  4.35126  5.07360  0.61958  0.77255  0.48576  0.95510
     96   3.51551  5.16293  4.17656  4.10153  5.01421  3.82493  5.11378
4.71707  4.20668  4.27537  5.36211  4.34900  0.28080  4.57809  4.37322
3.70338  3.99760  4.30069  6.03770  5.14909    448 P - - X
          2.68618  4.42225  2.77519  2.73123  3.46354  2.40513  3.72494
3.29354  2.67741  2.69355  4.24690  2.90347  2.73739  3.18146  2.89801
2.37887  2.77519  2.98518  4.58477  3.61503
          0.01934  4.35126  5.07360  0.61958  0.77255  0.48576  0.95510
```

```
     97   3.35219  4.58583  5.29913  4.81165  3.83633  4.87060  5.50919
1.10410  4.72382  2.22028  3.59975  4.99247  5.11511  4.96556  4.89170
4.28074  3.61080  1.04424  5.88968  4.68750    449 v - - X
          2.68618  4.42225  2.77519  2.73123  3.46354  2.40513  3.72494
3.29354  2.67741  2.69355  4.24690  2.90347  2.73739  3.18146  2.89801
2.37887  2.77519  2.98518  4.58477  3.61503
          0.01934  4.35126  5.07360  0.61958  0.77255  0.48576  0.95510
     98   3.23145  5.36544  3.59618  2.94764  4.84283  3.68165  3.77101
4.18744  2.00071  3.60857  4.47289  3.35216  4.20294  2.24583  1.00656
3.21796  3.39434  3.86096  5.63712  4.47208    450 r - - X
          2.68618  4.42225  2.77519  2.73123  3.46354  2.40513  3.72494
3.29354  2.67741  2.69355  4.24690  2.90347  2.73739  3.18146  2.89801
2.37887  2.77519  2.98518  4.58477  3.61503
          0.01934  4.35126  5.07360  0.61958  0.77255  0.48576  0.95510
     99   2.51393  4.59972  3.23110  2.89321  4.39374  3.28541  4.09146
3.81784  2.95125  3.40292  4.28297  3.01299  3.92069  3.14270  3.33697
1.04199  2.57826  3.37170  5.68708  4.40630    451 s - - X
          2.68618  4.42225  2.77519  2.73123  3.46354  2.40513  3.72494
3.29354  2.67741  2.69355  4.24690  2.90347  2.73739  3.18146  2.89801
2.37887  2.77519  2.98518  4.58477  3.61503
          0.01934  4.35126  5.07360  0.61958  0.77255  0.48576  0.95510
    100   3.93086  5.11990  4.92923  4.65414  0.80061  4.65137  3.50997
3.69700  4.48402  3.04085  4.27235  4.31030  4.95503  4.39792  4.47102
3.72838  4.14807  3.58710  3.76877  1.44176    452 f - - X
          2.68618  4.42225  2.77519  2.73123  3.46354  2.40513  3.72494
3.29354  2.67741  2.69355  4.24690  2.90347  2.73739  3.18146  2.89801
2.37887  2.77519  2.98518  4.58477  3.61503
          0.01934  4.35126  5.07360  0.61958  0.77255  0.48576  0.95510
    101   3.64006  5.39042  4.18729  3.62838  4.87378  3.99860  4.29692
4.47479  2.59431  3.90271  4.91188  3.93959  4.49769  3.51271  0.44369
3.71539  3.88579  4.18264  5.77229  4.72268    453 R - - X
          2.68618  4.42225  2.77519  2.73123  3.46354  2.40513  3.72494
3.29354  2.67741  2.69355  4.24690  2.90347  2.73739  3.18146  2.89801
2.37887  2.77519  2.98518  4.58477  3.61503
          0.01934  4.35126  5.07360  0.61958  0.77255  0.48576  0.95510
    102   2.98727  5.14803  3.45142  2.78724  4.48961  3.71782  3.73062
3.61200  1.12986  3.36262  3.52023  3.23673  4.07872  2.86647  2.07943
2.99989  3.17704  3.53665  5.51098  4.27623    454 k - - X
          2.68618  4.42225  2.77519  2.73123  3.46354  2.40513  3.72494
3.29354  2.67741  2.69355  4.24690  2.90347  2.73739  3.18146  2.89801
2.37887  2.77519  2.98518  4.58477  3.61503
          0.01934  4.35126  5.07360  0.61958  0.77255  0.48576  0.95510
    103   2.95176  5.02434  2.86786  2.80634  4.52473  3.41212  4.22934
4.29518  3.16168  3.91454  4.83409  0.73080  4.10556  3.48637  3.54205
2.93445  3.36596  3.83413  5.84997  4.43061    455 n - - X
          2.68618  4.42225  2.77519  2.73123  3.46354  2.40513  3.72494
3.29354  2.67741  2.69355  4.24690  2.90347  2.73739  3.18146  2.89801
2.37887  2.77519  2.98518  4.58477  3.61503
          0.02441  4.35126  4.48995  0.61958  0.77255  0.48576  0.95510
    104   2.62742  4.20110  3.91042  3.33386  3.32018  3.72260  4.02107
2.35894  3.24296  1.83976  3.00685  3.50390  4.09833  3.49969  3.49271
2.53542  2.19018  2.26674  4.82185  3.61879    456 l - - X
          2.68618  4.42225  2.77519  2.73123  3.46354  2.40513  3.72494
3.29354  2.67741  2.69355  4.24690  2.90347  2.73739  3.18146  2.89801
2.37887  2.77519  2.98518  4.58477  3.61503
          0.06034  4.34628  3.08781  0.61958  0.77255  0.48123  0.96239
```

```
    105   2.40335  4.29899  4.43359  3.85379  3.18581  4.03688  4.37097
2.32036  3.72861  1.50805  2.18485  4.05153  4.37146  3.92291  3.88643
3.34190  3.01721  1.88947  4.95948  3.79917    457 l - - X
          2.68618  4.42225  2.77520  2.73124  3.46354  2.40510  3.72495
3.29354  2.67741  2.69355  4.24683  2.90347  2.73740  3.18147  2.89801
2.37887  2.77518  2.98517  4.58477  3.61503
          0.05397  3.23479  4.32993  0.49299  0.94366  0.47180  0.97783
    106   1.59680  4.51894  3.32932  2.78428  3.50100  3.45903  3.73259
3.11796  2.76035  2.84067  3.70489  2.92924  3.93389  3.08689  3.16448
2.40894  2.66138  2.71402  5.16492  3.89860    459 a - - X
          2.68618  4.42225  2.77519  2.73123  3.46354  2.40513  3.72494
3.29354  2.67741  2.69355  4.24690  2.90347  2.73739  3.18146  2.89801
2.37887  2.77519  2.98518  4.58477  3.61503
          0.01964  4.33590  5.05825  0.61958  0.77255  0.48701  0.95312
    107   2.32646  4.70822  2.99248  2.48051  4.08879  3.09031  3.06181
3.50542  2.48843  3.10938  3.85676  2.75751  3.83759  2.78962  2.95179
2.27550  2.46097  2.83115  5.35412  3.85713    460 s - - X
          2.68618  4.42225  2.77519  2.73123  3.46354  2.40513  3.72494
3.29354  2.67741  2.69355  4.24690  2.90347  2.73739  3.18146  2.89801
2.37887  2.77519  2.98518  4.58477  3.61503
          0.01946  4.34484  5.06719  0.61958  0.77255  0.49514  0.94028
    108   2.62787  5.08079  2.92118  2.39499  4.39448  3.44626  3.50487
3.85208  2.28677  3.37492  4.14219  2.58808  2.99408  2.26558  2.87517
2.10708  2.64179  3.45710  5.54226  4.15902    461 s - - X
          2.68618  4.42225  2.77519  2.73123  3.46354  2.40513  3.72494
3.29354  2.67741  2.69355  4.24690  2.90347  2.73739  3.18146  2.89801
2.37887  2.77519  2.98518  4.58477  3.61503
          0.02565  4.34484  4.39424  0.61958  0.77255  0.49514  0.94028
    109   2.36901  4.48107  3.05979  2.48496  4.08319  3.47928  3.69126
3.49840  2.35966  2.94122  3.91858  2.99214  2.79911  2.67941  2.90273
2.14576  2.57929  3.10478  5.35046  4.01857    462 s - - X
          2.68621  4.42228  2.77522  2.73126  3.46356  2.40515  3.72497
3.29357  2.67737  2.69351  4.24692  2.90341  2.73735  3.18149  2.89803
2.37879  2.77522  2.98521  4.58480  3.61506
          0.11865  3.35495  2.56429  1.03474  0.43900  0.49828  0.93541
    110   2.54415  4.92452  2.86484  2.35992  4.09385  3.44236  3.64277
3.56345  2.27522  3.11886  3.98896  2.87454  3.02406  2.71725  2.86616
2.05266  2.74991  2.95853  5.40938  4.05282    469 s - - X
          2.68618  4.42225  2.77519  2.73123  3.46354  2.40513  3.72494
3.29354  2.67741  2.69355  4.24690  2.90347  2.73739  3.18146  2.89801
2.37887  2.77519  2.98518  4.58477  3.61503
          0.02103  4.26807  4.99042  0.61958  0.77255  0.47016  0.98057
    111   2.62638  5.09120  2.94468  2.34182  4.40719  3.22934  3.42408
3.85937  1.61873  3.37787  4.14911  2.92888  3.02537  2.69712  2.79370
2.52084  2.89477  3.39562  5.53903  4.16612    470 k - - X
          2.68620  4.42227  2.77518  2.73125  3.46355  2.40507  3.72496
3.29356  2.67738  2.69355  4.24691  2.90345  2.73739  3.18144  2.89802
2.37888  2.77521  2.98520  4.58478  3.61505
          0.06310  3.42719  3.55176  0.85932  0.55069  0.48947  0.94921
    112   2.52665  4.64142  2.94296  2.35847  4.33099  3.03433  3.38388
3.78376  2.36792  3.22025  4.08881  2.29907  3.29785  2.34334  2.87334
2.40971  2.80497  3.39856  5.49706  4.06507    478 n - - X
          2.68618  4.42225  2.77519  2.73123  3.46354  2.40513  3.72494
3.29354  2.67741  2.69355  4.24690  2.90347  2.73739  3.18146  2.89801
2.37887  2.77519  2.98518  4.58477  3.61503
          0.16553  4.31839  1.97161  0.61958  0.77255  0.53232  0.88489
```

113 2.32869 5.02963 2.81544 2.22237 4.33343 3.18253 2.99078
3.79144 2.30939 3.31724 4.08405 2.72422 3.54162 2.56714 2.81612
2.30826 2.78385 3.35467 5.48822 4.10254 479 e - - X
2.68618 4.42225 2.77519 2.73123 3.46354 2.40513 3.72494
3.29354 2.67741 2.69355 4.24690 2.90347 2.73739 3.18146 2.89801
2.37887 2.77519 2.98518 4.58477 3.61503
0.04187 4.17594 3.66357 0.61958 0.77255 0.40670 1.09615
114 2.63704 5.11623 2.76983 2.23364 4.44055 3.33119 3.57142
3.91216 1.96172 3.27766 4.16370 2.63022 3.15576 2.62820 2.85544
2.36232 2.60653 3.46524 5.56092 4.16238 480 k - - X
2.68618 4.42225 2.77519 2.73123 3.46354 2.40513 3.72494
3.29354 2.67741 2.69355 4.24690 2.90347 2.73739 3.18146 2.89801
2.37887 2.77519 2.98518 4.58477 3.61503
0.02004 4.31599 5.03834 0.61958 0.77255 0.53557 0.88028
115 2.65061 5.21553 2.51609 1.60517 4.53834 3.27119 3.67008
4.01901 2.36868 3.51189 4.27065 2.78090 3.78244 2.60752 2.93550
2.33873 2.83404 3.56997 5.65821 4.24235 481 e - - X
2.68599 4.42248 2.77535 2.73112 3.46396 2.40532 3.72546
3.29390 2.67723 2.69395 4.24726 2.90310 2.73747 3.18180 2.89852
2.37843 2.77403 2.98512 4.58528 3.61554
0.41454 1.38066 2.43104 2.12200 0.12760 0.51471 0.91050
116 2.47254 5.08980 2.54519 2.08988 4.41177 3.22205 3.39812
3.71051 2.21884 3.38510 4.13748 2.84255 3.01031 2.66978 2.77863
2.48854 2.73450 3.44089 5.53614 4.13826 502 e - - X
2.68618 4.42225 2.77519 2.73123 3.46354 2.40513 3.72494
3.29354 2.67741 2.69355 4.24690 2.90347 2.73739 3.18146 2.89801
2.37887 2.77519 2.98518 4.58477 3.61503
0.02497 4.24090 4.57903 0.61958 0.77255 0.60269 0.79261
117 2.06645 4.89675 2.94977 2.30864 4.16495 3.21259 3.65386
3.59333 2.43977 3.17484 3.93665 2.65498 3.56441 2.46358 2.90980
2.59838 2.79973 2.79689 5.39676 4.04617 503 a - - X
2.68618 4.42225 2.77519 2.73123 3.46354 2.40513 3.72494
3.29354 2.67741 2.69355 4.24690 2.90347 2.73739 3.18146 2.89801
2.37887 2.77519 2.98518 4.58477 3.61503
0.10147 4.25001 2.49822 0.61958 0.77255 0.61848 0.77385
118 2.52739 5.19065 2.24432 1.94347 4.51023 3.26768 3.64761
3.99072 2.41271 3.48945 4.25609 2.48169 2.96164 2.76483 2.95938
2.45739 2.89506 3.57111 5.64229 4.22229 504 e - - X
2.68618 4.42225 2.77519 2.73123 3.46354 2.40513 3.72494
3.29354 2.67741 2.69355 4.24690 2.90347 2.73739 3.18146 2.89801
2.37887 2.77519 2.98518 4.58477 3.61503
0.03879 4.17172 3.78891 0.61958 0.77255 0.70267 0.68372
119 2.08584 4.77500 2.95097 2.39231 4.03825 2.61021 3.67010
3.45011 2.34594 3.06294 3.88396 2.98173 3.64644 2.75516 2.93609
2.58487 2.83738 2.79173 5.31512 3.98802 505 a - - X
2.68618 4.42225 2.77519 2.73123 3.46354 2.40513 3.72494
3.29354 2.67741 2.69355 4.24690 2.90347 2.73739 3.18146 2.89801
2.37887 2.77519 2.98518 4.58477 3.61503
0.03147 4.15647 4.17861 0.61958 0.77255 0.69744 0.68887
120 2.22841 4.69725 3.08381 2.51213 3.92732 3.22045 3.67831
3.04458 1.98988 2.95861 3.79387 2.99629 3.85208 2.82368 2.89902
2.54438 2.82870 2.74366 5.23251 3.92639 506 k - - X
2.68618 4.42225 2.77519 2.73123 3.46354 2.40513 3.72494
3.29354 2.67741 2.69355 4.24690 2.90347 2.73739 3.18146 2.89801
2.37887 2.77519 2.98518 4.58477 3.61503
0.03478 4.15906 3.98693 0.61958 0.77255 0.58050 0.82011

121 2.18635 4.88209 2.96081 2.42838 4.14600 3.38753 3.64016
3.47941 2.34680 3.02562 3.95472 2.73427 3.00009 2.53580 2.85069
2.38698 2.79310 3.15130 5.37751 4.02877 507 a - - X
2.68618 4.42225 2.77519 2.73123 3.46354 2.40513 3.72494
3.29354 2.67741 2.69355 4.24690 2.90347 2.73739 3.18146 2.89801
2.37887 2.77519 2.98518 4.58477 3.61503
0.02817 4.21911 4.33804 0.61958 0.77255 0.60691 0.78753
122 2.41114 5.05642 2.71901 2.28244 4.35755 2.27007 3.64275
3.78172 2.35368 3.34665 4.12305 2.43204 3.78848 2.68064 2.84014
2.56187 2.90377 3.34797 5.52588 4.14081 508 g - - X
2.68618 4.42225 2.77519 2.73123 3.46354 2.40513 3.72494
3.29354 2.67741 2.69355 4.24690 2.90347 2.73739 3.18146 2.89801
2.37887 2.77519 2.98518 4.58477 3.61503
0.03126 4.23570 4.11646 0.61958 0.77255 0.63491 0.75498
123 2.20482 4.87797 2.95531 2.19843 3.91317 3.43864 3.55006
3.43129 2.27365 2.93532 3.94386 2.86474 3.52975 2.68505 2.83657
2.44533 2.83533 3.02676 5.36871 3.97293 509 e - - X
2.68618 4.42225 2.77519 2.73123 3.46354 2.40513 3.72494
3.29354 2.67741 2.69355 4.24690 2.90347 2.73739 3.18146 2.89801
2.37887 2.77519 2.98518 4.58477 3.61503
0.23983 4.22638 1.61629 0.61958 0.77255 0.63943 0.74992
124 2.00371 4.53200 2.97301 2.58364 3.87957 3.13251 3.72097
3.26994 2.58381 2.92516 3.63111 2.84460 3.80050 2.91819 3.01456
2.39155 2.52286 2.73278 5.21277 3.92077 510 a - - .
2.68618 4.42225 2.77519 2.73123 3.46354 2.40513 3.72494
3.29354 2.67741 2.69355 4.24690 2.90347 2.73739 3.18146 2.89801
2.37887 2.77519 2.98518 4.58477 3.61503
0.14343 4.01722 2.15749 0.61958 0.77255 0.43297 1.04577
125 2.43464 4.59288 2.60773 2.14792 4.33629 2.37718 3.61834
3.79275 2.31498 3.32433 4.09618 2.71980 3.50563 2.73590 2.84892
2.57425 2.87171 3.40251 5.50083 4.11555 511 e - - X
2.68593 4.42234 2.77532 2.73098 3.46368 2.40521 3.72508
3.29361 2.67676 2.69364 4.24691 2.90352 2.73752 3.18139 2.89832
2.37881 2.77531 2.98536 4.58510 3.61518
0.83247 1.15519 1.38619 1.63634 0.21653 0.52162 0.90032
126 2.34001 3.23783 3.23761 2.82201 3.63475 3.03267 3.82163
2.93395 2.78822 2.70352 3.57607 3.17798 3.59995 3.10065 3.16552
2.37099 2.25235 2.57795 5.04386 3.79807 519 t - - .
2.68618 4.42225 2.77519 2.73123 3.46354 2.40513 3.72494
3.29354 2.67741 2.69355 4.24690 2.90347 2.73739 3.18146 2.89801
2.37887 2.77519 2.98518 4.58477 3.61503
0.02705 4.01927 4.74161 0.61958 0.77255 0.68350 0.70289
127 2.22585 4.78794 2.95378 2.39956 4.18290 3.10294 3.60763
3.53770 2.08294 3.17993 3.97313 2.84007 3.56919 2.64498 2.53383
2.53545 2.77146 3.25951 5.38432 4.03382 520 k - - X
2.68618 4.42225 2.77519 2.73123 3.46354 2.40513 3.72494
3.29354 2.67741 2.69355 4.24690 2.90347 2.73739 3.18146 2.89801
2.37887 2.77519 2.98518 4.58477 3.61503
0.02469 4.10948 4.83182 0.61958 0.77255 0.70801 0.67850
128 2.45107 4.83445 2.97927 2.43099 4.11910 3.01656 3.63413
3.54338 2.09987 3.05728 3.93314 2.83055 3.62839 2.77548 2.78092
2.27492 2.61007 3.02664 5.35529 4.01344 521 k - - X
2.68618 4.42225 2.77519 2.73123 3.46354 2.40513 3.72494
3.29354 2.67741 2.69355 4.24690 2.90347 2.73739 3.18146 2.89801
2.37887 2.77519 2.98518 4.58477 3.61503
0.02399 4.13787 4.86022 0.61958 0.77255 0.36920 1.17534

```
     129   2.04212  2.90343  3.43411  2.88113  3.48680  2.76707  3.88884
3.08165  2.84821  2.77550  3.64859  3.18234  2.96489  3.16331  3.14407
2.41946  2.83332  2.69087  5.11621  3.86606    522 a - - X
           2.68618  4.42225  2.77519  2.73123  3.46354  2.40513  3.72494
3.29354  2.67741  2.69355  4.24690  2.90347  2.73739  3.18146  2.89801
2.37887  2.77519  2.98518  4.58477  3.61503
           0.01949  4.34351  5.06586  0.61958  0.77255  0.47876  0.96641
     130   2.28373  4.00374  3.61736  3.04890  3.44706  3.40978  3.74039
2.80370  2.98831  1.68269  3.15496  3.42511  3.35486  3.09018  3.31535
2.80923  2.81315  2.58517  4.90665  3.68258    523 l - - X
           2.68618  4.42225  2.77519  2.73123  3.46354  2.40513  3.72494
3.29354  2.67741  2.69355  4.24690  2.90347  2.73739  3.18146  2.89801
2.37887  2.77519  2.98518  4.58477  3.61503
           0.01934  4.35126  5.07360  0.61958  0.77255  0.48576  0.95510
     131   3.48197  4.75840  4.89050  4.47887  1.16875  4.42878  3.77577
3.17981  4.29992  2.01754  3.53706  4.25758  4.73771  4.27651  4.29823
3.76602  3.70199  3.07789  3.41843  1.59987    524 f - - X
           2.68618  4.42225  2.77519  2.73123  3.46354  2.40513  3.72494
3.29354  2.67741  2.69355  4.24690  2.90347  2.73739  3.18146  2.89801
2.37887  2.77519  2.98518  4.58477  3.61503
           0.01934  4.35126  5.07360  0.61958  0.77255  0.48576  0.95510
     132   3.24897  4.58748  5.01091  4.57844  3.83005  4.53357  5.30173
1.81056  4.44153  2.34956  3.65562  4.73645  4.92965  4.74610  4.62927
3.97057  3.54934  0.68402  5.81538  4.56385    525 V - - X
           2.68618  4.42225  2.77519  2.73123  3.46354  2.40513  3.72494
3.29354  2.67741  2.69355  4.24690  2.90347  2.73739  3.18146  2.89801
2.37887  2.77519  2.98518  4.58477  3.61503
           0.01934  4.35126  5.07360  0.61958  0.77255  0.48576  0.95510
     133   3.49633  5.41552  3.70075  3.26387  4.91697  3.89948  4.13367
4.38635  0.52868  3.85714  4.81741  3.65648  4.38598  3.31543  2.65694
3.52172  3.72501  4.07631  5.78899  4.68737    526 K - - X
           2.68618  4.42225  2.77519  2.73123  3.46354  2.40513  3.72494
3.29354  2.67741  2.69355  4.24690  2.90347  2.73739  3.18146  2.89801
2.37887  2.77519  2.98518  4.58477  3.61503
           0.01934  4.35126  5.07360  0.61958  0.77255  0.48576  0.95510
     134   3.32837  4.58276  5.23535  4.76344  3.86082  4.79740  5.48256
1.40888  4.66964  2.32735  3.63250  4.94030  5.08142  4.93186  4.84814
4.21583  3.59691  0.80691  5.90111  4.68381    527 v - - X
           2.68618  4.42225  2.77519  2.73123  3.46354  2.40513  3.72494
3.29354  2.67741  2.69355  4.24690  2.90347  2.73739  3.18146  2.89801
2.37887  2.77519  2.98518  4.58477  3.61503
           0.01934  4.35126  5.07360  0.61958  0.77255  0.48576  0.95510
     135   2.60251  4.33951  3.18849  2.66490  3.96373  3.46422  3.80264
3.39204  2.65395  3.03716  3.87793  2.24973  3.91180  2.99849  3.08391
1.75731  2.82083  3.08195  5.29798  2.94239    528 s - - X
           2.68618  4.42225  2.77519  2.73123  3.46354  2.40513  3.72494
3.29354  2.67741  2.69355  4.24690  2.90347  2.73739  3.18146  2.89801
2.37887  2.77519  2.98518  4.58477  3.61503
           0.01934  4.35126  5.07360  0.61958  0.77255  0.48576  0.95510
     136   3.36504  4.78324  4.80958  4.29339  3.28318  4.47948  4.87867
2.38391  4.04564  1.64299  1.00377  4.53378  4.78515  4.30027  4.19930
3.86190  3.62581  2.37306  5.30758  4.15065    529 m - - X
           2.68618  4.42225  2.77519  2.73123  3.46354  2.40513  3.72494
3.29354  2.67741  2.69355  4.24690  2.90347  2.73739  3.18146  2.89801
2.37887  2.77519  2.98518  4.58477  3.61503
           0.01934  4.35126  5.07360  0.61958  0.77255  0.48576  0.95510
```

137   3.32443 5.87233 0.67124 2.18893 5.19041 3.43848 4.10668
4.76325 3.24793 4.27032 5.19423 2.93008 4.12115 3.30238 3.89684
3.18188 3.64769 4.32619 6.36523 4.85371   530 D - - X
2.68618 4.42225 2.77519 2.73123 3.46354 2.40513 3.72494
3.29354 2.67741 2.69355 4.24690 2.90347 2.73739 3.18146 2.89801
2.37887 2.77519 2.98518 4.58477 3.61503
0.01934 4.35126 5.07360 0.61958 0.77255 0.48576 0.95510
138   3.36987 5.07041 4.13839 4.10947 5.19133 0.26594 5.17194
4.93531 4.35370 4.51632 5.51465 4.30529 4.47298 4.64076 4.51115
3.55933 3.88673 4.37049 6.13257 5.31844   531 G - - X
2.68618 4.42225 2.77519 2.73123 3.46354 2.40513 3.72494
3.29354 2.67741 2.69355 4.24690 2.90347 2.73739 3.18146 2.89801
2.37887 2.77519 2.98518 4.58477 3.61503
0.01934 4.35126 5.07360 0.61958 0.77255 0.48576 0.95510
139   1.66668 4.32875 3.69387 3.20579 3.53320 3.77415 4.14298
2.12316 3.25766 2.51556 3.48416 3.65214 4.17802 3.54352 3.55581
3.06552 2.99050 1.76378 5.05850 3.84007   532 a - - X
2.68618 4.42225 2.77519 2.73123 3.46354 2.40513 3.72494
3.29354 2.67741 2.69355 4.24690 2.90347 2.73739 3.18146 2.89801
2.37887 2.77519 2.98518 4.58477 3.61503
0.01934 4.35126 5.07360 0.61958 0.77255 0.48576 0.95510
140   2.42695 4.44130 3.67455 3.25930 4.07882 3.36907 4.26298
2.91533 3.18609 3.03163 4.00265 3.52169 1.14005 3.54064 3.50378
2.77118 2.83994 2.78511 5.53418 4.30014   533 p - - X
2.68618 4.42225 2.77519 2.73123 3.46354 2.40513 3.72494
3.29354 2.67741 2.69355 4.24690 2.90347 2.73739 3.18146 2.89801
2.37887 2.77519 2.98518 4.58477 3.61503
0.01934 4.35126 5.07360 0.61958 0.77255 0.48576 0.95510
141   3.18868 4.58467 4.53803 4.06013 2.79109 4.23870 4.13300
1.45371 3.87770 2.32770 3.52410 4.16222 4.58594 4.08805 4.01921
3.58467 3.43016 2.47566 4.47776 1.46399   534 i - - X
2.68618 4.42225 2.77519 2.73123 3.46354 2.40513 3.72494
3.29354 2.67741 2.69355 4.24690 2.90347 2.73739 3.18146 2.89801
2.37887 2.77519 2.98518 4.58477 3.61503
0.01934 4.35126 5.07360 0.61958 0.77255 0.48576 0.95510
142   2.84101 4.57542 3.80680 3.46116 3.80697 1.52620 4.36755
3.11583 3.39567 1.28459 3.85745 3.73764 4.20443 3.74770 3.66133
3.07633 3.20390 2.92460 5.35025 4.10193   535 l - - X
2.68618 4.42225 2.77519 2.73123 3.46354 2.40513 3.72494
3.29354 2.67741 2.69355 4.24690 2.90347 2.73739 3.18146 2.89801
2.37887 2.77519 2.98518 4.58477 3.61503
0.01934 4.35126 5.07360 0.61958 0.77255 0.48576 0.95510
143   3.64006 5.39042 4.18729 3.62838 4.87378 3.99860 4.29692
4.47479 2.59431 3.90271 4.91188 3.93959 4.49769 3.51271 0.44369
3.71539 3.88579 4.18264 5.77229 4.72268   536 R - - X
2.68618 4.42225 2.77519 2.73123 3.46354 2.40513 3.72494
3.29354 2.67741 2.69355 4.24690 2.90347 2.73739 3.18146 2.89801
2.37887 2.77519 2.98518 4.58477 3.61503
0.01934 4.35126 5.07360 0.61958 0.77255 0.48576 0.95510
144   3.49633 5.41552 3.70075 3.26387 4.91697 3.89948 4.13367
4.38635 0.52868 3.85714 4.81741 3.65648 4.38598 3.31543 2.65694
3.52172 3.72501 4.07631 5.78899 4.68737   537 K - - X
2.68618 4.42225 2.77519 2.73123 3.46354 2.40513 3.72494
3.29354 2.67741 2.69355 4.24690 2.90347 2.73739 3.18146 2.89801
2.37887 2.77519 2.98518 4.58477 3.61503
0.01934 4.35126 5.07360 0.61958 0.77255 0.48576 0.95510

145 3.29811 4.58568 5.13458 4.67310 3.84026 4.69122 5.39605
1.64668 4.56091 2.32574 3.63224 4.84899 5.01822 4.83996 4.74646
4.11344 3.57724 0.71921 5.85781 4.62880 538 v - - X
2.68618 4.42225 2.77519 2.73123 3.46354 2.40513 3.72494
3.29354 2.67741 2.69355 4.24690 2.90347 2.73739 3.18146 2.89801
2.37887 2.77519 2.98518 4.58477 3.61503
0.01934 4.35126 5.07360 0.61958 0.77255 0.48576 0.95510
146 3.24976 5.75844 0.70686 2.33541 5.09353 3.41072 4.09023
4.73135 3.22671 4.23867 5.14746 2.61861 4.09612 3.28807 3.86722
3.12784 3.58580 4.27300 6.31197 4.77918 539 d - - X
2.68618 4.42225 2.77519 2.73123 3.46354 2.40513 3.72494
3.29354 2.67741 2.69355 4.24690 2.90347 2.73739 3.18146 2.89801
2.37887 2.77519 2.98518 4.58477 3.61503
0.01934 4.35126 5.07360 0.61958 0.77255 0.48576 0.95510
147 3.65692 4.93199 5.31299 4.76425 3.22728 4.96025 5.26227
2.10625 4.57297 0.60785 2.94649 5.02073 5.07415 4.64231 4.64658
4.34784 3.87802 2.41038 5.41708 4.36927 540 L - - X
2.68618 4.42225 2.77519 2.73123 3.46354 2.40513 3.72494
3.29354 2.67741 2.69355 4.24690 2.90347 2.73739 3.18146 2.89801
2.37887 2.77519 2.98518 4.58477 3.61503
0.01934 4.35126 5.07360 0.61958 0.77255 0.48576 0.95510
148 2.61145 5.16867 3.05094 2.41963 4.51862 3.47229 3.67087
3.95521 1.48988 3.37133 4.22825 2.48917 3.92349 2.78445 2.36183
2.76643 2.88979 3.56610 5.57685 4.23746 541 k - - X
2.68618 4.42225 2.77519 2.73123 3.46354 2.40513 3.72494
3.29354 2.67741 2.69355 4.24690 2.90347 2.73739 3.18146 2.89801
2.37887 2.77519 2.98518 4.58477 3.61503
0.01934 4.35126 5.07360 0.61958 0.77255 0.48576 0.95510
149 1.84648 4.37282 3.53721 2.97647 3.54823 3.54862 3.90529
2.77967 2.92522 2.51845 2.73916 3.16965 4.00251 3.23269 3.27730
2.76772 2.20578 2.65420 4.99181 3.75745 542 a - - X
2.68618 4.42225 2.77519 2.73123 3.46354 2.40513 3.72494
3.29354 2.67741 2.69355 4.24690 2.90347 2.73739 3.18146 2.89801
2.37887 2.77519 2.98518 4.58477 3.61503
0.01934 4.35126 5.07360 0.61958 0.77255 0.48576 0.95510
150 2.80311 3.60722 3.71822 3.16627 2.86376 3.76843 2.97585
2.96713 3.09868 2.52970 3.57523 2.81097 4.14234 3.38264 3.41805
3.02745 3.03440 2.75397 4.56689 1.62434 543 y - - X
2.68618 4.42225 2.77519 2.73123 3.46354 2.40513 3.72494
3.29354 2.67741 2.69355 4.24690 2.90347 2.73739 3.18146 2.89801
2.37887 2.77519 2.98518 4.58477 3.61503
0.01934 4.35126 5.07360 0.61958 0.77255 0.48576 0.95510
151 2.65576 5.03106 2.36399 2.36638 4.46922 3.32667 3.63438
3.94298 2.07190 3.38927 4.18786 2.46133 3.83679 2.73813 2.57795
2.22544 2.76050 3.52243 5.58236 4.18225 544 k - - X
2.68618 4.42225 2.77519 2.73123 3.46354 2.40513 3.72494
3.29354 2.67741 2.69355 4.24690 2.90347 2.73739 3.18146 2.89801
2.37887 2.77519 2.98518 4.58477 3.61503
0.01934 4.35126 5.07360 0.61958 0.77255 0.48576 0.95510
152 2.39547 4.40720 3.26299 2.88889 4.39903 3.17675 4.06549
3.82731 2.93291 3.45390 4.26062 2.53479 3.88525 3.25535 3.34533
1.17149 2.56587 3.35981 5.68103 4.39710 545 s - - X
2.68618 4.42225 2.77519 2.73123 3.46354 2.40513 3.72494
3.29354 2.67741 2.69355 4.24690 2.90347 2.73739 3.18146 2.89801
2.37887 2.77519 2.98518 4.58477 3.61503
0.01934 4.35126 5.07360 0.61958 0.77255 0.48576 0.95510

153 4.01065 5.30186 4.58283 4.42361 2.48200 4.41739 3.95227
3.96534 4.27167 3.28879 4.59153 4.36362 4.88964 4.45365 4.34555
4.08855 4.30495 3.86082 4.13597 0.43493 546 Y - - X
2.68618 4.42225 2.77519 2.73123 3.46354 2.40513 3.72494
3.29354 2.67741 2.69355 4.24690 2.90347 2.73739 3.18146 2.89801
2.37887 2.77519 2.98518 4.58477 3.61503
0.01934 4.35126 5.07360 0.61958 0.77255 0.48576 0.95510
154 2.58147 5.12838 2.49563 1.96099 4.45714 3.40248 3.60515
3.78707 2.15595 3.31406 3.79564 2.87584 3.57710 2.31235 2.80434
2.62771 2.86160 3.40211 5.57049 4.16997 547 e - - X
2.68618 4.42225 2.77519 2.73123 3.46354 2.40513 3.72494
3.29354 2.67741 2.69355 4.24690 2.90347 2.73739 3.18146 2.89801
2.37887 2.77519 2.98518 4.58477 3.61503
0.01934 4.35126 5.07360 0.61958 0.77255 0.48576 0.95510
155 2.65202 5.20960 2.48822 1.81046 4.54624 3.38671 3.61349
4.02714 1.88906 3.50769 4.25719 2.79457 3.85404 2.69344 2.67355
2.67071 2.82750 3.59665 5.64084 4.23211 548 e - - X
2.68618 4.42225 2.77519 2.73123 3.46354 2.40513 3.72494
3.29354 2.67741 2.69355 4.24690 2.90347 2.73739 3.18146 2.89801
2.37887 2.77519 2.98518 4.58477 3.61503
0.01934 4.35126 5.07360 0.61958 0.77255 0.48576 0.95510
156 3.68838 4.98600 5.22645 4.71477 3.01671 4.90391 5.11271
2.39646 4.51282 0.55695 2.94047 4.95173 5.05008 4.59347 4.58867
4.30682 3.91532 2.59663 5.27088 4.08751 549 L - - X
2.68618 4.42225 2.77519 2.73123 3.46354 2.40513 3.72494
3.29354 2.67741 2.69355 4.24690 2.90347 2.73739 3.18146 2.89801
2.37887 2.77519 2.98518 4.58477 3.61503
0.01934 4.35126 5.07360 0.61958 0.77255 0.48576 0.95510
157 2.34525 4.17243 3.47278 2.87530 3.93934 3.33982 4.06215
3.42527 2.96402 3.04024 4.00398 3.34414 3.92819 3.30139 3.33847
1.10474 2.90794 3.07254 5.44775 4.18581 550 s - - X
2.68618 4.42225 2.77519 2.73123 3.46354 2.40513 3.72494
3.29354 2.67741 2.69355 4.24690 2.90347 2.73739 3.18146 2.89801
2.37887 2.77519 2.98518 4.58477 3.61503
0.01934 4.35126 5.07360 0.61958 0.77255 0.48576 0.95510
158 2.62521 4.75776 2.84869 2.55501 3.97141 3.21615 3.71522
3.32705 2.43166 2.56861 3.49316 2.98849 3.89279 2.88817 2.88706
2.12990 2.47346 2.93042 5.27869 3.67099 551 s - - X
2.68618 4.42225 2.77519 2.73123 3.46354 2.40513 3.72494
3.29354 2.67741 2.69355 4.24690 2.90347 2.73739 3.18146 2.89801
2.37887 2.77519 2.98518 4.58477 3.61503
0.01934 4.35126 5.07360 0.61958 0.77255 0.48576 0.95510
159 1.10435 4.36213 3.67007 3.13270 4.02203 3.02538 4.20340
3.30942 3.20045 3.08546 3.95960 3.47629 3.94394 3.50135 3.53218
2.61899 2.66877 2.38407 5.43231 4.21090 552 a - - X
2.68618 4.42225 2.77519 2.73123 3.46354 2.40513 3.72494
3.29354 2.67741 2.69355 4.24690 2.90347 2.73739 3.18146 2.89801
2.37887 2.77519 2.98518 4.58477 3.61503
0.01934 4.35126 5.07360 0.61958 0.77255 0.48576 0.95510
160 3.44999 4.75447 5.14941 4.61608 3.34970 4.74671 5.15598
2.12886 4.45811 0.82342 3.14269 4.83315 4.96022 4.58799 4.56660
4.12650 3.69112 1.67192 5.44352 4.34894 553 l - - X
2.68618 4.42225 2.77519 2.73123 3.46354 2.40513 3.72494
3.29354 2.67741 2.69355 4.24690 2.90347 2.73739 3.18146 2.89801
2.37887 2.77519 2.98518 4.58477 3.61503
0.01934 4.35126 5.07360 0.61958 0.77255 0.48576 0.95510

```
    161   2.68241  5.37610  2.12227  1.41153  4.69293  3.12563  3.59001
4.18774  2.40460  3.66870  4.44019  2.87853  3.90844  2.67365  3.10027
2.73190  3.09009  3.75452  5.81097  4.36860    554 e - - X
          2.68618  4.42225  2.77519  2.73123  3.46354  2.40513  3.72494
3.29354  2.67741  2.69355  4.24690  2.90347  2.73739  3.18146  2.89801
2.37887  2.77519  2.98518  4.58477  3.61503
          0.01934  4.35126  5.07360  0.61958  0.77255  0.48576  0.95510
    162   2.73830  5.23416  2.34331  2.03263  4.56952  3.44128  3.66885
4.04944  1.68464  3.31650  4.28419  2.70345  3.86761  2.61624  2.84889
2.69381  2.97271  3.62098  5.66264  4.25425    555 k - - X
          2.68618  4.42225  2.77519  2.73123  3.46354  2.40513  3.72494
3.29354  2.67741  2.69355  4.24690  2.90347  2.73739  3.18146  2.89801
2.37887  2.77519  2.98518  4.58477  3.61503
          0.02713  4.35126  4.27804  0.61958  0.77255  0.48576  0.95510
    163   3.52612  4.86689  5.04042  4.49910  3.20292  4.71824  5.01696
2.38811  4.27777  1.23011  1.16195  4.75302  4.91357  4.43340  4.38754
4.09592  3.75429  2.54898  5.30401  4.21215    556 m - - X
          2.68618  4.42225  2.77519  2.73123  3.46354  2.40513  3.72494
3.29354  2.67741  2.69355  4.24690  2.90347  2.73739  3.18146  2.89801
2.37887  2.77519  2.98518  4.58477  3.61503
          0.01949  4.34362  5.06596  0.61958  0.77255  0.49692  0.93752
    164   3.92883  5.19698  4.89634  4.72168  0.47303  4.45829  4.18247
3.42448  4.62500  2.69337  4.09528  4.58896  4.92218  4.66666  4.62647
4.15714  4.23818  3.44610  4.33306  2.65815    557 F - - X
          2.68618  4.42225  2.77519  2.73123  3.46354  2.40513  3.72494
3.29354  2.67741  2.69354  4.24690  2.90347  2.73739  3.18146  2.89801
2.37887  2.77519  2.98518  4.58477  3.61503
          0.03393  4.08039  4.10687  0.59565  0.80119  0.49692  0.93752
    165   2.64997  4.69128  3.12767  2.60281  3.71442  3.46111  3.45276
3.19180  2.57444  2.52766  3.65485  3.09783  3.90598  2.88677  2.42795
1.95435  2.71657  3.00172  5.22232  3.92393    559 s - - X
          2.68618  4.42225  2.77519  2.73123  3.46354  2.40513  3.72494
3.29354  2.67741  2.69355  4.24690  2.90347  2.73739  3.18146  2.89801
2.37887  2.77519  2.98518  4.58477  3.61503
          0.01969  4.33342  5.05576  0.61958  0.77255  0.51149  0.91529
    166   2.65766  2.78114  3.16205  2.60711  3.97997  2.50962  3.63499
3.37781  2.46180  3.01180  3.84863  3.09806  3.90696  2.84589  2.44325
2.34615  2.81448  3.07888  5.28484  3.98463    560 s - - X
          2.68618  4.42225  2.77519  2.73123  3.46354  2.40513  3.72494
3.29354  2.67741  2.69355  4.24690  2.90347  2.73739  3.18146  2.89801
2.37887  2.77519  2.98518  4.58477  3.61503
          0.01969  4.33342  5.05576  0.61958  0.77255  0.51149  0.91529
    167   3.05949  4.49428  4.46722  3.92831  1.29529  4.14169  4.24000
2.49514  3.79112  1.69023  3.24642  4.10134  3.55493  3.97291  3.94936
3.45918  3.29645  2.54453  4.67374  3.25311    561 f - - X
          2.68618  4.42225  2.77519  2.73123  3.46354  2.40513  3.72494
3.29354  2.67741  2.69355  4.24690  2.90347  2.73739  3.18146  2.89801
2.37887  2.77519  2.98518  4.58477  3.61503
          0.01969  4.33342  5.05576  0.61958  0.77255  0.48765  0.95210
    168   2.65864  4.26508  3.78758  3.22469  3.41242  3.67987  4.00521
2.18534  3.09867  2.00188  3.39311  3.55000  3.83401  3.42483  3.42313
2.67335  1.90182  2.46960  4.90223  3.69017    562 t - - X
          2.68618  4.42225  2.77520  2.73123  3.46354  2.40513  3.72495
3.29354  2.67741  2.69355  4.24690  2.90347  2.73740  3.18147  2.89801
2.37886  2.77518  2.98516  4.58477  3.61503
          0.03076  3.73036  5.06596  0.59217  0.80548  0.49692  0.93752
```

169 2.32799 4.30381 3.62723 2.89103 3.39798 3.62450 3.91544
2.06318 2.99959 2.41961 3.16018 3.40403 4.02730 3.21034 3.32483
2.55452 2.30288 2.51681 4.89803 3.67473 565 i - - X
2.68618 4.42225 2.77519 2.73123 3.46354 2.40513 3.72494
3.29354 2.67741 2.69355 4.24690 2.90347 2.73739 3.18146 2.89801
2.37887 2.77519 2.98518 4.58477 3.61503
0.15292 4.34362 2.04943 0.61958 0.77255 0.47885 0.96626
170 2.54152 4.30896 3.04705 2.50119 4.17620 1.48222 3.87538
3.58636 2.72521 3.21874 4.04758 3.11567 3.59537 3.06454 3.07741
2.55029 2.78313 3.21007 5.47263 4.17525 566 g - - X
2.68618 4.42225 2.77519 2.73123 3.46354 2.40513 3.72494
3.29354 2.67741 2.69355 4.24690 2.90347 2.73739 3.18146 2.89801
2.37887 2.77519 2.98518 4.58477 3.61503
0.04218 4.22164 3.62563 0.61958 0.77255 0.65058 0.73760
171 2.63400 4.92771 2.94087 2.41674 4.20011 3.43651 3.50778
3.63195 2.26898 3.05120 3.68840 2.62350 3.82819 1.97245 2.79181
2.56240 2.81152 3.08472 5.40306 4.05114 567 q - - X
2.68618 4.42193 2.77521 2.73130 3.46361 2.40520 3.72473
3.29342 2.67741 2.69342 4.24666 2.90353 2.73746 3.18142 2.89807
2.37878 2.77526 2.98525 4.58484 3.61502
0.24354 2.85171 1.84257 1.43208 0.27288 0.64566 0.74301
172 2.49960 2.07738 3.80735 3.25347 3.05887 3.50275 3.83640
2.62150 3.14548 2.36978 3.29836 3.52005 3.99227 3.41874 3.39401
2.87116 2.72773 2.41314 4.70588 2.95638 578 c - - X
2.68619 4.42226 2.77521 2.73124 3.46355 2.40514 3.72496
3.29355 2.67742 2.69356 4.24691 2.90348 2.73739 3.18147 2.89802
2.37880 2.77513 2.98519 4.58478 3.61504
0.13210 2.32934 3.63475 0.38533 1.14014 0.47618 0.97063
173 2.05813 4.79150 2.87397 2.39585 4.25980 2.04113 3.78989
3.68963 2.62908 3.28688 4.09727 2.81116 3.85209 2.94963 3.09332
2.24973 2.91213 3.20660 5.51567 4.17775 580 g - - X
2.68626 4.42234 2.77496 2.73124 3.46363 2.40522 3.72504
3.29363 2.67741 2.69364 4.24683 2.90330 2.73749 3.18076 2.89793
2.37893 2.77529 2.98528 4.58486 3.61512
0.26952 1.71177 2.88761 0.96591 0.47907 0.60076 0.79495
174 2.46401 4.70350 3.08004 2.55235 4.01055 3.11530 3.71831
3.41592 2.47704 2.76492 3.87060 3.04329 3.27009 2.85783 2.88385
1.80056 2.78422 3.09650 5.30515 3.99491 584 s - - X
2.68616 4.42191 2.77524 2.73126 3.46358 2.40517 3.72495
3.29358 2.67745 2.69351 4.24689 2.90351 2.73740 3.18151 2.89805
2.37865 2.77522 2.98523 4.58481 3.61507
0.31318 1.34084 4.92609 0.19616 1.72529 0.50154 0.93038
175 2.37275 4.27823 2.93625 2.38875 4.27095 3.26534 2.78641
3.71518 2.38370 3.26549 4.04498 2.51710 3.75160 2.41227 2.74332
2.49741 2.76318 3.34438 5.45800 3.96422 586 a - - X
2.68618 4.42225 2.77519 2.73123 3.46354 2.40513 3.72494
3.29354 2.67741 2.69355 4.24690 2.90347 2.73739 3.18146 2.89801
2.37887 2.77519 2.98518 4.58477 3.61503
0.02060 4.28872 5.01106 0.61958 0.77255 0.57135 0.83186
176 2.24162 4.80200 2.99587 2.58271 4.26694 1.67166 3.81663
3.68796 2.36860 3.28843 4.10708 2.84920 3.89097 2.94675 3.00863
2.62655 2.94956 3.22847 5.51668 4.19572 587 g - - X
2.68618 4.42225 2.77519 2.73123 3.46354 2.40513 3.72494
3.29354 2.67741 2.69355 4.24690 2.90347 2.73739 3.18146 2.89801
2.37887 2.77519 2.98518 4.58477 3.61503
0.02060 4.28872 5.01106 0.61958 0.77255 0.57135 0.83186

```
    177   2.35310  4.40538  3.41825  2.66597  3.44437  3.02147  3.82847
2.74109  2.79113  2.47275  2.91441  3.25478  3.86002  3.08968  3.19117
2.71422  2.27948  2.55182  4.98030  3.73503    588 t - - X
          2.68618  4.42225  2.77519  2.73123  3.46354  2.40513  3.72494
3.29354  2.67741  2.69355  4.24690  2.90347  2.73739  3.18146  2.89801
2.37887  2.77519  2.98518  4.58477  3.61503
          0.23815  4.28872  1.61850  0.61958  0.77255  0.57135  0.83186
    178   2.53688  4.17113  3.07727  2.52619  3.87787  3.21474  3.62708
3.27614  2.48924  2.81631  3.75331  2.87983  2.63131  2.68741  2.92615
2.39209  2.76690  2.84816  5.19700  3.89369    589 s - - X
          2.68618  4.42225  2.77519  2.73123  3.46354  2.40513  3.72494
3.29354  2.67741  2.69355  4.24690  2.90347  2.73739  3.18146  2.89801
2.37887  2.77519  2.98518  4.58477  3.61503
          0.02554  4.07610  4.79845  0.61958  0.77255  0.59859  0.79759
    179   2.50427  4.90017  2.92160  2.10474  4.17888  2.44403  3.64466
3.30244  2.40033  3.14885  3.98576  2.75897  3.82547  2.78224  2.84444
2.37378  2.82658  3.25962  5.40414  4.05118    590 e - - X
          2.68618  4.42225  2.77519  2.73123  3.46354  2.40513  3.72494
3.29354  2.67741  2.69355  4.24690  2.90347  2.73739  3.18146  2.89801
2.37887  2.77519  2.98518  4.58477  3.61503
          0.05133  4.18534  3.35769  0.61958  0.77255  0.46126  0.99557
    180   2.55784  4.46362  3.02333  2.34430  4.12565  3.24491  3.65939
3.37181  2.08351  3.05096  3.94301  2.98519  3.85258  2.65947  2.35512
2.64542  2.69622  2.77249  5.36721  4.02711    591 k - - X
          2.68618  4.42225  2.77519  2.73123  3.46354  2.40513  3.72494
3.29354  2.67741  2.69355  4.24690  2.90347  2.73739  3.18146  2.89801
2.37887  2.77519  2.98518  4.58477  3.61503
          0.02079  4.27936  5.00171  0.61958  0.77255  0.57679  0.82485
    181   2.80442  5.34023  1.74419  1.99313  4.65860  3.24304  3.54599
4.15399  2.40766  3.63374  4.40223  2.56882  3.87639  2.59607  3.06324
2.34948  3.05157  3.71916  5.77473  4.33273    592 d - - X
          2.68618  4.42225  2.77519  2.73123  3.46354  2.40513  3.72494
3.29354  2.67741  2.69355  4.24690  2.90347  2.73739  3.18146  2.89801
2.37887  2.77519  2.98518  4.58477  3.61503
          0.02073  4.28220  5.00455  0.61958  0.77255  0.57957  0.82130
    182   2.63071  4.23076  3.16024  2.59986  3.14639  2.78670  3.72398
3.21506  2.25793  2.85537  3.43941  3.06168  3.89331  2.73741  2.82488
2.32853  2.77758  2.86789  5.18892  3.89416    593 k - - X
          2.68618  4.42225  2.77519  2.73123  3.46354  2.40513  3.72494
3.29354  2.67741  2.69355  4.24690  2.90347  2.73739  3.18146  2.89801
2.37887  2.77519  2.98518  4.58477  3.61503
          0.02712  4.28220  4.34705  0.61958  0.77255  0.52197  0.89980
    183   2.64454  4.20360  3.84434  2.99257  3.31030  3.12552  3.98332
2.27931  3.17703  1.79823  2.48048  3.57451  4.07210  3.35942  3.43729
2.97090  2.75027  2.43524  4.80928  3.60516    594 l - - X
          2.68618  4.42225  2.77519  2.73123  3.46354  2.40513  3.72494
3.29354  2.67741  2.69355  4.24690  2.90347  2.73739  3.18146  2.89801
2.37887  2.77519  2.98518  4.58477  3.61503
          0.02032  4.30228  5.02463  0.61958  0.77255  0.51647  0.90789
    184   2.48678  4.88131  2.87679  2.32881  4.31829  3.44195  3.63557
3.50927  2.33805  3.30720  4.07923  2.38005  3.83461  2.59660  2.55219
2.24331  2.64757  3.22373  5.48863  4.11182    595 s - - X
          2.68620  4.42227  2.77495  2.73125  3.46356  2.40515  3.72496
3.29356  2.67743  2.69357  4.24692  2.90349  2.73742  3.18148  2.89803
2.37884  2.77521  2.98520  4.58479  3.61505
          0.12900  2.16664  5.04118  0.30982  1.32267  0.45806  1.00105
```

185 2.83680 5.35138 2.25384 1.37011 4.66727 3.12629 3.57499
4.15795 2.58785 3.64843 4.42346 2.80505 2.98367 2.87521 3.11273
2.68815 3.08635 3.65172 5.79820 4.35963 597 e - - X
2.68618 4.42225 2.77519 2.73123 3.46354 2.40513 3.72494
3.29354 2.67741 2.69355 4.24690 2.90347 2.73739 3.18146 2.89801
2.37887 2.77519 2.98518 4.58477 3.61503
0.02220 4.35126 4.70306 0.61958 0.77255 0.48576 0.95510
186 2.56752 4.81028 2.87371 2.22168 4.27500 2.68043 3.65725
3.66303 2.42569 3.27262 4.05471 2.83243 3.84944 2.78097 2.79194
2.07648 2.51064 3.20609 5.46965 4.02362 598 s - - X
2.68618 4.42225 2.77519 2.73123 3.46354 2.40513 3.72494
3.29354 2.67741 2.69355 4.24690 2.90347 2.73739 3.18146 2.89801
2.37887 2.77519 2.98518 4.58477 3.61503
0.03574 4.34845 3.80851 0.61958 0.77255 0.48989 0.94853
187 2.54228 4.72824 2.97378 2.18252 4.38833 3.46181 3.46558
3.84041 1.72016 3.35992 4.12860 2.92405 3.85268 2.72500 2.47954
2.53374 2.77862 3.30592 5.52192 4.14944 599 k - - X
2.68618 4.42225 2.77519 2.73123 3.46354 2.40513 3.72494
3.29354 2.67741 2.69355 4.24690 2.90347 2.73739 3.18146 2.89801
2.37887 2.77519 2.98518 4.58477 3.61503
0.03768 4.33241 3.73640 0.61958 0.77255 0.48148 0.96199
188 2.51541 4.35346 3.54726 2.97742 3.26062 3.63004 3.88323
2.63240 2.34194 1.80869 3.45530 3.37587 4.00887 3.22281 3.04130
2.76279 2.75777 2.53311 4.93842 3.70747 600 l - - X
2.68619 4.42230 2.77525 2.73129 3.46329 2.40518 3.72500
3.29335 2.67722 2.69360 4.24661 2.90344 2.73745 3.18152 2.89804
2.37884 2.77525 2.98524 4.58482 3.61508
0.33406 1.28163 5.05087 0.17127 1.84893 0.51835 0.90510
189 2.42639 4.41055 3.39993 2.87380 3.34645 3.59740 3.84307
2.82836 2.52561 2.27644 2.81643 3.23816 3.97830 3.14570 2.93371
2.38891 2.69233 2.54902 4.98548 3.67375 602 l - - X
2.68618 4.42225 2.77519 2.73123 3.46354 2.40513 3.72494
3.29354 2.67741 2.69355 4.24690 2.90347 2.73739 3.18146 2.89801
2.37887 2.77519 2.98518 4.58477 3.61503
0.01979 4.32853 5.05087 0.61958 0.77255 0.51835 0.90510
190 2.79767 5.28278 1.68565 2.32615 4.60071 2.55165 3.72524
4.08262 2.48655 3.58160 4.35582 2.32073 3.89053 2.71550 2.91668
2.74581 3.04410 3.48203 5.73555 4.31128 603 d - - X
2.68618 4.42225 2.77519 2.73123 3.46354 2.40513 3.72494
3.29354 2.67741 2.69355 4.24690 2.90347 2.73739 3.18146 2.89801
2.37887 2.77519 2.98518 4.58477 3.61503
0.03035 4.32853 4.09177 0.61958 0.77255 0.51835 0.90510
191 2.76965 4.38863 4.45128 3.88719 2.42809 4.07581 4.36711
2.40096 3.75118 1.06230 3.14659 4.08536 4.11036 3.94119 3.90632
3.34327 3.19025 2.40544 4.90306 3.68131 604 l - - X
2.68618 4.42225 2.77519 2.73123 3.46354 2.40513 3.72494
3.29354 2.67741 2.69355 4.24690 2.90347 2.73739 3.18146 2.89801
2.37887 2.77519 2.98518 4.58477 3.61503
0.02443 4.31817 4.52742 0.61958 0.77255 0.50223 0.92933
192 3.33800 4.67225 4.96611 4.39733 2.95544 4.58277 4.87740
2.13924 4.09879 0.78813 3.04713 4.61556 4.79722 4.36721 4.36990
3.92073 3.56174 2.15555 5.21858 4.10597 605 l - - X
2.68618 4.42225 2.77519 2.73123 3.46354 2.40513 3.72494
3.29354 2.67741 2.69355 4.24690 2.90347 2.73739 3.18146 2.89801
2.37887 2.77519 2.98518 4.58477 3.61503
0.01981 4.32710 5.04945 0.61958 0.77255 0.47429 0.97372

193 2.66800 5.13461 2.23420 2.36377 4.46058 3.43559 3.01702
3.93401 2.28942 3.40435 4.18143 2.36977 3.83477 2.39334 2.79661
2.57061 2.87347 3.32888 5.57707 3.84366 606 d - - X
2.68618 4.42225 2.77519 2.73123 3.46354 2.40513 3.72494
3.29354 2.67741 2.69355 4.24690 2.90347 2.73739 3.18146 2.89801
2.37887 2.77519 2.98518 4.58477 3.61503
0.01942 4.34705 5.06940 0.61958 0.77255 0.49193 0.94532
194 2.75076 4.98128 2.45081 2.51522 4.58181 1.40550 3.93187
4.04615 2.82622 3.61368 4.42409 2.91543 3.93204 3.09574 3.17434
2.09743 3.08352 3.60614 5.80926 4.43433 607 g - - X
2.68618 4.42225 2.77519 2.73123 3.46354 2.40513 3.72494
3.29354 2.67741 2.69355 4.24690 2.90347 2.73739 3.18146 2.89801
2.37887 2.77519 2.98518 4.58477 3.61503
0.01942 4.34705 5.06940 0.61958 0.77255 0.49193 0.94532
195 2.62633 4.72484 3.14704 2.63851 4.02130 3.27108 3.80006
3.48566 2.57929 3.11428 3.94539 3.11619 3.74005 2.80691 3.04206
1.41723 2.63687 3.15841 5.09569 4.07068 608 s - - X
2.68618 4.42229 2.77523 2.73127 3.46358 2.40482 3.72498
3.29358 2.67745 2.69359 4.24694 2.90351 2.73744 3.18150 2.89805
2.37882 2.77523 2.98522 4.58481 3.61507
0.23729 1.58497 5.06940 0.21071 1.66076 0.48193 0.96127
196 3.12203 5.73171 1.67744 1.03545 5.01996 3.40279 3.93775
4.54412 2.94909 4.03100 4.87496 2.85851 4.02114 3.09869 3.55070
3.00016 3.40891 3.93620 6.18280 4.66247 610 e - - X
2.68618 4.42225 2.77519 2.73123 3.46354 2.40513 3.72494
3.29354 2.67741 2.69355 4.24690 2.90347 2.73739 3.18146 2.89801
2.37887 2.77519 2.98518 4.58477 3.61503
0.01934 4.35126 5.07360 0.61958 0.77255 0.48576 0.95510
197 3.86619 5.14978 4.94975 4.67181 1.58551 4.67937 3.57706
3.68460 4.49632 2.93267 4.26030 4.32591 4.97615 4.40893 4.48614
4.04649 4.18390 3.52218 3.77299 0.72917 611 y - - X
2.68618 4.42225 2.77519 2.73123 3.46354 2.40513 3.72494
3.29354 2.67741 2.69355 4.24690 2.90347 2.73739 3.18146 2.89801
2.37887 2.77519 2.98518 4.58477 3.61503
0.01934 4.35126 5.07360 0.61958 0.77255 0.48576 0.95510
198 2.71862 4.34404 3.99324 3.46691 3.58192 3.78195 4.25233
2.37625 3.35135 2.50859 3.52195 3.75372 4.22459 3.35956 3.61835
3.10649 2.21536 1.24255 5.13912 3.92795 612 v - - X
2.68618 4.42225 2.77519 2.73123 3.46354 2.40513 3.72494
3.29354 2.67741 2.69355 4.24690 2.90347 2.73739 3.18146 2.89801
2.37887 2.77519 2.98518 4.58477 3.61503
0.01934 4.35126 5.07360 0.61958 0.77255 0.48576 0.95510
199 2.75014 4.35143 3.93441 3.39474 3.43767 3.75300 4.15863
2.56481 3.27304 1.24287 3.39014 3.69268 2.85474 3.57570 3.53630
2.93746 2.96918 2.41798 5.01614 3.81141 613 l - - X
2.68618 4.42225 2.77519 2.73123 3.46354 2.40513 3.72494
3.29354 2.67741 2.69355 4.24690 2.90347 2.73739 3.18146 2.89801
2.37887 2.77519 2.98518 4.58477 3.61503
0.01934 4.35126 5.07360 0.61958 0.77255 0.48576 0.95510
200 2.58307 4.34815 3.97096 3.54892 3.96047 3.43023 4.41693
2.84794 3.45959 2.91478 3.90202 3.70813 4.07675 3.77404 3.72688
2.73385 1.17494 1.85792 5.47697 4.26228 614 t - - X
2.68618 4.42225 2.77519 2.73123 3.46354 2.40513 3.72494
3.29354 2.67741 2.69355 4.24690 2.90347 2.73739 3.18146 2.89801
2.37887 2.77519 2.98518 4.58477 3.61503
0.01934 4.35126 5.07360 0.61958 0.77255 0.48576 0.95510

201 3.60329 4.43418 4.57111 4.27199 2.46133 4.25911 3.89717
3.59965 4.04591 3.06179 4.28613 4.21440 4.73127 4.25766 4.15844
3.75912 3.91287 3.46075 4.11975 0.56717 615 Y - - X
2.68618 4.42225 2.77519 2.73123 3.46354 2.40513 3.72494
3.29354 2.67741 2.69355 4.24690 2.90347 2.73739 3.18146 2.89801
2.37887 2.77519 2.98518 4.58477 3.61503
0.01934 4.35126 5.07360 0.61958 0.77255 0.48576 0.95510
202 3.07471 5.46157 2.52837 0.87807 4.74977 3.46517 3.93036
4.23278 2.77328 3.76276 4.65009 2.97291 4.04805 2.91521 3.22562
3.00885 3.35524 3.70841 5.94095 4.51533 616 e - - X
2.68618 4.42225 2.77519 2.73123 3.46354 2.40513 3.72494
3.29354 2.67741 2.69355 4.24690 2.90347 2.73739 3.18146 2.89801
2.37887 2.77519 2.98518 4.58477 3.61503
0.01934 4.35126 5.07360 0.61958 0.77255 0.48576 0.95510
203 3.59028 5.70934 0.42056 2.77070 5.17093 3.65316 4.46005
4.88711 3.64909 4.41509 5.42929 3.34237 4.33981 3.71706 4.21139
3.51519 3.94686 4.48203 6.23331 4.99747 617 D - - X
2.68618 4.42225 2.77519 2.73123 3.46354 2.40513 3.72494
3.29354 2.67741 2.69355 4.24690 2.90347 2.73739 3.18146 2.89801
2.37887 2.77519 2.98518 4.58477 3.61503
0.01934 4.35126 5.07360 0.61958 0.77255 0.48576 0.95510
204 2.80713 5.18034 2.94396 2.49758 4.52025 3.53286 3.68252
3.96104 1.37663 3.45616 4.24172 2.44872 3.93289 2.79945 2.48012
2.72875 3.02699 3.57563 5.58564 3.89697 618 k - - X
2.68618 4.42225 2.77519 2.73123 3.46354 2.40513 3.72494
3.29354 2.67741 2.69355 4.24690 2.90347 2.73739 3.18146 2.89801
2.37887 2.77519 2.98518 4.58477 3.61503
0.01934 4.35126 5.07360 0.61958 0.77255 0.48576 0.95510
205 3.25138 5.91960 0.95391 1.57239 5.19628 3.40398 4.01645
4.75841 3.12063 4.22573 5.10796 2.85724 4.06729 3.19347 3.77623
3.09784 3.55623 4.30244 6.36207 4.80327 619 d - - X
2.68618 4.42225 2.77519 2.73123 3.46354 2.40513 3.72494
3.29354 2.67741 2.69355 4.24690 2.90347 2.73739 3.18146 2.89801
2.37887 2.77519 2.98518 4.58477 3.61503
0.01934 4.35126 5.07360 0.61958 0.77255 0.48576 0.95510
206 2.28275 4.48803 3.29100 3.08684 4.70438 0.85389 4.43000
4.14602 3.43059 3.81509 4.63679 3.34056 3.92756 3.68898 3.76981
2.46706 3.00412 3.54669 6.01774 4.77823 620 g - - X
2.68618 4.42225 2.77519 2.73123 3.46354 2.40513 3.72494
3.29354 2.67741 2.69355 4.24690 2.90347 2.73739 3.18146 2.89801
2.37887 2.77519 2.98518 4.58477 3.61503
0.01934 4.35126 5.07360 0.61958 0.77255 0.48576 0.95510
207 3.30721 5.70644 0.61335 2.43323 5.06299 3.45674 4.17609
4.73424 3.33123 4.27036 5.20869 2.90831 4.14901 3.39045 3.95430
3.20233 3.65622 4.28896 6.27374 4.79731 621 D - - X
2.68618 4.42225 2.77519 2.73123 3.46354 2.40513 3.72494
3.29354 2.67741 2.69355 4.24690 2.90347 2.73739 3.18146 2.89801
2.37887 2.77519 2.98518 4.58477 3.61503
0.01934 4.35126 5.07360 0.61958 0.77255 0.48576 0.95510
208 2.85317 4.57728 3.73142 3.09933 3.55328 3.76165 3.86203
3.08260 2.45619 2.25782 3.66384 3.46406 4.12703 3.18285 2.24292
3.04991 3.06695 2.86849 1.85062 3.67285 622 w - - X
2.68618 4.42225 2.77519 2.73123 3.46354 2.40513 3.72494
3.29354 2.67741 2.69355 4.24690 2.90347 2.73739 3.18146 2.89801
2.37887 2.77519 2.98518 4.58477 3.61503
0.01934 4.35126 5.07360 0.61958 0.77255 0.48576 0.95510

209 3.26411 4.75396 4.66647 4.21887 3.36496 4.26820 4.83966
2.46440 3.96448 1.83544 0.93432 4.42067 4.68620 4.26671 4.12421
3.69667 3.56981 2.39452 5.34320 4.15398 623 m - - X
2.68618 4.42225 2.77519 2.73123 3.46354 2.40513 3.72494
3.29354 2.67741 2.69355 4.24690 2.90347 2.73739 3.18146 2.89801
2.37887 2.77519 2.98518 4.58477 3.61503
0.01934 4.35126 5.07360 0.61958 0.77255 0.48576 0.95510
210 3.74375 5.07852 4.96000 4.62732 3.39621 4.58724 5.13865
2.64756 4.39419 0.45084 3.31301 4.85979 4.95612 4.64163 4.48122
4.25687 4.02427 2.80429 5.42034 4.24618 624 L - - X
2.68618 4.42225 2.77519 2.73123 3.46354 2.40513 3.72494
3.29354 2.67741 2.69355 4.24690 2.90347 2.73739 3.18146 2.89801
2.37887 2.77519 2.98518 4.58477 3.61503
0.01934 4.35126 5.07360 0.61958 0.77255 0.48576 0.95510
211 2.99951 4.54152 4.82823 4.42970 3.88407 4.28713 5.18318
1.92661 4.29312 2.44663 3.71763 4.55509 4.77416 4.61373 4.48615
3.72954 3.45598 0.69246 5.80656 4.56015 625 V - - X
2.68618 4.42225 2.77519 2.73123 3.46354 2.40513 3.72494
3.29354 2.67741 2.69355 4.24690 2.90347 2.73739 3.18146 2.89801
2.37887 2.77519 2.98518 4.58477 3.61503
0.01934 4.35126 5.07360 0.61958 0.77255 0.48576 0.95510
212 3.03334 4.86986 3.75323 3.66964 4.93590 0.42146 4.82018
4.58421 3.82728 4.19592 5.12488 3.90895 4.27475 4.17631 3.69817
3.20035 3.53217 4.02631 6.04937 5.01541 626 G - - X
2.68618 4.42225 2.77519 2.73123 3.46354 2.40513 3.72494
3.29354 2.67741 2.69355 4.24690 2.90347 2.73739 3.18146 2.89801
2.37887 2.77519 2.98518 4.58477 3.61503
0.01934 4.35126 5.07360 0.61958 0.77255 0.48576 0.95510
213 3.59028 5.70934 0.42056 2.77070 5.17093 3.65316 4.46005
4.88711 3.64909 4.41509 5.42929 3.34237 4.33981 3.71706 4.21139
3.51519 3.94686 4.48203 6.23331 4.99747 627 D - - X
2.68618 4.42225 2.77519 2.73123 3.46354 2.40513 3.72494
3.29354 2.67741 2.69355 4.24690 2.90347 2.73739 3.18146 2.89801
2.37887 2.77519 2.98518 4.58477 3.61503
0.01934 4.35126 5.07360 0.61958 0.77255 0.48576 0.95510
214 3.30687 4.59232 5.10974 4.62173 3.76715 4.70719 5.31721
1.76393 4.49434 2.12904 3.57052 4.81774 5.00511 4.76603 4.67901
4.11046 3.57487 0.73396 5.77404 4.55117 628 v - - X
2.68618 4.42225 2.77519 2.73123 3.46354 2.40513 3.72494
3.29354 2.67741 2.69355 4.24690 2.90347 2.73739 3.18146 2.89801
2.37887 2.77519 2.98518 4.58477 3.61503
0.01934 4.35126 5.07360 0.61958 0.77255 0.48576 0.95510
215 3.51551 5.16293 4.17656 4.10153 5.01421 3.82493 5.11378
4.71707 4.20668 4.27537 5.36211 4.34900 0.28080 4.57809 4.37322
3.70338 3.99760 4.30069 6.03770 5.14909 629 P - - X
2.68618 4.42225 2.77519 2.73123 3.46354 2.40513 3.72494
3.29354 2.67741 2.69355 4.24690 2.90347 2.73739 3.18146 2.89801
2.37887 2.77519 2.98518 4.58477 3.61503
0.01934 4.35126 5.07360 0.61958 0.77255 0.48576 0.95510
216 4.24880 5.42805 4.89379 4.73576 3.32330 4.33122 4.61806
4.33045 4.47147 3.62991 4.93519 4.80088 4.89186 4.83078 4.50533
4.44805 4.57923 4.23115 0.30145 3.30497 630 W - - X
2.68618 4.42225 2.77519 2.73123 3.46354 2.40513 3.72494
3.29354 2.67741 2.69355 4.24690 2.90347 2.73739 3.18146 2.89801
2.37887 2.77519 2.98518 4.58477 3.61503
0.01934 4.35126 5.07360 0.61958 0.77255 0.48576 0.95510

217 2.76432 5.27820 2.42761 1.58349 4.60378 2.73228 3.09574
4.09161 2.36945 3.57440 4.33239 2.74198 3.77069 2.71784 2.95868
2.67459 3.00468 3.65995 5.71380 4.28833 631 e - - X
2.68618 4.42225 2.77519 2.73123 3.46354 2.40513 3.72494
3.29354 2.67741 2.69355 4.24690 2.90347 2.73739 3.18146 2.89801
2.37887 2.77519 2.98518 4.58477 3.61503
0.01934 4.35126 5.07360 0.61958 0.77255 0.48576 0.95510
218 3.10561 4.60304 4.65032 4.08622 3.29171 4.35539 4.68266
2.29508 3.92265 1.73103 1.17085 4.34082 4.64283 4.13812 4.09651
3.68225 3.42679 2.17384 5.19740 3.93103 632 m - - X
2.68618 4.42225 2.77519 2.73123 3.46354 2.40513 3.72494
3.29354 2.67741 2.69355 4.24690 2.90347 2.73739 3.18146 2.89801
2.37887 2.77519 2.98518 4.58477 3.61503
0.01934 4.35126 5.07360 0.61958 0.77255 0.48576 0.95510
219 3.94090 5.20688 4.90728 4.73466 0.46718 4.46700 4.18900
3.43665 4.63809 2.70396 4.10655 4.59889 4.93116 4.67774 4.63763
4.16826 4.25047 3.45871 4.33856 2.66322 633 F - - X
2.68618 4.42225 2.77519 2.73123 3.46354 2.40513 3.72494
3.29354 2.67741 2.69355 4.24690 2.90347 2.73739 3.18146 2.89801
2.37887 2.77519 2.98518 4.58477 3.61503
0.01934 4.35126 5.07360 0.61958 0.77255 0.48576 0.95510
220 2.88750 4.38716 4.56027 4.01685 3.64691 4.20830 4.68088
1.75408 3.91424 2.34181 3.52739 4.23903 4.57292 4.17154 4.12685
3.54262 2.05481 1.20156 5.35312 4.14880 634 v - - X
2.68618 4.42225 2.77519 2.73123 3.46354 2.40513 3.72494
3.29354 2.67741 2.69355 4.24690 2.90347 2.73739 3.18146 2.89801
2.37887 2.77519 2.98518 4.58477 3.61503
0.01934 4.35126 5.07360 0.61958 0.77255 0.48576 0.95510
221 2.61370 4.56759 2.34149 2.04980 4.44471 2.58107 3.66881
3.91099 2.43818 3.42703 4.19051 2.85491 3.85178 2.76938 2.93051
2.12475 2.80918 3.50319 5.58991 4.19436 635 e - - X
2.68618 4.42225 2.77519 2.73123 3.46354 2.40513 3.72494
3.29354 2.67741 2.69355 4.24690 2.90347 2.73739 3.18146 2.89801
2.37887 2.77519 2.98518 4.58477 3.61503
0.01934 4.35126 5.07360 0.61958 0.77255 0.48576 0.95510
222 2.34358 4.40256 3.63735 3.17887 4.19535 3.26156 4.20614
3.32101 3.14292 3.25375 4.10993 3.43800 3.91562 3.46108 3.49438
1.41460 1.61550 3.02368 5.56730 4.33283 636 s - - X
2.68618 4.42225 2.77519 2.73123 3.46354 2.40513 3.72494
3.29354 2.67741 2.69355 4.24690 2.90347 2.73739 3.18146 2.89801
2.37887 2.77519 2.98518 4.58477 3.61503
0.01934 4.35126 5.07360 0.61958 0.77255 0.48576 0.95510
223 2.36354 1.65519 4.57076 4.11019 3.76811 3.67339 4.67792
2.35859 3.97043 2.63673 3.69441 4.08695 4.27919 4.19956 4.11905
3.09041 3.07935 1.39573 5.39642 4.20779 637 v - - X
2.68618 4.42225 2.77519 2.73123 3.46354 2.40513 3.72494
3.29354 2.67741 2.69355 4.24690 2.90347 2.73739 3.18146 2.89801
2.37887 2.77519 2.98518 4.58477 3.61503
0.01934 4.35126 5.07360 0.61958 0.77255 0.48576 0.95510
224 3.30422 5.44348 3.73796 3.00530 4.96918 3.89182 3.75767
4.28305 0.93553 3.66257 4.52807 3.29619 4.23613 2.81065 1.72906
3.27800 3.44406 3.95547 5.65604 4.51987 638 k - - X
2.68618 4.42225 2.77519 2.73123 3.46354 2.40513 3.72494
3.29354 2.67741 2.69355 4.24690 2.90347 2.73739 3.18146 2.89801
2.37887 2.77519 2.98518 4.58477 3.61503
0.01934 4.35126 5.07360 0.61958 0.77255 0.48576 0.95510

225 3.40935 5.43850 3.92235 3.15704 4.97659 3.95263 3.81322
4.29194 1.71631 3.67961 4.57685 3.50019 4.30671 2.94885 0.83849
3.40111 3.54495 3.98702 5.66048 4.55969 639 r - - X
2.68618 4.42225 2.77519 2.73123 3.46354 2.40513 3.72494
3.29354 2.67741 2.69355 4.24690 2.90347 2.73739 3.18146 2.89801
2.37887 2.77519 2.98518 4.58477 3.61503
0.01934 4.35126 5.07360 0.61958 0.77255 0.48576 0.95510
226 3.63794 4.97580 5.10269 4.63387 3.20645 4.78307 5.11372
2.38164 4.35178 0.55606 3.01834 4.86824 5.00539 4.54780 4.44881
4.21926 3.88787 2.57109 5.34091 4.17540 640 L - - X
2.68618 4.42225 2.77519 2.73123 3.46354 2.40513 3.72494
3.29354 2.67741 2.69355 4.24690 2.90347 2.73739 3.18146 2.89801
2.37887 2.77519 2.98518 4.58477 3.61503
0.01934 4.35126 5.07360 0.61958 0.77255 0.48576 0.95510
227 3.34598 5.38635 3.83612 3.11345 4.91227 3.90987 3.80478
4.23722 1.81798 3.64719 4.53867 3.46499 4.27755 2.94234 0.84275
3.26473 3.49788 3.92670 5.64673 4.52541 641 r - - X
2.68618 4.42225 2.77519 2.73123 3.46354 2.40513 3.72494
3.29354 2.67741 2.69355 4.24690 2.90347 2.73739 3.18146 2.89801
2.37887 2.77519 2.98518 4.58477 3.61503
0.01934 4.35126 5.07360 0.61958 0.77255 0.48576 0.95510
228 3.36314 4.60198 5.28592 4.79588 3.79124 4.86370 5.48393
0.89734 4.69979 2.15381 3.55779 4.98218 5.10613 4.93326 4.86381
4.27267 3.62116 1.32641 5.85424 4.66235 642 i - - X
2.68618 4.42225 2.77519 2.73123 3.46354 2.40513 3.72494
3.29354 2.67741 2.69355 4.24690 2.90347 2.73739 3.18146 2.89801
2.37887 2.77519 2.98518 4.58477 3.61503
0.01934 4.35126 5.07360 0.61958 0.77255 0.48576 0.95510
229 3.52199 4.86828 5.02434 4.48647 3.21154 4.70543 5.00818
2.39432 4.25930 1.27741 1.12132 4.73999 4.90961 4.42680 4.37312
4.08520 3.75238 2.54971 5.30612 4.20551 643 m - - X
2.68618 4.42225 2.77519 2.73123 3.46354 2.40513 3.72494
3.29354 2.67741 2.69355 4.24690 2.90347 2.73739 3.18146 2.89801
2.37887 2.77519 2.98518 4.58477 3.61503
0.01934 4.35126 5.07360 0.61958 0.77255 0.48576 0.95510
230 3.35518 5.44733 3.82202 3.07562 4.98378 3.92009 3.78748
4.29940 0.86161 3.67983 4.56063 3.44349 4.27018 2.91680 1.75505
3.33580 3.49406 3.98069 5.66394 4.54488 644 k - - X
2.68618 4.42225 2.77519 2.73123 3.46354 2.40513 3.72494
3.29354 2.67741 2.69355 4.24690 2.90347 2.73739 3.18146 2.89801
2.37887 2.77519 2.98518 4.58477 3.61503
0.01934 4.35126 5.07360 0.61958 0.77255 0.48576 0.95510
231 2.60210 4.68174 3.16655 2.74461 4.29658 1.63153 3.66563
3.72374 2.76536 3.33950 4.15857 3.17858 3.90667 3.12068 3.00105
1.88825 2.57216 3.32160 5.57196 4.27130 645 g - - X
2.68618 4.42225 2.77519 2.73123 3.46354 2.40513 3.72494
3.29354 2.67741 2.69355 4.24690 2.90347 2.73739 3.18146 2.89801
2.37887 2.77519 2.98518 4.58477 3.61503
0.01934 4.35126 5.07360 0.61958 0.77255 0.48576 0.95510
232 2.52339 4.28736 3.52232 3.19475 4.43147 3.23945 4.27701
3.85080 2.89088 3.53877 4.38673 3.44399 3.94016 3.52142 3.43492
0.82615 2.94307 3.36879 5.75420 4.49955 646 s - - X
2.68618 4.42225 2.77519 2.73123 3.46354 2.40513 3.72494
3.29354 2.67741 2.69355 4.24690 2.90347 2.73739 3.18146 2.89801
2.37887 2.77519 2.98518 4.58477 3.61503
0.01934 4.35126 5.07360 0.61958 0.77255 0.48576 0.95510

```
    233   3.18327  5.85774  1.41665  1.14743  5.13369  3.39441  3.96589
4.68730  3.02588  4.14738  5.00226  2.71545  4.03646  3.13236  3.66158
3.03970  3.47761  4.22841  6.29297  4.73814    647 e - - X
          2.68618  4.42225  2.77519  2.73123  3.46354  2.40513  3.72494
3.29354  2.67741  2.69355  4.24690  2.90347  2.73739  3.18146  2.89801
2.37887  2.77519  2.98518  4.58477  3.61503
          0.01934  4.35126  5.07360  0.61958  0.77255  0.48576  0.95510
    234   1.30076  4.44624  3.56129  2.98759  3.76550  3.53396  4.08768
2.95182  3.05114  2.05787  3.72147  3.46513  4.05200  3.40103  3.37415
2.81961  2.98212  2.71496  5.23298  3.99308    648 a - - X
          2.68618  4.42225  2.77519  2.73123  3.46354  2.40513  3.72494
3.29354  2.67741  2.69355  4.24690  2.90347  2.73739  3.18146  2.89801
2.37887  2.77519  2.98518  4.58477  3.61503
          0.10643  4.35126  2.42965  0.61958  0.77255  0.48576  0.95510
    235   2.39562  4.26922  3.76752  3.25094  3.52239  3.59471  4.07278
1.47332  3.17765  2.55662  3.49113  3.09626  4.05860  3.47250  3.46575
2.65248  2.79254  2.35799  5.01506  3.80017    649 i - - X
          2.68618  4.42225  2.77519  2.73123  3.46354  2.40513  3.72494
3.29354  2.67741  2.69355  4.24690  2.90347  2.73739  3.18146  2.89801
2.37887  2.77519  2.98518  4.58477  3.61503
          0.06271  4.26591  3.06301  0.61958  0.77255  0.59961  0.79635
    236   2.54861  4.54357  3.06601  2.91976  4.44583  0.97448  3.91656
3.97850  3.12217  3.62183  4.47302  3.14763  3.89410  3.45185  3.46650
2.67938  2.81299  3.46810  5.76839  4.46431    650 g - - X
          2.68618  4.42225  2.77519  2.73123  3.46354  2.40513  3.72494
3.29354  2.67741  2.69355  4.24690  2.90347  2.73739  3.18146  2.89801
2.37887  2.77519  2.98518  4.58477  3.61503
          0.02196  4.22516  4.94751  0.61958  0.77255  0.64670  0.74185
    237   3.17966  4.57958  4.65489  4.09448  3.23462  4.10196  4.69000
2.16587  3.92292  0.86391  2.90907  4.34927  4.63267  4.13547  4.09081
3.69011  3.41631  2.20655  5.17670  4.04606    651 l - - X
          2.68618  4.42225  2.77519  2.73123  3.46354  2.40513  3.72494
3.29354  2.67741  2.69355  4.24690  2.90347  2.73739  3.18146  2.89801
2.37887  2.77519  2.98518  4.58477  3.61503
          0.03754  4.22516  3.80684  0.61958  0.77255  0.64670  0.74185
    238   0.96528  3.71306  3.87789  3.46763  4.18441  2.59432  4.36398
3.47886  3.40289  3.14511  4.12245  3.52797  3.82590  3.68435  3.67856
2.52655  2.64093  3.03667  5.58741  4.39908    652 a - - X
          2.68618  4.42225  2.77519  2.73123  3.46354  2.40513  3.72494
3.29354  2.67741  2.69355  4.24690  2.90347  2.73739  3.18146  2.89801
2.37887  2.77519  2.98518  4.58477  3.61503
          0.08992  4.20993  2.64299  0.61958  0.77255  0.66329  0.72393
    239   2.58704  4.56526  3.09025  2.87240  4.26374  3.23237  4.07914
3.69209  2.90583  3.32974  4.25643  3.23769  1.17354  3.11558  3.22862
2.71422  2.82110  3.29936  5.60205  4.31558    653 p - - X
          2.68618  4.42225  2.77519  2.73123  3.46354  2.40513  3.72494
3.29354  2.67741  2.69355  4.24690  2.90347  2.73739  3.18146  2.89801
2.37887  2.77519  2.98518  4.58477  3.61503
          0.04708  4.14385  3.50244  0.61958  0.77255  0.72955  0.65802
    240   3.10092  5.02920  3.64855  3.09605  4.43234  3.65598  3.86754
3.94590  2.19102  3.44904  4.40525  3.43730  4.13828  3.05577  0.85776
3.10270  3.35621  3.64054  5.47021  4.27653    654 r - - X
          2.68618  4.42225  2.77519  2.73123  3.46354  2.40513  3.72494
3.29354  2.67741  2.69355  4.24690  2.90347  2.73739  3.18146  2.89801
2.37887  2.77519  2.98518  4.58477  3.61503
          0.01649  4.11327        *  0.61958  0.77255  0.00000        *
//
HMMER3/f [3.3 | Nov 2019]
```

```
NAME  B3
ACC   PF02362.22
DESC  B3 DNA binding domain
LENG  101
ALPH  amino
RF    no
MM    no
CONS  yes
CS    yes
MAP   yes
DATE  Thu Apr 23 04:52:09 2020
NSEQ  87
EFFN  7.160614
CKSUM 2206534453
GA    30.10 30.10
TC    30.10 30.10
NC    30.00 30.00
STATS LOCAL MSV       -9.5159  0.71731
STATS LOCAL VITERBI  -10.3436  0.71731
STATS LOCAL FORWARD   -4.3590  0.71731
HMM          A        C        D        E        F        G        H
I        K        L        M        N        P        Q        R        S
T        V        W        Y
            m->m     m->i     m->d     i->m     i->i     d->m     d->d
  COMPO   2.76431  4.21684  3.01028  2.70665  2.92303  2.79235  3.70181
3.01354  2.54961  2.45966  3.63727  3.05848  3.46767  3.15259  2.81136
2.60094  2.84957  2.65603  4.01277  3.64747
          2.68618  4.42225  2.77519  2.73123  3.46354  2.40513  3.72494
3.29354  2.67741  2.69355  4.24690  2.90347  2.73739  3.18146  2.89801
2.37887  2.77519  2.98518  4.58477  3.61503
          0.01431  5.66164  4.53436  0.61958  0.77255  0.00000        *
      1   3.43940  3.81777  5.35331  4.76047  0.70292  4.59085  4.78293
2.99000  4.54818  2.39572  3.91735  4.71924  4.94947  3.93734  4.55410
3.90460  3.34705  3.05852  3.24749  2.81474      1 f - - E
          2.68618  4.42225  2.77519  2.73123  3.46354  2.40513  3.72494
3.29354  2.67741  2.69355  4.24690  2.90347  2.73739  3.18146  2.89801
2.37887  2.77519  2.98518  4.58477  3.61503
          0.07371  5.65255  2.69485  0.61958  0.77255  0.45673  1.00335
      2   2.78849  3.73278  3.60626  2.35089  2.07403  4.25849  3.92876
2.57073  3.44188  1.92943  3.51814  4.09476  4.63352  2.85235  3.47283
3.30476  2.92719  2.08286  5.41968  4.20726      2 l - - E
          2.68618  4.42225  2.77519  2.73123  3.46354  2.40513  3.72494
3.29354  2.67741  2.69355  4.24690  2.90347  2.73739  3.18146  2.89801
2.37887  2.77519  2.98518  4.58477  3.61503
          0.00554  5.59416  6.31650  0.61958  0.77255  0.37479  1.16293
      3   2.77430  5.34244  3.67779  3.04945  4.55934  4.07333  3.97716
3.09621  1.10230  2.85435  4.41973  3.62685  4.46168  3.30668  3.40793
2.54309  2.24215  2.80097  5.86020  4.54417      3 k - - E
          2.68618  4.42225  2.77519  2.73123  3.46354  2.40513  3.72494
3.29354  2.67741  2.69355  4.24690  2.90347  2.73739  3.18146  2.89801
2.37887  2.77519  2.98518  4.58477  3.61503
          0.00522  5.65443  6.37677  0.61958  0.77255  0.52427  0.89646
      4   2.61277  4.45583  3.96867  2.70960  3.30195  3.68220  4.41748
2.40158  2.67836  2.39835  4.16462  3.85047  3.59743  2.56321  3.13938
2.92604  2.35202  1.85802  5.63445  4.37977      4 v - - E
          2.68618  4.42225  2.77519  2.73123  3.46354  2.40513  3.72494
3.29354  2.67741  2.69355  4.24690  2.90347  2.73739  3.18146  2.89801
2.37887  2.77519  2.98518  4.58477  3.61503
```

```
          0.00522  5.65443  6.37677  0.61958  0.77255  0.52427  0.89646
      5   3.25368  4.93005  3.69878  2.87856  2.57503  3.97051  4.20329
3.03965  2.77476  1.39641  2.36803  3.39442  4.63024  3.87677  3.40984
2.95197  2.70356  2.71395  5.51774  3.67790      5 l - - -
          2.68632  4.41996  2.77534  2.73138  3.46368  2.40527  3.72509
3.29368  2.67755  2.69283  4.24704  2.90361  2.73754  3.18161  2.89815
2.37870  2.77534  2.98475  4.58491  3.61518
          0.23004  3.20903  1.80118  1.36410  0.29519  0.46242  0.99360
      6   2.96200  4.64120  4.64410  4.05634  3.19123  4.27256  4.57626
2.26959  2.51464  2.16561  2.83531  4.25877  4.11119  3.67939  3.21865
2.61947  1.90139  2.03701  5.25284  3.34977     11 t - - -
          2.68618  4.42225  2.77519  2.73123  3.46354  2.40513  3.72494
3.29354  2.67741  2.69355  4.24690  2.90347  2.73739  3.18146  2.89801
2.37887  2.77519  2.98518  4.58477  3.61503
          0.00618  5.48463  6.20697  0.61958  0.77255  0.90743  0.51678
      7   2.54178  5.60333  3.38481  2.82887  4.03241  3.90234  3.30354
3.92268  2.43601  3.90311  4.64740  2.59708  1.92388  2.69418  2.71938
2.48362  1.93225  3.98710  6.04371  4.64073     12 p - - H
          2.68618  4.42225  2.77519  2.73123  3.46354  2.40513  3.72494
3.29354  2.67741  2.69355  4.24690  2.90347  2.73739  3.18146  2.89801
2.37887  2.77519  2.98518  4.58477  3.61503
          0.00599  5.51604  6.23839  0.61958  0.77255  0.24985  1.50923
      8   2.38204  5.47447  3.41573  2.94347  4.72916  4.03524  3.59543
3.01023  2.74053  2.67663  4.16021  2.79092  2.39908  3.37327  3.28171
1.37583  3.34118  3.18820  5.96540  4.61670     13 s - - H
          2.68618  4.42225  2.77519  2.73123  3.46354  2.40513  3.72494
3.29354  2.67741  2.69355  4.24690  2.90347  2.73739  3.18146  2.89801
2.37887  2.77519  2.98518  4.58477  3.61503
          0.00518  5.66164  6.38398  0.61958  0.77255  0.48576  0.95510
      9   3.03034  5.65330  1.52659  2.85133  4.97230  3.31604  2.93364
4.44031  2.92669  3.94742  3.49398  2.88685  3.16145  3.28634  3.41476
1.86289  3.30200  3.71236  5.16798  2.64316     14 d - - H
          2.68618  4.42225  2.77519  2.73123  3.46354  2.40513  3.72494
3.29354  2.67741  2.69355  4.24690  2.90347  2.73739  3.18146  2.89801
2.37887  2.77519  2.98518  4.58477  3.61503
          0.00518  5.66164  6.38398  0.61958  0.77255  0.48576  0.95510
     10   2.73744  4.70694  2.88783  2.84021  3.56517  3.51812  3.60063
3.63986  3.21720  2.81894  2.91315  2.60831  4.50655  3.55222  2.88854
2.48112  2.88955  1.60184  5.75887  3.39249     15 v - - H
          2.68618  4.42225  2.77519  2.73123  3.46354  2.40513  3.72494
3.29354  2.67741  2.69355  4.24690  2.90347  2.73739  3.18146  2.89801
2.37887  2.77519  2.98518  4.58477  3.61503
          0.00518  5.66164  6.38398  0.61958  0.77255  0.48576  0.95510
     11   3.11714  5.08240  3.62673  2.73236  2.44510  2.28138  3.89777
2.83673  2.97817  2.04905  3.27313  2.97459  4.56100  3.68403  3.43912
2.43631  2.69858  2.66542  5.64773  3.65609     16 l - - S
          2.68618  4.42225  2.77519  2.73123  3.46354  2.40513  3.72494
3.29354  2.67741  2.69355  4.24690  2.90347  2.73739  3.18146  2.89801
2.37887  2.77519  2.98518  4.58477  3.61503
          0.00518  5.66164  6.38398  0.61958  0.77255  0.48576  0.95510
     12   2.67668  5.71288  3.19457  2.35088  3.93668  2.97566  3.72541
4.20826  1.88095  3.23817  4.08811  2.71153  3.89564  3.06954  2.47506
1.86382  2.72079  4.10314  6.14599  4.08924     17 s - - T
          2.68618  4.42225  2.77519  2.73123  3.46354  2.40513  3.72494
3.29354  2.67741  2.69355  4.24690  2.90347  2.73739  3.18146  2.89801
2.37887  2.77519  2.98518  4.58477  3.61503
          0.01383  5.66164  4.57967  0.61958  0.77255  0.48576  0.95510
```

```
      13   3.10304  5.01611  3.20845  2.58959  5.02800  2.88187  2.89769
 3.57771  2.40072  2.27823  4.06881  2.31506  4.36978  2.91978  2.59152
 2.12507  2.57250  4.07731  6.12857  3.81980      18 s - - T
           2.68620  4.42227  2.77521  2.73125  3.46356  2.40514  3.72496
 3.29356  2.67743  2.69357  4.24691  2.90348  2.73741  3.18148  2.89767
 2.37889  2.77521  2.98520  4.58479  3.61505
           0.05622  3.57799  3.62184  0.32330  1.28648  0.53153  0.88601
      14   2.50568  3.27910  2.23213  2.73903  3.47181  1.83483  3.05080
 3.78187  2.66317  3.96848  4.02272  2.38852  4.35689  3.05864  2.82473
 2.67210  3.41669  4.05254  6.10788  4.70406      20 g - - T
           2.68618  4.42225  2.77519  2.73123  3.46354  2.40513  3.72494
 3.29354  2.67741  2.69355  4.24690  2.90347  2.73739  3.18146  2.89801
 2.37887  2.77519  2.98518  4.58477  3.61503
           0.12105  5.62768  2.20356  0.61958  0.77255  0.65334  0.73460
      15   2.96529  3.92213  3.14587  2.72751  3.71913  2.57320  4.13251
 3.87700  2.74765  2.66545  3.38509  2.71286  4.32710  2.35077  1.69295
 2.86768  3.07751  3.79544  4.19014  4.13033      21 r - - E
           2.68618  4.42225  2.77519  2.73123  3.46354  2.40513  3.72494
 3.29354  2.67741  2.69355  4.24690  2.90347  2.73739  3.18146  2.89801
 2.37887  2.77519  2.98518  4.58477  3.61503
           0.00601  5.51265  6.23500  0.61958  0.77255  0.38999  1.13029
      16   3.29905  4.67809  5.22288  4.61019  2.56104  4.46583  4.79649
 2.25655  3.29304  1.06352  2.25952  4.61503  4.82630  4.07354  3.22728
 3.77604  3.53082  2.28310  5.29625  4.12269      22 l - - E
           2.68624  4.42231  2.77526  2.73129  3.46360  2.40519  3.72501
 3.29245  2.67747  2.69361  4.24696  2.90353  2.73746  3.18153  2.89807
 2.37893  2.77526  2.98525  4.58483  3.61430
           0.03356  3.46555  6.35003  0.92421  0.50558  0.39364  1.12268
      17   2.86491  4.38806  4.04472  3.79361  3.38460  3.30754  4.16475
 2.53560  3.31721  2.49736  3.53294  4.29691  4.23427  4.18102  2.77277
 2.09280  2.54797  1.66137  4.33299  2.70190      26 v - - E
           2.68618  4.42225  2.77519  2.73123  3.46354  2.40513  3.72494
 3.29354  2.67741  2.69355  4.24690  2.90347  2.73739  3.18146  2.89801
 2.37887  2.77519  2.98518  4.58477  3.61503
           0.05031  5.66164  3.08809  0.61958  0.77255  0.48576  0.95510
      18   3.69293  5.46510  6.25426  5.68821  2.90666  5.60258  6.00358
 0.98602  5.54052  1.09435  3.59249  5.77628  5.79055  5.56061  5.53501
 4.97160  4.44502  2.07859  6.22281  5.15554      27 i - - E
           2.68618  4.42225  2.77519  2.73123  3.46354  2.40513  3.72494
 3.29354  2.67741  2.69355  4.24690  2.90347  2.73739  3.18146  2.89801
 2.37887  2.77519  2.98518  4.58477  3.61503
           0.00542  5.61674  6.33909  0.61958  0.77255  0.70079  0.68556
      19   3.29691  4.04237  4.56138  4.02941  4.40714  3.33346  4.85430
 3.77435  3.93593  3.01976  4.38473  4.28354  0.56389  4.23315  3.95721
 2.76980  3.43701  3.50353  4.97766  4.64854      28 P - - -
           2.68618  4.42225  2.77519  2.73123  3.46354  2.40513  3.72494
 3.29354  2.67741  2.69355  4.24690  2.90347  2.73739  3.18146  2.89801
 2.37887  2.77519  2.98518  4.58477  3.61503
           0.00542  5.61674  6.33909  0.61958  0.77255  0.70079  0.68556
      20   2.97929  5.60356  2.93055  2.91124  4.91313  3.05468  3.92281
 3.40031  1.67785  2.82926  3.29696  2.78742  3.07045  3.27047  2.08078
 2.84074  3.27194  3.11989  6.05809  3.39229      29 k - - H
           2.68618  4.42225  2.77519  2.73123  3.46354  2.40513  3.72494
 3.29354  2.67741  2.69355  4.24690  2.90347  2.73739  3.18146  2.89801
 2.37887  2.77519  2.98518  4.58477  3.61503
           0.00542  5.61674  6.33909  0.61958  0.77255  0.37154  1.17013
```

21 2.77084 5.71610 3.00171 2.40899 3.61884 3.33631 3.27256
3.27157 2.03301 3.80163 4.75657 2.78656 2.99503 2.20446 2.42875
2.14821 3.28840 4.10712 6.14836 4.45162 30 k - - H
2.68618 4.42225 2.77519 2.73123 3.46354 2.40513 3.72494
3.29354 2.67741 2.69355 4.24690 2.90347 2.73739 3.18146 2.89801
2.37887 2.77519 2.98518 4.58477 3.61503
0.00518 5.66164 6.38398 0.61958 0.77255 0.48576 0.95510
22 2.51039 3.51515 3.46230 3.79902 1.02892 4.37021 2.77188
3.22666 3.98185 2.64923 3.88884 4.32451 4.74063 3.97400 4.18366
3.18113 3.51136 3.02991 4.22482 2.76764 31 f - - H
2.68625 4.42232 2.77526 2.73130 3.46360 2.40498 3.72501
3.29361 2.67747 2.69361 4.24696 2.90353 2.73746 3.18153 2.89807
2.37873 2.77511 2.98487 4.58484 3.61510
0.07421 3.22762 3.44605 0.77378 0.61853 0.48576 0.95510
23 1.30456 2.56677 5.14722 4.53772 3.75101 3.18603 4.75816
2.57623 4.34130 2.15637 3.08232 4.14438 4.79356 4.48864 4.37979
3.22264 2.54688 2.13789 5.27808 3.80580 35 a - - H
2.68618 4.42225 2.77519 2.73123 3.46354 2.40513 3.72494
3.29354 2.67741 2.69355 4.24690 2.90347 2.73739 3.18146 2.89801
2.37887 2.77519 2.98518 4.58477 3.61503
0.17073 5.63099 1.87495 0.61958 0.77255 0.49873 0.93471
24 2.46016 5.60979 2.82478 1.83731 4.95161 3.88184 4.06922
3.42016 2.04218 3.58802 4.65131 3.04969 4.27524 3.16661 1.98698
2.43010 2.73282 3.16422 6.04441 4.63544 36 e - - H
2.68618 4.42225 2.77519 2.73123 3.46354 2.40513 3.72494
3.29354 2.67741 2.69355 4.24690 2.90347 2.73739 3.18146 2.89801
2.37887 2.77519 2.98518 4.58477 3.61503
0.01637 5.48687 4.41525 0.61958 0.77255 0.22951 1.58437
25 2.30489 5.71202 2.60652 1.90332 4.31562 3.46321 3.18583
4.54491 1.98503 3.84202 4.33796 2.99635 4.36571 2.82250 2.45005
2.23166 2.94369 3.64488 6.14405 4.73245 37 e - - H
2.68618 4.42225 2.77519 2.73123 3.46354 2.40513 3.72494
3.29354 2.67741 2.69355 4.24690 2.90347 2.73739 3.18146 2.89801
2.37887 2.77519 2.98518 4.58477 3.61503
0.00522 5.65314 6.37548 0.61958 0.77255 0.53098 0.88680
26 3.27212 3.39979 4.22197 4.11394 2.98595 3.88739 1.95514
2.38555 3.99978 2.70883 3.45080 1.78839 4.73860 3.93757 4.19173
3.11125 3.50414 2.72399 5.36882 2.11224 38 n - - H
2.68656 4.42297 2.77471 2.73176 3.46099 2.40620 3.72632
3.29292 2.67839 2.69070 4.24547 2.90416 2.73397 3.18251 2.89912
2.37959 2.77658 2.98479 4.58473 3.61597
1.06999 1.19476 1.03787 1.71925 0.19748 0.53098 0.88680
27 2.18534 4.29584 2.98514 3.03294 2.35875 3.45663 4.11678
3.09938 2.72320 2.17844 3.77775 3.36018 3.53105 3.07321 3.14546
2.12977 3.02272 3.22575 5.47940 3.85043 47 s - - T
2.68618 4.42225 2.77519 2.73123 3.46354 2.40513 3.72494
3.29354 2.67741 2.69355 4.24690 2.90347 2.73739 3.18146 2.89801
2.37887 2.77519 2.98518 4.58477 3.61503
0.12085 5.21849 2.22180 0.61958 0.77255 0.19793 1.71716
28 2.38551 5.65530 2.96579 2.23689 4.59883 1.64516 3.83486
4.20337 2.34961 3.73485 3.79816 3.08939 3.28657 2.65496 2.98563
2.62269 3.15722 3.36895 6.08874 4.67855 48 g - - T
2.68618 4.42225 2.77519 2.73123 3.46354 2.40513 3.72494
3.29354 2.67741 2.69355 4.24690 2.90347 2.73739 3.18146 2.89801
2.37887 2.77519 2.98518 4.58477 3.61503
0.00572 5.56220 6.28455 0.61958 0.77255 0.68958 0.69673

29 2.74350 5.37620 2.88744 2.79980 4.10821 2.23184 3.80657
2.54573 2.53178 1.89938 3.45645 3.02073 3.82852 3.26603 3.32707
2.51661 3.40966 3.49150 5.87948 3.27648 49 l - - T
2.68618 4.42225 2.77519 2.73123 3.46354 2.40513 3.72494
3.29354 2.67741 2.69355 4.24690 2.90347 2.73739 3.18146 2.89801
2.37887 2.77519 2.98518 4.58477 3.61503
0.01884 5.59131 4.20419 0.61958 0.77255 0.80140 0.59548
30 2.91809 5.66259 2.67578 2.33155 5.00571 3.26953 3.78262
2.78643 2.13744 3.50840 4.70376 2.53125 2.56853 2.48893 3.27054
2.53742 3.01676 2.60760 6.09644 4.68666 50 k - - E
2.68618 4.42225 2.77519 2.73123 3.46354 2.40513 3.72494
3.29354 2.67741 2.69355 4.24690 2.90347 2.73739 3.18146 2.89801
2.37887 2.77519 2.98518 4.58477 3.61503
0.25582 5.57810 1.50537 0.61958 0.77255 0.34942 1.22111
31 2.60644 5.57676 3.32746 1.93326 4.91809 3.85032 3.34801
3.72064 1.74472 3.88040 4.61842 3.18250 2.28337 2.83765 3.05222
2.67942 2.46480 3.40052 6.01164 4.60307 51 k - - E
2.68618 4.42225 2.77519 2.73123 3.46354 2.40513 3.72494
3.29354 2.67741 2.69355 4.24690 2.90347 2.73739 3.18146 2.89801
2.37887 2.77519 2.98518 4.58477 3.61503
0.06412 5.42636 2.85237 0.61958 0.77255 1.24921 0.33790
32 2.12208 5.45340 3.34173 2.00698 4.75673 2.63430 4.03184
3.97111 2.64180 2.98235 4.12193 3.05515 2.70205 3.14357 2.37136
2.77575 2.54920 3.15896 5.00558 4.52764 52 e - - E
2.68610 4.42249 2.77508 2.73086 3.46378 2.40517 3.72519
3.29378 2.67765 2.69379 4.24714 2.90371 2.73764 3.18170 2.89825
2.37820 2.77480 2.98508 4.58501 3.61527
0.09398 2.64328 3.98588 1.59290 0.22732 0.28837 1.38424
33 2.94437 3.70586 3.15039 2.04325 3.86971 2.54071 3.89089
3.09308 2.15529 2.54162 4.44797 3.09295 4.34706 2.46054 2.47271
2.89212 2.71831 4.03757 6.09467 4.69179 60 e - - C
2.68618 4.42225 2.77519 2.73123 3.46354 2.40513 3.72494
3.29354 2.67741 2.69355 4.24690 2.90347 2.73739 3.18146 2.89801
2.37887 2.77519 2.98518 4.58477 3.61503
0.12935 5.60915 2.13987 0.61958 0.77255 0.73216 0.65560
34 2.77478 4.39171 2.85315 2.43077 4.41168 2.39967 3.53049
4.42845 2.69431 3.03457 4.64755 3.04965 2.46618 2.76560 3.16321
1.77366 2.62428 3.99367 6.04120 4.63317 61 s - - E
2.68618 4.42225 2.77519 2.73123 3.46354 2.40513 3.72494
3.29354 2.67741 2.69355 4.24690 2.90347 2.73739 3.18146 2.89801
2.37887 2.77519 2.98518 4.58477 3.61503
0.18660 5.48598 1.79531 0.61958 0.77255 1.11721 0.39629
35 2.66337 4.91371 3.71013 2.76775 2.89637 3.07538 4.19300
2.53805 2.08424 2.85900 2.79033 3.40606 4.34383 2.77500 2.91874
3.18738 2.74328 2.25933 5.47352 3.89939 62 k - - C
2.68618 4.42225 2.77519 2.73123 3.46354 2.40513 3.72494
3.29354 2.67741 2.69355 4.24690 2.90347 2.73739 3.18146 2.89801
2.37887 2.77519 2.98518 4.58477 3.61503
0.00739 5.30678 6.02912 0.61958 0.77255 0.50983 0.91779
36 2.81311 5.50011 2.68048 1.70696 4.78875 3.93525 4.12940
3.35123 2.20540 2.51443 3.45678 3.42901 4.32717 3.24787 3.37217
2.62418 1.93494 3.32688 5.96817 4.59410 63 e - - E
2.68618 4.42225 2.77519 2.73123 3.46354 2.40513 3.72494
3.29354 2.67741 2.69355 4.24690 2.90347 2.73739 3.18146 2.89801
2.37887 2.77519 2.98518 4.58477 3.61503
0.00590 5.53154 6.25389 0.61958 0.77255 0.26249 1.46590

```
     37   2.43863  4.75106  5.36435  4.75512  3.56119  4.57953  4.92435
1.52783  4.17009  1.62068  2.48484  4.74325  4.93596  4.67801  4.55632
3.89512  3.10282  1.55413  5.40957  4.23395     64 i - - E
          2.68618  4.42225  2.77519  2.73123  3.46354  2.40513  3.72494
3.29354  2.67741  2.69355  4.24690  2.90347  2.73739  3.18146  2.89801
2.37887  2.77519  2.98518  4.58477  3.61503
          0.00518  5.66164  6.38398  0.61958  0.77255  0.48576  0.95510
     38   2.85014  4.45540  3.11965  2.92838  3.65861  4.09486  4.30964
2.74723  2.35014  2.71721  3.19076  3.20375  4.08947  2.97405  2.60934
2.85455  1.93927  2.27272  5.81491  3.86681     65 t - - E
          2.68618  4.42225  2.77519  2.73123  3.46354  2.40513  3.72494
3.29354  2.67741  2.69355  4.24690  2.90347  2.73739  3.18146  2.89801
2.37887  2.77519  2.98518  4.58477  3.61503
          0.05031  5.66164  3.08809  0.61958  0.77255  0.48576  0.95510
     39   2.90088  4.93980  5.58023  4.97297  2.40153  4.80825  5.14970
2.17412  4.76918  0.90707  2.73716  4.97547  5.13036  4.86777  4.76841
4.13057  3.48736  1.96174  5.57530  4.43003     66 l - - E
          2.68618  4.42225  2.77519  2.73123  3.46354  2.40513  3.72494
3.29354  2.67741  2.69355  4.24690  2.90347  2.73739  3.18146  2.89801
2.37887  2.77519  2.98518  4.58477  3.61503
          0.00542  5.61674  6.33909  0.61958  0.77255  0.70079  0.68556
     40   2.90447  5.66374  3.04296  1.82921  4.99793  3.74423  4.14533
4.47629  2.13558  2.20173  2.96723  2.80416  4.35034  2.71315  2.40932
3.14663  3.10283  2.92487  6.10298  3.80878     67 e - - E
          2.68618  4.42225  2.77519  2.73123  3.46354  2.40513  3.72494
3.29354  2.67741  2.69355  4.24690  2.90347  2.73739  3.18146  2.89801
2.37887  2.77519  2.98518  4.58477  3.61503
          0.00542  5.61674  6.33909  0.61958  0.77255  0.37154  1.17013
     41   2.96630  5.71552  1.34659  2.81993  5.05926  2.84240  3.52965
4.54450  2.90431  4.02027  4.75670  2.29922  4.10856  3.05393  3.04670
2.24816  2.54290  3.41138  6.14897  4.73845     68 d - - E
          2.68621  4.42228  2.77523  2.73127  3.46330  2.40516  3.72498
3.29338  2.67744  2.69358  4.24693  2.90350  2.73743  3.18103  2.89804
2.37890  2.77523  2.98522  4.58480  3.61506
          0.07524  3.04841  3.68700  0.23329  1.56986  0.48576  0.95510
     42   3.08063  5.67722  3.44989  1.94441  5.01198  2.91618  4.15713
3.48480  2.25598  2.37842  4.72060  3.07424  2.47986  2.73368  2.44541
2.43634  3.33249  3.17720  6.11611  4.29813     70 e - - T
          2.68618  4.42225  2.77519  2.73123  3.46354  2.40513  3.72494
3.29354  2.67741  2.69355  4.24690  2.90347  2.73739  3.18146  2.89801
2.37887  2.77519  2.98518  4.58477  3.61503
          0.00530  5.63800  6.36035  0.61958  0.77255  0.60590  0.78874
     43   2.75549  5.70033  2.32355  2.40721  4.34153  2.97948  2.78339
3.88108  2.26688  3.28152  4.74106  2.80550  4.35759  2.92840  2.34275
2.09720  3.06889  4.09084  3.69574  4.18651     71 s - - T
          2.68618  4.42225  2.77519  2.73123  3.46354  2.40513  3.72494
3.29354  2.67741  2.69355  4.24690  2.90347  2.73739  3.18146  2.89801
2.37887  2.77519  2.98518  4.58477  3.61503
          0.00530  5.63800  6.36035  0.61958  0.77255  0.60590  0.78874
     44   3.17238  5.68900  3.32927  2.74858  5.01666  0.73813  4.30891
4.47176  3.08942  4.00831  3.78846  3.03619  4.48244  3.43169  3.25210
2.69719  3.37623  4.08115  6.18891  4.80546     72 g - - S
          2.68647  4.42254  2.77548  2.73152  3.46357  2.40541  3.72523
3.29383  2.67769  2.69384  4.24656  2.90375  2.73768  3.18175  2.89761
2.37663  2.77548  2.98547  4.58506  3.61532
          0.08812  2.49353  6.36035  1.41347  0.27878  0.41736  1.07523
```

```
     45   2.93410  4.79090  3.33389  2.55754  4.23387  3.69971  3.65313
3.74338  1.68375  2.95734  4.32312  2.55330  4.37704  2.88463  1.76460
2.71526  3.04984  3.22694  6.12520  4.36397     80 k - - E
          2.68618  4.42225  2.77519  2.73123  3.46354  2.40513  3.72494
3.29354  2.67741  2.69355  4.24690  2.90347  2.73739  3.18146  2.89801
2.37887  2.77519  2.98518  4.58477  3.61503
          0.00518  5.66164  6.38398  0.61958  0.77255  0.48576  0.95510
     46   2.74389  5.67182  3.47253  2.20344  4.47021  3.98672  4.17576
3.81335  1.92384  3.42359  4.71734  3.46157  3.48936  2.77276  2.72424
1.77717  2.36681  2.63505  6.11564  4.71605     81 s - - E
          2.68618  4.42225  2.77519  2.73123  3.46354  2.40513  3.72494
3.29354  2.67741  2.69355  4.24690  2.90347  2.73739  3.18146  2.89801
2.37887  2.77519  2.98518  4.58477  3.61503
          0.00518  5.66164  6.38398  0.61958  0.77255  0.48576  0.95510
     47   3.75897  4.54970  5.53143  4.99144  2.25763  4.85880  4.76098
3.34474  4.78361  3.24275  3.73099  4.89977  5.20837  4.86825  3.98085
3.90253  3.98837  3.38474  0.56944  2.99437     82 W - - E
          2.68618  4.42225  2.77519  2.73123  3.46354  2.40513  3.72494
3.29354  2.67741  2.69355  4.24690  2.90347  2.73739  3.18146  2.89801
2.37887  2.77519  2.98518  4.58477  3.61503
          0.00518  5.66164  6.38398  0.61958  0.77255  0.48576  0.95510
     48   3.03028  4.34107  2.56475  1.94284  4.56019  3.97762  3.96800
4.26464  2.11420  3.17829  4.45383  2.80099  3.07816  2.78817  2.37087
2.43671  2.43794  3.87156  6.14716  3.78185     83 e - - E
          2.68618  4.42225  2.77519  2.73123  3.46354  2.40513  3.72494
3.29354  2.67741  2.69355  4.24690  2.90347  2.73739  3.18146  2.89801
2.37887  2.77519  2.98518  4.58477  3.61503
          0.00518  5.66164  6.38398  0.61958  0.77255  0.48576  0.95510
     49   2.79838  4.67281  5.23275  4.61948  1.64099  4.46177  4.79465
2.79706  4.41032  2.28959  2.83725  4.61585  3.73091  4.54649  4.42779
2.50264  3.03260  1.34642  5.29578  4.11924     84 v - - E
          2.68618  4.42225  2.77519  2.73123  3.46354  2.40513  3.72494
3.29354  2.67741  2.69355  4.24690  2.90347  2.73739  3.18146  2.89801
2.37887  2.77519  2.98518  4.58477  3.61503
          0.00518  5.66164  6.38398  0.61958  0.77255  0.48576  0.95510
     50   2.61905  5.70719  3.18867  2.31578  5.04904  3.20170  3.70757
4.15337  1.89068  3.32123  3.78631  2.98771  4.37253  2.61474  2.02803
2.76656  3.19520  2.68192  6.14180  3.11105     85 k - - E
          2.68618  4.42225  2.77519  2.73123  3.46354  2.40513  3.72494
3.29354  2.67741  2.69355  4.24690  2.90347  2.73739  3.18146  2.89801
2.37887  2.77519  2.98518  4.58477  3.61503
          0.00518  5.66164  6.38398  0.61958  0.77255  0.48576  0.95510
     51   3.29173  2.94339  5.26618  4.65009  2.89176  4.46219  2.55072
2.52259  4.43248  1.51493  3.08994  4.62811  4.82571  4.56151  4.43675
3.77394  3.07644  2.02837  4.56710  1.95335     86 l - - E
          2.68622  4.42229  2.77524  2.73127  3.46358  2.40517  3.72499
3.29325  2.67745  2.69359  4.24694  2.90328  2.73744  3.18151  2.89805
2.37891  2.77524  2.98523  4.58481  3.61435
          0.56886  3.14224  0.93996  0.43422  1.04347  0.48576  0.95510
     52   2.79655  3.63670  3.72867  3.16392  3.43156  3.91841  3.34434
2.00220  2.41005  2.54106  3.87488  3.60234  4.30018  2.81953  2.31948
2.25187  3.20362  2.68130  5.34656  4.09633     89 i - - E
          2.68618  4.42225  2.77519  2.73123  3.46354  2.40513  3.72494
3.29354  2.67741  2.69355  4.24690  2.90347  2.73739  3.18146  2.89801
2.37887  2.77519  2.98518  4.58477  3.61503
          0.05831  5.16905  2.97682  0.61958  0.77255  1.62536  0.21920
```

```
     53   2.95886  4.51178  4.18301  3.60522  2.42784  3.45657  3.21984
2.62181  2.79314  2.40420  3.40415  3.38605  4.39096  3.60034  3.38432
2.82782  3.19078  2.64863  3.11470  1.86664     90 y - - E
          2.68644  4.42251  2.77533  2.73127  3.46380  2.40473  3.72520
3.29380  2.67766  2.69351  4.24715  2.90372  2.73707  3.18172  2.89791
2.37903  2.77483  2.98532  4.58044  3.61529
          0.29252  1.38343  5.84201  0.80001  0.59661  0.12452  2.14490
     54   2.92334  3.97314  2.56728  2.71329  4.34198  3.22036  4.17751
3.55023  2.32140  3.10160  4.71131  2.40701  4.38108  2.96423  2.05892
2.42318  2.72703  3.65717  2.93715  3.88277     96 r - - E
          2.68618  4.42225  2.77519  2.73123  3.46354  2.40513  3.72494
3.29354  2.67741  2.69355  4.24690  2.90347  2.73739  3.18146  2.89801
2.37887  2.77519  2.98518  4.58477  3.61503
          0.00518  5.66164  6.38398  0.61958  0.77255  0.48576  0.95510
     55   2.76565  5.70641  3.31026  2.64071  5.04792  2.72475  4.16674
3.78813  1.50830  4.01017  4.28431  2.24079  2.99940  3.15603  2.28891
2.98727  3.17143  2.99286  6.14123  4.03015     97 k - - T
          2.68618  4.42225  2.77519  2.73123  3.46354  2.40513  3.72494
3.29354  2.67741  2.69355  4.24690  2.90347  2.73739  3.18146  2.89801
2.37887  2.77519  2.98518  4.58477  3.61503
          0.00518  5.66164  6.38398  0.61958  0.77255  0.48576  0.95510
     56   2.90420  4.05664  2.75917  2.73410  5.06151  2.51708  3.47330
3.95347  1.95116  4.02138  4.75635  2.13476  4.37084  2.84389  2.63733
1.99895  2.72064  4.10680  6.14817  4.33035     98 k - - T
          2.68618  4.42225  2.77519  2.73123  3.46354  2.40513  3.72494
3.29354  2.67741  2.69355  4.24690  2.90347  2.73739  3.18146  2.89801
2.37887  2.77519  2.98518  4.58477  3.61503
          0.02458  5.66164  3.87264  0.61958  0.77255  0.48576  0.95510
     57   2.79005  3.92596  2.82344  2.78611  5.02936  2.46105  4.15691
4.51125  2.36157  3.50304  4.09347  3.34799  2.67863  3.03602  2.32230
1.57325  3.09133  3.91089  6.12628  4.31958     99 s - - C
          2.68618  4.42225  2.77519  2.73123  3.46354  2.40513  3.72494
3.29354  2.67741  2.69355  4.24690  2.90347  2.73739  3.18146  2.89801
2.37887  2.77519  2.98518  4.58477  3.61503
          0.01068  5.64234  4.95123  0.61958  0.77255  0.42830  1.05445
     58   3.12422  3.44780  2.91893  2.18135  5.05537  2.07873  4.16185
3.86204  2.54087  3.85490  3.74499  2.39256  4.36822  2.31745  2.16545
2.65537  2.98407  4.10110  6.14355  4.73281    100 g - - C
          2.68618  4.42225  2.77519  2.73123  3.46354  2.40513  3.72494
3.29354  2.67741  2.69355  4.24690  2.90347  2.73739  3.18146  2.89801
2.37887  2.77519  2.98518  4.58477  3.61503
          0.01070  5.65637  4.94081  0.61958  0.77255  0.51406  0.91147
     59   3.04945  5.66694  2.77325  2.55911  3.53035  2.25850  3.67632
4.17786  2.48432  3.96461  2.97542  3.45475  4.37319  2.79649  1.88702
2.26097  3.14458  3.39670  3.48268  4.32790    101 r - - E
          2.68623  4.41522  2.77509  2.73116  3.46347  2.40507  3.72507
3.29373  2.67756  2.69391  4.24726  2.90383  2.73776  3.18183  2.89792
2.37923  2.77522  2.98528  4.58513  3.61321
          0.25373  1.72280  3.08936  0.93748  0.49695  0.45188  1.01179
     60   2.70092  3.66006  5.05735  4.45277  2.12141  4.40890  2.98297
2.68956  3.77605  2.41575  3.21682  3.15046  4.77428  3.51042  4.33900
3.59492  2.54194  2.08002  5.27947  1.85901    107 y - - E
          2.68618  4.42225  2.77519  2.73123  3.46354  2.40513  3.72494
3.29354  2.67741  2.69355  4.24690  2.90347  2.73739  3.18146  2.89801
2.37887  2.77519  2.98518  4.58477  3.61503
          0.03109  5.61731  3.61282  0.61958  0.77255  0.62678  0.76424
```

```
     61   3.01120  4.62826  4.36035  4.58149  2.44627  4.05836  4.75045
2.64808  3.60715  1.93331  2.17338  4.57415  4.78176  4.50524  4.00750
3.30319  3.16498  1.60628  4.11275  2.25227    108 v - - E
          2.68618  4.42225  2.77519  2.73123  3.46354  2.40513  3.72494
3.29354  2.67741  2.69355  4.24690  2.90347  2.73739  3.18146  2.89801
2.37887  2.77519  2.98518  4.58477  3.61503
          0.02232  5.60070  3.99665  0.61958  0.77255  0.34365  1.23504
     62   3.11543  4.66597  5.22951  3.43691  2.27589  4.02742  4.78891
2.16347  4.40620  0.93259  2.76913  4.61118  4.81935  4.54130  4.42243
3.54604  3.36814  2.64370  5.28822  4.11226    109 l - - E
          2.68618  4.42225  2.77519  2.73123  3.46354  2.40513  3.72494
3.29354  2.67741  2.69355  4.24690  2.90347  2.73739  3.18146  2.89801
2.37887  2.77519  2.98518  4.58477  3.61503
          0.00526  5.64591  6.36826  0.61958  0.77255  0.43780  1.03692
     63   2.86510  5.71474  3.13666  2.41737  5.05991  2.46963  3.90225
4.54595  2.62650  4.02006  4.75537  3.19590  3.14559  2.52349  2.33989
2.42008  1.73986  3.30723  6.14736  3.78190    110 t - - E
          2.68644  4.42251  2.77433  2.73149  3.46380  2.40490  3.72521
3.29380  2.67767  2.69329  4.24716  2.90245  2.73766  3.18172  2.89827
2.37904  2.77546  2.98475  4.58503  3.61529
          0.12614  2.84510  2.80703  1.59475  0.22685  0.48576  0.95510
     64   2.99573  4.46839  2.70699  2.51003  4.46670  2.34897  3.93315
4.51166  2.00020  3.65767  4.72094  3.00385  4.03981  3.00616  2.26263
2.00566  2.35768  3.82812  6.11291  4.70185    118 k - - T
          2.68618  4.42225  2.77519  2.73123  3.46354  2.40513  3.72494
3.29354  2.67741  2.69355  4.24690  2.90347  2.73739  3.18146  2.89801
2.37887  2.77519  2.98518  4.58477  3.61503
          0.00550  5.60115  6.32349  0.61958  0.77255  0.34436  1.23330
     65   3.44744  3.67700  3.93831  4.16902  5.45901  0.35946  5.30841
4.89190  4.28313  4.54790  5.36578  3.08621  4.88782  4.55708  4.13765
3.31700  3.91043  3.76543  6.79041  5.55845    119 G - - T
          2.68618  4.42225  2.77519  2.73123  3.46354  2.40513  3.72494
3.29354  2.67741  2.69355  4.24690  2.90347  2.73739  3.18146  2.89801
2.37887  2.77519  2.98518  4.58477  3.61503
          0.00518  5.66164  6.38398  0.61958  0.77255  0.48576  0.95510
     66   3.46182  3.98640  5.12073  4.53987  3.75599  4.55022  4.79497
3.35143  4.30426  2.96404  4.02885  4.62055  4.94052  4.00306  4.41066
3.86738  2.81433  3.02405  0.56194  3.63875    120 W - - H
          2.68618  4.42225  2.77519  2.73123  3.46354  2.40513  3.72494
3.29354  2.67741  2.69355  4.24690  2.90347  2.73739  3.18146  2.89801
2.37887  2.77519  2.98518  4.58477  3.61503
          0.00518  5.66164  6.38398  0.61958  0.77255  0.48576  0.95510
     67   2.55302  4.81239  3.10692  2.47175  5.06153  2.91100  4.16433
4.27088  1.82486  3.79062  4.75636  2.96134  3.44281  2.78299  2.04310
1.99700  2.96214  3.72732  6.14818  4.31882    121 k - - H
          2.68618  4.42225  2.77519  2.73123  3.46354  2.40513  3.72494
3.29354  2.67741  2.69355  4.24690  2.90347  2.73739  3.18146  2.89801
2.37887  2.77519  2.98518  4.58477  3.61503
          0.00518  5.66164  6.38398  0.61958  0.77255  0.48576  0.95510
     68   2.88487  5.71739  2.81679  1.96631  5.06373  2.53077  3.47348
4.55060  2.11345  3.81444  4.75772  2.85704  3.86600  2.97086  2.08791
2.11188  2.98341  3.64877  6.14931  4.73760    122 e - - H
          2.68618  4.42225  2.77519  2.73123  3.46354  2.40513  3.72494
3.29354  2.67741  2.69355  4.24690  2.90347  2.73739  3.18146  2.89801
2.37887  2.77519  2.98518  4.58477  3.61503
          0.00518  5.66164  6.38398  0.61958  0.77255  0.48576  0.95510
```

```
     69   3.55581  4.27938  5.46347  4.87176  0.58070  4.71405  4.90128
2.96447  4.66195  2.58622  2.89864  4.84036  5.05799  4.76429  4.07290
4.03029  3.78555  3.13024  5.29703  3.00901    123 F - - H
          2.68618  4.42225  2.77519  2.73123  3.46354  2.40513  3.72494
3.29354  2.67741  2.69355  4.24690  2.90347  2.73739  3.18146  2.89801
2.37887  2.77519  2.98518  4.58477  3.61503
          0.00518  5.66164  6.38398  0.61958  0.77255  0.48576  0.95510
     70   2.06924  1.87198  5.20310  4.06800  3.41978  4.48109  4.81569
2.86562  4.39860  2.71940  3.82066  4.61830  4.84494  4.54632  3.91616
3.79044  3.28343  1.02387  5.33275  4.15437    124 v - - H
          2.68618  4.42225  2.77519  2.73123  3.46354  2.40513  3.72494
3.29354  2.67741  2.69355  4.24690  2.90347  2.73739  3.18146  2.89801
2.37887  2.77519  2.98518  4.58477  3.61503
          0.00518  5.66164  6.38398  0.61958  0.77255  0.48576  0.95510
     71   2.80731  4.57942  2.83838  2.25721  5.06148  3.97735  3.34541
4.03096  1.71975  3.50296  4.35643  2.53248  4.37083  2.26305  2.55974
2.35488  2.92542  3.40577  6.14815  4.73684    125 k - - H
          2.68618  4.42225  2.77519  2.73123  3.46354  2.40513  3.72494
3.29354  2.67741  2.69355  4.24690  2.90347  2.73739  3.18146  2.89801
2.37887  2.77519  2.98518  4.58477  3.61503
          0.00518  5.66164  6.38398  0.61958  0.77255  0.48576  0.95510
     72   1.39694  5.71348  1.68674  2.08582  4.50545  3.62889  4.17085
4.53954  2.80800  4.01748  4.75520  3.34096  4.37630  2.92426  3.26723
2.62319  3.13749  3.78395  6.14796  4.73834    126 a - - H
          2.68618  4.42225  2.77519  2.73123  3.46354  2.40513  3.72494
3.29354  2.67741  2.69355  4.24690  2.90347  2.73739  3.18146  2.89801
2.37887  2.77519  2.98518  4.58477  3.61503
          0.00518  5.66164  6.38398  0.61958  0.77255  0.48576  0.95510
     73   3.21069  5.68359  3.47407  2.73763  4.35965  3.98949  2.28761
4.16150  1.75468  3.98198  3.85415  1.27183  4.38246  2.84670  3.40154
3.09893  3.29332  4.06652  6.12428  4.29443    127 n - - C
          2.68618  4.42225  2.77519  2.73123  3.46354  2.40513  3.72494
3.29354  2.67741  2.69355  4.24690  2.90347  2.73739  3.18146  2.89801
2.37887  2.77519  2.98518  4.58477  3.61503
          0.00518  5.66164  6.38398  0.61958  0.77255  0.48576  0.95510
     74   2.77706  4.81282  2.55410  2.60295  5.06299  1.60162  2.96251
4.54969  2.35251  4.02260  4.28150  2.63572  3.97680  2.66159  2.40732
2.71725  3.29352  4.10808  6.14894  4.73737    128 g - - T
          2.68618  4.42225  2.77519  2.73123  3.46354  2.40513  3.72494
3.29354  2.67741  2.69355  4.24690  2.90347  2.73739  3.18146  2.89801
2.37887  2.77519  2.98518  4.58477  3.61503
          0.00518  5.66164  6.38398  0.61958  0.77255  0.48576  0.95510
     75   3.59032  5.27711  5.94883  5.35011  3.60445  5.22744  5.56838
2.34266  5.16421  0.52249  3.63235  5.39330  5.48601  5.21181  4.27139
4.56394  4.19335  1.98297  5.89561  4.79877    129 L - - -
          2.68618  4.42225  2.77519  2.73123  3.46354  2.40513  3.72494
3.29354  2.67741  2.69355  4.24690  2.90347  2.73739  3.18146  2.89801
2.37887  2.77519  2.98518  4.58477  3.61503
          0.00518  5.66164  6.38398  0.61958  0.77255  0.48576  0.95510
     76   2.71806  4.97887  2.74653  2.04823  5.05117  2.65158  3.89940
3.55155  1.71165  2.85497  4.21596  3.20508  4.37224  2.47537  2.70734
2.97341  3.16402  3.05995  6.14289  4.73348    130 k - - -
          2.68619  4.42226  2.77521  2.73124  3.46355  2.40514  3.72496
3.29355  2.67742  2.69356  4.24691  2.90325  2.73741  3.18147  2.89802
2.37888  2.77521  2.98519  4.58478  3.61504
          0.02099  3.95916  6.38398  0.38907  1.13223  0.48576  0.95510
```

```
     77   2.08111  5.56592  2.72174  1.92673  4.03668  3.46137  4.20581
2.77989  2.91079  3.60533  4.06646  3.26540  3.27734  2.89861  3.31134
2.97408  2.49563  2.10034  6.03590  4.66392    132 e - - T
          2.68618  4.42225  2.77519  2.73123  3.46354  2.40513  3.72494
3.29354  2.67741  2.69355  4.24690  2.90347  2.73739  3.18146  2.89801
2.37887  2.77519  2.98518  4.58477  3.61503
          0.00518  5.66164  6.38398  0.61958  0.77255  0.48576  0.95510
     78   3.25773  5.73509  3.33968  2.67665  5.06777  0.89999  3.99261
4.54318  2.72389  3.55333  4.78130  2.45372  4.41807  3.31606  2.75557
3.00118  3.48918  4.11955  6.17177  4.34539    133 g - - T
          2.68618  4.42225  2.77519  2.73123  3.46354  2.40513  3.72494
3.29354  2.67741  2.69355  4.24690  2.90347  2.73739  3.18146  2.89801
2.37887  2.77519  2.98518  4.58477  3.61503
          0.00518  5.66164  6.38398  0.61958  0.77255  0.48576  0.95510
     79   3.48526  6.00940  0.66269  2.07440  5.32831  4.11345  4.03342
4.82315  3.08587  4.30404  5.06645  3.57489  4.57883  3.24982  3.72477
2.95453  3.72845  4.38988  4.47927  4.40012    134 D - - S
          2.68618  4.42225  2.77519  2.73123  3.46354  2.40513  3.72494
3.29354  2.67741  2.69355  4.24690  2.90347  2.73739  3.18146  2.89801
2.37887  2.77519  2.98518  4.58477  3.61503
          0.01118  5.66164  4.87486  0.61958  0.77255  0.48576  0.95510
     80   2.60855  4.67323  5.16385  4.55425  1.77992  4.44500  4.77404
2.25152  3.92396  2.46880  3.25663  4.57911  3.99350  4.13339  4.39585
1.94367  2.44145  1.96737  5.29368  3.45401    135 f - - E
          2.68618  4.42225  2.77519  2.73123  3.46354  2.40513  3.72494
3.29354  2.67741  2.69355  4.24690  2.90347  2.73739  3.18146  2.89801
2.37887  2.77519  2.98518  4.58477  3.61503
          0.01596  5.65567  4.39553  0.61958  0.77255  0.46627  0.98709
     81   3.36767  1.97110  5.34902  4.73622  2.09916  4.55124  4.88998
2.13146  4.52201  1.63776  3.62408  4.71817  4.90712  4.64762  4.52620
3.86588  3.42623  1.63013  5.36892  3.48354    136 v - - E
          2.68618  4.42225  2.77519  2.73123  3.46354  2.40513  3.72494
3.29354  2.67741  2.69355  4.24690  2.90347  2.73739  3.18146  2.89801
2.37887  2.77519  2.98518  4.58477  3.61503
          0.00523  5.65101  6.37336  0.61958  0.77255  0.54192  0.87139
     82   3.27381  4.73310  4.80341  3.61972  3.22687  4.02877  4.69426
2.44353  2.97074  2.24055  3.41818  4.07463  4.75545  3.97363  3.75914
3.27524  1.71116  1.31738  5.34622  4.15712    137 v - - E
          2.68618  4.42225  2.77519  2.73123  3.46354  2.40513  3.72494
3.29354  2.67741  2.69355  4.24690  2.90347  2.73739  3.18146  2.89801
2.37887  2.77519  2.98518  4.58477  3.61503
          0.00523  5.65101  6.37336  0.61958  0.77255  0.54192  0.87139
     83   4.79063  5.97169  6.65130  6.13017  0.48994  6.08350  5.88872
2.71100  5.97357  1.58106  3.93981  6.14557  6.11577  5.75414  5.85554
5.47702  4.98619  3.39831  5.89678  4.12533    138 F - - E
          2.68618  4.42225  2.77519  2.73123  3.46354  2.40513  3.72494
3.29354  2.67741  2.69355  4.24690  2.90347  2.73739  3.18146  2.89801
2.37887  2.77519  2.98518  4.58477  3.61503
          0.00523  5.65101  6.37336  0.61958  0.77255  0.54192  0.87139
     84   3.08468  5.68537  3.45714  1.77532  5.02041  3.77464  3.30774
4.15510  1.88967  3.20265  2.94579  3.44778  4.36962  2.55446  2.54675
2.46698  2.87188  3.72479  6.12409  2.89205    139 e - - E
          2.68631  4.42238  2.77533  2.73137  3.46367  2.40520  3.72508
3.29367  2.67754  2.69353  4.24703  2.90360  2.73753  3.18160  2.89629
2.37893  2.77533  2.98511  4.58490  3.61516
          0.34202  2.41705  1.60701  0.67985  0.70662  0.54192  0.87139
```

```
     85   2.73267  4.58127  4.73365  4.14025  3.00386  3.05466  3.08801
3.02398  3.99823  1.33345  3.46095  4.29321  3.67454  4.19610  2.76643
3.55426  3.36998  2.57694  4.48479  2.05173    142 l - - E
          2.68618  4.42225  2.77519  2.73123  3.46354  2.40513  3.72494
3.29354  2.67741  2.69355  4.24690  2.90347  2.73739  3.18146  2.89801
2.37887  2.77519  2.98518  4.58477  3.61503
          0.08209  5.42939  2.59793  0.61958  0.77255  1.15333  0.37919
     86   2.75549  4.43208  2.41101  2.67005  3.76559  2.98304  3.85129
2.20640  2.78565  2.69127  3.10791  3.23412  4.33987  3.24476  3.32630
2.59717  2.83804  2.21979  5.59046  4.30408    143 i - - .
          2.68625  4.42232  2.77527  2.73130  3.46361  2.40474  3.72502
3.29361  2.67720  2.69362  4.24697  2.90331  2.73747  3.18153  2.89808
2.37894  2.77527  2.98525  4.58484  3.61510
          0.15475  2.06388  4.11007  0.14016  2.03421  0.74077  0.64769
     87   2.75203  5.60455  2.53564  2.60986  3.26693  1.84910  3.16107
4.43018  2.48269  3.69857  4.64605  2.94866  4.26977  2.64488  2.54809
2.39084  3.33260  3.99349  3.19386  4.63011    145 g - - S
          2.68618  4.42225  2.77519  2.73123  3.46354  2.40513  3.72494
3.29354  2.67741  2.69355  4.24690  2.90347  2.73739  3.18146  2.89801
2.37887  2.77519  2.98518  4.58477  3.61503
          0.23302  5.47676  1.59119  0.61958  0.77255  0.26155  1.46904
     88   2.65960  5.60637  2.10274  2.25803  4.95147  2.76393  3.47667
4.43750  2.79107  3.91167  4.64704  2.33268  3.59294  3.06487  2.21060
2.59463  2.60378  2.91521  6.03902  4.62799    146 d - - E
          2.68612  4.42214  2.77446  2.73108  3.46381  2.40478  3.72522
3.29362  2.67761  2.69358  4.24717  2.90374  2.73767  3.18173  2.89828
2.37871  2.77520  2.98517  4.58504  3.61530
          0.57831  0.87199  3.86137  0.04526  3.11791  0.21686  1.63496
     89   2.99479  4.12424  2.64322  1.94034  4.57458  2.52318  3.88246
4.52797  2.32810  3.65645  3.56707  2.89403  4.06317  3.07104  2.85388
1.94555  2.73508  3.11535  6.13454  4.22258    148 e - - T
          2.68618  4.42225  2.77519  2.73123  3.46354  2.40513  3.72494
3.29354  2.67741  2.69355  4.24690  2.90347  2.73739  3.18146  2.89801
2.37887  2.77519  2.98518  4.58477  3.61503
          0.00526  5.64591  6.36826  0.61958  0.77255  0.43780  1.03692
     90   2.85758  3.60456  2.15340  2.08067  5.06147  2.44325  3.78104
4.16782  2.38000  3.82806  4.75631  3.00253  3.34134  2.76527  2.66993
2.29536  2.81810  3.67815  4.79009  4.73683    149 e - - T
          2.68618  4.42225  2.77519  2.73123  3.46354  2.40513  3.72494
3.29354  2.67741  2.69355  4.24690  2.90347  2.73739  3.18146  2.89801
2.37887  2.77519  2.98518  4.58477  3.61503
          0.00518  5.66164  6.38398  0.61958  0.77255  0.48576  0.95510
     91   2.24849  5.46775  3.36366  2.68410  1.92174  2.06334  4.23774
3.93485  2.66543  2.43315  4.53409  3.28006  3.85582  3.27583  2.69011
3.23714  2.80509  3.34740  5.96016  4.61315    150 f - - S
          2.68618  4.42225  2.77519  2.73123  3.46354  2.40513  3.72494
3.29354  2.67741  2.69355  4.24690  2.90347  2.73739  3.18146  2.89801
2.37887  2.77519  2.98518  4.58477  3.61503
          0.29412  5.66164  1.38097  0.61958  0.77255  0.48576  0.95510
     92   3.04795  5.55731  3.20109  1.93141  4.90185  3.11498  3.55334
4.18009  2.06941  3.64671  4.25748  2.42870  3.25061  3.03324  2.52904
2.64114  2.05818  3.37364  5.99031  4.57969    151 e - - E
          2.68618  4.42225  2.77519  2.73123  3.46354  2.40513  3.72494
3.29354  2.67741  2.69355  4.24690  2.90347  2.73739  3.18146  2.89801
2.37887  2.77519  2.98518  4.58477  3.61503
          0.00691  5.37443  6.09677  0.61958  0.77255  1.34621  0.30141
```

```
     93   2.75478  4.09320  4.77742  4.18013  2.91439  4.24038  4.55716
 2.89577  3.69206  1.28522  2.96659  3.48395  1.86705  3.92771  4.13538
 3.53778  3.16631  2.78377  5.15008  3.01986    152 l - - E
          2.68618  4.42225  2.77519  2.73123  3.46354  2.40513  3.72494
 3.29354  2.67741  2.69355  4.24690  2.90347  2.73739  3.18146  2.89801
 2.37887  2.77519  2.98518  4.58477  3.61503
          0.00691  5.37443  6.09677  0.61958  0.77255  0.17763  1.81555
     94   2.95696  4.03094  4.41688  3.12429  2.85314  4.31104  3.51292
 2.78783  2.67277  2.22673  3.03756  3.12574  4.22051  3.49282  2.84885
 3.12553  2.44756  2.04966  3.22870  3.19032    153 v - - E
          2.68618  4.42225  2.77519  2.73123  3.46354  2.40513  3.72494
 3.29354  2.67741  2.69355  4.24690  2.90347  2.73739  3.18146  2.89801
 2.37887  2.77519  2.98518  4.58477  3.61503
          0.00518  5.66164  6.38398  0.61958  0.77255  0.48576  0.95510
     95   3.01447  4.66943  5.24895  4.63441  2.20481  4.46269  3.23361
 2.04437  4.42135  1.98694  3.58971  4.62230  4.20119  4.55430  4.43271
 3.57897  2.92717  1.17388  5.29228  4.11609    154 v - - E
          2.68618  4.42225  2.77519  2.73123  3.46354  2.40513  3.72494
 3.29354  2.67741  2.69355  4.24690  2.90347  2.73739  3.18146  2.89801
 2.37887  2.77519  2.98518  4.58477  3.61503
          0.00518  5.66164  6.38398  0.61958  0.77255  0.48576  0.95510
     96   3.05863  4.07572  3.00437  2.79815  4.91777  2.16684  2.91632
 3.82372  2.02691  2.73610  3.49353  3.26217  4.39201  2.75832  2.62681
 2.60447  2.98470  3.07430  4.67474  3.37117    155 k - - E
          2.68618  4.42225  2.77519  2.73123  3.46354  2.40513  3.72494
 3.29354  2.67741  2.69355  4.24690  2.90347  2.73739  3.18146  2.89801
 2.37887  2.77519  2.98518  4.58477  3.61503
          0.01431  5.66164  4.53436  0.61958  0.77255  0.48576  0.95510
     97   2.40831  4.20627  5.25690  4.64102  2.73962  3.49003  4.78959
 1.45435  3.96416  1.78199  3.49678  4.62057  4.81917  4.55383  4.42950
 3.76721  2.97186  1.88710  3.14570  4.10526    156 i - - E
          2.68618  4.42225  2.77519  2.73123  3.46354  2.40513  3.72494
 3.29354  2.67741  2.69355  4.24690  2.90347  2.73739  3.18146  2.89801
 2.37887  2.77519  2.98518  4.58477  3.61503
          0.00523  5.65255  6.37490  0.61958  0.77255  0.53401  0.88249
     98   3.27383  2.30514  4.76672  3.80308  1.80126  4.38103  3.49870
 2.31727  3.39987  2.22884  3.85772  3.91625  4.75014  3.96896  2.00130
 3.14161  3.50586  2.80518  5.35462  3.17058    157 f - - E
          2.68618  4.42225  2.77519  2.73123  3.46354  2.40513  3.72494
 3.29354  2.67741  2.69355  4.24690  2.90347  2.73739  3.18146  2.89801
 2.37887  2.77519  2.98518  4.58477  3.61503
          0.00523  5.65255  6.37490  0.61958  0.77255  0.53401  0.88249
     99   2.98395  5.71017  2.07855  2.39060  4.31820  3.36429  4.15931
 4.54184  2.50298  3.60509  4.41391  2.91102  3.33690  3.15277  1.55286
 2.06395  3.42981  3.94019  6.14263  4.73146    158 r - - C
          2.68618  4.42225  2.77519  2.73123  3.46354  2.40513  3.72494
 3.29354  2.67741  2.69355  4.24690  2.90347  2.73739  3.18146  2.89801
 2.37887  2.77519  2.98518  4.58477  3.61503
          0.00523  5.65255  6.37490  0.61958  0.77255  0.53401  0.88249
    100   2.15628  5.70609  3.44884  2.58258  5.04976  2.79198  3.37476
 3.91757  1.99372  3.51661  4.74712  2.75719  3.70333  2.80952  1.97906
 2.51699  2.90728  3.11655  6.13961  4.72951    159 r - - S
          2.68618  4.42225  2.77519  2.73123  3.46354  2.40513  3.72494
 3.29354  2.67741  2.69355  4.24690  2.90347  2.73739  3.18146  2.89801
 2.37887  2.77519  2.98518  4.58477  3.61503
          0.03001  5.65255  3.64760  0.61958  0.77255  0.53401  0.88249
```

```
    101   2.70745  5.68399  2.79617  2.42599  5.02418  2.91367  4.14809
3.11450  2.63369  3.81130  4.39752  2.47561  2.94790  2.66857  2.72849
2.14801  2.61163  2.63660  5.08030  4.26964    160 s - - S
          2.68618  4.42225  2.77519  2.73123  3.46354  2.40513  3.72494
3.29354  2.67741  2.69355  4.24690  2.90347  2.73739  3.18146  2.89801
2.37887  2.77519  2.98518  4.58477  3.61503
          0.00361  5.62615        *  0.61958  0.77255  0.00000        *
//
HMMER3/f [3.3 | Nov 2019]
NAME  BES1_N
ACC   PF05687.14
DESC  BES1/BZR1 plant transcription factor, N-terminal
LENG  151
ALPH  amino
RF    no
MM    no
CONS  yes
CS    no
MAP   yes
DATE  Fri Apr 17 10:30:43 2020
NSEQ  23
EFFN  1.075317
CKSUM 2037425146
GA    25.00 25.00
TC    25.30 25.30
NC    24.80 24.80
STATS LOCAL MSV      -10.2879  0.70928
STATS LOCAL VITERBI  -11.3277  0.70928
STATS LOCAL FORWARD   -4.0135  0.70928
HMM          A        C        D        E        F        G        H
I        K        L        M        N        P        Q        R        S
T        V        W        Y
            m->m     m->i     m->d     i->m     i->i     d->m     d->d
  COMPO   2.42919  4.22604  3.03310  2.60506  3.67900  2.78661  3.75096
3.12800  2.62591  2.66183  3.85386  2.97141  3.20070  3.09891  2.61684
2.43397  2.76435  2.84571  4.49912  3.58876
          2.68618  4.42225  2.77519  2.73123  3.46354  2.40513  3.72494
3.29354  2.67741  2.69355  4.24690  2.90347  2.73739  3.18146  2.89801
2.37887  2.77519  2.98518  4.58477  3.61503
          0.05222  4.23009  3.31507  0.61958  0.77255  0.00000        *
      1   1.95648  4.27362  3.59435  3.21934  4.36872  1.44617  4.27142
3.77493  3.24050  3.45655  4.27214  3.39309  3.79208  3.52614  3.57795
1.86134  2.60658  2.99015  5.70553  4.49035      1 g - - -
          2.68618  4.42225  2.77519  2.73123  3.46354  2.40513  3.72494
3.29354  2.67741  2.69355  4.24690  2.90347  2.73739  3.18146  2.89801
2.37887  2.77519  2.98518  4.58477  3.61503
          0.02252  4.20039  4.92274  0.61958  0.77255  0.52326  0.89792
      2   2.47085  4.78680  3.02240  2.53087  4.21286  2.07372  3.73101
3.63491  2.25421  3.22472  4.03386  3.02119  3.84453  2.88299  2.74214
2.23492  2.66829  3.26768  5.44455  4.12005      2 g - - -
          2.68618  4.42225  2.77519  2.73123  3.46354  2.40513  3.72494
3.29354  2.67741  2.69355  4.24690  2.90347  2.73739  3.18146  2.89801
2.37887  2.77519  2.98518  4.58477  3.61503
          0.02252  4.20039  4.92274  0.61958  0.77255  0.46301  0.99260
      3   2.56088  4.62257  3.20785  2.75331  4.26776  1.68956  3.89426
3.67996  2.65218  3.29623  4.11962  3.16662  3.86918  3.07473  2.33978
2.51902  2.31716  3.27741  5.52094  4.23926      3 g - - -
```

```
          2.68618  4.42225  2.77519  2.73123  3.46354  2.40513  3.72494
3.29354  2.67741  2.69355  4.24690  2.90347  2.73739  3.18146  2.89801
2.37887  2.77519  2.98518  4.58477  3.61503
          0.02185  4.23009  4.95244  0.61958  0.77255  0.48576  0.95510
      4   3.11969  5.31936  3.41530  2.43069  4.78047  3.73589  3.69227
4.12526  1.71397  3.54827  4.39686  3.23048  4.10474  2.81826  1.20934
3.09463  3.28601  3.78712  5.58710  4.39833      4 r - - -
          2.68618  4.42225  2.77519  2.73123  3.46354  2.40513  3.72494
3.29354  2.67741  2.69355  4.24690  2.90347  2.73739  3.18146  2.89801
2.37887  2.77519  2.98518  4.58477  3.61503
          0.02185  4.23009  4.95244  0.61958  0.77255  0.48576  0.95510
      5   2.63570  4.65836  3.16672  2.60045  3.85838  3.50482  3.71001
3.24628  2.26371  2.82701  3.05806  3.08908  3.89032  2.75841  2.44625
2.52796  2.61616  2.48879  5.18769  3.89755      5 k - - -
          2.68618  4.42225  2.77519  2.73123  3.46354  2.40513  3.72494
3.29354  2.67741  2.69355  4.24690  2.90347  2.73739  3.18146  2.89801
2.37887  2.77519  2.98518  4.58477  3.61503
          0.02185  4.23009  4.95244  0.61958  0.77255  0.48576  0.95510
      6   2.81272  5.09900  2.79811  1.90679  4.44890  3.44546  3.74611
3.88519  2.45934  3.42988  4.25063  2.98042  1.79992  2.89133  2.55054
2.80220  3.06323  3.51917  5.60266  4.25807      6 p - - -
          2.68618  4.42225  2.77519  2.73123  3.46354  2.40513  3.72494
3.29354  2.67741  2.69355  4.24690  2.90347  2.73739  3.18146  2.89801
2.37887  2.77519  2.98518  4.58477  3.61503
          0.02185  4.23009  4.95244  0.61958  0.77255  0.48576  0.95510
      7   2.60499  4.69954  3.17353  2.65444  4.16275  3.38929  3.78827
3.56805  2.53131  3.18108  4.00638  3.11014  3.22179  2.95760  2.19529
2.41519  1.78777  3.21210  5.41615  4.12167      7 t - - -
          2.68618  4.42225  2.77519  2.73123  3.46354  2.40513  3.72494
3.29354  2.67741  2.69355  4.24690  2.90347  2.73739  3.18146  2.89801
2.37887  2.77519  2.98518  4.58477  3.61503
          0.02185  4.23009  4.95244  0.61958  0.77255  0.48576  0.95510
      8   2.80955  4.61734  3.39710  2.15761  3.34291  3.66651  3.81525
3.09418  2.76441  2.44308  3.68272  3.33257  4.06723  3.17676  3.09445
2.96195  3.04264  2.88652  1.89926  3.41761      8 w - - -
          2.68618  4.42225  2.77519  2.73123  3.46354  2.40513  3.72494
3.29354  2.67741  2.69355  4.24690  2.90347  2.73739  3.18146  2.89801
2.37887  2.77519  2.98518  4.58477  3.61503
          0.02185  4.23009  4.95244  0.61958  0.77255  0.48576  0.95510
      9   3.11635  5.33052  3.44620  2.47203  4.80258  3.74100  3.68346
4.14635  1.45609  3.55816  4.40219  3.23108  4.10220  2.80610  1.38722
3.08838  3.28004  3.80286  5.58865  4.40099      9 r - - -
          2.68618  4.42225  2.77519  2.73123  3.46354  2.40513  3.72494
3.29354  2.67741  2.69355  4.24690  2.90347  2.73739  3.18146  2.89801
2.37887  2.77519  2.98518  4.58477  3.61503
          0.02185  4.23009  4.95244  0.61958  0.77255  0.48576  0.95510
     10   3.31599  5.45388  2.70627  0.60590  4.85371  3.54607  4.18506
4.38357  3.11607  3.94871  4.92394  3.20443  4.17267  3.40387  3.55064
3.27076  3.62839  4.03616  5.95539  4.69968     10 E - - -
          2.68618  4.42225  2.77519  2.73123  3.46354  2.40513  3.72494
3.29354  2.67741  2.69355  4.24690  2.90347  2.73739  3.18146  2.89801
2.37887  2.77519  2.98518  4.58477  3.61503
          0.02185  4.23009  4.95244  0.61958  0.77255  0.48576  0.95510
     11   3.27805  5.36476  3.77039  3.00970  4.88892  3.85470  3.71683
4.20216  1.36741  3.59192  4.47192  3.37650  4.20061  2.84667  1.17293
3.26219  3.41647  3.88703  5.58553  4.46463     11 r - - -
```

```
          2.68618  4.42225  2.77519  2.73123  3.46354  2.40513  3.72494
3.29354  2.67741  2.69355  4.24690  2.90347  2.73739  3.18146  2.89801
2.37887  2.77519  2.98518  4.58477  3.61503
          0.02185  4.23009  4.95244  0.61958  0.77255  0.48576  0.95510
     12   3.31599  5.45388  2.70627  0.60590  4.85371  3.54607  4.18506
4.38357  3.11607  3.94871  4.92394  3.20443  4.17267  3.40387  3.55064
3.27076  3.62839  4.03616  5.95539  4.69968     12 E - - -
          2.68618  4.42225  2.77519  2.73123  3.46354  2.40513  3.72494
3.29354  2.67741  2.69355  4.24690  2.90347  2.73739  3.18146  2.89801
2.37887  2.77519  2.98518  4.58477  3.61503
          0.02185  4.23009  4.95244  0.61958  0.77255  0.48576  0.95510
     13   2.92562  5.11568  3.06676  2.66150  4.50969  3.53830  3.78188
4.00173  2.30643  3.51090  4.35703  1.52985  4.01271  2.93490  1.75025
2.93119  3.17536  3.63478  5.58301  4.28756     13 n - - -
          2.68618  4.42225  2.77519  2.73123  3.46354  2.40513  3.72494
3.29354  2.67741  2.69355  4.24690  2.90347  2.73739  3.18146  2.89801
2.37887  2.77519  2.98518  4.58477  3.61503
          0.02185  4.23009  4.95244  0.61958  0.77255  0.48576  0.95510
     14   2.62462  4.68951  2.97858  2.72622  4.38032  3.27476  4.00997
3.79089  2.86245  3.46326  4.31741  1.45243  3.90359  3.21210  3.25683
2.71610  1.83623  3.37300  5.67129  4.34809     14 n - - -
          2.68618  4.42225  2.77519  2.73123  3.46354  2.40513  3.72494
3.29354  2.67741  2.69355  4.24690  2.90347  2.73739  3.18146  2.89801
2.37887  2.77519  2.98518  4.58477  3.61503
          0.02185  4.23009  4.95244  0.61958  0.77255  0.48576  0.95510
     15   3.25541  5.36085  3.72459  2.98135  4.87952  3.83866  3.71356
4.19876  1.07788  3.59039  4.46574  3.35757  4.18744  2.84234  1.52801
3.23768  3.39828  3.87885  5.58720  4.45716     15 k - - -
          2.68618  4.42225  2.77519  2.73123  3.46354  2.40513  3.72494
3.29354  2.67741  2.69355  4.24690  2.90347  2.73739  3.18146  2.89801
2.37887  2.77519  2.98518  4.58477  3.61503
          0.02185  4.23009  4.95244  0.61958  0.77255  0.48576  0.95510
     16   2.97418  4.91128  3.59199  2.92724  4.13053  3.73550  3.76032
3.47703  2.06324  1.99424  3.97812  3.32806  4.10101  2.94248  1.47130
3.05841  3.16738  3.23769  5.31919  4.10672     16 r - - -
          2.68618  4.42225  2.77519  2.73123  3.46354  2.40513  3.72494
3.29354  2.67741  2.69355  4.24690  2.90347  2.73739  3.18146  2.89801
2.37887  2.77519  2.98518  4.58477  3.61503
          0.02185  4.23009  4.95244  0.61958  0.77255  0.48576  0.95510
     17   3.45894  5.24202  3.99056  3.44454  4.69075  3.85739  4.15190
4.27144  2.47198  3.72220  4.72567  3.76998  4.35411  3.36562  0.54734
3.53400  3.70778  3.98282  5.63478  4.55480     17 R - - -
          2.68618  4.42225  2.77519  2.73123  3.46354  2.40513  3.72494
3.29354  2.67741  2.69355  4.24690  2.90347  2.73739  3.18146  2.89801
2.37887  2.77519  2.98518  4.58477  3.61503
          0.02185  4.23009  4.95244  0.61958  0.77255  0.48576  0.95510
     18   3.31599  5.45388  2.70627  0.60590  4.85371  3.54607  4.18506
4.38357  3.11607  3.94871  4.92394  3.20443  4.17267  3.40387  3.55064
3.27076  3.62839  4.03616  5.95539  4.69968     18 E - - -
          2.68618  4.42225  2.77519  2.73123  3.46354  2.40513  3.72494
3.29354  2.67741  2.69355  4.24690  2.90347  2.73739  3.18146  2.89801
2.37887  2.77519  2.98518  4.58477  3.61503
          0.02185  4.23009  4.95244  0.61958  0.77255  0.48576  0.95510
     19   3.45894  5.24202  3.99056  3.44454  4.69075  3.85739  4.15190
4.27144  2.47198  3.72220  4.72567  3.76998  4.35411  3.36562  0.54734
3.53400  3.70778  3.98282  5.63478  4.55480     19 R - - -
```

```
          2.68618  4.42225  2.77519  2.73123  3.46354  2.40513  3.72494
3.29354  2.67741  2.69355  4.24690  2.90347  2.73739  3.18146  2.89801
2.37887  2.77519  2.98518  4.58477  3.61503
          0.02185  4.23009  4.95244  0.61958  0.77255  0.48576  0.95510
     20   3.10401  5.19076  3.48380  2.88036  4.32069  3.73637  2.06263
3.98516  2.10519  3.44958  4.33041  3.29509  4.12611  2.90655  1.28284
3.11290  3.29238  3.67259  5.40030  4.10515     20 r - - -
          2.68618  4.42225  2.77519  2.73123  3.46354  2.40513  3.72494
3.29354  2.67741  2.69355  4.24690  2.90347  2.73739  3.18146  2.89801
2.37887  2.77519  2.98518  4.58477  3.61503
          0.02185  4.23009  4.95244  0.61958  0.77255  0.48576  0.95510
     21   3.15726  5.08905  3.67820  3.16561  4.67564  3.19755  4.00141
4.15159  2.36035  3.65139  4.56122  3.52174  4.19868  3.18564  0.74703
3.22547  3.43138  3.80069  5.62829  4.49923     21 r - - -
          2.68618  4.42225  2.77519  2.73123  3.46354  2.40513  3.72494
3.29354  2.67741  2.69355  4.24690  2.90347  2.73739  3.18146  2.89801
2.37887  2.77519  2.98518  4.58477  3.61503
          0.02185  4.23009  4.95244  0.61958  0.77255  0.48576  0.95510
     22   3.15726  5.08905  3.67820  3.16561  4.67564  3.19755  4.00141
4.15159  2.36035  3.65139  4.56122  3.52174  4.19868  3.18564  0.74703
3.22547  3.43138  3.80069  5.62829  4.49923     22 r - - -
          2.68618  4.42225  2.77519  2.73123  3.46354  2.40513  3.72494
3.29354  2.67741  2.69355  4.24690  2.90347  2.73739  3.18146  2.89801
2.37887  2.77519  2.98518  4.58477  3.61503
          0.02185  4.23009  4.95244  0.61958  0.77255  0.48576  0.95510
     23   0.79757  4.24609  3.68974  3.46387  4.49853  2.76549  4.49207
3.87666  3.53416  3.61484  4.44516  3.51431  3.82227  3.79902  3.79953
2.29230  2.83712  3.29954  5.85864  4.67360     23 a - - -
          2.68618  4.42225  2.77519  2.73123  3.46354  2.40513  3.72494
3.29354  2.67741  2.69355  4.24690  2.90347  2.73739  3.18146  2.89801
2.37887  2.77519  2.98518  4.58477  3.61503
          0.02185  4.23009  4.95244  0.61958  0.77255  0.48576  0.95510
     24   2.95684  4.39787  4.50089  4.02966  3.63012  3.60708  4.72133
0.97631  3.89375  2.32940  3.51923  4.22085  4.53263  4.19047  4.09618
3.48168  3.25165  1.75092  5.39622  4.17243     24 i - - -
          2.68618  4.42225  2.77519  2.73123  3.46354  2.40513  3.72494
3.29354  2.67741  2.69355  4.24690  2.90347  2.73739  3.18146  2.89801
2.37887  2.77519  2.98518  4.58477  3.61503
          0.02185  4.23009  4.95244  0.61958  0.77255  0.48576  0.95510
     25   1.17519  4.26533  3.76932  3.43869  4.29987  3.12046  4.41272
3.47609  3.41487  3.33219  4.22841  3.53284  3.86353  3.71450  3.69421
2.57186  1.60675  3.04892  5.70740  4.51217     25 a - - -
          2.68618  4.42225  2.77519  2.73123  3.46354  2.40513  3.72494
3.29354  2.67741  2.69355  4.24690  2.90347  2.73739  3.18146  2.89801
2.37887  2.77519  2.98518  4.58477  3.61503
          0.04436  4.23009  3.54592  0.61958  0.77255  0.48576  0.95510
     26   1.01711  4.24547  3.67410  3.40326  4.44010  3.03990  4.42852
3.80648  3.44407  3.54777  4.38682  3.48316  3.81052  3.72230  3.72694
1.68075  2.82135  3.25592  5.80302  4.60266     26 a - - -
          2.68618  4.42225  2.77519  2.73123  3.46354  2.40513  3.72494
3.29354  2.67741  2.69355  4.24690  2.90347  2.73739  3.18146  2.89801
2.37887  2.77519  2.98518  4.58477  3.61503
          0.02235  4.20808  4.93042  0.61958  0.77255  0.51380  0.91185
     27   3.15628  5.32102  3.58823  2.87112  4.78509  3.78030  3.23514
4.13585  1.18628  3.54078  4.39572  3.27665  4.12599  2.80098  1.60091
3.13468  3.30774  3.80514  5.55438  4.38585     27 k - - -
```

2.68618 4.42225 2.77519 2.73123 3.46354 2.40513 3.72494
3.29354 2.67741 2.69355 4.24690 2.90347 2.73739 3.18146 2.89801
2.37887 2.77519 2.98518 4.58477 3.61503
0.02235 4.20808 4.93042 0.61958 0.77255 0.51380 0.91185
28 3.26402 4.58454 4.92990 4.39228 3.33392 4.55183 4.95541
1.16046 4.23257 1.76335 2.01717 4.60960 4.79890 4.41431 4.36667
3.91714 3.50502 2.01045 5.34274 4.21945 28 i - - -
2.68618 4.42225 2.77519 2.73123 3.46354 2.40513 3.72494
3.29354 2.67741 2.69355 4.24690 2.90347 2.73739 3.18146 2.89801
2.37887 2.77519 2.98518 4.58477 3.61503
0.02235 4.20808 4.93042 0.61958 0.77255 0.51380 0.91185
29 3.36698 4.68824 4.68932 4.24067 1.78832 4.34029 3.80549
2.92762 4.08449 1.50192 3.53304 4.16673 4.63993 4.13000 4.15188
3.67199 3.58816 2.89035 4.00724 1.49557 29 y - - -
2.68618 4.42225 2.77519 2.73123 3.46354 2.40513 3.72494
3.29354 2.67741 2.69355 4.24690 2.90347 2.73739 3.18146 2.89801
2.37887 2.77519 2.98518 4.58477 3.61503
0.02235 4.20808 4.93042 0.61958 0.77255 0.46862 0.98314
30 1.58597 4.43668 3.37616 2.90391 4.17791 2.74544 4.00870
3.58738 2.89430 3.23881 4.06399 3.24984 3.83278 3.22160 3.04399
2.01773 2.15576 3.16858 5.50054 4.23802 30 a - - -
2.68618 4.42225 2.77519 2.73123 3.46354 2.40513 3.72494
3.29354 2.67741 2.69355 4.24690 2.90347 2.73739 3.18146 2.89801
2.37887 2.77519 2.98518 4.58477 3.61503
0.02185 4.23009 4.95244 0.61958 0.77255 0.48576 0.95510
31 3.17268 4.89696 3.91825 3.87003 4.97612 0.34278 4.95289
4.67814 4.09901 4.28099 5.27035 4.08435 4.29788 4.39553 4.28121
3.35714 3.68125 4.13697 5.96129 5.09530 31 G - - -
2.68618 4.42225 2.77519 2.73123 3.46354 2.40513 3.72494
3.29354 2.67741 2.69355 4.24690 2.90347 2.73739 3.18146 2.89801
2.37887 2.77519 2.98518 4.58477 3.61503
0.02185 4.23009 4.95244 0.61958 0.77255 0.48576 0.95510
32 2.95121 4.71432 4.48856 4.09486 3.32143 4.20764 4.77286
2.40723 3.88246 0.72962 3.20542 4.33009 4.62687 4.19189 4.04971
3.65963 3.53095 2.45128 5.29652 4.10928 32 l - - -
2.68618 4.42225 2.77519 2.73123 3.46354 2.40513 3.72494
3.29354 2.67741 2.69355 4.24690 2.90347 2.73739 3.18146 2.89801
2.37887 2.77519 2.98518 4.58477 3.61503
0.02185 4.23009 4.95244 0.61958 0.77255 0.48576 0.95510
33 3.04078 5.02857 3.61293 3.05993 4.53505 3.64720 3.86877
4.00366 2.19508 3.51404 4.42254 3.40621 4.13576 3.04192 0.89377
2.85403 3.30832 3.66175 5.55048 4.34569 33 r - - -
2.68618 4.42225 2.77519 2.73123 3.46354 2.40513 3.72494
3.29354 2.67741 2.69355 4.24690 2.90347 2.73739 3.18146 2.89801
2.37887 2.77519 2.98518 4.58477 3.61503
0.02185 4.23009 4.95244 0.61958 0.77255 0.48576 0.95510
34 1.98396 4.71198 3.11933 2.56163 3.93713 3.32137 3.69888
3.33279 2.50296 2.84675 3.23212 3.05822 3.87678 2.30808 2.68947
2.62809 2.86746 3.03883 5.24335 3.94079 34 a - - -
2.68618 4.42225 2.77519 2.73123 3.46354 2.40513 3.72494
3.29354 2.67741 2.69355 4.24690 2.90347 2.73739 3.18146 2.89801
2.37887 2.77519 2.98518 4.58477 3.61503
0.02185 4.23009 4.95244 0.61958 0.77255 0.48576 0.95510
35 2.78234 4.68791 3.35450 2.80539 3.23313 3.64226 2.65124
3.25970 2.67257 2.77970 3.54881 3.24609 4.02276 2.45944 3.03861
2.88672 3.00861 3.01047 4.73566 1.92667 35 y - - -

```
        2.68618  4.42225  2.77519  2.73123  3.46354  2.40513  3.72494
3.29354  2.67741  2.69355  4.24690  2.90347  2.73739  3.18146  2.89801
2.37887  2.77519  2.98518  4.58477  3.61503
        0.02185  4.23009  4.95244  0.61958  0.77255  0.48576  0.95510
     36  3.17268  4.89696  3.91825  3.87003  4.97612  0.34278  4.95289
4.67814  4.09901  4.28099  5.27035  4.08435  4.29788  4.39553  4.28121
3.35714  3.68125  4.13697  5.96129  5.09530     36 G - - -
        2.68618  4.42225  2.77519  2.73123  3.46354  2.40513  3.72494
3.29354  2.67741  2.69355  4.24690  2.90347  2.73739  3.18146  2.89801
2.37887  2.77519  2.98518  4.58477  3.61503
        0.02185  4.23009  4.95244  0.61958  0.77255  0.48576  0.95510
     37  2.87481  4.94537  2.81194  2.72953  4.61200  3.02004  4.16431
4.25719  3.09474  3.85719  4.74278  0.82496  4.02028  3.39985  3.49236
2.93137  3.28007  3.78183  5.85080  4.51517     37 n - - -
        2.68618  4.42225  2.77519  2.73123  3.46354  2.40513  3.72494
3.29354  2.67741  2.69355  4.24690  2.90347  2.73739  3.18146  2.89801
2.37887  2.77519  2.98518  4.58477  3.61503
        0.02185  4.23009  4.95244  0.61958  0.77255  0.48576  0.95510
     38  3.99090  5.12847  4.92907  4.69428  1.34347  4.65311  3.56765
3.63772  4.52002  2.90816  4.21247  4.28749  4.94510  4.38790  4.47906
4.04005  4.20601  3.57403  3.67714  0.85929     38 y - - -
        2.68618  4.42225  2.77519  2.73123  3.46354  2.40513  3.72494
3.29354  2.67741  2.69355  4.24690  2.90347  2.73739  3.18146  2.89801
2.37887  2.77519  2.98518  4.58477  3.61503
        0.02185  4.23009  4.95244  0.61958  0.77255  0.48576  0.95510
     39  2.70364  5.06721  2.85657  2.33683  4.39416  3.42774  3.65943
3.84023  1.96237  3.37035  4.15512  2.26556  2.66373  2.78326  2.83378
2.68765  2.56398  3.45631  5.54058  4.17255     39 k - - -
        2.68618  4.42225  2.77519  2.73123  3.46354  2.40513  3.72494
3.29354  2.67741  2.69355  4.24690  2.90347  2.73739  3.18146  2.89801
2.37887  2.77519  2.98518  4.58477  3.61503
        0.02185  4.23009  4.95244  0.61958  0.77255  0.48576  0.95510
     40  3.58371  4.94838  4.76373  4.41992  3.31418  4.43283  4.96586
2.55106  4.18544  0.53332  3.25101  4.66177  4.81590  4.46544  4.29681
4.06932  3.86659  2.67849  5.30709  4.10816     40 L - - -
        2.68618  4.42225  2.77519  2.73123  3.46354  2.40513  3.72494
3.29354  2.67741  2.69355  4.24690  2.90347  2.73739  3.18146  2.89801
2.37887  2.77519  2.98518  4.58477  3.61503
        0.02185  4.23009  4.95244  0.61958  0.77255  0.48576  0.95510
     41  2.87675  4.81299  3.39350  3.06764  4.41401  3.47606  4.13908
3.91689  2.81098  3.50350  4.42145  3.44336  0.94027  3.37106  2.48526
2.98931  3.23939  3.54567  5.61620  4.40851     41 p - - -
        2.68618  4.42225  2.77519  2.73123  3.46354  2.40513  3.72494
3.29354  2.67741  2.69355  4.24690  2.90347  2.73739  3.18146  2.89801
2.37887  2.77519  2.98518  4.58477  3.61503
        0.02185  4.23009  4.95244  0.61958  0.77255  0.48576  0.95510
     42  2.06491  4.76914  3.21523  2.69578  4.17455  3.47401  3.79514
3.43815  1.49988  3.14256  4.01030  3.15213  3.93537  2.96386  2.81109
2.77416  2.96999  2.75605  5.42453  4.14153     42 k - - -
        2.68618  4.42225  2.77519  2.73123  3.46354  2.40513  3.72494
3.29354  2.67741  2.69355  4.24690  2.90347  2.73739  3.18146  2.89801
2.37887  2.77519  2.98518  4.58477  3.61503
        0.02185  4.23009  4.95244  0.61958  0.77255  0.48576  0.95510
     43  3.07428  5.15281  3.42004  2.85824  4.20892  3.71106  1.58973
3.93897  2.16501  3.41977  4.30440  3.28186  4.11431  2.93132  1.63751
3.08927  3.27402  3.62875  5.35597  4.02697     43 h - - -
```

```
        2.68618  4.42225  2.77519  2.73123  3.46354  2.40513  3.72494
3.29354  2.67741  2.69355  4.24690  2.90347  2.73739  3.18146  2.89801
2.37887  2.77519  2.98518  4.58477  3.61503
        0.02185  4.23009  4.95244  0.61958  0.77255  0.48576  0.95510
     44   1.44926  1.34492  4.27369  3.94550  4.13836  3.14491  4.63032
3.21713  3.81207  3.16783  4.11782  3.77123  3.91324  4.06172  3.96969
2.61639  2.86617  2.85021  5.62146  4.45905      44 c - - -
        2.68618  4.42225  2.77519  2.73123  3.46354  2.40513  3.72494
3.29354  2.67741  2.69355  4.24690  2.90347  2.73739  3.18146  2.89801
2.37887  2.77519  2.98518  4.58477  3.61503
        0.02185  4.23009  4.95244  0.61958  0.77255  0.48576  0.95510
     45   3.42015  5.55612  0.51610  2.63456  4.99200  3.52397  4.30526
4.67165  3.46550  4.22020  5.22091  3.20369  4.20260  3.55501  4.01945
3.35182  3.77111  4.27692  6.08484  4.82466      45 D - - -
        2.68618  4.42225  2.77519  2.73123  3.46354  2.40513  3.72494
3.29354  2.67741  2.69355  4.24690  2.90347  2.73739  3.18146  2.89801
2.37887  2.77519  2.98518  4.58477  3.61503
        0.02185  4.23009  4.95244  0.61958  0.77255  0.48576  0.95510
     46   2.70714  4.52344  3.40900  2.89122  3.62175  3.61192  3.90280
2.58892  2.81472  2.59013  2.50285  1.82168  4.02456  3.17675  3.17845
2.88763  2.95691  2.66639  5.10540  3.82667      46 n - - -
        2.68618  4.42225  2.77519  2.73123  3.46354  2.40513  3.72494
3.29354  2.67741  2.69355  4.24690  2.90347  2.73739  3.18146  2.89801
2.37887  2.77519  2.98518  4.58477  3.61503
        0.02185  4.23009  4.95244  0.61958  0.77255  0.48576  0.95510
     47   3.07035  5.05155  2.97884  2.90842  4.47736  3.46564  4.27903
4.28335  3.22353  3.89188  4.85681  0.65304  4.14138  3.56540  3.57950
3.13176  3.47176  3.86743  5.74104  4.41006      47 N - - -
        2.68618  4.42225  2.77519  2.73123  3.46354  2.40513  3.72494
3.29354  2.67741  2.69355  4.24690  2.90347  2.73739  3.18146  2.89801
2.37887  2.77519  2.98518  4.58477  3.61503
        0.02185  4.23009  4.95244  0.61958  0.77255  0.48576  0.95510
     48   2.80790  5.63347  1.55946  1.25969  4.93212  3.33574  3.86150
4.44622  2.86426  3.93642  4.77373  2.79265  3.94769  3.02020  3.46250
2.91847  3.32001  4.00760  6.09449  4.58075      48 e - - -
        2.68618  4.42225  2.77519  2.73123  3.46354  2.40513  3.72494
3.29354  2.67741  2.69355  4.24690  2.90347  2.73739  3.18146  2.89801
2.37887  2.77519  2.98518  4.58477  3.61503
        0.02185  4.23009  4.95244  0.61958  0.77255  0.48576  0.95510
     49   3.10325  4.56437  4.61077  4.29931  3.84144  4.08347  5.04922
2.10548  4.17379  2.47442  3.76491  4.42851  4.63465  4.51429  4.35034
3.62119  3.46259  0.66758  5.68148  4.42988      49 V - - -
        2.68618  4.42225  2.77519  2.73123  3.46354  2.40513  3.72494
3.29354  2.67741  2.69355  4.24690  2.90347  2.73739  3.18146  2.89801
2.37887  2.77519  2.98518  4.58477  3.61503
        0.02185  4.23009  4.95244  0.61958  0.77255  0.48576  0.95510
     50   3.50413  4.79862  5.11474  4.58776  3.20525  4.78172  5.11218
1.84268  4.40297  0.75063  3.00544  4.83634  4.94949  4.52450  4.50292
4.17301  3.73430  2.27273  5.34291  4.24863      50 l - - -
        2.68618  4.42225  2.77519  2.73123  3.46354  2.40513  3.72494
3.29354  2.67741  2.69355  4.24690  2.90347  2.73739  3.18146  2.89801
2.37887  2.77519  2.98518  4.58477  3.61503
        0.02185  4.23009  4.95244  0.61958  0.77255  0.48576  0.95510
     51   1.94121  4.98165  3.19531  2.66957  4.40819  3.53408  3.73530
3.78499  1.42498  3.34140  4.17812  3.13263  3.97032  2.88065  2.44727
2.85068  3.06374  3.43995  5.50473  4.23679      51 k - - -
```

```
          2.68618  4.42225  2.77519  2.73123  3.46354  2.40513  3.72494
3.29354  2.67741  2.69355  4.24690  2.90347  2.73739  3.18146  2.89801
2.37887  2.77519  2.98518  4.58477  3.61503
          0.02185  4.23009  4.95244  0.61958  0.77255  0.48576  0.95510
     52   0.60057  4.41306  3.86798  3.69446  4.45030  3.22483  4.66788
3.63286  3.72125  3.49635  4.50006  3.74011  3.99923  4.03088  3.93531
2.76624  3.06929  3.22219  5.83939  4.68646     52 A - - -
          2.68618  4.42225  2.77519  2.73123  3.46354  2.40513  3.72494
3.29354  2.67741  2.69355  4.24690  2.90347  2.73739  3.18146  2.89801
2.37887  2.77519  2.98518  4.58477  3.61503
          0.02185  4.23009  4.95244  0.61958  0.77255  0.48576  0.95510
     53   3.58371  4.94838  4.76373  4.41992  3.31418  4.43283  4.96586
2.55106  4.18544  0.53332  3.25101  4.66177  4.81590  4.46544  4.29681
4.06932  3.86659  2.67849  5.30709  4.10816     53 L - - -
          2.68618  4.42225  2.77519  2.73123  3.46354  2.40513  3.72494
3.29354  2.67741  2.69355  4.24690  2.90347  2.73739  3.18146  2.89801
2.37887  2.77519  2.98518  4.58477  3.61503
          0.02185  4.23009  4.95244  0.61958  0.77255  0.48576  0.95510
     54   1.44926  1.34492  4.27369  3.94550  4.13836  3.14491  4.63032
3.21713  3.81207  3.16783  4.11782  3.77123  3.91324  4.06172  3.96969
2.61639  2.86617  2.85021  5.62146  4.45905     54 c - - -
          2.68618  4.42225  2.77519  2.73123  3.46354  2.40513  3.72494
3.29354  2.67741  2.69355  4.24690  2.90347  2.73739  3.18146  2.89801
2.37887  2.77519  2.98518  4.58477  3.61503
          0.02185  4.23009  4.95244  0.61958  0.77255  0.48576  0.95510
     55   2.58108  5.03092  2.84555  2.32122  4.33495  3.43264  3.62328
3.78243  2.27529  3.17896  4.09000  2.35707  3.83057  2.74088  2.07720
2.46895  2.88766  3.25651  5.48688  4.11746     55 r - - -
          2.68618  4.42225  2.77519  2.73123  3.46354  2.40513  3.72494
3.29354  2.67741  2.69355  4.24690  2.90347  2.73739  3.18146  2.89801
2.37887  2.77519  2.98518  4.58477  3.61503
          0.02185  4.23009  4.95244  0.61958  0.77255  0.48576  0.95510
     56   2.46090  5.19891  2.54678  1.01947  4.64268  3.37389  3.91546
4.00702  2.79858  3.64071  4.52334  2.95878  3.96890  3.08847  3.26766
2.89599  3.22719  3.63651  5.86091  4.46886     56 e - - -
          2.68618  4.42225  2.77519  2.73123  3.46354  2.40513  3.72494
3.29354  2.67741  2.69355  4.24690  2.90347  2.73739  3.18146  2.89801
2.37887  2.77519  2.98518  4.58477  3.61503
          0.02185  4.23009  4.95244  0.61958  0.77255  0.48576  0.95510
     57   0.60057  4.41306  3.86798  3.69446  4.45030  3.22483  4.66788
3.63286  3.72125  3.49635  4.50006  3.74011  3.99923  4.03088  3.93531
2.76624  3.06929  3.22219  5.83939  4.68646     57 A - - -
          2.68618  4.42225  2.77519  2.73123  3.46354  2.40513  3.72494
3.29354  2.67741  2.69355  4.24690  2.90347  2.73739  3.18146  2.89801
2.37887  2.77519  2.98518  4.58477  3.61503
          0.02185  4.23009  4.95244  0.61958  0.77255  0.48576  0.95510
     58   3.17268  4.89696  3.91825  3.87003  4.97612  0.34278  4.95289
4.67814  4.09901  4.28099  5.27035  4.08435  4.29788  4.39553  4.28121
3.35714  3.68125  4.13697  5.96129  5.09530     58 G - - -
          2.68618  4.42225  2.77519  2.73123  3.46354  2.40513  3.72494
3.29354  2.67741  2.69355  4.24690  2.90347  2.73739  3.18146  2.89801
2.37887  2.77519  2.98518  4.58477  3.61503
          0.02185  4.23009  4.95244  0.61958  0.77255  0.48576  0.95510
     59   3.99909  5.23259  4.68105  4.48618  3.12765  4.16892  4.41435
4.05735  4.21216  3.38330  4.67117  4.56875  4.71877  4.57990  4.27187
4.19910  4.32101  3.95644  0.39509  3.11360     59 W - - -
```

2.68618 4.42225 2.77519 2.73123 3.46354 2.40513 3.72494
3.29354 2.67741 2.69355 4.24690 2.90347 2.73739 3.18146 2.89801
2.37887 2.77519 2.98518 4.58477 3.61503
0.02185 4.23009 4.95244 0.61958 0.77255 0.48576 0.95510
60 2.64275 3.79051 4.01158 3.45323 3.41277 3.68986 4.14199
2.12037 3.35921 2.42434 3.38384 3.69900 4.12228 3.61630 3.60116
2.68744 1.95084 1.77646 4.94227 3.73936 60 v - - -
2.68618 4.42225 2.77519 2.73123 3.46354 2.40513 3.72494
3.29354 2.67741 2.69355 4.24690 2.90347 2.73739 3.18146 2.89801
2.37887 2.77519 2.98518 4.58477 3.61503
0.02185 4.23009 4.95244 0.61958 0.77255 0.48576 0.95510
61 3.10325 4.56437 4.61077 4.29931 3.84144 4.08347 5.04922
2.10548 4.17379 2.47442 3.76491 4.42851 4.63465 4.51429 4.35034
3.62119 3.46259 0.66758 5.68148 4.42988 61 V - - -
2.68618 4.42225 2.77519 2.73123 3.46354 2.40513 3.72494
3.29354 2.67741 2.69355 4.24690 2.90347 2.73739 3.18146 2.89801
2.37887 2.77519 2.98518 4.58477 3.61503
0.02185 4.23009 4.95244 0.61958 0.77255 0.48576 0.95510
62 2.75012 5.16767 2.54508 1.45164 4.45128 3.40583 3.18337
3.92320 2.47162 3.00940 4.23385 2.88347 3.86168 2.65699 2.95531
2.71404 2.99018 3.53371 5.61279 4.21267 62 e - - -
2.68618 4.42225 2.77519 2.73123 3.46354 2.40513 3.72494
3.29354 2.67741 2.69355 4.24690 2.90347 2.73739 3.18146 2.89801
2.37887 2.77519 2.98518 4.58477 3.61503
0.02185 4.23009 4.95244 0.61958 0.77255 0.48576 0.95510
63 2.43590 5.16262 2.38874 1.89164 4.49930 3.37283 3.72623
3.96331 2.56841 3.49695 4.28963 2.87540 2.08869 2.86163 3.07809
2.56398 3.01879 3.56438 5.67827 4.27026 63 e - - -
2.68618 4.42225 2.77519 2.73123 3.46354 2.40513 3.72494
3.29354 2.67741 2.69355 4.24690 2.90347 2.73739 3.18146 2.89801
2.37887 2.77519 2.98518 4.58477 3.61503
0.02185 4.23009 4.95244 0.61958 0.77255 0.48576 0.95510
64 3.42015 5.55612 0.51610 2.63456 4.99200 3.52397 4.30526
4.67165 3.46550 4.22020 5.22091 3.20369 4.20260 3.55501 4.01945
3.35182 3.77111 4.27692 6.08484 4.82466 64 D - - -
2.68618 4.42225 2.77519 2.73123 3.46354 2.40513 3.72494
3.29354 2.67741 2.69355 4.24690 2.90347 2.73739 3.18146 2.89801
2.37887 2.77519 2.98518 4.58477 3.61503
0.02185 4.23009 4.95244 0.61958 0.77255 0.48576 0.95510
65 3.17268 4.89696 3.91825 3.87003 4.97612 0.34278 4.95289
4.67814 4.09901 4.28099 5.27035 4.08435 4.29788 4.39553 4.28121
3.35714 3.68125 4.13697 5.96129 5.09530 65 G - - -
2.68618 4.42225 2.77519 2.73123 3.46354 2.40513 3.72494
3.29354 2.67741 2.69355 4.24690 2.90347 2.73739 3.18146 2.89801
2.37887 2.77519 2.98518 4.58477 3.61503
0.02185 4.23009 4.95244 0.61958 0.77255 0.48576 0.95510
66 2.65148 4.49220 3.79764 3.58613 4.33671 3.31492 4.56941
3.51085 3.53655 3.36081 4.39956 3.72269 4.05226 3.91579 3.76670
2.85828 0.68430 3.16397 5.74003 4.55800 66 T - - -
2.68618 4.42225 2.77519 2.73123 3.46354 2.40513 3.72494
3.29354 2.67741 2.69355 4.24690 2.90347 2.73739 3.18146 2.89801
2.37887 2.77519 2.98518 4.58477 3.61503
0.02185 4.23009 4.95244 0.61958 0.77255 0.48576 0.95510
67 2.65148 4.49220 3.79764 3.58613 4.33671 3.31492 4.56941
3.51085 3.53655 3.36081 4.39956 3.72269 4.05226 3.91579 3.76670
2.85828 0.68430 3.16397 5.74003 4.55800 67 T - - -

```
          2.68618  4.42225  2.77519  2.73123  3.46354  2.40513  3.72494
3.29354  2.67741  2.69355  4.24690  2.90347  2.73739  3.18146  2.89801
2.37887  2.77519  2.98518  4.58477  3.61503
          0.02185  4.23009  4.95244  0.61958  0.77255  0.48576  0.95510
     68   3.96998  5.12559  4.88874  4.64435  1.54769  4.63347  3.57342
3.62811  4.47444  2.90454  4.20795  4.27297  4.93154  4.36703  4.45196
4.02167  4.18794  3.56315  3.68771  0.75945     68 y - - -
          2.68618  4.42225  2.77519  2.73123  3.46354  2.40513  3.72494
3.29354  2.67741  2.69355  4.24690  2.90347  2.73739  3.18146  2.89801
2.37887  2.77519  2.98518  4.58477  3.61503
          0.02185  4.23009  4.95244  0.61958  0.77255  0.48576  0.95510
     69   3.03700  5.04223  3.54227  3.00500  4.56485  3.64496  3.89752
4.00275  2.28970  3.50925  4.40471  3.39114  3.04036  3.06704  0.92523
3.09413  3.29845  3.66192  5.57505  4.39538     69 r - - -
          2.68593  4.42264  2.77535  2.73095  3.46393  2.40536  3.72473
3.29314  2.67681  2.69351  4.24627  2.90386  2.73779  3.18186  2.89814
2.37889  2.77535  2.98500  4.58373  3.61488
          0.16223  1.94712  4.95244  2.36353  0.09881  0.48576  0.95510
     70   2.69069  5.16599  3.11430  2.57187  4.54937  3.56348  3.66749
3.95649  1.41354  3.44074  4.24967  3.06412  3.96047  2.14518  2.41873
2.67435  3.07649  3.58922  5.55154  4.26315     95 k - - -
          2.68618  4.42225  2.77519  2.73123  3.46354  2.40513  3.72494
3.29354  2.67741  2.69355  4.24690  2.90347  2.73739  3.18146  2.89801
2.37887  2.77519  2.98518  4.58477  3.61503
          0.02185  4.23009  4.95244  0.61958  0.77255  0.48576  0.95510
     71   2.28849  3.32173  3.88256  3.54515  4.42976  1.12806  4.47637
3.85278  3.51737  3.55445  4.37264  3.54526  3.80608  3.78482  3.78248
1.79775  2.79941  3.27324  5.79091  4.61210     96 g - - -
          2.68618  4.42225  2.77519  2.73123  3.46354  2.40513  3.72494
3.29354  2.67741  2.69355  4.24690  2.90347  2.73739  3.18146  2.89801
2.37887  2.77519  2.98518  4.58477  3.61503
          0.02185  4.23009  4.95244  0.61958  0.77255  0.48576  0.95510
     72   2.60502  2.34468  3.34569  2.79379  3.62897  3.52389  3.56947
3.00296  2.74823  2.69578  3.57237  3.22976  3.05645  2.88244  3.13125
2.49533  2.61447  2.75424  5.03675  3.56747     97 c - - -
          2.68618  4.42225  2.77519  2.73123  3.46354  2.40513  3.72494
3.29354  2.67741  2.69355  4.24690  2.90347  2.73739  3.18146  2.89801
2.37887  2.77519  2.98518  4.58477  3.61503
          0.02185  4.23009  4.95244  0.61958  0.77255  0.48576  0.95510
     73   2.64061  5.13102  3.08020  2.52160  4.49369  3.53037  3.64869
3.91316  1.45387  3.40669  4.20302  3.02569  3.48382  2.35961  2.40536
2.69260  3.01696  3.53906  5.53141  4.22058     98 k - - -
          2.68618  4.42225  2.77519  2.73123  3.46354  2.40513  3.72494
3.29354  2.67741  2.69355  4.24690  2.90347  2.73739  3.18146  2.89801
2.37887  2.77519  2.98518  4.58477  3.61503
          0.02185  4.23009  4.95244  0.61958  0.77255  0.48576  0.95510
     74   2.91810  4.79198  3.48510  3.26482  4.48142  3.46262  4.36544
4.03936  3.16647  3.63650  4.59753  3.59800  0.70627  3.65185  3.04424
3.05800  3.33558  3.64429  5.70250  4.53115     99 p - - -
          2.68618  4.42225  2.77519  2.73123  3.46354  2.40513  3.72494
3.29354  2.67741  2.69355  4.24690  2.90347  2.73739  3.18146  2.89801
2.37887  2.77519  2.98518  4.58477  3.61503
          0.18463  4.23009  1.87056  0.61958  0.77255  0.48576  0.95510
     75   2.59024  4.39505  3.44512  2.92403  3.62376  3.48685  3.91274
2.68581  2.83798  2.22377  3.55686  3.31650  2.12469  3.19321  3.18875
2.78906  2.46753  2.59310  5.10320  3.85434    100 p - - -
```

```
          2.68618  4.42225  2.77519  2.73123  3.46354  2.40513  3.72494
3.29354  2.67741  2.69355  4.24690  2.90347  2.73739  3.18146  2.89801
2.37887  2.77519  2.98518  4.58477  3.61503
          0.09043  4.07113  2.66782  0.61958  0.77255  0.66132  0.72603
     76   2.22820  4.63956  3.00070  2.43099  3.89243  2.98725  3.35383
3.29124  2.50329  2.66511  3.77260  2.99781  2.79805  2.85672  2.93439
2.64808  2.82117  2.99253  5.20927  3.90500    101 a - - -
          2.68620  4.42227  2.77522  2.73125  3.46356  2.40504  3.72497
3.29356  2.67743  2.69357  4.24692  2.90349  2.73720  3.18149  2.89803
2.37889  2.77522  2.98521  4.58479  3.61505
          0.41386  1.73932  1.81238  0.28973  1.38017  0.55092  0.85901
     77   2.34810  4.41082  3.16397  2.68433  3.91024  3.23176  3.79784
3.28901  2.67145  2.96023  3.80825  3.09381  2.87249  2.88242  3.07129
1.93299  2.60382  2.62373  5.25299  3.98011    103 s - - -
          2.68618  4.42225  2.77519  2.73123  3.46354  2.40513  3.72494
3.29354  2.67741  2.69355  4.24690  2.90347  2.73739  3.18146  2.89801
2.37887  2.77519  2.98518  4.58477  3.61503
          0.02877  3.95861  4.68096  0.61958  0.77255  0.71813  0.66877
     78   2.53236  4.86872  2.67414  1.70672  4.27805  3.28050  3.68218
3.70495  2.50751  3.28865  4.10209  2.86984  3.16617  2.83490  2.97381
2.36606  2.69641  3.32768  5.50216  4.14133    104 e - - -
          2.68618  4.42225  2.77519  2.73123  3.46354  2.40513  3.72494
3.29354  2.67741  2.69355  4.24690  2.90347  2.73739  3.18146  2.89801
2.37887  2.77519  2.98518  4.58477  3.61503
          0.02809  3.98226  4.70461  0.61958  0.77255  0.73778  0.65042
     79   2.29944  4.66692  3.05025  2.44386  3.89634  3.22019  3.45826
3.29212  2.41594  2.55205  3.76286  2.99992  3.47498  2.81655  2.39690
2.65944  2.82343  2.99817  5.19202  3.89350    105 a - - -
          2.68618  4.42225  2.77519  2.73123  3.46354  2.40513  3.72494
3.29354  2.67741  2.69355  4.24690  2.90347  2.73739  3.18146  2.89801
2.37887  2.77519  2.98518  4.58477  3.61503
          0.02809  3.98226  4.70461  0.61958  0.77255  0.38307  1.14496
     80   2.30498  4.65052  3.12712  2.56832  3.85352  3.31861  3.70084
3.14295  2.55085  2.89556  3.09419  2.88643  3.47691  2.64290  2.87022
2.32092  2.77965  2.72559  5.18787  3.88934    106 a - - -
          2.68618  4.42225  2.77519  2.73123  3.46354  2.40513  3.72494
3.29354  2.67741  2.69355  4.24690  2.90347  2.73739  3.18146  2.89801
2.37887  2.77519  2.98518  4.58477  3.61503
          0.02228  4.21102  4.93336  0.61958  0.77255  0.51014  0.91732
     81   2.41704  5.09656  2.04553  2.18953  3.87971  3.39601  3.31925
3.86772  2.40229  3.38537  4.15239  2.64043  3.81669  2.61366  2.89659
2.47224  2.89950  3.46691  5.55088  4.15319    107 d - - -
          2.68618  4.42225  2.77519  2.73123  3.46354  2.40513  3.72494
3.29354  2.67741  2.69355  4.24690  2.90347  2.73739  3.18146  2.89801
2.37887  2.77519  2.98518  4.58477  3.61503
          0.02228  4.21102  4.93336  0.61958  0.77255  0.51014  0.91732
     82   2.37362  4.31265  3.51282  2.94524  3.44914  3.39758  3.85279
2.42769  2.89283  2.41009  3.01387  3.34329  3.26764  2.63194  3.23879
2.84185  2.84074  2.22670  4.90185  3.67071    108 v - - -
          2.68618  4.42225  2.77519  2.73123  3.46354  2.40513  3.72494
3.29354  2.67741  2.69355  4.24690  2.90347  2.73739  3.18146  2.89801
2.37887  2.77519  2.98518  4.58477  3.61503
          0.03753  4.21102  3.81670  0.61958  0.77255  0.51014  0.91732
     83   2.42211  4.24629  3.78359  3.21691  3.38562  2.31313  4.00993
2.36623  3.14199  2.08247  3.10911  3.55010  4.07737  3.42209  3.43436
2.96685  2.89469  2.06211  4.90319  3.69128    109 v - - -
```

```
          2.68618  4.42225  2.77519  2.73123  3.46354  2.40513  3.72494
3.29354  2.67741  2.69355  4.24690  2.90347  2.73739  3.18146  2.89801
2.37887  2.77519  2.98518  4.58477  3.61503
          0.03900  4.19611  3.76371  0.61958  0.77255  0.45995  0.99781
     84   2.40257  4.56706  3.17420  2.70682  4.06185  1.80791  3.85803
3.14806  2.71468  3.10858  3.94531  3.14034  3.85561  2.80821  3.12972
2.15478  2.73788  3.10808  5.38177  4.09467    110 g - - -
          2.68618  4.42225  2.77519  2.73123  3.46354  2.40513  3.72494
3.29354  2.67741  2.69355  4.24690  2.90347  2.73739  3.18146  2.89801
2.37887  2.77519  2.98518  4.58477  3.61503
          0.02220  4.21462  4.93696  0.61958  0.77255  0.47355  0.97495
     85   2.48595  4.08588  3.31066  2.75180  2.65013  3.03586  3.79088
3.04647  2.71985  2.73294  3.60302  3.19835  3.44968  3.04556  2.55588
2.39817  2.60236  2.79000  5.06766  3.80635    111 s - - -
          2.68618  4.42225  2.77519  2.73123  3.46354  2.40513  3.72494
3.29354  2.67741  2.69355  4.24690  2.90347  2.73739  3.18146  2.89801
2.37887  2.77519  2.98518  4.58477  3.61503
          0.02185  4.23009  4.95244  0.61958  0.77255  0.48576  0.95510
     86   2.29291  4.79812  2.98039  2.50924  4.18942  3.11672  3.39552
3.61591  2.54239  3.21170  4.02181  3.00952  3.09549  2.72829  2.99578
1.73185  2.88757  3.25540  5.44112  4.10709    112 s - - -
          2.68618  4.42225  2.77519  2.73123  3.46354  2.40513  3.72494
3.29354  2.67741  2.69355  4.24690  2.90347  2.73739  3.18146  2.89801
2.37887  2.77519  2.98518  4.58477  3.61503
          0.02185  4.23009  4.95244  0.61958  0.77255  0.48576  0.95510
     87   2.01788  4.42258  3.37772  2.83010  3.69912  3.45889  3.85102
3.05769  2.79764  2.76194  3.40740  3.11484  2.96391  3.11736  3.18519
2.42299  2.57888  2.23789  5.10267  3.84954    113 a - - -
          2.68618  4.42225  2.77519  2.73123  3.46354  2.40513  3.72494
3.29354  2.67741  2.69355  4.24690  2.90347  2.73739  3.18146  2.89801
2.37887  2.77519  2.98518  4.58477  3.61503
          0.02185  4.23009  4.95244  0.61958  0.77255  0.48576  0.95510
     88   2.65506  4.94974  2.97141  2.44019  4.26828  3.18079  3.19732
3.70358  2.38651  3.25990  4.05196  2.74425  3.84471  2.78364  2.21671
2.05515  2.59560  3.33838  5.45294  4.10286    114 s - - -
          2.68619  4.42226  2.77521  2.73124  3.46355  2.40514  3.72496
3.29329  2.67742  2.69356  4.24691  2.90341  2.73741  3.18148  2.89802
2.37888  2.77521  2.98520  4.58478  3.61504
          0.28419  2.45004  1.82583  0.37540  1.16160  0.48576  0.95510
     89   1.98401  4.26221  3.50690  2.94129  3.23163  3.55766  3.61945
2.44412  2.88554  2.45573  3.23758  3.32914  3.23454  3.18538  3.22423
2.81256  2.80693  2.38520  4.86071  3.63008    116 a - - -
          2.68618  4.42225  2.77519  2.73123  3.46354  2.40513  3.72494
3.29354  2.67741  2.69355  4.24690  2.90347  2.73739  3.18146  2.89801
2.37887  2.77519  2.98518  4.58477  3.61503
          0.02588  4.06284  4.78518  0.61958  0.77255  0.38618  1.13834
     90   2.37178  4.31692  3.56313  3.31374  4.47241  3.07981  4.39179
3.86229  3.35479  3.58923  4.43753  3.45525  3.84107  3.66884  3.64835
0.87835  2.36393  3.31566  5.82314  4.59618    117 s - - -
          2.68618  4.42225  2.77519  2.73123  3.46354  2.40513  3.72494
3.29354  2.67741  2.69355  4.24690  2.90347  2.73739  3.18146  2.89801
2.37887  2.77519  2.98518  4.58477  3.61503
          0.02185  4.23009  4.95244  0.61958  0.77255  0.48576  0.95510
     91   2.49152  4.40265  3.41911  2.86101  3.37109  3.56551  3.84139
2.71515  2.81643  2.61159  3.51426  3.28553  2.25946  2.83252  3.18925
2.81418  2.76408  2.23699  5.00314  3.75756    118 v - - -
```

```
          2.68618  4.42225  2.77520  2.73124  3.46354  2.40513  3.72495
3.29354  2.67741  2.69355  4.24690  2.90347  2.73740  3.18147  2.89793
2.37887  2.77520  2.98519  4.58477  3.61503
          0.04627  3.26632  4.95244  0.50977  0.91788  0.48576  0.95510
     92   2.63719  2.54317  3.10666  2.12218  3.87032  3.48769  3.71943
3.26778  2.56351  2.91688  3.76407  2.96204  3.88729  2.67567  2.99168
2.54691  2.87179  2.98565  5.20508  3.35186    120 e - - -
          2.68618  4.42225  2.77519  2.73123  3.46354  2.40513  3.72494
3.29354  2.67741  2.69355  4.24690  2.90347  2.73739  3.18146  2.89801
2.37887  2.77519  2.98518  4.58477  3.61503
          0.02185  4.23009  4.95244  0.61958  0.77255  0.48576  0.95510
     93   2.37398  4.33557  3.50967  3.18232  4.41345  3.10139  4.27368
3.81889  3.21235  3.50233  4.32808  3.38823  3.44511  3.52351  3.54530
1.06288  2.07902  3.29266  5.74335  4.51530    121 s - - -
          2.68618  4.42225  2.77519  2.73123  3.46354  2.40513  3.72494
3.29354  2.67741  2.69355  4.24690  2.90347  2.73739  3.18146  2.89801
2.37887  2.77519  2.98518  4.58477  3.61503
          0.02185  4.23009  4.95244  0.61958  0.77255  0.48576  0.95510
     94   2.57766  4.65678  3.03678  2.70592  4.33840  3.27148  3.94648
3.78917  2.77781  3.39873  4.22622  3.13424  2.52224  2.83390  3.18000
1.31755  2.94264  3.36142  5.60992  4.30035    122 s - - -
          2.68618  4.42225  2.77519  2.73123  3.46354  2.40513  3.72494
3.29354  2.67741  2.69355  4.24690  2.90347  2.73739  3.18146  2.89801
2.37887  2.77519  2.98518  4.58477  3.61503
          0.02185  4.23009  4.95244  0.61958  0.77255  0.48576  0.95510
     95   2.50487  4.40261  3.38424  2.81924  3.55040  3.56174  3.36679
2.73764  2.78096  2.39589  3.37770  3.14020  3.38700  2.89358  3.16038
2.60751  2.77141  2.56342  4.97404  2.60539    123 l - - -
          2.68618  4.42225  2.77519  2.73123  3.46354  2.40513  3.72494
3.29354  2.67741  2.69355  4.24690  2.90347  2.73739  3.18146  2.89801
2.37887  2.77519  2.98518  4.58477  3.61503
          0.02185  4.23009  4.95244  0.61958  0.77255  0.48576  0.95510
     96   2.63545  4.90201  2.97192  2.43029  3.75492  3.44022  3.37942
3.59446  2.41929  3.00127  3.97250  2.70283  3.34371  2.00510  2.88231
2.34066  2.86768  3.25023  5.38595  4.03343    124 q - - -
          2.68618  4.42225  2.77519  2.73123  3.46354  2.40513  3.72494
3.29354  2.67741  2.69355  4.24690  2.90347  2.73739  3.18146  2.89801
2.37887  2.77519  2.98518  4.58477  3.61503
          0.02185  4.23009  4.95244  0.61958  0.77255  0.48576  0.95510
     97   2.26594  4.69043  3.10498  2.56844  3.97675  3.18105  3.72479
3.37853  2.55091  2.75267  3.84425  3.05844  2.37839  2.72155  2.91426
2.54223  2.58740  3.06780  5.28327  3.97672    125 a - - -
          2.68614  4.42229  2.77524  2.73128  3.46358  2.40510  3.72499
3.29358  2.67745  2.69336  4.24694  2.90341  2.73744  3.18151  2.89805
2.37878  2.77524  2.98523  4.58481  3.61507
          0.64641  1.37915  1.49485  0.19786  1.71751  0.48576  0.95510
     98   2.42269  4.61235  3.08625  2.55508  3.95127  3.36447  3.68131
3.33896  2.33382  2.97672  3.81491  3.02599  3.80937  2.86208  2.57457
2.00991  2.63116  2.82892  5.24177  3.94974    127 s - - -
          2.68618  4.42225  2.77519  2.73123  3.46354  2.40513  3.72494
3.29354  2.67741  2.69355  4.24690  2.90347  2.73739  3.18146  2.89801
2.37887  2.77519  2.98518  4.58477  3.61503
          0.02800  3.98519  4.70754  0.61958  0.77255  0.73545  0.65256
     99   2.36420  4.33105  3.37381  2.83387  3.49933  3.48258  3.53865
2.82907  2.76264  2.10375  3.46152  3.24223  2.85496  3.10088  3.11676
2.75343  2.80653  2.38390  4.94467  3.69584    128 l - - -
```

```
          2.68618  4.42225  2.77519  2.73123  3.46354  2.40513  3.72494
3.29354  2.67741  2.69355  4.24690  2.90347  2.73739  3.18146  2.89801
2.37887  2.77519  2.98518  4.58477  3.61503
          0.06942  3.98519  3.02667  0.61958  0.77255  0.73545  0.65256
    100   2.59177  4.69456  3.03913  2.48173  3.91887  3.42517  3.41641
3.31856  2.26529  2.58395  3.77493  2.67534  3.61829  2.62903  2.62983
2.65044  2.81886  2.73746  5.19819  3.89566    129 k - - -
          2.68618  4.42225  2.77519  2.73123  3.46354  2.40513  3.72494
3.29354  2.67741  2.69355  4.24690  2.90347  2.73739  3.18146  2.89801
2.37887  2.77519  2.98518  4.58477  3.61503
          0.02917  3.94495  4.66729  0.61958  0.77255  0.76638  0.62491
    101   2.42204  4.45604  2.86662  2.65614  4.22722  3.06485  3.93485
3.74724  2.82907  3.39013  4.23534  2.39251  3.73781  3.16084  3.20861
1.39458  2.83920  3.27697  5.54200  4.21120    130 s - - -
          2.68618  4.42225  2.77519  2.73123  3.46354  2.40513  3.72494
3.29354  2.67741  2.69355  4.24690  2.90347  2.73739  3.18146  2.89801
2.37887  2.77519  2.98518  4.58477  3.61503
          0.02917  3.94495  4.66729  0.61958  0.77255  0.76638  0.62491
    102   2.45435  3.11684  3.51196  2.99802  3.41378  3.34344  3.86725
2.88967  2.92029  2.61835  3.52485  3.31358  3.85674  3.23828  3.24057
1.85567  2.76955  2.62908  4.88699  2.91144    131 s - - -
          2.68618  4.42225  2.77519  2.73123  3.46354  2.40513  3.72494
3.29354  2.67741  2.69355  4.24690  2.90347  2.73739  3.18146  2.89801
2.37887  2.77519  2.98518  4.58477  3.61503
          0.02917  3.94495  4.66729  0.61958  0.77255  0.76638  0.62491
    103   1.47506  4.13745  3.39102  3.06133  4.21767  2.40081  4.13382
3.61416  3.11102  3.30607  4.12963  3.24370  3.65599  3.39626  3.44187
1.88011  2.47654  3.09319  5.56091  4.34582    132 a - - -
          2.68623  4.42230  2.77525  2.73128  3.46273  2.40518  3.72500
3.29344  2.67746  2.69346  4.24695  2.90352  2.73745  3.18152  2.89806
2.37892  2.77525  2.98501  4.58482  3.61508
          0.51563  0.93273  4.66729  0.17579  1.82506  0.70290  0.68349
    104   2.47969  4.92624  2.74633  2.34104  4.22429  2.94881  3.58217
3.66357  2.14453  3.21488  4.00358  2.87250  3.01661  2.62599  2.55990
2.59692  2.75143  3.29623  5.40652  4.04516    134 k - - -
          2.68618  4.42225  2.77519  2.73123  3.46354  2.40513  3.72494
3.29354  2.67741  2.69355  4.24690  2.90347  2.73739  3.18146  2.89801
2.37887  2.77519  2.98518  4.58477  3.61503
          0.02800  3.98519  4.70754  0.61958  0.77255  0.35633  1.20479
    105   2.20447  4.56734  3.12730  2.54773  4.26164  3.25648  3.96527
3.65198  2.83468  3.30745  4.13705  3.16611  3.85621  3.16257  3.24572
1.33078  2.49256  3.24296  5.56183  4.27335    135 s - - -
          2.68618  4.42225  2.77519  2.73123  3.46354  2.40513  3.72494
3.29354  2.67741  2.69355  4.24690  2.90347  2.73739  3.18146  2.89801
2.37887  2.77519  2.98518  4.58477  3.61503
          0.02185  4.23009  4.95244  0.61958  0.77255  0.48576  0.95510
    106   2.03484  4.34621  3.50205  3.22718  4.39835  3.12378  4.32260
3.69874  3.26554  3.44801  4.32059  3.42343  1.15331  3.58802  3.57596
2.58266  2.57826  3.22402  5.75727  4.53872    136 p - - -
          2.68618  4.42225  2.77519  2.73123  3.46354  2.40513  3.72494
3.29354  2.67741  2.69355  4.24690  2.90347  2.73739  3.18146  2.89801
2.37887  2.77519  2.98518  4.58477  3.61503
          0.02185  4.23009  4.95244  0.61958  0.77255  0.48576  0.95510
    107   2.54886  3.26570  3.96956  3.38874  3.26157  3.54137  4.01805
2.12189  3.27638  2.07038  3.25666  3.64669  4.08670  3.52847  2.63176
2.99558  2.86425  1.99667  4.77212  3.57354    137 v - - -
```

```
         2.68618  4.42225  2.77519  2.73123  3.46354  2.40513  3.72494
3.29354  2.67741  2.69355  4.24690  2.90347  2.73739  3.18146  2.89801
2.37887  2.77519  2.98518  4.58477  3.61503
         0.02185  4.23009  4.95244  0.61958  0.77255  0.48576  0.95510
    108  2.37848  4.99528  2.64096  2.01960  4.28978  2.82122  3.63256
3.49930  2.40554  3.15004  4.06391  2.91342  2.91107  2.75489  2.88839
2.63131  2.79834  3.36032  5.47462  4.09949    138 e - - -
         2.68618  4.42225  2.77519  2.73123  3.46354  2.40513  3.72494
3.29354  2.67741  2.69355  4.24690  2.90347  2.73739  3.18146  2.89801
2.37887  2.77519  2.98518  4.58477  3.61503
         0.02185  4.23009  4.95244  0.61958  0.77255  0.48576  0.95510
    109  2.50338  3.69667  3.44860  2.92603  3.84617  3.35015  3.94639
3.20656  2.87983  2.59017  3.77888  2.99597  3.88641  3.20732  3.25306
1.51718  2.41169  2.89626  5.23689  3.98679    139 s - - -
         2.68618  4.42225  2.77519  2.73123  3.46354  2.40513  3.72494
3.29354  2.67741  2.69355  4.24690  2.90347  2.73739  3.18146  2.89801
2.37887  2.77519  2.98518  4.58477  3.61503
         0.31867  4.23009  1.35349  0.61958  0.77255  0.48576  0.95510
    110  3.25936  4.80743  3.77201  3.42859  2.16135  3.95259  2.91125
3.41738  3.25574  2.84270  3.97588  3.61407  4.35051  3.57557  3.47831
3.33972  3.49869  3.24892  3.80842  1.09234    140 y - - -
         2.68618  4.42225  2.77519  2.73123  3.46354  2.40513  3.72494
3.29354  2.67741  2.69355  4.24690  2.90347  2.73739  3.18146  2.89801
2.37887  2.77519  2.98518  4.58477  3.61503
         0.02930  3.94072  4.66306  0.61958  0.77255  0.76950  0.62221
    111  2.75100  5.04564  2.64973  2.33528  4.22499  3.35634  2.87516
3.79790  2.34553  3.33067  4.16116  2.00039  3.83276  2.34468  2.74018
2.72944  2.99263  3.44168  5.42971  4.03977    141 n - - -
         2.68618  4.42225  2.77519  2.73123  3.46354  2.40513  3.72494
3.29354  2.67741  2.69355  4.24690  2.90347  2.73739  3.18146  2.89801
2.37887  2.77519  2.98518  4.58477  3.61503
         0.02930  3.94072  4.66306  0.61958  0.77255  0.76950  0.62221
    112  2.14260  4.43853  3.13430  2.64661  3.79963  3.11406  3.32537
3.18377  2.62923  2.85814  3.71603  3.08107  2.63455  2.97514  3.02851
2.60615  2.77423  2.54159  5.15918  3.88265    142 a - - -
         2.68618  4.42225  2.77519  2.73123  3.46354  2.40513  3.72494
3.29354  2.67741  2.69355  4.24690  2.90347  2.73739  3.18146  2.89801
2.37887  2.77519  2.98518  4.58477  3.61503
         0.02930  3.94072  4.66306  0.61958  0.77255  0.76950  0.62221
    113  2.52644  4.53350  2.94087  2.66285  3.87217  3.19316  3.19771
3.58536  2.69137  3.20394  4.08205  3.07118  3.80063  3.09050  3.04423
1.39968  2.89191  3.20260  5.25248  3.85509    143 s - - -
         2.68618  4.42225  2.77519  2.73123  3.46354  2.40513  3.72494
3.29354  2.67741  2.69355  4.24690  2.90347  2.73739  3.18146  2.89801
2.37887  2.77519  2.98518  4.58477  3.61503
         0.02930  3.94072  4.66306  0.61958  0.77255  0.53733  0.87780
    114  2.75693  4.87307  2.88182  2.58300  4.28574  3.36938  3.82581
3.77337  2.54870  3.33341  4.21686  3.06240  1.72314  1.99320  2.90601
2.79953  3.05901  3.42014  5.51499  4.20001    144 p - - -
         2.68618  4.42225  2.77519  2.73123  3.46354  2.40513  3.72494
3.29354  2.67741  2.69355  4.24690  2.90347  2.73739  3.18146  2.89801
2.37887  2.77519  2.98518  4.58477  3.61503
         0.02511  4.09297  4.81532  0.61958  0.77255  0.64046  0.74876
    115  1.96143  4.51836  3.16372  2.65407  3.95023  3.14791  3.78356
3.34197  2.64134  2.99654  3.83568  3.09487  3.81444  2.58762  3.05973
2.23685  2.39976  2.77764  5.27875  3.99309    145 a - - -
```

```
          2.68618  4.42225  2.77519  2.73123  3.46354  2.40513  3.72494
3.29354  2.67741  2.69355  4.24690  2.90347  2.73739  3.18146  2.89801
2.37887  2.77519  2.98518  4.58477  3.61503
          0.17804  4.09297  1.92138  0.61958  0.77255  0.64046  0.74876
    116   2.53249  4.54230  2.93926  2.65766  3.87026  3.20020  3.16234
3.58455  2.68413  3.20199  4.07933  3.06917  3.80360  3.08405  3.03836
1.40871  2.89471  3.20400  5.24984  3.85138    146 s - - -
          2.68618  4.42225  2.77519  2.73123  3.46354  2.40513  3.72494
3.29354  2.67741  2.69355  4.24690  2.90347  2.73739  3.18146  2.89801
2.37887  2.77519  2.98518  4.58477  3.61503
          0.02920  3.94413  4.66647  0.61958  0.77255  0.34312  1.23632
    117   2.16122  4.31489  3.55524  3.35660  4.51632  3.06655  4.45016
3.92703  3.44393  3.65866  4.51219  3.47581  3.84513  3.74077  3.72561
0.80512  2.88394  3.35504  5.87349  4.64340    147 s - - -
          2.68618  4.42225  2.77519  2.73123  3.46354  2.40513  3.72494
3.29354  2.67741  2.69355  4.24690  2.90347  2.73739  3.18146  2.89801
2.37887  2.77519  2.98518  4.58477  3.61503
          0.02185  4.23009  4.95244  0.61958  0.77255  0.48576  0.95510
    118   2.40985  4.40321  3.43273  2.90516  3.77228  3.41096  3.92052
3.08175  2.86350  2.82630  3.33943  3.12694  3.91151  3.18886  3.23811
1.58834  2.70560  2.33606  5.17804  3.92922    148 s - - -
          2.68618  4.42225  2.77519  2.73123  3.46354  2.40513  3.72494
3.29354  2.67741  2.69355  4.24690  2.90347  2.73739  3.18146  2.89801
2.37887  2.77519  2.98518  4.58477  3.61503
          0.02185  4.23009  4.95244  0.61958  0.77255  0.48576  0.95510
    119   2.82568  3.34842  4.48075  3.90534  1.78944  3.95833  4.12173
2.40956  3.74694  1.61687  3.16520  3.99615  4.29798  3.89574  3.84078
3.26642  3.05602  2.36580  4.56627  2.91283    149 l - - -
          2.68618  4.42225  2.77519  2.73123  3.46354  2.40513  3.72494
3.29354  2.67741  2.69355  4.24690  2.90347  2.73739  3.18146  2.89801
2.37887  2.77519  2.98518  4.58477  3.61503
          0.02185  4.23009  4.95244  0.61958  0.77255  0.48576  0.95510
    120   2.52688  4.88576  3.09901  2.54365  4.18235  3.49014  3.68155
3.58952  2.35990  3.17006  3.73039  3.04209  2.15939  2.55761  2.19831
2.73155  2.93212  3.26084  5.38690  4.07738    150 p - - -
          2.68637  4.42194  2.77474  2.73115  3.46342  2.40521  3.72514
3.29341  2.67737  2.69331  4.24641  2.90354  2.73759  3.18166  2.89820
2.37888  2.77521  2.98518  4.58496  3.61523
          0.25745  1.51454  4.95244  1.41339  0.27881  0.48576  0.95510
    121   2.13603  4.28514  3.61866  3.32878  4.47938  3.06289  4.38723
3.84282  3.36247  3.56971  4.39988  3.45498  3.82160  3.65787  3.66509
0.93810  2.38558  3.28942  5.82253  4.61659    156 s - - -
          2.68618  4.42225  2.77519  2.73123  3.46354  2.40513  3.72494
3.29354  2.67741  2.69355  4.24690  2.90347  2.73739  3.18146  2.89801
2.37887  2.77519  2.98518  4.58477  3.61503
          0.02185  4.23009  4.95244  0.61958  0.77255  0.48576  0.95510
    122   2.42745  4.40576  3.32620  3.09413  4.50563  2.87959  4.27975
3.96577  3.22731  3.61902  4.44121  3.34789  1.21277  3.52732  3.56986
1.90645  2.90192  3.40798  5.81420  4.57203    157 p - - -
          2.68618  4.42225  2.77519  2.73123  3.46354  2.40513  3.72494
3.29354  2.67741  2.69355  4.24690  2.90347  2.73739  3.18146  2.89801
2.37887  2.77519  2.98518  4.58477  3.61503
          0.02185  4.23009  4.95244  0.61958  0.77255  0.48576  0.95510
    123   2.17574  4.57727  3.19814  2.55299  3.57400  2.74937  3.74964
3.09768  2.62765  2.85998  3.71238  3.11819  3.52346  2.96142  3.05422
2.26261  2.62726  2.69427  5.16719  3.88288    158 a - - -
```

2.68618 4.42225 2.77519 2.73123 3.46354 2.40513 3.72494
3.29354 2.67741 2.69355 4.24690 2.90347 2.73739 3.18146 2.89801
2.37887 2.77519 2.98518 4.58477 3.61503
0.02185 4.23009 4.95244 0.61958 0.77255 0.48576 0.95510
124 2.62393 4.73816 3.15713 2.68345 4.30933 3.36320 3.82807
3.73853 2.52860 3.32548 4.14521 3.12638 3.16353 2.99160 2.16521
1.49297 2.93878 3.34353 5.52006 4.22379 159 s - - -
2.68618 4.42225 2.77519 2.73123 3.46354 2.40513 3.72494
3.29354 2.67741 2.69355 4.24690 2.90347 2.73739 3.18146 2.89801
2.37887 2.77519 2.98518 4.58477 3.61503
0.02185 4.23009 4.95244 0.61958 0.77255 0.48576 0.95510
125 2.62503 4.21939 3.74657 3.17547 3.06358 3.48674 3.93734
2.46591 3.09967 1.96057 3.31960 3.50515 4.03747 3.37315 3.38550
1.99294 2.85961 2.38284 4.80555 3.38621 160 l - - -
2.68618 4.42225 2.77519 2.73123 3.46354 2.40513 3.72494
3.29354 2.67741 2.69355 4.24690 2.90347 2.73739 3.18146 2.89801
2.37887 2.77519 2.98518 4.58477 3.61503
0.04243 4.23009 3.61223 0.61958 0.77255 0.48576 0.95510
126 2.72503 5.11917 1.77615 2.32730 4.46498 3.36653 3.36541
3.93356 2.53290 3.46482 4.25218 2.87669 3.06947 2.83768 3.03683
2.11340 2.82734 3.53164 5.64146 4.23796 161 d - - -
2.68618 4.42225 2.77519 2.73123 3.46354 2.40513 3.72494
3.29354 2.67741 2.69355 4.24690 2.90347 2.73739 3.18146 2.89801
2.37887 2.77519 2.98518 4.58477 3.61503
0.08096 4.20997 2.76585 0.61958 0.77255 0.51145 0.91536
127 2.60560 4.65519 3.09991 2.54712 3.44848 3.20791 3.33731
3.25207 2.52957 2.78371 3.73940 2.87110 3.85771 2.55373 2.96789
2.19833 2.83726 2.96751 5.16215 3.03976 162 s - - -
2.68618 4.42225 2.77519 2.73123 3.46354 2.40513 3.72494
3.29354 2.67741 2.69355 4.24690 2.90347 2.73739 3.18146 2.89801
2.37887 2.77519 2.98518 4.58477 3.61503
0.07956 4.15264 2.80087 0.61958 0.77255 0.57863 0.82250
128 2.48853 4.30048 3.18326 3.29381 3.50227 3.81383 4.15273
1.75775 3.24132 2.32136 3.42219 3.64948 4.18994 3.53194 3.55385
3.09838 2.98111 1.64891 5.07695 3.85222 163 v - - -
2.68618 4.42225 2.77519 2.73123 3.46354 2.40513 3.72494
3.29354 2.67741 2.69355 4.24690 2.90347 2.73739 3.18146 2.89801
2.37887 2.77519 2.98518 4.58477 3.61503
0.04561 4.09806 3.57628 0.61958 0.77255 0.63548 0.75434
129 2.47703 4.29960 3.43490 2.88535 3.52291 3.15469 3.83444
2.86444 2.83820 2.59150 3.03032 3.13621 3.63241 3.14885 3.19498
2.66637 2.22652 2.12509 4.95754 3.72105 164 v - - -
2.68618 4.42225 2.77519 2.73123 3.46354 2.40513 3.72494
3.29354 2.67741 2.69355 4.24690 2.90347 2.73739 3.18146 2.89801
2.37887 2.77519 2.98518 4.58477 3.61503
0.07965 4.07794 2.81986 0.61958 0.77255 0.65491 0.73290
130 2.51723 4.14505 3.78784 3.21238 3.23937 3.64627 3.65779
2.27012 3.11278 1.83695 2.99322 3.28173 4.01697 3.38635 3.37406
2.91923 2.82123 2.13960 4.75470 3.54655 165 l - - -
2.68618 4.42225 2.77519 2.73123 3.46354 2.40513 3.72494
3.29354 2.67741 2.69355 4.24690 2.90347 2.73739 3.18146 2.89801
2.37887 2.77519 2.98518 4.58477 3.61503
0.02689 4.02519 4.74753 0.61958 0.77255 0.70242 0.68396
131 1.66884 4.26391 3.34113 2.93999 4.17150 2.27155 4.03889
3.56944 2.96794 3.24273 4.06873 3.21704 2.71445 3.27292 3.33805
2.45106 2.22226 3.10486 5.50475 4.26246 166 a - - -

```
          2.68618  4.42225  2.77519  2.73123  3.46354  2.40513  3.72494
3.29354  2.67741  2.69355  4.24690  2.90347  2.73739  3.18146  2.89801
2.37887  2.77519  2.98518  4.58477  3.61503
          0.02689  4.02519  4.74753  0.61958  0.77255  0.70242  0.68396
    132   2.64510  4.98647  2.56924  1.95098  4.26459  2.61039  3.61756
3.17317  2.33461  3.26539  4.06160  2.66352  3.79232  2.75241  2.90481
2.63193  2.88493  3.34251  5.46619  4.08883    167 e - - -
          2.68628  4.42235  2.77500  2.73134  3.46364  2.40513  3.72478
3.29364  2.67751  2.69365  4.24700  2.90344  2.73735  3.18106  2.89800
2.37868  2.77530  2.98529  4.58487  3.61513
          0.54202  1.15361  2.27372  0.76968  0.62206  0.64558  0.74309
    133   2.57843  4.72703  2.89991  2.24903  3.99065  3.36877  3.63519
2.94124  2.45038  3.01603  3.84396  2.36834  3.39591  2.80117  2.89839
2.49087  2.66493  3.07656  5.27327  3.94876    171 e - - -
          2.68618  4.42225  2.77519  2.73123  3.46354  2.40513  3.72494
3.29354  2.67741  2.69355  4.24690  2.90347  2.73739  3.18146  2.89801
2.37887  2.77519  2.98518  4.58477  3.61503
          0.02865  3.96285  4.68520  0.61958  0.77255  0.71652  0.67031
    134   2.51452  4.54851  3.12444  2.56255  3.56603  3.17659  3.66305
3.12382  2.23444  2.48695  3.45356  2.94340  3.83190  2.87104  2.77731
2.59915  2.68722  2.78153  5.09022  3.80532    172 k - - -
          2.68618  4.42225  2.77519  2.73123  3.46354  2.40513  3.72494
3.29354  2.67741  2.69355  4.24690  2.90347  2.73739  3.18146  2.89801
2.37887  2.77519  2.98518  4.58477  3.61503
          0.02800  3.98540  4.70774  0.61958  0.77255  0.50610  0.92343
    135   2.43214  4.51268  3.21147  2.66439  3.71936  3.29995  3.74334
2.76741  2.63551  2.68673  3.63469  2.21456  3.88175  2.89145  3.04595
2.71717  2.68329  2.49825  5.09806  3.82437    173 n - - -
          2.68618  4.42225  2.77519  2.73123  3.46354  2.40513  3.72494
3.29354  2.67741  2.69355  4.24690  2.90347  2.73739  3.18146  2.89801
2.37887  2.77519  2.98518  4.58477  3.61503
          0.02419  4.12951  4.85186  0.61958  0.77255  0.41861  1.07283
    136   2.72993  5.20158  2.37016  2.03991  4.51729  2.26265  3.48467
3.99490  2.47768  3.49933  4.26978  2.60724  3.00930  2.79105  2.98721
2.57665  2.86637  3.58036  5.65706  4.23950    174 e - - -
          2.68618  4.42225  2.77519  2.73123  3.46354  2.40513  3.72494
3.29354  2.67741  2.69355  4.24690  2.90347  2.73739  3.18146  2.89801
2.37887  2.77519  2.98518  4.58477  3.61503
          0.02185  4.23009  4.95244  0.61958  0.77255  0.48576  0.95510
    137   2.58980  5.01694  2.89823  2.39529  4.35995  2.54152  3.64727
3.81037  2.08386  3.34292  4.11994  2.52026  3.59302  2.76776  2.69413
2.20504  2.89803  3.41970  5.51649  4.14521    175 k - - -
          2.68618  4.42225  2.77519  2.73123  3.46354  2.40513  3.72494
3.29354  2.67741  2.69355  4.24690  2.90347  2.73739  3.18146  2.89801
2.37887  2.77519  2.98518  4.58477  3.61503
          0.07291  4.23009  2.88670  0.61958  0.77255  0.48576  0.95510
    138   2.49055  4.40278  3.19770  2.78773  3.28515  2.88694  3.38859
2.92264  2.75397  2.62141  3.35681  3.11930  3.92492  3.06977  3.13862
2.71325  2.72161  2.31694  4.97231  2.82637    176 v - - -
          2.68618  4.42225  2.77519  2.73123  3.46354  2.40513  3.72494
3.29354  2.67741  2.69355  4.24690  2.90347  2.73739  3.18146  2.89801
2.37887  2.77519  2.98518  4.58477  3.61503
          0.02299  4.18017  4.90252  0.61958  0.77255  0.54741  0.86381
    139   2.50022  5.18478  2.31010  1.90213  4.50760  2.90697  3.66315
3.98514  2.47524  3.49121  4.26348  2.34749  3.82860  2.78424  2.98645
2.58350  2.60448  3.56983  5.65033  4.23207    177 e - - -
```

```
          2.68618  4.42225  2.77519  2.73123  3.46354  2.40513  3.72494
3.29354  2.67741  2.69355  4.24690  2.90347  2.73739  3.18146  2.89801
2.37887  2.77519  2.98518  4.58477  3.61503
          0.02299  4.18017  4.90252  0.61958  0.77255  0.47675  0.96968
    140   2.44159  4.84617  2.98763  2.45313  4.15677  2.49972  3.66639
3.39827  2.29792  3.16977  3.97117  2.56717  3.14643  2.80797  2.91897
2.38725  2.85689  3.09631  5.39397  4.04968    178 k - - -
          2.68618  4.42225  2.77519  2.73123  3.46354  2.40513  3.72494
3.29354  2.67741  2.69355  4.24690  2.90347  2.73739  3.18146  2.89801
2.37887  2.77519  2.98518  4.58477  3.61503
          0.02217  4.21579  4.93814  0.61958  0.77255  0.47444  0.97347
    141   2.33489  4.66936  3.11816  2.57424  3.90482  3.46517  3.71750
3.30007  2.55904  2.79117  3.78613  2.64527  3.87200  2.81820  2.99639
1.92714  2.76672  2.83101  5.23022  3.68360    179 s - - -
          2.68618  4.42225  2.77519  2.73123  3.46354  2.40513  3.72494
3.29354  2.67741  2.69355  4.24690  2.90347  2.73739  3.18146  2.89801
2.37887  2.77519  2.98518  4.58477  3.61503
          0.02185  4.23009  4.95244  0.61958  0.77255  0.48576  0.95510
    142   2.61997  3.71074  2.89714  2.33892  4.00410  3.45961  3.47782
3.41398  2.47984  2.93161  3.85233  3.00310  3.64712  2.82847  2.79210
2.00580  2.64408  3.10282  5.28868  3.58744    180 s - - -
          2.68618  4.42225  2.77519  2.73123  3.46354  2.40513  3.72494
3.29354  2.67741  2.69355  4.24690  2.90347  2.73739  3.18146  2.89801
2.37887  2.77519  2.98518  4.58477  3.61503
          0.10783  4.23009  2.43415  0.61958  0.77255  0.48576  0.95510
    143   2.63529  4.26984  3.71305  3.17026  3.38622  3.62758  3.98633
2.38573  3.07220  1.58254  3.35779  3.50399  3.18686  3.38019  3.36358
2.58062  2.89191  2.44407  4.91203  3.69126    181 l - - -
          2.68618  4.42225  2.77519  2.73123  3.46354  2.40513  3.72494
3.29354  2.67741  2.69355  4.24690  2.90347  2.73739  3.18146  2.89801
2.37887  2.77519  2.98518  4.58477  3.61503
          0.02379  4.14605  4.86840  0.61958  0.77255  0.58583  0.81338
    144   3.20674  4.49539  4.98193  4.43613  3.11352  4.56898  4.97710
1.23958  4.32793  1.56186  3.18409  4.63258  4.80126  4.47559  4.45550
3.92571  3.44411  1.59998  5.35273  4.21069    182 i - - -
          2.68618  4.42225  2.77519  2.73123  3.46354  2.40513  3.72494
3.29354  2.67741  2.69355  4.24690  2.90347  2.73739  3.18146  2.89801
2.37887  2.77519  2.98518  4.58477  3.61503
          0.02379  4.14605  4.86840  0.61958  0.77255  0.58583  0.81338
    145   2.63472  4.81582  2.54037  2.46404  4.34797  3.27846  3.82069
3.78632  2.69668  3.38309  4.20339  2.71830  1.68764  2.99102  3.16514
2.49865  2.69799  3.38556  5.60298  4.25214    183 p - - -
          2.68618  4.42225  2.77519  2.73123  3.46354  2.40513  3.72494
3.29354  2.67741  2.69355  4.24690  2.90347  2.73739  3.18146  2.89801
2.37887  2.77519  2.98518  4.58477  3.61503
          0.05880  4.14605  3.18753  0.61958  0.77255  0.58583  0.81338
    146   2.74543  4.25198  3.98748  3.43763  2.24463  3.77440  3.80629
2.72271  3.32902  2.40965  3.36843  3.65039  4.14274  3.55397  3.54562
2.61986  2.74896  2.53159  2.26751  2.59479    184 f - - -
          2.68618  4.42225  2.77519  2.73123  3.46354  2.40513  3.72494
3.29354  2.67741  2.69355  4.24690  2.90347  2.73739  3.18146  2.89801
2.37887  2.77519  2.98518  4.58477  3.61503
          0.02463  4.11189  4.83423  0.61958  0.77255  0.62167  0.77013
    147   3.28844  4.62954  4.84976  4.31069  3.22192  4.54097  4.91599
1.99386  4.15459  0.91634  3.02718  4.56761  4.77253  4.32909  4.30548
3.90447  3.52855  1.84511  5.28577  4.17170    185 l - - -
```

```
          2.68618  4.42225  2.77519  2.73123  3.46354  2.40513  3.72494
3.29354  2.67741  2.69355  4.24690  2.90347  2.73739  3.18146  2.89801
2.37887  2.77519  2.98518  4.58477  3.61503
          0.06164  4.11189  3.13723  0.61958  0.77255  0.62167  0.77013
    148   2.80492  5.12243  3.07387  2.50768  4.49247  3.15442  3.35940
3.90276  1.58088  3.38155  4.18518  2.79990  3.89980  2.53402  2.10242
2.78404  3.00963  3.53401  5.48890  4.19705    186 k - - -
          2.68618  4.42225  2.77519  2.73123  3.46354  2.40513  3.72494
3.29354  2.67741  2.69355  4.24690  2.90347  2.73739  3.18146  2.89801
2.37887  2.77519  2.98518  4.58477  3.61503
          0.06080  4.07579  3.16989  0.61958  0.77255  0.65694  0.73072
    149   2.50847  3.76414  3.10958  2.60426  3.92616  3.17643  3.49941
3.32881  2.59083  2.97504  3.81383  2.15531  3.80491  2.93898  3.01085
2.49531  2.25818  3.00335  5.25051  3.95825    187 n - - -
          2.68618  4.42225  2.77519  2.73123  3.46354  2.40513  3.72494
3.29354  2.67741  2.69355  4.24690  2.90347  2.73739  3.18146  2.89801
2.37887  2.77519  2.98518  4.58477  3.61503
          0.02645  4.04145  4.76379  0.61958  0.77255  0.68828  0.69804
    150   2.47726  4.30059  3.87115  3.36745  3.38440  3.37489  4.15654
2.31915  3.22869  1.35667  3.32586  3.65996  4.13437  3.55701  3.48802
3.02712  2.98203  2.27300  5.01804  3.80087    188 l - - -
          2.68618  4.42225  2.77519  2.73123  3.46354  2.40513  3.72494
3.29354  2.67741  2.69355  4.24690  2.90347  2.73739  3.18146  2.89801
2.37887  2.77519  2.98518  4.58477  3.61503
          0.04621  4.04145  3.59046  0.61958  0.77255  0.68828  0.69804
    151   2.23975  4.58989  2.93927  2.36990  4.15267  3.02158  3.78151
3.57047  2.63398  3.19076  4.01137  3.00365  3.22615  2.95981  3.07263
1.74936  2.70121  3.18176  5.43364  4.12180    189 s - - -
          2.68618  4.42225  2.77519  2.73123  3.46354  2.40513  3.72494
3.29354  2.67741  2.69355  4.24690  2.90347  2.73739  3.18146  2.89801
2.37887  2.77519  2.98518  4.58477  3.61503
          0.01824  4.01347        *  0.61958  0.77255  0.00000        *
//
HMMER3/f [3.3 | Nov 2019]
NAME  BSD
ACC   PF03909.18
DESC  BSD domain
LENG  58
ALPH  amino
RF    no
MM    no
CONS  yes
CS    no
MAP   yes
DATE  Thu Apr 16 22:39:23 2020
NSEQ  217
EFFN  15.850510
CKSUM 3973210315
GA    24.60 24.60
TC    24.60 24.60
NC    24.50 24.50
STATS LOCAL MSV       -8.2678  0.71898
STATS LOCAL VITERBI   -8.7970  0.71898
STATS LOCAL FORWARD   -4.1983  0.71898
HMM          A        C        D        E        F        G        H
I        K        L        M        N        P        Q        R        S
T        V        W        Y
            m->m     m->i     m->d     i->m     i->i     d->m     d->d
```

```
  COMPO   2.69729  5.01767  2.83458  2.44846  2.85150  3.72357  3.65146
3.05300  2.56086  2.37191  3.81349  3.03209  3.48405  2.88713  2.77295
2.65623  3.03164  2.87768  3.84113  3.22285
          2.68618  4.42225  2.77519  2.73123  3.46354  2.40513  3.72494
3.29354  2.67741  2.69355  4.24690  2.90347  2.73739  3.18146  2.89801
2.37887  2.77519  2.98518  4.58477  3.61503
          0.21330  6.39727  1.65852  0.61958  0.77255  0.00000        *
      1   3.31714  5.96030  1.63667  2.42125  3.19303  2.93430  4.52869
4.72614  3.28073  4.24891  5.01139  3.29984  4.72949  3.43503  3.37229
2.88205  2.94108  2.45234  1.65233  5.03058      1 d - - -
          2.68618  4.42225  2.77519  2.73123  3.46354  2.40513  3.72494
3.29354  2.67741  2.69355  4.24690  2.90347  2.73739  3.18146  2.89801
2.37887  2.77519  2.98518  4.58477  3.61503
          0.00306  6.18703  6.90938  0.61958  0.77255  0.35465  1.20871
      2   2.90396  3.35233  1.35353  2.54438  4.05883  4.43347  4.62152
2.76647  2.31633  3.07443  4.67121  3.03938  4.82672  2.85512  2.83847
2.50606  3.32649  3.87332  6.58258  5.17738      2 d - - -
          2.68618  4.42225  2.77519  2.73123  3.46354  2.40513  3.72494
3.29354  2.67741  2.69355  4.24690  2.90347  2.73739  3.18146  2.89801
2.37887  2.77519  2.98518  4.58477  3.61503
          0.00261  6.34697  7.06932  0.61958  0.77255  0.35476  1.20846
      3   2.49297  6.19957  2.03239  2.23728  3.03182  2.92830  3.77060
5.03261  2.11532  3.19957  5.23992  1.99594  4.85302  2.87860  3.66844
2.27492  3.50989  4.21756  6.63155  5.21989      3 n - - -
          2.68618  4.42225  2.77519  2.73123  3.46354  2.40513  3.72494
3.29354  2.67741  2.69355  4.24690  2.90347  2.73739  3.18146  2.89801
2.37887  2.77519  2.98518  4.58477  3.61503
          0.00250  6.39015  7.11249  0.61958  0.77255  0.43999  1.03295
      4   2.93657  6.20531  2.22681  2.20959  5.55176  2.12046  4.24402
5.03871  2.00791  4.30770  4.48205  2.40889  4.23602  2.30534  2.92974
2.44042  3.12538  3.56368  6.63716  5.22537      4 k - - -
          2.68618  4.42225  2.77519  2.73123  3.46354  2.40513  3.72494
3.29354  2.67741  2.69355  4.24690  2.90347  2.73739  3.18146  2.89801
2.37887  2.77519  2.98518  4.58477  3.61503
          0.00248  6.39727  7.11962  0.61958  0.77255  0.48576  0.95510
      5   3.94416  5.31103  5.92021  5.30478  0.84398  5.11986  5.44779
3.12136  5.08846  1.19372  4.06012  5.28633  5.47170  4.50148  4.60906
4.43368  4.00155  2.55441  5.01864  4.39530      5 f - - -
          2.68618  4.42225  2.77519  2.73123  3.46354  2.40513  3.72494
3.29354  2.67741  2.69355  4.24690  2.90347  2.73739  3.18146  2.89801
2.37887  2.77519  2.98518  4.58477  3.61503
          0.00248  6.39727  7.11962  0.61958  0.77255  0.48576  0.95510
      6   3.69160  6.20256  1.98959  2.55777  4.02820  3.89578  3.64663
4.55639  1.49751  3.11562  4.47125  2.06305  4.85881  3.59273  2.87577
2.31708  3.16746  3.40060  6.63517  4.54960      6 k - - -
          2.68636  4.42243  2.77510  2.73142  3.46322  2.40531  3.72513
3.29372  2.67759  2.69298  4.24264  2.90365  2.73743  3.18165  2.89791
2.37892  2.77538  2.98537  4.58495  3.61521
          0.45462  2.18541  1.37484  0.06433  2.77578  0.48576  0.95510
      7   2.27190  4.97895  5.57376  4.10433  2.79656  4.77475  5.10857
2.08455  4.74169  1.44342  3.33749  4.93888  4.33982  4.87190  4.74800
2.75047  3.22873  1.81294  5.60082  2.49416      8 l - - -
          2.68618  4.42225  2.77519  2.73123  3.46354  2.40513  3.72494
3.29354  2.67741  2.69355  4.24690  2.90347  2.73739  3.18146  2.89801
2.37887  2.77519  2.98518  4.58477  3.61503
          0.00331  6.10682  6.82917  0.61958  0.77255  1.49973  0.25256
```

```
      8   3.17547  4.81110  1.27770  1.51190  5.37245  4.06313  4.47338
4.85922  2.96879  4.33249  5.06788  2.14684  4.67895  3.11580  3.70314
2.58830  3.20312  3.90145  6.45929  5.04721      9 d - - -
          2.68643  4.42313  2.77299  2.72884  3.46442  2.40562  3.72583
3.29289  2.67702  2.69422  4.24778  2.90351  2.73828  3.18055  2.89889
2.37850  2.77600  2.98607  4.58565  3.61591
          0.71768  0.67126  6.85801  0.31179  1.31727  0.11221  2.24295
      9   1.99144  5.69179  4.33827  3.67843  4.47193  4.64099  3.47372
2.60488  1.56529  2.29104  4.12896  3.26122  3.41976  3.19231  3.37731
3.05399  2.74735  2.71919  3.98063  3.59784     14 k - - -
          2.68618  4.42225  2.77519  2.73123  3.46354  2.40513  3.72494
3.29354  2.67741  2.69355  4.24690  2.90347  2.73739  3.18146  2.89801
2.37887  2.77519  2.98518  4.58477  3.61503
          0.00248  6.39727  7.11962  0.61958  0.77255  0.48576  0.95510
     10   3.37206  6.19694  1.78111  3.24412  4.38812  3.94089  4.65401
4.23115  2.50548  4.50119  4.14100  2.62870  4.86012  2.38694  3.60732
2.64454  1.35111  4.28697  6.63108  3.04436     15 t - - -
          2.68618  4.42225  2.77519  2.73123  3.46354  2.40513  3.72494
3.29354  2.67741  2.69355  4.24690  2.90347  2.73739  3.18146  2.89801
2.37887  2.77519  2.98518  4.58477  3.61503
          0.00248  6.39727  7.11962  0.61958  0.77255  0.48576  0.95510
     11   2.18648  6.18901  2.44763  2.22687  3.18919  3.09031  3.46980
2.70263  2.59696  3.07804  4.51625  3.40917  2.91168  2.79023  3.01428
3.00114  3.29174  3.07552  6.62530  3.15599     16 a - - -
          2.68618  4.42225  2.77519  2.73123  3.46354  2.40513  3.72494
3.29354  2.67741  2.69355  4.24690  2.90347  2.73739  3.18146  2.89801
2.37887  2.77519  2.98518  4.58477  3.61503
          0.00248  6.39727  7.11962  0.61958  0.77255  0.48576  0.95510
     12   2.79058  6.20544  2.10719  0.94613  5.55177  4.18357  4.65200
5.03864  2.71690  4.01818  5.24578  3.54110  3.16359  2.46181  3.04400
3.40535  3.55376  3.51963  6.63736  5.22563     17 e - - -
          2.68618  4.42225  2.77519  2.73123  3.46354  2.40513  3.72494
3.29354  2.67741  2.69355  4.24690  2.90347  2.73739  3.18146  2.89801
2.37887  2.77519  2.98518  4.58477  3.61503
          0.00248  6.39727  7.11962  0.61958  0.77255  0.48576  0.95510
     13   3.82788  5.21441  4.22770  5.10619  4.01477  4.98889  2.61357
1.08909  3.78527  1.46730  3.50028  5.12633  5.35333  3.05764  4.94219
4.08723  3.53434  2.57756  3.27428  4.65727     18 i - - -
          2.68618  4.42225  2.77519  2.73123  3.46354  2.40513  3.72494
3.29354  2.67741  2.69355  4.24690  2.90347  2.73739  3.18146  2.89801
2.37887  2.77519  2.98518  4.58477  3.61503
          0.00248  6.39727  7.11962  0.61958  0.77255  0.48576  0.95510
     14   1.47428  6.17663  3.52484  2.49658  5.51017  4.47182  4.66011
2.21386  3.14843  4.47698  4.79582  3.40517  4.86503  1.59224  3.58989
2.17349  3.44167  3.32905  6.61627  5.21238     19 a - - -
          2.68618  4.42225  2.77519  2.73123  3.46354  2.40513  3.72494
3.29354  2.67741  2.69355  4.24690  2.90347  2.73739  3.18146  2.89801
2.37887  2.77519  2.98518  4.58477  3.61503
          0.00248  6.39727  7.11962  0.61958  0.77255  0.48576  0.95510
     15   2.20743  6.20296  2.98202  2.43583  4.85919  3.95134  3.27993
3.98677  2.45130  2.90011  3.60808  2.87806  4.85872  1.67085  2.52141
2.72977  2.75438  3.86721  6.63546  4.21647     20 q - - -
          2.68618  4.42225  2.77519  2.73123  3.46354  2.40513  3.72494
3.29354  2.67741  2.69355  4.24690  2.90347  2.73739  3.18146  2.89801
2.37887  2.77519  2.98518  4.58477  3.61503
          0.00248  6.39727  7.11962  0.61958  0.77255  0.48576  0.95510
```

```
     16   1.99259  5.02949  2.16124  3.57350  4.39646  4.71753  4.96156
2.89768  3.43882  1.57578  3.77260  3.94551  5.09880  3.62953  4.32283
1.56032  3.14681  3.30497  5.13345  4.87544     21 s - - -
          2.68618  4.42225  2.77519  2.73123  3.46354  2.40513  3.72494
3.29354  2.67741  2.69355  4.24690  2.90347  2.73739  3.18146  2.89801
2.37887  2.77519  2.98518  4.58477  3.61503
          0.00248  6.39727  7.11962  0.61958  0.77255  0.48576  0.95510
     17   3.73921  3.81893  5.93482  5.32426  3.69000  5.13899  5.48308
1.74414  5.11168  0.70635  3.36283  5.30671  5.49423  5.23992  5.11735
4.16958  3.27867  2.13306  5.96243  4.78875     22 l - - -
          2.68618  4.42225  2.77519  2.73123  3.46354  2.40513  3.72494
3.29354  2.67741  2.69355  4.24690  2.90347  2.73739  3.18146  2.89801
2.37887  2.77519  2.98518  4.58477  3.61503
          0.00248  6.39727  7.11962  0.61958  0.77255  0.48576  0.95510
     18   3.11341  5.98037  3.55531  1.73655  2.13572  3.76623  4.44575
4.41758  3.05292  1.47811  2.62429  3.19485  4.91809  3.31164  3.57434
2.90092  3.94186  3.54995  6.46890  4.01651     23 l - - -
          2.68618  4.42225  2.77519  2.73123  3.46354  2.40513  3.72494
3.29354  2.67741  2.69355  4.24690  2.90347  2.73739  3.18146  2.89801
2.37887  2.77519  2.98518  4.58477  3.61503
          0.00248  6.39727  7.11962  0.61958  0.77255  0.48576  0.95510
     19   2.69534  6.20516  3.22858  2.05323  5.55155  3.45992  4.03208
5.03846  1.56976  3.38981  4.70457  2.59237  4.85821  2.49445  2.25268
2.63363  2.74461  3.24208  6.63706  5.22531     24 k - - -
          2.68618  4.42225  2.77519  2.73123  3.46354  2.40513  3.72494
3.29354  2.67741  2.69355  4.24690  2.90347  2.73739  3.18146  2.89801
2.37887  2.77519  2.98518  4.58477  3.61503
          0.42888  6.39727  1.05815  0.61958  0.77255  0.48576  0.95510
     20   2.59012  5.90484  3.33650  1.81904  5.24953  4.16902  3.77340
4.30306  1.95240  2.24837  4.94560  3.41938  4.56248  2.28309  3.38697
2.21666  2.32254  4.03297  6.33775  4.92703     25 e - - -
          2.68618  4.42225  2.77519  2.73123  3.46354  2.40513  3.72494
3.29354  2.67741  2.69355  4.24690  2.90347  2.73739  3.18146  2.89801
2.37887  2.77519  2.98518  4.58477  3.61503
          0.00379  5.97218  6.69453  0.61958  0.77255  0.08094  2.55427
     21   2.87662  6.11292  1.53586  3.24083  4.12559  4.13180  2.83886
3.10416  2.96505  4.40171  5.16386  1.99779  4.88091  3.79027  3.91716
2.71228  3.58354  3.54481  6.56925  1.76136     26 d - - -
          2.68618  4.42225  2.77519  2.73123  3.46354  2.40513  3.72494
3.29354  2.67741  2.69355  4.24690  2.90347  2.73739  3.18146  2.89801
2.37887  2.77519  2.98518  4.58477  3.61503
          0.00248  6.39727  7.11962  0.61958  0.77255  0.48576  0.95510
     22   3.00450  6.20425  2.96568  2.44035  5.55021  3.21375  4.33354
5.03681  1.92034  3.70848  4.63596  3.67160  1.05137  3.50956  3.21130
2.83735  3.28985  4.03738  6.63640  5.22491     27 p - - -
          2.68618  4.42225  2.77519  2.73123  3.46354  2.40513  3.72494
3.29354  2.67741  2.69355  4.24690  2.90347  2.73739  3.18146  2.89801
2.37887  2.77519  2.98518  4.58477  3.61503
          0.00653  6.39727  5.32957  0.61958  0.77255  0.48576  0.95510
     23   2.15144  6.19639  2.43844  1.91755  4.34781  3.08835  4.65030
3.52455  2.99685  3.03322  4.69000  2.01158  3.91237  3.27055  3.07082
2.15493  3.34741  3.06613  6.62986  5.21970     28 e - - -
          2.68626  4.42233  2.77528  2.73132  3.46362  2.40500  3.72503
3.29362  2.67749  2.69363  4.24698  2.90355  2.73748  3.18155  2.89809
2.37895  2.77422  2.98527  4.58485  3.61511
          0.05476  2.94745  7.11557  0.12504  2.14101  0.45861  1.00011
```

```
     24   4.38833  5.70936  6.39021  5.79259  4.14965  5.63892  5.99561
2.48309  5.59959  0.54214  3.00898  4.71617  5.93708  5.68678  5.59872
4.97042  3.67110  1.70541  6.38512  5.25217      30 L - - -
          2.68618  4.42225  2.77519  2.73123  3.46354  2.40513  3.72494
3.29354  2.67741  2.69355  4.24690  2.90347  2.73739  3.18146  2.89801
2.37887  2.77519  2.98518  4.58477  3.61503
          0.00248  6.39727  7.11962  0.61958  0.77255  0.48576  0.95510
     25   2.30871  6.20427  3.57530  2.16512  4.37123  3.98411  3.18022
5.03688  2.08071  3.70438  3.77830  3.27231  4.52227  2.35809  1.59046
2.49664  3.17078  4.30873  4.65232  4.63824      31 r - - -
          2.68618  4.42225  2.77519  2.73123  3.46354  2.40513  3.72494
3.29354  2.67741  2.69355  4.24690  2.90347  2.73739  3.18146  2.89801
2.37887  2.77519  2.98518  4.58477  3.61503
          0.00248  6.39727  7.11962  0.61958  0.77255  0.48576  0.95510
     26   2.44809  6.20527  3.36899  2.40959  5.55172  3.17888  3.89396
4.42472  1.70401  4.51114  4.83582  2.60452  4.85819  2.14079  1.92257
2.49567  2.88740  4.00820  6.63714  5.22536      32 k - - -
          2.68618  4.42225  2.77519  2.73123  3.46354  2.40513  3.72494
3.29354  2.67741  2.69355  4.24690  2.90347  2.73739  3.18146  2.89801
2.37887  2.77519  2.98518  4.58477  3.61503
          0.00248  6.39727  7.11962  0.61958  0.77255  0.48576  0.95510
     27   2.16009  5.21265  5.73012  5.11899  3.41974  4.99079  5.32108
2.09255  3.16466  1.55162  2.17981  4.51793  5.35509  5.06030  3.30613
3.21801  1.95304  2.49997  5.83348  4.65607      33 l - - -
          2.68618  4.42225  2.77519  2.73123  3.46354  2.40513  3.72494
3.29354  2.67741  2.69355  4.24690  2.90347  2.73739  3.18146  2.89801
2.37887  2.77519  2.98518  4.58477  3.61503
          0.00248  6.39727  7.11962  0.61958  0.77255  0.48576  0.95510
     28   3.83456  5.28595  5.39465  4.79950  1.82780  4.94963  3.02447
3.73080  3.95331  3.09194  1.80180  4.96517  5.31710  4.19494  1.92395
3.75308  4.06605  3.23631  5.88436  1.44873      34 y - - -
          2.68618  4.42225  2.77519  2.73123  3.46354  2.40513  3.72494
3.29354  2.67741  2.69355  4.24690  2.90347  2.73739  3.18146  2.89801
2.37887  2.77519  2.98518  4.58477  3.61503
          0.00248  6.39727  7.11962  0.61958  0.77255  0.48576  0.95510
     29   2.70251  6.14000  2.45216  2.01311  2.80059  4.20830  4.67117
3.02724  2.71025  3.13699  2.86758  1.96730  4.87386  3.37789  3.35233
3.04091  2.75131  3.89933  6.58930  2.79627      35 n - - -
          2.68618  4.42225  2.77519  2.73123  3.46354  2.40513  3.72494
3.29354  2.67741  2.69355  4.24690  2.90347  2.73739  3.18146  2.89801
2.37887  2.77519  2.98518  4.58477  3.61503
          0.00248  6.39727  7.11962  0.61958  0.77255  0.48576  0.95510
     30   3.02488  6.20586  2.43383  1.06242  5.55230  4.46511  4.65210
5.03923  1.86166  4.14939  5.24618  2.85262  4.85868  2.92840  2.53254
2.95741  3.38977  4.04606  6.63769  5.22592      36 e - - -
          2.68618  4.42225  2.77519  2.73123  3.46354  2.40513  3.72494
3.29354  2.67741  2.69355  4.24690  2.90347  2.73739  3.18146  2.89801
2.37887  2.77519  2.98518  4.58477  3.61503
          0.00248  6.39727  7.11962  0.61958  0.77255  0.48576  0.95510
     31   1.93233  4.32975  5.78341  5.16875  3.80674  4.28895  4.71274
2.51602  4.95582  1.02043  3.37578  2.35336  5.36177  5.08891  4.96751
2.85513  3.36414  2.71008  5.82771  3.46124      37 l - - -
          2.68715  4.42451  2.77581  2.73057  3.46533  2.40726  3.72540
3.29549  2.67432  2.69186  4.24809  2.90519  2.73465  3.18236  2.89780
2.37791  2.77373  2.98602  4.58703  3.61729
          0.26483  1.46164  7.11962  1.74504  0.19193  0.48576  0.95510
```

```
     32   4.57630  2.63130  6.65849  6.12509  5.15817  6.00622  6.52282
2.07830  5.99019  2.75936  3.27312  6.17605  6.29909  6.18124  6.06208
4.53810  4.27129  0.44853  6.94785  5.74433     45 V - - -
          2.68618  4.42225  2.77519  2.73123  3.46354  2.40513  3.72494
3.29354  2.67741  2.69355  4.24690  2.90347  2.73739  3.18146  2.89801
2.37887  2.77519  2.98518  4.58477  3.61503
          0.00248  6.39727  7.11962  0.61958  0.77255  0.48576  0.95510
     33   4.43730  6.77953  4.18904  3.98445  6.33965  4.93927  4.30893
5.80349  3.58923  5.28988  6.10556  3.53788  0.42642  4.05429  4.51456
1.90379  3.64630  5.35697  7.41475  6.04379     46 P - - -
          2.68618  4.42225  2.77519  2.73123  3.46354  2.40513  3.72494
3.29354  2.67741  2.69355  4.24690  2.90347  2.73739  3.18146  2.89801
2.37887  2.77519  2.98518  4.58477  3.61503
          0.00248  6.39727  7.11962  0.61958  0.77255  0.48576  0.95510
     34   2.51161  4.73912  2.48726  2.29641  4.84209  3.19228  2.93795
3.83652  2.20657  3.77521  5.24366  2.87087  3.56067  3.47849  2.55192
2.06794  3.15242  2.40582  6.63556  4.60491     47 s - - -
          2.68618  4.42225  2.77519  2.73123  3.46354  2.40513  3.72494
3.29354  2.67741  2.69355  4.24690  2.90347  2.73739  3.18146  2.89801
2.37887  2.77519  2.98518  4.58477  3.61503
          0.06966  6.39727  2.72376  0.61958  0.77255  0.48576  0.95510
     35   2.10334  6.15407  3.20067  2.41267  4.89988  2.79885  3.12050
3.80682  1.80915  3.59939  4.68589  3.39596  3.68181  2.83533  2.60154
2.54769  2.94033  2.98042  6.58648  3.33341     48 k - - -
          2.68618  4.42225  2.77519  2.73123  3.46354  2.40513  3.72494
3.29354  2.67741  2.69355  4.24690  2.90347  2.73739  3.18146  2.89801
2.37887  2.77519  2.98518  4.58477  3.61503
          0.00265  6.33026  7.05260  0.61958  0.77255  0.25005  1.50851
     36   2.93834  5.20798  5.78662  4.37005  3.93780  5.00069  5.33398
2.04512  4.95902  1.78248  1.89188  5.16011  5.36436  2.44773  4.97051
3.49035  3.35052  1.35656  5.83033  4.65416     49 v - - -
          2.68618  4.42225  2.77519  2.73123  3.46354  2.40513  3.72494
3.29354  2.67741  2.69355  4.24690  2.90347  2.73739  3.18146  2.89801
2.37887  2.77519  2.98518  4.58477  3.61503
          0.00248  6.39727  7.11962  0.61958  0.77255  0.48576  0.95510
     37   3.20824  6.20361  2.20122  2.56404  2.01403  4.12414  4.65242
5.03546  2.57145  3.89750  5.24416  2.44138  2.94351  3.43775  3.74495
1.56288  2.52079  4.59450  6.63601  5.22475     50 s - - -
          2.68618  4.42225  2.77519  2.73123  3.46354  2.40513  3.72494
3.29354  2.67741  2.69355  4.24690  2.90347  2.73739  3.18146  2.89801
2.37887  2.77519  2.98518  4.58477  3.61503
          0.00248  6.39727  7.11962  0.61958  0.77255  0.48576  0.95510
     38   3.14953  6.19996  3.10819  1.28175  5.54393  4.46607  2.79595
5.02912  3.31216  4.50478  4.29832  2.28938  3.13804  3.57254  3.59745
3.12368  3.30268  3.43320  6.63331  1.74742     51 e - - -
          2.68618  4.42225  2.77519  2.73123  3.46354  2.40513  3.72494
3.29354  2.67741  2.69355  4.24690  2.90347  2.73739  3.18146  2.89801
2.37887  2.77519  2.98518  4.58477  3.61503
          0.00248  6.39727  7.11962  0.61958  0.77255  0.48576  0.95510
     39   2.13211  6.20523  2.92864  1.80183  5.55166  2.65504  4.16512
5.03859  2.20075  4.28876  4.37697  2.34062  4.13066  2.85399  3.15164
2.03411  3.16363  3.58465  6.63711  4.35272     52 e - - -
          2.68618  4.42225  2.77519  2.73123  3.46354  2.40513  3.72494
3.29354  2.67741  2.69355  4.24690  2.90347  2.73739  3.18146  2.89801
2.37887  2.77519  2.98518  4.58477  3.61503
          0.00248  6.39727  7.11962  0.61958  0.77255  0.48576  0.95510
```

```
     40   3.39968  4.09659  2.02034  1.81338  4.70032  3.96802  4.65636
4.35405  3.02110  2.84166  4.01629  3.28281  4.44502  1.72398  3.16146
3.15822  2.19210  3.46340  6.62521  3.81631     53 q - - -
          2.68618  4.42225  2.77519  2.73123  3.46354  2.40513  3.72494
3.29354  2.67741  2.69355  4.24690  2.90347  2.73739  3.18146  2.89801
2.37887  2.77519  2.98518  4.58477  3.61503
          0.00248  6.39727  7.11962  0.61958  0.77255  0.48576  0.95510
     41   7.23162  7.95227  7.59454  7.67317  0.01774  6.79070  6.86139
7.27129  7.72113  6.29306  7.74272  7.45883  7.35156  7.65835  7.46270
7.33199  7.56574  7.23053  6.96257  5.33161     54 F - - -
          2.68618  4.42225  2.77519  2.73123  3.46354  2.40513  3.72494
3.29354  2.67741  2.69355  4.24690  2.90347  2.73739  3.18146  2.89801
2.37887  2.77519  2.98518  4.58477  3.61503
          0.00248  6.39727  7.11962  0.61958  0.77255  0.48576  0.95510
     42   6.60302  7.42934  7.32164  7.36361  3.51381  6.89097  5.57507
5.98875  7.10845  4.28897  6.45324  6.51056  7.17187  6.69749  6.83964
6.42929  6.81115  6.06081  0.09069  3.88057     55 W - - -
          2.68618  4.42225  2.77519  2.73123  3.46354  2.40513  3.72494
3.29354  2.67741  2.69355  4.24690  2.90347  2.73739  3.18146  2.89801
2.37887  2.77519  2.98518  4.58477  3.61503
          0.00248  6.39727  7.11962  0.61958  0.77255  0.48576  0.95510
     43   2.81846  3.61528  3.95956  3.11060  4.59236  4.03591  3.86417
4.41794  2.24371  3.24613  3.97990  3.22288  4.86783  2.51339  2.33594
1.39957  2.29259  4.54608  6.60734  3.21083     56 s - - -
          2.68618  4.42225  2.77519  2.73123  3.46354  2.40513  3.72494
3.29354  2.67741  2.69355  4.24690  2.90347  2.73739  3.18146  2.89801
2.37887  2.77519  2.98518  4.58477  3.61503
          0.00248  6.39727  7.11962  0.61958  0.77255  0.48576  0.95510
     44   3.07887  5.06589  4.12928  3.57095  5.14103  4.55428  4.75867
2.31351  2.62200  3.97178  4.23516  2.78723  4.94378  3.55101  1.03165
2.85896  2.05496  3.63074  6.41182  5.08009     57 r - - -
          2.68618  4.42225  2.77519  2.73123  3.46354  2.40513  3.72494
3.29354  2.67741  2.69355  4.24690  2.90347  2.73739  3.18146  2.89801
2.37887  2.77519  2.98518  4.58477  3.61503
          0.00248  6.39727  7.11962  0.61958  0.77255  0.48576  0.95510
     45   6.63448  7.40854  7.26717  7.29039  1.86014  6.92114  5.42854
6.16927  6.87415  5.20527  6.64232  6.38705  7.15774  6.56981  1.49389
6.35606  6.80591  6.14479  5.49517  0.53052     58 Y - - -
          2.68618  4.42225  2.77519  2.73123  3.46354  2.40513  3.72494
3.29354  2.67741  2.69355  4.24690  2.90347  2.73739  3.18146  2.89801
2.37887  2.77519  2.98518  4.58477  3.61503
          0.00248  6.39727  7.11962  0.61958  0.77255  0.48576  0.95510
     46   3.95200  4.28818  5.93307  5.32259  0.75844  5.13633  5.46352
2.45548  5.10856  2.34610  4.39489  5.30147  5.49102  5.23208  5.11173
4.45173  3.31921  2.36202  5.93312  2.15535     59 f - - -
          2.68618  4.42225  2.77519  2.73123  3.46354  2.40513  3.72494
3.29354  2.67741  2.69355  4.24690  2.90347  2.73739  3.18146  2.89801
2.37887  2.77519  2.98518  4.58477  3.61503
          0.00248  6.39727  7.11962  0.61958  0.77255  0.48576  0.95510
     47   3.57646  5.87669  4.15069  3.49520  2.40202  3.49208  2.21483
3.93288  2.79456  3.16524  4.95107  2.75866  4.22371  2.56828  3.43120
2.50824  3.01046  2.76095  4.92317  1.74441     60 y - - -
          2.68618  4.42225  2.77519  2.73123  3.46354  2.40513  3.72494
3.29354  2.67741  2.69355  4.24690  2.90347  2.73739  3.18146  2.89801
2.37887  2.77519  2.98518  4.58477  3.61503
          0.00248  6.39727  7.11962  0.61958  0.77255  0.48576  0.95510
```

```
     48   3.10069  6.11693  3.98585  2.98839  5.42528  4.48758  3.90664
4.01257  2.39616  1.23067  3.98792  3.40423  4.88008  3.14984  2.06605
1.92584  3.09843  3.91031  6.57220  5.18464     61 l - - -
          2.68618  4.42225  2.77519  2.73123  3.46354  2.40513  3.72494
3.29354  2.67741  2.69355  4.24690  2.90347  2.73739  3.18146  2.89801
2.37887  2.77519  2.98518  4.58477  3.61503
          0.00248  6.39727  7.11962  0.61958  0.77255  0.48576  0.95510
     49   3.46235  5.74585  3.88669  2.86641  4.32949  4.61659  3.02545
2.82179  2.57755  1.35653  4.83126  4.20684  5.00309  2.99563  1.96102
3.83216  3.82680  1.91359  6.28393  4.57956     62 l - - -
          2.68618  4.42225  2.77519  2.73123  3.46354  2.40513  3.72494
3.29354  2.67741  2.69355  4.24690  2.90347  2.73739  3.18146  2.89801
2.37887  2.77519  2.98518  4.58477  3.61503
          0.00248  6.39727  7.11962  0.61958  0.77255  0.48576  0.95510
     50   3.28483  6.19326  3.17960  2.92258  4.38235  4.14448  1.97509
5.01735  2.86728  2.31261  4.71192  2.97858  4.86099  3.07349  1.48543
2.75354  3.75181  4.58171  5.48166  2.36762     63 r - - -
          2.68618  4.42225  2.77519  2.73123  3.46354  2.40513  3.72494
3.29354  2.67741  2.69355  4.24690  2.90347  2.73739  3.18146  2.89801
2.37887  2.77519  2.98518  4.58477  3.61503
          0.00248  6.39727  7.11962  0.61958  0.77255  0.48576  0.95510
     51   1.67534  4.29242  4.00338  2.98886  2.47055  3.63563  4.07533
4.83429  1.88377  2.68662  3.73777  3.12135  3.54556  2.73995  3.44575
2.40211  3.33644  3.19771  6.54873  4.34869     64 a - - -
          2.68618  4.42225  2.77519  2.73123  3.46354  2.40513  3.72494
3.29354  2.67741  2.69355  4.24690  2.90347  2.73739  3.18146  2.89801
2.37887  2.77519  2.98518  4.58477  3.61503
          0.00248  6.39727  7.11962  0.61958  0.77255  0.48576  0.95510
     52   3.48417  5.23959  5.53986  4.93991  3.26550  4.96225  1.55339
1.61768  2.66535  1.65675  4.35689  4.28016  5.32854  4.95220  2.89389
3.92441  3.66994  2.85227  5.85706  3.27558     65 h - - -
          2.68618  4.42225  2.77519  2.73123  3.46354  2.40513  3.72494
3.29354  2.67741  2.69355  4.24690  2.90347  2.73739  3.18146  2.89801
2.37887  2.77519  2.98518  4.58477  3.61503
          0.00248  6.39727  7.11962  0.61958  0.77255  0.48576  0.95510
     53   1.58146  4.65980  3.25433  2.09626  4.88395  4.47824  4.66777
3.09885  1.70250  2.46122  5.19768  3.95490  4.87115  2.89745  2.28700
3.39202  3.80182  3.49244  6.59745  5.20051     66 a - - -
          2.68618  4.42225  2.77519  2.73123  3.46354  2.40513  3.72494
3.29354  2.67741  2.69355  4.24690  2.90347  2.73739  3.18146  2.89801
2.37887  2.77519  2.98518  4.58477  3.61503
          0.00248  6.39727  7.11962  0.61958  0.77255  0.48576  0.95510
     54   2.89447  6.16437  2.55875  2.46159  5.49260  4.47480  4.04453
1.80313  2.69838  2.19249  4.46590  3.01453  4.86787  1.91391  3.89507
2.89427  2.60891  3.46657  6.60726  5.20668     67 i - - -
          2.68618  4.42225  2.77519  2.73123  3.46354  2.40513  3.72494
3.29354  2.67741  2.69355  4.24690  2.90347  2.73739  3.18146  2.89801
2.37887  2.77519  2.98518  4.58477  3.61503
          0.00729  6.39727  5.18546  0.61958  0.77255  0.48576  0.95510
     55   2.25305  6.20127  2.79384  1.45297  5.54751  4.20417  4.36397
5.03430  2.05303  3.91431  3.68234  3.92829  4.24697  3.04701  1.97946
2.36819  3.12190  3.80610  6.63326  5.22161     68 e - - -
          2.68618  4.42225  2.77519  2.73123  3.46354  2.40513  3.72494
3.29354  2.67741  2.69355  4.24690  2.90347  2.73739  3.18146  2.89801
2.37887  2.77519  2.98518  4.58477  3.61503
          0.02573  6.39247  3.74106  0.61958  0.77255  0.53893  0.87556
```

```
     56   3.02188  6.18364  2.77691  1.27874  5.52964  2.47708  3.45979
3.22246  2.78684  3.15601  5.22406  2.88594  4.83775  2.78743  2.93298
3.06575  2.58410  4.15107  6.61577  3.81450     69 e - - -
          2.68618  4.42225  2.77519  2.73123  3.46354  2.40513  3.72494
3.29354  2.67741  2.69355  4.24690  2.90347  2.73739  3.18146  2.89801
2.37887  2.77519  2.98518  4.58477  3.61503
          0.06002  6.36929  2.87273  0.61958  0.77255  0.75935  0.63105
     57   2.29762  6.14270  1.99931  1.71326  4.80872  3.77728  4.24722
4.97597  2.50798  4.44854  5.18302  2.28048  4.79577  2.55359  3.56485
2.03462  3.32502  3.21305  6.57460  5.16286     70 e - - -
          2.68618  4.42225  2.77519  2.73123  3.46354  2.40513  3.72494
3.29354  2.67741  2.69355  4.24690  2.90347  2.73739  3.18146  2.89801
2.37887  2.77519  2.98518  4.58477  3.61503
          0.20537  6.31196  1.69370  0.61958  0.77255  1.14456  0.38326
     58   2.10272  6.22297  4.07720  3.21437  5.61359  4.52536  4.62648
5.05812  1.74155  3.48869  5.26874  3.99618  4.90387  1.20222  1.71832
3.30043  3.99634  4.64164  6.59116  5.25320     71 q - - -
          2.68618  4.42225  2.77519  2.73123  3.46354  2.40513  3.72494
3.29354  2.67741  2.69355  4.24690  2.90347  2.73739  3.18146  2.89801
2.37887  2.77519  2.98518  4.58477  3.61503
          0.00223  6.10882        *  0.61958  0.77255  0.00000        *
//
HMMER3/f [3.3 | Nov 2019]
NAME  BTB
ACC   PF00651.32
DESC  BTB/POZ domain
LENG  111
ALPH  amino
RF    no
MM    no
CONS  yes
CS    yes
MAP   yes
DATE  Tue Apr 21 09:46:14 2020
NSEQ  83
EFFN  5.899261
CKSUM 3696956457
GA    20.50 20.50
TC    20.50 20.50
NC    20.40 20.40
STATS LOCAL MSV       -9.6452  0.71513
STATS LOCAL VITERBI  -10.4888  0.71513
STATS LOCAL FORWARD   -4.0502  0.71513
HMM          A        C        D        E        F        G        H
I        K        L        M        N        P        Q        R        S
T        V        W        Y
            m->m     m->i     m->d     i->m     i->i     d->m     d->d
  COMPO   2.53297  3.97624  2.94324  2.64399  3.05810  3.32449  3.57657
2.84330  2.69871  2.22451  3.52552  3.14503  3.57057  3.05624  3.05337
2.62695  2.89024  2.67421  5.28338  3.30607
          2.68618  4.42225  2.77519  2.73123  3.46354  2.40513  3.72494
3.29354  2.67741  2.69355  4.24690  2.90347  2.73739  3.18146  2.89801
2.37887  2.77519  2.98518  4.58477  3.61503
          0.02094  5.48996  4.09846  0.61958  0.77255  0.00000        *
      1   3.05362  3.98247  5.13435  4.51938  1.87432  4.34425  4.67746
2.67410  3.82922  1.11859  2.75316  3.80918  4.70765  4.43705  4.31497
3.65541  3.40732  2.38006  5.17192  3.05991      1 l - - H
```

2.68618 4.42225 2.77519 2.73123 3.46354 2.40513 3.72494
3.29354 2.67741 2.69355 4.24690 2.90347 2.73739 3.18146 2.89801
2.37887 2.77519 2.98518 4.58477 3.61503
0.00624 5.47526 6.19760 0.61958 0.77255 0.55077 0.85921
2 3.10065 5.60113 2.51535 2.38790 3.37042 3.04209 3.64992
3.68441 2.08994 3.38741 4.33043 1.68502 4.26938 2.69709 3.19018
2.72761 2.94064 3.68616 6.03638 3.85851 2 n - - H
2.68618 4.42225 2.77519 2.73123 3.46354 2.40513 3.72494
3.29354 2.67741 2.69355 4.24690 2.90347 2.73739 3.18146 2.89801
2.37887 2.77519 2.98518 4.58477 3.61503
0.00624 5.47526 6.19760 0.61958 0.77255 0.55077 0.85921
3 2.48448 5.61294 2.41426 1.83217 4.95889 3.16335 3.66634
4.44549 2.30392 3.24607 4.12296 2.88178 3.39829 2.44166 2.88509
2.31814 3.11514 3.48210 6.04509 4.29680 3 e - - H
2.68618 4.42225 2.77519 2.73123 3.46354 2.40513 3.72494
3.29354 2.67741 2.69355 4.24690 2.90347 2.73739 3.18146 2.89801
2.37887 2.77519 2.98518 4.58477 3.61503
0.00624 5.47526 6.19760 0.61958 0.77255 0.55077 0.85921
4 2.98134 4.31987 4.34235 3.76580 2.81253 4.20577 4.02705
3.06529 3.38985 1.41560 3.26427 3.67975 3.41719 2.06686 3.95194
2.50976 2.98302 2.88489 5.32180 3.01256 4 l - - H
2.68618 4.42225 2.77519 2.73123 3.46354 2.40513 3.72494
3.29354 2.67741 2.69355 4.24690 2.90347 2.73739 3.18146 2.89801
2.37887 2.77519 2.98518 4.58477 3.61503
0.00624 5.47526 6.19760 0.61958 0.77255 0.55077 0.85921
5 2.91580 5.10606 3.68928 2.57512 2.45124 4.01263 4.02561
3.67685 2.89611 2.12654 2.84141 3.09249 4.00088 3.21698 1.63005
2.87346 3.35624 3.40720 4.60979 3.71944 5 r - - H
2.68618 4.42225 2.77519 2.73123 3.46354 2.40513 3.72494
3.29354 2.67741 2.69355 4.24690 2.90347 2.73739 3.18146 2.89801
2.37887 2.77519 2.98518 4.58477 3.61503
0.00624 5.47526 6.19760 0.61958 0.77255 0.55077 0.85921
6 2.76853 4.09774 2.33563 1.92957 4.95666 3.68721 3.71921
4.44277 2.19009 2.72092 4.65200 2.33742 4.26747 2.42562 2.75568
2.58099 3.00320 4.00214 4.90557 4.63289 6 e - - H
2.68618 4.42225 2.77519 2.73123 3.46354 2.40513 3.72494
3.29354 2.67741 2.69355 4.24690 2.90347 2.73739 3.18146 2.89801
2.37887 2.77519 2.98518 4.58477 3.61503
0.06271 5.47526 2.87177 0.61958 0.77255 0.55077 0.85921
7 2.77452 3.44685 3.21827 2.34369 4.25358 3.18273 3.26134
4.39769 2.22372 2.81619 4.61458 2.55202 3.26775 2.30271 2.82145
2.17488 3.05353 3.96160 6.00786 3.58727 7 s - - H
2.68618 4.42225 2.77519 2.73123 3.46354 2.40513 3.72494
3.29354 2.67741 2.69355 4.24690 2.90347 2.73739 3.18146 2.89801
2.37887 2.77519 2.98518 4.58477 3.61503
0.14853 5.41915 2.01293 0.61958 0.77255 0.65571 0.73204
8 2.76726 4.84543 2.81826 1.98236 4.85153 1.82510 3.61469
3.87534 2.16095 3.81401 4.55211 2.98592 3.09724 2.93719 3.03830
2.78320 2.97238 3.89893 4.06380 3.89858 8 g - - T
2.68606 4.42236 2.77515 2.73125 3.46304 2.40507 3.72506
3.29365 2.67741 2.69366 4.24701 2.90358 2.73737 3.18084 2.89812
2.37898 2.77531 2.98529 4.58488 3.61514
0.19433 1.92835 3.46652 0.41190 1.08586 0.79707 0.59902
9 2.49406 4.74952 2.71972 2.18550 3.29295 3.81235 2.86988
3.25846 2.24662 2.93710 4.49174 3.29571 4.20486 2.49202 3.06887
2.29254 2.89201 2.90636 5.89664 4.50912 11 e - - T

2.68618 4.42225 2.77519 2.73123 3.46354 2.40513 3.72494
3.29354 2.67741 2.69355 4.24690 2.90347 2.73739 3.18146 2.89801
2.37887 2.77519 2.98518 4.58477 3.61503
0.00730 5.32002 6.04236 0.61958 0.77255 0.71066 0.67594
10 3.10054 4.62173 3.86281 3.65284 1.86266 3.17147 3.80266
2.97439 3.73657 1.60247 3.20214 3.69721 3.95463 3.34417 3.63994
3.45341 2.92440 2.87859 4.36593 2.28317 12 l - - T
2.68618 4.42225 2.77519 2.73123 3.46354 2.40513 3.72494
3.29354 2.67741 2.69355 4.24690 2.90347 2.73739 3.18146 2.89801
2.37887 2.77519 2.98518 4.58477 3.61503
0.00689 5.37744 6.09978 0.61958 0.77255 0.73713 0.65101
11 2.71192 1.48986 4.73042 4.13642 3.17197 4.24576 3.86900
2.91651 3.99221 2.59073 3.67817 4.28358 3.16081 4.18771 4.12387
2.01341 2.46795 2.33651 4.38558 3.99080 13 c - - S
2.68618 4.42225 2.77519 2.73123 3.46354 2.40513 3.72494
3.29354 2.67741 2.69355 4.24690 2.90347 2.73739 3.18146 2.89801
2.37887 2.77519 2.98518 4.58477 3.61503
0.00673 5.40090 6.12324 0.61958 0.77255 0.32349 1.28598
12 4.27488 7.09316 0.31064 2.86922 6.37468 4.26044 4.97904
6.00023 4.24199 5.41846 6.37038 2.95502 4.98535 4.17548 5.02553
4.07225 3.11173 5.48875 7.57028 5.88948 14 D - - -
2.68618 4.42225 2.77519 2.73123 3.46354 2.40513 3.72494
3.29354 2.67741 2.69355 4.24690 2.90347 2.73739 3.18146 2.89801
2.37887 2.77519 2.98518 4.58477 3.61503
0.00615 5.48996 6.21230 0.61958 0.77255 0.48576 0.95510
13 2.52187 3.00873 4.37801 4.63621 3.56034 4.47407 4.82208
1.90517 4.43140 2.06495 3.57265 4.63260 4.83143 4.57046 4.44997
3.78978 3.09206 1.03293 5.31476 4.13611 15 v - - E
2.68618 4.42225 2.77519 2.73123 3.46354 2.40513 3.72494
3.29354 2.67741 2.69355 4.24690 2.90347 2.73739 3.18146 2.89801
2.37887 2.77519 2.98518 4.58477 3.61503
0.00615 5.48996 6.21230 0.61958 0.77255 0.48576 0.95510
14 2.42625 4.19705 4.20379 3.48426 3.47079 4.17841 4.09047
2.63881 2.86149 2.59509 3.89384 3.98748 4.55458 2.97326 3.32448
2.82766 1.47988 2.02922 5.37966 3.68338 16 t - - E
2.68618 4.42225 2.77519 2.73123 3.46354 2.40513 3.72494
3.29354 2.67741 2.69355 4.24690 2.90347 2.73739 3.18146 2.89801
2.37887 2.77519 2.98518 4.58477 3.61503
0.00615 5.48996 6.21230 0.61958 0.77255 0.48576 0.95510
15 3.87866 3.36641 5.89458 5.31147 2.34745 5.18576 5.56033
1.39561 5.13769 0.95994 3.85296 5.35626 5.44127 5.20119 5.13728
4.53210 4.10861 2.15838 5.88441 4.77271 17 l - - E
2.68618 4.42225 2.77519 2.73123 3.46354 2.40513 3.72494
3.29354 2.67741 2.69355 4.24690 2.90347 2.73739 3.18146 2.89801
2.37887 2.77519 2.98518 4.58477 3.61503
0.00615 5.48996 6.21230 0.61958 0.77255 0.48576 0.95510
16 2.30518 5.12009 3.29774 2.99497 4.30578 4.01855 3.77290
3.22821 2.40975 2.29030 2.71583 3.61690 4.40464 2.46475 2.68705
3.23578 3.20509 1.98055 5.66186 3.16012 18 v - - E
2.68618 4.42225 2.77519 2.73123 3.46354 2.40513 3.72494
3.29354 2.67741 2.69355 4.24690 2.90347 2.73739 3.18146 2.89801
2.37887 2.77519 2.98518 4.58477 3.61503
0.00615 5.48996 6.21230 0.61958 0.77255 0.48576 0.95510
17 2.27402 2.63606 5.12517 4.51249 3.19110 3.84894 4.68969
2.38350 4.30394 2.21857 3.68612 4.50947 4.71938 4.44069 4.32221
2.87992 2.73562 1.17843 5.19143 4.01469 19 v - - E

```
          2.68618  4.42225  2.77519  2.73123  3.46354  2.40513  3.72494
3.29354  2.67741  2.69355  4.24690  2.90347  2.73739  3.18146  2.89801
2.37887  2.77519  2.98518  4.58477  3.61503
          0.00615  5.48996  6.21230  0.61958  0.77255  0.48576  0.95510
     18   2.89759  5.62027  2.37835  1.90213  4.96587  1.81227  4.06890
4.14197  2.21177  3.62875  4.66080  3.02020  4.27537  2.42182  2.84827
2.79961  3.33952  3.72267  6.05262  4.00089     20 g - - T
          2.68618  4.42225  2.77519  2.73123  3.46354  2.40513  3.72494
3.29354  2.67741  2.69355  4.24690  2.90347  2.73739  3.18146  2.89801
2.37887  2.77519  2.98518  4.58477  3.61503
          0.52276  5.48996  0.90885  0.61958  0.77255  0.48576  0.95510
     19   2.74093  5.34288  1.85331  2.26637  4.67230  3.08334  3.83728
3.38490  2.39554  3.33142  4.38782  2.15099  4.04123  2.93899  3.06942
2.74185  2.71865  3.72511  5.78494  3.52304     21 d - - T
          2.68638  4.42256  2.77496  2.73116  3.46385  2.40481  3.72492
3.29385  2.67759  2.69377  4.24721  2.90273  2.73745  3.18159  2.89770
2.37873  2.77550  2.98531  4.58508  3.61534
          0.97905  1.18718  1.14177  0.91619  0.51089  0.14528  2.00086
     20   2.44895  5.51106  2.38751  2.41644  4.85527  1.71392  3.67656
4.34069  2.43058  3.81596  4.55199  2.61541  4.16950  2.87142  2.87286
2.68493  2.59754  3.90125  5.07492  4.53368     26 g - - T
          2.68618  4.42225  2.77519  2.73123  3.46354  2.40513  3.72494
3.29354  2.67741  2.69355  4.24690  2.90347  2.73739  3.18146  2.89801
2.37887  2.77519  2.98518  4.58477  3.61503
          0.02470  5.28018  3.94742  0.61958  0.77255  0.27044  1.43989
     21   2.70205  5.56051  3.36274  2.07060  4.88681  3.55714  2.96805
3.21210  2.03557  2.80678  4.12167  3.35169  4.26967  2.27622  2.38852
2.65750  2.89682  2.95392  6.00458  4.60527     27 k - - E
          2.68618  4.42225  2.77519  2.73123  3.46354  2.40513  3.72494
3.29354  2.67741  2.69355  4.24690  2.90347  2.73739  3.18146  2.89801
2.37887  2.77519  2.98518  4.58477  3.61503
          0.03512  5.46323  3.49762  0.61958  0.77255  0.42027  1.06965
     22   2.88046  5.56025  3.06021  1.92836  3.95827  3.87588  3.42240
3.32699  2.14394  3.27534  3.39655  3.35083  3.50989  2.77967  2.21424
2.50949  2.52327  3.52136  4.98084  4.08976     28 e - - E
          2.68618  4.42225  2.77519  2.73123  3.46354  2.40513  3.72494
3.29354  2.67741  2.69355  4.24690  2.90347  2.73739  3.18146  2.89801
2.37887  2.77519  2.98518  4.58477  3.61503
          0.00633  5.46211  6.18445  0.61958  0.77255  0.41793  1.07414
     23   3.20028  4.57787  4.21374  4.52277  1.35328  4.36765  4.69553
1.78135  4.31421  2.07485  3.51853  4.51999  4.72987  4.44908  3.59937
3.67821  3.43234  2.32291  5.19218  2.45068     29 f - - E
          2.68618  4.42225  2.77519  2.73123  3.46354  2.40513  3.72494
3.29354  2.67741  2.69355  4.24690  2.90347  2.73739  3.18146  2.89801
2.37887  2.77519  2.98518  4.58477  3.61503
          0.00615  5.48996  6.21230  0.61958  0.77255  0.48576  0.95510
     24   3.01377  5.43181  3.45224  2.89519  3.24399  3.92376  2.36107
3.72942  2.00507  3.70559  3.68770  3.20738  2.50755  2.87146  2.11269
2.83323  3.23932  3.78292  4.64101  2.68365     30 k - - E
          2.68618  4.42225  2.77519  2.73123  3.46354  2.40513  3.72494
3.29354  2.67741  2.69355  4.24690  2.90347  2.73739  3.18146  2.89801
2.37887  2.77519  2.98518  4.58477  3.61503
          0.00615  5.48996  6.21230  0.61958  0.77255  0.48576  0.95510
     25   1.10889  4.06163  4.56924  3.98677  3.44852  3.92478  4.00967
2.95427  3.87813  2.87563  3.79293  4.21844  3.20796  4.10907  4.08144
2.88773  2.56688  1.83354  5.28791  4.09293     31 a - - E
```

2.68618 4.42225 2.77519 2.73123 3.46354 2.40513 3.72494
3.29354 2.67741 2.69355 4.24690 2.90347 2.73739 3.18146 2.89801
2.37887 2.77519 2.98518 4.58477 3.61503
0.00615 5.48996 6.21230 0.61958 0.77255 0.48576 0.95510
26 3.24592 3.10019 3.37186 3.22905 4.44517 4.06742 0.82373
3.87552 3.25264 2.85254 4.38934 2.75631 4.50575 3.59200 3.67205
3.08949 3.50002 3.59183 5.80587 4.49925 32 h - - -
2.68618 4.42225 2.77519 2.73123 3.46354 2.40513 3.72494
3.29354 2.67741 2.69355 4.24690 2.90347 2.73739 3.18146 2.89801
2.37887 2.77519 2.98518 4.58477 3.61503
0.00615 5.48996 6.21230 0.61958 0.77255 0.48576 0.95510
27 3.77133 6.04692 4.19652 3.48290 5.52026 4.44461 2.84562
4.88245 0.93285 4.28178 5.10355 3.92501 4.79020 3.49560 1.29473
3.16221 3.35839 4.51750 4.88039 5.09746 33 k - - H
2.68618 4.42225 2.77519 2.73123 3.46354 2.40513 3.72494
3.29354 2.67741 2.69355 4.24690 2.90347 2.73739 3.18146 2.89801
2.37887 2.77519 2.98518 4.58477 3.61503
0.00615 5.48996 6.21230 0.61958 0.77255 0.48576 0.95510
28 1.55894 3.92387 4.91660 4.31552 2.53785 3.89730 4.64104
2.78457 3.35286 1.75307 3.14307 3.53568 4.68606 4.11810 3.55085
3.12360 3.15806 2.08123 5.20807 3.61240 34 a - - H
2.68618 4.42225 2.77519 2.73123 3.46354 2.40513 3.72494
3.29354 2.67741 2.69355 4.24690 2.90347 2.73739 3.18146 2.89801
2.37887 2.77519 2.98518 4.58477 3.61503
0.00615 5.48996 6.21230 0.61958 0.77255 0.48576 0.95510
29 2.71914 4.79061 5.44639 4.85367 2.81409 4.69526 5.06471
1.29259 4.66005 2.54206 3.89524 4.85729 5.03630 4.80090 4.68302
4.02103 3.69033 1.08377 5.53356 3.26338 35 v - - H
2.68618 4.42225 2.77519 2.73123 3.46354 2.40513 3.72494
3.29354 2.67741 2.69355 4.24690 2.90347 2.73739 3.18146 2.89801
2.37887 2.77519 2.98518 4.58477 3.61503
0.00615 5.48996 6.21230 0.61958 0.77255 0.48576 0.95510
30 4.65668 5.84050 6.62358 6.03263 3.94829 6.06541 6.31506
2.92490 5.89519 0.28418 3.26233 6.24177 6.02172 5.67270 5.78790
5.46937 4.84864 2.62033 6.22145 4.17319 36 L - - H
2.68618 4.42225 2.77519 2.73123 3.46354 2.40513 3.72494
3.29354 2.67741 2.69355 4.24690 2.90347 2.73739 3.18146 2.89801
2.37887 2.77519 2.98518 4.58477 3.61503
0.00615 5.48996 6.21230 0.61958 0.77255 0.48576 0.95510
31 1.15890 2.87116 4.51973 3.94216 3.82757 3.90279 4.56891
3.08291 3.84214 2.37549 3.65667 4.19180 4.62674 3.73480 4.06510
1.74373 3.41405 2.71571 5.32566 4.12781 37 a - - H
2.68618 4.42225 2.77519 2.73123 3.46354 2.40513 3.72494
3.29354 2.67741 2.69355 4.24690 2.90347 2.73739 3.18146 2.89801
2.37887 2.77519 2.98518 4.58477 3.61503
0.00615 5.48996 6.21230 0.61958 0.77255 0.48576 0.95510
32 1.09394 3.76788 4.02746 3.57358 3.26658 3.91505 4.49325
3.02471 3.68696 2.63156 2.94017 3.66274 4.58641 3.69553 3.54771
2.31793 3.21704 2.72629 5.33314 3.50290 38 a - - H
2.68618 4.42225 2.77519 2.73123 3.46354 2.40513 3.72494
3.29354 2.67741 2.69355 4.24690 2.90347 2.73739 3.18146 2.89801
2.37887 2.77519 2.98518 4.58477 3.61503
0.00615 5.48996 6.21230 0.61958 0.77255 0.48576 0.95510
33 2.37341 2.02978 3.74297 3.18141 3.84804 3.51326 2.69110
3.62778 3.15980 3.30080 3.83664 3.28130 4.42226 3.27792 1.80426
2.48186 3.14523 3.20688 4.55604 3.37011 39 r - - H

```
          2.68618  4.42225  2.77519  2.73123  3.46354  2.40513  3.72494
3.29354  2.67741  2.69355  4.24690  2.90347  2.73739  3.18146  2.89801
2.37887  2.77519  2.98518  4.58477  3.61503
          0.00615  5.48996  6.21230  0.61958  0.77255  0.48576  0.95510
     34   2.43570  4.38050  4.63472  3.55836  5.24422  3.58016  5.20369
4.20930  4.18050  4.33520  5.15111  4.35189  4.72232  4.46573  4.51275
0.39486  3.37640  4.14123  6.59047  5.38174     40 S - - B
          2.68618  4.42225  2.77519  2.73123  3.46354  2.40513  3.72494
3.29354  2.67741  2.69355  4.24690  2.90347  2.73739  3.18146  2.89801
2.37887  2.77519  2.98518  4.58477  3.61503
          0.00615  5.48996  6.21230  0.61958  0.77255  0.48576  0.95510
     35   2.91669  5.61884  2.83115  2.09541  4.96391  3.45720  3.01235
4.44989  2.61367  3.92412  4.31822  2.78607  1.82755  2.87729  3.04524
2.23438  2.67441  3.45533  6.05152  4.00053     41 p - - H
          2.68618  4.42225  2.77519  2.73123  3.46354  2.40513  3.72494
3.29354  2.67741  2.69355  4.24690  2.90347  2.73739  3.18146  2.89801
2.37887  2.77519  2.98518  4.58477  3.61503
          0.00615  5.48996  6.21230  0.61958  0.77255  0.48576  0.95510
     36   3.03562  4.58607  5.13068  4.52078  2.14741  4.37152  4.28602
2.74209  4.31336  2.49761  3.50183  4.51807  4.73424  4.44868  3.91488
3.68185  3.43965  1.77063  5.17370  1.18069     42 y - - H
          2.68618  4.42225  2.77519  2.73123  3.46354  2.40513  3.72494
3.29354  2.67741  2.69355  4.24690  2.90347  2.73739  3.18146  2.89801
2.37887  2.77519  2.98518  4.58477  3.61503
          0.00615  5.48996  6.21230  0.61958  0.77255  0.48576  0.95510
     37   4.23068  5.50452  6.11905  5.54122  0.47851  5.48914  5.65617
2.95384  5.36935  1.84396  3.47607  5.61673  4.38097  5.32069  5.33529
4.84402  4.44441  3.12901  5.82222  4.62325     43 F - - H
          2.68618  4.42225  2.77519  2.73123  3.46354  2.40513  3.72494
3.29354  2.67741  2.69355  4.24690  2.90347  2.73739  3.18146  2.89801
2.37887  2.77519  2.98518  4.58477  3.61503
          0.00615  5.48996  6.21230  0.61958  0.77255  0.48576  0.95510
     38   2.82703  4.54955  2.90128  2.40704  4.96199  3.56030  3.31913
4.44753  1.59382  3.22138  4.65831  2.83704  4.27570  2.78469  1.94868
2.58570  3.33941  4.00782  5.17657  3.26960     44 k - - H
          2.68618  4.42225  2.77519  2.73123  3.46354  2.40513  3.72494
3.29354  2.67741  2.69355  4.24690  2.90347  2.73739  3.18146  2.89801
2.37887  2.77519  2.98518  4.58477  3.61503
          0.00615  5.48996  6.21230  0.61958  0.77255  0.48576  0.95510
     39   1.81958  4.82109  2.85949  2.38031  4.92496  3.73125  4.07568
3.83971  2.02069  3.71862  3.48122  2.82072  4.28057  2.89280  2.90776
2.41758  2.70953  2.63919  6.03138  4.62760     45 a - - H
          2.68618  4.42225  2.77519  2.73123  3.46354  2.40513  3.72494
3.29354  2.67741  2.69355  4.24690  2.90347  2.73739  3.18146  2.89801
2.37887  2.77519  2.98518  4.58477  3.61503
          0.00615  5.48996  6.21230  0.61958  0.77255  0.48576  0.95510
     40   2.93051  4.60825  4.82683  4.23035  3.54107  4.30767  4.62278
2.53152  3.43253  1.16382  1.70013  4.36012  4.67462  2.94937  3.91835
3.37552  3.17422  2.85835  5.22414  3.82090     46 l - - H
          2.68618  4.42225  2.77519  2.73123  3.46354  2.40513  3.72494
3.29354  2.67741  2.69355  4.24690  2.90347  2.73739  3.18146  2.89801
2.37887  2.77519  2.98518  4.58477  3.61503
          0.00615  5.48996  6.21230  0.61958  0.77255  0.48576  0.95510
     41   2.98902  4.59335  5.12630  4.00844  0.93933  4.37903  4.70742
2.79212  4.31155  1.72794  3.29872  4.52448  4.73976  4.44926  4.33668
3.43765  3.44496  2.62290  5.20890  3.60263     47 f - - H
```

```
          2.68618  4.42225  2.77519  2.73123  3.46354  2.40513  3.72494
3.29354  2.67741  2.69355  4.24690  2.90347  2.73739  3.18146  2.89801
2.37887  2.77519  2.98518  4.58477  3.61503
          0.00615  5.48996  6.21230  0.61958  0.77255  0.48576  0.95510
     42   2.83347  4.77082  3.05600  2.27164  3.36264  3.40473  3.84810
3.88352  2.56522  2.47606  4.56755  2.58123  4.29599  2.98779  2.96813
2.25238  1.99322  3.08484  5.97438  3.88272     48 t - - T
          2.68618  4.42225  2.77519  2.73123  3.46354  2.40513  3.72494
3.29354  2.67741  2.69355  4.24690  2.90347  2.73739  3.18146  2.89801
2.37887  2.77519  2.98518  4.58477  3.61503
          0.00615  5.48996  6.21230  0.61958  0.77255  0.48576  0.95510
     43   2.95408  5.61986  2.87733  2.27218  4.96528  2.06247  3.01128
4.45152  2.64070  3.36342  4.66044  2.15005  3.61600  2.75429  3.17845
2.00741  2.93266  3.64132  6.05233  4.64114     49 s - - S
          2.68618  4.42225  2.77519  2.73123  3.46354  2.40513  3.72494
3.29354  2.67741  2.69355  4.24690  2.90347  2.73739  3.18146  2.89801
2.37887  2.77519  2.98518  4.58477  3.61503
          0.01482  5.48996  4.54832  0.61958  0.77255  0.48576  0.95510
     44   2.93704  4.61896  2.52648  2.52864  3.36257  2.43515  3.27565
4.44292  2.14684  3.48512  4.26596  2.28853  3.18860  2.38834  3.20447
2.72656  2.61318  4.00319  6.04589  4.63529     50 k - - T
          2.68574  4.42335  2.77611  2.73174  3.46190  2.40352  3.72605
3.29298  2.67684  2.69415  4.24506  2.90406  2.73832  3.18256  2.89911
2.37879  2.77441  2.98450  4.58503  3.61613
          1.33287  1.03538  0.96446  1.50988  0.24966  0.52450  0.89613
     45   2.66913  5.32616  3.04827  2.19284  4.64573  2.37972  3.49676
4.11411  2.35344  3.31791  3.70099  3.14016  3.00041  2.85009  2.65995
2.64845  2.76831  3.45423  5.77440  2.61838     66 e - - T
          2.68618  4.42225  2.77519  2.73123  3.46354  2.40513  3.72494
3.29354  2.67741  2.69355  4.24690  2.90347  2.73739  3.18146  2.89801
2.37887  2.77519  2.98518  4.58477  3.61503
          0.23208  5.00464  1.60739  0.61958  0.77255  0.43554  1.04104
     46   2.79728  4.98564  2.84929  2.18728  2.78607  2.82698  4.07953
2.11635  2.60741  2.74614  2.80697  3.28831  4.24672  3.28214  3.37952
2.63139  2.97091  3.20453  5.52430  4.23268     67 i - - S
          2.68618  4.42225  2.77519  2.73123  3.46354  2.40513  3.72494
3.29354  2.67741  2.69355  4.24690  2.90347  2.73739  3.18146  2.89801
2.37887  2.77519  2.98518  4.58477  3.61503
          0.00821  5.20198  5.92432  0.61958  0.77255  0.44095  1.03121
     47   2.22588  3.73514  2.77985  2.10971  3.93253  2.96791  4.02495
4.08622  2.11092  3.42745  4.29843  3.02375  3.50121  2.52927  2.87546
2.27317  2.89528  3.95583  6.00110  4.06261     68 e - - T
          2.68618  4.42225  2.77519  2.73123  3.46354  2.40513  3.72494
3.29354  2.67741  2.69355  4.24690  2.90347  2.73739  3.18146  2.89801
2.37887  2.77519  2.98518  4.58477  3.61503
          0.00671  5.40320  6.12555  0.61958  0.77255  0.58849  0.81005
     48   2.44574  4.55377  2.27861  1.64257  4.93671  2.79630  4.04183
3.76865  2.28023  3.62752  4.63230  2.44463  3.82547  2.73498  3.16963
2.94326  2.86795  3.81053  6.02433  4.61337     69 e - - T
          2.68618  4.42225  2.77519  2.73123  3.46354  2.40513  3.72494
3.29354  2.67741  2.69355  4.24690  2.90347  2.73739  3.18146  2.89801
2.37887  2.77519  2.98518  4.58477  3.61503
          0.01383  5.43865  4.66847  0.61958  0.77255  0.63321  0.75691
     49   2.21775  4.23570  2.81785  2.49725  4.91873  3.45738  3.09968
3.08417  2.12111  3.21097  3.76177  2.76328  3.81127  2.51362  2.89010
2.10224  3.31330  3.54812  6.01540  4.60788     70 s - - E
```

2.68618 4.42225 2.77519 2.73123 3.46354 2.40513 3.72494
3.29354 2.67741 2.69355 4.24690 2.90347 2.73739 3.18146 2.89801
2.37887 2.77519 2.98518 4.58477 3.61503
0.01860 5.44003 4.26267 0.61958 0.77255 0.62207 0.76967
50 2.71953 5.59263 2.67190 2.27048 4.93834 2.63457 2.78268
4.42477 2.48706 3.52398 4.63312 2.36420 3.09460 3.00700 3.05387
2.13226 2.45134 3.98361 6.02492 3.85650 71 s - - S
2.68618 4.42225 2.77519 2.73123 3.46354 2.40513 3.72494
3.29354 2.67741 2.69355 4.24690 2.90347 2.73739 3.18146 2.89801
2.37887 2.77519 2.98518 4.58477 3.61503
0.18842 5.43763 1.78747 0.61958 0.77255 0.66179 0.72552
51 2.78993 4.94734 3.37759 3.06051 3.46867 3.65298 4.14157
2.93840 2.42461 2.77706 3.28571 3.06349 4.30025 2.30157 3.21840
2.58005 2.87486 1.71617 5.49788 3.91411 72 v - - C
2.68618 4.42225 2.77519 2.73123 3.46354 2.40513 3.72494
3.29354 2.67741 2.69355 4.24690 2.90347 2.73739 3.18146 2.89801
2.37887 2.77519 2.98518 4.58477 3.61503
0.01697 5.26293 4.45273 0.61958 0.77255 1.08837 0.41063
52 2.82085 3.31316 2.60950 2.46680 4.27616 3.79840 3.99314
2.96726 2.48731 3.12671 4.40696 2.88200 3.06472 2.81946 3.02539
2.19785 2.38493 2.68102 5.82049 4.21455 73 s - - E
2.68657 4.42265 2.77559 2.73136 3.46393 2.40515 3.72446
3.29188 2.67720 2.69382 4.24667 2.90324 2.73727 3.18144 2.89840
2.37857 2.77559 2.98558 4.58516 3.61395
0.40124 1.62271 2.01630 1.27643 0.32716 0.38590 1.13892
53 3.02622 5.38232 3.34203 1.74196 4.24373 3.65380 3.34043
3.32120 2.78387 3.17088 3.43741 2.83008 4.21798 2.50057 2.52043
2.81661 2.17925 2.72838 5.85201 4.47975 82 e - - E
2.68618 4.42225 2.77519 2.73123 3.46354 2.40513 3.72494
3.29354 2.67741 2.69355 4.24690 2.90347 2.73739 3.18146 2.89801
2.37887 2.77519 2.98518 4.58477 3.61503
0.00728 5.32157 6.04392 0.61958 0.77255 0.25436 1.49350
54 3.07237 4.56328 5.13479 3.94581 3.26076 4.35525 3.55720
1.30716 4.30945 1.58383 3.39678 4.51245 4.71861 4.44360 4.32336
3.66605 3.18626 1.79274 5.18614 3.45095 83 i - - E
2.68618 4.42225 2.77519 2.73123 3.46354 2.40513 3.72494
3.29354 2.67741 2.69355 4.24690 2.90347 2.73739 3.18146 2.89801
2.37887 2.77519 2.98518 4.58477 3.61503
0.11176 5.48996 2.28662 0.61958 0.77255 0.48576 0.95510
55 3.00481 5.49994 2.84311 2.03466 4.81823 3.83980 3.65833
3.08948 2.69537 3.13408 3.88362 2.80571 2.95722 2.86464 2.68417
2.69467 2.14614 2.86566 5.94899 3.14376 84 e - - E
2.68618 4.42225 2.77519 2.73123 3.46354 2.40513 3.72494
3.29354 2.67741 2.69355 4.24690 2.90347 2.73739 3.18146 2.89801
2.37887 2.77519 2.98518 4.58477 3.61503
0.08755 5.38504 2.53521 0.61958 0.77255 0.86313 0.54791
56 3.06683 4.49528 3.80003 3.42458 3.30712 4.19952 4.06521
1.70991 3.97917 1.30610 2.92428 4.25706 3.38374 4.16535 4.09246
2.97278 3.29894 2.29975 5.11172 3.92751 85 l - - E
2.68618 4.42225 2.77519 2.73123 3.46354 2.40513 3.72494
3.29354 2.67741 2.69355 4.24690 2.90347 2.73739 3.18146 2.89801
2.37887 2.77519 2.98518 4.58477 3.61503
0.00741 5.30489 6.02724 0.61958 0.77255 0.24393 1.53037
57 2.94918 4.55264 2.06302 2.09791 4.96514 3.31800 3.89079
3.87370 2.30832 3.43585 4.19187 2.69109 2.61013 2.68674 2.65348
2.49355 2.87811 3.67142 6.05213 4.64094 86 d - - T

2.68618 4.42225 2.77519 2.73123 3.46354 2.40513 3.72494
3.29354 2.67741 2.69355 4.24690 2.90347 2.73739 3.18146 2.89801
2.37887 2.77519 2.98518 4.58477 3.61503
0.00615 5.48996 6.21230 0.61958 0.77255 0.48576 0.95510
58 2.94108 4.82723 1.56908 2.16256 2.80858 2.77905 3.83687
3.97310 2.62130 3.39619 4.22199 2.49225 3.79459 3.00629 3.13711
2.96668 3.34064 3.52756 6.02556 4.16736 87 d - - T
2.68618 4.42225 2.77519 2.73123 3.46354 2.40513 3.72494
3.29354 2.67741 2.69355 4.24690 2.90347 2.73739 3.18146 2.89801
2.37887 2.77519 2.98518 4.58477 3.61503
0.00615 5.48996 6.21230 0.61958 0.77255 0.48576 0.95510
59 2.87202 3.18034 5.11338 3.70251 2.95944 4.02389 4.03283
1.58043 4.29186 2.46498 2.54203 4.49881 3.81993 4.04258 4.31096
3.54931 3.18248 1.41746 5.18142 4.00475 88 v - - S
2.68618 4.42225 2.77519 2.73123 3.46354 2.40513 3.72494
3.29354 2.67741 2.69355 4.24690 2.90347 2.73739 3.18146 2.89801
2.37887 2.77519 2.98518 4.58477 3.61503
0.00615 5.48996 6.21230 0.61958 0.77255 0.48576 0.95510
60 3.10815 5.62074 2.34473 1.92456 3.98660 3.02792 3.64220
4.45318 2.35805 3.92637 4.66119 2.54679 3.28849 2.83182 3.02384
1.98695 2.68455 3.19981 6.05293 4.64147 89 e - - G
2.68618 4.42225 2.77519 2.73123 3.46354 2.40513 3.72494
3.29354 2.67741 2.69355 4.24690 2.90347 2.73739 3.18146 2.89801
2.37887 2.77519 2.98518 4.58477 3.61503
0.00615 5.48996 6.21230 0.61958 0.77255 0.48576 0.95510
61 2.00878 4.27211 4.60598 4.02052 2.63522 4.27067 3.46959
3.10941 3.90549 2.77539 3.77385 4.23944 1.43553 4.12989 4.09566
2.63490 3.40613 2.70661 4.64236 2.40042 90 p - - H
2.68619 4.42226 2.77520 2.73124 3.46323 2.40514 3.72495
3.29355 2.67742 2.69356 4.24691 2.90347 2.73740 3.18147 2.89802
2.37888 2.77520 2.98519 4.58478 3.61504
0.02094 3.97808 6.21230 0.42219 1.06598 0.48576 0.95510
62 2.65109 4.19949 2.42713 1.61368 4.96330 3.54721 3.77518
4.44915 2.41196 3.30428 4.31878 3.06310 3.38003 2.83424 2.90266
2.15540 2.76592 3.45248 6.05119 4.64033 92 e - - H
2.68621 4.42228 2.77522 2.73126 3.46357 2.40516 3.72497
3.29357 2.67744 2.69358 4.24693 2.90350 2.73742 3.18112 2.89776
2.37890 2.77522 2.98521 4.58480 3.61506
0.02934 3.61524 6.21230 0.65684 0.73082 0.48576 0.95510
63 1.91195 5.24598 2.26789 2.55436 4.46274 3.31256 4.18538
2.42083 3.00977 3.49468 4.09172 3.29068 4.36417 3.35474 3.46302
2.42406 2.81731 2.03648 5.76428 3.95713 95 a - - H
2.68618 4.42225 2.77519 2.73123 3.46354 2.40513 3.72494
3.29354 2.67741 2.69355 4.24690 2.90347 2.73739 3.18146 2.89801
2.37887 2.77519 2.98518 4.58477 3.61503
0.00615 5.48996 6.21230 0.61958 0.77255 0.48576 0.95510
64 2.66908 4.67179 5.26912 4.65760 1.23111 4.06718 4.82684
2.70546 4.44840 1.61642 2.87962 4.65166 4.84042 4.57099 4.45712
3.80521 3.53848 1.93404 5.30029 4.13352 96 f - - H
2.68618 4.42225 2.77519 2.73123 3.46354 2.40513 3.72494
3.29354 2.67741 2.69355 4.24690 2.90347 2.73739 3.18146 2.89801
2.37887 2.77519 2.98518 4.58477 3.61503
0.00615 5.48996 6.21230 0.61958 0.77255 0.48576 0.95510
65 2.52388 4.52671 2.90880 1.77090 4.52842 2.63123 3.35138
4.05435 2.11975 3.46568 4.65893 3.22644 3.82906 2.40473 2.32576
2.88732 3.33932 3.71562 6.05104 3.86114 97 e - - H

```
         2.68618  4.42225  2.77519  2.73123  3.46354  2.40513  3.72494
3.29354  2.67741  2.69355  4.24690  2.90347  2.73739  3.18146  2.89801
2.37887  2.77519  2.98518  4.58477  3.61503
         0.00615  5.48996  6.21230  0.61958  0.77255  0.48576  0.95510
     66  1.69466  4.94586  3.09834  2.42090  3.72199  3.91877  4.11443
2.52520  2.52581  2.69934  3.69390  2.74419  3.48197  2.87859  2.89066
2.66690  3.11644  3.80517  5.92479  4.06274     98 a - - H
         2.68618  4.42225  2.77519  2.73123  3.46354  2.40513  3.72494
3.29354  2.67741  2.69355  4.24690  2.90347  2.73739  3.18146  2.89801
2.37887  2.77519  2.98518  4.58477  3.61503
         0.00615  5.48996  6.21230  0.61958  0.77255  0.48576  0.95510
     67  4.48141  5.69227  6.48565  5.89475  2.78979  5.86534  6.16447
1.76125  5.74916  0.76335  2.09040  6.04974  5.90416  5.59022  5.66232
5.25147  4.68410  2.15755  6.16742  5.21732     99 l - - H
         2.68618  4.42225  2.77519  2.73123  3.46354  2.40513  3.72494
3.29354  2.67741  2.69355  4.24690  2.90347  2.73739  3.18146  2.89801
2.37887  2.77519  2.98518  4.58477  3.61503
         0.00615  5.48996  6.21230  0.61958  0.77255  0.48576  0.95510
     68  4.71005  5.87709  6.70919  6.11750  3.18850  6.14925  6.40866
2.32691  5.99099  0.46411  2.40355  6.34161  6.06928  5.72647  5.86115
5.56422  4.89886  2.37986  6.26378  5.37611    100 L - - H
         2.68618  4.42225  2.77519  2.73123  3.46354  2.40513  3.72494
3.29354  2.67741  2.69355  4.24690  2.90347  2.73739  3.18146  2.89801
2.37887  2.77519  2.98518  4.58477  3.61503
         0.00615  5.48996  6.21230  0.61958  0.77255  0.48576  0.95510
     69  3.03664  4.63789  2.27999  1.73435  4.96660  3.59579  3.10416
4.45316  2.47067  3.58174  4.66114  2.52778  4.27505  2.71570  2.36535
2.33453  3.05065  3.66821  6.05288  3.69184    101 e - - H
         2.68618  4.42225  2.77519  2.73123  3.46354  2.40513  3.72494
3.29354  2.67741  2.69355  4.24690  2.90347  2.73739  3.18146  2.89801
2.37887  2.77519  2.98518  4.58477  3.61503
         0.02125  5.48996  4.08052  0.61958  0.77255  0.48576  0.95510
     70  3.17849  3.49318  5.12974  4.51609  1.27849  4.34600  4.67291
2.65004  4.30340  2.74283  3.50963  4.50364  4.70961  4.43589  4.31518
3.05512  3.02536  2.34219  4.22294  1.71393    102 f - - H
         2.68618  4.42225  2.77519  2.73123  3.46354  2.40513  3.72494
3.29354  2.67741  2.69355  4.24690  2.90347  2.73739  3.18146  2.89801
2.37887  2.77519  2.98518  4.58477  3.61503
         0.00624  5.47495  6.19730  0.61958  0.77255  0.55207  0.85745
     71  2.20819  3.70668  5.14319  4.52848  3.27836  4.35160  4.68594
1.57260  4.31426  1.64058  1.89733  4.51345  4.71463  4.44582  4.11523
3.40164  3.19841  2.54609  5.17943  4.00362    103 i - - H
         2.68621  4.42228  2.77522  2.73126  3.46357  2.40500  3.72497
3.29357  2.67743  2.69358  4.24692  2.90349  2.73742  3.18149  2.89803
2.37889  2.77522  2.98521  4.58480  3.61453
         0.02815  3.66042  6.19730  0.65553  0.73224  0.44643  1.02140
     72  5.49031  6.43547  6.14716  6.08191  2.98017  5.90333  3.20477
3.93443  5.74825  4.26573  5.65115  5.43451  6.18111  5.58880  5.62647
5.33595  5.69126  5.09017  4.65900  0.20106    106 Y - - H
         2.68618  4.42225  2.77519  2.73123  3.46354  2.40513  3.72494
3.29354  2.67741  2.69355  4.24690  2.90347  2.73739  3.18146  2.89801
2.37887  2.77519  2.98518  4.58477  3.61503
         0.01890  5.48996  4.22699  0.61958  0.77255  0.48576  0.95510
     73  2.62730  4.12235  3.21149  2.80843  4.72673  2.96878  3.89312
4.16932  2.87401  3.45397  4.50502  3.29508  2.60310  3.22982  2.86368
2.27904  1.37994  3.80182  5.92031  3.54077    107 t - - H
```

```
          2.68618  4.42225  2.77519  2.73123  3.46354  2.40513  3.72494
3.29354  2.67741  2.69355  4.24690  2.90347  2.73739  3.18146  2.89801
2.37887  2.77519  2.98518  4.58477  3.61503
          0.00623  5.47729  6.19963  0.61958  0.77255  0.45208  1.01144
     74   2.25055  4.84214  1.95214  2.36521  4.94116  1.63261  3.78690
4.09682  2.64373  3.90574  4.64639  3.25868  3.61542  3.17377  3.30463
2.39415  3.34188  3.66592  6.04086  4.63432    108 g - - S
          2.68618  4.42225  2.77519  2.73123  3.46354  2.40513  3.72494
3.29354  2.67741  2.69355  4.24690  2.90347  2.73739  3.18146  2.89801
2.37887  2.77519  2.98518  4.58477  3.61503
          0.03177  5.48996  3.60662  0.61958  0.77255  0.48576  0.95510
     75   2.60817  5.60091  3.01065  1.95609  4.31848  3.61948  3.41392
3.91885  2.21544  3.74296  4.32272  2.70001  3.35061  2.99667  2.45520
2.68581  2.15250  2.94748  6.03472  4.15237    109 e - - E
          2.68618  4.42225  2.77519  2.73123  3.46354  2.40513  3.72494
3.29354  2.67741  2.69355  4.24690  2.90347  2.73739  3.18146  2.89801
2.37887  2.77519  2.98518  4.58477  3.61503
          0.02017  5.46450  4.15184  0.61958  0.77255  0.59525  0.80168
     76   2.55491  4.15462  5.01109  4.40300  3.01562  3.28454  4.64056
2.07858  3.61344  1.38212  3.66580  3.89096  4.67751  3.94241  4.25768
3.61923  2.26188  1.93499  5.16765  3.98907    110 l - - E
          2.68618  4.42225  2.77519  2.73123  3.46354  2.40513  3.72494
3.29354  2.67741  2.69355  4.24690  2.90347  2.73739  3.18146  2.89801
2.37887  2.77519  2.98518  4.58477  3.61503
          0.00640  5.45072  6.17307  0.61958  0.77255  0.64880  0.73955
     77   2.74594  4.47936  2.43532  2.26427  3.52618  3.65963  3.89077
3.03822  2.70309  3.14966  3.88283  2.54839  2.80069  2.78897  2.89096
2.38431  2.63227  3.26566  5.97425  4.00283    111 e - - E
          2.68618  4.42225  2.77519  2.73123  3.46354  2.40513  3.72494
3.29354  2.67741  2.69355  4.24690  2.90347  2.73739  3.18146  2.89801
2.37887  2.77519  2.98518  4.58477  3.61503
          0.00640  5.45072  6.17307  0.61958  0.77255  0.64880  0.73955
     78   2.94847  4.77185  3.34667  3.21749  3.06151  4.14994  4.41718
1.76906  3.30088  2.23754  2.68970  3.01322  3.39227  3.46895  3.64008
2.67748  2.65443  2.23047  5.36438  4.14509    112 i - - C
          2.68618  4.42225  2.77519  2.73123  3.46354  2.40513  3.72494
3.29354  2.67741  2.69355  4.24690  2.90347  2.73739  3.18146  2.89801
2.37887  2.77519  2.98518  4.58477  3.61503
          0.24993  5.45072  1.52854  0.61958  0.77255  0.39584  1.11814
     79   2.72930  3.52515  2.92039  2.34206  4.07670  3.03351  3.59156
4.21244  2.33627  3.11529  4.02007  2.80147  2.43396  2.90808  3.20322
2.47227  2.35779  2.95463  5.88142  4.48884    113 k - - -
          2.68614  4.42293  2.77516  2.73092  3.46422  2.40537  3.72319
3.29304  2.67775  2.69391  4.24758  2.90373  2.73786  3.18136  2.89869
2.37793  2.77470  2.98559  4.58251  3.61571
          1.55076  1.10435  0.78419  1.32015  0.31074  0.45897  0.99949
     80   2.68322  5.31732  2.81308  2.47682  3.05683  3.40985  3.81334
4.12167  2.25299  3.61587  3.99489  2.84532  3.19017  2.59964  2.47557
1.89604  2.88760  3.69948  5.75953  3.79411    125 s - - T
          2.68618  4.42225  2.77519  2.73123  3.46354  2.40513  3.72494
3.29354  2.67741  2.69355  4.24690  2.90347  2.73739  3.18146  2.89801
2.37887  2.77519  2.98518  4.58477  3.61503
          0.14676  4.92328  2.04620  0.61958  0.77255  0.18883  1.75986
     81   2.61065  5.53227  2.62841  1.92242  3.66198  3.30664  3.82369
3.55898  2.37006  2.97142  4.24046  3.30609  3.05910  2.87635  2.72029
2.39075  2.60916  3.91561  3.46908  4.57052    126 e - - G
```

2.68618 4.42225 2.77519 2.73123 3.46354 2.40513 3.72494
3.29354 2.67741 2.69355 4.24690 2.90347 2.73739 3.18146 2.89801
2.37887 2.77519 2.98518 4.58477 3.61503
0.00683 5.38511 6.10745 0.61958 0.77255 0.59572 0.80110
82 2.30858 4.79976 2.58515 1.99706 4.92306 2.70153 4.03854
3.46403 2.23481 3.39712 3.27751 2.82965 4.03540 2.72192 3.11110
2.27506 2.83668 3.96988 6.01499 4.60567 127 e - - G
2.68618 4.42225 2.77519 2.73123 3.46354 2.40513 3.72494
3.29354 2.67741 2.69355 4.24690 2.90347 2.73739 3.18146 2.89801
2.37887 2.77519 2.98518 4.58477 3.61503
0.00654 5.42957 6.15192 0.61958 0.77255 0.66232 0.72495
83 2.90432 4.61125 2.39478 2.48768 4.90222 3.85950 4.04765
4.05392 2.48448 3.67890 3.24150 1.73684 3.33401 2.85497 3.09931
2.67788 2.37503 3.64900 6.00648 3.09299 128 n - - C
2.68618 4.42225 2.77519 2.73123 3.46354 2.40513 3.72494
3.29354 2.67741 2.69355 4.24690 2.90347 2.73739 3.18146 2.89801
2.37887 2.77519 2.98518 4.58477 3.61503
0.00648 5.43865 6.16099 0.61958 0.77255 0.37509 1.16228
84 2.25657 4.61133 3.73311 4.20604 3.71005 4.30224 3.66334
1.84959 4.05882 1.76486 2.53474 3.84888 3.99084 3.98082 3.90661
3.18613 3.19047 1.79123 5.22684 3.70395 129 l - - H
2.68618 4.42225 2.77519 2.73123 3.46354 2.40513 3.72494
3.29354 2.67741 2.69355 4.24690 2.90347 2.73739 3.18146 2.89801
2.37887 2.77519 2.98518 4.58477 3.61503
0.00615 5.48996 6.21230 0.61958 0.77255 0.48576 0.95510
85 2.30035 5.56988 2.40999 2.21279 4.47536 3.23448 3.74948
3.38320 2.48551 2.83023 3.54332 2.88293 2.85761 2.46818 3.22668
3.08231 3.25651 2.54492 6.01521 4.25325 130 e - - H
2.68618 4.42225 2.77519 2.73123 3.46354 2.40513 3.72494
3.29354 2.67741 2.69355 4.24690 2.90347 2.73739 3.18146 2.89801
2.37887 2.77519 2.98518 4.58477 3.61503
0.00615 5.48996 6.21230 0.61958 0.77255 0.48576 0.95510
86 2.27323 5.61703 2.22150 2.09541 4.96133 3.27127 2.96273
3.92697 2.41560 3.46346 4.12036 3.08915 3.34945 2.61234 3.12231
2.51176 2.91712 3.30380 6.05018 3.31522 131 e - - H
2.68618 4.42225 2.77519 2.73123 3.46354 2.40513 3.72494
3.29354 2.67741 2.69355 4.24690 2.90347 2.73739 3.18146 2.89801
2.37887 2.77519 2.98518 4.58477 3.61503
0.00615 5.48996 6.21230 0.61958 0.77255 0.48576 0.95510
87 2.99876 4.57556 4.29394 4.40022 3.05834 4.33271 4.65891
2.11304 4.21428 1.45760 3.54789 3.46852 4.69778 3.88336 3.83620
2.98182 2.89378 1.55061 5.19515 3.19529 132 l - - H
2.68618 4.42225 2.77519 2.73123 3.46354 2.40513 3.72494
3.29354 2.67741 2.69355 4.24690 2.90347 2.73739 3.18146 2.89801
2.37887 2.77519 2.98518 4.58477 3.61503
0.00615 5.48996 6.21230 0.61958 0.77255 0.48576 0.95510
88 3.54971 4.34957 5.50080 4.88903 2.83848 4.74213 5.05569
2.62875 4.68471 0.53833 3.06282 4.90123 5.05337 4.76544 4.06045
4.06195 3.77599 3.04502 5.45973 3.51705 133 L - - H
2.68618 4.42225 2.77519 2.73123 3.46354 2.40513 3.72494
3.29354 2.67741 2.69355 4.24690 2.90347 2.73739 3.18146 2.89801
2.37887 2.77519 2.98518 4.58477 3.61503
0.00615 5.48996 6.21230 0.61958 0.77255 0.48576 0.95510
89 1.97209 5.58771 3.06582 2.21718 3.86788 3.88817 3.45240
3.92376 2.58307 2.54046 4.63190 3.13768 3.83911 2.36340 2.71210
2.33000 2.89327 3.59258 6.02848 3.30747 134 a - - H

2.68618 4.42225 2.77519 2.73123 3.46354 2.40513 3.72494
3.29354 2.67741 2.69355 4.24690 2.90347 2.73739 3.18146 2.89801
2.37887 2.77519 2.98518 4.58477 3.61503
0.00615 5.48996 6.21230 0.61958 0.77255 0.48576 0.95510
90 1.57059 4.09895 3.77504 3.38265 3.91230 3.44243 3.60547
2.74967 2.88386 1.72417 3.32939 3.98283 4.55289 3.84400 3.87235
3.03331 2.39415 2.59695 5.38222 4.16409 135 a - - H
2.68618 4.42225 2.77519 2.73123 3.46354 2.40513 3.72494
3.29354 2.67741 2.69355 4.24690 2.90347 2.73739 3.18146 2.89801
2.37887 2.77519 2.98518 4.58477 3.61503
0.00615 5.48996 6.21230 0.61958 0.77255 0.48576 0.95510
91 0.83634 4.43511 3.56703 3.44170 4.17768 2.78706 4.42432
3.30291 3.40595 2.71085 4.12747 3.85323 3.68903 3.71676 3.78197
2.74522 3.41568 3.07497 5.59936 4.35437 136 a - - H
2.68533 4.42235 2.77530 2.73133 3.46364 2.40523 3.72504
3.29342 2.67751 2.69352 4.24624 2.90357 2.73750 3.18135 2.89811
2.37897 2.77530 2.98528 4.58487 3.61513
0.17043 1.86632 6.21230 0.10782 2.28068 0.48576 0.95510
92 3.10816 4.97789 1.78238 2.30127 4.96331 3.70950 2.93360
3.92844 2.35364 3.33627 4.33637 2.64709 4.27547 2.66340 2.49349
2.33708 3.08611 3.56082 6.05119 3.82264 138 d - - H
2.68618 4.42225 2.77519 2.73123 3.46354 2.40513 3.72494
3.29354 2.67741 2.69355 4.24690 2.90347 2.73739 3.18146 2.89801
2.37887 2.77519 2.98518 4.58477 3.61503
0.00615 5.48996 6.21230 0.61958 0.77255 0.48576 0.95510
93 3.15730 4.37908 3.79400 3.05683 2.64179 4.17652 3.70309
2.47170 2.15414 1.76840 3.03720 3.98259 4.55281 3.84372 2.63862
2.91339 3.38914 2.89970 5.38236 2.58474 139 l - - H
2.68618 4.42225 2.77519 2.73123 3.46354 2.40513 3.72494
3.29354 2.67741 2.69355 4.24690 2.90347 2.73739 3.18146 2.89801
2.37887 2.77519 2.98518 4.58477 3.61503
0.00615 5.48996 6.21230 0.61958 0.77255 0.48576 0.95510
94 4.71550 5.87294 6.16263 5.85933 1.64174 5.62085 4.73237
4.09223 5.64344 0.84431 3.91266 5.40758 5.86833 5.44150 5.50737
4.98998 4.92377 4.13989 4.02495 1.41243 140 l - - H
2.68618 4.42225 2.77519 2.73123 3.46354 2.40513 3.72494
3.29354 2.67741 2.69355 4.24690 2.90347 2.73739 3.18146 2.89801
2.37887 2.77519 2.98518 4.58477 3.61503
0.00615 5.48996 6.21230 0.61958 0.77255 0.48576 0.95510
95 2.70989 4.84493 2.33967 1.88903 4.96357 2.89545 3.26370
4.02308 2.63906 2.71576 4.65930 2.98056 4.27550 1.85978 2.61369
2.98400 3.26590 4.00919 6.05136 4.64047 141 q - - T
2.68618 4.42225 2.77519 2.73123 3.46354 2.40513 3.72494
3.29354 2.67741 2.69355 4.24690 2.90347 2.73739 3.18146 2.89801
2.37887 2.77519 2.98518 4.58477 3.61503
0.00615 5.48996 6.21230 0.61958 0.77255 0.48576 0.95510
96 3.01398 3.50344 5.12362 2.96895 3.04784 4.35026 4.68289
1.55869 3.74869 1.75223 2.27378 4.50506 4.71404 4.43588 4.31668
3.48010 3.11483 1.97031 5.18340 4.00688 142 i - - B
2.68618 4.42225 2.77519 2.73123 3.46354 2.40513 3.72494
3.29354 2.67741 2.69355 4.24690 2.90347 2.73739 3.18146 2.89801
2.37887 2.77519 2.98518 4.58477 3.61503
0.00615 5.48996 6.21230 0.61958 0.77255 0.48576 0.95510
97 2.70569 5.61456 2.23179 2.41479 4.95778 3.43852 3.58184
4.10621 2.21771 3.56445 4.15886 3.07402 1.97947 2.63688 2.82060
2.89007 2.92508 3.51910 4.72116 3.40272 143 p - - H

```
        2.68618  4.42225  2.77519  2.73123  3.46354  2.40513  3.72494
3.29354  2.67741  2.69355  4.24690  2.90347  2.73739  3.18146  2.89801
2.37887  2.77519  2.98518  4.58477  3.61503
        0.00615  5.48996  6.21230  0.61958  0.77255  0.48576  0.95510
     98  2.44346  3.45528  2.67921  2.49933  3.81120  2.75755  2.82140
4.40639  2.70480  3.57880  3.80024  2.71933  3.51208  3.06822  2.11568
2.36215  3.11957  3.59319  6.03295  3.67920    144 r - - H
        2.68618  4.42225  2.77519  2.73123  3.46354  2.40513  3.72494
3.29354  2.67741  2.69355  4.24690  2.90347  2.73739  3.18146  2.89801
2.37887  2.77519  2.98518  4.58477  3.61503
        0.02934  5.48996  3.69743  0.61958  0.77255  0.48576  0.95510
     99  2.64836  4.56144  4.22072  4.45625  3.36890  4.33931  4.67020
2.29392  4.25813  1.09798  3.39959  4.47692  3.74037  4.40365  4.29323
3.11669  3.40848  1.65267  5.18593  4.00826    145 l - - H
        2.68618  4.42225  2.77519  2.73123  3.46354  2.40513  3.72494
3.29354  2.67741  2.69355  4.24690  2.90347  2.73739  3.18146  2.89801
2.37887  2.77519  2.98518  4.58477  3.61503
        0.00630  5.46691  6.18926  0.61958  0.77255  0.58548  0.81382
    100  2.53280  4.99940  3.64783  2.73649  3.56541  4.04856  3.87027
2.12361  2.04419  2.58818  3.36683  3.69649  4.43212  2.88661  3.00563
2.87098  2.66462  2.25734  4.49612  4.29563    146 k - - H
        2.68618  4.42225  2.77519  2.73123  3.46354  2.40513  3.72494
3.29354  2.67741  2.69355  4.24690  2.90347  2.73739  3.18146  2.89801
2.37887  2.77519  2.98518  4.58477  3.61503
        0.00630  5.46691  6.18926  0.61958  0.77255  0.42810  1.05482
    101  2.37779  4.56384  2.26180  2.20707  4.94957  3.88390  3.45759
3.22618  2.19403  3.57601  3.86624  2.84249  4.03413  2.56144  2.54834
2.92906  2.43655  3.44648  4.65400  4.63581    147 k - - H
        2.68618  4.42225  2.77519  2.73123  3.46354  2.40513  3.72494
3.29354  2.67741  2.69355  4.24690  2.90347  2.73739  3.18146  2.89801
2.37887  2.77519  2.98518  4.58477  3.61503
        0.00615  5.48996  6.21230  0.61958  0.77255  0.48576  0.95510
    102  2.12161  5.21600  3.60282  2.64818  3.73789  3.98498  2.93599
2.61886  2.17696  2.22150  3.46197  3.28926  4.37298  2.54486  3.07402
2.85137  3.25571  2.67240  5.73999  4.43019    148 a - - H
        2.68618  4.42225  2.77519  2.73123  3.46354  2.40513  3.72494
3.29354  2.67741  2.69355  4.24690  2.90347  2.73739  3.18146  2.89801
2.37887  2.77519  2.98518  4.58477  3.61503
        0.00615  5.48996  6.21230  0.61958  0.77255  0.48576  0.95510
    103  2.46436  1.10495  4.30250  4.18373  3.71541  3.77938  4.61090
2.79612  4.04057  2.20368  3.41016  3.85516  4.66611  3.97248  3.93341
3.25495  2.99316  2.57410  5.23107  3.31102    149 c - - H
        2.68618  4.42225  2.77519  2.73123  3.46354  2.40513  3.72494
3.29354  2.67741  2.69355  4.24690  2.90347  2.73739  3.18146  2.89801
2.37887  2.77519  2.98518  4.58477  3.61503
        0.00615  5.48996  6.21230  0.61958  0.77255  0.48576  0.95510
    104  2.70063  4.13159  3.30387  1.51169  4.17774  3.36137  3.48642
3.72463  2.64146  3.37561  3.26673  3.22452  3.34437  2.85589  2.92737
2.35528  2.59993  3.13722  5.99364  4.60309    150 e - - H
        2.68618  4.42225  2.77519  2.73123  3.46354  2.40513  3.72494
3.29354  2.67741  2.69355  4.24690  2.90347  2.73739  3.18146  2.89801
2.37887  2.77519  2.98518  4.58477  3.61503
        0.00615  5.48996  6.21230  0.61958  0.77255  0.48576  0.95510
    105  2.64636  5.60783  2.60212  1.82621  3.58826  3.56847  3.71235
3.65958  2.21295  3.58091  3.95526  2.92234  4.01102  2.64738  2.79981
2.65649  2.67613  2.96031  6.04339  3.47015    151 e - - H
```

```
          2.68618  4.42225  2.77519  2.73123  3.46354  2.40513  3.72494
3.29354  2.67741  2.69355  4.24690  2.90347  2.73739  3.18146  2.89801
2.37887  2.77519  2.98518  4.58477  3.61503
          0.00615  5.48996  6.21230  0.61958  0.77255  0.48576  0.95510
    106   2.57390  4.89183  3.48673  2.88288  1.97776  3.89920  3.38663
2.27822  2.76463  2.28212  3.27764  3.38593  4.13516  3.53901  3.74563
2.69490  3.26294  2.93673  5.47066  2.71221    152 f - - H
          2.68618  4.42225  2.77519  2.73123  3.46354  2.40513  3.72494
3.29354  2.67741  2.69355  4.24690  2.90347  2.73739  3.18146  2.89801
2.37887  2.77519  2.98518  4.58477  3.61503
          0.01681  5.48996  4.37861  0.61958  0.77255  0.48576  0.95510
    107   2.90535  3.47011  4.46280  3.86321  3.11923  4.04188  4.64146
2.08382  3.88982  1.04979  3.11847  3.92610  4.28320  3.81569  3.38469
3.45328  3.27828  2.42184  5.19650  4.01568    153 l - - H
          2.68618  4.42225  2.77519  2.73123  3.46354  2.40513  3.72494
3.29354  2.67741  2.69355  4.24690  2.90347  2.73739  3.18146  2.89801
2.37887  2.77519  2.98518  4.58477  3.61503
          0.01236  5.47936  4.81392  0.61958  0.77255  0.49058  0.94744
    108   2.92300  2.72109  2.73197  2.62643  4.75508  3.90739  3.88945
2.59461  2.24322  2.81269  4.52409  3.20740  3.51799  2.34686  3.00901
2.61529  2.70461  2.77066  5.93666  3.82923    154 k - - H
          2.68618  4.42225  2.77519  2.73123  3.46354  2.40513  3.72494
3.29354  2.67741  2.69355  4.24690  2.90347  2.73739  3.18146  2.89801
2.37887  2.77519  2.98518  4.58477  3.61503
          0.00622  5.47914  6.20149  0.61958  0.77255  0.45669  1.00341
    109   2.59448  5.61969  2.94133  2.12710  4.49436  2.86747  3.28821
4.45141  2.23235  3.61620  4.66024  2.74437  3.99545  2.09067  2.72900
2.60047  2.57472  3.43049  5.14057  3.54488    155 q - - H
          2.68618  4.42225  2.77519  2.73123  3.46354  2.40513  3.72494
3.29354  2.67741  2.69355  4.24690  2.90347  2.73739  3.18146  2.89801
2.37887  2.77519  2.98518  4.58477  3.61503
          0.01414  5.48996  4.61430  0.61958  0.77255  0.48576  0.95510
    110   3.02028  4.57859  2.86880  2.76453  3.79998  3.45645  3.19192
3.27295  2.13791  3.45239  4.13984  2.36555  3.87572  2.41591  2.90020
1.83908  2.73150  3.78805  6.03053  4.21925    156 s - - C
          2.68618  4.42225  2.77519  2.73123  3.46354  2.40513  3.72494
3.29354  2.67741  2.69355  4.24690  2.90347  2.73739  3.18146  2.89801
2.37887  2.77519  2.98518  4.58477  3.61503
          0.03900  5.48202  3.37878  0.61958  0.77255  0.52150  0.90049
    111   2.80040  5.50088  3.27780  2.49611  4.38205  2.91288  3.70579
3.16495  2.25976  1.85691  4.55215  3.08384  3.46057  2.89755  3.07397
2.10454  2.76764  3.69781  5.95800  3.92921    157 l - - X
          2.68618  4.42225  2.77519  2.73123  3.46354  2.40513  3.72494
3.29354  2.67741  2.69355  4.24690  2.90347  2.73739  3.18146  2.89801
2.37887  2.77519  2.98518  4.58477  3.61503
          0.00432  5.44734        *  0.61958  0.77255  0.00000        *
//
HMMER3/f [3.3 | Nov 2019]
NAME  C1_2
ACC   PF03107.17
DESC  C1 domain
LENG  47
ALPH  amino
RF    no
MM    no
CONS  yes
CS    no
MAP   yes
```

```
DATE  Mon Apr 20 11:59:05 2020
NSEQ  218
EFFN  7.713959
CKSUM 1375319583
GA    26.00 26.00
TC    26.00 26.00
NC    25.90 25.90
STATS LOCAL MSV       -8.5457  0.71926
STATS LOCAL VITERBI   -9.3201  0.71926
STATS LOCAL FORWARD   -3.8560  0.71926
HMM          A        C        D        E        F        G        H
I        K        L        M        N        P        Q        R        S
T        V        W        Y
            m->m     m->i     m->d     i->m     i->i     d->m     d->d
  COMPO   2.85033  2.30679  2.88107  2.76889  3.17226  3.10123  2.85345
3.20264  2.75026  2.57126  4.06103  3.14625  3.31373  3.29579  3.04661
2.60305  3.01307  3.09715  4.87185  3.19351
          2.68618  4.42225  2.77519  2.73123  3.46354  2.40513  3.72494
3.29354  2.67741  2.69355  4.24690  2.90347  2.73739  3.18146  2.89801
2.37887  2.77519  2.98518  4.58477  3.61503
          0.05788  5.72855  2.93772  0.61958  0.77255  0.00000        *
      1   2.87923  5.70025  3.36219  2.35950  2.53511  3.63893  3.42028
4.28491  2.23582  3.59785  4.74349  2.83986  1.61886  2.98882  3.20489
2.54559  3.32492  3.91683  6.13884  3.42927      1 p - - -
          2.68618  4.42225  2.77519  2.73123  3.46354  2.40513  3.72494
3.29354  2.67741  2.69355  4.24690  2.90347  2.73739  3.18146  2.89801
2.37887  2.77519  2.98518  4.58477  3.61503
          0.00511  5.67578  6.39812  0.61958  0.77255  0.46523  0.98884
      2   3.10901  4.06458  3.76863  3.52662  3.30335  3.27445  4.15346
2.96637  2.78654  1.90858  4.07790  3.91806  4.66272  3.90254  2.41891
1.75392  2.79443  2.93989  3.90270  2.64226      2 s - - -
          2.68618  4.42225  2.77519  2.73123  3.46354  2.40513  3.72494
3.29354  2.67741  2.69355  4.24690  2.90347  2.73739  3.18146  2.89801
2.37887  2.77519  2.98518  4.58477  3.61503
          0.00493  5.71147  6.43381  0.61958  0.77255  0.51548  0.90936
      3   4.10462  4.53726  5.12778  4.73691  3.74074  4.88114  0.29055
4.51657  4.38108  4.08483  5.11575  4.13372  5.40505  4.81267  4.56651
4.28810  4.43409  3.59290  5.36062  3.32945      3 H - - -
          2.68618  4.42225  2.77519  2.73123  3.46354  2.40513  3.72494
3.29354  2.67741  2.69355  4.24690  2.90347  2.73739  3.18146  2.89801
2.37887  2.77519  2.98518  4.58477  3.61503
          0.00489  5.71860  6.44094  0.61958  0.77255  0.49892  0.93441
      4   2.90872  5.75158  2.15310  2.04780  4.68637  3.85895  3.22386
4.44908  2.01211  3.56687  3.99054  3.11812  1.73958  3.03286  3.02942
2.90075  3.40853  4.03216  5.45553  4.77279      4 p - - -
          2.68635  4.42175  2.77490  2.73087  3.46349  2.40532  3.72512
3.29386  2.67690  2.69364  4.24722  2.90342  2.73772  3.17995  2.89802
2.37915  2.77546  2.98551  4.58509  3.61535
          0.46858  1.00480  4.82911  0.08810  2.47297  0.46726  0.98541
      5   4.18365  4.64351  4.20894  4.15826  4.04541  4.83560  0.31061
4.67968  3.43646  4.21347  5.20461  4.51209  5.30717  4.38808  4.03860
4.27096  4.43919  4.43566  5.60639  3.29211      7 H - - -
          2.68618  4.42225  2.77519  2.73123  3.46354  2.40513  3.72494
3.29354  2.67741  2.69355  4.24690  2.90347  2.73739  3.18146  2.89801
2.37887  2.77519  2.98518  4.58477  3.61503
          0.00487  5.72216  6.44451  0.61958  0.77255  0.52232  0.89929
```

```
      6   3.19777  5.25379  3.33284  2.88299  4.54502  3.68446  3.31555
4.12140  2.53711  3.10849  3.56750  2.78728  1.53840  3.05098  2.33200
2.61499  2.64857  3.53113  6.13395  3.65285      8 p - - -
          2.68621  4.42228  2.77522  2.73126  3.46357  2.40515  3.72480
3.29357  2.67743  2.69338  4.24692  2.90349  2.73742  3.18149  2.89803
2.37882  2.77522  2.98521  4.58480  3.61490
          0.03877  3.31201  6.44451  0.26595  1.45448  0.46356  0.99166
      7   4.96880  6.12658  6.95728  6.36240  3.79598  6.40932  6.63784
2.58538  6.23681  0.25679  3.46268  6.60118  6.29860  5.94216  6.09236
5.82968  5.15210  2.98809  6.46213  4.43807     10 L - - -
          2.68618  4.42225  2.77519  2.73123  3.46354  2.40513  3.72494
3.29354  2.67741  2.69355  4.24690  2.90347  2.73739  3.18146  2.89801
2.37887  2.77519  2.98518  4.58477  3.61503
          0.00484  5.72855  6.45090  0.61958  0.77255  0.48576  0.95510
      8   2.89061  5.48918  3.63991  2.87567  3.28748  4.08252  4.11916
2.30363  2.21894  3.50059  4.31932  2.97209  4.47273  2.83850  2.55625
2.19934  1.90398  3.04933  5.98567  3.62546     11 t - - -
          2.68618  4.42225  2.77519  2.73123  3.46354  2.40513  3.72494
3.29354  2.67741  2.69355  4.24690  2.90347  2.73739  3.18146  2.89801
2.37887  2.77519  2.98518  4.58477  3.61503
          0.00484  5.72855  6.45090  0.61958  0.77255  0.48576  0.95510
      9   3.33502  4.71937  5.23662  4.62549  2.08773  4.49744  3.95059
2.94288  3.96193  0.76159  3.68299  4.63916  4.51365  4.12379  3.83125
3.80606  3.44904  2.79563  4.74303  3.22136     12 l - - -
          2.68618  4.42225  2.77519  2.73123  3.46354  2.40513  3.72494
3.29354  2.67741  2.69355  4.24690  2.90347  2.73739  3.18146  2.89801
2.37887  2.77519  2.98518  4.58477  3.61503
          0.00484  5.72855  6.45090  0.61958  0.77255  0.48576  0.95510
     10   3.15088  3.61739  2.73362  2.52805  3.08076  3.86675  2.58166
3.32577  2.58501  2.54251  3.74315  3.17526  4.22565  2.94566  3.16666
2.27031  2.43817  2.69088  6.02379  4.05157     13 s - - -
          2.68851  4.42407  2.77362  2.73275  3.46599  2.40547  3.72071
3.28754  2.67847  2.69439  4.23971  2.90535  2.73668  3.18096  2.89880
2.37838  2.77621  2.98392  4.58699  3.61323
          1.64761  0.84892  0.96861  1.99712  0.14587  0.48576  0.95510
     11   2.91506  5.31169  2.87984  2.51292  4.09735  2.69058  3.80874
2.37813  2.33135  3.16351  4.03952  2.87143  3.60545  3.01900  2.88165
2.59509  2.81623  3.22453  4.96921  2.55868     36 k - - -
          2.68618  4.42225  2.77519  2.73123  3.46354  2.40513  3.72494
3.29354  2.67741  2.69355  4.24690  2.90347  2.73739  3.18146  2.89801
2.37887  2.77519  2.98518  4.58477  3.61503
          0.01384  5.25368  4.76593  0.61958  0.77255  0.94526  0.49197
     12   2.94290  5.08973  2.65969  2.34930  4.12735  2.14625  3.63260
4.04842  2.56679  3.31925  4.60473  2.80487  2.44434  3.00606  2.88919
2.15777  2.93358  3.53459  5.99875  3.77384     37 g - - -
          2.68618  4.42225  2.77519  2.73123  3.46354  2.40513  3.72494
3.29354  2.67741  2.69355  4.24690  2.90347  2.73739  3.18146  2.89801
2.37887  2.77519  2.98518  4.58477  3.61503
          0.00667  5.40883  6.13118  0.61958  0.77255  1.07042  0.41986
     13   2.68297  5.57368  2.87118  1.95360  4.28415  3.51795  3.56758
3.29367  2.38769  2.71217  4.23596  2.96361  3.50977  3.12408  2.22493
2.32899  2.93265  3.10482  5.13044  4.34453     38 e - - -
          2.68618  4.42225  2.77519  2.73123  3.46354  2.40513  3.72494
3.29354  2.67741  2.69355  4.24690  2.90347  2.73739  3.18146  2.89801
2.37887  2.77519  2.98518  4.58477  3.61503
          0.01754  5.47180  4.32900  0.61958  0.77255  0.31934  1.29694
```

```
     14   2.80566  5.64644  2.24532  2.62642  3.37306  3.33880  3.78842
  3.71301  2.63713  2.59939  3.99561  2.59067  2.36829  3.01945  3.32518
  2.65740  2.51835  3.08190  6.09879  3.71129     39 d - - -
          2.68618  4.42225  2.77519  2.73123  3.46354  2.40513  3.72494
  3.29354  2.67741  2.69355  4.24690  2.90347  2.73739  3.18146  2.89801
  2.37887  2.77519  2.98518  4.58477  3.61503
          0.00511  5.67573  6.39808  0.61958  0.77255  0.44088  1.03133
     15   2.60272  4.57642  2.57042  2.29506  4.48341  2.95863  3.44836
  3.80219  2.44516  3.42979  4.44280  3.18368  3.48356  3.19862  2.42298
  2.00642  2.92491  2.81666  5.15933  3.35502     40 s - - -
          2.68618  4.42225  2.77519  2.73123  3.46354  2.40513  3.72494
  3.29354  2.67741  2.69355  4.24690  2.90347  2.73739  3.18146  2.89801
  2.37887  2.77519  2.98518  4.58477  3.61503
          0.00491  5.71495  6.43730  0.61958  0.77255  0.52337  0.89776
     16   2.86719  4.68266  2.37563  2.40221  4.27595  3.35843  3.10983
  3.73394  2.10640  3.15207  4.78630  2.82971  3.33427  2.81921  2.97625
  2.01641  3.01035  3.97450  5.41341  3.00626     41 s - - -
          2.68618  4.42225  2.77519  2.73123  3.46354  2.40513  3.72494
  3.29354  2.67741  2.69355  4.24690  2.90347  2.73739  3.18146  2.89801
  2.37887  2.77519  2.98518  4.58477  3.61503
          0.00968  5.71912  5.05958  0.61958  0.77255  0.53918  0.87521
     17   2.41533  5.70616  2.55825  2.09704  4.72090  2.10259  3.93827
  2.44812  2.72132  3.61772  4.12168  3.04595  4.08678  3.17982  3.03847
  2.27755  2.96543  3.48456  4.70016  4.30041     42 e - - -
          2.68618  4.42225  2.77519  2.73123  3.46354  2.40513  3.72494
  3.29354  2.67741  2.69355  4.24690  2.90347  2.73739  3.18146  2.89801
  2.37887  2.77519  2.98518  4.58477  3.61503
          0.01167  5.71435  4.79162  0.61958  0.77255  0.52118  0.90096
     18   2.89449  4.96640  2.39543  2.30384  4.34642  3.24350  3.98814
  3.11311  2.38055  2.61814  3.23281  2.66261  3.70992  3.12028  2.81547
  2.24787  2.65182  3.50882  5.02821  4.13899     43 s - - -
          2.68621  4.42234  2.77512  2.73126  3.46363  2.40508  3.72504
  3.29340  2.67750  2.69351  4.24699  2.90356  2.73739  3.18155  2.89787
  2.37890  2.77522  2.98505  4.58441  3.61512
          0.07005  2.71736  6.43477  0.57506  0.82707  0.51428  0.91112
     19   3.16430  4.68612  3.22212  3.18537  1.88271  2.63353  3.70491
  3.42536  2.22077  2.66013  3.45788  3.55083  4.37845  3.29843  2.76985
  2.75115  2.87161  3.05847  3.92394  3.03332     46 f - - -
          2.68618  4.42225  2.77519  2.73123  3.46354  2.40513  3.72494
  3.29354  2.67741  2.69355  4.24690  2.90347  2.73739  3.18146  2.89801
  2.37887  2.77519  2.98518  4.58477  3.61503
          0.00489  5.71912  6.44146  0.61958  0.77255  0.45375  1.00852
     20   3.06208  4.70415  3.25950  2.46624  3.35476  4.03591  3.60925
  3.67492  2.34439  2.79656  4.71794  2.13963  2.98290  3.02141  2.62543
  2.52936  2.61498  3.06361  3.07190  4.22983     47 n - - -
          2.68618  4.42225  2.77519  2.73123  3.46354  2.40513  3.72494
  3.29354  2.67741  2.69355  4.24690  2.90347  2.73739  3.18146  2.89801
  2.37887  2.77519  2.98518  4.58477  3.61503
          0.00484  5.72855  6.45090  0.61958  0.77255  0.48576  0.95510
     21   5.79102  0.02375  6.89345  6.96636  7.19240  5.75517  7.43306
  7.19073  7.03690  6.63065  7.84655  6.93829  6.46707  7.30890  6.83866
  6.06698  6.32816  6.69384  7.83763  7.41600     48 C - - -
          2.68618  4.42225  2.77519  2.73123  3.46354  2.40513  3.72494
  3.29354  2.67741  2.69355  4.24690  2.90347  2.73739  3.18146  2.89801
  2.37887  2.77519  2.98518  4.58477  3.61503
          0.00484  5.72855  6.45090  0.61958  0.77255  0.48576  0.95510
```

```
     22   2.99781  4.96687  2.19444  2.34207  4.23948  2.86376  3.60200
3.42806  2.67687  3.60217  4.79332  2.06834  4.41058  2.65679  2.80110
1.91183  3.22630  3.95103  6.18553  3.82550     49 s - - -
          2.68618  4.42225  2.77519  2.73123  3.46354  2.40513  3.72494
3.29354  2.67741  2.69355  4.24690  2.90347  2.73739  3.18146  2.89801
2.37887  2.77519  2.98518  4.58477  3.61503
          0.00484  5.72855  6.45090  0.61958  0.77255  0.48576  0.95510
     23   1.49670  3.26416  5.27478  4.66108  3.41361  2.62544  4.83385
2.31695  4.45119  2.27010  3.83201  4.65616  4.86543  4.58687  3.89602
3.45567  2.78687  1.84542  2.80773  4.15811     50 a - - -
          2.68619  4.42226  2.77521  2.73124  3.46355  2.40514  3.72495
3.29355  2.67725  2.69356  4.24691  2.90348  2.73741  3.18147  2.89802
2.37888  2.77521  2.98519  4.58478  3.61504
          0.01834  4.09888  6.45090  0.40182  1.10595  0.48576  0.95510
     24   5.79102  0.02375  6.89345  6.96636  7.19240  5.75517  7.43306
7.19073  7.03690  6.63065  7.84655  6.93829  6.46707  7.30890  6.83866
6.06698  6.32816  6.69384  7.83763  7.41600     52 C - - -
          2.68618  4.42225  2.77519  2.73123  3.46354  2.40513  3.72494
3.29354  2.67741  2.69355  4.24690  2.90347  2.73739  3.18146  2.89801
2.37887  2.77519  2.98518  4.58477  3.61503
          0.01244  5.72855  4.69785  0.61958  0.77255  0.48576  0.95510
     25   2.54921  5.74505  2.44990  2.17105  5.08826  1.69676  3.98454
3.37960  2.32260  4.04954  4.25295  3.36233  3.36332  2.97997  2.46861
3.09485  3.23046  3.12287  5.42471  4.76902     53 g - - -
          2.68618  4.42225  2.77519  2.73123  3.46354  2.40513  3.72494
3.29354  2.67741  2.69355  4.24690  2.90347  2.73739  3.18146  2.89801
2.37887  2.77519  2.98518  4.58477  3.61503
          0.00488  5.72099  6.44333  0.61958  0.77255  0.52885  0.88985
     26   3.05180  4.52517  3.19643  2.12668  4.62204  3.92222  3.55326
4.07402  2.11394  2.45013  4.09619  2.60024  4.41214  2.45593  2.07200
3.08419  2.67536  3.38439  3.94940  4.08121     54 r - - -
          2.68618  4.42225  2.77519  2.73123  3.46354  2.40513  3.72494
3.29354  2.67741  2.69355  4.24690  2.90347  2.73739  3.18146  2.89801
2.37887  2.77519  2.98518  4.58477  3.61503
          0.00992  5.72099  5.02101  0.61958  0.77255  0.52885  0.88985
     27   2.90921  5.73943  2.29697  2.28522  5.08147  3.58437  3.89967
3.41672  1.90719  2.90709  4.14506  3.01355  2.41172  3.07751  3.08038
2.60063  2.57568  3.64964  5.40917  3.80122     55 k - - -
          2.68639  4.42005  2.77527  2.73161  3.46373  2.40503  3.72507
3.29334  2.67785  2.69252  4.24741  2.90392  2.73774  3.18184  2.89674
2.37913  2.77558  2.98486  4.58528  3.61465
          0.46764  1.31141  2.26259  0.81765  0.58245  0.44406  1.02562
     28   2.81688  4.89743  3.63182  3.52178  3.93217  2.34323  4.50220
1.87789  3.12582  2.45175  3.25293  4.01921  3.90616  3.68130  3.80031
1.97564  2.52018  2.29174  5.48686  3.99422     60 i - - -
          2.68618  4.42225  2.77519  2.73123  3.46354  2.40513  3.72494
3.29354  2.67741  2.69355  4.24690  2.90347  2.73739  3.18146  2.89801
2.37887  2.77519  2.98518  4.58477  3.61503
          0.19629  5.62186  1.74523  0.61958  0.77255  0.49826  0.93544
     29   2.74776  3.80149  2.10456  2.59980  2.40364  3.04688  3.45838
4.14259  2.41241  3.82459  4.49152  2.34317  3.80470  2.85854  3.06521
2.51357  2.71757  3.88560  6.05153  4.05548     61 d - - -
          2.68618  4.42225  2.77519  2.73123  3.46354  2.40513  3.72494
3.29354  2.67741  2.69355  4.24690  2.90347  2.73739  3.18146  2.89801
2.37887  2.77519  2.98518  4.58477  3.61503
          0.01795  5.50993  4.28721  0.61958  0.77255  0.37053  1.17237
```

```
     30   2.86352  4.51938  2.38098  2.35538  4.12578  1.56238  3.84277
4.23879  2.76992  3.86503  4.42987  2.88894  2.95910  2.99802  3.08397
2.63543  3.11696  3.85463  4.63862  3.28260     62 g - - -
          2.68618  4.42225  2.77519  2.73123  3.46354  2.40513  3.72494
3.29354  2.67741  2.69355  4.24690  2.90347  2.73739  3.18146  2.89801
2.37887  2.77519  2.98518  4.58477  3.61503
          0.01520  5.66943  4.45340  0.61958  0.77255  0.77501  0.61748
     31   3.17514  5.23728  2.84788  2.67420  1.46129  3.66570  4.00916
3.28503  2.82006  2.65322  3.59779  3.07283  4.20972  3.53207  3.36418
2.48445  2.80833  3.13530  4.92777  3.32243     63 f - - -
          2.68618  4.42225  2.77519  2.73123  3.46354  2.40513  3.72494
3.29354  2.67741  2.69355  4.24690  2.90347  2.73739  3.18146  2.89801
2.37887  2.77519  2.98518  4.58477  3.61503
          0.02969  5.65942  3.65851  0.61958  0.77255  0.81528  0.58432
     32   2.93634  4.08234  2.88422  2.54216  2.17985  2.82507  3.75762
3.37106  2.75687  2.82849  3.12683  3.00810  4.12672  3.09741  2.47485
2.87863  3.21746  3.40133  5.42423  2.65776     64 f - - -
          2.68622  4.42176  2.77579  2.73185  3.46011  2.40494  3.72520
3.29366  2.67798  2.69376  4.24760  2.90338  2.73704  3.18188  2.89838
2.37873  2.77544  2.98527  4.57985  3.61530
          0.61122  0.78619  6.35739  0.68779  0.69854  0.29152  1.37486
     33   5.94871  6.73629  6.63388  6.67767  2.36407  6.28817  4.79874
5.37663  6.43156  3.17151  5.85924  5.76114  6.51570  5.96173  6.14729
5.71364  6.12427  5.38936  4.85980  0.20909     67 Y - - -
          2.68618  4.42225  2.77519  2.73123  3.46354  2.40513  3.72494
3.29354  2.67741  2.69355  4.24690  2.90347  2.73739  3.18146  2.89801
2.37887  2.77519  2.98518  4.58477  3.61503
          0.00484  5.72855  6.45090  0.61958  0.77255  0.48576  0.95510
     34   2.99625  4.33149  3.55011  2.46051  3.66173  3.45647  2.81221
2.59649  2.20847  3.78623  3.94985  3.02820  4.46443  3.41036  2.32964
2.15647  2.65718  3.00830  5.20814  3.03403     68 s - - -
          2.68618  4.42225  2.77519  2.73123  3.46354  2.40513  3.72494
3.29354  2.67741  2.69355  4.24690  2.90347  2.73739  3.18146  2.89801
2.37887  2.77519  2.98518  4.58477  3.61503
          0.00484  5.72855  6.45090  0.61958  0.77255  0.48576  0.95510
     35   5.79102  0.02375  6.89345  6.96636  7.19240  5.75517  7.43306
7.19073  7.03690  6.63065  7.84655  6.93829  6.46707  7.30890  6.83866
6.06698  6.32816  6.69384  7.83763  7.41600     69 C - - -
          2.68620  4.42227  2.77506  2.73125  3.46356  2.40509  3.72496
3.29356  2.67743  2.69357  4.24691  2.90340  2.73741  3.18148  2.89802
2.37889  2.77521  2.98520  4.58479  3.61505
          0.17001  3.66307  2.03495  0.32498  1.28209  0.48576  0.95510
     36   3.13671  5.30236  2.35777  2.46041  4.17730  3.67369  3.85208
3.37840  2.37487  2.99169  4.39256  2.96843  2.91732  2.93953  2.98548
1.58183  2.88625  3.37435  6.09237  4.33115     71 s - - -
          2.68618  4.42225  2.77519  2.73123  3.46354  2.40513  3.72494
3.29354  2.67741  2.69355  4.24690  2.90347  2.73739  3.18146  2.89801
2.37887  2.77519  2.98518  4.58477  3.61503
          0.00556  5.59031  6.31266  0.61958  0.77255  0.24760  1.51720
     37   2.87544  5.69749  3.09957  1.74274  4.20528  3.68121  3.94126
2.35684  2.23250  3.36978  3.27442  3.37677  3.32191  2.59375  2.86930
2.95701  2.88051  2.98625  6.14482  3.73066     72 e - - -
          2.68655  4.42267  2.77493  2.73049  3.46396  2.40484  3.72483
3.29396  2.67689  2.69393  4.24732  2.90324  2.73773  3.18066  2.89826
2.37908  2.77521  2.98546  4.58519  3.61533
          0.37900  1.15876  6.45090  0.46807  0.98406  0.48576  0.95510
```

```
     38   3.72729  0.27417  3.29346  4.11296  5.49410  3.85959  5.46708
4.99972  4.60427  4.67910  5.55406  4.44885  5.08522  4.13329  4.95928
3.59445  4.19392  4.53888  6.89865  4.85954     76 C - - -
          2.68627  4.42234  2.77486  2.73105  3.46357  2.40519  3.72503
3.29363  2.67743  2.69363  4.24698  2.90355  2.73722  3.18155  2.89809
2.37875  2.77528  2.98527  4.58486  3.61512
          0.10085  2.56034  3.98158  0.39225  1.12558  0.48576  0.95510
     39   3.18308  3.93850  1.40399  2.42685  4.87644  3.11338  4.01894
4.45532  2.49539  4.04495  4.57225  1.98643  4.24735  3.20635  2.58071
2.72643  3.46420  3.66772  4.97316  4.11995     79 d - - -
          2.68633  4.42241  2.77485  2.73057  3.46353  2.40528  3.72510
3.29329  2.67756  2.69346  4.24705  2.90362  2.73755  3.18162  2.89808
2.37902  2.77511  2.98534  4.58492  3.61458
          0.08297  2.55087  6.43367  0.86180  0.54888  0.43057  1.05021
     40   3.04830  4.75401  5.32332  4.71393  0.87037  4.54584  4.61975
2.78465  4.50198  2.45291  3.86665  4.69786  4.90784  4.63136  4.51350
3.75812  3.43407  2.41621  5.32510  2.08658     84 f - - -
          2.68618  4.42225  2.77519  2.73123  3.46354  2.40513  3.72494
3.29354  2.67741  2.69355  4.24690  2.90347  2.73739  3.18146  2.89801
2.37887  2.77519  2.98518  4.58477  3.61503
          0.00484  5.72855  6.45090  0.61958  0.77255  0.48576  0.95510
     41   2.41337  4.33664  3.00517  3.31268  3.14513  3.38911  4.43050
2.46405  2.68928  2.82908  3.18660  3.01142  4.58302  2.61593  3.75980
2.19324  2.50881  2.29481  5.72699  4.10076     85 s - - -
          2.68618  4.42225  2.77519  2.73123  3.46354  2.40513  3.72494
3.29354  2.67741  2.69355  4.24690  2.90347  2.73739  3.18146  2.89801
2.37887  2.77519  2.98518  4.58477  3.61503
          0.00484  5.72855  6.45090  0.61958  0.77255  0.48576  0.95510
     42   2.90245  4.42147  5.31543  4.69923  2.91657  4.50964  4.84394
2.05073  4.48122  0.96082  3.10922  4.67634  4.87291  4.60973  4.48471
3.62727  3.09749  2.00467  4.53099  3.75154     86 l - - -
          2.68619  4.42226  2.77521  2.73124  3.46336  2.40506  3.72496
3.29355  2.67742  2.69356  4.24691  2.90348  2.73741  3.18148  2.89802
2.37888  2.77521  2.98520  4.58478  3.61504
          0.02102  3.95150  6.45090  0.37593  1.16043  0.48576  0.95510
     43   3.97894  3.13464  1.33010  3.33944  5.84301  3.99878  0.76525
5.38543  3.97121  4.90574  5.74479  3.33702  4.98550  4.13420  4.54125
3.77265  4.30349  4.93182  7.06208  5.58833     88 h - - -
          2.68620  4.42227  2.77521  2.73125  3.46356  2.40515  3.72496
3.29356  2.67743  2.69344  4.24692  2.90348  2.73741  3.18148  2.89803
2.37889  2.77521  2.98498  4.58479  3.61505
          0.02829  3.63757  6.45090  0.32050  1.29384  0.48576  0.95510
     44   2.57258  5.08183  3.57994  2.15371  3.22435  3.82637  3.60132
2.67449  2.56003  2.53517  3.58628  3.61347  3.77097  2.86113  3.39815
2.23434  3.02312  2.11875  5.88336  4.40915     90 v - - -
          2.68618  4.42225  2.77519  2.73123  3.46354  2.40513  3.72494
3.29354  2.67741  2.69355  4.24690  2.90347  2.73739  3.18146  2.89801
2.37887  2.77519  2.98518  4.58477  3.61503
          0.00484  5.72855  6.45090  0.61958  0.77255  0.48576  0.95510
     45   2.72429  5.06746  2.59617  2.35068  4.35056  3.63573  3.95048
4.20848  2.40243  3.80688  4.79436  2.83924  4.15734  2.58987  1.91235
1.99489  2.78113  2.95763  6.18639  4.19985     91 r - - -
          2.68618  4.42225  2.77519  2.73123  3.46354  2.40513  3.72494
3.29354  2.67741  2.69355  4.24690  2.90347  2.73739  3.18146  2.89801
2.37887  2.77519  2.98518  4.58477  3.61503
          0.00484  5.72855  6.45090  0.61958  0.77255  0.48576  0.95510
```

```
     46   5.79102  0.02375  6.89345  6.96636  7.19240  5.75517  7.43306
7.19073  7.03690  6.63065  7.84655  6.93829  6.46707  7.30890  6.83866
6.06698  6.32816  6.69384  7.83763  7.41600     92 C - - -
          2.68618  4.42225  2.77519  2.73123  3.46354  2.40513  3.72494
3.29354  2.67741  2.69355  4.24690  2.90347  2.73739  3.18146  2.89801
2.37887  2.77519  2.98518  4.58477  3.61503
          0.00484  5.72855  6.45090  0.61958  0.77255  0.48576  0.95510
     47   1.00090  4.73450  5.12549  4.19200  3.41074  3.97799  4.80576
2.30084  4.34220  2.53780  3.51433  4.58620  4.84691  4.17477  4.41025
2.34921  3.17799  2.16105  4.90639  4.17331     93 a - - -
          2.68618  4.42225  2.77519  2.73123  3.46354  2.40513  3.72494
3.29354  2.67741  2.69355  4.24690  2.90347  2.73739  3.18146  2.89801
2.37887  2.77519  2.98518  4.58477  3.61503
          0.00326  5.72697        *  0.61958  0.77255  0.00000        *
//
HMMER3/f [3.3 | Nov 2019]
NAME  CAF1C_H4-bd
ACC   PF12265.9
DESC  Histone-binding protein RBBP4 or subunit C of CAF1 complex
LENG  67
ALPH  amino
RF    no
MM    no
CONS  yes
CS    yes
MAP   yes
DATE  Sat Apr 18 22:41:23 2020
NSEQ  233
EFFN  7.931870
CKSUM 3604089371
GA    22.70 22.70
TC    22.70 22.90
NC    22.60 22.60
STATS LOCAL MSV       -8.4599  0.71879
STATS LOCAL VITERBI   -9.3663  0.71879
STATS LOCAL FORWARD   -3.8783  0.71879
HMM          A        C        D        E        F        G        H
I        K        L        M        N        P        Q        R        S
T        V        W        Y
            m->m     m->i     m->d     i->m     i->i     d->m     d->d
  COMPO   2.78991  4.08672  2.96056  2.73549  3.38496  3.10416  3.61082
2.90457  2.79560  2.31335  3.67859  2.97922  3.03488  3.02451  3.20551
2.57221  2.67902  2.76068  3.89464  3.15382
          2.68618  4.42225  2.77519  2.73123  3.46354  2.40513  3.72494
3.29354  2.67741  2.69355  4.24690  2.90347  2.73739  3.18146  2.89801
2.37887  2.77519  2.98518  4.58477  3.61503
          0.23647  5.75372  1.57300  0.61958  0.77255  0.00000        *
      1   2.97575  4.77868  1.64210  1.29133  5.03553  2.74528  4.12786
4.52385  2.70612  3.99665  4.73497  3.02315  4.32866  2.64503  3.15098
3.13254  3.40761  4.08221  6.12404  4.70823      1 e - - H
          2.68618  4.42225  2.77519  2.73123  3.46354  2.40513  3.72494
3.29354  2.67741  2.69355  4.24690  2.90347  2.73739  3.18146  2.89801
2.37887  2.77519  2.98518  4.58477  3.61503
          0.00595  5.52320  6.24554  0.61958  0.77255  0.39757  1.11459
      2   3.05206  5.74082  3.22784  0.95014  5.08694  3.99988  4.18740
4.57330  2.27431  4.04619  4.51999  2.62253  4.39412  2.61341  2.79228
3.08643  3.03625  4.01220  6.17243  4.76128      2 e - - H
```

2.68618 4.42225 2.77519 2.73123 3.46354 2.40513 3.72494
3.29354 2.67741 2.69355 4.24690 2.90347 2.73739 3.18146 2.89801
2.37887 2.77519 2.98518 4.58477 3.61503
0.00501 5.69389 6.41624 0.61958 0.77255 0.47955 0.96511
3 3.25307 5.60881 3.47905 1.94612 2.98672 4.05304 3.23792
3.40270 2.66265 3.78950 4.66552 3.19493 4.44484 2.41370 2.95589
3.19261 3.40559 2.95812 6.07851 1.51779 3 y - - H
2.68618 4.42225 2.77519 2.73123 3.46354 2.40513 3.72494
3.29354 2.67741 2.69355 4.24690 2.90347 2.73739 3.18146 2.89801
2.37887 2.77519 2.98518 4.58477 3.61503
0.00483 5.73201 6.45435 0.61958 0.77255 0.41687 1.07618
4 3.23451 5.07052 3.27632 2.70999 4.70740 3.54484 3.64642
4.26357 1.91201 1.54168 4.27507 3.00410 4.43578 2.59462 2.15735
2.94986 3.12561 3.98856 4.00351 4.46584 4 l - - H
2.68618 4.42225 2.77519 2.73123 3.46354 2.40513 3.72494
3.29354 2.67741 2.69355 4.24690 2.90347 2.73739 3.18146 2.89801
2.37887 2.77519 2.98518 4.58477 3.61503
0.00472 5.75372 6.47607 0.61958 0.77255 0.48576 0.95510
5 3.17900 5.66116 2.82509 1.81622 4.81172 4.05622 3.76655
1.82842 2.58296 2.61424 4.48092 2.67950 4.44851 3.00492 3.04143
3.24924 2.95157 2.50518 6.12138 4.50181 5 e - - H
2.68618 4.42225 2.77519 2.73123 3.46354 2.40513 3.72494
3.29354 2.67741 2.69355 4.24690 2.90347 2.73739 3.18146 2.89801
2.37887 2.77519 2.98518 4.58477 3.61503
0.00472 5.75372 6.47607 0.61958 0.77255 0.48576 0.95510
6 2.77239 3.38389 5.29878 4.68474 2.76608 4.52002 4.55371
3.10812 4.34330 2.84872 2.93922 4.67660 3.05522 4.60785 4.48772
3.83057 3.58485 2.35063 0.98231 3.22697 6 w - - H
2.68618 4.42225 2.77519 2.73123 3.46354 2.40513 3.72494
3.29354 2.67741 2.69355 4.24690 2.90347 2.73739 3.18146 2.89801
2.37887 2.77519 2.98518 4.58477 3.61503
0.00472 5.75372 6.47607 0.61958 0.77255 0.48576 0.95510
7 3.27570 5.77111 1.58085 2.80926 5.11332 4.04866 4.23258
3.88672 1.19748 3.55186 4.81343 3.51814 4.44181 3.16992 2.43722
2.90340 3.50601 3.51137 6.20340 4.79910 7 k - - H
2.68618 4.42225 2.77519 2.73123 3.46354 2.40513 3.72494
3.29354 2.67741 2.69355 4.24690 2.90347 2.73739 3.18146 2.89801
2.37887 2.77519 2.98518 4.58477 3.61503
0.00472 5.75372 6.47607 0.61958 0.77255 0.48576 0.95510
8 3.25845 5.76773 3.42450 2.15563 5.11228 4.03240 4.14510
4.59783 1.35655 3.15206 4.66588 2.62200 2.77558 2.71240 2.58410
2.66433 3.35344 3.13165 6.20069 3.74891 8 k - - H
2.68618 4.42225 2.77519 2.73123 3.46354 2.40513 3.72494
3.29354 2.67741 2.69355 4.24690 2.90347 2.73739 3.18146 2.89801
2.37887 2.77519 2.98518 4.58477 3.61503
0.00472 5.75372 6.47607 0.61958 0.77255 0.48576 0.95510
9 3.20414 3.99387 3.55557 2.75992 4.97476 3.65228 3.76351
4.43284 2.72581 3.05095 4.72220 1.23890 4.44670 3.29439 3.17425
1.84816 2.84491 3.92231 6.12847 3.18183 9 n - - H
2.68618 4.42225 2.77519 2.73123 3.46354 2.40513 3.72494
3.29354 2.67741 2.69355 4.24690 2.90347 2.73739 3.18146 2.89801
2.37887 2.77519 2.98518 4.58477 3.61503
0.00472 5.75372 6.47607 0.61958 0.77255 0.48576 0.95510
10 1.38047 2.82927 4.79729 4.21078 3.92453 4.44033 4.74548
2.86905 4.09214 2.75602 3.87140 3.66385 4.81128 3.32323 4.27484
2.83521 1.83267 1.91640 5.43204 4.23986 10 a - - H

2.68618 4.42225 2.77519 2.73123 3.46354 2.40513 3.72494
3.29354 2.67741 2.69355 4.24690 2.90347 2.73739 3.18146 2.89801
2.37887 2.77519 2.98518 4.58477 3.61503
0.44781 5.75372 1.02777 0.61958 0.77255 0.48576 0.95510
11 2.86551 5.26717 3.41445 2.85578 4.51649 3.42989 3.93872
3.13579 2.60882 3.29390 4.22204 3.37858 1.26456 2.93991 2.57399
2.49066 3.26417 3.38133 5.76163 4.41996 11 p - - H
2.68618 4.42225 2.77519 2.73123 3.46354 2.40513 3.72494
3.29354 2.67741 2.69355 4.24690 2.90347 2.73739 3.18146 2.89801
2.37887 2.77519 2.98518 4.58477 3.61503
0.00734 5.31326 6.03560 0.61958 0.77255 1.62827 0.21849
12 3.08344 4.46216 4.15592 4.40383 1.58347 4.24896 4.28983
2.47523 4.19521 1.62680 3.27128 4.40041 4.61249 4.13920 4.21338
3.51616 3.31555 2.24799 4.70283 2.17028 12 f - - H
2.68618 4.42225 2.77519 2.73123 3.46354 2.40513 3.72494
3.29354 2.67741 2.69355 4.24690 2.90347 2.73739 3.18146 2.89801
2.37887 2.77519 2.98518 4.58477 3.61503
0.00734 5.31326 6.03560 0.61958 0.77255 1.62827 0.21849
13 3.30623 4.67817 5.17963 4.57204 3.61444 4.48115 4.07283
2.54218 4.37377 0.66772 2.45404 4.33543 4.81473 4.49890 4.40563
3.79450 3.53511 2.63997 5.26442 3.91393 13 L - - H
2.68618 4.42225 2.77519 2.73123 3.46354 2.40513 3.72494
3.29354 2.67741 2.69355 4.24690 2.90347 2.73739 3.18146 2.89801
2.37887 2.77519 2.98518 4.58477 3.61503
0.00734 5.31326 6.03560 0.61958 0.77255 0.19985 1.70844
14 5.94932 6.78713 6.57861 6.63252 3.16754 6.19567 4.89873
5.55571 6.42162 4.60694 6.04716 5.81885 6.50471 6.03080 6.16945
5.74097 6.16794 5.53206 4.96541 0.10657 14 Y - - E
2.68618 4.42225 2.77519 2.73123 3.46354 2.40513 3.72494
3.29354 2.67741 2.69355 4.24690 2.90347 2.73739 3.18146 2.89801
2.37887 2.77519 2.98518 4.58477 3.61503
0.00846 5.72269 5.26819 0.61958 0.77255 0.54883 0.86186
15 3.24742 5.75810 0.97673 2.15286 5.10305 4.01993 3.70688
4.29686 2.70170 3.82064 4.79889 2.74499 4.17876 3.12058 2.90937
2.89356 3.42897 3.57598 6.19071 4.62632 15 d - - E
2.68618 4.42225 2.77519 2.73123 3.46354 2.40513 3.72494
3.29354 2.67741 2.69355 4.24690 2.90347 2.73739 3.18146 2.89801
2.37887 2.77519 2.98518 4.58477 3.61503
0.00483 5.73083 6.45317 0.61958 0.77255 0.41373 1.08229
16 2.93324 3.93704 5.30549 4.53749 2.05440 4.52013 4.16493
2.97115 4.47793 1.77120 1.67390 3.75681 4.88393 4.61104 4.22107
3.07842 3.06355 2.80049 3.81280 2.24807 16 m - - E
2.68618 4.42225 2.77519 2.73123 3.46354 2.40513 3.72494
3.29354 2.67741 2.69355 4.24690 2.90347 2.73739 3.18146 2.89801
2.37887 2.77519 2.98518 4.58477 3.61503
0.00472 5.75372 6.47607 0.61958 0.77255 0.48576 0.95510
17 3.31074 3.47018 5.34075 4.72464 2.81215 4.53464 4.57407
1.73273 4.50663 1.25419 3.34001 4.70164 4.89719 4.63440 4.30390
3.84677 3.59497 1.70323 5.35694 2.92556 17 l - - E
2.68618 4.42225 2.77519 2.73123 3.46354 2.40513 3.72494
3.29354 2.67741 2.69355 4.24690 2.90347 2.73739 3.18146 2.89801
2.37887 2.77519 2.98518 4.58477 3.61503
0.00472 5.75372 6.47607 0.61958 0.77255 0.48576 0.95510
18 2.94824 4.76330 5.06791 4.46786 2.44203 4.48720 1.64716
2.35635 4.30306 2.23199 2.46772 3.70202 4.85346 3.96328 4.12467
2.24726 2.89330 2.73817 5.38086 3.44122 18 h - - E

```
        2.68619  4.42227  2.77521  2.73125  3.46355  2.40496  3.72496
3.29356  2.67742  2.69356  4.24691  2.90348  2.73741  3.18148  2.89802
2.37888  2.77521  2.98520  4.58478  3.61505
        0.02418  3.80089  6.47607  0.34483  1.23216  0.48576  0.95510
     19  2.76106  3.30961  2.85096  2.28069  5.10335  3.80943  3.24434
3.59776  2.89626  4.06541  4.17504  2.91592  4.26097  2.87445  2.72614
1.79152  1.75451  4.03920  6.19612  4.78711      20 t - - E
        2.68618  4.42225  2.77519  2.73123  3.46354  2.40513  3.72494
3.29354  2.67741  2.69355  4.24690  2.90347  2.73739  3.18146  2.89801
2.37887  2.77519  2.98518  4.58477  3.61503
        0.00472  5.75372  6.47607  0.61958  0.77255  0.48576  0.95510
     20  2.76810  3.85117  3.93830  3.81019  3.04177  4.35683  1.76851
2.40171  3.34100  2.50900  3.37011  2.61398  2.99061  3.65916  3.54475
3.46649  2.37462  2.74628  4.99838  3.56978      21 h - - E
        2.68618  4.42225  2.77519  2.73123  3.46354  2.40513  3.72494
3.29354  2.67741  2.69355  4.24690  2.90347  2.73739  3.18146  2.89801
2.37887  2.77519  2.98518  4.58477  3.61503
        0.00472  5.75372  6.47607  0.61958  0.77255  0.48576  0.95510
     21  2.14462  5.76595  3.21854  2.46953  5.10977  3.76205  3.86436
3.28065  2.57937  3.38639  4.60193  2.35337  3.30238  2.54908  2.69520
2.12844  2.27372  3.27674  6.19939  4.78919      22 s - - E
        2.68618  4.42225  2.77519  2.73123  3.46354  2.40513  3.72494
3.29354  2.67741  2.69355  4.24690  2.90347  2.73739  3.18146  2.89801
2.37887  2.77519  2.98518  4.58477  3.61503
        0.00667  5.75372  5.66229  0.61958  0.77255  0.48576  0.95510
     22  3.00339  4.49573  4.26993  4.46599  3.85863  4.48581  4.56683
3.09901  4.30129  0.98657  3.12736  3.97406  4.46031  4.02741  4.39479
3.49295  1.86400  2.09803  5.37969  3.79992      23 l - - -
        2.68618  4.42225  2.77519  2.73123  3.46354  2.40513  3.72494
3.29354  2.67741  2.69355  4.24690  2.90347  2.73739  3.18146  2.89801
2.37887  2.77519  2.98518  4.58477  3.61503
        0.00473  5.75179  6.47413  0.61958  0.77255  0.49729  0.93694
     23  3.04219  5.76420  2.38151  1.67815  4.83314  2.79353  4.21871
4.48084  2.24529  2.84471  4.68098  3.31268  2.47028  2.94961  3.32460
2.95352  2.61309  2.87667  6.19780  4.78776      24 e - - S
        2.68618  4.42225  2.77519  2.73123  3.46354  2.40513  3.72494
3.29354  2.67741  2.69355  4.24690  2.90347  2.73739  3.18146  2.89801
2.37887  2.77519  2.98518  4.58477  3.61503
        0.00473  5.75179  6.47413  0.61958  0.77255  0.49729  0.93694
     24  2.68288  4.83669  4.72022  3.83766  3.14496  4.42574  3.77651
2.99411  4.02670  2.94463  3.95058  4.37477  4.79636  3.66733  3.93920
3.32880  3.16645  3.09208  0.78275  3.53623      25 w - - S
        2.68618  4.42225  2.77519  2.73123  3.46354  2.40513  3.72494
3.29354  2.67741  2.69355  4.24690  2.90347  2.73739  3.18146  2.89801
2.37887  2.77519  2.98518  4.58477  3.61503
        0.00473  5.75179  6.47413  0.61958  0.77255  0.47862  0.96663
     25  3.84891  6.15805  3.89512  3.39851  5.80689  4.50278  4.96743
5.22015  3.55665  4.73709  5.59304  4.16788  0.34880  3.13940  3.48058
3.51665  4.30724  4.40003  6.86730  5.58867      26 P - - -
        2.68618  4.42225  2.77519  2.73123  3.46354  2.40513  3.72494
3.29354  2.67741  2.69355  4.24690  2.90347  2.73739  3.18146  2.89801
2.37887  2.77519  2.98518  4.58477  3.61503
        0.00472  5.75372  6.47607  0.61958  0.77255  0.48576  0.95510
     26  3.14959  1.53477  3.43084  3.35734  4.04514  4.18734  4.45345
3.87140  3.33842  3.48642  4.39302  3.76052  3.47219  3.67088  3.48744
1.22224  2.48073  3.59759  5.84938  4.42853      27 s - - -
```

2.68618 4.42225 2.77519 2.73123 3.46354 2.40513 3.72494
3.29354 2.67741 2.69355 4.24690 2.90347 2.73739 3.18146 2.89801
2.37887 2.77519 2.98518 4.58477 3.61503
0.03590 5.75372 3.43917 0.61958 0.77255 0.48576 0.95510
27 4.33682 5.65916 6.21386 5.63445 4.10950 5.55048 5.93884
3.30182 5.43603 0.30163 3.08561 5.74106 4.12347 5.44744 5.43169
4.91613 2.87573 3.55111 6.16122 5.14115 28 L - - S
2.68618 4.42225 2.77519 2.73123 3.46354 2.40513 3.72494
3.29354 2.67741 2.69355 4.24690 2.90347 2.73739 3.18146 2.89801
2.37887 2.77519 2.98518 4.58477 3.61503
0.00487 5.72269 6.44504 0.61958 0.77255 0.54883 0.86186
28 3.12265 4.21508 5.00840 4.51258 4.20493 3.75851 5.23590
3.77403 4.42840 3.79149 4.68137 4.60591 4.91505 4.66923 4.66545
0.94856 1.07247 3.25291 6.16331 4.97564 29 s - - -
2.68618 4.42225 2.77519 2.73123 3.46354 2.40513 3.72494
3.29354 2.67741 2.69355 4.24690 2.90347 2.73739 3.18146 2.89801
2.37887 2.77519 2.98518 4.58477 3.61503
0.00481 5.73436 6.45670 0.61958 0.77255 0.42329 1.06388
29 2.74446 2.43376 5.48058 4.87509 1.37565 4.69627 5.04976
2.08023 4.66760 2.41269 3.90900 4.86257 5.05066 4.80282 4.67940
4.01459 3.19886 1.52891 5.53241 4.35455 30 f - - E
2.68618 4.42225 2.77519 2.73123 3.46354 2.40513 3.72494
3.29354 2.67741 2.69355 4.24690 2.90347 2.73739 3.18146 2.89801
2.37887 2.77519 2.98518 4.58477 3.61503
0.00472 5.75372 6.47607 0.61958 0.77255 0.48576 0.95510
30 3.25774 5.30573 1.32007 2.25938 5.14624 3.38996 3.73275
4.63357 2.95182 4.10647 4.84316 3.48193 4.44681 1.53551 2.90108
2.72107 3.45656 4.19213 6.23333 4.81989 31 d - - E
2.68618 4.42225 2.77519 2.73123 3.46354 2.40513 3.72494
3.29354 2.67741 2.69355 4.24690 2.90347 2.73739 3.18146 2.89801
2.37887 2.77519 2.98518 4.58477 3.61503
0.02991 5.75372 3.63846 0.61958 0.77255 0.48576 0.95510
31 3.33826 4.58285 5.29989 4.68499 1.66339 4.50717 4.66061
2.11290 4.47010 2.72629 3.83181 4.66864 4.13222 4.60128 3.95264
3.81845 3.27195 2.08512 1.43844 3.36854 32 w - - E
2.68846 4.42345 2.77308 2.72895 3.46513 2.40570 3.72532
3.29128 2.67541 2.69120 4.24764 2.90254 2.73985 3.18296 2.89598
2.38000 2.77659 2.98620 4.58881 3.61602
0.46592 1.11252 3.13014 2.71935 0.06819 0.40808 1.09339
32 3.32164 4.71541 4.50114 4.15762 1.74728 2.68952 4.80461
2.62963 4.36971 1.60711 2.87722 3.75521 4.84247 3.52995 4.41977
3.78359 3.55379 2.86041 4.66859 1.92604 61 l - - -
2.68618 4.42225 2.77519 2.73123 3.46354 2.40513 3.72494
3.29354 2.67741 2.69355 4.24690 2.90347 2.73739 3.18146 2.89801
2.37887 2.77519 2.98518 4.58477 3.61503
0.00492 5.71176 6.43411 0.61958 0.77255 0.70586 0.68059
33 3.21329 5.75092 3.47484 2.95690 5.03537 3.94493 4.08466
4.47473 2.97662 3.66208 4.82620 3.57034 0.62057 3.35731 3.55388
3.07896 3.39360 4.11223 6.22690 4.86106 62 P - - S
2.68618 4.42225 2.77519 2.73123 3.46354 2.40513 3.72494
3.29354 2.67741 2.69355 4.24690 2.90347 2.73739 3.18146 2.89801
2.37887 2.77519 2.98518 4.58477 3.61503
0.13101 5.71176 2.12460 0.61958 0.77255 0.70586 0.68059
34 2.66853 5.57265 1.79612 2.45233 4.46342 3.58874 2.68549
3.59584 2.75719 2.34127 3.97544 3.14886 3.96905 2.95304 3.38576
2.46836 3.19199 3.10419 6.03055 3.20203 63 d - - -

```
          2.68688  4.42459  2.77365  2.73026  3.46342  2.40604  3.72669
3.29335  2.67655  2.69352  4.24631  2.90294  2.73802  3.18181  2.89907
2.37867  2.77375  2.98506  4.58711  3.61671
          0.54358  0.87343  6.30869  1.99692  0.14590  0.35347  1.21149
     35   3.07116  4.27223  2.72494  2.31813  4.46939  3.80049  3.74563
3.05283  2.57396  3.24516  4.49670  3.37875  2.92896  2.69734  3.22831
2.14996  1.89127  2.93379  6.13276  3.59773     88 t - - B
          2.68702  4.42337  2.77489  2.73019  3.46326  2.40618  3.72587
3.29259  2.67753  2.69403  4.24792  2.90307  2.73788  3.18105  2.89817
2.37717  2.77505  2.98563  4.58611  3.61560
          0.89728  0.77019  2.04485  1.21766  0.35087  0.50371  0.92706
     36   2.38930  3.82510  2.59902  2.48552  4.57004  2.80976  4.01421
3.17329  2.79597  2.98885  3.12199  2.43849  3.91960  3.21261  3.36490
2.66825  2.88420  2.42573  5.92576  3.11054     96 a - - -
          2.68618  4.42225  2.77519  2.73123  3.46354  2.40513  3.72494
3.29354  2.67741  2.69355  4.24690  2.90347  2.73739  3.18146  2.89801
2.37887  2.77519  2.98518  4.58477  3.61503
          0.43529  5.60518  1.05198  0.61958  0.77255  0.86581  0.54595
     37   2.40346  5.48671  2.54211  2.35652  4.83235  3.00884  3.63545
4.31878  2.16995  3.47315  4.52725  2.16168  3.31453  2.55136  3.16258
2.08940  3.04891  3.80194  5.91902  4.50762     97 s - - -
          2.68618  4.42225  2.77519  2.73123  3.46354  2.40513  3.72494
3.29354  2.67741  2.69355  4.24690  2.90347  2.73739  3.18146  2.89801
2.37887  2.77519  2.98518  4.58477  3.61503
          0.06331  5.22282  2.88319  0.61958  0.77255  1.72938  0.19528
     38   2.76702  5.45896  2.62823  1.94437  4.80379  2.74542  3.32286
3.96305  2.60706  3.69802  4.49970  2.17184  3.32602  2.61657  3.08497
2.16067  2.99464  3.79356  5.89178  3.85227     98 e - - -
          2.68618  4.42225  2.77519  2.73123  3.46354  2.40513  3.72494
3.29354  2.67741  2.69355  4.24690  2.90347  2.73739  3.18146  2.89801
2.37887  2.77519  2.98518  4.58477  3.61503
          0.00850  5.16801  5.89036  0.61958  0.77255  1.62788  0.21859
     39   2.53746  4.53769  3.68059  2.97040  2.91272  3.93007  3.96890
2.30884  2.88875  2.22455  3.31135  2.89402  4.26903  3.60921  3.27081
2.70235  2.55259  2.64312  5.26045  2.45786     99 l - - E
          2.68618  4.42225  2.77519  2.73123  3.46354  2.40513  3.72494
3.29354  2.67741  2.69355  4.24690  2.90347  2.73739  3.18146  2.89801
2.37887  2.77519  2.98518  4.58477  3.61503
          0.00824  5.19831  5.92066  0.61958  0.77255  1.62382  0.21958
     40   2.59354  5.00185  3.14815  2.86876  3.93970  2.90227  3.86579
2.50571  2.85375  2.66288  3.80720  2.70866  4.25511  3.28783  3.17014
2.12411  2.17979  2.61448  5.53945  3.79749    100 s - - E
          2.68618  4.42225  2.77519  2.73123  3.46354  2.40513  3.72494
3.29354  2.67741  2.69355  4.24690  2.90347  2.73739  3.18146  2.89801
2.37887  2.77519  2.98518  4.58477  3.61503
          0.06382  5.22282  2.87475  0.61958  0.77255  1.22689  0.34701
     41   2.69297  5.30979  3.20805  2.42985  4.16871  3.60987  4.01582
3.45262  2.01512  3.42502  3.41618  2.82136  4.20941  2.91658  2.69956
2.50841  2.45764  2.35028  4.19140  3.35962    101 k - - E
          2.68618  4.42225  2.77519  2.73123  3.46354  2.40513  3.72494
3.29354  2.67741  2.69355  4.24690  2.90347  2.73739  3.18146  2.89801
2.37887  2.77519  2.98518  4.58477  3.61503
          0.00758  5.28126  6.00360  0.61958  0.77255  1.61758  0.22112
     42   2.71380  5.44332  3.28999  2.69532  4.53984  3.79622  1.93615
4.22196  2.73337  3.73516  4.49251  2.62011  4.18911  1.55427  3.06635
2.44264  3.14884  3.39949  5.89471  3.88113    102 q - - E
```

```
          2.68618  4.42225  2.77519  2.73123  3.46354  2.40513  3.72494
3.29354  2.67741  2.69355  4.24690  2.90347  2.73739  3.18146  2.89801
2.37887  2.77519  2.98518  4.58477  3.61503
          0.01139  5.28944  5.06949  0.61958  0.77255  1.65682  0.21164
     43   3.00740  5.51073  3.12483  2.45799  4.85368  3.78166  3.89882
3.43210  2.14594  3.81452  4.55217  2.50207  4.04806  2.54727  1.42605
2.59749  2.88513  3.90028  5.63205  4.40478    103 r - - E
          2.68674  4.42301  2.77528  2.73084  3.46300  2.40544  3.72464
3.29237  2.67817  2.69411  4.24766  2.90265  2.73696  3.18088  2.89852
2.37847  2.77537  2.98544  4.58553  3.61443
          0.15897  1.93434  6.00795  2.15506  0.12318  0.12643  2.13062
     44   3.17984  3.34001  5.33013  4.71379  2.60651  4.52336  4.85761
1.84587  4.49549  1.51851  3.43193  4.51170  4.18243  4.62403  4.49871
3.83523  2.77195  2.24454  3.68279  2.01074    114 l - - E
          2.68618  4.42225  2.77519  2.73123  3.46354  2.40513  3.72494
3.29354  2.67741  2.69355  4.24690  2.90347  2.73739  3.18146  2.89801
2.37887  2.77519  2.98518  4.58477  3.61503
          0.00472  5.75372  6.47607  0.61958  0.77255  0.48576  0.95510
     45   2.59041  2.85882  5.33135  4.71522  3.29418  4.31480  4.86038
1.97735  4.49734  1.11831  3.16102  4.69253  4.88938  4.62613  4.50108
3.53147  3.58712  2.16247  5.35026  2.77147    115 l - - E
          2.68618  4.42225  2.77519  2.73123  3.46354  2.40513  3.72494
3.29354  2.67741  2.69355  4.24690  2.90347  2.73739  3.18146  2.89801
2.37887  2.77519  2.98518  4.58477  3.61503
          0.00472  5.75372  6.47607  0.61958  0.77255  0.48576  0.95510
     46   2.73997  2.79530  5.32884  4.71270  2.83631  4.52406  4.13444
2.07208  4.49493  1.42629  3.71021  4.69033  4.88759  4.62391  4.49894
3.69071  3.17797  1.46104  5.34859  3.43551    116 l - - E
          2.68373  4.42135  2.77551  2.73154  3.46385  2.40533  3.72526
3.29385  2.67772  2.69386  4.24721  2.90378  2.73771  3.18177  2.89832
2.37860  2.77502  2.98518  4.58508  3.61534
          0.44726  1.02420  6.47607  0.03983  3.24293  0.48576  0.95510
     47   2.29772  3.48326  6.09716  6.06679  6.32413  0.34464  6.48314
5.83366  5.90648  5.53157  6.25792  5.19378  5.12267  5.94692  5.84028
2.59232  3.22739  4.87132  7.67192  6.68560    118 G - - -
          2.68618  4.42225  2.77519  2.73123  3.46354  2.40513  3.72494
3.29354  2.67741  2.69355  4.24690  2.90347  2.73739  3.18146  2.89801
2.37887  2.77519  2.98518  4.58477  3.61503
          0.00472  5.75372  6.47607  0.61958  0.77255  0.48576  0.95510
     48   3.35349  3.31207  4.48219  3.18655  3.66474  4.33768  4.73293
3.54525  3.85768  3.00530  4.18352  4.24531  4.77064  4.13656  4.15687
2.15638  0.71401  3.24936  5.66888  4.45369    119 t - - -
          2.68618  4.42225  2.77519  2.73123  3.46354  2.40513  3.72494
3.29354  2.67741  2.69355  4.24690  2.90347  2.73739  3.18146  2.89801
2.37887  2.77519  2.98518  4.58477  3.61503
          0.00472  5.75372  6.47607  0.61958  0.77255  0.48576  0.95510
     49   3.28082  4.61173  3.71773  3.05598  2.51971  4.12498  2.09150
4.05656  3.10059  3.14559  4.50074  2.82461  4.51379  1.30622  2.77485
3.27924  3.51205  3.34059  5.93805  3.33976    120 q - - -
          2.68618  4.42225  2.77519  2.73123  3.46354  2.40513  3.72494
3.29354  2.67741  2.69355  4.24690  2.90347  2.73739  3.18146  2.89801
2.37887  2.77519  2.98518  4.58477  3.61503
          0.00856  5.75372  5.22972  0.61958  0.77255  0.48576  0.95510
     50   1.46392  4.76896  3.83965  3.29980  4.41137  3.69740  3.95837
3.79248  3.33677  3.13760  4.32831  3.82941  4.03971  3.66664  3.75462
2.53042  1.17462  3.53069  5.78851  3.82133    121 t - - -
```

```
          2.68643  4.42250  2.77537  2.73118  3.46379  2.40471  3.72381
3.29379  2.67766  2.69380  4.24715  2.90372  2.73698  3.18147  2.89788
2.37912  2.77462  2.98525  4.58502  3.61528
          0.09547  2.60421  4.06921  1.30120  0.31774  0.47188  0.97770
     51   2.42346  4.82076  2.07398  2.16134  5.07834  3.57515  4.21433
4.30806  2.95217  2.90973  4.05973  2.56496  3.94395  2.91442  3.44362
1.63155  2.70364  3.95890  5.68083  4.77265    127 s - - T
          2.68618  4.42225  2.77519  2.73123  3.46354  2.40513  3.72494
3.29354  2.67741  2.69355  4.24690  2.90347  2.73739  3.18146  2.89801
2.37887  2.77519  2.98518  4.58477  3.61503
          0.06538  5.73811  2.81235  0.61958  0.77255  0.57450  0.82780
     52   3.03491  5.31033  2.34070  2.41636  5.07211  1.59870  4.07343
4.33027  2.12829  4.03177  4.43309  2.64864  3.81213  2.91839  2.71783
2.82149  2.64160  3.92441  6.15819  4.74666    128 g - - T
          2.68606  4.42288  2.77541  2.73067  3.46416  2.40559  3.72557
3.29308  2.67777  2.69398  4.24731  2.90262  2.73783  3.18039  2.89863
2.37905  2.77455  2.98550  4.58172  3.61389
          0.17775  1.83931  5.54101  1.53127  0.24368  0.42698  1.05692
     53   2.44995  5.75847  2.82710  2.21621  4.79719  2.72080  4.11820
4.51194  1.82981  3.82455  3.83163  2.76271  2.98812  2.25737  2.65736
2.68923  3.14381  3.60082  6.19048  4.77884    139 k - - S
          2.68618  4.42225  2.77519  2.73123  3.46354  2.40513  3.72494
3.29354  2.67741  2.69355  4.24690  2.90347  2.73739  3.18146  2.89801
2.37887  2.77519  2.98518  4.58477  3.61503
          0.00482  5.73222  6.45456  0.61958  0.77255  0.58554  0.81374
     54   2.54112  5.47718  2.11001  1.68593  4.27832  3.61710  3.58892
4.59030  2.15775  3.00228  4.32107  3.06529  4.26578  2.70073  3.14171
2.39246  2.90560  3.73619  6.19074  4.56381    140 e - - S
          2.68618  4.42225  2.77519  2.73123  3.46354  2.40513  3.72494
3.29354  2.67741  2.69355  4.24690  2.90347  2.73739  3.18146  2.89801
2.37887  2.77519  2.98518  4.58477  3.61503
          0.00481  5.73436  6.45670  0.61958  0.77255  0.42329  1.06388
     55   2.95352  5.76810  2.27859  2.59137  5.11286  3.59944  4.07195
3.96434  2.30002  3.28159  4.59882  2.77811  2.28792  1.87625  3.10652
2.73590  2.68425  3.17813  5.20913  4.79019    141 q - - -
          2.68618  4.42225  2.77519  2.73123  3.46354  2.40513  3.72494
3.29354  2.67741  2.69355  4.24690  2.90347  2.73739  3.18146  2.89801
2.37887  2.77519  2.98518  4.58477  3.61503
          0.00472  5.75372  6.47607  0.61958  0.77255  0.48576  0.95510
     56   3.31477  5.94851  2.08964  2.22133  5.28324  4.11666  4.11183
4.77384  3.14164  4.25023  4.99895  0.91959  4.55242  3.47263  3.64861
2.51707  3.33167  3.71023  6.38333  4.65053    142 n - - E
          2.68618  4.42225  2.77519  2.73123  3.46354  2.40513  3.72494
3.29354  2.67741  2.69355  4.24690  2.90347  2.73739  3.18146  2.89801
2.37887  2.77519  2.98518  4.58477  3.61503
          0.00472  5.75372  6.47607  0.61958  0.77255  0.48576  0.95510
     57   3.09756  4.63755  3.54508  2.27459  3.14627  3.81145  2.95261
3.99664  2.63031  3.71029  4.73937  2.91312  4.44203  2.48201  2.97382
2.10044  2.81352  3.41013  6.14293  1.86995    143 y - - E
          2.68618  4.42225  2.77519  2.73123  3.46354  2.40513  3.72494
3.29354  2.67741  2.69355  4.24690  2.90347  2.73739  3.18146  2.89801
2.37887  2.77519  2.98518  4.58477  3.61503
          0.00472  5.75372  6.47607  0.61958  0.77255  0.48576  0.95510
     58   4.21552  5.80420  6.73242  6.21929  4.49496  6.22097  6.74644
0.96216  6.14165  0.91437  3.35622  6.40196  6.28288  6.11453  6.15300
5.65521  4.86454  2.10859  6.75357  5.74405    144 l - - E
```

```
          2.68618  4.42225  2.77519  2.73123  3.46354  2.40513  3.72494
3.29354  2.67741  2.69355  4.24690  2.90347  2.73739  3.18146  2.89801
2.37887  2.77519  2.98518  4.58477  3.61503
          0.00472  5.75372  6.47607  0.61958  0.77255  0.48576  0.95510
     59   2.98344  5.03993  4.17826  3.48713  4.03683  3.82579  3.19044
2.26887  2.63643  2.48780  2.74489  3.35346  4.66813  2.65022  3.74535
2.66948  2.93710  2.37161  5.62084  1.99424    145 y - - E
          2.68618  4.42225  2.77519  2.73123  3.46354  2.40513  3.72494
3.29354  2.67741  2.69355  4.24690  2.90347  2.73739  3.18146  2.89801
2.37887  2.77519  2.98518  4.58477  3.61503
          0.00472  5.75372  6.47607  0.61958  0.77255  0.48576  0.95510
     60   3.93683  5.22967  5.96679  5.39702  4.00643  5.26460  5.68290
1.19745  4.95195  1.81976  4.02377  5.43075  5.56240  4.97123  5.26086
4.61054  4.07984  0.93312  6.09207  4.65678    146 v - - E
          2.68618  4.42225  2.77519  2.73123  3.46354  2.40513  3.72494
3.29354  2.67741  2.69355  4.24690  2.90347  2.73739  3.18146  2.89801
2.37887  2.77519  2.98518  4.58477  3.61503
          0.00472  5.75372  6.47607  0.61958  0.77255  0.48576  0.95510
     61   1.66939  3.65707  5.30397  4.68942  3.22078  3.21510  4.85289
2.14049  3.52834  1.78792  1.90751  4.67857  4.88376  4.61024  4.48910
2.96687  3.50973  2.77374  4.03109  3.84044    147 a - - E
          2.68618  4.42225  2.77519  2.73123  3.46354  2.40513  3.72494
3.29354  2.67741  2.69355  4.24690  2.90347  2.73739  3.18146  2.89801
2.37887  2.77519  2.98518  4.58477  3.61503
          0.00472  5.75372  6.47607  0.61958  0.77255  0.48576  0.95510
     62   3.00538  5.77201  2.59778  2.19807  5.11841  3.55593  3.84159
4.60530  1.62180  4.07784  4.81233  3.12635  4.42502  2.07578  2.32109
2.10396  3.32756  3.76175  6.20388  4.53719    148 k - - E
          2.68618  4.42225  2.77519  2.73123  3.46354  2.40513  3.72494
3.29354  2.67741  2.69355  4.24690  2.90347  2.73739  3.18146  2.89801
2.37887  2.77519  2.98518  4.58477  3.61503
          0.00472  5.75372  6.47607  0.61958  0.77255  0.48576  0.95510
     63   2.78791  3.99131  5.33549  4.71957  3.66272  4.53034  4.86514
2.07266  4.50185  1.52714  2.47261  4.69698  4.89350  4.63068  4.50564
3.57365  3.15715  1.45900  2.69069  4.17909    149 v - - E
          2.68618  4.42225  2.77519  2.73123  3.46354  2.40513  3.72494
3.29354  2.67741  2.69355  4.24690  2.90347  2.73739  3.18146  2.89801
2.37887  2.77519  2.98518  4.58477  3.61503
          0.06691  5.75372  2.78794  0.61958  0.77255  0.48576  0.95510
     64   2.39826  5.18758  2.29469  2.29832  3.65783  3.34442  3.21063
4.38274  2.41816  3.42332  4.77029  3.24955  4.28245  2.72796  3.16203
1.68419  2.55874  3.84818  5.66236  4.58763    150 s - - E
          2.68618  4.42225  2.77519  2.73123  3.46354  2.40513  3.72494
3.29354  2.67741  2.69355  4.24690  2.90347  2.73739  3.18146  2.89801
2.37887  2.77519  2.98518  4.58477  3.61503
          0.02111  5.69184  4.04462  0.61958  0.77255  0.79292  0.60243
     65   3.13504  4.84804  3.75170  2.97743  3.49340  3.55319  3.52833
2.32455  2.99008  1.58903  4.16067  1.82415  4.55569  3.11430  3.50809
2.95548  3.08083  2.94819  4.67096  4.41947    151 l - - E
          2.68618  4.42225  2.77519  2.73123  3.46354  2.40513  3.72494
3.29354  2.67741  2.69355  4.24690  2.90347  2.73739  3.18146  2.89801
2.37887  2.77519  2.98518  4.58477  3.61503
          0.01629  5.67583  4.36345  0.61958  0.77255  0.85662  0.55268
     66   3.44091  4.83387  5.23750  4.63758  3.86755  4.60277  4.93524
2.71853  4.45846  1.30610  2.83725  3.75150  1.14858  4.61487  4.52571
3.91194  3.59124  2.61632  5.45916  4.29139    152 p - - E
```

```
          2.68618  4.42225  2.77519  2.73123  3.46354  2.40513  3.72494
3.29354  2.67741  2.69355  4.24690  2.90347  2.73739  3.18146  2.89801
2.37887  2.77519  2.98518  4.58477  3.61503
          0.22788  5.66470  1.60786  0.61958  0.77255  0.89810  0.52314
     67   2.98484  2.98543  2.90575  2.61479  4.92348  3.85934  2.28969
4.40612  2.25836  3.16420  4.62506  2.14879  4.25270  2.75790  2.83268
2.21485  2.72333  3.58917  6.01868  4.22649    153 n - - X
          2.68618  4.42225  2.77519  2.73123  3.46354  2.40513  3.72494
3.29354  2.67741  2.69355  4.24690  2.90347  2.73739  3.18146  2.89801
2.37887  2.77519  2.98518  4.58477  3.61503
          0.00434  5.44116        *  0.61958  0.77255  0.00000        *
//
HMMER3/f [3.3 | Nov 2019]
NAME  CBFB_NFYA
ACC   PF02045.16
DESC  CCAAT-binding transcription factor (CBF-B/NF-YA) subunit B
LENG  56
ALPH  amino
RF    no
MM    no
CONS  yes
CS    no
MAP   yes
DATE  Thu Apr 16 09:56:53 2020
NSEQ  56
EFFN  2.594238
CKSUM 3197731095
GA    20.10 20.10
TC    20.40 20.10
NC    19.00 19.90
STATS LOCAL MSV       -8.5715  0.71901
STATS LOCAL VITERBI   -9.0660  0.71901
STATS LOCAL FORWARD   -3.5881  0.71901
HMM          A        C        D        E        F        G        H
I        K        L        M        N        P        Q        R        S
T        V        W        Y
            m->m     m->i     m->d     i->m     i->i     d->m     d->d
  COMPO   2.58481  4.75289  3.34151  2.73117  3.45341  2.96595  3.29199
3.15793  2.39994  2.80319  3.79036  2.92473  3.43175  2.77890  1.99722
2.77567  3.15172  3.06101  5.29215  3.27333
          2.68618  4.42225  2.77519  2.73123  3.46354  2.40513  3.72494
3.29354  2.67741  2.69355  4.24690  2.90347  2.73739  3.18146  2.89801
2.37887  2.77519  2.98518  4.58477  3.61503
          0.19937  4.81512  1.75651  0.61958  0.77255  0.00000        *
      1   2.43533  5.24605  2.98670  1.65000  4.58392  3.03946  3.14411
4.06311  2.24378  3.54655  4.29008  2.99265  3.73801  2.36260  2.78438
2.60844  2.98741  3.63312  5.68104  4.27661      1 e - - -
          2.68618  4.42225  2.77519  2.73123  3.46354  2.40513  3.72494
3.29354  2.67741  2.69355  4.24690  2.90347  2.73739  3.18146  2.89801
2.37887  2.77519  2.98518  4.58477  3.61503
          0.01459  4.63034  5.35269  0.61958  0.77255  0.69084  0.69546
      2   2.34614  4.61491  3.66216  3.20431  4.19072  3.34459  4.24888
3.55975  3.18421  3.26120  4.12360  3.53705  1.05080  3.50935  3.54774
2.87391  2.64092  3.06409  5.57321  3.75980      2 p - - -
          2.68618  4.42225  2.77519  2.73123  3.46354  2.40513  3.72494
3.29354  2.67741  2.69355  4.24690  2.90347  2.73739  3.18146  2.89801
2.37887  2.77519  2.98518  4.58477  3.61503
          0.01398  4.67259  5.39494  0.61958  0.77255  0.63031  0.76020
```

```
      3   2.83296  4.23865  4.57516  3.97613  3.13772  3.98638  4.31045
1.77693  3.80857  1.65168  3.21903  4.07089  4.34543  3.98050  2.78819
3.28962  2.84175  1.86419  4.86820  3.48346      3 l - - -
          2.68618  4.42225  2.77519  2.73123  3.46354  2.40513  3.72494
3.29354  2.67741  2.69355  4.24690  2.90347  2.73739  3.18146  2.89801
2.37887  2.77519  2.98518  4.58477  3.61503
          0.01343  4.71260  5.43495  0.61958  0.77255  0.69145  0.69485
      4   3.03212  4.51644  4.31687  3.76514  2.17898  4.07100  3.44412
2.98527  3.64971  2.29958  3.43349  3.95396  3.77190  3.42828  3.84935
3.35805  3.26185  2.79659  4.56112  1.22898      4 y - - -
          2.68618  4.42225  2.77519  2.73123  3.46354  2.40513  3.72494
3.29354  2.67741  2.69355  4.24690  2.90347  2.73739  3.18146  2.89801
2.37887  2.77519  2.98518  4.58477  3.61503
          0.01343  4.71260  5.43495  0.61958  0.77255  0.37577  1.16079
      5   3.69527  4.90322  5.72910  5.24893  4.07776  5.26468  5.92462
1.91579  5.16493  1.96395  3.82651  5.41519  5.47161  5.37107  5.30436
4.69125  3.95149  0.57248  6.20091  5.02157      5 V - - -
          2.68618  4.42225  2.77519  2.73123  3.46354  2.40513  3.72494
3.29354  2.67741  2.69355  4.24690  2.90347  2.73739  3.18146  2.89801
2.37887  2.77519  2.98518  4.58477  3.61503
          0.01212  4.81512  5.53746  0.61958  0.77255  0.48576  0.95510
      6   3.90183  5.76247  3.75991  3.77243  5.32683  4.16140  5.17165
5.36137  4.23599  4.88817  5.90678  0.22323  4.87929  4.52629  4.55699
3.97810  4.35202  4.86597  6.48096  5.26900      6 N - - -
          2.68618  4.42225  2.77519  2.73123  3.46354  2.40513  3.72494
3.29354  2.67741  2.69355  4.24690  2.90347  2.73739  3.18146  2.89801
2.37887  2.77519  2.98518  4.58477  3.61503
          0.01212  4.81512  5.53746  0.61958  0.77255  0.48576  0.95510
      7   1.53516  4.77441  3.22188  2.75474  3.95927  3.70104  3.92942
3.16361  2.81256  3.01015  3.54605  3.31208  2.56467  2.91332  3.23798
2.77518  3.04313  2.41504  5.32406  4.04149      7 a - - -
          2.68618  4.42225  2.77519  2.73123  3.46354  2.40513  3.72494
3.29354  2.67741  2.69355  4.24690  2.90347  2.73739  3.18146  2.89801
2.37887  2.77519  2.98518  4.58477  3.61503
          0.01212  4.81512  5.53746  0.61958  0.77255  0.48576  0.95510
      8   2.67014  5.55930  3.60994  2.97393  5.00553  3.94667  3.64547
4.38477  0.84242  3.81080  4.62831  3.07440  4.31377  3.06122  2.17195
3.24420  3.45602  4.01700  5.86197  4.62598      8 k - - -
          2.68618  4.42225  2.77519  2.73123  3.46354  2.40513  3.72494
3.29354  2.67741  2.69355  4.24690  2.90347  2.73739  3.18146  2.89801
2.37887  2.77519  2.98518  4.58477  3.61503
          0.01212  4.81512  5.53746  0.61958  0.77255  0.48576  0.95510
      9   4.14586  5.92642  4.04072  3.90547  5.25889  4.36083  5.02791
5.17326  3.68479  4.55064  5.64459  4.33861  4.97899  0.23183  3.90644
4.20076  4.48801  4.85672  6.34999  5.19881      9 Q - - -
          2.68618  4.42225  2.77519  2.73123  3.46354  2.40513  3.72494
3.29354  2.67741  2.69355  4.24690  2.90347  2.73739  3.18146  2.89801
2.37887  2.77519  2.98518  4.58477  3.61503
          0.01212  4.81512  5.53746  0.61958  0.77255  0.48576  0.95510
     10   4.43051  5.52305  5.57943  5.36129  1.62651  5.16915  4.06056
3.88946  5.15147  1.84718  4.43960  4.82937  5.42557  4.92531  5.02444
4.55023  4.63533  3.88846  4.15318  0.73884     10 y - - -
          2.68618  4.42225  2.77519  2.73123  3.46354  2.40513  3.72494
3.29354  2.67741  2.69355  4.24690  2.90347  2.73739  3.18146  2.89801
2.37887  2.77519  2.98518  4.58477  3.61503
          0.01212  4.81512  5.53746  0.61958  0.77255  0.48576  0.95510
```

```
     11   2.62325  5.31310  2.78557  2.39616  4.65621  3.42908  2.26475
4.14042  2.20541  3.61711  4.35459  2.22060  3.97419  2.38643  2.62259
2.67326  2.95169  3.55142  5.74640  4.33710      11 k - - -
          2.68618  4.42225  2.77519  2.73123  3.46354  2.40513  3.72494
3.29354  2.67741  2.69355  4.24690  2.90347  2.73739  3.18146  2.89801
2.37887  2.77519  2.98518  4.58477  3.61503
          0.01212  4.81512  5.53746  0.61958  0.77255  0.48576  0.95510
     12   2.43223  3.35497  3.42483  2.85925  3.98766  2.92019  3.94064
3.37239  2.79049  3.03594  3.89303  3.33332  4.10509  3.06485  1.37990
2.85053  3.06341  3.10381  4.71020  3.79338      12 r - - -
          2.68618  4.42225  2.77519  2.73123  3.46354  2.40513  3.72494
3.29354  2.67741  2.69355  4.24690  2.90347  2.73739  3.18146  2.89801
2.37887  2.77519  2.98518  4.58477  3.61503
          0.01212  4.81512  5.53746  0.61958  0.77255  0.48576  0.95510
     13   3.78228  5.01207  5.71517  5.22753  3.84687  5.23878  5.79586
0.54827  5.07547  2.19791  2.74056  5.40074  5.43365  5.22696  5.16768
4.67032  4.03430  2.15987  6.00025  4.87778      13 I - - -
          2.68618  4.42225  2.77519  2.73123  3.46354  2.40513  3.72494
3.29354  2.67741  2.69355  4.24690  2.90347  2.73739  3.18146  2.89801
2.37887  2.77519  2.98518  4.58477  3.61503
          0.01212  4.81512  5.53746  0.61958  0.77255  0.48576  0.95510
     14   2.84438  4.37670  4.12145  3.53840  3.47021  3.91330  4.19679
2.49245  3.02913  1.50330  2.25167  3.81689  4.28224  2.57445  3.34334
3.18895  3.07545  2.35625  4.97837  3.78085      14 l - - -
          2.68618  4.42225  2.77519  2.73123  3.46354  2.40513  3.72494
3.29354  2.67741  2.69355  4.24690  2.90347  2.73739  3.18146  2.89801
2.37887  2.77519  2.98518  4.58477  3.61503
          0.01212  4.81512  5.53746  0.61958  0.77255  0.48576  0.95510
     15   3.06342  5.44120  3.55514  2.74752  4.82752  3.88910  3.64593
2.76905  1.16852  3.68752  4.50057  3.38036  4.25161  2.95511  1.71951
3.15233  3.35873  3.86043  5.78240  4.52378      15 k - - -
          2.68618  4.42225  2.77519  2.73123  3.46354  2.40513  3.72494
3.29354  2.67741  2.69355  4.24690  2.90347  2.73739  3.18146  2.89801
2.37887  2.77519  2.98518  4.58477  3.61503
          0.01212  4.81512  5.53746  0.61958  0.77255  0.48576  0.95510
     16   3.60181  5.47301  4.40154  3.62416  4.68056  4.24099  4.23809
4.06336  2.48871  3.48536  2.98967  3.92986  4.63309  3.41630  0.56687
3.68546  3.79783  3.87635  5.81663  4.64340      16 R - - -
          2.68618  4.42225  2.77519  2.73123  3.46354  2.40513  3.72494
3.29354  2.67741  2.69355  4.24690  2.90347  2.73739  3.18146  2.89801
2.37887  2.77519  2.98518  4.58477  3.61503
          0.01212  4.81512  5.53746  0.61958  0.77255  0.48576  0.95510
     17   3.93570  5.88949  4.61254  3.65990  5.55546  4.39604  4.13666
4.80301  1.81723  4.10774  5.02562  3.93510  4.71790  3.26764  0.52525
3.91110  4.01880  4.51093  6.01818  4.99337      17 R - - -
          2.68618  4.42225  2.77519  2.73123  3.46354  2.40513  3.72494
3.29354  2.67741  2.69355  4.24690  2.90347  2.73739  3.18146  2.89801
2.37887  2.77519  2.98518  4.58477  3.61503
          0.01212  4.81512  5.53746  0.61958  0.77255  0.48576  0.95510
     18   2.22012  5.11392  3.06793  2.19835  4.38440  3.61421  3.81112
2.36432  2.37244  3.02370  4.17511  2.99559  4.00546  2.28849  2.77672
2.66157  3.03667  3.10446  5.59391  4.08586      18 e - - -
          2.68618  4.42225  2.77519  2.73123  3.46354  2.40513  3.72494
3.29354  2.67741  2.69355  4.24690  2.90347  2.73739  3.18146  2.89801
2.37887  2.77519  2.98518  4.58477  3.61503
          0.01212  4.81512  5.53746  0.61958  0.77255  0.48576  0.95510
```

```
     19   1.70141  5.17511  3.13475  2.50125  4.46606  3.61524  3.64490
3.91121  2.02325  3.45449  3.30960  3.10993  4.00633  2.82985  2.48056
2.60307  2.58599  3.53665  5.63959  4.27094     19 a - - -
          2.68618  4.42225  2.77519  2.73123  3.46354  2.40513  3.72494
3.29354  2.67741  2.69355  4.24690  2.90347  2.73739  3.18146  2.89801
2.37887  2.77519  2.98518  4.58477  3.61503
          0.01212  4.81512  5.53746  0.61958  0.77255  0.48576  0.95510
     20   4.42929  6.01337  5.02713  4.44544  5.63928  4.60490  4.97673
5.34744  3.22588  4.68534  5.72526  4.70283  5.12327  4.21411  0.18950
4.51656  4.67147  5.04026  6.37171  5.45790     20 R - - -
          2.68618  4.42225  2.77519  2.73123  3.46354  2.40513  3.72494
3.29354  2.67741  2.69355  4.24690  2.90347  2.73739  3.18146  2.89801
2.37887  2.77519  2.98518  4.58477  3.61503
          0.01212  4.81512  5.53746  0.61958  0.77255  0.48576  0.95510
     21   1.59627  5.28659  2.67688  2.07467  4.61287  3.46344  3.78521
4.08638  2.41499  3.05134  4.33338  3.06299  3.98735  2.40541  2.90628
2.68961  3.05130  3.66732  5.72992  4.32927     21 a - - -
          2.68618  4.42225  2.77519  2.73123  3.46354  2.40513  3.72494
3.29354  2.67741  2.69355  4.24690  2.90347  2.73739  3.18146  2.89801
2.37887  2.77519  2.98518  4.58477  3.61503
          0.01212  4.81512  5.53746  0.61958  0.77255  0.48576  0.95510
     22   2.40643  5.06667  3.19149  2.63100  3.78635  3.63633  3.69049
3.49903  1.61500  2.95456  3.91316  3.15447  4.02578  2.73043  2.11914
2.83841  3.04807  3.40821  5.55473  3.63119     22 k - - -
          2.68618  4.42225  2.77519  2.73123  3.46354  2.40513  3.72494
3.29354  2.67741  2.69355  4.24690  2.90347  2.73739  3.18146  2.89801
2.37887  2.77519  2.98518  4.58477  3.61503
          0.01929  4.81512  4.51004  0.61958  0.77255  0.48576  0.95510
     23   2.34090  4.36147  4.09238  3.51155  3.46498  3.89198  4.17767
2.43332  3.40895  1.32699  3.24060  3.79316  4.26307  2.78172  2.98255
3.16606  2.99192  2.57076  4.51213  3.76413     23 l - - -
          2.68618  4.42225  2.77519  2.73123  3.46354  2.40513  3.72494
3.29354  2.67741  2.69355  4.24690  2.90347  2.73739  3.18146  2.89801
2.37887  2.77519  2.98518  4.58477  3.61503
          0.02082  4.80803  4.38715  0.61958  0.77255  0.47563  0.97152
     24   2.55290  5.40386  2.23325  1.46123  4.74136  3.35885  3.65143
4.23123  2.49185  3.56047  4.45084  2.80784  4.01709  2.23128  3.09203
2.83496  3.11849  3.79126  5.83628  4.41457     24 e - - -
          2.68618  4.42225  2.77519  2.73123  3.46354  2.40513  3.72494
3.29354  2.67741  2.69355  4.24690  2.90347  2.73739  3.18146  2.89801
2.37887  2.77519  2.98518  4.58477  3.61503
          0.01222  4.80667  5.52901  0.61958  0.77255  0.50529  0.92466
     25   1.89468  5.15914  2.89378  2.07968  4.04385  3.48066  3.79527
3.89263  2.48286  2.59177  3.51045  2.85274  3.85017  2.73420  3.03879
2.46162  2.82080  3.51802  5.62847  4.25575     25 a - - -
          2.68646  4.42274  2.77539  2.73128  3.46356  2.40543  3.72544
3.29333  2.67654  2.69338  4.24739  2.90361  2.73743  3.18045  2.89752
2.37936  2.77535  2.98500  4.58526  3.61498
          0.25695  1.60955  3.62621  1.87205  0.16701  0.47374  0.97462
     26   2.58818  5.18906  2.95962  1.94455  4.09857  3.58848  2.89345
3.50967  2.45874  2.88795  3.40529  2.93728  3.98073  2.50546  2.58389
2.56224  2.93603  3.20869  5.65013  4.04648     35 e - - -
          2.68618  4.42225  2.77519  2.73123  3.46354  2.40513  3.72494
3.29354  2.67741  2.69355  4.24690  2.90347  2.73739  3.18146  2.89801
2.37887  2.77519  2.98518  4.58477  3.61503
          0.04528  4.79240  3.32490  0.61958  0.77255  0.45497  1.00640
```

```
     27   2.79379  4.99697  2.84509  2.56741  4.23565  2.48336  3.16967
3.04202  2.50554  2.34095  3.11434  2.61879  4.01454  2.97796  2.86170
2.82744  2.68722  3.32908  5.49914  3.86016      36 l - - -
          2.68618  4.42225  2.77519  2.73123  3.46354  2.40513  3.72494
3.29354  2.67741  2.69355  4.24690  2.90347  2.73739  3.18146  2.89801
2.37887  2.77519  2.98518  4.58477  3.61503
          0.01251  4.78339  5.50573  0.61958  0.77255  0.55641  0.85159
     28   2.23372  5.20980  3.11691  2.55566  4.51779  3.49723  3.49284
3.50722  1.75048  3.49450  4.26319  3.00894  3.99504  2.84676  1.96943
2.70723  2.74246  3.43962  5.66163  4.28695      37 k - - -
          2.68618  4.42225  2.77519  2.73123  3.46354  2.40513  3.72494
3.29354  2.67741  2.69355  4.24690  2.90347  2.73739  3.18146  2.89801
2.37887  2.77519  2.98518  4.58477  3.61503
          0.01251  4.78339  5.50573  0.61958  0.77255  0.55641  0.85159
     29   2.63190  4.51582  3.68413  2.96463  3.65187  3.63760  4.03469
2.54768  2.79840  1.73839  3.24768  3.21642  3.57711  3.37094  2.97882
2.59114  2.71105  2.35496  5.09826  3.86476      38 l - - -
          2.68604  4.42237  2.77532  2.73136  3.46366  2.40525  3.72483
3.29366  2.67672  2.69353  4.24682  2.90334  2.73752  3.18159  2.89813
2.37889  2.77521  2.98523  4.58489  3.61515
          0.53138  0.94721  3.71375  0.08985  2.45418  0.55641  0.85159
     30   2.59237  5.25175  2.59928  2.23460  4.57915  3.35957  3.57422
3.66316  2.38150  3.12609  3.88702  2.84467  2.75633  2.80263  2.34257
2.36594  2.97509  3.15268  5.69515  4.29513      40 e - - -
          2.68618  4.42225  2.77519  2.73123  3.46354  2.40513  3.72494
3.29354  2.67741  2.69355  4.24690  2.90347  2.73739  3.18146  2.89801
2.37887  2.77519  2.98518  4.58477  3.61503
          0.01277  4.76285  5.48520  0.61958  0.77255  0.59851  0.79769
     31   2.65954  5.22782  3.06162  2.16767  4.26359  3.56962  3.75931
3.70603  1.84618  3.06992  3.99786  2.80376  3.78529  2.65112  2.29174
2.67254  2.61053  3.32619  5.67691  4.28344      41 k - - -
          2.68618  4.42225  2.77519  2.73123  3.46354  2.40513  3.72494
3.29354  2.67741  2.69355  4.24690  2.90347  2.73739  3.18146  2.89801
2.37887  2.77519  2.98518  4.58477  3.61503
          0.01836  4.76285  4.64117  0.61958  0.77255  0.59851  0.79769
     32   2.17356  4.20344  3.07867  2.28952  4.48132  3.08082  3.59459
3.93722  2.44010  3.46669  4.23306  2.23254  3.83357  2.69827  2.82181
2.49768  2.58009  3.01812  5.64058  3.87959      42 a - - -
          2.68618  4.42225  2.77519  2.73123  3.46354  2.40513  3.72494
3.29354  2.67741  2.69355  4.24690  2.90347  2.73739  3.18146  2.89801
2.37887  2.77519  2.98518  4.58477  3.61503
          0.08115  4.75734  2.66856  0.61958  0.77255  0.58969  0.80856
     33   2.28418  5.30210  3.07778  2.22613  4.65235  3.27948  3.75890
4.11809  2.08389  3.59457  4.34864  2.77390  3.98696  2.63984  1.67800
2.80379  3.06375  3.69711  5.71793  4.33731      43 r - - -
          2.68646  4.42277  2.77442  2.73135  3.46345  2.40503  3.72547
3.29345  2.67699  2.69385  4.24742  2.90364  2.73727  3.17909  2.89832
2.37918  2.77554  2.98570  4.58529  3.61555
          0.27037  1.64611  3.12114  2.04427  0.13866  0.64736  0.74113
     34   3.17121  5.48303  3.35641  2.83143  4.92663  3.81497  3.89323
4.32606  0.99191  3.76343  4.57840  3.31187  3.87885  1.96996  2.60912
2.88759  3.37325  3.94647  5.82762  4.56314      55 k - - -
          2.68618  4.42225  2.77519  2.73123  3.46354  2.40513  3.72494
3.29354  2.67741  2.69355  4.24690  2.90347  2.73739  3.18146  2.89801
2.37887  2.77519  2.98518  4.58477  3.61503
          0.01391  4.67819  5.40054  0.61958  0.77255  0.49485  0.94074
```

35 2.68112 5.20948 2.92901 2.51964 4.51954 3.22587 3.76953
3.97983 1.91288 2.75576 4.26146 2.83089 2.05438 2.81523 2.87501
2.67296 2.86878 3.58157 5.66437 4.27783 56 k - - -
2.68618 4.42225 2.77519 2.73123 3.46354 2.40513 3.72494
3.29354 2.67741 2.69355 4.24690 2.90347 2.73739 3.18146 2.89801
2.37887 2.77519 2.98518 4.58477 3.61503
0.01279 4.76137 5.48372 0.61958 0.77255 0.41968 1.07078
36 2.76313 4.80401 4.49937 4.00997 2.61584 4.28914 4.10879
3.02321 3.89580 2.92541 3.94098 3.59712 4.66789 4.09041 4.08602
3.60183 3.56581 3.09393 4.48657 0.75141 57 y - - -
2.68618 4.42225 2.77519 2.73123 3.46354 2.40513 3.72494
3.29354 2.67741 2.69355 4.24690 2.90347 2.73739 3.18146 2.89801
2.37887 2.77519 2.98518 4.58477 3.61503
0.01212 4.81512 5.53746 0.61958 0.77255 0.48576 0.95510
37 2.84025 4.45073 3.92205 3.34541 3.56238 2.83716 4.13134
2.50005 2.39078 1.39622 2.82972 3.68823 4.23975 3.27762 3.52612
3.12960 3.07115 2.70501 5.04369 3.83218 58 l - - -
2.68618 4.42225 2.77519 2.73123 3.46354 2.40513 3.72494
3.29354 2.67741 2.69355 4.24690 2.90347 2.73739 3.18146 2.89801
2.37887 2.77519 2.98518 4.58477 3.61503
0.01212 4.81512 5.53746 0.61958 0.77255 0.48576 0.95510
38 2.93008 5.17775 3.16014 2.23387 4.26019 3.69103 1.45231
3.86355 2.65999 3.22032 4.24980 2.95384 4.10156 2.88959 3.09722
2.93965 3.16067 3.52782 5.54229 2.58815 59 h - - -
2.68618 4.42225 2.77519 2.73123 3.46354 2.40513 3.72494
3.29354 2.67741 2.69355 4.24690 2.90347 2.73739 3.18146 2.89801
2.37887 2.77519 2.98518 4.58477 3.61503
0.01212 4.81512 5.53746 0.61958 0.77255 0.48576 0.95510
39 2.90196 5.39735 2.89576 1.16074 4.72657 3.60543 3.48780
4.20849 2.42165 3.57940 4.44928 2.92641 3.32233 2.95284 3.10606
2.35912 3.13861 3.78094 5.83505 4.42092 60 e - - -
2.68630 4.42237 2.77531 2.73135 3.46366 2.40524 3.72506
3.29366 2.67752 2.69367 4.24701 2.90358 2.73660 3.18158 2.89812
2.37867 2.77531 2.98530 4.58489 3.61405
0.10326 2.36261 5.53746 1.18241 0.36606 0.48576 0.95510
40 3.00944 5.00020 3.63385 3.47351 5.05133 3.62568 4.68949
4.58049 2.97780 4.20241 5.05430 3.77415 4.35652 3.93069 3.78165
0.46775 3.47791 4.01696 6.30023 5.03849 65 S - - -
2.68618 4.42225 2.77519 2.73123 3.46354 2.40513 3.72494
3.29354 2.67741 2.69355 4.24690 2.90347 2.73739 3.18146 2.89801
2.37887 2.77519 2.98518 4.58477 3.61503
0.01212 4.81512 5.53746 0.61958 0.77255 0.48576 0.95510
41 3.84632 5.68800 4.61408 3.81882 4.91931 4.35708 4.34081
4.45355 2.53229 3.18040 4.90596 4.08797 4.76712 3.52463 0.42813
3.90457 4.03185 4.23048 5.92972 4.75679 66 R - - -
2.68618 4.42225 2.77519 2.73123 3.46354 2.40513 3.72494
3.29354 2.67741 2.69355 4.24690 2.90347 2.73739 3.18146 2.89801
2.37887 2.77519 2.98518 4.58477 3.61503
0.01212 4.81512 5.53746 0.61958 0.77255 0.48576 0.95510
42 3.06701 5.29429 3.19398 2.78974 4.60636 3.74393 1.08144
4.07516 2.66135 3.62462 4.44942 3.29546 2.79786 2.84762 2.92377
2.76653 3.31225 3.72236 5.77824 4.44467 67 h - - -
2.68618 4.42225 2.77519 2.73123 3.46354 2.40513 3.72494
3.29354 2.67741 2.69355 4.24690 2.90347 2.73739 3.18146 2.89801
2.37887 2.77519 2.98518 4.58477 3.61503
0.01212 4.81512 5.53746 0.61958 0.77255 0.48576 0.95510

```
     43   2.63741  5.27658  3.01158  2.45264  4.60543  3.59743  3.77997
3.87965  1.74197  2.69982  4.32279  2.88522  3.98941  2.17617  2.29622
2.79049  2.86192  3.57690  5.71605  4.32278      68 k - - -
          2.68618  4.42225  2.77519  2.73123  3.46354  2.40513  3.72494
3.29354  2.67741  2.69355  4.24690  2.90347  2.73739  3.18146  2.89801
2.37887  2.77519  2.98518  4.58477  3.61503
          0.01212  4.81512  5.53746  0.61958  0.77255  0.48576  0.95510
     44   3.42178  5.38632  3.75238  3.25836  3.49753  4.10885  0.85588
4.07458  2.80621  3.55037  4.50904  3.66798  4.50615  2.31805  3.07429
3.44937  3.63305  3.81315  4.04277  3.41113      69 h - - -
          2.68627  4.42234  2.77528  2.73132  3.46363  2.40489  3.72503
3.29283  2.67749  2.69364  4.24698  2.90355  2.73748  3.18084  2.89809
2.37895  2.77528  2.98527  4.58486  3.61512
          0.10326  2.36261  5.53746  0.95808  0.48391  0.48576  0.95510
     45   0.44211  2.47415  4.97130  4.76857  4.95636  3.49197  5.34815
4.13736  4.62429  4.04086  4.93513  4.30245  4.32598  4.80267  4.69174
2.98855  3.30688  3.61086  6.38978  5.29005      73 A - - -
          2.68618  4.42225  2.77519  2.73123  3.46354  2.40513  3.72494
3.29354  2.67741  2.69355  4.24690  2.90347  2.73739  3.18146  2.89801
2.37887  2.77519  2.98518  4.58477  3.61503
          0.01212  4.81512  5.53746  0.61958  0.77255  0.48576  0.95510
     46   2.82805  4.07891  4.28651  3.69954  3.40875  3.93102  4.23447
2.58033  2.86506  1.91494  1.65317  3.57108  4.30007  3.80080  3.75894
3.21720  2.48680  2.25551  4.92029  3.72848      74 m - - -
          2.68618  4.42225  2.77519  2.73123  3.46354  2.40513  3.72494
3.29354  2.67741  2.69355  4.24690  2.90347  2.73739  3.18146  2.89801
2.37887  2.77519  2.98518  4.58477  3.61503
          0.01212  4.81512  5.53746  0.61958  0.77255  0.48576  0.95510
     47   2.97589  5.39772  3.25161  2.67453  4.76987  3.32955  3.83914
4.21423  1.85763  3.41352  4.44821  1.99171  4.10324  2.80793  1.60703
2.75526  3.19171  3.80668  5.78423  4.44119      75 r - - -
          2.68618  4.42225  2.77519  2.73123  3.46354  2.40513  3.72494
3.29354  2.67741  2.69355  4.24690  2.90347  2.73739  3.18146  2.89801
2.37887  2.77519  2.98518  4.58477  3.61503
          0.01212  4.81512  5.53746  0.61958  0.77255  0.48576  0.95510
     48   4.42929  6.01337  5.02713  4.44544  5.63928  4.60490  4.97673
5.34744  3.22588  4.68534  5.72526  4.70283  5.12327  4.21411  0.18950
4.51656  4.67147  5.04026  6.37171  5.45790      76 R - - -
          2.68618  4.42225  2.77519  2.73123  3.46354  2.40513  3.72494
3.29354  2.67741  2.69355  4.24690  2.90347  2.73739  3.18146  2.89801
2.37887  2.77519  2.98518  4.58477  3.61503
          0.01212  4.81512  5.53746  0.61958  0.77255  0.48576  0.95510
     49   2.58381  4.88024  3.30262  2.49325  4.08516  3.67368  3.88556
2.64433  2.54034  2.69316  3.83746  3.24282  1.91096  2.89435  2.62933
2.88549  2.96564  2.94097  5.40683  4.10224      77 p - - -
          2.68618  4.42225  2.77519  2.73123  3.46354  2.40513  3.72494
3.29354  2.67741  2.69355  4.24690  2.90347  2.73739  3.18146  2.89801
2.37887  2.77519  2.98518  4.58477  3.61503
          0.01212  4.81512  5.53746  0.61958  0.77255  0.48576  0.95510
     50   4.42929  6.01337  5.02713  4.44544  5.63928  4.60490  4.97673
5.34744  3.22588  4.68534  5.72526  4.70283  5.12327  4.21411  0.18950
4.51656  4.67147  5.04026  6.37171  5.45790      78 R - - -
          2.68618  4.42225  2.77519  2.73123  3.46354  2.40513  3.72494
3.29354  2.67741  2.69355  4.24690  2.90347  2.73739  3.18146  2.89801
2.37887  2.77519  2.98518  4.58477  3.61503
          0.02226  4.81512  4.27545  0.61958  0.77255  0.48576  0.95510
```

```
      51   2.35676  5.08558  2.65595  2.69368  4.46554  1.24957  3.95794
3.89575  2.77761  3.33002  3.80074  2.91088  4.07495  3.10676  3.24964
2.82593  3.13091  3.52905  5.70787  4.35862     79 g - - -
          2.68618  4.42225  2.77519  2.73123  3.46354  2.40513  3.72494
3.29354  2.67741  2.69355  4.24690  2.90347  2.73739  3.18146  2.89801
2.37887  2.77519  2.98518  4.58477  3.61503
          0.01224  4.80510  5.52744  0.61958  0.77255  0.47159  0.97820
      52   2.70372  4.13416  3.04312  2.17723  4.54636  3.59176  3.60701
4.00955  2.36991  3.43155  4.28400  2.84334  1.93412  2.83395  2.96601
2.18777  2.75552  3.60704  5.68707  4.29715     80 p - - -
          2.68618  4.42225  2.77519  2.73123  3.46354  2.40513  3.72494
3.29354  2.67741  2.69355  4.24690  2.90347  2.73739  3.18146  2.89801
2.37887  2.77519  2.98518  4.58477  3.61503
          0.01212  4.81512  5.53746  0.61958  0.77255  0.48576  0.95510
      53   2.86648  5.36631  2.37581  2.51079  4.70162  1.80081  3.42618
4.18510  2.23593  3.66840  4.41601  2.32399  4.01224  2.92213  3.01451
2.42951  2.95597  3.75303  5.80497  4.39132     81 g - - -
          2.68618  4.42225  2.77519  2.73123  3.46354  2.40513  3.72494
3.29354  2.67741  2.69355  4.24690  2.90347  2.73739  3.18146  2.89801
2.37887  2.77519  2.98518  4.58477  3.61503
          0.01212  4.81512  5.53746  0.61958  0.77255  0.48576  0.95510
      54   4.18040  5.77045  5.01670  5.05113  6.01114  0.10436  6.00694
5.92994  5.33751  5.40701  6.45199  5.18500  5.16755  5.59123  5.38544
4.38977  4.71899  5.28747  6.77565  6.17198     82 G - - -
          2.68618  4.42225  2.77519  2.73123  3.46354  2.40513  3.72494
3.29354  2.67741  2.69355  4.24690  2.90347  2.73739  3.18146  2.89801
2.37887  2.77519  2.98518  4.58477  3.61503
          0.03069  4.81512  3.81154  0.61958  0.77255  0.48576  0.95510
      55   3.86375  5.87277  4.51640  3.55649  5.52988  4.35524  4.06403
4.75105  1.70491  4.05094  4.95440  3.85035  4.65826  2.89906  0.61720
3.82733  3.93861  4.45519  5.97565  4.93815     83 R - - -
          2.68618  4.42225  2.77519  2.73123  3.46354  2.40513  3.72494
3.29354  2.67741  2.69355  4.24690  2.90347  2.73739  3.18146  2.89801
2.37887  2.77519  2.98518  4.58477  3.61503
          0.01234  4.79677  5.51912  0.61958  0.77255  0.52749  0.89181
      56   4.76896  5.74418  5.61717  5.53759  0.37436  5.27640  4.00382
4.27671  5.32541  3.42203  4.81211  4.86377  5.54818  5.02314  5.15094
4.70828  4.97698  4.26620  4.08119  1.97122     84 F - - -
          2.68618  4.42225  2.77519  2.73123  3.46354  2.40513  3.72494
3.29354  2.67741  2.69355  4.24690  2.90347  2.73739  3.18146  2.89801
2.37887  2.77519  2.98518  4.58477  3.61503
          0.00832  4.79275        *  0.61958  0.77255  0.00000        *
//
HMMER3/f [3.3 | Nov 2019]
NAME  CCT
ACC   PF06203.15
DESC  CCT motif
LENG  44
ALPH  amino
RF    no
MM    no
CONS  yes
CS    no
MAP   yes
DATE  Wed Apr 22 22:54:58 2020
NSEQ  88
EFFN  4.670166
CKSUM 2217445830
```

```
GA    28.40 28.40
TC    28.40 28.40
NC    28.30 28.10
STATS LOCAL MSV       -7.8867  0.71935
STATS LOCAL VITERBI   -8.1313  0.71935
STATS LOCAL FORWARD   -4.0698  0.71935
HMM          A        C        D        E        F        G        H
I        K        L        M        N        P        Q        R        S
T        V        W        Y
            m->m     m->i     m->d     i->m     i->i     d->m     d->d
  COMPO   2.59120  4.67018  3.40169  2.81816  3.11482  3.21545  4.01986
3.39319  2.00787  3.09307  4.09849  3.25421  3.98478  3.03999  1.69076
2.94495  3.20797  2.90668  4.89206  3.08517
          2.68618  4.42225  2.77519  2.73123  3.46354  2.40513  3.72494
3.29354  2.67741  2.69355  4.24690  2.90347  2.73739  3.18146  2.89801
2.37887  2.77519  2.98518  4.58477  3.61503
          0.00753  5.28844  6.01079  0.61958  0.77255  0.00000        *
      1   4.54511  6.36682  5.26667  4.32898  6.10417  4.89379  4.72485
5.43483  2.62008  4.70655  5.65820  4.55675  5.26324  3.87074  0.23180
4.54079  4.64467  5.13976  6.53654  5.56470      1 R - - -
          2.68618  4.42225  2.77519  2.73123  3.46354  2.40513  3.72494
3.29354  2.67741  2.69355  4.24690  2.90347  2.73739  3.18146  2.89801
2.37887  2.77519  2.98518  4.58477  3.61503
          0.02032  5.28844  4.19519  0.61958  0.77255  0.48576  0.95510
      2   2.03342  5.50574  2.91697  1.76152  3.97507  3.56334  3.75141
3.94925  2.20223  3.38719  4.25824  2.81480  4.16700  2.37004  2.43453
2.87228  3.23031  3.70016  5.93944  4.52953      2 e - - -
          2.68618  4.42225  2.77519  2.73123  3.46354  2.40513  3.72494
3.29354  2.67741  2.69355  4.24690  2.90347  2.73739  3.18146  2.89801
2.37887  2.77519  2.98518  4.58477  3.61503
          0.00763  5.27575  5.99809  0.61958  0.77255  0.45782  1.00148
      3   1.78315  4.87310  3.07632  1.50319  4.83486  3.78271  3.97063
4.11058  2.54318  3.34559  4.54049  3.07009  4.17605  2.22997  2.98715
2.77060  3.08627  3.74090  5.93514  4.52907      3 e - - -
          2.68618  4.42225  2.77519  2.73123  3.46354  2.40513  3.72494
3.29354  2.67741  2.69355  4.24690  2.90347  2.73739  3.18146  2.89801
2.37887  2.77519  2.98518  4.58477  3.61503
          0.00753  5.28844  6.01079  0.61958  0.77255  0.48576  0.95510
      4   1.86277  5.46079  3.30746  2.74806  4.78074  3.80894  3.99229
3.99474  1.72858  3.75216  4.24293  3.28864  4.20053  2.48561  1.86467
2.56208  3.25596  3.45516  5.90600  4.10206      4 k - - -
          2.68618  4.42225  2.77519  2.73123  3.46354  2.40513  3.72494
3.29354  2.67741  2.69355  4.24690  2.90347  2.73739  3.18146  2.89801
2.37887  2.77519  2.98518  4.58477  3.61503
          0.00753  5.28844  6.01079  0.61958  0.77255  0.48576  0.95510
      5   3.54120  4.85628  5.48761  4.91003  3.92937  4.83113  5.22431
1.51854  2.90692  1.35578  3.41556  4.97074  5.13792  4.88598  4.78943
4.16745  3.77796  1.29865  5.66731  4.50623      5 v - - -
          2.68618  4.42225  2.77519  2.73123  3.46354  2.40513  3.72494
3.29354  2.67741  2.69355  4.24690  2.90347  2.73739  3.18146  2.89801
2.37887  2.77519  2.98518  4.58477  3.61503
          0.00753  5.28844  6.01079  0.61958  0.77255  0.48576  0.95510
      6   2.53486  5.50441  2.65378  2.27349  4.84455  3.05406  3.81690
3.95638  2.30153  2.80353  2.98096  2.64698  4.17445  2.44001  2.46766
2.70108  2.73249  3.51678  5.32372  4.53209      6 e - - -
          2.68618  4.42225  2.77519  2.73123  3.46354  2.40513  3.72494
3.29354  2.67741  2.69355  4.24690  2.90347  2.73739  3.18146  2.89801
2.37887  2.77519  2.98518  4.58477  3.61503
```

```
          0.00753  5.28844  6.01079  0.61958  0.77255  0.48576  0.95510
      7   3.65362  6.14199  4.69005  3.80793  5.79525  4.61882  4.40412
5.04190  1.79989  4.36904  5.25343  3.75680  4.94512  3.52731  0.51268
4.05039  3.39181  4.72214  6.31758  5.24821      7 R - - -
          2.68618  4.42225  2.77519  2.73123  3.46354  2.40513  3.72494
3.29354  2.67741  2.69355  4.24690  2.90347  2.73739  3.18146  2.89801
2.37887  2.77519  2.98518  4.58477  3.61503
          0.00753  5.28844  6.01079  0.61958  0.77255  0.48576  0.95510
      8   5.56734  6.33871  6.22317  6.27597  1.66212  5.87966  4.37342
5.08259  6.03062  4.13408  5.56399  5.34140  6.11055  5.55183  5.74125
5.30221  5.73988  5.05575  2.72123  0.41175      8 Y - - -
          2.68618  4.42225  2.77519  2.73123  3.46354  2.40513  3.72494
3.29354  2.67741  2.69355  4.24690  2.90347  2.73739  3.18146  2.89801
2.37887  2.77519  2.98518  4.58477  3.61503
          0.00753  5.28844  6.01079  0.61958  0.77255  0.48576  0.95510
      9   3.07670  5.35738  3.43643  2.86678  3.62865  3.87952  3.33487
4.06046  1.62629  2.34594  4.17658  2.99907  4.26622  3.00089  1.54427
3.08604  3.30289  3.71157  4.96624  4.48034      9 r - - -
          2.68618  4.42225  2.77519  2.73123  3.46354  2.40513  3.72494
3.29354  2.67741  2.69355  4.24690  2.90347  2.73739  3.18146  2.89801
2.37887  2.77519  2.98518  4.58477  3.61503
          0.00753  5.28844  6.01079  0.61958  0.77255  0.48576  0.95510
     10   2.32632  5.51289  2.89610  1.39936  4.85651  3.77981  3.96711
3.73030  2.39622  3.65653  4.26707  2.99680  4.17334  2.28707  3.04285
2.35250  3.02333  3.90275  5.94642  4.53635     10 e - - -
          2.68618  4.42225  2.77519  2.73123  3.46354  2.40513  3.72494
3.29354  2.67741  2.69355  4.24690  2.90347  2.73739  3.18146  2.89801
2.37887  2.77519  2.98518  4.58477  3.61503
          0.00753  5.28844  6.01079  0.61958  0.77255  0.48576  0.95510
     11   4.47710  6.36047  5.03132  4.19078  6.08697  4.84201  4.66391
5.38831  0.23815  4.66270  5.60247  4.45549  5.20667  3.80445  2.70415
4.45641  4.57352  5.08933  6.51264  5.52232     11 K - - -
          2.68618  4.42225  2.77519  2.73123  3.46354  2.40513  3.72494
3.29354  2.67741  2.69355  4.24690  2.90347  2.73739  3.18146  2.89801
2.37887  2.77519  2.98518  4.58477  3.61503
          0.00753  5.28844  6.01079  0.61958  0.77255  0.48576  0.95510
     12   4.33245  6.30594  5.08293  4.01624  6.03597  4.78548  4.44481
5.21333  1.22472  4.47531  5.38858  3.41061  5.07494  3.56790  0.66217
4.28809  4.38264  4.92348  6.37064  5.36604     12 R - - -
          2.68618  4.42225  2.77519  2.73123  3.46354  2.40513  3.72494
3.29354  2.67741  2.69355  4.24690  2.90347  2.73739  3.18146  2.89801
2.37887  2.77519  2.98518  4.58477  3.61503
          0.02070  5.28844  4.17092  0.61958  0.77255  0.48576  0.95510
     13   2.93510  4.94747  3.12433  2.35105  4.85126  3.61292  3.55953
4.33633  1.47603  3.30896  3.60559  2.66493  4.16701  2.39050  2.29629
2.63851  3.00678  3.89732  5.94046  4.53037     13 k - - -
          2.68618  4.42225  2.77519  2.73123  3.46354  2.40513  3.72494
3.29354  2.67741  2.69355  4.24690  2.90347  2.73739  3.18146  2.89801
2.37887  2.77519  2.98518  4.58477  3.61503
          0.00763  5.27537  5.99772  0.61958  0.77255  0.53347  0.88326
     14   2.71448  5.50877  3.16175  2.23762  4.85336  3.31460  3.43734
4.33888  2.27624  3.14764  4.54959  2.32722  4.16681  2.75097  1.97366
2.61851  2.18345  3.89916  5.94165  4.53115     14 r - - -
          2.68618  4.42225  2.77519  2.73123  3.46354  2.40513  3.72494
3.29354  2.67741  2.69355  4.24690  2.90347  2.73739  3.18146  2.89801
2.37887  2.77519  2.98518  4.58477  3.61503
          0.00763  5.27537  5.99772  0.61958  0.77255  0.45705  1.00281
```

```
     15   4.13024  5.91940  5.13200  4.18296  4.97251  4.74669  4.61561
3.45009  2.78441  3.33821  4.90458  4.41892  5.10418  3.79832  0.35006
4.22885  4.29465  4.15441  6.16053  4.96736      15 R - - -
         2.68622  4.42229  2.77524  2.73127  3.46358  2.40517  3.72499
3.29358  2.67745  2.69328  4.24694  2.90351  2.73744  3.18150  2.89766
2.37891  2.77524  2.98522  4.58481  3.61507
         0.04714  3.13285  6.01079  0.66274  0.72451  0.48576  0.95510
     16   2.92299  2.50997  3.77418  3.20986  3.25406  3.80071  3.58646
3.13613  2.20785  2.35487  2.86409  2.36377  4.35662  3.19131  3.10096
2.63293  2.90736  2.68595  5.41659  4.16408      18 k - - -
         2.68618  4.42225  2.77519  2.73123  3.46354  2.40513  3.72494
3.29354  2.67741  2.69355  4.24690  2.90347  2.73739  3.18146  2.89801
2.37887  2.77519  2.98518  4.58477  3.61503
         0.00753  5.28844  6.01079  0.61958  0.77255  0.48576  0.95510
     17   3.75906  5.08371  5.39188  4.90641  0.82481  4.79991  4.44736
3.55653  4.70639  2.75543  3.69339  4.75372  5.13810  4.75018  3.36372
3.72408  3.98551  3.39008  2.05711  2.18703      19 f - - -
         2.68591  4.42253  2.77548  2.73123  3.46382  2.40494  3.72523
3.29382  2.67729  2.69328  4.24718  2.90354  2.73725  3.18108  2.89829
2.37885  2.77504  2.98547  4.58505  3.61531
         0.18060  2.02488  3.40456  1.30097  0.31782  0.48576  0.95510
     18   2.22910  5.49141  2.21672  2.12922  4.32903  2.81623  3.15801
3.49277  2.28040  3.51423  4.06507  2.77742  4.15900  2.82523  3.11428
2.44427  3.08488  3.28043  5.92652  4.51801      25 e - - -
         2.68618  4.42225  2.77519  2.73123  3.46354  2.40513  3.72494
3.29354  2.67741  2.69355  4.24690  2.90347  2.73739  3.18146  2.89801
2.37887  2.77519  2.98518  4.58477  3.61503
         0.00777  5.25719  5.97954  0.61958  0.77255  0.42289  1.06465
     19   3.22159  5.66875  3.27268  2.90411  5.05039  3.95595  4.07952
4.50087  0.86641  3.95263  4.71631  3.13590  3.07129  2.93385  2.26226
3.18368  3.10366  4.08255  6.04806  4.69875      26 k - - -
         2.68618  4.42225  2.77519  2.73123  3.46354  2.40513  3.72494
3.29354  2.67741  2.69355  4.24690  2.90347  2.73739  3.18146  2.89801
2.37887  2.77519  2.98518  4.58477  3.61503
         0.00753  5.28844  6.01079  0.61958  0.77255  0.48576  0.95510
     20   2.89783  5.49626  3.26881  2.59387  4.83240  3.78699  3.62718
4.31090  1.49855  3.44515  4.25556  3.25830  3.31628  2.54655  2.13575
2.67251  2.33687  3.28601  5.93362  4.52991      27 k - - -
         2.68618  4.42225  2.77519  2.73123  3.46354  2.40513  3.72494
3.29354  2.67741  2.69355  4.24690  2.90347  2.73739  3.18146  2.89801
2.37887  2.77519  2.98518  4.58477  3.61503
         0.00753  5.28844  6.01079  0.61958  0.77255  0.48576  0.95510
     21   2.50601  4.53531  4.99113  4.39286  3.65416  4.32800  3.72125
1.18480  4.20918  2.26502  3.66032  3.88803  4.68859  4.37372  4.27059
3.63896  3.38604  1.44733  5.20295  4.02094      28 i - - -
         2.68588  4.42230  2.77524  2.73128  3.46358  2.40517  3.72499
3.29359  2.67745  2.69359  4.24694  2.90351  2.73744  3.18151  2.89805
2.37866  2.77524  2.98523  4.58481  3.61508
         0.10598  3.03649  2.94582  0.66465  0.72248  0.48576  0.95510
     22   3.01066  5.47728  3.01245  2.71776  4.81285  3.78602  3.37093
4.28389  2.04867  3.19467  4.26158  3.25973  4.17746  2.96232  1.29691
2.41061  2.72981  3.86326  5.91095  4.51792      31 r - - -
         2.68618  4.42225  2.77519  2.73123  3.46354  2.40513  3.72494
3.29354  2.67741  2.69355  4.24690  2.90347  2.73739  3.18146  2.89801
2.37887  2.77519  2.98518  4.58477  3.61503
         0.00793  5.23704  5.95938  0.61958  0.77255  0.39118  1.12782
```

```
     23   4.37204  5.90199  5.20997  5.15634  3.14965  4.92693  4.63620
4.96001  5.12295  4.23345  5.50124  4.97349  5.52509  5.21289  5.17477
2.85611  4.79221  4.73473  4.81501  0.25185      32 Y - - -
          2.68618  4.42225  2.77519  2.73123  3.46354  2.40513  3.72494
3.29354  2.67741  2.69355  4.24690  2.90347  2.73739  3.18146  2.89801
2.37887  2.77519  2.98518  4.58477  3.61503
          0.00753  5.28844  6.01079  0.61958  0.77255  0.48576  0.95510
     24   2.03825  5.51685  2.41102  1.63988  4.86136  3.46007  3.63223
4.10510  2.70322  3.82191  4.55775  2.58890  3.67170  2.38164  3.19613
2.58353  3.06991  3.45949  5.94982  4.53895      33 e - - -
          2.68618  4.42225  2.77519  2.73123  3.46354  2.40513  3.72494
3.29354  2.67741  2.69355  4.24690  2.90347  2.73739  3.18146  2.89801
2.37887  2.77519  2.98518  4.58477  3.61503
          0.00753  5.28844  6.01079  0.61958  0.77255  0.48576  0.95510
     25   2.69121  2.07455  4.43015  3.84768  3.66740  3.45601  4.44087
2.85828  3.60123  2.69533  3.67452  3.72985  4.51098  3.97538  3.95047
1.75964  2.72763  1.69447  5.16926  3.60992      34 v - - -
          2.68618  4.42225  2.77519  2.73123  3.46354  2.40513  3.72494
3.29354  2.67741  2.69355  4.24690  2.90347  2.73739  3.18146  2.89801
2.37887  2.77519  2.98518  4.58477  3.61503
          0.00753  5.28844  6.01079  0.61958  0.77255  0.48576  0.95510
     26   5.29767  6.67668  5.89031  5.34712  6.44439  5.26133  5.79145
6.29488  4.06078  5.54940  6.62685  5.57212  5.80692  5.07301  0.07758
5.41245  5.54893  5.96862  7.03636  6.28386      35 R - - -
          2.68618  4.42225  2.77519  2.73123  3.46354  2.40513  3.72494
3.29354  2.67741  2.69355  4.24690  2.90347  2.73739  3.18146  2.89801
2.37887  2.77519  2.98518  4.58477  3.61503
          0.00753  5.28844  6.01079  0.61958  0.77255  0.48576  0.95510
     27   3.10640  5.92851  4.01840  3.32530  5.42066  4.29378  4.24188
4.78397  0.83348  4.17032  4.98783  3.77226  4.64512  2.15411  1.83399
3.01370  3.80029  4.40863  6.18745  4.97375      36 k - - -
          2.68618  4.42225  2.77519  2.73123  3.46354  2.40513  3.72494
3.29354  2.67741  2.69355  4.24690  2.90347  2.73739  3.18146  2.89801
2.37887  2.77519  2.98518  4.58477  3.61503
          0.00753  5.28844  6.01079  0.61958  0.77255  0.48576  0.95510
     28   2.04465  5.46267  3.27777  2.41012  4.53032  3.78938  3.77925
3.73344  2.10133  2.81280  4.50926  2.62017  4.18229  2.45014  2.46773
2.82803  2.49786  2.97307  5.90893  4.51193      37 a - - -
          2.68618  4.42225  2.77519  2.73123  3.46354  2.40513  3.72494
3.29354  2.67741  2.69355  4.24690  2.90347  2.73739  3.18146  2.89801
2.37887  2.77519  2.98518  4.58477  3.61503
          0.00753  5.28844  6.01079  0.61958  0.77255  0.48576  0.95510
     29   2.91006  4.15260  4.47697  3.89147  3.24240  3.93506  4.44855
2.86563  3.11238  1.58428  3.05462  2.69795  4.51868  4.00379  2.81770
2.80937  2.90718  1.85481  5.15025  3.20290      38 l - - -
          2.68618  4.42225  2.77519  2.73123  3.46354  2.40513  3.72494
3.29354  2.67741  2.69355  4.24690  2.90347  2.73739  3.18146  2.89801
2.37887  2.77519  2.98518  4.58477  3.61503
          0.00753  5.28844  6.01079  0.61958  0.77255  0.48576  0.95510
     30   0.27875  5.22998  3.25359  4.04502  5.70769  3.88231  5.40675
5.17935  4.58977  4.86063  5.66611  4.24173  4.70101  4.69802  4.93055
3.02638  3.79451  4.44719  7.03281  5.81303      39 A - - -
          2.68618  4.42225  2.77519  2.73123  3.46354  2.40513  3.72494
3.29354  2.67741  2.69355  4.24690  2.90347  2.73739  3.18146  2.89801
2.37887  2.77519  2.98518  4.58477  3.61503
          0.00753  5.28844  6.01079  0.61958  0.77255  0.48576  0.95510
```

```
     31   2.53142  5.51189  1.54391  1.98749  4.84982  3.59063  3.77273
4.33159  2.63237  3.14816  4.55481  2.60197  4.18227  2.74319  3.21059
2.84637  3.24732  2.73909  5.94847  4.54049      40 d - - -
          2.68618  4.42225  2.77519  2.73123  3.46354  2.40513  3.72494
3.29354  2.67741  2.69355  4.24690  2.90347  2.73739  3.18146  2.89801
2.37887  2.77519  2.98518  4.58477  3.61503
          0.00753  5.28844  6.01079  0.61958  0.77255  0.48576  0.95510
     32   2.61296  4.83454  3.19983  2.59530  4.27560  3.37152  3.97635
4.04500  1.76729  3.61619  4.02940  3.02800  4.18033  2.71401  2.56144
2.01179  2.17200  3.35581  5.91621  4.51662      41 k - - -
          2.68618  4.42225  2.77519  2.73123  3.46354  2.40513  3.72494
3.29354  2.67741  2.69355  4.24690  2.90347  2.73739  3.18146  2.89801
2.37887  2.77519  2.98518  4.58477  3.61503
          0.00753  5.28844  6.01079  0.61958  0.77255  0.48576  0.95510
     33   3.79948  4.67544  4.45068  3.63606  5.22912  4.47152  4.34178
4.54395  2.31848  4.02091  2.75841  4.00353  4.80087  3.01967  0.56156
3.82425  3.94742  4.25797  6.10807  4.96734      42 R - - -
          2.68618  4.42225  2.77519  2.73123  3.46354  2.40513  3.72494
3.29354  2.67741  2.69355  4.24690  2.90347  2.73739  3.18146  2.89801
2.37887  2.77519  2.98518  4.58477  3.61503
          0.00753  5.28844  6.01079  0.61958  0.77255  0.48576  0.95510
     34   2.94197  5.39200  3.32582  2.62968  4.68654  3.81316  3.79297
4.13624  2.05823  2.71221  3.90193  3.16208  1.56116  2.47214  2.86812
3.00834  3.17640  2.96014  5.85482  4.48007      43 p - - -
          2.68618  4.42225  2.77519  2.73123  3.46354  2.40513  3.72494
3.29354  2.67741  2.69355  4.24690  2.90347  2.73739  3.18146  2.89801
2.37887  2.77519  2.98518  4.58477  3.61503
          0.00753  5.28844  6.01079  0.61958  0.77255  0.48576  0.95510
     35   5.29767  6.67668  5.89031  5.34712  6.44439  5.26133  5.79145
6.29488  4.06078  5.54940  6.62685  5.57212  5.80692  5.07301  0.07758
5.41245  5.54893  5.96862  7.03636  6.28386      44 R - - -
          2.68618  4.42225  2.77519  2.73123  3.46354  2.40513  3.72494
3.29354  2.67741  2.69355  4.24690  2.90347  2.73739  3.18146  2.89801
2.37887  2.77519  2.98518  4.58477  3.61503
          0.00753  5.28844  6.01079  0.61958  0.77255  0.48576  0.95510
     36   3.05800  4.47845  4.75978  3.73261  3.00039  4.19593  3.17958
1.99083  3.99938  2.36231  3.04300  3.39048  4.56240  4.17900  3.54104
3.00073  3.29011  1.18941  5.09569  3.91270      45 v - - -
          2.68618  4.42225  2.77519  2.73123  3.46354  2.40513  3.72494
3.29354  2.67741  2.69355  4.24690  2.90347  2.73739  3.18146  2.89801
2.37887  2.77519  2.98518  4.58477  3.61503
          0.00753  5.28844  6.01079  0.61958  0.77255  0.48576  0.95510
     37   4.11433  6.20692  4.69146  3.79363  5.85480  3.86028  4.38872
5.09230  0.58466  4.39209  5.27524  3.80838  4.95161  2.95832  1.58902
4.07061  4.20780  4.77433  6.32350  5.25699      46 K - - -
          2.68618  4.42225  2.77519  2.73123  3.46354  2.40513  3.72494
3.29354  2.67741  2.69355  4.24690  2.90347  2.73739  3.18146  2.89801
2.37887  2.77519  2.98518  4.58477  3.61503
          0.00753  5.28844  6.01079  0.61958  0.77255  0.48576  0.95510
     38   3.50422  5.37386  5.16550  5.16841  5.83527  0.19166  6.02388
5.29151  5.28942  5.07303  5.98671  4.91332  4.98839  5.48398  5.36232
3.72001  4.08179  3.30037  7.11863  6.07155      47 G - - -
          2.68618  4.42225  2.77519  2.73123  3.46354  2.40513  3.72494
3.29354  2.67741  2.69355  4.24690  2.90347  2.73739  3.18146  2.89801
2.37887  2.77519  2.98518  4.58477  3.61503
          0.00753  5.28844  6.01079  0.61958  0.77255  0.48576  0.95510
```

```
     39   3.84393  6.03861  4.29249  3.54186  5.58767  3.53566  4.32310
4.89991  2.20578  4.26264  5.11266  3.94087  4.79173  2.20531  0.62985
3.80986  3.25922  4.55400  6.25051  5.10366     48 R - - -
          2.68618  4.42225  2.77519  2.73123  3.46354  2.40513  3.72494
3.29354  2.67741  2.69355  4.24690  2.90347  2.73739  3.18146  2.89801
2.37887  2.77519  2.98518  4.58477  3.61503
          0.00753  5.28844  6.01079  0.61958  0.77255  0.48576  0.95510
     40   4.73844  6.08176  5.45750  5.43449  0.20724  5.21414  4.67997
4.87300  5.46631  4.11584  5.45603  3.46607  5.73643  5.41325  5.44614
4.80134  5.10509  4.75715  4.81422  3.07667     49 F - - -
          2.68618  4.42225  2.77519  2.73123  3.46354  2.40513  3.72494
3.29354  2.67741  2.69355  4.24690  2.90347  2.73739  3.18146  2.89801
2.37887  2.77519  2.98518  4.58477  3.61503
          0.00753  5.28844  6.01079  0.61958  0.77255  0.48576  0.95510
     41   1.67014  4.68537  5.23303  4.65267  3.87361  4.56638  4.96565
1.85403  4.48421  2.25747  3.83621  4.70524  4.91944  4.65468  4.55140
3.58569  2.96742  1.13306  5.48052  4.29369     50 v - - -
          2.68618  4.42225  2.77519  2.73123  3.46354  2.40513  3.72494
3.29354  2.67741  2.69355  4.24690  2.90347  2.73739  3.18146  2.89801
2.37887  2.77519  2.98518  4.58477  3.61503
          0.06379  5.28844  2.86918  0.61958  0.77255  0.48576  0.95510
     42   2.95460  5.51516  3.33626  2.77070  4.87107  3.62653  3.82477
4.33542  1.20948  3.81579  4.56971  3.30537  3.61998  3.09566  2.19476
2.03354  2.78381  3.91530  5.94189  4.56356     51 k - - -
          2.68618  4.42225  2.77519  2.73123  3.46354  2.40513  3.72494
3.29354  2.67741  2.69355  4.24690  2.90347  2.73739  3.18146  2.89801
2.37887  2.77519  2.98518  4.58477  3.61503
          0.08311  5.23262  2.59812  0.61958  0.77255  0.67165  0.71512
     43   2.10934  5.44990  2.66244  2.26079  4.79292  3.71863  3.90590
4.08719  2.17142  3.75430  3.98899  2.38437  3.87532  2.53483  2.61259
2.55258  2.91398  2.76326  5.88380  4.47410     52 a - - -
          2.68618  4.42225  2.77519  2.73123  3.46354  2.40513  3.72494
3.29354  2.67741  2.69355  4.24690  2.90347  2.73739  3.18146  2.89801
2.37887  2.77519  2.98518  4.58477  3.61503
          0.21155  5.15809  1.68786  0.61958  0.77255  0.86409  0.54720
     44   2.67562  5.37169  2.35230  1.89810  4.71636  2.92559  3.82136
4.20235  2.19130  3.67681  4.41263  2.49694  3.13965  2.85215  2.96007
2.37221  2.84547  3.47627  5.80449  4.39326     53 e - - -
          2.68618  4.42225  2.77519  2.73123  3.46354  2.40513  3.72494
3.29354  2.67741  2.69355  4.24690  2.90347  2.73739  3.18146  2.89801
2.37887  2.77519  2.98518  4.58477  3.61503
          0.00708  4.95363        *  0.61958  0.77255  0.00000        *
//
HMMER3/f [3.3 | Nov 2019]
NAME  CG-1
ACC   PF03859.17
DESC  CG-1 domain
LENG  116
ALPH  amino
RF    no
MM    no
CONS  yes
CS    no
MAP   yes
DATE  Thu Apr 16 22:20:22 2020
NSEQ  27
EFFN  1.196411
CKSUM 2500231289
```

```
GA    23.50 23.50
TC    23.90 23.50
NC    22.90 23.40
STATS LOCAL MSV       -9.8408  0.71419
STATS LOCAL VITERBI  -10.5243  0.71419
STATS LOCAL FORWARD   -4.5406  0.71419
HMM          A        C        D        E        F        G        H
I        K        L        M        N        P        Q        R        S
T        V        W        Y
            m->m     m->i     m->d     i->m     i->i     d->m     d->d
  COMPO   2.71221  4.37504  2.97410  2.60820  3.30487  3.11910  3.46302
2.89829  2.48548  2.48827  3.75714  3.02664  3.46042  2.99295  2.67614
2.76108  2.94613  2.73486  4.03665  3.16935
          2.68618  4.42225  2.77519  2.73123  3.46354  2.40513  3.72494
3.29354  2.67741  2.69355  4.24690  2.90347  2.73739  3.18146  2.89801
2.37887  2.77519  2.98518  4.58477  3.61503
          0.13640  4.29155  2.17312  0.61958  0.77255  0.00000        *
      1   2.71430  5.18416  2.52193  1.71114  4.49792  3.37568  3.64863
3.97287  2.24729  3.47521  4.24555  2.59851  3.82768  2.67606  2.93580
2.56703  2.47159  3.56024  5.62986  4.21749      1 e - - -
          2.68618  4.42225  2.77519  2.73123  3.46354  2.40513  3.72494
3.29354  2.67741  2.69355  4.24690  2.90347  2.73739  3.18146  2.89801
2.37887  2.77519  2.98518  4.58477  3.61503
          0.02303  4.17818  4.90053  0.61958  0.77255  0.55482  0.85373
      2   1.69710  4.43615  3.34842  2.79440  3.58347  3.54581  3.43742
2.71897  2.74288  2.66250  3.54475  3.23269  3.93718  2.94184  3.12176
2.70717  2.85230  2.65720  5.00029  3.38773      2 a - - -
          2.68618  4.42225  2.77519  2.73123  3.46354  2.40513  3.72494
3.29354  2.67741  2.69355  4.24690  2.90347  2.73739  3.18146  2.89801
2.37887  2.77519  2.98518  4.58477  3.61503
          0.02223  4.21337  4.93571  0.61958  0.77255  0.42832  1.05442
      3   2.62060  4.38226  3.02510  2.45638  4.33427  3.47764  3.64642
3.76928  1.75530  3.30571  4.09107  2.97924  3.86776  2.42083  2.41879
2.44905  2.63905  3.39923  5.47935  4.13124      3 k - - -
          2.68618  4.42225  2.77519  2.73123  3.46354  2.40513  3.72494
3.29354  2.67741  2.69355  4.24690  2.90347  2.73739  3.18146  2.89801
2.37887  2.77519  2.98518  4.58477  3.61503
          0.02054  4.29155  5.01389  0.61958  0.77255  0.48576  0.95510
      4   2.47015  4.94941  2.79703  2.41889  4.22080  3.44595  3.25935
3.09493  2.37187  3.22245  4.01054  2.64989  3.83764  2.59139  2.66328
2.48756  2.40586  3.29987  5.42870  3.67407      4 k - - -
          2.68618  4.42225  2.77519  2.73123  3.46354  2.40513  3.72494
3.29354  2.67741  2.69355  4.24690  2.90347  2.73739  3.18146  2.89801
2.37887  2.77519  2.98518  4.58477  3.61503
          0.02054  4.29155  5.01389  0.61958  0.77255  0.48576  0.95510
      5   2.76109  5.09532  2.82908  2.27513  4.41455  3.49847  3.67057
3.84488  2.23590  3.37024  4.16361  2.99377  3.90320  2.79461  1.56308
2.64553  2.98544  3.18746  5.53026  4.18983      5 r - - -
          2.68618  4.42225  2.77519  2.73123  3.46354  2.40513  3.72494
3.29354  2.67741  2.69355  4.24690  2.90347  2.73739  3.18146  2.89801
2.37887  2.77519  2.98518  4.58477  3.61503
          0.02054  4.29155  5.01389  0.61958  0.77255  0.48576  0.95510
      6   4.12486  5.33116  4.78874  4.61253  3.22493  4.25077  4.51635
4.19419  4.34279  3.50684  4.80357  4.68577  4.80617  4.70640  4.38951
4.32441  4.45106  4.09423  0.34433  3.20864      6 W - - -
          2.68618  4.42225  2.77519  2.73123  3.46354  2.40513  3.72494
3.29354  2.67741  2.69355  4.24690  2.90347  2.73739  3.18146  2.89801
2.37887  2.77519  2.98518  4.58477  3.61503
```

```
          0.02054  4.29155  5.01389  0.61958  0.77255  0.48576  0.95510
      7   2.80720  4.38513  3.90227  3.34591  2.97055  3.84056  4.08415
2.62394  3.24948  1.32579  3.01512  2.59648  4.20187  3.52983  3.52591
3.11953  3.04124  2.51073  4.87468  3.60582      7 l - - -
          2.68618  4.42225  2.77519  2.73123  3.46354  2.40513  3.72494
3.29354  2.67741  2.69355  4.24690  2.90347  2.73739  3.18146  2.89801
2.37887  2.77519  2.98518  4.58477  3.61503
          0.02054  4.29155  5.01389  0.61958  0.77255  0.48576  0.95510
      8   2.76704  5.03876  3.12881  2.54639  4.36507  3.53569  3.67076
3.77720  1.66241  3.31407  4.11781  2.84493  3.92281  2.79890  2.15820
2.77064  2.37308  3.07504  5.47876  4.16688      8 k - - -
          2.68618  4.42225  2.77519  2.73123  3.46354  2.40513  3.72494
3.29354  2.67741  2.69355  4.24690  2.90347  2.73739  3.18146  2.89801
2.37887  2.77519  2.98518  4.58477  3.61503
          0.02054  4.29155  5.01389  0.61958  0.77255  0.48576  0.95510
      9   2.67318  4.98800  2.92845  2.44313  4.35304  3.42360  3.69304
3.79658  2.08587  3.34449  4.13066  2.44764  2.40248  2.82131  2.90154
2.38037  2.53797  3.41188  5.52994  4.16826      9 k - - -
          2.68618  4.42225  2.77519  2.73123  3.46354  2.40513  3.72494
3.29354  2.67741  2.69355  4.24690  2.90347  2.73739  3.18146  2.89801
2.37887  2.77519  2.98518  4.58477  3.61503
          0.02054  4.29155  5.01389  0.61958  0.77255  0.48576  0.95510
     10   2.49780  5.09789  2.71118  2.09557  4.41705  3.42149  3.61959
3.88571  2.32740  3.30382  4.14702  2.67429  3.20367  2.24337  2.85755
2.56960  2.62383  3.26569  5.54640  4.15017     10 e - - -
          2.68618  4.42225  2.77519  2.73123  3.46354  2.40513  3.72494
3.29354  2.67741  2.69355  4.24690  2.90347  2.73739  3.18146  2.89801
2.37887  2.77519  2.98518  4.58477  3.61503
          0.02054  4.29155  5.01389  0.61958  0.77255  0.48576  0.95510
     11   3.10728  5.50288  2.51888  0.89242  4.83887  3.46409  3.91526
4.30480  2.47383  3.82668  4.69743  2.96658  4.04408  3.08023  3.14813
3.02980  3.37888  3.92189  5.95278  4.55915     11 e - - -
          2.68618  4.42225  2.77519  2.73123  3.46354  2.40513  3.72494
3.29354  2.67741  2.69355  4.24690  2.90347  2.73739  3.18146  2.89801
2.37887  2.77519  2.98518  4.58477  3.61503
          0.02054  4.29155  5.01389  0.61958  0.77255  0.48576  0.95510
     12   3.31029  4.55240  5.22386  4.72067  3.73620  4.80324  5.38388
1.15399  4.62753  2.02125  3.50833  4.90713  5.04204  4.84976  4.78741
4.19971  3.56362  1.11557  5.76848  4.58359     12 v - - -
          2.68618  4.42225  2.77519  2.73123  3.46354  2.40513  3.72494
3.29354  2.67741  2.69355  4.24690  2.90347  2.73739  3.18146  2.89801
2.37887  2.77519  2.98518  4.58477  3.61503
          0.02054  4.29155  5.01389  0.61958  0.77255  0.48576  0.95510
     13   2.41698  2.97899  4.19725  3.60559  3.05810  3.74695  3.58588
2.46200  3.46719  1.69451  3.09483  3.76922  4.11425  3.66946  3.61187
3.03940  2.85942  2.19290  4.68475  2.87983     13 l - - -
          2.68618  4.42225  2.77519  2.73123  3.46354  2.40513  3.72494
3.29354  2.67741  2.69355  4.24690  2.90347  2.73739  3.18146  2.89801
2.37887  2.77519  2.98518  4.58477  3.61503
          0.02054  4.29155  5.01389  0.61958  0.77255  0.48576  0.95510
     14   2.40164  5.02024  2.67827  2.20576  3.44179  3.32018  3.63463
3.76157  2.39882  3.30220  4.07652  2.60331  3.83072  2.22010  2.88181
2.52606  2.87894  3.38166  5.48628  3.61432     14 e - - -
          2.68618  4.42225  2.77519  2.73123  3.46354  2.40513  3.72494
3.29354  2.67741  2.69355  4.24690  2.90347  2.73739  3.18146  2.89801
2.37887  2.77519  2.98518  4.58477  3.61503
          0.02054  4.29155  5.01389  0.61958  0.77255  0.48576  0.95510
```

```
     15   3.34132  4.60045  5.18050  4.64967  3.54142  4.77256  5.24889
0.95093  4.53901  1.75180  3.10259  4.85268  4.98527  4.70492  4.67262
4.15027  3.58340  1.66392  5.59144  4.46385      15 i - - -
          2.68618  4.42225  2.77519  2.73123  3.46354  2.40513  3.72494
3.29354  2.67741  2.69355  4.24690  2.90347  2.73739  3.18146  2.89801
2.37887  2.77519  2.98518  4.58477  3.61503
          0.02054  4.29155  5.01389  0.61958  0.77255  0.48576  0.95510
     16   3.55902  4.83558  5.22759  4.69682  3.24093  4.86583  5.20195
1.76905  4.51988  0.73984  3.02983  4.93700  5.01406  4.61175  4.60239
4.25978  3.78668  2.28816  5.39598  4.32023      16 l - - -
          2.68618  4.42225  2.77519  2.73123  3.46354  2.40513  3.72494
3.29354  2.67741  2.69355  4.24690  2.90347  2.73739  3.18146  2.89801
2.37887  2.77519  2.98518  4.58477  3.61503
          0.02054  4.29155  5.01389  0.61958  0.77255  0.48576  0.95510
     17   2.65737  4.81851  3.08178  2.44875  4.05267  3.49023  3.68396
3.07488  2.24272  2.44448  3.89256  3.03007  3.87807  2.30875  2.69174
2.45535  2.67568  3.14810  5.32007  3.99999      17 k - - -
          2.68558  4.42229  2.77524  2.73127  3.46358  2.40517  3.72499
3.29358  2.67745  2.69359  4.24694  2.90351  2.73744  3.18151  2.89805
2.37891  2.77524  2.98523  4.58481  3.61507
          0.28314  2.11312  2.07368  0.66320  0.72402  0.48576  0.95510
     18   2.58974  4.02670  3.00631  2.54403  4.10917  3.15046  3.23640
3.55084  2.55742  3.16092  3.98275  1.71627  3.83907  2.91635  2.99157
2.42381  2.87408  3.19790  5.38709  4.05958      20 n - - -
          2.68618  4.42225  2.77519  2.73123  3.46354  2.40513  3.72494
3.29354  2.67741  2.69355  4.24690  2.90347  2.73739  3.18146  2.89801
2.37887  2.77519  2.98518  4.58477  3.61503
          0.02335  4.16476  4.88711  0.61958  0.77255  0.63877  0.75065
     19   2.34716  4.33556  3.74745  3.19701  2.48307  3.72009  2.90624
2.81892  3.12202  2.50330  3.44604  3.51019  4.09351  3.38954  3.41298
2.98848  2.96238  2.45275  4.47951  2.00779      21 y - - -
          2.68618  4.42225  2.77519  2.73123  3.46354  2.40513  3.72494
3.29354  2.67741  2.69355  4.24690  2.90347  2.73739  3.18146  2.89801
2.37887  2.77519  2.98518  4.58477  3.61503
          0.02335  4.16476  4.88711  0.61958  0.77255  0.48632  0.95421
     20   2.45115  5.10564  2.09813  2.27235  4.42591  3.41409  3.49892
3.89326  2.08856  3.16259  4.15581  2.89060  3.81881  2.54709  2.71465
2.62892  2.81021  3.48347  5.54940  4.15525      22 k - - -
          2.68618  4.42225  2.77519  2.73123  3.46354  2.40513  3.72494
3.29354  2.67741  2.69355  4.24690  2.90347  2.73739  3.18146  2.89801
2.37887  2.77519  2.98518  4.58477  3.61503
          0.02154  4.24435  4.96669  0.61958  0.77255  0.44883  1.01715
     21   2.57979  4.51082  2.98731  2.29637  4.22725  3.28878  3.64560
3.66192  2.14630  2.74800  4.01552  2.95896  2.75552  2.66253  2.54963
2.50304  2.87823  3.30550  5.42994  4.07326      23 k - - -
          2.68618  4.42225  2.77519  2.73123  3.46354  2.40513  3.72494
3.29354  2.67741  2.69355  4.24690  2.90347  2.73739  3.18146  2.89801
2.37887  2.77519  2.98518  4.58477  3.61503
          0.24121  4.29155  1.60626  0.61958  0.77255  0.48576  0.95510
     22   2.50868  4.58694  3.13812  2.57575  3.77351  3.46735  3.18935
3.16557  2.31915  2.60895  3.67371  3.06096  3.13431  2.88310  2.80225
2.68824  2.82010  2.69070  5.11888  3.08683      24 k - - -
          2.68618  4.42225  2.77519  2.73123  3.46354  2.40513  3.72494
3.29354  2.67741  2.69355  4.24690  2.90347  2.73739  3.18146  2.89801
2.37887  2.77519  2.98518  4.58477  3.61503
          0.02554  4.07589  4.79823  0.61958  0.77255  0.72427  0.66296
```

```
     23   2.63726  4.87189  2.81008  2.43434  4.33784  2.33040  3.71039
3.77662  1.98215  3.33825  4.14551  2.70901  3.82123  2.85839  2.88914
2.14004  2.92098  3.38421  5.52717  4.18444     25 k - - -
          2.68618  4.42225  2.77519  2.73123  3.46354  2.40513  3.72494
3.29354  2.67741  2.69355  4.24690  2.90347  2.73739  3.18146  2.89801
2.37887  2.77519  2.98518  4.58477  3.61503
          0.02554  4.07589  4.79823  0.61958  0.77255  0.36115  1.19361
     24   2.50493  4.13961  3.17889  2.75887  2.35538  3.01038  3.83299
2.91017  2.82552  2.37644  3.50103  2.82208  3.96744  3.13604  3.19898
2.82479  2.85989  2.51711  4.97484  3.57666     26 f - - -
          2.68618  4.42225  2.77519  2.73123  3.46354  2.40513  3.72494
3.29354  2.67741  2.69355  4.24690  2.90347  2.73739  3.18146  2.89801
2.37887  2.77519  2.98518  4.58477  3.61503
          0.02054  4.29155  5.01389  0.61958  0.77255  0.48576  0.95510
     25   2.49908  3.26054  2.98924  2.26805  3.94006  3.49048  3.48334
3.23237  2.42455  2.64303  3.80863  2.94916  3.32712  2.70514  2.92141
2.64305  2.86544  3.04569  3.93232  3.94113     27 e - - -
          2.68618  4.42225  2.77519  2.73123  3.46354  2.40513  3.72494
3.29354  2.67741  2.69355  4.24690  2.90347  2.73739  3.18146  2.89801
2.37887  2.77519  2.98518  4.58477  3.61503
          0.02054  4.29155  5.01389  0.61958  0.77255  0.48576  0.95510
     26   2.81396  4.24393  4.35012  3.76990  3.04182  3.96552  4.28586
1.40323  3.63994  1.88907  3.20985  3.96853  3.88483  3.52320  3.80384
3.26613  3.04735  1.98742  4.89968  3.71954     28 i - - -
          2.68618  4.42225  2.77519  2.73123  3.46354  2.40513  3.72494
3.29354  2.67741  2.69355  4.24690  2.90347  2.73739  3.18146  2.89801
2.37887  2.77519  2.98518  4.58477  3.61503
          0.02054  4.29155  5.01389  0.61958  0.77255  0.48576  0.95510
     27   2.50147  4.18454  3.15423  2.59469  3.86658  3.50505  3.05632
3.25913  2.41133  2.71966  3.75385  2.91104  3.89346  2.83734  3.01194
2.36137  2.34219  2.63954  5.20326  3.90620     29 t - - -
          2.68618  4.42225  2.77519  2.73123  3.46354  2.40513  3.72494
3.29354  2.67741  2.69355  4.24690  2.90347  2.73739  3.18146  2.89801
2.37887  2.77519  2.98518  4.58477  3.61503
          0.02054  4.29155  5.01389  0.61958  0.77255  0.48576  0.95510
     28   2.57717  4.94207  2.97756  2.22375  3.60539  3.44748  3.04008
3.64623  2.31025  3.21403  4.00405  2.84197  3.10690  2.48188  2.89248
2.60319  2.58801  2.91359  5.42304  4.06400     30 e - - -
          2.68618  4.42225  2.77519  2.73123  3.46354  2.40513  3.72494
3.29354  2.67741  2.69355  4.24690  2.90347  2.73739  3.18146  2.89801
2.37887  2.77519  2.98518  4.58477  3.61503
          0.02054  4.29155  5.01389  0.61958  0.77255  0.48576  0.95510
     29   2.49298  5.10260  2.79717  2.00954  4.42141  3.42529  3.62669
3.88771  2.03748  3.39583  4.15413  2.90400  3.82754  2.39302  2.85624
2.29830  2.81208  3.22994  5.55110  4.15762     31 e - - -
          2.68618  4.42225  2.77519  2.73123  3.46354  2.40513  3.72494
3.29354  2.67741  2.69355  4.24690  2.90347  2.73739  3.18146  2.89801
2.37887  2.77519  2.98518  4.58477  3.61503
          0.02054  4.29155  5.01389  0.61958  0.77255  0.48576  0.95510
     30   2.43931  4.39456  3.45160  2.88817  3.55533  3.58082  3.85055
2.56256  2.83985  2.21602  3.51001  3.30673  2.54573  3.15573  2.99688
2.45655  2.78874  2.58197  4.98771  3.74598     32 l - - -
          2.68618  4.42225  2.77519  2.73123  3.46354  2.40513  3.72494
3.29354  2.67741  2.69355  4.24690  2.90347  2.73739  3.18146  2.89801
2.37887  2.77519  2.98518  4.58477  3.61503
          0.02054  4.29155  5.01389  0.61958  0.77255  0.48576  0.95510
```

```
     31   2.64506  4.74068  3.11269  2.56346  3.97538  3.48732  3.71532
3.24656  2.52641  2.90331  3.84123  2.88029  2.12431  2.69642  2.84738
2.42418  2.88195  2.80935  5.27801  3.96946     33 p - - -
          2.68618  4.42226  2.77520  2.73124  3.46354  2.40513  3.72495
3.29354  2.67741  2.69346  4.24690  2.90347  2.73740  3.18147  2.89801
2.37887  2.77520  2.98519  4.58477  3.61504
          0.05372  3.08661  5.01389  0.47303  0.97581  0.48576  0.95510
     32   2.63860  4.92674  2.74121  2.42954  4.19072  3.44944  3.29101
3.62331  2.42757  2.96094  3.99029  2.60512  2.99131  2.62181  2.90098
2.57627  2.42383  2.93921  5.41138  3.66415     35 t - - -
          2.68618  4.42225  2.77519  2.73123  3.46354  2.40513  3.72494
3.29354  2.67741  2.69355  4.24690  2.90347  2.73739  3.18146  2.89801
2.37887  2.77519  2.98518  4.58477  3.61503
          0.02054  4.29155  5.01389  0.61958  0.77255  0.48576  0.95510
     33   2.82052  5.14163  3.16588  2.56031  4.49527  3.56908  3.65727
3.91047  1.85122  3.26055  3.91250  2.86060  3.94451  2.44192  1.69518
2.70468  2.93921  3.54144  5.52786  4.22410     36 r - - -
          2.68618  4.42225  2.77519  2.73123  3.46354  2.40513  3.72494
3.29354  2.67741  2.69355  4.24690  2.90347  2.73739  3.18146  2.89801
2.37887  2.77519  2.98518  4.58477  3.61503
          0.02054  4.29155  5.01389  0.61958  0.77255  0.48576  0.95510
     34   3.40778  5.07202  4.06240  3.97555  4.90212  3.73699  4.99900
4.58121  4.07395  4.15041  5.23109  4.23115  0.32126  4.44812  4.25302
3.59265  3.88492  4.17347  5.94956  5.03458     37 P - - -
          2.68618  4.42225  2.77519  2.73123  3.46354  2.40513  3.72494
3.29354  2.67741  2.69355  4.24690  2.90347  2.73739  3.18146  2.89801
2.37887  2.77519  2.98518  4.58477  3.61503
          0.02054  4.29155  5.01389  0.61958  0.77255  0.48576  0.95510
     35   2.35774  3.42514  3.15460  2.42506  3.86849  3.50433  3.72574
3.26008  2.50080  2.75455  3.75578  3.09005  2.63248  2.59620  3.01270
2.66705  2.80890  2.76760  5.20513  3.90838     38 a - - -
          2.68618  4.42225  2.77519  2.73123  3.46354  2.40513  3.72494
3.29354  2.67741  2.69355  4.24690  2.90347  2.73739  3.18146  2.89801
2.37887  2.77519  2.98518  4.58477  3.61503
          0.02054  4.29155  5.01389  0.61958  0.77255  0.48576  0.95510
     36   2.56516  4.85928  2.89546  2.53549  4.36961  2.87533  3.35179
3.82073  2.65946  3.40129  4.20844  2.44853  3.88319  2.98952  3.11623
1.45660  2.96844  3.41816  5.60551  4.25699     39 s - - -
          2.68618  4.42225  2.77519  2.73123  3.46354  2.40513  3.72494
3.29354  2.67741  2.69355  4.24690  2.90347  2.73739  3.18146  2.89801
2.37887  2.77519  2.98518  4.58477  3.61503
          0.02054  4.29155  5.01389  0.61958  0.77255  0.48576  0.95510
     37   2.51715  4.45696  3.46901  3.38450  4.72412  0.70896  4.58394
4.26277  3.63340  3.94169  4.78476  3.54529  3.94589  3.89456  3.90967
2.41431  3.04298  3.61392  6.02379  4.82074     40 g - - -
          2.68618  4.42225  2.77519  2.73123  3.46354  2.40513  3.72494
3.29354  2.67741  2.69355  4.24690  2.90347  2.73739  3.18146  2.89801
2.37887  2.77519  2.98518  4.58477  3.61503
          0.02054  4.29155  5.01389  0.61958  0.77255  0.48576  0.95510
     38   2.38079  4.11336  2.65213  2.45787  4.18414  3.43767  3.67360
3.61332  2.45910  3.19473  3.99249  2.97934  3.84608  2.53604  2.84607
2.01878  2.42794  3.26190  5.41510  4.06600     41 s - - -
          2.68618  4.42225  2.77519  2.73123  3.46354  2.40513  3.72494
3.29354  2.67741  2.69355  4.24690  2.90347  2.73739  3.18146  2.89801
2.37887  2.77519  2.98518  4.58477  3.61503
          0.02054  4.29155  5.01389  0.61958  0.77255  0.48576  0.95510
```

```
     39   2.63846  4.12908  4.04241  3.45868  3.04150  3.72996  3.55834
2.19206  3.34408  1.71083  3.23060  3.68591  4.09843  2.94640  3.54135
3.01297  2.87016  2.23037  4.28049  3.08790     42 l - - -
          2.68618  4.42225  2.77519  2.73123  3.46354  2.40513  3.72494
3.29354  2.67741  2.69355  4.24690  2.90347  2.73739  3.18146  2.89801
2.37887  2.77519  2.98518  4.58477  3.61503
          0.02054  4.29155  5.01389  0.61958  0.77255  0.48576  0.95510
     40   2.95488  4.39006  4.39238  3.85132  1.51247  4.04106  4.14882
2.54129  3.71110  1.62799  3.28209  3.60252  4.38929  3.90241  3.86644
3.35509  3.19276  2.27780  4.60351  3.17498     43 f - - -
          2.68618  4.42225  2.77519  2.73123  3.46354  2.40513  3.72494
3.29354  2.67741  2.69355  4.24690  2.90347  2.73739  3.18146  2.89801
2.37887  2.77519  2.98518  4.58477  3.61503
          0.02054  4.29155  5.01389  0.61958  0.77255  0.48576  0.95510
     41   3.46065  4.73778  5.25548  4.67527  2.75196  4.76705  5.06045
1.61508  4.54180  0.93544  2.58187  4.86147  4.90952  4.53777  4.57073
4.12080  3.67428  2.39336  5.25579  4.21563     44 l - - -
          2.68618  4.42225  2.77519  2.73123  3.46354  2.40513  3.72494
3.29354  2.67741  2.69355  4.24690  2.90347  2.73739  3.18146  2.89801
2.37887  2.77519  2.98518  4.58477  3.61503
          0.02054  4.29155  5.01389  0.61958  0.77255  0.48576  0.95510
     42   3.20240  4.54448  4.71421  4.21398  1.44985  4.25972  3.96115
2.32695  4.05387  1.90678  3.41435  4.18302  4.56979  4.12276  4.11465
3.58269  3.42662  2.69982  4.21132  1.83075     45 f - - -
          2.68618  4.42225  2.77519  2.73123  3.46354  2.40513  3.72494
3.29354  2.67741  2.69355  4.24690  2.90347  2.73739  3.18146  2.89801
2.37887  2.77519  2.98518  4.58477  3.61503
          0.02054  4.29155  5.01389  0.61958  0.77255  0.48576  0.95510
     43   2.77438  5.24881  1.83559  2.32110  4.58213  3.40029  3.71252
4.06416  2.52320  3.56389  4.33725  2.12861  2.81313  2.83458  2.73080
2.61428  3.02335  3.64318  5.71668  4.29527     46 d - - -
          2.68618  4.42225  2.77519  2.73123  3.46354  2.40513  3.72494
3.29354  2.67741  2.69355  4.24690  2.90347  2.73739  3.18146  2.89801
2.37887  2.77519  2.98518  4.58477  3.61503
          0.02054  4.29155  5.01389  0.61958  0.77255  0.48576  0.95510
     44   2.75542  4.93591  3.18228  2.63663  4.35616  3.50603  3.74089
3.76151  2.29527  3.31839  4.13621  3.11145  3.29262  2.88431  1.47451
2.41260  2.73222  3.40395  5.49583  4.20594     47 r - - -
          2.68618  4.42225  2.77519  2.73123  3.46354  2.40513  3.72494
3.29354  2.67741  2.69355  4.24690  2.90347  2.73739  3.18146  2.89801
2.37887  2.77519  2.98518  4.58477  3.61503
          0.02054  4.29155  5.01389  0.61958  0.77255  0.48576  0.95510
     45   2.90728  5.11016  3.29704  2.44722  4.44633  3.64213  3.69343
3.82799  1.44936  2.37609  4.17775  3.15170  4.01288  2.82714  2.11826
2.91334  3.10501  3.49963  5.48900  4.23036     48 k - - -
          2.68619  4.42226  2.77521  2.73125  3.46355  2.40514  3.72496
3.29315  2.67742  2.69356  4.24691  2.90348  2.73741  3.18148  2.89802
2.37888  2.77521  2.98520  4.58478  3.61504
          0.09794  2.44582  5.01389  0.36379  1.18757  0.48576  0.95510
     46   2.50467  4.58934  2.65335  2.67935  3.77531  3.54367  3.77153
2.51456  2.20203  2.81127  3.34586  3.15771  3.92971  2.99168  3.07688
2.76692  2.81965  2.30410  5.15171  3.87151     50 k - - -
          2.68618  4.42225  2.77519  2.73123  3.46354  2.40513  3.72494
3.29354  2.67741  2.69355  4.24690  2.90347  2.73739  3.18146  2.89801
2.37887  2.77519  2.98518  4.58477  3.61503
          0.02054  4.29155  5.01389  0.61958  0.77255  0.48576  0.95510
```

```
     47   2.62907  3.97246  3.47334  2.68240  3.51256  2.97115  3.84922
2.67613  2.53771  2.03652  3.36155  3.12053  3.97942  3.16855  3.22028
2.84166  2.86088  2.27933  4.95132  3.71312     51 l - - -
          2.68618  4.42225  2.77519  2.73123  3.46354  2.40513  3.72494
3.29354  2.67741  2.69355  4.24690  2.90347  2.73739  3.18146  2.89801
2.37887  2.77519  2.98518  4.58477  3.61503
          0.02054  4.29155  5.01389  0.61958  0.77255  0.48576  0.95510
     48   2.50468  5.04729  3.23450  2.66233  4.45581  3.57218  3.72016
3.85098  2.02177  3.37723  4.19792  3.13369  3.98244  2.85550  1.47596
2.37846  3.07058  3.49767  5.52100  4.24865     52 r - - -
          2.68618  4.42225  2.77519  2.73123  3.46354  2.40513  3.72494
3.29354  2.67741  2.69355  4.24690  2.90347  2.73739  3.18146  2.89801
2.37887  2.77519  2.98518  4.58477  3.61503
          0.02054  4.29155  5.01389  0.61958  0.77255  0.48576  0.95510
     49   2.64551  4.49050  3.04565  2.78135  2.74668  3.17286  3.79103
3.02273  2.45734  2.70902  3.58741  2.90111  3.95520  3.07260  3.14655
2.80428  2.87705  2.77883  3.24871  2.66079     53 k - - -
          2.68618  4.42225  2.77519  2.73123  3.46354  2.40513  3.72494
3.29354  2.67741  2.69355  4.24690  2.90347  2.73739  3.18146  2.89801
2.37887  2.77519  2.98518  4.58477  3.61503
          0.07520  4.29155  2.83433  0.61958  0.77255  0.48576  0.95510
     50   3.98849  5.09363  5.04305  4.81593  0.91001  4.67867  3.55078
3.66351  4.61688  2.95579  4.22374  4.31219  4.95620  4.42576  4.52399
4.05230  4.19471  3.57729  2.15692  1.61586     54 f - - -
          2.68618  4.42225  2.77519  2.73123  3.46354  2.40513  3.72494
3.29354  2.67741  2.69355  4.24690  2.90347  2.73739  3.18146  2.89801
2.37887  2.77519  2.98518  4.58477  3.61503
          0.02168  4.23803  4.96037  0.61958  0.77255  0.44443  1.02496
     51   3.29751  5.42421  3.77901  3.00481  4.95796  3.88451  3.72454
4.26024  1.25859  3.63248  4.50141  3.38043  4.21860  2.68427  1.28324
3.27106  3.42906  3.93757  5.62192  4.49738     55 k - - -
          2.68618  4.42225  2.77519  2.73123  3.46354  2.40513  3.72494
3.29354  2.67741  2.69355  4.24690  2.90347  2.73739  3.18146  2.89801
2.37887  2.77519  2.98518  4.58477  3.61503
          0.02054  4.29155  5.01389  0.61958  0.77255  0.48576  0.95510
     52   2.63020  5.18082  3.03492  2.48885  4.54269  3.52305  3.65539
3.97831  1.55401  3.45883  4.24054  2.55865  3.91684  2.31849  2.43306
2.64956  3.01228  3.58579  5.57641  4.24221     56 k - - -
          2.68618  4.42225  2.77519  2.73123  3.46354  2.40513  3.72494
3.29354  2.67741  2.69355  4.24690  2.90347  2.73739  3.18146  2.89801
2.37887  2.77519  2.98518  4.58477  3.61503
          0.02054  4.29155  5.01389  0.61958  0.77255  0.48576  0.95510
     53   3.50502  5.63344  0.46547  2.70166  5.08200  3.58809  4.38221
4.77985  3.55697  4.31808  5.32537  3.27215  4.27088  3.63548  4.11541
3.43308  3.85880  4.37983  6.15976  4.91119     57 D - - -
          2.68618  4.42225  2.77519  2.73123  3.46354  2.40513  3.72494
3.29354  2.67741  2.69355  4.24690  2.90347  2.73739  3.18146  2.89801
2.37887  2.77519  2.98518  4.58477  3.61503
          0.02054  4.29155  5.01389  0.61958  0.77255  0.48576  0.95510
     54   2.78218  4.64578  3.63866  3.45078  4.36410  0.67688  4.51753
3.85774  3.49642  2.91130  4.55696  3.68794  4.11485  3.86063  3.76309
2.95886  3.24744  3.46289  5.71817  4.48038     58 G - - -
          2.68618  4.42225  2.77519  2.73123  3.46354  2.40513  3.72494
3.29354  2.67741  2.69355  4.24690  2.90347  2.73739  3.18146  2.89801
2.37887  2.77519  2.98518  4.58477  3.61503
          0.02054  4.29155  5.01389  0.61958  0.77255  0.48576  0.95510
```

55 3.79269 5.10473 4.60017 4.22066 2.09745 4.50585 1.70344
3.72723 4.03247 3.07436 4.27402 4.11238 4.81603 4.12640 4.15541
3.84266 4.00455 3.59227 2.28997 1.19043 59 y - - -
2.68618 4.42225 2.77519 2.73123 3.46354 2.40513 3.72494
3.29354 2.67741 2.69355 4.24690 2.90347 2.73739 3.18146 2.89801
2.37887 2.77519 2.98518 4.58477 3.61503
0.02054 4.29155 5.01389 0.61958 0.77255 0.48576 0.95510
56 2.63405 4.20079 3.02555 2.51639 3.90300 3.49774 3.33309
2.58708 2.55386 2.94202 3.61669 2.40851 3.88567 2.63444 2.99764
2.65138 2.48917 2.91236 5.22770 3.92337 60 n - - -
2.68618 4.42225 2.77519 2.73123 3.46354 2.40513 3.72494
3.29354 2.67741 2.69355 4.24690 2.90347 2.73739 3.18146 2.89801
2.37887 2.77519 2.98518 4.58477 3.61503
0.02054 4.29155 5.01389 0.61958 0.77255 0.48576 0.95510
57 3.92348 5.13419 4.82750 4.53269 2.10420 4.58241 3.63546
3.71541 4.27039 3.02332 4.27881 4.26190 4.90466 4.31517 4.30206
3.99081 4.14431 3.61757 0.99716 1.52470 61 w - - -
2.68618 4.42225 2.77519 2.73123 3.46354 2.40513 3.72494
3.29354 2.67741 2.69355 4.24690 2.90347 2.73739 3.18146 2.89801
2.37887 2.77519 2.98518 4.58477 3.61503
0.02054 4.29155 5.01389 0.61958 0.77255 0.48576 0.95510
58 2.78823 4.97101 3.21780 2.61180 4.25434 3.57670 3.68554
3.17622 1.91389 3.21114 3.15795 3.10009 3.95133 2.46570 1.81251
2.81312 3.00031 3.32968 5.40828 4.12133 62 r - - -
2.68618 4.42225 2.77519 2.73123 3.46354 2.40513 3.72494
3.29354 2.67741 2.69355 4.24690 2.90347 2.73739 3.18146 2.89801
2.37887 2.77519 2.98518 4.58477 3.61503
0.02054 4.29155 5.01389 0.61958 0.77255 0.48576 0.95510
59 2.85297 5.08468 3.16146 2.63350 4.50197 3.55848 3.72693
3.90110 1.28113 3.42005 4.23971 3.11362 2.70186 2.86026 2.50074
2.85947 2.84129 3.53934 5.55806 4.27529 63 k - - -
2.68618 4.42225 2.77519 2.73123 3.46354 2.40513 3.72494
3.29354 2.67741 2.69355 4.24690 2.90347 2.73739 3.18146 2.89801
2.37887 2.77519 2.98518 4.58477 3.61503
0.02054 4.29155 5.01389 0.61958 0.77255 0.48576 0.95510
60 3.32694 5.41973 3.83109 3.05083 4.96078 3.89877 3.74473
4.26608 1.18089 3.64209 4.52104 3.41419 4.23907 2.87234 1.28813
3.30641 3.45970 3.94881 5.62552 4.51116 64 k - - -
2.68618 4.42225 2.77519 2.73123 3.46354 2.40513 3.72494
3.29354 2.67741 2.69355 4.24690 2.90347 2.73739 3.18146 2.89801
2.37887 2.77519 2.98518 4.58477 3.61503
0.02054 4.29155 5.01389 0.61958 0.77255 0.48576 0.95510
61 2.78274 5.15389 3.05969 2.49436 4.50444 3.52540 3.18424
3.93746 1.46736 3.42709 4.21045 2.72623 3.61419 2.67085 2.43247
2.76028 2.75342 3.55167 5.55251 4.22079 65 k - - -
2.68618 4.42225 2.77519 2.73123 3.46354 2.40513 3.72494
3.29354 2.67741 2.69355 4.24690 2.90347 2.73739 3.18146 2.89801
2.37887 2.77519 2.98518 4.58477 3.61503
0.02054 4.29155 5.01389 0.61958 0.77255 0.48576 0.95510
62 2.95099 5.51573 1.47346 2.26382 4.81913 3.38666 3.80756
4.32811 2.50882 3.80496 4.60582 1.94466 3.93802 2.44904 3.24568
2.85943 3.21365 3.89297 5.94921 4.47832 66 d - - -
2.68618 4.42225 2.77519 2.73123 3.46354 2.40513 3.72494
3.29354 2.67741 2.69355 4.24690 2.90347 2.73739 3.18146 2.89801
2.37887 2.77519 2.98518 4.58477 3.61503
0.02054 4.29155 5.01389 0.61958 0.77255 0.48576 0.95510

```
     63   2.72315  4.85149  2.83825  2.62968  4.54351  1.22067  3.98374
3.99334  2.50488  3.58188  4.41360  2.77772  3.93845  3.16864  3.21631
2.54455  3.08364  3.55392  5.77466  4.44800     67 g - - -
          2.68618  4.42225  2.77519  2.73123  3.46354  2.40513  3.72494
3.29354  2.67741  2.69355  4.24690  2.90347  2.73739  3.18146  2.89801
2.37887  2.77519  2.98518  4.58477  3.61503
          0.02054  4.29155  5.01389  0.61958  0.77255  0.48576  0.95510
     64   2.74378  4.88353  3.19596  2.60718  4.14798  3.55297  3.69988
2.93219  1.65504  3.13269  3.96566  3.09700  3.93563  2.85460  2.19221
2.59088  2.96439  2.89982  5.36039  4.06832     68 k - - -
          2.68618  4.42225  2.77519  2.73123  3.46354  2.40513  3.72494
3.29354  2.67741  2.69355  4.24690  2.90347  2.73739  3.18146  2.89801
2.37887  2.77519  2.98518  4.58477  3.61503
          0.02054  4.29155  5.01389  0.61958  0.77255  0.48576  0.95510
     65   2.49981  4.70335  3.11954  2.58882  3.95974  2.52354  3.74160
3.36128  2.57531  2.99957  3.83703  2.65055  3.88897  2.78172  3.01124
2.71039  2.00018  3.06027  4.22507  3.97182     69 t - - -
          2.68619  4.42226  2.77520  2.73124  3.46355  2.40513  3.72495
3.29355  2.67741  2.69356  4.24690  2.90347  2.73740  3.18147  2.89786
2.37887  2.77520  2.98519  4.58478  3.61504
          0.06280  2.91460  5.01389  0.44501  1.02393  0.48576  0.95510
     66   2.64178  3.61006  4.21369  3.62581  2.73512  3.76395  4.09124
2.03648  3.49100  2.27993  3.21456  3.45597  4.13602  3.69689  3.64062
2.90805  2.41443  1.76227  4.72937  3.54011     71 v - - -
          2.68618  4.42225  2.77519  2.73123  3.46354  2.40513  3.72494
3.29354  2.67741  2.69355  4.24690  2.90347  2.73739  3.18146  2.89801
2.37887  2.77519  2.98518  4.58477  3.61503
          0.02054  4.29155  5.01389  0.61958  0.77255  0.48576  0.95510
     67   2.75666  5.17611  3.29963  2.71615  4.58181  3.15618  3.71434
3.96074  2.01229  3.44899  4.28002  3.17678  4.04366  2.62720  1.26244
2.96518  3.16786  3.61695  5.55449  4.31263     72 r - - -
          2.68618  4.42225  2.77519  2.73123  3.46354  2.40513  3.72494
3.29354  2.67741  2.69355  4.24690  2.90347  2.73739  3.18146  2.89801
2.37887  2.77519  2.98518  4.58477  3.61503
          0.02054  4.29155  5.01389  0.61958  0.77255  0.48576  0.95510
     68   3.39297  5.52624  2.75926  0.54903  4.93768  3.60383  4.25599
4.48256  3.19742  4.03920  5.01803  3.26687  4.23510  3.47743  3.63449
3.34437  3.70781  4.13065  6.02588  4.77906     73 E - - -
          2.68618  4.42225  2.77519  2.73123  3.46354  2.40513  3.72494
3.29354  2.67741  2.69355  4.24690  2.90347  2.73739  3.18146  2.89801
2.37887  2.77519  2.98518  4.58477  3.61503
          0.02054  4.29155  5.01389  0.61958  0.77255  0.48576  0.95510
     69   1.83902  4.95375  1.54377  2.47882  4.52855  3.15606  3.88476
3.95493  2.77608  3.55022  4.36111  2.99261  3.89866  3.04392  3.27293
2.73564  2.60498  3.53008  5.75846  4.38198     74 d - - -
          2.68618  4.42225  2.77519  2.73123  3.46354  2.40513  3.72494
3.29354  2.67741  2.69355  4.24690  2.90347  2.73739  3.18146  2.89801
2.37887  2.77519  2.98518  4.58477  3.61503
          0.02054  4.29155  5.01389  0.61958  0.77255  0.48576  0.95510
     70   2.77547  4.91062  3.10714  2.60872  4.08628  3.53586  1.70691
3.60145  2.41768  3.19018  4.03082  2.63281  3.95379  2.90582  2.56475
2.81447  3.00949  2.91337  5.34545  4.00406     75 h - - -
          2.68618  4.42225  2.77519  2.73123  3.46354  2.40513  3.72494
3.29354  2.67741  2.69355  4.24690  2.90347  2.73739  3.18146  2.89801
2.37887  2.77519  2.98518  4.58477  3.61503
          0.02054  4.29155  5.01389  0.61958  0.77255  0.48576  0.95510
```

```
     71   2.51810  4.68904  3.20191  1.65475  3.86476  3.55875  3.79173
3.10047  2.63375  2.48096  3.20955  3.15269  3.95143  2.98660  3.05919
2.78996  2.92378  2.70692  5.25268  3.96254     76 e - - -
          2.68618  4.42225  2.77519  2.73123  3.46354  2.40513  3.72494
3.29354  2.67741  2.69355  4.24690  2.90347  2.73739  3.18146  2.89801
2.37887  2.77519  2.98518  4.58477  3.61503
          0.02054  4.29155  5.01389  0.61958  0.77255  0.48576  0.95510
     72   3.04032  5.32015  3.34530  2.72548  4.76495  3.69939  3.69229
4.14036  1.34682  3.56345  4.38598  2.46250  4.06843  2.68410  1.75636
3.00997  3.22063  3.77686  5.61102  4.38073     77 k - - -
          2.68618  4.42225  2.77519  2.73123  3.46354  2.40513  3.72494
3.29354  2.67741  2.69355  4.24690  2.90347  2.73739  3.18146  2.89801
2.37887  2.77519  2.98518  4.58477  3.61503
          0.02054  4.29155  5.01389  0.61958  0.77255  0.48576  0.95510
     73   3.66400  5.01337  4.86321  4.52480  3.35447  4.51099  5.05294
2.59863  4.29078  0.48980  3.28075  4.76169  4.88668  4.55447  4.38991
4.16368  3.94564  2.74100  5.36364  4.17732     78 L - - -
          2.68618  4.42225  2.77519  2.73123  3.46354  2.40513  3.72494
3.29354  2.67741  2.69355  4.24690  2.90347  2.73739  3.18146  2.89801
2.37887  2.77519  2.98518  4.58477  3.61503
          0.02054  4.29155  5.01389  0.61958  0.77255  0.48576  0.95510
     74   3.05253  5.19642  3.50573  2.84070  4.60550  3.73434  3.72744
3.94806  1.06978  3.44112  4.29983  3.26714  4.10434  2.86184  2.01866
3.05931  3.23772  3.09921  5.54131  4.33460     79 k - - -
          2.68618  4.42225  2.77519  2.73123  3.46354  2.40513  3.72494
3.29354  2.67741  2.69355  4.24690  2.90347  2.73739  3.18146  2.89801
2.37887  2.77519  2.98518  4.58477  3.61503
          0.02054  4.29155  5.01389  0.61958  0.77255  0.48576  0.95510
     75   2.67596  4.36129  4.66269  4.13980  3.66487  4.21494  4.76493
1.40440  4.02939  2.41286  3.53991  4.31540  4.59771  4.28197  4.21609
3.28645  3.24891  1.15745  5.39348  4.18604     80 v - - -
          2.68618  4.42225  2.77519  2.73123  3.46354  2.40513  3.72494
3.29354  2.67741  2.69355  4.24690  2.90347  2.73739  3.18146  2.89801
2.37887  2.77519  2.98518  4.58477  3.61503
          0.02054  4.29155  5.01389  0.61958  0.77255  0.48576  0.95510
     76   2.75708  5.10782  2.66712  2.39736  4.46002  1.78732  3.73678
3.91428  2.52712  3.45340  4.24837  2.32847  3.89012  2.74086  2.68838
2.59425  3.01280  3.52498  5.63444  4.25317     81 g - - -
          2.68618  4.42225  2.77519  2.73123  3.46354  2.40513  3.72494
3.29354  2.67741  2.69355  4.24690  2.90347  2.73739  3.18146  2.89801
2.37887  2.77519  2.98518  4.58477  3.61503
          0.02054  4.29155  5.01389  0.61958  0.77255  0.48576  0.95510
     77   2.64458  4.85656  2.95075  2.54670  4.40175  1.90888  3.81440
3.84557  2.60509  3.41454  4.21235  2.14056  3.87450  2.96257  2.75047
2.29024  2.81675  3.43100  5.61000  4.26696     82 g - - -
          2.68621  4.42228  2.77522  2.73126  3.46357  2.40515  3.72478
3.29331  2.67743  2.69358  4.24692  2.90349  2.73728  3.18149  2.89803
2.37884  2.77522  2.98521  4.58480  3.61506
          0.12181  2.22526  5.01389  0.50314  0.92794  0.48576  0.95510
     78   2.49792  4.79574  2.92745  2.10205  4.02214  3.47595  3.42637
2.85166  2.49975  3.04885  3.87090  2.88552  3.86746  2.70539  2.95749
2.62878  2.64465  2.50159  5.30736  3.98218     85 e - - -
          2.68618  4.42225  2.77519  2.73123  3.46354  2.40513  3.72494
3.29354  2.67741  2.69355  4.24690  2.90347  2.73739  3.18146  2.89801
2.37887  2.77519  2.98518  4.58477  3.61503
          0.02054  4.29155  5.01389  0.61958  0.77255  0.48576  0.95510
```

```
     79   2.72042  5.21594  2.29996  1.79287  4.53976  3.40771  3.66212
4.02226  2.44255  3.51244  4.26996  2.52907  3.22222  2.37900  2.94629
2.67508  2.95904  3.20937  5.65662  4.23863     86 e - - -
          2.68618  4.42225  2.77519  2.73123  3.46354  2.40513  3.72494
3.29354  2.67741  2.69355  4.24690  2.90347  2.73739  3.18146  2.89801
2.37887  2.77519  2.98518  4.58477  3.61503
          0.02054  4.29155  5.01389  0.61958  0.77255  0.48576  0.95510
     80   2.08146  3.43249  3.93978  3.35863  3.25384  3.70045  3.61375
2.39052  3.25896  2.14054  2.87758  3.62273  4.07231  3.50352  3.05968
2.97743  2.74785  2.10725  4.75278  3.55369     87 a - - -
          2.68618  4.42225  2.77519  2.73123  3.46354  2.40513  3.72494
3.29354  2.67741  2.69355  4.24690  2.90347  2.73739  3.18146  2.89801
2.37887  2.77519  2.98518  4.58477  3.61503
          0.02054  4.29155  5.01389  0.61958  0.77255  0.48576  0.95510
     81   3.41914  4.68106  5.22412  4.69228  3.41545  4.81407  5.24563
1.13128  4.55794  1.25929  3.19423  4.90108  5.00144  4.68550  4.66453
4.19766  3.65694  1.90073  5.51601  4.41524     88 i - - -
          2.68618  4.42225  2.77519  2.73123  3.46354  2.40513  3.72494
3.29354  2.67741  2.69355  4.24690  2.90347  2.73739  3.18146  2.89801
2.37887  2.77519  2.98518  4.58477  3.61503
          0.02054  4.29155  5.01389  0.61958  0.77255  0.48576  0.95510
     82   2.64026  4.81838  3.04651  2.34432  4.04997  3.47306  2.78665
3.12403  2.48233  2.85807  3.89176  2.42794  3.86394  2.83459  2.94029
2.48067  2.46301  3.14736  5.32399  3.71927     89 e - - -
          2.68618  4.42225  2.77519  2.73123  3.46354  2.40513  3.72494
3.29354  2.67741  2.69355  4.24690  2.90347  2.73739  3.18146  2.89801
2.37887  2.77519  2.98518  4.58477  3.61503
          0.02054  4.29155  5.01389  0.61958  0.77255  0.48576  0.95510
     83   1.87095  1.44938  4.16978  3.74064  3.92646  2.57130  4.44251
3.14836  3.63944  2.99720  3.89888  3.70740  3.93159  3.87625  3.84132
2.65439  2.84761  2.39737  5.39728  4.22281     90 c - - -
          2.68618  4.42225  2.77519  2.73123  3.46354  2.40513  3.72494
3.29354  2.67741  2.69355  4.24690  2.90347  2.73739  3.18146  2.89801
2.37887  2.77519  2.98518  4.58477  3.61503
          0.02054  4.29155  5.01389  0.61958  0.77255  0.48576  0.95510
     84   2.66405  3.93362  3.57499  3.00429  3.44772  3.63780  3.87641
2.84497  2.39870  2.28346  3.47209  3.39152  4.02042  3.22929  3.20258
2.89571  2.68371  2.62627  4.90718  1.98626     91 y - - -
          2.68618  4.42225  2.77519  2.73123  3.46354  2.40513  3.72494
3.29354  2.67741  2.69355  4.24690  2.90347  2.73739  3.18146  2.89801
2.37887  2.77519  2.98518  4.58477  3.61503
          0.02054  4.29155  5.01389  0.61958  0.77255  0.48576  0.95510
     85   3.27630  3.88918  4.21517  3.77599  2.39677  4.10695  3.23316
3.35644  3.57764  2.88405  3.96209  3.88491  4.50119  3.83735  3.77343
3.45156  3.52797  3.17742  4.03993  0.86055     92 y - - -
          2.68618  4.42225  2.77519  2.73123  3.46354  2.40513  3.72494
3.29354  2.67741  2.69355  4.24690  2.90347  2.73739  3.18146  2.89801
2.37887  2.77519  2.98518  4.58477  3.61503
          0.02054  4.29155  5.01389  0.61958  0.77255  0.48576  0.95510
     86   1.72941  4.29482  3.77121  3.24504  3.66093  3.49570  4.11339
2.61100  3.18422  2.68983  3.61532  3.53141  4.01771  3.47423  3.49565
2.21178  2.76404  1.84446  5.13828  3.92072     93 a - - -
          2.68618  4.42225  2.77519  2.73123  3.46354  2.40513  3.72494
3.29354  2.67741  2.69355  4.24690  2.90347  2.73739  3.18146  2.89801
2.37887  2.77519  2.98518  4.58477  3.61503
          0.02054  4.29155  5.01389  0.61958  0.77255  0.48576  0.95510
```

```
     87   2.84799  4.94437  3.23412  2.68661  3.80835  3.61994  1.69812
3.60101  2.37984  3.16793  4.02942  3.15999  4.00729  2.63931  2.44457
2.88619  3.06351  3.30481  5.14776  2.95914     94 h - - -
          2.68623  4.42230  2.77524  2.73128  3.46359  2.40517  3.72499
3.29279  2.67745  2.69360  4.24694  2.90351  2.73744  3.18151  2.89805
2.37891  2.77509  2.98504  4.58482  3.61508
          0.15013  2.01920  5.01389  0.66505  0.72206  0.48576  0.95510
     88   2.38356  4.85902  2.93559  2.52702  4.38255  2.23206  3.80244
3.82575  2.38760  3.39690  4.19305  2.55795  3.86702  2.94927  3.06736
1.82252  2.93907  3.41543  5.59777  4.24954     97 s - - -
          2.68618  4.42225  2.77519  2.73123  3.46354  2.40513  3.72494
3.29354  2.67741  2.69355  4.24690  2.90347  2.73739  3.18146  2.89801
2.37887  2.77519  2.98518  4.58477  3.61503
          0.02054  4.29155  5.01389  0.61958  0.77255  0.48576  0.95510
     89   2.08173  5.14900  2.49252  1.87111  4.46011  2.89063  3.66140
3.92824  2.28699  3.27588  4.20836  2.89406  3.84701  2.71423  2.93454
2.67211  2.94254  3.52298  5.60283  4.20091     98 e - - -
          2.68618  4.42225  2.77519  2.73123  3.46354  2.40513  3.72494
3.29354  2.67741  2.69355  4.24690  2.90347  2.73739  3.18146  2.89801
2.37887  2.77519  2.98518  4.58477  3.61503
          0.02054  4.29155  5.01389  0.61958  0.77255  0.48576  0.95510
     90   2.60975  4.93112  2.32643  2.17517  4.18978  3.45226  3.52507
2.63742  2.40047  2.79117  3.99684  2.96325  3.85202  2.54796  2.91810
2.66744  2.88774  3.27391  5.41738  4.06368     99 e - - -
          2.68618  4.42225  2.77519  2.73123  3.46354  2.40513  3.72494
3.29354  2.67741  2.69355  4.24690  2.90347  2.73739  3.18146  2.89801
2.37887  2.77519  2.98518  4.58477  3.61503
          0.02054  4.29155  5.01389  0.61958  0.77255  0.48576  0.95510
     91   2.64959  4.87434  2.60059  2.39438  4.12075  3.11466  3.67493
3.53994  2.41450  2.54418  3.94694  2.39130  3.85869  2.82162  2.94159
2.67560  2.69743  2.65259  5.37478  4.03279    100 n - - -
          2.68618  4.42225  2.77519  2.73123  3.46354  2.40513  3.72494
3.29354  2.67741  2.69355  4.24690  2.90347  2.73739  3.18146  2.89801
2.37887  2.77519  2.98518  4.58477  3.61503
          0.02054  4.29155  5.01389  0.61958  0.77255  0.48576  0.95510
     92   2.64157  5.17216  2.56247  1.99040  4.48451  3.41452  3.68098
3.95303  2.46966  3.46712  4.23996  2.89339  2.16558  2.57650  2.88950
2.40098  2.97128  3.54883  5.63092  4.22679    101 e - - -
          2.68627  4.42234  2.77528  2.73086  3.46363  2.40505  3.72503
3.29283  2.67728  2.69364  4.24698  2.90355  2.73748  3.18155  2.89809
2.37895  2.77528  2.98527  4.58486  3.61512
          0.09794  2.44582  5.01389  1.35643  0.29784  0.48576  0.95510
     93   2.65291  4.85872  3.03405  2.48492  4.10593  3.29947  3.67544
3.52248  2.31170  3.12077  3.93329  2.48957  3.86583  2.62603  2.82208
2.68419  2.06274  2.78913  5.35826  3.81670    108 t - - -
          2.68618  4.42225  2.77519  2.73123  3.46354  2.40513  3.72494
3.29354  2.67741  2.69355  4.24690  2.90347  2.73739  3.18146  2.89801
2.37887  2.77519  2.98518  4.58477  3.61503
          0.02054  4.29155  5.01389  0.61958  0.77255  0.48576  0.95510
     94   3.41327  4.73773  4.97238  4.43396  1.20882  4.57920  4.55956
2.47076  4.28217  1.47567  2.44712  4.56464  4.79307  4.33976  4.35604
3.92112  3.63212  2.59960  4.79740  3.40125    109 f - - -
          2.68618  4.42225  2.77519  2.73123  3.46354  2.40513  3.72494
3.29354  2.67741  2.69355  4.24690  2.90347  2.73739  3.18146  2.89801
2.37887  2.77519  2.98518  4.58477  3.61503
          0.02054  4.29155  5.01389  0.61958  0.77255  0.48576  0.95510
```

```
     95   2.60521  4.98327  2.96519  2.44045  3.89560  3.46417  2.46971
3.69470  2.41832  3.25245  4.04967  2.44864  3.86438  2.07700  2.87321
2.68410  2.91256  3.33983  5.40839  3.79135    110 q - - -
          2.68618  4.42225  2.77519  2.73123  3.46354  2.40513  3.72494
3.29354  2.67741  2.69355  4.24690  2.90347  2.73739  3.18146  2.89801
2.37887  2.77519  2.98518  4.58477  3.61503
          0.04118  4.29155  3.62474  0.61958  0.77255  0.48576  0.95510
     96   3.51909  5.29168  4.05598  3.50529  4.75202  3.90442  4.19946
4.33924  2.51137  3.78228  4.78752  3.82597  4.40177  3.41369  0.50997
3.59408  3.76676  4.04941  5.68051  4.61060    111 R - - -
          2.68618  4.42225  2.77519  2.73123  3.46354  2.40513  3.72494
3.29354  2.67741  2.69355  4.24690  2.90347  2.73739  3.18146  2.89801
2.37887  2.77519  2.98518  4.58477  3.61503
          0.02096  4.27133  4.99368  0.61958  0.77255  0.46900  0.98251
     97   3.54915  5.31634  4.08864  3.53577  4.78244  3.92787  4.22346
4.37301  2.53153  3.81225  4.81842  3.85408  4.42560  3.43802  0.49246
3.62417  3.79628  4.08260  5.70333  4.63846    112 R - - -
          2.68618  4.42225  2.77519  2.73123  3.46354  2.40513  3.72494
3.29354  2.67741  2.69355  4.24690  2.90347  2.73739  3.18146  2.89801
2.37887  2.77519  2.98518  4.58477  3.61503
          0.02054  4.29155  5.01389  0.61958  0.77255  0.48576  0.95510
     98   2.65784  2.58190  4.21142  3.65318  3.37971  3.72364  4.21736
1.70751  3.53246  2.39719  3.36186  3.82105  4.16545  3.76233  3.71706
2.28390  2.83366  2.06020  4.92130  3.72811    113 i - - -
          2.68618  4.42225  2.77519  2.73123  3.46354  2.40513  3.72494
3.29354  2.67741  2.69355  4.24690  2.90347  2.73739  3.18146  2.89801
2.37887  2.77519  2.98518  4.58477  3.61503
          0.02054  4.29155  5.01389  0.61958  0.77255  0.48576  0.95510
     99   3.92766  5.17282  4.72242  4.47810  2.04373  4.55508  3.66766
3.66790  4.31023  2.97481  4.28052  4.25641  4.90812  4.33367  4.35772
3.98688  4.17228  3.59280  3.80839  0.59099    114 Y - - -
          2.68618  4.42225  2.77519  2.73123  3.46354  2.40513  3.72494
3.29354  2.67741  2.69355  4.24690  2.90347  2.73739  3.18146  2.89801
2.37887  2.77519  2.98518  4.58477  3.61503
          0.02054  4.29155  5.01389  0.61958  0.77255  0.48576  0.95510
    100   2.65312  3.83158  3.65352  3.08402  2.93295  3.64921  3.89321
2.79803  2.41743  2.51443  3.42203  3.44386  4.03249  3.29877  3.29736
2.57208  2.88824  2.58259  2.18156  3.54469    115 w - - -
          2.68618  4.42225  2.77519  2.73123  3.46354  2.40513  3.72494
3.29354  2.67741  2.69355  4.24690  2.90347  2.73739  3.18146  2.89801
2.37887  2.77519  2.98518  4.58477  3.61503
          0.02054  4.29155  5.01389  0.61958  0.77255  0.48576  0.95510
    101   3.05552  4.43403  4.70326  4.11305  2.86911  4.24073  4.54226
2.20858  3.97402  1.13816  2.26750  4.28768  4.51788  3.65020  4.07516
3.55379  3.27880  2.12191  4.98930  3.87972    116 l - - -
          2.68618  4.42225  2.77519  2.73123  3.46354  2.40513  3.72494
3.29354  2.67741  2.69355  4.24690  2.90347  2.73739  3.18146  2.89801
2.37887  2.77519  2.98518  4.58477  3.61503
          0.02054  4.29155  5.01389  0.61958  0.77255  0.48576  0.95510
    102   2.71527  4.26387  3.92670  3.35149  2.75567  3.77268  4.04544
2.47605  3.24292  1.48768  3.25913  3.65242  2.92992  3.51167  3.11192
3.04897  2.94738  2.46456  4.83042  3.61575    117 l - - -
          2.68618  4.42225  2.77519  2.73123  3.46354  2.40513  3.72494
3.29354  2.67741  2.69355  4.24690  2.90347  2.73739  3.18146  2.89801
2.37887  2.77519  2.98518  4.58477  3.61503
          0.02054  4.29155  5.01389  0.61958  0.77255  0.48576  0.95510
```

```
    103   2.91288  5.48302  1.53385  1.79014  4.78946  3.38311  3.78531
4.29759  2.67586  3.77310  4.56270  2.70733  2.95338  2.49127  3.22598
2.82597  3.17233  3.85833  5.91752  4.44740    118 d - - -
          2.68618  4.42225  2.77519  2.73123  3.46354  2.40513  3.72494
3.29354  2.67741  2.69355  4.24690  2.90347  2.73739  3.18146  2.89801
2.37887  2.77519  2.98518  4.58477  3.61503
          0.02054  4.29155  5.01389  0.61958  0.77255  0.48576  0.95510
    104   2.64124  4.34874  2.61076  2.26397  4.38416  3.19721  3.61795
3.84796  2.32037  3.36282  3.86039  2.68196  3.01643  2.51679  2.78287
2.16736  2.87272  3.44462  5.52400  4.13328    119 s - - -
          2.68618  4.42225  2.77519  2.73123  3.46354  2.40513  3.72494
3.29354  2.67741  2.69355  4.24690  2.90347  2.73739  3.18146  2.89801
2.37887  2.77519  2.98518  4.58477  3.61503
          0.02054  4.29155  5.01389  0.61958  0.77255  0.48576  0.95510
    105   2.40857  5.15235  2.37421  2.06301  4.47705  2.90745  3.51914
3.95240  2.41186  3.45228  4.20884  2.69185  3.12522  2.75309  2.90956
2.38722  2.75853  3.53415  5.60284  4.19582    120 e - - -
          2.68623  4.42230  2.77524  2.73128  3.46349  2.40517  3.72484
3.29359  2.67739  2.69339  4.24694  2.90351  2.73744  3.18151  2.89805
2.37891  2.77524  2.98523  4.58482  3.61422
          0.66820  1.39245  1.43168  0.19109  1.74902  0.48576  0.95510
    106   2.53694  5.02329  2.37555  1.96804  4.32496  3.22760  3.58214
3.78350  2.35241  3.31010  3.73472  2.83777  3.77235  2.50102  2.76288
2.53778  2.77371  3.39154  5.48270  4.09325    122 e - - -
          2.68618  4.42225  2.77519  2.73123  3.46354  2.40513  3.72494
3.29354  2.67741  2.69355  4.24690  2.90347  2.73739  3.18146  2.89801
2.37887  2.77519  2.98518  4.58477  3.61503
          0.02684  4.02724  4.74959  0.61958  0.77255  0.47028  0.98037
    107   2.62937  4.25508  2.66535  2.36332  4.31205  3.15025  2.48397
3.76492  2.36744  3.29909  4.07089  2.69098  3.80984  2.45632  2.69818
2.47208  2.86154  3.37990  5.47678  4.09793    123 e - - -
          2.68618  4.42225  2.77519  2.73123  3.46354  2.40513  3.72494
3.29354  2.67741  2.69355  4.24690  2.90347  2.73739  3.18146  2.89801
2.37887  2.77519  2.98518  4.58477  3.61503
          0.02235  4.20793  4.93027  0.61958  0.77255  0.42497  1.06071
    108   2.60438  4.20804  3.99443  3.42244  3.16035  3.76441  3.24561
1.60888  3.29594  2.29472  3.30159  3.67643  4.13335  3.55573  3.51712
3.04776  2.92752  2.34770  4.69797  2.58304    124 i - - -
          2.68618  4.42225  2.77519  2.73123  3.46354  2.40513  3.72494
3.29354  2.67741  2.69355  4.24690  2.90347  2.73739  3.18146  2.89801
2.37887  2.77519  2.98518  4.58477  3.61503
          0.02054  4.29155  5.01389  0.61958  0.77255  0.48576  0.95510
    109   2.82422  4.26596  4.30548  3.75493  2.54209  3.92635  4.11313
2.45507  3.62346  2.30725  3.34151  3.91431  4.29444  3.82969  3.78513
3.09398  3.06503  1.23894  4.63466  2.84599    125 v - - -
          2.68618  4.42225  2.77519  2.73123  3.46354  2.40513  3.72494
3.29354  2.67741  2.69355  4.24690  2.90347  2.73739  3.18146  2.89801
2.37887  2.77519  2.98518  4.58477  3.61503
          0.03538  4.29155  3.85964  0.61958  0.77255  0.48576  0.95510
    110   3.59954  4.88757  5.13843  4.63635  2.12425  4.77259  4.75440
2.44433  4.48406  0.72778  3.01543  4.78361  4.94105  4.50016  4.53391
4.15899  3.81909  2.63666  4.91288  3.54000    126 l - - -
          2.68618  4.42225  2.77519  2.73123  3.46354  2.40513  3.72494
3.29354  2.67741  2.69355  4.24690  2.90347  2.73739  3.18146  2.89801
2.37887  2.77519  2.98518  4.58477  3.61503
          0.02084  4.27701  4.99936  0.61958  0.77255  0.50560  0.92418
```

```
    111   3.14494  4.59788  4.67372  4.36746  3.88501  4.13019  5.11246
2.12340  4.24332  2.51178  3.80444  4.48853  4.68156  4.58212  4.41514
3.67402  3.50480  0.63112  5.73158  4.48170    127 V - - -
          2.68618  4.42225  2.77519  2.73123  3.46354  2.40513  3.72494
3.29354  2.67741  2.69355  4.24690  2.90347  2.73739  3.18146  2.89801
2.37887  2.77519  2.98518  4.58477  3.61503
          0.02084  4.27701  4.99936  0.61958  0.77255  0.50560  0.92418
    112   3.16121  5.16046  3.10485  2.86385  3.66166  3.66639  0.97920
3.98014  2.64918  3.45707  4.43543  3.31359  4.19114  2.95325  2.92385
3.18228  3.43065  3.69051  5.10480  3.57279    128 h - - -
          2.68618  4.42225  2.77519  2.73123  3.46354  2.40513  3.72494
3.29354  2.67741  2.69355  4.24690  2.90347  2.73739  3.18146  2.89801
2.37887  2.77519  2.98518  4.58477  3.61503
          0.02084  4.27701  4.99936  0.61958  0.77255  0.50560  0.92418
    113   3.88413  5.20643  4.46291  4.28437  2.43172  4.32289  3.88689
3.84508  4.13286  3.18944  4.47923  4.25856  4.79448  4.33574  4.22516
3.97252  4.17680  3.73660  4.08506  0.49092    129 Y - - -
          2.68618  4.42225  2.77519  2.73123  3.46354  2.40513  3.72494
3.29354  2.67741  2.69355  4.24690  2.90347  2.73739  3.18146  2.89801
2.37887  2.77519  2.98518  4.58477  3.61503
          0.02084  4.27701  4.99936  0.61958  0.77255  0.50560  0.92418
    114   2.93945  4.51960  4.00949  3.43273  2.86457  3.94707  4.13027
2.67268  3.16515  1.21354  3.28273  3.76265  4.29229  3.55579  2.36238
3.24187  3.16816  2.62323  4.91323  3.62343    130 l - - -
          2.68618  4.42225  2.77519  2.73123  3.46354  2.40513  3.72494
3.29354  2.67741  2.69355  4.24690  2.90347  2.73739  3.18146  2.89801
2.37887  2.77519  2.98518  4.58477  3.61503
          0.04260  4.27701  3.58183  0.61958  0.77255  0.50560  0.92418
    115   2.75639  5.27530  2.22671  2.02456  4.60006  3.39242  3.46142
4.08909  2.20234  3.56977  4.33183  2.00381  3.85330  2.60115  2.97616
2.70018  2.87551  3.65704  5.70807  4.27982    131 n - - -
          2.68618  4.42225  2.77519  2.73123  3.46354  2.40513  3.72494
3.29354  2.67741  2.69355  4.24690  2.90347  2.73739  3.18146  2.89801
2.37887  2.77519  2.98518  4.58477  3.61503
          0.24576  4.25570  1.59106  0.61958  0.77255  0.53349  0.88322
    116   2.56640  4.38375  3.32117  2.53838  3.61719  3.47652  3.81753
2.74513  2.75818  2.64959  3.55085  3.22279  3.90581  3.09217  3.13327
2.51142  2.36351  1.99802  5.04280  3.79098    132 v - - -
          2.68618  4.42225  2.77519  2.73123  3.46354  2.40513  3.72494
3.29354  2.67741  2.69355  4.24690  2.90347  2.73739  3.18146  2.89801
2.37887  2.77519  2.98518  4.58477  3.61503
          0.01797  4.02791        *  0.61958  0.77255  0.00000        *
//
HMMER3/f [3.3 | Nov 2019]
NAME  CSD
ACC   PF00313.23
DESC  'Cold-shock' DNA-binding domain
LENG  66
ALPH  amino
RF    no
MM    no
CONS  yes
CS    yes
MAP   yes
DATE  Mon Apr 20 17:02:05 2020
NSEQ  34
EFFN  2.809814
CKSUM 2228867964
```

```
GA    20.80 20.80
TC    20.80 20.80
NC    20.70 20.70
STATS LOCAL MSV       -8.7493  0.71880
STATS LOCAL VITERBI   -9.1249  0.71880
STATS LOCAL FORWARD   -4.3329  0.71880
HMM          A        C        D        E        F        G        H
I        K        L        M        N        P        Q        R        S
T        V        W        Y
            m->m     m->i     m->d     i->m     i->i     d->m     d->d
  COMPO   2.62108  4.64282  2.92850  2.58388  2.81465  2.56079  3.48201
2.90289  2.55941  2.78142  4.01901  3.03916  3.83142  2.96182  3.04229
2.74201  2.91423  2.45586  4.75077  3.63020
          2.68618  4.42225  2.77519  2.73123  3.46354  2.40513  3.72494
3.29354  2.67741  2.69355  4.24690  2.90347  2.73739  3.18146  2.89801
2.37887  2.77519  2.98518  4.58477  3.61503
          0.13757  4.87608  2.11278  0.61958  0.77255  0.00000        *
      1   2.50697  3.63033  3.65543  2.48332  3.37235  3.76361  4.01600
3.01193  2.89784  2.24672  2.42974  3.49630  4.14311  2.99027  2.63537
2.82444  3.01813  2.25035  5.09369  3.85837      1 l - - E
          2.68618  4.42225  2.77519  2.73123  3.46354  2.40513  3.72494
3.29354  2.67741  2.69355  4.24690  2.90347  2.73739  3.18146  2.89801
2.37887  2.77519  2.98518  4.58477  3.61503
          0.01292  4.75143  5.47377  0.61958  0.77255  0.35357  1.21126
      2   2.83426  5.19881  3.14647  2.58849  4.48987  3.63119  3.82213
3.59235  2.13107  3.07051  4.25460  2.78441  4.02263  2.26217  2.47526
2.72947  1.88147  3.38304  5.66403  3.56905      2 t - - E
          2.68618  4.42225  2.77519  2.73123  3.46354  2.40513  3.72494
3.29354  2.67741  2.69355  4.24690  2.90347  2.73739  3.18146  2.89801
2.37887  2.77519  2.98518  4.58477  3.61503
          0.01140  4.87608  5.59843  0.61958  0.77255  0.48576  0.95510
      3   2.22812  4.84920  4.41817  4.39535  5.48279  0.37732  5.42686
4.96702  4.63267  4.67657  5.48701  4.26596  4.44149  4.80273  4.78850
3.13751  3.50764  4.17139  6.75628  5.69956      3 G - - E
          2.68618  4.42225  2.77519  2.73123  3.46354  2.40513  3.72494
3.29354  2.67741  2.69355  4.24690  2.90347  2.73739  3.18146  2.89801
2.37887  2.77519  2.98518  4.58477  3.61503
          0.01140  4.87608  5.59843  0.61958  0.77255  0.48576  0.95510
      4   2.84011  4.55212  3.73202  3.16373  3.68773  3.82288  4.07682
2.45347  2.50012  2.52736  3.65581  3.56531  4.20188  3.12976  3.31668
2.61311  1.71825  2.22532  5.13499  3.28809      4 t - - E
          2.68618  4.42225  2.77519  2.73123  3.46354  2.40513  3.72494
3.29354  2.67741  2.69355  4.24690  2.90347  2.73739  3.18146  2.89801
2.37887  2.77519  2.98518  4.58477  3.61503
          0.01140  4.87608  5.59843  0.61958  0.77255  0.48576  0.95510
      5   3.59076  3.43961  5.67576  5.20630  4.26315  5.08261  5.86276
1.81023  5.12376  2.77981  4.03084  5.30502  5.39190  5.37466  5.27158
4.50744  3.86633  0.51207  6.26225  5.03307      5 V - - E
          2.68618  4.42225  2.77519  2.73123  3.46354  2.40513  3.72494
3.29354  2.67741  2.69355  4.24690  2.90347  2.73739  3.18146  2.89801
2.37887  2.77519  2.98518  4.58477  3.61503
          0.01140  4.87608  5.59843  0.61958  0.77255  0.48576  0.95510
      6   2.64694  3.89809  3.17954  2.11342  4.66900  3.68565  3.87602
4.11547  1.10401  3.62648  4.40110  2.96969  4.09228  2.99079  2.98342
2.91754  3.16761  3.72040  5.77592  4.41353      6 k - - E
          2.68618  4.42225  2.77519  2.73123  3.46354  2.40513  3.72494
3.29354  2.67741  2.69355  4.24690  2.90347  2.73739  3.18146  2.89801
2.37887  2.77519  2.98518  4.58477  3.61503
```

```
         0.01140  4.87608  5.59843  0.61958  0.77255  0.48576  0.95510
      7  2.59772  3.19970  4.05857  3.49149  3.66199  3.84843  4.23607
3.01464  2.78370  2.74966  3.65795  3.78304  4.28013  3.66164  3.60138
3.14800  2.32346  2.79386  1.33335  3.92374      7 w - - E
         2.68618  4.42225  2.77519  2.73123  3.46354  2.40513  3.72494
3.29354  2.67741  2.69355  4.24690  2.90347  2.73739  3.18146  2.89801
2.37887  2.77519  2.98518  4.58477  3.61503
         0.01140  4.87608  5.59843  0.61958  0.77255  0.48576  0.95510
      8  3.36336  4.70230  5.16992  4.59986  0.99361  4.52711  4.60825
2.80833  4.41585  1.77158  2.92662  4.60658  4.82230  4.47983  4.43093
3.85099  3.58770  2.33021  4.92233  2.83416      8 f - - E
         2.68618  4.42225  2.77519  2.73123  3.46354  2.40513  3.72494
3.29354  2.67741  2.69355  4.24690  2.90347  2.73739  3.18146  2.89801
2.37887  2.77519  2.98518  4.58477  3.61503
         0.01140  4.87608  5.59843  0.61958  0.77255  0.48576  0.95510
      9  2.92633  5.41014  2.26104  2.55326  4.73978  3.63380  3.87239
4.21919  2.03314  3.03548  4.46651  1.50561  4.06268  2.98170  3.12892
2.45616  3.16334  3.79514  5.85270  4.44205      9 n - - E
         2.68618  4.42225  2.77519  2.73123  3.46354  2.40513  3.72494
3.29354  2.67741  2.69355  4.24690  2.90347  2.73739  3.18146  2.89801
2.37887  2.77519  2.98518  4.58477  3.61503
         0.01140  4.87608  5.59843  0.61958  0.77255  0.48576  0.95510
     10  2.10003  5.27798  2.28073  2.34320  4.59743  3.17108  3.80401
4.06576  2.46333  3.57255  4.32627  2.71259  4.00606  2.90965  2.81955
2.62057  2.56149  2.39420  5.72689  4.33162     10 a - - T
         2.68618  4.42225  2.77519  2.73123  3.46354  2.40513  3.72494
3.29354  2.67741  2.69355  4.24690  2.90347  2.73739  3.18146  2.89801
2.37887  2.77519  2.98518  4.58477  3.61503
         0.21924  4.87608  1.66470  0.61958  0.77255  0.48576  0.95510
     11  2.37617  5.25639  2.57124  1.95026  4.59159  3.37079  3.72785
4.07149  1.85915  3.00866  4.30075  3.00253  3.93102  2.66176  2.96123
2.35547  2.99840  3.64193  5.69426  4.28767     11 k - - T
         2.68618  4.42225  2.77519  2.73123  3.46354  2.40513  3.72494
3.29354  2.67741  2.69355  4.24690  2.90347  2.73739  3.18146  2.89801
2.37887  2.77519  2.98518  4.58477  3.61503
         0.01401  4.67084  5.39319  0.61958  0.77255  0.86572  0.54602
     12  2.71473  5.31232  3.22775  2.66740  4.70444  3.67691  3.81414
4.13633  1.19727  3.61542  4.39888  2.86666  4.07421  2.92764  2.39780
2.23933  3.16809  3.74101  5.72884  4.40394     12 k - - T
         2.68618  4.42225  2.77519  2.73123  3.46354  2.40513  3.72494
3.29354  2.67741  2.69355  4.24690  2.90347  2.73739  3.18146  2.89801
2.37887  2.77519  2.98518  4.58477  3.61503
         0.01401  4.67084  5.39319  0.61958  0.77255  0.30536  1.33505
     13  2.65598  5.25437  3.15100  2.69396  4.67725  1.24195  3.95853
4.12864  2.70278  3.66162  4.44296  2.82185  4.10968  2.59460  2.70870
2.53355  3.19014  3.72287  5.83187  4.46676     13 g - - T
         2.68618  4.42225  2.77519  2.73123  3.46354  2.40513  3.72494
3.29354  2.67741  2.69355  4.24690  2.90347  2.73739  3.18146  2.89801
2.37887  2.77519  2.98518  4.58477  3.61503
         0.01140  4.87608  5.59843  0.61958  0.77255  0.48576  0.95510
     14  4.22486  5.46972  5.19884  4.86699  0.89463  4.97311  4.05180
4.03724  2.74008  3.41407  4.61592  4.63768  5.28280  4.67813  4.67361
4.33276  4.43964  3.91567  4.20396  1.14990     14 f - - E
         2.68618  4.42225  2.77519  2.73123  3.46354  2.40513  3.72494
3.29354  2.67741  2.69355  4.24690  2.90347  2.73739  3.18146  2.89801
2.37887  2.77519  2.98518  4.58477  3.61503
         0.01140  4.87608  5.59843  0.61958  0.77255  0.48576  0.95510
```

```
     15   4.28989  5.86342  5.13151  5.17220  6.11447  0.09285  6.11164
6.05588  5.46131  5.51774  6.56989  5.30007  5.25843  5.71159  5.49451
4.50189  4.82999  5.40615  6.85651  6.27972      15 G - - E
          2.68618  4.42225  2.77519  2.73123  3.46354  2.40513  3.72494
3.29354  2.67741  2.69355  4.24690  2.90347  2.73739  3.18146  2.89801
2.37887  2.77519  2.98518  4.58477  3.61503
          0.01140  4.87608  5.59843  0.61958  0.77255  0.48576  0.95510
     16   4.46832  5.62291  5.68773  5.46669  0.40285  5.25674  4.45399
3.49244  5.26265  2.25775  4.08480  5.10605  5.53069  5.11236  5.17129
4.74069  4.71198  3.63363  4.53600  2.83959      16 F - - E
          2.68618  4.42225  2.77519  2.73123  3.46354  2.40513  3.72494
3.29354  2.67741  2.69355  4.24690  2.90347  2.73739  3.18146  2.89801
2.37887  2.77519  2.98518  4.58477  3.61503
          0.01140  4.87608  5.59843  0.61958  0.77255  0.48576  0.95510
     17   3.77064  4.99060  5.80332  5.38654  4.20916  5.29819  6.14745
0.46747  5.29821  2.58172  3.95376  5.53249  5.56483  5.54869  5.45184
4.77396  4.05009  1.80141  6.39918  5.18624      17 I - - E
          2.68618  4.42225  2.77519  2.73123  3.46354  2.40513  3.72494
3.29354  2.67741  2.69355  4.24690  2.90347  2.73739  3.18146  2.89801
2.37887  2.77519  2.98518  4.58477  3.61503
          0.01140  4.87608  5.59843  0.61958  0.77255  0.48576  0.95510
     18   2.52072  4.32710  3.15592  2.10298  4.44064  3.63252  3.83077
3.05730  2.59924  3.43886  4.22180  2.31085  4.02534  2.61839  3.07709
2.38844  1.97618  3.51617  5.63772  4.27148      18 t - - E
          2.68618  4.42225  2.77519  2.73123  3.46354  2.40513  3.72494
3.29354  2.67741  2.69355  4.24690  2.90347  2.73739  3.18146  2.89801
2.37887  2.77519  2.98518  4.58477  3.61503
          0.01140  4.87608  5.59843  0.61958  0.77255  0.48576  0.95510
     19   2.70183  3.63388  2.85981  2.59370  4.45561  3.41355  3.82574
3.89935  2.50936  3.45121  4.23113  3.12851  2.13090  2.60539  2.06489
2.82648  2.77425  3.35653  5.64529  3.29427      19 r - - E
          2.68618  4.42225  2.77519  2.73123  3.46354  2.40513  3.72494
3.29354  2.67741  2.69355  4.24690  2.90347  2.73739  3.18146  2.89801
2.37887  2.77519  2.98518  4.58477  3.61503
          0.01140  4.87608  5.59843  0.61958  0.77255  0.48576  0.95510
     20   2.49009  5.35266  2.16873  1.81055  4.69354  3.19961  3.80557
4.17834  2.54890  3.65664  4.39640  2.85180  3.73805  2.63909  3.04376
2.05522  2.86320  3.53171  5.78801  4.37628      20 e - - T
          2.68618  4.42225  2.77519  2.73123  3.46354  2.40513  3.72494
3.29354  2.67741  2.69355  4.24690  2.90347  2.73739  3.18146  2.89801
2.37887  2.77519  2.98518  4.58477  3.61503
          0.01140  4.87608  5.59843  0.61958  0.77255  0.48576  0.95510
     21   2.88878  5.33621  1.48873  2.23395  4.65154  2.69622  3.85339
3.44417  2.62265  3.63087  4.39231  2.64126  4.04367  2.96503  3.11799
2.63835  3.12454  3.00298  5.78985  4.38895      21 d - - T
          2.68618  4.42225  2.77519  2.73123  3.46354  2.40513  3.72494
3.29354  2.67741  2.69355  4.24690  2.90347  2.73739  3.18146  2.89801
2.37887  2.77519  2.98518  4.58477  3.61503
          0.01140  4.87608  5.59843  0.61958  0.77255  0.48576  0.95510
     22   2.85225  5.07964  3.22056  2.59520  4.34561  1.55454  3.29315
3.76730  2.65890  3.35801  4.16498  3.06390  4.06361  3.02330  2.71217
2.78300  2.27535  2.89913  5.58792  4.24857      22 g - - S
          2.68618  4.42225  2.77519  2.73123  3.46354  2.40513  3.72494
3.29354  2.67741  2.69355  4.24690  2.90347  2.73739  3.18146  2.89801
2.37887  2.77519  2.98518  4.58477  3.61503
          0.01140  4.87608  5.59843  0.61958  0.77255  0.48576  0.95510
```

```
     23   2.83492  5.33996  2.17936  2.30982  4.68181  2.63072  3.79519
4.16616  2.53346  3.64387  4.38211  2.57209  3.99988  2.57649  2.29375
2.21658  2.93795  3.28700  5.77430  4.36410      23 d - - S
          2.68618  4.42225  2.77519  2.73123  3.46354  2.40513  3.72494
3.29354  2.67741  2.69355  4.24690  2.90347  2.73739  3.18146  2.89801
2.37887  2.77519  2.98518  4.58477  3.61503
          0.13306  4.87608  2.14593  0.61958  0.77255  0.48576  0.95510
     24   2.21026  5.23792  2.85628  2.01130  4.56072  3.38743  3.75521
3.40174  1.80648  3.53391  4.28523  3.04160  3.49283  2.85947  2.98821
2.43595  2.82450  3.33166  5.68487  4.28800      24 k - - -
          2.68620  4.42227  2.77522  2.73125  3.46356  2.40515  3.72496
3.29356  2.67711  2.69357  4.24692  2.90349  2.73742  3.18148  2.89803
2.37889  2.77522  2.98520  4.58479  3.61505
          0.08271  2.58750  5.47823  0.30701  1.33046  0.35682  1.20363
     25   3.22781  5.80276  0.97710  1.89822  5.12488  3.70105  4.07974
4.63062  2.42275  4.09006  4.87802  3.15314  4.23075  3.21022  2.75914
3.13914  3.48252  4.18666  6.21758  4.75914      26 d - - E
          2.68618  4.42225  2.77519  2.73123  3.46354  2.40513  3.72494
3.29354  2.67741  2.69355  4.24690  2.90347  2.73739  3.18146  2.89801
2.37887  2.77519  2.98518  4.58477  3.61503
          0.01140  4.87608  5.59843  0.61958  0.77255  0.48576  0.95510
     26   3.75837  4.95513  5.84007  5.34703  4.03956  5.34030  5.95767
1.46975  5.27045  1.41888  3.78009  5.49979  5.51481  5.41511  5.37012
4.76129  4.00647  0.95573  6.17448  5.04125      27 v - - E
          2.68618  4.42225  2.77519  2.73123  3.46354  2.40513  3.72494
3.29354  2.67741  2.69355  4.24690  2.90347  2.73739  3.18146  2.89801
2.37887  2.77519  2.98518  4.58477  3.61503
          0.01140  4.87608  5.59843  0.61958  0.77255  0.48576  0.95510
     27   4.08133  5.49456  4.75999  4.53231  0.44724  4.74210  4.30970
3.89803  4.36203  3.15479  4.46219  4.57350  5.17398  2.70618  4.48815
4.23270  4.35813  3.86339  4.54533  2.84778      28 F - - E
          2.68618  4.42225  2.77519  2.73123  3.46354  2.40513  3.72494
3.29354  2.67741  2.69355  4.24690  2.90347  2.73739  3.18146  2.89801
2.37887  2.77519  2.98518  4.58477  3.61503
          0.01140  4.87608  5.59843  0.61958  0.77255  0.48576  0.95510
     28   2.59284  3.94126  5.00046  4.42002  2.04278  4.32646  4.71744
2.40790  4.24912  2.27079  3.55520  4.46950  4.67922  4.40921  4.31141
3.65480  3.34885  0.99843  5.21516  4.02345      29 v - - E
          2.68618  4.42225  2.77519  2.73123  3.46354  2.40513  3.72494
3.29354  2.67741  2.69355  4.24690  2.90347  2.73739  3.18146  2.89801
2.37887  2.77519  2.98518  4.58477  3.61503
          0.01140  4.87608  5.59843  0.61958  0.77255  0.48576  0.95510
     29   4.70121  6.13353  4.72229  4.65426  4.57186  4.71657  0.14301
5.60960  4.52114  4.90906  6.10292  4.98873  5.34507  5.06341  4.67253
4.80161  5.07551  5.31548  5.96037  4.51621      30 H - - E
          2.68618  4.42225  2.77519  2.73123  3.46354  2.40513  3.72494
3.29354  2.67741  2.69355  4.24690  2.90347  2.73739  3.18146  2.89801
2.37887  2.77519  2.98518  4.58477  3.61503
          0.01140  4.87608  5.59843  0.61958  0.77255  0.48576  0.95510
     30   2.86824  3.25483  4.54452  3.94973  2.11875  4.00031  3.88855
2.53384  3.79178  2.48004  3.40790  4.05986  4.36621  2.12094  3.89734
3.29885  3.10020  2.08289  4.17618  2.25433      31 v - - T
          2.68618  4.42225  2.77519  2.73123  3.46354  2.40513  3.72494
3.29354  2.67741  2.69355  4.24690  2.90347  2.73739  3.18146  2.89801
2.37887  2.77519  2.98518  4.58477  3.61503
          0.01140  4.87608  5.59843  0.61958  0.77255  0.48576  0.95510
```

```
     31   2.94854  5.13594  3.39252  2.88861  4.64632  3.69433  4.05074
4.06866  2.24404  3.63201  4.43748  3.36233  4.17358  3.19503  2.92599
1.04649  2.26308  3.67749  5.81591  4.50571      32 s - - T
          2.68618  4.42225  2.77519  2.73123  3.46354  2.40513  3.72494
3.29354  2.67741  2.69355  4.24690  2.90347  2.73739  3.18146  2.89801
2.37887  2.77519  2.98518  4.58477  3.61503
          0.01140  4.87608  5.59843  0.61958  0.77255  0.48576  0.95510
     32   1.56801  5.31590  2.91992  1.94836  4.64408  3.44491  3.81713
4.11809  2.56748  3.61615  4.36689  2.71001  4.01580  2.58903  3.05938
2.46259  2.92923  3.41237  5.76361  4.36168      33 a - - T
          2.68618  4.42225  2.77519  2.73123  3.46354  2.40513  3.72494
3.29354  2.67741  2.69355  4.24690  2.90347  2.73739  3.18146  2.89801
2.37887  2.77519  2.98518  4.58477  3.61503
          0.01140  4.87608  5.59843  0.61958  0.77255  0.48576  0.95510
     33   3.23369  4.56873  5.15754  4.56805  2.38200  4.45628  4.74555
1.03656  4.38206  1.89850  3.42636  4.59301  4.76634  4.48370  4.40165
3.78240  3.46417  2.12338  5.14238  2.99112      34 i - - S
          2.68618  4.42225  2.77519  2.73123  3.46354  2.40513  3.72494
3.29354  2.67741  2.69355  4.24690  2.90347  2.73739  3.18146  2.89801
2.37887  2.77519  2.98518  4.58477  3.61503
          0.01140  4.87608  5.59843  0.61958  0.77255  0.48576  0.95510
     34   2.58995  5.30109  3.09125  2.31467  4.63093  3.60772  3.79624
3.60140  2.38106  3.59955  4.34610  2.19035  4.00105  1.86706  2.84179
2.25442  2.83729  3.39342  5.74278  4.34202      35 q - - -
          2.68618  4.42225  2.77519  2.73123  3.46354  2.40513  3.72494
3.29354  2.67741  2.69355  4.24690  2.90347  2.73739  3.18146  2.89801
2.37887  2.77519  2.98518  4.58477  3.61503
          0.01140  4.87608  5.59843  0.61958  0.77255  0.48576  0.95510
     35   2.65924  5.26886  2.69093  2.54740  4.58625  2.29430  3.80187
4.05308  2.33168  3.14116  2.78414  2.78594  3.50880  2.83080  2.67311
2.30676  2.74830  3.64456  5.71855  4.32503      36 g - - S
          2.68618  4.42225  2.77519  2.73123  3.46354  2.40513  3.72494
3.29354  2.67741  2.69355  4.24690  2.90347  2.73739  3.18146  2.89801
2.37887  2.77519  2.98518  4.58477  3.61503
          0.01140  4.87608  5.59843  0.61958  0.77255  0.48576  0.95510
     36   2.86505  5.38051  1.97266  2.17675  4.72241  2.86903  3.81802
4.21011  2.56578  3.68445  4.42412  2.10673  4.01781  2.58534  2.59351
2.53367  2.78017  3.76986  5.81322  4.39751      37 d - - T
          2.68618  4.42225  2.77519  2.73123  3.46354  2.40513  3.72494
3.29354  2.67741  2.69355  4.24690  2.90347  2.73739  3.18146  2.89801
2.37887  2.77519  2.98518  4.58477  3.61503
          0.01140  4.87608  5.59843  0.61958  0.77255  0.48576  0.95510
     37   2.84639  5.03648  2.37302  2.60838  4.27344  1.78169  3.88364
3.00721  2.69231  2.61979  4.11174  2.52454  4.06471  3.03748  3.15617
2.88259  3.07936  2.97877  5.54475  4.21071      38 g - - T
          2.68623  4.42246  2.77540  2.73102  3.46318  2.40533  3.72515
3.29375  2.67677  2.69362  4.24710  2.90349  2.73714  3.18167  2.89821
2.37907  2.77512  2.98539  4.58498  3.61420
          0.39696  1.97766  1.66470  1.32322  0.30962  0.48576  0.95510
     38   2.34202  4.26956  4.38105  3.79687  1.67815  3.93815  4.18687
2.69079  3.65648  2.02773  3.36107  3.94981  4.30227  3.35705  3.79812
3.23267  3.05759  2.33634  4.76501  2.38768      44 f - - .
          2.68618  4.42225  2.77519  2.73123  3.46354  2.40513  3.72494
3.29354  2.67741  2.69355  4.24690  2.90347  2.73739  3.18146  2.89801
2.37887  2.77519  2.98518  4.58477  3.61503
          0.01401  4.67084  5.39319  0.61958  0.77255  0.45219  1.01124
```

```
     39   2.97144  5.44044  2.78671  2.10799  4.81409  3.69132  3.84220
4.27409  1.75761  3.72710  4.48713  3.15881  4.09053  2.55229  1.52462
2.92914  3.19207  3.84932  5.82698  4.46153      45 r - - T
          2.68618  4.42225  2.77519  2.73123  3.46354  2.40513  3.72494
3.29354  2.67741  2.69355  4.24690  2.90347  2.73739  3.18146  2.89801
2.37887  2.77519  2.98518  4.58477  3.61503
          0.01211  4.81520  5.53755  0.61958  0.77255  0.40860  1.09237
     40   2.88921  5.29212  2.65597  2.03332  4.62661  3.62813  3.87886
4.08641  2.65505  3.61324  4.38280  3.12898  4.05562  2.99617  3.14614
1.65356  1.86166  3.26146  5.78406  4.39281      46 s - - S
          2.68618  4.42225  2.77519  2.73123  3.46354  2.40513  3.72494
3.29354  2.67741  2.69355  4.24690  2.90347  2.73739  3.18146  2.89801
2.37887  2.77519  2.98518  4.58477  3.61503
          0.01140  4.87608  5.59843  0.61958  0.77255  0.48576  0.95510
     41   4.18522  5.37136  6.09842  5.54782  3.56777  5.62812  5.96482
2.28195  5.40914  0.40373  3.29852  5.79302  5.60810  5.27045  5.36532
5.05831  4.39480  2.48843  5.87386  4.93290      47 L - - -
          2.68618  4.42225  2.77519  2.73123  3.46354  2.40513  3.72494
3.29354  2.67741  2.69355  4.24690  2.90347  2.73739  3.18146  2.89801
2.37887  2.77519  2.98518  4.58477  3.61503
          0.01140  4.87608  5.59843  0.61958  0.77255  0.48576  0.95510
     42   2.38678  5.32508  2.52198  2.12452  4.21385  3.01225  3.79064
3.59473  2.05545  3.62795  4.36732  2.97081  3.99615  2.02674  3.01977
2.79221  2.65680  3.71271  5.76107  4.35336      48 q - - -
          2.68618  4.42225  2.77519  2.73123  3.46354  2.40513  3.72494
3.29354  2.67741  2.69355  4.24690  2.90347  2.73739  3.18146  2.89801
2.37887  2.77519  2.98518  4.58477  3.61503
          0.01140  4.87608  5.59843  0.61958  0.77255  0.48576  0.95510
     43   2.23225  5.26517  2.44801  1.61781  4.57868  3.61504  3.80988
4.04244  2.47155  3.12289  4.31555  3.09604  3.13778  2.91809  3.04789
2.81116  2.78578  2.88964  5.71818  4.32662      49 e - - S
          2.68618  4.42225  2.77519  2.73123  3.46354  2.40513  3.72494
3.29354  2.67741  2.69355  4.24690  2.90347  2.73739  3.18146  2.89801
2.37887  2.77519  2.98518  4.58477  3.61503
          0.01140  4.87608  5.59843  0.61958  0.77255  0.48576  0.95510
     44   3.44119  5.87007  2.73120  2.11755  5.40969  0.76638  4.38705
4.94320  3.41111  4.44270  5.30218  2.24988  4.39380  3.56666  3.97444
3.36379  3.77988  4.46385  6.58767  5.10441      50 g - - T
          2.68618  4.42225  2.77519  2.73123  3.46354  2.40513  3.72494
3.29354  2.67741  2.69355  4.24690  2.90347  2.73739  3.18146  2.89801
2.37887  2.77519  2.98518  4.58477  3.61503
          0.01140  4.87608  5.59843  0.61958  0.77255  0.48576  0.95510
     45   3.05219  5.58085  1.77121  1.73441  4.90037  3.65680  3.96440
4.39366  2.77695  2.88536  4.64108  3.11743  4.13293  1.64223  3.29250
2.99042  3.29739  3.96060  6.01379  4.57709      51 q - - S
          2.68618  4.42225  2.77519  2.73123  3.46354  2.40513  3.72494
3.29354  2.67741  2.69355  4.24690  2.90347  2.73739  3.18146  2.89801
2.37887  2.77519  2.98518  4.58477  3.61503
          0.01140  4.87608  5.59843  0.61958  0.77255  0.48576  0.95510
     46   2.44087  5.32693  2.53108  1.94999  4.66757  3.60227  3.78976
4.15019  1.97762  3.63015  3.57850  2.89672  3.99580  2.71137  2.30071
2.67913  3.05849  3.06720  5.76209  4.35415      52 e - - E
          2.68618  4.42225  2.77519  2.73123  3.46354  2.40513  3.72494
3.29354  2.67741  2.69355  4.24690  2.90347  2.73739  3.18146  2.89801
2.37887  2.77519  2.98518  4.58477  3.61503
          0.01140  4.87608  5.59843  0.61958  0.77255  0.48576  0.95510
```

```
      47   3.75247  5.13308  5.46949  5.24431  4.54705  4.74184  5.96377
 2.53130  5.15613  3.10990  4.43077  5.27595  5.31052  5.48056  5.27290
 4.38610  4.12555  0.29769  6.44639  5.22810     53 V - - E
           2.68618  4.42225  2.77519  2.73123  3.46354  2.40513  3.72494
 3.29354  2.67741  2.69355  4.24690  2.90347  2.73739  3.18146  2.89801
 2.37887  2.77519  2.98518  4.58477  3.61503
           0.01140  4.87608  5.59843  0.61958  0.77255  0.48576  0.95510
      48   2.84065  5.22117  3.12581  1.32467  4.51820  3.62827  3.82572
 3.96961  2.58192  3.00658  3.68963  3.01203  4.02411  2.60617  3.06160
 2.38002  2.61213  3.58609  5.68548  4.30751     54 e - - E
           2.68618  4.42225  2.77519  2.73123  3.46354  2.40513  3.72494
 3.29354  2.67741  2.69355  4.24690  2.90347  2.73739  3.18146  2.89801
 2.37887  2.77519  2.98518  4.58477  3.61503
           0.01140  4.87608  5.59843  0.61958  0.77255  0.48576  0.95510
      49   4.94383  5.86025  5.74301  5.70252  0.39571  5.39730  4.04685
 4.46054  5.48036  3.58251  4.98050  4.94501  5.65625  5.12183  5.26940
 4.82569  5.14182  4.44254  4.11858  1.74728     55 F - - E
           2.68618  4.42225  2.77519  2.73123  3.46354  2.40513  3.72494
 3.29354  2.67741  2.69355  4.24690  2.90347  2.73739  3.18146  2.89801
 2.37887  2.77519  2.98518  4.58477  3.61503
           0.01140  4.87608  5.59843  0.61958  0.77255  0.48576  0.95510
      50   2.69333  3.42212  2.08139  1.86084  4.44738  3.63134  3.82830
 3.61342  2.59585  3.44456  4.22609  3.13086  4.02377  2.95132  3.07465
 2.17942  2.80837  3.32296  5.64139  3.26102     56 e - - E
           2.68618  4.42225  2.77519  2.73123  3.46354  2.40513  3.72494
 3.29354  2.67741  2.69355  4.24690  2.90347  2.73739  3.18146  2.89801
 2.37887  2.77519  2.98518  4.58477  3.61503
           0.01140  4.87608  5.59843  0.61958  0.77255  0.48576  0.95510
      51   2.80341  4.36699  4.58813  3.63346  3.49527  4.10384  4.43276
 1.57183  3.84234  2.08938  3.48783  4.14593  4.46386  4.05260  2.94499
 3.40527  3.02839  1.42126  5.04319  3.85562     57 v - - E
           2.68618  4.42225  2.77519  2.73123  3.46354  2.40513  3.72494
 3.29354  2.67741  2.69355  4.24690  2.90347  2.73739  3.18146  2.89801
 2.37887  2.77519  2.98518  4.58477  3.61503
           0.01140  4.87608  5.59843  0.61958  0.77255  0.48576  0.95510
      52   2.83127  4.97649  3.26712  2.22008  4.19964  3.33196  3.56802
 2.71302  2.51284  3.03970  4.05176  3.22130  4.06431  2.53976  3.15510
 2.37271  2.47501  2.11783  5.48990  4.16761     58 v - - E
           2.68618  4.42225  2.77519  2.73123  3.46354  2.40513  3.72494
 3.29354  2.67741  2.69355  4.24690  2.90347  2.73739  3.18146  2.89801
 2.37887  2.77519  2.98518  4.58477  3.61503
           0.01140  4.87608  5.59843  0.61958  0.77255  0.48576  0.95510
      53   2.82747  5.33100  2.38470  1.94954  4.67304  3.60141  3.34621
 3.31073  2.35072  3.01207  4.37253  2.47858  3.99524  2.31566  2.79897
 2.44056  3.05866  3.72003  5.76535  4.35622     59 e - - E
           2.68632  4.42239  2.77488  2.73137  3.46368  2.40502  3.72508
 3.29368  2.67755  2.69369  4.24704  2.90321  2.73753  3.18108  2.89755
 2.37886  2.77533  2.98532  4.58491  3.61517
           0.28289  1.41597  5.59843  0.51102  0.91600  0.48576  0.95510
      54   2.93767  5.38849  2.89367  2.59286  4.73650  1.46644  3.88176
 4.20582  2.39381  3.69950  4.46163  2.63739  4.07883  2.99277  2.18570
 2.53107  3.17452  3.78795  5.84100  4.44757     62 g - - C
           2.68618  4.42225  2.77519  2.73123  3.46354  2.40513  3.72494
 3.29354  2.67741  2.69355  4.24690  2.90347  2.73739  3.18146  2.89801
 2.37887  2.77519  2.98518  4.58477  3.61503
           0.01140  4.87608  5.59843  0.61958  0.77255  0.48576  0.95510
```

```
     55   2.54186  5.32655  2.54479  2.39359  4.66641  2.86912  3.79071
4.14887  2.25478  2.93771  4.36870  2.59414  3.07376  2.38444  3.02005
2.63037  2.33049  3.71441  5.76231  4.35425      63 k - - S
          2.68618  4.42225  2.77519  2.73123  3.46354  2.40513  3.72494
3.29354  2.67741  2.69355  4.24690  2.90347  2.73739  3.18146  2.89801
2.37887  2.77519  2.98518  4.58477  3.61503
          0.01140  4.87608  5.59843  0.61958  0.77255  0.48576  0.95510
     56   3.15416  5.53279  3.40357  2.83438  4.95479  2.20775  3.93389
4.37484  1.30728  3.81280  4.60180  2.34774  4.23794  3.04423  1.95735
3.11924  3.36034  3.97549  5.88604  4.58711      64 k - - S
          2.68618  4.42225  2.77519  2.73123  3.46354  2.40513  3.72494
3.29354  2.67741  2.69355  4.24690  2.90347  2.73739  3.18146  2.89801
2.37887  2.77519  2.98518  4.58477  3.61503
          0.01140  4.87608  5.59843  0.61958  0.77255  0.48576  0.95510
     57   3.39454  5.55287  3.62497  3.16707  5.15614  0.96548  4.17422
4.54557  1.52939  3.99929  4.84621  3.60139  4.45022  3.31457  2.55707
3.40107  3.63766  4.15999  6.04871  4.84015      65 g - - C
          2.68618  4.42225  2.77519  2.73123  3.46354  2.40513  3.72494
3.29354  2.67741  2.69355  4.24690  2.90347  2.73739  3.18146  2.89801
2.37887  2.77519  2.98518  4.58477  3.61503
          0.01140  4.87608  5.59843  0.61958  0.77255  0.48576  0.95510
     58   2.83076  3.48460  2.93216  2.51990  4.20200  3.67438  3.88234
3.61032  2.69807  2.67582  3.72896  2.82691  1.76023  3.04495  2.85688
2.74406  2.89017  2.94278  5.49148  4.16864      66 p - - E
          2.68618  4.42225  2.77519  2.73123  3.46354  2.40513  3.72494
3.29354  2.67741  2.69355  4.24690  2.90347  2.73739  3.18146  2.89801
2.37887  2.77519  2.98518  4.58477  3.61503
          0.01140  4.87608  5.59843  0.61958  0.77255  0.48576  0.95510
     59   2.54648  5.28472  3.09782  1.94011  4.60821  3.60981  3.27965
3.28580  2.33367  3.58053  4.33125  3.08618  4.00277  2.03565  2.89244
2.17291  2.83652  3.24716  5.73033  4.33334      67 e - - E
          2.68618  4.42225  2.77519  2.73123  3.46354  2.40513  3.72494
3.29354  2.67741  2.69355  4.24690  2.90347  2.73739  3.18146  2.89801
2.37887  2.77519  2.98518  4.58477  3.61503
          0.01140  4.87608  5.59843  0.61958  0.77255  0.48576  0.95510
     60   0.21672  5.03756  4.82733  4.77644  5.39749  3.82121  5.61495
4.73148  4.82865  4.53880  5.50664  4.57183  4.64725  5.06554  4.92224
3.41031  3.75496  4.16090  6.67239  5.66052      68 A - - E
          2.68618  4.42225  2.77519  2.73123  3.46354  2.40513  3.72494
3.29354  2.67741  2.69355  4.24690  2.90347  2.73739  3.18146  2.89801
2.37887  2.77519  2.98518  4.58477  3.61503
          0.01140  4.87608  5.59843  0.61958  0.77255  0.48576  0.95510
     61   1.81137  3.41249  3.24213  2.39359  3.95151  3.73363  3.95925
2.68796  2.64333  3.00528  3.86544  3.35055  4.11881  3.04961  3.27419
2.65293  2.44437  2.65921  5.32479  3.41900      69 a - - E
          2.68618  4.42225  2.77519  2.73123  3.46354  2.40513  3.72494
3.29354  2.67741  2.69355  4.24690  2.90347  2.73739  3.18146  2.89801
2.37887  2.77519  2.98518  4.58477  3.61503
          0.01140  4.87608  5.59843  0.61958  0.77255  0.48576  0.95510
     62   2.63052  5.36952  2.62672  2.26656  4.70747  3.28177  3.55700
4.19193  2.09731  3.67175  4.41437  1.53299  4.01931  2.92082  3.06413
2.64914  3.09823  3.47042  5.80483  4.39213      70 n - - E
          2.68618  4.42225  2.77519  2.73123  3.46354  2.40513  3.72494
3.29354  2.67741  2.69355  4.24690  2.90347  2.73739  3.18146  2.89801
2.37887  2.77519  2.98518  4.58477  3.61503
          0.01140  4.87608  5.59843  0.61958  0.77255  0.48576  0.95510
```

```
     63   3.74770  4.94033  5.82611  5.36077  4.20940  5.37134  6.09781
1.80195  5.29728  2.21118  3.94123  5.52862  5.57024  5.52657  5.45232
4.81128  4.00629  0.52581  6.36282  5.16487     71 V - - E
          2.68618  4.42225  2.77519  2.73123  3.46354  2.40513  3.72494
3.29354  2.67741  2.69355  4.24690  2.90347  2.73739  3.18146  2.89801
2.37887  2.77519  2.98518  4.58477  3.61503
          0.01140  4.87608  5.59843  0.61958  0.77255  0.48576  0.95510
     64   2.83550  4.71912  3.49731  2.93450  3.88393  3.75387  3.98528
2.75328  2.79040  2.94364  3.81285  2.99451  4.13764  2.66598  2.66547
2.50115  2.01635  2.41323  3.51033  3.06126     72 t - - E
          2.68618  4.42225  2.77519  2.73123  3.46354  2.40513  3.72494
3.29354  2.67741  2.69355  4.24690  2.90347  2.73739  3.18146  2.89801
2.37887  2.77519  2.98518  4.58477  3.61503
          0.03072  4.87608  3.78855  0.61958  0.77255  0.48576  0.95510
     65   2.55705  4.97512  3.25802  2.69931  4.20131  2.26462  3.87452
3.42044  2.07376  2.44386  4.05115  3.21208  3.13314  3.03615  2.70054
2.69786  2.86056  2.74414  5.48828  4.16500     73 k - - E
          2.68618  4.42225  2.77519  2.73123  3.46354  2.40513  3.72494
3.29354  2.67741  2.69355  4.24690  2.90347  2.73739  3.18146  2.89801
2.37887  2.77519  2.98518  4.58477  3.61503
          0.22221  4.85697  1.65297  0.61958  0.77255  0.53183  0.88559
     66   2.57100  4.30593  3.97850  3.40201  3.42364  3.80955  4.10115
2.15828  3.31582  2.06922  3.42046  3.69793  2.41534  3.31859  3.57185
2.73417  2.56263  2.09024  4.91534  3.71080     74 l - - E
          2.68618  4.42225  2.77519  2.73123  3.46354  2.40513  3.72494
3.29354  2.67741  2.69355  4.24690  2.90347  2.73739  3.18146  2.89801
2.37887  2.77519  2.98518  4.58477  3.61503
          0.00966  4.64442        *  0.61958  0.77255  0.00000        *
//
HMMER3/f [3.3 | Nov 2019]
NAME  DDT
ACC   PF02791.18
DESC  DDT domain
LENG  60
ALPH  amino
RF    no
MM    no
CONS  yes
CS    no
MAP   yes
DATE  Thu Apr 16 14:58:24 2020
NSEQ  159
EFFN  8.952484
CKSUM 1687141266
GA    21.80 21.80
TC    21.80 21.80
NC    21.70 21.70
STATS LOCAL MSV       -8.5312  0.71893
STATS LOCAL VITERBI   -9.0722  0.71893
STATS LOCAL FORWARD   -3.8580  0.71893
HMM          A        C        D        E        F        G        H
I        K        L        M        N        P        Q        R        S
T        V        W        Y
            m->m     m->i     m->d     i->m     i->i     d->m     d->d
  COMPO   2.70343  3.82612  2.85777  2.65579  2.67778  3.09384  3.58756
2.96895  3.09160  1.89317  3.49465  3.35140  3.56862  3.19140  3.26906
2.69151  2.98120  2.64880  4.56012  3.81454
```

```
         2.68618  4.42225  2.77519  2.73123  3.46354  2.40513  3.72494
3.29354  2.67741  2.69355  4.24690  2.90347  2.73739  3.18146  2.89801
2.37887  2.77519  2.98518  4.58477  3.61503
         0.21262  5.86384  1.66762  0.61958  0.77255  0.00000        *
      1  2.94675  4.73213  2.56375  1.27064  5.06397  3.97610  2.19571
4.55079  2.29316  4.02356  4.75843  2.91610  4.37042  2.61009  3.20766
2.71208  3.43567  4.10911  6.14983  4.73802      1 e - - -
         2.68618  4.42225  2.77519  2.73123  3.46354  2.40513  3.72494
3.29354  2.67741  2.69355  4.24690  2.90347  2.73739  3.18146  2.89801
2.37887  2.77519  2.98518  4.58477  3.61503
         0.00521  5.65643  6.37878  0.61958  0.77255  0.47942  0.96533
      2  2.12582  2.20475  1.96731  2.96168  4.91902  3.90635  2.93148
3.96908  3.01662  2.78920  4.69354  2.96478  4.48224  3.17528  3.53026
2.24021  2.60837  3.79477  6.10773  3.87096      2 d - - -
         2.68618  4.42225  2.77519  2.73123  3.46354  2.40513  3.72494
3.29354  2.67741  2.69355  4.24690  2.90347  2.73739  3.18146  2.89801
2.37887  2.77519  2.98518  4.58477  3.61503
         0.00455  5.79046  6.51281  0.61958  0.77255  0.50522  0.92476
      3  3.83014  5.15813  5.82914  5.23331  0.99866  5.07517  5.43385
2.24815  5.03902  2.50474  3.41483  5.24188  5.39203  5.14802  5.04702
4.40396  3.62744  1.26797  5.85523  4.30805      3 f - - -
         2.68618  4.42225  2.77519  2.73123  3.46354  2.40513  3.72494
3.29354  2.67741  2.69355  4.24690  2.90347  2.73739  3.18146  2.89801
2.37887  2.77519  2.98518  4.58477  3.61503
         0.00436  5.83355  6.55590  0.61958  0.77255  0.40559  1.09836
      4  2.40252  5.76499  2.75307  2.72951  4.43454  1.40332  4.30444
4.33130  2.91105  3.20689  3.41861  3.59393  2.48951  2.94275  3.53920
2.16759  3.55949  3.89735  6.21594  4.82369      4 g - - -
         2.68618  4.42225  2.77519  2.73123  3.46354  2.40513  3.72494
3.29354  2.67741  2.69355  4.24690  2.90347  2.73739  3.18146  2.89801
2.37887  2.77519  2.98518  4.58477  3.61503
         0.00424  5.86157  6.58392  0.61958  0.77255  0.47645  0.97017
      5  3.09368  5.83371  1.37603  2.48879  4.74400  4.10011  3.41523
4.66272  2.27744  3.60248  4.46586  2.68344  3.43199  3.03772  3.30134
2.77003  3.44057  2.17189  6.26711  4.48540      5 d - - -
         2.68618  4.42225  2.77519  2.73123  3.46354  2.40513  3.72494
3.29354  2.67741  2.69355  4.24690  2.90347  2.73739  3.18146  2.89801
2.37887  2.77519  2.98518  4.58477  3.61503
         0.00423  5.86384  6.58619  0.61958  0.77255  0.48576  0.95510
      6  2.21834  1.68614  5.41057  4.79458  2.41611  4.60603  4.94051
2.80275  4.57697  1.54205  2.41682  4.77239  4.96910  4.70565  4.58090
3.78475  3.30355  2.33633  5.43006  4.04455      6 l - - -
         2.68618  4.42225  2.77519  2.73123  3.46354  2.40513  3.72494
3.29354  2.67741  2.69355  4.24690  2.90347  2.73739  3.18146  2.89801
2.37887  2.77519  2.98518  4.58477  3.61503
         0.00423  5.86384  6.58619  0.61958  0.77255  0.48576  0.95510
      7  3.82023  4.76551  6.16171  5.55936  2.86441  5.42802  5.76099
2.85121  5.37095  0.40240  3.41612  5.59988  5.67489  5.39827  5.34793
4.76566  4.06993  2.47858  6.06442  4.97997      7 L - - -
         2.68618  4.42225  2.77519  2.73123  3.46354  2.40513  3.72494
3.29354  2.67741  2.69355  4.24690  2.90347  2.73739  3.18146  2.89801
2.37887  2.77519  2.98518  4.58477  3.61503
         0.00423  5.86384  6.58619  0.61958  0.77255  0.48576  0.95510
      8  2.74251  5.13784  3.72430  2.33584  3.82184  3.61218  4.36877
4.24227  3.00322  3.65813  1.53643  3.57661  4.55711  1.72117  3.62865
2.34248  2.86903  3.67353  6.07001  4.01889      8 m - - -
```

2.68618 4.42225 2.77519 2.73123 3.46354 2.40513 3.72494
3.29354 2.67741 2.69355 4.24690 2.90347 2.73739 3.18146 2.89801
2.37887 2.77519 2.98518 4.58477 3.61503
0.00423 5.86384 6.58619 0.61958 0.77255 0.48576 0.95510
9 2.91579 4.07355 5.63712 5.04200 3.90285 4.87832 5.25368
2.03331 4.84615 2.87202 4.14322 5.04289 5.23075 4.99203 4.87114
3.50101 2.54283 0.64286 5.73982 4.55770 9 V - - -
2.68618 4.42225 2.77519 2.73123 3.46354 2.40513 3.72494
3.29354 2.67741 2.69355 4.24690 2.90347 2.73739 3.18146 2.89801
2.37887 2.77519 2.98518 4.58477 3.61503
0.00423 5.86384 6.58619 0.61958 0.77255 0.48576 0.95510
10 3.49054 4.86193 5.44030 4.31668 2.84242 4.65457 4.93866
3.15568 4.61775 2.07037 3.29273 4.36486 5.01591 4.74202 4.62398
3.96689 3.72235 2.55925 1.34863 1.44645 10 w - - -
2.68618 4.42225 2.77519 2.73123 3.46354 2.40513 3.72494
3.29354 2.67741 2.69355 4.24690 2.90347 2.73739 3.18146 2.89801
2.37887 2.77519 2.98518 4.58477 3.61503
0.00423 5.86384 6.58619 0.61958 0.77255 0.48576 0.95510
11 2.97961 5.83762 2.03397 1.13387 5.18322 3.45124 3.91576
4.12360 2.88579 4.14313 4.56487 3.10258 4.49280 2.66523 3.31312
2.41962 3.14775 3.68086 6.26999 4.07202 11 e - - -
2.68618 4.42225 2.77519 2.73123 3.46354 2.40513 3.72494
3.29354 2.67741 2.69355 4.24690 2.90347 2.73739 3.18146 2.89801
2.37887 2.77519 2.98518 4.58477 3.61503
0.00423 5.86384 6.58619 0.61958 0.77255 0.48576 0.95510
12 3.50731 3.94273 5.45553 4.84733 0.55696 4.68191 5.01237
2.98117 4.63905 2.94610 3.87232 4.83941 5.04302 4.77191 4.65467
3.84945 2.74271 2.57067 5.49769 4.05876 12 F - - -
2.68618 4.42225 2.77519 2.73123 3.46354 2.40513 3.72494
3.29354 2.67741 2.69355 4.24690 2.90347 2.73739 3.18146 2.89801
2.37887 2.77519 2.98518 4.58477 3.61503
0.00423 5.86384 6.58619 0.61958 0.77255 0.48576 0.95510
13 3.69759 3.63386 5.88716 5.27851 1.74708 5.11917 5.45292
2.39809 5.07625 0.68753 3.44455 5.28892 5.41949 5.14940 5.06521
4.44464 3.81656 2.85929 5.83987 4.71531 13 L - - -
2.68618 4.42225 2.77519 2.73123 3.46354 2.40513 3.72494
3.29354 2.67741 2.69355 4.24690 2.90347 2.73739 3.18146 2.89801
2.37887 2.77519 2.98518 4.58477 3.61503
0.00423 5.86384 6.58619 0.61958 0.77255 0.48576 0.95510
14 3.33429 2.64500 3.64293 2.98130 4.99697 4.01314 1.56624
3.93810 2.93773 3.63066 4.53336 1.78954 4.52329 3.25222 2.24798
2.75826 2.97494 3.96190 6.17150 4.17899 14 h - - -
2.68618 4.42225 2.77519 2.73123 3.46354 2.40513 3.72494
3.29354 2.67741 2.69355 4.24690 2.90347 2.73739 3.18146 2.89801
2.37887 2.77519 2.98518 4.58477 3.61503
0.00423 5.86384 6.58619 0.61958 0.77255 0.48576 0.95510
15 2.52542 4.69146 4.21989 3.65339 3.94643 3.77012 3.42897
2.98193 3.48771 3.20059 4.04401 2.66828 4.73157 3.60187 3.59025
1.97905 1.90189 1.51161 5.70769 4.46846 15 v - - -
2.68618 4.42225 2.77519 2.73123 3.46354 2.40513 3.72494
3.29354 2.67741 2.69355 4.24690 2.90347 2.73739 3.18146 2.89801
2.37887 2.77519 2.98518 4.58477 3.61503
0.00423 5.86384 6.58619 0.61958 0.77255 0.48576 0.95510
16 5.33674 6.42647 6.46986 6.26306 0.37258 6.03999 3.73651
5.02729 4.89651 3.83992 4.33025 5.68991 6.31842 4.72113 5.87825
5.41750 5.54206 4.92009 5.00732 1.67652 16 F - - -

```
         2.68618  4.42225  2.77519  2.73123  3.46354  2.40513  3.72494
3.29354  2.67741  2.69355  4.24690  2.90347  2.73739  3.18146  2.89801
2.37887  2.77519  2.98518  4.58477  3.61503
         0.00423  5.86384  6.58619  0.61958  0.77255  0.48576  0.95510
     17  2.58222  2.32183  3.35227  2.59912  5.15602  1.34567  3.15573
4.63647  2.73506  3.92761  4.86152  3.19895  4.49691  3.26350  2.75066
2.38869  3.45641  4.20521  5.10241  4.85003      17 g - - -
         2.68618  4.42225  2.77519  2.73123  3.46354  2.40513  3.72494
3.29354  2.67741  2.69355  4.24690  2.90347  2.73739  3.18146  2.89801
2.37887  2.77519  2.98518  4.58477  3.61503
         0.00423  5.86384  6.58619  0.61958  0.77255  0.48576  0.95510
     18  3.23313  5.83873  2.56110  1.83623  4.65036  2.10905  3.71126
3.26441  2.26015  4.14446  4.87911  2.75676  4.13997  3.02655  2.12859
2.72019  2.84308  4.11323  5.39937  4.85920      18 e - - -
         2.68618  4.42225  2.77519  2.73123  3.46354  2.40513  3.72494
3.29354  2.67741  2.69355  4.24690  2.90347  2.73739  3.18146  2.89801
2.37887  2.77519  2.98518  4.58477  3.61503
         0.00423  5.86384  6.58619  0.61958  0.77255  0.48576  0.95510
     19  2.99337  4.91903  4.76242  3.54044  4.02631  4.49335  4.78788
2.49483  3.51049  1.45983  3.74442  4.43024  2.64673  3.22378  3.78382
3.38960  2.05890  1.76988  5.52674  3.99678      19 l - - -
         2.68618  4.42225  2.77519  2.73123  3.46354  2.40513  3.72494
3.29354  2.67741  2.69355  4.24690  2.90347  2.73739  3.18146  2.89801
2.37887  2.77519  2.98518  4.58477  3.61503
         0.00423  5.86384  6.58619  0.61958  0.77255  0.48576  0.95510
     20  4.49710  6.03240  6.78691  6.19355  2.39086  6.15616  6.35925
2.75538  6.03677  0.32313  3.99364  6.32529  6.19594  5.85967  5.93654
5.53604  5.01375  3.21024  6.36824  4.03831      20 L - - -
         2.68618  4.42225  2.77519  2.73123  3.46354  2.40513  3.72494
3.29354  2.67741  2.69355  4.24690  2.90347  2.73739  3.18146  2.89801
2.37887  2.77519  2.98518  4.58477  3.61503
         0.00423  5.86384  6.58619  0.61958  0.77255  0.48576  0.95510
     21  3.11201  4.70028  2.76425  2.82394  3.46521  2.20737  1.91908
3.95390  2.37536  3.48170  4.09266  3.03711  4.27105  3.06864  2.55986
2.20555  3.44356  3.59099  6.25266  3.96251      21 h - - -
         2.68618  4.42225  2.77519  2.73123  3.46354  2.40513  3.72494
3.29354  2.67741  2.69355  4.24690  2.90347  2.73739  3.18146  2.89801
2.37887  2.77519  2.98518  4.58477  3.61503
         0.00423  5.86384  6.58619  0.61958  0.77255  0.48576  0.95510
     22  3.37788  5.15767  5.79988  5.19398  2.87469  5.03357  5.37624
2.17550  4.99233  0.52498  3.76934  5.20065  5.35057  5.08720  4.99162
3.96267  3.81767  2.62842  5.79372  4.65228      22 L - - -
         2.68511  4.42784  2.76961  2.72606  3.46630  2.40103  3.73064
3.29823  2.67741  2.69853  4.23880  2.90867  2.73288  3.18362  2.90075
2.38233  2.78080  2.97524  4.59003  3.61482
         0.51997  0.90614  6.58619  2.69923  0.06963  0.48576  0.95510
     23  3.22347  4.78203  1.58741  2.13457  4.03900  3.36580  4.01552
4.64177  2.82388  4.12427  4.32392  3.11752  4.11384  3.27259  3.51940
1.55289  2.61901  4.20890  3.98538  4.24486      54 s - - -
         2.68618  4.42225  2.77519  2.73123  3.46354  2.40513  3.72494
3.29354  2.67741  2.69355  4.24690  2.90347  2.73739  3.18146  2.89801
2.37887  2.77519  2.98518  4.58477  3.61503
         0.13745  5.86384  2.07482  0.61958  0.77255  0.48576  0.95510
     24  2.79400  4.95297  3.46711  3.03626  4.83733  1.69706  3.73493
3.69220  3.03346  3.65413  4.62710  3.32500  1.59403  3.10565  3.40908
2.31431  2.60951  3.74702  6.04583  4.30671      55 p - - -
```

```
          2.68618  4.42225  2.77519  2.73123  3.46354  2.40513  3.72494
3.29354  2.67741  2.69355  4.24690  2.90347  2.73739  3.18146  2.89801
2.37887  2.77519  2.98518  4.58477  3.61503
          0.00483  5.73123  6.45357  0.61958  0.77255  1.09052  0.40954
     25   3.40520  3.96967  5.38109  4.76699  1.13323  4.58315  4.91770
1.37090  4.55193  2.49535  3.06279  4.74892  4.11482  4.67662  4.55585
3.89674  3.63714  2.53710  5.39740  3.62813     56 f - - -
          2.68618  4.42225  2.77519  2.73123  3.46354  2.40513  3.72494
3.29354  2.67741  2.69355  4.24690  2.90347  2.73739  3.18146  2.89801
2.37887  2.77519  2.98518  4.58477  3.61503
          0.00483  5.73123  6.45357  0.61958  0.77255  1.09052  0.40954
     26   3.06195  5.75048  3.26386  2.06277  5.09071  3.75927  4.21376
4.57331  2.87489  3.90285  4.79298  3.24107  4.01427  3.25613  2.74138
2.29636  1.03308  4.01646  6.18627  4.77803     57 t - - -
          2.68619  4.42226  2.77521  2.73124  3.46317  2.40514  3.72496
3.29355  2.67742  2.69356  4.24691  2.90348  2.73741  3.18147  2.89802
2.37888  2.77521  2.98519  4.58478  3.61504
          0.01896  4.06275  6.45357  0.39503  1.11981  0.23593  1.55986
     27   3.46717  5.08609  5.70825  5.09425  1.92498  4.92072  5.23905
2.65031  4.88273  0.87962  3.25383  5.08762  5.24583  4.97157  4.87353
4.23908  3.96052  2.76594  2.11699  3.90671     59 l - - -
          2.68618  4.42225  2.77519  2.73123  3.46354  2.40513  3.72494
3.29354  2.67741  2.69355  4.24690  2.90347  2.73739  3.18146  2.89801
2.37887  2.77519  2.98518  4.58477  3.61503
          0.00423  5.86384  6.58619  0.61958  0.77255  0.48576  0.95510
     28   3.02996  4.83420  1.23105  1.31013  5.21887  3.34604  4.09292
4.70626  2.90455  4.17985  4.91710  3.30535  4.25606  3.13875  3.55411
2.78250  3.59270  4.26523  6.30746  4.59407     60 d - - -
          2.68618  4.42225  2.77519  2.73123  3.46354  2.40513  3.72494
3.29354  2.67741  2.69355  4.24690  2.90347  2.73739  3.18146  2.89801
2.37887  2.77519  2.98518  4.58477  3.61503
          0.00423  5.86384  6.58619  0.61958  0.77255  0.48576  0.95510
     29   3.06768  5.77373  1.25027  2.28957  5.09192  3.92718  4.30393
3.64852  2.95786  3.22986  2.10953  3.34191  4.50664  2.91852  3.40627
2.91633  2.73972  3.80933  6.22284  4.82869     61 d - - -
          2.68618  4.42225  2.77519  2.73123  3.46354  2.40513  3.72494
3.29354  2.67741  2.69355  4.24690  2.90347  2.73739  3.18146  2.89801
2.37887  2.77519  2.98518  4.58477  3.61503
          0.00423  5.86384  6.58619  0.61958  0.77255  0.48576  0.95510
     30   4.99679  6.17951  6.92587  6.35255  0.92093  6.33681  6.35424
3.33132  6.20198  0.82668  3.23555  6.47138  6.31320  5.94996  6.06758
5.73243  5.18418  3.70411  6.30304  3.55435     62 l - - -
          2.68618  4.42225  2.77519  2.73123  3.46354  2.40513  3.72494
3.29354  2.67741  2.69355  4.24690  2.90347  2.73739  3.18146  2.89801
2.37887  2.77519  2.98518  4.58477  3.61503
          0.00423  5.86384  6.58619  0.61958  0.77255  0.48576  0.95510
     31   2.15338  3.15871  4.21066  1.42846  4.27630  4.09767  4.13335
2.49466  3.60373  2.74347  3.62343  4.06376  4.72883  2.28859  3.71911
3.38545  3.28041  2.28571  5.71212  4.47179     63 e - - -
          2.68618  4.42225  2.77519  2.73123  3.46354  2.40513  3.72494
3.29354  2.67741  2.69355  4.24690  2.90347  2.73739  3.18146  2.89801
2.37887  2.77519  2.98518  4.58477  3.61503
          0.00423  5.86384  6.58619  0.61958  0.77255  0.48576  0.95510
     32   2.46995  4.82306  1.82847  2.04815  5.18460  3.18246  4.28600
4.23609  2.45806  3.88474  4.57653  2.92999  4.49254  2.43739  2.75651
2.11608  2.99642  4.06949  6.27066  3.86109     64 d - - -
```

2.68618 4.42225 2.77519 2.73123 3.46354 2.40513 3.72494
3.29354 2.67741 2.69355 4.24690 2.90347 2.73739 3.18146 2.89801
2.37887 2.77519 2.98518 4.58477 3.61503
0.00423 5.86384 6.58619 0.61958 0.77255 0.48576 0.95510
33 0.62789 3.54756 5.97358 5.72863 5.64198 2.52655 6.16341
5.02298 5.57259 4.77484 5.62120 5.18315 5.18688 5.68212 5.59569
2.38064 3.75630 1.68135 7.08665 5.98273 65 A - - -
2.68618 4.42225 2.77519 2.73123 3.46354 2.40513 3.72494
3.29354 2.67741 2.69355 4.24690 2.90347 2.73739 3.18146 2.89801
2.37887 2.77519 2.98518 4.58477 3.61503
0.00423 5.86384 6.58619 0.61958 0.77255 0.48576 0.95510
34 5.37248 6.45789 7.36850 6.77294 3.30128 6.92664 7.09450
2.57358 6.68190 0.32330 2.27088 7.14260 6.61265 6.22427 6.46302
6.41204 5.53355 3.39185 6.69932 5.90550 66 L - - -
2.68618 4.42225 2.77519 2.73123 3.46354 2.40513 3.72494
3.29354 2.67741 2.69355 4.24690 2.90347 2.73739 3.18146 2.89801
2.37887 2.77519 2.98518 4.58477 3.61503
0.00423 5.86384 6.58619 0.61958 0.77255 0.48576 0.95510
35 3.06775 3.47836 4.40246 3.83147 2.03171 3.66384 3.29533
3.19868 2.89362 1.96875 3.37331 3.43700 4.78243 2.70020 3.07532
2.16389 2.94119 2.49834 5.63158 4.05108 67 l - - -
2.68618 4.42225 2.77519 2.73123 3.46354 2.40513 3.72494
3.29354 2.67741 2.69355 4.24690 2.90347 2.73739 3.18146 2.89801
2.37887 2.77519 2.98518 4.58477 3.61503
0.22268 5.86384 1.62562 0.61958 0.77255 0.48576 0.95510
36 3.06421 2.57134 2.95246 2.53799 3.52197 2.86371 3.68973
3.85935 2.94656 3.19687 4.08513 2.58948 3.88455 3.20360 3.49126
1.60892 3.16097 3.48822 3.64746 3.29806 68 s - - -
2.68618 4.42225 2.77519 2.73123 3.46354 2.40513 3.72494
3.29354 2.67741 2.69355 4.24690 2.90347 2.73739 3.18146 2.89801
2.37887 2.77519 2.98518 4.58477 3.61503
0.00526 5.64642 6.36877 0.61958 0.77255 1.32177 0.31015
37 2.90652 5.70619 2.85034 2.24542 4.76589 3.40116 3.58250
3.80054 2.33466 3.44236 4.38463 2.47188 4.09325 1.76039 3.00888
2.40041 2.55785 3.44368 6.13875 3.89743 69 q - - -
2.68618 4.42225 2.77519 2.73123 3.46354 2.40513 3.72494
3.29354 2.67741 2.69355 4.24690 2.90347 2.73739 3.18146 2.89801
2.37887 2.77519 2.98518 4.58477 3.61503
0.00526 5.64642 6.36877 0.61958 0.77255 0.31872 1.29857
38 3.05929 5.03184 1.58499 1.93072 2.60064 2.73836 2.95858
4.42637 2.86698 3.72824 4.82635 3.12342 4.12894 3.27488 3.50220
2.44168 3.25079 3.69954 5.01482 3.92745 70 d - - -
2.68618 4.42225 2.77519 2.73123 3.46354 2.40513 3.72494
3.29354 2.67741 2.69355 4.24690 2.90347 2.73739 3.18146 2.89801
2.37887 2.77519 2.98518 4.58477 3.61503
0.14030 5.82655 2.05606 0.61958 0.77255 0.70482 0.68161
39 2.87541 5.72795 3.01868 2.13958 5.07135 3.99566 3.43493
3.61471 2.59462 3.84030 4.40610 3.00552 1.76844 1.95526 3.13829
2.81863 2.90959 3.03784 6.16164 4.39265 71 p - - -
2.68618 4.42225 2.77519 2.73123 3.46354 2.40513 3.72494
3.29354 2.67741 2.69355 4.24690 2.90347 2.73739 3.18146 2.89801
2.37887 2.77519 2.98518 4.58477 3.61503
0.33542 5.69127 1.26731 0.61958 0.77255 1.20860 0.35470
40 2.64588 5.53583 2.42345 2.25126 4.87309 3.50807 3.88027
4.35365 2.74605 3.83779 1.97232 2.79928 2.29154 2.63295 3.16394
2.73910 2.88686 3.65499 5.97320 4.56688 72 m - - -

```
          2.68618  4.42225  2.77519  2.73123  3.46354  2.40513  3.72494
 3.29354  2.67741  2.69355  4.24690  2.90347  2.73739  3.18146  2.89801
 2.37887  2.77519  2.98518  4.58477  3.61503
          0.03095  5.36284  3.65772  0.61958  0.77255  1.77667  0.18537
      41  3.04169  5.22441  3.45340  1.90977  3.33634  1.39490  4.08238
 3.75362  2.80316  3.39055  4.15737  3.23109  3.68585  3.17296  3.25618
 2.89130  3.04309  2.82641  5.73068  3.78261     73 g - - -
          2.68618  4.42225  2.77519  2.73123  3.46354  2.40513  3.72494
 3.29354  2.67741  2.69355  4.24690  2.90347  2.73739  3.18146  2.89801
 2.37887  2.77519  2.98518  4.58477  3.61503
          0.02000  5.33904  4.19976  0.61958  0.77255  0.62076  0.77119
      42  2.98526  5.68214  2.45711  2.08178  5.02081  3.79524  4.15063
 4.50229  2.65665  3.03336  4.72448  3.23566  1.63124  2.93147  3.25080
 2.13370  2.98340  3.32338  4.90063  4.34461     74 p - - -
          2.68683  4.42388  2.77645  2.72781  3.46517  2.40530  3.72561
 3.29480  2.67858  2.69483  4.24683  2.90233  2.73811  3.17947  2.89727
 2.37763  2.77410  2.98608  4.58640  3.61666
          1.51105  0.81639  1.08679  1.54395  0.24021  0.20076  1.70433
      43  2.87387  2.54458  2.17979  2.18548  4.93780  3.91962  4.10849
 4.08091  2.60379  3.40601  4.65455  2.73866  3.05911  3.05906  3.33987
 1.92427  2.97282  3.09510  6.05230  3.73917     83 s - - -
          2.68618  4.42225  2.77519  2.73123  3.46354  2.40513  3.72494
 3.29354  2.67741  2.69355  4.24690  2.90347  2.73739  3.18146  2.89801
 2.37887  2.77519  2.98518  4.58477  3.61503
          0.20868  5.54464  1.69045  0.61958  0.77255  1.47561  0.25960
      44  2.69858  4.85424  3.08133  1.46531  4.84377  3.81633  4.00492
 3.77280  2.66725  3.26127  4.32215  3.05157  3.17069  2.55223  2.81751
 2.47105  2.50573  3.18841  5.95442  3.96429     84 e - - -
          2.68618  4.42225  2.77519  2.73123  3.46354  2.40513  3.72494
 3.29354  2.67741  2.69355  4.24690  2.90347  2.73739  3.18146  2.89801
 2.37887  2.77519  2.98518  4.58477  3.61503
          0.00707  5.35095  6.07329  0.61958  0.77255  1.78976  0.18272
      45  4.74132  5.87392  6.73391  6.14799  3.86049  6.24029  6.48190
 2.44395  6.03933  0.50759  1.67510  6.43626  6.06808  5.70958  5.88683
 5.68562  4.92220  2.90219  6.22822  5.38171     85 L - - -
          2.68734  4.42341  2.77635  2.73239  3.46470  2.40521  3.72610
 3.29470  2.67857  2.69471  4.24805  2.90462  2.73706  3.18030  2.89916
 2.37378  2.77635  2.98252  4.58593  3.61260
          0.18863  1.77427  6.07329  2.36058  0.09912  0.11031  2.25910
      46  3.50344  4.87269  5.47275  4.85793  1.60963  4.67880  5.01181
 2.60238  3.62678  0.92913  3.40247  4.84304  5.03434  4.76644  4.64787
 3.87548  3.60001  2.27661  5.49044  4.00764     98 l - - -
          2.68618  4.42225  2.77519  2.73123  3.46354  2.40513  3.72494
 3.29354  2.67741  2.69355  4.24690  2.90347  2.73739  3.18146  2.89801
 2.37887  2.77519  2.98518  4.58477  3.61503
          0.00423  5.86384  6.58619  0.61958  0.77255  0.48576  0.95510
      47  1.74680  3.66451  2.42605  2.84203  4.15749  2.92093  4.34701
 3.74436  3.12328  3.12490  4.19384  2.98161  4.23163  2.68476  3.47840
 2.06408  2.81812  2.52656  6.11741  4.50376     99 a - - -
          2.68618  4.42225  2.77519  2.73123  3.46354  2.40513  3.72494
 3.29354  2.67741  2.69355  4.24690  2.90347  2.73739  3.18146  2.89801
 2.37887  2.77519  2.98518  4.58477  3.61503
          0.00423  5.86384  6.58619  0.61958  0.77255  0.48576  0.95510
      48  2.99795  5.27553  1.51726  1.06493  5.21315  4.11539  4.09377
 4.70021  2.93081  4.17396  4.91100  3.58201  4.15304  3.04992  3.39848
 2.60530  3.33587  4.25938  6.30156  4.88818    100 e - - -
```

```
         2.68618  4.42225  2.77519  2.73123  3.46354  2.40513  3.72494
3.29354  2.67741  2.69355  4.24690  2.90347  2.73739  3.18146  2.89801
2.37887  2.77519  2.98518  4.58477  3.61503
         0.00423  5.86384  6.58619  0.61958  0.77255  0.48576  0.95510
     49  3.15904  4.21029  5.42099  4.80682  3.91830  4.62483  4.96149
1.48832  4.59194  1.76903  3.33969  4.78900  4.98726  4.72314  4.59937
2.33422  3.04724  1.40008  5.45280  3.80531    101 v - - -
         2.68618  4.42225  2.77519  2.73123  3.46354  2.40513  3.72494
3.29354  2.67741  2.69355  4.24690  2.90347  2.73739  3.18146  2.89801
2.37887  2.77519  2.98518  4.58477  3.61503
         0.00423  5.86384  6.58619  0.61958  0.77255  0.48576  0.95510
     50  3.27901  4.30895  5.37653  4.76223  3.06942  4.59563  1.23180
2.94592  4.55043  1.39780  3.33130  3.77228  4.95947  4.46973  4.56392
3.90626  3.33852  2.49078  3.81336  3.54080    102 h - - -
         2.68618  4.42225  2.77519  2.73123  3.46354  2.40513  3.72494
3.29354  2.67741  2.69355  4.24690  2.90347  2.73739  3.18146  2.89801
2.37887  2.77519  2.98518  4.58477  3.61503
         0.00423  5.86384  6.58619  0.61958  0.77255  0.48576  0.95510
     51  2.70147  2.38073  5.36198  4.74865  1.86891  4.59364  4.09379
1.83229  4.20075  2.63236  3.04169  4.74646  4.95762  2.79982  3.97028
3.30042  2.98074  1.92978  5.42809  3.98019    103 i - - -
         2.68618  4.42225  2.77519  2.73123  3.46354  2.40513  3.72494
3.29354  2.67741  2.69355  4.24690  2.90347  2.73739  3.18146  2.89801
2.37887  2.77519  2.98518  4.58477  3.61503
         0.00423  5.86384  6.58619  0.61958  0.77255  0.48576  0.95510
     52  1.51466  3.11196  4.03933  3.47656  3.29496  2.15754  3.65394
3.78523  3.01751  3.03030  3.27689  3.48885  4.67443  3.29169  3.11044
2.35794  3.04723  2.61146  5.80613  4.54149    104 a - - -
         2.68618  4.42225  2.77519  2.73123  3.46354  2.40513  3.72494
3.29354  2.67741  2.69355  4.24690  2.90347  2.73739  3.18146  2.89801
2.37887  2.77519  2.98518  4.58477  3.61503
         0.00423  5.86384  6.58619  0.61958  0.77255  0.48576  0.95510
     53  3.26617  4.52456  5.44449  4.83045  3.09761  4.65242  4.98794
2.79686  4.61666  0.56366  3.36926  4.07000  5.01170  4.74473  4.62404
3.61767  3.70962  2.39207  5.47424  4.30128    105 L - - -
         2.68618  4.42225  2.77519  2.73123  3.46354  2.40513  3.72494
3.29354  2.67741  2.69355  4.24690  2.90347  2.73739  3.18146  2.89801
2.37887  2.77519  2.98518  4.58477  3.61503
         0.00423  5.86384  6.58619  0.61958  0.77255  0.48576  0.95510
     54  5.32364  6.41974  7.32492  6.73295  3.47496  6.86890  7.05926
2.63129  6.63928  0.28521  2.74684  7.08122  6.58959  6.21114  6.43592
6.34495  5.49027  2.92262  6.69487  5.88925    106 L - - -
         2.68618  4.42225  2.77519  2.73123  3.46354  2.40513  3.72494
3.29354  2.67741  2.69355  4.24690  2.90347  2.73739  3.18146  2.89801
2.37887  2.77519  2.98518  4.58477  3.61503
         0.00423  5.86384  6.58619  0.61958  0.77255  0.48576  0.95510
     55  3.11495  2.19132  3.58063  2.84494  5.16314  3.41446  3.54751
4.13657  1.91317  3.92161  4.86586  2.49555  4.49576  2.69039  1.96374
2.28955  3.01176  3.33624  6.25977  4.65189    107 k - - -
         2.68618  4.42225  2.77519  2.73123  3.46354  2.40513  3.72494
3.29354  2.67741  2.69355  4.24690  2.90347  2.73739  3.18146  2.89801
2.37887  2.77519  2.98518  4.58477  3.61503
         0.00423  5.86384  6.58619  0.61958  0.77255  0.48576  0.95510
     56  1.24772  3.22767  4.44187  4.21800  3.85316  4.27985  4.79904
2.92499  3.38123  1.82937  3.13970  3.68173  4.87215  3.23633  4.31329
2.99292  2.41623  2.72246  5.51779  4.05977    108 a - - -
```

```
          2.68618  4.42225  2.77519  2.73123  3.46354  2.40513  3.72494
3.29354  2.67741  2.69355  4.24690  2.90347  2.73739  3.18146  2.89801
2.37887  2.77519  2.98518  4.58477  3.61503
          0.00423  5.86384  6.58619  0.61958  0.77255  0.48576  0.95510
     57   2.22707  4.10513  4.70592  4.76712  3.61922  4.30902  4.46200
1.25722  4.55440  1.19707  3.64844  4.75591  4.96077  4.29666  4.56640
3.77550  3.66092  2.57234  5.42699  4.25078    109 l - - -
          2.68618  4.42225  2.77519  2.73123  3.46354  2.40513  3.72494
3.29354  2.67741  2.69355  4.24690  2.90347  2.73739  3.18146  2.89801
2.37887  2.77519  2.98518  4.58477  3.61503
          0.01496  5.86384  4.42240  0.61958  0.77255  0.48576  0.95510
     58   3.35591  4.30296  5.32095  4.08509  2.35623  4.58193  4.91219
1.93881  3.69615  1.54561  3.23424  4.72372  4.94623  3.83943  1.97822
3.71625  3.49389  1.91718  5.42471  4.24730    110 l - - -
          2.68618  4.42225  2.77519  2.73123  3.46354  2.40513  3.72494
3.29354  2.67741  2.69355  4.24690  2.90347  2.73739  3.18146  2.89801
2.37887  2.77519  2.98518  4.58477  3.61503
          0.04991  5.85316  3.08321  0.61958  0.77255  0.55443  0.85425
     59   2.71217  3.75900  2.93047  2.53819  5.14997  3.51777  3.77547
4.63665  2.17687  3.96195  4.84428  3.10432  4.45778  2.09313  1.49844
2.40772  3.04718  3.97290  6.23596  4.82441    111 r - - -
          2.68618  4.42225  2.77519  2.73123  3.46354  2.40513  3.72494
3.29354  2.67741  2.69355  4.24690  2.90347  2.73739  3.18146  2.89801
2.37887  2.77519  2.98518  4.58477  3.61503
          0.06595  5.80773  2.79982  0.61958  0.77255  0.79695  0.59911
     60   2.73879  5.76356  1.44235  1.89673  5.10267  4.03567  2.99373
4.58515  2.88657  2.03577  4.80611  3.00792  4.43158  3.14897  3.45873
2.77086  3.25982  3.90189  6.19949  4.79099    112 d - - -
          2.68618  4.42225  2.77519  2.73123  3.46354  2.40513  3.72494
3.29354  2.67741  2.69355  4.24690  2.90347  2.73739  3.18146  2.89801
2.37887  2.77519  2.98518  4.58477  3.61503
          0.00320  5.74498        *  0.61958  0.77255  0.00000        *
//
HMMER3/f [3.3 | Nov 2019]
NAME  DEAD
ACC   PF00270.30
DESC  DEAD/DEAH box helicase
LENG  176
ALPH  amino
RF    yes
MM    no
CONS  yes
CS    yes
MAP   yes
DATE  Mon Apr  6 11:52:26 2020
NSEQ  181
EFFN  4.565323
CKSUM 326570816
GA    26.00 24.10
TC    26.00 24.10
NC    25.90 24.00
STATS LOCAL MSV       -10.3196  0.70702
STATS LOCAL VITERBI   -11.4043  0.70702
STATS LOCAL FORWARD    -4.7208  0.70702
HMM          A        C        D        E        F        G        H
I        K        L        M        N        P        Q        R        S
T        V        W        Y
            m->m     m->i     m->d     i->m     i->i     d->m     d->d
```

```
  COMPO   2.53704  4.35697  2.94302  2.70487  3.45006  2.80640  3.77734
2.79666  2.66684  2.38519  3.72681  3.15704  3.24441  3.00138  2.99415
2.61043  2.67149  2.64162  4.96540  3.70471
          2.68618  4.42225  2.77519  2.73123  3.46354  2.40513  3.72494
3.29354  2.67741  2.69355  4.24690  2.90347  2.73739  3.18146  2.89801
2.37887  2.77519  2.98518  4.58477  3.61503
          0.00768  5.26922  5.99156  0.61958  0.77255  0.00000        *
      1   3.02261  3.96044  3.50444  3.21034  2.77456  3.96786  4.20885
3.10605  3.01431  3.02294  3.86581  2.86563  4.34974  3.49245  2.65164
2.60858  1.34890  3.11209  4.74116  3.10044      1 t x - -
          2.68618  4.42225  2.77519  2.73123  3.46354  2.40513  3.72494
3.29354  2.67741  2.69355  4.24690  2.90347  2.73739  3.18146  2.89801
2.37887  2.77519  2.98518  4.58477  3.61503
          0.00768  5.26922  5.99156  0.61958  0.77255  0.48576  0.95510
      2   2.30355  5.49762  1.94037  2.40706  4.83878  3.56850  3.86505
4.15517  2.44869  3.42956  4.41165  3.17375  2.04037  2.96096  2.57969
2.65118  3.04470  3.70385  5.93264  4.28926      2 d x - H
          2.68618  4.42225  2.77519  2.73123  3.46354  2.40513  3.72494
3.29354  2.67741  2.69355  4.24690  2.90347  2.73739  3.18146  2.89801
2.37887  2.77519  2.98518  4.58477  3.61503
          0.00768  5.26922  5.99156  0.61958  0.77255  0.48576  0.95510
      3   2.70185  4.20567  3.30737  3.68336  3.35990  3.48048  3.77971
1.24350  3.63482  2.55747  3.60771  3.63956  3.92739  3.88459  3.83094
3.29826  3.27300  1.98292  5.15146  3.16963      3 i x - H
          2.68618  4.42226  2.77520  2.73124  3.46354  2.40513  3.72495
3.29354  2.67741  2.69355  4.24690  2.90340  2.73740  3.18140  2.89801
2.37887  2.77520  2.98519  4.58477  3.61504
          0.02741  4.06833  4.61181  0.47367  0.97475  0.48576  0.95510
      4   3.00403  5.74241  3.56349  3.32269  5.29285  4.11170  3.89654
4.81505  3.12883  4.31776  5.18125  3.78897  4.68186  0.46016  3.42181
3.59785  3.61074  4.40191  6.39574  5.08110      5 Q x - H
          2.68618  4.42225  2.77519  2.73123  3.46354  2.40513  3.72494
3.29354  2.67741  2.69355  4.24690  2.90347  2.73739  3.18146  2.89801
2.37887  2.77519  2.98518  4.58477  3.61503
          0.00773  5.26175  5.98410  0.61958  0.77255  0.46920  0.98217
      5   1.66847  5.02283  2.92645  2.30553  4.73645  3.61823  3.90960
3.65167  2.30114  2.92004  3.95808  3.26732  4.03070  2.61915  2.58815
2.49231  2.99666  3.54846  4.73420  3.78585      6 a x - H
          2.68618  4.42225  2.77519  2.73123  3.46354  2.40513  3.72494
3.29354  2.67741  2.69355  4.24690  2.90347  2.73739  3.18146  2.89801
2.37887  2.77519  2.98518  4.58477  3.61503
          0.00768  5.26922  5.99156  0.61958  0.77255  0.48576  0.95510
      6   2.41046  4.97183  3.08452  1.60824  4.82411  3.71862  3.71899
3.52773  2.28911  3.01377  4.21918  3.01631  4.16655  2.25049  2.70244
2.65921  2.97801  3.57629  5.92496  4.03011      7 e x - H
          2.68618  4.42225  2.77519  2.73123  3.46354  2.40513  3.72494
3.29354  2.67741  2.69355  4.24690  2.90347  2.73739  3.18146  2.89801
2.37887  2.77519  2.98518  4.58477  3.61503
          0.00768  5.26922  5.99156  0.61958  0.77255  0.48576  0.95510
      7   1.61108  3.70856  4.53703  3.82741  3.52060  3.93581  4.45849
2.53218  3.82485  2.66780  3.57843  4.14475  4.52115  4.04014  3.99605
1.81085  2.38207  1.99719  5.12835  3.73810      8 a x - H
          2.68618  4.42225  2.77519  2.73123  3.46354  2.40513  3.72494
3.29354  2.67741  2.69355  4.24690  2.90347  2.73739  3.18146  2.89801
2.37887  2.77519  2.98518  4.58477  3.61503
          0.02925  5.26922  3.74322  0.61958  0.77255  0.48576  0.95510
```

8 2.86714 3.96905 5.00575 4.39022 2.73990 4.11923 4.54261
1.14693 4.00565 1.91289 3.17298 4.37225 4.57249 4.30506 3.98778
3.40355 3.27134 2.20169 3.86168 3.49132 9 i x - H
2.68618 4.42225 2.77519 2.73123 3.46354 2.40513 3.72494
3.29354 2.67741 2.69355 4.24690 2.90347 2.73739 3.18146 2.89801
2.37887 2.77519 2.98518 4.58477 3.61503
0.00784 5.24781 5.97016 0.61958 0.77255 0.56103 0.84542
9 2.64457 5.48547 2.75655 2.24999 4.65564 3.62382 3.58346
4.23123 2.39983 3.44521 4.12437 2.72123 1.51931 2.91964 3.01335
2.77296 3.00020 3.79048 5.92097 4.31688 10 p x - H
2.68618 4.42225 2.77519 2.73123 3.46354 2.40513 3.72494
3.29354 2.67741 2.69355 4.24690 2.90347 2.73739 3.18146 2.89801
2.37887 2.77519 2.98518 4.58477 3.61503
0.00784 5.24781 5.97016 0.61958 0.77255 0.56103 0.84542
10 2.39323 4.14211 3.24226 2.67973 3.97597 3.82019 3.72221
2.69870 2.94782 1.64420 3.78073 3.16707 3.42959 3.06973 3.16892
2.75483 2.73726 2.80395 4.37699 3.91089 11 l x - H
2.68618 4.42225 2.77519 2.73123 3.46354 2.40513 3.72494
3.29354 2.67741 2.69355 4.24690 2.90347 2.73739 3.18146 2.89801
2.37887 2.77519 2.98518 4.58477 3.61503
0.01446 5.24781 4.69973 0.61958 0.77255 0.56103 0.84542
11 2.26854 4.15080 4.97254 4.35908 2.95946 3.34199 4.53422
1.32113 4.14987 1.78206 3.39826 4.35564 4.56583 4.12888 4.16746
3.45588 2.87071 2.27862 4.53710 3.78878 12 i x - H
2.68618 4.42225 2.77519 2.73123 3.46354 2.40513 3.72494
3.29354 2.67741 2.69355 4.24690 2.90347 2.73739 3.18146 2.89801
2.37887 2.77519 2.98518 4.58477 3.61503
0.01291 5.24124 4.88823 0.61958 0.77255 0.58272 0.81731
12 2.76969 4.27849 4.02595 3.34040 3.53239 3.94007 3.89216
2.52552 3.29610 1.11737 3.18872 3.79755 4.06233 3.71999 3.52873
3.03909 3.00430 2.56655 5.15425 3.15923 13 l x - H
2.68637 4.42255 2.77519 2.73136 3.46375 2.40502 3.72418
3.29373 2.67756 2.69354 4.24548 2.90334 2.73736 3.18142 2.89774
2.37891 2.77546 2.98534 4.58507 3.61480
0.34165 1.71993 2.20431 1.13753 0.38656 0.42108 1.06809
13 2.65350 5.45163 2.78533 2.09090 4.18031 2.08230 3.67998
4.19458 2.37483 3.57617 4.18698 2.85782 3.39368 2.56672 2.86633
2.34851 2.91173 3.84147 5.88521 3.69808 24 g x - T
2.68618 4.42225 2.77519 2.73123 3.46354 2.40513 3.72494
3.29354 2.67741 2.69355 4.24690 2.90347 2.73739 3.18146 2.89801
2.37887 2.77519 2.98518 4.58477 3.61503
0.00857 5.15912 5.88147 0.61958 0.77255 0.63390 0.75613
14 2.84878 5.47167 2.91773 2.45113 4.54410 1.22149 3.89600
4.28805 2.42967 3.72025 4.51572 2.68331 3.85180 2.92462 2.99717
2.84887 3.01795 3.85753 5.90924 4.50200 25 g x - T
2.68613 4.42245 2.77534 2.73141 3.46315 2.40533 3.72479
3.29354 2.67729 2.69364 4.24710 2.90332 2.73707 3.18163 2.89765
2.37891 2.77528 2.98524 4.58497 3.61515
0.12732 2.59107 3.10979 1.53788 0.24186 0.39554 1.11876
15 2.84755 5.27439 2.89869 2.15888 4.15176 1.95554 3.26435
4.31177 1.96929 3.74756 4.27672 3.02294 4.04557 2.84092 2.14742
2.79955 3.17726 3.83859 5.91524 4.39054 38 g x - H
2.68619 4.42197 2.77522 2.73126 3.46356 2.40515 3.72490
3.29356 2.67739 2.69357 4.24692 2.90346 2.73742 3.18132 2.89795
2.37886 2.77522 2.98521 4.58479 3.61503
0.05998 2.88975 5.94379 0.27905 1.41263 0.39787 1.11397

```
     16   2.96221  5.15332  1.16888  2.68441  4.79619  3.50779  3.64284
4.26920  2.69708  3.76746  3.82885  2.29826  4.09094  3.02669  3.07345
2.57065  3.23687  3.45502  5.91466  4.51484      40 d x - E
          2.68618  4.42225  2.77519  2.73123  3.46354  2.40513  3.72494
3.29354  2.67741  2.69355  4.24690  2.90347  2.73739  3.18146  2.89801
2.37887  2.77519  2.98518  4.58477  3.61503
          0.00768  5.26922  5.99156  0.61958  0.77255  0.48576  0.95510
     17   2.66679  3.49225  5.02666  4.41270  3.13058  4.23787  4.57324
2.15092  4.19947  2.04389  2.72774  4.23635  4.60055  4.16497  4.20976
3.54969  2.94972  1.13262  5.06720  3.80678      41 v x - E
          2.68618  4.42225  2.77519  2.73123  3.46354  2.40513  3.72494
3.29354  2.67741  2.69355  4.24690  2.90347  2.73739  3.18146  2.89801
2.37887  2.77519  2.98518  4.58477  3.61503
          0.00768  5.26922  5.99156  0.61958  0.77255  0.48576  0.95510
     18   2.81420  3.28933  5.01277  3.86595  2.61392  4.21981  4.55330
1.82941  4.18272  1.33333  3.03950  4.26011  4.58350  4.20445  4.12591
3.39078  3.09685  2.09888  4.83325  3.70406      42 l x - E
          2.68623  4.42233  2.77510  2.73133  3.46369  2.40535  3.72490
3.29361  2.67751  2.69315  4.24532  2.90350  2.73725  3.18162  2.89751
2.37899  2.77542  2.98529  4.58488  3.61524
          0.07809  2.62247  5.99156  1.76461  0.18784  0.48576  0.95510
     19   2.25489  4.09676  4.99661  4.38753  3.56134  2.55763  4.59009
2.04590  4.18644  2.08672  2.04935  4.39914  4.61615  4.25154  4.21533
3.56443  3.27747  1.59596  5.09810  3.92049      51 v x - E
          2.68618  4.42225  2.77519  2.73123  3.46354  2.40513  3.72494
3.29354  2.67741  2.69355  4.24690  2.90347  2.73739  3.18146  2.89801
2.37887  2.77519  2.98518  4.58477  3.61503
          0.00768  5.26922  5.99156  0.61958  0.77255  0.48576  0.95510
     20   1.86763  3.24768  3.19934  2.57535  4.54877  3.18358  3.63493
3.22493  2.66409  3.15184  3.96006  3.15335  4.20746  2.26149  2.88777
2.33156  3.08743  2.81182  5.77190  4.09768      52 a x - E
          2.68618  4.42225  2.77519  2.73123  3.46354  2.40513  3.72494
3.29354  2.67741  2.69355  4.24690  2.90347  2.73739  3.18146  2.89801
2.37887  2.77519  2.98518  4.58477  3.61503
          0.00768  5.26922  5.99156  0.61958  0.77255  0.48576  0.95510
     21   1.02962  4.68660  4.10426  3.53867  3.84521  2.53301  4.36383
2.75816  3.47543  2.74949  3.03566  3.88421  4.43965  3.65883  3.78489
2.71319  3.02673  2.97625  5.31310  4.09558      53 a x - E
          2.68618  4.42225  2.77519  2.73123  3.46354  2.40513  3.72494
3.29354  2.67741  2.69355  4.24690  2.90347  2.73739  3.18146  2.89801
2.37887  2.77519  2.98518  4.58477  3.61503
          0.00768  5.26922  5.99156  0.61958  0.77255  0.48576  0.95510
     22   2.38699  5.50731  2.78790  1.73019  4.85194  3.32186  3.69648
4.33768  2.48339  3.81238  4.54815  3.15544  2.06543  2.51985  2.74066
2.79552  3.22865  3.24877  5.94018  4.52935      54 e x - S
          2.68618  4.42225  2.77519  2.73123  3.46354  2.40513  3.72494
3.29354  2.67741  2.69355  4.24690  2.90347  2.73739  3.18146  2.89801
2.37887  2.77519  2.98518  4.58477  3.61503
          0.00768  5.26922  5.99156  0.61958  0.77255  0.48576  0.95510
     23   2.98103  4.92854  4.50110  4.10265  4.72051  3.87930  4.99299
4.07150  3.83159  3.80838  4.67561  4.06051  3.97031  4.33885  4.32241
2.90790  0.49945  2.59486  6.13842  4.94623      55 T x - T
          2.68618  4.42225  2.77519  2.73123  3.46354  2.40513  3.72494
3.29354  2.67741  2.69355  4.24690  2.90347  2.73739  3.18146  2.89801
2.37887  2.77519  2.98518  4.58477  3.61503
          0.00768  5.26922  5.99156  0.61958  0.77255  0.48576  0.95510
```

```
     24   2.53443  5.04547  4.93096  4.94823  5.89697  0.29087  5.86245
5.41763  5.17473  5.10508  5.84643  4.59494  4.68490  5.25874  5.25438
2.82744  3.70863  4.47081  7.22385  6.17492     56 G x - T
          2.68618  4.42225  2.77519  2.73123  3.46354  2.40513  3.72494
3.29354  2.67741  2.69355  4.24690  2.90347  2.73739  3.18146  2.89801
2.37887  2.77519  2.98518  4.58477  3.61503
          0.00768  5.26922  5.99156  0.61958  0.77255  0.48576  0.95510
     25   2.40767  4.40637  4.18770  3.61242  3.35773  2.97176  4.35847
2.84642  3.53655  2.76632  3.29206  3.85910  4.45424  3.74891  3.81406
1.14974  2.62908  2.87900  4.85559  3.25993     57 s x - S
          2.68618  4.42225  2.77519  2.73123  3.46354  2.40513  3.72494
3.29354  2.67741  2.69355  4.24690  2.90347  2.73739  3.18146  2.89801
2.37887  2.77519  2.98518  4.58477  3.61503
          0.00768  5.26922  5.99156  0.61958  0.77255  0.48576  0.95510
     26   4.99673  6.45377  5.84570  5.91455  6.74411  0.04532  6.74547
6.81678  6.20439  6.18124  7.28276  6.02020  5.82930  6.44120  6.14952
5.22612  5.53970  6.14351  7.35435  6.93573     58 G x - S
          2.68618  4.42225  2.77519  2.73123  3.46354  2.40513  3.72494
3.29354  2.67741  2.69355  4.24690  2.90347  2.73739  3.18146  2.89801
2.37887  2.77519  2.98518  4.58477  3.61503
          0.00768  5.26922  5.99156  0.61958  0.77255  0.48576  0.95510
     27   5.01328  6.59203  5.28076  4.81690  6.35512  5.09487  5.44144
6.00271  0.10780  5.30352  6.32757  5.12163  5.61158  4.66299  3.81184
5.06274  5.22700  5.67797  6.92068  6.08154     59 K x - H
          2.68618  4.42225  2.77519  2.73123  3.46354  2.40513  3.72494
3.29354  2.67741  2.69355  4.24690  2.90347  2.73739  3.18146  2.89801
2.37887  2.77519  2.98518  4.58477  3.61503
          0.00768  5.26922  5.99156  0.61958  0.77255  0.48576  0.95510
     28   3.12568  5.04404  5.00049  4.91719  5.70172  3.83032  5.75274
5.17074  4.94317  4.89219  5.67934  4.59070  4.68741  5.13756  5.05633
1.91618  0.37296  4.36479  7.04915  5.95287     60 T x - H
          2.68618  4.42225  2.77519  2.73123  3.46354  2.40513  3.72494
3.29354  2.67741  2.69355  4.24690  2.90347  2.73739  3.18146  2.89801
2.37887  2.77519  2.98518  4.58477  3.61503
          0.00768  5.26922  5.99156  0.61958  0.77255  0.48576  0.95510
     29   2.20805  4.81364  3.62618  2.30898  3.30545  2.16043  4.14385
2.96111  3.13670  1.79396  3.52327  3.18619  4.35672  3.51144  3.57821
3.18958  2.78151  2.82365  5.38594  4.00947     61 l x - H
          2.68618  4.42225  2.77519  2.73123  3.46354  2.40513  3.72494
3.29354  2.67741  2.69355  4.24690  2.90347  2.73739  3.18146  2.89801
2.37887  2.77519  2.98518  4.58477  3.61503
          0.00768  5.26922  5.99156  0.61958  0.77255  0.48576  0.95510
     30   1.13345  3.16009  4.80594  4.20907  3.58887  3.80470  4.54290
2.46733  4.04276  2.50080  3.46789  4.29648  4.57438  4.21697  4.13226
2.90512  3.05711  1.86819  5.11360  3.93155     62 a x - H
          2.68623  4.42230  2.77525  2.73125  3.46327  2.40515  3.72500
3.29332  2.67732  2.69345  4.24695  2.90352  2.73729  3.18152  2.89806
2.37892  2.77525  2.98524  4.58482  3.61508
          0.04671  3.55683  4.06809  1.09524  0.40715  0.48576  0.95510
     31   1.97990  4.51490  4.84256  4.25070  1.24463  3.87056  4.49447
2.86200  4.07938  2.66602  3.63464  4.31955  4.60749  4.24206  4.15923
3.36949  3.15232  2.76912  4.03556  2.05621     68 f x - H
          2.68618  4.42225  2.77519  2.73123  3.46354  2.40513  3.72494
3.29354  2.67741  2.69355  4.24690  2.90347  2.73739  3.18146  2.89801
2.37887  2.77519  2.98518  4.58477  3.61503
          0.00779  5.25450  5.97684  0.61958  0.77255  0.51979  0.90299
```

32 2.51399 3.61322 4.43018 3.17239 3.13391 3.23265 4.11718
2.75071 3.73668 1.81895 2.87382 3.93290 4.31141 2.95792 3.73398
1.95292 3.16083 2.27999 4.12564 3.85966 69 l x - H
2.68618 4.42225 2.77519 2.73123 3.46354 2.40513 3.72494
3.29354 2.67741 2.69355 4.24690 2.90347 2.73739 3.18146 2.89801
2.37887 2.77519 2.98518 4.58477 3.61503
0.00777 5.25765 5.98000 0.61958 0.77255 0.46062 0.99667
33 2.78500 4.44302 4.89296 4.11759 3.05854 4.13783 4.40910
1.30459 3.53621 1.52035 3.43290 4.32703 4.56976 3.89098 3.49439
3.47650 3.18949 2.35450 4.92852 3.58755 70 i x - H
2.68618 4.42225 2.77519 2.73123 3.46354 2.40513 3.72494
3.29354 2.67741 2.69355 4.24690 2.90347 2.73739 3.18146 2.89801
2.37887 2.77519 2.98518 4.58477 3.61503
0.00768 5.26922 5.99156 0.61958 0.77255 0.48576 0.95510
34 2.11654 4.66378 3.67635 3.19099 3.83305 3.54781 4.04573
3.29854 3.15904 2.92285 3.94432 3.26519 1.17625 3.47706 3.54958
2.98211 3.04671 3.00257 4.66328 3.92990 71 p x - H
2.68618 4.42225 2.77519 2.73123 3.46354 2.40513 3.72494
3.29354 2.67741 2.69355 4.24690 2.90347 2.73739 3.18146 2.89801
2.37887 2.77519 2.98518 4.58477 3.61503
0.00768 5.26922 5.99156 0.61958 0.77255 0.48576 0.95510
35 2.01410 3.96022 4.99312 4.37937 3.36647 3.87911 4.55096
1.83738 4.16914 1.81071 2.86098 4.37364 4.39111 4.30453 3.95368
3.33815 3.12181 1.62591 5.05113 3.62695 72 v x - H
2.68618 4.42225 2.77519 2.73123 3.46354 2.40513 3.72494
3.29354 2.67741 2.69355 4.24690 2.90347 2.73739 3.18146 2.89801
2.37887 2.77519 2.98518 4.58477 3.61503
0.00768 5.26922 5.99156 0.61958 0.77255 0.48576 0.95510
36 2.58702 3.90840 5.00092 4.16179 2.79404 4.21830 4.41670
1.56397 4.17425 1.45302 3.43602 3.76297 4.58211 4.30794 3.84826
3.52902 3.28257 2.04877 4.70632 3.48576 73 l x - H
2.68617 4.42229 2.77507 2.73127 3.46326 2.40505 3.72498
3.29358 2.67745 2.69359 4.24672 2.90350 2.73743 3.18150 2.89804
2.37891 2.77523 2.98516 4.58481 3.61507
0.15242 3.52905 2.18890 0.86716 0.54497 0.48576 0.95510
37 2.50321 4.95139 2.67758 2.24427 4.78418 3.65746 3.73482
4.00115 2.36754 3.56395 4.48482 2.83425 4.03136 1.55257 2.90996
2.60588 3.08856 3.37702 5.87819 3.58727 77 q x - H
2.68618 4.42225 2.77519 2.73123 3.46354 2.40513 3.72494
3.29354 2.67741 2.69355 4.24690 2.90347 2.73739 3.18146 2.89801
2.37887 2.77519 2.98518 4.58477 3.61503
0.00863 5.15320 5.87554 0.61958 0.77255 0.82530 0.57644
38 2.45764 3.78125 3.43845 2.63137 4.07859 3.62198 3.40780
2.05301 2.46305 2.76773 3.79130 3.10476 4.21774 2.86191 2.64890
2.59370 2.70082 3.07180 4.30865 3.78683 78 i x - H
2.68618 4.42225 2.77519 2.73123 3.46354 2.40513 3.72494
3.29354 2.67741 2.69355 4.24690 2.90347 2.73739 3.18146 2.89801
2.37887 2.77519 2.98518 4.58477 3.61503
0.01164 5.15320 5.15067 0.61958 0.77255 0.82530 0.57644
39 2.79029 4.25519 4.94606 4.33138 2.61386 4.15869 4.38558
1.88021 4.11802 1.55046 3.29898 4.31873 4.52197 4.14857 3.97753
3.29460 3.19530 1.53787 4.49127 3.81128 79 v x - H
2.68618 4.42225 2.77519 2.73123 3.46354 2.40513 3.72494
3.29354 2.67741 2.69355 4.24690 2.90347 2.73739 3.18146 2.89801
2.37887 2.77519 2.98518 4.58477 3.61503
0.21132 5.15021 1.68909 0.61958 0.77255 0.83227 0.57103

40 2.52470 4.39283 4.20843 3.60027 3.12026 3.78149 3.83258
2.59662 3.20833 2.07440 3.31694 3.56300 4.32806 3.55296 3.74948
2.46596 2.48250 2.58649 3.97729 1.93979 80 y x - H
2.68618 4.42225 2.77519 2.73123 3.46354 2.40513 3.72494
3.29354 2.67741 2.69355 4.24690 2.90347 2.73739 3.18146 2.89801
2.37887 2.77519 2.98518 4.58477 3.61503
0.07645 4.94947 2.71041 0.61958 0.77255 0.85872 0.55113
41 2.73113 5.37540 2.72043 1.67266 4.72036 3.48471 3.24594
4.20622 2.25638 3.54124 4.41611 2.54711 3.98700 2.71995 2.54974
2.33112 2.88930 3.73246 5.11055 4.28137 81 e x - H
2.68618 4.42225 2.77519 2.73123 3.46354 2.40513 3.72494
3.29354 2.67741 2.69355 4.24690 2.90347 2.73739 3.18146 2.89801
2.37887 2.77519 2.98518 4.58477 3.61503
0.26272 4.97429 1.49553 0.61958 0.77255 1.14984 0.38080
42 2.72227 5.07217 2.89665 2.41732 4.26654 3.06636 3.53132
3.77474 2.53020 2.74495 3.66486 2.97034 3.97333 2.74207 2.85011
2.64661 1.72245 3.39307 5.55409 3.81948 82 t x - S
2.68727 4.42350 2.77389 2.73010 3.46433 2.40512 3.72586
3.29372 2.67521 2.69324 4.24664 2.90365 2.73793 3.17812 2.89869
2.37981 2.77604 2.98639 4.58565 3.61632
1.20980 0.36035 5.44718 1.85123 0.17084 0.70153 0.68484
43 2.79840 5.36582 2.74588 2.26943 3.73072 3.40534 3.56757
4.01020 1.60225 3.17546 4.34022 2.90117 3.24464 2.85566 2.48813
2.72768 2.81885 3.51078 5.80256 4.22563 95 k x - T
2.68622 4.42212 2.77516 2.73127 3.46353 2.40517 3.72498
3.29353 2.67728 2.69359 4.24694 2.90350 2.73728 3.18150 2.89805
2.37891 2.77516 2.98514 4.58481 3.61507
0.08047 2.79054 4.13972 0.59512 0.80183 0.45021 1.01471
44 2.80626 4.57690 2.87761 2.41377 4.76136 2.09180 3.67888
3.64778 2.33587 2.74225 4.23663 2.69719 3.70265 2.72138 2.63655
2.56008 2.69421 3.35748 5.87048 4.37192 98 g x - T
2.68618 4.42225 2.77519 2.73123 3.46354 2.40513 3.72494
3.29354 2.67741 2.69355 4.24690 2.90347 2.73739 3.18146 2.89801
2.37887 2.77519 2.98518 4.58477 3.61503
0.00846 5.17275 5.89509 0.61958 0.77255 0.34847 1.22339
45 2.80353 4.92523 2.51347 2.36559 3.73007 3.34311 3.81820
3.65884 1.74635 2.95804 4.19713 2.99093 3.37335 2.82058 2.76960
2.64986 2.76931 3.13549 4.89378 4.03220 99 k x - T
2.68618 4.42225 2.77519 2.73123 3.46354 2.40513 3.72494
3.29354 2.67741 2.69355 4.24690 2.90347 2.73739 3.18146 2.89801
2.37887 2.77519 2.98518 4.58477 3.61503
0.00770 5.26669 5.98903 0.61958 0.77255 0.49505 0.94044
46 2.67344 4.43677 2.64469 2.35008 4.72042 2.81484 3.65829
3.25929 2.46927 3.51928 4.40716 2.62221 3.00065 2.81420 2.88607
2.63914 2.00103 3.55923 5.92414 4.05193 100 t x - T
2.68618 4.42225 2.77519 2.73123 3.46354 2.40513 3.72494
3.29354 2.67741 2.69355 4.24690 2.90347 2.73739 3.18146 2.89801
2.37887 2.77519 2.98518 4.58477 3.61503
0.00770 5.26669 5.98903 0.61958 0.77255 0.49505 0.94044
47 2.49081 5.19710 2.93853 2.59425 4.82762 1.93139 3.51988
4.30878 2.21255 3.58528 4.38531 3.07995 3.64773 2.91939 2.71625
2.68344 2.02943 3.49329 5.92621 4.30821 101 g x - S
2.68762 4.38775 2.77650 2.73619 3.43833 2.39956 3.71334
3.29521 2.66816 2.69208 4.24660 2.90395 2.74661 3.18041 2.90860
2.38605 2.78101 2.98777 4.57665 3.61335
0.29033 1.60960 2.95616 5.09467 0.00615 0.49505 0.94044

```
     48   2.66394  4.62991  3.53142  2.96841  3.56075  3.09363  4.12365
2.94627  2.63393  2.56053  3.89543  3.14888  1.69876  3.30357  3.09316
2.72532  2.88522  2.74534  5.46399  3.49652    308 p x - -
          2.68618  4.42225  2.77519  2.73123  3.46354  2.40513  3.72494
3.29354  2.67741  2.69355  4.24690  2.90347  2.73739  3.18146  2.89801
2.37887  2.77519  2.98518  4.58477  3.61503
          0.00810  5.21591  5.93825  0.61958  0.77255  0.40879  1.09199
     49   2.99870  5.35418  3.31484  2.71914  3.93405  3.40661  3.81407
3.65033  1.63167  3.13579  3.58320  3.25920  4.01510  2.24303  2.02654
2.77776  2.99136  3.33707  5.82389  3.49811    309 k x - E
          2.68618  4.42225  2.77519  2.73123  3.46354  2.40513  3.72494
3.29354  2.67741  2.69355  4.24690  2.90347  2.73739  3.18146  2.89801
2.37887  2.77519  2.98518  4.58477  3.61503
          0.00771  5.26512  5.98747  0.61958  0.77255  0.47652  0.97006
     50   1.33164  3.04585  4.75130  4.15256  3.31606  3.18587  4.50597
2.53337  3.91313  2.53726  3.54613  4.25859  4.55260  4.17057  4.09104
3.02421  2.34682  1.97892  5.08677  3.79174    310 a x - E
          2.68618  4.42225  2.77519  2.73123  3.46354  2.40513  3.72494
3.29354  2.67741  2.69355  4.24690  2.90347  2.73739  3.18146  2.89801
2.37887  2.77519  2.98518  4.58477  3.61503
          0.00768  5.26922  5.99156  0.61958  0.77255  0.48576  0.95510
     51   3.01658  4.79622  5.62580  5.04088  3.01168  4.91622  5.29309
1.69592  4.86458  0.91100  3.50919  5.08084  5.19479  4.95266  4.87564
4.25657  3.85753  1.76039  5.66037  4.53332    311 l x - E
          2.68618  4.42225  2.77519  2.73123  3.46354  2.40513  3.72494
3.29354  2.67741  2.69355  4.24690  2.90347  2.73739  3.18146  2.89801
2.37887  2.77519  2.98518  4.58477  3.61503
          0.00768  5.26922  5.99156  0.61958  0.77255  0.48576  0.95510
     52   3.08776  3.93339  5.05428  4.44109  3.00629  4.26325  4.24258
1.38192  4.22753  2.03607  3.47744  4.42551  4.62367  4.35742  4.23579
3.57598  3.32010  1.57271  5.08349  2.46819    312 i x - E
          2.68618  4.42225  2.77519  2.73123  3.46354  2.40513  3.72494
3.29354  2.67741  2.69355  4.24690  2.90347  2.73739  3.18146  2.89801
2.37887  2.77519  2.98518  4.58477  3.61503
          0.00768  5.26922  5.99156  0.61958  0.77255  0.48576  0.95510
     53   3.22529  4.49192  5.19893  4.59686  3.33254  4.43710  4.79283
1.68983  4.39618  1.59063  2.99987  4.59741  4.78538  4.53205  4.41483
3.26470  3.10833  1.26855  5.27189  4.09709    313 v x - E
          2.68618  4.42225  2.77519  2.73123  3.46354  2.40513  3.72494
3.29354  2.67741  2.69355  4.24690  2.90347  2.73739  3.18146  2.89801
2.37887  2.77519  2.98518  4.58477  3.61503
          0.00768  5.26922  5.99156  0.61958  0.77255  0.48576  0.95510
     54   1.90184  4.04852  3.86413  1.89032  3.76149  3.99492  4.24497
3.23243  3.25621  2.40816  3.87473  3.60371  4.37530  3.56245  3.62047
2.25438  2.73670  2.08669  5.35146  3.77158    314 e x - E
          2.68618  4.42225  2.77519  2.73123  3.46354  2.40513  3.72494
3.29354  2.67741  2.69355  4.24690  2.90347  2.73739  3.18146  2.89801
2.37887  2.77519  2.98518  4.58477  3.61503
          0.00768  5.26922  5.99156  0.61958  0.77255  0.48576  0.95510
     55   3.22779  5.17120  4.36713  4.33428  5.62877  3.88106  5.02388
5.20396  4.49933  4.85725  5.67402  4.37946  0.29964  4.80604  4.71079
3.19945  3.15137  4.44417  6.95482  5.74963    315 P x - S
          2.68618  4.42225  2.77519  2.73123  3.46354  2.40513  3.72494
3.29354  2.67741  2.69355  4.24690  2.90347  2.73739  3.18146  2.89801
2.37887  2.77519  2.98518  4.58477  3.61503
          0.00768  5.26922  5.99156  0.61958  0.77255  0.48576  0.95510
```

```
     56   3.02820  4.74521  3.91189  3.06562  3.88139  4.00911  4.26283
3.10162  3.29667  2.19173  3.16326  3.71619  4.38834  3.52995  3.04411
1.96665  1.41793  2.83679  5.32752  3.57098    316 t x - S
          2.68618  4.42225  2.77519  2.73123  3.46354  2.40513  3.72494
3.29354  2.67741  2.69355  4.24690  2.90347  2.73739  3.18146  2.89801
2.37887  2.77519  2.98518  4.58477  3.61503
          0.00768  5.26922  5.99156  0.61958  0.77255  0.48576  0.95510
     57   2.86665  5.38478  3.32620  2.60509  4.67949  3.69649  3.44009
3.18754  2.03298  3.37843  4.44004  3.30233  4.20134  3.11509  1.27253
2.73970  2.89381  3.42043  5.84647  4.37281    317 r x - H
          2.68618  4.42225  2.77519  2.73123  3.46354  2.40513  3.72494
3.29354  2.67741  2.69355  4.24690  2.90347  2.73739  3.18146  2.89801
2.37887  2.77519  2.98518  4.58477  3.61503
          0.00768  5.26922  5.99156  0.61958  0.77255  0.48576  0.95510
     58   2.05750  5.32281  3.26921  1.24868  4.59966  3.61778  4.00818
3.55394  2.77961  3.16017  4.38579  3.23458  3.66645  3.13436  3.25664
2.40746  3.11019  3.09277  5.80328  4.43975    318 e x - H
          2.68618  4.42225  2.77519  2.73123  3.46354  2.40513  3.72494
3.29354  2.67741  2.69355  4.24690  2.90347  2.73739  3.18146  2.89801
2.37887  2.77519  2.98518  4.58477  3.61503
          0.00768  5.26922  5.99156  0.61958  0.77255  0.48576  0.95510
     59   4.07302  5.39304  5.79759  5.24482  3.81189  5.28206  5.61941
2.77638  4.46358  0.31274  3.52157  5.40403  5.49645  5.08664  4.95528
4.25000  4.30583  3.12335  5.88239  4.81037    319 L x - H
          2.68618  4.42225  2.77519  2.73123  3.46354  2.40513  3.72494
3.29354  2.67741  2.69355  4.24690  2.90347  2.73739  3.18146  2.89801
2.37887  2.77519  2.98518  4.58477  3.61503
          0.00768  5.26922  5.99156  0.61958  0.77255  0.48576  0.95510
     60   1.23876  3.30740  4.36614  4.00125  3.60047  3.82013  4.46925
2.27191  3.80332  2.61500  2.72981  3.87044  4.52866  3.84186  3.87769
3.08468  2.67704  2.26259  5.11324  3.92486    320 a x - H
          2.68618  4.42225  2.77519  2.73123  3.46354  2.40513  3.72494
3.29354  2.67741  2.69355  4.24690  2.90347  2.73739  3.18146  2.89801
2.37887  2.77519  2.98518  4.58477  3.61503
          0.00768  5.26922  5.99156  0.61958  0.77255  0.48576  0.95510
     61   2.67176  5.47857  3.02584  1.66083  3.87619  3.77494  3.60946
3.86687  2.38335  2.89590  4.08412  2.54791  3.74543  2.41629  2.73448
2.75250  3.02103  3.11095  5.07966  4.35368    321 e x - H
          2.68618  4.42225  2.77519  2.73123  3.46354  2.40513  3.72494
3.29354  2.67741  2.69355  4.24690  2.90347  2.73739  3.18146  2.89801
2.37887  2.77519  2.98518  4.58477  3.61503
          0.00768  5.26922  5.99156  0.61958  0.77255  0.48576  0.95510
     62   2.80555  5.68213  2.30371  2.44304  4.63948  3.83347  4.09110
4.50626  2.79711  3.98195  4.73329  2.79721  4.27558  0.96566  3.31801
3.03665  3.39663  4.06778  6.11489  4.68857    322 q x - H
          2.68618  4.42225  2.77519  2.73123  3.46354  2.40513  3.72494
3.29354  2.67741  2.69355  4.24690  2.90347  2.73739  3.18146  2.89801
2.37887  2.77519  2.98518  4.58477  3.61503
          0.00768  5.26922  5.99156  0.61958  0.77255  0.48576  0.95510
     63   3.00041  4.15101  4.44487  3.86017  3.64099  4.13412  3.20777
1.97606  3.23531  2.51229  2.59758  4.09131  4.50448  2.67598  3.77595
3.34574  1.85315  1.90442  5.14497  3.95034    323 t x - H
          2.68618  4.42225  2.77519  2.73123  3.46354  2.40513  3.72494
3.29354  2.67741  2.69355  4.24690  2.90347  2.73739  3.18146  2.89801
2.37887  2.77519  2.98518  4.58477  3.61503
          0.00768  5.26922  5.99156  0.61958  0.77255  0.48576  0.95510
```

```
     64   2.34904  4.11616  4.56730  3.03757  2.76702  3.36080  3.65239
2.75425  3.39009  1.64537  3.26384  4.16063  4.52467  3.67450  3.92417
3.26305  2.93806  2.13241  5.11771  2.40209    324 l x - H
          2.68618  4.42225  2.77519  2.73123  3.46354  2.40513  3.72494
3.29354  2.67741  2.69355  4.24690  2.90347  2.73739  3.18146  2.89801
2.37887  2.77519  2.98518  4.58477  3.61503
          0.00768  5.26922  5.99156  0.61958  0.77255  0.48576  0.95510
     65   2.75811  5.50719  2.54929  1.96816  4.85254  3.53020  3.61941
3.60651  2.27678  3.40723  4.16626  1.90988  4.16306  2.67515  2.55689
2.63109  2.87250  3.89798  5.93971  4.52860    325 n x - H
          2.68618  4.42225  2.77519  2.73123  3.46354  2.40513  3.72494
3.29354  2.67741  2.69355  4.24690  2.90347  2.73739  3.18146  2.89801
2.37887  2.77519  2.98518  4.58477  3.61503
          0.00768  5.26922  5.99156  0.61958  0.77255  0.48576  0.95510
     66   2.79914  5.50218  2.74309  2.12884  4.60345  3.77055  3.66973
4.04168  2.42477  3.74402  4.54335  1.83144  4.16399  2.73118  2.46082
2.68879  2.55215  2.69957  5.93601  4.52622    326 n x - H
          2.68618  4.42225  2.77519  2.73123  3.46354  2.40513  3.72494
3.29354  2.67741  2.69355  4.24690  2.90347  2.73739  3.18146  2.89801
2.37887  2.77519  2.98518  4.58477  3.61503
          0.00768  5.26922  5.99156  0.61958  0.77255  0.48576  0.95510
     67   2.48429  3.74628  5.02171  4.40592  2.17713  4.03166  4.55543
2.36339  4.18929  1.55496  3.20805  4.38609  4.58505  4.31919  4.19509
3.53308  2.82526  1.68772  4.24142  3.41636    327 l x - H
          2.68620  4.42227  2.77518  2.73115  3.46356  2.40515  3.72497
3.29349  2.67743  2.69354  4.24676  2.90345  2.73742  3.18148  2.89803
2.37886  2.77517  2.98520  4.58479  3.61505
          0.03334  3.83795  4.48723  0.74872  0.64050  0.48576  0.95510
     68   2.70149  5.20498  3.17747  2.23106  4.51221  3.45789  3.83939
3.24583  1.53066  3.28471  4.52515  2.95335  4.16251  2.62348  2.45063
2.51812  2.86605  3.42731  5.92009  4.46578    331 k x - H
          2.68618  4.42225  2.77519  2.73123  3.46354  2.40513  3.72494
3.29354  2.67741  2.69355  4.24690  2.90347  2.73739  3.18146  2.89801
2.37887  2.77519  2.98518  4.58477  3.61503
          0.00774  5.26042  5.98277  0.61958  0.77255  0.46638  0.98689
     69   2.68101  5.50616  2.98406  2.13462  4.72300  3.65426  3.84255
4.16729  1.79391  3.63884  4.54686  2.84896  3.97926  1.92201  2.72237
2.59732  2.68592  3.68864  5.54886  3.75036    332 k x - H
          2.68618  4.42225  2.77519  2.73123  3.46354  2.40513  3.72494
3.29354  2.67741  2.69355  4.24690  2.90347  2.73739  3.18146  2.89801
2.37887  2.77519  2.98518  4.58477  3.61503
          0.00768  5.26922  5.99156  0.61958  0.77255  0.48576  0.95510
     70   2.68817  4.16075  4.42343  3.85810  1.81608  4.17081  4.48381
2.58792  3.37773  1.86024  3.25277  4.02199  3.80137  3.92838  2.73450
2.67220  3.18459  2.50397  4.27360  2.82590    333 f x - H
          2.68618  4.42225  2.77519  2.73123  3.46354  2.40513  3.72494
3.29354  2.67741  2.69355  4.24690  2.90347  2.73739  3.18146  2.89801
2.37887  2.77519  2.98518  4.58477  3.61503
          0.00913  5.26922  5.53708  0.61958  0.77255  0.48576  0.95510
     71   2.58008  3.81674  3.95275  3.37111  2.64390  2.40847  4.18339
2.57345  2.00330  2.17097  3.11810  3.42847  3.89429  3.71107  3.67480
2.94117  2.86369  2.79642  5.26053  3.71958    334 k x - T
          2.68618  4.42225  2.77519  2.73123  3.46354  2.40513  3.72494
3.29354  2.67741  2.69355  4.24690  2.90347  2.73739  3.18146  2.89801
2.37887  2.77519  2.98518  4.58477  3.61503
          0.00769  5.26778  5.99012  0.61958  0.77255  0.48247  0.96040
```

```
     72   2.54506  4.84554  2.79910  2.30636  4.18189  3.03110  3.72324
3.35145  1.54472  3.59605  4.53205  2.87908  3.89431  2.75896  3.06547
2.34155  3.09543  3.76676  5.10814  3.82781    335 k x - T
          2.68629  4.42170  2.77508  2.73128  3.46348  2.40508  3.72471
3.29340  2.67755  2.69335  4.24713  2.90356  2.73721  3.18150  2.89811
2.37895  2.77540  2.98529  4.58466  3.61483
          0.25041  2.23109  2.17060  1.29697  0.31932  0.48576  0.95510
     73   2.71328  5.09771  3.22353  2.65346  3.17483  2.68369  3.51884
3.48847  2.49944  2.98800  4.05869  3.00861  3.01965  3.14298  3.08773
2.72012  2.84073  3.23617  4.47379  1.99982    344 y x - T
          2.68631  4.42191  2.77513  2.73118  3.46367  2.40519  3.72502
3.29344  2.67719  2.69350  4.24696  2.90328  2.73742  3.18159  2.89798
2.37900  2.77523  2.98514  4.58490  3.61508
          0.37442  1.51714  2.37543  0.31423  1.31064  0.77665  0.61608
     74   2.42543  3.82155  4.21300  3.66863  3.23663  3.80316  4.12240
1.64072  3.58457  1.95253  3.27922  3.55257  4.01978  3.86563  3.55054
2.90041  2.42977  2.41820  4.75670  3.39955    349 i x - S
          2.68618  4.42225  2.77519  2.73123  3.46354  2.40513  3.72494
3.29354  2.67741  2.69355  4.24690  2.90347  2.73739  3.18146  2.89801
2.37887  2.77519  2.98518  4.58477  3.61503
          0.04059  5.06778  3.39683  0.61958  0.77255  0.96155  0.48175
     75   2.57931  5.16938  1.83485  2.30511  4.39922  2.15180  3.74024
4.15904  2.38232  3.63640  4.43708  2.85396  3.88744  2.88089  2.91676
2.59863  2.89396  3.67298  5.83054  4.24909    350 d x - T
          2.68596  4.42272  2.77503  2.73166  3.46400  2.40546  3.72536
3.29401  2.67649  2.69401  4.24736  2.90218  2.73622  3.18188  2.89774
2.37932  2.77548  2.98539  4.58524  3.61550
          0.51553  1.27957  2.08219  1.31854  0.31133  0.51751  0.90635
     76   2.73997  4.18836  4.25270  3.99408  3.29503  3.88942  4.30929
2.15029  3.34365  1.17269  3.24845  3.91861  3.81916  3.93994  3.61800
3.25589  3.08412  2.29177  4.98492  3.31960    365 l x - S
          2.68618  4.42225  2.77519  2.73123  3.46354  2.40513  3.72494
3.29354  2.67741  2.69355  4.24690  2.90347  2.73739  3.18146  2.89801
2.37887  2.77519  2.98518  4.58477  3.61503
          0.00941  5.06658  5.78893  0.61958  0.77255  0.52966  0.88868
     77   2.71685  5.42537  3.13986  2.39883  4.43874  3.37310  3.89272
3.45781  2.09592  3.35224  4.00913  3.06967  3.71339  2.63201  1.62953
2.59973  2.89486  3.27283  5.86844  4.40692    366 r x - E
          2.68618  4.42225  2.77519  2.73123  3.46354  2.40513  3.72494
3.29354  2.67741  2.69355  4.24690  2.90347  2.73739  3.18146  2.89801
2.37887  2.77519  2.98518  4.58477  3.61503
          0.01133  5.18302  5.17503  0.61958  0.77255  0.73386  0.65403
     78   2.51897  3.87156  4.13682  2.06821  3.40664  3.91688  4.11197
2.57623  3.57120  2.08760  3.47604  3.95891  3.81209  3.88598  3.62573
3.08822  2.71021  1.70696  4.39263  3.39669    367 v x - E
          2.68618  4.42225  2.77519  2.73123  3.46354  2.40513  3.72494
3.29354  2.67741  2.69355  4.24690  2.90347  2.73739  3.18146  2.89801
2.37887  2.77519  2.98518  4.58477  3.61503
          0.04243  5.18338  3.32604  0.61958  0.77255  0.75088  0.63857
     79   2.57757  2.99846  3.53367  2.63302  3.94765  3.43100  3.36866
2.88411  2.81331  1.86331  3.54293  3.09715  3.97462  3.16882  3.10743
2.55544  2.92414  2.52018  5.39974  3.91368    368 l x - E
          2.68617  4.42232  2.77518  2.73123  3.46361  2.40520  3.72495
3.29326  2.67743  2.69362  4.24697  2.90324  2.73745  3.18149  2.89803
2.37887  2.77522  2.98509  4.58484  3.61510
          0.28165  1.85545  2.41825  0.14135  2.02635  0.75869  0.63164
```

```
     80   2.14905  3.54365  3.79666  3.23417  3.29085  3.36012  4.22663
2.93333  3.15195  1.57979  3.66449  3.34886  4.22769  3.57592  3.25100
2.29948  3.00857  2.55603  4.42945  3.77932    370 l x - E
          2.68618  4.42225  2.77519  2.73123  3.46354  2.40513  3.72494
3.29354  2.67741  2.69355  4.24690  2.90347  2.73739  3.18146  2.89801
2.37887  2.77519  2.98518  4.58477  3.61503
          0.00933  5.07528  5.79762  0.61958  0.77255  0.84892  0.55840
     81   2.66572  3.96166  4.04876  2.94269  3.15938  3.35246  4.09211
1.56025  3.22616  2.11021  3.46540  3.94178  4.42456  3.78117  3.56680
3.15382  2.86970  2.12518  3.79948  3.85389    371 i x - E
          2.68622  4.42234  2.77528  2.73130  3.46359  2.40522  3.72490
3.29354  2.67747  2.69334  4.24699  2.90351  2.73727  3.18148  2.89802
2.37883  2.77521  2.98524  4.58368  3.61499
          0.11780  2.57021  3.36374  0.91950  0.50869  0.30755  1.32895
     82   2.96542  4.20817  4.26395  3.90151  3.11448  4.16813  4.11568
1.51638  3.41220  1.91005  3.42470  4.24690  4.30105  3.77340  3.69271
3.09434  2.67499  2.21309  4.88108  2.48767    375 i x - E
          2.68618  4.42225  2.77519  2.73123  3.46354  2.40513  3.72494
3.29354  2.67741  2.69355  4.24690  2.90347  2.73739  3.18146  2.89801
2.37887  2.77519  2.98518  4.58477  3.61503
          0.00795  5.23412  5.95647  0.61958  0.77255  0.60557  0.78914
     83   2.85094  5.46363  3.02242  2.53166  4.79017  1.26305  3.77504
4.26365  2.47535  3.61211  4.51118  3.06380  3.96873  3.04000  3.17160
2.40568  2.55721  3.78564  5.90842  4.50773    376 g x - T
          2.68635  4.42242  2.77536  2.73118  3.46371  2.40524  3.72511
3.29336  2.67717  2.69357  4.24693  2.90348  2.73756  3.18130  2.89798
2.37885  2.77509  2.98476  4.58494  3.61520
          0.16222  2.15299  3.39305  0.98880  0.46525  0.48904  0.94988
     84   2.65300  5.43727  2.86796  2.67117  4.76053  1.21285  3.95248
4.23113  2.60323  3.57748  4.48473  2.97634  3.89708  2.66834  3.06245
2.63047  3.05741  3.70929  5.50110  4.48665    380 g x - S
          2.68627  4.42234  2.77529  2.73114  3.46325  2.40496  3.72504
3.29331  2.67750  2.69364  4.24699  2.90356  2.73749  3.18127  2.89810
2.37883  2.77529  2.98504  4.58486  3.61512
          0.04876  3.55512  3.96242  1.51169  0.24915  0.40226  1.10505
     85   2.61801  5.16173  2.28319  2.48110  4.58742  3.45223  3.19085
3.62551  2.32998  3.55501  4.04516  2.96338  3.62572  2.85197  2.93492
2.53917  2.70852  2.02962  5.89812  4.27860    389 v x - S
          2.68618  4.42225  2.77519  2.73123  3.46354  2.40513  3.72494
3.29354  2.67741  2.69355  4.24690  2.90347  2.73739  3.18146  2.89801
2.37887  2.77519  2.98518  4.58477  3.61503
          0.00780  5.25341  5.97575  0.61958  0.77255  0.54204  0.87123
     86   1.89240  5.49193  2.85590  2.25764  4.83396  3.30148  3.87773
4.19532  2.35271  3.42522  4.22319  3.04424  2.91591  2.81548  2.90200
2.00307  2.75134  3.46441  5.92644  4.51733    390 a x - C
          2.68618  4.42225  2.77519  2.73123  3.46354  2.40513  3.72494
3.29354  2.67741  2.69355  4.24690  2.90347  2.73739  3.18146  2.89801
2.37887  2.77519  2.98518  4.58477  3.61503
          0.00780  5.25341  5.97575  0.61958  0.77255  0.54204  0.87123
     87   1.93022  5.23019  3.11371  2.55540  3.79550  3.68326  4.02259
2.95713  2.52344  2.62400  3.89386  3.23654  3.43195  2.80246  2.36953
2.82048  2.69209  3.11699  5.72623  3.31845    391 a x - H
          2.68620  4.42227  2.77522  2.73125  3.46356  2.40515  3.72497
3.29356  2.67743  2.69357  4.24664  2.90349  2.73742  3.18148  2.89790
2.37887  2.77517  2.98520  4.58456  3.61492
          0.10266  3.05778  2.98431  0.30229  1.34370  0.52422  0.89653
```

88 2.71540 5.46969 2.83841 2.12757 4.73460 3.27893 3.46102
3.72355 2.19133 3.27700 3.94773 2.92180 3.76710 2.76975 1.80548
2.61654 2.87645 3.69691 4.95764 4.14094 393 r x - H
2.68618 4.42225 2.77519 2.73123 3.46354 2.40513 3.72494
3.29354 2.67741 2.69355 4.24690 2.90347 2.73739 3.18146 2.89801
2.37887 2.77519 2.98518 4.58477 3.61503
0.01082 5.20737 5.24305 0.61958 0.77255 0.50412 0.92644
89 2.68118 5.47841 1.76556 2.26461 4.59809 3.69122 3.33766
3.43688 2.36553 3.02916 4.44244 3.09814 3.37607 2.61551 2.82502
2.58290 3.04591 3.29628 5.91471 3.85950 394 d x - H
2.68618 4.42225 2.77519 2.73123 3.46354 2.40513 3.72494
3.29354 2.67741 2.69355 4.24690 2.90347 2.73739 3.18146 2.89801
2.37887 2.77519 2.98518 4.58477 3.61503
0.00791 5.23953 5.96188 0.61958 0.77255 0.58826 0.81033
90 2.86856 5.15835 2.89422 2.01612 4.78277 3.76277 3.82452
3.35561 2.42905 3.02995 3.93966 2.81366 4.15585 1.53439 2.99124
2.87007 2.98806 3.20123 5.89650 4.36287 395 q x - H
2.68618 4.42225 2.77519 2.73123 3.46354 2.40513 3.72494
3.29354 2.67741 2.69355 4.24690 2.90347 2.73739 3.18146 2.89801
2.37887 2.77519 2.98518 4.58477 3.61503
0.01071 5.23953 5.23154 0.61958 0.77255 0.57357 0.82899
91 2.59930 4.65853 3.64327 3.03774 3.36477 3.41208 3.74909
2.60199 2.78972 1.65166 3.56186 3.53772 3.67287 3.09236 2.68545
2.98961 2.93270 2.61775 3.98882 3.43567 396 l x - H
2.68618 4.42225 2.77519 2.73123 3.46354 2.40513 3.72494
3.29354 2.67741 2.69355 4.24690 2.90347 2.73739 3.18146 2.89801
2.37887 2.77519 2.98518 4.58477 3.61503
0.00791 5.23931 5.96165 0.61958 0.77255 0.58900 0.80941
92 2.49129 5.48713 2.91793 2.08954 3.98871 3.28015 3.87375
4.26714 2.18900 3.41431 4.28935 2.88190 3.94204 2.75275 2.66946
1.80133 2.78179 3.87657 5.92114 3.61981 397 s x - H
2.68618 4.42225 2.77519 2.73123 3.46354 2.40513 3.72494
3.29354 2.67741 2.69355 4.24690 2.90347 2.73739 3.18146 2.89801
2.37887 2.77519 2.98518 4.58477 3.61503
0.00791 5.23931 5.96165 0.61958 0.77255 0.58900 0.80941
93 2.49484 5.25432 2.81668 2.31090 3.52751 3.59028 3.68720
3.80224 2.06763 3.02730 4.29709 3.09576 3.95477 2.64056 2.58843
2.83781 2.89536 2.15666 5.01065 4.31659 398 k x - H
2.68618 4.42225 2.77519 2.73123 3.46354 2.40513 3.72494
3.29354 2.67741 2.69355 4.24690 2.90347 2.73739 3.18146 2.89801
2.37887 2.77519 2.98518 4.58477 3.61503
0.00791 5.23931 5.96165 0.61958 0.77255 0.58900 0.80941
94 2.83686 4.41846 4.55375 4.22118 3.12954 4.19465 4.52455
2.09966 3.94369 1.01946 3.23000 4.13299 4.55902 4.09670 3.85162
3.37754 3.19994 2.15972 4.80229 3.52649 399 l x - H
2.68630 4.42247 2.77518 2.73122 3.46363 2.40499 3.72508
3.29347 2.67738 2.69371 4.24611 2.90328 2.73761 3.18133 2.89820
2.37903 2.77521 2.98484 4.58499 3.61470
0.54335 2.24326 1.16126 1.28371 0.32436 0.58900 0.80941
95 2.59485 5.32501 1.88850 2.15653 4.66653 3.56561 3.58417
4.05170 2.15696 3.41123 4.13570 2.89580 3.91206 2.77411 2.37095
2.61362 2.91893 3.51056 5.32650 4.35106 406 d x - H
2.68629 4.42224 2.77530 2.73095 3.46352 2.40522 3.72505
3.29365 2.67743 2.69356 4.24701 2.90325 2.73744 3.18139 2.89809
2.37894 2.77510 2.98526 4.58488 3.61450
0.20376 1.73500 4.83509 0.60328 0.79190 0.25345 1.49664

96 2.41665 3.86004 3.04609 2.50155 4.55434 3.23721 3.66708
3.67706 2.04646 3.25679 3.92257 1.98952 4.15437 2.71968 2.70428
2.52373 2.97953 3.45854 5.89227 3.83551 409 n x - H
2.68618 4.42225 2.77519 2.73123 3.46354 2.40513 3.72494
3.29354 2.67741 2.69355 4.24690 2.90347 2.73739 3.18146 2.89801
2.37887 2.77519 2.98518 4.58477 3.61503
0.01058 5.23536 5.25838 0.61958 0.77255 0.50382 0.92689
97 2.87490 5.21934 2.92006 2.34018 4.36546 1.74703 3.45551
4.32245 2.21558 3.63070 4.36115 2.60098 4.07474 2.88562 2.50508
2.66756 2.64120 3.82291 5.92759 4.28137 410 g x - T
2.68623 4.42255 2.77538 2.73108 3.46358 2.40478 3.72515
3.29359 2.67723 2.69367 4.24720 2.90343 2.73729 3.18125 2.89808
2.37899 2.77528 2.98526 4.58507 3.61509
0.34954 1.23418 5.54302 0.68735 0.69898 0.46018 0.99743
98 2.15018 3.04747 4.63094 3.88079 3.58782 3.81369 4.47623
2.25267 3.48512 2.29052 3.60427 4.08116 2.60779 3.89782 4.03623
2.92247 2.76366 1.62048 5.10272 3.47033 414 v x - C
2.68618 4.42225 2.77519 2.73123 3.46354 2.40513 3.72494
3.29354 2.67741 2.69355 4.24690 2.90347 2.73739 3.18146 2.89801
2.37887 2.77519 2.98518 4.58477 3.61503
0.00771 5.26529 5.98764 0.61958 0.77255 0.47690 0.96945
99 2.75808 5.50969 1.49185 2.34681 4.85539 3.55935 2.87836
4.17399 2.43522 3.66461 4.55024 2.72128 3.21768 2.56313 2.85017
2.74657 3.20027 3.85136 5.94192 4.53045 415 d x - S
2.68618 4.42225 2.77519 2.73123 3.46354 2.40513 3.72494
3.29354 2.67741 2.69355 4.24690 2.90347 2.73739 3.18146 2.89801
2.37887 2.77519 2.98518 4.58477 3.61503
0.00768 5.26922 5.99156 0.61958 0.77255 0.48576 0.95510
100 2.98901 4.17760 5.01501 4.40081 3.13133 4.22902 4.56299
1.07375 4.18798 2.11903 3.27921 4.09632 4.59216 4.32091 4.19936
3.17997 3.29099 1.83923 4.81311 3.46951 416 i x - E
2.68618 4.42225 2.77519 2.73123 3.46354 2.40513 3.72494
3.29354 2.67741 2.69355 4.24690 2.90347 2.73739 3.18146 2.89801
2.37887 2.77519 2.98518 4.58477 3.61503
0.00768 5.26922 5.99156 0.61958 0.77255 0.48576 0.95510
101 2.95772 4.24917 5.01834 4.40514 3.02551 4.23900 4.57382
2.18617 3.97756 1.53585 3.52657 4.39677 4.32002 4.32858 4.20821
3.55043 2.85012 1.25850 5.07044 3.34263 417 v x - E
2.68618 4.42225 2.77519 2.73123 3.46354 2.40513 3.72494
3.29354 2.67741 2.69355 4.24690 2.90347 2.73739 3.18146 2.89801
2.37887 2.77519 2.98518 4.58477 3.61503
0.00768 5.26922 5.99156 0.61958 0.77255 0.48576 0.95510
102 3.09061 4.36195 5.02772 4.41773 3.13178 4.26647 4.60228
1.71799 4.21207 2.30760 3.51067 3.66817 4.62769 4.35071 4.23316
3.40145 3.32345 1.06828 5.10175 2.99381 418 v x - E
2.68618 4.42225 2.77519 2.73123 3.46354 2.40513 3.72494
3.29354 2.67741 2.69355 4.24690 2.90347 2.73739 3.18146 2.89801
2.37887 2.77519 2.98518 4.58477 3.61503
0.01255 5.26922 4.91620 0.61958 0.77255 0.48576 0.95510
103 2.21094 4.35641 3.69992 2.90138 4.04588 1.54966 4.17680
3.26738 3.02634 2.86913 3.27772 3.29321 4.32550 3.43270 3.51209
2.63027 2.22518 2.82152 5.44563 4.18322 419 g x - E
2.68618 4.42225 2.77519 2.73123 3.46354 2.40513 3.72494
3.29354 2.67741 2.69355 4.24690 2.90347 2.73739 3.18146 2.89801
2.37887 2.77519 2.98518 4.58477 3.61503
0.00771 5.26438 5.98672 0.61958 0.77255 0.50342 0.92750

```
    104   2.90167  4.87980  3.59539  2.82281  4.23058  3.90090  4.15175
3.47303  3.01687  3.14712  3.95715  3.06369  4.30237  3.27633  3.44130
2.88923  0.87277  3.29225  5.58254  4.29630    420 t x - S
          2.68618  4.42225  2.77519  2.73123  3.46354  2.40513  3.72494
3.29354  2.67741  2.69355  4.24690  2.90347  2.73739  3.18146  2.89801
2.37887  2.77519  2.98518  4.58477  3.61503
          0.00771  5.26438  5.98672  0.61958  0.77255  0.50342  0.92750
    105   2.91109  4.82703  3.57907  2.96701  4.09276  3.90373  3.33470
3.46564  2.98663  3.24268  3.91488  3.50264  1.01667  3.24046  3.12407
3.07165  2.79331  3.31102  5.54818  3.81488    421 p x - H
          2.68595  4.42236  2.77512  2.73134  3.46291  2.40511  3.72505
3.29365  2.67751  2.69365  4.24700  2.90357  2.73750  3.18157  2.89811
2.37897  2.77512  2.98529  4.58447  3.61429
          0.05687  3.37758  3.85605  1.47544  0.25965  0.47488  0.97276
    106   2.70008  5.47713  2.90814  1.94946  4.48587  1.36126  3.89802
4.29008  2.58191  3.71578  4.39640  3.19833  3.63352  2.97463  3.10178
2.60754  3.13583  3.74364  5.91634  4.51156    431 g x - H
          2.68618  4.42225  2.77519  2.73123  3.46354  2.40513  3.72494
3.29354  2.67741  2.69355  4.24690  2.90347  2.73739  3.18146  2.89801
2.37887  2.77519  2.98518  4.58477  3.61503
          0.00782  5.25049  5.97284  0.61958  0.77255  0.55199  0.85756
    107   2.76271  5.20819  3.23372  2.83229  3.93828  3.51695  4.02827
3.39121  2.30773  3.06191  3.61352  3.31301  4.21521  3.01882  1.25374
2.74368  2.97666  3.20395  5.70852  4.23025    432 r x - H
          2.68618  4.42225  2.77519  2.73123  3.46354  2.40513  3.72494
3.29354  2.67741  2.69355  4.24690  2.90347  2.73739  3.18146  2.89801
2.37887  2.77519  2.98518  4.58477  3.61503
          0.00782  5.25049  5.97284  0.61958  0.77255  0.55199  0.85756
    108   2.83314  4.17716  3.67759  4.36648  2.91251  4.21554  4.54744
2.28155  4.15907  0.98487  3.41634  4.36695  4.57852  4.29584  4.17895
3.52564  3.19311  2.15705  3.79480  3.74840    433 l x - H
          2.68618  4.42225  2.77519  2.73123  3.46354  2.40513  3.72494
3.29354  2.67741  2.69355  4.24690  2.90347  2.73739  3.18146  2.89801
2.37887  2.77519  2.98518  4.58477  3.61503
          0.00782  5.25049  5.97284  0.61958  0.77255  0.55199  0.85756
    109   2.51130  4.54610  1.98626  2.95304  3.21569  3.63647  3.52060
2.71409  3.01188  2.10420  3.46546  3.30847  4.22743  3.07721  3.26436
2.90684  3.08890  2.50175  4.93917  3.52859    434 d x - H
          2.68618  4.42225  2.77519  2.73123  3.46354  2.40513  3.72494
3.29354  2.67741  2.69355  4.24690  2.90347  2.73739  3.18146  2.89801
2.37887  2.77519  2.98518  4.58477  3.61503
          0.02315  5.25049  4.03783  0.61958  0.77255  0.55199  0.85756
    110   2.71216  5.45796  1.41191  2.54843  4.39776  3.46749  3.45045
3.68647  2.44187  3.27294  4.50226  2.98000  4.15274  2.95506  2.90676
2.39603  2.92389  3.59518  5.89898  3.77435    435 d x - H
          2.68611  4.42231  2.77523  2.73125  3.46360  2.40514  3.72501
3.29356  2.67747  2.69350  4.24658  2.90350  2.73727  3.18147  2.89807
2.37885  2.77516  2.98520  4.58483  3.61509
          0.23236  2.05320  2.53815  0.15440  1.94440  0.48297  0.95959
    111   2.96507  4.23630  3.90748  3.40532  2.96265  3.98780  2.55167
2.17229  3.85756  1.43311  2.81712  3.55517  4.11087  4.05510  3.99330
3.15638  3.13678  2.33211  4.84993  3.59442    437 l x - H
          2.68618  4.42225  2.77519  2.73123  3.46354  2.40513  3.72494
3.29354  2.67741  2.69355  4.24690  2.90347  2.73739  3.18146  2.89801
2.37887  2.77519  2.98518  4.58477  3.61503
          0.00840  5.17908  5.90143  0.61958  0.77255  0.76196  0.62876
```

```
    112   2.83034  3.61154  4.72071  4.14596  2.75332  3.75557  4.49438
2.19014  3.33062  1.53772  3.15669  4.06100  4.52776  4.23723  3.98932
3.26941  2.81904  1.65023  4.79889  3.61403    438 l x - H
          2.68620  4.42272  2.77566  2.73138  3.46358  2.40540  3.72332
3.29350  2.67739  2.69375  4.24645  2.90323  2.73775  3.18167  2.89688
2.37910  2.77546  2.98503  4.58396  3.61443
          0.62184  0.86715  3.14897  1.36354  0.29538  0.69499  0.69131
    113   2.68263  5.03497  2.90442  2.08448  4.21733  3.64625  3.67450
3.98376  2.31100  3.05018  4.47491  2.48048  3.84091  2.60151  2.82686
1.86554  2.90523  3.60092  4.39958  4.16158    446 s x - H
          2.68620  4.42227  2.77521  2.73125  3.46329  2.40514  3.72487
3.29356  2.67742  2.69351  4.24691  2.90341  2.73741  3.18148  2.89802
2.37888  2.77521  2.98520  4.58479  3.61505
          0.17276  3.14492  2.15769  0.33540  1.25545  0.47971  0.96486
    114   2.69366  5.39851  2.76468  2.48018  4.59281  3.58784  3.89467
3.84494  2.35499  3.51829  4.10154  2.83105  3.82659  2.68188  2.28547
2.64694  1.70549  3.27656  5.11685  4.31940    448 t x - C
          2.68618  4.42225  2.77519  2.73123  3.46354  2.40513  3.72494
3.29354  2.67741  2.69355  4.24690  2.90347  2.73739  3.18146  2.89801
2.37887  2.77519  2.98518  4.58477  3.61503
          0.01442  5.11649  4.78932  0.61958  0.77255  0.76458  0.62648
    115   2.84441  5.42647  3.11408  2.27714  4.29607  1.81922  3.60732
4.05905  2.18309  3.63711  4.46940  2.75249  3.76823  2.73022  2.42693
2.70016  3.02417  3.09828  4.97271  4.45865    449 g x - T
          2.68618  4.42225  2.77519  2.73123  3.46354  2.40513  3.72494
3.29354  2.67741  2.69355  4.24690  2.90347  2.73739  3.18146  2.89801
2.37887  2.77519  2.98518  4.58477  3.61503
          0.00874  5.14036  5.86271  0.61958  0.77255  0.82912  0.57347
    116   2.62581  5.12213  3.00508  2.37330  4.18265  3.14701  3.25793
3.77098  1.70606  2.84053  4.37073  3.01048  3.62774  2.91596  2.57056
2.54299  2.90910  3.40865  5.85187  3.47404    450 k x - S
          2.68623  4.42195  2.77524  2.73125  3.46322  2.40516  3.72499
3.29359  2.67746  2.69347  4.24695  2.90348  2.73744  3.18151  2.89806
2.37877  2.77515  2.98520  4.58482  3.61508
          0.33988  2.81619  1.47706  0.64665  0.74192  0.82401  0.57744
    117   2.85530  4.22566  4.83444  4.22481  2.43183  4.10782  4.43678
2.07935  3.79040  1.13490  2.96813  4.24766  4.46058  3.92654  4.05667
3.41837  3.11819  2.34511  4.93161  3.65985    453 l x - S
          2.68618  4.42225  2.77519  2.73123  3.46354  2.40513  3.72494
3.29354  2.67741  2.69355  4.24690  2.90347  2.73739  3.18146  2.89801
2.37887  2.77519  2.98518  4.58477  3.61503
          0.01417  4.89457  5.02254  0.61958  0.77255  1.24179  0.34090
    118   2.83364  4.25863  2.75028  2.51864  4.60860  3.39382  3.67049
3.90203  2.38303  3.39538  4.33377  1.84543  3.46034  2.66763  2.83247
2.30749  2.75820  2.85472  5.73382  4.33743    454 n x - H
          2.68620  4.42222  2.77535  2.73123  3.46325  2.40511  3.72510
3.29331  2.67735  2.69356  4.24706  2.90314  2.73753  3.18151  2.89790
2.37897  2.77532  2.98512  4.58493  3.61513
          0.18210  1.81497  5.61664  0.95283  0.48719  0.20446  1.68789
    119   2.87200  3.80763  4.98585  4.21447  2.57148  4.21451  4.42158
2.49476  4.10465  1.00763  3.27820  4.25811  4.42992  4.29896  3.84107
3.18914  2.84930  2.43150  4.50446  3.34056    459 l x - C
          2.68618  4.42225  2.77519  2.73123  3.46354  2.40513  3.72494
3.29354  2.67741  2.69355  4.24690  2.90347  2.73739  3.18146  2.89801
2.37887  2.77519  2.98518  4.58477  3.61503
          0.00771  5.26555  5.98789  0.61958  0.77255  0.47746  0.96853
```

```
    120   2.89373  5.50707  2.79554  2.37513  4.48172  3.05277  3.60516
4.09642  2.00148  3.60342  4.30560  2.85915  4.03695  2.36515  2.73526
1.64775  3.06875  3.89784  5.93961  4.37322    460 s x - T
          2.68618  4.42225  2.77519  2.73123  3.46354  2.40513  3.72494
3.29354  2.67741  2.69355  4.24690  2.90347  2.73739  3.18146  2.89801
2.37887  2.77519  2.98518  4.58477  3.61503
          0.00768  5.26922  5.99156  0.61958  0.77255  0.48576  0.95510
    121   2.57739  5.50736  2.75854  2.43880  4.21348  3.00579  2.88422
4.33902  2.34440  3.59931  4.54793  2.42720  4.08317  1.89872  2.66872
2.41610  3.08986  3.71714  5.93983  4.11723    461 q x - T
          2.68618  4.42225  2.77519  2.73123  3.46354  2.40513  3.72494
3.29354  2.67741  2.69355  4.24690  2.90347  2.73739  3.18146  2.89801
2.37887  2.77519  2.98518  4.58477  3.61503
          0.00768  5.26922  5.99156  0.61958  0.77255  0.48576  0.95510
    122   2.95678  3.43278  4.96575  4.35371  3.21856  4.03841  4.54521
2.34435  3.90595  1.53140  3.46503  4.36071  4.02220  4.10447  3.92805
3.11989  2.94395  1.30567  5.05333  3.77087    462 v x - E
          2.68604  4.42229  2.77521  2.73127  3.46358  2.40517  3.72498
3.29354  2.67739  2.69353  4.24694  2.90351  2.73741  3.18133  2.89805
2.37887  2.77523  2.98512  4.58481  3.61507
          0.05987  3.15545  4.16737  0.66189  0.72541  0.48576  0.95510
    123   2.83957  5.21217  3.11724  2.36027  4.84234  2.80509  3.95153
4.12939  1.98341  3.60085  4.53926  2.94291  4.06259  2.79855  1.68570
2.17404  2.91354  3.88838  5.93144  4.52140    465 r x - E
          2.68618  4.42225  2.77519  2.73123  3.46354  2.40513  3.72494
3.29354  2.67741  2.69355  4.24690  2.90347  2.73739  3.18146  2.89801
2.37887  2.77519  2.98518  4.58477  3.61503
          0.00778  5.25614  5.97848  0.61958  0.77255  0.51661  0.90768
    124   2.97396  3.95570  5.00342  4.38842  1.68816  4.06846  4.25392
2.55953  4.17433  1.72046  3.10118  4.37422  4.57714  4.30643  4.18420
3.15303  2.84492  2.35479  4.09935  2.17346    466 f x - E
          2.68612  4.42229  2.77519  2.73127  3.46358  2.40510  3.72498
3.29358  2.67744  2.69352  4.24693  2.90350  2.73743  3.18142  2.89783
2.37890  2.77517  2.98522  4.58481  3.61507
          0.04458  3.19231  5.98120  0.66092  0.72645  0.46311  0.99243
    125   3.22269  4.60239  5.21138  4.60336  2.94652  4.44377  4.78506
2.05009  4.23625  1.07769  3.00374  4.60457  4.78431  4.51739  4.29911
3.76160  3.02536  1.64968  5.24573  4.08478    469 l x - E
          2.68618  4.42225  2.77519  2.73123  3.46354  2.40513  3.72494
3.29354  2.67741  2.69355  4.24690  2.90347  2.73739  3.18146  2.89801
2.37887  2.77519  2.98518  4.58477  3.61503
          0.00768  5.26922  5.99156  0.61958  0.77255  0.48576  0.95510
    126   3.35322  5.18977  6.17863  5.74376  4.70649  5.72281  6.61857
1.22481  5.72294  3.10503  4.09169  5.90305  5.92906  6.02185  5.91838
5.18632  4.28140  0.60249  6.89992  5.64342    470 V x - E
          2.68618  4.42225  2.77519  2.73123  3.46354  2.40513  3.72494
3.29354  2.67741  2.69355  4.24690  2.90347  2.73739  3.18146  2.89801
2.37887  2.77519  2.98518  4.58477  3.61503
          0.00768  5.26922  5.99156  0.61958  0.77255  0.48576  0.95510
    127   3.46786  4.86071  5.51421  4.90986  2.41534  4.76553  5.10283
1.64457  4.71439  1.01644  3.02423  4.93209  5.05486  4.78369  4.70682
4.09417  3.56743  1.99024  5.47616  4.10657    471 l x - E
          2.68618  4.42225  2.77519  2.73123  3.46354  2.40513  3.72494
3.29354  2.67741  2.69355  4.24690  2.90347  2.73739  3.18146  2.89801
2.37887  2.77519  2.98518  4.58477  3.61503
          0.00768  5.26922  5.99156  0.61958  0.77255  0.48576  0.95510
```

```
    128   4.28608  6.99512  0.24231  2.71477  6.31933  4.20295  4.96579
6.01675  4.27400  5.42309  6.42807  3.67781  4.93881  4.17963  5.06386
4.07735  4.63758  5.50856  7.44785  5.86395    472 D x - E
          2.68618  4.42225  2.77519  2.73123  3.46354  2.40513  3.72494
3.29354  2.67741  2.69355  4.24690  2.90347  2.73739  3.18146  2.89801
2.37887  2.77519  2.98518  4.58477  3.61503
          0.00768  5.26922  5.99156  0.61958  0.77255  0.48576  0.95510
    129   4.43206  6.81754  3.16383  0.18611  6.29222  4.39502  5.16853
6.01780  4.36852  5.44376  6.44537  3.95948  5.10694  4.39667  4.99437
4.27722  4.78170  5.54275  7.30519  5.95206    473 E x - T
          2.68618  4.42225  2.77519  2.73123  3.46354  2.40513  3.72494
3.29354  2.67741  2.69355  4.24690  2.90347  2.73739  3.18146  2.89801
2.37887  2.77519  2.98518  4.58477  3.61503
          0.00768  5.26922  5.99156  0.61958  0.77255  0.48576  0.95510
    130   1.05508  3.78906  4.34547  3.01781  3.27913  3.64209  4.40669
2.35810  3.66881  2.61401  3.67837  4.03286  4.48704  3.39758  3.89987
3.28797  3.21110  2.43203  5.17197  3.97222    474 a x - H
          2.68618  4.42225  2.77519  2.73123  3.46354  2.40513  3.72494
3.29354  2.67741  2.69355  4.24690  2.90347  2.73739  3.18146  2.89801
2.37887  2.77519  2.98518  4.58477  3.61503
          0.00768  5.26922  5.99156  0.61958  0.77255  0.48576  0.95510
    131   3.92449  6.61716  0.82272  2.90797  5.79756  4.05913  1.27709
5.46561  3.74442  4.90436  5.79380  3.50496  4.72678  3.12249  4.37023
3.76306  4.09481  5.00263  6.99090  5.40605    475 d x - H
          2.68618  4.42225  2.77519  2.73123  3.46354  2.40513  3.72494
3.29354  2.67741  2.69355  4.24690  2.90347  2.73739  3.18146  2.89801
2.37887  2.77519  2.98518  4.58477  3.61503
          0.00768  5.26922  5.99156  0.61958  0.77255  0.48576  0.95510
    132   2.80368  3.42817  3.11463  2.15786  4.30629  2.22378  3.78850
3.94646  2.54953  2.77978  3.69640  3.16534  4.19071  2.93835  1.95540
2.91469  2.89039  3.31123  5.83105  4.05105    476 r x - H
          2.68618  4.42225  2.77519  2.73123  3.46354  2.40513  3.72494
3.29354  2.67741  2.69355  4.24690  2.90347  2.73739  3.18146  2.89801
2.37887  2.77519  2.98518  4.58477  3.61503
          0.00768  5.26922  5.99156  0.61958  0.77255  0.48576  0.95510
    133   2.97935  4.21714  5.00232  4.38786  2.61756  4.21929  3.86192
2.34686  4.17553  1.26294  2.20585  4.37757  4.58299  4.19650  4.18813
2.98606  3.17118  2.21109  5.04940  3.19138    477 l x - H
          2.68618  4.42225  2.77519  2.73123  3.46354  2.40513  3.72494
3.29354  2.67741  2.69355  4.24690  2.90347  2.73739  3.18146  2.89801
2.37887  2.77519  2.98518  4.58477  3.61503
          0.00768  5.26922  5.99156  0.61958  0.77255  0.48576  0.95510
    134   2.74843  4.94277  2.97998  2.90111  3.61158  2.89980  3.78794
2.86677  3.00973  1.46973  3.34399  3.34318  4.30623  3.01868  3.18895
2.42574  3.01779  2.98170  5.49462  4.14160    478 l x - H
          2.68618  4.42225  2.77519  2.73123  3.46354  2.40513  3.72494
3.29354  2.67741  2.69355  4.24690  2.90347  2.73739  3.18146  2.89801
2.37887  2.77519  2.98518  4.58477  3.61503
          0.00768  5.26922  5.99156  0.61958  0.77255  0.48576  0.95510
    135   2.86304  5.49933  1.80785  2.32086  4.47262  2.87918  3.86200
4.32473  2.55051  3.72113  4.54087  2.94179  4.00130  2.64369  3.14530
1.82236  2.97973  3.15751  5.93399  3.96318    479 d x - H
          2.68618  4.42225  2.77519  2.73123  3.46354  2.40513  3.72494
3.29354  2.67741  2.69355  4.24690  2.90347  2.73739  3.18146  2.89801
2.37887  2.77519  2.98518  4.58477  3.61503
          0.02655  5.26922  3.86089  0.61958  0.77255  0.48576  0.95510
```

```
    136   2.78802  5.43382  2.92495  2.25820  4.37272  3.57419  3.81454
3.92694  2.39822  2.99977  2.78006  2.83132  4.03130  1.81515  2.93389
2.60258  3.08532  3.39561  3.48748  4.25590    480 q x - T
          2.68618  4.42220  2.77529  2.73131  3.46332  2.40488  3.72395
3.29356  2.67747  2.69361  4.24678  2.90358  2.73754  3.18144  2.89810
2.37897  2.77532  2.98506  4.58491  3.61511
          0.34434  1.85685  2.00137  0.59470  0.80236  0.55199  0.85756
    137   2.72418  5.35910  2.59042  2.49381  4.67349  1.52563  3.90111
4.13841  2.50626  3.29592  4.13213  2.89639  4.10305  2.94919  3.13652
2.32252  2.98172  3.24074  5.81068  4.13251    483 g x - T
          2.68618  4.42225  2.77519  2.73123  3.46354  2.40513  3.72494
3.29354  2.67741  2.69355  4.24690  2.90347  2.73739  3.18146  2.89801
2.37887  2.77519  2.98518  4.58477  3.61503
          0.00902  5.10823  5.83058  0.61958  0.77255  0.29497  1.36473
    138   2.94940  4.67549  3.96626  3.43698  1.46939  3.98341  3.86894
3.07851  3.28628  2.75841  3.56430  3.84109  4.42206  2.94949  2.63197
2.95267  3.26381  2.90969  4.74255  2.03202    484 f x - T
          2.68618  4.42225  2.77519  2.73123  3.46354  2.40513  3.72494
3.29354  2.67741  2.69355  4.24690  2.90347  2.73739  3.18146  2.89801
2.37887  2.77519  2.98518  4.58477  3.61503
          0.00770  5.26627  5.98861  0.61958  0.77255  0.49657  0.93806
    139   2.85983  5.48207  3.11435  2.27759  4.54704  2.29891  3.90003
3.66035  2.40609  3.14990  4.37512  3.21797  3.39903  2.43358  2.39965
1.94528  3.19152  3.54792  5.92071  3.83315    485 s x - H
          2.68618  4.42225  2.77519  2.73123  3.46354  2.40513  3.72494
3.29354  2.67741  2.69355  4.24690  2.90347  2.73739  3.18146  2.89801
2.37887  2.77519  2.98518  4.58477  3.61503
          0.00770  5.26627  5.98861  0.61958  0.77255  0.47906  0.96591
    140   2.57048  4.89726  1.66552  2.19494  4.67207  3.38066  3.85473
4.04003  2.54402  3.68652  4.54065  2.94901  2.48695  2.82738  2.68831
2.70991  3.17675  3.49745  5.29903  4.35098    486 d x - H
          2.68618  4.42225  2.77519  2.73123  3.46354  2.40513  3.72494
3.29354  2.67741  2.69355  4.24690  2.90347  2.73739  3.18146  2.89801
2.37887  2.77519  2.98518  4.58477  3.61503
          0.00768  5.26922  5.99156  0.61958  0.77255  0.48576  0.95510
    141   2.83915  4.90017  2.35251  2.59543  2.27010  3.44981  3.38052
2.94253  2.73589  2.84281  4.22593  3.18830  4.15595  2.47983  3.23512
2.72894  2.86280  2.91921  3.45617  4.08280    487 f x - H
          2.68618  4.42225  2.77519  2.73123  3.46354  2.40513  3.72494
3.29354  2.67741  2.69355  4.24690  2.90347  2.73739  3.18146  2.89801
2.37887  2.77519  2.98518  4.58477  3.61503
          0.00768  5.26922  5.99156  0.61958  0.77255  0.48576  0.95510
    142   2.63029  3.68956  5.01803  4.40240  3.23216  4.22024  4.31597
1.48723  4.18637  1.67836  3.08256  4.38392  4.58390  4.03299  4.19316
3.42368  3.01866  2.08690  3.75319  2.84100    488 i x - H
          2.68618  4.42225  2.77519  2.73123  3.46354  2.40513  3.72494
3.29354  2.67741  2.69355  4.24690  2.90347  2.73739  3.18146  2.89801
2.37887  2.77519  2.98518  4.58477  3.61503
          0.00768  5.26922  5.99156  0.61958  0.77255  0.48576  0.95510
    143   2.54204  5.50244  2.79682  2.04034  4.32747  3.55436  3.64927
3.44023  2.29755  3.33523  4.34190  2.03799  3.80675  2.75743  2.47763
2.57828  2.95910  3.79624  5.93618  3.71057    489 n x - H
          2.68618  4.42225  2.77519  2.73123  3.46354  2.40513  3.72494
3.29354  2.67741  2.69355  4.24690  2.90347  2.73739  3.18146  2.89801
2.37887  2.77519  2.98518  4.58477  3.61503
          0.00768  5.26922  5.99156  0.61958  0.77255  0.48576  0.95510
```

```
     144    2.51048  5.48417  3.07497  2.25809  4.45418  3.46434  3.37482
4.21154  2.21673  2.75278  4.12858  2.96104  4.16711  2.80466  1.85249
2.56073  2.68371  3.74359  5.14648  3.75486    490 r x - H
         2.68619  4.42226  2.77521  2.73124  3.46355  2.40514  3.72496
3.29330  2.67735  2.69356  4.24691  2.90348  2.73741  3.18148  2.89802
2.37888  2.77521  2.98520  4.58478  3.61504
         0.10568  4.28514  2.44740  0.75229  0.63731  0.48576  0.95510
     145    2.87236  3.47673  4.53854  4.25407  3.48531  3.80888  4.09798
1.63805  3.74037  1.97060  1.91352  4.18050  4.52335  3.94134  4.01234
3.13078  3.14318  2.02039  5.01179  3.75409    494 i x - H
         2.68618  4.42225  2.77519  2.73123  3.46354  2.40513  3.72494
3.29354  2.67741  2.69355  4.24690  2.90347  2.73739  3.18146  2.89801
2.37887  2.77519  2.98518  4.58477  3.61503
         0.00838  5.18146  5.90380  0.61958  0.77255  0.43745  1.03755
     146    2.50080  4.41866  3.94907  3.89131  3.14974  3.76424  2.17653
2.16721  3.50924  1.67056  2.83062  4.04941  4.51580  3.95568  3.62899
3.28003  3.26430  2.14849  5.10164  3.79129    495 l x - H
         2.68618  4.42225  2.77519  2.73123  3.46354  2.40513  3.72494
3.29354  2.67741  2.69355  4.24690  2.90347  2.73739  3.18146  2.89801
2.37887  2.77519  2.98518  4.58477  3.61503
         0.00785  5.24746  5.96981  0.61958  0.77255  0.56219  0.84387
     147    2.51632  5.48987  3.02283  2.24560  4.83223  3.00384  3.62902
3.76803  2.36671  3.62499  3.74587  1.94753  4.07318  2.94422  2.32903
2.28377  2.79593  3.87905  5.28330  4.51486    496 n x - H
         2.68618  4.42225  2.77519  2.73123  3.46354  2.40513  3.72494
3.29354  2.67741  2.69355  4.24690  2.90347  2.73739  3.18146  2.89801
2.37887  2.77519  2.98518  4.58477  3.61503
         0.00785  5.24746  5.96981  0.61958  0.77255  0.56219  0.84387
     148    2.41951  4.76493  2.97924  2.43416  4.21184  3.66672  2.93378
3.78072  2.54217  3.08597  3.78752  3.02632  4.16059  1.79346  2.36794
2.61896  2.85387  3.65382  5.89616  4.05673    497 q x - H
         2.68618  4.42225  2.77519  2.73123  3.46354  2.40513  3.72494
3.29354  2.67741  2.69355  4.24690  2.90347  2.73739  3.18146  2.89801
2.37887  2.77519  2.98518  4.58477  3.61503
         0.00785  5.24746  5.96981  0.61958  0.77255  0.56219  0.84387
     149    2.88377  4.08366  3.70525  4.28287  3.12363  3.81770  4.52272
1.55129  3.51586  1.45419  2.82254  4.32021  4.23317  4.24387  4.13922
3.50137  2.86669  2.25471  5.05066  3.87201    498 l x - S
         2.68618  4.42225  2.77519  2.73123  3.46354  2.40513  3.72494
3.29354  2.67741  2.69355  4.24690  2.90347  2.73739  3.18146  2.89801
2.37887  2.77519  2.98518  4.58477  3.61503
         0.31756  5.24746  1.32119  0.61958  0.77255  0.56219  0.84387
     150    2.53173  5.33379  2.73981  2.50589  4.36253  3.54391  3.57405
3.88536  2.12935  3.31036  4.37759  2.90571  2.09266  2.74449  2.40576
2.54288  3.08290  3.71806  5.77359  3.61701    499 p x - H
         2.68618  4.42225  2.77519  2.73123  3.46354  2.40513  3.72494
3.29354  2.67741  2.69355  4.24690  2.90347  2.73739  3.18146  2.89801
2.37887  2.77519  2.98518  4.58477  3.61503
         0.08886  4.94058  2.55262  0.61958  0.77255  1.19567  0.36025
     151    2.74844  5.30775  2.69643  2.26746  3.82636  3.49883  3.20650
3.71480  2.33671  3.49208  4.35106  2.98803  3.90038  1.81238  2.42783
2.64257  2.82659  3.53172  5.40242  4.08192    500 q x - H
         2.68618  4.42225  2.77519  2.73123  3.46354  2.40513  3.72494
3.29354  2.67741  2.69355  4.24690  2.90347  2.73739  3.18146  2.89801
2.37887  2.77519  2.98518  4.58477  3.61503
         0.01562  4.86326  4.85687  0.61958  0.77255  1.24363  0.34015
```

152 2.38344 4.72768 3.11914 2.86110 3.71669 3.60116 3.97745
1.82707 2.51664 2.46306 3.64607 3.19107 3.42718 3.08349 3.01760
2.88695 2.87154 2.69822 5.28436 3.85280 501 i x - H
2.68618 4.42225 2.77519 2.73123 3.46354 2.40513 3.72494
3.29354 2.67741 2.69355 4.24690 2.90347 2.73739 3.18146 2.89801
2.37887 2.77519 2.98518 4.58477 3.61503
0.04079 4.87067 3.43269 0.61958 0.77255 1.13820 0.38624
153 2.73204 3.75712 3.34417 2.68944 3.46554 3.04044 3.95178
3.15111 2.44392 2.69853 3.88627 3.12372 3.91983 3.16915 3.06460
2.64505 1.75247 2.93815 5.34359 3.27081 502 t x - H
2.68618 4.42225 2.77519 2.73123 3.46354 2.40513 3.72494
3.29354 2.67741 2.69355 4.24690 2.90347 2.73739 3.18146 2.89801
2.37887 2.77519 2.98518 4.58477 3.61503
0.01135 4.87994 5.60229 0.61958 0.77255 1.23430 0.34395
154 2.53379 2.09233 2.84928 2.43597 4.29073 3.02191 3.46710
3.73825 2.49446 3.05602 3.78640 2.87093 3.16967 2.92501 2.98700
2.64624 2.91388 3.31241 5.61847 4.25885 503 c x - C
2.68618 4.42225 2.77519 2.73123 3.46354 2.40513 3.72494
3.29354 2.67741 2.69355 4.24690 2.90347 2.73739 3.18146 2.89801
2.37887 2.77519 2.98518 4.58477 3.61503
0.03561 4.88877 3.59514 0.61958 0.77255 1.22789 0.34660
155 2.74828 4.93131 1.82352 2.47506 4.35211 3.24170 3.61226
3.59503 2.34300 2.85653 3.98954 2.83980 3.49069 2.51050 2.73743
2.67133 2.90040 3.62657 5.73238 4.14796 504 d x - C
2.68618 4.42225 2.77519 2.73123 3.46354 2.40513 3.72494
3.29354 2.67741 2.69355 4.24690 2.90347 2.73739 3.18146 2.89801
2.37887 2.77519 2.98518 4.58477 3.61503
0.07954 4.87269 2.67640 0.61958 0.77255 1.17925 0.36746
156 2.59914 4.28494 3.22197 2.68906 3.55069 1.98661 3.86044
3.13461 2.69855 2.60024 3.83258 3.28822 3.45137 2.80380 2.92703
2.69522 2.69297 2.72894 5.35820 3.81437 505 g x - S
2.68618 4.42225 2.77519 2.73123 3.46354 2.40513 3.72494
3.29354 2.67741 2.69355 4.24690 2.90347 2.73739 3.18146 2.89801
2.37887 2.77519 2.98518 4.58477 3.61503
0.01616 4.83250 4.82082 0.61958 0.77255 0.56851 0.83556
157 2.85228 5.07522 3.10460 2.46275 4.35498 3.52968 3.67644
4.21543 1.72601 3.15540 4.44326 2.54178 2.01340 2.75264 2.85234
2.67770 3.00627 3.78585 5.83828 4.27893 506 k x - T
2.68617 4.42240 2.77529 2.73135 3.46369 2.40525 3.72509
3.29356 2.67747 2.69364 4.24704 2.90350 2.73743 3.18157 2.89643
2.37900 2.77531 2.98529 4.58492 3.61518
0.72088 1.08672 1.73525 0.07801 2.58963 0.70747 0.67902
158 2.52463 5.20387 3.02467 2.36869 4.28344 2.99817 3.86136
3.61147 2.13838 2.00780 4.26203 3.04759 3.13042 2.73436 3.01383
2.64812 2.95072 3.17028 5.44360 3.92825 508 l x - T
2.68618 4.42225 2.77519 2.73123 3.46354 2.40513 3.72494
3.29354 2.67741 2.69355 4.24690 2.90347 2.73739 3.18146 2.89801
2.37887 2.77519 2.98518 4.58477 3.61503
0.02159 4.96401 4.24214 0.61958 0.77255 1.16421 0.37421
159 2.78060 5.36441 2.55048 2.35578 4.70835 3.51955 3.57784
4.09338 2.23203 3.66920 4.09406 2.68604 3.46322 1.78383 2.95733
2.28200 2.79618 3.58574 5.79774 4.23900 509 q x - T
2.68630 4.42232 2.77453 2.73119 3.46329 2.40571 3.72533
3.29304 2.67753 2.69260 4.24702 2.90332 2.73683 3.18123 2.89853
2.37924 2.77537 2.98592 4.58555 3.61452
0.48790 2.18812 1.29479 2.66014 0.07250 0.79436 0.60124

160 2.58693 4.53851 3.12071 2.59537 3.71585 3.37292 3.48018
3.03080 2.34822 2.95668 3.93209 2.95359 3.58232 3.00029 2.03367
2.56102 2.93698 2.72098 5.45802 3.52112 536 r x - S
2.68618 4.42225 2.77519 2.73123 3.46354 2.40513 3.72494
3.29354 2.67741 2.69355 4.24690 2.90347 2.73739 3.18146 2.89801
2.37887 2.77519 2.98518 4.58477 3.61503
0.01262 4.77484 5.49718 0.61958 0.77255 1.36673 0.29429
161 2.76531 4.94812 3.03702 2.46165 4.55282 3.45820 3.68332
3.44910 2.24015 3.35747 4.06142 2.88549 3.32351 1.72225 2.30253
2.76874 2.80706 3.55795 5.68290 4.29034 537 q x - E
2.68628 4.42240 2.77534 2.73138 3.46361 2.40518 3.72412
3.29355 2.67736 2.69336 4.24705 2.90355 2.73711 3.18098 2.89816
2.37902 2.77530 2.98513 4.58492 3.61518
0.29238 1.38858 5.50017 0.63333 0.75677 0.17216 1.84419
162 2.83783 3.89685 4.71544 4.11825 3.14451 4.17983 4.13710
2.01809 2.82548 2.31319 3.38486 4.05859 4.54670 4.03719 3.06538
2.83871 2.24966 1.56826 4.61389 3.81122 540 v x - E
2.68618 4.42225 2.77519 2.73123 3.46354 2.40513 3.72494
3.29354 2.67741 2.69355 4.24690 2.90347 2.73739 3.18146 2.89801
2.37887 2.77519 2.98518 4.58477 3.61503
0.00768 5.26922 5.99156 0.61958 0.77255 0.48576 0.95510
163 2.75180 4.13175 5.01736 4.40338 3.39166 3.62975 4.56690
1.50137 4.19095 1.60728 2.50979 4.39203 4.59542 4.17412 4.20274
3.46049 3.29400 1.84066 5.06235 3.70279 541 i x - E
2.68618 4.42225 2.77519 2.73123 3.46354 2.40513 3.72494
3.29354 2.67741 2.69355 4.24690 2.90347 2.73739 3.18146 2.89801
2.37887 2.77519 2.98518 4.58477 3.61503
0.00768 5.26922 5.99156 0.61958 0.77255 0.48576 0.95510
164 2.27651 3.77107 4.98338 4.37013 2.78642 2.96030 4.54805
2.63828 3.89138 1.67605 2.70336 4.36851 4.58007 4.13527 4.18062
3.20191 3.11205 1.54124 4.68946 3.81254 542 v x - E
2.68618 4.42225 2.77519 2.73123 3.46354 2.40513 3.72494
3.29354 2.67741 2.69355 4.24690 2.90347 2.73739 3.18146 2.89801
2.37887 2.77519 2.98518 4.58477 3.61503
0.00768 5.26922 5.99156 0.61958 0.77255 0.48576 0.95510
165 2.86935 2.10025 5.00118 4.38679 1.72501 3.71904 4.55173
2.60776 4.17464 1.69713 2.75551 4.37688 4.58262 4.30835 4.18755
3.18729 3.02229 2.53181 4.90033 3.71067 543 l x - E
2.68618 4.42225 2.77519 2.73123 3.46354 2.40513 3.72494
3.29354 2.67741 2.69355 4.24690 2.90347 2.73739 3.18146 2.89801
2.37887 2.77519 2.98518 4.58477 3.61503
0.00768 5.26922 5.99156 0.61958 0.77255 0.48576 0.95510
166 3.11747 5.04218 4.99194 4.89694 5.73929 3.82194 5.74837
5.22746 4.93416 4.93251 5.70575 4.57939 4.67953 5.12151 5.05861
0.39785 1.80669 4.38795 7.07600 5.98265 544 S x - E
2.68618 4.42225 2.77519 2.73123 3.46354 2.40513 3.72494
3.29354 2.67741 2.69355 4.24690 2.90347 2.73739 3.18146 2.89801
2.37887 2.77519 2.98518 4.58477 3.61503
0.00768 5.26922 5.99156 0.61958 0.77255 0.48576 0.95510
167 0.22265 4.57380 5.35267 5.26033 5.72619 3.52046 5.87175
5.16598 5.16745 4.90400 5.66915 4.24196 4.66571 5.29870 5.19861
3.29595 3.39000 4.33118 7.08553 6.04381 545 A x - S
2.68618 4.42225 2.77519 2.73123 3.46354 2.40513 3.72494
3.29354 2.67741 2.69355 4.24690 2.90347 2.73739 3.18146 2.89801
2.37887 2.77519 2.98518 4.58477 3.61503
0.00768 5.26922 5.99156 0.61958 0.77255 0.48576 0.95510

```
    168   2.95338  4.43439  5.39868  5.24514  5.57189  3.80877  5.81241
4.88895  5.08407  4.70838  5.51984  4.66619  4.66723  5.24601  5.12775
2.93042  0.26954  3.92918  6.96471  5.89772    546 T x - S
          2.68618  4.42225  2.77519  2.73123  3.46354  2.40513  3.72494
3.29354  2.67741  2.69355  4.24690  2.90347  2.73739  3.18146  2.89801
2.37887  2.77519  2.98518  4.58477  3.61503
          0.00768  5.26922  5.99156  0.61958  0.77255  0.48576  0.95510
    169   2.45431  4.43240  4.98030  4.36742  2.90714  4.21786  4.47229
2.36378  4.16021  1.26859  2.45670  4.36853  2.58625  3.97311  4.18091
3.52765  3.23852  2.36220  4.39425  3.57513    547 l x - B
          2.68618  4.42225  2.77519  2.73123  3.46354  2.40513  3.72494
3.29354  2.67741  2.69355  4.24690  2.90347  2.73739  3.18146  2.89801
2.37887  2.77519  2.98518  4.58477  3.61503
          0.02683  5.26922  3.84792  0.61958  0.77255  0.48576  0.95510
    170   2.70655  5.41754  2.94902  2.57656  3.76089  3.39410  2.22025
3.22920  2.62244  3.70868  4.46704  2.80649  2.07854  3.07522  3.14665
2.42180  2.46100  3.73892  5.87050  4.48033    548 p x - S
          2.68628  4.42246  2.77533  2.73130  3.46370  2.40523  3.72515
3.29374  2.67756  2.69355  4.24626  2.90356  2.73705  3.18167  2.89815
2.37802  2.77540  2.98534  4.58497  3.61518
          0.67712  0.92861  2.33490  0.05820  2.87277  0.44594  1.02227
    171   2.77406  5.44538  2.61117  2.34092  2.33234  3.54626  3.75149
3.98608  2.17189  3.70970  4.35240  3.09599  3.36250  2.63981  2.26396
2.38840  2.88368  3.67681  5.64194  4.37704    550 k x - H
          2.68618  4.42225  2.77519  2.73123  3.46354  2.40513  3.72494
3.29354  2.67741  2.69355  4.24690  2.90347  2.73739  3.18146  2.89801
2.37887  2.77519  2.98518  4.58477  3.61503
          0.00846  5.17216  5.89450  0.61958  0.77255  0.33806  1.24881
    172   2.64663  5.48931  1.74193  2.29093  4.12056  3.31327  3.96072
4.06399  2.53243  3.74064  4.53192  2.41963  3.28455  2.90006  3.09360
2.61245  2.55524  3.18141  5.22493  3.85883    551 d x - H
          2.68618  4.42225  2.77519  2.73123  3.46354  2.40513  3.72494
3.29354  2.67741  2.69355  4.24690  2.90347  2.73739  3.18146  2.89801
2.37887  2.77519  2.98518  4.58477  3.61503
          0.00768  5.26922  5.99156  0.61958  0.77255  0.48576  0.95510
    173   2.54480  4.20640  4.60554  3.59274  3.47115  4.00414  4.32591
2.22665  3.71373  2.01817  3.49302  3.95884  2.88786  3.54429  3.68307
3.12442  2.92492  1.44098  5.11011  3.17515    552 v x - H
          2.68618  4.42225  2.77519  2.73123  3.46354  2.40513  3.72494
3.29354  2.67741  2.69355  4.24690  2.90347  2.73739  3.18146  2.89801
2.37887  2.77519  2.98518  4.58477  3.61503
          0.00768  5.26922  5.99156  0.61958  0.77255  0.48576  0.95510
    174   2.62622  5.18882  2.59550  1.96624  4.48737  3.62865  3.72558
3.62893  1.65132  3.32424  4.13779  2.91551  4.16403  2.60528  2.91153
2.58039  2.86915  3.66458  5.93537  4.10245    553 k x - H
          2.68618  4.42225  2.77519  2.73123  3.46354  2.40513  3.72494
3.29354  2.67741  2.69355  4.24690  2.90347  2.73739  3.18146  2.89801
2.37887  2.77519  2.98518  4.58477  3.61503
          0.00768  5.26922  5.99156  0.61958  0.77255  0.48576  0.95510
    175   2.74271  5.50383  2.35474  2.09692  4.60475  3.77022  3.91132
3.87891  1.61957  3.21581  3.76404  2.81635  4.09833  2.84676  2.49739
2.78080  2.71534  3.89384  5.93721  4.52699    554 k x - H
          2.68618  4.42225  2.77519  2.73123  3.46354  2.40513  3.72494
3.29354  2.67741  2.69355  4.24690  2.90347  2.73739  3.18146  2.89801
2.37887  2.77519  2.98518  4.58477  3.61503
          0.00768  5.26922  5.99156  0.61958  0.77255  0.48576  0.95510
```

```
    176   2.51458  4.42977  4.76031  4.37224  2.72161  4.11692  4.36561
2.01015  4.16350  1.26284  3.42624  4.36989  4.58087  4.30016  4.18187
3.15919  2.79284  1.96317  4.79565  3.80637    555 l x - H
          2.68618  4.42225  2.77519  2.73123  3.46354  2.40513  3.72494
3.29354  2.67741  2.69355  4.24690  2.90347  2.73739  3.18146  2.89801
2.37887  2.77519  2.98518  4.58477  3.61503
          0.00517  5.26671        *  0.61958  0.77255  0.00000        *
//
HMMER3/f [3.3 | Nov 2019]
NAME  dsrm
ACC   PF00035.27
DESC  Double-stranded RNA binding motif
LENG  67
ALPH  amino
RF    no
MM    no
CONS  yes
CS    yes
MAP   yes
DATE  Wed Apr 22 15:11:30 2020
NSEQ  77
EFFN  21.247375
CKSUM 854144823
GA    23.00 21.00
TC    23.00 21.00
NC    22.90 20.90
STATS LOCAL MSV       -8.3458  0.71879
STATS LOCAL VITERBI   -9.2401  0.71879
STATS LOCAL FORWARD   -3.4639  0.71879
HMM          A        C        D        E        F        G        H
I        K        L        M        N        P        Q        R        S
T        V        W        Y
            m->m     m->i     m->d     i->m     i->i     d->m     d->d
  COMPO   2.34863  3.87137  3.38036  2.65848  3.37788  2.86369  3.67599
3.01119  2.32381  2.57027  3.86131  3.08075  3.20976  2.89671  2.95837
2.66919  2.90779  2.82375  4.95246  3.36283
          2.68618  4.42225  2.77519  2.73123  3.46354  2.40513  3.72494
3.29354  2.67741  2.69355  4.24690  2.90347  2.73739  3.18146  2.89801
2.37887  2.77519  2.98518  4.58477  3.61503
          0.03087  6.67751  3.53581  0.61958  0.77255  0.00000        *
      1   1.60728  4.28708  5.21938  3.85362  3.74913  5.05891  4.56144
3.59630  4.55746  3.75614  4.02335  4.94067  1.27417  3.04716  4.81505
2.42217  3.41711  3.81563  3.73326  2.04173      1 p - - H
          2.68618  4.42225  2.77519  2.73123  3.46354  2.40513  3.72494
3.29354  2.67741  2.69355  4.24690  2.90347  2.73739  3.18146  2.89801
2.37887  2.77519  2.98518  4.58477  3.61503
          0.00193  6.64857  7.37092  0.61958  0.77255  0.70206  0.68431
      2   4.05524  3.97891  5.88986  5.27934  3.95936  5.20996  5.53309
2.13283  0.67701  3.63563  3.25809  5.32617  5.57392  5.24167  2.66027
4.51660  4.28706  2.33633  6.07131  3.55424      2 K - - H
          2.68618  4.42225  2.77519  2.73123  3.46354  2.40513  3.72494
3.29354  2.67741  2.69355  4.24690  2.90347  2.73739  3.18146  2.89801
2.37887  2.77519  2.98518  4.58477  3.61503
          0.02141  6.65494  3.91705  0.61958  0.77255  0.33944  1.24539
      3   2.96585  4.21939  3.22303  3.41828  5.75084  2.81513  4.85160
5.23761  2.59806  4.08018  4.39555  1.95952  3.13427  2.77080  2.24102
1.55170  2.28019  4.79588  6.83663  5.42499      3 s - - H
```

```
         2.68648  4.42256  2.77550  2.73154  3.46058  2.40543  3.72525
3.29384  2.67771  2.69385  4.24720  2.90377  2.73455  3.18177  2.89831
2.37917  2.77550  2.98549  4.58507  3.61534
         0.05033  3.02707  7.38083  1.04716  0.43222  0.35607  1.20539
      4  2.79205  6.36535  3.24573  2.63509  3.11949  4.05507  2.99570
3.10841  1.72383  2.10261  4.00296  4.17072  4.24485  2.57285  2.32074
3.55536  3.09243  2.69481  6.81204  5.41538      7 k - - H
         2.68618  4.42225  2.77519  2.73123  3.46354  2.40513  3.72494
3.29354  2.67741  2.69355  4.24690  2.90347  2.73739  3.18146  2.89801
2.37887  2.77519  2.98518  4.58477  3.61503
         0.00187  6.67751  7.39985  0.61958  0.77255  0.48576  0.95510
      5  6.13481  7.19860  8.16392  7.59613  2.37744  7.78725  7.99216
1.91865  7.53628  0.36684  4.76623  8.00480  7.42868  7.06503  7.32339
7.31308  6.30356  3.05091  7.52806  6.72963      8 L - - H
         2.68618  4.42225  2.77519  2.73123  3.46354  2.40513  3.72494
3.29354  2.67741  2.69355  4.24690  2.90347  2.73739  3.18146  2.89801
2.37887  2.77519  2.98518  4.58477  3.61503
         0.00187  6.67751  7.39985  0.61958  0.77255  0.48576  0.95510
      6  3.05162  6.35606  4.18774  2.51850  3.70550  4.69639  2.62386
3.96001  3.63256  3.18422  3.70393  1.91590  3.14948  1.39536  2.73278
3.30302  3.49038  4.73214  6.80521  2.85784      9 q - - H
         2.68618  4.42225  2.77519  2.73123  3.46354  2.40513  3.72494
3.29354  2.67741  2.69355  4.24690  2.90347  2.73739  3.18146  2.89801
2.37887  2.77519  2.98518  4.58477  3.61503
         0.00187  6.67751  7.39985  0.61958  0.77255  0.48576  0.95510
      7  3.02001  6.42023  2.90428  1.14172  5.76668  3.73241  4.19759
5.25363  2.66134  4.05689  5.46053  3.73131  5.07311  1.60473  2.79933
2.48537  3.22039  4.12699  6.85209  5.44030     10 e - - H
         2.68663  4.42271  2.77565  2.73140  3.46399  2.40558  3.72457
3.29037  2.67757  2.69311  4.24476  2.90392  2.73785  3.18192  2.89661
2.37932  2.77565  2.98564  4.58522  3.61312
         0.23415  1.56950  7.39985  0.02779  3.59705  0.48576  0.95510
      8  4.06090  3.10664  6.03735  5.42109  1.84184  5.23152  3.82785
2.58199  4.26816  1.50641  4.55287  5.39810  5.59510  4.48588  4.38045
4.54335  3.59467  3.09931  3.14528  1.32638     12 y - - H
         2.68618  4.42225  2.77519  2.73123  3.46354  2.40513  3.72494
3.29354  2.67741  2.69355  4.24690  2.90347  2.73739  3.18146  2.89801
2.37887  2.77519  2.98518  4.58477  3.61503
         0.00187  6.67751  7.39985  0.61958  0.77255  0.48576  0.95510
      9  1.68244  1.42277  6.01440  5.39968  2.90723  3.66005  5.56136
2.52492  5.18659  2.22661  3.24743  3.97950  5.59209  3.44323  4.33352
3.08649  3.42947  2.43554  6.05786  4.26966     13 c - - H
         2.68618  4.42225  2.77519  2.73123  3.46354  2.40513  3.72494
3.29354  2.67741  2.69355  4.24690  2.90347  2.73739  3.18146  2.89801
2.37887  2.77519  2.98518  4.58477  3.61503
         0.00187  6.67751  7.39985  0.61958  0.77255  0.48576  0.95510
     10  3.37992  6.41675  3.45363  2.46035  5.76163  3.36272  2.62542
3.63429  2.06209  2.36784  4.64061  3.38123  5.07388  1.30038  2.83703
3.53669  3.34647  3.46949  6.84955  4.39346     14 q - - H
         2.68618  4.42225  2.77519  2.73123  3.46354  2.40513  3.72494
3.29354  2.67741  2.69355  4.24690  2.90347  2.73739  3.18146  2.89801
2.37887  2.77519  2.98518  4.58477  3.61503
         0.00187  6.67751  7.39985  0.61958  0.77255  0.48576  0.95510
     11  3.30983  4.47495  4.16826  3.37109  4.54676  3.46470  2.95362
3.44557  1.47760  4.10420  3.92852  3.21768  3.14937  1.89833  1.83119
3.12257  3.41337  3.58371  6.83160  4.32910     15 k - - H
```

```
          2.68618  4.42225  2.77519  2.73123  3.46354  2.40513  3.72494
3.29354  2.67741  2.69355  4.24690  2.90347  2.73739  3.18146  2.89801
2.37887  2.77519  2.98518  4.58477  3.61503
          0.00187  6.67751  7.39985  0.61958  0.77255  0.48576  0.95510
     12   2.56057  6.40466  3.55698  2.65323  2.86111  3.52303  3.46882
3.17259  2.10925  2.68979  2.93896  2.29852  3.14934  3.33938  2.49294
3.31072  2.61557  4.28141  6.84078  3.37243     16 k - - T
          2.68618  4.42225  2.77519  2.73123  3.46354  2.40513  3.72494
3.29354  2.67741  2.69355  4.24690  2.90347  2.73739  3.18146  2.89801
2.37887  2.77519  2.98518  4.58477  3.61503
          0.01102  6.67751  4.63559  0.61958  0.77255  0.48576  0.95510
     13   3.56445  4.40230  3.74776  3.34217  5.74063  1.44701  2.85209
4.46194  1.88546  3.01552  5.44192  3.02116  3.14211  3.96092  1.92977
3.08983  3.26951  4.78829  3.52152  4.59836     17 g - - T
          2.68599  4.42139  2.77602  2.73198  3.46444  2.40543  3.71754
3.29408  2.67656  2.69294  4.24913  2.90514  2.73776  3.18169  2.89948
2.37842  2.77617  2.98320  4.58416  3.61407
          0.42319  1.29627  2.63814  1.30722  0.31549  0.41303  1.08365
     14   2.81774  6.21733  3.57889  2.75450  2.90658  3.17932  3.06203
3.08951  2.05034  2.90771  3.50975  3.31606  2.33577  3.26559  3.60197
3.24263  3.48854  3.06237  3.07737  2.47708     24 k - - S
          2.68618  4.42225  2.77519  2.73123  3.46354  2.40513  3.72494
3.29354  2.67741  2.69355  4.24690  2.90347  2.73739  3.18146  2.89801
2.37887  2.77519  2.98518  4.58477  3.61503
          0.00201  6.60469  7.32703  0.61958  0.77255  0.78808  0.60645
     15   2.91599  6.37406  2.46473  2.45751  4.41957  2.54342  4.82896
4.00108  2.20798  2.86551  3.18450  3.21827  2.06937  2.82102  2.62759
3.83039  2.56102  4.15798  6.80774  3.36670     25 p - - E
          2.68618  4.42225  2.77519  2.73123  3.46354  2.40513  3.72494
3.29354  2.67741  2.69355  4.24690  2.90347  2.73739  3.18146  2.89801
2.37887  2.77519  2.98518  4.58477  3.61503
          0.00197  6.62734  7.34968  0.61958  0.77255  0.48425  0.95753
     16   3.39875  5.48012  4.46927  2.61284  3.70837  3.49125  3.94534
2.51571  3.35614  2.18296  2.85315  5.16688  1.19201  5.06717  5.01112
4.43859  3.11608  2.64341  4.83824  2.81666     26 p - - E
          2.68618  4.42225  2.77519  2.73123  3.46354  2.40513  3.72494
3.29354  2.67741  2.69355  4.24690  2.90347  2.73739  3.18146  2.89801
2.37887  2.77519  2.98518  4.58477  3.61503
          0.00191  6.65861  7.38096  0.61958  0.77255  0.54886  0.86182
     17   3.32261  6.40963  2.84432  2.18609  5.75503  3.95589  4.15925
3.07071  2.89812  3.44851  5.45020  1.77327  1.95679  2.61255  3.24125
2.66103  3.43306  2.55329  4.78921  5.43096     27 n - - E
          2.68618  4.42225  2.77519  2.73123  3.46354  2.40513  3.72494
3.29354  2.67741  2.69355  4.24690  2.90347  2.73739  3.18146  2.89801
2.37887  2.77519  2.98518  4.58477  3.61503
          0.00189  6.66705  7.38940  0.61958  0.77255  0.40433  1.10090
     18   3.60051  3.53123  3.11917  2.81519  1.97847  2.92863  5.17866
3.16629  3.73115  2.92668  3.60858  2.72407  3.23765  4.46917  3.82053
3.40052  2.79376  3.25555  6.35174  1.50476     28 y - - E
          2.68618  4.42225  2.77519  2.73123  3.46354  2.40513  3.72494
3.29354  2.67741  2.69355  4.24690  2.90347  2.73739  3.18146  2.89801
2.37887  2.77519  2.98518  4.58477  3.61503
          0.00187  6.67751  7.39985  0.61958  0.77255  0.48576  0.95510
     19   3.57296  5.01932  3.71982  1.95696  3.49399  4.68938  4.11045
3.65420  2.75452  3.54581  4.64875  3.22360  2.94509  2.68379  2.35805
2.47562  2.76474  2.35836  6.82434  2.13880     29 e - - E
```

```
        2.68618  4.42225  2.77519  2.73123  3.46354  2.40513  3.72494
3.29354  2.67741  2.69355  4.24690  2.90347  2.73739  3.18146  2.89801
2.37887  2.77519  2.98518  4.58477  3.61503
        0.01984  6.67751  3.99611  0.61958  0.77255  0.48576  0.95510
     20  3.67484  3.57065  4.28456  2.97024  2.48586  5.03199  3.81826
2.73230  2.55722  2.58589  4.23857  3.91950  5.40735  4.15529  3.87970
2.51243  2.17239  2.41396  3.78330  1.77497     30 y - - E
        2.68618  4.42225  2.77519  2.73123  3.46354  2.40513  3.72494
3.29354  2.67741  2.69355  4.24690  2.90347  2.73739  3.18146  2.89801
2.37887  2.77519  2.98518  4.58477  3.61503
        0.00191  6.65957  7.38192  0.61958  0.77255  0.57202  0.83099
     21  3.43269  3.83052  5.20776  2.85445  3.49104  5.06692  3.08602
2.26129  2.97323  2.53068  4.03648  3.11761  2.29891  4.81050  2.72925
2.75446  2.61168  1.82772  4.77851  3.74726     31 v - - E
        2.68618  4.42225  2.77519  2.73123  3.46354  2.40513  3.72494
3.29354  2.67741  2.69355  4.24690  2.90347  2.73739  3.18146  2.89801
2.37887  2.77519  2.98518  4.58477  3.61503
        0.02570  6.66633  3.72562  0.61958  0.77255  0.39975  1.11014
     22  3.22986  6.39138  3.05221  2.10550  3.81956  3.24035  3.82221
2.48609  2.46249  3.14695  4.25020  3.01573  3.91722  3.28707  2.87295
1.65056  2.92177  2.98616  6.82606  3.86786     32 s - - E
        2.68618  4.42225  2.77519  2.73123  3.46354  2.40513  3.72494
3.29354  2.67741  2.69355  4.24690  2.90347  2.73739  3.18146  2.89801
2.37887  2.77519  2.98518  4.58477  3.61503
        0.00192  6.65402  7.37636  0.61958  0.77255  0.33537  1.25552
     23  3.62257  3.24634  3.22470  1.95123  5.74867  3.58045  2.78598
3.10734  2.77056  4.08689  4.49115  4.15178  3.57581  2.14397  2.43421
2.61871  2.83665  2.03339  6.84310  4.39184     33 e - - E
        2.68618  4.42225  2.77519  2.73123  3.46354  2.40513  3.72494
3.29354  2.67741  2.69355  4.24690  2.90347  2.73739  3.18146  2.89801
2.37887  2.77519  2.98518  4.58477  3.61503
        0.00187  6.67751  7.39985  0.61958  0.77255  0.48576  0.95510
     24  2.35983  3.61643  2.73547  1.72621  4.63459  2.62653  4.24674
4.10273  2.39549  3.68704  5.45777  3.03020  2.93251  2.71725  3.82457
2.74185  2.37097  3.14604  4.84220  5.43887     34 e - - E
        2.68618  4.42225  2.77519  2.73123  3.46354  2.40513  3.72494
3.29354  2.67741  2.69355  4.24690  2.90347  2.73739  3.18146  2.89801
2.37887  2.77519  2.98518  4.58477  3.61503
        0.00187  6.67751  7.39985  0.61958  0.77255  0.48576  0.95510
     25  3.90654  6.41805  3.40254  2.98221  5.76349  1.06383  4.19626
5.24972  2.67844  4.17016  4.41827  3.16472  5.07365  2.92783  3.03433
2.86756  1.91364  2.66419  6.85050  5.43933     35 g - - S
        2.68618  4.42225  2.77519  2.73123  3.46354  2.40513  3.72494
3.29354  2.67741  2.69355  4.24690  2.90347  2.73739  3.18146  2.89801
2.37887  2.77519  2.98518  4.58477  3.61503
        0.00187  6.67751  7.39985  0.61958  0.77255  0.48576  0.95510
     26  3.90645  6.41811  3.32120  2.70406  4.68377  1.97557  3.34525
3.87530  2.52113  4.72357  3.89868  3.06878  1.36983  3.10426  3.40339
2.09616  4.13760  3.25112  6.85053  5.43932     36 p - - S
        2.68618  4.42225  2.77519  2.73123  3.46354  2.40513  3.72494
3.29354  2.67741  2.69355  4.24690  2.90347  2.73739  3.18146  2.89801
2.37887  2.77519  2.98518  4.58477  3.61503
        0.04994  6.67751  3.04794  0.61958  0.77255  0.48576  0.95510
     27  2.14969  4.85931  2.37508  2.51198  5.72717  2.99783  3.06939
3.67021  2.81237  4.68689  5.42168  3.09008  1.99528  3.92512  2.50778
2.20654  2.71559  3.26170  6.81342  5.40196     37 p - - T
```

```
          2.68618  4.42225  2.77519  2.73123  3.46354  2.40513  3.72494
3.29354  2.67741  2.69355  4.24690  2.90347  2.73739  3.18146  2.89801
2.37887  2.77519  2.98518  4.58477  3.61503
          0.01287  6.62953  4.46806  0.61958  0.77255  1.02049  0.44694
     28   3.27312  4.11219  2.63591  3.38728  5.65352  2.31036  1.52487
3.39876  2.44385  3.44239  4.47168  3.36226  3.83933  3.49346  3.79135
2.55967  2.42286  2.86883  6.77207  3.76294     38 h - - S
          2.68618  4.42225  2.77519  2.73123  3.46354  2.40513  3.72494
3.29354  2.67741  2.69355  4.24690  2.90347  2.73739  3.18146  2.89801
2.37887  2.77519  2.98518  4.58477  3.61503
          0.02876  6.61864  3.61139  0.61958  0.77255  1.10782  0.40089
     29   2.20844  4.66356  2.91877  2.47645  4.64660  3.17363  2.61188
4.27336  2.27328  4.65808  3.13808  2.45839  3.12405  3.33095  2.93517
1.95765  2.83352  3.73581  6.78441  5.37282     39 s - - S
          2.68618  4.42225  2.77519  2.73123  3.46354  2.40513  3.72494
3.29354  2.67741  2.69355  4.24690  2.90347  2.73739  3.18146  2.89801
2.37887  2.77519  2.98518  4.58477  3.61503
          0.51578  6.59192  0.91231  0.61958  0.77255  1.29131  0.32146
     30   2.59725  3.19004  3.27828  2.28171  5.28341  3.30333  3.29493
2.72534  2.25222  2.12155  4.99396  2.38518  3.15710  3.06607  3.00415
3.15069  3.69916  2.78959  6.39001  4.98630     40 l - - S
          2.68618  4.42225  2.77519  2.73123  3.46354  2.40513  3.72494
3.29354  2.67741  2.69355  4.24690  2.90347  2.73739  3.18146  2.89801
2.37887  2.77519  2.98518  4.58477  3.61503
          0.01878  6.07955  4.11541  0.61958  0.77255  0.44378  1.02613
     31   3.76689  6.24532  4.02848  3.47249  4.07112  4.54569  3.98970
5.05376  1.92043  4.00111  2.35599  2.67806  1.79454  2.59978  2.10307
2.69748  2.49286  3.64000  6.68595  5.28328     41 p - - E
          2.68643  4.41530  2.77545  2.73149  3.46222  2.40538  3.72520
3.29324  2.67766  2.69323  4.24715  2.90372  2.73765  3.18030  2.89826
2.37912  2.77545  2.98544  4.58502  3.61528
          0.16234  1.96660  4.61347  0.04815  3.05750  0.26864  1.44570
     32   3.89171  6.22272  4.21989  2.57963  4.20929  3.30938  4.89228
2.56298  1.95767  2.36127  3.70987  3.72340  3.73484  2.49444  2.46084
2.53115  2.21843  2.60241  6.69803  4.46106     43 k - - E
          2.68618  4.42225  2.77519  2.73123  3.46354  2.40513  3.72494
3.29354  2.67741  2.69355  4.24690  2.90347  2.73739  3.18146  2.89801
2.37887  2.77519  2.98518  4.58477  3.61503
          0.00194  6.63948  7.36183  0.61958  0.77255  0.66937  0.71750
     33   4.26281  4.05612  6.18758  4.11621  0.46911  5.41116  4.34464
3.26706  5.38292  3.80042  4.74623  5.53810  5.76969  5.48834  5.37951
3.97771  4.49403  3.20116  2.87425  2.40127     44 F - - E
          2.68618  4.42225  2.77519  2.73123  3.46354  2.40513  3.72494
3.29354  2.67741  2.69355  4.24690  2.90347  2.73739  3.18146  2.89801
2.37887  2.77519  2.98518  4.58477  3.61503
          0.00192  6.65214  7.37449  0.61958  0.77255  0.59015  0.80798
     34   3.61873  6.34665  4.17409  2.56425  5.66593  2.90558  3.58280
2.54918  2.42289  3.42159  3.97885  3.63010  3.89486  2.93037  2.76193
2.26190  2.00830  1.98557  6.79508  3.36070     45 v - - E
          2.68618  4.42225  2.77519  2.73123  3.46354  2.40513  3.72494
3.29354  2.67741  2.69355  4.24690  2.90347  2.73739  3.18146  2.89801
2.37887  2.77519  2.98518  4.58477  3.61503
          0.00190  6.66219  7.38453  0.61958  0.77255  0.69235  0.69394
     35   1.90615  1.87568  5.95395  5.34240  3.74496  2.79764  5.53955
2.48774  2.58821  3.61915  3.16259  5.35299  4.16887  5.28111  5.16722
3.20689  2.42733  1.67499  6.04995  3.67252     46 v - - E
```

2.68644 4.42251 2.77545 2.73016 3.46380 2.40539 3.72520
3.29380 2.67640 2.69381 4.24111 2.90373 2.73765 3.18172 2.89827
2.37913 2.77545 2.98544 4.58503 3.61529
0.04268 3.19034 7.38453 1.03914 0.43658 0.37536 1.16169
36 2.83903 3.84955 3.78138 2.12237 5.75645 3.57682 3.48187
3.40869 2.56552 4.71771 4.16751 4.14948 3.90424 2.66452 2.71442
2.02251 1.57395 2.73871 6.84698 5.43712 50 t - - E
2.68618 4.42225 2.77519 2.73123 3.46354 2.40513 3.72494
3.29354 2.67741 2.69355 4.24690 2.90347 2.73739 3.18146 2.89801
2.37887 2.77519 2.98518 4.58477 3.61503
0.00187 6.67751 7.39985 0.61958 0.77255 0.48576 0.95510
37 2.33811 2.20470 6.04705 5.43111 2.68486 5.24100 5.57597
1.85755 4.10441 2.28546 3.06964 5.40800 5.60409 3.12835 5.21662
4.55313 4.30109 1.21363 6.06519 4.88957 51 v - - E
2.68618 4.42225 2.77519 2.73123 3.46354 2.40513 3.72494
3.29354 2.67741 2.69355 4.24690 2.90347 2.73739 3.18146 2.89801
2.37887 2.77519 2.98518 4.58477 3.61503
0.03019 6.67751 3.55854 0.61958 0.77255 0.48576 0.95510
38 3.90220 4.09516 3.67028 2.22081 4.48771 4.71263 4.91023
2.69267 1.78526 3.77306 4.54707 2.63055 5.10378 3.64637 2.49181
2.75135 2.41034 2.15849 4.27762 2.77152 52 k - - E
2.68618 4.42225 2.77519 2.73123 3.46354 2.40513 3.72494
3.29354 2.67741 2.69355 4.24690 2.90347 2.73739 3.18146 2.89801
2.37887 2.77519 2.98518 4.58477 3.61503
0.00193 6.64924 7.37159 0.61958 0.77255 0.83684 0.56753
39 3.43756 5.40980 6.01640 5.39994 2.05069 5.20821 5.54255
1.70916 5.18128 1.82291 3.63956 5.37568 3.73038 5.30953 5.18394
3.31077 3.40441 1.24436 4.71196 3.27151 53 v - - E
2.68798 4.42405 2.77480 2.73052 3.46345 2.40464 3.72233
3.29066 2.67701 2.69399 4.24869 2.90440 2.73608 3.18247 2.89832
2.38015 2.77540 2.98428 4.58657 3.61449
0.35775 1.85627 1.93448 1.65773 0.21143 0.31588 1.30618
40 3.12570 4.23798 1.77668 1.95197 4.18693 2.13807 4.74934
5.13574 2.38181 3.84831 5.34295 1.81023 3.61303 3.58596 3.03783
2.73391 4.02023 4.69390 6.73456 5.32287 62 d - - T
2.68618 4.42225 2.77519 2.73123 3.46354 2.40513 3.72494
3.29354 2.67741 2.69355 4.24690 2.90347 2.73739 3.18146 2.89801
2.37887 2.77519 2.98518 4.58477 3.61503
0.00218 6.52685 7.24919 0.61958 0.77255 1.07806 0.41590
41 2.96397 6.32980 2.06895 2.28048 5.67605 1.13540 4.10160
5.16291 2.85473 4.63558 2.75169 2.57864 4.98279 3.58123 3.47052
2.87025 3.61945 4.72108 6.76175 5.34998 63 g - - T
2.68623 4.42230 2.77524 2.73128 3.46283 2.40518 3.72499
3.29359 2.67746 2.69321 4.24695 2.90352 2.73744 3.18151 2.89806
2.37892 2.77524 2.98523 4.58482 3.61508
0.02805 3.61297 7.28277 0.18445 1.78118 1.30085 0.31787
42 3.82492 6.28995 3.25046 2.49592 2.98032 3.96991 3.19532
2.90325 1.81765 3.24542 2.95816 3.10801 4.99896 2.84538 2.41915
3.47131 2.42431 2.93039 6.73402 2.75358 65 k - - E
2.68618 4.42225 2.77519 2.73123 3.46354 2.40513 3.72494
3.29354 2.67741 2.69355 4.24690 2.90347 2.73739 3.18146 2.89801
2.37887 2.77519 2.98518 4.58477 3.61503
0.00209 6.56867 7.29101 0.61958 0.77255 1.42436 0.27531
43 3.57987 6.30281 3.20457 2.06305 3.53994 3.53095 3.57354
2.23669 2.21303 1.89735 5.34693 3.62784 4.27411 3.07783 2.42874
3.56700 2.62649 2.74666 6.74344 5.34054 66 l - - E

```
         2.68618  4.42225  2.77519  2.73123  3.46354  2.40513  3.72494
3.29354  2.67741  2.69355  4.24690  2.90347  2.73739  3.18146  2.89801
2.37887  2.77519  2.98518  4.58477  3.61503
         0.00209  6.56867  7.29101  0.61958  0.77255  1.42436  0.27531
     44  2.90291  3.87138  5.94800  5.33162  2.33034  5.14075  4.13844
1.67024  5.11319  2.47893  3.63836  5.30787  2.61189  5.24164  3.42634
4.45264  3.12135  2.23462  3.22798  1.74361     67 i - - E
         2.68618  4.42225  2.77519  2.73123  3.46354  2.40513  3.72494
3.29354  2.67741  2.69355  4.24690  2.90347  2.73739  3.18146  2.89801
2.37887  2.77519  2.98518  4.58477  3.61503
         0.05954  6.56867  2.87532  0.61958  0.77255  1.42436  0.27531
     45  2.11303  4.09958  3.85973  2.91864  4.19523  1.57284  4.83699
2.49062  3.16717  3.57361  3.47720  3.76450  3.91997  3.22075  2.71105
2.59510  2.65811  3.30049  6.51155  3.20389     68 g - - E
         2.68661  4.41858  2.77387  2.72998  3.46397  2.40528  3.72537
3.29397  2.67784  2.69398  4.24733  2.90390  2.73689  3.18189  2.89844
2.37930  2.77439  2.98561  4.58520  3.61546
         0.08628  2.50176  7.23368  1.01719  0.44881  0.12355  2.15222
     46  2.41199  3.47595  4.23348  2.60675  5.56595  3.24341  4.91297
2.26545  2.65189  4.11456  3.55237  3.89993  4.48025  2.43333  2.54401
2.11397  2.26859  2.90833  6.74766  3.12742     73 s - - E
         2.68618  4.42225  2.77519  2.73123  3.46354  2.40513  3.72494
3.29354  2.67741  2.69355  4.24690  2.90347  2.73739  3.18146  2.89801
2.37887  2.77519  2.98518  4.58477  3.61503
         0.00187  6.67751  7.39985  0.61958  0.77255  0.48576  0.95510
     47  1.95575  6.56519  3.40548  3.31103  5.91432  0.62843  5.04011
5.39764  3.80952  4.23673  5.63646  3.76435  4.11217  3.76865  4.31055
3.37843  2.54504  4.96480  7.02608  5.61179     74 G - - E
         2.68618  4.42225  2.77519  2.73123  3.46354  2.40513  3.72494
3.29354  2.67741  2.69355  4.24690  2.90347  2.73739  3.18146  2.89801
2.37887  2.77519  2.98518  4.58477  3.61503
         0.00187  6.67751  7.39985  0.61958  0.77255  0.48576  0.95510
     48  2.89666  4.65308  2.93298  2.38460  2.99847  4.04162  3.59140
2.56812  2.12719  3.61937  5.40769  3.72066  3.92388  3.34051  3.12472
2.68740  2.33491  2.05642  3.05388  5.41308     75 v - - E
         2.68618  4.42225  2.77519  2.73123  3.46354  2.40513  3.72494
3.29354  2.67741  2.69355  4.24690  2.90347  2.73739  3.18146  2.89801
2.37887  2.77519  2.98518  4.58477  3.61503
         0.00187  6.67751  7.39985  0.61958  0.77255  0.48576  0.95510
     49  2.20030  3.89035  3.33115  3.00399  5.42961  0.95897  4.95249
4.85454  3.42163  4.44250  5.24474  4.27068  3.43617  4.09251  4.21010
2.11430  3.69413  2.72636  4.62564  3.42104     76 g - - E
         2.68727  4.42334  2.77628  2.73001  3.46463  2.40621  3.72603
3.28654  2.67630  2.69464  4.24798  2.90455  2.73848  3.17893  2.89909
2.37995  2.77146  2.98560  4.58586  3.61612
         0.11528  2.46650  3.72979  1.71954  0.19741  0.48576  0.95510
     50  2.40134  4.39278  3.72799  3.15653  4.05036  3.49419  4.33661
5.23248  2.32832  4.70575  3.40206  2.86730  3.22056  3.02206  2.07573
1.40420  2.43729  4.79119  6.83237  4.49973     84 s - - S
         2.68652  4.42259  2.77312  2.73157  3.46329  2.40547  3.72528
3.29388  2.67717  2.69389  4.24724  2.90381  2.73663  3.18180  2.89786
2.37921  2.77554  2.98552  4.58178  3.61537
         0.22581  1.83529  3.15669  0.03662  3.32544  0.33463  1.25739
     51  4.30569  6.64095  4.58750  4.10852  6.24553  4.99774  5.31191
5.70133  3.24409  5.16852  5.93826  1.02061  4.29595  4.42799  3.25564
1.10347  2.04477  5.23705  7.26812  5.93015     86 n - - S
```

2.68618 4.42225 2.77519 2.73123 3.46354 2.40513 3.72494
3.29354 2.67741 2.69355 4.24690 2.90347 2.73739 3.18146 2.89801
2.37887 2.77519 2.98518 4.58477 3.61503
0.00195 6.63569 7.35803 0.61958 0.77255 0.96705 0.47836
52 4.06970 4.39707 5.12079 4.52302 4.83300 5.07364 5.30557
2.96590 0.52040 2.38913 4.80923 4.87005 5.44514 4.05745 2.95656
3.74283 3.68240 2.98246 6.28365 5.07294 87 K - - H
2.68625 4.42232 2.77526 2.73130 3.46361 2.40520 3.72501
3.29252 2.67748 2.69318 4.24696 2.90353 2.73746 3.18153 2.89808
2.37894 2.77526 2.98525 4.58484 3.61510
0.03543 3.37634 7.35803 0.14531 2.00063 0.27153 1.43639
53 3.68938 6.44567 2.80572 3.24599 5.79491 4.70448 3.96929
5.27952 0.60838 4.74987 5.48642 3.11968 5.09815 2.54463 2.49481
3.89674 3.80998 3.82073 6.87311 5.46568 89 K - - H
2.68618 4.42225 2.77519 2.73123 3.46354 2.40513 3.72494
3.29354 2.67741 2.69355 4.24690 2.90347 2.73739 3.18146 2.89801
2.37887 2.77519 2.98518 4.58477 3.61503
0.00187 6.67751 7.39985 0.61958 0.77255 0.48576 0.95510
54 2.82497 6.41169 2.26981 1.59701 5.75424 4.68170 4.86902
3.90833 1.95147 2.03181 4.40791 3.72242 5.07512 2.18834 3.17351
3.58224 3.20006 3.19752 6.84588 4.35836 90 e - - H
2.68618 4.42225 2.77519 2.73123 3.46354 2.40513 3.72494
3.29354 2.67741 2.69355 4.24690 2.90347 2.73739 3.18146 2.89801
2.37887 2.77519 2.98518 4.58477 3.61503
0.00187 6.67751 7.39985 0.61958 0.77255 0.48576 0.95510
55 0.23883 6.34514 6.99553 7.06461 7.35893 3.92244 7.47459
6.90075 6.96163 6.59145 7.29198 6.12114 6.03771 6.95224 6.84219
1.78786 5.03576 5.84469 8.69002 7.73981 91 A - - H
2.68618 4.42225 2.77519 2.73123 3.46354 2.40513 3.72494
3.29354 2.67741 2.69355 4.24690 2.90347 2.73739 3.18146 2.89801
2.37887 2.77519 2.98518 4.58477 3.61503
0.00187 6.67751 7.39985 0.61958 0.77255 0.48576 0.95510
56 3.06368 4.76299 4.15760 1.11033 5.76543 3.40595 3.52919
4.17735 1.30028 4.72501 4.49959 4.14970 5.07570 2.35777 2.95788
3.56850 4.13988 4.81075 6.85183 5.44118 92 e - - H
2.68618 4.42225 2.77519 2.73123 3.46354 2.40513 3.72494
3.29354 2.67741 2.69355 4.24690 2.90347 2.73739 3.18146 2.89801
2.37887 2.77519 2.98518 4.58477 3.61503
0.00187 6.67751 7.39985 0.61958 0.77255 0.48576 0.95510
57 3.07852 6.40382 3.40395 3.60633 5.74280 4.68374 2.57754
2.75767 2.22159 2.51352 4.31168 2.11208 5.07706 1.56090 3.09363
2.35319 3.29431 4.06660 6.84017 5.43291 93 q - - H
2.68618 4.42225 2.77519 2.73123 3.46354 2.40513 3.72494
3.29354 2.67741 2.69355 4.24690 2.90347 2.73739 3.18146 2.89801
2.37887 2.77519 2.98518 4.58477 3.61503
0.00187 6.67751 7.39985 0.61958 0.77255 0.48576 0.95510
58 2.52615 3.97988 2.82749 3.05672 4.34786 3.99891 4.87244
3.07319 2.53500 1.96683 5.44309 2.38155 5.07788 2.21959 2.35233
1.96004 4.13913 3.82686 6.83770 5.43137 94 s - - H
2.68618 4.42225 2.77519 2.73123 3.46354 2.40513 3.72494
3.29354 2.67741 2.69355 4.24690 2.90347 2.73739 3.18146 2.89801
2.37887 2.77519 2.98518 4.58477 3.61503
0.00187 6.67751 7.39985 0.61958 0.77255 0.48576 0.95510
59 0.27669 2.04333 7.02188 6.93052 6.70289 5.15549 7.21183
4.19289 6.72460 5.82186 6.66598 6.08420 6.01315 6.76154 6.63428
3.59912 3.58849 3.98123 8.14947 7.08951 95 A - - H

2.68618 4.42225 2.77519 2.73123 3.46354 2.40513 3.72494
3.29354 2.67741 2.69355 4.24690 2.90347 2.73739 3.18146 2.89801
2.37887 2.77519 2.98518 4.58477 3.61503
0.00187 6.67751 7.39985 0.61958 0.77255 0.48576 0.95510
60 0.16113 4.24525 7.06443 7.08744 7.20856 4.05777 7.42224
6.73994 6.90461 6.44013 7.16872 6.11656 6.02720 6.91538 6.79103
2.60465 5.01770 5.77386 8.56859 3.96133 96 A - - H
2.68618 4.42225 2.77519 2.73123 3.46354 2.40513 3.72494
3.29354 2.67741 2.69355 4.24690 2.90347 2.73739 3.18146 2.89801
2.37887 2.77519 2.98518 4.58477 3.61503
0.00187 6.67751 7.39985 0.61958 0.77255 0.48576 0.95510
61 2.36527 5.94244 4.52305 1.85220 4.07701 4.84729 4.33602
3.15996 1.83708 2.22268 2.58312 4.44756 5.23329 2.81991 2.91248
3.46579 3.78847 2.32304 6.48505 3.58037 97 k - - H
2.68618 4.42225 2.77519 2.73123 3.46354 2.40513 3.72494
3.29354 2.67741 2.69355 4.24690 2.90347 2.73739 3.18146 2.89801
2.37887 2.77519 2.98518 4.58477 3.61503
0.00187 6.67751 7.39985 0.61958 0.77255 0.48576 0.95510
62 2.29786 6.41851 2.00582 2.41580 5.76419 4.67997 2.72746
3.89238 2.08860 2.39157 3.85359 2.80928 5.07345 2.58825 2.78844
2.94562 3.24637 3.23811 6.85082 5.43950 98 d - - H
2.68618 4.42225 2.77519 2.73123 3.46354 2.40513 3.72494
3.29354 2.67741 2.69355 4.24690 2.90347 2.73739 3.18146 2.89801
2.37887 2.77519 2.98518 4.58477 3.61503
0.00187 6.67751 7.39985 0.61958 0.77255 0.48576 0.95510
63 0.70559 3.85517 6.03572 5.41980 3.90392 3.45659 5.56692
2.45469 5.20247 2.63921 2.57453 5.39827 5.59610 5.33182 5.20716
3.26090 3.01654 2.69006 6.05781 4.88206 99 a - - H
2.68618 4.42225 2.77519 2.73123 3.46354 2.40513 3.72494
3.29354 2.67741 2.69355 4.24690 2.90347 2.73739 3.18146 2.89801
2.37887 2.77519 2.98518 4.58477 3.61503
0.01473 6.67751 4.31493 0.61958 0.77255 0.48576 0.95510
64 4.08643 2.11095 6.06609 5.44990 2.98737 5.25941 5.59305
2.17544 5.23187 0.76053 3.99861 5.42709 5.61951 5.35670 5.23343
4.57200 3.52548 2.58457 4.05993 3.19589 100 l - - H
2.68618 4.42225 2.77519 2.73123 3.46354 2.40513 3.72494
3.29354 2.67741 2.69355 4.24690 2.90347 2.73739 3.18146 2.89801
2.37887 2.77519 2.98518 4.58477 3.61503
0.00190 6.66467 7.38702 0.61958 0.77255 0.66183 0.72548
65 2.53573 4.79208 3.00420 1.76191 5.75621 4.66947 3.77373
4.36098 2.39746 2.79043 5.45016 2.43440 5.06296 1.86902 1.97806
2.82312 3.62639 4.80118 6.84175 5.43002 101 e - - H
2.68618 4.42225 2.77519 2.73123 3.46354 2.40513 3.72494
3.29354 2.67741 2.69355 4.24690 2.90347 2.73739 3.18146 2.89801
2.37887 2.77519 2.98518 4.58477 3.61503
0.00190 6.66467 7.38702 0.61958 0.77255 0.66183 0.72548
66 3.12033 6.37014 3.19404 2.51110 4.54446 4.05882 3.26805
3.19413 1.93091 2.73622 4.26620 2.63545 5.07277 2.31980 3.42023
3.04985 3.49977 2.30730 4.81053 2.23565 102 k - - H
2.68618 4.42225 2.77519 2.73123 3.46354 2.40513 3.72494
3.29354 2.67741 2.69355 4.24690 2.90347 2.73739 3.18146 2.89801
2.37887 2.77519 2.98518 4.58477 3.61503
0.02002 6.66467 3.98748 0.61958 0.77255 0.66183 0.72548
67 4.02637 5.44531 5.73593 3.94419 4.54212 5.16509 4.45803
2.71307 3.72926 0.45323 3.25474 4.28411 5.53142 4.20314 4.16780
3.93792 4.25842 3.04235 6.06252 3.47614 103 L - - H

```
          2.68618  4.42225  2.77519  2.73123  3.46354  2.40513  3.72494
3.29354  2.67741  2.69355  4.24690  2.90347  2.73739  3.18146  2.89801
2.37887  2.77519  2.98518  4.58477  3.61503
          0.00130  6.64595        *  0.61958  0.77255  0.00000        *
//
HMMER3/f [3.3 | Nov 2019]
NAME  DUF547
ACC   PF04784.15
DESC  Protein of unknown function, DUF547
LENG  125
ALPH  amino
RF    no
MM    no
CONS  yes
CS    no
MAP   yes
DATE  Fri Apr 17 05:00:08 2020
NSEQ  219
EFFN  4.160385
CKSUM 2276703782
GA    20.20 20.20
TC    20.20 20.60
NC    20.10 19.80
STATS LOCAL MSV       -9.7751  0.71267
STATS LOCAL VITERBI  -10.7043  0.71267
STATS LOCAL FORWARD   -4.2894  0.71267
HMM          A        C        D        E        F        G        H
I        K        L        M        N        P        Q        R        S
T        V        W        Y
            m->m     m->i     m->d     i->m     i->i     d->m     d->d
  COMPO   2.50482  4.19327  3.04889  2.67822  3.18670  3.05756  3.59147
2.82329  2.70256  2.35675  3.69657  2.89103  3.28858  3.04599  2.92183
2.65913  2.91357  2.80367  4.52827  3.34161
          2.68618  4.42225  2.77519  2.73123  3.46354  2.40513  3.72494
3.29354  2.67741  2.69355  4.24690  2.90347  2.73739  3.18146  2.89801
2.37887  2.77519  2.98518  4.58477  3.61503
          0.30713  5.19127  1.35140  0.61958  0.77255  0.00000        *
      1   2.83472  4.27411  4.57868  3.97873  2.76336  3.99845  4.31842
2.32083  3.76124  1.54854  3.32861  4.00438  2.10416  3.84345  3.84120
3.16150  3.01060  2.10352  4.89190  3.52193      1 l - - -
          2.68618  4.42225  2.77519  2.73123  3.46354  2.40513  3.72494
3.29354  2.67741  2.69355  4.24690  2.90347  2.73739  3.18146  2.89801
2.37887  2.77519  2.98518  4.58477  3.61503
          0.01118  4.89532  5.61767  0.61958  0.77255  0.71885  0.66809
      2   2.50214  5.38450  2.48162  2.31503  4.62179  3.46273  3.73823
3.93868  2.37539  3.27605  4.42564  2.74481  3.90967  2.49402  2.12595
2.16317  2.83184  3.54937  5.81826  4.40841      2 r - - -
          2.68618  4.42225  2.77519  2.73123  3.46354  2.40513  3.72494
3.29354  2.67741  2.69355  4.24690  2.90347  2.73739  3.18146  2.89801
2.37887  2.77519  2.98518  4.58477  3.61503
          0.01000  5.00638  5.72873  0.61958  0.77255  0.80624  0.59155
      3   2.60744  4.72273  2.64775  2.29315  4.08496  2.87533  3.61057
4.21941  2.29527  3.03346  4.25280  2.53233  3.79393  2.67481  2.43149
2.17969  2.90134  3.65960  5.82859  4.41961      3 s - - -
          2.68618  4.42225  2.77519  2.73123  3.46354  2.40513  3.72494
3.29354  2.67741  2.69355  4.24690  2.90347  2.73739  3.18146  2.89801
2.37887  2.77519  2.98518  4.58477  3.61503
          0.00969  5.03715  5.75949  0.61958  0.77255  0.51511  0.90989
```

4 2.86272 4.35979 4.87419 4.13180 2.59896 4.13748 4.46668
2.76186 4.06221 1.12871 2.43089 4.27834 4.34870 4.09975 4.09307
3.26189 2.88713 2.60649 2.97260 3.38889 4 l - - -
2.68618 4.42225 2.77519 2.73123 3.46354 2.40513 3.72494
3.29354 2.67741 2.69355 4.24690 2.90347 2.73739 3.18146 2.89801
2.37887 2.77519 2.98518 4.58477 3.61503
0.00886 5.12651 5.84885 0.61958 0.77255 0.40613 1.09728
5 2.46339 5.46537 2.74958 2.44185 4.43955 3.16192 3.91786
4.29499 2.01809 3.59601 4.50627 2.43873 3.27881 2.90673 2.97811
1.91769 2.42997 3.85556 5.89856 4.48809 5 s - - -
2.68627 4.42234 2.77526 2.73120 3.46363 2.40521 3.72504
3.29363 2.67748 2.69364 4.24699 2.90326 2.73749 3.18132 2.89810
2.37874 2.77480 2.98517 4.58486 3.61512
0.21871 1.64131 5.90761 0.11528 2.21745 0.47330 0.97536
6 2.73199 4.59156 2.69198 2.03726 4.81617 3.32413 2.75918
4.21534 2.22234 3.74236 4.51127 2.65043 3.56615 2.85900 1.91754
2.72085 3.00883 3.75084 5.90315 4.08552 7 r - - -
2.68618 4.42225 2.77519 2.73123 3.46354 2.40513 3.72494
3.29354 2.67741 2.69355 4.24690 2.90347 2.73739 3.18146 2.89801
2.37887 2.77519 2.98518 4.58477 3.61503
0.00830 5.19127 5.91362 0.61958 0.77255 0.48576 0.95510
7 2.41289 5.47266 2.16791 1.86475 4.81819 3.56798 3.72461
4.24324 2.34756 3.74268 4.51326 2.22118 3.64338 2.36336 3.02564
2.80701 3.17026 3.26231 5.90500 4.49359 8 e - - -
2.68618 4.42225 2.77519 2.73123 3.46354 2.40513 3.72494
3.29354 2.67741 2.69355 4.24690 2.90347 2.73739 3.18146 2.89801
2.37887 2.77519 2.98518 4.58477 3.61503
0.00830 5.19127 5.91362 0.61958 0.77255 0.48576 0.95510
8 2.56169 5.47050 2.59439 1.25611 4.81355 3.56633 3.66068
4.22246 2.60175 3.77486 4.51188 3.20354 4.13027 2.25782 2.92615
2.82689 2.84501 3.81832 5.90416 4.04859 9 e - - -
2.68618 4.42225 2.77519 2.73123 3.46354 2.40513 3.72494
3.29354 2.67741 2.69355 4.24690 2.90347 2.73739 3.18146 2.89801
2.37887 2.77519 2.98518 4.58477 3.61503
0.00830 5.19127 5.91362 0.61958 0.77255 0.48576 0.95510
9 2.65306 5.46604 3.27465 2.61679 4.80477 3.73855 3.71753
3.76777 1.53429 3.32340 4.51136 3.25082 4.16723 2.12724 1.85387
2.97281 2.92218 3.85393 5.89510 4.30402 10 k - - -
2.68618 4.42225 2.77519 2.73123 3.46354 2.40513 3.72494
3.29354 2.67741 2.69355 4.24690 2.90347 2.73739 3.18146 2.89801
2.37887 2.77519 2.98518 4.58477 3.61503
0.00830 5.19127 5.91362 0.61958 0.77255 0.48576 0.95510
10 2.93234 4.42759 4.70616 3.81253 3.15615 4.14460 4.46308
2.35150 2.79225 1.06119 2.97400 4.21539 4.51078 3.77532 4.04688
3.44356 2.79114 2.48342 5.04459 3.48457 11 l - - -
2.68618 4.42225 2.77519 2.73123 3.46354 2.40513 3.72494
3.29354 2.67741 2.69355 4.24690 2.90347 2.73739 3.18146 2.89801
2.37887 2.77519 2.98518 4.58477 3.61503
0.00830 5.19127 5.91362 0.61958 0.77255 0.48576 0.95510
11 0.52161 2.42361 5.12258 4.81762 5.19953 3.73976 5.48321
4.61796 4.71452 4.33442 5.15203 4.48032 3.58350 4.89212 4.85250
2.27601 2.99663 3.90976 6.60048 5.48609 12 A - - -
2.68618 4.42225 2.77519 2.73123 3.46354 2.40513 3.72494
3.29354 2.67741 2.69355 4.24690 2.90347 2.73739 3.18146 2.89801
2.37887 2.77519 2.98518 4.58477 3.61503
0.00830 5.19127 5.91362 0.61958 0.77255 0.48576 0.95510

```
     12   3.71274  5.02616  5.44936  4.93762  0.76105  4.81791  4.59898
2.88156  4.74289  2.40595  3.77944  4.82676  5.12489  4.77013  4.73311
4.14864  3.76354  2.86946  4.83436  1.83538     13 f - - -
          2.68618  4.42225  2.77519  2.73123  3.46354  2.40513  3.72494
3.29354  2.67741  2.69355  4.24690  2.90347  2.73739  3.18146  2.89801
2.37887  2.77519  2.98518  4.58477  3.61503
          0.00830  5.19127  5.91362  0.61958  0.77255  0.48576  0.95510
     13   3.39957  4.98651  5.24311  4.74822  1.61526  4.31715  2.65588
3.46134  4.56460  2.29578  4.11664  4.63795  5.03003  4.62591  4.59144
4.01002  3.87358  3.18309  1.15708  2.60763     14 w - - -
          2.68618  4.42225  2.77519  2.73123  3.46354  2.40513  3.72494
3.29354  2.67741  2.69355  4.24690  2.90347  2.73739  3.18146  2.89801
2.37887  2.77519  2.98518  4.58477  3.61503
          0.00830  5.19127  5.91362  0.61958  0.77255  0.48576  0.95510
     14   3.05188  4.31888  5.08463  4.48447  3.42968  3.54183  4.71222
1.05525  4.29199  1.56681  3.50316  4.50481  4.71101  4.43783  4.32668
3.67561  2.96129  2.24779  5.20947  4.03213     15 i - - -
          2.68618  4.42225  2.77519  2.73123  3.46354  2.40513  3.72494
3.29354  2.67741  2.69355  4.24690  2.90347  2.73739  3.18146  2.89801
2.37887  2.77519  2.98518  4.58477  3.61503
          0.00830  5.19127  5.91362  0.61958  0.77255  0.48576  0.95510
     15   4.54639  6.28962  4.42104  4.47111  5.95641  4.71037  5.84743
6.14129  5.01198  5.59371  6.65624  0.10794  5.43895  5.26516  5.28994
4.64164  5.02130  5.59481  7.00122  5.92134     16 N - - -
          2.68618  4.42225  2.77519  2.73123  3.46354  2.40513  3.72494
3.29354  2.67741  2.69355  4.24690  2.90347  2.73739  3.18146  2.89801
2.37887  2.77519  2.98518  4.58477  3.61503
          0.00830  5.19127  5.91362  0.61958  0.77255  0.48576  0.95510
     16   1.82829  4.50436  5.01681  4.41857  3.62971  3.97248  4.67648
1.74890  4.23371  1.56098  3.50255  4.45705  4.68124  4.38944  4.28375
3.63868  2.75496  1.79704  5.19269  4.01232     17 l - - -
          2.68618  4.42225  2.77519  2.73123  3.46354  2.40513  3.72494
3.29354  2.67741  2.69355  4.24690  2.90347  2.73739  3.18146  2.89801
2.37887  2.77519  2.98518  4.58477  3.61503
          0.00830  5.19127  5.91362  0.61958  0.77255  0.48576  0.95510
     17   3.92003  5.26189  5.23549  4.78087  2.60935  4.87063  2.06390
3.27201  4.62829  2.56516  4.39902  4.69103  5.20141  3.48802  4.70437
4.19110  4.14336  3.60902  3.75785  0.73305     18 y - - -
          2.68618  4.42225  2.77519  2.73123  3.46354  2.40513  3.72494
3.29354  2.67741  2.69355  4.24690  2.90347  2.73739  3.18146  2.89801
2.37887  2.77519  2.98518  4.58477  3.61503
          0.00830  5.19127  5.91362  0.61958  0.77255  0.48576  0.95510
     18   3.64834  6.04936  3.11809  2.98651  5.31960  4.03287  3.16938
4.93635  3.18288  4.40428  5.25713  0.54933  4.60493  2.84879  3.57420
3.57418  3.65453  4.52762  6.44878  5.02408     19 N - - -
          2.68618  4.42225  2.77519  2.73123  3.46354  2.40513  3.72494
3.29354  2.67741  2.69355  4.24690  2.90347  2.73739  3.18146  2.89801
2.37887  2.77519  2.98518  4.58477  3.61503
          0.00830  5.19127  5.91362  0.61958  0.77255  0.48576  0.95510
     19   1.23964  4.10378  4.79995  4.19593  3.28605  3.87000  4.48172
2.31102  4.01725  2.03644  3.35812  4.26146  4.52242  4.18040  4.08581
2.83342  2.83141  2.21320  5.02986  3.57594     20 a - - -
          2.68618  4.42225  2.77519  2.73123  3.46354  2.40513  3.72494
3.29354  2.67741  2.69355  4.24690  2.90347  2.73739  3.18146  2.89801
2.37887  2.77519  2.98518  4.58477  3.61503
          0.00830  5.19127  5.91362  0.61958  0.77255  0.48576  0.95510
```

```
     20   2.75558  3.11472  4.96548  4.35049  2.36102  3.97790  4.40027
2.38726  4.03992  1.46569  2.00859  4.26453  4.53954  4.26868  4.14650
3.23334  2.91429  2.61754  4.13475  3.35100     21 l - - -
          2.68618  4.42225  2.77519  2.73123  3.46354  2.40513  3.72494
3.29354  2.67741  2.69355  4.24690  2.90347  2.73739  3.18146  2.89801
2.37887  2.77519  2.98518  4.58477  3.61503
          0.00830  5.19127  5.91362  0.61958  0.77255  0.48576  0.95510
     21   2.57906  4.00568  4.93942  4.32629  2.90443  4.17327  4.50524
1.85685  4.11817  2.14609  2.50715  4.01418  4.53724  3.70236  4.13754
3.41333  2.12602  1.76490  5.00835  3.38316     22 v - - -
          2.68618  4.42225  2.77519  2.73123  3.46354  2.40513  3.72494
3.29354  2.67741  2.69355  4.24690  2.90347  2.73739  3.18146  2.89801
2.37887  2.77519  2.98518  4.58477  3.61503
          0.00830  5.19127  5.91362  0.61958  0.77255  0.48576  0.95510
     22   2.61386  4.39279  4.92966  4.31759  2.92855  3.63050  4.50773
1.90707  3.91168  1.62958  1.88720  4.32374  4.54003  3.78026  4.02327
3.48584  3.24357  2.08490  5.01438  3.83770     23 l - - -
          2.68618  4.42225  2.77519  2.73123  3.46354  2.40513  3.72494
3.29354  2.67741  2.69355  4.24690  2.90347  2.73739  3.18146  2.89801
2.37887  2.77519  2.98518  4.58477  3.61503
          0.00830  5.19127  5.91362  0.61958  0.77255  0.48576  0.95510
     23   2.86210  5.46617  2.92385  2.47240  4.80709  3.74078  1.42328
4.28892  2.25382  3.28245  4.50841  2.86757  4.13499  2.36937  2.94407
2.81105  3.19912  3.85469  5.90029  4.10273     24 h - - -
          2.68618  4.42225  2.77519  2.73123  3.46354  2.40513  3.72494
3.29354  2.67741  2.69355  4.24690  2.90347  2.73739  3.18146  2.89801
2.37887  2.77519  2.98518  4.58477  3.61503
          0.00830  5.19127  5.91362  0.61958  0.77255  0.48576  0.95510
     24   1.59576  5.12728  3.39707  2.69201  4.35369  2.79432  3.66565
3.47890  2.31439  2.24191  3.38792  3.29852  4.19959  2.78055  3.26051
2.77888  2.97338  3.26261  5.63829  4.31301     25 a - - -
          2.68618  4.42225  2.77519  2.73123  3.46354  2.40513  3.72494
3.29354  2.67741  2.69355  4.24690  2.90347  2.73739  3.18146  2.89801
2.37887  2.77519  2.98518  4.58477  3.61503
          0.00830  5.19127  5.91362  0.61958  0.77255  0.48576  0.95510
     25   3.00722  3.34716  4.96792  4.35278  2.35729  4.17618  3.74137
1.68544  4.13827  2.15612  3.35325  4.02199  4.53990  4.05970  4.14745
3.48734  2.70618  2.06725  3.35674  2.41533     26 i - - -
          2.68618  4.42225  2.77519  2.73123  3.46354  2.40513  3.72494
3.29354  2.67741  2.69355  4.24690  2.90347  2.73739  3.18146  2.89801
2.37887  2.77519  2.98518  4.58477  3.61503
          0.00830  5.19127  5.91362  0.61958  0.77255  0.48576  0.95510
     26   2.63039  3.97523  4.71089  4.17466  3.19292  4.18026  4.51316
1.82567  4.12783  1.23945  2.85095  4.33378  4.54381  4.26437  3.98670
3.26323  3.06538  2.09170  5.01458  3.83797     27 l - - -
          2.68567  4.42304  2.77565  2.73083  3.46420  2.40543  3.72519
3.29375  2.67615  2.69382  4.24618  2.90354  2.73745  3.18147  2.89757
2.37945  2.77544  2.98535  4.58521  3.61405
          0.67675  1.83928  1.10021  2.18548  0.11926  0.48576  0.95510
     27   2.07140  4.63979  2.47911  1.87767  4.58485  3.57538  3.76453
3.99796  2.31478  3.40688  4.30445  3.02278  3.96844  2.55186  2.85309
2.58218  2.89428  2.98609  5.45780  4.30375     40 e - - -
          2.68618  4.42225  2.77519  2.73123  3.46354  2.40513  3.72494
3.29354  2.67741  2.69355  4.24690  2.90347  2.73739  3.18146  2.89801
2.37887  2.77519  2.98518  4.58477  3.61503
          0.09353  4.79065  2.51347  0.61958  0.77255  1.24048  0.34143
```

28 2.66927 4.39267 3.68356 3.11514 2.92718 3.38892 2.95956
2.89922 2.78033 2.42469 3.47062 2.93577 4.13776 3.10876 3.41022
3.00644 2.97649 2.53359 4.68247 1.98635 41 y - - -
2.68618 4.42225 2.77519 2.73123 3.46354 2.40513 3.72494
3.29354 2.67741 2.69355 4.24690 2.90347 2.73739 3.18146 2.89801
2.37887 2.77519 2.98518 4.58477 3.61503
0.03585 4.71058 3.64146 0.61958 0.77255 1.31998 0.31080
29 2.82012 5.00775 2.97437 2.62217 4.30846 1.34779 3.88120
3.72707 2.57757 3.33009 4.14828 3.06934 3.46138 3.03904 3.04288
2.75336 3.07251 2.76782 5.57161 4.23660 42 g - - -
2.68640 4.42267 2.77505 2.73125 3.46234 2.40541 3.72536
3.29330 2.67776 2.69280 4.24732 2.90377 2.73745 3.18165 2.89807
2.37876 2.77477 2.98533 4.58519 3.61545
0.31757 1.41677 3.52047 1.65175 0.21284 1.33980 0.30367
30 2.44960 4.12290 3.66329 3.44217 2.54839 3.82706 3.90350
1.88126 3.37983 2.16920 3.28960 3.14059 3.57427 3.57402 3.42683
2.93323 2.82287 2.26957 4.88337 3.63908 56 i - - -
2.68618 4.42225 2.77519 2.73123 3.46354 2.40513 3.72494
3.29354 2.67741 2.69355 4.24690 2.90347 2.73739 3.18146 2.89801
2.37887 2.77519 2.98518 4.58477 3.61503
0.01412 4.66305 5.38540 0.61958 0.77255 1.21386 0.35247
31 2.73234 4.61834 3.47096 2.80168 3.20625 3.69985 3.93542
2.99841 2.87699 2.76729 3.71424 3.29436 1.71900 2.78385 3.08773
2.62711 2.92297 2.76426 4.20792 3.59663 57 p - - -
2.68618 4.42225 2.77519 2.73123 3.46354 2.40513 3.72494
3.29354 2.67741 2.69355 4.24690 2.90347 2.73739 3.18146 2.89801
2.37887 2.77519 2.98518 4.58477 3.61503
0.01346 4.71058 5.43292 0.61958 0.77255 1.31998 0.31080
32 2.51390 5.09758 2.72991 2.09133 4.26203 3.14671 3.32853
3.99831 2.10929 3.39070 3.75260 2.91713 3.74456 2.60177 2.57087
2.30672 2.79945 3.55425 5.69681 4.28993 58 e - - -
2.68618 4.42225 2.77519 2.73123 3.46354 2.40513 3.72494
3.29354 2.67741 2.69355 4.24690 2.90347 2.73739 3.18146 2.89801
2.37887 2.77519 2.98518 4.58477 3.61503
0.11720 4.71058 2.28681 0.61958 0.77255 0.62720 0.76375
33 2.83427 5.33954 2.32592 2.36943 4.68190 2.59102 3.46014
4.16629 2.34555 3.64364 4.31064 2.40356 4.00098 2.77082 2.92807
2.01967 2.56849 3.72876 5.77364 4.05073 59 s - - -
2.68618 4.42225 2.77519 2.73123 3.46354 2.40513 3.72494
3.29354 2.67741 2.69355 4.24690 2.90347 2.73739 3.18146 2.89801
2.37887 2.77519 2.98518 4.58477 3.61503
0.01126 4.88787 5.61022 0.61958 0.77255 0.51845 0.90496
34 2.67974 5.21318 2.65786 2.40557 4.34654 3.62159 3.04112
3.38465 2.48144 3.25369 4.09458 2.20927 3.38584 2.50310 2.62779
2.44010 2.90005 3.23091 5.83015 3.76996 60 n - - -
2.68771 4.42083 2.77507 2.73274 3.46241 2.40581 3.72615
3.28821 2.67661 2.69316 4.24831 2.90291 2.73623 3.18246 2.89932
2.37839 2.77589 2.98530 4.58558 3.61518
0.57591 0.94721 2.99602 2.70050 0.06953 0.80875 0.58953
35 2.40201 4.51847 3.41307 2.62952 2.88747 3.88922 3.91904
2.69762 3.29531 2.28354 3.18526 3.58058 4.10748 3.53126 3.38581
3.00228 3.10504 2.14980 2.21539 3.80716 94 v - - -
2.68618 4.42225 2.77519 2.73123 3.46354 2.40513 3.72494
3.29354 2.67741 2.69355 4.24690 2.90347 2.73739 3.18146 2.89801
2.37887 2.77519 2.98518 4.58477 3.61503
0.23292 5.02479 1.60340 0.61958 0.77255 0.90685 0.51717

36 2.78382 5.28078 2.70171 2.26845 4.61377 3.44361 3.76610
3.95947 1.75642 2.86491 3.25303 2.86386 3.97117 2.51084 2.68145
2.42356 2.99968 3.52080 5.72061 4.31739 95 k - - -
2.68618 4.42225 2.77519 2.73123 3.46354 2.40513 3.72494
3.29354 2.67741 2.69355 4.24690 2.90347 2.73739 3.18146 2.89801
2.37887 2.77519 2.98518 4.58477 3.61503
0.01225 4.80411 5.52646 0.61958 0.77255 1.22580 0.34747
37 2.72162 3.79259 2.96313 2.30102 4.48190 3.47638 3.78740
3.67080 2.17247 2.75365 4.15396 2.91283 3.67526 2.81141 1.92155
2.59493 2.86354 3.23251 5.64836 4.17458 96 r - - -
2.68618 4.42225 2.77519 2.73123 3.46354 2.40513 3.72494
3.29354 2.67741 2.69355 4.24690 2.90347 2.73739 3.18146 2.89801
2.37887 2.77519 2.98518 4.58477 3.61503
0.01225 4.80411 5.52646 0.61958 0.77255 1.22580 0.34747
38 2.53816 4.57593 3.61185 2.95791 3.07629 3.76699 3.60683
2.98589 2.61937 2.23075 3.33993 3.26436 4.05664 2.82802 2.72407
2.98302 2.36773 2.45588 4.52848 3.03742 97 l - - -
2.68618 4.42225 2.77519 2.73123 3.46354 2.40513 3.72494
3.29354 2.67741 2.69355 4.24690 2.90347 2.73739 3.18146 2.89801
2.37887 2.77519 2.98518 4.58477 3.61503
0.03202 4.80411 3.75864 0.61958 0.77255 1.22580 0.34747
39 2.28665 4.99684 3.03052 2.51721 3.99321 3.39709 3.82890
3.06483 2.37726 2.72257 3.76731 3.04886 3.83806 2.95327 2.82427
2.04831 2.44987 3.19769 5.49913 3.90927 98 s - - -
2.68618 4.42225 2.77519 2.73123 3.46354 2.40513 3.72494
3.29354 2.67741 2.69355 4.24690 2.90347 2.73739 3.18146 2.89801
2.37887 2.77519 2.98518 4.58477 3.61503
0.01550 4.78459 4.95910 0.61958 0.77255 1.17663 0.36862
40 2.73474 4.27157 4.23885 3.61595 2.28430 2.97998 3.92535
2.57749 3.44319 1.52126 3.19716 3.92932 4.29242 3.55539 3.49736
2.89777 3.00584 2.33612 4.07772 3.51560 99 l - - -
2.68618 4.42225 2.77519 2.73123 3.46354 2.40513 3.72494
3.29354 2.67741 2.69355 4.24690 2.90347 2.73739 3.18146 2.89801
2.37887 2.77519 2.98518 4.58477 3.61503
0.01229 4.80121 5.52355 0.61958 0.77255 1.21812 0.35067
41 2.81381 4.23371 4.51831 3.91915 1.77061 3.95204 4.27061
2.45456 3.27323 1.89565 2.76131 3.84669 4.31846 3.73839 3.24023
2.98310 3.04592 2.32418 4.39807 3.03325 100 f - - -
2.68626 4.42233 2.77522 2.73126 3.46306 2.40521 3.72503
3.29362 2.67749 2.69328 4.24574 2.90355 2.73748 3.18154 2.89809
2.37890 2.77528 2.98526 4.58485 3.61467
0.29340 1.38510 5.52646 0.12781 2.12046 0.39567 1.11849
42 2.73764 5.22876 2.41090 2.40354 4.76707 3.12493 3.09193
3.18669 2.23285 3.06164 4.22647 2.39731 3.94077 2.38666 2.74822
2.51930 2.83941 3.81353 5.85775 4.44795 102 k - - -
2.68618 4.42225 2.77519 2.73123 3.46354 2.40513 3.72494
3.29354 2.67741 2.69355 4.24690 2.90347 2.73739 3.18146 2.89801
2.37887 2.77519 2.98518 4.58477 3.61503
0.00911 5.09926 5.82160 0.61958 0.77255 0.74824 0.64093
43 2.66002 5.42117 2.69189 2.33503 4.45360 3.51703 3.28356
3.89375 1.49153 3.18640 4.13957 3.03454 4.08621 2.61278 2.65186
2.61534 2.87062 3.71178 5.85570 4.28656 103 k - - -
2.68618 4.42225 2.77520 2.73124 3.46354 2.40513 3.72495
3.29354 2.67741 2.69355 4.24690 2.90347 2.73740 3.18147 2.89801
2.37887 2.77511 2.98519 4.58477 3.61503
0.02092 4.03222 5.82160 0.49444 0.94139 0.35090 1.21758

```
     44   1.71888  5.10870  3.18907  2.68685  2.79343  3.61845  3.84998
2.85860  2.27634  3.23550  4.18439  3.08458  3.36741  3.16599  2.94294
2.78470  3.03097  2.72482  5.62340  3.60483    105 a - - -
          2.68618  4.42225  2.77519  2.73123  3.46354  2.40513  3.72494
3.29354  2.67741  2.69355  4.24690  2.90347  2.73739  3.18146  2.89801
2.37887  2.77519  2.98518  4.58477  3.61503
          0.00830  5.19127  5.91362  0.61958  0.77255  0.48576  0.95510
     45   2.20631  4.28962  2.85513  2.71040  3.00950  3.38863  3.70121
3.21869  2.47000  3.14919  3.18645  3.19590  4.20729  2.82830  2.88133
2.25306  2.68266  3.01449  4.40586  3.51196    106 a - - -
          2.68618  4.42225  2.77519  2.73123  3.46354  2.40513  3.72494
3.29354  2.67741  2.69355  4.24690  2.90347  2.73739  3.18146  2.89801
2.37887  2.77519  2.98518  4.58477  3.61503
          0.00830  5.19127  5.91362  0.61958  0.77255  0.48576  0.95510
     46   2.95274  3.07968  4.89263  3.17312  2.75413  3.55843  4.22094
2.16653  4.08432  2.19328  3.44734  4.30375  4.53156  4.23010  4.11986
3.31135  3.18050  2.40676  3.93911  1.47776    107 y - - -
          2.68618  4.42225  2.77519  2.73123  3.46354  2.40513  3.72494
3.29354  2.67741  2.69355  4.24690  2.90347  2.73739  3.18146  2.89801
2.37887  2.77519  2.98518  4.58477  3.61503
          0.00830  5.19127  5.91362  0.61958  0.77255  0.48576  0.95510
     47   2.87637  3.97784  2.89647  2.44605  4.63701  3.74083  3.69231
3.66515  2.30857  3.24566  4.29011  2.15610  3.54464  2.91971  2.30636
2.48031  2.27850  3.10544  5.87091  4.19380    108 n - - -
          2.68618  4.42225  2.77519  2.73123  3.46354  2.40513  3.72494
3.29354  2.67741  2.69355  4.24690  2.90347  2.73739  3.18146  2.89801
2.37887  2.77519  2.98518  4.58477  3.61503
          0.00830  5.19127  5.91362  0.61958  0.77255  0.48576  0.95510
     48   3.76145  4.99629  5.82623  5.31056  3.87395  5.25249  5.80867
0.98933  4.37085  2.24699  4.07212  5.40788  5.50779  5.39483  5.29823
4.63850  4.01072  0.99117  6.18484  4.99196    109 i - - -
          2.68618  4.42225  2.77519  2.73123  3.46354  2.40513  3.72494
3.29354  2.67741  2.69355  4.24690  2.90347  2.73739  3.18146  2.89801
2.37887  2.77519  2.98518  4.58477  3.61503
          0.01855  5.19127  4.35727  0.61958  0.77255  0.48576  0.95510
     49   2.54194  4.87379  3.27081  2.71782  3.89617  1.14464  4.02161
3.76890  2.81346  3.04576  4.11547  3.09972  4.20016  3.07658  3.29356
2.61939  3.18037  3.45649  5.64350  4.31831    110 g - - -
          2.68611  4.42256  2.77468  2.73154  3.46138  2.40543  3.72525
3.29385  2.67772  2.69348  4.24541  2.90377  2.73770  3.18177  2.89785
2.37918  2.77509  2.98549  4.58004  3.61534
          0.06233  2.85252  5.90346  2.10265  0.13026  0.51970  0.90312
     50   3.28864  5.81896  2.54196  2.70385  5.13776  0.88467  3.60685
4.63198  3.02057  4.11360  4.89005  2.77909  4.34545  2.60575  3.32484
3.08707  3.49732  4.20017  6.25649  4.81407    123 g - - -
          2.68618  4.42225  2.77519  2.73123  3.46354  2.40513  3.72494
3.29354  2.67741  2.69355  4.24690  2.90347  2.73739  3.18146  2.89801
2.37887  2.77519  2.98518  4.58477  3.61503
          0.00839  5.18111  5.90346  0.61958  0.77255  0.51970  0.90312
     51   2.92404  3.26852  2.84744  2.34235  4.69815  3.02278  2.86392
3.75752  2.29684  2.56801  3.57352  2.97826  4.13600  2.31663  2.67114
2.88025  3.01520  3.23258  5.83840  3.55889    124 k - - -
          2.68622  4.42229  2.77523  2.73127  3.46357  2.40475  3.72498
3.29358  2.67744  2.69358  4.24693  2.90350  2.73743  3.18150  2.89804
2.37890  2.77523  2.98522  4.58480  3.61507
          0.08757  2.51187  5.90346  0.22465  1.60345  0.51970  0.90312
```

52 2.62651 4.76005 2.91542 2.47724 4.25317 3.68266 3.92811
3.77312 2.00866 3.15576 4.10921 2.95766 3.56547 2.71304 2.66731
1.96408 2.45033 3.08379 5.59983 4.29141 126 s - - -
2.68618 4.42225 2.77519 2.73123 3.46354 2.40513 3.72494
3.29354 2.67741 2.69355 4.24690 2.90347 2.73739 3.18146 2.89801
2.37887 2.77519 2.98518 4.58477 3.61503
0.00839 5.18111 5.90346 0.61958 0.77255 0.51970 0.90312
53 2.95589 4.27399 4.86689 3.16180 2.04711 4.15905 4.27280
2.43898 3.99299 1.69974 3.15386 4.28891 4.52371 4.21362 3.51461
3.46603 2.69037 2.27624 4.52389 2.18499 127 l - - -
2.68618 4.42225 2.77519 2.73123 3.46354 2.40513 3.72494
3.29354 2.67741 2.69355 4.24690 2.90347 2.73739 3.18146 2.89801
2.37887 2.77519 2.98518 4.58477 3.61503
0.00839 5.18111 5.90346 0.61958 0.77255 0.51970 0.90312
54 2.85206 5.07555 3.22319 2.84420 4.59949 3.77047 3.95724
3.98168 2.84980 3.61009 4.40189 2.13829 4.20445 3.19941 3.31901
1.26179 2.09875 3.21680 5.82176 4.47170 128 s - - -
2.68618 4.42225 2.77519 2.73123 3.46354 2.40513 3.72494
3.29354 2.67741 2.69355 4.24690 2.90347 2.73739 3.18146 2.89801
2.37887 2.77519 2.98518 4.58477 3.61503
0.00839 5.18111 5.90346 0.61958 0.77255 0.51970 0.90312
55 1.78320 4.10265 4.87817 4.27281 3.35007 4.20129 4.53110
2.60216 4.08704 1.09365 2.72046 4.32136 3.78171 4.23954 4.14082
3.43909 3.27514 2.55103 5.05801 3.88256 129 l - - -
2.68618 4.42225 2.77519 2.73123 3.46354 2.40513 3.72494
3.29354 2.67741 2.69355 4.24690 2.90347 2.73739 3.18146 2.89801
2.37887 2.77519 2.98518 4.58477 3.61503
0.00839 5.18111 5.90346 0.61958 0.77255 0.51970 0.90312
56 2.24949 5.28815 2.03748 2.46979 4.76628 3.41997 3.02954
4.01990 2.61604 2.89219 4.23284 2.18798 4.12709 2.73358 3.02929
2.48824 3.10462 3.77951 4.62805 3.82368 130 d - - -
2.68618 4.42225 2.77519 2.73123 3.46354 2.40513 3.72494
3.29354 2.67741 2.69355 4.24690 2.90347 2.73739 3.18146 2.89801
2.37887 2.77519 2.98518 4.58477 3.61503
0.00839 5.18111 5.90346 0.61958 0.77255 0.51970 0.90312
57 2.22121 5.04454 1.64907 2.24624 4.51164 3.59298 3.81564
3.75546 2.67494 3.59173 3.13729 2.97312 4.13870 2.92424 2.90622
2.77920 2.62056 3.42889 4.94633 4.31066 131 d - - -
2.68618 4.42225 2.77519 2.73123 3.46354 2.40513 3.72494
3.29354 2.67741 2.69355 4.24690 2.90347 2.73739 3.18146 2.89801
2.37887 2.77519 2.98518 4.58477 3.61503
0.00839 5.18111 5.90346 0.61958 0.77255 0.51970 0.90312
58 3.91263 5.14467 5.94449 5.40400 3.99463 5.36035 5.81414
0.71853 5.27851 1.47549 3.70752 5.51922 5.54747 5.34771 5.31473
4.74387 4.14802 1.99786 6.04392 4.19256 132 i - - -
2.68618 4.42225 2.77519 2.73123 3.46354 2.40513 3.72494
3.29354 2.67741 2.69355 4.24690 2.90347 2.73739 3.18146 2.89801
2.37887 2.77519 2.98518 4.58477 3.61503
0.00839 5.18111 5.90346 0.61958 0.77255 0.51970 0.90312
59 2.91511 5.44399 3.21514 1.26837 4.53531 3.30475 3.69367
3.98870 2.31350 3.49324 4.20535 3.17507 4.12732 2.14655 2.79726
2.85213 3.18810 3.82965 5.88193 3.66082 133 e - - -
2.68627 4.42234 2.77529 2.73112 3.46319 2.40522 3.72504
3.29363 2.67750 2.69344 4.24699 2.90356 2.73685 3.18156 2.89810
2.37896 2.77529 2.98501 4.58486 3.61513
0.03682 3.39861 5.90346 1.45257 0.26653 0.51970 0.90312

```
     60   2.95804  4.93118  3.21362  2.70751  3.56983  2.77171  1.68781
4.04548  2.65332  3.61736  4.20562  2.26265  4.14624  2.51796  3.06137
2.61388  2.96649  3.69697  5.79948  3.55023    141 h - - -
          2.68618  4.42225  2.77519  2.73123  3.46354  2.40513  3.72494
3.29354  2.67741  2.69355  4.24690  2.90347  2.73739  3.18146  2.89801
2.37887  2.77519  2.98518  4.58477  3.61503
          0.00839  5.18111  5.90346  0.61958  0.77255  0.51970  0.90312
     61   2.87934  4.23593  2.97702  2.41359  3.39707  1.78029  3.89910
4.03904  2.36894  3.45582  4.29910  2.72701  4.13961  2.98897  2.61661
2.50635  3.03233  3.45302  5.82407  3.19028    142 g - - -
          2.68618  4.42225  2.77519  2.73123  3.46354  2.40513  3.72494
3.29354  2.67741  2.69355  4.24690  2.90347  2.73739  3.18146  2.89801
2.37887  2.77519  2.98518  4.58477  3.61503
          0.00839  5.18111  5.90346  0.61958  0.77255  0.51970  0.90312
     62   3.08762  4.25517  5.04822  4.44713  3.56053  4.33443  4.68273
0.95222  3.64072  1.85205  3.26592  4.47417  4.68725  4.40418  4.08518
3.64963  3.21722  2.08262  5.18586  4.00845    143 i - - -
          2.68618  4.42225  2.77519  2.73123  3.46354  2.40513  3.72494
3.29354  2.67741  2.69355  4.24690  2.90347  2.73739  3.18146  2.89801
2.37887  2.77519  2.98518  4.58477  3.61503
          0.00839  5.18111  5.90346  0.61958  0.77255  0.46511  0.98904
     63   3.79345  5.09266  5.72300  5.12342  3.28752  5.05182  4.43405
1.83417  4.94627  0.57944  3.36826  5.20312  5.26481  4.95187  4.92988
4.39236  4.01133  2.58713  5.61577  4.54103    144 L - - -
          2.68618  4.42225  2.77520  2.73123  3.46354  2.40513  3.72495
3.29354  2.67741  2.69355  4.24690  2.90347  2.73740  3.18147  2.89801
2.37887  2.77513  2.98519  4.58477  3.61503
          0.04093  4.25830  3.65127  0.51422  0.91123  0.48576  0.95510
     64   2.99463  4.05512  3.49343  2.91643  4.42646  2.62141  3.96543
3.74662  2.55042  3.13038  4.26195  3.38788  4.25684  3.09234  1.05194
3.07053  3.27024  3.52077  5.67585  3.68779    146 r - - -
          2.68618  4.42225  2.77519  2.73123  3.46354  2.40513  3.72494
3.29354  2.67741  2.69355  4.24690  2.90347  2.73739  3.18146  2.89801
2.37887  2.77519  2.98518  4.58477  3.61503
          0.00850  5.16774  5.89009  0.61958  0.77255  0.56217  0.84391
     65   2.47447  2.68762  3.16083  2.57065  4.36445  2.42479  3.18354
3.69661  2.24174  3.33181  3.90409  3.10576  2.82486  2.75287  2.72503
2.67060  3.13731  3.47129  5.23868  4.08302    147 k - - -
          2.68618  4.42225  2.77519  2.73123  3.46354  2.40513  3.72494
3.29354  2.67741  2.69355  4.24690  2.90347  2.73739  3.18146  2.89801
2.37887  2.77519  2.98518  4.58477  3.61503
          0.05494  5.16774  3.04156  0.61958  0.77255  0.56217  0.84391
     66   2.92779  5.43293  2.85127  2.40834  4.38019  3.21366  3.78572
3.72227  2.29964  3.73722  4.47420  1.96458  2.98192  2.57854  2.16293
2.75973  2.66552  3.67215  4.88033  4.45745    148 n - - -
          2.68652  4.42067  2.77580  2.73141  3.46363  2.40531  3.72376
3.29342  2.67723  2.69301  4.24748  2.90428  2.73766  3.18107  2.89764
2.37872  2.77523  2.98452  4.58475  3.61576
          0.99184  1.36640  0.98328  1.85588  0.16998  0.65756  0.73005
     67   2.64948  3.86933  3.10154  2.56388  4.19858  2.94733  3.39526
3.58758  2.43186  2.83338  3.99239  2.89353  3.28955  2.73238  2.25505
2.62232  2.47241  3.31257  5.49715  3.42655    166 r - - -
          2.68679  4.42262  2.77487  2.73136  3.46302  2.40597  3.72563
3.29366  2.67753  2.69176  4.24787  2.90458  2.73741  3.18158  2.89675
2.37935  2.77541  2.98516  4.57783  3.61501
          1.09071  0.41633  5.39094  1.78506  0.18367  1.31558  0.31241
```

```
     68   2.62456  4.58858  3.43661  2.67413  2.46283  3.18177  3.51251
3.00136  2.76672  1.93396  3.60019  3.32714  3.40527  3.06838  3.04888
2.67261  2.96372  2.82304  5.00829  3.67283    190 l - - -
          2.68618  4.42225  2.77519  2.73123  3.46354  2.40513  3.72494
3.29354  2.67741  2.69355  4.24690  2.90347  2.73739  3.18146  2.89801
2.37887  2.77519  2.98518  4.58477  3.61503
          0.12744  4.68136  2.20379  0.61958  0.77255  0.73595  0.65210
     69   2.34866  4.17037  3.76551  3.12279  3.37673  3.40984  4.07280
2.90060  2.73150  2.19696  3.02638  3.53945  2.60519  3.29646  3.34092
2.56025  2.67185  2.70017  4.41795  2.86665    191 l - - -
          2.68618  4.42225  2.77519  2.73123  3.46354  2.40513  3.72494
3.29354  2.67741  2.69355  4.24690  2.90347  2.73739  3.18146  2.89801
2.37887  2.77519  2.98518  4.58477  3.61503
          0.02221  4.81793  4.27740  0.61958  0.77255  1.21029  0.35398
     70   2.48511  4.67496  2.88636  2.54853  3.91382  2.86208  3.57391
3.82419  2.42833  3.03407  4.17578  2.97178  2.69600  2.89971  3.05576
2.08710  2.67871  3.46565  5.59426  3.07398    192 s - - -
          2.68618  4.42225  2.77519  2.73123  3.46354  2.40513  3.72494
3.29354  2.67741  2.69355  4.24690  2.90347  2.73739  3.18146  2.89801
2.37887  2.77519  2.98518  4.58477  3.61503
          0.01220  4.80793  5.53027  0.61958  0.77255  1.22156  0.34923
     71   2.50074  5.17623  2.79537  2.52866  3.98134  3.38332  3.62898
3.65145  2.38532  2.91590  3.83118  2.58156  3.58017  2.81425  2.24209
2.40690  2.54142  3.38249  4.81423  4.02640    193 r - - -
          2.68618  4.42225  2.77519  2.73123  3.46354  2.40513  3.72494
3.29354  2.67741  2.69355  4.24690  2.90347  2.73739  3.18146  2.89801
2.37887  2.77519  2.98518  4.58477  3.61503
          0.01220  4.80793  5.53027  0.61958  0.77255  1.22156  0.34923
     72   2.70342  4.17723  2.63384  2.67585  3.81739  3.48229  3.54469
3.16474  2.61530  1.91904  3.49079  3.11036  2.86197  3.03116  3.08696
2.52886  2.91509  3.06533  5.00182  3.91791    194 l - - -
          2.68618  4.42225  2.77519  2.73123  3.46354  2.40513  3.72494
3.29354  2.67741  2.69355  4.24690  2.90347  2.73739  3.18146  2.89801
2.37887  2.77519  2.98518  4.58477  3.61503
          0.01220  4.80793  5.53027  0.61958  0.77255  1.22156  0.34923
     73   2.72651  5.15272  2.62095  2.50869  3.36027  3.24166  3.79711
3.61993  1.83752  3.04357  3.86794  2.89825  3.57333  2.89829  2.65413
2.37270  2.95465  3.32403  4.17489  3.99157    195 k - - -
          2.68618  4.42225  2.77519  2.73123  3.46354  2.40513  3.72494
3.29354  2.67741  2.69355  4.24690  2.90347  2.73739  3.18146  2.89801
2.37887  2.77519  2.98518  4.58477  3.61503
          0.08982  4.80793  2.55440  0.61958  0.77255  1.22156  0.34923
     74   2.63270  5.24414  2.86125  2.40771  3.90126  3.29379  3.70015
3.62112  1.91694  3.34814  4.21958  2.80623  3.46714  2.36118  2.41732
2.41873  2.91879  3.39775  5.39202  4.28569    196 k - - -
          2.68618  4.42225  2.77519  2.73123  3.46354  2.40513  3.72494
3.29354  2.67741  2.69355  4.24690  2.90347  2.73739  3.18146  2.89801
2.37887  2.77519  2.98518  4.58477  3.61503
          0.01318  4.73129  5.45364  0.61958  0.77255  1.30060  0.31796
     75   2.64523  3.75675  3.09899  2.59509  3.53228  3.29912  3.67836
3.27968  1.94157  2.81005  3.99372  3.13166  2.65302  2.89727  2.73703
2.79418  2.94178  3.12952  5.43220  3.25245    197 k - - -
          2.68618  4.42225  2.77519  2.73123  3.46354  2.40513  3.72494
3.29354  2.67741  2.69355  4.24690  2.90347  2.73739  3.18146  2.89801
2.37887  2.77519  2.98518  4.58477  3.61503
          0.06626  4.73129  2.89495  0.61958  0.77255  1.30060  0.31796
```

76 2.18057 4.31846 3.44609 2.98050 2.45519 3.47990 3.93292
2.74613 2.79257 2.20692 3.50302 3.40104 3.95645 3.18559 3.22082
2.76276 2.83068 2.41121 4.21517 3.54308 198 a - - -
2.68632 4.42331 2.77466 2.73131 3.46306 2.40505 3.72487
3.29363 2.67747 2.69372 4.24730 2.90423 2.73727 3.18146 2.89725
2.37857 2.77590 2.98519 4.58511 3.61467
1.27888 0.33249 5.40127 1.74149 0.19269 1.33740 0.30453
77 2.74054 5.21910 1.70516 2.25573 4.33337 3.15307 3.65247
3.90506 2.36511 3.09631 4.16980 2.84273 3.93128 2.75762 2.82669
2.57859 2.81494 3.40670 5.66393 4.26532 220 d - - -
2.68618 4.42225 2.77519 2.73123 3.46354 2.40513 3.72494
3.29354 2.67741 2.69355 4.24690 2.90347 2.73739 3.18146 2.89801
2.37887 2.77519 2.98518 4.58477 3.61503
0.01385 4.68217 5.40451 0.61958 0.77255 1.19009 0.36268
78 2.53111 5.20673 2.72227 2.04623 4.11264 3.12787 3.67286
3.39537 2.32148 2.99033 4.14146 2.71869 2.84554 2.77296 2.83791
2.60592 2.94433 3.14410 5.65837 3.99319 221 e - - -
2.68618 4.42225 2.77519 2.73123 3.46354 2.40513 3.72494
3.29354 2.67741 2.69355 4.24690 2.90347 2.73739 3.18146 2.89801
2.37887 2.77519 2.98518 4.58477 3.61503
0.01318 4.73129 5.45364 0.61958 0.77255 1.30060 0.31796
79 2.66386 5.17640 2.82369 2.41429 4.47919 3.30277 3.46037
3.87832 2.35800 3.40393 3.44397 3.05100 3.32393 2.65544 1.91106
2.58159 2.95167 3.43354 3.34140 3.97605 222 r - - -
2.68618 4.42225 2.77519 2.73123 3.46354 2.40513 3.72494
3.29354 2.67741 2.69355 4.24690 2.90347 2.73739 3.18146 2.89801
2.37887 2.77519 2.98518 4.58477 3.61503
0.01318 4.73129 5.45364 0.61958 0.77255 1.05449 0.42828
80 2.70782 4.95088 3.16067 2.55990 4.15112 3.44552 3.35238
3.65708 2.25897 2.66995 4.07484 2.96367 3.96473 2.47020 2.26967
2.38931 2.98633 3.30337 3.09135 3.70918 223 k - - -
2.68618 4.42225 2.77519 2.73123 3.46354 2.40513 3.72494
3.29354 2.67741 2.69355 4.24690 2.90347 2.73739 3.18146 2.89801
2.37887 2.77519 2.98518 4.58477 3.61503
0.01220 4.80793 5.53027 0.61958 0.77255 1.22156 0.34923
81 2.12648 5.23504 2.83638 2.43218 4.54938 3.40530 3.71972
3.69106 2.01787 2.87630 4.28390 3.01655 3.27633 2.45804 2.86351
2.71194 2.62226 3.42451 5.68655 3.55794 224 k - - -
2.68618 4.42225 2.77519 2.73123 3.46354 2.40513 3.72494
3.29354 2.67741 2.69355 4.24690 2.90347 2.73739 3.18146 2.89801
2.37887 2.77519 2.98518 4.58477 3.61503
0.01220 4.80793 5.53027 0.61958 0.77255 1.22156 0.34923
82 2.77479 4.90902 3.04900 2.44033 2.72463 3.28598 3.51276
3.42002 2.61242 2.72734 3.98738 3.09468 4.04658 2.63840 2.80606
2.77782 3.03547 2.99745 5.11899 2.18828 225 y - - -
2.68618 4.42225 2.77519 2.73123 3.46354 2.40513 3.72494
3.29354 2.67741 2.69355 4.24690 2.90347 2.73739 3.18146 2.89801
2.37887 2.77519 2.98518 4.58477 3.61503
0.01220 4.80793 5.53027 0.61958 0.77255 1.22156 0.34923
83 1.84364 3.44847 4.11664 3.67949 2.64927 2.62813 4.21656
2.61809 3.56146 2.19434 3.33788 3.89136 4.28416 3.78300 3.40136
2.65246 3.02026 2.28999 4.90455 3.11790 226 a - - -
2.68618 4.42225 2.77519 2.73123 3.46354 2.40513 3.72494
3.29354 2.67741 2.69355 4.24690 2.90347 2.73739 3.18146 2.89801
2.37887 2.77519 2.98518 4.58477 3.61503
0.01220 4.80793 5.53027 0.61958 0.77255 1.18424 0.36525

```
     84   2.33131  3.78331  3.29892  3.68101  3.33614  3.92819  4.15080
1.86867  3.60809  1.69025  3.25148  3.72395  3.89141  3.76273  3.77493
3.09761  2.94081  2.10672  4.89754  3.70739    227 l - - -
          2.68618  4.42225  2.77519  2.73123  3.46354  2.40513  3.72494
3.29354  2.67741  2.69355  4.24690  2.90347  2.73739  3.18146  2.89801
2.37887  2.77519  2.98518  4.58477  3.61503
          0.02252  4.81793  4.25546  0.61958  0.77255  1.02284  0.44562
     85   2.67769  5.31455  2.77119  1.96631  4.65278  3.18118  3.45492
4.08669  2.30013  2.62722  4.29519  2.97498  2.95312  2.58529  2.82380
2.39474  2.82538  3.65994  5.75133  3.87235    228 e - - -
          2.68618  4.42225  2.77519  2.73123  3.46354  2.40513  3.72494
3.29354  2.67741  2.69355  4.24690  2.90347  2.73739  3.18146  2.89801
2.37887  2.77519  2.98518  4.58477  3.61503
          0.01157  4.86071  5.58305  0.61958  0.77255  0.21842  1.62857
     86   2.79942  4.89939  3.26929  2.68738  2.87291  3.78403  3.34575
3.58084  2.54942  3.08641  3.45902  3.22230  2.99992  2.31382  3.14666
2.59406  2.66826  3.18959  3.25327  2.62348    229 q - - -
          2.68618  4.42225  2.77519  2.73123  3.46354  2.40513  3.72494
3.29354  2.67741  2.69355  4.24690  2.90347  2.73739  3.18146  2.89801
2.37887  2.77519  2.98518  4.58477  3.61503
          0.00830  5.19127  5.91362  0.61958  0.77255  0.48576  0.95510
     87   2.46335  4.56176  2.80026  2.64483  3.91248  2.84928  3.67582
4.20447  2.37500  3.21578  4.00273  2.77865  2.15442  2.66373  2.84108
2.49141  2.92792  3.07374  5.86035  4.46406    230 p - - -
          2.68618  4.42225  2.77519  2.73123  3.46354  2.40513  3.72494
3.29354  2.67741  2.69355  4.24690  2.90347  2.73739  3.18146  2.89801
2.37887  2.77519  2.98518  4.58477  3.61503
          0.00830  5.19127  5.91362  0.61958  0.77255  0.48576  0.95510
     88   3.63972  6.31112  0.92615  1.23783  4.91134  3.93296  4.44766
5.14311  3.44980  4.59335  5.41624  2.88405  4.54137  3.22766  4.06205
3.51036  3.66096  4.67929  6.73308  5.19818    231 d - - -
          2.68618  4.42225  2.77519  2.73123  3.46354  2.40513  3.72494
3.29354  2.67741  2.69355  4.24690  2.90347  2.73739  3.18146  2.89801
2.37887  2.77519  2.98518  4.58477  3.61503
          0.00830  5.19127  5.91362  0.61958  0.77255  0.48576  0.95510
     89   2.24787  4.67852  3.62407  2.93731  3.79257  3.89198  4.12821
3.44183  2.87244  2.73324  3.98734  3.44699  1.15507  3.36757  3.41130
2.91662  3.18156  3.18599  5.44911  3.98459    232 p - - -
          2.68618  4.42225  2.77519  2.73123  3.46354  2.40513  3.72494
3.29354  2.67741  2.69355  4.24690  2.90347  2.73739  3.18146  2.89801
2.37887  2.77519  2.98518  4.58477  3.61503
          0.00830  5.19127  5.91362  0.61958  0.77255  0.48576  0.95510
     90   3.02406  5.17259  3.56369  2.93408  4.15554  3.84298  3.82352
3.79630  2.73535  1.96377  4.19072  2.87262  4.29999  3.22904  1.13573
3.04153  3.30712  3.46921  5.66633  4.37576    233 r - - -
          2.68619  4.42226  2.77520  2.73124  3.46355  2.40513  3.72495
3.29355  2.67741  2.69356  4.24690  2.90339  2.73740  3.18147  2.89801
2.37883  2.77520  2.98519  4.58478  3.61504
          0.02544  3.79784  5.91362  0.44226  1.02886  0.48576  0.95510
     91   3.25631  4.77049  5.46982  4.89941  3.65688  4.78141  5.19845
1.11068  4.73297  1.68756  3.82999  4.93818  5.09413  4.88406  4.77813
4.12283  3.26609  1.37248  5.64160  4.12677    235 i - - -
          2.68618  4.42225  2.77519  2.73123  3.46354  2.40513  3.72494
3.29354  2.67741  2.69355  4.24690  2.90347  2.73739  3.18146  2.89801
2.37887  2.77519  2.98518  4.58477  3.61503
          0.00830  5.19127  5.91362  0.61958  0.77255  0.48576  0.95510
```

92 2.86979 3.61075 4.53420 3.94384 3.51733 3.95397 1.34649
2.74267 3.81256 1.98444 2.99902 3.78655 4.48370 4.02115 3.83967
3.29725 2.82967 2.60373 5.07338 2.92469 236 h - - -
2.68618 4.42225 2.77519 2.73123 3.46354 2.40513 3.72494
3.29354 2.67741 2.69355 4.24690 2.90347 2.73739 3.18146 2.89801
2.37887 2.77519 2.98518 4.58477 3.61503
0.00830 5.19127 5.91362 0.61958 0.77255 0.48576 0.95510
93 2.80966 3.95825 4.93477 4.32811 0.91825 4.02600 4.52302
2.66841 4.13007 2.46106 3.08967 4.34572 4.57307 4.27139 4.16389
3.52103 3.28242 2.58733 5.02214 2.91223 237 f - - -
2.68618 4.42225 2.77519 2.73123 3.46354 2.40513 3.72494
3.29354 2.67741 2.69355 4.24690 2.90347 2.73739 3.18146 2.89801
2.37887 2.77519 2.98518 4.58477 3.61503
0.00830 5.19127 5.91362 0.61958 0.77255 0.48576 0.95510
94 0.93748 3.72787 4.60167 4.03741 3.73672 2.86967 4.55879
2.92368 3.91363 2.17441 3.74174 4.18321 4.51720 4.12770 4.08239
3.35592 3.12070 2.36543 5.24925 4.06116 238 a - - -
2.68618 4.42225 2.77519 2.73123 3.46354 2.40513 3.72494
3.29354 2.67741 2.69355 4.24690 2.90347 2.73739 3.18146 2.89801
2.37887 2.77519 2.98518 4.58477 3.61503
0.00830 5.19127 5.91362 0.61958 0.77255 0.48576 0.95510
95 3.70973 5.00908 5.68378 5.08671 2.57503 4.98057 5.31716
2.11296 4.90756 0.72301 3.20078 5.14474 5.21720 4.93547 4.89326
4.32110 3.25168 2.08199 5.61132 4.53086 239 l - - -
2.68618 4.42225 2.77519 2.73123 3.46354 2.40513 3.72494
3.29354 2.67741 2.69355 4.24690 2.90347 2.73739 3.18146 2.89801
2.37887 2.77519 2.98518 4.58477 3.61503
0.00830 5.19127 5.91362 0.61958 0.77255 0.48576 0.95510
96 2.80734 2.29038 3.75753 3.36581 2.98547 3.98565 4.24587
3.09955 2.79095 2.87782 3.70316 2.01936 4.36385 3.60685 3.64914
2.52466 2.83890 1.92733 5.25672 3.82444 240 v - - -
2.68618 4.42225 2.77519 2.73123 3.46354 2.40513 3.72494
3.29354 2.67741 2.69355 4.24690 2.90347 2.73739 3.18146 2.89801
2.37887 2.77519 2.98518 4.58477 3.61503
0.00830 5.19127 5.91362 0.61958 0.77255 0.48576 0.95510
97 2.82421 1.57980 3.19222 2.52280 3.38447 3.71244 4.00164
3.77900 2.74605 3.43205 3.99282 2.90028 4.18791 2.91232 2.96694
2.14946 2.85516 3.50561 5.67488 4.17524 241 c - - -
2.68618 4.42225 2.77519 2.73123 3.46354 2.40513 3.72494
3.29354 2.67741 2.69355 4.24690 2.90347 2.73739 3.18146 2.89801
2.37887 2.77519 2.98518 4.58477 3.61503
0.00830 5.19127 5.91362 0.61958 0.77255 0.48576 0.95510
98 1.81773 3.80581 3.72428 2.62801 3.82830 1.29800 4.18352
3.37454 3.13400 3.07037 3.72939 3.60562 4.30943 3.45342 3.52627
3.12209 3.22629 2.97938 5.41503 3.90962 242 g - - -
2.68619 4.42226 2.77520 2.73124 3.46355 2.40513 3.72479
3.29355 2.67741 2.69356 4.24690 2.90347 2.73740 3.18147 2.89801
2.37882 2.77520 2.98519 4.58478 3.61504
0.02614 3.76805 5.91362 0.43726 1.03791 0.48576 0.95510
99 1.71589 4.67921 3.34972 2.67012 4.00953 3.57244 3.41662
3.36229 2.82166 3.32642 3.69098 2.67864 4.19613 3.16965 3.18634
1.69815 2.63425 3.38674 5.64832 4.31998 244 s - - -
2.68618 4.42225 2.77519 2.73123 3.46354 2.40513 3.72494
3.29354 2.67741 2.69355 4.24690 2.90347 2.73739 3.18146 2.89801
2.37887 2.77519 2.98518 4.58477 3.61503
0.00830 5.19127 5.91362 0.61958 0.77255 0.48576 0.95510

```
     100   2.39556  4.59969  3.92236  2.95589  3.15115  3.98232  2.88190
2.45840  2.33317  2.24867  3.66450  3.63304  4.10070  3.56860  2.64299
2.93212  3.07443  2.32172  3.86543  3.35503    245 l - - -
           2.68618  4.42225  2.77519  2.73123  3.46354  2.40513  3.72494
3.29354  2.67741  2.69355  4.24690  2.90347  2.73739  3.18146  2.89801
2.37887  2.77519  2.98518  4.58477  3.61503
           0.00830  5.19127  5.91362  0.61958  0.77255  0.48576  0.95510
     101   2.97100  5.08471  3.47274  2.88382  4.35125  2.22655  3.97996
3.34989  2.93791  3.28074  4.22119  3.27384  4.24934  3.28239  3.38166
1.05764  2.91757  3.44746  5.66114  4.16858    246 s - - -
           2.68618  4.42225  2.77519  2.73123  3.46354  2.40513  3.72494
3.29354  2.67741  2.69355  4.24690  2.90347  2.73739  3.18146  2.89801
2.37887  2.77519  2.98518  4.58477  3.61503
           0.00830  5.19127  5.91362  0.61958  0.77255  0.48576  0.95510
     102   2.92015  1.71318  2.32506  2.89601  4.35873  3.06794  4.06378
3.63516  2.88705  3.29065  3.96034  3.39801  3.09456  3.23144  3.33982
1.94054  3.00398  3.36971  5.65492  4.33772    247 c - - -
           2.68618  4.42225  2.77519  2.73123  3.46354  2.40513  3.72494
3.29354  2.67741  2.69355  4.24690  2.90347  2.73739  3.18146  2.89801
2.37887  2.77519  2.98518  4.58477  3.61503
           0.00830  5.19127  5.91362  0.61958  0.77255  0.48576  0.95510
     103   2.99023  5.06811  4.25469  4.13455  5.50679  3.78992  5.27767
4.95385  3.85352  4.61812  5.42313  4.23066  0.39328  4.56302  4.50611
2.75393  3.08793  4.26091  6.80495  5.63434    248 P - - -
           2.68618  4.42225  2.77519  2.73123  3.46354  2.40513  3.72494
3.29354  2.67741  2.69355  4.24690  2.90347  2.73739  3.18146  2.89801
2.37887  2.77519  2.98518  4.58477  3.61503
           0.00830  5.19127  5.91362  0.61958  0.77255  0.48576  0.95510
     104   1.91606  4.36857  2.94249  2.34693  4.72081  3.55348  3.93570
4.04567  2.52087  3.35410  4.08948  2.75052  1.85746  2.97880  2.90704
2.70818  3.10193  3.39354  5.85284  4.45915    249 p - - -
           2.68618  4.42225  2.77519  2.73123  3.46354  2.40513  3.72494
3.29354  2.67741  2.69355  4.24690  2.90347  2.73739  3.18146  2.89801
2.37887  2.77519  2.98518  4.58477  3.61503
           0.00830  5.19127  5.91362  0.61958  0.77255  0.48576  0.95510
     105   3.39765  4.89626  5.58877  5.01063  3.63998  4.90586  5.29536
1.73681  4.84319  0.95250  3.44080  5.06418  4.51358  4.94157  4.86671
4.24938  3.83240  1.48728  5.66575  4.53580    250 l - - -
           2.68618  4.42225  2.77519  2.73123  3.46354  2.40513  3.72494
3.29354  2.67741  2.69355  4.24690  2.90347  2.73739  3.18146  2.89801
2.37887  2.77519  2.98518  4.58477  3.61503
           0.00830  5.19127  5.91362  0.61958  0.77255  0.48576  0.95510
     106   2.81738  5.42207  3.05619  2.58135  4.54996  3.59089  3.75962
4.06826  2.28789  2.55462  4.39096  3.09556  3.72874  2.39966  1.47242
2.71611  3.10249  3.80186  4.87085  4.38260    251 r - - -
           2.68632  4.42256  2.77482  2.72996  3.46379  2.40529  3.72511
3.29380  2.67746  2.69380  4.24720  2.90317  2.73754  3.18152  2.89809
2.37901  2.77524  2.98530  4.58508  3.61528
           0.38146  1.15698  5.91362  0.68665  0.69969  0.48576  0.95510
     107   1.83416  4.06538  4.84349  4.23652  2.64876  4.16088  4.20239
1.72911  3.94455  2.31168  3.51903  4.28094  2.88510  4.20335  4.02840
2.99413  3.01705  2.02340  5.02063  3.39077    254 i - - -
           2.68618  4.42225  2.77519  2.73123  3.46354  2.40513  3.72494
3.29354  2.67741  2.69355  4.24690  2.90347  2.73739  3.18146  2.89801
2.37887  2.77519  2.98518  4.58477  3.61503
           0.00830  5.19127  5.91362  0.61958  0.77255  0.48576  0.95510
```

```
    108   4.00276  5.26388  5.63199  5.20404  1.82275  5.02186  4.47777
2.66228  4.99632  2.17499  3.85511  4.93741  5.31762  4.93636  4.93491
4.36502  4.22274  3.51237  4.30485  0.73508    255 y - - -
          2.68618  4.42225  2.77519  2.73123  3.46354  2.40513  3.72494
3.29354  2.67741  2.69355  4.24690  2.90347  2.73739  3.18146  2.89801
2.37887  2.77519  2.98518  4.58477  3.61503
          0.00830  5.19127  5.91362  0.61958  0.77255  0.48576  0.95510
    109   2.92566  5.45967  2.79493  2.35888  4.58333  3.73491  3.79138
3.99688  2.17883  3.55140  4.42793  2.47621  4.12828  2.88952  2.49021
2.47367  1.67359  3.43320  5.89497  4.41561    256 t - - -
          2.68618  4.42225  2.77519  2.73123  3.46354  2.40513  3.72494
3.29354  2.67741  2.69355  4.24690  2.90347  2.73739  3.18146  2.89801
2.37887  2.77519  2.98518  4.58477  3.61503
          0.00830  5.19127  5.91362  0.61958  0.77255  0.48576  0.95510
    110   1.78769  5.15475  3.38746  2.74949  4.40431  2.47111  4.02204
3.81930  2.80710  3.13731  4.24218  3.34858  1.87295  3.08827  3.28840
2.36204  2.93405  3.36449  5.67535  4.05827    257 a - - -
          2.68618  4.42225  2.77519  2.73123  3.46354  2.40513  3.72494
3.29354  2.67741  2.69355  4.24690  2.90347  2.73739  3.18146  2.89801
2.37887  2.77519  2.98518  4.58477  3.61503
          0.00830  5.19127  5.91362  0.61958  0.77255  0.48576  0.95510
    111   2.40145  5.47196  2.14339  1.74434  4.81756  3.28080  3.85034
4.15248  2.20723  3.63004  4.51252  2.73532  3.11346  2.77496  3.08630
2.55005  2.97978  3.81811  5.90430  4.41466    258 e - - -
          2.68618  4.42225  2.77519  2.73123  3.46354  2.40513  3.72494
3.29354  2.67741  2.69355  4.24690  2.90347  2.73739  3.18146  2.89801
2.37887  2.77519  2.98518  4.58477  3.61503
          0.00830  5.19127  5.91362  0.61958  0.77255  0.48576  0.95510
    112   2.84472  5.25800  2.57190  2.25518  4.12171  3.03502  3.58707
4.29772  2.28810  3.22344  4.24941  2.18205  4.12671  2.79012  2.23424
2.61759  2.43772  3.85822  5.90115  4.36877    259 n - - -
          2.68618  4.42225  2.77519  2.73123  3.46354  2.40513  3.72494
3.29354  2.67741  2.69355  4.24690  2.90347  2.73739  3.18146  2.89801
2.37887  2.77519  2.98518  4.58477  3.61503
          0.00830  5.19127  5.91362  0.61958  0.77255  0.48576  0.95510
    113   3.20579  4.55849  5.16965  4.56651  3.11386  4.41455  4.76506
2.11061  4.28078  1.16395  3.08760  4.46480  4.75727  4.49591  4.38460
3.65256  3.21296  1.41605  5.23501  4.06703    260 l - - -
          2.68618  4.42225  2.77519  2.73123  3.46354  2.40513  3.72494
3.29354  2.67741  2.69355  4.24690  2.90347  2.73739  3.18146  2.89801
2.37887  2.77519  2.98518  4.58477  3.61503
          0.00830  5.19127  5.91362  0.61958  0.77255  0.48576  0.95510
    114   2.87946  5.42305  1.96578  1.75906  3.13278  3.58453  3.54891
3.99190  2.35459  3.62869  3.91872  2.80838  4.13465  2.88299  3.00468
2.81152  3.01472  3.14003  4.55180  3.79803    261 e - - -
          2.68618  4.42225  2.77519  2.73123  3.46354  2.40513  3.72494
3.29354  2.67741  2.69355  4.24690  2.90347  2.73739  3.18146  2.89801
2.37887  2.77519  2.98518  4.58477  3.61503
          0.00830  5.19127  5.91362  0.61958  0.77255  0.48576  0.95510
    115   2.25835  5.46840  2.96454  1.86378  4.61526  3.59383  3.06235
4.23367  2.45495  3.67244  3.64090  2.95916  4.01597  2.41768  2.39948
2.26363  2.83527  3.81641  5.90144  4.49086    262 e - - -
          2.68618  4.42225  2.77519  2.73123  3.46354  2.40513  3.72494
3.29354  2.67741  2.69355  4.24690  2.90347  2.73739  3.18146  2.89801
2.37887  2.77519  2.98518  4.58477  3.61503
          0.00830  5.19127  5.91362  0.61958  0.77255  0.48576  0.95510
```

```
    116   2.83101  5.45370  2.59705  1.90905  4.78694  3.49086  3.93424
4.03232  2.64203  3.00399  4.12357  3.11695  4.13809  1.32484  3.16654
2.88941  3.18030  3.83805  5.89311  4.48845    263 q - - -
          2.68618  4.42225  2.77519  2.73123  3.46354  2.40513  3.72494
3.29354  2.67741  2.69355  4.24690  2.90347  2.73739  3.18146  2.89801
2.37887  2.77519  2.98518  4.58477  3.61503
          0.00830  5.19127  5.91362  0.61958  0.77255  0.48576  0.95510
    117   4.12871  5.38381  6.02578  5.42727  2.72340  5.43048  5.69171
2.73850  4.34854  0.38819  3.09288  5.57546  5.52998  5.17585  5.21964
4.79187  4.33259  3.07327  5.79658  4.79293    264 L - - -
          2.68618  4.42225  2.77519  2.73123  3.46354  2.40513  3.72494
3.29354  2.67741  2.69355  4.24690  2.90347  2.73739  3.18146  2.89801
2.37887  2.77519  2.98518  4.58477  3.61503
          0.00830  5.19127  5.91362  0.61958  0.77255  0.48576  0.95510
    118   2.66586  5.47295  2.40512  1.50297  4.81872  3.67649  3.64690
4.21532  2.48339  3.77848  4.33260  2.42283  4.12711  2.55993  2.45408
2.66368  3.10142  3.76241  5.90513  4.49369    265 e - - -
          2.68618  4.42225  2.77519  2.73123  3.46354  2.40513  3.72494
3.29354  2.67741  2.69355  4.24690  2.90347  2.73739  3.18146  2.89801
2.37887  2.77519  2.98518  4.58477  3.61503
          0.00830  5.19127  5.91362  0.61958  0.77255  0.48576  0.95510
    119   2.33037  4.43953  2.64383  2.07265  4.79700  3.55682  3.79032
3.74293  2.31580  3.33360  4.37254  2.60840  4.12855  2.60326  2.84365
2.07467  2.91149  3.10385  5.89321  4.24464    266 e - - -
          2.68618  4.42225  2.77519  2.73123  3.46354  2.40513  3.72494
3.29354  2.67741  2.69355  4.24690  2.90347  2.73739  3.18146  2.89801
2.37887  2.77519  2.98518  4.58477  3.61503
          0.01368  5.19127  4.82538  0.61958  0.77255  0.48576  0.95510
    120   1.31808  4.68677  3.24703  2.85582  4.31878  3.41608  4.02500
3.35317  2.84602  3.05634  4.11991  2.81522  4.20422  2.48410  3.25504
2.47998  3.11772  2.74420  5.61566  4.29654    267 a - - -
          2.68618  4.42225  2.77519  2.73123  3.46354  2.40513  3.72494
3.29354  2.67741  2.69355  4.24690  2.90347  2.73739  3.18146  2.89801
2.37887  2.77519  2.98518  4.58477  3.61503
          0.00835  5.18594  5.90828  0.61958  0.77255  0.50377  0.92697
    121   1.69946  3.48641  3.54958  2.98853  4.12538  3.14105  4.08793
3.34969  1.98888  2.96762  3.00527  3.47081  4.25082  3.22240  3.09320
2.93559  2.16584  3.19253  5.48816  4.20448    268 a - - -
          2.68618  4.42225  2.77519  2.73123  3.46354  2.40513  3.72494
3.29354  2.67741  2.69355  4.24690  2.90347  2.73739  3.18146  2.89801
2.37887  2.77519  2.98518  4.58477  3.61503
          0.00835  5.18594  5.90828  0.61958  0.77255  0.50377  0.92697
    122   2.62292  5.21560  3.14670  1.89869  4.55224  3.73659  3.63518
3.78591  2.12398  3.19793  3.45772  2.91762  4.12973  2.81212  1.96402
2.76307  2.58263  3.52198  5.87559  4.39566    269 e - - -
          2.68618  4.42225  2.77519  2.73123  3.46354  2.40513  3.72494
3.29354  2.67741  2.69355  4.24690  2.90347  2.73739  3.18146  2.89801
2.37887  2.77519  2.98518  4.58477  3.61503
          0.01099  5.18594  5.23259  0.61958  0.77255  0.50377  0.92697
    123   2.43540  5.46353  2.41411  1.59334  4.58394  3.55652  3.85911
4.29261  2.29293  3.44709  4.28992  2.95955  4.12324  2.89307  2.19553
2.64965  3.02625  3.85356  5.36106  4.15595    270 e - - -
          2.68618  4.42225  2.77519  2.73123  3.46354  2.40513  3.72494
3.29354  2.67741  2.69355  4.24690  2.90347  2.73739  3.18146  2.89801
2.37887  2.77519  2.98518  4.58477  3.61503
          0.04426  5.18331  3.27856  0.61958  0.77255  0.51248  0.91382
```

```
    124   5.40531  6.19414  6.08725  6.12888  0.71199  5.73937  4.24684
4.92858  5.88489  3.99327  5.41473  5.20885  5.97207  5.41619  5.60185
5.15977  5.57993  4.89803  2.63381  1.07008    271 f - - -
          2.68618  4.42225  2.77519  2.73123  3.46354  2.40513  3.72494
3.29354  2.67741  2.69355  4.24690  2.90347  2.73739  3.18146  2.89801
2.37887  2.77519  2.98518  4.58477  3.61503
          0.21815  5.14773  1.65976  0.61958  0.77255  0.62159  0.77022
    125   3.86534  5.07010  5.91705  5.39032  3.86362  5.37874  5.87419
1.26425  5.28955  1.05347  3.32391  5.54180  5.51392  5.32788  5.32546
4.78358  4.10001  1.54741  6.01712  4.95913    272 l - - -
          2.68618  4.42225  2.77519  2.73123  3.46354  2.40513  3.72494
3.29354  2.67741  2.69355  4.24690  2.90347  2.73739  3.18146  2.89801
2.37887  2.77519  2.98518  4.58477  3.61503
          0.00720  4.93678        *  0.61958  0.77255  0.00000        *
//
HMMER3/f [3.3 | Nov 2019]
NAME  DUF573
ACC   PF04504.15
DESC  Protein of unknown function, DUF573
LENG  95
ALPH  amino
RF    no
MM    no
CONS  yes
CS    no
MAP   yes
DATE  Wed Apr 22 04:10:50 2020
NSEQ  74
EFFN  2.795776
CKSUM 358131143
GA    29.50 29.50
TC    29.50 29.50
NC    29.40 29.40
STATS LOCAL MSV       -9.3948  0.71833
STATS LOCAL VITERBI  -10.3276  0.71833
STATS LOCAL FORWARD   -3.5457  0.71833
HMM          A        C        D        E        F        G        H
I        K        L        M        N        P        Q        R        S
T        V        W        Y
            m->m     m->i     m->d     i->m     i->i     d->m     d->d
  COMPO   2.69178  4.70351  2.83355  2.53442  3.24394  3.18572  3.66748
2.93304  2.34571  2.47199  3.81237  3.11834  3.63766  2.94163  2.74633
2.57185  2.89931  2.90806  3.96738  3.46753
          2.68618  4.42225  2.77519  2.73123  3.46354  2.40513  3.72494
3.29354  2.67741  2.69355  4.24690  2.90347  2.73739  3.18146  2.89801
2.37887  2.77519  2.98518  4.58477  3.61503
          0.19205  4.87222  1.78928  0.61958  0.77255  0.00000        *
      1   2.67611  4.29512  4.05833  3.47842  1.62295  3.83829  4.12749
2.63792  3.24466  2.07443  3.40765  3.56072  3.17883  3.33331  3.61661
2.86954  2.99852  2.44739  4.89901  3.44099      1 f - - -
          2.68618  4.42225  2.77519  2.73123  3.46354  2.40513  3.72494
3.29354  2.67741  2.69355  4.24690  2.90347  2.73739  3.18146  2.89801
2.37887  2.77519  2.98518  4.58477  3.61503
          0.01369  4.69386  5.41620  0.61958  0.77255  0.45627  1.00414
      2   2.75300  5.28707  2.92060  2.42547  4.62022  3.39953  2.67888
4.09761  2.25638  3.58707  3.88357  2.98642  3.13869  1.76514  2.90807
2.50766  3.03684  3.17288  5.72676  4.32346      2 q - - -
```

```
          2.68618  4.42225  2.77519  2.73123  3.46354  2.40513  3.72494
3.29354  2.67741  2.69355  4.24690  2.90347  2.73739  3.18146  2.89801
2.37887  2.77519  2.98518  4.58477  3.61503
          0.01208  4.81837  5.54071  0.61958  0.77255  0.54324  0.86956
      3   3.26556  5.45586  3.72287  3.03749  4.84307  3.97826  3.95972
4.20793  2.15333  3.32726  3.81281  3.48292  4.33172  2.55470  0.88182
3.16790  3.24593  3.87744  5.78659  4.56482      3 r - - -
          2.68618  4.42225  2.77519  2.73123  3.46354  2.40513  3.72494
3.29354  2.67741  2.69355  4.24690  2.90347  2.73739  3.18146  2.89801
2.37887  2.77519  2.98518  4.58477  3.61503
          0.01187  4.83574  5.55809  0.61958  0.77255  0.52263  0.89885
      4   2.83701  4.36096  4.14181  3.56127  3.47196  3.91133  4.20250
1.86136  3.10406  1.67082  3.47431  2.76558  4.28343  3.70780  3.48454
2.74664  2.99133  2.09880  4.96942  3.76985      4 l - - -
          2.68618  4.42225  2.77519  2.73123  3.46354  2.40513  3.72494
3.29354  2.67741  2.69355  4.24690  2.90347  2.73739  3.18146  2.89801
2.37887  2.77519  2.98518  4.58477  3.61503
          0.01172  4.84838  5.57073  0.61958  0.77255  0.45181  1.01191
      5   4.78993  5.80410  5.69602  5.58795  1.91479  5.27575  4.16533
4.31308  5.34354  3.44944  4.84091  4.98417  5.58725  5.12161  5.19524
4.78624  5.01456  4.31899  0.47219  2.50477      5 W - - -
          2.68618  4.42225  2.77519  2.73123  3.46354  2.40513  3.72494
3.29354  2.67741  2.69355  4.24690  2.90347  2.73739  3.18146  2.89801
2.37887  2.77519  2.98518  4.58477  3.61503
          0.01144  4.87222  5.59457  0.61958  0.77255  0.48576  0.95510
      6   2.69698  4.24683  3.51234  2.99438  4.48676  3.59305  4.15297
3.90836  2.86108  3.52659  4.33690  2.85065  3.73326  3.32581  3.41885
1.11779  1.95060  3.50982  5.76693  4.46541      6 s - - -
          2.68618  4.42225  2.77519  2.73123  3.46354  2.40513  3.72494
3.29354  2.67741  2.69355  4.24690  2.90347  2.73739  3.18146  2.89801
2.37887  2.77519  2.98518  4.58477  3.61503
          0.01144  4.87222  5.59457  0.61958  0.77255  0.48576  0.95510
      7   2.82738  5.32486  2.46212  1.56481  4.66428  3.54274  3.79014
4.14635  2.22234  3.26969  4.36721  3.07086  3.55477  2.84681  2.80385
2.40716  2.62688  3.38779  5.76079  4.35310      7 e - - -
          2.68618  4.42225  2.77519  2.73123  3.46354  2.40513  3.72494
3.29354  2.67741  2.69355  4.24690  2.90347  2.73739  3.18146  2.89801
2.37887  2.77519  2.98518  4.58477  3.61503
          0.01144  4.87222  5.59457  0.61958  0.77255  0.48576  0.95510
      8   2.39055  5.34368  2.34813  1.60858  4.68654  3.50238  3.57823
4.17196  2.36376  3.64798  4.38546  2.99346  2.93872  2.76571  2.86122
2.64751  2.83117  3.73323  5.77713  4.36580      8 e - - -
          2.68618  4.42225  2.77519  2.73123  3.46354  2.40513  3.72494
3.29354  2.67741  2.69355  4.24690  2.90347  2.73739  3.18146  2.89801
2.37887  2.77519  2.98518  4.58477  3.61503
          0.01144  4.87222  5.59457  0.61958  0.77255  0.48576  0.95510
      9   3.71598  6.52273  0.51464  2.10998  5.75883  3.75073  4.41983
5.36498  3.61518  4.79004  5.71076  3.06051  4.45221  3.19342  4.34364
3.52224  4.03700  4.87688  6.93721  5.29341      9 D - - -
          2.68618  4.42225  2.77519  2.73123  3.46354  2.40513  3.72494
3.29354  2.67741  2.69355  4.24690  2.90347  2.73739  3.18146  2.89801
2.37887  2.77519  2.98518  4.58477  3.61503
          0.01144  4.87222  5.59457  0.61958  0.77255  0.48576  0.95510
     10   3.20828  5.93205  2.71537  0.54264  5.35611  3.73996  4.31230
4.86768  3.26424  4.36234  5.22163  3.24507  4.37566  3.26689  3.78921
3.03895  3.75658  4.42123  6.50996  5.02898     10 E - - -
```

```
          2.68618  4.42225  2.77519  2.73123  3.46354  2.40513  3.72494
3.29354  2.67741  2.69355  4.24690  2.90347  2.73739  3.18146  2.89801
2.37887  2.77519  2.98518  4.58477  3.61503
          0.01144  4.87222  5.59457  0.61958  0.77255  0.48576  0.95510
     11   3.47737  4.89436  5.72627  5.21201  3.97503  5.19056  5.74394
0.87287  5.11512  1.65459  3.39679  5.34331  5.39546  5.25681  5.20197
4.58796  3.91988  1.52814  6.02328  4.88707     11 i - - -
          2.68618  4.42225  2.77519  2.73123  3.46354  2.40513  3.72494
3.29354  2.67741  2.69355  4.24690  2.90347  2.73739  3.18146  2.89801
2.37887  2.77519  2.98518  4.58477  3.61503
          0.01144  4.87222  5.59457  0.61958  0.77255  0.48576  0.95510
     12   2.43370  5.17669  2.94593  2.13292  4.46089  3.28532  3.82207
3.59766  2.39083  2.95933  3.98445  3.12369  4.01905  2.94278  2.15051
2.46104  2.86933  2.46828  5.64763  4.27681     12 e - - -
          2.68618  4.42225  2.77519  2.73123  3.46354  2.40513  3.72494
3.29354  2.67741  2.69355  4.24690  2.90347  2.73739  3.18146  2.89801
2.37887  2.77519  2.98518  4.58477  3.61503
          0.01144  4.87222  5.59457  0.61958  0.77255  0.48576  0.95510
     13   3.23310  4.57097  4.50809  4.56402  2.35193  4.45917  4.79941
1.37086  4.37914  1.33691  2.93408  4.60629  4.76233  4.47656  4.39780
3.78426  3.46219  2.18237  5.21080  4.07479     13 l - - -
          2.68618  4.42225  2.77519  2.73123  3.46354  2.40513  3.72494
3.29354  2.67741  2.69355  4.24690  2.90347  2.73739  3.18146  2.89801
2.37887  2.77519  2.98518  4.58477  3.61503
          0.01144  4.87222  5.59457  0.61958  0.77255  0.48576  0.95510
     14   3.93609  5.20583  5.70728  5.13680  3.52355  5.23109  5.53430
2.22156  4.89885  0.44365  3.29583  5.33860  5.35200  4.95277  4.29566
4.61288  4.15264  2.75396  5.68288  4.66484     14 L - - -
          2.68618  4.42225  2.77519  2.73123  3.46354  2.40513  3.72494
3.29354  2.67741  2.69355  4.24690  2.90347  2.73739  3.18146  2.89801
2.37887  2.77519  2.98518  4.58477  3.61503
          0.01144  4.87222  5.59457  0.61958  0.77255  0.48576  0.95510
     15   2.86471  5.36241  3.09718  1.90570  4.71172  3.36723  3.80670
4.19101  2.06460  3.66301  4.40512  2.93939  4.02174  1.77140  2.44300
2.50440  2.98746  3.75601  5.78627  4.38613     15 q - - -
          2.68618  4.42225  2.77519  2.73123  3.46354  2.40513  3.72494
3.29354  2.67741  2.69355  4.24690  2.90347  2.73739  3.18146  2.89801
2.37887  2.77519  2.98518  4.58477  3.61503
          0.01144  4.87222  5.59457  0.61958  0.77255  0.48576  0.95510
     16   2.33794  4.95850  3.30627  2.64119  4.21174  1.39501  3.92065
3.28776  2.73407  3.11587  4.07005  3.16139  4.08496  3.09027  2.72741
2.71127  3.08329  3.19785  5.50701  4.19318     16 g - - -
          2.68618  4.42225  2.77519  2.73123  3.46354  2.40513  3.72494
3.29354  2.67741  2.69355  4.24690  2.90347  2.73739  3.18146  2.89801
2.37887  2.77519  2.98518  4.58477  3.61503
          0.01144  4.87222  5.59457  0.61958  0.77255  0.48576  0.95510
     17   2.81619  4.31162  4.86210  4.25057  2.45072  4.11061  4.02466
1.85653  4.04675  1.38390  2.39840  4.25989  4.46125  4.17281  4.06538
3.42260  3.16896  2.32205  4.92017  3.47934     17 l - - -
          2.68618  4.42225  2.77519  2.73123  3.46354  2.40513  3.72494
3.29354  2.67741  2.69355  4.24690  2.90347  2.73739  3.18146  2.89801
2.37887  2.77519  2.98518  4.58477  3.61503
          0.01144  4.87222  5.59457  0.61958  0.77255  0.48576  0.95510
     18   2.22443  4.28287  4.70076  3.90544  3.29078  3.87827  4.38480
1.87454  3.92053  1.46002  3.39977  4.16175  4.41453  4.08355  3.98703
3.28398  3.07693  1.83644  4.92573  3.63824     18 l - - -
```

```
         2.68618  4.42225  2.77519  2.73123  3.46354  2.40513  3.72494
3.29354  2.67741  2.69355  4.24690  2.90347  2.73739  3.18146  2.89801
2.37887  2.77519  2.98518  4.58477  3.61503
         0.01144  4.87222  5.59457  0.61958  0.77255  0.48576  0.95510
    19   2.23961  5.33544  1.81546  2.24975  4.67574  3.45675  3.79497
4.15902  2.40323  3.63870  4.37808  2.87657  3.99937  2.85430  2.47473
2.39815  2.78644  3.59597  5.77074  4.36144     19 d - - -
         2.68618  4.42225  2.77519  2.73123  3.46354  2.40513  3.72494
3.29354  2.67741  2.69355  4.24690  2.90347  2.73739  3.18146  2.89801
2.37887  2.77519  2.98518  4.58477  3.61503
         0.06937  4.87222  2.82410  0.61958  0.77255  0.48576  0.95510
    20   2.91531  3.76056  3.94709  3.53220  1.86056  3.73078  2.52648
2.94275  3.44870  2.66040  3.58543  3.81448  4.32546  3.69814  3.70492
2.47568  3.14800  2.74054  4.82796  1.90981     20 f - - -
         2.68619  4.42226  2.77520  2.73124  3.46355  2.40513  3.72495
3.29355  2.67741  2.69356  4.24690  2.90347  2.73740  3.18147  2.89801
2.37887  2.77520  2.98502  4.58478  3.61504
         0.07149  3.39709  3.33746  0.43816  1.03627  0.41262  1.08445
    21   2.48952  5.30139  2.68742  2.27503  3.99978  3.44668  3.50607
3.87182  2.09141  3.44144  4.34451  2.97920  3.98367  2.63867  2.30809
2.62632  2.19327  3.46984  5.73967  4.33445     22 k - - -
         2.68618  4.42225  2.77519  2.73123  3.46354  2.40513  3.72494
3.29354  2.67741  2.69355  4.24690  2.90347  2.73739  3.18146  2.89801
2.37887  2.77519  2.98518  4.58477  3.61503
         0.01179  4.84193  5.56428  0.61958  0.77255  0.53492  0.88121
    22   2.18087  5.25321  2.96653  2.33299  4.38644  3.46370  3.79179
3.88060  2.16518  3.54585  3.34424  2.75108  3.99414  2.89851  2.70811
1.92300  2.69733  3.40582  5.70398  4.31159     23 s - - -
         2.68618  4.42225  2.77519  2.73123  3.46354  2.40513  3.72494
3.29354  2.67741  2.69355  4.24690  2.90347  2.73739  3.18146  2.89801
2.37887  2.77519  2.98518  4.58477  3.61503
         0.09737  4.84767  2.46593  0.61958  0.77255  0.54420  0.86823
    23   2.64518  5.29027  2.75913  2.00983  4.63279  3.56000  3.35501
4.11661  1.84012  3.59416  4.33178  2.84324  3.95382  2.36647  2.71317
2.68947  2.67127  3.06739  5.72414  4.31520     24 k - - -
         2.68618  4.42225  2.77519  2.73123  3.46354  2.40513  3.72494
3.29354  2.67741  2.69355  4.24690  2.90347  2.73739  3.18146  2.89801
2.37887  2.77519  2.98518  4.58477  3.61503
         0.01277  4.76307  5.48542  0.61958  0.77255  0.71417  0.67256
    24   2.81510  5.25639  3.08482  2.52948  4.58494  3.28668  2.49872
4.04925  2.10339  3.55000  4.30650  2.74709  3.80931  2.87743  2.39986
2.63591  1.89714  3.63924  5.69672  4.31053     25 t - - -
         2.68618  4.42225  2.77519  2.73123  3.46354  2.40513  3.72494
3.29354  2.67741  2.69355  4.24690  2.90347  2.73739  3.18146  2.89801
2.37887  2.77519  2.98518  4.58477  3.61503
         0.01277  4.76307  5.48542  0.61958  0.77255  0.71417  0.67256
    25   2.73536  5.12839  2.98233  2.48306  4.40666  1.38997  3.81878
3.84143  2.31278  3.16820  3.89161  3.11731  3.64059  2.94945  2.97100
2.82800  3.05678  3.48452  5.61013  4.25213     26 g - - -
         2.68618  4.42225  2.77519  2.73123  3.46354  2.40513  3.72494
3.29354  2.67741  2.69355  4.24690  2.90347  2.73739  3.18146  2.89801
2.37887  2.77519  2.98518  4.58477  3.61503
         0.02443  4.76307  4.16085  0.61958  0.77255  0.71417  0.67256
    26   2.46156  4.98523  3.02588  2.49386  3.83409  3.26213  3.48235
3.00726  2.24682  2.66413  4.05567  3.02447  3.82656  2.90775  2.86284
2.54218  2.28836  2.88245  5.48738  3.83458     27 k - - -
```

```
          2.68611  4.42228  2.77522  2.73126  3.46356  2.40515  3.72497
3.29357  2.67743  2.69357  4.24692  2.90349  2.73742  3.18149  2.89767
2.37889  2.77522  2.98521  4.58479  3.61506
          0.21500  2.38762  2.28657  0.27364  1.42965  0.64092  0.74826
     27   2.75635  5.10717  2.71316  2.30839  3.64476  3.55608  3.75185
3.83674  2.51668  2.49850  4.16461  2.61984  3.01045  2.71989  2.99672
2.06080  2.72670  3.29373  5.57821  4.20711     29 s - - -
          2.68623  4.42230  2.77524  2.73128  3.46326  2.40518  3.72499
3.29359  2.67720  2.69360  4.24695  2.90352  2.73744  3.18151  2.89806
2.37882  2.77517  2.98523  4.58482  3.61475
          0.41134  2.42581  1.39097  0.70496  0.68147  0.63226  0.75799
     28   2.19740  4.49092  3.66304  3.19975  4.19679  3.36824  4.22976
3.53208  3.16444  2.99678  4.11515  3.48766  1.20376  3.48725  3.51941
2.49484  2.68716  2.97284  5.57277  4.33604     33 p - - -
          2.68618  4.42225  2.77519  2.73123  3.46354  2.40513  3.72494
3.29354  2.67741  2.69355  4.24690  2.90347  2.73739  3.18146  2.89801
2.37887  2.77519  2.98518  4.58477  3.61503
          0.06669  4.49019  2.93182  0.61958  0.77255  0.83276  0.57066
     29   2.58740  4.72846  3.23674  2.67663  3.31652  3.22718  3.36902
3.31278  2.21941  2.90857  3.81302  3.04445  3.67699  2.88777  3.09754
2.35603  2.68315  2.73935  4.80419  2.71763     34 k - - -
          2.68626  4.42244  2.77538  2.73142  3.46358  2.40499  3.72405
3.29373  2.67739  2.69352  4.24691  2.90356  2.73749  3.18115  2.89770
2.37890  2.77522  2.98537  4.58496  3.61497
          0.55202  0.90487  3.93108  0.60782  0.78644  0.42803  1.05495
     30   2.71374  5.26216  2.37977  2.31711  4.29974  3.47839  3.55630
3.75135  2.38764  3.28926  4.30677  2.94512  2.50517  2.55955  2.75635
2.22787  3.01885  3.57952  5.70388  3.34176     37 s - - -
          2.68618  4.42225  2.77519  2.73123  3.46354  2.40513  3.72494
3.29354  2.67741  2.69355  4.24690  2.90347  2.73739  3.18146  2.89801
2.37887  2.77519  2.98518  4.58477  3.61503
          0.01264  4.77268  5.49502  0.61958  0.77255  0.40384  1.10186
     31   2.83697  5.33151  1.47228  2.43185  4.66823  3.39743  3.46585
4.14948  2.53903  3.63296  4.37522  2.68005  4.00085  2.66616  2.53149
2.64171  2.95694  3.50009  5.76821  3.96360     38 d - - -
          2.68618  4.42225  2.77519  2.73123  3.46354  2.40513  3.72494
3.29354  2.67741  2.69355  4.24690  2.90347  2.73739  3.18146  2.89801
2.37887  2.77519  2.98518  4.58477  3.61503
          0.01156  4.86185  5.58420  0.61958  0.77255  0.51104  0.91597
     32   2.69630  4.41828  3.98291  3.40768  3.53505  3.02901  3.63150
2.50073  2.97209  2.01079  2.55218  3.59953  3.73972  3.59933  3.05203
2.97510  2.28926  2.44723  3.41390  3.80785     39 l - - -
          2.68618  4.42225  2.77519  2.73123  3.46354  2.40513  3.72494
3.29354  2.67741  2.69355  4.24690  2.90347  2.73739  3.18146  2.89801
2.37887  2.77519  2.98518  4.58477  3.61503
          0.01156  4.86185  5.58420  0.61958  0.77255  0.47027  0.98038
     33   2.41005  5.32412  2.02947  2.40698  4.66188  3.03043  3.55204
4.04489  2.53218  3.62638  4.36716  2.32713  3.76616  2.89202  2.88578
2.07678  2.82332  3.71092  5.76120  4.08740     40 d - - -
          2.68618  4.42225  2.77519  2.73123  3.46354  2.40513  3.72494
3.29354  2.67741  2.69355  4.24690  2.90347  2.73739  3.18146  2.89801
2.37887  2.77519  2.98518  4.58477  3.61503
          0.01144  4.87222  5.59457  0.61958  0.77255  0.48576  0.95510
     34   1.91452  5.31393  2.78255  2.26804  4.64966  2.63869  3.79071
4.12913  2.34669  3.39727  4.35718  2.69233  3.35411  2.74047  2.96974
2.57909  2.55682  3.42424  5.75229  4.34693     41 a - - -
```

```
          2.68618  4.42225  2.77519  2.73123  3.46354  2.40513  3.72494
3.29354  2.67741  2.69355  4.24690  2.90347  2.73739  3.18146  2.89801
2.37887  2.77519  2.98518  4.58477  3.61503
          0.01144  4.87222  5.59457  0.61958  0.77255  0.48576  0.95510
     35   2.92711  4.50360  5.01438  4.41969  1.34841  4.32153  4.56601
2.16841  4.22895  1.53182  3.37124  4.44234  4.64585  4.33245  4.25229
3.63885  3.37316  2.52448  4.97511  2.85342      42 f - - -
          2.68618  4.42225  2.77519  2.73123  3.46354  2.40513  3.72494
3.29354  2.67741  2.69355  4.24690  2.90347  2.73739  3.18146  2.89801
2.37887  2.77519  2.98518  4.58477  3.61503
          0.01144  4.87222  5.59457  0.61958  0.77255  0.48576  0.95510
     36   2.64100  3.30062  4.41050  3.81891  2.69100  3.96443  2.64518
2.37126  3.68153  2.44481  3.41304  3.98434  4.33253  3.73224  3.47761
3.13829  3.07862  2.46757  4.90422  1.64202      43 y - - -
          2.68630  4.42237  2.77499  2.73135  3.46366  2.40502  3.72506
3.29366  2.67753  2.69307  4.24702  2.90359  2.73752  3.18158  2.89813
2.37899  2.77532  2.98530  4.58291  3.61440
          0.06937  2.75987  5.59457  1.43539  0.27184  0.48576  0.95510
     37   2.24847  4.45714  1.88258  1.78996  4.64733  3.07487  3.60227
3.96464  2.53624  3.55472  4.35806  2.95919  3.99931  2.83907  2.90256
2.71462  2.98646  3.69834  5.75355  4.34896      50 e - - -
          2.68618  4.42225  2.77519  2.73123  3.46354  2.40513  3.72494
3.29354  2.67741  2.69355  4.24690  2.90347  2.73739  3.18146  2.89801
2.37887  2.77519  2.98518  4.58477  3.61503
          0.01144  4.87222  5.59457  0.61958  0.77255  0.48576  0.95510
     38   2.09249  5.02729  3.06488  2.55848  2.64950  3.66000  3.22361
3.37683  2.25064  3.07960  3.90162  3.19126  4.04975  2.79824  2.76836
2.47681  2.89631  3.19431  5.53029  3.27939      51 a - - -
          2.68618  4.42225  2.77519  2.73123  3.46354  2.40513  3.72494
3.29354  2.67741  2.69355  4.24690  2.90347  2.73739  3.18146  2.89801
2.37887  2.77519  2.98518  4.58477  3.61503
          0.01144  4.87222  5.59457  0.61958  0.77255  0.48576  0.95510
     39   2.50069  3.20092  4.81860  4.20624  2.49165  4.04522  4.04515
1.86053  3.99713  1.82573  3.05194  4.20130  4.40646  4.13172  4.01296
3.35686  3.10458  1.71020  4.87652  3.70085      52 v - - -
          2.68618  4.42225  2.77519  2.73123  3.46354  2.40513  3.72494
3.29354  2.67741  2.69355  4.24690  2.90347  2.73739  3.18146  2.89801
2.37887  2.77519  2.98518  4.58477  3.61503
          0.01144  4.87222  5.59457  0.61958  0.77255  0.48576  0.95510
     40   2.49179  5.31085  3.02178  2.53997  4.64513  3.01871  3.55645
4.12019  1.64716  3.44302  4.35542  3.08454  4.00444  2.65068  2.22816
2.23660  3.06530  3.43645  5.74845  4.34896      53 k - - -
          2.68618  4.42225  2.77519  2.73123  3.46354  2.40513  3.72494
3.29354  2.67741  2.69355  4.24690  2.90347  2.73739  3.18146  2.89801
2.37887  2.77519  2.98518  4.58477  3.61503
          0.01144  4.87222  5.59457  0.61958  0.77255  0.48576  0.95510
     41   2.83421  5.34088  2.55596  2.44519  4.68343  2.38212  3.36186
4.16822  2.02179  3.64496  4.38282  2.74918  3.31783  2.89157  2.82253
1.93745  2.98996  3.73029  5.77459  4.36406      54 s - - -
          2.68618  4.42225  2.77519  2.73123  3.46354  2.40513  3.72494
3.29354  2.67741  2.69355  4.24690  2.90347  2.73739  3.18146  2.89801
2.37887  2.77519  2.98518  4.58477  3.61503
          0.01144  4.87222  5.59457  0.61958  0.77255  0.48576  0.95510
     42   2.82759  4.17119  3.18887  2.50023  3.76445  3.64457  3.84391
3.56919  2.06554  3.18890  4.16076  2.95343  4.03526  2.83141  2.67593
1.73456  2.43325  3.43878  4.76004  4.06581      55 s - - -
```

```
          2.68618  4.42225  2.77519  2.73123  3.46354  2.40513  3.72494
3.29354  2.67741  2.69355  4.24690  2.90347  2.73739  3.18146  2.89801
2.37887  2.77519  2.98518  4.58477  3.61503
          0.01144  4.87222  5.59457  0.61958  0.77255  0.48576  0.95510
     43   2.85215  4.23800  4.74064  4.13072  2.44657  3.54283  4.34410
1.79555  3.93240  1.58510  2.96610  4.15153  4.37794  4.07753  3.96735
3.11176  2.49210  2.30938  4.85963  3.38224     56 l - - -
          2.68618  4.42225  2.77519  2.73123  3.46354  2.40513  3.72494
3.29354  2.67741  2.69355  4.24690  2.90347  2.73739  3.18146  2.89801
2.37887  2.77519  2.98518  4.58477  3.61503
          0.01144  4.87222  5.59457  0.61958  0.77255  0.48576  0.95510
     44   2.82552  5.24310  2.55952  2.41947  4.13130  3.26655  3.06696
2.91643  2.55686  2.96927  4.29368  2.71996  3.79703  2.62835  2.98431
1.84935  2.87003  3.51311  5.69859  4.31112     57 s - - -
          2.68618  4.42225  2.77519  2.73123  3.46354  2.40513  3.72494
3.29354  2.67741  2.69355  4.24690  2.90347  2.73739  3.18146  2.89801
2.37887  2.77519  2.98518  4.58477  3.61503
          0.09352  4.87222  2.50564  0.61958  0.77255  0.48576  0.95510
     45   2.54971  4.63943  2.90709  2.95096  2.40414  3.62157  3.97235
3.03159  2.92086  2.56814  3.73641  3.40030  3.49445  3.02398  3.31596
2.06443  2.53913  2.43598  5.20522  3.94820     58 s - - -
          2.68656  4.42207  2.77557  2.73161  3.46392  2.40551  3.72056
3.29392  2.67779  2.69393  4.24728  2.90385  2.73644  3.17770  2.89839
2.37925  2.77557  2.98556  4.58515  3.61541
          0.12568  2.17092  5.51346  2.08368  0.13293  0.38925  1.13184
     46   2.83432  5.34716  1.96407  2.07568  4.69219  3.49933  3.57715
4.17880  2.22114  3.65223  4.38833  2.75062  2.86190  2.60879  2.50960
2.63512  2.91951  3.73787  5.77920  4.36710     72 d - - -
          2.68618  4.42225  2.77519  2.73123  3.46354  2.40513  3.72494
3.29354  2.67741  2.69355  4.24690  2.90347  2.73739  3.18146  2.89801
2.37887  2.77519  2.98518  4.58477  3.61503
          0.01144  4.87222  5.59457  0.61958  0.77255  0.48576  0.95510
     47   2.29909  4.40458  4.03479  3.27495  2.38840  3.65897  4.17515
2.86921  2.87426  2.52464  3.51555  3.76510  3.40962  3.63720  3.18808
2.85088  3.01657  1.95388  3.45786  2.62814     73 v - - -
          2.68618  4.42225  2.77519  2.73123  3.46354  2.40513  3.72494
3.29354  2.67741  2.69355  4.24690  2.90347  2.73739  3.18146  2.89801
2.37887  2.77519  2.98518  4.58477  3.61503
          0.01144  4.87222  5.59457  0.61958  0.77255  0.48576  0.95510
     48   2.73488  5.27089  2.94860  2.37163  4.58788  3.27186  3.80981
3.69793  2.50771  3.56540  4.32244  2.45810  4.00994  2.91745  2.93628
1.80920  1.90359  3.64706  5.72399  4.33105     74 s - - -
          2.68618  4.42225  2.77519  2.73123  3.46354  2.40513  3.72494
3.29354  2.67741  2.69355  4.24690  2.90347  2.73739  3.18146  2.89801
2.37887  2.77519  2.98518  4.58477  3.61503
          0.01144  4.87222  5.59457  0.61958  0.77255  0.48576  0.95510
     49   2.63265  4.21543  3.06421  2.68179  3.51110  3.66383  3.75122
3.23127  1.45239  2.93970  3.99839  2.97757  3.68657  2.91713  2.94293
2.76360  2.82874  3.17648  5.51779  3.73763     75 k - - -
          2.68623  4.42238  2.77514  2.73136  3.46367  2.40517  3.72489
3.29359  2.67735  2.69358  4.24703  2.90359  2.73752  3.18138  2.89795
2.37884  2.77494  2.98496  4.58490  3.61503
          0.21718  1.65287  5.59457  0.65591  0.73183  0.48576  0.95510
     50   2.59262  5.31947  2.77147  2.11680  4.65784  3.60088  3.54796
3.62330  2.32350  3.62189  4.36192  2.35976  3.39962  2.84337  2.83190
2.14552  2.38661  3.28004  5.75618  4.34928     78 e - - -
```

2.68618 4.42225 2.77519 2.73123 3.46354 2.40513 3.72494
3.29354 2.67741 2.69355 4.24690 2.90347 2.73739 3.18146 2.89801
2.37887 2.77519 2.98518 4.58477 3.61503
0.01144 4.87222 5.59457 0.61958 0.77255 0.48576 0.95510
51 3.08966 5.61209 2.62872 2.22057 4.93519 3.24090 3.98635
4.42470 2.64428 3.70825 4.67679 2.99441 4.15645 0.99293 3.29047
3.02533 3.33512 3.99455 6.04102 4.60918 79 q - - -
2.68618 4.42225 2.77519 2.73123 3.46354 2.40513 3.72494
3.29354 2.67741 2.69355 4.24690 2.90347 2.73739 3.18146 2.89801
2.37887 2.77519 2.98518 4.58477 3.61503
0.01144 4.87222 5.59457 0.61958 0.77255 0.48576 0.95510
52 3.46464 4.77923 5.40281 4.79911 2.29870 4.70270 5.01157
2.15288 4.61551 0.85397 2.96534 4.85736 4.94657 4.64233 4.60214
4.03619 3.68505 2.10748 5.31679 3.71382 80 l - - -
2.68618 4.42225 2.77519 2.73123 3.46354 2.40513 3.72494
3.29354 2.67741 2.69355 4.24690 2.90347 2.73739 3.18146 2.89801
2.37887 2.77519 2.98518 4.58477 3.61503
0.01144 4.87222 5.59457 0.61958 0.77255 0.48576 0.95510
53 2.49754 4.53342 3.75515 3.18662 3.43804 3.36050 4.08420
2.64584 2.68613 2.56590 3.37221 3.58095 4.20555 3.00967 3.20125
2.22008 2.75552 2.11167 5.11852 2.57247 81 v - - -
2.68618 4.42225 2.77519 2.73123 3.46354 2.40513 3.72494
3.29354 2.67741 2.69355 4.24690 2.90347 2.73739 3.18146 2.89801
2.37887 2.77519 2.98518 4.58477 3.61503
0.01144 4.87222 5.59457 0.61958 0.77255 0.48576 0.95510
54 2.84253 5.35176 2.02568 1.67224 4.69377 3.17867 3.80045
4.17944 2.35359 3.65571 4.39411 2.82684 4.00361 2.44893 3.03632
2.66288 2.76671 3.50883 5.78533 4.01160 82 e - - -
2.68618 4.42225 2.77519 2.73123 3.46354 2.40513 3.72494
3.29354 2.67741 2.69355 4.24690 2.90347 2.73739 3.18146 2.89801
2.37887 2.77519 2.98518 4.58477 3.61503
0.01144 4.87222 5.59457 0.61958 0.77255 0.48576 0.95510
55 3.94500 5.91398 4.36999 3.65826 5.54374 4.38031 4.27040
4.88119 0.38971 4.21286 5.13550 3.97721 4.76293 3.41129 2.43253
3.92911 4.07754 4.57055 6.11776 5.06343 83 K - - -
2.68618 4.42225 2.77519 2.73123 3.46354 2.40513 3.72494
3.29354 2.67741 2.69355 4.24690 2.90347 2.73739 3.18146 2.89801
2.37887 2.77519 2.98518 4.58477 3.61503
0.01144 4.87222 5.59457 0.61958 0.77255 0.48576 0.95510
56 2.92900 4.42502 4.99918 4.40176 3.48519 4.28930 4.64271
1.55519 4.21417 1.45552 3.16999 4.43289 4.62646 4.35086 4.24881
3.60944 3.18915 1.66943 5.11769 3.01797 84 l - - -
2.68618 4.42225 2.77519 2.73123 3.46354 2.40513 3.72494
3.29354 2.67741 2.69355 4.24690 2.90347 2.73739 3.18146 2.89801
2.37887 2.77519 2.98518 4.58477 3.61503
0.01144 4.87222 5.59457 0.61958 0.77255 0.48576 0.95510
57 2.82901 5.15052 3.24724 2.67756 4.42309 3.21109 3.55225
3.84773 2.24799 3.17036 3.79536 3.19750 4.07302 2.86247 1.30223
2.73552 3.10757 3.50261 4.45863 4.07814 85 r - - -
2.68618 4.42225 2.77519 2.73123 3.46354 2.40513 3.72494
3.29354 2.67741 2.69355 4.24690 2.90347 2.73739 3.18146 2.89801
2.37887 2.77519 2.98518 4.58477 3.61503
0.01144 4.87222 5.59457 0.61958 0.77255 0.48576 0.95510
58 2.70219 5.29938 3.12751 2.56991 4.36714 3.30438 3.64008
4.09286 2.27414 3.59259 4.34825 2.64967 4.02413 2.80599 1.43001
2.46154 2.86476 3.68198 5.73848 4.35169 86 r - - -

```
          2.68618  4.42225  2.77519  2.73123  3.46354  2.40513  3.72494
3.29354  2.67741  2.69355  4.24690  2.90347  2.73739  3.18146  2.89801
2.37887  2.77519  2.98518  4.58477  3.61503
          0.01144  4.87222  5.59457  0.61958  0.77255  0.48576  0.95510
     59   3.96841  4.42351  5.88276  5.30357  3.52926  5.33491  5.65555
2.11531  5.15935  0.47538  2.88466  5.48485  5.40987  5.07563  5.13060
4.71677  4.17887  2.76779  5.71898  4.74345      87 L - - -
          2.68618  4.42225  2.77519  2.73123  3.46354  2.40513  3.72494
3.29354  2.67741  2.69355  4.24690  2.90347  2.73739  3.18146  2.89801
2.37887  2.77519  2.98518  4.58477  3.61503
          0.01144  4.87222  5.59457  0.61958  0.77255  0.48576  0.95510
     60   3.81369  5.88903  4.38456  3.50276  5.52620  4.33883  4.09598
4.77381  0.75736  4.08417  4.97073  3.33069  4.65419  3.21783  1.39263
3.77527  3.91130  4.45876  6.01923  4.95141      88 k - - -
          2.68618  4.42225  2.77519  2.73123  3.46354  2.40513  3.72494
3.29354  2.67741  2.69355  4.24690  2.90347  2.73739  3.18146  2.89801
2.37887  2.77519  2.98518  4.58477  3.61503
          0.01144  4.87222  5.59457  0.61958  0.77255  0.48576  0.95510
     61   2.83162  5.28653  2.92658  2.54597  4.61114  3.61288  3.68777
3.89157  1.53120  3.25559  3.35841  2.80608  4.00533  2.64093  2.20693
2.71895  2.82156  3.66624  5.73035  4.02866      89 k - - -
          2.68618  4.42225  2.77519  2.73123  3.46354  2.40513  3.72494
3.29354  2.67741  2.69355  4.24690  2.90347  2.73739  3.18146  2.89801
2.37887  2.77519  2.98518  4.58477  3.61503
          0.01144  4.87222  5.59457  0.61958  0.77255  0.48576  0.95510
     62   3.64611  5.74407  4.24861  3.38912  5.21601  4.25305  4.05830
4.56054  0.74708  3.66622  4.81241  3.74938  4.56988  3.18568  1.58082
3.62685  3.76999  4.24861  4.23118  4.78784      90 k - - -
          2.68618  4.42225  2.77519  2.73123  3.46354  2.40513  3.72494
3.29354  2.67741  2.69355  4.24690  2.90347  2.73739  3.18146  2.89801
2.37887  2.77519  2.98518  4.58477  3.61503
          0.01144  4.87222  5.59457  0.61958  0.77255  0.48576  0.95510
     63   4.47964  5.58926  5.49985  5.27016  1.12037  5.15866  3.50319
4.19992  4.26474  3.09274  4.76323  4.78381  5.44002  4.89598  4.97963
4.53155  4.68535  4.09473  4.13386  0.79632      91 y - - -
          2.68618  4.42225  2.77519  2.73123  3.46354  2.40513  3.72494
3.29354  2.67741  2.69355  4.24690  2.90347  2.73739  3.18146  2.89801
2.37887  2.77519  2.98518  4.58477  3.61503
          0.01144  4.87222  5.59457  0.61958  0.77255  0.48576  0.95510
     64   2.61726  5.31293  2.52250  2.14870  3.68175  3.11737  3.36178
4.02819  2.20525  3.23207  4.06772  2.90199  3.99513  2.72689  2.13985
2.70265  2.86212  3.22639  5.75122  4.34596      92 r - - -
          2.68618  4.42225  2.77519  2.73123  3.46354  2.40513  3.72494
3.29354  2.67741  2.69355  4.24690  2.90347  2.73739  3.18146  2.89801
2.37887  2.77519  2.98518  4.58477  3.61503
          0.03224  4.87222  3.72686  0.61958  0.77255  0.48576  0.95510
     65   2.09727  5.25189  3.03386  2.23105  4.56634  3.01002  3.57817
4.03124  2.29492  2.88287  4.13813  2.16293  3.99618  2.90116  2.88456
2.63668  2.75479  3.25600  5.70333  4.07140      93 a - - -
          2.68618  4.42225  2.77519  2.73123  3.46354  2.40513  3.72494
3.29354  2.67741  2.69355  4.24690  2.90347  2.73739  3.18146  2.89801
2.37887  2.77519  2.98518  4.58477  3.61503
          0.04637  4.85166  3.28359  0.61958  0.77255  0.53503  0.88105
     66   2.43780  3.80352  3.47014  3.01649  3.75552  3.76215  3.48901
2.37959  2.49587  2.50338  2.80784  2.58442  4.14368  3.22056  3.11182
2.88339  2.50910  2.55572  5.17840  3.93000      94 i - - -
```

```
         2.68618  4.42225  2.77519  2.73123  3.46354  2.40513  3.72494
3.29354  2.67741  2.69355  4.24690  2.90347  2.73739  3.18146  2.89801
2.37887  2.77519  2.98518  4.58477  3.61503
         0.01209  4.81738  5.53973  0.61958  0.77255  0.61012  0.78369
     67  2.14659  4.56913  3.63561  2.87289  3.51402  3.77762  4.02563
2.98240  2.94142  2.37599  3.23286  3.39034  4.15805  2.97327  2.83971
2.22840  2.79614  2.08958  4.42494  3.90508     95 v - - -
         2.68618  4.42225  2.77519  2.73123  3.46354  2.40513  3.72494
3.29354  2.67741  2.69355  4.24690  2.90347  2.73739  3.18146  2.89801
2.37887  2.77519  2.98518  4.58477  3.61503
         0.05592  4.81738  3.07259  0.61958  0.77255  0.61012  0.78369
     68  2.41658  5.25520  2.88536  2.32249  4.58232  2.98534  3.75801
3.77199  1.92314  3.21824  4.11497  2.85186  3.96209  2.76296  2.84963
1.96149  2.91529  3.42346  5.69877  4.29901     96 k - - -
         2.68618  4.42225  2.77519  2.73123  3.46354  2.40513  3.72494
3.29354  2.67741  2.69355  4.24690  2.90347  2.73739  3.18146  2.89801
2.37887  2.77519  2.98518  4.58477  3.61503
         0.03306  4.77408  3.72655  0.61958  0.77255  0.66268  0.72457
     69  2.59237  5.21007  3.17367  2.60312  3.96158  3.63712  3.67642
3.59687  1.59986  3.48715  4.26707  2.91711  4.02442  2.91674  1.73870
2.71124  3.07861  3.58314  5.65280  4.29850     97 k - - -
         2.68618  4.42225  2.77519  2.73123  3.46354  2.40513  3.72494
3.29354  2.67741  2.69355  4.24690  2.90347  2.73739  3.18146  2.89801
2.37887  2.77519  2.98518  4.58477  3.61503
         0.20122  4.76285  1.75029  0.61958  0.77255  0.71456  0.67218
     70  2.39824  4.09193  3.36450  2.38999  3.22232  3.06631  3.95340
2.15483  2.98599  2.54126  3.03936  3.27149  4.07911  3.28743  3.33956
2.61796  2.95164  2.46045  5.02188  3.78769     98 i - - -
         2.68618  4.42225  2.77519  2.73123  3.46354  2.40513  3.72494
3.29354  2.67741  2.69355  4.24690  2.90347  2.73739  3.18146  2.89801
2.37887  2.77519  2.98518  4.58477  3.61503
         0.12539  4.57704  2.22965  0.61958  0.77255  0.80404  0.59333
     71  2.36067  5.14843  2.83612  2.28455  4.46491  2.94438  3.68470
3.77535  2.14201  3.04719  4.19704  2.77813  3.88729  2.79127  2.50590
2.17231  2.94092  3.31602  5.59865  4.20611     99 k - - -
         2.68621  4.42244  2.77538  2.73105  3.46373  2.40505  3.72493
3.29373  2.67694  2.69365  4.24708  2.90365  2.73734  3.18165  2.89805
2.37864  2.77538  2.98511  4.58496  3.61522
         0.34663  1.24572  5.25888  0.91179  0.51384  0.37723  1.15760
     72  2.44477  5.29365  2.80180  2.26563  4.63126  2.85476  3.61785
4.11199  2.20728  3.33507  4.33628  2.74316  3.45438  2.86397  2.23507
2.49118  2.62851  3.33449  5.73081  3.87927    105 k - - -
         2.68618  4.42225  2.77519  2.73123  3.46354  2.40513  3.72494
3.29354  2.67741  2.69355  4.24690  2.90347  2.73739  3.18146  2.89801
2.37887  2.77519  2.98518  4.58477  3.61503
         0.01934  4.80909  4.50981  0.61958  0.77255  0.59131  0.80654
     73  2.56162  5.24424  2.82385  2.17632  4.56133  1.83971  3.61948
3.22201  2.36631  3.53740  4.29244  2.96707  3.98062  2.76333  2.66492
2.77935  2.84815  3.52537  5.06822  4.30072    106 g - - -
         2.68618  4.42225  2.77519  2.73123  3.46354  2.40513  3.72494
3.29354  2.67741  2.69355  4.24690  2.90347  2.73739  3.18146  2.89801
2.37887  2.77519  2.98518  4.58477  3.61503
         0.01216  4.81167  5.53402  0.61958  0.77255  0.57309  0.82961
     74  2.53790  5.28362  2.88909  2.16778  4.61462  2.79029  3.77475
3.76996  1.93400  2.70503  4.32805  2.99965  3.97945  2.71950  2.51661
2.59248  2.66269  3.58866  5.72472  4.32262    107 k - - -
```

```
         2.68618  4.42225  2.77519  2.73123  3.46354  2.40513  3.72494
3.29354  2.67741  2.69355  4.24690  2.90347  2.73739  3.18146  2.89801
2.37887  2.77519  2.98518  4.58477  3.61503
         0.02869  4.82475  3.89919  0.61958  0.77255  0.44595  1.02226
     75  2.32753  4.42399  1.95567  2.11548  4.64775  2.54719  3.78179
4.12846  2.32009  3.47370  4.35316  2.88914  3.73132  2.75300  2.89956
2.71928  3.04892  3.45424  5.74761  4.34111    108 d - - -
         2.68618  4.42225  2.77519  2.73123  3.46354  2.40513  3.72494
3.29354  2.67741  2.69355  4.24690  2.90347  2.73739  3.18146  2.89801
2.37887  2.77519  2.98518  4.58477  3.61503
         0.01170  4.84976  5.57211  0.61958  0.77255  0.53942  0.87488
     76  2.61961  4.91741  3.13080  2.48319  3.29661  3.47019  3.88839
3.16324  2.54746  2.54075  3.82424  2.77832  2.10810  3.00247  2.95928
2.82020  3.05199  2.65292  5.44001  4.12853    109 p - - -
         2.68618  4.42225  2.77519  2.73123  3.46354  2.40513  3.72494
3.29354  2.67741  2.69355  4.24690  2.90347  2.73739  3.18146  2.89801
2.37887  2.77519  2.98518  4.58477  3.61503
         0.01807  4.84976  4.59762  0.61958  0.77255  0.51310  0.91288
     77  2.46848  5.16064  3.02003  2.47700  3.94199  3.02537  3.67250
3.53688  2.27172  2.85941  4.21859  2.52221  3.73043  2.93732  2.99359
2.09369  2.52502  2.97941  5.63329  4.26424    110 s - - -
         2.68618  4.42225  2.77519  2.73123  3.46354  2.40513  3.72494
3.29354  2.67741  2.69355  4.24690  2.90347  2.73739  3.18146  2.89801
2.37887  2.77519  2.98518  4.58477  3.61503
         0.01170  4.85025  5.57259  0.61958  0.77255  0.51386  0.91176
     78  2.60003  4.18154  3.49774  3.04680  1.71945  3.48693  4.09685
2.89779  3.17800  2.08606  3.60378  3.42550  2.89050  3.46825  3.35795
2.76296  3.06299  2.70343  5.08672  3.86239    111 f - - -
         2.68618  4.42225  2.77519  2.73123  3.46354  2.40513  3.72494
3.29354  2.67741  2.69355  4.24690  2.90347  2.73739  3.18146  2.89801
2.37887  2.77519  2.98518  4.58477  3.61503
         0.01162  4.85650  5.57884  0.61958  0.77255  0.52375  0.89721
     79  2.51559  5.24480  2.71019  2.47117  4.55586  3.33771  3.79782
4.01851  2.09806  2.63171  3.99289  3.08882  3.54697  2.86072  2.70454
2.10928  2.42214  3.40384  5.69839  4.00462    112 k - - -
         2.68618  4.42225  2.77519  2.73123  3.46354  2.40513  3.72494
3.29354  2.67741  2.69355  4.24690  2.90347  2.73739  3.18146  2.89801
2.37887  2.77519  2.98518  4.58477  3.61503
         0.01162  4.85650  5.57884  0.61958  0.77255  0.52375  0.89721
     80  2.75611  5.33136  2.68404  2.24087  4.67391  3.47435  3.78597
4.15830  1.83403  3.54055  4.37324  2.35458  3.99135  2.88365  2.79111
2.09181  2.49360  3.72076  5.76520  4.35530    113 k - - -
         2.68618  4.42225  2.77519  2.73123  3.46354  2.40513  3.72494
3.29354  2.67741  2.69355  4.24690  2.90347  2.73739  3.18146  2.89801
2.37887  2.77519  2.98518  4.58477  3.61503
         0.01162  4.85650  5.57884  0.61958  0.77255  0.46272  0.99309
     81  2.36666  5.23130  2.61809  2.33775  4.29714  3.07526  3.81250
3.99292  2.56739  3.27345  4.28532  3.04904  1.86397  2.87714  3.05222
2.49856  2.81181  3.28280  5.69195  4.30781    114 p - - -
         2.68618  4.42225  2.77519  2.73123  3.46354  2.40513  3.72494
3.29354  2.67741  2.69355  4.24690  2.90347  2.73739  3.18146  2.89801
2.37887  2.77519  2.98518  4.58477  3.61503
         0.01144  4.87222  5.59457  0.61958  0.77255  0.48576  0.95510
     82  2.83062  5.22160  2.48831  2.34168  4.20381  3.02448  1.63924
3.80950  2.57333  3.12524  4.27537  3.10900  4.01475  2.82644  3.05699
2.63742  2.99249  3.35549  5.68339  4.30220    115 h - - -
```

2.68618 4.42225 2.77519 2.73123 3.46354 2.40513 3.72494
3.29354 2.67741 2.69355 4.24690 2.90347 2.73739 3.18146 2.89801
2.37887 2.77519 2.98518 4.58477 3.61503
0.01144 4.87222 5.59457 0.61958 0.77255 0.48576 0.95510
83 3.18453 5.74512 1.07104 1.65576 5.04738 3.67771 4.06438
4.55534 2.94402 4.03370 4.81994 3.13255 4.20487 2.83279 3.48942
3.10236 3.15986 4.11901 6.17742 3.49410 116 d - - -
2.68618 4.42225 2.77519 2.73123 3.46354 2.40513 3.72494
3.29354 2.67741 2.69355 4.24690 2.90347 2.73739 3.18146 2.89801
2.37887 2.77519 2.98518 4.58477 3.61503
0.01144 4.87222 5.59457 0.61958 0.77255 0.48576 0.95510
84 2.68766 5.31358 2.93537 2.24277 4.64904 3.42848 3.58542
3.97936 2.33039 2.98045 4.35718 3.07821 3.99963 1.98548 1.87251
2.65226 3.06069 3.51943 5.75109 4.34829 117 r - - -
2.68618 4.42225 2.77519 2.73123 3.46354 2.40513 3.72494
3.29354 2.67741 2.69355 4.24690 2.90347 2.73739 3.18146 2.89801
2.37887 2.77519 2.98518 4.58477 3.61503
0.01144 4.87222 5.59457 0.61958 0.77255 0.48576 0.95510
85 2.48328 5.31857 2.76795 2.30926 4.65648 3.60225 3.78966
3.91845 1.84604 3.53182 4.36124 2.88281 3.99555 2.52660 2.16715
2.65665 2.89428 2.81369 5.75530 4.34932 118 k - - -
2.68618 4.42225 2.77519 2.73123 3.46354 2.40513 3.72494
3.29354 2.67741 2.69355 4.24690 2.90347 2.73739 3.18146 2.89801
2.37887 2.77519 2.98518 4.58477 3.61503
0.01144 4.87222 5.59457 0.61958 0.77255 0.48576 0.95510
86 2.29392 2.76213 3.93276 3.90121 3.28839 3.60451 4.29158
2.17020 3.18399 1.86141 3.00627 4.02961 4.34282 3.93714 3.86596
2.93328 2.84285 1.94708 4.89147 3.70689 119 l - - -
2.68618 4.42225 2.77519 2.73123 3.46354 2.40513 3.72494
3.29354 2.67741 2.69355 4.24690 2.90347 2.73739 3.18146 2.89801
2.37887 2.77519 2.98518 4.58477 3.61503
0.01144 4.87222 5.59457 0.61958 0.77255 0.48576 0.95510
87 3.94331 5.19313 5.31831 4.94792 0.94782 4.84953 4.10302
3.65614 4.75753 2.36020 4.28038 4.65383 5.16112 4.70182 4.73002
3.52660 4.16473 3.35673 4.25442 1.26830 120 f - - -
2.68618 4.42225 2.77519 2.73123 3.46354 2.40513 3.72494
3.29354 2.67741 2.69355 4.24690 2.90347 2.73739 3.18146 2.89801
2.37887 2.77519 2.98518 4.58477 3.61503
0.01144 4.87222 5.59457 0.61958 0.77255 0.48576 0.95510
88 2.88701 5.40683 2.01175 1.75193 4.74966 2.95860 3.63914
4.23825 2.06960 3.70981 4.45133 3.08073 4.03101 2.21499 2.87694
2.84230 3.12106 3.79691 5.83654 4.41955 121 e - - -
2.68618 4.42225 2.77519 2.73123 3.46354 2.40513 3.72494
3.29354 2.67741 2.69355 4.24690 2.90347 2.73739 3.18146 2.89801
2.37887 2.77519 2.98518 4.58477 3.61503
0.01144 4.87222 5.59457 0.61958 0.77255 0.48576 0.95510
89 3.12062 4.48589 4.98847 4.38517 3.10809 4.29570 4.25167
2.07654 4.19378 0.82313 3.15002 4.42233 4.61856 4.29958 4.21932
3.61069 3.34798 2.59901 4.07614 3.62115 122 l - - -
2.68618 4.42225 2.77519 2.73123 3.46354 2.40513 3.72494
3.29354 2.67741 2.69355 4.24690 2.90347 2.73739 3.18146 2.89801
2.37887 2.77519 2.98518 4.58477 3.61503
0.01144 4.87222 5.59457 0.61958 0.77255 0.48576 0.95510
90 2.20860 3.36276 4.01678 3.46052 3.70293 3.52873 4.25001
3.06033 3.38793 2.64632 3.69174 3.75584 4.25132 3.66244 3.67767
1.19761 3.10167 2.75196 3.14595 3.96130 123 s - - -

```
          2.68618  4.42225  2.77519  2.73123  3.46354  2.40513  3.72494
3.29354  2.67741  2.69355  4.24690  2.90347  2.73739  3.18146  2.89801
2.37887  2.77519  2.98518  4.58477  3.61503
          0.01144  4.87222  5.59457  0.61958  0.77255  0.48576  0.95510
     91   2.84065  5.31118  3.10641  2.45922  4.64529  3.38236  3.37037
4.11822  1.51387  3.48149  3.80302  2.92206  4.00938  2.74179  2.14648
2.51415  2.85817  3.46222  5.74737  4.35102    124 k - - -
          2.68618  4.42225  2.77519  2.73123  3.46354  2.40513  3.72494
3.29354  2.67741  2.69355  4.24690  2.90347  2.73739  3.18146  2.89801
2.37887  2.77519  2.98518  4.58477  3.61503
          0.01144  4.87222  5.59457  0.61958  0.77255  0.48576  0.95510
     92   2.65896  4.82973  3.00856  2.19175  4.11325  3.61912  3.59644
3.43720  1.63019  3.17323  3.47908  3.10723  4.01134  2.85953  2.56967
2.44520  2.88409  3.40335  5.67947  4.29831    125 k - - -
          2.68618  4.42225  2.77519  2.73123  3.46354  2.40513  3.72494
3.29354  2.67741  2.69355  4.24690  2.90347  2.73739  3.18146  2.89801
2.37887  2.77519  2.98518  4.58477  3.61503
          0.01144  4.87222  5.59457  0.61958  0.77255  0.48576  0.95510
     93   3.77821  4.96543  5.87078  5.39152  4.09864  5.39741  6.06530
0.75434  5.32752  1.80601  3.82761  5.55688  5.56499  5.49363  5.44614
4.83138  4.02943  1.47682  6.26877  5.12302    126 i - - -
          2.68618  4.42225  2.77519  2.73123  3.46354  2.40513  3.72494
3.29354  2.67741  2.69355  4.24690  2.90347  2.73739  3.18146  2.89801
2.37887  2.77519  2.98518  4.58477  3.61503
          0.01144  4.87222  5.59457  0.61958  0.77255  0.48576  0.95510
     94   4.82471  5.83988  5.59691  5.47383  2.54486  5.25427  4.12538
4.51500  5.14859  3.68033  5.03916  4.92710  5.57591  5.05479  5.05632
4.76519  5.04402  4.46011  0.43290  2.01062    127 W - - -
          2.68618  4.42225  2.77519  2.73123  3.46354  2.40513  3.72494
3.29354  2.67741  2.69355  4.24690  2.90347  2.73739  3.18146  2.89801
2.37887  2.77519  2.98518  4.58477  3.61503
          0.02534  4.87222  4.05310  0.61958  0.77255  0.48576  0.95510
     95   2.63626  5.37899  2.37017  2.54092  4.72936  1.37731  3.72349
4.19995  2.73673  3.71712  4.49044  3.03600  4.09211  3.05549  3.15027
2.18134  3.06576  3.78782  5.88306  4.47802    128 g - - -
          2.68618  4.42225  2.77519  2.73123  3.46354  2.40513  3.72494
3.29354  2.67741  2.69355  4.24690  2.90347  2.73739  3.18146  2.89801
2.37887  2.77519  2.98518  4.58477  3.61503
          0.00782  4.85470        *  0.61958  0.77255  0.00000        *
//
HMMER3/f [3.3 | Nov 2019]
NAME  DUF632
ACC   PF04782.13
DESC  Protein of unknown function (DUF632)
LENG  321
ALPH  amino
RF    no
MM    no
CONS  yes
CS    no
MAP   yes
DATE  Fri Apr 17 04:55:25 2020
NSEQ  125
EFFN  2.788544
CKSUM 77574005
GA    21.40 21.40
TC    21.40 21.40
NC    21.20 21.30
```

```
STATS LOCAL MSV       -11.3569  0.70075
STATS LOCAL VITERBI   -12.1247  0.70075
STATS LOCAL FORWARD    -5.3377  0.70075
HMM          A        C        D        E        F        G        H
I        K        L        M        N        P        Q        R        S
T        V        W        Y
            m->m     m->i     m->d     i->m     i->i     d->m     d->d
  COMPO   2.57229  4.34674  3.01255  2.48388  3.54965  3.25132  3.54434
2.91065  2.50237  2.38469  3.60524  3.10866  3.64521  2.91790  2.85475
2.53619  2.87081  2.75117  4.06029  3.61864
          2.68618  4.42225  2.77519  2.73123  3.46354  2.40513  3.72494
3.29354  2.67741  2.69355  4.24690  2.90347  2.73739  3.18146  2.89801
2.37887  2.77519  2.98518  4.58477  3.61503
          0.13283  4.87023  2.14804  0.61958  0.77255  0.00000        *
      1   3.67672  4.94910  5.60086  5.00466  2.26574  4.97059  5.26921
2.47005  4.84386  0.84586  2.37472  5.12167  5.12463  4.79885  4.81431
4.32333  3.88882  1.90826  5.44674  4.41959      1 l - - -
          2.68618  4.42225  2.77519  2.73123  3.46354  2.40513  3.72494
3.29354  2.67741  2.69355  4.24690  2.90347  2.73739  3.18146  2.89801
2.37887  2.77519  2.98518  4.58477  3.61503
          0.01293  4.75033  5.47268  0.61958  0.77255  0.51183  0.91479
      2   2.27871  4.44885  3.84908  2.92510  3.18690  3.44600  3.98243
2.85008  3.00382  2.01515  2.58478  3.51901  3.90141  3.49763  2.94850
2.58043  2.89763  2.29294  4.61340  3.55230      2 l - - -
          2.68618  4.42225  2.77519  2.73123  3.46354  2.40513  3.72494
3.29354  2.67741  2.69355  4.24690  2.90347  2.73739  3.18146  2.89801
2.37887  2.77519  2.98518  4.58477  3.61503
          0.01209  4.81719  5.53954  0.61958  0.77255  0.45970  0.99825
      3   2.61749  5.36899  2.62201  1.40056  4.70952  3.11831  3.81087
4.19616  2.42254  3.67238  4.41298  2.91218  4.01053  2.59354  2.98030
2.33448  2.93889  3.75761  5.80264  4.38788      3 e - - -
          2.68618  4.42225  2.77519  2.73123  3.46354  2.40513  3.72494
3.29354  2.67741  2.69355  4.24690  2.90347  2.73739  3.18146  2.89801
2.37887  2.77519  2.98518  4.58477  3.61503
          0.01161  4.85767  5.58002  0.61958  0.77255  0.46719  0.98553
      4   2.21453  3.65423  4.21393  3.95300  3.34808  3.84034  4.34713
1.85360  3.80204  2.47132  3.43023  4.07542  4.38036  3.99063  3.91855
2.58370  3.10991  1.39778  4.94559  3.76046      4 v - - -
          2.68618  4.42225  2.77519  2.73123  3.46354  2.40513  3.72494
3.29354  2.67741  2.69355  4.24690  2.90347  2.73739  3.18146  2.89801
2.37887  2.77519  2.98518  4.58477  3.61503
          0.01146  4.87023  5.59257  0.61958  0.77255  0.48576  0.95510
      5   2.28693  4.30820  4.83612  4.23276  3.19524  3.90639  4.46436
1.92983  4.04071  1.92913  2.27571  4.25961  4.47776  4.18824  4.07977
3.43451  2.98510  1.56346  4.97205  3.79470      5 v - - -
          2.68618  4.42225  2.77519  2.73123  3.46354  2.40513  3.72494
3.29354  2.67741  2.69355  4.24690  2.90347  2.73739  3.18146  2.89801
2.37887  2.77519  2.98518  4.58477  3.61503
          0.01146  4.87023  5.59257  0.61958  0.77255  0.48576  0.95510
      6   2.54968  5.32684  2.86713  2.27635  4.52944  3.39672  3.72039
4.00310  1.49929  3.56161  4.36854  2.82733  3.99354  2.53442  2.49732
2.74768  2.88989  3.45720  5.76128  4.35307      6 k - - -
          2.68618  4.42225  2.77519  2.73123  3.46354  2.40513  3.72494
3.29354  2.67741  2.69355  4.24690  2.90347  2.73739  3.18146  2.89801
2.37887  2.77519  2.98518  4.58477  3.61503
          0.01146  4.87023  5.59257  0.61958  0.77255  0.48576  0.95510
```

```
      7   2.50106  5.45471  1.96332  1.33964  4.78887  3.23530  3.87230
4.27893  2.54573  3.75544  4.50442  3.00185  4.05938  2.77069  3.05372
2.85281  3.17127  3.66832  5.88851  4.46436      7 e - - -
          2.68618  4.42225  2.77519  2.73123  3.46354  2.40513  3.72494
3.29354  2.67741  2.69355  4.24690  2.90347  2.73739  3.18146  2.89801
2.37887  2.77519  2.98518  4.58477  3.61503
          0.01146  4.87023  5.59257  0.61958  0.77255  0.48576  0.95510
      8   3.53659  4.81733  5.49999  4.92898  3.63378  4.86915  5.24958
1.02946  4.77118  1.34083  3.46526  5.01625  5.10660  4.84835  4.79863
4.22196  3.76755  1.79537  4.68375  4.44717      8 i - - -
          2.68618  4.42225  2.77519  2.73123  3.46354  2.40513  3.72494
3.29354  2.67741  2.69355  4.24690  2.90347  2.73739  3.18146  2.89801
2.37887  2.77519  2.98518  4.58477  3.61503
          0.01146  4.87023  5.59257  0.61958  0.77255  0.48576  0.95510
      9   2.94457  5.53196  1.91490  1.42417  4.86749  3.55278  3.91160
4.36357  2.01928  3.83164  4.58418  2.81122  4.09216  2.60220  3.10956
2.93184  3.23108  3.91920  5.95959  4.52631      9 e - - -
          2.68618  4.42225  2.77519  2.73123  3.46354  2.40513  3.72494
3.29354  2.67741  2.69355  4.24690  2.90347  2.73739  3.18146  2.89801
2.37887  2.77519  2.98518  4.58477  3.61503
          0.01146  4.87023  5.59257  0.61958  0.77255  0.48576  0.95510
     10   2.60112  4.53769  2.07569  1.78998  4.43207  3.25068  3.33193
3.58403  2.47417  3.51699  4.32624  3.01185  3.99971  2.90040  2.91141
2.57268  2.67433  3.30519  5.72602  3.94993     10 e - - -
          2.68618  4.42225  2.77519  2.73123  3.46354  2.40513  3.72494
3.29354  2.67741  2.69355  4.24690  2.90347  2.73739  3.18146  2.89801
2.37887  2.77519  2.98518  4.58477  3.61503
          0.01146  4.87023  5.59257  0.61958  0.77255  0.48576  0.95510
     11   2.83256  4.94765  3.29021  2.57215  4.16321  3.68337  2.52128
3.32459  2.67553  2.28251  3.69580  3.09364  4.07150  2.09378  2.75237
2.85690  3.06358  3.26706  5.09984  2.66088     11 q - - -
          2.68618  4.42225  2.77519  2.73123  3.46354  2.40513  3.72494
3.29354  2.67741  2.69355  4.24690  2.90347  2.73739  3.18146  2.89801
2.37887  2.77519  2.98518  4.58477  3.61503
          0.01146  4.87023  5.59257  0.61958  0.77255  0.48576  0.95510
     12   3.60884  4.28040  5.20154  4.80485  0.51762  4.53552  4.37587
3.28698  4.60022  2.58578  4.04988  4.62705  4.98348  4.67014  4.63511
3.62775  3.91473  3.19438  4.59085  2.94508     12 F - - -
          2.68618  4.42225  2.77519  2.73123  3.46354  2.40513  3.72494
3.29354  2.67741  2.69355  4.24690  2.90347  2.73739  3.18146  2.89801
2.37887  2.77519  2.98518  4.58477  3.61503
          0.01146  4.87023  5.59257  0.61958  0.77255  0.48576  0.95510
     13   2.69272  4.62759  2.83122  2.44171  3.33582  3.61621  4.02774
2.59345  2.79981  1.82150  3.31978  3.47507  4.16631  3.27490  3.38614
2.60796  2.86909  2.41410  5.19947  3.95131     13 l - - -
          2.68618  4.42225  2.77519  2.73123  3.46354  2.40513  3.72494
3.29354  2.67741  2.69355  4.24690  2.90347  2.73739  3.18146  2.89801
2.37887  2.77519  2.98518  4.58477  3.61503
          0.01146  4.87023  5.59257  0.61958  0.77255  0.48576  0.95510
     14   2.84482  5.30542  3.05379  2.36261  4.63684  3.50624  3.80263
3.32175  1.62026  3.60078  4.35142  3.03528  4.01363  2.80550  1.93061
2.42577  2.91606  3.53841  5.74262  4.34971     14 k - - -
          2.68618  4.42225  2.77519  2.73123  3.46354  2.40513  3.72494
3.29354  2.67741  2.69355  4.24690  2.90347  2.73739  3.18146  2.89801
2.37887  2.77519  2.98518  4.58477  3.61503
          0.01146  4.87023  5.59257  0.61958  0.77255  0.48576  0.95510
```

```
     15   0.71525  4.57467  4.35634  3.86834  4.07205  3.60675  4.64022
2.58409  3.77763  2.99526  4.02578  4.01951  4.37023  4.05061  4.02993
3.09242  3.08193  2.61011  5.58088  4.38236      15 a - - -
          2.68618  4.42225  2.77519  2.73123  3.46354  2.40513  3.72494
3.29354  2.67741  2.69355  4.24690  2.90347  2.73739  3.18146  2.89801
2.37887  2.77519  2.98518  4.58477  3.61503
          0.01146  4.87023  5.59257  0.61958  0.77255  0.48576  0.95510
     16   1.71966  2.96280  3.89881  3.33176  3.56143  3.83526  4.01366
2.98775  3.26824  2.64969  3.61661  3.67851  4.23560  3.55042  3.57742
1.65462  2.99495  2.69047  5.09916  3.04545      16 s - - -
          2.68618  4.42225  2.77519  2.73123  3.46354  2.40513  3.72494
3.29354  2.67741  2.69355  4.24690  2.90347  2.73739  3.18146  2.89801
2.37887  2.77519  2.98518  4.58477  3.61503
          0.03104  4.87023  3.77706  0.61958  0.77255  0.48576  0.95510
     17   2.52350  5.35762  2.16648  1.65678  4.69763  2.87536  3.80499
4.18330  2.55250  3.66111  4.28087  2.44383  4.00502  2.67128  3.04894
2.59996  3.02138  3.53664  5.79251  4.37893      17 e - - -
          2.68618  4.42225  2.77519  2.73123  3.46354  2.40513  3.72494
3.29354  2.67741  2.69355  4.24690  2.90347  2.73739  3.18146  2.89801
2.37887  2.77519  2.98518  4.58477  3.61503
          0.01169  4.85088  5.57322  0.61958  0.77255  0.45782  1.00147
     18   1.78150  2.16309  3.91282  3.38205  3.53943  3.02841  4.14539
3.38587  3.33765  3.09254  3.96973  3.53680  4.21375  3.63140  3.67917
1.48179  3.02435  3.09595  5.44038  4.21439      18 s - - -
          2.68618  4.42225  2.77519  2.73123  3.46354  2.40513  3.72494
3.29354  2.67741  2.69355  4.24690  2.90347  2.73739  3.18146  2.89801
2.37887  2.77519  2.98518  4.58477  3.61503
          0.01146  4.87023  5.59257  0.61958  0.77255  0.48576  0.95510
     19   1.80134  4.26861  3.76298  3.14021  4.40124  1.08125  4.33173
3.60655  3.24228  3.46645  4.30282  3.24772  4.17968  3.55965  3.63451
2.69677  2.95891  3.31868  5.74994  4.49339      19 g - - -
          2.68618  4.42225  2.77519  2.73123  3.46354  2.40513  3.72494
3.29354  2.67741  2.69355  4.24690  2.90347  2.73739  3.18146  2.89801
2.37887  2.77519  2.98518  4.58477  3.61503
          0.01146  4.87023  5.59257  0.61958  0.77255  0.48576  0.95510
     20   2.60157  5.33209  2.80860  2.22924  4.51075  3.24039  3.10856
3.98810  1.79451  3.57693  4.24801  2.53305  3.99136  2.70971  2.55121
2.37875  2.91442  3.72221  5.76531  4.22278      20 k - - -
          2.68618  4.42225  2.77519  2.73123  3.46354  2.40513  3.72494
3.29354  2.67741  2.69355  4.24690  2.90347  2.73739  3.18146  2.89801
2.37887  2.77519  2.98518  4.58477  3.61503
          0.01146  4.87023  5.59257  0.61958  0.77255  0.48576  0.95510
     21   2.76945  5.42024  2.14514  1.27057  4.75725  3.36823  3.57728
4.24575  2.57037  3.72188  4.46712  3.08226  3.73871  2.59344  2.99408
2.81588  3.13871  3.67919  5.85345  4.43362      21 e - - -
          2.68618  4.42225  2.77519  2.73123  3.46354  2.40513  3.72494
3.29354  2.67741  2.69355  4.24690  2.90347  2.73739  3.18146  2.89801
2.37887  2.77519  2.98518  4.58477  3.61503
          0.01146  4.87023  5.59257  0.61958  0.77255  0.48576  0.95510
     22   3.33407  4.85446  5.70168  5.20525  4.13157  5.19222  5.81511
1.56347  5.12331  1.99608  3.70730  5.34073  5.41983  5.32986  5.24957
4.59780  3.89154  0.71650  6.15391  4.96661      22 v - - -
          2.68618  4.42225  2.77519  2.73123  3.46354  2.40513  3.72494
3.29354  2.67741  2.69355  4.24690  2.90347  2.73739  3.18146  2.89801
2.37887  2.77519  2.98518  4.58477  3.61503
          0.01146  4.87023  5.59257  0.61958  0.77255  0.48576  0.95510
```

```
     23   2.00142  4.75564  3.77618  3.26955  4.45657  3.35456  4.34784
3.85850  3.24968  3.08547  4.35046  3.60924  3.00615  3.56827  3.64448
0.97232  2.89781  3.23491  5.79497  4.53626      23 s - - -
          2.68618  4.42225  2.77519  2.73123  3.46354  2.40513  3.72494
3.29354  2.67741  2.69355  4.24690  2.90347  2.73739  3.18146  2.89801
2.37887  2.77519  2.98518  4.58477  3.61503
          0.01146  4.87023  5.59257  0.61958  0.77255  0.48576  0.95510
     24   2.69544  5.26952  2.95832  2.24612  4.34223  3.31370  3.79864
3.80621  2.00029  3.05914  3.67789  3.08712  3.66689  2.57298  2.09545
2.60701  2.91845  2.93205  5.71838  4.32406      24 k - - -
          2.68618  4.42225  2.77519  2.73123  3.46354  2.40513  3.72494
3.29354  2.67741  2.69355  4.24690  2.90347  2.73739  3.18146  2.89801
2.37887  2.77519  2.98518  4.58477  3.61503
          0.01146  4.87023  5.59257  0.61958  0.77255  0.48576  0.95510
     25   3.01408  4.40066  4.81696  4.21391  3.27537  4.18029  4.00264
2.33538  3.90111  1.34527  1.53831  4.28745  4.52077  4.17763  4.09426
3.48919  3.13341  2.37113  5.00410  3.84499      25 l - - -
          2.68618  4.42225  2.77519  2.73123  3.46354  2.40513  3.72494
3.29354  2.67741  2.69355  4.24690  2.90347  2.73739  3.18146  2.89801
2.37887  2.77519  2.98518  4.58477  3.61503
          0.01146  4.87023  5.59257  0.61958  0.77255  0.48576  0.95510
     26   4.32246  5.49243  6.17369  5.60627  3.14981  5.72963  5.90197
2.73289  5.46755  0.33828  3.02413  5.86852  5.63569  5.24207  5.38051
5.15762  4.51173  2.94577  5.75975  4.77599      26 L - - -
          2.68618  4.42225  2.77519  2.73123  3.46354  2.40513  3.72494
3.29354  2.67741  2.69355  4.24690  2.90347  2.73739  3.18146  2.89801
2.37887  2.77519  2.98518  4.58477  3.61503
          0.01146  4.87023  5.59257  0.61958  0.77255  0.48576  0.95510
     27   3.63962  6.35551  1.99725  0.54013  5.64061  3.75369  4.38861
5.22357  3.52156  4.66691  5.55709  3.20281  4.09324  3.56789  4.20079
3.47301  3.95139  4.74697  6.80053  5.21342      27 E - - -
          2.68618  4.42225  2.77519  2.73123  3.46354  2.40513  3.72494
3.29354  2.67741  2.69355  4.24690  2.90347  2.73739  3.18146  2.89801
2.37887  2.77519  2.98518  4.58477  3.61503
          0.01146  4.87023  5.59257  0.61958  0.77255  0.48576  0.95510
     28   1.60742  4.33205  4.32803  3.49747  3.35470  3.82088  4.27405
2.28269  3.62302  2.24673  3.45104  3.94882  4.33417  3.84361  3.80352
3.03706  2.51810  1.76523  4.95613  3.76390      28 a - - -
          2.68618  4.42225  2.77519  2.73123  3.46354  2.40513  3.72494
3.29354  2.67741  2.69355  4.24690  2.90347  2.73739  3.18146  2.89801
2.37887  2.77519  2.98518  4.58477  3.61503
          0.01146  4.87023  5.59257  0.61958  0.77255  0.48576  0.95510
     29   2.72545  4.92283  2.82190  2.53516  4.62460  2.16219  3.79574
3.95649  2.43948  3.59458  4.34176  2.33924  3.35859  2.89791  2.68704
2.06543  2.63708  3.67748  5.73938  4.33895      29 s - - -
          2.68618  4.42225  2.77519  2.73123  3.46354  2.40513  3.72494
3.29354  2.67741  2.69355  4.24690  2.90347  2.73739  3.18146  2.89801
2.37887  2.77519  2.98518  4.58477  3.61503
          0.01146  4.87023  5.59257  0.61958  0.77255  0.48576  0.95510
     30   2.84651  5.31327  2.95251  2.55374  4.64810  3.35599  3.72226
3.97417  1.47783  3.60959  4.06879  2.73184  4.01392  2.90364  1.92729
2.66203  2.96569  3.69883  5.33022  4.35421      30 k - - -
          2.68628  4.42195  2.77533  2.73137  3.46343  2.40521  3.72478
3.29361  2.67754  2.69315  4.24608  2.90360  2.73702  3.18150  2.89799
2.37897  2.77529  2.98524  4.58490  3.61507
          0.39549  1.36804  2.63049  0.45771  1.00166  0.48576  0.95510
```

```
     31   2.23215  4.34416  3.64124  3.50302  3.17553  3.87772  3.85254
2.35169  3.41127  2.14572  3.14881  3.55148  2.86710  3.54973  3.40774
2.73766  2.91285  2.06020  4.58044  3.04729     34 v - - -
          2.68618  4.42225  2.77519  2.73123  3.46354  2.40513  3.72494
3.29354  2.67741  2.69355  4.24690  2.90347  2.73739  3.18146  2.89801
2.37887  2.77519  2.98518  4.58477  3.61503
          0.18482  4.79946  1.82936  0.61958  0.77255  0.64208  0.74696
     32   2.69768  5.18786  2.93945  2.29349  4.05794  3.46565  2.59517
3.67143  2.41016  3.31987  4.23519  2.92616  3.33458  2.12246  2.30605
2.62330  2.97015  3.42861  5.63543  4.15308     35 q - - -
          2.68618  4.42225  2.77519  2.73123  3.46354  2.40513  3.72494
3.29354  2.67741  2.69355  4.24690  2.90347  2.73739  3.18146  2.89801
2.37887  2.77519  2.98518  4.58477  3.61503
          0.01461  4.62925  5.35159  0.61958  0.77255  0.42422  1.06213
     33   2.81507  4.31363  4.20125  3.61739  2.54601  3.52921  3.37837
2.69039  3.50935  2.07737  3.32893  3.75712  3.52834  3.60901  3.71451
2.57718  2.92782  2.45321  4.53833  1.87185     36 y - - -
          2.68618  4.42225  2.77519  2.73123  3.46354  2.40513  3.72494
3.29354  2.67741  2.69355  4.24690  2.90347  2.73739  3.18146  2.89801
2.37887  2.77519  2.98518  4.58477  3.61503
          0.02624  4.81020  4.03133  0.61958  0.77255  0.59759  0.79881
     34   2.73283  5.27664  2.58887  2.42398  4.25647  3.00100  2.34786
3.96835  2.47022  3.22858  4.32088  2.93883  3.97068  2.51068  2.30135
2.41438  2.92436  3.66000  5.71745  3.68650     37 r - - -
          2.68618  4.42225  2.77519  2.73123  3.46354  2.40513  3.72494
3.29354  2.67741  2.69355  4.24690  2.90347  2.73739  3.18146  2.89801
2.37887  2.77519  2.98518  4.58477  3.61503
          0.16438  4.80250  1.94232  0.61958  0.77255  0.63606  0.75369
     35   2.67366  4.89997  3.17770  2.61920  3.44796  3.51822  3.65173
3.15823  2.34293  2.85943  3.97454  3.03187  3.18470  2.77573  2.75401
1.89496  2.72435  3.22446  5.41184  3.46667     38 s - - -
          2.68618  4.42225  2.77519  2.73123  3.46354  2.40513  3.72494
3.29354  2.67741  2.69355  4.24690  2.90347  2.73739  3.18146  2.89801
2.37887  2.77519  2.98518  4.58477  3.61503
          0.01427  4.65239  5.37474  0.61958  0.77255  0.86035  0.54994
     36   2.70848  4.55818  3.09824  2.47469  3.82309  3.05521  3.45361
3.52995  2.47751  2.77422  4.10030  2.42723  3.59853  2.79017  2.57247
2.07489  2.77332  3.22412  5.52174  4.16575     39 s - - -
          2.68618  4.42225  2.77519  2.73123  3.46354  2.40513  3.72494
3.29354  2.67741  2.69355  4.24690  2.90347  2.73739  3.18146  2.89801
2.37887  2.77519  2.98518  4.58477  3.61503
          0.02709  4.65882  4.06004  0.61958  0.77255  0.87233  0.54124
     37   2.55034  4.72504  2.66422  2.65471  2.51083  3.52765  3.68308
3.22070  2.73098  2.22641  3.81248  3.09066  3.27895  2.98400  3.16029
2.47971  2.85976  2.85252  5.26805  3.80564     40 l - - -
          2.68618  4.42225  2.77519  2.73123  3.46354  2.40513  3.72494
3.29354  2.67741  2.69355  4.24690  2.90347  2.73739  3.18146  2.89801
2.37887  2.77519  2.98518  4.58477  3.61503
          0.15412  4.64610  2.01566  0.61958  0.77255  0.86130  0.54924
     38   2.11685  5.16182  2.75552  2.23537  4.48534  3.10766  3.56851
3.95635  2.22343  3.08431  4.20832  2.56301  3.77462  2.70210  2.68415
2.55832  2.82777  3.20173  5.60753  4.20999     41 a - - -
          2.68618  4.42225  2.77519  2.73123  3.46354  2.40513  3.72494
3.29354  2.67741  2.69355  4.24690  2.90347  2.73739  3.18146  2.89801
2.37887  2.77519  2.98518  4.58477  3.61503
          0.01633  4.51859  5.24094  0.61958  0.77255  0.71985  0.66714
```

39 2.52493 5.21989 2.44251 2.02270 4.55111 3.52135 3.23660
3.09415 2.39950 3.40147 4.26424 2.95105 3.12576 2.43582 2.78891
2.53636 2.83257 3.54440 5.66091 4.08469 42 e - - -
2.68618 4.42225 2.77519 2.73123 3.46354 2.40513 3.72494
3.29354 2.67741 2.69355 4.24690 2.90347 2.73739 3.18146 2.89801
2.37887 2.77519 2.98518 4.58477 3.61503
0.05609 4.64209 3.10311 0.61958 0.77255 0.48700 0.95313
40 2.75026 4.87160 3.00059 2.34644 4.56034 2.77090 3.37699
3.29097 2.25766 2.81718 4.28376 2.63770 3.39745 2.46630 2.66982
2.37691 2.74364 3.61603 5.68330 4.28607 43 k - - -
2.68618 4.42225 2.77519 2.73123 3.46354 2.40513 3.72494
3.29354 2.67741 2.69355 4.24690 2.90347 2.73739 3.18146 2.89801
2.37887 2.77519 2.98518 4.58477 3.61503
0.02025 4.75260 4.47297 0.61958 0.77255 0.63297 0.75718
41 2.65402 5.20333 3.03505 2.37240 3.85515 2.69794 3.77039
3.81782 1.71773 3.14423 4.25450 3.06393 3.97210 2.88081 2.30105
2.41387 2.93716 3.47979 5.65915 4.27349 44 k - - -
2.68618 4.42225 2.77519 2.73123 3.46354 2.40513 3.72494
3.29354 2.67741 2.69355 4.24690 2.90347 2.73739 3.18146 2.89801
2.37887 2.77519 2.98518 4.58477 3.61503
0.05637 4.77320 3.07135 0.61958 0.77255 0.66645 0.72058
42 2.53983 4.90846 2.98194 2.45854 4.26580 2.51511 3.44705
3.83856 1.88186 3.31450 4.15074 2.60964 3.95421 2.82424 2.95433
2.14373 2.88682 3.50108 5.66054 4.26914 45 k - - -
2.68618 4.42225 2.77519 2.73123 3.46354 2.40513 3.72494
3.29354 2.67741 2.69355 4.24690 2.90347 2.73739 3.18146 2.89801
2.37887 2.77519 2.98518 4.58477 3.61503
0.26772 4.73739 1.48670 0.61958 0.77255 0.75432 0.63550
43 2.55753 5.12000 2.86810 2.23547 4.09545 3.17558 2.86946
3.89387 2.28753 3.41065 4.16985 2.86361 3.87378 2.75388 2.78350
2.18823 2.34457 3.49231 5.57344 3.83047 46 s - - -
2.68618 4.42225 2.77519 2.73123 3.46354 2.40513 3.72494
3.29354 2.67741 2.69355 4.24690 2.90347 2.73739 3.18146 2.89801
2.37887 2.77519 2.98518 4.58477 3.61503
0.03597 4.48653 3.72667 0.61958 0.77255 1.06753 0.42138
44 2.57505 4.20164 3.82611 3.28556 2.89968 3.40185 4.04763
2.01864 3.22741 2.21895 3.22397 3.35227 3.97461 3.55759 3.54275
2.82751 2.73380 1.88235 4.80843 3.60911 47 v - - -
2.68618 4.42225 2.77519 2.73123 3.46354 2.40513 3.72494
3.29354 2.67741 2.69355 4.24690 2.90347 2.73739 3.18146 2.89801
2.37887 2.77519 2.98518 4.58477 3.61503
0.05742 4.46775 3.11617 0.61958 0.77255 1.08492 0.41238
45 2.55028 4.75051 2.55818 2.59996 3.16822 3.53513 3.52734
3.30206 2.51427 2.74724 3.83112 3.02251 3.92305 2.92950 2.62050
2.60079 2.90558 3.01829 5.27461 2.52762 48 k - - -
2.68618 4.42225 2.77519 2.73123 3.46354 2.40513 3.72494
3.29354 2.67741 2.69355 4.24690 2.90347 2.73739 3.18146 2.89801
2.37887 2.77519 2.98518 4.58477 3.61503
0.01789 4.42823 5.15057 0.61958 0.77255 1.11957 0.39515
46 2.70507 5.14165 2.93066 2.35529 4.46773 3.48114 2.35215
3.76386 2.15033 3.35918 4.19016 2.95958 3.60522 2.50948 2.35067
2.35496 2.93344 3.52269 5.57939 4.19584 49 k - - -
2.68618 4.42225 2.77519 2.73123 3.46354 2.40513 3.72494
3.29354 2.67741 2.69355 4.24690 2.90347 2.73739 3.18146 2.89801
2.37887 2.77519 2.98518 4.58477 3.61503
0.38129 4.42823 1.18717 0.61958 0.77255 0.54469 0.86755

```
     47   2.29717  4.28100  3.04404  2.32464  4.18528  3.18588  3.41728
3.48838  2.12314  3.19656  3.99715  3.01235  3.37138  2.83345  2.65282
2.51006  2.90685  2.68349  5.42234  3.91710     50 k - - -
          2.68655  4.41500  2.77675  2.73282  3.46287  2.40639  3.72512
3.29349  2.67818  2.69216  4.24418  2.90558  2.73670  3.18271  2.89723
2.37621  2.77566  2.98383  4.58678  3.61618
          0.77846  0.62530  5.15155  3.07068  0.04750  1.04308  0.43443
     48   2.36306  5.09210  2.83300  2.19122  4.16067  3.34200  3.58783
3.61624  2.17228  3.37982  4.01981  2.88722  3.53019  2.74127  2.66215
2.24138  2.75623  3.07492  5.54996  3.99358     89 k - - -
          2.68618  4.42225  2.77519  2.73123  3.46354  2.40513  3.72494
3.29354  2.67741  2.69355  4.24690  2.90347  2.73739  3.18146  2.89801
2.37887  2.77519  2.98518  4.58477  3.61503
          0.12085  4.45910  2.28020  0.61958  0.77255  0.92862  0.50269
     49   2.54735  4.67148  2.82924  2.26122  4.30614  2.49142  3.67343
3.64581  2.41228  3.16733  4.08110  2.87951  2.90102  2.79496  2.87926
2.25615  2.85030  3.23083  5.49523  4.01429     90 s - - -
          2.68618  4.42225  2.77519  2.73123  3.46354  2.40513  3.72494
3.29354  2.67741  2.69355  4.24690  2.90347  2.73739  3.18146  2.89801
2.37887  2.77519  2.98518  4.58477  3.61503
          0.03774  4.42884  3.68442  0.61958  0.77255  1.07342  0.41830
     50   2.41767  5.02425  2.96050  2.40059  4.31037  3.15848  3.67418
3.75489  2.35679  3.09760  4.08460  2.26419  3.48757  2.79560  2.68785
2.21189  2.86584  2.99171  5.49797  4.12807     91 s - - -
          2.68618  4.42225  2.77519  2.73123  3.46354  2.40513  3.72494
3.29354  2.67741  2.69355  4.24690  2.90347  2.73739  3.18146  2.89801
2.37887  2.77519  2.98518  4.58477  3.61503
          0.19327  4.42755  1.80912  0.61958  0.77255  0.57981  0.82099
     51   2.26089  4.02895  3.34152  2.73612  3.78816  3.61737  3.52622
3.01756  2.61067  2.84519  3.40493  3.24828  3.73437  3.08369  3.08568
1.74610  2.85538  2.75627  5.17253  3.51669     92 s - - -
          2.68618  4.42225  2.77519  2.73123  3.46354  2.40513  3.72494
3.29354  2.67741  2.69355  4.24690  2.90347  2.73739  3.18146  2.89801
2.37887  2.77519  2.98518  4.58477  3.61503
          0.03421  4.54011  3.77416  0.61958  0.77255  0.51281  0.91332
     52   2.08227  5.14857  3.03835  2.38941  4.44169  3.18886  3.53309
3.68199  2.37788  3.31774  3.61791  2.83706  3.82324  2.37964  2.91497
2.36985  2.64086  2.87174  5.61366  4.23645     93 a - - -
          2.68618  4.42225  2.77519  2.73123  3.46354  2.40513  3.72494
3.29354  2.67741  2.69355  4.24690  2.90347  2.73739  3.18146  2.89801
2.37887  2.77519  2.98518  4.58477  3.61503
          0.02441  4.72843  4.18152  0.61958  0.77255  0.69954  0.68679
     53   2.33016  4.57923  3.05684  2.50033  4.52718  3.19654  3.42003
3.76707  1.74348  3.50408  4.10553  2.68699  3.75846  2.78193  2.47448
2.29881  3.00800  3.58648  5.22279  4.27128     94 k - - -
          2.68618  4.42225  2.77519  2.73123  3.46354  2.40513  3.72494
3.29354  2.67741  2.69355  4.24690  2.90347  2.73739  3.18146  2.89801
2.37887  2.77519  2.98518  4.58477  3.61503
          0.01309  4.73799  5.46034  0.61958  0.77255  0.75332  0.63639
     54   2.57674  4.45365  3.49086  2.83757  3.28854  3.58261  4.04313
2.64981  3.11845  2.28158  2.67351  3.26725  4.04994  3.41005  2.89100
2.68238  2.74404  1.90145  5.04189  3.81646     95 v - - -
          2.68618  4.42225  2.77519  2.73123  3.46354  2.40513  3.72494
3.29354  2.67741  2.69355  4.24690  2.90347  2.73739  3.18146  2.89801
2.37887  2.77519  2.98518  4.58477  3.61503
          0.01309  4.73799  5.46034  0.61958  0.77255  0.75332  0.63639
```

55 2.53679 4.29409 3.67922 3.54847 2.80375 3.78244 4.16012
2.47259 3.44629 1.79112 2.61822 2.99477 4.23713 3.68430 3.38739
2.71885 2.74485 2.18338 4.90172 3.70428 96 l - - -
2.68618 4.42225 2.77519 2.73123 3.46354 2.40513 3.72494
3.29354 2.67741 2.69355 4.24690 2.90347 2.73739 3.18146 2.89801
2.37887 2.77519 2.98518 4.58477 3.61503
0.02176 4.73799 4.36077 0.61958 0.77255 0.71567 0.67112
56 2.54552 5.27862 3.03853 2.46613 4.61886 3.18902 3.38685
4.09818 1.96108 3.57986 4.32176 2.32113 3.86845 2.59026 2.50685
2.02992 2.97471 3.66693 5.71247 4.30901 97 k - - -
2.68618 4.42225 2.77519 2.73123 3.46354 2.40513 3.72494
3.29354 2.67741 2.69355 4.24690 2.90347 2.73739 3.18146 2.89801
2.37887 2.77519 2.98518 4.58477 3.61503
0.01306 4.74035 5.46269 0.61958 0.77255 0.74939 0.63990
57 2.11437 4.53823 3.50456 3.03773 3.56530 3.74614 3.82138
2.88283 2.96371 2.67974 3.53352 3.20884 2.74158 3.30568 3.36584
2.53407 2.49790 2.12955 5.11530 3.01905 98 a - - -
2.68618 4.42225 2.77519 2.73123 3.46354 2.40513 3.72494
3.29354 2.67741 2.69355 4.24690 2.90347 2.73739 3.18146 2.89801
2.37887 2.77519 2.98518 4.58477 3.61503
0.01888 4.74035 4.60882 0.61958 0.77255 0.74939 0.63990
58 2.59513 4.20685 4.64159 4.03771 3.11741 3.97523 4.30563
1.69384 3.85440 1.61677 3.31944 4.09078 4.15201 4.01259 3.91338
2.98600 2.94244 1.90023 4.84027 3.33099 99 l - - -
2.68618 4.42225 2.77519 2.73123 3.46354 2.40513 3.72494
3.29354 2.67741 2.69355 4.24690 2.90347 2.73739 3.18146 2.89801
2.37887 2.77519 2.98518 4.58477 3.61503
0.02622 4.73461 4.06935 0.61958 0.77255 0.75891 0.63145
59 2.06869 3.88512 3.18705 2.81183 3.89828 3.66936 3.76661
3.22845 2.79097 2.91271 3.81105 2.99965 3.81354 3.12260 3.21280
2.07706 2.01787 2.78974 4.50340 3.99125 100 t - - -
2.68618 4.42225 2.77520 2.73123 3.46347 2.40513 3.72495
3.29354 2.67741 2.69352 4.24690 2.90347 2.73740 3.18147 2.89801
2.37887 2.77520 2.98519 4.58477 3.61503
0.11938 3.78911 2.40890 0.51427 0.91115 0.77979 0.61342
60 2.61995 3.60084 4.06137 3.39971 3.38095 3.36457 4.11591
2.72933 3.37766 2.09999 3.28582 3.55373 4.18926 3.62556 3.60431
2.74807 2.97621 2.50171 1.68602 3.67806 102 w - - -
2.68618 4.42225 2.77519 2.73123 3.46354 2.40513 3.72494
3.29354 2.67741 2.69355 4.24690 2.90347 2.73739 3.18146 2.89801
2.37887 2.77519 2.98518 4.58477 3.61503
0.02627 4.63223 4.12330 0.61958 0.77255 0.90745 0.51676
61 2.66046 5.07386 3.00178 2.47302 3.55527 3.16147 2.65274
3.62070 2.37597 3.00709 4.13230 2.37033 3.81181 2.73085 2.97974
2.17433 2.75844 3.34025 5.54752 4.17962 103 s - - -
2.68618 4.42225 2.77519 2.73123 3.46354 2.40513 3.72494
3.29354 2.67741 2.69355 4.24690 2.90347 2.73739 3.18146 2.89801
2.37887 2.77519 2.98518 4.58477 3.61503
0.02161 4.62070 4.46293 0.61958 0.77255 0.88630 0.53133
62 2.74264 4.98292 2.98936 2.51850 4.03657 3.56409 3.51648
3.65692 2.45685 3.17556 3.87409 3.02616 3.62915 2.85942 1.73463
2.53457 2.97338 3.32198 2.95479 3.87783 104 r - - -
2.68618 4.42225 2.77519 2.73123 3.46354 2.40513 3.72494
3.29354 2.67741 2.69355 4.24690 2.90347 2.73739 3.18146 2.89801
2.37887 2.77519 2.98518 4.58477 3.61503
0.01466 4.62553 5.34788 0.61958 0.77255 0.91596 0.51105

63 2.57076 4.90846 3.01067 2.60553 4.15265 3.24528 3.78934
3.56555 2.59637 3.17711 3.99485 3.11937 3.96675 2.94589 2.77975
1.52183 2.47347 3.09727 4.93339 3.90819 105 s - - -
2.68618 4.42225 2.77519 2.73123 3.46354 2.40513 3.72494
3.29354 2.67741 2.69355 4.24690 2.90347 2.73739 3.18146 2.89801
2.37887 2.77519 2.98518 4.58477 3.61503
0.01876 4.62553 4.73444 0.61958 0.77255 0.91596 0.51105
64 2.45224 4.55572 3.47280 2.81988 2.76597 3.09729 3.91632
2.90308 2.78067 2.52853 3.43718 2.96045 3.26347 3.19383 3.26585
2.21905 2.60539 2.55940 5.12392 3.87062 106 s - - -
2.68619 4.42226 2.77521 2.73115 3.46355 2.40509 3.72496
3.29355 2.67742 2.69356 4.24691 2.90348 2.73741 3.18148 2.89802
2.37884 2.77521 2.98516 4.58478 3.61504
0.26644 2.70988 1.78766 0.35212 1.21468 0.88773 0.53033
65 2.69367 5.01727 3.05466 2.46420 3.93371 3.30467 3.71112
3.73997 1.91557 3.24332 4.09437 3.03035 3.91374 2.84128 2.70373
1.67816 2.95643 3.38341 5.49804 4.14998 108 s - - -
2.68618 4.42225 2.77519 2.73123 3.46354 2.40513 3.72494
3.29354 2.67741 2.69355 4.24690 2.90347 2.73739 3.18146 2.89801
2.37887 2.77519 2.98518 4.58477 3.61503
0.03931 4.45687 3.61373 0.61958 0.77255 1.07206 0.41901
66 2.57210 4.70382 3.24136 2.76381 4.27579 2.59939 3.94194
3.69545 2.71509 3.13146 4.13193 3.12432 3.09562 3.11812 3.20859
1.31656 2.94770 3.17950 5.55873 4.25544 109 s - - -
2.68618 4.42225 2.77519 2.73123 3.46354 2.40513 3.72494
3.29354 2.67741 2.69355 4.24690 2.90347 2.73739 3.18146 2.89801
2.37887 2.77519 2.98518 4.58477 3.61503
0.01761 4.44408 5.16643 0.61958 0.77255 1.10598 0.40180
67 2.64256 4.76334 3.17047 2.60891 3.97151 3.54420 3.39238
3.18396 2.47790 2.39108 3.56203 3.11002 3.14571 2.72263 2.14989
2.72704 2.75136 2.89579 5.28662 3.98108 110 r - - -
2.68664 4.42265 2.77596 2.73184 3.46309 2.40564 3.72547
3.29377 2.67786 2.69314 4.24708 2.90362 2.73735 3.18205 2.89774
2.37596 2.77564 2.98575 4.58535 3.61536
0.56337 0.92563 3.36885 2.01270 0.14344 0.89389 0.52604
68 2.38110 5.04917 2.90175 2.42810 4.33387 3.20785 3.57096
3.77918 2.45800 2.85290 3.93741 2.73092 3.09393 2.54568 2.88004
2.16033 2.64965 3.22108 5.52022 4.14918 121 s - - -
2.68618 4.42225 2.77519 2.73123 3.46354 2.40513 3.72494
3.29354 2.67741 2.69355 4.24690 2.90347 2.73739 3.18146 2.89801
2.37887 2.77519 2.98518 4.58477 3.61503
0.06498 4.50343 2.95957 0.61958 0.77255 0.99482 0.46170
69 2.31387 4.55535 3.04454 2.54962 3.93689 3.37760 3.73302
3.42566 2.47303 3.11580 3.59399 2.55083 3.37339 2.78643 3.00186
2.04334 2.72062 2.90624 5.36943 3.89991 122 s - - -
2.68618 4.42225 2.77519 2.73123 3.46354 2.40513 3.72494
3.29354 2.67741 2.69355 4.24690 2.90347 2.73739 3.18146 2.89801
2.37887 2.77519 2.98518 4.58477 3.61503
0.02768 4.47747 4.13912 0.61958 0.77255 1.05713 0.42687
70 2.34714 4.45164 3.10858 2.51980 4.08656 3.16887 3.73266
3.29818 2.28575 2.87377 3.82175 2.74674 3.62305 2.80043 2.93681
2.20379 2.64852 2.81906 5.36604 4.03823 123 s - - -
2.68618 4.42225 2.77519 2.73123 3.46354 2.40513 3.72494
3.29354 2.67741 2.69355 4.24690 2.90347 2.73739 3.18146 2.89801
2.37887 2.77519 2.98518 4.58477 3.61503
0.02506 4.47406 4.31629 0.61958 0.77255 1.07915 0.41534

```
     71   2.68933  5.15974  2.56390  2.32512  4.48891  3.18923  3.44839
3.96315  1.85962  3.27289  4.05878  2.76640  3.86170  2.75868  2.57594
2.29926  2.76657  3.54186  4.97664  4.20143    124 k - - -
          2.68618  4.42225  2.77519  2.73123  3.46354  2.40513  3.72494
3.29354  2.67741  2.69355  4.24690  2.90347  2.73739  3.18146  2.89801
2.37887  2.77519  2.98518  4.58477  3.61503
          0.01722  4.46621  5.18856  0.61958  0.77255  1.03474  0.43900
     72   2.64391  5.14429  2.06594  2.29500  4.46243  3.06964  3.56368
3.93037  2.39251  3.35076  4.19255  2.68457  3.87276  2.73349  2.81310
2.28363  2.83654  3.26577  5.59316  3.45390    125 d - - -
          2.68618  4.42225  2.77519  2.73123  3.46354  2.40513  3.72494
3.29354  2.67741  2.69355  4.24690  2.90347  2.73739  3.18146  2.89801
2.37887  2.77519  2.98518  4.58477  3.61503
          0.01688  4.48572  5.20806  0.61958  0.77255  1.01257  0.45143
     73   2.45009  5.07546  2.42838  2.41405  4.21094  2.68278  3.68865
3.59968  2.37672  3.19866  4.13030  2.98626  3.88723  2.75053  2.55819
2.26562  2.75159  3.15179  5.54059  4.02113    126 s - - -
          2.68618  4.42225  2.77519  2.73123  3.46354  2.40513  3.72494
3.29354  2.67741  2.69355  4.24690  2.90347  2.73739  3.18146  2.89801
2.37887  2.77519  2.98518  4.58477  3.61503
          0.02345  4.50641  4.41093  0.61958  0.77255  1.04841  0.43155
     74   2.49466  4.87190  2.81415  2.51634  3.67707  3.33848  3.62761
3.22399  2.26212  2.86395  3.61501  2.93705  3.62077  2.83551  2.85295
2.49633  2.49088  2.69413  5.37944  3.89852    127 k - - -
          2.68618  4.42225  2.77519  2.73123  3.46354  2.40513  3.72494
3.29354  2.67741  2.69355  4.24690  2.90347  2.73739  3.18146  2.89801
2.37887  2.77519  2.98518  4.58477  3.61503
          0.02587  4.49961  4.23868  0.61958  0.77255  1.05504  0.42799
     75   2.60248  5.11005  2.02612  1.99636  4.12939  2.96848  3.60297
3.87264  2.43838  3.39783  4.16263  2.93173  3.70629  2.75896  2.92429
2.59013  2.86789  3.38830  3.70618  4.18214    128 e - - -
          2.68618  4.42225  2.77519  2.73123  3.46354  2.40513  3.72494
3.29354  2.67741  2.69355  4.24690  2.90347  2.73739  3.18146  2.89801
2.37887  2.77519  2.98518  4.58477  3.61503
          0.01680  4.49054  5.21288  0.61958  0.77255  0.99802  0.45983
     76   2.59194  5.15894  2.23133  2.13146  4.21606  3.04608  3.67410
3.95264  2.36189  3.26921  3.93761  2.78615  3.62491  2.77807  2.80828
2.32480  2.60631  3.38925  5.60516  4.20784    129 e - - -
          2.68618  4.42225  2.77519  2.73123  3.46354  2.40513  3.72494
3.29354  2.67741  2.69355  4.24690  2.90347  2.73739  3.18146  2.89801
2.37887  2.77519  2.98518  4.58477  3.61503
          0.01639  4.51490  5.23725  0.61958  0.77255  0.70936  0.67719
     77   2.67375  4.56504  2.94949  2.38201  3.03770  3.12799  3.56357
3.26928  2.63275  2.70749  3.52352  2.63906  3.40990  2.94770  3.00345
2.34477  2.84070  3.00486  5.37676  3.36121    130 s - - -
          2.68618  4.42225  2.77519  2.73123  3.46354  2.40513  3.72494
3.29354  2.67741  2.69355  4.24690  2.90347  2.73739  3.18146  2.89801
2.37887  2.77519  2.98518  4.58477  3.61503
          0.02879  4.64495  3.97562  0.61958  0.77255  0.89092  0.52810
     78   2.55087  4.41664  2.69681  2.20285  3.52847  2.99016  3.51609
3.48468  2.44262  2.99773  3.91782  2.83105  3.94096  2.82991  2.99864
2.20290  2.64105  3.20912  5.51839  3.82354    131 e - - -
          2.68618  4.42225  2.77519  2.73123  3.46354  2.40513  3.72494
3.29354  2.67741  2.69355  4.24690  2.90347  2.73739  3.18146  2.89801
2.37887  2.77519  2.98518  4.58477  3.61503
          0.03062  4.63075  3.89165  0.61958  0.77255  0.90934  0.51548
```

```
     79   2.70737  5.17077  2.68070  2.23944  3.97429  2.76147  3.70947
3.71669  2.25602  2.93863  4.21934  2.75550  3.52201  2.77081  2.88267
2.07403  2.81547  3.54541  5.62157  3.93113    132 s - - -
          2.68618  4.42225  2.77519  2.73123  3.46354  2.40513  3.72494
3.29354  2.67741  2.69355  4.24690  2.90347  2.73739  3.18146  2.89801
2.37887  2.77519  2.98518  4.58477  3.61503
          0.17548  4.61495  1.89020  0.61958  0.77255  0.92915  0.50234
     80   2.54897  5.11714  2.27633  2.16340  4.15162  3.21086  3.66421
3.81634  2.41433  3.07938  4.16704  2.55478  3.86529  2.77247  2.84520
2.17778  2.87786  3.41891  5.57003  4.00395    133 e - - -
          2.68660  4.42209  2.77455  2.73116  3.46185  2.40532  3.72546
3.29328  2.67779  2.69350  4.24660  2.90365  2.73776  3.18198  2.89822
2.37847  2.77535  2.98499  4.58499  3.61495
          0.84196  0.57358  5.17919  1.31467  0.31274  0.97224  0.47519
     81   2.62613  5.20145  2.25390  1.72454  4.52956  2.84464  3.68536
3.75194  2.39674  3.49972  4.24884  2.83378  3.88432  2.70049  2.88865
2.59621  2.90201  3.31675  5.64364  4.23821    147 e - - -
          2.68618  4.42225  2.77519  2.73123  3.46354  2.40513  3.72494
3.29354  2.67741  2.69355  4.24690  2.90347  2.73739  3.18146  2.89801
2.37887  2.77519  2.98518  4.58477  3.61503
          0.01656  4.50463  5.22698  0.61958  0.77255  0.73141  0.65629
     82   2.73858  4.92499  2.35365  1.94690  4.26619  2.96566  3.42250
3.79502  2.48153  3.35564  4.08229  2.69662  3.92459  2.83927  2.90873
2.50174  2.96980  3.13832  4.10340  3.19499    148 e - - -
          2.68618  4.42225  2.77519  2.73123  3.46354  2.40513  3.72494
3.29354  2.67741  2.69355  4.24690  2.90347  2.73739  3.18146  2.89801
2.37887  2.77519  2.98518  4.58477  3.61503
          0.01459  4.63075  5.35309  0.61958  0.77255  0.88031  0.53555
     83   2.65967  4.02709  3.12607  2.06876  2.94546  2.78237  3.59713
3.27336  2.36173  2.90754  3.77939  3.01378  3.85509  2.80667  3.00753
2.45987  2.93240  3.02592  5.41079  3.97801    149 e - - -
          2.68618  4.42225  2.77519  2.73123  3.46354  2.40513  3.72494
3.29354  2.67741  2.69355  4.24690  2.90347  2.73739  3.18146  2.89801
2.37887  2.77519  2.98518  4.58477  3.61503
          0.01445  4.64000  5.36235  0.61958  0.77255  0.88168  0.53458
     84   2.50822  2.72536  3.19652  2.64705  3.96817  2.45653  3.83588
3.28901  2.63887  3.01195  3.77772  3.13271  3.32953  2.99243  2.94099
2.51524  2.50137  2.95090  5.30808  3.44742    150 g - - -
          2.68618  4.42225  2.77519  2.73123  3.46354  2.40513  3.72494
3.29354  2.67741  2.69355  4.24690  2.90347  2.73739  3.18146  2.89801
2.37887  2.77519  2.98518  4.58477  3.61503
          0.17828  4.64495  1.87285  0.61958  0.77255  0.80847  0.58976
     85   2.54240  3.40276  4.12306  3.53791  3.18270  3.09458  3.87316
2.49812  3.42359  2.30664  1.77713  3.67330  4.16219  3.64968  3.61603
2.81408  2.76616  2.30851  4.79562  3.60207    151 m - - -
          2.68618  4.42225  2.77519  2.73123  3.46354  2.40513  3.72494
3.29354  2.67741  2.69355  4.24690  2.90347  2.73739  3.18146  2.89801
2.37887  2.77519  2.98518  4.58477  3.61503
          0.01640  4.51453  5.23688  0.61958  0.77255  0.54030  0.87365
     86   2.67516  5.03962  2.78134  2.44476  3.72957  2.63419  3.78677
2.67311  2.41851  3.02007  3.79503  2.67251  3.97801  2.92246  2.41561
2.53780  2.96370  3.05328  5.52826  4.17699    152 r - - -
          2.68618  4.42225  2.77519  2.73123  3.46354  2.40513  3.72494
3.29354  2.67741  2.69355  4.24690  2.90347  2.73739  3.18146  2.89801
2.37887  2.77519  2.98518  4.58477  3.61503
          0.01331  4.72158  5.44393  0.61958  0.77255  0.72256  0.66458
```

```
     87   2.36248  4.18289  3.40952  2.84780  3.46475  3.31645  3.75445
3.24522  2.82451  2.85935  3.78715  3.16611  3.09142  3.06504  3.23933
1.60185  2.87452  2.76738  5.24899  3.48027    153 s - - -
          2.68618  4.42225  2.77519  2.73123  3.46354  2.40513  3.72494
3.29354  2.67741  2.69355  4.24690  2.90347  2.73739  3.18146  2.89801
2.37887  2.77519  2.98518  4.58477  3.61503
          0.01309  4.73848  5.46082  0.61958  0.77255  0.73144  0.65627
     88   2.71157  5.04065  3.07772  2.56542  4.29836  1.58713  3.80383
3.61653  2.37471  3.30808  4.10931  3.04310  3.99543  2.94243  2.63854
2.55585  2.97958  2.99401  4.98794  4.18633    154 g - - -
          2.68618  4.42225  2.77519  2.73123  3.46354  2.40513  3.72494
3.29354  2.67741  2.69355  4.24690  2.90347  2.73739  3.18146  2.89801
2.37887  2.77519  2.98518  4.58477  3.61503
          0.01301  4.74449  5.46684  0.61958  0.77255  0.66811  0.71882
     89   2.62962  5.17467  2.98421  2.55752  4.48658  3.40868  3.43532
3.93668  2.51840  3.47935  4.06367  2.32453  3.99866  2.93496  3.06177
1.35576  3.05354  3.46581  5.66444  4.28881    155 s - - -
          2.68618  4.42225  2.77519  2.73123  3.46354  2.40513  3.72494
3.29354  2.67741  2.69355  4.24690  2.90347  2.73739  3.18146  2.89801
2.37887  2.77519  2.98518  4.58477  3.61503
          0.01273  4.76599  5.48834  0.61958  0.77255  0.36964  1.17436
     90   3.31720  4.94736  4.14404  3.67026  3.34667  4.19481  1.18954
3.25079  3.32538  1.40047  3.95699  3.82768  4.59870  3.80756  3.55564
3.54055  3.55954  3.19468  4.94616  3.43436    156 h - - -
          2.68618  4.42225  2.77519  2.73123  3.46354  2.40513  3.72494
3.29354  2.67741  2.69355  4.24690  2.90347  2.73739  3.18146  2.89801
2.37887  2.77519  2.98518  4.58477  3.61503
          0.01146  4.87023  5.59257  0.61958  0.77255  0.48576  0.95510
     91   2.03404  2.92004  3.37336  2.81624  4.08051  3.38936  3.81993
3.37827  2.71192  3.04120  3.96883  3.30408  4.09724  2.70925  3.23299
1.57483  2.93568  3.18768  5.41836  4.12315    157 s - - -
          2.68618  4.42225  2.77519  2.73123  3.46354  2.40513  3.72494
3.29354  2.67741  2.69355  4.24690  2.90347  2.73739  3.18146  2.89801
2.37887  2.77519  2.98518  4.58477  3.61503
          0.01146  4.87023  5.59257  0.61958  0.77255  0.48576  0.95510
     92   2.67224  4.11033  3.58284  3.01880  3.68686  3.53418  4.02130
3.11277  2.88309  2.59820  3.38860  3.45892  4.16000  3.08986  3.37169
1.32520  2.47203  2.88762  5.22088  3.96904    158 s - - -
          2.68618  4.42225  2.77519  2.73123  3.46354  2.40513  3.72494
3.29354  2.67741  2.69355  4.24690  2.90347  2.73739  3.18146  2.89801
2.37887  2.77519  2.98518  4.58477  3.61503
          0.01146  4.87023  5.59257  0.61958  0.77255  0.48576  0.95510
     93   2.78123  4.61970  4.38959  3.94371  4.27029  3.70395  4.75129
3.03354  3.84427  3.25957  4.21514  4.04658  4.36514  4.13288  4.09432
2.85056  0.67641  2.47888  5.76952  4.57322    159 T - - -
          2.68618  4.42225  2.77519  2.73123  3.46354  2.40513  3.72494
3.29354  2.67741  2.69355  4.24690  2.90347  2.73739  3.18146  2.89801
2.37887  2.77519  2.98518  4.58477  3.61503
          0.01146  4.87023  5.59257  0.61958  0.77255  0.48576  0.95510
     94   4.16705  5.36943  6.04553  5.49365  3.55027  5.57128  5.90615
2.56950  5.33082  0.39350  3.21958  5.73359  5.57593  5.22330  5.30001
4.99744  4.37977  2.35462  5.84367  4.88926    160 L - - -
          2.68618  4.42225  2.77519  2.73123  3.46354  2.40513  3.72494
3.29354  2.67741  2.69355  4.24690  2.90347  2.73739  3.18146  2.89801
2.37887  2.77519  2.98518  4.58477  3.61503
          0.01146  4.87023  5.59257  0.61958  0.77255  0.48576  0.95510
```

```
     95   2.96895  5.50456  1.61692  1.57679  4.84048  3.04413  3.65326
4.33462  2.40506  3.80469  4.55534  3.09387  4.07981  2.52420  2.98269
2.91369  3.20851  3.89176  5.93351  4.50366    161 e - - -
          2.68618  4.42225  2.77519  2.73123  3.46354  2.40513  3.72494
3.29354  2.67741  2.69355  4.24690  2.90347  2.73739  3.18146  2.89801
2.37887  2.77519  2.98518  4.58477  3.61503
          0.01146  4.87023  5.59257  0.61958  0.77255  0.48576  0.95510
     96   3.65177  5.80099  4.17878  3.35480  5.37843  4.23868  4.05379
4.66335  0.99229  4.00758  4.87080  3.72570  4.56211  2.89416  1.21407
3.43117  3.33113  4.33105  5.97670  4.86234    162 k - - -
          2.68618  4.42225  2.77519  2.73123  3.46354  2.40513  3.72494
3.29354  2.67741  2.69355  4.24690  2.90347  2.73739  3.18146  2.89801
2.37887  2.77519  2.98518  4.58477  3.61503
          0.01146  4.87023  5.59257  0.61958  0.77255  0.48576  0.95510
     97   4.32108  5.48611  6.19971  5.62463  3.49602  5.76001  6.00384
2.54641  5.47300  0.35039  2.83892  5.91193  5.65216  5.26348  5.39178
5.19359  4.51082  2.93799  5.83680  4.94034    163 L - - -
          2.68618  4.42225  2.77519  2.73123  3.46354  2.40513  3.72494
3.29354  2.67741  2.69355  4.24690  2.90347  2.73739  3.18146  2.89801
2.37887  2.77519  2.98518  4.58477  3.61503
          0.01146  4.87023  5.59257  0.61958  0.77255  0.48576  0.95510
     98   3.23903  3.67232  4.90166  4.36848  2.47435  4.32040  3.38180
3.06426  4.18839  1.93451  3.72954  4.07710  4.67031  4.28565  4.23178
3.63496  3.46801  2.82406  3.67767  1.03116    164 y - - -
          2.68618  4.42225  2.77519  2.73123  3.46354  2.40513  3.72494
3.29354  2.67741  2.69355  4.24690  2.90347  2.73739  3.18146  2.89801
2.37887  2.77519  2.98518  4.58477  3.61503
          0.01146  4.87023  5.59257  0.61958  0.77255  0.48576  0.95510
     99   1.32105  4.46385  3.76649  3.06241  3.39628  3.86470  4.13586
2.82689  3.26170  2.44869  2.97268  3.27152  4.24091  3.54138  3.56679
3.12350  2.88056  2.21870  5.06063  3.84387    165 a - - -
          2.68618  4.42225  2.77519  2.73123  3.46354  2.40513  3.72494
3.29354  2.67741  2.69355  4.24690  2.90347  2.73739  3.18146  2.89801
2.37887  2.77519  2.98518  4.58477  3.61503
          0.01146  4.87023  5.59257  0.61958  0.77255  0.48576  0.95510
    100   3.00403  5.16136  3.83679  2.92794  3.83847  4.07240  4.45149
3.83752  3.42370  3.45515  4.46608  3.94294  4.62163  3.92054  3.66845
3.56969  3.72112  3.60404  0.60292  3.87271    166 W - - -
          2.68618  4.42225  2.77519  2.73123  3.46354  2.40513  3.72494
3.29354  2.67741  2.69355  4.24690  2.90347  2.73739  3.18146  2.89801
2.37887  2.77519  2.98518  4.58477  3.61503
          0.01146  4.87023  5.59257  0.61958  0.77255  0.48576  0.95510
    101   4.31017  6.28993  3.47007  0.19447  5.85197  4.30443  5.11648
5.58499  4.18561  5.04635  6.09422  4.06160  4.97822  4.38535  4.62774
4.24327  4.65613  5.19312  6.77364  5.68848    167 E - - -
          2.68618  4.42225  2.77519  2.73123  3.46354  2.40513  3.72494
3.29354  2.67741  2.69355  4.24690  2.90347  2.73739  3.18146  2.89801
2.37887  2.77519  2.98518  4.58477  3.61503
          0.01146  4.87023  5.59257  0.61958  0.77255  0.48576  0.95510
    102   3.45994  5.69559  3.89766  3.06883  5.19850  4.10412  4.00489
4.13107  0.79393  3.91937  4.75535  3.58587  4.44445  2.67916  1.83787
3.29797  3.47946  4.18183  5.92878  4.75018    168 k - - -
          2.68618  4.42225  2.77519  2.73123  3.46354  2.40513  3.72494
3.29354  2.67741  2.69355  4.24690  2.90347  2.73739  3.18146  2.89801
2.37887  2.77519  2.98518  4.58477  3.61503
          0.01146  4.87023  5.59257  0.61958  0.77255  0.48576  0.95510
```

103 3.93259 5.91234 4.40711 3.64240 5.54870 4.38239 4.22983
4.85920 0.41076 4.18350 5.10085 3.95558 4.74701 3.36645 2.33141
3.91211 4.05216 4.55149 6.09397 5.04278 169 K - - -
2.68618 4.42225 2.77519 2.73123 3.46354 2.40513 3.72494
3.29354 2.67741 2.69355 4.24690 2.90347 2.73739 3.18146 2.89801
2.37887 2.77519 2.98518 4.58477 3.61503
0.01146 4.87023 5.59257 0.61958 0.77255 0.48576 0.95510
104 4.28737 5.50713 5.97498 5.51453 3.53214 5.53020 5.86365
2.66324 5.26770 0.30913 3.32266 5.74213 5.60043 5.23457 5.24235
5.04240 4.51627 3.03645 5.80408 4.75196 170 L - - -
2.68618 4.42225 2.77519 2.73123 3.46354 2.40513 3.72494
3.29354 2.67741 2.69355 4.24690 2.90347 2.73739 3.18146 2.89801
2.37887 2.77519 2.98518 4.58477 3.61503
0.01146 4.87023 5.59257 0.61958 0.77255 0.48576 0.95510
105 4.84590 5.81128 5.64731 5.53475 2.03298 5.34375 3.19391
4.48555 5.31217 3.27733 5.00459 4.87526 5.60177 5.03238 5.16343
4.74115 5.03850 4.42102 4.09311 0.39037 171 Y - - -
2.68618 4.42225 2.77519 2.73123 3.46354 2.40513 3.72494
3.29354 2.67741 2.69355 4.24690 2.90347 2.73739 3.18146 2.89801
2.37887 2.77519 2.98518 4.58477 3.61503
0.01146 4.87023 5.59257 0.61958 0.77255 0.48576 0.95510
106 2.71148 5.34043 1.97503 2.00589 4.68548 3.42827 3.21582
4.17127 2.11966 3.43142 4.38145 2.93686 3.99509 2.31334 2.63124
2.79173 3.06116 3.61381 5.77252 4.36170 172 d - - -
2.68618 4.42225 2.77519 2.73123 3.46354 2.40513 3.72494
3.29354 2.67741 2.69355 4.24690 2.90347 2.73739 3.18146 2.89801
2.37887 2.77519 2.98518 4.58477 3.61503
0.01146 4.87023 5.59257 0.61958 0.77255 0.48576 0.95510
107 3.19667 5.85374 2.52583 0.64432 5.15134 3.71324 4.18552
4.64462 3.12332 3.39357 4.98468 3.06831 4.29334 3.33895 3.67529
3.23682 3.60314 4.22758 6.31981 4.84456 173 E - - -
2.68618 4.42225 2.77519 2.73123 3.46354 2.40513 3.72494
3.29354 2.67741 2.69355 4.24690 2.90347 2.73739 3.18146 2.89801
2.37887 2.77519 2.98518 4.58477 3.61503
0.01146 4.87023 5.59257 0.61958 0.77255 0.48576 0.95510
108 3.73029 4.92374 5.80640 5.33556 4.21408 5.35491 6.07086
1.74828 5.27295 2.47261 3.64182 5.50446 5.55433 5.50544 5.43033
4.78945 3.98752 0.51321 6.35304 5.15556 174 V - - -
2.68618 4.42225 2.77519 2.73123 3.46354 2.40513 3.72494
3.29354 2.67741 2.69355 4.24690 2.90347 2.73739 3.18146 2.89801
2.37887 2.77519 2.98518 4.58477 3.61503
0.01146 4.87023 5.59257 0.61958 0.77255 0.48576 0.95510
109 3.58503 5.71124 4.12785 3.33141 5.24301 4.19918 4.05695
4.54184 0.61185 3.93450 4.25682 3.70799 4.53536 3.18101 2.07302
3.56770 3.31160 4.21779 5.94275 4.81062 175 K - - -
2.68613 4.41991 2.77598 2.73190 3.46207 2.40591 3.72417
3.29060 2.67737 2.69335 4.24768 2.90390 2.73789 3.18072 2.89764
2.37949 2.77537 2.98457 4.58555 3.61485
0.08634 2.53837 5.59257 2.97499 0.05240 0.48576 0.95510
110 1.58830 4.57093 2.28676 2.42288 4.54416 3.33123 3.68977
4.00325 2.56284 3.52711 4.29045 2.67986 4.00931 2.84734 3.04828
2.54703 2.91136 3.19121 5.69612 4.13653 212 a - - -
2.68618 4.42225 2.77519 2.73123 3.46354 2.40513 3.72494
3.29354 2.67741 2.69355 4.24690 2.90347 2.73739 3.18146 2.89801
2.37887 2.77519 2.98518 4.58477 3.61503
0.01146 4.87023 5.59257 0.61958 0.77255 0.48576 0.95510

111 2.56801 4.90336 3.13212 1.60653 4.35105 2.29460 3.81540
3.53718 2.53408 3.40067 4.25602 2.99282 4.01399 2.93188 2.66553
2.41935 2.98984 3.56347 4.87875 4.28947 213 e - - -
2.68618 4.42225 2.77519 2.73123 3.46354 2.40513 3.72494
3.29354 2.67741 2.69355 4.24690 2.90347 2.73739 3.18146 2.89801
2.37887 2.77519 2.98518 4.58477 3.61503
0.01146 4.87023 5.59257 0.61958 0.77255 0.48576 0.95510
112 3.27476 5.97848 2.15604 0.72758 5.27710 3.11126 4.18055
4.80638 3.07316 4.26708 5.07851 3.04487 4.28770 3.25187 3.55145
3.12911 3.62332 4.35271 6.40914 4.90023 214 e - - -
2.68618 4.42225 2.77519 2.73123 3.46354 2.40513 3.72494
3.29354 2.67741 2.69355 4.24690 2.90347 2.73739 3.18146 2.89801
2.37887 2.77519 2.98518 4.58477 3.61503
0.01146 4.87023 5.59257 0.61958 0.77255 0.48576 0.95510
113 2.60300 4.45362 3.30730 2.72084 3.57746 3.30582 3.71106
2.97091 1.76362 2.73798 3.39103 3.15101 4.07594 2.95694 2.44603
2.48551 2.95023 3.10733 5.43986 3.97998 215 k - - -
2.68618 4.42225 2.77519 2.73123 3.46354 2.40513 3.72494
3.29354 2.67741 2.69355 4.24690 2.90347 2.73739 3.18146 2.89801
2.37887 2.77519 2.98518 4.58477 3.61503
0.01146 4.87023 5.59257 0.61958 0.77255 0.48576 0.95510
114 2.55016 4.29986 4.75362 4.15199 3.40106 4.08624 4.42286
1.77180 3.96969 1.63331 2.29235 3.83664 4.44539 4.12763 4.02808
3.39537 2.57766 2.04108 4.95169 3.77301 216 l - - -
2.68618 4.42225 2.77519 2.73123 3.46354 2.40513 3.72494
3.29354 2.67741 2.69355 4.24690 2.90347 2.73739 3.18146 2.89801
2.37887 2.77519 2.98518 4.58477 3.61503
0.01146 4.87023 5.59257 0.61958 0.77255 0.48576 0.95510
115 2.95627 4.13939 3.38998 2.71918 4.67977 3.65704 3.89159
4.09610 1.79178 3.60387 4.40062 3.19731 4.16808 2.83008 1.19207
2.94955 3.24240 3.73220 4.83790 4.43338 217 r - - -
2.68618 4.42225 2.77519 2.73123 3.46354 2.40513 3.72494
3.29354 2.67741 2.69355 4.24690 2.90347 2.73739 3.18146 2.89801
2.37887 2.77519 2.98518 4.58477 3.61503
0.01146 4.87023 5.59257 0.61958 0.77255 0.48576 0.95510
116 2.65643 4.45881 3.74597 3.22472 3.39176 3.86256 4.02860
1.96403 2.52245 2.17262 3.06057 3.67847 4.23843 3.22157 2.80128
2.96198 2.87852 2.12027 5.05347 3.63247 218 i - - -
2.68618 4.42225 2.77519 2.73123 3.46354 2.40513 3.72494
3.29354 2.67741 2.69355 4.24690 2.90347 2.73739 3.18146 2.89801
2.37887 2.77519 2.98518 4.58477 3.61503
0.01146 4.87023 5.59257 0.61958 0.77255 0.48576 0.95510
117 2.37492 5.26075 2.70519 1.78357 4.57585 3.61026 3.80074
3.15324 2.43757 2.76392 3.91907 3.01906 4.00301 2.47961 2.88686
2.43766 2.90560 3.54464 5.71178 4.31968 219 e - - -
2.68618 4.42225 2.77519 2.73123 3.46354 2.40513 3.72494
3.29354 2.67741 2.69355 4.24690 2.90347 2.73739 3.18146 2.89801
2.37887 2.77519 2.98518 4.58477 3.61503
0.01146 4.87023 5.59257 0.61958 0.77255 0.48576 0.95510
118 3.85997 5.23323 4.89699 4.45649 2.37065 4.72062 1.85958
3.58411 4.31510 2.55158 4.36585 4.43165 5.04613 3.91310 4.45451
4.03694 4.07989 3.62035 4.27230 0.82223 220 y - - -
2.68618 4.42225 2.77519 2.73123 3.46354 2.40513 3.72494
3.29354 2.67741 2.69355 4.24690 2.90347 2.73739 3.18146 2.89801
2.37887 2.77519 2.98518 4.58477 3.61503
0.01146 4.87023 5.59257 0.61958 0.77255 0.48576 0.95510

```
     119   2.72206  5.35006  2.00914  1.52233  4.69214  3.53470  3.68049
4.17733  2.40539  3.65376  3.95422  2.91652  4.00326  2.49764  2.85975
2.75441  3.07405  3.66907  5.78337  4.37191    221 e - - -
           2.68618  4.42225  2.77519  2.73123  3.46354  2.40513  3.72494
3.29354  2.67741  2.69355  4.24690  2.90347  2.73739  3.18146  2.89801
2.37887  2.77519  2.98518  4.58477  3.61503
           0.01146  4.87023  5.59257  0.61958  0.77255  0.48576  0.95510
     120   3.03839  5.47584  3.40906  2.47148  4.87087  3.82058  3.89141
4.13059  1.08677  3.74371  4.03328  3.24620  4.19340  2.77248  1.92801
3.02064  3.19956  3.89802  5.82886  4.52110    222 k - - -
           2.68618  4.42225  2.77519  2.73123  3.46354  2.40513  3.72494
3.29354  2.67741  2.69355  4.24690  2.90347  2.73739  3.18146  2.89801
2.37887  2.77519  2.98518  4.58477  3.61503
           0.01146  4.87023  5.59257  0.61958  0.77255  0.48576  0.95510
     121   2.80712  4.90244  3.16574  2.56061  4.61076  3.51546  3.70929
3.90360  1.13195  3.57410  4.33935  2.70270  4.04400  2.93416  2.73612
2.64741  3.10334  3.66765  5.72567  4.02634    223 k - - -
           2.68618  4.42225  2.77519  2.73123  3.46354  2.40513  3.72494
3.29354  2.67741  2.69355  4.24690  2.90347  2.73739  3.18146  2.89801
2.37887  2.77519  2.98518  4.58477  3.61503
           0.01146  4.87023  5.59257  0.61958  0.77255  0.48576  0.95510
     122   2.84233  1.91948  4.37380  3.78347  3.32551  3.79674  2.91835
2.69575  3.52790  2.08215  3.35114  3.96337  4.32421  3.86111  3.38860
2.68982  2.97757  2.11294  4.52392  2.93533    224 c - - -
           2.68618  4.42225  2.77519  2.73123  3.46354  2.40513  3.72494
3.29354  2.67741  2.69355  4.24690  2.90347  2.73739  3.18146  2.89801
2.37887  2.77519  2.98518  4.58477  3.61503
           0.01146  4.87023  5.59257  0.61958  0.77255  0.48576  0.95510
     123   2.20319  5.28490  3.01652  2.39642  4.60948  3.29320  3.72677
4.08097  1.79583  3.17085  3.50648  2.90552  3.99960  2.86448  2.43225
2.30784  2.86340  3.58920  5.72990  4.33206    225 k - - -
           2.68618  4.42225  2.77520  2.73123  3.46354  2.40513  3.72495
3.29354  2.67741  2.69355  4.24690  2.90347  2.73735  3.18146  2.89801
2.37887  2.77520  2.98518  4.58477  3.61503
           0.01997  4.13246  5.59257  0.54102  0.87264  0.48576  0.95510
     124   2.65803  4.96933  3.26793  2.43947  3.87220  3.67382  3.32409
3.32596  2.42903  2.26970  3.97723  3.22107  4.06264  2.10242  2.52889
2.82230  2.86806  2.99215  3.69229  3.96648    227 q - - -
           2.68618  4.42225  2.77519  2.73123  3.46354  2.40513  3.72494
3.29354  2.67741  2.69355  4.24690  2.90347  2.73739  3.18146  2.89801
2.37887  2.77519  2.98518  4.58477  3.61503
           0.01146  4.87023  5.59257  0.61958  0.77255  0.48576  0.95510
     125   3.86563  5.38853  5.93870  5.39660  3.52258  5.46321  5.81603
2.69808  5.18573  0.35560  3.00230  5.63064  5.51851  5.13596  5.17444
4.88865  4.36894  2.86492  5.80127  4.84498    228 L - - -
           2.68618  4.42225  2.77519  2.73123  3.46354  2.40513  3.72494
3.29354  2.67741  2.69355  4.24690  2.90347  2.73739  3.18146  2.89801
2.37887  2.77519  2.98518  4.58477  3.61503
           0.01146  4.87023  5.59257  0.61958  0.77255  0.48576  0.95510
     126   3.01421  5.44607  3.30272  2.71108  4.82981  3.63442  3.62188
4.27372  1.64043  3.61927  4.49285  2.62190  4.13605  2.42057  1.53125
2.83950  3.01940  3.86096  5.81947  4.48057    229 r - - -
           2.68618  4.42225  2.77519  2.73123  3.46354  2.40513  3.72494
3.29354  2.67741  2.69355  4.24690  2.90347  2.73739  3.18146  2.89801
2.37887  2.77519  2.98518  4.58477  3.61503
           0.01146  4.87023  5.59257  0.61958  0.77255  0.48576  0.95510
```

```
    127   2.46976  5.32183  2.82093  2.46291  4.45677  3.59978  2.91701
3.87149  1.98059  3.39056  4.23154  2.29377  3.99311  2.72267  2.34525
2.40489  2.96544  3.61239  5.75763  4.35006    230 k - - -
          2.68618  4.42225  2.77519  2.73123  3.46354  2.40513  3.72494
3.29354  2.67741  2.69355  4.24690  2.90347  2.73739  3.18146  2.89801
2.37887  2.77519  2.98518  4.58477  3.61503
          0.01146  4.87023  5.59257  0.61958  0.77255  0.48576  0.95510
    128   2.84645  4.64450  3.61227  2.97126  3.70003  3.79242  3.51141
3.16174  2.77403  1.62658  3.04844  3.47852  4.17307  1.80615  3.29552
3.02953  3.07707  2.86228  4.60266  3.96312    231 l - - -
          2.68618  4.42225  2.77519  2.73123  3.46354  2.40513  3.72494
3.29354  2.67741  2.69355  4.24690  2.90347  2.73739  3.18146  2.89801
2.37887  2.77519  2.98518  4.58477  3.61503
          0.01146  4.87023  5.59257  0.61958  0.77255  0.48576  0.95510
    129   2.82530  4.51514  1.76923  1.75135  3.92163  3.50247  3.69331
3.97276  2.23182  3.58221  4.11198  3.00089  3.99963  2.85267  3.02861
2.74079  3.01611  3.30841  5.73093  4.33266    232 e - - -
          2.68618  4.42225  2.77519  2.73123  3.46354  2.40513  3.72494
3.29354  2.67741  2.69355  4.24690  2.90347  2.73739  3.18146  2.89801
2.37887  2.77519  2.98518  4.58477  3.61503
          0.01146  4.87023  5.59257  0.61958  0.77255  0.48576  0.95510
    130   2.19429  5.02264  2.56236  2.14791  4.60191  3.34357  3.79636
3.94843  2.18209  3.57504  3.89489  3.03557  3.99973  2.58098  2.99727
2.30444  2.93681  2.79223  5.72590  3.49018    233 e - - -
          2.68618  4.42225  2.77519  2.73123  3.46354  2.40513  3.72494
3.29354  2.67741  2.69355  4.24690  2.90347  2.73739  3.18146  2.89801
2.37887  2.77519  2.98518  4.58477  3.61503
          0.01997  4.87023  4.41478  0.61958  0.77255  0.48576  0.95510
    131   2.86141  5.34366  3.12038  2.40384  4.69104  3.18273  3.39743
4.06178  1.74369  3.49647  4.38704  2.72266  4.02113  2.83268  1.76513
2.49964  3.01092  3.73628  5.76710  4.37432    234 k - - -
          2.68618  4.42225  2.77519  2.73123  3.46354  2.40513  3.72494
3.29354  2.67741  2.69355  4.24690  2.90347  2.73739  3.18146  2.89801
2.37887  2.77519  2.98518  4.58477  3.61503
          0.01156  4.86182  5.58417  0.61958  0.77255  0.50629  0.92314
    132   2.86233  5.45483  2.37156  2.44964  4.77895  1.27209  3.91390
4.25829  2.67002  3.75518  4.52084  2.82588  4.09066  2.98483  2.74183
2.75241  3.21635  3.73699  5.90545  4.48897    235 g - - -
          2.68616  4.42226  2.77520  2.73124  3.46355  2.40513  3.72495
3.29355  2.67741  2.69356  4.24663  2.90347  2.73740  3.18147  2.89801
2.37888  2.77515  2.98519  4.58478  3.61504
          0.03737  3.41352  5.58417  0.43302  1.04568  0.50629  0.92314
    133   1.67383  5.25699  2.61255  2.18679  4.57144  3.09886  3.44296
4.03641  2.28527  3.22357  4.30591  3.08785  4.00067  2.83576  3.03382
2.74752  2.88202  3.03488  5.70841  3.94772    237 a - - -
          2.68618  4.42225  2.77520  2.73124  3.46354  2.40513  3.72495
3.29354  2.67741  2.69351  4.24690  2.90347  2.73740  3.18147  2.89801
2.37884  2.77520  2.98519  4.58477  3.61503
          0.01912  4.18787  5.58417  0.63075  0.75970  0.50629  0.92314
    134   2.80420  5.34259  1.98261  1.68120  4.68319  3.27099  3.52384
3.74633  2.44079  3.64595  4.38524  2.49730  3.99923  2.89480  2.97045
2.48087  2.96266  3.73098  5.77709  4.18653    240 e - - -
          2.68618  4.42225  2.77519  2.73123  3.46354  2.40513  3.72494
3.29354  2.67741  2.69355  4.24690  2.90347  2.73739  3.18146  2.89801
2.37887  2.77519  2.98518  4.58477  3.61503
          0.01156  4.86182  5.58417  0.61958  0.77255  0.50629  0.92314
```

```
    135   2.21881  5.20967  2.93250  2.38480  4.25613  3.33655  3.80864
3.96054  2.53424  3.08406  4.26322  2.57365  2.78935  2.69852  2.89833
2.18048  2.92115  3.22967  4.51698  3.41741    241 s - - -
          2.68618  4.42225  2.77519  2.73123  3.46354  2.40513  3.72494
3.29354  2.67741  2.69355  4.24690  2.90347  2.73739  3.18146  2.89801
2.37887  2.77519  2.98518  4.58477  3.61503
          0.01156  4.86182  5.58417  0.61958  0.77255  0.50629  0.92314
    136   2.61457  5.15537  2.88265  2.24389  4.07800  3.53543  3.13022
3.31273  2.53201  3.07169  3.73974  2.99071  3.92085  2.64915  2.78657
1.98029  2.82253  2.82562  4.79455  4.15171    242 s - - -
          2.68618  4.42225  2.77519  2.73123  3.46354  2.40513  3.72494
3.29354  2.67741  2.69355  4.24690  2.90347  2.73739  3.18146  2.89801
2.37887  2.77519  2.98518  4.58477  3.61503
          0.01156  4.86182  5.58417  0.61958  0.77255  0.50629  0.92314
    137   2.47653  4.65013  2.88579  2.42638  4.57196  3.60746  3.59890
3.94095  1.52676  3.23079  4.30578  3.04681  4.00017  2.79461  2.92529
2.29660  2.69748  3.15856  5.70811  4.31633    243 k - - -
          2.68618  4.42225  2.77519  2.73123  3.46354  2.40513  3.72494
3.29354  2.67741  2.69355  4.24690  2.90347  2.73739  3.18146  2.89801
2.37887  2.77519  2.98518  4.58477  3.61503
          0.01156  4.86182  5.58417  0.61958  0.77255  0.50629  0.92314
    138   2.90719  4.58669  5.23218  4.66886  3.78022  4.60381  5.03241
1.16864  4.51827  1.85696  3.24959  4.73970  4.91725  4.69014  4.59215
3.94573  2.81546  1.45902  5.50054  4.31761    244 i - - -
          2.68618  4.42225  2.77519  2.73123  3.46354  2.40513  3.72494
3.29354  2.67741  2.69355  4.24690  2.90347  2.73739  3.18146  2.89801
2.37887  2.77519  2.98518  4.58477  3.61503
          0.01156  4.86182  5.58417  0.61958  0.77255  0.47314  0.97562
    139   3.14294  5.70534  1.11541  1.64013  5.01505  3.66775  3.81551
4.52300  2.89066  3.79136  4.77355  2.82078  4.18014  2.92371  3.42932
3.06494  3.39611  4.08241  6.13453  3.91313    245 d - - -
          2.68618  4.42225  2.77519  2.73123  3.46354  2.40513  3.72494
3.29354  2.67741  2.69355  4.24690  2.90347  2.73739  3.18146  2.89801
2.37887  2.77519  2.98518  4.58477  3.61503
          0.01146  4.87023  5.59257  0.61958  0.77255  0.48576  0.95510
    140   2.40395  5.14600  3.17531  2.51750  4.03463  3.64223  3.83529
3.85595  1.38528  2.93204  3.94358  3.14697  4.03310  2.96279  2.84168
2.29962  2.93962  3.49809  5.62293  4.26420    246 k - - -
          2.68618  4.42225  2.77519  2.73123  3.46354  2.40513  3.72494
3.29354  2.67741  2.69355  4.24690  2.90347  2.73739  3.18146  2.89801
2.37887  2.77519  2.98518  4.58477  3.61503
          0.01146  4.87023  5.59257  0.61958  0.77255  0.48576  0.95510
    141   2.48744  4.58389  3.57885  3.14957  3.75123  3.79925  4.08989
2.84717  3.10241  2.63725  3.71196  3.42452  4.19781  3.41085  3.31911
2.93294  1.18311  2.61412  5.19094  3.95415    247 t - - -
          2.68618  4.42225  2.77519  2.73123  3.46354  2.40513  3.72494
3.29354  2.67741  2.69355  4.24690  2.90347  2.73739  3.18146  2.89801
2.37887  2.77519  2.98518  4.58477  3.61503
          0.01146  4.87023  5.59257  0.61958  0.77255  0.48576  0.95510
    142   3.04300  5.02233  3.37441  2.42994  4.77106  3.79122  3.88272
4.19628  1.78304  3.67415  4.45926  3.27405  4.16655  2.71668  1.28817
2.91360  3.20106  3.81157  3.57925  4.46988    248 r - - -
          2.68618  4.42225  2.77519  2.73123  3.46354  2.40513  3.72494
3.29354  2.67741  2.69355  4.24690  2.90347  2.73739  3.18146  2.89801
2.37887  2.77519  2.98518  4.58477  3.61503
          0.01146  4.87023  5.59257  0.61958  0.77255  0.48576  0.95510
```

```
    143   1.52934  4.93811  3.12178  2.73150  4.01385  3.68176  3.89266
3.39288  2.35484  2.86140  3.66846  3.00886  4.07030  3.06495  3.12780
2.36989  2.63736  2.99465  5.45880  4.14454    249 a - - -
          2.68618  4.42225  2.77519  2.73123  3.46354  2.40513  3.72494
3.29354  2.67741  2.69355  4.24690  2.90347  2.73739  3.18146  2.89801
2.37887  2.77519  2.98518  4.58477  3.61503
          0.01146  4.87023  5.59257  0.61958  0.77255  0.48576  0.95510
    144   2.02096  4.41418  3.38244  2.56544  3.59207  3.49709  4.01442
3.00824  2.97512  2.33087  3.46298  3.27681  4.15698  3.29241  3.36432
2.03194  2.72727  2.41323  5.22068  3.76753    250 a - - -
          2.68618  4.42225  2.77519  2.73123  3.46354  2.40513  3.72494
3.29354  2.67741  2.69355  4.24690  2.90347  2.73739  3.18146  2.89801
2.37887  2.77519  2.98518  4.58477  3.61503
          0.01146  4.87023  5.59257  0.61958  0.77255  0.48576  0.95510
    145   2.73147  4.34683  4.86964  4.27217  3.47270  4.17977  4.53155
1.56384  4.08681  2.28021  3.31055  4.13701  4.53305  4.24354  4.13683
3.49513  3.08881  1.17924  4.08326  3.86304    251 v - - -
          2.68618  4.42225  2.77519  2.73123  3.46354  2.40513  3.72494
3.29354  2.67741  2.69355  4.24690  2.90347  2.73739  3.18146  2.89801
2.37887  2.77519  2.98518  4.58477  3.61503
          0.01146  4.87023  5.59257  0.61958  0.77255  0.48576  0.95510
    146   2.83593  5.33966  2.88224  2.11552  4.68478  3.49989  3.79156
4.16781  1.82241  3.64294  4.10822  2.83490  4.00054  2.71580  1.84988
2.48877  2.90140  3.73052  5.77032  4.36348    252 k - - -
          2.68618  4.42225  2.77519  2.73123  3.46354  2.40513  3.72494
3.29354  2.67741  2.69355  4.24690  2.90347  2.73739  3.18146  2.89801
2.37887  2.77519  2.98518  4.58477  3.61503
          0.01146  4.87023  5.59257  0.61958  0.77255  0.48576  0.95510
    147   2.77537  4.46239  2.05698  2.37325  4.65270  3.32477  3.07177
3.96093  1.91433  3.14287  4.00707  2.79948  3.99393  2.88742  2.68286
2.41481  2.98903  3.70193  5.75298  4.23298    253 k - - -
          2.68618  4.42225  2.77519  2.73123  3.46354  2.40513  3.72494
3.29354  2.67741  2.69355  4.24690  2.90347  2.73739  3.18146  2.89801
2.37887  2.77519  2.98518  4.58477  3.61503
          0.01146  4.87023  5.59257  0.61958  0.77255  0.48576  0.95510
    148   2.92691  4.51002  4.67643  4.09199  3.11806  4.21451  3.84321
2.70382  3.92776  0.74512  3.34293  4.24753  4.55721  4.11885  4.05431
3.41723  3.31678  2.56744  5.04369  3.83182    254 l - - -
          2.68618  4.42225  2.77519  2.73123  3.46354  2.40513  3.72494
3.29354  2.67741  2.69355  4.24690  2.90347  2.73739  3.18146  2.89801
2.37887  2.77519  2.98518  4.58477  3.61503
          0.01146  4.87023  5.59257  0.61958  0.77255  0.48576  0.95510
    149   2.82558  3.67043  2.96435  2.18760  4.52912  3.61707  2.18546
3.98586  2.56261  3.15328  4.27915  3.07738  4.00946  2.43392  2.75467
2.15890  3.05674  3.49123  5.68597  3.28976    255 s - - -
          2.68618  4.42225  2.77519  2.73123  3.46354  2.40513  3.72494
3.29354  2.67741  2.69355  4.24690  2.90347  2.73739  3.18146  2.89801
2.37887  2.77519  2.98518  4.58477  3.61503
          0.01146  4.87023  5.59257  0.61958  0.77255  0.48576  0.95510
    150   2.32878  4.22628  4.14620  3.66728  4.49519  3.55360  4.61680
3.85876  3.61518  3.57571  3.90505  3.84454  4.23468  3.90580  3.94075
1.33830  1.14280  3.44179  5.88348  4.67228    256 t - - -
          2.68618  4.42225  2.77519  2.73123  3.46354  2.40513  3.72494
3.29354  2.67741  2.69355  4.24690  2.90347  2.73739  3.18146  2.89801
2.37887  2.77519  2.98518  4.58477  3.61503
          0.01146  4.87023  5.59257  0.61958  0.77255  0.48576  0.95510
```

```
    151   3.02453  5.44877  3.08047  2.66424  4.83099  3.58328  3.86845
4.27229  1.54177  3.17891  4.49644  3.23592  4.14391  2.27409  1.55467
2.99003  3.23695  3.86297  5.82139  4.48568    257 k - - -
          2.68618  4.42225  2.77519  2.73123  3.46354  2.40513  3.72494
3.29354  2.67741  2.69355  4.24690  2.90347  2.73739  3.18146  2.89801
2.37887  2.77519  2.98518  4.58477  3.61503
          0.01146  4.87023  5.59257  0.61958  0.77255  0.48576  0.95510
    152   3.03604  4.40453  4.91940  4.31534  3.38690  4.22026  4.32954
1.13867  4.12375  1.84552  2.50639  4.35323  4.55840  4.25327  4.15856
3.53449  3.26664  2.28961  5.01910  2.93641    258 i - - -
          2.68618  4.42225  2.77519  2.73123  3.46354  2.40513  3.72494
3.29354  2.67741  2.69355  4.24690  2.90347  2.73739  3.18146  2.89801
2.37887  2.77519  2.98518  4.58477  3.61503
          0.01146  4.87023  5.59257  0.61958  0.77255  0.48576  0.95510
    153   2.71742  5.02428  2.98484  2.67239  4.26119  3.26964  3.77496
2.64280  2.36894  2.90149  3.31618  2.87873  4.04946  2.78332  2.32415
2.28490  2.74658  2.99507  4.39729  4.19345    259 s - - -
          2.68618  4.42225  2.77519  2.73123  3.46354  2.40513  3.72494
3.29354  2.67741  2.69355  4.24690  2.90347  2.73739  3.18146  2.89801
2.37887  2.77519  2.98518  4.58477  3.61503
          0.01146  4.87023  5.59257  0.61958  0.77255  0.48576  0.95510
    154   2.97847  4.43950  4.77738  4.20189  3.62293  4.23067  4.62462
1.60063  4.04456  2.58941  3.51911  4.32064  4.59898  4.25217  3.77749
3.03988  3.03237  1.02450  5.20934  4.01955    260 v - - -
          2.68618  4.42225  2.77519  2.73123  3.46354  2.40513  3.72494
3.29354  2.67741  2.69355  4.24690  2.90347  2.73739  3.18146  2.89801
2.37887  2.77519  2.98518  4.58477  3.61503
          0.01146  4.87023  5.59257  0.61958  0.77255  0.48576  0.95510
    155   1.70770  4.26805  2.79006  2.81765  4.04122  3.50605  3.93727
2.87483  2.80160  2.72670  3.93564  3.08547  4.09823  3.13857  3.23622
1.96542  2.77841  3.08051  5.38816  4.09599    261 a - - -
          2.68618  4.42225  2.77519  2.73123  3.46354  2.40513  3.72494
3.29354  2.67741  2.69355  4.24690  2.90347  2.73739  3.18146  2.89801
2.37887  2.77519  2.98518  4.58477  3.61503
          0.01146  4.87023  5.59257  0.61958  0.77255  0.48576  0.95510
    156   2.78777  4.32449  4.28615  3.37741  3.42867  3.94163  4.01689
1.38006  3.46818  2.24580  2.78713  3.91495  4.31140  3.58857  3.62336
2.56149  2.84945  2.26682  4.93536  3.74265    262 i - - -
          2.68618  4.42225  2.77519  2.73123  3.46354  2.40513  3.72494
3.29354  2.67741  2.69355  4.24690  2.90347  2.73739  3.18146  2.89801
2.37887  2.77519  2.98518  4.58477  3.61503
          0.01146  4.87023  5.59257  0.61958  0.77255  0.48576  0.95510
    157   2.84778  5.32615  2.99997  2.39506  4.66603  3.62135  2.71930
4.14048  2.24045  3.44978  4.37004  3.09310  4.01303  1.63456  2.21325
2.73292  3.07683  3.71448  5.16389  3.99782    263 q - - -
          2.68618  4.42225  2.77519  2.73123  3.46354  2.40513  3.72494
3.29354  2.67741  2.69355  4.24690  2.90347  2.73739  3.18146  2.89801
2.37887  2.77519  2.98518  4.58477  3.61503
          0.01146  4.87023  5.59257  0.61958  0.77255  0.48576  0.95510
    158   1.99246  4.45663  3.42716  2.86746  3.99980  3.43921  3.96404
3.38445  2.69101  3.05092  3.74052  3.34373  4.11317  3.18014  2.84210
1.69835  2.68370  2.34207  5.36258  4.08047    264 s - - -
          2.68618  4.42225  2.77519  2.73123  3.46354  2.40513  3.72494
3.29354  2.67741  2.69355  4.24690  2.90347  2.73739  3.18146  2.89801
2.37887  2.77519  2.98518  4.58477  3.61503
          0.01146  4.87023  5.59257  0.61958  0.77255  0.48576  0.95510
```

159 2.11246 4.59334 5.21151 4.65161 3.81553 4.60804 5.04451
1.42172 4.50762 2.47828 2.66921 4.73545 4.92550 4.69121 4.59556
3.95016 3.39561 1.18775 5.52968 4.34075 265 v - - -
2.68618 4.42225 2.77519 2.73123 3.46354 2.40513 3.72494
3.29354 2.67741 2.69355 4.24690 2.90347 2.73739 3.18146 2.89801
2.37887 2.77519 2.98518 4.58477 3.61503
0.01146 4.87023 5.59257 0.61958 0.77255 0.48576 0.95510
160 2.65925 5.39722 1.77754 1.76729 4.73505 3.39706 3.83336
4.22247 2.54793 3.62914 4.44292 2.38035 4.02890 2.82473 3.09019
2.53578 2.90492 3.78457 5.83135 4.14846 266 e - - -
2.68618 4.42225 2.77519 2.73123 3.46354 2.40513 3.72494
3.29354 2.67741 2.69355 4.24690 2.90347 2.73739 3.18146 2.89801
2.37887 2.77519 2.98518 4.58477 3.61503
0.01146 4.87023 5.59257 0.61958 0.77255 0.48576 0.95510
161 2.17073 5.13361 3.09540 2.56751 4.02699 3.53188 3.70209
3.83988 2.31343 3.21223 3.98282 2.99546 4.02655 2.96087 2.92691
1.70254 2.34345 3.48329 5.61405 3.85339 267 s - - -
2.68618 4.42225 2.77519 2.73123 3.46354 2.40513 3.72494
3.29354 2.67741 2.69355 4.24690 2.90347 2.73739 3.18146 2.89801
2.37887 2.77519 2.98518 4.58477 3.61503
0.01146 4.87023 5.59257 0.61958 0.77255 0.48576 0.95510
162 2.84668 4.51557 4.95170 4.38019 3.13603 4.39414 4.79348
1.08258 4.23671 2.49502 3.62127 4.49181 4.73815 4.42578 4.34601
3.71999 1.84870 1.98238 5.33457 4.14744 268 i - - -
2.68618 4.42225 2.77519 2.73123 3.46354 2.40513 3.72494
3.29354 2.67741 2.69355 4.24690 2.90347 2.73739 3.18146 2.89801
2.37887 2.77519 2.98518 4.58477 3.61503
0.01146 4.87023 5.59257 0.61958 0.77255 0.48576 0.95510
163 2.57399 3.17689 4.12098 3.57146 3.75775 3.80258 4.33807
2.92691 3.49489 2.75415 3.61557 3.83294 4.28767 3.76215 3.77089
1.06372 2.87176 2.32157 5.24117 4.03468 269 s - - -
2.68618 4.42225 2.77519 2.73123 3.46354 2.40513 3.72494
3.29354 2.67741 2.69355 4.24690 2.90347 2.73739 3.18146 2.89801
2.37887 2.77519 2.98518 4.58477 3.61503
0.01146 4.87023 5.59257 0.61958 0.77255 0.48576 0.95510
164 2.49237 5.24082 3.03262 2.37602 4.33556 3.25080 3.80528
3.63187 1.93156 3.04937 3.79022 2.91295 4.00638 2.83032 2.71043
1.89291 2.92198 3.32005 5.69662 4.30954 270 s - - -
2.68618 4.42225 2.77519 2.73123 3.46354 2.40513 3.72494
3.29354 2.67741 2.69355 4.24690 2.90347 2.73739 3.18146 2.89801
2.37887 2.77519 2.98518 4.58477 3.61503
0.01146 4.87023 5.59257 0.61958 0.77255 0.48576 0.95510
165 2.47520 4.81226 3.09977 2.09286 4.62406 3.61130 3.79743
4.09592 2.04055 3.51351 4.11334 2.95876 4.00398 2.90016 1.80656
2.45614 2.64994 3.67737 5.73700 4.34008 271 r - - -
2.68618 4.42225 2.77519 2.73123 3.46354 2.40513 3.72494
3.29354 2.67741 2.69355 4.24690 2.90347 2.73739 3.18146 2.89801
2.37887 2.77519 2.98518 4.58477 3.61503
0.01146 4.87023 5.59257 0.61958 0.77255 0.48576 0.95510
166 3.78634 4.99932 5.79970 5.30189 3.95537 5.31273 5.90361
0.55311 5.19681 2.30484 2.75382 5.47050 5.49215 5.33369 5.29477
4.73366 4.03574 1.98059 6.11031 4.99653 272 I - - -
2.68618 4.42225 2.77519 2.73123 3.46354 2.40513 3.72494
3.29354 2.67741 2.69355 4.24690 2.90347 2.73739 3.18146 2.89801
2.37887 2.77519 2.98518 4.58477 3.61503
0.01146 4.87023 5.59257 0.61958 0.77255 0.48576 0.95510

```
    167   2.73457  4.99346  3.06950  1.80455  4.57129  3.61137  2.72670
3.22758  2.55031  3.03421  4.30680  2.18768  4.00428  2.47864  3.03697
2.55431  3.01901  3.51564  5.70956  4.31850    273 e - - -
          2.68618  4.42225  2.77519  2.73123  3.46354  2.40513  3.72494
3.29354  2.67741  2.69355  4.24690  2.90347  2.73739  3.18146  2.89801
2.37887  2.77519  2.98518  4.58477  3.61503
          0.01146  4.87023  5.59257  0.61958  0.77255  0.48576  0.95510
    168   2.76082  5.34050  2.80593  2.00002  4.68510  3.38870  3.60660
4.16882  1.53617  3.64409  4.38209  2.92287  3.99974  2.80658  2.37994
2.79781  2.69282  3.60479  5.77187  4.36380    274 k - - -
          2.68618  4.42225  2.77519  2.73123  3.46354  2.40513  3.72494
3.29354  2.67741  2.69355  4.24690  2.90347  2.73739  3.18146  2.89801
2.37887  2.77519  2.98518  4.58477  3.61503
          0.01146  4.87023  5.59257  0.61958  0.77255  0.48576  0.95510
    169   4.04514  5.24334  6.01855  5.44955  3.56998  5.48465  5.82415
1.52619  5.32687  0.67830  2.70871  5.64724  5.50927  5.20085  5.27916
4.88515  4.25383  2.42790  5.81278  4.86252    275 L - - -
          2.68618  4.42225  2.77519  2.73123  3.46354  2.40513  3.72494
3.29354  2.67741  2.69355  4.24690  2.90347  2.73739  3.18146  2.89801
2.37887  2.77519  2.98518  4.58477  3.61503
          0.01146  4.87023  5.59257  0.61958  0.77255  0.48576  0.95510
    170   3.61759  5.57566  4.31908  3.50738  5.03705  4.24117  4.13694
3.43077  2.25208  3.79667  4.72019  3.83142  4.60065  3.28476  0.56527
3.65680  3.57327  4.02366  5.89761  4.77636    276 R - - -
          2.68618  4.42225  2.77519  2.73123  3.46354  2.40513  3.72494
3.29354  2.67741  2.69355  4.24690  2.90347  2.73739  3.18146  2.89801
2.37887  2.77519  2.98518  4.58477  3.61503
          0.01146  4.87023  5.59257  0.61958  0.77255  0.48576  0.95510
    171   3.73906  6.55614  0.51071  2.09133  5.79595  3.75207  4.43903
5.41327  3.66246  4.83621  5.76889  2.77592  4.46222  3.62995  4.41453
3.54026  4.06637  4.91837  6.98137  5.32388    277 D - - -
          2.68618  4.42225  2.77519  2.73123  3.46354  2.40513  3.72494
3.29354  2.67741  2.69355  4.24690  2.90347  2.73739  3.18146  2.89801
2.37887  2.77519  2.98518  4.58477  3.61503
          0.01146  4.87023  5.59257  0.61958  0.77255  0.48576  0.95510
    172   2.78520  5.35640  2.68933  1.33236  4.69724  3.47662  3.80568
4.18228  2.20890  3.65967  4.39960  2.92165  4.00799  2.67056  2.98009
2.57093  2.74135  3.58152  5.79031  4.37833    278 e - - -
          2.68618  4.42225  2.77519  2.73123  3.46354  2.40513  3.72494
3.29354  2.67741  2.69355  4.24690  2.90347  2.73739  3.18146  2.89801
2.37887  2.77519  2.98518  4.58477  3.61503
          0.01146  4.87023  5.59257  0.61958  0.77255  0.48576  0.95510
    173   3.25682  5.84010  2.45742  0.76075  5.14758  3.69757  3.54565
4.66101  2.98374  4.12542  4.92070  3.14861  4.24345  2.59790  3.38242
3.16253  3.51660  4.21823  6.25928  4.78760    279 e - - -
          2.68618  4.42225  2.77519  2.73123  3.46354  2.40513  3.72494
3.29354  2.67741  2.69355  4.24690  2.90347  2.73739  3.18146  2.89801
2.37887  2.77519  2.98518  4.58477  3.61503
          0.01146  4.87023  5.59257  0.61958  0.77255  0.48576  0.95510
    174   4.48791  5.70531  5.78318  5.51155  3.85587  5.24687  5.90741
3.16812  5.31141  0.21752  3.71317  5.72780  5.57400  5.40709  5.29078
5.10656  4.76051  3.43922  5.97401  4.90344    280 L - - -
          2.68618  4.42225  2.77519  2.73123  3.46354  2.40513  3.72494
3.29354  2.67741  2.69355  4.24690  2.90347  2.73739  3.18146  2.89801
2.37887  2.77519  2.98518  4.58477  3.61503
          0.01146  4.87023  5.59257  0.61958  0.77255  0.48576  0.95510
```

175 2.61641 4.36844 3.66643 2.93535 3.30741 3.67971 3.08049
3.09205 3.05867 2.52682 3.68812 3.51874 4.18284 2.04789 3.32820
2.88439 3.06728 2.69640 2.85822 2.36975 281 q - - -
2.68618 4.42225 2.77519 2.73123 3.46354 2.40513 3.72494
3.29354 2.67741 2.69355 4.24690 2.90347 2.73739 3.18146 2.89801
2.37887 2.77519 2.98518 4.58477 3.61503
0.01146 4.87023 5.59257 0.61958 0.77255 0.48576 0.95510
176 2.93758 4.87441 3.50391 2.98332 3.63031 3.78366 3.77787
3.48811 2.84411 3.01677 4.01387 3.43821 1.05406 3.17324 2.98485
2.97012 3.08800 3.21901 5.40781 4.11321 282 p - - -
2.68618 4.42225 2.77519 2.73123 3.46354 2.40513 3.72494
3.29354 2.67741 2.69355 4.24690 2.90347 2.73739 3.18146 2.89801
2.37887 2.77519 2.98518 4.58477 3.61503
0.01146 4.87023 5.59257 0.61958 0.77255 0.48576 0.95510
177 3.21754 5.41611 3.62797 2.99017 4.75630 3.94334 3.97066
4.14149 2.15413 2.90778 4.16386 3.45211 4.30890 0.92982 2.51209
3.22100 3.40994 3.81622 5.77375 4.52495 283 q - - -
2.68618 4.42225 2.77519 2.73123 3.46354 2.40513 3.72494
3.29354 2.67741 2.69355 4.24690 2.90347 2.73739 3.18146 2.89801
2.37887 2.77519 2.98518 4.58477 3.61503
0.01146 4.87023 5.59257 0.61958 0.77255 0.48576 0.95510
178 3.91942 5.13942 5.87949 5.31747 3.61715 5.34262 5.71787
1.87589 5.19178 0.59926 3.12449 5.49369 5.43234 5.14179 5.18342
4.73171 3.95509 2.11527 5.80129 4.80828 284 L - - -
2.68618 4.42225 2.77519 2.73123 3.46354 2.40513 3.72494
3.29354 2.67741 2.69355 4.24690 2.90347 2.73739 3.18146 2.89801
2.37887 2.77519 2.98518 4.58477 3.61503
0.01146 4.87023 5.59257 0.61958 0.77255 0.48576 0.95510
179 2.73846 4.36690 3.50938 2.77011 3.67513 3.82244 4.07829
2.32285 2.82687 2.05241 3.39389 2.84848 4.20127 3.33562 3.40285
2.96014 2.60276 1.87903 5.12493 3.89356 285 v - - -
2.68618 4.42225 2.77519 2.73123 3.46354 2.40513 3.72494
3.29354 2.67741 2.69355 4.24690 2.90347 2.73739 3.18146 2.89801
2.37887 2.77519 2.98518 4.58477 3.61503
0.01146 4.87023 5.59257 0.61958 0.77255 0.48576 0.95510
180 2.51517 4.88431 2.69734 1.22804 4.68925 3.42535 3.58156
4.17209 2.46606 3.65366 4.39615 2.91592 4.01106 2.62377 2.98415
2.81419 3.02677 3.73869 5.78774 4.37747 286 e - - -
2.68618 4.42225 2.77519 2.73123 3.46354 2.40513 3.72494
3.29354 2.67741 2.69355 4.24690 2.90347 2.73739 3.18146 2.89801
2.37887 2.77519 2.98518 4.58477 3.61503
0.01146 4.87023 5.59257 0.61958 0.77255 0.48576 0.95510
181 4.12600 5.34417 5.95569 5.39345 3.08046 5.47868 5.71354
2.49966 5.24748 0.38519 3.25398 5.60858 5.50098 5.12480 5.21068
4.87862 3.92416 2.93606 5.70434 4.67958 287 L - - -
2.68618 4.42225 2.77519 2.73123 3.46354 2.40513 3.72494
3.29354 2.67741 2.69355 4.24690 2.90347 2.73739 3.18146 2.89801
2.37887 2.77519 2.98518 4.58477 3.61503
0.01146 4.87023 5.59257 0.61958 0.77255 0.48576 0.95510
182 2.77847 3.26262 4.78692 3.99979 3.10410 4.05729 4.39428
1.41053 3.98203 1.93358 3.29635 4.19812 4.41879 4.12795 4.01612
3.04288 2.72776 1.90807 4.90572 3.72728 288 i - - -
2.68618 4.42225 2.77519 2.73123 3.46354 2.40513 3.72494
3.29354 2.67741 2.69355 4.24690 2.90347 2.73739 3.18146 2.89801
2.37887 2.77519 2.98518 4.58477 3.61503
0.01146 4.87023 5.59257 0.61958 0.77255 0.48576 0.95510

```
     183   2.65407  5.01325  2.72386  2.27092  4.52073  3.24401  2.77799
4.06201  2.26691  3.24206  4.36510  2.83792  3.99275  1.75877  2.89267
2.67146  2.98536  3.60243  5.75869  4.22070    289 q - - -
           2.68618  4.42225  2.77519  2.73123  3.46354  2.40513  3.72494
3.29354  2.67741  2.69355  4.24690  2.90347  2.73739  3.18146  2.89801
2.37887  2.77519  2.98518  4.58477  3.61503
           0.01146  4.87023  5.59257  0.61958  0.77255  0.48576  0.95510
     184   2.56056  4.53675  3.44547  2.87658  4.40062  1.09825  4.04685
3.80800  2.63475  3.42237  4.24534  3.37198  4.13941  3.21572  2.61202
2.79966  3.15367  3.31724  5.66395  4.36596    290 g - - -
           2.68618  4.42225  2.77519  2.73123  3.46354  2.40513  3.72494
3.29354  2.67741  2.69355  4.24690  2.90347  2.73739  3.18146  2.89801
2.37887  2.77519  2.98518  4.58477  3.61503
           0.01146  4.87023  5.59257  0.61958  0.77255  0.48576  0.95510
     185   3.13819  4.51644  4.92938  4.32885  2.07544  4.30891  4.61653
2.67424  4.15095  0.94730  2.62495  4.41306  4.62911  3.97966  4.20659
3.50985  2.99569  2.65666  5.07880  3.92522    291 l - - -
           2.68618  4.42225  2.77519  2.73123  3.46354  2.40513  3.72494
3.29354  2.67741  2.69355  4.24690  2.90347  2.73739  3.18146  2.89801
2.37887  2.77519  2.98518  4.58477  3.61503
           0.01146  4.87023  5.59257  0.61958  0.77255  0.48576  0.95510
     186   2.20878  4.23025  4.05071  3.29798  3.51091  3.69662  4.17922
2.15307  3.06416  2.28278  2.38060  3.69210  4.26930  3.64805  3.02804
2.67946  2.37259  2.43569  4.74360  3.79429    292 i - - -
           2.68618  4.42225  2.77519  2.73123  3.46354  2.40513  3.72494
3.29354  2.67741  2.69355  4.24690  2.90347  2.73739  3.18146  2.89801
2.37887  2.77519  2.98518  4.58477  3.61503
           0.01146  4.87023  5.59257  0.61958  0.77255  0.48576  0.95510
     187   2.82483  4.48152  2.92494  2.37874  4.64284  3.18072  3.27850
3.95304  2.16764  3.41741  4.35222  2.90932  3.99576  2.63826  1.70350
2.53174  2.88701  3.69344  5.74767  3.68636    293 r - - -
           2.68618  4.42225  2.77519  2.73123  3.46354  2.40513  3.72494
3.29354  2.67741  2.69355  4.24690  2.90347  2.73739  3.18146  2.89801
2.37887  2.77519  2.98518  4.58477  3.61503
           0.01146  4.87023  5.59257  0.61958  0.77255  0.48576  0.95510
     188   2.97001  4.53042  4.33906  3.80596  3.66907  3.97612  4.44114
2.83991  3.59547  2.47400  0.88910  3.78347  4.42754  3.92463  3.79900
3.17101  2.87589  2.70531  5.22130  4.01556    294 m - - -
           2.68618  4.42225  2.77519  2.73123  3.46354  2.40513  3.72494
3.29354  2.67741  2.69355  4.24690  2.90347  2.73739  3.18146  2.89801
2.37887  2.77519  2.98518  4.58477  3.61503
           0.01146  4.87023  5.59257  0.61958  0.77255  0.48576  0.95510
     189   5.32281  6.25912  5.76884  5.74769  4.28168  5.02990  5.55290
5.57208  5.58378  4.75558  6.12455  5.79372  5.62614  5.89784  5.50583
5.53168  5.68657  5.46235  0.09980  4.25226    295 W - - -
           2.68618  4.42225  2.77519  2.73123  3.46354  2.40513  3.72494
3.29354  2.67741  2.69355  4.24690  2.90347  2.73739  3.18146  2.89801
2.37887  2.77519  2.98518  4.58477  3.61503
           0.01146  4.87023  5.59257  0.61958  0.77255  0.48576  0.95510
     190   2.72233  5.35778  3.04042  2.23299  4.70722  3.58268  3.80475
4.18503  1.55137  3.65760  4.40041  2.78036  4.02166  2.67701  1.95535
2.64264  3.02659  3.75145  5.78088  4.28614    296 k - - -
           2.68618  4.42225  2.77519  2.73123  3.46354  2.40513  3.72494
3.29354  2.67741  2.69355  4.24690  2.90347  2.73739  3.18146  2.89801
2.37887  2.77519  2.98518  4.58477  3.61503
           0.01146  4.87023  5.59257  0.61958  0.77255  0.48576  0.95510
```

191 2.36439 4.80938 2.96768 2.67862 3.45433 3.54546 3.94131
2.97914 2.81847 2.96543 3.67754 3.04852 4.10530 3.10920 3.24745
2.00770 2.20034 2.34168 5.35278 4.06701 297 s - - -
2.68618 4.42225 2.77519 2.73123 3.46354 2.40513 3.72494
3.29354 2.67741 2.69355 4.24690 2.90347 2.73739 3.18146 2.89801
2.37887 2.77519 2.98518 4.58477 3.61503
0.01146 4.87023 5.59257 0.61958 0.77255 0.48576 0.95510
192 3.24568 4.63298 4.90664 4.31709 3.50464 4.42833 4.75103
2.44480 3.62430 1.77878 0.98716 4.47830 4.73723 4.32224 4.25467
3.74258 3.36578 2.32393 5.26029 4.12384 298 m - - -
2.68618 4.42225 2.77519 2.73123 3.46354 2.40513 3.72494
3.29354 2.67741 2.69355 4.24690 2.90347 2.73739 3.18146 2.89801
2.37887 2.77519 2.98518 4.58477 3.61503
0.01146 4.87023 5.59257 0.61958 0.77255 0.48576 0.95510
193 2.33182 4.16115 3.89023 3.49644 3.12230 3.90096 3.35790
2.85056 3.40973 1.45839 3.33617 3.38749 4.27391 3.32375 3.20354
3.12882 3.01045 2.58144 4.48952 2.60196 299 l - - -
2.68618 4.42225 2.77519 2.73123 3.46354 2.40513 3.72494
3.29354 2.67741 2.69355 4.24690 2.90347 2.73739 3.18146 2.89801
2.37887 2.77519 2.98518 4.58477 3.61503
0.01146 4.87023 5.59257 0.61958 0.77255 0.48576 0.95510
194 2.73812 5.31734 2.41521 1.35759 4.42373 3.60111 3.55533
3.79398 2.32210 3.49292 4.08950 2.99360 3.99472 2.65819 2.89745
2.76614 3.05640 3.65214 5.75450 4.17584 300 e - - -
2.68618 4.42225 2.77519 2.73123 3.46354 2.40513 3.72494
3.29354 2.67741 2.69355 4.24690 2.90347 2.73739 3.18146 2.89801
2.37887 2.77519 2.98518 4.58477 3.61503
0.01146 4.87023 5.59257 0.61958 0.77255 0.48576 0.95510
195 2.80788 1.40650 3.91821 3.34749 3.33732 3.86650 2.89896
2.95365 3.27065 2.68048 3.58575 3.62134 4.24837 3.55620 3.35337
2.51885 3.08321 2.64136 5.05145 3.40749 301 c - - -
2.68618 4.42225 2.77519 2.73123 3.46354 2.40513 3.72494
3.29354 2.67741 2.69355 4.24690 2.90347 2.73739 3.18146 2.89801
2.37887 2.77519 2.98518 4.58477 3.61503
0.01146 4.87023 5.59257 0.61958 0.77255 0.48576 0.95510
196 4.13374 5.66898 4.43444 4.15815 2.79034 4.67891 0.48515
4.36939 3.81400 3.51853 4.87955 4.31462 5.09899 4.26509 3.99949
4.13808 4.37740 4.21603 4.44051 2.43197 302 H - - -
2.68618 4.42225 2.77519 2.73123 3.46354 2.40513 3.72494
3.29354 2.67741 2.69355 4.24690 2.90347 2.73739 3.18146 2.89801
2.37887 2.77519 2.98518 4.58477 3.61503
0.01146 4.87023 5.59257 0.61958 0.77255 0.48576 0.95510
197 2.83779 5.34265 2.96652 1.96059 4.68750 3.51790 2.63738
4.17098 2.20978 3.64601 4.38432 2.94804 4.00149 1.76424 2.68175
2.66707 3.06853 3.68070 5.77348 4.16648 303 q - - -
2.68618 4.42225 2.77519 2.73123 3.46354 2.40513 3.72494
3.29354 2.67741 2.69355 4.24690 2.90347 2.73739 3.18146 2.89801
2.37887 2.77519 2.98518 4.58477 3.61503
0.01146 4.87023 5.59257 0.61958 0.77255 0.48576 0.95510
198 2.34462 3.92865 3.64499 3.14265 3.61185 3.81431 3.86738
2.46082 2.39202 2.51457 3.14893 3.33167 4.19371 3.34395 2.60358
2.57391 2.57866 2.26107 4.83244 3.90526 304 v - - -
2.68618 4.42225 2.77519 2.73123 3.46354 2.40513 3.72494
3.29354 2.67741 2.69355 4.24690 2.90347 2.73739 3.18146 2.89801
2.37887 2.77519 2.98518 4.58477 3.61503
0.01146 4.87023 5.59257 0.61958 0.77255 0.48576 0.95510

```
    199   3.77414  5.83423  3.51346  3.33270  4.79547  4.13019  4.04759
4.86430  3.03999  4.25627  5.23522  3.79697  4.69497  0.41702  3.28559
3.75934  4.05282  4.52152  6.02204  4.62653    305 Q - - -
         2.68618  4.42225  2.77519  2.73123  3.46354  2.40513  3.72494
3.29354  2.67741  2.69355  4.24690  2.90347  2.73739  3.18146  2.89801
2.37887  2.77519  2.98518  4.58477  3.61503
         0.01146  4.87023  5.59257  0.61958  0.77255  0.48576  0.95510
    200   2.75546  3.03485  3.71574  3.14819  2.76017  3.56511  3.58437
2.96762  2.37095  2.04596  3.56616  2.68631  4.19515  3.29638  3.32984
2.70425  2.76228  2.77152  5.13703  3.10381    306 l - - -
         2.68618  4.42225  2.77519  2.73123  3.46354  2.40513  3.72494
3.29354  2.67741  2.69355  4.24690  2.90347  2.73739  3.18146  2.89801
2.37887  2.77519  2.98518  4.58477  3.61503
         0.01146  4.87023  5.59257  0.61958  0.77255  0.48576  0.95510
    201   2.74422  5.29674  3.02914  2.42506  4.62596  3.46315  2.60865
3.43111  2.16906  2.88118  4.34183  2.84307  3.99952  2.15171  2.21843
2.71776  2.98841  3.53746  5.73844  4.33871    307 q - - -
         2.68618  4.42225  2.77519  2.73123  3.46354  2.40513  3.72494
3.29354  2.67741  2.69355  4.24690  2.90347  2.73739  3.18146  2.89801
2.37887  2.77519  2.98518  4.58477  3.61503
         0.01146  4.87023  5.59257  0.61958  0.77255  0.48576  0.95510
    202   1.82992  4.36162  4.32571  3.60110  3.48374  3.98691  4.30366
1.51861  3.62779  2.49955  3.37938  3.96283  4.35895  3.69591  3.81989
3.21893  2.25126  2.21631  5.00206  3.80788    308 i - - -
         2.68618  4.42225  2.77519  2.73123  3.46354  2.40513  3.72494
3.29354  2.67741  2.69355  4.24690  2.90347  2.73739  3.18146  2.89801
2.37887  2.77519  2.98518  4.58477  3.61503
         0.01146  4.87023  5.59257  0.61958  0.77255  0.48576  0.95510
    203   2.49837  4.38444  4.88355  4.29244  3.52838  4.22935  4.59068
1.33027  4.11900  2.12576  2.90396  4.35093  4.58036  4.28440  4.18441
3.01952  3.14446  1.58257  5.11228  3.92953    309 i - - -
         2.68618  4.42225  2.77519  2.73123  3.46354  2.40513  3.72494
3.29354  2.67741  2.69355  4.24690  2.90347  2.73739  3.18146  2.89801
2.37887  2.77519  2.98518  4.58477  3.61503
         0.01146  4.87023  5.59257  0.61958  0.77255  0.48576  0.95510
    204   2.60559  5.28858  2.67178  2.38564  3.96752  3.49119  3.61886
4.08751  2.28234  3.23124  3.81310  2.99339  3.99821  2.14692  2.88128
1.93880  2.75503  3.30030  5.73277  4.24366    310 s - - -
         2.68618  4.42225  2.77519  2.73123  3.46354  2.40513  3.72494
3.29354  2.67741  2.69355  4.24690  2.90347  2.73739  3.18146  2.89801
2.37887  2.77519  2.98518  4.58477  3.61503
         0.01617  4.87023  4.78383  0.61958  0.77255  0.48576  0.95510
    205   2.73730  5.32172  2.67387  1.69837  4.40031  3.22006  3.65989
3.95950  2.48993  3.02384  4.36367  2.64778  3.82365  2.12200  2.78279
2.71243  2.95875  3.55927  5.75726  4.34919    311 e - - -
         2.68618  4.42225  2.77519  2.73123  3.46354  2.40513  3.72494
3.29354  2.67741  2.69355  4.24690  2.90347  2.73739  3.18146  2.89801
2.37887  2.77519  2.98518  4.58477  3.61503
         0.01152  4.86558  5.58792  0.61958  0.77255  0.49719  0.93711
    206   1.65531  3.21980  4.50095  3.90698  3.38793  3.82708  4.30474
2.58256  3.75657  1.85835  3.21683  4.03587  4.34890  3.94613  3.87603
2.59648  2.89591  1.95325  4.90811  3.72325    312 a - - -
         2.68618  4.42225  2.77519  2.73123  3.46354  2.40513  3.72494
3.29354  2.67741  2.69355  4.24690  2.90347  2.73739  3.18146  2.89801
2.37887  2.77519  2.98518  4.58477  3.61503
         0.02045  4.86558  4.37901  0.61958  0.77255  0.47869  0.96652
```

207 2.85389 5.30522 3.12595 2.44891 4.22558 3.62939 3.72223
4.10328 1.42459 3.59815 4.35196 2.63418 4.02051 2.84985 2.25443
2.58845 3.02841 3.68931 5.73950 3.21446 313 k - - -
2.68618 4.42225 2.77519 2.73123 3.46354 2.40513 3.72494
3.29354 2.67741 2.69355 4.24690 2.90347 2.73739 3.18146 2.89801
2.37887 2.77519 2.98518 4.58477 3.61503
0.01156 4.86143 5.58378 0.61958 0.77255 0.47258 0.97655
208 2.67623 5.06710 3.03633 2.64618 3.93949 2.77816 3.04396
3.26316 2.52440 2.64299 4.13439 2.65017 3.90726 2.99250 2.79565
2.02020 2.92949 3.18237 5.56176 3.27547 314 s - - -
2.68618 4.42225 2.77519 2.73123 3.46354 2.40513 3.72494
3.29354 2.67741 2.69355 4.24690 2.90347 2.73739 3.18146 2.89801
2.37887 2.77519 2.98518 4.58477 3.61503
0.03104 4.87023 3.77706 0.61958 0.77255 0.48576 0.95510
209 2.62970 4.34539 3.45273 3.09838 2.99421 3.71549 2.99746
2.99914 2.99504 1.44672 3.67512 3.19297 4.04970 3.15294 3.23787
2.67076 2.94320 2.73121 5.15172 3.91259 315 l - - -
2.68618 4.42225 2.77519 2.73123 3.46354 2.40513 3.72494
3.29354 2.67741 2.69355 4.24690 2.90347 2.73739 3.18146 2.89801
2.37887 2.77519 2.98518 4.58477 3.61503
0.01169 4.85088 5.57322 0.61958 0.77255 0.53213 0.88516
210 2.72218 4.49866 2.18294 2.47015 4.26871 2.86864 3.56830
3.65458 2.38561 2.96933 4.03531 2.66945 4.00007 2.78145 2.81702
2.26649 2.69835 3.27216 4.81396 4.29841 316 d - - -
2.68618 4.42225 2.77519 2.73123 3.46354 2.40513 3.72494
3.29354 2.67741 2.69355 4.24690 2.90347 2.73739 3.18146 2.89801
2.37887 2.77519 2.98518 4.58477 3.61503
0.02070 4.85088 4.36873 0.61958 0.77255 0.53213 0.88516
211 2.34646 3.36118 3.29462 3.08114 3.40698 3.78826 3.61772
2.65129 2.89591 2.11691 3.49140 2.64612 3.89612 3.34867 3.40860
2.18657 2.78621 2.68557 5.15552 3.91465 317 l - - -
2.68618 4.42225 2.77519 2.73123 3.46354 2.40513 3.72494
3.29354 2.67741 2.69355 4.24690 2.90347 2.73739 3.18146 2.89801
2.37887 2.77519 2.98518 4.58477 3.61503
0.29677 4.84197 1.39072 0.61958 0.77255 0.55249 0.85687
212 2.46997 4.86286 3.09890 2.59163 4.08647 3.22761 3.53577
3.09619 2.58251 2.84724 3.74539 2.86839 2.99267 2.79998 2.71315
2.11431 2.69027 3.04361 4.78156 3.86513 318 s - - -
2.68618 4.42225 2.77519 2.73123 3.46354 2.40513 3.72494
3.29354 2.67741 2.69355 4.24690 2.90347 2.73739 3.18146 2.89801
2.37887 2.77519 2.98518 4.58477 3.61503
0.01565 4.56084 5.28319 0.61958 0.77255 0.99199 0.46337
213 2.54322 5.09826 2.97607 2.40488 4.18760 3.29821 3.40798
3.84578 2.23501 3.06768 4.01400 2.88296 2.75914 2.61221 2.90535
2.43160 2.24035 3.35253 5.56194 4.18334 319 k - - -
2.68588 4.42231 2.77538 2.73141 3.46375 2.40527 3.72472
3.29363 2.67707 2.69373 4.24711 2.90360 2.73731 3.18126 2.89790
2.37871 2.77525 2.98525 4.58498 3.61516
0.12370 2.24927 4.52086 1.79041 0.18259 0.28859 1.38357
214 2.44339 3.91948 3.50564 3.22494 3.53633 3.14870 4.10978
2.25845 2.74348 2.39888 2.73547 3.39163 4.22030 3.49868 3.39521
2.24921 2.91239 2.41933 5.06564 3.14010 332 s - - -
2.68618 4.42225 2.77519 2.73123 3.46354 2.40513 3.72494
3.29354 2.67741 2.69355 4.24690 2.90347 2.73739 3.18146 2.89801
2.37887 2.77519 2.98518 4.58477 3.61503
0.02797 4.85326 3.92292 0.61958 0.77255 0.47725 0.96887

215 2.38852 4.73716 3.45327 2.83185 3.90774 2.96137 3.85710
2.78799 2.78555 2.84138 2.89839 3.02157 3.19114 3.19650 3.06028
2.25066 2.49638 2.39785 5.29103 4.01885 333 s - - -
2.68628 4.42232 2.77535 2.73138 3.46366 2.40515 3.72480
3.29352 2.67723 2.69363 4.24653 2.90335 2.73749 3.18163 2.89820
2.37867 2.77474 2.98522 4.58499 3.61525
0.61594 1.07508 2.13204 0.63582 0.75396 0.45670 1.00341
216 2.62861 5.25431 2.72470 2.04973 4.58448 2.83555 3.49906
3.47886 2.03969 3.43371 4.13489 2.99396 3.68171 2.75010 2.79706
2.61358 2.50237 3.10773 5.69601 4.10107 336 k - - -
2.68618 4.42225 2.77519 2.73123 3.46354 2.40513 3.72494
3.29354 2.67741 2.69355 4.24690 2.90347 2.73739 3.18146 2.89801
2.37887 2.77519 2.98518 4.58477 3.61503
0.04074 4.75088 3.46475 0.61958 0.77255 0.35800 1.20088
217 2.61851 4.98658 3.23915 2.28832 3.20792 3.65698 3.77094
3.01472 2.25411 2.79690 3.96295 3.00777 2.69786 2.97775 3.06383
2.17148 2.82386 3.06014 5.49563 4.04135 337 s - - -
2.68618 4.42225 2.77519 2.73123 3.46354 2.40513 3.72494
3.29354 2.67741 2.69355 4.24690 2.90347 2.73739 3.18146 2.89801
2.37887 2.77519 2.98518 4.58477 3.61503
0.01175 4.84590 5.56824 0.61958 0.77255 0.54359 0.86908
218 2.81614 3.82369 3.04369 2.37501 3.90188 3.61897 3.58227
3.78844 2.53321 2.82805 4.21478 2.83520 2.86873 2.37180 2.88821
2.63496 1.94373 3.51222 5.62978 4.26135 338 t - - -
2.68618 4.42225 2.77519 2.73123 3.46354 2.40513 3.72494
3.29354 2.67741 2.69355 4.24690 2.90347 2.73739 3.18146 2.89801
2.37887 2.77519 2.98518 4.58477 3.61503
0.01175 4.84590 5.56824 0.61958 0.77255 0.47058 0.97987
219 2.77592 5.28726 2.91786 2.54013 4.61689 2.65401 3.80213
4.08871 2.51582 3.50961 4.33972 2.64695 3.43906 2.86231 2.63242
1.61401 2.47847 3.67124 5.73801 4.33981 339 s - - -
2.68618 4.42225 2.77519 2.73123 3.46354 2.40513 3.72494
3.29354 2.67741 2.69355 4.24690 2.90347 2.73739 3.18146 2.89801
2.37887 2.77519 2.98518 4.58477 3.61503
0.01152 4.86499 5.58734 0.61958 0.77255 0.49861 0.93490
220 2.70541 5.34616 2.11234 1.75454 4.68901 2.60415 3.59841
3.97673 2.40386 3.52729 4.38810 2.72322 3.50397 2.68243 3.02874
2.59914 3.06800 3.73574 5.77943 4.36728 340 e - - -
2.68618 4.42225 2.77519 2.73123 3.46354 2.40513 3.72494
3.29354 2.67741 2.69355 4.24690 2.90347 2.73739 3.18146 2.89801
2.37887 2.77519 2.98518 4.58477 3.61503
0.02107 4.86499 4.33216 0.61958 0.77255 0.47781 0.96795
221 2.55890 4.82070 2.89573 2.55520 4.13843 3.64772 3.10299
2.96819 2.64158 2.79080 4.12353 3.05254 3.07692 2.85086 2.67256
2.00494 2.73739 3.11027 5.19641 3.69878 341 s - - -
2.68618 4.42225 2.77519 2.73123 3.46354 2.40513 3.72494
3.29354 2.67741 2.69355 4.24690 2.90347 2.73739 3.18146 2.89801
2.37887 2.77519 2.98518 4.58477 3.61503
0.01157 4.86084 5.58318 0.61958 0.77255 0.50864 0.91957
222 2.40162 5.06599 3.20062 2.57889 4.31602 3.29175 1.70508
3.63957 2.55995 2.91350 4.13316 3.11569 3.73948 2.57677 2.89189
2.74921 3.05551 3.19220 4.91559 3.85169 342 h - - -
2.68618 4.42225 2.77519 2.73123 3.46354 2.40513 3.72494
3.29354 2.67741 2.69355 4.24690 2.90347 2.73739 3.18146 2.89801
2.37887 2.77519 2.98518 4.58477 3.61503
0.01157 4.86084 5.58318 0.61958 0.77255 0.47172 0.97798

```
    223   2.14512  3.87759  3.34834  2.78836  3.86672  3.49960  2.85065
3.35779  2.62405  2.64236  3.59010  3.28349  3.78679  2.99797  1.90698
2.61864  3.06139  3.10076  5.39857  3.78612    343 r - - -
          2.68618  4.42225  2.77519  2.73123  3.46354  2.40513  3.72494
3.29354  2.67741  2.69355  4.24690  2.90347  2.73739  3.18146  2.89801
2.37887  2.77519  2.98518  4.58477  3.61503
          0.01146  4.87023  5.59257  0.61958  0.77255  0.48576  0.95510
    224   2.80355  5.32989  2.51346  2.11557  4.49062  3.59952  3.43589
4.15688  2.00548  2.95065  4.11157  3.01618  3.83385  1.95992  2.55155
2.75814  2.99111  3.71942  5.76356  4.35451    344 q - - -
          2.68618  4.42225  2.77519  2.73123  3.46354  2.40513  3.72494
3.29354  2.67741  2.69355  4.24690  2.90347  2.73739  3.18146  2.89801
2.37887  2.77519  2.98518  4.58477  3.61503
          0.01146  4.87023  5.59257  0.61958  0.77255  0.48576  0.95510
    225   1.40810  4.78120  3.34144  2.70009  3.81603  3.72892  3.79264
2.97818  2.84320  2.67756  3.87087  3.34261  4.11434  3.05951  2.86719
2.50021  2.92866  2.75049  5.32935  4.04981    345 a - - -
          2.68618  4.42225  2.77519  2.73123  3.46354  2.40513  3.72494
3.29354  2.67741  2.69355  4.24690  2.90347  2.73739  3.18146  2.89801
2.37887  2.77519  2.98518  4.58477  3.61503
          0.01146  4.87023  5.59257  0.61958  0.77255  0.48576  0.95510
    226   1.97111  4.36658  4.17509  3.59436  3.40266  3.92589  4.22376
2.56791  3.49496  2.42085  3.03626  3.85457  4.29918  3.73717  3.72008
2.97297  1.39583  2.53197  4.63934  3.77967    346 t - - -
          2.68618  4.42225  2.77519  2.73123  3.46354  2.40513  3.72494
3.29354  2.67741  2.69355  4.24690  2.90347  2.73739  3.18146  2.89801
2.37887  2.77519  2.98518  4.58477  3.61503
          0.01146  4.87023  5.59257  0.61958  0.77255  0.48576  0.95510
    227   2.49617  4.19913  3.77846  3.30539  3.38098  3.77601  4.12545
2.47657  2.60683  1.81031  2.84862  3.47286  4.23332  3.38794  2.66518
2.55685  2.98338  2.47223  5.06268  3.77897    347 l - - -
          2.68618  4.42225  2.77519  2.73123  3.46354  2.40513  3.72494
3.29354  2.67741  2.69355  4.24690  2.90347  2.73739  3.18146  2.89801
2.37887  2.77519  2.98518  4.58477  3.61503
          0.02569  4.87023  4.03476  0.61958  0.77255  0.48576  0.95510
    228   2.54898  5.32485  2.64415  2.14167  4.66772  3.59377  3.16854
4.15193  2.19977  3.29988  4.20638  2.72061  3.98743  1.74830  2.88358
2.78312  2.97997  3.64272  5.75864  4.21451    348 q - - -
          2.68618  4.42225  2.77519  2.73123  3.46354  2.40513  3.72494
3.29354  2.67741  2.69355  4.24690  2.90347  2.73739  3.18146  2.89801
2.37887  2.77519  2.98518  4.58477  3.61503
          0.01163  4.85616  5.57851  0.61958  0.77255  0.46507  0.98910
    229   4.25728  5.45507  6.07493  5.54482  3.23616  5.61038  5.82525
2.71622  5.39357  0.34373  3.26370  5.76852  5.59923  5.22744  5.33411
5.04920  4.46612  2.74986  5.73581  4.67706    349 L - - -
          2.68618  4.42225  2.77519  2.73123  3.46354  2.40513  3.72494
3.29354  2.67741  2.69355  4.24690  2.90347  2.73739  3.18146  2.89801
2.37887  2.77519  2.98518  4.58477  3.61503
          0.01146  4.87023  5.59257  0.61958  0.77255  0.48576  0.95510
    230   2.82814  4.56811  3.15034  1.23578  4.35323  3.38189  3.84393
3.54731  2.43758  2.98724  4.16038  3.15935  4.03562  2.81873  3.01297
2.79858  3.05930  3.15495  4.47959  4.14498    350 e - - -
          2.68618  4.42225  2.77519  2.73123  3.46354  2.40513  3.72494
3.29354  2.67741  2.69355  4.24690  2.90347  2.73739  3.18146  2.89801
2.37887  2.77519  2.98518  4.58477  3.61503
          0.01146  4.87023  5.59257  0.61958  0.77255  0.48576  0.95510
```

```
    231   2.18198  4.84297  3.09853  2.63224  3.53952  3.65681  3.39267
3.13511  2.59140  2.16800  3.43817  2.99321  4.09546  3.09067  2.90449
2.62866  2.61995  2.60493  5.38047  3.99822    351 l - - -
          2.68618  4.42225  2.77519  2.73123  3.46354  2.40513  3.72494
3.29354  2.67741  2.69355  4.24690  2.90347  2.73739  3.18146  2.89801
2.37887  2.77519  2.98518  4.58477  3.61503
          0.01146  4.87023  5.59257  0.61958  0.77255  0.48576  0.95510
    232   2.41521  5.25846  2.96704  1.23965  4.56871  3.61683  3.81307
3.77607  2.50488  3.54872  4.13732  3.09940  4.01328  2.56727  3.04973
2.77495  3.00920  3.20973  5.71318  4.32426    352 e - - -
          2.68618  4.42225  2.77519  2.73123  3.46354  2.40513  3.72494
3.29354  2.67741  2.69355  4.24690  2.90347  2.73739  3.18146  2.89801
2.37887  2.77519  2.98518  4.58477  3.61503
          0.01146  4.87023  5.59257  0.61958  0.77255  0.48576  0.95510
    233   3.20240  4.75986  5.40797  4.82875  3.48698  4.76590  5.14145
2.04870  4.66964  0.92949  2.79892  4.91046  5.02118  4.75174  4.69828
4.10963  3.55183  1.58015  5.48710  4.37852    353 l - - -
          2.68618  4.42225  2.77519  2.73123  3.46354  2.40513  3.72494
3.29354  2.67741  2.69355  4.24690  2.90347  2.73739  3.18146  2.89801
2.37887  2.77519  2.98518  4.58477  3.61503
          0.01146  4.87023  5.59257  0.61958  0.77255  0.48576  0.95510
    234   2.82449  4.97792  2.94400  2.31349  4.57229  3.25348  3.51346
3.39044  2.47322  2.54286  4.30706  2.85882  4.00337  2.37252  2.28052
2.31504  2.65400  3.42803  5.70977  4.05408    354 r - - -
          2.68618  4.42225  2.77519  2.73123  3.46354  2.40513  3.72494
3.29354  2.67741  2.69355  4.24690  2.90347  2.73739  3.18146  2.89801
2.37887  2.77519  2.98518  4.58477  3.61503
          0.01146  4.87023  5.59257  0.61958  0.77255  0.48576  0.95510
    235   2.50756  4.50133  2.88071  2.23977  4.34410  3.47388  3.57492
4.13316  2.08888  3.55786  4.35833  1.93785  3.99386  2.68322  2.87742
2.32008  2.93161  3.57415  5.75302  3.82341    355 n - - -
          2.68618  4.42225  2.77519  2.73123  3.46354  2.40513  3.72494
3.29354  2.67741  2.69355  4.24690  2.90347  2.73739  3.18146  2.89801
2.37887  2.77519  2.98518  4.58477  3.61503
          0.01146  4.87023  5.59257  0.61958  0.77255  0.48576  0.95510
    236   4.39230  5.51734  5.57870  5.32294  2.05911  5.16540  4.11647
3.87580  5.10243  2.28844  4.41829  4.84838  5.42667  4.92496  5.00305
4.54014  4.59913  3.87406  0.64145  2.39884    356 W - - -
          2.68618  4.42225  2.77519  2.73123  3.46354  2.40513  3.72494
3.29354  2.67741  2.69355  4.24690  2.90347  2.73739  3.18146  2.89801
2.37887  2.77519  2.98518  4.58477  3.61503
          0.01146  4.87023  5.59257  0.61958  0.77255  0.48576  0.95510
    237   2.75355  2.87131  3.17081  2.71792  4.17542  3.25135  2.53429
2.92483  2.62004  3.12644  4.03334  3.03809  4.06563  2.87982  2.46181
2.49565  2.79049  3.27755  5.47347  2.84789    357 r - - -
          2.68618  4.42225  2.77519  2.73123  3.46354  2.40513  3.72494
3.29354  2.67741  2.69355  4.24690  2.90347  2.73739  3.18146  2.89801
2.37887  2.77519  2.98518  4.58477  3.61503
          0.01146  4.87023  5.59257  0.61958  0.77255  0.48576  0.95510
    238   2.31875  4.84410  3.09653  2.52115  4.26438  3.52250  3.62471
3.41423  2.61604  2.74199  4.05859  2.54122  4.03026  2.79528  2.75566
1.79145  2.72372  3.25500  5.59853  4.24286    358 s - - -
          2.68618  4.42225  2.77519  2.73123  3.46354  2.40513  3.72494
3.29354  2.67741  2.69355  4.24690  2.90347  2.73739  3.18146  2.89801
2.37887  2.77519  2.98518  4.58477  3.61503
          0.01146  4.87023  5.59257  0.61958  0.77255  0.48576  0.95510
```

239 2.57454 2.50917 3.10976 2.61681 4.39249 3.33752 3.64807
3.82666 2.61317 3.39692 4.09338 2.59252 4.02974 2.59399 2.92943
1.73979 2.84134 3.47317 5.60827 4.11664 359 s - - -
2.68618 4.42225 2.77519 2.73123 3.46354 2.40513 3.72494
3.29354 2.67741 2.69355 4.24690 2.90347 2.73739 3.18146 2.89801
2.37887 2.77519 2.98518 4.58477 3.61503
0.01146 4.87023 5.59257 0.61958 0.77255 0.48576 0.95510
240 4.10115 5.32098 5.69558 5.23739 0.64187 5.22519 4.76576
3.01165 5.05486 1.62120 3.19770 5.15133 5.37829 4.95145 5.00990
4.59765 4.30760 3.21250 4.86536 3.22095 360 F - - -
2.68618 4.42225 2.77519 2.73123 3.46354 2.40513 3.72494
3.29354 2.67741 2.69355 4.24690 2.90347 2.73739 3.18146 2.89801
2.37887 2.77519 2.98518 4.58477 3.61503
0.01146 4.87023 5.59257 0.61958 0.77255 0.48576 0.95510
241 2.45198 2.86509 2.59059 2.26080 4.44405 3.46379 3.58348
4.09197 2.44852 3.42848 4.19439 2.53923 3.99780 2.67654 2.87933
2.10704 2.68923 3.57366 5.73480 4.33489 361 s - - -
2.68618 4.42225 2.77519 2.73123 3.46354 2.40513 3.72494
3.29354 2.67741 2.69355 4.24690 2.90347 2.73739 3.18146 2.89801
2.37887 2.77519 2.98518 4.58477 3.61503
0.01146 4.87023 5.59257 0.61958 0.77255 0.48576 0.95510
242 2.43484 4.99226 2.74892 2.15693 4.43139 3.12800 3.58060
3.90148 1.96935 3.54933 4.36795 2.29810 3.99215 2.88358 2.64929
2.35527 2.83954 3.63869 5.76109 4.35219 362 k - - -
2.68618 4.42225 2.77519 2.73123 3.46354 2.40513 3.72494
3.29354 2.67741 2.69355 4.24690 2.90347 2.73739 3.18146 2.89801
2.37887 2.77519 2.98518 4.58477 3.61503
0.01146 4.87023 5.59257 0.61958 0.77255 0.48576 0.95510
243 3.08444 4.23989 4.81850 4.24049 3.09781 4.21571 4.35084
2.78341 4.05968 1.53524 3.41771 4.27748 4.56212 4.19465 4.12097
3.52555 3.21333 2.64025 1.38972 2.63795 363 w - - -
2.68618 4.42225 2.77519 2.73123 3.46354 2.40513 3.72494
3.29354 2.67741 2.69355 4.24690 2.90347 2.73739 3.18146 2.89801
2.37887 2.77519 2.98518 4.58477 3.61503
0.01146 4.87023 5.59257 0.61958 0.77255 0.48576 0.95510
244 3.07590 4.66284 5.37966 4.82930 3.47935 4.75720 5.21894
1.09168 4.68926 2.41319 2.87521 4.90282 5.05095 4.86034 4.76344
4.11188 3.43118 1.19592 5.65302 4.47154 364 i - - -
2.68618 4.42225 2.77519 2.73123 3.46354 2.40513 3.72494
3.29354 2.67741 2.69355 4.24690 2.90347 2.73739 3.18146 2.89801
2.37887 2.77519 2.98518 4.58477 3.61503
0.01146 4.87023 5.59257 0.61958 0.77255 0.48576 0.95510
245 2.53340 5.32617 2.61503 2.42044 4.66770 3.20785 3.66178
3.89314 2.05328 3.62992 4.27654 2.44028 3.99238 2.77679 2.61026
2.23559 2.34283 3.55030 5.76096 4.35218 365 k - - -
2.68618 4.42225 2.77519 2.73123 3.46354 2.40513 3.72494
3.29354 2.67741 2.69355 4.24690 2.90347 2.73739 3.18146 2.89801
2.37887 2.77519 2.98518 4.58477 3.61503
0.01146 4.87023 5.59257 0.61958 0.77255 0.48576 0.95510
246 1.68958 4.30381 3.69336 3.07306 3.22597 3.72865 3.25807
2.63998 3.01312 2.67086 3.67120 3.31827 4.18948 3.34191 3.44192
2.09023 2.68738 2.77391 5.14888 3.36130 366 a - - -
2.68618 4.42225 2.77519 2.73123 3.46354 2.40513 3.72494
3.29354 2.67741 2.69355 4.24690 2.90347 2.73739 3.18146 2.89801
2.37887 2.77519 2.98518 4.58477 3.61503
0.01146 4.87023 5.59257 0.61958 0.77255 0.48576 0.95510

247 3.14176 5.32760 3.44938 2.91516 4.03908 3.88276 2.38902
4.02757 2.63304 3.46398 4.16713 3.39907 4.27507 1.00372 2.97482
3.14902 3.35861 3.71176 5.39735 3.57395 367 q - - -
2.68618 4.42225 2.77519 2.73123 3.46354 2.40513 3.72494
3.29354 2.67741 2.69355 4.24690 2.90347 2.73739 3.18146 2.89801
2.37887 2.77519 2.98518 4.58477 3.61503
0.01146 4.87023 5.59257 0.61958 0.77255 0.48576 0.95510
248 3.12538 5.55024 3.44074 2.95239 4.97864 3.93912 3.94828
4.08920 1.36343 3.80192 4.61004 3.42294 4.30246 2.93684 1.20054
3.21270 3.08296 3.99373 5.86491 4.61196 368 r - - -
2.68618 4.42225 2.77519 2.73123 3.46354 2.40513 3.72494
3.29354 2.67741 2.69355 4.24690 2.90347 2.73739 3.18146 2.89801
2.37887 2.77519 2.98518 4.58477 3.61503
0.01146 4.87023 5.59257 0.61958 0.77255 0.48576 0.95510
249 2.13986 4.61005 2.37088 2.22123 4.31438 2.87652 3.73211
4.11331 2.51287 3.42912 4.34864 2.65623 3.99655 2.67108 3.02306
1.98961 3.05631 3.58680 5.74503 4.34194 369 s - - -
2.68618 4.42225 2.77519 2.73123 3.46354 2.40513 3.72494
3.29354 2.67741 2.69355 4.24690 2.90347 2.73739 3.18146 2.89801
2.37887 2.77519 2.98518 4.58477 3.61503
0.01146 4.87023 5.59257 0.61958 0.77255 0.48576 0.95510
250 4.40138 3.77019 5.50926 5.27417 1.86700 5.11385 3.72737
4.12834 5.06891 3.47025 4.70661 4.78017 5.40774 4.89524 4.97972
4.48801 4.61672 4.01819 4.14472 0.46820 370 Y - - -
2.68618 4.42225 2.77519 2.73123 3.46354 2.40513 3.72494
3.29354 2.67741 2.69355 4.24690 2.90347 2.73739 3.18146 2.89801
2.37887 2.77519 2.98518 4.58477 3.61503
0.01146 4.87023 5.59257 0.61958 0.77255 0.48576 0.95510
251 2.91695 4.77820 5.56972 5.05222 4.05726 5.01868 5.57382
1.39227 4.94804 2.14270 3.41530 5.16317 5.27857 5.14756 5.05751
4.40149 3.78680 0.85596 5.96951 4.77802 371 v - - -
2.68618 4.42225 2.77519 2.73123 3.46354 2.40513 3.72494
3.29354 2.67741 2.69355 4.24690 2.90347 2.73739 3.18146 2.89801
2.37887 2.77519 2.98518 4.58477 3.61503
0.01146 4.87023 5.59257 0.61958 0.77255 0.48576 0.95510
252 2.83377 4.67112 3.08773 1.79868 4.67384 3.35166 2.85902
4.15488 1.97626 3.29899 4.37297 2.93846 4.00015 2.48897 2.25984
2.71604 2.98959 3.72059 5.76295 4.35817 372 e - - -
2.68618 4.42225 2.77519 2.73123 3.46354 2.40513 3.72494
3.29354 2.67741 2.69355 4.24690 2.90347 2.73739 3.18146 2.89801
2.37887 2.77519 2.98518 4.58477 3.61503
0.01146 4.87023 5.59257 0.61958 0.77255 0.48576 0.95510
253 1.38764 4.64828 3.59277 2.96781 3.57440 3.47114 4.03325
2.96502 2.99783 2.81025 3.76266 3.13697 4.16225 3.31406 3.38414
2.21300 2.82100 2.55147 5.23370 3.98230 373 a - - -
2.68618 4.42225 2.77519 2.73123 3.46354 2.40513 3.72494
3.29354 2.67741 2.69355 4.24690 2.90347 2.73739 3.18146 2.89801
2.37887 2.77519 2.98518 4.58477 3.61503
0.01146 4.87023 5.59257 0.61958 0.77255 0.48576 0.95510
254 4.21893 5.39393 6.13821 5.57203 3.54119 5.67601 5.97682
2.23937 5.44151 0.40175 3.15054 5.83181 5.61808 5.26610 5.38028
5.10140 4.41916 2.62340 5.85885 4.94457 374 L - - -
2.68618 4.42225 2.77519 2.73123 3.46354 2.40513 3.72494
3.29354 2.67741 2.69355 4.24690 2.90347 2.73739 3.18146 2.89801
2.37887 2.77519 2.98518 4.58477 3.61503
0.01146 4.87023 5.59257 0.61958 0.77255 0.48576 0.95510

```
     255   2.59261  4.35427  2.95748  2.59485  4.20640  3.51938  2.86028
  3.88503  2.56047  3.44028  4.22214  1.57522  4.02163  2.92028  3.07227
  2.52576  2.59733  3.51820  5.63754  3.62672    375 n - - -
           2.68618  4.42225  2.77519  2.73123  3.46354  2.40513  3.72494
  3.29354  2.67741  2.69355  4.24690  2.90347  2.73739  3.18146  2.89801
  2.37887  2.77519  2.98518  4.58477  3.61503
           0.01146  4.87023  5.59257  0.61958  0.77255  0.48576  0.95510
     256   2.62130  5.30144  3.01235  2.11514  4.62871  1.77643  3.80222
  4.10230  2.46710  3.41346  4.34842  2.85047  4.00475  2.77144  2.89690
  2.19427  2.89519  3.60233  5.74560  4.34518    376 g - - -
           2.68618  4.42225  2.77519  2.73123  3.46354  2.40513  3.72494
  3.29354  2.67741  2.69355  4.24690  2.90347  2.73739  3.18146  2.89801
  2.37887  2.77519  2.98518  4.58477  3.61503
           0.01146  4.87023  5.59257  0.61958  0.77255  0.48576  0.95510
     257   4.75254  5.88439  5.47698  5.33176  2.91378  5.06493  4.43751
  4.58741  4.95126  3.77361  5.13227  5.04741  5.50667  5.12285  4.93847
  4.80223  5.02910  4.53690  0.29236  2.72631    377 W - - -
           2.68618  4.42225  2.77519  2.73123  3.46354  2.40513  3.72494
  3.29354  2.67741  2.69355  4.24690  2.90347  2.73739  3.18146  2.89801
  2.37887  2.77519  2.98518  4.58477  3.61503
           0.01146  4.87023  5.59257  0.61958  0.77255  0.48576  0.95510
     258   3.49970  5.13784  5.80082  5.22770  3.22196  5.25744  5.58952
  2.08948  5.09295  0.51942  3.31119  5.39857  5.36424  5.04013  5.08298
  4.63300  4.11055  2.46515  5.70108  4.69515    378 L - - -
           2.68618  4.42225  2.77519  2.73123  3.46354  2.40513  3.72494
  3.29354  2.67741  2.69355  4.24690  2.90347  2.73739  3.18146  2.89801
  2.37887  2.77519  2.98518  4.58477  3.61503
           0.01146  4.87023  5.59257  0.61958  0.77255  0.48576  0.95510
     259   2.75018  5.08305  3.19571  2.63806  4.05080  3.41607  3.40524
  3.57568  2.08389  2.48762  2.94788  2.94939  4.03734  2.55205  2.31535
  2.45286  3.02044  3.07690  5.57424  4.22636    379 k - - -
           2.68618  4.42225  2.77519  2.73123  3.46354  2.40513  3.72494
  3.29354  2.67741  2.69355  4.24690  2.90347  2.73739  3.18146  2.89801
  2.37887  2.77519  2.98518  4.58477  3.61503
           0.01146  4.87023  5.59257  0.61958  0.77255  0.48576  0.95510
     260   3.06391  5.24827  3.49296  2.87900  4.34661  3.84062  3.82677
  3.94084  1.49052  1.76841  4.31870  3.06363  4.21308  3.07235  1.98830
  3.01909  3.21029  3.61801  5.67005  4.39118    380 k - - -
           2.68618  4.42225  2.77519  2.73123  3.46354  2.40513  3.72494
  3.29354  2.67741  2.69355  4.24690  2.90347  2.73739  3.18146  2.89801
  2.37887  2.77519  2.98518  4.58477  3.61503
           0.01146  4.87023  5.59257  0.61958  0.77255  0.48576  0.95510
     261   2.78172  1.88204  4.17103  3.58952  2.88527  3.56448  4.21324
  2.74237  3.48958  2.55550  3.47109  2.89168  4.29156  3.73044  3.57463
  2.69739  2.35480  2.61437  2.96700  3.44250    381 c - - -
           2.68618  4.42225  2.77519  2.73123  3.46354  2.40513  3.72494
  3.29354  2.67741  2.69355  4.24690  2.90347  2.73739  3.18146  2.89801
  2.37887  2.77519  2.98518  4.58477  3.61503
           0.06957  4.87023  2.82125  0.61958  0.77255  0.48576  0.95510
     262   2.71196  4.28788  4.79868  4.19399  2.90239  3.91694  4.42810
  1.99058  4.00243  1.22828  3.04393  3.96998  4.44516  4.14662  4.04222
  3.40289  3.14460  1.94053  4.93298  3.75952    382 l - - -
           2.68618  4.42225  2.77519  2.73123  3.46354  2.40513  3.72494
  3.29354  2.67741  2.69355  4.24690  2.90347  2.73739  3.18146  2.89801
  2.37887  2.77519  2.98518  4.58477  3.61503
           0.37623  4.81280  1.18604  0.61958  0.77255  0.61522  0.77766
```

```
    263   2.55085  4.27149  3.56359  3.21860  2.75123  3.54991  3.98753
2.28372  2.89796  2.08877  3.17830  3.55157  3.75028  3.30516  2.87388
2.79091  2.90745  2.23321  4.86917  3.51284    383 l - - -
          2.68618  4.42225  2.77519  2.73123  3.46354  2.40513  3.72494
3.29354  2.67741  2.69355  4.24690  2.90347  2.73739  3.18146  2.89801
2.37887  2.77519  2.98518  4.58477  3.61503
          0.05408  4.45401  3.19388  0.61958  0.77255  1.09726  0.40614
    264   2.66974  3.85697  3.23243  2.62068  3.51542  3.55573  3.56716
3.22707  2.65138  2.60605  3.74257  3.15711  2.34873  2.37360  3.07992
2.48664  2.79378  2.88212  5.19717  3.91046    384 p - - -
          2.68618  4.42225  2.77519  2.73123  3.46354  2.40513  3.72494
3.29354  2.67741  2.69355  4.24690  2.90347  2.73739  3.18146  2.89801
2.37887  2.77519  2.98518  4.58477  3.61503
          0.01808  4.41800  5.14035  0.61958  0.77255  0.99168  0.46355
    265   2.59045  4.33018  2.74726  2.41431  3.39271  3.37907  3.77944
2.68853  2.57873  2.75541  3.79591  3.14630  2.69135  2.97599  3.02762
2.60839  2.67147  2.80738  5.24704  3.95133    385 e - - -
          2.68618  4.42225  2.77519  2.73123  3.46354  2.40513  3.72494
3.29354  2.67741  2.69355  4.24690  2.90347  2.73739  3.18146  2.89801
2.37887  2.77519  2.98518  4.58477  3.61503
          0.01709  4.47351  5.19586  0.61958  0.77255  1.07965  0.41508
    266   2.68914  4.85498  2.71741  2.08888  4.27632  3.20535  3.45219
3.88382  2.41822  2.93011  4.02440  2.86072  3.04029  2.51989  2.70263
2.39678  2.87852  3.02768  5.56610  4.17737    386 e - - -
          2.68618  4.42225  2.77519  2.73123  3.46354  2.40513  3.72494
3.29354  2.67741  2.69355  4.24690  2.90347  2.73739  3.18146  2.89801
2.37887  2.77519  2.98518  4.58477  3.61503
          0.01709  4.47351  5.19586  0.61958  0.77255  1.07965  0.41508
    267   2.64339  5.18529  2.49075  2.02540  4.52332  3.13768  3.51399
3.88604  2.09872  3.31566  4.22795  2.74987  3.65881  2.69127  2.68703
2.35396  2.85316  3.38643  5.62203  4.21526    387 e - - -
          2.68618  4.42225  2.77519  2.73123  3.46354  2.40513  3.72494
3.29354  2.67741  2.69355  4.24690  2.90347  2.73739  3.18146  2.89801
2.37887  2.77519  2.98518  4.58477  3.61503
          0.01709  4.47351  5.19586  0.61958  0.77255  0.99873  0.45941
    268   2.56823  5.09456  2.78429  2.35766  3.52524  3.24903  3.68321
3.72750  2.31540  3.38054  4.14728  2.58387  3.04481  2.76147  2.45679
2.32123  2.78909  3.28683  5.16660  3.90860    388 k - - -
          2.68603  4.42211  2.77542  2.73135  3.46376  2.40533  3.72475
3.29388  2.67741  2.69343  4.24732  2.90357  2.73719  3.18164  2.89790
2.37854  2.77502  2.98529  4.58519  3.61484
          0.51850  0.91826  5.22652  1.39088  0.28616  0.93913  0.49589
    269   2.47908  5.09233  2.93206  2.43527  4.38725  3.33111  3.69995
3.83907  2.27639  2.73723  4.14672  2.88195  3.03633  2.63492  2.61318
2.14171  2.71067  3.31106  5.55626  4.00743    395 s - - -
          2.68618  4.42225  2.77519  2.73123  3.46354  2.40513  3.72494
3.29354  2.67741  2.69355  4.24690  2.90347  2.73739  3.18146  2.89801
2.37887  2.77519  2.98518  4.58477  3.61503
          0.02678  4.54555  4.14681  0.61958  0.77255  0.39230  1.12548
    270   2.64840  4.37019  2.96451  2.22894  4.45371  3.45883  3.11458
3.96479  1.98398  3.04517  4.06670  2.84157  3.00113  2.72682  2.40281
2.51532  3.02659  3.25489  5.70805  4.30751    396 k - - -
          2.68618  4.42225  2.77519  2.73123  3.46354  2.40513  3.72494
3.29354  2.67741  2.69355  4.24690  2.90347  2.73739  3.18146  2.89801
2.37887  2.77519  2.98518  4.58477  3.61503
          0.11549  4.79407  2.29473  0.61958  0.77255  0.61387  0.77925
```

271 2.65975 5.23011 3.03036 2.14029 4.55715 2.93681 3.36351
3.93438 2.30164 3.47628 4.02138 2.92380 3.76298 2.34500 2.52919
2.33129 2.95349 3.50548 5.67361 2.86803 397 e - - -
2.68618 4.42225 2.77519 2.73123 3.46354 2.40513 3.72494
3.29354 2.67741 2.69355 4.24690 2.90347 2.73739 3.18146 2.89801
2.37887 2.77519 2.98518 4.58477 3.61503
0.01355 4.70396 5.42630 0.61958 0.77255 0.80739 0.59063
272 2.76368 5.25042 2.88776 1.79109 4.40638 3.44895 3.72899
3.78456 2.08253 3.55142 4.15735 2.94181 3.30186 2.71331 2.43841
2.33967 2.94836 3.25295 5.68894 4.16523 398 e - - -
2.68618 4.42225 2.77519 2.73123 3.46354 2.40513 3.72494
3.29354 2.67741 2.69355 4.24690 2.90347 2.73739 3.18146 2.89801
2.37887 2.77519 2.98518 4.58477 3.61503
0.03664 4.70396 3.61488 0.61958 0.77255 0.80739 0.59063
273 2.71544 5.20355 2.87815 2.37261 4.52309 3.40553 3.72979
3.77038 2.38502 3.43649 4.25111 2.97777 2.56310 2.33466 2.32998
2.30259 2.88119 2.96348 5.65169 4.14005 399 s - - -
2.68618 4.42225 2.77519 2.73123 3.46354 2.40513 3.72494
3.29354 2.67741 2.69355 4.24690 2.90347 2.73739 3.18146 2.89801
2.37887 2.77519 2.98518 4.58477 3.61503
0.02841 4.68118 3.97701 0.61958 0.77255 0.84106 0.56432
274 2.63602 5.22889 2.82934 1.70671 4.56043 3.18119 3.71828
3.94191 2.33512 3.22826 4.08160 3.00248 3.50778 2.62999 2.68508
2.24202 2.89574 3.36385 5.66972 4.26715 400 e - - -
2.68618 4.42225 2.77519 2.73123 3.46354 2.40513 3.72494
3.29354 2.67741 2.69355 4.24690 2.90347 2.73739 3.18146 2.89801
2.37887 2.77519 2.98518 4.58477 3.61503
0.10108 4.66683 2.44492 0.61958 0.77255 0.86132 0.54923
275 2.57437 5.18391 2.78154 1.94847 4.16517 3.21559 3.69343
3.66237 2.34777 3.41117 4.22997 2.97965 3.52279 2.26905 2.80097
2.27991 2.81778 3.22527 5.62888 4.23046 401 e - - -
2.68618 4.42225 2.77519 2.73123 3.46354 2.40513 3.72494
3.29354 2.67741 2.69355 4.24690 2.90347 2.73739 3.18146 2.89801
2.37887 2.77519 2.98518 4.58477 3.61503
0.06892 4.58109 2.87605 0.61958 0.77255 0.96933 0.47697
276 2.52729 4.80800 3.18557 2.63259 4.04280 3.55722 3.78705
3.44340 2.54986 3.07549 3.64691 2.92909 2.63764 2.88081 2.95938
2.77687 1.71075 3.00705 5.34804 4.03842 402 t - - -
2.68618 4.42225 2.77519 2.73123 3.46354 2.40513 3.72494
3.29354 2.67741 2.69355 4.24690 2.90347 2.73739 3.18146 2.89801
2.37887 2.77519 2.98518 4.58477 3.61503
0.06272 4.52834 2.99574 0.61958 0.77255 1.00580 0.45532
277 2.23818 5.12365 2.64473 2.30028 4.42308 3.49028 3.65108
3.87377 2.53450 3.41909 4.20342 2.98930 1.85631 2.73955 3.02234
2.55022 3.00015 3.38265 5.60880 4.22721 403 p - - -
2.68618 4.42225 2.77519 2.73123 3.46354 2.40513 3.72494
3.29354 2.67741 2.69355 4.24690 2.90347 2.73739 3.18146 2.89801
2.37887 2.77519 2.98518 4.58477 3.61503
0.06395 4.49016 2.98118 0.61958 0.77255 0.91515 0.51159
278 2.64358 4.50421 1.78239 2.32245 4.43814 3.38089 3.50442
3.90070 2.39390 3.41759 4.17725 2.88231 3.66906 2.75831 2.50898
2.68085 2.88108 3.30964 5.58047 3.69302 404 d - - -
2.68618 4.42225 2.77519 2.73123 3.46354 2.40513 3.72494
3.29354 2.67741 2.69355 4.24690 2.90347 2.73739 3.18146 2.89801
2.37887 2.77519 2.98518 4.58477 3.61503
0.05706 4.50314 3.11476 0.61958 0.77255 1.05161 0.42982

279 2.60575 4.18913 2.76995 2.38339 4.26560 2.06503 3.69225
3.58768 2.43530 3.21127 3.92932 3.00017 3.34482 2.73821 2.59689
2.62147 2.88123 3.17969 5.47514 4.11462 405 g - - -
2.68618 4.42225 2.77519 2.73123 3.46354 2.40513 3.72494
3.29354 2.67741 2.69355 4.24690 2.90347 2.73739 3.18146 2.89801
2.37887 2.77519 2.98518 4.58477 3.61503
0.02434 4.46334 4.37987 0.61958 0.77255 0.76885 0.62277
280 2.27514 4.16404 4.33300 3.58087 2.71964 3.84875 4.16159
2.25538 3.59460 1.88048 3.28034 3.88620 3.83184 3.33170 3.65777
2.99804 2.67588 1.91239 4.19381 3.49964 406 l - - -
2.68618 4.42225 2.77519 2.73123 3.46354 2.40513 3.72494
3.29354 2.67741 2.69355 4.24690 2.90347 2.73739 3.18146 2.89801
2.37887 2.77519 2.98518 4.58477 3.61503
0.02117 4.58894 4.53003 0.61958 0.77255 0.92401 0.50571
281 2.21396 4.64301 3.08229 2.53090 3.91936 3.47981 3.59045
3.44512 2.57994 3.10377 3.97094 2.73864 2.58408 2.85323 2.96994
2.23114 2.67522 2.89171 4.80502 4.07808 407 a - - -
2.68631 4.42239 2.77533 2.73137 3.46367 2.40524 3.72508
3.29359 2.67750 2.69337 4.24703 2.90360 2.73596 3.18160 2.89809
2.37898 2.77533 2.98532 4.58490 3.61517
1.10738 0.67829 1.81958 0.08372 2.52182 0.95291 0.48714
282 2.67599 4.74780 2.97181 2.46318 2.25512 3.53666 3.51009
3.28359 2.45516 2.99070 3.82877 3.10471 3.92467 2.93151 2.47405
2.65133 2.72134 3.06114 5.27253 3.31138 409 f - - -
2.68630 4.42237 2.77511 2.73135 3.46365 2.40522 3.72506
3.29366 2.67752 2.69366 4.24689 2.90343 2.73751 3.18158 2.89805
2.37806 2.77531 2.98530 4.58488 3.61505
0.73837 0.66109 5.14656 0.09572 2.39379 1.12294 0.39352
283 2.68195 5.04704 2.92604 2.37361 4.10586 3.28797 3.41091
3.69038 2.40418 3.32780 4.10335 2.71081 2.04535 2.64576 2.65804
2.58473 2.91310 3.40800 5.51313 3.90400 411 p - - -
2.68618 4.42225 2.77519 2.73123 3.46354 2.40513 3.72494
3.29354 2.67741 2.69355 4.24690 2.90347 2.73739 3.18146 2.89801
2.37887 2.77519 2.98518 4.58477 3.61503
0.05841 4.42422 3.10661 0.61958 0.77255 0.98624 0.46677
284 2.47873 4.59336 2.94089 2.42059 3.98029 2.43873 3.43972
3.66306 2.41947 3.05446 4.02999 2.93186 3.71941 2.68059 2.74905
2.40398 2.52100 3.10005 5.45154 4.09585 412 s - - -
2.68643 4.42226 2.77544 2.73143 3.46356 2.40532 3.72510
3.29280 2.67749 2.69323 4.24637 2.90366 2.73760 3.18152 2.89696
2.37881 2.77528 2.98529 4.58501 3.61528
0.73402 0.66493 5.16432 0.72115 0.66590 1.10781 0.40090
285 2.55385 4.25539 2.95695 2.43918 3.97912 1.97662 3.75272
3.26128 2.58819 2.60357 3.74622 3.02152 3.77197 2.93111 3.03588
2.55681 2.91042 3.03018 5.29465 3.98544 416 g - - -
2.68618 4.42225 2.77519 2.73123 3.46354 2.40513 3.72494
3.29354 2.67741 2.69355 4.24690 2.90347 2.73739 3.18146 2.89801
2.37887 2.77519 2.98518 4.58477 3.61503
0.01764 4.44197 5.16432 0.61958 0.77255 0.48499 0.95634
286 1.51492 4.91572 3.14182 2.55459 4.02946 2.89247 3.62280
3.54645 2.64418 2.95763 3.99196 3.16536 3.50161 2.89700 3.06062
2.57990 2.81761 3.01394 5.07688 4.10878 417 a - - -
2.68618 4.42225 2.77519 2.73123 3.46354 2.40513 3.72494
3.29354 2.67741 2.69355 4.24690 2.90347 2.73739 3.18146 2.89801
2.37887 2.77519 2.98518 4.58477 3.61503
0.01324 4.72672 5.44907 0.61958 0.77255 0.34118 1.24109

```
    287   2.48351  4.98011  3.31214  2.79873  4.29833  3.66975  3.42219
3.70934  2.78581  3.27912  4.15531  3.20527  1.28096  3.04558  3.18009
2.41636  3.12118  3.15673  5.58462  4.27009    418 p - - -
          2.68618  4.42225  2.77519  2.73123  3.46354  2.40513  3.72494
3.29354  2.67741  2.69355  4.24690  2.90347  2.73739  3.18146  2.89801
2.37887  2.77519  2.98518  4.58477  3.61503
          0.01146  4.87023  5.59257  0.61958  0.77255  0.48576  0.95510
    288   2.55383  5.18578  2.93874  2.32529  4.47260  3.32045  3.74835
3.91928  2.52846  2.98508  3.91383  3.12106  1.66161  2.88098  3.02212
2.41943  3.05991  3.41740  5.65543  4.28277    419 p - - -
          2.68618  4.42225  2.77519  2.73123  3.46354  2.40513  3.72494
3.29354  2.67741  2.69355  4.24690  2.90347  2.73739  3.18146  2.89801
2.37887  2.77519  2.98518  4.58477  3.61503
          0.01146  4.87023  5.59257  0.61958  0.77255  0.48576  0.95510
    289   2.37435  4.47927  5.06653  4.48404  3.47240  4.39979  4.78388
1.25762  4.31403  1.90003  3.19316  4.53540  4.73329  4.47316  4.37136
3.54670  3.38082  1.56183  5.27161  4.09231    420 i - - -
          2.68618  4.42225  2.77519  2.73123  3.46354  2.40513  3.72494
3.29354  2.67741  2.69355  4.24690  2.90347  2.73739  3.18146  2.89801
2.37887  2.77519  2.98518  4.58477  3.61503
          0.01146  4.87023  5.59257  0.61958  0.77255  0.48576  0.95510
    290   2.70004  4.43927  3.96956  3.39375  1.56735  3.88271  3.82680
2.91001  3.08232  2.26534  3.54877  3.72332  4.25709  3.03742  2.99290
3.14693  2.99939  2.60082  5.02769  2.59444    421 f - - -
          2.68618  4.42225  2.77519  2.73123  3.46354  2.40513  3.72494
3.29354  2.67741  2.69355  4.24690  2.90347  2.73739  3.18146  2.89801
2.37887  2.77519  2.98518  4.58477  3.61503
          0.01146  4.87023  5.59257  0.61958  0.77255  0.48576  0.95510
    291   2.35323  4.68192  3.31347  2.87480  3.84031  3.04451  3.99958
2.40955  2.82091  2.84199  3.63242  3.42372  4.14662  3.17521  3.03432
2.36197  2.65745  1.86581  5.24580  3.98679    422 v - - -
          2.68618  4.42225  2.77519  2.73123  3.46354  2.40513  3.72494
3.29354  2.67741  2.69355  4.24690  2.90347  2.73739  3.18146  2.89801
2.37887  2.77519  2.98518  4.58477  3.61503
          0.01146  4.87023  5.59257  0.61958  0.77255  0.48576  0.95510
    292   2.76545  4.41133  4.98055  4.38307  2.68967  4.27136  4.62541
1.52198  4.19609  1.52305  3.33785  4.41424  4.61121  4.33501  4.23223
3.59085  2.55555  1.92048  5.10569  3.93686    423 i - - -
          2.68618  4.42225  2.77519  2.73123  3.46354  2.40513  3.72494
3.29354  2.67741  2.69355  4.24690  2.90347  2.73739  3.18146  2.89801
2.37887  2.77519  2.98518  4.58477  3.61503
          0.01146  4.87023  5.59257  0.61958  0.77255  0.48576  0.95510
    293   2.75404  1.20759  4.53444  3.98181  3.63870  3.92920  4.49368
2.67408  3.84618  2.24288  3.63088  4.09818  4.40297  4.06321  4.00541
2.27996  3.18288  2.34648  5.17476  3.98705    424 c - - -
          2.68618  4.42225  2.77519  2.73123  3.46354  2.40513  3.72494
3.29354  2.67741  2.69355  4.24690  2.90347  2.73739  3.18146  2.89801
2.37887  2.77519  2.98518  4.58477  3.61503
          0.01146  4.87023  5.59257  0.61958  0.77255  0.48576  0.95510
    294   2.78436  5.31889  2.70785  2.07020  4.65740  3.25852  2.87351
3.57046  2.38999  3.42998  4.13890  1.94883  3.99339  2.75348  2.68995
2.64971  3.00870  3.31917  5.75549  4.12820    425 n - - -
          2.68618  4.42225  2.77519  2.73123  3.46354  2.40513  3.72494
3.29354  2.67741  2.69355  4.24690  2.90347  2.73739  3.18146  2.89801
2.37887  2.77519  2.98518  4.58477  3.61503
          0.01146  4.87023  5.59257  0.61958  0.77255  0.48576  0.95510
```

```
    295   2.50281  4.24197  2.03402  1.89423  4.65349  3.44779  3.57989
3.80772  2.42966  3.61812  4.15956  2.99444  3.99378  2.33652  2.78430
2.61934  2.98681  3.48311  5.75343  4.10585    426 e - - -
          2.68618  4.42225  2.77519  2.73123  3.46354  2.40513  3.72494
3.29354  2.67741  2.69355  4.24690  2.90347  2.73739  3.18146  2.89801
2.37887  2.77519  2.98518  4.58477  3.61503
          0.01146  4.87023  5.59257  0.61958  0.77255  0.48576  0.95510
    296   4.49854  5.73719  5.47021  5.23018  3.07450  4.97708  4.58259
4.10429  4.77728  2.75546  4.69974  5.05194  5.42183  5.04781  4.80110
4.67924  4.78598  4.12268  0.34367  3.06402    427 W - - -
          2.68618  4.42225  2.77519  2.73123  3.46354  2.40513  3.72494
3.29354  2.67741  2.69355  4.24690  2.90347  2.73739  3.18146  2.89801
2.37887  2.77519  2.98518  4.58477  3.61503
          0.01146  4.87023  5.59257  0.61958  0.77255  0.48576  0.95510
    297   2.69150  4.72000  3.24915  2.59863  3.41315  3.36141  2.59612
3.38454  2.45767  2.41201  4.06934  3.05007  4.05608  2.49520  3.14218
1.97087  2.99562  3.32263  5.50505  3.82218    428 s - - -
          2.68618  4.42225  2.77519  2.73123  3.46354  2.40513  3.72494
3.29354  2.67741  2.69355  4.24690  2.90347  2.73739  3.18146  2.89801
2.37887  2.77519  2.98518  4.58477  3.61503
          0.01146  4.87023  5.59257  0.61958  0.77255  0.48576  0.95510
    298   2.49730  5.32145  2.68285  2.22762  4.66051  3.50265  2.85309
4.14199  2.40748  2.62831  4.36382  3.00313  3.99454  2.11583  2.35467
2.69635  2.86424  3.70888  5.75749  4.35055    429 q - - -
          2.68618  4.42225  2.77519  2.73123  3.46354  2.40513  3.72494
3.29354  2.67741  2.69355  4.24690  2.90347  2.73739  3.18146  2.89801
2.37887  2.77519  2.98518  4.58477  3.61503
          0.01146  4.87023  5.59257  0.61958  0.77255  0.48576  0.95510
    299   1.57543  4.53764  3.48417  2.78328  3.72350  3.09374  3.69658
3.08233  2.75497  2.45713  3.61446  3.20276  4.13177  3.11089  3.05236
2.54633  2.98717  2.72616  4.98308  3.73513    430 a - - -
          2.68618  4.42225  2.77519  2.73123  3.46354  2.40513  3.72494
3.29354  2.67741  2.69355  4.24690  2.90347  2.73739  3.18146  2.89801
2.37887  2.77519  2.98518  4.58477  3.61503
          0.01146  4.87023  5.59257  0.61958  0.77255  0.48576  0.95510
    300   2.90788  4.44053  4.99849  4.39486  3.04499  4.28245  4.62077
1.90278  4.20352  1.14527  2.29951  4.17837  4.61300  4.32325  4.22933
3.60029  3.23874  2.16117  5.07907  3.92579    431 l - - -
          2.68618  4.42225  2.77519  2.73123  3.46354  2.40513  3.72494
3.29354  2.67741  2.69355  4.24690  2.90347  2.73739  3.18146  2.89801
2.37887  2.77519  2.98518  4.58477  3.61503
          0.01146  4.87023  5.59257  0.61958  0.77255  0.48576  0.95510
    301   2.77758  5.02875  1.66844  1.81461  4.70223  3.11250  3.80875
4.18790  2.32333  3.66503  4.40508  2.71902  4.00996  2.80093  3.00232
2.72364  2.99171  3.60203  5.79568  4.38259    432 d - - -
          2.68618  4.42225  2.77519  2.73123  3.46354  2.40513  3.72494
3.29354  2.67741  2.69355  4.24690  2.90347  2.73739  3.18146  2.89801
2.37887  2.77519  2.98518  4.58477  3.61503
          0.01146  4.87023  5.59257  0.61958  0.77255  0.48576  0.95510
    302   2.55794  5.27223  2.87608  2.48222  4.59184  3.25254  3.79814
3.88293  2.06486  3.45350  3.65589  2.75203  4.00120  2.87441  1.93786
2.20686  2.96920  3.40066  4.52386  4.32549    433 r - - -
          2.68618  4.42225  2.77520  2.73123  3.46354  2.40513  3.72495
3.29354  2.67741  2.69355  4.24690  2.90347  2.73740  3.18140  2.89801
2.37887  2.77520  2.98518  4.58477  3.61503
          0.01873  4.21076  5.59257  0.55108  0.85880  0.48576  0.95510
```

```
    303   2.75877  4.27693  4.29286  4.02943  3.17569  4.02265  4.35008
1.80264  3.85989  1.39935  3.39510  3.66615  4.38717  4.03148  3.94333
2.97618  3.05230  1.91249  4.91040  3.72846    435 l - - -
          2.68618  4.42225  2.77515  2.73124  3.46354  2.40513  3.72482
3.29354  2.67741  2.69355  4.24690  2.90347  2.73740  3.18147  2.89801
2.37887  2.77520  2.98519  4.58477  3.61504
          0.02904  3.69301  5.59257  0.47741  0.96860  0.48576  0.95510
    304   2.83658  4.79349  2.94734  2.56232  4.56953  3.31835  3.36089
4.03098  2.57017  3.55051  4.31237  2.94895  1.80457  2.67028  2.95862
1.86402  3.00154  3.63065  5.71552  4.32751    437 p - - -
          2.68618  4.42225  2.77519  2.73123  3.46354  2.40513  3.72494
3.29354  2.67741  2.69355  4.24690  2.90347  2.73739  3.18146  2.89801
2.37887  2.77519  2.98518  4.58477  3.61503
          0.01146  4.87023  5.59257  0.61958  0.77255  0.48576  0.95510
    305   2.64581  4.34876  2.01250  1.64195  4.62566  3.30785  3.79352
3.81567  2.41594  3.59511  4.34171  3.03553  3.99764  2.89531  2.90223
2.59292  2.70228  3.49430  5.73915  4.12549    438 e - - -
          2.68618  4.42225  2.77519  2.73123  3.46354  2.40513  3.72494
3.29354  2.67741  2.69355  4.24690  2.90347  2.73739  3.18146  2.89801
2.37887  2.77519  2.98518  4.58477  3.61503
          0.01146  4.87023  5.59257  0.61958  0.77255  0.48576  0.95510
    306   2.53266  5.32340  2.83889  1.94794  4.66362  3.60051  3.78775
3.93174  1.65453  3.62635  4.15621  2.91021  3.99390  2.71965  2.60757
2.75798  2.56128  3.41173  5.75875  4.35131    439 k - - -
          2.68618  4.42225  2.77519  2.73123  3.46354  2.40513  3.72494
3.29354  2.67741  2.69355  4.24690  2.90347  2.73739  3.18146  2.89801
2.37887  2.77519  2.98518  4.58477  3.61503
          0.01146  4.87023  5.59257  0.61958  0.77255  0.48576  0.95510
    307   2.54101  5.28753  2.44602  1.80626  4.61316  3.52605  3.72424
3.78190  2.22176  3.15583  4.11393  2.91302  3.39668  2.85486  2.87986
2.76061  2.99565  2.51936  5.73201  4.33310    440 e - - -
          2.68618  4.42225  2.77519  2.73123  3.46354  2.40513  3.72494
3.29354  2.67741  2.69355  4.24690  2.90347  2.73739  3.18146  2.89801
2.37887  2.77519  2.98518  4.58477  3.61503
          0.01146  4.87023  5.59257  0.61958  0.77255  0.48576  0.95510
    308   1.75937  4.41077  4.67725  4.09767  3.29018  4.16294  4.53663
2.45189  3.95717  2.16077  3.54149  4.23352  4.53236  4.15630  4.08794
3.33845  3.00660  1.17451  5.13574  3.94702    441 v - - -
          2.68618  4.42225  2.77519  2.73123  3.46354  2.40513  3.72494
3.29354  2.67741  2.69355  4.24690  2.90347  2.73739  3.18146  2.89801
2.37887  2.77519  2.98518  4.58477  3.61503
          0.01146  4.87023  5.59257  0.61958  0.77255  0.48576  0.95510
    309   2.54377  3.85440  4.00153  3.17051  3.46282  3.78521  4.16472
2.47576  2.89630  1.98537  3.40889  3.61775  4.25965  3.61366  3.41854
2.06070  2.62353  2.03076  5.01618  3.80757    442 l - - -
          2.68618  4.42225  2.77519  2.73123  3.46354  2.40513  3.72494
3.29354  2.67741  2.69355  4.24690  2.90347  2.73739  3.18146  2.89801
2.37887  2.77519  2.98518  4.58477  3.61503
          0.01146  4.87023  5.59257  0.61958  0.77255  0.48576  0.95510
    310   2.82447  4.56207  2.06438  1.70274  4.21905  3.48559  3.78803
4.14133  2.25261  3.62332  4.36298  2.76508  3.99358  2.85817  2.83221
2.45310  2.82293  3.57552  5.75699  3.99488    443 e - - -
          2.68618  4.42225  2.77519  2.73123  3.46354  2.40513  3.72494
3.29354  2.67741  2.69355  4.24690  2.90347  2.73739  3.18146  2.89801
2.37887  2.77519  2.98518  4.58477  3.61503
          0.01146  4.87023  5.59257  0.61958  0.77255  0.48576  0.95510
```

```
     311   1.31317  4.66075  3.37727  2.83123  4.38065  2.59787  4.00348
3.79957  2.67074  3.40843  4.21939  3.30931  4.09068  3.16295  2.76021
2.10413  3.00280  3.30279  5.64894  4.33052    444 a - - -
         2.68618  4.42225  2.77519  2.73123  3.46354  2.40513  3.72494
3.29354  2.67741  2.69355  4.24690  2.90347  2.73739  3.18146  2.89801
2.37887  2.77519  2.98518  4.58477  3.61503
         0.01146  4.87023  5.59257  0.61958  0.77255  0.48576  0.95510
     312   3.74727  4.97972  5.75193  5.20361  3.73017  5.18348  5.61949
1.01256  5.08006  1.70648  1.76854  5.33767  5.34847  5.11861  5.10737
4.56577  3.97821  1.93969  5.81761  4.75504    445 i - - -
         2.68618  4.42225  2.77519  2.73123  3.46354  2.40513  3.72494
3.29354  2.67741  2.69355  4.24690  2.90347  2.73739  3.18146  2.89801
2.37887  2.77519  2.98518  4.58477  3.61503
         0.01146  4.87023  5.59257  0.61958  0.77255  0.48576  0.95510
     313   2.79991  5.33317  2.54827  2.25407  4.67754  3.55386  3.38388
4.07964  1.60573  3.49739  4.19372  2.85173  3.99272  2.49894  2.40507
2.63109  3.05661  3.64746  5.76591  3.93984    446 k - - -
         2.68618  4.42225  2.77519  2.73123  3.46354  2.40513  3.72494
3.29354  2.67741  2.69355  4.24690  2.90347  2.73739  3.18146  2.89801
2.37887  2.77519  2.98518  4.58477  3.61503
         0.01146  4.87023  5.59257  0.61958  0.77255  0.48576  0.95510
     314   2.16109  5.23748  2.88927  2.35719  3.42160  2.82703  3.38895
3.92293  2.55815  3.52608  3.97745  2.70070  4.00700  2.80712  2.97661
1.89485  2.77197  3.44793  5.69415  4.30789    447 s - - -
         2.68618  4.42225  2.77519  2.73123  3.46354  2.40513  3.72494
3.29354  2.67741  2.69355  4.24690  2.90347  2.73739  3.18146  2.89801
2.37887  2.77519  2.98518  4.58477  3.61503
         0.01146  4.87023  5.59257  0.61958  0.77255  0.48576  0.95510
     315   3.09385  4.13251  4.93187  4.33317  1.12761  4.27103  4.57786
2.51187  4.15155  1.64896  3.09877  4.38947  4.60294  4.27750  4.19718
3.26270  3.32434  2.49271  5.03872  3.85198    448 f - - -
         2.68618  4.42225  2.77519  2.73123  3.46354  2.40513  3.72494
3.29354  2.67741  2.69355  4.24690  2.90347  2.73739  3.18146  2.89801
2.37887  2.77519  2.98518  4.58477  3.61503
         0.01146  4.87023  5.59257  0.61958  0.77255  0.48576  0.95510
     316   1.75923  4.35077  4.19184  3.30047  3.29027  3.46989  4.21882
2.45869  3.44594  2.06252  3.16444  3.86046  4.29543  3.74426  3.47964
2.63734  2.89763  1.91779  4.95872  3.76145    449 a - - -
         2.68618  4.42225  2.77519  2.73123  3.46354  2.40513  3.72494
3.29354  2.67741  2.69355  4.24690  2.90347  2.73739  3.18146  2.89801
2.37887  2.77519  2.98518  4.58477  3.61503
         0.01146  4.87023  5.59257  0.61958  0.77255  0.48576  0.95510
     317   2.00200  4.90775  3.07402  2.24857  4.53445  3.31076  3.67550
3.76243  2.21985  3.09608  3.68581  2.78376  4.00810  2.88186  2.84388
2.24457  2.50269  3.49545  5.68890  4.18061    450 a - - -
         2.68618  4.42225  2.77519  2.73123  3.46354  2.40513  3.72494
3.29354  2.67741  2.69355  4.24690  2.90347  2.73739  3.18146  2.89801
2.37887  2.77519  2.98518  4.58477  3.61503
         0.02712  4.87023  3.95875  0.61958  0.77255  0.48576  0.95510
     318   2.38152  4.69070  2.51397  2.48540  4.36964  2.91819  3.68515
2.97942  2.48410  3.11077  4.16961  2.86558  4.02448  2.90071  2.87439
2.46208  2.92601  2.25549  5.59142  4.23607    451 v - - -
         2.68618  4.42225  2.77519  2.73123  3.46354  2.40513  3.72494
3.29354  2.67741  2.69355  4.24690  2.90347  2.73739  3.18146  2.89801
2.37887  2.77519  2.98518  4.58477  3.61503
         0.02471  4.85475  4.09761  0.61958  0.77255  0.52310  0.89815
```

```
    319   3.18546  4.59237  5.27375  4.70819  3.34626  4.61984  5.04381
1.28296  4.55224  2.09087  2.96506  4.76664  4.92480  4.70748  4.61006
3.96355  3.08462  1.19435  5.48525  4.31198    452 v - - -
          2.68618  4.42225  2.77519  2.73123  3.46354  2.40513  3.72494
3.29354  2.67741  2.69355  4.24690  2.90347  2.73739  3.18146  2.89801
2.37887  2.77519  2.98518  4.58477  3.61503
          0.04836  4.84183  3.23614  0.61958  0.77255  0.55279  0.85647
    320   2.65358  5.28631  3.02759  2.43653  4.61961  2.97348  1.99087
4.09428  2.34635  3.23336  4.33102  2.71211  3.98059  2.64727  2.22742
2.64865  3.00184  3.67082  5.72408  4.32478    453 h - - -
          2.68618  4.42225  2.77519  2.73123  3.46354  2.40513  3.72494
3.29354  2.67741  2.69355  4.24690  2.90347  2.73739  3.18146  2.89801
2.37887  2.77519  2.98518  4.58477  3.61503
          0.11475  4.80571  2.30033  0.61958  0.77255  0.62964  0.76096
    321   2.20105  5.17073  3.05641  2.22533  4.47487  3.39247  2.93148
3.93276  2.37170  3.45829  3.93332  2.93723  3.94748  2.67298  2.73546
2.25970  2.58854  2.77168  5.62866  4.24416    454 a - - -
          2.68618  4.42225  2.77519  2.73123  3.46354  2.40513  3.72494
3.29354  2.67741  2.69355  4.24690  2.90347  2.73739  3.18146  2.89801
2.37887  2.77519  2.98518  4.58477  3.61503
          0.00914  4.70010        *  0.61958  0.77255  0.00000        *
//
HMMER3/f [3.3 | Nov 2019]
NAME  DUF702
ACC   PF05142.13
DESC  Domain of unknown function (DUF702)
LENG  151
ALPH  amino
RF    no
MM    no
CONS  yes
CS    no
MAP   yes
DATE  Fri Apr 17 07:19:20 2020
NSEQ  53
EFFN  0.934875
CKSUM 2638512836
GA    20.50 20.50
TC    20.60 20.50
NC    20.40 20.40
STATS LOCAL MSV      -10.1407  0.70932
STATS LOCAL VITERBI  -11.1841  0.70932
STATS LOCAL FORWARD   -3.9256  0.70932
HMM          A        C        D        E        F        G        H
I        K        L        M        N        P        Q        R        S
T        V        W        Y
            m->m     m->i     m->d     i->m     i->i     d->m     d->d
  COMPO   2.46598  3.29251  2.99745  2.68984  3.49601  2.84120  3.50160
3.11474  2.52466  2.81576  3.89069  3.10455  3.45731  2.93963  2.69419
2.55579  2.75735  2.75201  4.83684  3.61731
          2.68618  4.42225  2.77519  2.73123  3.46354  2.40513  3.72494
3.29354  2.67741  2.69355  4.24690  2.90347  2.73739  3.18146  2.89801
2.37887  2.77519  2.98518  4.58477  3.61503
          0.11643  4.15374  2.36230  0.61958  0.77255  0.00000        *
      1   2.42500  4.49617  3.17813  2.56680  3.82383  2.01682  3.76578
3.08857  2.63616  2.76457  3.63379  3.11104  3.83993  2.98423  3.04143
2.56106  2.64880  2.82786  5.18200  3.90587      1 g - - -
```

```
          2.68618  4.42225  2.77519  2.73123  3.46354  2.40513  3.72494
3.29354  2.67741  2.69355  4.24690  2.90347  2.73739  3.18146  2.89801
2.37887  2.77519  2.98518  4.58477  3.61503
          0.02588  4.06319  4.78553  0.61958  0.77255  0.50267  0.92865
      2   2.51155  4.60777  3.06231  2.46724  4.00374  2.15406  3.75314
3.22542  2.59823  3.04357  3.87660  2.94482  3.82166  2.93850  3.03148
2.29733  2.42529  3.00876  5.31336  4.01167      2 g - - -
          2.68618  4.42225  2.77519  2.73123  3.46354  2.40513  3.72494
3.29354  2.67741  2.69355  4.24690  2.90347  2.73739  3.18146  2.89801
2.37887  2.77519  2.98518  4.58477  3.61503
          0.02468  4.11007  4.83242  0.61958  0.77255  0.48363  0.95852
      3   2.40973  4.29962  3.47789  2.90973  3.43975  3.56586  3.82367
2.37907  2.75575  2.51172  2.88219  3.31069  3.78229  3.16026  2.82206
2.61805  2.58046  2.38143  4.88658  3.65410      3 i - - -
          2.68618  4.42225  2.77519  2.73123  3.46354  2.40513  3.72494
3.29354  2.67741  2.69355  4.24690  2.90347  2.73739  3.18146  2.89801
2.37887  2.77519  2.98518  4.58477  3.61503
          0.02398  4.13832  4.86067  0.61958  0.77255  0.47446  0.97344
      4   2.45957  4.68408  3.09260  2.55413  4.03029  3.39222  3.71177
3.43371  2.51079  3.05811  3.88337  2.76815  3.83471  2.88090  2.45060
2.03265  2.48468  2.79565  5.31389  4.00485      4 s - - -
          2.68618  4.42225  2.77519  2.73123  3.46354  2.40513  3.72494
3.29354  2.67741  2.69355  4.24690  2.90347  2.73739  3.18146  2.89801
2.37887  2.77519  2.98518  4.58477  3.61503
          0.02361  4.15374  4.87609  0.61958  0.77255  0.48576  0.95510
      5   2.95080  0.51307  4.52067  4.33116  4.26996  3.50590  4.91763
3.47094  4.16388  3.36857  4.50213  4.24499  4.23359  4.49865  4.23484
3.22163  3.44180  3.19375  5.61467  4.55296      5 C - - -
          2.68618  4.42225  2.77519  2.73123  3.46354  2.40513  3.72494
3.29354  2.67741  2.69355  4.24690  2.90347  2.73739  3.18146  2.89801
2.37887  2.77519  2.98518  4.58477  3.61503
          0.02361  4.15374  4.87609  0.61958  0.77255  0.48576  0.95510
      6   2.84499  5.13332  3.01833  2.38762  4.47688  3.52807  3.55498
3.88808  2.06241  3.38824  4.20412  3.02176  3.93303  1.55418  2.38415
2.82799  3.05481  3.41830  5.51520  4.21959      6 q - - -
          2.68618  4.42225  2.77519  2.73123  3.46354  2.40513  3.72494
3.29354  2.67741  2.69355  4.24690  2.90347  2.73739  3.18146  2.89801
2.37887  2.77519  2.98518  4.58477  3.61503
          0.02361  4.15374  4.87609  0.61958  0.77255  0.48576  0.95510
      7   3.11657  5.69854  0.95327  1.89065  4.98745  3.29840  3.90728
4.52670  2.99254  4.03055  4.91839  2.76870  3.95531  3.08817  3.62051
2.98130  3.42187  4.09532  6.16794  4.64586      7 d - - -
          2.68618  4.42225  2.77519  2.73123  3.46354  2.40513  3.72494
3.29354  2.67741  2.69355  4.24690  2.90347  2.73739  3.18146  2.89801
2.37887  2.77519  2.98518  4.58477  3.61503
          0.02361  4.15374  4.87609  0.61958  0.77255  0.48576  0.95510
      8   2.95080  0.51307  4.52067  4.33116  4.26996  3.50590  4.91763
3.47094  4.16388  3.36857  4.50213  4.24499  4.23359  4.49865  4.23484
3.22163  3.44180  3.19375  5.61467  4.55296      8 C - - -
          2.68618  4.42225  2.77519  2.73123  3.46354  2.40513  3.72494
3.29354  2.67741  2.69355  4.24690  2.90347  2.73739  3.18146  2.89801
2.37887  2.77519  2.98518  4.58477  3.61503
          0.02361  4.15374  4.87609  0.61958  0.77255  0.48576  0.95510
      9   2.69788  4.59662  3.36666  2.92888  3.82034  1.24111  3.92871
3.44355  2.75053  3.07809  3.97459  3.31567  3.96991  3.20878  2.81784
2.83339  3.01471  3.13617  4.15476  3.86628      9 g - - -
```

```
          2.68618  4.42225  2.77519  2.73123  3.46354  2.40513  3.72494
3.29354  2.67741  2.69355  4.24690  2.90347  2.73739  3.18146  2.89801
2.37887  2.77519  2.98518  4.58477  3.61503
          0.02361  4.15374  4.87609  0.61958  0.77255  0.48576  0.95510
     10   2.84493  4.99371  2.78777  2.53450  3.92244  3.42983  3.37357
3.79425  2.58968  3.13120  4.24600  1.29814  3.95754  3.01510  2.96603
2.86057  3.12535  3.45847  5.29457  3.82191     10 n - - -
          2.68618  4.42225  2.77520  2.73124  3.46354  2.40513  3.72495
3.29354  2.67741  2.69355  4.24690  2.90347  2.73740  3.18147  2.89793
2.37887  2.77520  2.98519  4.58477  3.61503
          0.04925  3.20815  4.87609  0.51240  0.91393  0.48576  0.95510
     11   2.86299  5.09902  3.13722  2.59055  4.44015  3.56216  3.65157
3.85103  2.11175  3.17095  4.18273  2.87602  3.95563  1.58697  2.12212
2.85829  3.06867  3.50810  5.47749  4.20088     12 q - - -
          2.68618  4.42225  2.77519  2.73123  3.46354  2.40513  3.72494
3.29354  2.67741  2.69355  4.24690  2.90347  2.73739  3.18146  2.89801
2.37887  2.77519  2.98518  4.58477  3.61503
          0.02361  4.15374  4.87609  0.61958  0.77255  0.48576  0.95510
     12   0.77774  4.25890  3.67017  3.47207  4.33156  3.07190  4.47908
3.53498  3.50039  3.40709  4.34862  3.53510  3.84821  3.80979  3.74237
2.50651  2.87427  3.09129  5.75328  4.53619     13 a - - -
          2.68618  4.42225  2.77519  2.73123  3.46354  2.40513  3.72494
3.29354  2.67741  2.69355  4.24690  2.90347  2.73739  3.18146  2.89801
2.37887  2.77519  2.98518  4.58477  3.61503
          0.02361  4.15374  4.87609  0.61958  0.77255  0.48576  0.95510
     13   3.25424  5.20763  3.44752  3.02803  4.65946  3.70196  3.94962
4.10925  0.70826  3.61725  4.57248  3.43648  4.18977  3.13154  2.52931
3.28311  3.49084  3.80448  5.60456  4.46281     14 k - - -
          2.68618  4.42225  2.77519  2.73123  3.46354  2.40513  3.72494
3.29354  2.67741  2.69355  4.24690  2.90347  2.73739  3.18146  2.89801
2.37887  2.77519  2.98518  4.58477  3.61503
          0.02361  4.15374  4.87609  0.61958  0.77255  0.48576  0.95510
     14   2.62806  5.08232  3.26407  2.73937  4.54295  3.59110  3.71543
3.89494  1.10945  3.41314  4.27491  3.17622  4.02126  2.85884  2.27778
2.96254  3.16847  3.55961  5.52684  4.30234     15 k - - -
          2.68618  4.42225  2.77519  2.73123  3.46354  2.40513  3.72494
3.29354  2.67741  2.69355  4.24690  2.90347  2.73739  3.18146  2.89801
2.37887  2.77519  2.98518  4.58477  3.61503
          0.02361  4.15374  4.87609  0.61958  0.77255  0.48576  0.95510
     15   2.87277  5.36827  1.38697  1.94316  4.64697  2.81519  3.75551
4.14434  2.67569  3.65855  4.47150  2.79324  3.87747  2.90356  3.22565
2.79619  3.13763  3.73674  5.82657  3.76203     16 d - - -
          2.68618  4.42225  2.77519  2.73123  3.46354  2.40513  3.72494
3.29354  2.67741  2.69355  4.24690  2.90347  2.73739  3.18146  2.89801
2.37887  2.77519  2.98518  4.58477  3.61503
          0.02361  4.15374  4.87609  0.61958  0.77255  0.48576  0.95510
     16   2.95080  0.51307  4.52067  4.33116  4.26996  3.50590  4.91763
3.47094  4.16388  3.36857  4.50213  4.24499  4.23359  4.49865  4.23484
3.22163  3.44180  3.19375  5.61467  4.55296     17 C - - -
          2.68618  4.42225  2.77519  2.73123  3.46354  2.40513  3.72494
3.29354  2.67741  2.69355  4.24690  2.90347  2.73739  3.18146  2.89801
2.37887  2.77519  2.98518  4.58477  3.61503
          0.02361  4.15374  4.87609  0.61958  0.77255  0.48576  0.95510
     17   2.36318  4.54786  3.18644  2.59738  3.73832  3.48383  3.72023
2.98963  2.57006  2.66727  3.64778  3.10510  3.14729  2.76392  3.02477
2.41482  2.48844  2.54130  5.10535  3.82537     18 a - - -
```

```
          2.68618  4.42225  2.77519  2.73123  3.46354  2.40513  3.72494
3.29354  2.67741  2.69355  4.24690  2.90347  2.73739  3.18146  2.89801
2.37887  2.77519  2.98518  4.58477  3.61503
          0.02361  4.15374  4.87609  0.61958  0.77255  0.48576  0.95510
     18   3.20044  4.78875  3.91870  3.44405  1.94168  4.02827  1.54684
3.35417  3.34448  2.84457  3.91303  3.66895  4.38283  3.41904  3.61995
3.31846  3.42518  3.17190  3.95805  1.90186     19 h - - -
          2.68618  4.42225  2.77519  2.73123  3.46354  2.40513  3.72494
3.29354  2.67741  2.69355  4.24690  2.90347  2.73739  3.18146  2.89801
2.37887  2.77519  2.98518  4.58477  3.61503
          0.04925  4.15374  3.43106  0.61958  0.77255  0.48576  0.95510
     19   2.62320  4.48715  3.30595  2.64435  3.65322  3.37001  3.66175
2.97164  2.59312  2.39700  2.43266  3.18136  3.91459  2.83362  2.45771
2.77260  2.85061  2.77845  5.03503  3.78009     20 l - - -
          2.68618  4.42225  2.77519  2.73123  3.46354  2.40513  3.72494
3.29354  2.67741  2.69355  4.24690  2.90347  2.73739  3.18146  2.89801
2.37887  2.77519  2.98518  4.58477  3.61503
          0.02421  4.12871  4.85105  0.61958  0.77255  0.51524  0.90971
     20   3.04818  4.99699  3.67758  3.03570  4.25331  3.69256  3.79538
3.69105  2.12902  3.20258  3.88826  3.38952  4.12291  2.97653  0.99256
3.11752  3.27562  3.44018  5.38801  4.15884     21 r - - -
          2.68618  4.42225  2.77519  2.73123  3.46354  2.40513  3.72494
3.29354  2.67741  2.69355  4.24690  2.90347  2.73739  3.18146  2.89801
2.37887  2.77519  2.98518  4.58477  3.61503
          0.02421  4.12871  4.85105  0.61958  0.77255  0.46776  0.98457
     21   2.95080  0.51307  4.52067  4.33116  4.26996  3.50590  4.91763
3.47094  4.16388  3.36857  4.50213  4.24499  4.23359  4.49865  4.23484
3.22163  3.44180  3.19375  5.61467  4.55296     22 C - - -
          2.68618  4.42225  2.77519  2.73123  3.46354  2.40513  3.72494
3.29354  2.67741  2.69355  4.24690  2.90347  2.73739  3.18146  2.89801
2.37887  2.77519  2.98518  4.58477  3.61503
          0.02361  4.15374  4.87609  0.61958  0.77255  0.48576  0.95510
     22   3.35187  5.15246  3.87394  3.33738  4.58016  3.77324  4.06898
4.14982  2.40540  3.61470  4.61546  3.67131  4.26934  3.28229  0.62255
3.42748  3.60321  3.86349  5.55312  4.45522     23 R - - -
          2.68618  4.42225  2.77519  2.73123  3.46354  2.40513  3.72494
3.29354  2.67741  2.69355  4.24690  2.90347  2.73739  3.18146  2.89801
2.37887  2.77519  2.98518  4.58477  3.61503
          0.02361  4.15374  4.87609  0.61958  0.77255  0.48576  0.95510
     23   2.36634  4.27908  3.54383  3.27624  4.26899  3.08877  4.31290
3.56249  3.26496  3.35814  4.24825  3.43144  3.82847  3.60731  3.54457
2.25195  1.01638  3.11417  5.65701  4.42708     24 t - - -
          2.68618  4.42225  2.77519  2.73123  3.46354  2.40513  3.72494
3.29354  2.67741  2.69355  4.24690  2.90347  2.73739  3.18146  2.89801
2.37887  2.77519  2.98518  4.58477  3.61503
          0.02361  4.15374  4.87609  0.61958  0.77255  0.48576  0.95510
     24   2.95080  0.51307  4.52067  4.33116  4.26996  3.50590  4.91763
3.47094  4.16388  3.36857  4.50213  4.24499  4.23359  4.49865  4.23484
3.22163  3.44180  3.19375  5.61467  4.55296     25 C - - -
          2.68618  4.42225  2.77519  2.73123  3.46354  2.40513  3.72494
3.29354  2.67741  2.69355  4.24690  2.90347  2.73739  3.18146  2.89801
2.37887  2.77519  2.98518  4.58477  3.61503
          0.04925  4.15374  3.43106  0.61958  0.77255  0.48576  0.95510
     25   2.91432  0.54160  4.48126  4.28488  4.22706  3.47624  4.87659
3.42180  4.11656  3.32160  4.45384  4.20490  4.20229  4.45238  4.19263
3.18535  3.40316  3.14686  5.57940  4.51074     26 C - - -
```

```
        2.68618  4.42225  2.77519  2.73123  3.46354  2.40513  3.72494
3.29354  2.67741  2.69355  4.24690  2.90347  2.73739  3.18146  2.89801
2.37887  2.77519  2.98518  4.58477  3.61503
        0.02421  4.12871  4.85105  0.61958  0.77255  0.51524  0.90971
     26  2.97719  5.15238  3.21479  2.69946  4.60430  3.32398  3.68352
3.98556  1.11128  3.46045  4.30842  3.05190  4.01722  2.82135  2.19699
2.97346  3.18345  3.63957  5.53501  4.31121      27 k - - -
        2.68618  4.42225  2.77519  2.73123  3.46354  2.40513  3.72494
3.29354  2.67741  2.69355  4.24690  2.90347  2.73739  3.18146  2.89801
2.37887  2.77519  2.98518  4.58477  3.61503
        0.02421  4.12871  4.85105  0.61958  0.77255  0.51524  0.90971
     27  2.10443  4.25170  3.42066  3.18856  4.47310  2.62756  4.31550
3.90464  3.30262  3.58908  4.40570  3.35190  3.75764  3.58208  3.61771
0.98859  2.79211  3.31142  5.80034  4.58251      28 s - - -
        2.68618  4.42225  2.77519  2.73123  3.46354  2.40513  3.72494
3.29354  2.67741  2.69355  4.24690  2.90347  2.73739  3.18146  2.89801
2.37887  2.77519  2.98518  4.58477  3.61503
        0.02421  4.12871  4.85105  0.61958  0.77255  0.46776  0.98457
     28  3.13681  5.24017  3.51259  2.86343  4.66578  3.74556  3.51036
4.03055  1.84408  3.47067  4.33959  3.16179  4.11114  2.74378  1.09016
3.13102  3.29668  3.71667  5.50603  4.33444      29 r - - -
        2.68618  4.42225  2.77519  2.73123  3.46354  2.40513  3.72494
3.29354  2.67741  2.69355  4.24690  2.90347  2.73739  3.18146  2.89801
2.37887  2.77519  2.98518  4.58477  3.61503
        0.02361  4.15374  4.87609  0.61958  0.77255  0.48576  0.95510
     29  2.32132  4.57840  2.72574  2.70801  4.50582  1.15480  4.09745
3.95292  3.07653  3.59835  4.43510  2.89943  3.83749  3.32051  3.49781
2.63614  2.96583  3.44939  5.80429  4.49525      30 g - - -
        2.68618  4.42225  2.77519  2.73123  3.46354  2.40513  3.72494
3.29354  2.67741  2.69355  4.24690  2.90347  2.73739  3.18146  2.89801
2.37887  2.77519  2.98518  4.58477  3.61503
        0.02361  4.15374  4.87609  0.61958  0.77255  0.48576  0.95510
     30  2.89198  4.55106  4.41479  3.93746  1.26797  4.13493  3.76321
2.87610  3.79965  2.17099  3.52995  3.97384  4.46991  3.92588  3.93136
3.45421  3.38620  2.77149  4.03688  1.93514      31 f - - -
        2.68618  4.42225  2.77519  2.73123  3.46354  2.40513  3.72494
3.29354  2.67741  2.69355  4.24690  2.90347  2.73739  3.18146  2.89801
2.37887  2.77519  2.98518  4.58477  3.61503
        0.02361  4.15374  4.87609  0.61958  0.77255  0.48576  0.95510
     31  2.62646  5.09329  2.17068  2.16985  4.40303  3.38039  2.93293
3.87059  2.38029  3.34446  4.14818  2.76145  3.73534  2.44286  2.87248
2.52479  2.88690  3.14281  5.54404  4.14490      32 e - - -
        2.68618  4.42225  2.77519  2.73123  3.46354  2.40513  3.72494
3.29354  2.67741  2.69355  4.24690  2.90347  2.73739  3.18146  2.89801
2.37887  2.77519  2.98518  4.58477  3.61503
        0.02361  4.15374  4.87609  0.61958  0.77255  0.48576  0.95510
     32  2.95080  0.51307  4.52067  4.33116  4.26996  3.50590  4.91763
3.47094  4.16388  3.36857  4.50213  4.24499  4.23359  4.49865  4.23484
3.22163  3.44180  3.19375  5.61467  4.55296      33 C - - -
        2.68618  4.42225  2.77519  2.73123  3.46354  2.40513  3.72494
3.29354  2.67741  2.69355  4.24690  2.90347  2.73739  3.18146  2.89801
2.37887  2.77519  2.98518  4.58477  3.61503
        0.02361  4.15374  4.87609  0.61958  0.77255  0.48576  0.95510
     33  2.29494  4.98616  2.77133  2.25676  4.29347  3.38576  3.63998
3.73943  2.42368  3.29062  4.07811  2.79506  2.71083  2.35387  2.90283
2.36442  2.84293  3.36475  5.48434  4.11067      34 e - - -
```

```
         2.68618  4.42225  2.77519  2.73123  3.46354  2.40513  3.72494
3.29354  2.67741  2.69355  4.24690  2.90347  2.73739  3.18146  2.89801
2.37887  2.77519  2.98518  4.58477  3.61503
         0.02361  4.15374  4.87609  0.61958  0.77255  0.48576  0.95510
     34  2.23210  4.26429  3.55000  3.28081  4.30837  2.78574  4.32601
3.57071  3.29451  3.37300  4.24976  3.43034  3.81843  3.61652  3.57956
2.54079  1.01320  3.11295  5.68476  4.47853      35 t - - -
         2.68618  4.42225  2.77519  2.73123  3.46354  2.40513  3.72494
3.29354  2.67741  2.69355  4.24690  2.90347  2.73739  3.18146  2.89801
2.37887  2.77519  2.98518  4.58477  3.61503
         0.02361  4.15374  4.87609  0.61958  0.77255  0.48576  0.95510
     35  3.35455  5.10491  3.44950  3.24666  3.52205  3.71961  0.66629
4.10203  3.08393  3.55586  4.62826  3.64086  4.29195  3.61922  3.32794
3.42549  3.67138  3.83208  4.98080  3.46686      36 H - - -
         2.68618  4.42225  2.77519  2.73123  3.46354  2.40513  3.72494
3.29354  2.67741  2.69355  4.24690  2.90347  2.73739  3.18146  2.89801
2.37887  2.77519  2.98518  4.58477  3.61503
         0.02361  4.15374  4.87609  0.61958  0.77255  0.48576  0.95510
     36  3.15641  4.48186  4.85602  4.36947  3.65205  4.48431  5.08967
1.67018  4.23963  2.13194  3.47128  4.58144  4.81784  4.52593  4.44177
3.88058  3.43069  0.86747  5.61762  4.38980      37 v - - -
         2.68618  4.42225  2.77519  2.73123  3.46354  2.40513  3.72494
3.29354  2.67741  2.69355  4.24690  2.90347  2.73739  3.18146  2.89801
2.37887  2.77519  2.98518  4.58477  3.61503
         0.02361  4.15374  4.87609  0.61958  0.77255  0.48576  0.95510
     37  3.17019  5.27669  3.57818  2.90195  4.76040  3.75993  3.68869
4.10254  1.01693  3.52257  4.39867  3.29353  4.12788  2.82228  1.81331
3.15982  3.32996  3.78267  5.54073  4.38912      38 k - - -
         2.68619  4.42226  2.77520  2.73124  3.46354  2.40513  3.72495
3.29355  2.67741  2.69355  4.24690  2.90347  2.73735  3.18147  2.89801
2.37887  2.77513  2.98519  4.58477  3.61504
         0.06955  2.82082  4.87609  0.45231  1.01104  0.48576  0.95510
     38  2.34808  4.29257  3.46139  3.24434  4.40879  3.04351  4.34268
3.81521  3.30259  3.54624  4.41130  3.40159  3.80618  3.62554  3.59188
0.88659  2.59928  3.27884  5.76884  4.52361      40 s - - -
         2.68618  4.42225  2.77519  2.73123  3.46354  2.40513  3.72494
3.29354  2.67741  2.69355  4.24690  2.90347  2.73739  3.18146  2.89801
2.37887  2.77519  2.98518  4.58477  3.61503
         0.02361  4.15374  4.87609  0.61958  0.77255  0.48576  0.95510
     39  2.23075  4.25583  3.57914  3.29925  4.27679  3.07953  4.32517
3.51045  3.28643  3.33632  4.22417  3.43924  3.82083  3.61851  3.56577
2.36342  1.02654  3.06908  5.66812  4.45419      41 t - - -
         2.68618  4.42225  2.77519  2.73123  3.46354  2.40513  3.72494
3.29354  2.67741  2.69355  4.24690  2.90347  2.73739  3.18146  2.89801
2.37887  2.77519  2.98518  4.58477  3.61503
         0.02361  4.15374  4.87609  0.61958  0.77255  0.48576  0.95510
     40  3.42849  4.91143  4.13019  3.74440  2.37716  4.09318  2.88833
3.54715  3.38556  2.96924  4.10746  3.86984  4.50258  3.78880  3.56126
3.54770  3.67236  3.38668  1.03270  2.32476      42 w - - -
         2.68618  4.42225  2.77519  2.73123  3.46354  2.40513  3.72494
3.29354  2.67741  2.69355  4.24690  2.90347  2.73739  3.18146  2.89801
2.37887  2.77519  2.98518  4.58477  3.61503
         0.02361  4.15374  4.87609  0.61958  0.77255  0.48576  0.95510
     41  3.14358  4.47799  4.84693  4.37654  3.67690  4.44994  5.10524
1.64730  4.25145  2.19647  3.49573  4.57661  4.80659  4.54073  4.45250
3.85757  3.42612  0.85895  5.64109  4.41088      43 v - - -
```

```
          2.68618  4.42225  2.77519  2.73123  3.46354  2.40513  3.72494
3.29354  2.67741  2.69355  4.24690  2.90347  2.73739  3.18146  2.89801
2.37887  2.77519  2.98518  4.58477  3.61503
          0.02361  4.15374  4.87609  0.61958  0.77255  0.48576  0.95510
     42   2.49272  4.43624  3.29389  3.11946  4.38032  3.13894  4.28780
3.87732  3.22182  3.55890  4.44997  3.37524  1.00131  3.57114  3.52258
2.36197  2.96857  3.37955  5.71385  4.45370     44 p - - -
          2.68618  4.42225  2.77519  2.73123  3.46354  2.40513  3.72494
3.29354  2.67741  2.69355  4.24690  2.90347  2.73739  3.18146  2.89801
2.37887  2.77519  2.98518  4.58477  3.61503
          0.02361  4.15374  4.87609  0.61958  0.77255  0.48576  0.95510
     43   1.24288  4.25936  3.72044  3.25595  3.85359  3.28976  4.17994
2.75940  3.19103  2.85194  3.79388  3.48128  3.91386  3.50089  3.50010
2.44852  2.84231  2.31433  5.32336  4.10047     45 a - - -
          2.68618  4.42225  2.77520  2.73123  3.46354  2.40513  3.72495
3.29354  2.67741  2.69355  4.24690  2.90347  2.73740  3.18147  2.89801
2.37883  2.77520  2.98519  4.58477  3.61503
          0.04339  3.35725  4.87609  0.53322  0.88362  0.48576  0.95510
     44   1.46860  4.36862  3.39959  2.92713  3.82134  3.30498  3.95421
3.19629  2.90034  2.91614  3.79442  3.27761  3.86618  3.22718  3.26458
2.08670  2.82762  2.77531  5.22750  3.43170     47 a - - -
          2.68618  4.42225  2.77519  2.73123  3.46354  2.40513  3.72494
3.29354  2.67741  2.69355  4.24690  2.90347  2.73739  3.18146  2.89801
2.37887  2.77519  2.98518  4.58477  3.61503
          0.02361  4.15374  4.87609  0.61958  0.77255  0.48576  0.95510
     45   2.93193  5.19201  3.29860  2.65588  4.59955  3.62698  3.26898
3.97990  1.47368  3.43437  4.25535  3.11794  3.99038  2.51645  1.80346
2.91354  2.84841  3.62623  5.51063  4.27018     48 k - - -
          2.68618  4.42225  2.77519  2.73123  3.46354  2.40513  3.72494
3.29354  2.67741  2.69355  4.24690  2.90347  2.73739  3.18146  2.89801
2.37887  2.77519  2.98518  4.58477  3.61503
          0.02361  4.15374  4.87609  0.61958  0.77255  0.48576  0.95510
     46   2.81229  3.62102  3.47311  2.85352  4.04375  3.57853  3.42876
3.50711  2.26568  3.10478  3.98466  3.25664  4.00088  2.94559  1.29304
2.90233  3.05240  3.21669  5.26793  4.00308     49 r - - -
          2.68618  4.42225  2.77519  2.73123  3.46354  2.40513  3.72494
3.29354  2.67741  2.69355  4.24690  2.90347  2.73739  3.18146  2.89801
2.37887  2.77519  2.98518  4.58477  3.61503
          0.02361  4.15374  4.87609  0.61958  0.77255  0.48576  0.95510
     47   2.84171  4.87664  3.39703  2.88153  4.41516  3.50360  3.81079
3.85820  2.24096  3.40215  4.27548  3.26346  4.00604  2.98108  1.16046
2.37732  3.12912  3.49633  5.49874  4.26482     50 r - - -
          2.68618  4.42225  2.77519  2.73123  3.46354  2.40513  3.72494
3.29354  2.67741  2.69355  4.24690  2.90347  2.73739  3.18146  2.89801
2.37887  2.77519  2.98518  4.58477  3.61503
          0.03257  4.15374  4.11421  0.61958  0.77255  0.48576  0.95510
     48   2.81414  5.19857  2.53948  1.29688  4.50042  3.35979  3.30045
3.98189  2.54944  3.51335  4.32857  2.85719  3.35094  2.87579  3.02221
2.64317  3.07258  3.59619  5.67877  4.27393     51 e - - -
          2.68618  4.42225  2.77519  2.73123  3.46354  2.40513  3.72494
3.29354  2.67741  2.69355  4.24690  2.90347  2.73739  3.18146  2.89801
2.37887  2.77519  2.98518  4.58477  3.61503
          0.04604  4.14499  3.53508  0.61958  0.77255  0.49625  0.93856
     49   3.02453  5.10323  3.41680  2.83510  4.57940  3.26189  3.70869
3.94619  1.92185  3.43150  4.30010  3.24092  4.06099  2.85536  1.12763
3.04438  3.22894  3.61872  5.50024  4.31737     52 r - - -
```

```
          2.68621  4.42228  2.77523  2.73126  3.46357  2.40516  3.72393
  3.29357  2.67744  2.69349  4.24693  2.90350  2.73743  3.18149  2.89804
  2.37890  2.77523  2.98521  4.58480  3.61506
          0.22701  2.94081  1.89535  1.01691  0.44896  0.52140  0.90064
     50   2.78038  4.93073  2.96964  2.52538  3.90738  3.47061  2.80160
  3.61993  2.26088  2.98395  4.03111  3.01102  3.89771  1.78112  2.58620
  2.79729  3.00610  3.31449  5.19435  3.79532     58 q - - -
          2.68618  4.42225  2.77519  2.73123  3.46354  2.40513  3.72494
  3.29354  2.67741  2.69355  4.24690  2.90347  2.73739  3.18146  2.89801
  2.37887  2.77519  2.98518  4.58477  3.61503
          0.02845  3.96967  4.69202  0.61958  0.77255  0.67090  0.71590
     51   2.58242  4.92017  2.86083  2.45575  4.12599  3.41061  3.07863
  3.64733  2.29564  3.15251  4.05120  2.91527  3.85876  1.76670  2.64843
  2.74476  2.96885  3.31772  5.34236  3.99497     59 q - - -
          2.68618  4.42225  2.77519  2.73123  3.46354  2.40513  3.72494
  3.29354  2.67741  2.69355  4.24690  2.90347  2.73739  3.18146  2.89801
  2.37887  2.77519  2.98518  4.58477  3.61503
          0.02845  3.96967  4.69202  0.61958  0.77255  0.67090  0.71590
     52   2.49970  4.79595  2.94335  2.41016  4.04222  3.40605  3.17716
  3.28820  2.35044  3.02561  3.88882  2.94880  3.81592  2.03576  2.75611
  2.52555  2.85724  3.14561  5.28676  3.96448     60 q - - -
          2.68618  4.42225  2.77519  2.73123  3.46354  2.40513  3.72494
  3.29354  2.67741  2.69355  4.24690  2.90347  2.73739  3.18146  2.89801
  2.37887  2.77519  2.98518  4.58477  3.61503
          0.02845  3.96967  4.69202  0.61958  0.77255  0.67090  0.71590
     53   2.62887  4.37938  3.41104  2.86402  3.43011  3.55953  3.35760
  2.72353  2.68448  1.78956  3.43049  3.27249  3.94566  2.83871  3.00215
  2.83030  2.72399  2.62681  4.89707  3.63111     61 l - - -
          2.68618  4.42225  2.77519  2.73123  3.46354  2.40513  3.72494
  3.29354  2.67741  2.69355  4.24690  2.90347  2.73739  3.18146  2.89801
  2.37887  2.77519  2.98518  4.58477  3.61503
          0.02845  3.96967  4.69202  0.61958  0.77255  0.67090  0.71590
     54   2.12095  4.56470  3.05853  2.50699  3.82946  3.32695  3.55311
  3.22161  2.52535  2.74612  3.71900  2.99198  3.71713  2.78033  2.94833
  2.33501  2.54251  2.88944  5.16298  3.87099     62 a - - -
          2.68618  4.42225  2.77519  2.73123  3.46354  2.40513  3.72494
  3.29354  2.67741  2.69355  4.24690  2.90347  2.73739  3.18146  2.89801
  2.37887  2.77519  2.98518  4.58477  3.61503
          0.02845  3.96967  4.69202  0.61958  0.77255  0.67090  0.71590
     55   1.97217  4.42030  3.20422  2.63925  3.74497  3.28721  3.74915
  3.11276  2.64312  2.77216  3.52329  3.10759  3.41039  2.93479  3.04487
  2.49607  2.40660  2.71365  5.11583  3.84780     63 a - - -
          2.68618  4.42225  2.77519  2.73123  3.46354  2.40513  3.72494
  3.29354  2.67741  2.69355  4.24690  2.90347  2.73739  3.18146  2.89801
  2.37887  2.77519  2.98518  4.58477  3.61503
          0.05139  3.96967  3.46693  0.61958  0.77255  0.67090  0.71590
     56   2.32068  4.24521  3.46158  2.90561  3.37314  3.42158  3.81981
  2.50548  2.84811  2.11642  3.38862  3.29027  3.23946  3.15625  3.19095
  2.63977  2.67646  2.46695  4.88360  3.65201     64 l - - -
          2.68618  4.42225  2.77519  2.73123  3.46354  2.40513  3.72494
  3.29354  2.67741  2.69355  4.24690  2.90347  2.73739  3.18146  2.89801
  2.37887  2.77519  2.98518  4.58477  3.61503
          0.02910  3.94738  4.66973  0.61958  0.77255  0.68917  0.69714
     57   2.36768  4.56136  3.07709  2.53940  3.49700  3.39049  3.66613
  3.12252  2.51007  2.84781  3.69845  3.02048  3.61944  2.24923  2.93393
  2.46468  2.65677  2.87001  5.14463  3.85061     65 q - - -
```

2.68618 4.42225 2.77519 2.73123 3.46354 2.40513 3.72494
3.29354 2.67741 2.69355 4.24690 2.90347 2.73739 3.18146 2.89801
2.37887 2.77519 2.98518 4.58477 3.61503
0.05608 3.94738 3.34585 0.61958 0.77255 0.68917 0.69714
58 2.28014 4.76849 2.92333 2.41490 4.05051 3.29154 3.43277
3.46569 2.35969 3.01591 3.88016 2.92455 3.37860 2.35524 2.65712
2.47430 2.82238 3.13682 5.29248 3.96818 66 a - - -
2.68618 4.42225 2.77519 2.73123 3.46354 2.40513 3.72494
3.29354 2.67741 2.69355 4.24690 2.90347 2.73739 3.18146 2.89801
2.37887 2.77519 2.98518 4.58477 3.61503
0.04418 3.92119 3.75500 0.61958 0.77255 0.47798 0.96768
59 2.59176 4.89563 2.88786 2.34064 4.16501 3.19450 3.24306
3.59429 2.37238 2.85975 3.97011 2.77971 3.72390 2.11197 2.82120
2.43188 2.85747 3.24869 5.37863 4.03057 67 q - - -
2.68618 4.42225 2.77519 2.73123 3.46354 2.40513 3.72494
3.29354 2.67741 2.69355 4.24690 2.90347 2.73739 3.18146 2.89801
2.37887 2.77519 2.98518 4.58477 3.61503
0.05725 4.06405 3.25812 0.61958 0.77255 0.58450 0.81505
60 2.28114 4.68490 2.99967 2.47928 3.90980 3.28748 3.54712
3.20582 2.45070 2.83279 3.67087 2.98382 3.71587 2.23913 2.85306
2.59883 2.70836 2.95987 5.21004 3.89792 68 q - - -
2.68618 4.42225 2.77519 2.73123 3.46354 2.40513 3.72494
3.29354 2.67741 2.69355 4.24690 2.90347 2.73739 3.18146 2.89801
2.37887 2.77519 2.98518 4.58477 3.61503
0.04649 4.03347 3.58580 0.61958 0.77255 0.61423 0.77883
61 2.22825 4.69471 3.02477 2.45474 3.91598 3.35331 3.33484
3.31852 2.41300 2.79958 3.62120 2.97886 3.70613 2.32126 2.86579
2.61500 2.74668 3.01832 5.21046 3.78656 69 a - - -
2.68618 4.42225 2.77519 2.73123 3.46354 2.40513 3.72494
3.29354 2.67741 2.69355 4.24690 2.90347 2.73739 3.18146 2.89801
2.37887 2.77519 2.98518 4.58477 3.61503
0.08961 4.01418 2.69331 0.61958 0.77255 0.62100 0.77091
62 2.34937 4.83033 2.81441 2.24183 4.09765 3.27478 3.54000
3.52288 2.38704 3.07701 3.91551 2.88947 3.35895 2.33539 2.84996
2.50625 2.81996 2.99679 5.33475 3.98768 70 e - - -
2.68618 4.42225 2.77519 2.73123 3.46354 2.40513 3.72494
3.29354 2.67741 2.69355 4.24690 2.90347 2.73739 3.18146 2.89801
2.37887 2.77519 2.98518 4.58477 3.61503
0.06567 3.96039 3.11206 0.61958 0.77255 0.67859 0.70792
63 2.32293 4.47115 3.14013 2.57634 3.67297 3.18062 3.56050
3.05190 2.56668 2.46174 3.58876 3.06730 3.61308 2.73308 2.97423
2.54299 2.73464 2.57368 5.04499 3.77133 71 a - - -
2.68618 4.42225 2.77519 2.73123 3.46354 2.40513 3.72494
3.29354 2.67741 2.69355 4.24690 2.90347 2.73739 3.18146 2.89801
2.37887 2.77519 2.98518 4.58477 3.61503
0.09187 3.92450 2.68791 0.61958 0.77255 0.69018 0.69612
64 2.39405 4.84418 2.62454 2.30373 4.14987 2.92625 3.59853
3.58007 2.39237 3.15819 3.96645 2.85185 3.75962 2.38533 2.73336
2.40332 2.83308 3.18997 5.37526 4.02243 72 e - - -
2.68618 4.42225 2.77519 2.73123 3.46354 2.40513 3.72494
3.29354 2.67741 2.69355 4.24690 2.90347 2.73739 3.18146 2.89801
2.37887 2.77519 2.98518 4.58477 3.61503
0.03129 3.87586 4.59821 0.61958 0.77255 0.74311 0.64556
65 2.36720 4.54496 3.05454 2.47850 3.63667 2.95576 3.52743
2.94668 2.49479 2.79456 3.64507 2.81648 3.80014 2.67982 2.92125
2.63272 2.72942 2.74323 5.09380 3.80257 73 a - - -

2.68618 4.42225 2.77519 2.73123 3.46354 2.40513 3.72494
3.29354 2.67741 2.69355 4.24690 2.90347 2.73739 3.18146 2.89801
2.37887 2.77519 2.98518 4.58477 3.61503
0.05235 3.87586 3.49762 0.61958 0.77255 0.52745 0.89186
66 2.37135 4.79730 2.90825 2.25776 4.06458 2.96743 3.55828
3.48587 2.41114 3.08122 3.89123 2.78049 3.47471 2.59073 2.87426
2.54840 2.58644 2.92038 5.31652 3.97598 74 e - - -
2.68594 4.42242 2.77532 2.73135 3.46370 2.40509 3.72500
3.29359 2.67757 2.69371 4.24706 2.90331 2.73730 3.18083 2.89813
2.37877 2.77521 2.98525 4.58493 3.61520
0.54857 0.90147 4.11974 0.96261 0.48109 0.55605 0.85208
67 2.40037 4.70617 3.02044 2.46597 3.93447 2.99624 3.55173
3.17684 2.43057 2.81387 3.79041 2.59846 3.43143 2.77688 2.72486
2.56727 2.73028 2.91433 5.22812 3.91025 83 a - - -
2.68618 4.42225 2.77519 2.73123 3.46354 2.40513 3.72494
3.29354 2.67741 2.69355 4.24690 2.90347 2.73739 3.18146 2.89801
2.37887 2.77519 2.98518 4.58477 3.61503
0.22498 4.04979 1.69261 0.61958 0.77255 0.58431 0.81529
68 2.31195 4.66585 2.90105 2.36038 3.82368 3.03105 3.61992
3.33670 2.44664 2.96136 3.79233 2.82220 3.65202 2.64140 2.89292
2.34450 2.74763 3.02462 5.22507 3.77999 84 a - - -
2.68618 4.42225 2.77519 2.73123 3.46354 2.40513 3.72494
3.29354 2.67741 2.69355 4.24690 2.90347 2.73739 3.18146 2.89801
2.37887 2.77519 2.98518 4.58477 3.61503
0.03159 3.86651 4.58886 0.61958 0.77255 0.71409 0.67263
69 2.21785 4.43367 3.05975 2.60877 3.52465 2.89301 3.71333
3.06237 2.60386 2.75508 3.66989 3.05534 3.46239 2.93367 3.01974
2.21032 2.74933 2.84509 5.12143 3.84255 85 s - - -
2.68618 4.42225 2.77519 2.73123 3.46354 2.40513 3.72494
3.29354 2.67741 2.69355 4.24690 2.90347 2.73739 3.18146 2.89801
2.37887 2.77519 2.98518 4.58477 3.61503
0.03815 3.89077 4.07442 0.61958 0.77255 0.73241 0.65537
70 2.33833 4.61380 2.92824 2.46823 3.82300 2.67874 3.56699
3.31016 2.45912 2.88451 3.77353 2.92386 3.65211 2.81412 2.67958
2.43729 2.77801 2.95177 5.20798 3.89942 86 a - - -
2.68618 4.42225 2.77519 2.73123 3.46354 2.40513 3.72494
3.29354 2.67741 2.69355 4.24690 2.90347 2.73739 3.18146 2.89801
2.37887 2.77519 2.98518 4.58477 3.61503
0.03104 3.88367 4.60601 0.61958 0.77255 0.72394 0.66327
71 2.28977 4.64662 2.75063 2.43020 4.10810 2.46943 3.69291
3.53049 2.53313 3.13963 3.96211 2.91779 3.50349 2.63649 2.93193
2.23796 2.81009 3.16244 5.37909 4.05393 87 s - - -
2.68618 4.42225 2.77519 2.73123 3.46354 2.40513 3.72494
3.29354 2.67741 2.69355 4.24690 2.90347 2.73739 3.18146 2.89801
2.37887 2.77519 2.98518 4.58477 3.61503
0.06753 3.89275 3.10297 0.61958 0.77255 0.73097 0.65670
72 2.48807 4.38372 2.80695 2.35445 3.96146 2.92069 3.51385
3.37305 2.44842 2.99186 3.81520 2.92404 3.40786 2.78962 2.90064
2.25879 2.64633 2.99883 5.24795 3.92670 88 s - - -
2.68618 4.42225 2.77519 2.73123 3.46354 2.40513 3.72494
3.29354 2.67741 2.69355 4.24690 2.90347 2.73739 3.18146 2.89801
2.37887 2.77519 2.98518 4.58477 3.61503
0.03189 3.85711 4.57945 0.61958 0.77255 0.70730 0.67920
73 2.29197 4.31054 3.05211 2.51518 3.90146 2.93901 3.58661
3.30400 2.48205 2.93831 3.77055 2.79197 3.64033 2.84035 2.62183
2.41045 2.62243 2.92435 5.20779 3.90796 89 a - - -

2.68618 4.42225 2.77519 2.73123 3.46354 2.40513 3.72494
3.29354 2.67741 2.69355 4.24690 2.90347 2.73739 3.18146 2.89801
2.37887 2.77519 2.98518 4.58477 3.61503
0.08461 3.89081 2.80180 0.61958 0.77255 0.71681 0.67003
74 2.34274 4.69178 2.62057 2.31209 4.03238 2.88127 3.63343
3.44838 2.47468 3.06109 3.88329 2.83443 3.37300 2.79879 2.93785
2.40604 2.79649 2.85533 5.30971 3.97678 90 e - - -
2.68618 4.42225 2.77519 2.73123 3.46354 2.40513 3.72494
3.29354 2.67741 2.69355 4.24690 2.90347 2.73739 3.18146 2.89801
2.37887 2.77519 2.98518 4.58477 3.61503
0.03214 3.84958 4.57193 0.61958 0.77255 0.74755 0.64155
75 2.32650 4.64034 2.87788 2.44596 3.98519 2.64822 3.63766
3.39350 2.39270 3.01163 3.83913 2.93714 3.58936 2.76888 2.65309
2.45807 2.74179 2.99231 5.26383 3.95106 91 a - - -
2.68619 4.42226 2.77519 2.73125 3.46355 2.40514 3.72496
3.29338 2.67742 2.69354 4.24691 2.90340 2.73741 3.18148 2.89802
2.37886 2.77521 2.98520 4.58478 3.61504
0.09617 2.50780 4.58144 0.64514 0.74358 0.36631 1.18184
76 2.39794 4.56243 3.01131 2.46445 3.95109 3.21722 3.59642
3.46867 2.42995 3.07375 3.88695 2.76111 2.89755 2.78385 2.82553
2.14322 2.68650 3.13768 5.31758 3.98683 94 s - - -
2.68618 4.42225 2.77519 2.73123 3.46354 2.40513 3.72494
3.29354 2.67741 2.69355 4.24690 2.90347 2.73739 3.18146 2.89801
2.37887 2.77519 2.98518 4.58477 3.61503
0.03311 4.13990 4.09551 0.61958 0.77255 0.48872 0.95039
77 3.04219 5.18576 3.20918 2.73880 4.61426 3.62246 3.71236
3.98752 1.02835 3.46610 4.34808 3.17714 4.05471 2.69617 2.30568
3.03867 3.13517 3.66157 5.55275 4.33372 95 k - - -
2.68618 4.42225 2.77519 2.73123 3.46354 2.40513 3.72494
3.29354 2.67741 2.69355 4.24690 2.90347 2.73739 3.18146 2.89801
2.37887 2.77519 2.98518 4.58477 3.61503
0.02399 4.13801 4.86036 0.61958 0.77255 0.50447 0.92590
78 3.11574 5.21343 3.54814 2.87731 4.68817 3.72998 3.67881
4.02777 1.68925 3.47050 4.34151 3.27173 3.85355 2.81481 1.14616
3.11581 3.28451 3.70900 5.51345 4.35392 96 r - - -
2.68623 4.42230 2.77524 2.73119 3.46359 2.40518 3.72476
3.29344 2.67746 2.69351 4.24695 2.90352 2.73736 3.18138 2.89806
2.37879 2.77515 2.98523 4.58482 3.61508
0.06751 3.03022 4.07616 1.32492 0.30901 0.47424 0.97381
79 2.56481 4.53109 3.21643 2.66102 3.68445 3.50447 3.37803
2.99869 2.60849 2.50130 3.62389 3.12996 2.34046 2.80250 3.01092
2.73528 2.75957 2.82828 5.06529 3.28742 106 p - - -
2.68618 4.42225 2.77519 2.73123 3.46354 2.40513 3.72494
3.29354 2.67741 2.69355 4.24690 2.90347 2.73739 3.18146 2.89801
2.37887 2.77519 2.98518 4.58477 3.61503
0.02383 4.14462 4.86697 0.61958 0.77255 0.49669 0.93788
80 2.96485 5.05260 3.41694 2.78748 4.40499 3.65546 3.56609
3.79885 1.98481 3.11239 4.17416 3.20995 4.03708 2.82757 1.24391
2.75222 3.15816 3.48928 5.42053 4.20139 107 r - - -
2.68619 4.42232 2.77502 2.73121 3.46361 2.40511 3.72476
3.29344 2.67748 2.69362 4.24653 2.90337 2.73747 3.18154 2.89808
2.37887 2.77527 2.98513 4.58484 3.61510
0.32291 2.49319 1.64342 1.32647 0.30845 0.49669 0.93788
81 2.53662 4.61228 2.98000 2.07032 3.83468 3.40635 3.65452
3.06607 2.49163 2.49253 3.71698 2.99629 3.72408 2.84511 2.92119
2.51782 2.70890 2.86535 5.16610 3.86570 118 e - - -

```
          2.68617  4.42229  2.77518  2.73127  3.46358  2.40508  3.72499
3.29350  2.67745  2.69343  4.24694  2.90351  2.73744  3.18150  2.89800
2.37891  2.77524  2.98501  4.58481  3.61507
          0.39304  1.15346  4.66164  0.20723  1.67576  0.60002  0.79584
     82   2.41515  4.44647  2.87039  2.38369  3.89749  2.83737  3.50984
3.25911  2.45020  2.92994  3.75945  2.77119  3.80223  2.79596  2.86351
2.49958  2.72621  2.86881  4.42863  3.88469    120 e - - -
          2.68610  4.42239  2.77491  2.73129  3.46368  2.40506  3.72509
3.29364  2.67742  2.69355  4.24704  2.90352  2.73748  3.18135  2.89815
2.37870  2.77521  2.98526  4.58491  3.61517
          0.54059  1.37998  1.79569  1.22662  0.34712  0.55936  0.84764
     83   2.43642  4.78510  2.85418  2.32982  3.95533  3.31148  3.57401
3.46063  2.33489  3.00536  3.78350  2.77883  3.35498  2.54480  2.70116
2.47090  2.66420  3.12746  5.28326  3.94250    134 e - - -
          2.68618  4.42225  2.77519  2.73123  3.46354  2.40513  3.72494
3.29354  2.67741  2.69355  4.24690  2.90347  2.73739  3.18146  2.89801
2.37887  2.77519  2.98518  4.58477  3.61503
          0.05329  3.89013  3.45929  0.61958  0.77255  0.67055  0.71627
     84   2.35781  4.58959  2.94946  2.49110  3.81227  3.29593  3.52702
3.00045  2.47799  2.85256  3.62000  2.98536  3.57677  2.67025  2.91592
2.38204  2.58723  2.87319  5.14002  3.83932    135 a - - -
          2.68618  4.42225  2.77519  2.73123  3.46354  2.40513  3.72494
3.29354  2.67741  2.69355  4.24690  2.90347  2.73739  3.18146  2.89801
2.37887  2.77519  2.98518  4.58477  3.61503
          0.03020  3.91096  4.63331  0.61958  0.77255  0.68032  0.70614
     85   2.26157  4.18921  3.25477  2.67138  3.50343  3.18425  3.72182
2.89389  2.66193  2.50511  3.50219  3.02353  3.75328  2.89857  3.05475
2.62058  2.52386  2.50498  4.96988  3.71287    136 a - - -
          2.68618  4.42225  2.77519  2.73123  3.46354  2.40513  3.72494
3.29354  2.67741  2.69355  4.24690  2.90347  2.73739  3.18146  2.89801
2.37887  2.77519  2.98518  4.58477  3.61503
          0.04549  3.93521  3.69172  0.61958  0.77255  0.54647  0.86510
     86   2.34269  4.24820  2.86120  2.50687  3.92810  2.89578  3.66827
3.33257  2.50294  2.96438  3.79343  2.88127  3.37734  2.78630  2.94955
2.53710  2.41068  2.80253  5.23416  3.92442    137 a - - -
          2.68618  4.42225  2.77519  2.73123  3.46354  2.40513  3.72494
3.29354  2.67741  2.69355  4.24690  2.90347  2.73739  3.18146  2.89801
2.37887  2.77519  2.98518  4.58477  3.61503
          0.06193  4.02020  3.16771  0.61958  0.77255  0.61375  0.77939
     87   2.28342  4.70181  2.99502  2.48451  4.03481  2.94651  3.48274
3.44439  2.42408  3.05423  3.87859  2.81822  3.80086  2.81921  2.41921
2.62303  2.35410  3.11338  5.29658  3.98470    138 a - - -
          2.68618  4.42225  2.77519  2.73123  3.46354  2.40513  3.72494
3.29354  2.67741  2.69355  4.24690  2.90347  2.73739  3.18146  2.89801
2.37887  2.77519  2.98518  4.58477  3.61503
          0.02776  3.99399  4.71633  0.61958  0.77255  0.58547  0.81383
     88   2.45853  4.49088  3.18543  2.62765  3.68117  3.10097  3.61384
2.88872  2.57025  2.52510  3.59746  3.09601  3.79283  2.90143  2.85989
2.22551  2.68829  2.69216  5.05576  3.78239    139 s - - -
          2.68618  4.42225  2.77519  2.73123  3.46354  2.40513  3.72494
3.29354  2.67741  2.69355  4.24690  2.90347  2.73739  3.18146  2.89801
2.37887  2.77519  2.98518  4.58477  3.61503
          0.02668  4.03305  4.75539  0.61958  0.77255  0.59052  0.80752
     89   2.34496  4.62949  3.07144  2.51602  3.74076  3.04220  2.99949
3.24273  2.49654  2.84498  3.43675  3.01248  3.13674  2.84275  2.85749
2.48239  2.77516  2.92805  5.17038  3.86769    140 a - - -
```

```
          2.68618  4.42225  2.77519  2.73123  3.46354  2.40513  3.72494
3.29354  2.67741  2.69355  4.24690  2.90347  2.73739  3.18146  2.89801
2.37887  2.77519  2.98518  4.58477  3.61503
          0.02630  4.04695  4.76930  0.61958  0.77255  0.58957  0.80870
     90   2.13785  4.32559  3.19026  2.87403  4.14411  3.03592  3.99876
3.54041  2.91753  3.21145  4.04188  3.19368  3.54059  3.22677  3.29989
1.80954  1.74829  3.10429  5.47737  4.22332    141 t - - -
          2.68618  4.42225  2.77519  2.73123  3.46354  2.40513  3.72494
3.29354  2.67741  2.69355  4.24690  2.90347  2.73739  3.18146  2.89801
2.37887  2.77519  2.98518  4.58477  3.61503
          0.02613  4.05363  4.77598  0.61958  0.77255  0.59483  0.80219
     91   1.89708  4.38741  3.26720  2.77654  3.78414  3.22330  3.87071
3.33688  2.76663  3.00982  3.85421  3.15765  3.30259  3.03004  3.16471
1.83041  2.54630  2.92532  5.30124  4.03528    142 s - - -
          2.68618  4.42225  2.77519  2.73123  3.46354  2.40513  3.72494
3.29354  2.67741  2.69355  4.24690  2.90347  2.73739  3.18146  2.89801
2.37887  2.77519  2.98518  4.58477  3.61503
          0.02613  4.05363  4.77598  0.61958  0.77255  0.59483  0.80219
     92   2.47720  4.34606  3.38614  2.82915  3.52347  3.51461  3.80157
2.65800  2.77239  2.23273  3.33962  2.86624  3.91511  3.09868  3.13838
2.68806  2.17990  2.58369  4.95828  3.71374    143 t - - -
          2.68618  4.42225  2.77519  2.73123  3.46354  2.40513  3.72494
3.29354  2.67741  2.69355  4.24690  2.90347  2.73739  3.18146  2.89801
2.37887  2.77519  2.98518  4.58477  3.61503
          0.02613  4.05363  4.77598  0.61958  0.77255  0.52195  0.89984
     93   2.25633  4.70488  2.88694  2.45252  4.06118  3.11139  3.68568
3.47802  2.50603  3.08919  3.90464  2.88336  3.24405  2.84603  2.96278
2.18722  2.44065  2.94841  5.33596  4.01046    144 s - - -
          2.68620  4.42138  2.77531  2.73147  3.46373  2.40526  3.72518
3.29349  2.67764  2.69346  4.24680  2.90329  2.73739  3.18143  2.89797
2.37881  2.77474  2.98506  4.58485  3.61527
          0.69690  0.94639  2.17390  1.23858  0.34220  0.51125  0.91566
     94   2.16765  4.43094  3.00636  2.53068  3.92338  3.34615  3.58297
3.32561  2.52674  2.96261  3.79463  2.67779  2.91297  2.86825  2.96642
2.44685  2.68354  2.80352  5.23531  3.93125    155 a - - -
          2.68618  4.42225  2.77519  2.73123  3.46354  2.40513  3.72494
3.29354  2.67741  2.69355  4.24690  2.90347  2.73739  3.18146  2.89801
2.37887  2.77519  2.98518  4.58477  3.61503
          0.04116  4.00851  3.80916  0.61958  0.77255  0.63722  0.75239
     95   2.23346  4.73412  2.69016  2.38076  4.07600  3.32328  3.66894
3.49179  2.50231  3.10289  3.92356  2.75843  3.78705  2.79291  2.96308
2.36279  2.23059  3.10543  5.34909  4.01617    156 t - - -
          2.68618  4.42225  2.77519  2.73123  3.46354  2.40513  3.72494
3.29354  2.67741  2.69355  4.24690  2.90347  2.73739  3.18146  2.89801
2.37887  2.77519  2.98518  4.58477  3.61503
          0.02773  3.99507  4.71742  0.61958  0.77255  0.51377  0.91189
     96   2.51723  4.75786  2.94043  2.45941  4.08683  3.36786  3.49941
3.50443  2.39867  3.10869  3.92646  2.71034  2.86429  2.83162  2.92020
2.39607  2.17760  3.16538  5.34877  4.02074    157 t - - -
          2.68618  4.42225  2.77519  2.73123  3.46354  2.40513  3.72494
3.29354  2.67741  2.69355  4.24690  2.90347  2.73739  3.18146  2.89801
2.37887  2.77519  2.98518  4.58477  3.61503
          0.09889  4.07795  2.56116  0.61958  0.77255  0.57038  0.83312
     97   2.49676  4.47659  3.19115  2.64459  3.75841  3.38941  3.72663
3.08471  2.59663  2.71663  3.66532  3.09610  2.82092  2.94757  2.82452
2.39586  2.36513  2.62994  5.12025  3.84608    158 t - - -
```

2.68618 4.42225 2.77519 2.73123 3.46354 2.40513 3.72494
3.29354 2.67741 2.69355 4.24690 2.90347 2.73739 3.18146 2.89801
2.37887 2.77519 2.98518 4.58477 3.61503
0.02741 4.00646 4.72881 0.61958 0.77255 0.61465 0.77834
98 2.57775 4.77083 2.88225 2.41975 3.78610 3.25331 3.60954
3.42809 2.29941 2.81912 3.84665 2.82928 3.79597 2.72208 2.59870
2.31833 2.53663 3.10612 5.27557 3.94382 159 k - - -
2.68618 4.42225 2.77519 2.73123 3.46354 2.40513 3.72494
3.29354 2.67741 2.69355 4.24690 2.90347 2.73739 3.18146 2.89801
2.37887 2.77519 2.98518 4.58477 3.61503
0.02701 4.02092 4.74327 0.61958 0.77255 0.54964 0.86075
99 2.52205 4.58663 3.03948 2.38974 4.04177 3.07339 3.76827
3.43264 2.60642 3.07953 3.82864 3.04512 3.80715 2.95442 3.03007
1.68509 2.52970 2.96333 5.34513 4.04497 160 s - - -
2.68611 4.42233 2.77496 2.73154 3.46270 2.40529 3.72473
3.29373 2.67757 2.69368 4.24732 2.90379 2.73757 3.18119 2.89815
2.37844 2.77492 2.98531 4.58519 3.61509
0.30598 1.60596 2.76622 2.29360 0.10636 0.55033 0.85981
100 2.59961 4.90503 2.77261 2.17711 4.17784 2.80892 3.59641
3.61401 2.37711 2.99732 3.97459 2.85280 3.68048 2.56876 2.80014
2.25289 2.77576 3.25809 5.38889 3.90328 181 e - - -
2.68630 4.42167 2.77537 2.73090 3.46397 2.40463 3.72533
3.29351 2.67781 2.69331 4.24599 2.90376 2.73744 3.18151 2.89828
2.37897 2.77486 2.98507 4.58485 3.61546
0.35864 1.22849 4.75125 2.06978 0.13492 0.42073 1.06876
101 2.51553 4.64218 2.96237 2.40691 4.17699 3.32967 2.98740
3.60800 2.32695 3.17669 3.97095 2.68340 3.81328 2.69780 2.39775
2.24123 2.80529 3.14375 5.38475 4.03401 213 s - - -
2.68618 4.42225 2.77519 2.73123 3.46354 2.40513 3.72494
3.29354 2.67741 2.69355 4.24690 2.90347 2.73739 3.18146 2.89801
2.37887 2.77519 2.98518 4.58477 3.61503
0.02376 4.14759 4.86994 0.61958 0.77255 0.48117 0.96249
102 3.34984 4.67471 4.76743 4.27918 1.35163 4.40044 4.05474
2.65276 4.11116 1.35071 3.21349 4.29248 4.66220 4.17118 4.18194
3.73982 3.56963 2.72835 3.94941 2.68323 214 l - - -
2.68618 4.42225 2.77519 2.73123 3.46354 2.40513 3.72494
3.29354 2.67741 2.69355 4.24690 2.90347 2.73739 3.18146 2.89801
2.37887 2.77519 2.98518 4.58477 3.61503
0.02361 4.15374 4.87609 0.61958 0.77255 0.48576 0.95510
103 2.19544 4.40368 3.38360 3.12755 4.22855 3.17598 4.23577
3.53160 3.14205 3.14773 4.22087 3.38389 1.14441 3.50847 3.44692
2.64835 2.92978 3.14073 5.62247 4.36854 215 p - - -
2.68618 4.42225 2.77519 2.73123 3.46354 2.40513 3.72494
3.29354 2.67741 2.69355 4.24690 2.90347 2.73739 3.18146 2.89801
2.37887 2.77519 2.98518 4.58477 3.61503
0.02361 4.15374 4.87609 0.61958 0.77255 0.48576 0.95510
104 2.30872 4.86623 2.84829 2.39161 4.19518 2.77021 3.64741
3.62869 2.42801 3.20226 3.99704 2.93955 2.84114 2.78333 2.48087
2.43971 2.81858 3.20382 5.41403 4.06189 216 a - - -
2.68618 4.42225 2.77519 2.73123 3.46354 2.40513 3.72494
3.29354 2.67741 2.69355 4.24690 2.90347 2.73739 3.18146 2.89801
2.37887 2.77519 2.98518 4.58477 3.61503
0.02361 4.15374 4.87609 0.61958 0.77255 0.48576 0.95510
105 2.74763 5.13981 2.79439 1.55245 4.45616 3.42732 3.26769
3.90628 2.22437 3.41408 4.20334 2.90751 3.85892 2.45392 2.70196
2.71630 2.97685 3.37510 5.56215 4.19679 217 e - - -

```
          2.68618  4.42225  2.77519  2.73123  3.46354  2.40513  3.72494
3.29354  2.67741  2.69355  4.24690  2.90347  2.73739  3.18146  2.89801
2.37887  2.77519  2.98518  4.58477  3.61503
          0.02361  4.15374  4.87609  0.61958  0.77255  0.48576  0.95510
    106   3.18406  4.48794  4.89698  4.38044  3.57139  4.55058  5.06060
1.69547  4.26024  1.84916  3.38066  4.60539  4.83175  4.50827  4.44925
3.92575  3.43986  0.95709  5.54395  4.34931    218 v - - -
          2.68618  4.42225  2.77519  2.73123  3.46354  2.40513  3.72494
3.29354  2.67741  2.69355  4.24690  2.90347  2.73739  3.18146  2.89801
2.37887  2.77519  2.98518  4.58477  3.61503
          0.02361  4.15374  4.87609  0.61958  0.77255  0.48576  0.95510
    107   2.69206  4.93015  2.96760  2.39805  4.26021  3.43875  3.55934
3.68240  2.35128  3.24488  4.05551  2.85870  3.86404  2.80599  2.16432
1.83298  2.79164  3.33147  5.43753  4.11221    219 s - - -
          2.68618  4.42225  2.77519  2.73123  3.46354  2.40513  3.72494
3.29354  2.67741  2.69355  4.24690  2.90347  2.73739  3.18146  2.89801
2.37887  2.77519  2.98518  4.58477  3.61503
          0.02361  4.15374  4.87609  0.61958  0.77255  0.48576  0.95510
    108   1.81537  4.33313  3.46804  2.97515  3.90862  3.15762  3.99642
3.22501  2.93884  2.65199  3.83447  3.29782  3.84456  3.25919  3.30278
1.67760  2.71436  2.70042  5.29968  4.05725    220 s - - -
          2.68618  4.42225  2.77519  2.73123  3.46354  2.40513  3.72494
3.29354  2.67741  2.69355  4.24690  2.90347  2.73739  3.18146  2.89801
2.37887  2.77519  2.98518  4.58477  3.61503
          0.02361  4.15374  4.87609  0.61958  0.77255  0.48576  0.95510
    109   2.59931  5.05779  2.41574  2.16149  4.35564  3.37852  3.64334
3.81011  2.43947  3.34928  4.13347  2.83892  2.30985  2.56796  2.93102
2.55711  2.91443  3.17193  5.53408  4.14527    221 e - - -
          2.68618  4.42225  2.77519  2.73123  3.46354  2.40513  3.72494
3.29354  2.67741  2.69355  4.24690  2.90347  2.73739  3.18146  2.89801
2.37887  2.77519  2.98518  4.58477  3.61503
          0.02361  4.15374  4.87609  0.61958  0.77255  0.48576  0.95510
    110   0.67374  4.35772  3.77269  3.58841  4.35656  3.17269  4.57363
3.52606  3.61264  3.39375  4.40194  3.65904  3.93945  3.92912  3.83673
2.70940  3.00683  3.13030  5.75460  4.59006    222 A - - -
          2.68618  4.42225  2.77519  2.73123  3.46354  2.40513  3.72494
3.29354  2.67741  2.69355  4.24690  2.90347  2.73739  3.18146  2.89801
2.37887  2.77519  2.98518  4.58477  3.61503
          0.02361  4.15374  4.87609  0.61958  0.77255  0.48576  0.95510
    111   2.63533  4.32481  3.20674  2.88312  3.45416  3.60545  3.75738
2.70960  2.91637  2.27223  3.43250  3.36049  3.99772  3.23136  3.25095
2.87858  2.84198  1.60719  4.92392  3.61775    223 v - - -
          2.68618  4.42225  2.77519  2.73123  3.46354  2.40513  3.72494
3.29354  2.67741  2.69355  4.24690  2.90347  2.73739  3.18146  2.89801
2.37887  2.77519  2.98518  4.58477  3.61503
          0.02361  4.15374  4.87609  0.61958  0.77255  0.48576  0.95510
    112   3.64002  4.96169  4.62697  4.40310  0.64358  4.24624  4.03529
3.13902  4.30558  2.44755  3.83219  4.34869  4.70462  4.39759  4.35519
3.88945  3.94440  3.14903  4.21629  2.55435    224 F - - -
          2.68618  4.42225  2.77519  2.73123  3.46354  2.40513  3.72494
3.29354  2.67741  2.69355  4.24690  2.90347  2.73739  3.18146  2.89801
2.37887  2.77519  2.98518  4.58477  3.61503
          0.02361  4.15374  4.87609  0.61958  0.77255  0.48576  0.95510
    113   3.18122  5.29343  3.62089  2.90247  4.76671  3.78379  3.55803
4.09937  1.60405  3.50896  4.37820  3.28992  4.13073  2.70819  1.15659
3.16632  3.32601  3.78328  5.52565  4.37842    225 r - - -
```

```
         2.68618  4.42225  2.77519  2.73123  3.46354  2.40513  3.72494
3.29354  2.67741  2.69355  4.24690  2.90347  2.73739  3.18146  2.89801
2.37887  2.77519  2.98518  4.58477  3.61503
         0.02361  4.15374  4.87609  0.61958  0.77255  0.48576  0.95510
    114  2.64852  1.12819  3.96646  3.50212  3.87674  3.40782  4.24201
3.20120  3.08770  2.96673  3.94000  3.66659  4.03039  3.60956  2.51398
2.88147  3.03877  2.92109  5.28724  4.08128    226 c - - -
         2.68618  4.42225  2.77519  2.73123  3.46354  2.40513  3.72494
3.29354  2.67741  2.69355  4.24690  2.90347  2.73739  3.18146  2.89801
2.37887  2.77519  2.98518  4.58477  3.61503
         0.02361  4.15374  4.87609  0.61958  0.77255  0.48576  0.95510
    115  2.73451  4.23304  4.06093  3.49636  3.34796  3.83691  3.73404
2.09311  3.36543  2.27037  3.17142  3.76864  4.20858  3.64833  3.59951
3.13461  2.83976  1.32916  4.93387  3.72271    227 v - - -
         2.68618  4.42225  2.77519  2.73123  3.46354  2.40513  3.72494
3.29354  2.67741  2.69355  4.24690  2.90347  2.73739  3.18146  2.89801
2.37887  2.77519  2.98518  4.58477  3.61503
         0.02361  4.15374  4.87609  0.61958  0.77255  0.48576  0.95510
    116  3.12703  5.25773  3.54891  2.85413  4.68705  3.75280  3.17080
4.04768  1.71830  3.47623  4.33787  3.25759  4.10376  2.74826  1.18108
3.11520  3.28140  3.72875  5.50620  4.33373    228 r - - -
         2.68618  4.42225  2.77519  2.73123  3.46354  2.40513  3.72494
3.29354  2.67741  2.69355  4.24690  2.90347  2.73739  3.18146  2.89801
2.37887  2.77519  2.98518  4.58477  3.61503
         0.02361  4.15374  4.87609  0.61958  0.77255  0.48576  0.95510
    117  3.15038  4.47008  4.82874  4.27985  3.42072  4.47566  4.89063
1.83822  4.16450  1.69862  2.77370  4.50526  4.73661  4.36135  4.32998
3.82224  3.39078  1.10422  5.35872  4.21307    229 v - - -
         2.68618  4.42225  2.77519  2.73123  3.46354  2.40513  3.72494
3.29354  2.67741  2.69355  4.24690  2.90347  2.73739  3.18146  2.89801
2.37887  2.77519  2.98518  4.58477  3.61503
         0.02361  4.15374  4.87609  0.61958  0.77255  0.48576  0.95510
    118  2.31946  4.29116  3.44251  3.10818  4.38905  2.26683  4.21585
3.81292  3.16389  3.47836  4.29092  3.32450  3.77118  3.45826  3.51204
1.33467  1.99984  3.26949  5.70948  4.48017    230 s - - -
         2.68618  4.42225  2.77519  2.73123  3.46354  2.40513  3.72494
3.29354  2.67741  2.69355  4.24690  2.90347  2.73739  3.18146  2.89801
2.37887  2.77519  2.98518  4.58477  3.61503
         0.04339  4.15374  3.62114  0.61958  0.77255  0.48576  0.95510
    119  1.92949  4.22841  3.28324  2.80176  4.15018  2.43501  3.75142
3.56881  2.80330  3.20792  4.02674  3.17147  2.87176  3.12825  3.21232
1.82375  2.78610  3.14807  5.46051  4.18561    231 s - - -
         2.68618  4.42225  2.77519  2.73123  3.46354  2.40513  3.72494
3.29354  2.67741  2.69355  4.24690  2.90347  2.73739  3.18146  2.89801
2.37887  2.77519  2.98518  4.58477  3.61503
         0.02407  4.13443  4.85678  0.61958  0.77255  0.50864  0.91958
    120  2.80920  4.23154  4.32755  3.75967  3.36195  3.99087  4.35634
1.59841  3.64533  2.16304  2.86182  3.98327  3.79921  3.88008  3.83965
3.29917  2.85192  1.53251  5.01288  3.82474    232 v - - -
         2.68618  4.42225  2.77517  2.73120  3.46354  2.40513  3.72495
3.29354  2.67741  2.69355  4.24690  2.90347  2.73740  3.18147  2.89801
2.37887  2.77520  2.98519  4.58477  3.61503
         0.07065  3.34595  3.41175  0.53429  0.88209  0.50864  0.91958
    121  2.93464  5.51206  1.34582  1.82293  4.80183  3.01343  3.61341
4.31832  2.74988  3.80445  4.62828  2.61474  3.87514  2.92866  3.33410
2.66793  3.21125  3.88557  5.96424  4.46756    234 d - - -
```

2.68618 4.42225 2.77519 2.73123 3.46354 2.40513 3.72494
3.29354 2.67741 2.69355 4.24690 2.90347 2.73739 3.18146 2.89801
2.37887 2.77519 2.98518 4.58477 3.61503
0.02471 4.10890 4.83125 0.61958 0.77255 0.53745 0.87763
122 2.78898 5.45249 1.50665 1.82854 4.75200 3.19037 3.74943
4.26071 2.69083 3.74770 4.55806 2.43999 3.86203 2.84283 3.26043
2.53942 3.16006 3.82851 5.90544 4.42323 235 d - - -
2.68619 4.42226 2.77518 2.73121 3.46355 2.40508 3.72495
3.29355 2.67741 2.69356 4.24690 2.90347 2.73740 3.18147 2.89801
2.37887 2.77520 2.98519 4.58478 3.61504
0.07541 2.73866 4.83125 0.44612 1.02194 0.45474 1.00679
123 2.03553 4.87343 2.67089 2.33379 4.27205 2.24013 3.71676
3.71018 2.54835 3.28910 4.09289 2.76981 3.42328 2.86521 3.02814
2.55631 2.80614 3.33273 5.50565 4.14721 237 a - - -
2.68618 4.42225 2.77519 2.73123 3.46354 2.40513 3.72494
3.29354 2.67741 2.69355 4.24690 2.90347 2.73739 3.18146 2.89801
2.37887 2.77519 2.98518 4.58477 3.61503
0.02361 4.15374 4.87609 0.61958 0.77255 0.48576 0.95510
124 2.76921 5.25533 1.94516 1.63526 4.56027 3.35233 3.68170
4.03936 2.35021 3.54362 4.32704 2.81923 3.84159 2.75326 3.02625
2.66305 3.01736 3.23819 5.70247 4.27432 238 e - - -
2.68618 4.42225 2.77519 2.73123 3.46354 2.40513 3.72494
3.29354 2.67741 2.69355 4.24690 2.90347 2.73739 3.18146 2.89801
2.37887 2.77519 2.98518 4.58477 3.61503
0.02361 4.15374 4.87609 0.61958 0.77255 0.48576 0.95510
125 2.46961 5.26976 1.85454 1.96828 4.58264 3.08785 3.67704
4.07118 2.51487 3.56494 4.34112 2.55547 3.39993 2.63212 3.03995
2.65385 3.01018 3.64729 5.71758 4.28070 239 d - - -
2.68608 4.42226 2.77520 2.73124 3.46355 2.40512 3.72495
3.29355 2.67741 2.69356 4.24690 2.90347 2.73740 3.18147 2.89801
2.37887 2.77520 2.98519 4.58478 3.61504
0.07590 2.72627 4.87609 0.43654 1.03922 0.48576 0.95510
126 2.75241 5.10682 2.80804 1.57458 4.40792 3.43266 3.65465
3.72341 2.34571 3.37309 4.08111 2.92378 3.86827 2.17648 2.69719
2.72931 2.98395 3.48076 5.54091 4.18154 241 e - - -
2.68618 4.42225 2.77519 2.73123 3.46354 2.40513 3.72494
3.29354 2.67741 2.69355 4.24690 2.90347 2.73739 3.18146 2.89801
2.37887 2.77519 2.98518 4.58477 3.61503
0.02361 4.15374 4.87609 0.61958 0.77255 0.48576 0.95510
127 2.73707 3.91493 4.29175 3.71652 2.49315 3.85797 4.04982
2.32206 3.57923 2.02148 3.18800 3.86017 4.20926 3.76171 3.71693
3.15757 2.96868 1.99862 4.56563 2.01643 242 v - - -
2.68618 4.42225 2.77519 2.73123 3.46354 2.40513 3.72494
3.29354 2.67741 2.69355 4.24690 2.90347 2.73739 3.18146 2.89801
2.37887 2.77519 2.98518 4.58477 3.61503
0.02361 4.15374 4.87609 0.61958 0.77255 0.48576 0.95510
128 0.87034 4.21410 3.67982 3.41534 4.33016 2.69471 4.41190
3.59011 3.43344 3.40792 4.28656 3.48537 3.80327 3.72311 3.69915
2.50733 2.80854 2.97587 5.72326 4.52581 243 a - - -
2.68618 4.42225 2.77519 2.73123 3.46354 2.40513 3.72494
3.29354 2.67741 2.69355 4.24690 2.90347 2.73739 3.18146 2.89801
2.37887 2.77519 2.98518 4.58477 3.61503
0.02361 4.15374 4.87609 0.61958 0.77255 0.48576 0.95510
129 3.67898 5.05277 4.26641 4.05671 2.36299 4.16794 3.78809
3.65234 3.90700 3.03117 4.29919 4.08770 4.63892 4.14320 4.02904
3.78391 3.96911 3.53721 4.01463 0.60146 244 Y - - -

```
         2.68618  4.42225  2.77519  2.73123  3.46354  2.40513  3.72494
3.29354  2.67741  2.69355  4.24690  2.90347  2.73739  3.18146  2.89801
2.37887  2.77519  2.98518  4.58477  3.61503
         0.02361  4.15374  4.87609  0.61958  0.77255  0.48576  0.95510
    130  2.70035  4.89011  3.04729  2.47475  4.10121  3.48564  3.32815
3.55960  2.29694  3.03897  3.83588  3.01142  3.87872  1.68853  2.71894
2.68963  2.92327  3.23895  5.32681  3.65989    245 q - - -
         2.68618  4.42225  2.77519  2.73123  3.46354  2.40513  3.72494
3.29354  2.67741  2.69355  4.24690  2.90347  2.73739  3.18146  2.89801
2.37887  2.77519  2.98518  4.58477  3.61503
         0.02361  4.15374  4.87609  0.61958  0.77255  0.48576  0.95510
    131  1.52100  4.23742  3.68142  3.34804  4.22491  3.09582  4.33664
3.38116  3.32357  3.24711  4.15309  3.47207  3.82788  3.63331  3.60975
2.54415  1.35566  2.97401  5.63643  4.43415    246 t - - -
         2.68618  4.42225  2.77519  2.73123  3.46354  2.40513  3.72494
3.29354  2.67741  2.69355  4.24690  2.90347  2.73739  3.18146  2.89801
2.37887  2.77519  2.98518  4.58477  3.61503
         0.02361  4.15374  4.87609  0.61958  0.77255  0.48576  0.95510
    132  1.91670  4.42436  3.32669  2.78759  3.73790  3.41135  3.82931
3.08905  2.70682  2.79550  3.45315  3.04733  3.87762  3.08105  3.14218
2.48207  2.15156  2.57739  5.12871  3.87074    247 a - - -
         2.68618  4.42225  2.77519  2.73123  3.46354  2.40513  3.72494
3.29354  2.67741  2.69355  4.24690  2.90347  2.73739  3.18146  2.89801
2.37887  2.77519  2.98518  4.58477  3.61503
         0.02361  4.15374  4.87609  0.61958  0.77255  0.48576  0.95510
    133  3.03310  4.47454  4.60576  4.21566  3.68976  4.13962  4.95956
1.91257  4.06311  2.28865  3.58118  4.37308  4.62948  4.39884  4.26196
3.59169  3.36553  0.80825  5.58848  4.31365    248 v - - -
         2.68618  4.42225  2.77519  2.73123  3.46354  2.40513  3.72494
3.29354  2.67741  2.69355  4.24690  2.90347  2.73739  3.18146  2.89801
2.37887  2.77519  2.98518  4.58477  3.61503
         0.02361  4.15374  4.87609  0.61958  0.77255  0.48576  0.95510
    134  2.64961  4.99273  2.86883  2.37643  4.30306  3.19556  3.37548
3.74715  2.05684  3.29022  4.07565  2.42567  3.81971  2.72123  2.83053
2.20805  2.54415  3.37182  5.47386  4.10792    249 k - - -
         2.68618  4.42225  2.77519  2.73123  3.46354  2.40513  3.72494
3.29354  2.67741  2.69355  4.24690  2.90347  2.73739  3.18146  2.89801
2.37887  2.77519  2.98518  4.58477  3.61503
         0.02361  4.15374  4.87609  0.61958  0.77255  0.48576  0.95510
    135  3.25682  4.60987  4.71761  4.35166  3.58004  4.35724  5.03646
0.78444  4.18563  2.11817  3.48159  4.55922  4.76995  4.51072  4.35146
3.88812  3.55441  1.89597  5.52259  4.27933    250 i - - -
         2.68618  4.42225  2.77519  2.73123  3.46354  2.40513  3.72494
3.29354  2.67741  2.69355  4.24690  2.90347  2.73739  3.18146  2.89801
2.37887  2.77519  2.98518  4.58477  3.61503
         0.02361  4.15374  4.87609  0.61958  0.77255  0.48576  0.95510
    136  1.86824  4.34931  3.25846  2.99297  4.43992  1.40884  4.18795
3.87954  3.14561  3.52818  4.33907  2.92725  3.78003  3.42191  3.51574
2.13462  2.82252  3.32865  5.74940  4.50257    251 g - - -
         2.68618  4.42225  2.77519  2.73123  3.46354  2.40513  3.72494
3.29354  2.67741  2.69355  4.24690  2.90347  2.73739  3.18146  2.89801
2.37887  2.77519  2.98518  4.58477  3.61503
         0.02361  4.15374  4.87609  0.61958  0.77255  0.48576  0.95510
    137  3.05382  4.79169  3.78440  3.72406  4.84291  0.40257  4.81787
4.52014  3.94306  4.13547  5.12012  3.94993  4.19102  4.24549  4.13940
3.23524  3.55679  3.99395  5.85436  4.95750    252 G - - -
```

2.68618 4.42225 2.77519 2.73123 3.46354 2.40513 3.72494
3.29354 2.67741 2.69355 4.24690 2.90347 2.73739 3.18146 2.89801
2.37887 2.77519 2.98518 4.58477 3.61503
0.02361 4.15374 4.87609 0.61958 0.77255 0.48576 0.95510
138 3.06027 5.01828 3.23304 2.88747 3.68215 3.62360 1.10550
3.84203 2.50319 3.34794 4.30201 3.31208 4.11866 3.14402 2.55787
3.10607 3.32012 3.55171 5.07502 3.59915 253 h - - -
2.68618 4.42225 2.77519 2.73123 3.46354 2.40513 3.72494
3.29354 2.67741 2.69355 4.24690 2.90347 2.73739 3.18146 2.89801
2.37887 2.77519 2.98518 4.58477 3.61503
0.02361 4.15374 4.87609 0.61958 0.77255 0.48576 0.95510
139 3.05469 4.41015 4.66108 4.12382 3.57207 4.34625 4.81762
1.79009 4.00691 1.97738 3.31303 4.36414 4.66448 4.27111 4.21796
3.69344 3.02563 1.01417 5.42118 4.22840 254 v - - -
2.68618 4.42225 2.77519 2.73123 3.46354 2.40513 3.72494
3.29354 2.67741 2.69355 4.24690 2.90347 2.73739 3.18146 2.89801
2.37887 2.77519 2.98518 4.58477 3.61503
0.02361 4.15374 4.87609 0.61958 0.77255 0.48576 0.95510
140 3.39390 4.75027 4.69829 4.29008 0.99639 4.37313 4.10147
2.37438 4.13807 1.97388 3.35428 4.30982 4.70282 4.23855 4.23593
3.76850 3.65077 2.62384 4.31377 2.69792 255 f - - -
2.68618 4.42225 2.77519 2.73123 3.46354 2.40513 3.72494
3.29354 2.67741 2.69355 4.24690 2.90347 2.73739 3.18146 2.89801
2.37887 2.77519 2.98518 4.58477 3.61503
0.02361 4.15374 4.87609 0.61958 0.77255 0.48576 0.95510
141 3.17885 5.28890 3.61130 2.90983 4.78126 3.77150 3.68094
4.11551 1.07356 3.52747 4.40150 3.29785 4.13139 2.81239 1.68316
3.16603 3.33303 3.79522 5.54086 4.39462 256 k - - -
2.68618 4.42225 2.77519 2.73123 3.46354 2.40513 3.72494
3.29354 2.67741 2.69355 4.24690 2.90347 2.73739 3.18146 2.89801
2.37887 2.77519 2.98518 4.58477 3.61503
0.02361 4.15374 4.87609 0.61958 0.77255 0.48576 0.95510
142 3.05382 4.79169 3.78440 3.72406 4.84291 0.40257 4.81787
4.52014 3.94306 4.13547 5.12012 3.94993 4.19102 4.24549 4.13940
3.23524 3.55679 3.99395 5.85436 4.95750 257 G - - -
2.68618 4.42225 2.77519 2.73123 3.46354 2.40513 3.72494
3.29354 2.67741 2.69355 4.24690 2.90347 2.73739 3.18146 2.89801
2.37887 2.77519 2.98518 4.58477 3.61503
0.02361 4.15374 4.87609 0.61958 0.77255 0.48576 0.95510
143 3.14215 4.45431 4.89693 4.34010 2.38420 4.44229 4.78189
1.30708 4.21651 1.70918 3.13669 4.50687 4.69588 4.35015 4.32469
3.78570 3.37626 1.74307 5.16507 3.97956 258 i - - -
2.68618 4.42225 2.77519 2.73123 3.46354 2.40513 3.72494
3.29354 2.67741 2.69355 4.24690 2.90347 2.73739 3.18146 2.89801
2.37887 2.77519 2.98518 4.58477 3.61503
0.02361 4.15374 4.87609 0.61958 0.77255 0.48576 0.95510
144 3.48662 4.87077 4.64049 4.29080 3.26797 4.33624 4.85946
2.49612 4.05625 0.59265 3.21920 4.53931 4.72902 4.35596 4.18248
3.95442 3.77122 2.60478 5.23946 4.02466 259 L - - -
2.68618 4.42225 2.77519 2.73123 3.46354 2.40513 3.72494
3.29354 2.67741 2.69355 4.24690 2.90347 2.73739 3.18146 2.89801
2.37887 2.77519 2.98518 4.58477 3.61503
0.02361 4.15374 4.87609 0.61958 0.77255 0.48576 0.95510
145 3.12294 4.82178 3.68095 3.21946 2.55627 3.89762 2.70845
3.42071 2.94343 2.93384 3.95987 3.52561 4.28052 3.38413 2.91449
3.22135 3.34745 3.21343 4.16752 1.20933 260 y - - -

```
          2.68618  4.42225  2.77519  2.73123  3.46354  2.40513  3.72494
3.29354  2.67741  2.69355  4.24690  2.90347  2.73739  3.18146  2.89801
2.37887  2.77519  2.98518  4.58477  3.61503
          0.02361  4.15374  4.87609  0.61958  0.77255  0.48576  0.95510
    146   2.90574  5.32785  1.14868  2.24254  4.55669  3.21294  3.80767
4.13108  2.77716  3.67266  4.51428  2.67729  3.90138  2.97998  3.32720
2.76792  3.19093  3.73457  5.80649  3.76571    261 d - - -
          2.68618  4.42225  2.77519  2.73123  3.46354  2.40513  3.72494
3.29354  2.67741  2.69355  4.24690  2.90347  2.73739  3.18146  2.89801
2.37887  2.77519  2.98518  4.58477  3.61503
          0.02361  4.15374  4.87609  0.61958  0.77255  0.48576  0.95510
    147   2.67788  4.76871  3.10503  2.56319  3.79494  3.49964  2.83223
3.38818  2.45220  2.96512  3.84908  3.05621  3.89198  1.91836  2.85158
2.73476  2.90599  2.72695  5.14179  3.39097    262 q - - -
          2.68618  4.42225  2.77519  2.73123  3.46354  2.40513  3.72494
3.29354  2.67741  2.69355  4.24690  2.90347  2.73739  3.18146  2.89801
2.37887  2.77519  2.98518  4.58477  3.61503
          0.02361  4.15374  4.87609  0.61958  0.77255  0.48576  0.95510
    148   3.05382  4.79169  3.78440  3.72406  4.84291  0.40257  4.81787
4.52014  3.94306  4.13547  5.12012  3.94993  4.19102  4.24549  4.13940
3.23524  3.55679  3.99395  5.85436  4.95750    263 G - - -
          2.68618  4.42225  2.77519  2.73123  3.46354  2.40513  3.72494
3.29354  2.67741  2.69355  4.24690  2.90347  2.73739  3.18146  2.89801
2.37887  2.77519  2.98518  4.58477  3.61503
          0.02361  4.15374  4.87609  0.61958  0.77255  0.48576  0.95510
    149   2.45258  4.34138  3.47834  2.91714  3.50134  3.56352  3.77803
2.74668  2.85761  2.27605  3.30472  3.32096  2.28703  3.17451  3.21156
2.82074  2.70116  2.34399  4.95736  3.72151    264 l - - -
          2.68618  4.42225  2.77519  2.73123  3.46354  2.40513  3.72494
3.29354  2.67741  2.69355  4.24690  2.90347  2.73739  3.18146  2.89801
2.37887  2.77519  2.98518  4.58477  3.61503
          0.03216  4.15374  4.13900  0.61958  0.77255  0.48576  0.95510
    150   2.54625  5.37001  1.73537  1.69487  4.67202  3.27010  3.44421
4.16999  2.60908  3.66113  4.45419  2.58157  3.85659  2.85669  3.15429
2.66412  3.09412  3.74376  5.81651  4.35828    265 e - - -
          2.68618  4.42225  2.77519  2.73123  3.46354  2.40513  3.72494
3.29354  2.67741  2.69355  4.24690  2.90347  2.73739  3.18146  2.89801
2.37887  2.77519  2.98518  4.58477  3.61503
          0.16545  4.14539  1.99030  0.61958  0.77255  0.49577  0.93931
    151   2.47683  5.03178  2.54128  2.18097  4.33155  3.34440  3.33709
3.79107  2.24436  3.31783  4.09274  2.54559  3.65499  2.65911  2.85183
2.29154  2.73234  3.39945  5.49145  4.09966    266 e - - -
          2.68618  4.42225  2.77519  2.73123  3.46354  2.40513  3.72494
3.29354  2.67741  2.69355  4.24690  2.90347  2.73739  3.18146  2.89801
2.37887  2.77519  2.98518  4.58477  3.61503
          0.01851  3.99845        *  0.61958  0.77255  0.00000        *
//
HMMER3/f [3.3 | Nov 2019]
NAME  E2F_TDP
ACC   PF02319.21
DESC  E2F/DP family winged-helix DNA-binding domain
LENG  69
ALPH  amino
RF    no
MM    no
CONS  yes
CS    no
MAP   yes
```

```
DATE  Tue Apr 21 21:18:27 2020
NSEQ  169
EFFN  3.535446
CKSUM 2655815412
GA    25.70 25.70
TC    25.70 25.80
NC    25.50 25.60
STATS LOCAL MSV       -8.8194  0.71875
STATS LOCAL VITERBI   -9.5243  0.71875
STATS LOCAL FORWARD   -4.1857  0.71875
HMM          A        C        D        E        F        G        H
I        K        L        M        N        P        Q        R        S
T        V        W        Y
            m->m     m->i     m->d     i->m     i->i     d->m     d->d
  COMPO   2.65389  4.34445  3.00218  2.76433  3.51399  3.01462  3.87565
2.70085  2.48954  2.29318  3.69305  3.00009  3.75426  3.14429  2.71899
2.62694  2.88525  2.61764  4.32230  3.44820
          2.68618  4.42225  2.77519  2.73123  3.46354  2.40513  3.72494
3.29354  2.67741  2.69355  4.24690  2.90347  2.73739  3.18146  2.89801
2.37887  2.77519  2.98518  4.58477  3.61503
          0.01214  5.05763  5.16697  0.61958  0.77255  0.00000        *
      1   2.72857  5.18415  3.11501  2.48310  2.87578  3.46155  3.68647
3.92734  1.63301  3.49152  4.27928  2.83380  3.96772  2.85796  2.57827
2.24506  2.90079  3.44278  5.78581  3.81130      1 k - - -
          2.68618  4.42225  2.77519  2.73123  3.46354  2.40513  3.72494
3.29354  2.67741  2.69355  4.24690  2.90347  2.73739  3.18146  2.89801
2.37887  2.77519  2.98518  4.58477  3.61503
          0.00952  5.05501  5.77736  0.61958  0.77255  0.48093  0.96288
      2   2.80279  5.40307  1.83452  1.96903  4.74403  2.79225  3.63846
2.89996  2.51724  3.66438  4.33884  2.93013  3.96858  2.68129  2.90480
2.48049  3.03050  3.79151  5.83812  4.42941      2 d - - -
          2.68618  4.42225  2.77519  2.73123  3.46354  2.40513  3.72494
3.29354  2.67741  2.69355  4.24690  2.90347  2.73739  3.18146  2.89801
2.37887  2.77519  2.98518  4.58477  3.61503
          0.00949  5.05763  5.77998  0.61958  0.77255  0.48576  0.95510
      3   2.85389  5.38610  3.01238  2.53931  4.72073  3.32768  3.53388
4.19902  1.29081  3.65702  4.23255  2.76320  4.07220  2.92055  2.87942
2.27191  2.77901  3.45076  5.08567  4.42084      3 k - - -
          2.68618  4.42225  2.77519  2.73123  3.46354  2.40513  3.72494
3.29354  2.67741  2.69355  4.24690  2.90347  2.73739  3.18146  2.89801
2.37887  2.77519  2.98518  4.58477  3.61503
          0.00949  5.05763  5.77998  0.61958  0.77255  0.48576  0.95510
      4   2.81293  4.85077  4.53861  4.30435  5.44910  1.51357  5.29571
4.93476  4.37630  4.58938  5.34734  4.22081  3.81502  4.58915  4.62774
0.68710  2.75804  4.13999  6.75620  5.61777      4 S - - -
          2.68618  4.42225  2.77519  2.73123  3.46354  2.40513  3.72494
3.29354  2.67741  2.69355  4.24690  2.90347  2.73739  3.18146  2.89801
2.37887  2.77519  2.98518  4.58477  3.61503
          0.00949  5.05763  5.77998  0.61958  0.77255  0.48576  0.95510
      5   4.54094  5.67575  6.48874  5.89822  3.36590  6.01563  6.21610
2.51152  5.78488  0.36493  2.40144  6.19565  5.83438  5.45324  5.63486
5.45663  4.71681  3.24434  5.96862  5.10857      5 L - - -
          2.68618  4.42225  2.77519  2.73123  3.46354  2.40513  3.72494
3.29354  2.67741  2.69355  4.24690  2.90347  2.73739  3.18146  2.89801
2.37887  2.77519  2.98518  4.58477  3.61503
          0.00949  5.05763  5.77998  0.61958  0.77255  0.48576  0.95510
```

```
      6   2.46782  4.67653  3.25820  2.58289  4.22772  1.89638  3.68651
3.87914  2.34507  3.32985  3.97578  3.20898  4.10951  2.96626  2.28117
2.50147  2.68574  3.29919  4.09124  4.08409      6 g - - -
          2.68618  4.42225  2.77519  2.73123  3.46354  2.40513  3.72494
3.29354  2.67741  2.69355  4.24690  2.90347  2.73739  3.18146  2.89801
2.37887  2.77519  2.98518  4.58477  3.61503
          0.00949  5.05763  5.77998  0.61958  0.77255  0.48576  0.95510
      7   2.38102  4.30006  4.11351  3.33920  3.48101  3.97839  2.87342
2.60270  3.36931  1.92969  3.17853  3.09357  4.35213  3.42156  3.25202
2.87397  2.99484  1.82933  5.09544  3.26765      7 v - - -
          2.68618  4.42225  2.77519  2.73123  3.46354  2.40513  3.72494
3.29354  2.67741  2.69355  4.24690  2.90347  2.73739  3.18146  2.89801
2.37887  2.77519  2.98518  4.58477  3.61503
          0.00949  5.05763  5.77998  0.61958  0.77255  0.48576  0.95510
      8   2.99761  4.44839  4.97530  4.36652  2.32895  4.25016  4.57932
2.33355  4.16972  0.98818  2.98462  4.39147  4.59574  4.29715  4.19687
3.38950  2.63600  2.56134  5.06078  3.83451      8 l - - -
          2.68618  4.42225  2.77519  2.73123  3.46354  2.40513  3.72494
3.29354  2.67741  2.69355  4.24690  2.90347  2.73739  3.18146  2.89801
2.37887  2.77519  2.98518  4.58477  3.61503
          0.00949  5.05763  5.77998  0.61958  0.77255  0.48576  0.95510
      9   2.30108  1.59065  4.87474  4.51431  4.89803  3.64534  5.22256
4.28144  4.41895  4.01583  4.85520  4.30307  4.43563  4.62323  4.59632
1.41589  1.33230  3.59434  6.31397  5.17408      9 t - - -
          2.68618  4.42225  2.77519  2.73123  3.46354  2.40513  3.72494
3.29354  2.67741  2.69355  4.24690  2.90347  2.73739  3.18146  2.89801
2.37887  2.77519  2.98518  4.58477  3.61503
          0.00949  5.05763  5.77998  0.61958  0.77255  0.48576  0.95510
     10   2.77831  5.37585  2.94291  2.51411  4.58541  3.67975  3.18780
3.86335  1.90387  3.29005  3.26744  3.10815  4.07286  1.94375  2.48839
2.56814  2.79302  3.38951  5.81730  4.19897     10 k - - -
          2.68618  4.42225  2.77519  2.73123  3.46354  2.40513  3.72494
3.29354  2.67741  2.69355  4.24690  2.90347  2.73739  3.18146  2.89801
2.37887  2.77519  2.98518  4.58477  3.61503
          0.00949  5.05763  5.77998  0.61958  0.77255  0.48576  0.95510
     11   2.80633  5.04386  3.20256  2.64463  4.62431  3.15774  3.88743
3.73262  1.37234  3.02640  3.97379  2.73064  4.09095  2.90134  2.18729
2.72320  3.09588  3.56208  5.77232  4.38944     11 k - - -
          2.68618  4.42225  2.77519  2.73123  3.46354  2.40513  3.72494
3.29354  2.67741  2.69355  4.24690  2.90347  2.73739  3.18146  2.89801
2.37887  2.77519  2.98518  4.58477  3.61503
          0.00949  5.05763  5.77998  0.61958  0.77255  0.48576  0.95510
     12   3.89807  5.15474  5.85130  5.27276  1.15467  5.22328  5.50336
2.58379  5.11638  1.27612  3.36095  5.37325  5.37896  5.07906  5.08952
4.58547  4.11510  1.69275  5.66957  4.56713     12 f - - -
          2.68618  4.42225  2.77519  2.73123  3.46354  2.40513  3.72494
3.29354  2.67741  2.69355  4.24690  2.90347  2.73739  3.18146  2.89801
2.37887  2.77519  2.98518  4.58477  3.61503
          0.00949  5.05763  5.77998  0.61958  0.77255  0.48576  0.95510
     13   2.83170  2.61200  4.89926  4.28436  3.18730  4.10902  4.44259
1.81780  4.07029  1.71249  2.91517  4.27008  4.47245  4.20221  3.70900
3.30206  3.13816  1.72472  4.57828  3.52688     13 l - - -
          2.68618  4.42225  2.77519  2.73123  3.46354  2.40513  3.72494
3.29354  2.67741  2.69355  4.24690  2.90347  2.73739  3.18146  2.89801
2.37887  2.77519  2.98518  4.58477  3.61503
          0.00949  5.05763  5.77998  0.61958  0.77255  0.48576  0.95510
```

```
     14   2.50409  5.40234  2.64832  2.10911  4.65238  3.41231  3.56172
4.06056  2.07654  3.70616  3.28683  2.98030  4.06827  2.08949  2.84289
2.68970  3.00403  3.70360  5.83708  2.97094     14 k - - -
          2.68618  4.42225  2.77519  2.73123  3.46354  2.40513  3.72494
3.29354  2.67741  2.69355  4.24690  2.90347  2.73739  3.18146  2.89801
2.37887  2.77519  2.98518  4.58477  3.61503
          0.00949  5.05763  5.77998  0.61958  0.77255  0.48576  0.95510
     15   2.87320  3.86656  4.20569  3.43532  3.35749  3.99808  3.11943
2.68271  2.23256  1.34012  3.00496  3.90401  4.37033  3.73856  3.39792
3.27207  3.09270  2.61295  5.06768  3.26690     15 l - - -
          2.68618  4.42225  2.77519  2.73123  3.46354  2.40513  3.72494
3.29354  2.67741  2.69355  4.24690  2.90347  2.73739  3.18146  2.89801
2.37887  2.77519  2.98518  4.58477  3.61503
          0.00949  5.05763  5.77998  0.61958  0.77255  0.48576  0.95510
     16   2.96839  3.63592  4.92624  4.31204  1.89484  4.13974  4.47100
2.38299  4.09913  1.58742  3.18575  4.30001  4.49952  4.08448  4.02988
3.45162  3.20023  1.89788  4.95875  2.51848     16 l - - -
          2.68620  4.42227  2.77522  2.73126  3.46356  2.40512  3.72497
3.29356  2.67743  2.69328  4.24692  2.90349  2.73739  3.18149  2.89803
2.37889  2.77522  2.98521  4.58479  3.61505
          0.33970  2.77330  1.48915  0.28708  1.38811  0.48576  0.95510
     17   2.74406  5.12924  3.05917  2.14086  4.62901  3.57854  3.61573
4.10863  1.75015  3.33692  3.97766  2.89008  3.02806  2.32044  2.48177
2.62646  3.00299  3.52453  5.46235  4.21527     18 k - - -
          2.68618  4.42225  2.77519  2.73123  3.46354  2.40513  3.72494
3.29354  2.67741  2.69355  4.24690  2.90347  2.73739  3.18146  2.89801
2.37887  2.77519  2.98518  4.58477  3.61503
          0.01223  4.80599  5.52834  0.61958  0.77255  0.67849  0.70802
     18   2.52933  4.77819  2.56178  2.02834  4.66065  3.38607  3.64571
4.14032  2.43806  2.65313  4.04869  2.82235  3.90465  2.60708  2.26960
2.42157  3.02370  3.45388  5.76266  4.28736     19 e - - -
          2.68619  4.42149  2.77522  2.73115  3.46387  2.40532  3.72521
3.29341  2.67756  2.69384  4.24723  2.90340  2.73699  3.18145  2.89830
2.37875  2.77492  2.98511  4.58510  3.61459
          0.62490  1.43557  1.48411  1.27633  0.32720  0.84708  0.55978
     19   2.03064  4.53772  2.76826  2.39169  4.28430  3.43728  3.66693
3.87896  2.41110  3.30816  4.18355  2.84090  3.59078  2.70163  2.94133
1.87360  2.86687  3.40067  5.17400  4.21331     30 s - - -
          2.68618  4.42225  2.77519  2.73123  3.46354  2.40513  3.72494
3.29354  2.67741  2.69355  4.24690  2.90347  2.73739  3.18146  2.89801
2.37887  2.77519  2.98518  4.58477  3.61503
          0.06028  4.64773  3.01756  0.61958  0.77255  1.18116  0.36662
     20   2.17648  5.15792  2.84509  2.27230  4.47008  3.51643  3.63168
3.60414  2.19833  3.10394  4.10378  2.72387  2.67615  2.75114  2.88181
2.48869  2.75666  3.53076  5.61078  3.98474     31 a - - -
          2.68631  4.42294  2.77589  2.73133  3.46423  2.40368  3.72404
3.29319  2.67698  2.69310  4.24759  2.90346  2.73809  3.18216  2.89870
2.37914  2.77465  2.98588  4.58546  3.61429
          0.18932  1.90149  3.76664  2.59341  0.07771  0.27023  1.44056
     21   2.77420  5.38078  2.32950  2.07238  4.72222  2.99666  3.74134
3.77001  1.97800  2.81155  4.10070  2.61234  3.39437  2.89151  2.71777
2.77271  2.96220  3.53456  5.81564  4.24493     50 k - - -
          2.68618  4.42225  2.77519  2.73123  3.46354  2.40513  3.72494
3.29354  2.67741  2.69355  4.24690  2.90347  2.73739  3.18146  2.89801
2.37887  2.77519  2.98518  4.58477  3.61503
          0.00998  5.00778  5.73012  0.61958  0.77255  0.56668  0.83795
```

```
     22   2.62673  5.09001  2.67103  2.32804  4.41626  1.81435  3.69909
4.17209  2.37254  3.62866  4.08982  2.88418  3.97936  2.72651  2.67040
2.70149  2.96244  2.85985  5.80387  4.21512      51 g - - -
          2.68618  4.42225  2.77519  2.73123  3.46354  2.40513  3.72494
3.29354  2.67741  2.69355  4.24690  2.90347  2.73739  3.18146  2.89801
2.37887  2.77519  2.98518  4.58477  3.61503
          0.09566  5.01988  2.46951  0.61958  0.77255  0.59072  0.80727
     23   2.75844  4.57468  3.33343  2.72851  3.91496  3.36931  3.77733
2.64660  2.77569  2.59145  3.72451  3.37640  3.71571  3.21371  2.98875
2.76778  1.70617  2.34834  5.03460  3.89337      52 t - - -
          2.68640  4.42247  2.77523  2.73063  3.46375  2.40497  3.72510
3.29365  2.67748  2.69369  4.24711  2.90368  2.73761  3.18150  2.89808
2.37896  2.77532  2.98462  4.58498  3.61425
          0.17239  1.86525  5.65730  1.22147  0.34927  0.33702  1.25139
     24   2.96731  3.90630  4.92196  4.30838  2.94870  4.13993  4.47408
1.74517  4.09690  1.52876  3.16569  4.29906  4.50018  4.22790  4.10857
3.45180  2.15610  1.98206  4.69255  3.58890      58 l - - -
          2.68618  4.42225  2.77519  2.73123  3.46354  2.40513  3.72494
3.29354  2.67741  2.69355  4.24690  2.90347  2.73739  3.18146  2.89801
2.37887  2.77519  2.98518  4.58477  3.61503
          0.00949  5.05763  5.77998  0.61958  0.77255  0.48576  0.95510
     25   2.75147  4.07400  2.00415  2.51062  4.52038  3.32780  3.67184
4.05952  2.49819  3.67293  4.41900  2.72430  3.91622  2.87635  3.10036
1.84647  2.22943  3.75656  5.81626  4.17144      59 s - - -
          2.68618  4.42225  2.77519  2.73123  3.46354  2.40513  3.72494
3.29354  2.67741  2.69355  4.24690  2.90347  2.73739  3.18146  2.89801
2.37887  2.77519  2.98518  4.58477  3.61503
          0.00949  5.05763  5.77998  0.61958  0.77255  0.48576  0.95510
     26   2.87942  4.14525  4.69557  4.09404  3.39482  4.09648  4.41655
2.46156  3.92172  1.05677  3.41339  4.18294  4.46044  3.75027  3.35527
3.39815  3.10217  2.49625  4.97871  2.28836      60 l - - -
          2.68618  4.42225  2.77519  2.73123  3.46354  2.40513  3.72494
3.29354  2.67741  2.69355  4.24690  2.90347  2.73739  3.18146  2.89801
2.37887  2.77519  2.98518  4.58477  3.61503
          0.00949  5.05763  5.77998  0.61958  0.77255  0.48576  0.95510
     27   2.80534  4.75035  2.13620  2.25806  4.75018  3.57395  3.31963
4.11396  2.39283  3.71151  4.44832  1.79693  4.06844  2.91066  2.85183
2.58841  2.49589  3.72819  5.84088  4.43102      61 n - - -
          2.68618  4.42225  2.77519  2.73123  3.46354  2.40513  3.72494
3.29354  2.67741  2.69355  4.24690  2.90347  2.73739  3.18146  2.89801
2.37887  2.77519  2.98518  4.58477  3.61503
          0.00949  5.05763  5.77998  0.61958  0.77255  0.48576  0.95510
     28   2.23816  5.14746  2.24585  1.73634  4.48142  3.56796  3.60026
3.95357  2.39642  3.53868  3.82857  2.99512  4.07100  2.55347  3.03124
2.75549  2.78025  3.42479  5.45031  3.81438      62 e - - -
          2.68618  4.42225  2.77519  2.73123  3.46354  2.40513  3.72494
3.29354  2.67741  2.69355  4.24690  2.90347  2.73739  3.18146  2.89801
2.37887  2.77519  2.98518  4.58477  3.61503
          0.00949  5.05763  5.77998  0.61958  0.77255  0.48576  0.95510
     29   1.21141  4.45610  4.87968  4.30342  3.72197  4.11888  4.72910
2.30312  4.15994  2.24164  3.62836  4.42365  4.70135  4.35661  4.28157
3.65201  3.20238  1.44243  5.31157  4.12263      63 a - - -
          2.68603  4.42231  2.77502  2.73129  3.46359  2.40518  3.72500
3.29321  2.67746  2.69360  4.24695  2.90352  2.73745  3.18152  2.89806
2.37877  2.77525  2.98524  4.58482  3.61509
          0.04288  3.24706  5.77998  1.05865  0.42606  0.48576  0.95510
```

```
     30   1.14557  3.63403  4.23314  3.65835  3.55530  3.98218  4.18892
2.95088  3.46400  2.57694  3.54873  3.92380  4.37703  3.81345  3.80289
2.57708  2.87758  2.04523  5.10585  3.90532     68 a - - -
          2.68618  4.42225  2.77519  2.73123  3.46354  2.40513  3.72494
3.29354  2.67741  2.69355  4.24690  2.90347  2.73739  3.18146  2.89801
2.37887  2.77519  2.98518  4.58477  3.61503
          0.00949  5.05763  5.77998  0.61958  0.77255  0.48576  0.95510
     31   2.51655  5.41088  2.31113  1.97049  4.75608  3.46866  3.76626
3.90032  1.81653  3.71620  4.21744  2.69331  4.06707  2.73249  2.71723
2.64117  2.73492  3.70444  5.84346  4.43244     69 k - - -
          2.68618  4.42225  2.77519  2.73123  3.46354  2.40513  3.72494
3.29354  2.67741  2.69355  4.24690  2.90347  2.73739  3.18146  2.89801
2.37887  2.77519  2.98518  4.58477  3.61503
          0.00949  5.05763  5.77998  0.61958  0.77255  0.48576  0.95510
     32   2.33978  5.04579  3.07057  1.61405  4.61984  3.69139  3.66209
3.14560  2.27556  3.34510  3.77735  2.96895  4.08385  2.79609  2.76496
2.81550  2.78225  2.94407  5.77049  4.04098     70 e - - -
          2.68618  4.42225  2.77519  2.73123  3.46354  2.40513  3.72494
3.29354  2.67741  2.69355  4.24690  2.90347  2.73739  3.18146  2.89801
2.37887  2.77519  2.98518  4.58477  3.61503
          0.00949  5.05763  5.77998  0.61958  0.77255  0.48576  0.95510
     33   4.23413  5.41808  6.22843  5.66031  3.59755  5.69558  6.02962
2.04018  5.54428  0.49742  3.10125  5.86770  5.68843  5.38230  5.47800
5.10547  4.43986  2.17544  5.97068  5.03643     71 L - - -
          2.68618  4.42225  2.77519  2.73123  3.46354  2.40513  3.72494
3.29354  2.67741  2.69355  4.24690  2.90347  2.73739  3.18146  2.89801
2.37887  2.77519  2.98518  4.58477  3.61503
          0.00949  5.05763  5.77998  0.61958  0.77255  0.48576  0.95510
     34   2.70801  4.52047  3.24046  2.78177  3.97559  2.47909  3.95491
2.57742  2.72623  2.35737  3.81539  2.37300  4.02101  2.85375  3.18664
2.85499  3.09750  2.36747  5.43662  3.48590     72 l - - -
          2.68506  4.42432  2.77396  2.72982  3.46428  2.40543  3.72561
3.29498  2.67815  2.69344  4.24602  2.90302  2.73733  3.17864  2.89904
2.37926  2.77572  2.98560  4.58625  3.61694
          1.61342  0.92926  0.90153  2.56133  0.08034  0.48576  0.95510
     35   2.56536  5.17191  2.59137  2.29161  4.26418  3.36841  3.52346
3.96901  2.33299  2.91937  4.02514  2.82990  3.22768  2.16290  2.73064
2.53962  2.73501  3.36116  5.61674  4.21822    107 q - - -
          2.68618  4.42225  2.77519  2.73123  3.46354  2.40513  3.72494
3.29354  2.67741  2.69355  4.24690  2.90347  2.73739  3.18146  2.89801
2.37887  2.77519  2.98518  4.58477  3.61503
          0.01595  4.54202  5.26437  0.61958  0.77255  1.27575  0.32742
     36   2.35775  4.77214  3.09333  2.33606  3.37196  3.37248  3.60149
3.60557  2.10352  3.09955  3.78958  2.90195  3.50402  2.84709  2.86360
2.44290  2.85423  3.17069  5.43512  2.97293    108 k - - -
          2.68618  4.42225  2.77519  2.73123  3.46354  2.40513  3.72494
3.29354  2.67741  2.69355  4.24690  2.90347  2.73739  3.18146  2.89801
2.37887  2.77519  2.98518  4.58477  3.61503
          0.01595  4.54202  5.26437  0.61958  0.77255  1.27575  0.32742
     37   2.27701  5.19238  2.16417  2.36864  4.52347  3.48999  3.62371
3.90122  2.02732  3.41989  4.13420  2.75222  3.88503  2.57828  2.91201
2.37916  2.82106  3.57557  5.63318  4.11220    109 k - - -
          2.68618  4.42225  2.77519  2.73123  3.46354  2.40513  3.72494
3.29354  2.67741  2.69355  4.24690  2.90347  2.73739  3.18146  2.89801
2.37887  2.77519  2.98518  4.58477  3.61503
          0.01595  4.54202  5.26437  0.61958  0.77255  1.27575  0.32742
```

```
     38   2.70917  4.84471  3.03079  2.25547  3.38555  3.20144  3.57582
3.34977  2.54128  2.98593  2.44481  3.03792  3.73355  2.41286  2.97337
2.76257  2.78215  3.09133  5.36005  3.47123    110 e - - -
          2.68635  4.42242  2.77537  2.73137  3.46371  2.40530  3.72512
3.29350  2.67571  2.69372  4.24707  2.90358  2.73735  3.18143  2.89798
2.37904  2.77537  2.98536  4.58494  3.61520
          1.17025  0.37901  5.26437  0.06723  2.73301  0.19192  1.74509
     39   2.61830  4.54212  4.09039  3.52076  3.50506  3.97955  4.28927
2.76919  3.44835  2.75509  3.61685  2.37952  4.36772  3.71976  3.73364
3.09193  1.77957  1.46818  5.16969  3.95802    112 v - - -
          2.68619  4.42226  2.77521  2.73125  3.46355  2.40512  3.72496
3.29355  2.67736  2.69356  4.24691  2.90345  2.73741  3.18148  2.89794
2.37885  2.77521  2.98520  4.58478  3.61505
          0.04751  3.13940  5.77998  0.35095  1.21745  0.48576  0.95510
     40   2.87252  5.10072  3.11775  2.12980  4.67887  3.50106  3.36686
2.61031  1.79164  3.39579  3.93858  3.10231  3.83234  2.10957  2.85126
2.76834  3.04060  3.33460  5.80261  4.40577    114 k - - -
          2.68618  4.42225  2.77519  2.73123  3.46354  2.40513  3.72494
3.29354  2.67741  2.69355  4.24690  2.90347  2.73739  3.18146  2.89801
2.37887  2.77519  2.98518  4.58477  3.61503
          0.00949  5.05763  5.77998  0.61958  0.77255  0.48576  0.95510
     41   2.91516  3.87825  3.32776  2.64793  4.33308  3.74900  3.82031
2.69051  1.62772  3.28365  4.17017  3.16016  3.96241  3.01122  1.94887
2.95401  2.87395  2.80258  5.60169  4.09592    115 k - - -
          2.68618  4.42225  2.77519  2.73123  3.46354  2.40513  3.72494
3.29354  2.67741  2.69355  4.24690  2.90347  2.73739  3.18146  2.89801
2.37887  2.77519  2.98518  4.58477  3.61503
          0.00949  5.05763  5.77998  0.61958  0.77255  0.48576  0.95510
     42   4.15823  6.10343  4.85617  3.85153  5.74201  4.60394  3.78224
5.00101  2.11704  4.28418  5.20442  4.11218  4.90639  3.41625  0.40595
4.12315  4.21673  4.71745  6.17680  5.14892    116 R - - -
          2.68618  4.42225  2.77519  2.73123  3.46354  2.40513  3.72494
3.29354  2.67741  2.69355  4.24690  2.90347  2.73739  3.18146  2.89801
2.37887  2.77519  2.98518  4.58477  3.61503
          0.00949  5.05763  5.77998  0.61958  0.77255  0.48576  0.95510
     43   4.87263  6.35416  5.47779  4.90681  6.05478  4.94134  5.38523
5.83331  3.63574  5.12644  6.18497  5.14355  5.47330  4.64221  0.11986
4.97217  5.11796  5.51659  6.71074  5.87739    117 R - - -
          2.68618  4.42225  2.77519  2.73123  3.46354  2.40513  3.72494
3.29354  2.67741  2.69355  4.24690  2.90347  2.73739  3.18146  2.89801
2.37887  2.77519  2.98518  4.58477  3.61503
          0.00949  5.05763  5.77998  0.61958  0.77255  0.48576  0.95510
     44   3.47207  4.48264  5.47788  4.89751  3.17810  4.77360  5.16240
1.26413  4.72448  1.46971  2.69007  4.93744  5.05761  4.82595  4.74285
4.11540  3.55955  1.56175  5.54239  4.40567    118 i - - -
          2.68618  4.42225  2.77519  2.73123  3.46354  2.40513  3.72494
3.29354  2.67741  2.69355  4.24690  2.90347  2.73739  3.18146  2.89801
2.37887  2.77519  2.98518  4.58477  3.61503
          0.00949  5.05763  5.77998  0.61958  0.77255  0.48576  0.95510
     45   4.37334  5.84244  4.50727  4.40340  2.87834  4.84989  4.28544
4.61181  4.30874  3.89814  5.13761  3.80272  5.32401  3.91422  4.46524
4.37060  4.65571  4.45311  4.53865  0.31369    119 Y - - -
          2.68618  4.42225  2.77519  2.73123  3.46354  2.40513  3.72494
3.29354  2.67741  2.69355  4.24690  2.90347  2.73739  3.18146  2.89801
2.37887  2.77519  2.98518  4.58477  3.61503
          0.00949  5.05763  5.77998  0.61958  0.77255  0.48576  0.95510
```

```
      46   3.91407  6.69011  0.36761  2.47799  5.98869  3.90609  4.63074
5.61858  3.89022  5.04944  6.00486  3.35877  4.63212  3.82976  4.66113
3.30842  4.25837  5.11165  7.18965  5.52341    120 D - - -
           2.68618  4.42225  2.77519  2.73123  3.46354  2.40513  3.72494
3.29354  2.67741  2.69355  4.24690  2.90347  2.73739  3.18146  2.89801
2.37887  2.77519  2.98518  4.58477  3.61503
           0.00949  5.05763  5.77998  0.61958  0.77255  0.48576  0.95510
      47   2.11895  4.95025  5.73765  5.27152  4.38156  5.14938  5.93777
0.67663  5.19244  2.90379  4.14893  5.37389  5.47675  5.44218  5.34517
4.57150  3.96925  1.58832  6.36516  5.14383    121 I - - -
           2.68618  4.42225  2.77519  2.73123  3.46354  2.40513  3.72494
3.29354  2.67741  2.69355  4.24690  2.90347  2.73739  3.18146  2.89801
2.37887  2.77519  2.98518  4.58477  3.61503
           0.00949  5.05763  5.77998  0.61958  0.77255  0.48576  0.95510
      48   1.83275  4.38273  4.69636  4.09918  3.36733  3.66753  4.44719
2.26094  3.93680  1.76305  3.41538  4.19942  4.48410  4.11441  4.03252
3.42136  1.87586  2.05942  5.01968  3.83697    122 l - - -
           2.68618  4.42225  2.77519  2.73123  3.46354  2.40513  3.72494
3.29354  2.67741  2.69355  4.24690  2.90347  2.73739  3.18146  2.89801
2.37887  2.77519  2.98518  4.58477  3.61503
           0.00949  5.05763  5.77998  0.61958  0.77255  0.48576  0.95510
      49   3.29657  5.11056  2.91127  2.97483  5.28298  3.49580  4.50129
4.77919  3.33210  4.32680  5.14904  0.53624  4.43780  3.67777  3.95001
2.92816  3.66652  4.27968  6.49462  5.09200    123 N - - -
           2.68618  4.42225  2.77519  2.73123  3.46354  2.40513  3.72494
3.29354  2.67741  2.69355  4.24690  2.90347  2.73739  3.18146  2.89801
2.37887  2.77519  2.98518  4.58477  3.61503
           0.00949  5.05763  5.77998  0.61958  0.77255  0.48576  0.95510
      50   3.66640  5.05224  6.02576  5.59399  4.53501  5.57971  6.48096
1.40257  5.56918  2.83306  4.23023  5.76043  5.78439  5.86596  5.76614
5.04715  4.14394  0.53963  6.74872  5.49766    124 V - - -
           2.68618  4.42225  2.77519  2.73123  3.46354  2.40513  3.72494
3.29354  2.67741  2.69355  4.24690  2.90347  2.73739  3.18146  2.89801
2.37887  2.77519  2.98518  4.58477  3.61503
           0.00949  5.05763  5.77998  0.61958  0.77255  0.48576  0.95510
      51   4.53869  5.67917  6.45946  5.87488  2.65110  5.97684  6.10426
2.90954  5.75111  0.38661  2.34421  6.14073  5.81514  5.42640  5.60112
5.41201  4.71416  3.30537  5.89440  4.94445    125 L - - -
           2.68618  4.42225  2.77519  2.73123  3.46354  2.40513  3.72494
3.29354  2.67741  2.69355  4.24690  2.90347  2.73739  3.18146  2.89801
2.37887  2.77519  2.98518  4.58477  3.61503
           0.00949  5.05763  5.77998  0.61958  0.77255  0.48576  0.95510
      52   2.73291  4.08309  2.82172  1.49677  4.65944  3.68590  3.87611
3.62381  2.46289  3.50322  2.84992  3.04472  4.07868  2.41408  3.03878
2.45386  3.04507  3.63518  5.79217  4.21339    126 e - - -
           2.68618  4.42225  2.77519  2.73123  3.46354  2.40513  3.72494
3.29354  2.67741  2.69355  4.24690  2.90347  2.73739  3.18146  2.89801
2.37887  2.77519  2.98518  4.58477  3.61503
           0.00949  5.05763  5.77998  0.61958  0.77255  0.48576  0.95510
      53   1.41846  3.75048  4.72486  4.35707  5.23479  1.48928  5.22814
4.69025  4.30151  4.36270  5.14466  4.23431  4.10085  4.53422  4.54855
1.17597  2.99165  3.99971  6.57429  5.43033    127 s - - -
           2.68618  4.42225  2.77519  2.73123  3.46354  2.40513  3.72494
3.29354  2.67741  2.69355  4.24690  2.90347  2.73739  3.18146  2.89801
2.37887  2.77519  2.98518  4.58477  3.61503
           0.00949  5.05763  5.77998  0.61958  0.77255  0.48576  0.95510
```

```
     54   3.13115  4.34925  5.21298  4.61965  3.56684  4.48291  4.85074
1.45936  4.43126  1.35173  2.41454  4.63971  4.81151  4.56122  4.45621
3.53860  3.47598  1.78656  5.30451  4.14021    128 l - - -
          2.68618  4.42225  2.77519  2.73123  3.46354  2.40513  3.72494
3.29354  2.67741  2.69355  4.24690  2.90347  2.73739  3.18146  2.89801
2.37887  2.77519  2.98518  4.58477  3.61503
          0.00949  5.05763  5.77998  0.61958  0.77255  0.48576  0.95510
     55   2.89438  5.45528  2.55566  2.35456  4.79764  1.62771  3.35889
4.28504  2.42786  3.75911  4.49845  2.24042  4.09415  2.61194  2.94869
2.74531  3.13985  3.84481  5.88746  4.47250    129 g - - -
          2.68618  4.42225  2.77519  2.73123  3.46354  2.40513  3.72494
3.29354  2.67741  2.69355  4.24690  2.90347  2.73739  3.18146  2.89801
2.37887  2.77519  2.98518  4.58477  3.61503
          0.00949  5.05763  5.77998  0.61958  0.77255  0.48576  0.95510
     56   3.17670  4.29785  5.23394  4.62877  3.12358  4.48463  4.82401
1.49106  4.43108  1.07258  2.99130  4.64503  4.79972  4.52755  4.43606
3.80755  3.49918  2.17887  5.24345  3.66852    130 l - - -
          2.68618  4.42225  2.77519  2.73123  3.46354  2.40513  3.72494
3.29354  2.67741  2.69355  4.24690  2.90347  2.73739  3.18146  2.89801
2.37887  2.77519  2.98518  4.58477  3.61503
          0.00949  5.05763  5.77998  0.61958  0.77255  0.48576  0.95510
     57   3.24722  4.24745  5.65666  5.13290  4.15964  5.06016  5.60064
0.77269  5.01609  2.13575  3.68317  5.21664  5.33672  5.21427  5.11003
4.43675  3.65175  1.46959  6.01340  4.81399    131 i - - -
          2.68618  4.42225  2.77519  2.73123  3.46354  2.40513  3.72494
3.29354  2.67741  2.69355  4.24690  2.90347  2.73739  3.18146  2.89801
2.37887  2.77519  2.98518  4.58477  3.61503
          0.00949  5.05763  5.77998  0.61958  0.77255  0.48576  0.95510
     58   2.64485  5.40911  2.97131  1.71752  4.75353  3.12102  3.75464
4.23900  2.27496  3.71408  4.22459  2.97099  4.06746  2.61841  2.39816
2.20737  2.54877  3.68872  5.84215  4.33245    132 e - - -
          2.68618  4.42225  2.77519  2.73123  3.46354  2.40513  3.72494
3.29354  2.67741  2.69355  4.24690  2.90347  2.73739  3.18146  2.89801
2.37887  2.77519  2.98518  4.58477  3.61503
          0.00949  5.05763  5.77998  0.61958  0.77255  0.48576  0.95510
     59   3.95926  6.02370  4.56677  3.65504  5.66405  4.48351  3.96114
4.90821  0.56381  4.21571  5.10420  3.97590  4.79401  3.34949  1.71205
3.41872  4.05246  4.59606  6.14814  5.08549    133 K - - -
          2.68618  4.42225  2.77519  2.73123  3.46354  2.40513  3.72494
3.29354  2.67741  2.69355  4.24690  2.90347  2.73739  3.18146  2.89801
2.37887  2.77519  2.98518  4.58477  3.61503
          0.00949  5.05763  5.77998  0.61958  0.77255  0.48576  0.95510
     60   2.86613  4.90842  2.60692  1.97807  4.52154  3.57983  3.77595
3.31749  2.10649  3.29924  3.90858  3.01451  3.99343  2.94238  2.31444
2.79079  2.70888  2.55279  4.89678  4.22280    134 e - - -
          2.68618  4.42225  2.77519  2.73123  3.46354  2.40513  3.72494
3.29354  2.67741  2.69355  4.24690  2.90347  2.73739  3.18146  2.89801
2.37887  2.77519  2.98518  4.58477  3.61503
          0.14897  5.05763  2.02465  0.61958  0.77255  0.48576  0.95510
     61   2.41580  4.89706  2.91218  2.50154  4.65125  2.65880  2.94524
4.12746  2.49629  3.53775  4.36456  2.47208  3.37688  2.83149  2.90004
1.88725  2.80219  3.66125  5.76135  3.86731    135 s - - -
          2.68629  4.42282  2.77560  2.73136  3.46411  2.40489  3.72473
3.29337  2.67753  2.69384  4.24676  2.90374  2.73786  3.18179  2.89728
2.37849  2.77476  2.98444  4.58534  3.61560
          0.32175  1.33643  4.39508  1.67948  0.20637  0.33972  1.24468
```

```
     62   3.06872  5.51595  3.22119  2.71694  4.88267  3.86321  3.98073
4.31447  0.82414  3.79027  4.57267  3.34442  4.25155  3.03342  2.56426
2.99590  3.29992  3.30263  5.90233  4.57464    144 k - - -
          2.68623  4.42230  2.77525  2.73129  3.46359  2.40518  3.72500
3.29346  2.67726  2.69360  4.24695  2.90352  2.73745  3.18133  2.89783
2.37886  2.77525  2.98502  4.58482  3.61508
          0.07326  3.00111  3.86748  0.90911  0.51564  0.46365  0.99151
     63   2.92544  5.42015  3.06956  2.53412  4.75799  3.59159  3.88179
4.23903  2.04604  3.72150  4.46516  1.60889  2.32173  2.67408  3.11156
2.56406  2.93889  3.80741  5.85583  4.31691    148 n - - -
          2.68618  4.42225  2.77519  2.73123  3.46354  2.40513  3.72494
3.29354  2.67741  2.69355  4.24690  2.90347  2.73739  3.18146  2.89801
2.37887  2.77519  2.98518  4.58477  3.61503
          0.01671  5.03991  4.59570  0.61958  0.77255  0.53689  0.87842
     64   2.38538  4.25405  2.95280  2.17583  4.70924  3.40760  2.63611
4.09067  2.21380  3.45365  4.19934  2.90673  4.06056  2.62981  2.78180
2.34601  2.68179  3.23016  5.81385  3.54144    149 e - - -
          2.68618  4.42225  2.77519  2.73123  3.46354  2.40513  3.72494
3.29354  2.67741  2.69355  4.24690  2.90347  2.73739  3.18146  2.89801
2.37887  2.77519  2.98518  4.58477  3.61503
          0.00973  5.03294  5.75528  0.61958  0.77255  0.52347  0.89762
     65   3.08779  4.50658  5.08173  4.48463  1.91189  4.34312  4.62647
1.44678  4.28553  2.30681  3.42300  4.48432  4.68319  4.40399  4.30228
3.66153  3.38346  2.07414  5.06261  1.99450    150 i - - -
          2.68618  4.42225  2.77519  2.73123  3.46354  2.40513  3.72494
3.29354  2.67741  2.69355  4.24690  2.90347  2.73739  3.18146  2.89801
2.37887  2.77519  2.98518  4.58477  3.61503
          0.02440  5.03991  4.03818  0.61958  0.77255  0.45507  1.00623
     66   2.31863  4.90928  3.18174  2.43211  4.30626  3.63883  3.73054
3.75799  1.96835  3.29869  4.20798  3.16689  4.07647  2.30166  1.95878
2.66036  2.87422  3.30642  5.77030  3.91689    151 r - - -
          2.68618  4.42225  2.77519  2.73123  3.46354  2.40513  3.72494
3.29354  2.67741  2.69355  4.24690  2.90347  2.73739  3.18146  2.89801
2.37887  2.77519  2.98518  4.58477  3.61503
          0.00963  5.04330  5.76564  0.61958  0.77255  0.52739  0.89194
     67   4.59432  4.32022  5.71165  5.49498  2.43252  5.29215  4.19681
4.26901  5.26542  3.57529  4.84329  4.95943  5.58302  5.07778  5.15797
4.67495  4.81084  4.18156  0.44012  2.23703    152 W - - -
          2.68627  4.42234  2.77528  2.73132  3.46363  2.40492  3.72503
3.29342  2.67749  2.69364  4.24698  2.90355  2.73748  3.18155  2.89809
2.37895  2.77528  2.98474  4.58486  3.61412
          0.11935  2.44287  3.66539  0.84857  0.55867  0.52739  0.89194
     68   2.91201  4.19561  4.30768  3.62709  3.22478  4.00300  3.17412
1.67682  2.90720  2.04031  3.29322  3.11980  4.37348  3.63496  3.29010
3.19216  2.93107  2.06470  4.86710  3.42480    156 i - - -
          2.68618  4.42225  2.77519  2.73123  3.46354  2.40513  3.72494
3.29354  2.67741  2.69355  4.24690  2.90347  2.73739  3.18146  2.89801
2.37887  2.77519  2.98518  4.58477  3.61503
          0.01200  5.02058  5.23441  0.61958  0.77255  0.56112  0.84529
     69   2.82881  4.99225  3.47412  3.03969  4.40338  0.90439  4.19621
3.80640  3.00961  3.25558  4.06048  3.38276  4.24793  3.29907  3.40099
2.40254  3.25714  3.43912  5.72144  4.34177    157 g - - -
          2.68618  4.42225  2.77519  2.73123  3.46354  2.40513  3.72494
3.29354  2.67741  2.69355  4.24690  2.90347  2.73739  3.18146  2.89801
2.37887  2.77519  2.98518  4.58477  3.61503
          0.00662  5.02127        *  0.61958  0.77255  0.00000        *
//
HMMER3/f [3.3 | Nov 2019]
```

```
NAME  EIN3
ACC   PF04873.14
DESC  Ethylene insensitive 3
LENG  253
ALPH  amino
RF    no
MM    no
CONS  yes
CS    yes
MAP   yes
DATE  Fri Apr 17 05:34:01 2020
NSEQ  10
EFFN  0.786133
CKSUM 1042994250
GA    27.00 27.00
TC    27.90 27.40
NC    23.40 23.20
STATS LOCAL MSV       -11.0204  0.70281
STATS LOCAL VITERBI   -11.7808  0.70281
STATS LOCAL FORWARD    -5.2715  0.70281
HMM          A        C        D        E        F        G        H
I        K        L        M        N        P        Q        R        S
T        V        W        Y
            m->m     m->i     m->d     i->m     i->i     d->m     d->d
  COMPO   2.56203  4.32702  2.90142  2.65028  3.57115  2.87266  3.67540
3.00358  2.49398  2.50562  3.58819  3.14243  3.10280  2.94715  2.77587
2.63977  2.94196  2.76754  4.01832  3.69817
          2.68618  4.42225  2.77519  2.73123  3.46354  2.40513  3.72494
3.29354  2.67741  2.69355  4.24690  2.90347  2.73739  3.18146  2.89801
2.37887  2.77519  2.98518  4.58477  3.61503
          0.02580  4.06597  4.78831  0.61958  0.77255  0.00000        *
      1   2.48277  5.12258  2.28649  1.82022  4.43760  3.34220  3.62459
3.90500  2.41655  3.41972  4.19768  2.82209  3.79893  2.74745  2.50737
2.45754  2.92574  3.50164  5.58340  4.17851      1 e - - X
          2.68618  4.42225  2.77519  2.73123  3.46354  2.40513  3.72494
3.29354  2.67741  2.69355  4.24690  2.90347  2.73739  3.18146  2.89801
2.37887  2.77519  2.98518  4.58477  3.61503
          0.02580  4.06597  4.78831  0.61958  0.77255  0.48576  0.95510
      2   3.01225  5.60602  1.32129  1.50728  4.88993  3.25338  3.81602
4.41832  2.85536  3.91286  4.77382  2.71446  3.88637  2.98531  3.46533
2.88546  3.30492  3.98519  6.06004  4.54343      2 d - - X
          2.68618  4.42225  2.77519  2.73123  3.46354  2.40513  3.72494
3.29354  2.67741  2.69355  4.24690  2.90347  2.73739  3.18146  2.89801
2.37887  2.77519  2.98518  4.58477  3.61503
          0.02580  4.06597  4.78831  0.61958  0.77255  0.48576  0.95510
      3   2.87976  2.88119  4.41686  3.90612  3.27024  3.90916  4.47092
2.28211  3.71172  1.09622  3.21641  4.07115  4.35099  3.97709  3.87971
3.30355  3.17696  2.20954  5.05677  3.84267      3 l - - X
          2.68618  4.42225  2.77519  2.73123  3.46354  2.40513  3.72494
3.29354  2.67741  2.69355  4.24690  2.90347  2.73739  3.18146  2.89801
2.37887  2.77519  2.98518  4.58477  3.61503
          0.02580  4.06597  4.78831  0.61958  0.77255  0.48576  0.95510
      4   2.51652  5.00469  2.78217  1.95132  4.33336  3.37578  3.63034
3.76974  1.94725  3.31391  4.10906  2.88783  3.81644  2.76133  2.80798
2.33444  2.74230  3.39493  5.49463  4.13239      4 k - - X
          2.68618  4.42225  2.77519  2.73123  3.46354  2.40513  3.72494
3.29354  2.67741  2.69355  4.24690  2.90347  2.73739  3.18146  2.89801
2.37887  2.77519  2.98518  4.58477  3.61503
```

```
          0.02580  4.06597  4.78831  0.61958  0.77255  0.48576  0.95510
      5   2.40948  5.07484  3.19775  2.60008  4.45447  3.55679  3.61387
3.83845  1.58507  3.33400  4.16014  3.06893  3.93769  2.57024  1.92222
2.84622  3.04889  3.49532  5.44946  4.19525      5 k - - X
          2.68618  4.42225  2.77519  2.73123  3.46354  2.40513  3.72494
3.29354  2.67741  2.69355  4.24690  2.90347  2.73739  3.18146  2.89801
2.37887  2.77519  2.98518  4.58477  3.61503
          0.02580  4.06597  4.78831  0.61958  0.77255  0.48576  0.95510
      6   2.79663  5.12110  2.41505  2.43064  4.46743  3.44764  3.63576
3.89741  2.06574  3.40189  4.21361  2.94627  3.88224  2.76644  1.63303
2.77195  3.02089  3.52774  5.53079  4.20676      6 r - - X
          2.68618  4.42225  2.77519  2.73123  3.46354  2.40513  3.72494
3.29354  2.67741  2.69355  4.24690  2.90347  2.73739  3.18146  2.89801
2.37887  2.77519  2.98518  4.58477  3.61503
          0.02580  4.06597  4.78831  0.61958  0.77255  0.48576  0.95510
      7   3.23847  4.60072  4.72937  4.16408  3.12532  4.45846  4.75728
1.96969  4.00878  1.34643  1.56538  4.43811  4.67503  4.17514  4.16247
3.79462  3.46343  2.23891  5.15643  4.06304      7 l - - X
          2.68618  4.42225  2.77519  2.73123  3.46354  2.40513  3.72494
3.29354  2.67741  2.69355  4.24690  2.90347  2.73739  3.18146  2.89801
2.37887  2.77519  2.98518  4.58477  3.61503
          0.02580  4.06597  4.78831  0.61958  0.77255  0.48576  0.95510
      8   2.76599  4.46883  3.71731  3.30783  2.91383  3.55426  3.84807
3.17714  3.10244  2.78859  3.79695  3.54307  4.08550  3.49548  3.34451
2.29578  3.09278  2.94151  1.48500  2.93273      8 w - - X
          2.68618  4.42225  2.77519  2.73123  3.46354  2.40513  3.72494
3.29354  2.67741  2.69355  4.24690  2.90347  2.73739  3.18146  2.89801
2.37887  2.77519  2.98518  4.58477  3.61503
          0.02580  4.06597  4.78831  0.61958  0.77255  0.48576  0.95510
      9   3.11866  5.22743  3.55503  2.85759  4.70539  3.72154  3.63839
4.04231  1.28087  3.46534  4.33823  3.24887  4.08207  2.77092  1.49988
3.10925  3.27584  3.72386  5.48940  4.33716      9 k - - X
          2.68618  4.42225  2.77519  2.73123  3.46354  2.40513  3.72494
3.29354  2.67741  2.69355  4.24690  2.90347  2.73739  3.18146  2.89801
2.37887  2.77519  2.98518  4.58477  3.61503
          0.02580  4.06597  4.78831  0.61958  0.77255  0.48576  0.95510
     10   2.97911  5.47428  1.12493  2.16121  4.79286  3.24241  3.84258
4.36819  2.89351  3.88675  4.75630  2.18191  3.88744  3.02333  3.49079
2.87687  3.29106  3.93259  6.00601  4.49849     10 d - - X
          2.68618  4.42225  2.77519  2.73123  3.46354  2.40513  3.72494
3.29354  2.67741  2.69355  4.24690  2.90347  2.73739  3.18146  2.89801
2.37887  2.77519  2.98518  4.58477  3.61503
          0.02580  4.06597  4.78831  0.61958  0.77255  0.48576  0.95510
     11   2.82667  5.10832  3.11053  2.54181  4.48806  2.80832  3.60549
3.88817  1.78251  3.37081  4.18398  3.02672  3.91512  2.30875  1.89908
2.81151  3.02996  3.52840  5.47827  4.20255     11 k - - X
          2.68618  4.42225  2.77519  2.73123  3.46354  2.40513  3.72494
3.29354  2.67741  2.69355  4.24690  2.90347  2.73739  3.18146  2.89801
2.37887  2.77519  2.98518  4.58477  3.61503
          0.02580  4.06597  4.78831  0.61958  0.77255  0.48576  0.95510
     12   2.59813  4.42922  3.31190  2.75263  3.57433  3.52546  3.76396
2.73346  2.68149  2.60026  2.60195  2.84691  3.91111  2.44645  3.05881
2.76665  2.83120  2.44092  4.99713  3.74157     12 v - - X
          2.68618  4.42225  2.77519  2.73123  3.46354  2.40513  3.72494
3.29354  2.67741  2.69355  4.24690  2.90347  2.73739  3.18146  2.89801
2.37887  2.77519  2.98518  4.58477  3.61503
          0.02580  4.06597  4.78831  0.61958  0.77255  0.48576  0.95510
```

```
     13   2.35269  4.66774  3.26286  2.68135  3.89248  3.52984  3.69254
3.24253  2.05673  2.14908  3.76526  3.13556  3.91782  2.88873  2.33311
2.79314  2.91910  2.97846  5.18117  3.92521     13 k - - X
          2.68618  4.42225  2.77519  2.73123  3.46354  2.40513  3.72494
3.29354  2.67741  2.69355  4.24690  2.90347  2.73739  3.18146  2.89801
2.37887  2.77519  2.98518  4.58477  3.61503
          0.02580  4.06597  4.78831  0.61958  0.77255  0.48576  0.95510
     14   2.90195  3.01289  4.44098  3.87158  3.17116  4.04489  4.41255
2.23070  3.71724  1.21039  2.62027  4.07880  4.37943  3.92546  3.87821
3.36959  3.14685  2.18356  4.96495  3.80650     14 l - - X
          2.68618  4.42225  2.77519  2.73123  3.46354  2.40513  3.72494
3.29354  2.67741  2.69355  4.24690  2.90347  2.73739  3.18146  2.89801
2.37887  2.77519  2.98518  4.58477  3.61503
          0.02580  4.06597  4.78831  0.61958  0.77255  0.48576  0.95510
     15   2.59550  3.58326  3.20911  2.64142  3.74984  2.90505  3.70283
3.12483  2.02466  2.79202  2.95419  3.10679  3.86995  2.92049  2.75491
2.71262  2.83083  2.85832  5.10048  3.82948     15 k - - X
          2.68618  4.42225  2.77519  2.73123  3.46354  2.40513  3.72494
3.29354  2.67741  2.69355  4.24690  2.90347  2.73739  3.18146  2.89801
2.37887  2.77519  2.98518  4.58477  3.61503
          0.02580  4.06597  4.78831  0.61958  0.77255  0.48576  0.95510
     16   2.88336  5.08924  3.24176  2.64019  4.49486  3.57291  3.62383
3.87572  1.64999  3.36110  4.19408  3.09723  2.93503  2.75461  1.67688
2.88139  3.08221  3.53258  5.46123  4.22203     16 k - - X
          2.68618  4.42225  2.77519  2.73123  3.46354  2.40513  3.72494
3.29354  2.67741  2.69355  4.24690  2.90347  2.73739  3.18146  2.89801
2.37887  2.77519  2.98518  4.58477  3.61503
          0.02580  4.06597  4.78831  0.61958  0.77255  0.48576  0.95510
     17   3.22011  4.53245  4.87581  4.33057  3.28509  4.53828  4.93436
1.65180  4.20574  1.15363  3.07956  4.57121  4.76466  4.37001  4.35489
3.89528  3.45739  1.66565  5.31825  4.20546     17 l - - X
          2.68618  4.42225  2.77519  2.73123  3.46354  2.40513  3.72494
3.29354  2.67741  2.69355  4.24690  2.90347  2.73739  3.18146  2.89801
2.37887  2.77519  2.98518  4.58477  3.61503
          0.02580  4.06597  4.78831  0.61958  0.77255  0.48576  0.95510
     18   2.36167  5.06907  2.80767  2.08663  4.42174  3.42222  3.65682
3.82376  1.58170  3.36682  4.18721  2.93798  3.87489  2.79458  2.66250
2.76073  3.01088  3.46414  5.52989  4.20182     18 k - - X
          2.68618  4.42225  2.77519  2.73123  3.46354  2.40513  3.72494
3.29354  2.67741  2.69355  4.24690  2.90347  2.73739  3.18146  2.89801
2.37887  2.77519  2.98518  4.58477  3.61503
          0.02580  4.06597  4.78831  0.61958  0.77255  0.48576  0.95510
     19   2.81043  5.19711  2.46445  1.62086  4.53381  2.17815  3.72098
4.00001  2.55624  3.53056  4.35245  2.82042  3.85332  2.48918  3.03146
2.76293  3.07643  3.60955  5.70308  4.29885     19 e - - X
          2.68618  4.42225  2.77519  2.73123  3.46354  2.40513  3.72494
3.29354  2.67741  2.69355  4.24690  2.90347  2.73739  3.18146  2.89801
2.37887  2.77519  2.98518  4.58477  3.61503
          0.02580  4.06597  4.78831  0.61958  0.77255  0.48576  0.95510
     20   2.63022  4.84386  3.01145  2.45074  4.10586  3.10872  3.61364
3.52122  2.32978  2.92766  3.38339  2.59378  3.82597  2.42163  2.25474
2.65571  2.85694  3.19224  5.32336  3.99932     20 r - - X
          2.68618  4.42225  2.77519  2.73123  3.46354  2.40513  3.72494
3.29354  2.67741  2.69355  4.24690  2.90347  2.73739  3.18146  2.89801
2.37887  2.77519  2.98518  4.58477  3.61503
          0.02580  4.06597  4.78831  0.61958  0.77255  0.48576  0.95510
```

```
     21   2.43438  4.77996  2.99279  2.35307  3.22799  3.42192  3.62236
3.43169  2.24292  3.03662  3.85446  2.95712  3.81385  2.42757  2.66148
2.47867  2.69770  3.11333  5.28072  3.95412     21 k - - X
          2.68618  4.42225  2.77519  2.73123  3.46354  2.40513  3.72494
3.29354  2.67741  2.69355  4.24690  2.90347  2.73739  3.18146  2.89801
2.37887  2.77519  2.98518  4.58477  3.61503
          0.02580  4.06597  4.78831  0.61958  0.77255  0.48576  0.95510
     22   2.70993  4.99665  3.03887  2.46732  4.31984  3.08302  3.59762
3.73655  1.75073  3.03446  4.06598  2.97258  3.34403  2.56633  2.24122
2.70776  2.92674  3.38071  5.42137  4.10786     22 k - - X
          2.68618  4.42225  2.77519  2.73123  3.46354  2.40513  3.72494
3.29354  2.67741  2.69355  4.24690  2.90347  2.73739  3.18146  2.89801
2.37887  2.77519  2.98518  4.58477  3.61503
          0.06102  4.06597  3.16886  0.61958  0.77255  0.48576  0.95510
     23   2.52298  5.07711  2.28136  2.26486  4.38314  2.55473  3.16953
3.84627  2.30491  3.37475  4.15869  2.57181  3.78890  2.74867  2.92781
2.50013  2.91032  3.45322  5.55155  4.15127     23 e - - X
          2.68618  4.42225  2.77519  2.73123  3.46354  2.40513  3.72494
3.29354  2.67741  2.69355  4.24690  2.90347  2.73739  3.18146  2.89801
2.37887  2.77519  2.98518  4.58477  3.61503
          0.10853  4.03166  2.46389  0.61958  0.77255  0.52245  0.89910
     24   2.64567  4.96763  2.71244  2.16467  4.27922  2.68646  3.60678
3.71883  2.25698  3.26851  4.06921  2.54689  3.78639  2.74313  2.64976
2.30209  2.88821  3.35107  5.45849  4.09523     24 e - - .
          2.68618  4.42225  2.77519  2.73123  3.46354  2.40513  3.72494
3.29354  2.67741  2.69355  4.24690  2.90347  2.73739  3.18146  2.89801
2.37887  2.77519  2.98518  4.58477  3.61503
          0.02896  3.95209  4.67444  0.61958  0.77255  0.47593  0.97102
     25   2.56937  3.66419  3.68315  3.12600  3.24303  2.67758  3.88180
2.64714  3.03292  1.89036  3.31686  3.44566  3.97747  3.32365  3.31739
2.85630  2.81901  2.44160  4.74689  2.84176     25 l - - .
          2.68618  4.42225  2.77519  2.73123  3.46354  2.40513  3.72494
3.29354  2.67741  2.69355  4.24690  2.90347  2.73739  3.18146  2.89801
2.37887  2.77519  2.98518  4.58477  3.61503
          0.13826  4.03006  2.19501  0.61958  0.77255  0.46251  0.99344
     26   2.07912  4.44481  3.15363  2.69019  3.79998  2.45773  3.77971
3.13130  2.47965  2.39996  3.71832  3.11672  3.82626  3.01322  3.01062
2.65165  2.80979  2.84337  5.16767  3.90065     26 a - - X
          2.68618  4.42225  2.77519  2.73123  3.46354  2.40513  3.72494
3.29354  2.67741  2.69355  4.24690  2.90347  2.73739  3.18146  2.89801
2.37887  2.77519  2.98518  4.58477  3.61503
          0.02868  3.96160  4.68395  0.61958  0.77255  0.58996  0.80822
     27   2.09939  4.48412  2.86341  2.59868  3.83288  2.95665  3.73638
3.20378  2.62252  2.46565  3.73430  3.05281  3.79671  2.94964  3.04394
2.46543  2.52952  2.90010  5.18495  3.90103     27 a - - X
          2.68618  4.42225  2.77519  2.73123  3.46354  2.40513  3.72494
3.29354  2.67741  2.69355  4.24690  2.90347  2.73739  3.18146  2.89801
2.37887  2.77519  2.98518  4.58477  3.61503
          0.02868  3.96160  4.68395  0.61958  0.77255  0.42572  1.05929
     28   2.35642  4.79723  3.00342  2.45028  4.05069  3.42488  3.62251
3.46276  2.37774  3.06106  3.14413  2.69039  3.49297  2.41639  2.45144
2.64317  2.83914  3.14040  5.29715  3.97287     28 a - - X
          2.68618  4.42225  2.77519  2.73123  3.46354  2.40513  3.72494
3.29354  2.67741  2.69355  4.24690  2.90347  2.73739  3.18146  2.89801
2.37887  2.77519  2.98518  4.58477  3.61503
          0.02580  4.06597  4.78831  0.61958  0.77255  0.48576  0.95510
```

```
     29   2.27836  4.51787  3.15038  2.63426  3.79796  2.85348  3.73956
3.17791  2.60985  2.30300  3.70375  3.09349  3.84367  2.61885  3.01885
2.42863  2.66256  2.89200  5.15449  3.87629     29 a - - X
          2.68618  4.42225  2.77519  2.73123  3.46354  2.40513  3.72494
3.29354  2.67741  2.69355  4.24690  2.90347  2.73739  3.18146  2.89801
2.37887  2.77519  2.98518  4.58477  3.61503
          0.08382  4.06597  2.76059  0.61958  0.77255  0.48576  0.95510
     30   2.21329  4.64668  2.97617  2.37994  3.95942  2.82754  3.68188
3.36017  2.51580  2.99393  3.82751  2.98621  3.80287  2.63055  2.95528
2.31322  2.81699  2.70699  5.26282  3.95284     30 a - - X
          2.68618  4.42225  2.77519  2.73123  3.46354  2.40513  3.72494
3.29354  2.67741  2.69355  4.24690  2.90347  2.73739  3.18146  2.89801
2.37887  2.77519  2.98518  4.58477  3.61503
          0.18529  4.00947  1.89049  0.61958  0.77255  0.54485  0.86733
     31   2.10065  4.54944  2.39367  2.44249  4.22694  2.46355  3.78122
3.65563  2.69276  3.27586  4.09832  2.90429  3.70174  2.96703  3.15214
2.08187  2.79846  3.22686  5.50642  4.17757     31 s - - .
          2.68608  4.42238  2.77533  2.73136  3.46367  2.40485  3.72474
3.29367  2.67754  2.69335  4.24703  2.90360  2.73753  3.18160  2.89799
2.37900  2.77533  2.98448  4.58490  3.61516
          0.36636  1.21579  4.57845  1.14564  0.38276  0.38281  1.14553
     32   2.41332  4.18862  3.63271  3.06020  2.80421  3.30984  3.86342
2.43022  2.98453  2.06518  3.29005  3.40988  3.96874  3.05042  2.97937
2.85338  2.80125  2.34152  4.78332  3.56654     36 l - - X
          2.68618  4.42225  2.77519  2.73123  3.46354  2.40513  3.72494
3.29354  2.67741  2.69355  4.24690  2.90347  2.73739  3.18146  2.89801
2.37887  2.77519  2.98518  4.58477  3.61503
          0.02580  4.06597  4.78831  0.61958  0.77255  0.48576  0.95510
     33   2.63801  4.93434  2.47797  2.14438  4.23051  2.41507  3.64445
3.66633  2.46071  2.90082  4.04225  2.87695  3.80326  2.78591  2.94439
2.40656  2.71848  3.30719  5.45351  4.08556     37 e - - X
          2.68618  4.42225  2.77519  2.73123  3.46354  2.40513  3.72494
3.29354  2.67741  2.69355  4.24690  2.90347  2.73739  3.18146  2.89801
2.37887  2.77519  2.98518  4.58477  3.61503
          0.02580  4.06597  4.78831  0.61958  0.77255  0.48576  0.95510
     34   2.43163  4.00436  3.09373  2.29646  3.82140  3.44211  3.66920
2.77748  2.27582  2.86230  3.70687  3.03128  3.51217  2.86182  2.94985
2.45878  2.68319  2.92661  5.15533  3.85829     38 k - - X
          2.68618  4.42225  2.77519  2.73123  3.46354  2.40513  3.72494
3.29354  2.67741  2.69355  4.24690  2.90347  2.73739  3.18146  2.89801
2.37887  2.77519  2.98518  4.58477  3.61503
          0.02580  4.06597  4.78831  0.61958  0.77255  0.48576  0.95510
     35   2.59903  4.78647  2.92957  2.20503  4.04080  2.90961  3.63750
2.90990  2.44225  3.06181  3.87999  2.94371  3.25359  2.58764  2.89896
2.31459  2.83716  3.13200  5.30772  3.97488     39 e - - X
          2.68618  4.42225  2.77519  2.73123  3.46354  2.40513  3.72494
3.29354  2.67741  2.69355  4.24690  2.90347  2.73739  3.18146  2.89801
2.37887  2.77519  2.98518  4.58477  3.61503
          0.12272  4.06597  2.31928  0.61958  0.77255  0.48576  0.95510
     36   2.66920  4.96595  2.82465  2.21575  4.27395  3.38583  3.59862
3.69989  1.93204  3.24654  4.05173  2.89374  3.81059  2.50119  2.69387
2.41086  2.51389  3.34271  5.42812  4.08815     40 k - - X
          2.68618  4.42225  2.77519  2.73123  3.46354  2.40513  3.72494
3.29354  2.67741  2.69355  4.24690  2.90347  2.73739  3.18146  2.89801
2.37887  2.77519  2.98518  4.58477  3.61503
          0.02839  3.97164  4.69398  0.61958  0.77255  0.58084  0.81968
```

```
     37   2.38590  4.71008  2.94019  2.45018  4.02985  3.34903  3.16594
3.44303  2.33145  3.05316  3.87584  2.95054  2.94155  2.80975  2.87558
2.23757  2.64470  3.11150  5.29791  3.97720     41 s - - X
          2.68618  4.42225  2.77519  2.73123  3.46354  2.40513  3.72494
3.29354  2.67741  2.69355  4.24690  2.90347  2.73739  3.18146  2.89801
2.37887  2.77519  2.98518  4.58477  3.61503
          0.02839  3.97164  4.69398  0.61958  0.77255  0.58084  0.81968
     38   2.44238  4.95649  2.58010  2.32455  4.25311  3.35953  3.29270
3.69602  2.06646  3.24131  4.03030  2.85929  3.77742  2.70979  2.64603
2.25530  2.84963  3.32559  5.42706  4.06140     42 k - - X
          2.68624  4.42247  2.77541  2.73058  3.46375  2.40525  3.72516
3.29347  2.67762  2.69365  4.24711  2.90368  2.73761  3.18149  2.89822
2.37817  2.77524  2.98498  4.58498  3.61525
          0.23360  1.61359  4.69398  1.76242  0.18829  0.43059  1.05018
     39   2.62521  5.06037  2.61009  2.30991  4.38105  3.37388  3.57700
3.84424  2.14077  3.35075  4.11401  2.52524  3.34297  2.37155  2.54925
2.59732  2.66590  3.43920  5.50361  4.11531     56 k - - X
          2.68618  4.42225  2.77519  2.73123  3.46354  2.40513  3.72494
3.29354  2.67741  2.69355  4.24690  2.90347  2.73739  3.18146  2.89801
2.37887  2.77519  2.98518  4.58477  3.61503
          0.02580  4.06597  4.78831  0.61958  0.77255  0.48576  0.95510
     40   2.57084  4.28618  3.46157  2.89460  3.05671  3.54929  3.80854
2.35131  2.83522  2.48589  3.12759  3.29532  2.91447  3.14484  2.84066
2.54254  2.80458  2.54991  4.87055  3.63550     57 i - - X
          2.68618  4.42225  2.77519  2.73123  3.46354  2.40513  3.72494
3.29354  2.67741  2.69355  4.24690  2.90347  2.73739  3.18146  2.89801
2.37887  2.77519  2.98518  4.58477  3.61503
          0.02580  4.06597  4.78831  0.61958  0.77255  0.48576  0.95510
     41   2.59442  4.79057  2.93770  2.17462  4.05794  3.39516  3.63594
3.47229  2.43348  3.07610  3.89156  2.94324  3.31886  2.53986  2.88906
2.30642  2.58655  2.71742  5.31786  3.98372     58 e - - X
          2.68618  4.42225  2.77519  2.73123  3.46354  2.40513  3.72494
3.29354  2.67741  2.69355  4.24690  2.90347  2.73739  3.18146  2.89801
2.37887  2.77519  2.98518  4.58477  3.61503
          0.02580  4.06597  4.78831  0.61958  0.77255  0.48576  0.95510
     42   2.28658  5.15066  2.31308  1.58172  4.50190  2.63678  3.71304
3.96403  2.59825  3.50678  4.31569  2.80317  3.82662  2.86025  3.11961
2.71332  3.02628  3.56499  5.69488  4.27667     59 e - - X
          2.68618  4.42225  2.77519  2.73123  3.46354  2.40513  3.72494
3.29354  2.67741  2.69355  4.24690  2.90347  2.73739  3.18146  2.89801
2.37887  2.77519  2.98518  4.58477  3.61503
          0.02580  4.06597  4.78831  0.61958  0.77255  0.48576  0.95510
     43   2.44797  5.01326  2.92693  2.40891  4.32580  3.43075  3.14819
3.76193  2.25377  3.28883  4.08344  2.53753  3.83377  2.15856  2.31281
2.67793  2.91237  3.39469  5.44829  4.10848     60 q - - X
          2.68618  4.42225  2.77519  2.73123  3.46354  2.40513  3.72494
3.29354  2.67741  2.69355  4.24690  2.90347  2.73739  3.18146  2.89801
2.37887  2.77519  2.98518  4.58477  3.61503
          0.02580  4.06597  4.78831  0.61958  0.77255  0.48576  0.95510
     44   2.04778  4.27996  3.52936  2.98959  2.72336  3.54059  3.82471
2.79992  2.93871  2.51291  3.42747  3.35286  3.95570  3.23277  3.26843
2.34165  2.83622  2.57639  4.75846  2.76949     61 a - - X
          2.68618  4.42225  2.77519  2.73123  3.46354  2.40513  3.72494
3.29354  2.67741  2.69355  4.24690  2.90347  2.73739  3.18146  2.89801
2.37887  2.77519  2.98518  4.58477  3.61503
          0.02580  4.06597  4.78831  0.61958  0.77255  0.48576  0.95510
```

```
     45   2.66067  4.78925  3.08509  2.53839  4.07715  2.84270  3.65189
3.48049  2.32682  2.69658  3.91052  3.02544  3.86024  2.65247  1.99233
2.49026  2.89493  3.16498  5.30265  4.00679      62 r - - X
          2.68618  4.42225  2.77519  2.73123  3.46354  2.40513  3.72494
3.29354  2.67741  2.69355  4.24690  2.90347  2.73739  3.18146  2.89801
2.37887  2.77519  2.98518  4.58477  3.61503
          0.02580  4.06597  4.78831  0.61958  0.77255  0.48576  0.95510
     46   2.88321  4.99054  3.32956  2.72268  4.39921  3.58092  3.65578
3.75229  1.90191  3.28348  4.13922  3.15394  3.97915  2.80130  1.45134
2.91119  2.51252  3.43297  5.41824  4.19931      63 r - - X
          2.68618  4.42225  2.77519  2.73123  3.46354  2.40513  3.72494
3.29354  2.67741  2.69355  4.24690  2.90347  2.73739  3.18146  2.89801
2.37887  2.77519  2.98518  4.58477  3.61503
          0.02580  4.06597  4.78831  0.61958  0.77255  0.48576  0.95510
     47   2.82690  4.91832  3.29078  2.68665  4.24184  3.56841  3.65846
3.57530  1.41757  3.16287  4.03087  3.13379  3.95765  2.81194  2.20310
2.86787  3.03761  2.65348  5.35841  4.11412      64 k - - X
          2.68618  4.42225  2.77519  2.73123  3.46354  2.40513  3.72494
3.29354  2.67741  2.69355  4.24690  2.90347  2.73739  3.18146  2.89801
2.37887  2.77519  2.98518  4.58477  3.61503
          0.02580  4.06597  4.78831  0.61958  0.77255  0.48576  0.95510
     48   2.27437  4.56301  3.17631  2.64091  3.02849  3.45321  3.72659
3.13518  1.91898  2.82454  3.70066  3.10576  3.87168  2.94049  2.94160
2.71001  2.60653  2.86726  5.14595  3.86155      65 k - - X
          2.68618  4.42225  2.77519  2.73123  3.46354  2.40513  3.72494
3.29354  2.67741  2.69355  4.24690  2.90347  2.73739  3.18146  2.89801
2.37887  2.77519  2.98518  4.58477  3.61503
          0.02580  4.06597  4.78831  0.61958  0.77255  0.48576  0.95510
     49   2.83227  4.35514  4.17559  3.64487  3.35339  3.89495  4.37453
2.23722  3.50042  1.99328  1.68397  3.90890  4.30276  3.78927  3.73291
3.23499  2.80453  1.86764  5.11081  3.93650      66 m - - X
          2.68618  4.42225  2.77519  2.73123  3.46354  2.40513  3.72494
3.29354  2.67741  2.69355  4.24690  2.90347  2.73739  3.18146  2.89801
2.37887  2.77519  2.98518  4.58477  3.61503
          0.02580  4.06597  4.78831  0.61958  0.77255  0.48576  0.95510
     50   2.28106  4.62053  2.64741  2.53405  3.82413  3.42994  3.68446
3.22241  2.54669  2.50268  3.72913  3.02762  3.83969  2.88224  2.98481
2.25625  2.82625  2.93850  5.16727  3.34446      67 s - - X
          2.68618  4.42225  2.77519  2.73123  3.46354  2.40513  3.72494
3.29354  2.67741  2.69355  4.24690  2.90347  2.73739  3.18146  2.89801
2.37887  2.77519  2.98518  4.58477  3.61503
          0.02580  4.06597  4.78831  0.61958  0.77255  0.48576  0.95510
     51   2.95024  5.13900  3.21992  2.66551  4.55123  3.59277  3.63629
3.93456  1.86329  3.40708  4.25141  2.51316  3.98885  2.77101  1.41873
2.94238  3.14394  3.59555  5.48451  4.25725      68 r - - X
          2.68618  4.42225  2.77519  2.73123  3.46354  2.40513  3.72494
3.29354  2.67741  2.69355  4.24690  2.90347  2.73739  3.18146  2.89801
2.37887  2.77519  2.98518  4.58477  3.61503
          0.02580  4.06597  4.78831  0.61958  0.77255  0.48576  0.95510
     52   1.69729  4.56343  3.03146  2.25005  4.00780  3.30526  3.79912
3.34843  2.66005  3.04271  3.89405  3.06644  3.82340  2.99851  3.07877
2.35531  2.84309  2.77198  5.33574  4.04156      69 a - - X
          2.68618  4.42225  2.77519  2.73123  3.46354  2.40513  3.72494
3.29354  2.67741  2.69355  4.24690  2.90347  2.73739  3.18146  2.89801
2.37887  2.77519  2.98518  4.58477  3.61503
          0.02580  4.06597  4.78831  0.61958  0.77255  0.48576  0.95510
```

53 3.04918 5.03175 3.04542 2.80766 4.22524 3.50323 3.96612
3.89013 2.61596 3.37270 4.39941 3.26749 4.06220 0.92537 2.89446
3.09074 3.34803 3.60901 5.46468 4.17314 70 q - - X
2.68618 4.42225 2.77519 2.73123 3.46354 2.40513 3.72494
3.29354 2.67741 2.69355 4.24690 2.90347 2.73739 3.18146 2.89801
2.37887 2.77519 2.98518 4.58477 3.61503
0.02580 4.06597 4.78831 0.61958 0.77255 0.48576 0.95510
54 3.20895 5.35366 0.67572 2.47659 4.75938 3.36761 4.11707
4.39404 3.24020 3.96944 4.95589 3.04114 4.03443 3.35942 3.77958
3.15229 3.55307 4.01393 5.88916 4.60562 71 D - - X
2.68618 4.42225 2.77519 2.73123 3.46354 2.40513 3.72494
3.29354 2.67741 2.69355 4.24690 2.90347 2.73739 3.18146 2.89801
2.37887 2.77519 2.98518 4.58477 3.61503
0.02580 4.06597 4.78831 0.61958 0.77255 0.48576 0.95510
55 2.33042 4.28679 3.24274 3.09786 4.46583 1.08688 4.29772
3.95896 3.30252 3.63538 4.47142 3.30526 3.75697 3.57924 3.61286
2.06571 2.83361 3.36184 5.78193 4.55103 72 g - - X
2.68618 4.42225 2.77519 2.73123 3.46354 2.40513 3.72494
3.29354 2.67741 2.69355 4.24690 2.90347 2.73739 3.18146 2.89801
2.37887 2.77519 2.98518 4.58477 3.61503
0.02580 4.06597 4.78831 0.61958 0.77255 0.48576 0.95510
56 3.14218 4.44640 4.88041 4.39038 3.59589 4.50506 5.09827
1.13020 4.27707 2.08227 3.39570 4.60092 4.81211 4.53729 4.46784
3.90048 3.40936 1.28882 5.58483 4.38256 73 i - - X
2.68618 4.42225 2.77519 2.73123 3.46354 2.40513 3.72494
3.29354 2.67741 2.69355 4.24690 2.90347 2.73739 3.18146 2.89801
2.37887 2.77519 2.98518 4.58477 3.61503
0.02580 4.06597 4.78831 0.61958 0.77255 0.48576 0.95510
57 3.37882 4.78611 4.49943 4.14431 3.22022 4.22585 4.73971
2.43905 3.91022 0.66895 3.19014 4.40099 4.63059 4.23187 4.05293
3.82563 3.66575 2.52585 5.16535 3.93195 74 L - - X
2.68618 4.42225 2.77519 2.73123 3.46354 2.40513 3.72494
3.29354 2.67741 2.69355 4.24690 2.90347 2.73739 3.18146 2.89801
2.37887 2.77519 2.98518 4.58477 3.61503
0.02580 4.06597 4.78831 0.61958 0.77255 0.48576 0.95510
58 3.10703 5.21870 3.52241 2.84468 4.68974 3.70996 3.64217
4.03274 1.16904 3.46140 4.33425 3.24070 4.07615 2.77572 1.68112
3.09858 3.26871 3.71336 5.48968 4.33144 75 k - - X
2.68618 4.42225 2.77519 2.73123 3.46354 2.40513 3.72494
3.29354 2.67741 2.69355 4.24690 2.90347 2.73739 3.18146 2.89801
2.37887 2.77519 2.98518 4.58477 3.61503
0.02580 4.06597 4.78831 0.61958 0.77255 0.48576 0.95510
59 3.37000 4.91410 3.87614 3.51629 2.19115 4.06123 2.42074
3.53895 3.33980 2.94506 4.08202 3.69331 4.44443 3.64944 3.56593
3.43231 3.60199 3.36963 3.84025 1.05092 76 y - - X
2.68618 4.42225 2.77519 2.73123 3.46354 2.40513 3.72494
3.29354 2.67741 2.69355 4.24690 2.90347 2.73739 3.18146 2.89801
2.37887 2.77519 2.98518 4.58477 3.61503
0.02580 4.06597 4.78831 0.61958 0.77255 0.48576 0.95510
60 3.13199 4.65245 4.26270 3.87495 3.30719 3.98147 4.56542
2.50279 3.63121 1.87626 1.05027 4.12728 4.44675 4.00241 3.81296
3.49937 3.44300 2.53053 5.17137 3.94826 77 m - - X
2.68618 4.42225 2.77519 2.73123 3.46354 2.40513 3.72494
3.29354 2.67741 2.69355 4.24690 2.90347 2.73739 3.18146 2.89801
2.37887 2.77519 2.98518 4.58477 3.61503
0.02580 4.06597 4.78831 0.61958 0.77255 0.48576 0.95510

```
     61   2.79879  4.35153  4.00047  3.45979  3.24359  3.83135  4.18130
2.43039  3.29617  1.28125  2.71561  3.75694  4.21536  3.60739  3.53770
2.75007  3.05305  2.35317  4.94332  3.73134      78 l - - X
          2.68618  4.42225  2.77519  2.73123  3.46354  2.40513  3.72494
3.29354  2.67741  2.69355  4.24690  2.90347  2.73739  3.18146  2.89801
2.37887  2.77519  2.98518  4.58477  3.61503
          0.02580  4.06597  4.78831  0.61958  0.77255  0.48576  0.95510
     62   3.15895  5.12095  3.34717  2.93780  4.55191  3.62263  3.88116
3.99448  0.80088  3.51942  4.47451  3.35216  4.11266  3.06460  2.48782
3.19066  3.39982  3.69284  5.53001  4.37266      79 k - - X
          2.68618  4.42225  2.77519  2.73123  3.46354  2.40513  3.72494
3.29354  2.67741  2.69355  4.24690  2.90347  2.73739  3.18146  2.89801
2.37887  2.77519  2.98518  4.58477  3.61503
          0.02580  4.06597  4.78831  0.61958  0.77255  0.48576  0.95510
     63   2.94236  4.37788  4.35509  3.78448  3.20942  4.09950  4.42286
1.99536  3.65667  1.56018  1.91090  4.05337  4.40583  3.87985  3.85626
3.40733  2.75848  2.15981  5.02522  3.88488      80 l - - X
          2.68618  4.42225  2.77519  2.73123  3.46354  2.40513  3.72494
3.29354  2.67741  2.69355  4.24690  2.90347  2.73739  3.18146  2.89801
2.37887  2.77519  2.98518  4.58477  3.61503
          0.02580  4.06597  4.78831  0.61958  0.77255  0.48576  0.95510
     64   3.13199  4.65245  4.26270  3.87495  3.30719  3.98147  4.56542
2.50279  3.63121  1.87626  1.05027  4.12728  4.44675  4.00241  3.81296
3.49937  3.44300  2.53053  5.17137  3.94826      81 m - - X
          2.68618  4.42225  2.77519  2.73123  3.46354  2.40513  3.72494
3.29354  2.67741  2.69355  4.24690  2.90347  2.73739  3.18146  2.89801
2.37887  2.77519  2.98518  4.58477  3.61503
          0.02580  4.06597  4.78831  0.61958  0.77255  0.48576  0.95510
     65   3.13201  5.27001  2.58681  0.77842  4.64254  3.40845  4.01772
4.13568  2.92371  3.72353  4.69358  3.06030  4.02361  3.23223  3.34935
3.09731  3.43920  3.80121  5.77750  4.50534      82 e - - X
          2.68618  4.42225  2.77519  2.73123  3.46354  2.40513  3.72494
3.29354  2.67741  2.69355  4.24690  2.90347  2.73739  3.18146  2.89801
2.37887  2.77519  2.98518  4.58477  3.61503
          0.02580  4.06597  4.78831  0.61958  0.77255  0.48576  0.95510
     66   2.17340  4.30286  3.65856  3.15375  3.62778  3.49984  4.04223
2.61392  2.98096  2.58297  3.56657  3.46859  4.00163  3.37196  2.82526
2.84467  2.89557  1.51700  5.12342  3.89803      83 v - - X
          2.68618  4.42225  2.77519  2.73123  3.46354  2.40513  3.72494
3.29354  2.67741  2.69355  4.24690  2.90347  2.73739  3.18146  2.89801
2.37887  2.77519  2.98518  4.58477  3.61503
          0.02580  4.06597  4.78831  0.61958  0.77255  0.48576  0.95510
     67   2.69059  1.39633  4.02234  3.56823  3.12372  3.55942  4.07340
2.77076  3.37970  2.53984  3.58964  3.71968  4.10080  3.70033  3.58213
2.97871  3.02731  2.57296  4.70897  2.68898      84 c - - X
          2.68618  4.42225  2.77519  2.73123  3.46354  2.40513  3.72494
3.29354  2.67741  2.69355  4.24690  2.90347  2.73739  3.18146  2.89801
2.37887  2.77519  2.98518  4.58477  3.61503
          0.02580  4.06597  4.78831  0.61958  0.77255  0.48576  0.95510
     68   2.84519  5.11825  2.91089  2.49237  4.48767  3.47462  3.65110
3.91327  1.76523  3.41359  4.23994  1.81389  3.91533  2.78792  2.35688
2.82711  3.06859  3.55199  5.52707  4.22800      85 k - - X
          2.68618  4.42225  2.77519  2.73123  3.46354  2.40513  3.72494
3.29354  2.67741  2.69355  4.24690  2.90347  2.73739  3.18146  2.89801
2.37887  2.77519  2.98518  4.58477  3.61503
          0.02580  4.06597  4.78831  0.61958  0.77255  0.48576  0.95510
```

```
     69   1.13413  4.22226  3.80613  3.44490  3.94755  3.21089  4.36353
2.80115  3.38096  2.83585  3.88289  3.56750  3.90728  3.69556  3.64763
2.65307  2.85692  2.13744  5.49200  4.27177     86 a - - X
          2.68618  4.42225  2.77519  2.73123  3.46354  2.40513  3.72494
3.29354  2.67741  2.69355  4.24690  2.90347  2.73739  3.18146  2.89801
2.37887  2.77519  2.98518  4.58477  3.61503
          0.02580  4.06597  4.78831  0.61958  0.77255  0.48576  0.95510
     70   2.97089  5.12451  3.26534  2.70811  4.48912  3.60547  3.65577
3.91407  2.03366  3.37957  4.24883  3.14847  4.00648  1.70797  1.64836
2.97044  3.16749  3.58925  5.46051  4.22897     87 r - - X
          2.68618  4.42225  2.77519  2.73123  3.46354  2.40513  3.72494
3.29354  2.67741  2.69355  4.24690  2.90347  2.73739  3.18146  2.89801
2.37887  2.77519  2.98518  4.58477  3.61503
          0.02580  4.06597  4.78831  0.61958  0.77255  0.48576  0.95510
     71   2.92343  4.67539  3.63649  3.56258  4.69323  0.48449  4.66708
4.34379  3.76993  3.97234  4.95260  3.80138  4.07247  4.07908  3.98092
3.10155  3.41972  3.83474  5.73323  4.80306     88 G - - X
          2.68618  4.42225  2.77519  2.73123  3.46354  2.40513  3.72494
3.29354  2.67741  2.69355  4.24690  2.90347  2.73739  3.18146  2.89801
2.37887  2.77519  2.98518  4.58477  3.61503
          0.02580  4.06597  4.78831  0.61958  0.77255  0.48576  0.95510
     72   3.51469  4.86097  4.50462  4.25959  0.74124  4.15137  3.97776
3.01886  4.16279  2.34599  3.72269  4.24241  4.60843  4.27827  4.23449
3.77205  3.81736  3.02314  4.17644  2.52206     89 f - - X
          2.68618  4.42225  2.77519  2.73123  3.46354  2.40513  3.72494
3.29354  2.67741  2.69355  4.24690  2.90347  2.73739  3.18146  2.89801
2.37887  2.77519  2.98518  4.58477  3.61503
          0.02580  4.06597  4.78831  0.61958  0.77255  0.48576  0.95510
     73   2.96339  4.45718  4.38985  4.06301  3.69890  3.92024  4.83368
2.06096  3.93383  2.35484  3.63848  4.22069  4.47294  4.28154  4.12712
3.43917  3.32210  0.81171  5.51471  4.25663     90 v - - X
          2.68618  4.42225  2.77519  2.73123  3.46354  2.40513  3.72494
3.29354  2.67741  2.69355  4.24690  2.90347  2.73739  3.18146  2.89801
2.37887  2.77519  2.98518  4.58477  3.61503
          0.02580  4.06597  4.78831  0.61958  0.77255  0.48576  0.95510
     74   3.53803  4.94815  4.12940  3.89878  2.32685  4.05986  3.72610
3.52159  3.75101  2.92451  4.17717  3.96967  4.53092  4.00980  3.89333
3.65386  3.82662  3.40178  3.97672  0.69604     91 y - - X
          2.68618  4.42225  2.77519  2.73123  3.46354  2.40513  3.72494
3.29354  2.67741  2.69355  4.24690  2.90347  2.73739  3.18146  2.89801
2.37887  2.77519  2.98518  4.58477  3.61503
          0.02580  4.06597  4.78831  0.61958  0.77255  0.48576  0.95510
     75   2.92343  4.67539  3.63649  3.56258  4.69323  0.48449  4.66708
4.34379  3.76993  3.97234  4.95260  3.80138  4.07247  4.07908  3.98092
3.10155  3.41972  3.83474  5.73323  4.80306     92 G - - X
          2.68618  4.42225  2.77519  2.73123  3.46354  2.40513  3.72494
3.29354  2.67741  2.69355  4.24690  2.90347  2.73739  3.18146  2.89801
2.37887  2.77519  2.98518  4.58477  3.61503
          0.02580  4.06597  4.78831  0.61958  0.77255  0.48576  0.95510
     76   3.14393  4.47792  4.80237  4.33109  3.53463  4.43696  5.03762
0.99150  4.19096  2.01595  3.35537  4.54451  4.77204  4.46719  4.38062
3.84406  3.42030  1.57031  5.53119  4.32013     93 i - - X
          2.68618  4.42225  2.77519  2.73123  3.46354  2.40513  3.72494
3.29354  2.67741  2.69355  4.24690  2.90347  2.73739  3.18146  2.89801
2.37887  2.77519  2.98518  4.58477  3.61503
          0.02580  4.06597  4.78831  0.61958  0.77255  0.48576  0.95510
```

77 3.14181 4.44550 4.88164 4.39135 3.59771 4.50603 5.09930
1.13859 4.27855 2.08472 3.39727 4.60174 4.81273 4.53871 4.46940
3.90127 3.40886 1.27726 5.58607 4.38366 94 i - - X
2.68618 4.42225 2.77519 2.73123 3.46354 2.40513 3.72494
3.29354 2.67741 2.69355 4.24690 2.90347 2.73739 3.18146 2.89801
2.37887 2.77519 2.98518 4.58477 3.61503
0.02580 4.06597 4.78831 0.61958 0.77255 0.48576 0.95510
78 2.67467 4.83114 2.28699 2.44277 4.12886 3.34286 3.75703
3.54009 2.62223 2.77135 4.04744 2.95715 1.91523 2.93739 3.07983
2.71133 2.95528 3.21707 5.43507 4.07900 95 p - - X
2.68618 4.42225 2.77519 2.73123 3.46354 2.40513 3.72494
3.29354 2.67741 2.69355 4.24690 2.90347 2.73739 3.18146 2.89801
2.37887 2.77519 2.98518 4.58477 3.61503
0.02580 4.06597 4.78831 0.61958 0.77255 0.48576 0.95510
79 2.92511 5.27370 2.45671 1.15594 4.44626 3.35247 3.17427
4.05423 2.58777 3.57603 4.44118 2.85943 3.90852 2.93919 3.02445
2.86725 3.19050 3.68683 5.65754 4.23531 96 e - - X
2.68618 4.42225 2.77519 2.73123 3.46354 2.40513 3.72494
3.29354 2.67741 2.69355 4.24690 2.90347 2.73739 3.18146 2.89801
2.37887 2.77519 2.98518 4.58477 3.61503
0.02580 4.06597 4.78831 0.61958 0.77255 0.48576 0.95510
80 2.47374 4.93794 2.89119 2.47534 4.36871 3.38687 3.68100
3.78682 1.53043 3.33678 4.15348 2.64612 3.85796 2.82475 2.69193
2.47602 2.96681 3.41317 5.50349 4.18640 97 k - - X
2.68618 4.42225 2.77519 2.73123 3.46354 2.40513 3.72494
3.29354 2.67741 2.69355 4.24690 2.90347 2.73739 3.18146 2.89801
2.37887 2.77519 2.98518 4.58477 3.61503
0.02580 4.06597 4.78831 0.61958 0.77255 0.48576 0.95510
81 2.92343 4.67539 3.63649 3.56258 4.69323 0.48449 4.66708
4.34379 3.76993 3.97234 4.95260 3.80138 4.07247 4.07908 3.98092
3.10155 3.41972 3.83474 5.73323 4.80306 98 G - - X
2.68618 4.42225 2.77519 2.73123 3.46354 2.40513 3.72494
3.29354 2.67741 2.69355 4.24690 2.90347 2.73739 3.18146 2.89801
2.37887 2.77519 2.98518 4.58477 3.61503
0.02580 4.06597 4.78831 0.61958 0.77255 0.48576 0.95510
82 2.80796 4.99196 3.02766 2.34792 4.32137 3.50021 3.66165
3.65849 1.39054 3.24980 4.10329 3.03756 3.92156 2.80784 2.52400
2.82147 3.03020 2.92543 5.43988 4.15618 99 k - - X
2.68618 4.42225 2.77519 2.73123 3.46354 2.40513 3.72494
3.29354 2.67741 2.69355 4.24690 2.90347 2.73739 3.18146 2.89801
2.37887 2.77519 2.98518 4.58477 3.61503
0.02580 4.06597 4.78831 0.61958 0.77255 0.48576 0.95510
83 2.43854 4.36781 3.31799 3.07237 4.24972 3.12006 4.19488
3.57332 3.09528 3.30093 4.23046 3.33299 1.21823 3.46360 3.39976
2.60458 2.40345 3.15765 5.60560 4.37384 100 p - - X
2.68618 4.42225 2.77519 2.73123 3.46354 2.40513 3.72494
3.29354 2.67741 2.69355 4.24690 2.90347 2.73739 3.18146 2.89801
2.37887 2.77519 2.98518 4.58477 3.61503
0.02580 4.06597 4.78831 0.61958 0.77255 0.48576 0.95510
84 3.01572 4.42827 4.54316 4.04712 3.48532 4.19711 4.75891
1.92835 3.89140 2.04402 2.85284 4.27576 4.57966 4.18705 4.10078
3.57869 3.29724 1.01422 5.37314 4.16308 101 v - - X
2.68618 4.42225 2.77519 2.73123 3.46354 2.40513 3.72494
3.29354 2.67741 2.69355 4.24690 2.90347 2.73739 3.18146 2.89801
2.37887 2.77519 2.98518 4.58477 3.61503
0.02580 4.06597 4.78831 0.61958 0.77255 0.48576 0.95510

```
     85   1.99374  4.20656  3.48041  3.15898  4.30358  2.99403  4.22276
3.65259  3.18321  3.38329  4.22026  3.32811  3.73043  3.48635  3.50140
1.20440  2.27966  3.14147  5.65129  4.43546    102 s - - X
          2.68618  4.42225  2.77519  2.73123  3.46354  2.40513  3.72494
3.29354  2.67741  2.69355  4.24690  2.90347  2.73739  3.18146  2.89801
2.37887  2.77519  2.98518  4.58477  3.61503
          0.02580  4.06597  4.78831  0.61958  0.77255  0.48576  0.95510
     86   2.31873  4.27862  3.25014  3.09121  4.46327  1.15445  4.28627
3.95073  3.28772  3.62286  4.45331  3.29840  3.74967  3.56257  3.60345
1.93975  2.81998  3.35228  5.77941  4.54850    103 g - - X
          2.68618  4.42225  2.77519  2.73123  3.46354  2.40513  3.72494
3.29354  2.67741  2.69355  4.24690  2.90347  2.73739  3.18146  2.89801
2.37887  2.77519  2.98518  4.58477  3.61503
          0.02580  4.06597  4.78831  0.61958  0.77255  0.48576  0.95510
     87   1.40291  4.19006  3.50145  3.21797  4.31933  2.97610  4.27576
3.68216  3.26350  3.41978  4.26341  3.35453  3.72965  3.55500  3.56636
1.41562  2.74448  3.15628  5.67823  4.46199    104 a - - X
          2.68618  4.42225  2.77519  2.73123  3.46354  2.40513  3.72494
3.29354  2.67741  2.69355  4.24690  2.90347  2.73739  3.18146  2.89801
2.37887  2.77519  2.98518  4.58477  3.61503
          0.02580  4.06597  4.78831  0.61958  0.77255  0.48576  0.95510
     88   2.42075  4.34145  3.38622  3.21538  4.27507  3.06832  4.31764
3.82606  3.30239  3.53276  4.44528  3.40582  3.82400  3.63716  3.57937
0.83261  2.91617  3.31815  5.63277  4.36086    105 s - - X
          2.68618  4.42225  2.77519  2.73123  3.46354  2.40513  3.72494
3.29354  2.67741  2.69355  4.24690  2.90347  2.73739  3.18146  2.89801
2.37887  2.77519  2.98518  4.58477  3.61503
          0.02580  4.06597  4.78831  0.61958  0.77255  0.48576  0.95510
     89   3.20895  5.35366  0.67572  2.47659  4.75938  3.36761  4.11707
4.39404  3.24020  3.96944  4.95589  3.04114  4.03443  3.35942  3.77958
3.15229  3.55307  4.01393  5.88916  4.60562    106 D - - X
          2.68618  4.42225  2.77519  2.73123  3.46354  2.40513  3.72494
3.29354  2.67741  2.69355  4.24690  2.90347  2.73739  3.18146  2.89801
2.37887  2.77519  2.98518  4.58477  3.61503
          0.02580  4.06597  4.78831  0.61958  0.77255  0.48576  0.95510
     90   2.56178  4.66485  2.76540  2.57226  4.36707  3.16515  3.92553
3.89821  2.81013  3.50939  4.34738  1.54633  3.81296  3.12797  3.22480
1.86000  2.95149  3.43273  5.64924  4.28993    107 n - - X
          2.68618  4.42225  2.77519  2.73123  3.46354  2.40513  3.72494
3.29354  2.67741  2.69355  4.24690  2.90347  2.73739  3.18146  2.89801
2.37887  2.77519  2.98518  4.58477  3.61503
          0.02580  4.06597  4.78831  0.61958  0.77255  0.48576  0.95510
     91   3.32845  4.65525  4.86371  4.33781  3.16368  4.57780  4.91516
1.73005  4.15758  0.92455  2.98316  4.59816  4.78969  4.33623  4.29650
3.95534  3.56413  2.12405  5.24547  4.11473    108 l - - X
          2.68618  4.42225  2.77519  2.73123  3.46354  2.40513  3.72494
3.29354  2.67741  2.69355  4.24690  2.90347  2.73739  3.18146  2.89801
2.37887  2.77519  2.98518  4.58477  3.61503
          0.02580  4.06597  4.78831  0.61958  0.77255  0.48576  0.95510
     92   3.23578  5.05316  3.74734  3.22314  4.45741  3.68117  3.98214
4.01613  2.33942  3.49703  4.49575  3.56619  4.17759  3.19599  0.71938
3.31284  3.49063  3.73260  5.46392  4.34662    109 r - - X
          2.68618  4.42225  2.77519  2.73123  3.46354  2.40513  3.72494
3.29354  2.67741  2.69355  4.24690  2.90347  2.73739  3.18146  2.89801
2.37887  2.77519  2.98518  4.58477  3.61503
          0.02580  4.06597  4.78831  0.61958  0.77255  0.48576  0.95510
```

93 2.13158 4.77941 2.90444 2.27200 4.16359 2.57564 3.68183
3.58650 2.47137 3.17876 3.99052 2.95313 3.80482 2.83515 2.60314
2.30233 2.85586 3.22764 5.40398 4.07054 110 a - - X
2.68618 4.42225 2.77519 2.73123 3.46354 2.40513 3.72494
3.29354 2.67741 2.69355 4.24690 2.90347 2.73739 3.18146 2.89801
2.37887 2.77519 2.98518 4.58477 3.61503
0.02580 4.06597 4.78831 0.61958 0.77255 0.48576 0.95510
94 3.67704 4.97923 4.39985 4.15673 2.89117 3.95783 4.15897
3.71339 3.87713 3.07301 4.33751 4.26783 4.49253 4.25464 3.97000
3.87873 3.98817 3.60894 0.57145 2.88383 111 W - - X
2.68618 4.42225 2.77519 2.73123 3.46354 2.40513 3.72494
3.29354 2.67741 2.69355 4.24690 2.90347 2.73739 3.18146 2.89801
2.37887 2.77519 2.98518 4.58477 3.61503
0.02580 4.06597 4.78831 0.61958 0.77255 0.48576 0.95510
95 3.67704 4.97923 4.39985 4.15673 2.89117 3.95783 4.15897
3.71339 3.87713 3.07301 4.33751 4.26783 4.49253 4.25464 3.97000
3.87873 3.98817 3.60894 0.57145 2.88383 112 W - - X
2.68618 4.42225 2.77519 2.73123 3.46354 2.40513 3.72494
3.29354 2.67741 2.69355 4.24690 2.90347 2.73739 3.18146 2.89801
2.37887 2.77519 2.98518 4.58477 3.61503
0.02580 4.06597 4.78831 0.61958 0.77255 0.48576 0.95510
96 3.15895 5.12095 3.34717 2.93780 4.55191 3.62263 3.88116
3.99448 0.80088 3.51942 4.47451 3.35216 4.11266 3.06460 2.48782
3.19066 3.39982 3.69284 5.53001 4.37266 113 k - - X
2.68618 4.42225 2.77519 2.73123 3.46354 2.40513 3.72494
3.29354 2.67741 2.69355 4.24690 2.90347 2.73739 3.18146 2.89801
2.37887 2.77519 2.98518 4.58477 3.61503
0.02580 4.06597 4.78831 0.61958 0.77255 0.48576 0.95510
97 2.99068 5.56556 1.65250 1.24803 4.85376 3.26006 3.80529
4.37531 2.82227 3.87388 4.72809 2.72377 3.88247 2.97161 3.41787
2.87160 3.27940 3.94635 6.02100 4.51701 114 e - - X
2.68618 4.42225 2.77519 2.73123 3.46354 2.40513 3.72494
3.29354 2.67741 2.69355 4.24690 2.90347 2.73739 3.18146 2.89801
2.37887 2.77519 2.98518 4.58477 3.61503
0.02580 4.06597 4.78831 0.61958 0.77255 0.48576 0.95510
98 2.79539 5.21067 2.35215 2.29484 4.54248 3.35712 3.67161
4.00929 1.56595 3.51454 4.31936 2.29719 3.85146 2.80738 2.84897
2.74611 3.04244 3.61236 5.65527 4.26533 115 k - - X
2.68618 4.42225 2.77519 2.73123 3.46354 2.40513 3.72494
3.29354 2.67741 2.69355 4.24690 2.90347 2.73739 3.18146 2.89801
2.37887 2.77519 2.98518 4.58477 3.61503
0.02580 4.06597 4.78831 0.61958 0.77255 0.48576 0.95510
99 2.96339 4.45718 4.38985 4.06301 3.69890 3.92024 4.83368
2.06096 3.93383 2.35484 3.63848 4.22069 4.47294 4.28154 4.12712
3.43917 3.32210 0.81171 5.51471 4.25663 116 v - - X
2.68618 4.42225 2.77519 2.73123 3.46354 2.40513 3.72494
3.29354 2.67741 2.69355 4.24690 2.90347 2.73739 3.18146 2.89801
2.37887 2.77519 2.98518 4.58477 3.61503
0.02580 4.06597 4.78831 0.61958 0.77255 0.48576 0.95510
100 2.54409 5.02561 3.11146 2.54041 4.38077 3.51074 3.61338
3.77994 1.95660 3.29582 4.11290 3.02585 3.90049 2.45674 1.77333
2.55220 2.99360 3.43250 5.43523 4.15445 117 r - - X
2.68618 4.42225 2.77519 2.73123 3.46354 2.40513 3.72494
3.29354 2.67741 2.69355 4.24690 2.90347 2.73739 3.18146 2.89801
2.37887 2.77519 2.98518 4.58477 3.61503
0.02580 4.06597 4.78831 0.61958 0.77255 0.48576 0.95510

```
     101   3.51469  4.86097  4.50462  4.25959  0.74124  4.15137  3.97776
 3.01886  4.16279  2.34599  3.72269  4.24241  4.60843  4.27827  4.23449
 3.77205  3.81736  3.02314  4.17644  2.52206    118 f - - X
           2.68618  4.42225  2.77519  2.73123  3.46354  2.40513  3.72494
 3.29354  2.67741  2.69355  4.24690  2.90347  2.73739  3.18146  2.89801
 2.37887  2.77519  2.98518  4.58477  3.61503
           0.02580  4.06597  4.78831  0.61958  0.77255  0.48576  0.95510
     102   3.20895  5.35366  0.67572  2.47659  4.75938  3.36761  4.11707
 4.39404  3.24020  3.96944  4.95589  3.04114  4.03443  3.35942  3.77958
 3.15229  3.55307  4.01393  5.88916  4.60562    119 D - - X
           2.68618  4.42225  2.77519  2.73123  3.46354  2.40513  3.72494
 3.29354  2.67741  2.69355  4.24690  2.90347  2.73739  3.18146  2.89801
 2.37887  2.77519  2.98518  4.58477  3.61503
           0.02580  4.06597  4.78831  0.61958  0.77255  0.48576  0.95510
     103   2.89879  5.06236  3.31714  2.67747  4.42892  3.60647  3.62018
 3.79796  1.84420  3.29959  4.14333  3.12489  3.97422  2.43407  1.51956
 2.90634  3.08734  3.09456  5.41822  4.19015    120 r - - X
           2.68618  4.42225  2.77519  2.73123  3.46354  2.40513  3.72494
 3.29354  2.67741  2.69355  4.24690  2.90347  2.73739  3.18146  2.89801
 2.37887  2.77519  2.98518  4.58477  3.61503
           0.02580  4.06597  4.78831  0.61958  0.77255  0.48576  0.95510
     104   2.04192  4.53461  2.96796  2.66835  4.27432  3.17153  3.92237
 3.63666  2.78670  3.32092  4.16126  1.63417  3.79494  3.12005  3.19081
 2.58493  2.43929  3.21828  5.56781  4.26659    121 n - - X
           2.68618  4.42225  2.77519  2.73123  3.46354  2.40513  3.72494
 3.29354  2.67741  2.69355  4.24690  2.90347  2.73739  3.18146  2.89801
 2.37887  2.77519  2.98518  4.58477  3.61503
           0.02580  4.06597  4.78831  0.61958  0.77255  0.48576  0.95510
     105   1.94399  4.26548  3.36131  3.20233  4.45997  1.05649  4.35385
 3.84159  3.36842  3.57828  4.44093  3.36973  3.77006  3.64689  3.65940
 2.50862  2.83828  3.28766  5.78069  4.58983    122 g - - X
           2.68618  4.42225  2.77519  2.73123  3.46354  2.40513  3.72494
 3.29354  2.67741  2.69355  4.24690  2.90347  2.73739  3.18146  2.89801
 2.37887  2.77519  2.98518  4.58477  3.61503
           0.02580  4.06597  4.78831  0.61958  0.77255  0.48576  0.95510
     106   3.02861  4.74922  3.65739  3.52739  4.49912  3.42598  4.58547
 4.09872  3.60213  3.70274  4.76356  3.81267  0.53536  3.98492  3.82221
 3.20306  3.48618  3.72125  5.62838  4.62373    123 P - - X
           2.68618  4.42225  2.77519  2.73123  3.46354  2.40513  3.72494
 3.29354  2.67741  2.69355  4.24690  2.90347  2.73739  3.18146  2.89801
 2.37887  2.77519  2.98518  4.58477  3.61503
           0.02580  4.06597  4.78831  0.61958  0.77255  0.48576  0.95510
     107   1.84199  4.39608  3.30115  2.76688  3.71361  3.38399  3.80917
 3.04884  2.73172  2.76783  3.18503  2.78672  3.85329  3.06175  3.12116
 2.52696  2.56663  2.59673  5.10701  3.84909    124 a - - X
           2.68618  4.42225  2.77519  2.73123  3.46354  2.40513  3.72494
 3.29354  2.67741  2.69355  4.24690  2.90347  2.73739  3.18146  2.89801
 2.37887  2.77519  2.98518  4.58477  3.61503
           0.02580  4.06597  4.78831  0.61958  0.77255  0.48576  0.95510
     108   0.76718  4.29954  3.66918  3.47445  4.25490  3.11796  4.47219
 3.41012  3.49596  3.28238  4.29640  3.57189  3.87593  3.82006  3.73013
 2.64994  2.94097  3.03063  5.66250  4.48579    125 a - - X
           2.68618  4.42225  2.77519  2.73123  3.46354  2.40513  3.72494
 3.29354  2.67741  2.69355  4.24690  2.90347  2.73739  3.18146  2.89801
 2.37887  2.77519  2.98518  4.58477  3.61503
           0.02580  4.06597  4.78831  0.61958  0.77255  0.48576  0.95510
```

```
     109   3.19558  4.49000  4.90940  4.38070  3.42165  4.56450  5.02242
1.18176  4.26027  1.60568  3.21758  4.61099  4.80849  4.46449  4.42631
3.93407  3.44149  1.58295  5.43852  4.28651    126 i - - X
          2.68618  4.42225  2.77519  2.73123  3.46354  2.40513  3.72494
3.29354  2.67741  2.69355  4.24690  2.90347  2.73739  3.18146  2.89801
2.37887  2.77519  2.98518  4.58477  3.61503
          0.02580  4.06597  4.78831  0.61958  0.77255  0.48576  0.95510
     110   1.95245  4.23107  3.69838  3.16986  3.54270  3.49030  4.03789
2.17335  3.10469  2.53656  3.49149  3.47607  3.98111  3.40112  3.41584
2.28935  2.84443  2.05683  5.04305  3.82201    127 a - - X
          2.68618  4.42225  2.77519  2.73123  3.46354  2.40513  3.72494
3.29354  2.67741  2.69355  4.24690  2.90347  2.73739  3.18146  2.89801
2.37887  2.77519  2.98518  4.58477  3.61503
          0.02580  4.06597  4.78831  0.61958  0.77255  0.48576  0.95510
     111   2.75730  5.12811  2.31831  2.31375  4.49162  2.62434  3.68554
3.94946  1.57957  3.47469  4.28023  2.85712  3.84177  2.82477  2.90018
2.72382  3.01456  3.55443  5.63147  4.24972    128 k - - X
          2.68618  4.42225  2.77519  2.73123  3.46354  2.40513  3.72494
3.29354  2.67741  2.69355  4.24690  2.90347  2.73739  3.18146  2.89801
2.37887  2.77519  2.98518  4.58477  3.61503
          0.02580  4.06597  4.78831  0.61958  0.77255  0.48576  0.95510
     112   2.71938  4.61976  3.26237  2.74026  3.33197  3.56132  3.13802
3.20281  2.56572  2.83279  3.72958  3.18103  3.34875  3.00147  2.64425
2.83025  2.94874  2.94925  4.79792  1.93759    129 y - - X
          2.68621  4.42228  2.77523  2.73126  3.46357  2.40516  3.72497
3.29357  2.67744  2.69327  4.24693  2.90350  2.73743  3.18149  2.89804
2.37890  2.77506  2.98521  4.58480  3.61506
          0.21019  2.50089  2.22974  0.84137  0.56408  0.48576  0.95510
     113   2.40771  5.03245  2.60715  1.96515  4.35263  3.33184  3.65478
3.77561  2.40383  3.33394  4.16564  2.85053  3.82352  1.94820  2.82329
2.71401  2.98403  3.41889  5.52937  4.16940    133 q - - X
          2.68618  4.42225  2.77519  2.73123  3.46354  2.40513  3.72494
3.29354  2.67741  2.69355  4.24690  2.90347  2.73739  3.18146  2.89801
2.37887  2.77519  2.98518  4.58477  3.61503
          0.02869  3.96146  4.68381  0.61958  0.77255  0.59008  0.80807
     114   1.80665  4.55113  3.10361  2.46611  3.87796  3.37221  3.72553
3.19972  2.51344  2.89656  3.76730  3.07206  3.83416  2.93029  2.59785
2.67084  2.83586  2.69542  5.20742  3.93068    134 a - - X
          2.68618  4.42225  2.77519  2.73123  3.46354  2.40513  3.72494
3.29354  2.67741  2.69355  4.24690  2.90347  2.73739  3.18146  2.89801
2.37887  2.77519  2.98518  4.58477  3.61503
          0.02869  3.96146  4.68381  0.61958  0.77255  0.59008  0.80807
     115   2.42155  5.25559  1.68238  1.93631  4.55318  3.25765  3.67410
4.02466  2.37910  3.54962  4.36338  2.72971  3.80125  2.82095  3.10097
2.71545  3.04789  3.62401  5.72681  4.28530    135 d - - X
          2.68618  4.42225  2.77519  2.73123  3.46354  2.40513  3.72494
3.29354  2.67741  2.69355  4.24690  2.90347  2.73739  3.18146  2.89801
2.37887  2.77519  2.98518  4.58477  3.61503
          0.02869  3.96146  4.68381  0.61958  0.77255  0.59008  0.80807
     116   2.57371  3.93818  3.07330  2.53241  3.79328  3.42486  3.34802
2.89532  2.47164  2.85268  3.70297  2.28886  3.82421  2.58578  2.88389
2.66005  2.80917  2.92246  5.12732  3.82461    136 n - - X
          2.68618  4.42225  2.77519  2.73123  3.46354  2.40513  3.72494
3.29354  2.67741  2.69355  4.24690  2.90347  2.73739  3.18146  2.89801
2.37887  2.77519  2.98518  4.58477  3.61503
          0.02869  3.96146  4.68381  0.61958  0.77255  0.59008  0.80807
```

117 2.41639 4.30302 3.37437 2.85048 3.16504 3.46865 3.79987
2.81118 2.80132 1.98629 3.45481 2.88701 3.89503 3.12370 3.15331
2.44755 2.79870 2.58022 4.90258 3.63441 137 l - - X
2.68618 4.42225 2.77519 2.73123 3.46354 2.40513 3.72494
3.29354 2.67741 2.69355 4.24690 2.90347 2.73739 3.18146 2.89801
2.37887 2.77519 2.98518 4.58477 3.61503
0.02869 3.96146 4.68381 0.61958 0.77255 0.50601 0.92356
118 2.27304 4.28766 4.23494 3.66924 3.30396 4.00576 4.35280
1.91042 3.57131 1.65157 2.88011 3.94629 4.33711 3.81122 3.79913
3.30835 3.07979 1.80513 5.03408 3.86171 138 l - - X
2.68618 4.42225 2.77519 2.73123 3.46354 2.40513 3.72494
3.29354 2.67741 2.69355 4.24690 2.90347 2.73739 3.18146 2.89801
2.37887 2.77519 2.98518 4.58477 3.61503
0.02720 4.01411 4.73646 0.61958 0.77255 0.45314 1.00958
119 2.00013 4.41135 3.26634 2.73535 3.35965 2.99308 3.79482
3.11352 2.71526 2.61990 3.66215 3.15752 3.15636 3.03934 3.11484
2.22769 2.78838 2.82624 5.11993 3.85531 139 a - - X
2.68618 4.42225 2.77519 2.73123 3.46354 2.40513 3.72494
3.29354 2.67741 2.69355 4.24690 2.90347 2.73739 3.18146 2.89801
2.37887 2.77519 2.98518 4.58477 3.61503
0.02580 4.06597 4.78831 0.61958 0.77255 0.48576 0.95510
120 2.33609 4.65925 2.51602 2.50893 4.14055 2.42878 3.73523
3.55655 2.57230 3.17118 3.98387 2.98834 3.78442 2.89889 3.02885
2.33259 2.41672 2.91503 5.41067 4.08528 140 s - - X
2.68618 4.42225 2.77519 2.73123 3.46354 2.40513 3.72494
3.29354 2.67741 2.69355 4.24690 2.90347 2.73739 3.18146 2.89801
2.37887 2.77519 2.98518 4.58477 3.61503
0.12272 4.06597 2.31928 0.61958 0.77255 0.48576 0.95510
121 2.57112 4.81108 2.76286 2.38038 3.67376 2.83040 3.58766
3.48789 2.20515 3.07660 3.54476 2.65263 3.77775 2.72997 2.64176
2.59319 2.80296 3.15259 5.30450 3.96113 141 k - - X
2.68618 4.42225 2.77519 2.73123 3.46354 2.40513 3.72494
3.29354 2.67741 2.69355 4.24690 2.90347 2.73739 3.18146 2.89801
2.37887 2.77519 2.98518 4.58477 3.61503
0.02839 3.97164 4.69398 0.61958 0.77255 0.58084 0.81968
122 2.35535 5.06656 2.17659 1.93850 4.41225 2.87236 3.65878
3.87421 2.52469 3.41679 4.21837 2.78360 3.78293 2.80150 3.03980
2.26661 2.95067 3.47878 5.60675 4.19869 142 e - - X
2.68618 4.42225 2.77519 2.73123 3.46354 2.40513 3.72494
3.29354 2.67741 2.69355 4.24690 2.90347 2.73739 3.18146 2.89801
2.37887 2.77519 2.98518 4.58477 3.61503
0.02839 3.97164 4.69398 0.61958 0.77255 0.58084 0.81968
123 2.71809 5.14042 2.15682 2.11016 4.43713 3.29658 3.63800
3.90220 2.31981 3.42977 4.22967 2.77637 3.79270 2.59808 2.97142
1.96405 2.96861 3.51200 5.60768 4.19766 143 s - - X
2.68618 4.42225 2.77519 2.73123 3.46354 2.40513 3.72494
3.29354 2.67741 2.69355 4.24690 2.90347 2.73739 3.18146 2.89801
2.37887 2.77519 2.98518 4.58477 3.61503
0.02839 3.97164 4.69398 0.61958 0.77255 0.58084 0.81968
124 2.58725 4.86984 2.69590 2.25717 4.14177 3.12808 3.59135
3.57388 2.37716 2.81859 3.94759 2.73207 3.02416 2.72861 2.68823
2.42142 2.82333 3.22397 5.36359 4.00723 144 e - - X
2.68618 4.42225 2.77519 2.73123 3.46354 2.40513 3.72494
3.29354 2.67741 2.69355 4.24690 2.90347 2.73739 3.18146 2.89801
2.37887 2.77519 2.98518 4.58477 3.61503
0.02839 3.97164 4.69398 0.61958 0.77255 0.58084 0.81968

```
    125   2.56855  4.11220  2.66025  2.37436  4.08775  3.04604  3.58634
  3.51403  2.36459  2.92948  3.89907  2.75404  3.77404  2.55952  2.51501
  2.48410  2.80237  3.17231  5.32008  3.97366    145 k - - X
          2.68603  4.42230  2.77525  2.73115  3.46359  2.40518  3.72500
  3.29359  2.67746  2.69360  4.24695  2.90352  2.73732  3.18152  2.89806
  2.37867  2.77525  2.98524  4.58482  3.61508
          0.52490  1.56219  1.61590  0.63341  0.75668  0.43059  1.05018
    126   1.99950  4.20409  3.57278  3.04966  3.49506  3.40854  3.94659
  2.59303  2.96869  2.46223  2.70313  3.37228  3.90219  3.28690  3.28619
  2.54917  2.78663  2.16958  4.99657  3.77161    149 a - - .
          2.68618  4.42225  2.77519  2.73123  3.46354  2.40513  3.72494
  3.29354  2.67741  2.69355  4.24690  2.90347  2.73739  3.18146  2.89801
  2.37887  2.77519  2.98518  4.58477  3.61503
          0.03127  3.87663  4.59897  0.61958  0.77255  0.43981  1.03326
    127   2.06025  4.28475  3.25936  2.92217  4.26724  1.82217  4.06745
  3.67135  2.99505  3.33893  4.16149  3.20333  2.22773  3.29769  3.36385
  2.45312  2.49277  3.17566  5.58623  4.34068    150 g - - .
          2.68618  4.42225  2.77519  2.73123  3.46354  2.40513  3.72494
  3.29354  2.67741  2.69355  4.24690  2.90347  2.73739  3.18146  2.89801
  2.37887  2.77519  2.98518  4.58477  3.61503
          0.02676  4.03006  4.75240  0.61958  0.77255  0.46251  0.99344
    128   2.40242  3.42607  2.79709  2.51144  3.91535  2.90833  3.66945
  3.31766  2.50400  2.95168  3.78324  2.71780  3.81383  2.84647  2.79189
  2.51521  2.67162  2.82784  5.22524  3.91607    151 a - - X
          2.68618  4.42225  2.77519  2.73123  3.46354  2.40513  3.72494
  3.29354  2.67741  2.69355  4.24690  2.90347  2.73739  3.18146  2.89801
  2.37887  2.77519  2.98518  4.58477  3.61503
          0.02580  4.06597  4.78831  0.61958  0.77255  0.48576  0.95510
    129   2.62123  4.82515  2.97649  2.45433  4.12813  2.88377  3.63682
  3.54547  2.27465  2.91321  3.94487  2.79005  3.82234  2.78452  2.44806
  2.06421  2.86188  3.20760  5.35030  4.02527    152 s - - X
          2.68618  4.42225  2.77519  2.73123  3.46354  2.40513  3.72494
  3.29354  2.67741  2.69355  4.24690  2.90347  2.73739  3.18146  2.89801
  2.37887  2.77519  2.98518  4.58477  3.61503
          0.02580  4.06597  4.78831  0.61958  0.77255  0.48576  0.95510
    130   2.56538  4.50540  3.16631  2.62973  3.26978  2.68548  3.71754
  3.09932  2.61760  2.77124  3.63265  2.74714  3.85565  2.94840  3.03421
  2.45227  2.48262  2.83101  5.07564  3.22919    153 s - - -
          2.68618  4.42225  2.77519  2.73123  3.46354  2.40513  3.72494
  3.29354  2.67741  2.69355  4.24690  2.90347  2.73739  3.18146  2.89801
  2.37887  2.77519  2.98518  4.58477  3.61503
          0.02580  4.06597  4.78831  0.61958  0.77255  0.48576  0.95510
    131   2.41577  4.48753  3.24281  2.69468  3.65869  3.49563  3.74751
  2.69224  2.63156  2.34571  3.57795  3.15236  2.76446  2.53360  3.02187
  2.73990  2.83543  2.75119  5.06140  3.79634    154 l - - -
          2.68618  4.42225  2.77519  2.73123  3.46354  2.40513  3.72494
  3.29354  2.67741  2.69355  4.24690  2.90347  2.73739  3.18146  2.89801
  2.37887  2.77519  2.98518  4.58477  3.61503
          0.02580  4.06597  4.78831  0.61958  0.77255  0.48576  0.95510
    132   2.64497  3.86756  3.27513  2.73383  3.13261  3.52250  2.01653
  3.09621  2.63532  2.74500  3.64421  3.17662  3.92249  2.72224  3.00810
  2.77631  2.88372  2.84758  4.86761  3.46585    155 h - - -
          2.68618  4.42225  2.77519  2.73123  3.46354  2.40513  3.72494
  3.29354  2.67741  2.69355  4.24690  2.90347  2.73739  3.18146  2.89801
  2.37887  2.77519  2.98518  4.58477  3.61503
          0.02580  4.06597  4.78831  0.61958  0.77255  0.48576  0.95510
```

133 2.57027 4.41302 3.28232 2.71924 2.98608 3.15069 3.74030
2.94563 2.49588 2.63791 3.07966 3.16765 3.88534 3.00630 2.83670
2.39096 2.67144 2.57798 4.98094 3.72383 156 s - - -
2.68618 4.42225 2.77519 2.73123 3.46354 2.40513 3.72494
3.29354 2.67741 2.69355 4.24690 2.90347 2.73739 3.18146 2.89801
2.37887 2.77519 2.98518 4.58477 3.61503
0.02580 4.06597 4.78831 0.61958 0.77255 0.48576 0.95510
134 3.37882 4.78611 4.49943 4.14431 3.22022 4.22585 4.73971
2.43905 3.91022 0.66895 3.19014 4.40099 4.63059 4.23187 4.05293
3.82563 3.66575 2.52585 5.16535 3.93195 157 L - - S
2.68618 4.42225 2.77519 2.73123 3.46354 2.40513 3.72494
3.29354 2.67741 2.69355 4.24690 2.90347 2.73739 3.18146 2.89801
2.37887 2.77519 2.98518 4.58477 3.61503
0.02580 4.06597 4.78831 0.61958 0.77255 0.48576 0.95510
135 2.50747 4.60715 3.16686 2.60992 3.75444 3.48930 2.58466
3.16940 2.49425 2.42323 3.33993 3.08703 3.87688 2.44688 2.88192
2.72422 2.85316 2.90828 5.11027 3.81466 158 l - - T
2.68618 4.42225 2.77519 2.73123 3.46354 2.40513 3.72494
3.29354 2.67741 2.69355 4.24690 2.90347 2.73739 3.18146 2.89801
2.37887 2.77519 2.98518 4.58477 3.61503
0.02580 4.06597 4.78831 0.61958 0.77255 0.48576 0.95510
136 2.99780 5.58484 1.51269 1.34099 4.87092 3.25682 3.80762
4.39608 2.83303 3.89095 4.74638 2.71832 3.88289 2.97446 3.43530
2.87508 3.28766 3.96404 6.03808 4.52752 159 e - - T
2.68618 4.42225 2.77519 2.73123 3.46354 2.40513 3.72494
3.29354 2.67741 2.69355 4.24690 2.90347 2.73739 3.18146 2.89801
2.37887 2.77519 2.98518 4.58477 3.61503
0.02580 4.06597 4.78831 0.61958 0.77255 0.48576 0.95510
137 3.33120 4.66020 4.85789 4.33286 3.15904 4.57543 4.91062
1.75810 4.15034 0.91082 2.98005 4.59453 4.78813 4.33090 4.28990
3.95360 3.56705 2.13537 5.24141 4.10919 160 l - - S
2.68618 4.42225 2.77519 2.73123 3.46354 2.40513 3.72494
3.29354 2.67741 2.69355 4.24690 2.90347 2.73739 3.18146 2.89801
2.37887 2.77519 2.98518 4.58477 3.61503
0.02580 4.06597 4.78831 0.61958 0.77255 0.48576 0.95510
138 3.04918 5.03175 3.04542 2.80766 4.22524 3.50323 3.96612
3.89013 2.61596 3.37270 4.39941 3.26749 4.06220 0.92537 2.89446
3.09074 3.34803 3.60901 5.46468 4.17314 161 q - - -
2.68618 4.42225 2.77519 2.73123 3.46354 2.40513 3.72494
3.29354 2.67741 2.69355 4.24690 2.90347 2.73739 3.18146 2.89801
2.37887 2.77519 2.98518 4.58477 3.61503
0.02580 4.06597 4.78831 0.61958 0.77255 0.48576 0.95510
139 3.20895 5.35366 0.67572 2.47659 4.75938 3.36761 4.11707
4.39404 3.24020 3.96944 4.95589 3.04114 4.03443 3.35942 3.77958
3.15229 3.55307 4.01393 5.88916 4.60562 162 D - - H
2.68618 4.42225 2.77519 2.73123 3.46354 2.40513 3.72494
3.29354 2.67741 2.69355 4.24690 2.90347 2.73739 3.18146 2.89801
2.37887 2.77519 2.98518 4.58477 3.61503
0.02580 4.06597 4.78831 0.61958 0.77255 0.48576 0.95510
140 1.81762 4.20132 3.52496 3.15618 4.18937 3.02750 4.18523
3.52046 3.15384 3.26293 4.10871 3.33610 3.74241 3.45665 3.47519
1.96231 1.52064 3.05744 5.55475 4.34007 163 t - - H
2.68618 4.42225 2.77519 2.73123 3.46354 2.40513 3.72494
3.29354 2.67741 2.69355 4.24690 2.90347 2.73739 3.18146 2.89801
2.37887 2.77519 2.98518 4.58477 3.61503
0.02580 4.06597 4.78831 0.61958 0.77255 0.48576 0.95510

```
    141   2.52838  4.37237  3.61240  3.37882  4.15210  3.20391  4.37977
3.30338  3.32563  3.15988  4.20136  3.55332  3.92452  3.71164  3.57205
2.73226  0.88151  2.97705  5.57256  4.37008    164 t - - H
          2.68618  4.42225  2.77519  2.73123  3.46354  2.40513  3.72494
3.29354  2.67741  2.69355  4.24690  2.90347  2.73739  3.18146  2.89801
2.37887  2.77519  2.98518  4.58477  3.61503
          0.02580  4.06597  4.78831  0.61958  0.77255  0.48576  0.95510
    142   3.37882  4.78611  4.49943  4.14431  3.22022  4.22585  4.73971
2.43905  3.91022  0.66895  3.19014  4.40099  4.63059  4.23187  4.05293
3.82563  3.66575  2.52585  5.16535  3.93195    165 L - - H
          2.68618  4.42225  2.77519  2.73123  3.46354  2.40513  3.72494
3.29354  2.67741  2.69355  4.24690  2.90347  2.73739  3.18146  2.89801
2.37887  2.77519  2.98518  4.58477  3.61503
          0.02580  4.06597  4.78831  0.61958  0.77255  0.48576  0.95510
    143   2.92343  4.67539  3.63649  3.56258  4.69323  0.48449  4.66708
4.34379  3.76993  3.97234  4.95260  3.80138  4.07247  4.07908  3.98092
3.10155  3.41972  3.83474  5.73323  4.80306    166 G - - H
          2.68618  4.42225  2.77519  2.73123  3.46354  2.40513  3.72494
3.29354  2.67741  2.69355  4.24690  2.90347  2.73739  3.18146  2.89801
2.37887  2.77519  2.98518  4.58477  3.61503
          0.02580  4.06597  4.78831  0.61958  0.77255  0.48576  0.95510
    144   1.98709  4.22116  3.41930  3.18665  4.35162  2.98121  4.28327
3.73083  3.25911  3.46996  4.32625  3.34208  3.74134  3.56319  3.55721
1.03241  2.77407  3.19795  5.71045  4.47678    167 s - - H
          2.68618  4.42225  2.77519  2.73123  3.46354  2.40513  3.72494
3.29354  2.67741  2.69355  4.24690  2.90347  2.73739  3.18146  2.89801
2.37887  2.77519  2.98518  4.58477  3.61503
          0.02580  4.06597  4.78831  0.61958  0.77255  0.48576  0.95510
    145   3.23350  4.64162  4.64454  4.16043  3.17983  4.35479  4.78418
2.16995  3.95765  0.87146  3.03819  4.41190  4.66902  4.20446  4.12278
3.75454  3.49960  2.01014  5.21235  4.03473    168 l - - H
          2.68618  4.42225  2.77519  2.73123  3.46354  2.40513  3.72494
3.29354  2.67741  2.69355  4.24690  2.90347  2.73739  3.18146  2.89801
2.37887  2.77519  2.98518  4.58477  3.61503
          0.02580  4.06597  4.78831  0.61958  0.77255  0.48576  0.95510
    146   3.37882  4.78611  4.49943  4.14431  3.22022  4.22585  4.73971
2.43905  3.91022  0.66895  3.19014  4.40099  4.63059  4.23187  4.05293
3.82563  3.66575  2.52585  5.16535  3.93195    169 L - - H
          2.68618  4.42225  2.77519  2.73123  3.46354  2.40513  3.72494
3.29354  2.67741  2.69355  4.24690  2.90347  2.73739  3.18146  2.89801
2.37887  2.77519  2.98518  4.58477  3.61503
          0.02580  4.06597  4.78831  0.61958  0.77255  0.48576  0.95510
    147   2.42075  4.34145  3.38622  3.21538  4.27507  3.06832  4.31764
3.82606  3.30239  3.53276  4.44528  3.40582  3.82400  3.63716  3.57937
0.83261  2.91617  3.31815  5.63277  4.36086    170 s - - H
          2.68618  4.42225  2.77519  2.73123  3.46354  2.40513  3.72494
3.29354  2.67741  2.69355  4.24690  2.90347  2.73739  3.18146  2.89801
2.37887  2.77519  2.98518  4.58477  3.61503
          0.02580  4.06597  4.78831  0.61958  0.77255  0.48576  0.95510
    148   1.07946  4.17606  3.53521  3.26980  4.30234  2.97433  4.30860
3.63954  3.31167  3.40256  4.25778  3.38075  3.73499  3.60234  3.59807
1.87052  2.74784  3.12638  5.67501  4.46400    171 a - - H
          2.68618  4.42225  2.77519  2.73123  3.46354  2.40513  3.72494
3.29354  2.67741  2.69355  4.24690  2.90347  2.73739  3.18146  2.89801
2.37887  2.77519  2.98518  4.58477  3.61503
          0.02580  4.06597  4.78831  0.61958  0.77255  0.48576  0.95510
```

149 3.37882 4.78611 4.49943 4.14431 3.22022 4.22585 4.73971
2.43905 3.91022 0.66895 3.19014 4.40099 4.63059 4.23187 4.05293
3.82563 3.66575 2.52585 5.16535 3.93195 172 L - - H
2.68618 4.42225 2.77519 2.73123 3.46354 2.40513 3.72494
3.29354 2.67741 2.69355 4.24690 2.90347 2.73739 3.18146 2.89801
2.37887 2.77519 2.98518 4.58477 3.61503
0.02580 4.06597 4.78831 0.61958 0.77255 0.48576 0.95510
150 3.14642 4.51266 4.68899 4.12274 2.65139 4.35136 4.62194
1.86938 3.99650 1.56661 1.61601 4.35330 4.59369 4.12931 4.13257
3.67897 3.37213 2.25407 5.01987 3.86071 173 l - - S
2.68618 4.42225 2.77519 2.73123 3.46354 2.40513 3.72494
3.29354 2.67741 2.69355 4.24690 2.90347 2.73739 3.18146 2.89801
2.37887 2.77519 2.98518 4.58477 3.61503
0.02580 4.06597 4.78831 0.61958 0.77255 0.48576 0.95510
151 2.79329 4.92869 2.86760 2.56655 4.30974 3.38554 3.79845
3.80948 2.47889 3.34960 4.24086 3.05036 3.05729 1.33526 2.82064
2.82271 3.08339 3.46117 5.51326 4.19774 174 q - - S
2.68618 4.42225 2.77519 2.73123 3.46354 2.40513 3.72494
3.29354 2.67741 2.69355 4.24690 2.90347 2.73739 3.18146 2.89801
2.37887 2.77519 2.98518 4.58477 3.61503
0.02580 4.06597 4.78831 0.61958 0.77255 0.48576 0.95510
152 3.22923 5.00496 3.33584 3.11929 3.43719 3.62566 0.79230
3.96225 2.95784 3.42994 4.49131 3.51913 4.19088 3.48923 3.20982
3.29938 3.54090 3.69387 4.89871 3.38345 175 h - - S
2.68618 4.42225 2.77519 2.73123 3.46354 2.40513 3.72494
3.29354 2.67741 2.69355 4.24690 2.90347 2.73739 3.18146 2.89801
2.37887 2.77519 2.98518 4.58477 3.61503
0.02580 4.06597 4.78831 0.61958 0.77255 0.48576 0.95510
153 2.82616 0.62012 4.38432 4.17110 4.12291 3.40456 4.77610
3.30307 4.00070 3.20778 4.33683 4.10703 4.12629 4.33906 4.08927
3.09758 3.30953 3.03349 5.49328 4.40808 176 C - - S
2.68618 4.42225 2.77519 2.73123 3.46354 2.40513 3.72494
3.29354 2.67741 2.69355 4.24690 2.90347 2.73739 3.18146 2.89801
2.37887 2.77519 2.98518 4.58477 3.61503
0.02580 4.06597 4.78831 0.61958 0.77255 0.48576 0.95510
154 2.72827 5.15919 1.85066 1.96758 4.45936 3.32079 3.66431
3.92494 2.51321 3.45628 4.25059 2.53533 3.81380 2.79956 3.02933
2.50872 2.98034 2.97179 5.63575 4.21908 177 d - - S
2.68618 4.42225 2.77519 2.73123 3.46354 2.40513 3.72494
3.29354 2.67741 2.69355 4.24690 2.90347 2.73739 3.18146 2.89801
2.37887 2.77519 2.98518 4.58477 3.61503
0.02580 4.06597 4.78831 0.61958 0.77255 0.48576 0.95510
155 3.02861 4.74922 3.65739 3.52739 4.49912 3.42598 4.58547
4.09872 3.60213 3.70274 4.76356 3.81267 0.53536 3.98492 3.82221
3.20306 3.48618 3.72125 5.62838 4.62373 178 P - - S
2.68618 4.42225 2.77519 2.73123 3.46354 2.40513 3.72494
3.29354 2.67741 2.69355 4.24690 2.90347 2.73739 3.18146 2.89801
2.37887 2.77519 2.98518 4.58477 3.61503
0.02580 4.06597 4.78831 0.61958 0.77255 0.48576 0.95510
156 2.72426 4.72018 2.96616 2.74679 4.21529 3.30724 3.97662
3.73894 2.76689 3.32115 4.25872 3.17570 1.22587 2.74798 3.09580
2.81530 3.08705 3.38315 5.50823 4.21601 179 p - - -
2.68618 4.42225 2.77519 2.73123 3.46354 2.40513 3.72494
3.29354 2.67741 2.69355 4.24690 2.90347 2.73739 3.18146 2.89801
2.37887 2.77519 2.98518 4.58477 3.61503
0.02580 4.06597 4.78831 0.61958 0.77255 0.48576 0.95510

157 3.04918 5.03175 3.04542 2.80766 4.22524 3.50323 3.96612
3.89013 2.61596 3.37270 4.39941 3.26749 4.06220 0.92537 2.89446
3.09074 3.34803 3.60901 5.46468 4.17314 180 q - - T
2.68618 4.42225 2.77519 2.73123 3.46354 2.40513 3.72494
3.29354 2.67741 2.69355 4.24690 2.90347 2.73739 3.18146 2.89801
2.37887 2.77519 2.98518 4.58477 3.61503
0.02580 4.06597 4.78831 0.61958 0.77255 0.48576 0.95510
158 3.23578 5.05316 3.74734 3.22314 4.45741 3.68117 3.98214
4.01613 2.33942 3.49703 4.49575 3.56619 4.17759 3.19599 0.71938
3.31284 3.49063 3.73260 5.46392 4.34662 181 r - - T
2.68618 4.42225 2.77519 2.73123 3.46354 2.40513 3.72494
3.29354 2.67741 2.69355 4.24690 2.90347 2.73739 3.18146 2.89801
2.37887 2.77519 2.98518 4.58477 3.61503
0.02580 4.06597 4.78831 0.61958 0.77255 0.48576 0.95510
159 2.87347 5.04885 3.24471 2.65776 4.46369 3.56177 3.63718
3.84507 1.77668 3.34443 4.18207 3.10797 3.95916 2.77338 1.58373
2.36272 3.08072 3.50466 5.45237 4.21536 182 r - - S
2.68618 4.42225 2.77519 2.73123 3.46354 2.40513 3.72494
3.29354 2.67741 2.69355 4.24690 2.90347 2.73739 3.18146 2.89801
2.37887 2.77519 2.98518 4.58477 3.61503
0.02580 4.06597 4.78831 0.61958 0.77255 0.48576 0.95510
160 3.64795 4.88999 4.65618 4.34275 0.96674 4.41106 3.52645
3.29712 4.18714 2.61985 3.89772 4.11014 4.72200 4.16139 4.21988
3.78470 3.87235 3.23599 3.66803 1.54733 183 f - - -
2.68618 4.42225 2.77519 2.73123 3.46354 2.40513 3.72494
3.29354 2.67741 2.69355 4.24690 2.90347 2.73739 3.18146 2.89801
2.37887 2.77519 2.98518 4.58477 3.61503
0.02580 4.06597 4.78831 0.61958 0.77255 0.48576 0.95510
161 2.71249 4.52860 3.47622 3.10776 3.71109 3.45216 4.09778
3.04731 2.99624 2.11190 3.79269 3.45306 1.39736 3.41082 3.29474
2.89854 3.06242 2.85240 5.23167 3.93481 184 p - - -
2.68618 4.42225 2.77519 2.73123 3.46354 2.40513 3.72494
3.29354 2.67741 2.69355 4.24690 2.90347 2.73739 3.18146 2.89801
2.37887 2.77519 2.98518 4.58477 3.61503
0.02580 4.06597 4.78831 0.61958 0.77255 0.48576 0.95510
162 3.37882 4.78611 4.49943 4.14431 3.22022 4.22585 4.73971
2.43905 3.91022 0.66895 3.19014 4.40099 4.63059 4.23187 4.05293
3.82563 3.66575 2.52585 5.16535 3.93195 185 L - - T
2.68618 4.42225 2.77519 2.73123 3.46354 2.40513 3.72494
3.29354 2.67741 2.69355 4.24690 2.90347 2.73739 3.18146 2.89801
2.37887 2.77519 2.98518 4.58477 3.61503
0.02580 4.06597 4.78831 0.61958 0.77255 0.48576 0.95510
163 2.98459 5.52735 1.85722 1.14113 4.82021 3.26702 3.80780
4.33382 2.81244 3.84384 4.70052 2.73792 3.88554 2.97534 3.39433
2.87281 3.27284 3.91347 5.99052 4.50133 186 e - - T
2.68618 4.42225 2.77519 2.73123 3.46354 2.40513 3.72494
3.29354 2.67741 2.69355 4.24690 2.90347 2.73739 3.18146 2.89801
2.37887 2.77519 2.98518 4.58477 3.61503
0.02580 4.06597 4.78831 0.61958 0.77255 0.48576 0.95510
164 3.09940 5.20848 3.49475 2.83744 4.67259 3.69994 3.64827
4.02166 1.11596 3.45692 4.33148 3.23649 4.07278 2.78361 1.79263
3.09288 3.26565 3.70263 5.48932 4.32647 187 k - - T
2.68618 4.42225 2.77519 2.73123 3.46354 2.40513 3.72494
3.29354 2.67741 2.69355 4.24690 2.90347 2.73739 3.18146 2.89801
2.37887 2.77519 2.98518 4.58477 3.61503
0.02580 4.06597 4.78831 0.61958 0.77255 0.48576 0.95510

```
    165   2.92343  4.67539  3.63649  3.56258  4.69323  0.48449  4.66708
4.34379  3.76993  3.97234  4.95260  3.80138  4.07247  4.07908  3.98092
3.10155  3.41972  3.83474  5.73323  4.80306    188 G - - -
          2.68618  4.42225  2.77519  2.73123  3.46354  2.40513  3.72494
3.29354  2.67741  2.69355  4.24690  2.90347  2.73739  3.18146  2.89801
2.37887  2.77519  2.98518  4.58477  3.61503
          0.02580  4.06597  4.78831  0.61958  0.77255  0.48576  0.95510
    166   2.98768  4.36983  4.53060  3.98584  3.43922  4.23207  4.66350
1.82481  3.86894  1.61349  3.28119  4.23417  4.55320  4.11887  4.07746
3.56830  2.92121  1.30738  5.27179  4.09423    189 v - - -
          2.68618  4.42225  2.77519  2.73123  3.46354  2.40513  3.72494
3.29354  2.67741  2.69355  4.24690  2.90347  2.73739  3.18146  2.89801
2.37887  2.77519  2.98518  4.58477  3.61503
          0.02580  4.06597  4.78831  0.61958  0.77255  0.48576  0.95510
    167   1.83881  4.27104  3.38711  3.04354  4.20626  3.06597  4.13219
3.51438  3.06128  3.25449  4.11738  3.28738  1.63937  3.38423  3.39819
2.49602  2.38486  3.07397  5.56332  4.33248    190 p - - H
          2.68618  4.42225  2.77519  2.73123  3.46354  2.40513  3.72494
3.29354  2.67741  2.69355  4.24690  2.90347  2.73739  3.18146  2.89801
2.37887  2.77519  2.98518  4.58477  3.61503
          0.02580  4.06597  4.78831  0.61958  0.77255  0.48576  0.95510
    168   3.02861  4.74922  3.65739  3.52739  4.49912  3.42598  4.58547
4.09872  3.60213  3.70274  4.76356  3.81267  0.53536  3.98492  3.82221
3.20306  3.48618  3.72125  5.62838  4.62373    191 P - - H
          2.68618  4.42225  2.77519  2.73123  3.46354  2.40513  3.72494
3.29354  2.67741  2.69355  4.24690  2.90347  2.73739  3.18146  2.89801
2.37887  2.77519  2.98518  4.58477  3.61503
          0.02580  4.06597  4.78831  0.61958  0.77255  0.48576  0.95510
    169   3.02861  4.74922  3.65739  3.52739  4.49912  3.42598  4.58547
4.09872  3.60213  3.70274  4.76356  3.81267  0.53536  3.98492  3.82221
3.20306  3.48618  3.72125  5.62838  4.62373    192 P - - H
          2.68618  4.42225  2.77519  2.73123  3.46354  2.40513  3.72494
3.29354  2.67741  2.69355  4.24690  2.90347  2.73739  3.18146  2.89801
2.37887  2.77519  2.98518  4.58477  3.61503
          0.02580  4.06597  4.78831  0.61958  0.77255  0.48576  0.95510
    170   3.67704  4.97923  4.39985  4.15673  2.89117  3.95783  4.15897
3.71339  3.87713  3.07301  4.33751  4.26783  4.49253  4.25464  3.97000
3.87873  3.98817  3.60894  0.57145  2.88383    193 W - - H
          2.68618  4.42225  2.77519  2.73123  3.46354  2.40513  3.72494
3.29354  2.67741  2.69355  4.24690  2.90347  2.73739  3.18146  2.89801
2.37887  2.77519  2.98518  4.58477  3.61503
          0.02580  4.06597  4.78831  0.61958  0.77255  0.48576  0.95510
    171   3.67704  4.97923  4.39985  4.15673  2.89117  3.95783  4.15897
3.71339  3.87713  3.07301  4.33751  4.26783  4.49253  4.25464  3.97000
3.87873  3.98817  3.60894  0.57145  2.88383    194 W - - -
          2.68618  4.42225  2.77519  2.73123  3.46354  2.40513  3.72494
3.29354  2.67741  2.69355  4.24690  2.90347  2.73739  3.18146  2.89801
2.37887  2.77519  2.98518  4.58477  3.61503
          0.02580  4.06597  4.78831  0.61958  0.77255  0.48576  0.95510
    172   3.02861  4.74922  3.65739  3.52739  4.49912  3.42598  4.58547
4.09872  3.60213  3.70274  4.76356  3.81267  0.53536  3.98492  3.82221
3.20306  3.48618  3.72125  5.62838  4.62373    195 P - - -
          2.68618  4.42225  2.77519  2.73123  3.46354  2.40513  3.72494
3.29354  2.67741  2.69355  4.24690  2.90347  2.73739  3.18146  2.89801
2.37887  2.77519  2.98518  4.58477  3.61503
          0.02580  4.06597  4.78831  0.61958  0.77255  0.48576  0.95510
```

```
    173   2.59354  4.75465  2.90003  2.18223  4.08803  3.35718  3.68571
3.49404  2.50164  3.11185  3.93503  2.64270  3.81373  2.84894  2.95064
2.37496  2.08691  2.94271  5.35851  4.02964    196 t - - -
          2.68618  4.42225  2.77519  2.73123  3.46354  2.40513  3.72494
3.29354  2.67741  2.69355  4.24690  2.90347  2.73739  3.18146  2.89801
2.37887  2.77519  2.98518  4.58477  3.61503
          0.02580  4.06597  4.78831  0.61958  0.77255  0.48576  0.95510
    174   2.92343  4.67539  3.63649  3.56258  4.69323  0.48449  4.66708
4.34379  3.76993  3.97234  4.95260  3.80138  4.07247  4.07908  3.98092
3.10155  3.41972  3.83474  5.73323  4.80306    197 G - - S
          2.68608  4.42237  2.77531  2.73135  3.46365  2.40508  3.72506
3.29326  2.67752  2.69366  4.24701  2.90358  2.73751  3.18158  2.89759
2.37898  2.77508  2.98472  4.58488  3.61515
          0.12272  2.23340  4.78831  1.57603  0.23167  0.48576  0.95510
    175   2.63447  5.07843  2.52220  2.17815  4.39706  3.04592  3.58490
3.86343  2.12307  3.36955  4.13365  2.55980  3.78238  2.69708  2.48705
2.60303  2.61117  3.45629  5.52353  4.12875    206 k - - -
          2.68618  4.42225  2.77519  2.73123  3.46354  2.40513  3.72494
3.29354  2.67741  2.69355  4.24690  2.90347  2.73739  3.18146  2.89801
2.37887  2.77519  2.98518  4.58477  3.61503
          0.02580  4.06597  4.78831  0.61958  0.77255  0.48576  0.95510
    176   2.26458  5.02592  2.53448  1.27614  4.46101  3.29878  3.81656
3.80295  2.68073  3.45756  4.33561  2.89886  3.87608  2.98960  3.13744
2.78357  3.09140  3.44630  5.69832  4.32349    207 e - - -
          2.68618  4.42225  2.77519  2.73123  3.46354  2.40513  3.72494
3.29354  2.67741  2.69355  4.24690  2.90347  2.73739  3.18146  2.89801
2.37887  2.77519  2.98518  4.58477  3.61503
          0.02580  4.06597  4.78831  0.61958  0.77255  0.48576  0.95510
    177   2.46235  4.91895  2.31177  2.08305  4.18173  3.37239  3.64298
3.60072  2.46132  2.46891  4.00341  2.88610  3.32914  2.78869  2.94121
2.64988  2.88587  3.25873  5.42059  4.05906    208 e - - H
          2.68618  4.42225  2.77519  2.73123  3.46354  2.40513  3.72494
3.29354  2.67741  2.69355  4.24690  2.90347  2.73739  3.18146  2.89801
2.37887  2.77519  2.98518  4.58477  3.61503
          0.02580  4.06597  4.78831  0.61958  0.77255  0.48576  0.95510
    178   2.76354  4.42895  3.80573  3.35433  3.05611  3.59310  3.93632
2.97028  3.10668  2.59187  3.64876  3.59583  4.10125  3.51433  3.33964
2.99729  2.36650  2.77821  1.52011  3.14636    209 w - - H
          2.68618  4.42225  2.77519  2.73123  3.46354  2.40513  3.72494
3.29354  2.67741  2.69355  4.24690  2.90347  2.73739  3.18146  2.89801
2.37887  2.77519  2.98518  4.58477  3.61503
          0.02580  4.06597  4.78831  0.61958  0.77255  0.48576  0.95510
    179   3.04084  4.50546  4.25909  3.75961  2.69035  4.00205  3.96328
2.14519  3.50407  2.15354  3.37938  3.92110  4.37573  3.80338  3.67834
3.36758  3.28991  2.57056  1.54294  2.82348    210 w - - H
          2.68618  4.42225  2.77519  2.73123  3.46354  2.40513  3.72494
3.29354  2.67741  2.69355  4.24690  2.90347  2.73739  3.18146  2.89801
2.37887  2.77519  2.98518  4.58477  3.61503
          0.02580  4.06597  4.78831  0.61958  0.77255  0.48576  0.95510
    180   2.27631  4.58612  2.79767  2.56170  3.86987  2.54219  3.70864
2.83199  2.56898  2.91025  3.75548  3.04201  3.20633  2.90306  3.00588
2.64385  2.81152  2.77690  5.20528  3.90808    211 a - - H
          2.68618  4.42225  2.77519  2.73123  3.46354  2.40513  3.72494
3.29354  2.67741  2.69355  4.24690  2.90347  2.73739  3.18146  2.89801
2.37887  2.77519  2.98518  4.58477  3.61503
          0.02580  4.06597  4.78831  0.61958  0.77255  0.48576  0.95510
```

```
    181   2.34327  4.87593  2.90184  2.14950  4.14469  3.17339  3.61038
3.57216  2.29178  2.89350  3.95064  2.91220  3.80021  2.45206  2.84127
2.49663  2.65312  3.22693  5.36649  4.01714    212 e - - H
          2.68618  4.42225  2.77519  2.73123  3.46354  2.40513  3.72494
3.29354  2.67741  2.69355  4.24690  2.90347  2.73739  3.18146  2.89801
2.37887  2.77519  2.98518  4.58477  3.61503
          0.02580  4.06597  4.78831  0.61958  0.77255  0.48576  0.95510
    182   2.67841  4.61799  3.17977  2.69104  3.74874  3.50269  3.76296
3.13452  2.55082  1.95585  3.68255  3.15116  3.01222  2.26187  2.90590
2.79341  2.92333  2.90939  5.15066  3.86709    213 l - - H
          2.68618  4.42225  2.77519  2.73123  3.46354  2.40513  3.72494
3.29354  2.67741  2.69355  4.24690  2.90347  2.73739  3.18146  2.89801
2.37887  2.77519  2.98518  4.58477  3.61503
          0.02580  4.06597  4.78831  0.61958  0.77255  0.48576  0.95510
    183   1.82173  4.20311  3.38328  3.15350  4.42510  1.33210  4.28143
3.84517  3.28542  3.53307  4.34952  3.31375  3.71562  3.55099  3.60277
2.09592  2.74702  3.25720  5.75697  4.54541    214 g - - T
          2.68618  4.42225  2.77519  2.73123  3.46354  2.40513  3.72494
3.29354  2.67741  2.69355  4.24690  2.90347  2.73739  3.18146  2.89801
2.37887  2.77519  2.98518  4.58477  3.61503
          0.29602  4.06597  1.43095  0.61958  0.77255  0.48576  0.95510
    184   2.24733  4.71170  2.62890  1.97376  4.14633  3.20727  3.68803
3.52010  2.52767  3.16253  4.01588  2.86097  2.75280  2.86743  2.96004
2.60997  2.86862  3.17180  5.41729  4.08432    215 e - - .
          2.68618  4.42225  2.77519  2.73123  3.46354  2.40513  3.72494
3.29354  2.67741  2.69355  4.24690  2.90347  2.73739  3.18146  2.89801
2.37887  2.77519  2.98518  4.58477  3.61503
          0.03367  3.80362  4.52597  0.61958  0.77255  0.36589  1.18279
    185   2.26285  4.25055  4.10889  3.54992  3.40018  3.88961  4.27230
1.94526  3.45709  1.79183  3.31050  3.83202  4.26066  3.72176  3.70702
3.19317  2.71146  1.76490  5.03905  3.84168    216 v - - -
          2.68618  4.42225  2.77519  2.73123  3.46354  2.40513  3.72494
3.29354  2.67741  2.69355  4.24690  2.90347  2.73739  3.18146  2.89801
2.37887  2.77519  2.98518  4.58477  3.61503
          0.02580  4.06597  4.78831  0.61958  0.77255  0.48576  0.95510
    186   2.28490  4.67292  2.94097  2.53471  4.18195  3.28011  3.76239
3.60257  2.56914  3.21073  4.03316  3.00561  2.24237  2.41532  2.99219
2.24476  2.85930  3.22447  5.44239  4.12577    217 p - - -
          2.68618  4.42225  2.77519  2.73123  3.46354  2.40513  3.72494
3.29354  2.67741  2.69355  4.24690  2.90347  2.73739  3.18146  2.89801
2.37887  2.77519  2.98518  4.58477  3.61503
          0.02580  4.06597  4.78831  0.61958  0.77255  0.48576  0.95510
    187   2.28798  4.40858  3.29150  2.72968  3.11349  3.50222  3.74831
2.92679  2.36501  2.63241  3.22925  2.98782  3.88967  3.01634  3.07897
2.73798  2.48678  2.49951  4.98275  3.72632    218 a - - T
          2.68618  4.42225  2.77519  2.73123  3.46354  2.40513  3.72494
3.29354  2.67741  2.69355  4.24690  2.90347  2.73739  3.18146  2.89801
2.37887  2.77519  2.98518  4.58477  3.61503
          0.02580  4.06597  4.78831  0.61958  0.77255  0.48576  0.95510
    188   2.63337  4.97405  2.33718  2.15806  4.25656  2.91767  3.22113
3.70195  2.41792  3.25853  4.05008  2.86333  3.79473  2.75038  2.90498
2.34775  2.87272  2.81633  5.45837  4.08184    219 e - - T
          2.68618  4.42225  2.77519  2.73123  3.46354  2.40513  3.72494
3.29354  2.67741  2.69355  4.24690  2.90347  2.73739  3.18146  2.89801
2.37887  2.77519  2.98518  4.58477  3.61503
          0.02580  4.06597  4.78831  0.61958  0.77255  0.48576  0.95510
```

189 2.63088 4.49506 3.29222 2.73298 3.06652 3.54018 3.17190
2.80526 2.65770 2.33341 3.55384 3.18312 3.92096 2.16932 3.04036
2.77651 2.86070 2.77186 4.91273 3.56859 220 q - - -
2.68618 4.42225 2.77519 2.73123 3.46354 2.40513 3.72494
3.29354 2.67741 2.69355 4.24690 2.90347 2.73739 3.18146 2.89801
2.37887 2.77519 2.98518 4.58477 3.61503
0.02580 4.06597 4.78831 0.61958 0.77255 0.48576 0.95510
190 2.63012 4.78896 2.93759 2.49521 4.14639 2.09916 2.90611
3.59083 2.43474 3.18075 4.00532 2.98746 3.83504 2.85674 2.56411
2.36925 2.89754 3.24214 5.38430 4.05927 221 g - - -
2.68580 4.42230 2.77524 2.73128 3.46358 2.40507 3.72499
3.29359 2.67745 2.69359 4.24694 2.90351 2.73744 3.18151 2.89805
2.37891 2.77524 2.98505 4.58481 3.61508
0.32580 1.81959 2.15453 0.66454 0.72259 0.48576 0.95510
191 2.26617 4.36242 3.33267 2.90279 3.72670 3.31429 3.93546
2.94423 2.83260 2.21862 3.68074 3.26225 2.05804 3.20620 3.17005
2.68310 2.84224 2.69983 5.19486 3.93141 224 p - - .
2.68618 4.42225 2.77519 2.73123 3.46354 2.40513 3.72494
3.29354 2.67741 2.69355 4.24690 2.90347 2.73739 3.18146 2.89801
2.37887 2.77519 2.98518 4.58477 3.61503
0.02896 3.95209 4.67444 0.61958 0.77255 0.42125 1.06777
192 2.74655 4.73025 3.23118 2.83720 4.24683 3.39039 3.90383
3.71125 2.53842 3.29812 4.19558 3.24310 1.38250 3.10970 2.18816
2.84016 3.07081 3.36126 5.44902 4.21439 225 p - - -
2.68618 4.42225 2.77519 2.73123 3.46354 2.40513 3.72494
3.29354 2.67741 2.69355 4.24690 2.90347 2.73739 3.18146 2.89801
2.37887 2.77519 2.98518 4.58477 3.61503
0.02580 4.06597 4.78831 0.61958 0.77255 0.48576 0.95510
193 3.02861 4.74922 3.65739 3.52739 4.49912 3.42598 4.58547
4.09872 3.60213 3.70274 4.76356 3.81267 0.53536 3.98492 3.82221
3.20306 3.48618 3.72125 5.62838 4.62373 226 P - - -
2.68618 4.42225 2.77519 2.73123 3.46354 2.40513 3.72494
3.29354 2.67741 2.69355 4.24690 2.90347 2.73739 3.18146 2.89801
2.37887 2.77519 2.98518 4.58477 3.61503
0.02580 4.06597 4.78831 0.61958 0.77255 0.48576 0.95510
194 2.99234 4.72381 3.61321 3.12909 2.83024 3.75600 3.68686
3.30076 2.72735 2.85005 3.86966 3.45753 4.16871 3.26850 2.33964
3.11910 3.22975 3.09407 4.40190 1.35052 227 y - - -
2.68618 4.42225 2.77519 2.73123 3.46354 2.40513 3.72494
3.29354 2.67741 2.69355 4.24690 2.90347 2.73739 3.18146 2.89801
2.37887 2.77519 2.98518 4.58477 3.61503
0.02580 4.06597 4.78831 0.61958 0.77255 0.48576 0.95510
195 3.13038 5.21914 3.57764 2.87833 4.69498 3.72825 3.64447
4.03156 1.48835 3.45898 4.33716 3.26308 4.09044 2.77941 1.27476
3.12505 3.28725 3.71796 5.48423 4.33688 228 r - - -
2.68618 4.42225 2.77519 2.73123 3.46354 2.40513 3.72494
3.29354 2.67741 2.69355 4.24690 2.90347 2.73739 3.18146 2.89801
2.37887 2.77519 2.98518 4.58477 3.61503
0.02580 4.06597 4.78831 0.61958 0.77255 0.48576 0.95510
196 2.97750 5.14370 3.15515 2.66483 4.37662 3.58834 2.73039
3.92706 1.21060 3.40371 4.26778 3.11972 4.00262 2.81342 2.32966
2.96931 3.17690 3.59863 5.42084 4.13460 229 k - - -
2.68618 4.42225 2.77519 2.73123 3.46354 2.40513 3.72494
3.29354 2.67741 2.69355 4.24690 2.90347 2.73739 3.18146 2.89801
2.37887 2.77519 2.98518 4.58477 3.61503
0.02580 4.06597 4.78831 0.61958 0.77255 0.48576 0.95510

```
    197   3.02861  4.74922  3.65739  3.52739  4.49912  3.42598  4.58547
4.09872  3.60213  3.70274  4.76356  3.81267  0.53536  3.98492  3.82221
3.20306  3.48618  3.72125  5.62838  4.62373    230 P - - G
          2.68618  4.42225  2.77519  2.73123  3.46354  2.40513  3.72494
3.29354  2.67741  2.69355  4.24690  2.90347  2.73739  3.18146  2.89801
2.37887  2.77519  2.98518  4.58477  3.61503
          0.02580  4.06597  4.78831  0.61958  0.77255  0.48576  0.95510
    198   2.94509  4.98735  3.23397  2.75445  3.91918  3.58622  1.55179
3.75164  2.22266  3.26705  4.16656  3.18932  4.01923  2.91701  2.06606
2.97717  3.16781  3.45177  5.18323  3.79854    231 h - - G
          2.68618  4.42225  2.77519  2.73123  3.46354  2.40513  3.72494
3.29354  2.67741  2.69355  4.24690  2.90347  2.73739  3.18146  2.89801
2.37887  2.77519  2.98518  4.58477  3.61503
          0.02580  4.06597  4.78831  0.61958  0.77255  0.48576  0.95510
    199   2.93451  5.35948  1.21850  2.22566  4.44553  3.30007  2.69273
4.17637  2.75965  3.68902  4.55058  2.79690  3.89126  2.97348  3.29551
2.85487  3.21737  3.78139  5.71636  4.22405    232 d - - G
          2.68618  4.42225  2.77519  2.73123  3.46354  2.40513  3.72494
3.29354  2.67741  2.69355  4.24690  2.90347  2.73739  3.18146  2.89801
2.37887  2.77519  2.98518  4.58477  3.61503
          0.02580  4.06597  4.78831  0.61958  0.77255  0.48576  0.95510
    200   2.73098  4.42202  3.67834  3.30509  3.53013  2.53674  4.17643
2.79443  3.19649  1.18045  3.57849  3.60014  4.08592  3.56653  3.45564
2.98522  3.06412  2.63757  5.11106  3.85167    233 l - - -
          2.68618  4.42225  2.77519  2.73123  3.46354  2.40513  3.72494
3.29354  2.67741  2.69355  4.24690  2.90347  2.73739  3.18146  2.89801
2.37887  2.77519  2.98518  4.58477  3.61503
          0.02580  4.06597  4.78831  0.61958  0.77255  0.48576  0.95510
    201   3.10998  5.22177  3.53163  2.84768  4.69502  3.71326  3.64061
4.03614  1.19317  3.46284  4.33539  3.24254  4.07754  2.77371  1.63672
3.10104  3.27025  3.71680  5.48977  4.33315    234 k - - -
          2.68618  4.42225  2.77519  2.73123  3.46354  2.40513  3.72494
3.29354  2.67741  2.69355  4.24690  2.90347  2.73739  3.18146  2.89801
2.37887  2.77519  2.98518  4.58477  3.61503
          0.02580  4.06597  4.78831  0.61958  0.77255  0.48576  0.95510
    202   3.09733  5.20517  3.48614  2.83557  4.66713  3.69679  3.65052
4.01821  1.10348  3.45562  4.33091  3.23547  4.07192  2.78650  1.82212
3.09149  3.26510  3.69936  5.48927  4.32502    235 k - - H
          2.68618  4.42225  2.77519  2.73123  3.46354  2.40513  3.72494
3.29354  2.67741  2.69355  4.24690  2.90347  2.73739  3.18146  2.89801
2.37887  2.77519  2.98518  4.58477  3.61503
          0.02580  4.06597  4.78831  0.61958  0.77255  0.48576  0.95510
    203   1.96529  4.36307  3.45189  2.88550  3.51814  3.55925  3.83709
2.75731  2.78481  2.33488  3.17921  3.29723  3.95357  3.13539  2.67023
2.81657  2.84768  2.31284  4.97714  3.73957    236 a - - H
          2.68618  4.42225  2.77519  2.73123  3.46354  2.40513  3.72494
3.29354  2.67741  2.69355  4.24690  2.90347  2.73739  3.18146  2.89801
2.37887  2.77519  2.98518  4.58477  3.61503
          0.02580  4.06597  4.78831  0.61958  0.77255  0.48576  0.95510
    204   3.27178  4.81327  3.96106  3.53614  2.35473  3.98758  2.49001
3.42877  3.21044  2.88264  3.98668  3.71828  4.38383  3.61607  3.41637
3.39104  3.50824  3.25569  1.33329  2.30432    237 w - - H
          2.68618  4.42225  2.77519  2.73123  3.46354  2.40513  3.72494
3.29354  2.67741  2.69355  4.24690  2.90347  2.73739  3.18146  2.89801
2.37887  2.77519  2.98518  4.58477  3.61503
          0.02580  4.06597  4.78831  0.61958  0.77255  0.48576  0.95510
```

205 2.85038 4.99180 3.06515 2.63955 4.44653 3.47323 3.73570
3.85457 1.23484 3.39140 4.25197 3.10221 2.78613 2.89041 2.53976
2.87180 3.10410 3.50397 5.51151 4.25702 238 k - - H
2.68618 4.42225 2.77519 2.73123 3.46354 2.40513 3.72494
3.29354 2.67741 2.69355 4.24690 2.90347 2.73739 3.18146 2.89801
2.37887 2.77519 2.98518 4.58477 3.61503
0.12272 4.06597 2.31928 0.61958 0.77255 0.48576 0.95510
206 3.04806 4.39542 4.67830 4.18766 3.54286 4.33963 4.92295
1.49388 4.06790 2.06597 3.36408 4.41647 4.68154 4.34937 4.27965
3.72750 3.32154 1.08706 5.48620 4.26981 239 v - - H
2.68618 4.42225 2.77519 2.73123 3.46354 2.40513 3.72494
3.29354 2.67741 2.69355 4.24690 2.90347 2.73739 3.18146 2.89801
2.37887 2.77519 2.98518 4.58477 3.61503
0.02839 3.97164 4.69398 0.61958 0.77255 0.58084 0.81968
207 2.25955 4.22907 3.19450 2.98218 4.35390 1.82598 4.16305
3.82435 3.13660 3.49019 4.31593 3.21365 3.68717 3.42262 3.47120
1.41232 2.74457 3.25440 5.67046 4.42997 240 s - - H
2.68618 4.42225 2.77519 2.73123 3.46354 2.40513 3.72494
3.29354 2.67741 2.69355 4.24690 2.90347 2.73739 3.18146 2.89801
2.37887 2.77519 2.98518 4.58477 3.61503
0.02839 3.97164 4.69398 0.61958 0.77255 0.58084 0.81968
208 3.01953 4.42855 4.48895 4.00180 3.39388 4.19569 4.71242
1.90795 3.84132 1.65627 3.26977 4.24751 4.56392 4.14172 4.05304
3.58135 3.29662 1.17592 5.30382 4.07150 241 v - - H
2.68618 4.42225 2.77519 2.73123 3.46354 2.40513 3.72494
3.29354 2.67741 2.69355 4.24690 2.90347 2.73739 3.18146 2.89801
2.37887 2.77519 2.98518 4.58477 3.61503
0.02839 3.97164 4.69398 0.61958 0.77255 0.43059 1.05018
209 3.37882 4.78611 4.49943 4.14431 3.22022 4.22585 4.73971
2.43905 3.91022 0.66895 3.19014 4.40099 4.63059 4.23187 4.05293
3.82563 3.66575 2.52585 5.16535 3.93195 242 L - - H
2.68618 4.42225 2.77519 2.73123 3.46354 2.40513 3.72494
3.29354 2.67741 2.69355 4.24690 2.90347 2.73739 3.18146 2.89801
2.37887 2.77519 2.98518 4.58477 3.61503
0.02580 4.06597 4.78831 0.61958 0.77255 0.48576 0.95510
210 2.22167 4.36815 3.32528 2.80891 3.80363 3.30271 3.85880
2.77597 2.78091 2.85929 3.72407 3.19546 2.92816 3.10648 3.16780
2.22548 2.08834 2.84090 5.18708 3.93151 243 t - - H
2.68618 4.42225 2.77519 2.73123 3.46354 2.40513 3.72494
3.29354 2.67741 2.69355 4.24690 2.90347 2.73739 3.18146 2.89801
2.37887 2.77519 2.98518 4.58477 3.61503
0.02580 4.06597 4.78831 0.61958 0.77255 0.48576 0.95510
211 1.32097 4.19013 3.46169 3.16675 4.35661 2.05644 4.24997
3.77278 3.24435 3.45666 4.27229 3.32257 3.71284 3.51615 3.56794
1.94317 2.72807 3.21011 5.69374 4.48370 244 a - - H
2.68618 4.42225 2.77519 2.73123 3.46354 2.40513 3.72494
3.29354 2.67741 2.69355 4.24690 2.90347 2.73739 3.18146 2.89801
2.37887 2.77519 2.98518 4.58477 3.61503
0.02580 4.06597 4.78831 0.61958 0.77255 0.48576 0.95510
212 2.17634 4.29157 4.15920 3.76839 3.76408 3.63408 4.59771
2.14545 3.67774 2.49102 3.64741 3.91397 4.22967 3.98924 3.91267
3.06409 3.05965 1.08390 5.47518 4.25328 245 v - - H
2.68618 4.42225 2.77519 2.73123 3.46354 2.40513 3.72494
3.29354 2.67741 2.69355 4.24690 2.90347 2.73739 3.18146 2.89801
2.37887 2.77519 2.98518 4.58477 3.61503
0.02580 4.06597 4.78831 0.61958 0.77255 0.48576 0.95510

```
     213   2.77904  4.36491  3.92952  3.49283  3.51196  3.73965  4.31659
 1.26618  3.37432  2.29491  3.45737  3.77359  2.87570  3.71493  3.62820
 3.13176  3.08377  2.16268  5.17573  3.94362    246 i - - H
           2.68618  4.42225  2.77519  2.73123  3.46354  2.40513  3.72494
 3.29354  2.67741  2.69355  4.24690  2.90347  2.73739  3.18146  2.89801
 2.37887  2.77519  2.98518  4.58477  3.61503
           0.02580  4.06597  4.78831  0.61958  0.77255  0.48576  0.95510
     214   2.37628  5.01452  3.07949  2.54668  4.39357  3.49459  3.63216
 3.79092  1.50622  3.31309  4.13497  2.80331  3.90398  2.76682  2.26276
 2.79080  3.00702  3.44080  5.45290  4.17298    247 k - - H
           2.68618  4.42225  2.77519  2.73123  3.46354  2.40513  3.72494
 3.29354  2.67741  2.69355  4.24690  2.90347  2.73739  3.18146  2.89801
 2.37887  2.77519  2.98518  4.58477  3.61503
           0.02580  4.06597  4.78831  0.61958  0.77255  0.48576  0.95510
     215   2.94509  4.98735  3.23397  2.75445  3.91918  3.58622  1.55179
 3.75164  2.22266  3.26705  4.16656  3.18932  4.01923  2.91701  2.06606
 2.97717  3.16781  3.45177  5.18323  3.79854    248 h - - H
           2.68618  4.42225  2.77519  2.73123  3.46354  2.40513  3.72494
 3.29354  2.67741  2.69355  4.24690  2.90347  2.73739  3.18146  2.89801
 2.37887  2.77519  2.98518  4.58477  3.61503
           0.02580  4.06597  4.78831  0.61958  0.77255  0.48576  0.95510
     216   2.34937  4.38479  4.04261  3.51151  3.31227  3.85513  4.27499
 2.36044  3.36135  1.82848  1.72122  3.81002  4.25487  3.66938  3.61044
 3.18399  3.09272  2.28567  5.05509  3.87009    249 m - - T
           2.68618  4.42225  2.77519  2.73123  3.46354  2.40513  3.72494
 3.29354  2.67741  2.69355  4.24690  2.90347  2.73739  3.18146  2.89801
 2.37887  2.77519  2.98518  4.58477  3.61503
           0.02580  4.06597  4.78831  0.61958  0.77255  0.48576  0.95510
     217   1.89857  4.32838  3.41903  2.91237  3.26567  3.35115  3.90258
 2.99014  2.87544  2.55667  3.64546  3.27427  3.87011  3.19468  3.23784
 1.87690  2.80616  2.71959  5.09839  3.82809    250 s - - G
           2.68618  4.42225  2.77519  2.73123  3.46354  2.40513  3.72494
 3.29354  2.67741  2.69355  4.24690  2.90347  2.73739  3.18146  2.89801
 2.37887  2.77519  2.98518  4.58477  3.61503
           0.02580  4.06597  4.78831  0.61958  0.77255  0.48576  0.95510
     218   2.41475  4.36984  3.21204  3.00804  4.30032  3.07697  4.17957
 3.77674  3.10440  3.45566  4.33211  3.28000  1.21287  3.44871  3.42126
 2.19199  2.87986  3.28670  5.63456  4.36959    251 p - - G
           2.68618  4.42225  2.77519  2.73123  3.46354  2.40513  3.72494
 3.29354  2.67741  2.69355  4.24690  2.90347  2.73739  3.18146  2.89801
 2.37887  2.77519  2.98518  4.58477  3.61503
           0.02580  4.06597  4.78831  0.61958  0.77255  0.48576  0.95510
     219   2.69493  5.11890  2.08914  2.27404  4.45156  3.33280  3.64304
 3.92312  2.44134  3.43976  4.22325  2.27261  3.80567  2.77051  2.46499
 2.31478  2.94487  3.51782  5.60227  4.19766    252 d - - G
           2.68618  4.42225  2.77519  2.73123  3.46354  2.40513  3.72494
 3.29354  2.67741  2.69355  4.24690  2.90347  2.73739  3.18146  2.89801
 2.37887  2.77519  2.98518  4.58477  3.61503
           0.02580  4.06597  4.78831  0.61958  0.77255  0.48576  0.95510
     220   2.77034  4.24197  4.14685  3.57876  2.38529  3.87980  4.15709
 1.81247  3.46796  1.83185  3.13354  3.82608  3.07584  3.69236  3.67293
 3.17581  3.00501  2.24059  4.79351  3.55657    253 i - - H
           2.68618  4.42225  2.77519  2.73123  3.46354  2.40513  3.72494
 3.29354  2.67741  2.69355  4.24690  2.90347  2.73739  3.18146  2.89801
 2.37887  2.77519  2.98518  4.58477  3.61503
           0.02580  4.06597  4.78831  0.61958  0.77255  0.48576  0.95510
```

221 2.40683 5.14837 2.03316 1.95772 4.49111 3.02063 3.68313
3.96243 2.54604 3.48984 4.28471 2.80166 3.81313 2.82089 3.06727
2.12275 2.98988 3.55612 5.66801 4.24844 254 e - - H
2.68618 4.42225 2.77519 2.73123 3.46354 2.40513 3.72494
3.29354 2.67741 2.69355 4.24690 2.90347 2.73739 3.18146 2.89801
2.37887 2.77519 2.98518 4.58477 3.61503
0.02580 4.06597 4.78831 0.61958 0.77255 0.48576 0.95510
222 2.90399 5.18287 3.19417 2.59760 4.59540 3.57870 3.60188
3.98091 1.61439 3.42940 4.24920 2.70543 3.95412 2.27944 1.83557
2.87951 3.09243 3.62097 5.50135 4.25360 255 k - - H
2.68618 4.42225 2.77519 2.73123 3.46354 2.40513 3.72494
3.29354 2.67741 2.69355 4.24690 2.90347 2.73739 3.18146 2.89801
2.37887 2.77519 2.98518 4.58477 3.61503
0.02580 4.06597 4.78831 0.61958 0.77255 0.48576 0.95510
223 3.17012 4.47563 4.87369 4.32097 3.31924 4.50699 4.90548
1.29988 4.20743 1.54932 2.71781 4.54497 4.73890 4.36789 4.35150
3.85622 3.40531 1.68241 5.30999 4.19558 256 i - - H
2.68618 4.42225 2.77519 2.73123 3.46354 2.40513 3.72494
3.29354 2.67741 2.69355 4.24690 2.90347 2.73739 3.18146 2.89801
2.37887 2.77519 2.98518 4.58477 3.61503
0.02580 4.06597 4.78831 0.61958 0.77255 0.48576 0.95510
224 2.92464 5.07282 3.30442 2.70515 4.49155 3.59589 3.64367
3.86256 1.83348 3.35422 4.20321 3.14295 3.19808 2.78186 1.38719
2.93341 3.12334 3.53183 5.45311 4.23599 257 r - - H
2.68618 4.42225 2.77519 2.73123 3.46354 2.40513 3.72494
3.29354 2.67741 2.69355 4.24690 2.90347 2.73739 3.18146 2.89801
2.37887 2.77519 2.98518 4.58477 3.61503
0.02580 4.06597 4.78831 0.61958 0.77255 0.48576 0.95510
225 2.73565 5.10174 2.92613 2.14788 4.44992 3.44829 3.59584
3.88135 1.88444 3.37441 4.16426 2.50510 3.85141 2.71403 2.13984
2.71054 2.77456 3.49879 5.50310 4.17034 258 k - - H
2.68618 4.42225 2.77519 2.73123 3.46354 2.40513 3.72494
3.29354 2.67741 2.69355 4.24690 2.90347 2.73739 3.18146 2.89801
2.37887 2.77519 2.98518 4.58477 3.61503
0.02580 4.06597 4.78831 0.61958 0.77255 0.48576 0.95510
226 2.62440 3.72238 3.50709 2.92613 3.44123 3.57499 3.15769
2.84202 2.70882 1.81203 3.44889 3.31529 3.95930 3.12551 2.58534
2.84040 2.85648 2.62429 4.88538 3.62850 259 l - - T
2.68618 4.42225 2.77519 2.73123 3.46354 2.40513 3.72494
3.29354 2.67741 2.69355 4.24690 2.90347 2.73739 3.18146 2.89801
2.37887 2.77519 2.98518 4.58477 3.61503
0.02580 4.06597 4.78831 0.61958 0.77255 0.48576 0.95510
227 2.36229 4.42977 3.32950 2.44010 3.72997 3.53210 3.98976
2.61161 2.92467 2.66345 3.64421 3.33493 4.01022 3.26926 3.28080
2.87499 2.94152 1.54563 5.21492 3.96158 260 v - - T
2.68618 4.42225 2.77519 2.73123 3.46354 2.40513 3.72494
3.29354 2.67741 2.69355 4.24690 2.90347 2.73739 3.18146 2.89801
2.37887 2.77519 2.98518 4.58477 3.61503
0.02580 4.06597 4.78831 0.61958 0.77255 0.48576 0.95510
228 2.76777 4.83191 3.22054 2.28937 3.99375 3.54644 3.65271
3.47585 2.23909 3.06006 3.92031 3.10536 3.92990 2.83171 1.72969
2.82097 2.97990 3.18715 3.25637 3.92691 261 r - - T
2.68618 4.42225 2.77519 2.73123 3.46354 2.40513 3.72494
3.29354 2.67741 2.69355 4.24690 2.90347 2.73739 3.18146 2.89801
2.37887 2.77519 2.98518 4.58477 3.61503
0.02580 4.06597 4.78831 0.61958 0.77255 0.48576 0.95510

229 2.97083 5.18391 3.28569 2.67614 4.60119 3.62352 3.61616
3.97195 1.78427 3.42096 4.26149 3.12319 3.99636 1.76621 1.79102
2.95380 3.15022 3.63189 5.48756 4.26999 262 q - - T
2.68618 4.42225 2.77519 2.73123 3.46354 2.40513 3.72494
3.29354 2.67741 2.69355 4.24690 2.90347 2.73739 3.18146 2.89801
2.37887 2.77519 2.98518 4.58477 3.61503
0.02580 4.06597 4.78831 0.61958 0.77255 0.48576 0.95510
230 2.59865 4.67342 2.90170 2.62040 4.23066 3.24820 3.86331
3.72791 2.64801 3.33164 4.19161 3.06137 3.84301 2.41767 3.01456
1.36792 2.95111 3.32997 5.51004 4.17997 263 s - - S
2.68618 4.42225 2.77519 2.73123 3.46354 2.40513 3.72494
3.29354 2.67741 2.69355 4.24690 2.90347 2.73739 3.18146 2.89801
2.37887 2.77519 2.98518 4.58477 3.61503
0.02580 4.06597 4.78831 0.61958 0.77255 0.48576 0.95510
231 2.93001 5.19639 3.08859 2.19769 4.60034 3.56218 3.62480
3.98389 1.33990 3.44416 4.27523 3.05473 3.96235 2.75218 2.12149
2.90333 3.12401 3.63051 5.52467 4.27374 264 k - - S
2.68618 4.42225 2.77519 2.73123 3.46354 2.40513 3.72494
3.29354 2.67741 2.69355 4.24690 2.90347 2.73739 3.18146 2.89801
2.37887 2.77519 2.98518 4.58477 3.61503
0.02580 4.06597 4.78831 0.61958 0.77255 0.48576 0.95510
232 2.57663 2.66268 3.12599 2.30004 3.87034 3.14978 3.71178
3.25856 2.52429 2.90928 3.75796 3.06909 3.84481 2.91112 2.49578
2.46707 2.83345 2.96426 5.19483 3.91089 265 e - - S
2.68618 4.42225 2.77519 2.73123 3.46354 2.40513 3.72494
3.29354 2.67741 2.69355 4.24690 2.90347 2.73739 3.18146 2.89801
2.37887 2.77519 2.98518 4.58477 3.61503
0.02580 4.06597 4.78831 0.61958 0.77255 0.48576 0.95510
233 2.92132 4.58794 3.80535 3.25828 3.42719 3.79426 4.02804
2.75672 2.76170 1.23157 3.42005 3.60401 4.19314 3.34781 2.38741
3.16280 3.16472 2.68450 5.04281 3.78736 266 l - - S
2.68618 4.42225 2.77519 2.73123 3.46354 2.40513 3.72494
3.29354 2.67741 2.69355 4.24690 2.90347 2.73739 3.18146 2.89801
2.37887 2.77519 2.98518 4.58477 3.61503
0.02580 4.06597 4.78831 0.61958 0.77255 0.48576 0.95510
234 2.76617 4.90189 2.80534 2.55006 4.38182 2.49633 3.83343
3.88530 2.56131 3.44556 4.31040 3.03458 3.89900 1.36933 2.92087
2.79887 3.07892 3.50128 5.57316 4.25809 267 q - - T
2.68618 4.42225 2.77519 2.73123 3.46354 2.40513 3.72494
3.29354 2.67741 2.69355 4.24690 2.90347 2.73739 3.18146 2.89801
2.37887 2.77519 2.98518 4.58477 3.61503
0.02580 4.06597 4.78831 0.61958 0.77255 0.48576 0.95510
235 2.86549 5.40031 1.44212 1.98726 4.67687 3.29057 3.09460
4.18594 2.62837 3.67923 4.49169 2.74760 3.84734 2.56125 3.17042
2.77671 3.12893 3.76763 5.83269 4.36660 268 d - - T
2.68618 4.42225 2.77519 2.73123 3.46354 2.40513 3.72494
3.29354 2.67741 2.69355 4.24690 2.90347 2.73739 3.18146 2.89801
2.37887 2.77519 2.98518 4.58477 3.61503
0.02580 4.06597 4.78831 0.61958 0.77255 0.48576 0.95510
236 3.09733 5.20517 3.48614 2.83557 4.66713 3.69679 3.65052
4.01821 1.10348 3.45562 4.33091 3.23547 4.07192 2.78650 1.82212
3.09149 3.26510 3.69936 5.48927 4.32502 269 k - - T
2.68618 4.42225 2.77519 2.73123 3.46354 2.40513 3.72494
3.29354 2.67741 2.69355 4.24690 2.90347 2.73739 3.18146 2.89801
2.37887 2.77519 2.98518 4.58477 3.61503
0.02580 4.06597 4.78831 0.61958 0.77255 0.48576 0.95510

```
    237   2.77794  4.56333  3.29554  2.39446  3.60442  3.60646  3.93810
2.83209  2.79957  2.44944  1.67735  3.33019  4.04764  3.21571  3.12118
2.96249  3.03259  2.68069  5.13576  3.88232    270 m - - -
          2.68618  4.42225  2.77519  2.73123  3.46354  2.40513  3.72494
3.29354  2.67741  2.69355  4.24690  2.90347  2.73739  3.18146  2.89801
2.37887  2.77519  2.98518  4.58477  3.61503
          0.02580  4.06597  4.78831  0.61958  0.77255  0.48576  0.95510
    238   2.56583  4.52556  3.21429  2.66446  3.80198  3.42319  3.73537
3.17069  2.55992  2.84120  3.13397  3.11590  3.85657  2.95077  2.53952
2.23477  2.14798  2.88949  5.15055  3.87811    271 t - - -
          2.68618  4.42225  2.77519  2.73123  3.46354  2.40513  3.72494
3.29354  2.67741  2.69355  4.24690  2.90347  2.73739  3.18146  2.89801
2.37887  2.77519  2.98518  4.58477  3.61503
          0.02580  4.06597  4.78831  0.61958  0.77255  0.48576  0.95510
    239   1.15859  4.17899  3.47966  3.20229  4.35099  2.23754  4.27536
3.75608  3.28114  3.45387  4.27513  3.33980  3.71528  3.55185  3.59208
2.09866  2.73028  3.19750  5.69607  4.49132    272 a - - S
          2.68618  4.42225  2.77519  2.73123  3.46354  2.40513  3.72494
3.29354  2.67741  2.69355  4.24690  2.90347  2.73739  3.18146  2.89801
2.37887  2.77519  2.98518  4.58477  3.61503
          0.02580  4.06597  4.78831  0.61958  0.77255  0.48576  0.95510
    240   2.86747  5.07735  3.17482  2.61823  4.49754  2.75619  3.63664
3.88559  1.44071  3.37603  4.20706  3.08102  3.94779  2.76946  1.99495
2.86631  3.07702  3.53508  5.47811  4.23061    273 k - - H
          2.68618  4.42225  2.77519  2.73123  3.46354  2.40513  3.72494
3.29354  2.67741  2.69355  4.24690  2.90347  2.73739  3.18146  2.89801
2.37887  2.77519  2.98518  4.58477  3.61503
          0.02580  4.06597  4.78831  0.61958  0.77255  0.48576  0.95510
    241   2.79111  5.23857  2.30152  1.45791  4.54887  3.33776  3.67596
4.02009  2.47376  3.52871  4.33092  2.81332  3.84039  2.81195  2.45447
2.73436  3.04036  3.62031  5.68186  4.27214    274 e - - H
          2.68618  4.42225  2.77519  2.73123  3.46354  2.40513  3.72494
3.29354  2.67741  2.69355  4.24690  2.90347  2.73739  3.18146  2.89801
2.37887  2.77519  2.98518  4.58477  3.61503
          0.02580  4.06597  4.78831  0.61958  0.77255  0.48576  0.95510
    242   2.35200  4.56403  3.13732  2.60169  3.92844  2.96856  3.72899
3.07351  2.55841  2.96798  3.80583  3.06231  3.81534  2.91936  2.55238
2.04233  2.52688  3.00170  5.24560  3.95429    275 s - - H
          2.68618  4.42225  2.77519  2.73123  3.46354  2.40513  3.72494
3.29354  2.67741  2.69355  4.24690  2.90347  2.73739  3.18146  2.89801
2.37887  2.77519  2.98518  4.58477  3.61503
          0.02580  4.06597  4.78831  0.61958  0.77255  0.48576  0.95510
    243   2.15749  4.80502  2.17048  2.40989  4.21828  2.71916  3.70323
3.64496  2.54077  2.96257  4.04976  2.92232  3.79662  2.85686  3.01609
2.35437  2.86953  3.27171  5.46489  4.11288    276 a - - H
          2.68618  4.42225  2.77519  2.73123  3.46354  2.40513  3.72494
3.29354  2.67741  2.69355  4.24690  2.90347  2.73739  3.18146  2.89801
2.37887  2.77519  2.98518  4.58477  3.61503
          0.02580  4.06597  4.78831  0.61958  0.77255  0.48576  0.95510
    244   2.64671  4.17849  3.91019  3.33614  3.18771  3.71210  3.99202
2.14580  3.21276  2.05587  3.21597  3.61652  4.08246  3.48455  3.45176
2.99607  2.05750  2.31805  3.31057  3.51005    277 l - - H
          2.68618  4.42225  2.77519  2.73123  3.46354  2.40513  3.72494
3.29354  2.67741  2.69355  4.24690  2.90347  2.73739  3.18146  2.89801
2.37887  2.77519  2.98518  4.58477  3.61503
          0.02580  4.06597  4.78831  0.61958  0.77255  0.48576  0.95510
```

```
    245   2.63578  3.47694  2.74011  2.94412  3.29826  3.53364  3.82218
2.92479  2.89739  2.62342  3.54754  3.32848  3.96702  3.21489  3.23962
2.83701  2.89579  2.69483  2.01246  3.41671    278 w - - H
          2.68618  4.42225  2.77519  2.73123  3.46354  2.40513  3.72494
3.29354  2.67741  2.69355  4.24690  2.90347  2.73739  3.18146  2.89801
2.37887  2.77519  2.98518  4.58477  3.61503
          0.02580  4.06597  4.78831  0.61958  0.77255  0.48576  0.95510
    246   2.15594  4.31126  3.41116  2.92192  3.67149  3.35133  3.90818
2.99027  2.87570  1.89979  3.62263  3.27920  3.23964  3.20396  3.22410
2.21025  2.80475  2.72034  5.10056  3.85988    279 l - - H
          2.68618  4.42225  2.77519  2.73123  3.46354  2.40513  3.72494
3.29354  2.67741  2.69355  4.24690  2.90347  2.73739  3.18146  2.89801
2.37887  2.77519  2.98518  4.58477  3.61503
          0.02580  4.06597  4.78831  0.61958  0.77255  0.48576  0.95510
    247   1.98601  4.74951  2.99579  2.49131  4.11522  2.88368  3.67420
3.52888  2.27925  3.12732  3.94466  2.98484  3.02105  2.83050  2.56261
2.63422  2.84972  3.18336  5.35705  4.03926    280 a - - H
          2.68618  4.42225  2.77519  2.73123  3.46354  2.40513  3.72494
3.29354  2.67741  2.69355  4.24690  2.90347  2.73739  3.18146  2.89801
2.37887  2.77519  2.98518  4.58477  3.61503
          0.02580  4.06597  4.78831  0.61958  0.77255  0.48576  0.95510
    248   2.38414  4.43075  2.73339  2.76374  3.68051  3.47589  3.82744
2.67901  2.76614  2.70104  3.60423  3.20366  3.91108  3.08490  3.16346
2.53895  2.84246  1.89285  5.10369  3.84014    281 v - - H
          2.68618  4.42225  2.77519  2.73123  3.46354  2.40513  3.72494
3.29354  2.67741  2.69355  4.24690  2.90347  2.73739  3.18146  2.89801
2.37887  2.77519  2.98518  4.58477  3.61503
          0.02580  4.06597  4.78831  0.61958  0.77255  0.48576  0.95510
    249   2.81960  4.30204  4.17568  3.62190  3.29235  3.93063  4.29795
1.95973  3.49225  1.37926  3.20783  3.89024  4.29395  3.75928  3.71757
2.84670  3.06892  1.99822  4.99911  3.80364    282 l - - H
          2.68618  4.42225  2.77519  2.73123  3.46354  2.40513  3.72494
3.29354  2.67741  2.69355  4.24690  2.90347  2.73739  3.18146  2.89801
2.37887  2.77519  2.98518  4.58477  3.61503
          0.02580  4.06597  4.78831  0.61958  0.77255  0.48576  0.95510
    250   2.62137  4.89843  2.91627  2.39366  4.17506  2.87968  3.60778
3.60641  2.26477  3.17430  3.97411  2.33511  3.35027  2.74312  2.48437
2.63057  2.85328  3.25711  5.37890  3.45080    283 k - - T
          2.68618  4.42225  2.77519  2.73123  3.46354  2.40513  3.72494
3.29354  2.67741  2.69355  4.24690  2.90347  2.73739  3.18146  2.89801
2.37887  2.77519  2.98518  4.58477  3.61503
          0.02580  4.06597  4.78831  0.61958  0.77255  0.48576  0.95510
    251   2.98775  5.19860  3.32605  2.69488  4.62831  3.63874  3.61294
3.99221  1.72471  3.43232  4.27340  3.13610  4.00541  1.88441  1.69565
2.96912  3.16234  3.65205  5.49017  4.28135    284 r - - T
          2.68618  4.42225  2.77519  2.73123  3.46354  2.40513  3.72494
3.29354  2.67741  2.69355  4.24690  2.90347  2.73739  3.18146  2.89801
2.37887  2.77519  2.98518  4.58477  3.61503
          0.02580  4.06597  4.78831  0.61958  0.77255  0.48576  0.95510
    252   2.89631  5.18445  2.63701  1.21970  4.51420  3.40033  3.74622
3.96774  2.44338  3.49992  4.35991  2.93282  3.91822  2.90401  2.48319
2.86269  3.15005  3.61038  5.63634  4.29627    285 e - - T
          2.68618  4.42225  2.77519  2.73123  3.46354  2.40513  3.72494
3.29354  2.67741  2.69355  4.24690  2.90347  2.73739  3.18146  2.89801
2.37887  2.77519  2.98518  4.58477  3.61503
          0.12272  4.06597  2.31928  0.61958  0.77255  0.48576  0.95510
```

```
    253   2.73698  5.16219  2.41772  1.63015  4.47530  3.33154  3.61925
3.93313  2.17720  3.44074  4.23819  2.80618  3.80738  2.75031  2.55015
2.68941  2.97699  3.54062  5.59359  4.20410    286 e - - T
          2.68618  4.42225  2.77519  2.73123  3.46354  2.40513  3.72494
3.29354  2.67741  2.69355  4.24690  2.90347  2.73739  3.18146  2.89801
2.37887  2.77519  2.98518  4.58477  3.61503
          0.01920  3.96245        *  0.61958  0.77255  0.00000        *
//
HMMER3/f [3.3 | Nov 2019]
NAME  FHA
ACC   PF00498.27
DESC  FHA domain
LENG  69
ALPH  amino
RF    no
MM    no
CONS  yes
CS    yes
MAP   yes
DATE  Thu Apr 23 03:29:28 2020
NSEQ  195
EFFN  10.048141
CKSUM 80165375
GA    20.70 20.70
TC    20.70 20.70
NC    20.60 20.60
STATS LOCAL MSV       -8.8106  0.71875
STATS LOCAL VITERBI   -9.3775  0.71875
STATS LOCAL FORWARD   -3.8446  0.71875
HMM          A        C        D        E        F        G        H
I        K        L        M        N        P        Q        R        S
T        V        W        Y
            m->m     m->i     m->d     i->m     i->i     d->m     d->d
  COMPO   2.91704  4.27949  2.79759  2.85392  3.39845  2.59567  3.36071
2.65400  2.76742  2.47296  4.07852  2.92986  3.45177  3.12304  2.71942
2.52339  2.90300  2.62541  4.81946  3.66000
          2.68618  4.42225  2.77519  2.73123  3.46354  2.40513  3.72494
3.29354  2.67741  2.69355  4.24690  2.90347  2.73739  3.18146  2.89801
2.37887  2.77519  2.98518  4.58477  3.61503
          0.04427  5.96995  3.20038  0.61958  0.77255  0.00000        *
      1   3.39702  2.95020  5.42191  4.54462  3.16247  3.63453  3.73745
2.19945  3.73781  2.31282  3.80720  3.85365  5.00581  4.73037  4.04320
3.95254  2.46400  1.76857  2.89802  1.79284      1 v - - E
          2.68618  4.42225  2.77519  2.73123  3.46354  2.40513  3.72494
3.29354  2.67741  2.69355  4.24690  2.90347  2.73739  3.18146  2.89801
2.37887  2.77519  2.98518  4.58477  3.61503
          0.00396  5.92965  6.65199  0.61958  0.77255  0.35295  1.21272
      2   3.17876  4.80636  2.97268  3.13900  3.96738  4.08916  4.18753
2.33740  2.17319  2.60290  3.98382  3.65980  4.70181  3.67970  2.64829
2.26097  1.81553  2.50525  5.07344  4.13327      2 t - - E
          2.68618  4.42225  2.77519  2.73123  3.46354  2.40513  3.72494
3.29354  2.67741  2.69355  4.24690  2.90347  2.73739  3.18146  2.89801
2.37887  2.77519  2.98518  4.58477  3.61503
          0.00380  5.96995  6.69230  0.61958  0.77255  0.48576  0.95510
      3   3.38812  4.47617  5.59979  4.99039  2.49749  4.56554  5.15253
1.08382  4.77877  1.75199  2.84171  4.66963  5.16231  4.90999  4.78626
4.12240  3.84578  1.69356  5.63484  4.45890      3 i - - E
```

2.68618 4.42225 2.77519 2.73123 3.46354 2.40513 3.72494
3.29354 2.67741 2.69355 4.24690 2.90347 2.73739 3.18146 2.89801
2.37887 2.77519 2.98518 4.58477 3.61503
0.00380 5.96995 6.69230 0.61958 0.77255 0.48576 0.95510
4 6.22964 7.44145 6.96725 7.05361 7.72632 0.01456 7.72065
7.96000 7.30436 7.18124 8.37483 7.18536 6.76853 7.55116 7.13726
6.49445 6.75577 7.33159 8.16554 7.95249 4 G - - E
2.68618 4.42225 2.77519 2.73123 3.46354 2.40513 3.72494
3.29354 2.67741 2.69355 4.24690 2.90347 2.73739 3.18146 2.89801
2.37887 2.77519 2.98518 4.58477 3.61503
0.00380 5.96995 6.69230 0.61958 0.77255 0.48576 0.95510
5 4.21200 5.16630 5.11442 4.27478 6.21079 5.07538 4.93912
5.49301 2.76519 4.85622 5.72522 4.34644 5.42438 3.46954 0.30462
2.88174 4.26819 5.15555 6.83532 5.73382 5 R - - S
2.68618 4.42225 2.77519 2.73123 3.46354 2.40513 3.72494
3.29354 2.67741 2.69355 4.24690 2.90347 2.73739 3.18146 2.89801
2.37887 2.77519 2.98518 4.58477 3.61503
0.00380 5.96995 6.69230 0.61958 0.77255 0.48576 0.95510
6 2.53618 5.90500 2.33366 2.42056 4.37768 2.85998 2.72545
4.15283 2.59510 3.45926 4.54956 2.47416 3.34048 3.24111 3.01781
1.71976 3.44943 3.65477 6.33747 3.99646 6 s - - S
2.68589 4.42272 2.77360 2.73221 3.46345 2.40586 3.72575
3.29445 2.67771 2.69426 4.24837 2.90243 2.73746 3.18191 2.89843
2.37630 2.77592 2.98508 4.58662 3.61624
0.65295 0.98472 2.24482 1.49943 0.25265 0.48576 0.95510
7 2.56440 5.27548 2.87199 2.48453 4.84319 2.67313 3.66615
4.28604 2.21096 3.45584 4.87514 3.02154 2.16017 3.02723 2.71625
1.92687 2.98301 3.70874 5.69462 4.58469 18 s - - T
2.68618 4.42225 2.77519 2.73123 3.46354 2.40513 3.72494
3.29354 2.67741 2.69355 4.24690 2.90347 2.73739 3.18146 2.89801
2.37887 2.77519 2.98518 4.58477 3.61503
0.00425 5.85935 6.58170 0.61958 0.77255 0.47747 0.96850
8 2.82537 5.87845 2.05482 2.26847 3.25270 3.18976 3.90081
3.82033 2.26202 3.91462 4.67095 3.02459 2.99593 2.93559 2.80608
2.10614 2.72472 3.27166 5.09763 4.90017 19 d - - T
2.68620 4.42227 2.77521 2.73125 3.46356 2.40515 3.72496
3.29356 2.67743 2.69357 4.24692 2.90349 2.73742 3.18148 2.89794
2.37884 2.77502 2.98520 4.58479 3.61505
0.02466 3.76929 6.65245 0.30832 1.32681 0.35403 1.21017
9 2.29261 1.73602 3.62928 2.92211 4.61478 4.02273 4.09639
3.63719 3.26717 3.46255 4.07959 2.23460 3.76536 2.97034 3.39632
2.07018 2.67983 2.90641 5.25986 4.15441 21 c - - S
2.68618 4.42225 2.77519 2.73123 3.46354 2.40513 3.72494
3.29354 2.67741 2.69355 4.24690 2.90347 2.73739 3.18146 2.89801
2.37887 2.77519 2.98518 4.58477 3.61503
0.00380 5.96995 6.69230 0.61958 0.77255 0.48576 0.95510
10 2.96436 5.40857 1.08258 2.78635 5.24729 4.04283 3.39526
4.32633 3.05476 3.75488 4.94366 2.21521 4.15553 3.10907 2.98292
2.52836 2.74357 4.09590 6.33590 4.29024 22 d - - S
2.68618 4.42225 2.77519 2.73123 3.46354 2.40513 3.72494
3.29354 2.67741 2.69355 4.24690 2.90347 2.73739 3.18146 2.89801
2.37887 2.77519 2.98518 4.58477 3.61503
0.00380 5.96995 6.69230 0.61958 0.77255 0.48576 0.95510
11 3.24904 4.26677 5.48143 4.86510 2.65493 4.22255 4.28061
1.21606 4.64683 1.79875 3.60506 4.84171 3.80846 4.77542 4.65012
3.46935 3.61171 1.86341 3.42878 3.12299 23 i - - E

2.68618 4.42225 2.77519 2.73123 3.46354 2.40513 3.72494
3.29354 2.67741 2.69355 4.24690 2.90347 2.73739 3.18146 2.89801
2.37887 2.77519 2.98518 4.58477 3.61503
0.00380 5.96995 6.69230 0.61958 0.77255 0.48576 0.95510
12 2.64488 4.12197 3.29128 3.43945 3.60860 4.44110 4.08205
2.40369 3.43811 2.47378 3.93245 3.82851 3.33387 2.18664 2.86858
3.14721 3.14762 1.53185 4.61457 3.77522 24 v - - E
2.68601 4.42243 2.77537 2.73141 3.46372 2.40488 3.72489
3.29372 2.67731 2.69373 4.24708 2.90322 2.73757 3.18094 2.89819
2.37867 2.77537 2.98536 4.58438 3.61521
0.06104 2.84782 6.69230 1.01756 0.44860 0.48576 0.95510
13 3.34098 4.45497 4.49321 4.84867 2.61888 4.67261 4.65144
1.70844 4.63430 1.14988 3.63313 3.70514 4.76915 4.76618 3.79767
3.83834 3.40252 1.79148 5.50079 3.98626 28 l - - T
2.68618 4.42225 2.77519 2.73123 3.46354 2.40513 3.72494
3.29354 2.67741 2.69355 4.24690 2.90347 2.73739 3.18146 2.89801
2.37887 2.77519 2.98518 4.58477 3.61503
0.00380 5.96995 6.69230 0.61958 0.77255 0.48576 0.95510
14 2.99949 5.33387 1.87618 2.67180 4.24300 3.34479 3.68665
3.91220 2.68643 3.19755 4.53213 2.93293 2.32294 2.42196 3.03639
2.15728 2.97877 3.28497 6.33441 4.20363 29 d - - T
2.68680 4.42384 2.77298 2.73114 3.46386 2.40528 3.72537
3.29282 2.67754 2.69424 4.24797 2.90129 2.73749 3.18246 2.89869
2.37910 2.77578 2.98412 4.58665 3.61548
0.37455 1.16744 6.69230 1.69514 0.20282 0.48576 0.95510
15 2.85692 4.53859 1.61228 2.57914 3.34044 2.75125 2.01297
4.68277 3.10260 3.69795 4.55733 2.85158 3.87590 3.15843 3.53341
2.23074 3.44451 4.13909 4.56351 3.99730 44 d - - S
2.68641 4.42248 2.77493 2.73146 3.46377 2.40489 3.72518
3.29360 2.67764 2.69315 4.24676 2.90364 2.73682 3.18157 2.89798
2.37876 2.77543 2.98541 4.58500 3.61526
0.12095 2.20652 5.56373 0.68083 0.70561 0.48576 0.95510
16 2.44948 5.89984 3.11511 2.50458 4.30057 3.64066 4.01608
3.82666 2.02146 3.49284 4.26834 3.20165 1.97598 2.82601 2.71809
2.02214 2.97297 3.88135 5.54666 4.15153 48 p - - T
2.68618 4.42225 2.77519 2.73123 3.46354 2.40513 3.72494
3.29354 2.67741 2.69355 4.24690 2.90347 2.73739 3.18146 2.89801
2.37887 2.77519 2.98518 4.58477 3.61503
0.00381 5.96736 6.68970 0.61958 0.77255 0.47400 0.97421
17 3.07359 5.51556 3.07597 2.66005 3.32129 3.06911 3.81174
3.90739 2.49061 3.17145 4.27740 3.44922 4.57675 2.76880 2.68074
1.61500 2.38139 2.52069 5.44202 3.87160 49 s - - T
2.68628 4.42235 2.77530 2.73133 3.46348 2.40523 3.72446
3.29289 2.67751 2.69360 4.24663 2.90357 2.73750 3.18156 2.89788
2.37893 2.77524 2.98476 4.58487 3.61513
0.09296 2.43573 6.69230 0.18828 1.76248 0.48576 0.95510
18 3.11302 3.59735 5.58393 4.97419 3.75655 4.78945 5.13549
1.14952 4.76213 2.16486 3.37916 4.95651 5.14725 4.89453 4.76995
3.81990 3.83098 1.17097 5.62039 3.93257 52 i - - S
2.68618 4.42225 2.77519 2.73123 3.46354 2.40513 3.72494
3.29354 2.67741 2.69355 4.24690 2.90347 2.73739 3.18146 2.89801
2.37887 2.77519 2.98518 4.58477 3.61503
0.00380 5.96995 6.69230 0.61958 0.77255 0.48576 0.95510
19 3.05690 6.03213 3.42037 3.21903 5.36198 4.12418 4.57465
4.82153 3.12781 3.86755 5.11462 3.70196 4.76124 3.43816 3.42157
0.46306 3.86269 4.42255 6.50010 5.11318 53 S - - T

```
         2.68618  4.42225  2.77519  2.73123  3.46354  2.40513  3.72494
3.29354  2.67741  2.69355  4.24690  2.90347  2.73739  3.18146  2.89801
2.37887  2.77519  2.98518  4.58477  3.61503
         0.00380  5.96995  6.69230  0.61958  0.77255  0.48576  0.95510
     20  3.23837  5.90078  3.44281  2.85540  4.94090  2.95151  4.15141
4.72906  2.17736  3.77604  4.94186  3.28343  3.39130  3.33535  0.96606
2.85997  2.75007  4.29056  5.69147  4.16470     54 r - - S
         2.68618  4.42225  2.77519  2.73123  3.46354  2.40513  3.72494
3.29354  2.67741  2.69355  4.24690  2.90347  2.73739  3.18146  2.89801
2.37887  2.77519  2.98518  4.58477  3.61503
         0.00380  5.96995  6.69230  0.61958  0.77255  0.48576  0.95510
     21  3.30906  4.38450  3.56878  2.74512  3.50288  4.19233  2.84264
3.54560  1.89516  3.45732  3.88432  2.35062  4.58463  2.20948  2.29778
3.19810  3.22630  2.61921  6.25870  2.94204     55 k - - S
         2.68618  4.42225  2.77519  2.73123  3.46354  2.40513  3.72494
3.29354  2.67741  2.69355  4.24690  2.90347  2.73739  3.18146  2.89801
2.37887  2.77519  2.98518  4.58477  3.61503
         0.00380  5.96995  6.69230  0.61958  0.77255  0.48576  0.95510
     22  5.42287  7.21512  4.96863  4.90385  5.57437  5.51754  0.12653
6.59592  4.59310  5.82808  6.93639  5.32905  6.13135  3.08465  4.78589
5.39511  5.73141  6.24493  6.95195  5.44354     56 H - - E
         2.68618  4.42225  2.77519  2.73123  3.46354  2.40513  3.72494
3.29354  2.67741  2.69355  4.24690  2.90347  2.73739  3.18146  2.89801
2.37887  2.77519  2.98518  4.58477  3.61503
         0.00380  5.96995  6.69230  0.61958  0.77255  0.48576  0.95510
     23  0.87051  1.80376  5.45888  4.85585  2.86508  3.52974  5.05230
3.09907  4.65244  2.29606  4.05580  4.84934  5.05812  4.79221  4.67768
3.87565  3.76943  3.04942  5.56383  4.38695     57 a - - E
         2.68618  4.42225  2.77519  2.73123  3.46354  2.40513  3.72494
3.29354  2.67741  2.69355  4.24690  2.90347  2.73739  3.18146  2.89801
2.37887  2.77519  2.98518  4.58477  3.61503
         0.00380  5.96995  6.69230  0.61958  0.77255  0.48576  0.95510
     24  2.80544  3.98473  3.70875  1.98749  4.05095  4.33125  4.55587
2.59996  3.21163  1.97212  4.14181  3.79524  4.71657  2.53846  2.41492
2.91865  2.66120  2.20256  5.93256  4.43438     58 l - - E
         2.68626  4.42140  2.77527  2.73131  3.46275  2.40521  3.72436
3.29362  2.67749  2.69339  4.24698  2.90355  2.73748  3.18154  2.89809
2.37895  2.77527  2.98526  4.58376  3.61511
         0.03126  3.59578  5.70356  0.94942  0.48933  0.48576  0.95510
     25  4.95873  6.11102  7.04771  6.52640  2.60028  6.55770  7.01815
0.82812  6.45249  1.12879  4.31419  6.74307  6.54276  6.33032  6.41967
6.00583  5.17995  2.12965  6.91286  5.94617     62 i - - E
         2.68636  4.42148  2.77534  2.73142  3.46355  2.40534  3.72519
3.29322  2.67742  2.69338  4.24661  2.90364  2.73756  3.18157  2.89703
2.37896  2.77544  2.98481  4.58502  3.61492
         0.08333  2.54199  6.69020  1.03662  0.43796  0.47622  0.97055
     26  3.22505  4.48870  2.70351  2.55787  3.20575  4.08154  3.63717
3.31490  2.27713  2.71812  4.52970  3.13063  3.95028  2.66373  2.63338
2.48490  2.58608  2.40682  4.34111  3.24673     66 k - - E
         2.68618  4.42225  2.77519  2.73123  3.46354  2.40513  3.72494
3.29354  2.67741  2.69355  4.24690  2.90347  2.73739  3.18146  2.89801
2.37887  2.77519  2.98518  4.58477  3.61503
         0.01452  5.96995  4.43415  0.61958  0.77255  0.48576  0.95510
     27  3.08789  3.39512  4.04280  3.79815  2.19811  4.53021  3.92017
2.42312  3.05349  2.51244  3.39445  2.75091  4.12094  3.56943  2.53674
2.96095  3.62681  2.37092  3.27449  2.38801     67 f - - E
```

```
         2.68618  4.42225  2.77519  2.73123  3.46354  2.40513  3.72494
3.29354  2.67741  2.69355  4.24690  2.90347  2.73739  3.18146  2.89801
2.37887  2.77519  2.98518  4.58477  3.61503
         0.01680  5.95927  4.26306  0.61958  0.77255  0.56182  0.84436
     28  3.10159  5.04364  1.90583  2.18950  3.80226  3.44771  3.37210
3.17118  2.41549  3.46682  3.23556  2.49099  3.55363  2.89374  2.48745
2.87065  2.90106  3.86577  6.31947  4.32015     68 d - - E
         2.68618  4.42225  2.77519  2.73123  3.46354  2.40513  3.72494
3.29354  2.67741  2.69355  4.24690  2.90347  2.73739  3.18146  2.89801
2.37887  2.77519  2.98518  4.58477  3.61503
         0.00389  5.94636  6.66871  0.61958  0.77255  0.64568  0.74298
     29  2.97164  5.88878  2.46090  2.25445  3.94588  2.44238  4.33912
4.25277  2.23349  3.27563  3.83622  2.41413  2.66197  3.07118  3.01344
2.26872  2.89466  3.41225  6.32150  4.91057     69 k - - T
         2.68618  4.42225  2.77519  2.73123  3.46354  2.40513  3.72494
3.29354  2.67741  2.69355  4.24690  2.90347  2.73739  3.18146  2.89801
2.37887  2.77519  2.98518  4.58477  3.61503
         0.00643  5.94636  5.57356  0.61958  0.77255  0.64568  0.74298
     30  3.01290  5.09171  2.43489  2.18575  5.23253  2.15420  3.74592
4.55311  2.78018  3.44191  3.47989  2.23961  2.82967  2.44183  3.33916
2.62001  2.79049  3.62542  5.23702  4.58252     70 g - - T
         2.68739  4.42552  2.77408  2.73076  3.46061  2.40594  3.72056
3.29256  2.67660  2.69492  4.24887  2.90495  2.73748  3.18062  2.90018
2.37708  2.77733  2.98488  4.58568  3.61281
         1.40151  1.15633  0.82295  2.37993  0.09712  0.66120  0.72615
     31  2.86810  5.54654  2.37951  2.49016  4.88785  3.14345  3.66533
2.87548  2.49491  3.25277  4.58818  3.10610  3.79615  2.54719  2.33613
2.11029  2.41933  3.36593  5.98147  4.57279     97 s - - C
         2.68618  4.42225  2.77519  2.73123  3.46354  2.40513  3.72494
3.29354  2.67741  2.69355  4.24690  2.90347  2.73739  3.18146  2.89801
2.37887  2.77519  2.98518  4.58477  3.61503
         0.05643  5.36782  2.99165  0.61958  0.77255  1.07570  0.41712
     32  2.97616  4.27288  3.23612  2.72258  3.51694  1.80094  4.04796
3.59831  2.68021  2.71074  4.41610  2.94623  2.96363  3.07238  2.41822
3.01621  3.26565  3.17586  3.18722  3.39600     98 g - - E
         2.68618  4.42225  2.77519  2.73123  3.46354  2.40513  3.72494
3.29354  2.67741  2.69355  4.24690  2.90347  2.73739  3.18146  2.89801
2.37887  2.77519  2.98518  4.58477  3.61503
         0.00596  5.52181  6.24415  0.61958  0.77255  0.16418  1.88776
     33  2.63496  5.25983  3.05293  2.51013  4.87326  2.48236  4.07484
3.95900  2.29608  2.86096  3.90543  2.38060  4.17638  2.77296  2.43223
2.39483  2.79961  2.99597  6.31658  3.59897     99 k - - E
         2.68618  4.42225  2.77519  2.73123  3.46354  2.40513  3.72494
3.29354  2.67741  2.69355  4.24690  2.90347  2.73739  3.18146  2.89801
2.37887  2.77519  2.98518  4.58477  3.61503
         0.00389  5.94636  6.66871  0.61958  0.77255  0.39736  1.11502
     34  3.11649  4.13940  5.48030  4.86405  1.84550  4.67473  4.43787
2.07962  4.64605  2.28997  3.90175  4.84124  3.84044  4.21004  4.64975
3.90807  3.68385  1.70918  2.25620  2.22598    100 v - - E
         2.68618  4.42225  2.77519  2.73123  3.46354  2.40513  3.72494
3.29354  2.67741  2.69355  4.24690  2.90347  2.73739  3.18146  2.89801
2.37887  2.77519  2.98518  4.58477  3.61503
         0.00380  5.96995  6.69230  0.61958  0.77255  0.48576  0.95510
     35  3.20490  3.58092  3.88561  2.44465  2.54627  2.55313  3.64214
3.06113  3.46147  2.57464  4.13439  4.17034  4.81524  3.47629  2.45479
3.20894  2.68232  2.72536  4.05193  1.97037    101 y - - E
```

```
          2.68618  4.42225  2.77519  2.73123  3.46354  2.40513  3.72494
3.29354  2.67741  2.69355  4.24690  2.90347  2.73739  3.18146  2.89801
2.37887  2.77519  2.98518  4.58477  3.61503
          0.00380  5.96995  6.69230  0.61958  0.77255  0.48576  0.95510
     36   3.61032  5.28045  5.96678  5.37773  4.05910  5.22570  5.60426
1.31401  5.18914  1.10610  4.25453  5.39207  5.54274  5.31145  4.06616
4.55814  4.19508  1.40143  6.03384  4.57970    102 l - - E
          2.68618  4.42225  2.77519  2.73123  3.46354  2.40513  3.72494
3.29354  2.67741  2.69355  4.24690  2.90347  2.73739  3.18146  2.89801
2.37887  2.77519  2.98518  4.58477  3.61503
          0.00380  5.96995  6.69230  0.61958  0.77255  0.48576  0.95510
     37   3.14157  5.10016  3.07029  1.97598  4.59516  4.18228  3.24127
2.33324  2.24225  3.84420  3.64730  3.27122  4.57506  2.76983  2.00895
3.20775  2.74277  2.60719  5.10612  3.87255    103 e - - E
          2.68618  4.42225  2.77519  2.73123  3.46354  2.40513  3.72494
3.29354  2.67741  2.69355  4.24690  2.90347  2.73739  3.18146  2.89801
2.37887  2.77519  2.98518  4.58477  3.61503
          0.00380  5.96995  6.69230  0.61958  0.77255  0.48576  0.95510
     38   3.43683  4.75999  0.65443  3.22402  4.80221  4.27462  4.49321
3.13294  3.31481  3.45519  4.44756  2.99449  3.12669  3.65860  3.77129
3.28417  3.67031  3.61743  6.09626  4.77457    104 D - - E
          2.68641  4.42259  2.77443  2.73133  3.46374  2.40433  3.72499
3.29388  2.67762  2.69336  4.24627  2.90345  2.73752  3.18110  2.89834
2.37920  2.77535  2.98552  4.58511  3.61537
          0.24223  2.64445  1.93737  1.40453  0.28168  0.48576  0.95510
     39   3.18965  3.87734  5.18452  3.90016  2.57204  3.94612  4.07655
2.69915  3.57301  0.96343  3.06953  3.54039  4.90603  3.47583  3.68424
3.67572  3.31304  2.38212  4.18858  4.23240    110 l - - E
          2.68618  4.42225  2.77519  2.73123  3.46354  2.40513  3.72494
3.29354  2.67741  2.69355  4.24690  2.90347  2.73739  3.18146  2.89801
2.37887  2.77519  2.98518  4.58477  3.61503
          0.07093  5.81582  2.72579  0.61958  0.77255  0.31313  1.31361
     40   3.24413  4.10865  2.72104  2.92446  5.19089  1.31192  3.58863
4.10821  2.25227  4.15224  4.88898  2.41013  3.97635  3.21619  2.56857
2.89987  2.90261  4.09246  4.83527  4.02611    111 g - - S
          2.68599  4.41986  2.77524  2.73154  3.46252  2.40565  3.72507
3.29363  2.67700  2.69325  4.24742  2.90297  2.73780  3.18165  2.89752
2.37926  2.77556  2.98516  4.58529  3.61476
          0.21852  2.21698  2.43778  1.50815  0.25016  0.27949  1.41129
     41   3.29639  5.20369  3.64539  2.86139  5.09663  3.32270  4.17343
4.55777  2.87738  4.07646  4.58645  3.38249  4.01543  3.45373  3.37741
0.62584  3.10767  4.15660  6.24026  4.85363    118 S - - S
          2.68618  4.42225  2.77519  2.73123  3.46354  2.40513  3.72494
3.29354  2.67741  2.69355  4.24690  2.90347  2.73739  3.18146  2.89801
2.37887  2.77519  2.98518  4.58477  3.61503
          0.00413  5.88730  6.60965  0.61958  0.77255  0.27706  1.41885
     42   2.89319  4.83906  3.21587  2.73358  5.22166  3.11635  4.23889
4.24117  1.84934  2.43430  4.73774  3.40866  4.56491  3.29048  2.32371
1.92286  2.01333  3.62354  6.32285  4.38745    119 k - - S
          2.68618  4.42225  2.77519  2.73123  3.46354  2.40513  3.72494
3.29354  2.67741  2.69355  4.24690  2.90347  2.73739  3.18146  2.89801
2.37887  2.77519  2.98518  4.58477  3.61503
          0.00380  5.96995  6.69230  0.61958  0.77255  0.48576  0.95510
     43   2.87209  5.70540  3.65758  3.04201  3.86882  3.68005  3.04113
3.22289  3.06978  3.72555  4.53560  0.87495  4.60854  3.35186  3.44225
2.89012  3.16055  4.05348  6.18773  3.21060    120 n - - S
```

```
         2.68618  4.42225  2.77519  2.73123  3.46354  2.40513  3.72494
3.29354  2.67741  2.69355  4.24690  2.90347  2.73739  3.18146  2.89801
2.37887  2.77519  2.98518  4.58477  3.61503
         0.00380  5.96995  6.69230  0.61958  0.77255  0.48576  0.95510
     44  3.28695  4.93351  3.66046  2.99403  4.99306  0.62519  4.20369
4.42987  2.96117  3.66935  4.78265  3.61171  4.07150  3.42419  3.41708
3.03021  3.46121  3.77679  6.20077  4.49729    121 G - - S
         2.68618  4.42225  2.77519  2.73123  3.46354  2.40513  3.72494
3.29354  2.67741  2.69355  4.24690  2.90347  2.73739  3.18146  2.89801
2.37887  2.77519  2.98518  4.58477  3.61503
         0.00380  5.96995  6.69230  0.61958  0.77255  0.48576  0.95510
     45  2.76910  3.99301  5.41497  4.80571  3.99717  4.67610  5.01313
2.35174  4.60562  2.53753  3.91482  4.82031  4.80038  4.74973  4.40493
3.15310  0.78268  2.17410  5.52692  4.34928    122 t - - E
         2.68620  4.42227  2.77522  2.73126  3.46341  2.40515  3.72497
3.29356  2.67743  2.69347  4.24692  2.90349  2.73742  3.18149  2.89794
2.37889  2.77522  2.98521  4.58396  3.61505
         0.04759  3.64115  3.89947  0.27954  1.41112  0.48576  0.95510
     46  3.38132  4.13838  5.32321  4.11455  1.35396  4.64177  4.48194
3.11837  2.88277  2.40448  2.88420  4.51389  5.00676  3.94883  4.27349
3.32332  3.27305  2.84778  3.57764  1.64739    124 f - - E
         2.68618  4.42225  2.77519  2.73123  3.46354  2.40513  3.72494
3.29354  2.67741  2.69355  4.24690  2.90347  2.73739  3.18146  2.89801
2.37887  2.77519  2.98518  4.58477  3.61503
         0.00388  5.95076  6.67310  0.61958  0.77255  0.41113  1.08737
     47  3.51479  4.88725  5.48987  4.87391  3.27421  4.37406  5.02020
1.85635  4.65644  1.58679  3.65443  4.85227  5.04873  4.24646  4.15899
3.99816  3.74690  0.95230  5.09388  3.93287    125 v - - E
         2.68637  4.42244  2.77504  2.73123  3.46347  2.40491  3.72513
3.29373  2.67730  2.69365  4.24709  2.90343  2.73746  3.18136  2.89787
2.37896  2.77504  2.98537  4.58496  3.61503
         0.10393  2.98741  3.03037  1.17169  0.37084  0.48576  0.95510
     48  3.43661  6.00884  2.46157  2.91711  5.34813  3.35446  4.29410
4.83771  3.09797  4.31134  4.65544  0.65758  4.29789  3.22399  3.31540
3.18242  3.61780  4.39714  6.44075  5.02041    131 N - - T
         2.68618  4.42225  2.77519  2.73123  3.46354  2.40513  3.72494
3.29354  2.67741  2.69355  4.24690  2.90347  2.73739  3.18146  2.89801
2.37887  2.77519  2.98518  4.58477  3.61503
         0.00399  5.92175  6.64410  0.61958  0.77255  0.78588  0.60829
     49  2.97678  5.87369  2.81969  2.64082  3.25451  1.34562  3.22164
4.03765  2.37762  3.79105  4.91428  2.54983  4.24389  3.07456  2.55521
2.76173  3.40427  4.26448  6.30622  4.89510    132 g - - T
         2.68642  4.42158  2.77535  2.73137  3.46371  2.40459  3.72528
3.29371  2.67744  2.69388  4.24723  2.90364  2.73761  3.18042  2.89820
2.37902  2.77507  2.98462  4.58427  3.61536
         0.10085  2.35778  6.64410  1.18494  0.36494  0.78588  0.60829
     50  3.03009  5.17149  3.56934  2.24792  4.00144  3.82060  3.33961
4.35203  2.15103  3.38931  4.77487  2.95662  4.53245  1.87290  2.36925
2.55643  2.66077  2.39922  6.29612  4.19532    139 q - - E
         2.68618  4.42225  2.77519  2.73123  3.46354  2.40513  3.72494
3.29354  2.67741  2.69355  4.24690  2.90347  2.73739  3.18146  2.89801
2.37887  2.77519  2.98518  4.58477  3.61503
         0.00399  5.92175  6.64410  0.61958  0.77255  0.33549  1.25522
     51  3.12412  5.85080  3.43435  2.80463  3.77350  4.17963  3.97533
3.94494  1.78076  2.68184  4.28271  3.65653  2.83043  2.83813  1.36200
2.83448  3.47852  3.42207  6.29755  4.64217    140 r - - E
```

```
          2.68618  4.42225  2.77519  2.73123  3.46354  2.40513  3.72494
3.29354  2.67741  2.69355  4.24690  2.90347  2.73739  3.18146  2.89801
2.37887  2.77519  2.98518  4.58477  3.61503
          0.00380  5.96995  6.69230  0.61958  0.77255  0.48576  0.95510
     52   3.26486  4.17523  4.41222  4.83208  3.33153  4.41627  4.16930
1.39082  4.62159  1.45560  3.24018  4.55490  3.91486  3.93314  4.07143
3.86641  3.19263  1.68449  5.50274  4.32630    141 i - - E
          2.68618  4.42225  2.77519  2.73123  3.46354  2.40513  3.72494
3.29354  2.67741  2.69355  4.24690  2.90347  2.73739  3.18146  2.89801
2.37887  2.77519  2.98518  4.58477  3.61503
          0.01540  5.96995  4.36413  0.61958  0.77255  0.48576  0.95510
     53   3.18192  5.21513  3.07844  2.44113  4.84075  2.15583  3.01086
3.88945  2.38456  3.04064  4.56803  3.16953  3.01440  2.39473  2.24880
2.42377  2.89714  3.51808  6.32952  4.32654    142 g - - S
          2.68618  4.42225  2.77519  2.73123  3.46354  2.40513  3.72494
3.29354  2.67741  2.69355  4.24690  2.90347  2.73739  3.18146  2.89801
2.37887  2.77519  2.98518  4.58477  3.61503
          0.04845  5.95840  3.10755  0.61958  0.77255  0.51678  0.90742
     54   3.03962  5.29724  3.02105  2.71853  5.21925  3.24327  3.27875
4.08718  1.83668  3.56948  4.58595  2.60033  2.24313  2.32178  2.60675
2.40776  2.93588  3.66208  6.30530  4.34702    143 k - - T
          2.68566  4.42337  2.77570  2.73018  3.46438  2.40474  3.72397
3.29351  2.67755  2.69453  4.24801  2.90306  2.73660  3.18156  2.89786
2.37935  2.77477  2.98585  4.58420  3.61615
          0.71722  1.76603  1.07622  1.79985  0.18071  0.80260  0.59450
     55   2.88153  5.61301  2.40980  2.70371  3.11347  1.84753  3.70139
3.78437  2.38104  3.31426  4.65518  2.40597  4.28529  2.73095  3.06288
2.27097  3.14819  3.83058  6.04915  4.64168    158 g - - T
          2.68618  4.42225  2.77519  2.73123  3.46354  2.40513  3.72494
3.29354  2.67741  2.69355  4.24690  2.90347  2.73739  3.18146  2.89801
2.37887  2.77519  2.98518  4.58477  3.61503
          0.03341  5.50366  3.54797  0.61958  0.77255  0.90318  0.51966
     56   2.95381  5.71514  2.80707  1.85249  3.97579  3.71525  3.93660
4.13925  1.99445  3.78745  4.75560  2.14478  2.50544  2.69820  2.84743
2.93307  2.85397  3.33509  6.14737  4.73595    159 e - - S
          2.68618  4.42225  2.77519  2.73123  3.46354  2.40513  3.72494
3.29354  2.67741  2.69355  4.24690  2.90347  2.73739  3.18146  2.89801
2.37887  2.77519  2.98518  4.58477  3.61503
          0.00519  5.65978  6.38212  0.61958  0.77255  0.59118  0.80670
     57   2.93672  4.85568  2.46881  2.26528  4.70193  3.47846  4.27183
4.21218  2.28751  3.66778  4.22450  3.42892  1.89884  3.05684  2.75872
2.47900  2.83951  2.72551  6.23918  3.27059    160 p - - E
          2.68618  4.42225  2.77519  2.73123  3.46354  2.40513  3.72494
3.29354  2.67741  2.69355  4.24690  2.90347  2.73739  3.18146  2.89801
2.37887  2.77519  2.98518  4.58477  3.61503
          0.00436  5.83379  6.55613  0.61958  0.77255  0.22019  1.62133
     58   2.41980  4.38913  3.96350  3.23016  3.72411  3.97121  2.57507
2.54533  3.50222  2.80715  3.98321  3.90353  4.82201  2.74132  2.97559
3.11573  2.50361  1.88468  4.92155  2.25988    161 v - - E
          2.68618  4.42225  2.77519  2.73123  3.46354  2.40513  3.72494
3.29354  2.67741  2.69355  4.24690  2.90347  2.73739  3.18146  2.89801
2.37887  2.77519  2.98518  4.58477  3.61503
          0.00380  5.96995  6.69230  0.61958  0.77255  0.48576  0.95510
     59   2.82036  5.86099  2.54414  1.79544  4.92178  4.17719  4.36629
2.85110  2.65436  2.82106  3.62467  3.38508  2.93234  2.89796  1.98559
3.05820  3.12720  2.86160  5.28043  4.54142    162 e - - E
```

```
          2.68618  4.42225  2.77519  2.73123  3.46354  2.40513  3.72494
3.29354  2.67741  2.69355  4.24690  2.90347  2.73739  3.18146  2.89801
2.37887  2.77519  2.98518  4.58477  3.61503
          0.00380  5.96995  6.69230  0.61958  0.77255  0.48576  0.95510
     60   3.82930  5.27487  5.91373  5.30395  3.64915  5.14426  5.47652
2.28568  5.09992  0.48397  3.78514  5.31192  5.45269  5.18078  4.43798
4.46748  3.93312  2.22329  5.87745  4.28010    163 L - - E
          2.68618  4.42225  2.77519  2.73123  3.46354  2.40513  3.72494
3.29354  2.67741  2.69355  4.24690  2.90347  2.73739  3.18146  2.89801
2.37887  2.77519  2.98518  4.58477  3.61503
          0.00380  5.96995  6.69230  0.61958  0.77255  0.48576  0.95510
     61   2.32248  5.90419  3.51341  2.73465  3.89600  3.42942  3.02257
4.37394  1.92356  3.86220  4.42555  2.99800  3.23167  2.11229  2.41555
2.07878  3.04007  3.88902  5.41811  4.27027    164 k - - S
          2.68618  4.42225  2.77519  2.73123  3.46354  2.40513  3.72494
3.29354  2.67741  2.69355  4.24690  2.90347  2.73739  3.18146  2.89801
2.37887  2.77519  2.98518  4.58477  3.61503
          0.00380  5.96995  6.69230  0.61958  0.77255  0.48576  0.95510
     62   2.99562  5.90042  2.01437  2.46411  5.24378  4.16820  2.56336
3.10756  2.90584  3.96837  4.94152  2.23900  3.51170  2.94352  3.58331
1.83254  2.72803  3.00705  6.33412  4.41788    165 s - - T
          2.68618  4.42225  2.77519  2.73123  3.46354  2.40513  3.72494
3.29354  2.67741  2.69355  4.24690  2.90347  2.73739  3.18146  2.89801
2.37887  2.77519  2.98518  4.58477  3.61503
          0.00380  5.96995  6.69230  0.61958  0.77255  0.48576  0.95510
     63   3.31911  4.40289  2.09393  2.95458  4.87157  0.94467  4.36087
4.47635  2.51873  4.20334  4.94181  2.74202  4.56640  3.21918  3.36438
3.05319  3.39200  4.28828  6.33496  4.92627    166 g - - T
          2.68618  4.42225  2.77519  2.73123  3.46354  2.40513  3.72494
3.29354  2.67741  2.69355  4.24690  2.90347  2.73739  3.18146  2.89801
2.37887  2.77519  2.98518  4.58477  3.61503
          0.00380  5.96995  6.69230  0.61958  0.77255  0.48576  0.95510
     64   2.96232  3.45875  0.88849  2.60652  4.02063  3.96236  4.16458
4.37421  3.15364  3.75504  4.10319  3.32422  4.59138  3.41994  3.63530
2.46340  2.77485  3.83328  6.23837  4.86273    167 d - - E
          2.68618  4.42225  2.77519  2.73123  3.46354  2.40513  3.72494
3.29354  2.67741  2.69355  4.24690  2.90347  2.73739  3.18146  2.89801
2.37887  2.77519  2.98518  4.58477  3.61503
          0.00380  5.96995  6.69230  0.61958  0.77255  0.48576  0.95510
     65   3.22630  5.43089  3.81270  1.97372  4.48508  4.10474  4.18674
2.05529  3.22978  2.54894  3.73872  3.68382  4.17987  3.09226  2.32247
3.30428  2.43323  1.87197  5.97035  4.03745    168 v - - E
          2.68608  4.42227  2.77521  2.73125  3.46356  2.40515  3.72496
3.29356  2.67732  2.69357  4.24692  2.90349  2.73741  3.18135  2.89803
2.37889  2.77521  2.98520  4.58479  3.61505
          0.02294  3.84277  6.69230  0.31418  1.31078  0.48576  0.95510
     66   4.53510  5.76788  6.59188  6.04435  2.91218  5.96527  6.38569
0.75304  5.91107  1.57643  4.36014  6.13596  6.14979  5.95025  5.92084
5.34535  4.76760  1.63310  6.59686  4.49911    170 i - - E
          2.68618  4.42225  2.77519  2.73123  3.46354  2.40513  3.72494
3.29354  2.67741  2.69355  4.24690  2.90347  2.73739  3.18146  2.89801
2.37887  2.77519  2.98518  4.58477  3.61503
          0.00380  5.96995  6.69230  0.61958  0.77255  0.48576  0.95510
     67   2.81433  5.30547  2.50637  2.22256  5.24731  3.59215  2.99659
3.80344  2.32098  2.63307  4.40106  2.93898  4.56110  2.44801  2.43261
2.69330  2.37818  3.10291  5.85687  4.41898    171 e - - E
```

```
          2.68618  4.42225  2.77519  2.73123  3.46354  2.40513  3.72494
3.29354  2.67741  2.69355  4.24690  2.90347  2.73739  3.18146  2.89801
2.37887  2.77519  2.98518  4.58477  3.61503
          0.00380  5.96995  6.69230  0.61958  0.77255  0.48576  0.95510
     68   4.01586  5.62078  6.30191  5.69297  1.35985  5.56247  5.87368
1.60971  5.50075  1.04002  3.38139  5.73604  5.79216  5.50066  5.46339
4.89975  4.53506  2.64389  4.80144  5.07620    172 l - - E
          2.68618  4.42225  2.77519  2.73123  3.46354  2.40513  3.72494
3.29354  2.67741  2.69355  4.24690  2.90347  2.73739  3.18146  2.89801
2.37887  2.77519  2.98518  4.58477  3.61503
          0.11655  5.96995  2.23063  0.61958  0.77255  0.48576  0.95510
     69   2.77635  4.82162  4.13349  3.74453  4.72855  0.54807  4.47163
3.41966  3.71533  3.79249  4.65159  4.14563  3.99079  4.03762  3.61237
2.74994  3.74204  3.65311  6.11052  4.85285    173 G - - T
          2.68618  4.42225  2.77519  2.73123  3.46354  2.40513  3.72494
3.29354  2.67741  2.69355  4.24690  2.90347  2.73739  3.18146  2.89801
2.37887  2.77519  2.98518  4.58477  3.61503
          0.00287  5.85627        *  0.61958  0.77255  0.00000        *
//
HMMER3/f [3.3 | Nov 2019]
NAME  FYRC
ACC   PF05965.15
DESC  F/Y rich C-terminus
LENG  84
ALPH  amino
RF    no
MM    no
CONS  yes
CS    no
MAP   yes
DATE  Fri Apr 17 12:05:44 2020
NSEQ  110
EFFN  5.481873
CKSUM 2479452943
GA    20.80 20.80
TC    20.80 20.80
NC    20.70 20.70
STATS LOCAL MSV       -8.8673  0.71849
STATS LOCAL VITERBI   -9.7388  0.71849
STATS LOCAL FORWARD   -4.0031  0.71849
HMM          A        C        D        E        F        G        H
I        K        L        M        N        P        Q        R        S
T        V        W        Y
            m->m     m->i     m->d     i->m     i->i     d->m     d->d
  COMPO   2.65764  4.08547  3.05203  2.55004  3.13732  2.91608  3.68044
2.94089  2.63554  2.48304  3.70307  3.07607  3.17590  2.97593  2.92059
2.61822  2.88830  2.68893  4.36808  3.49634
          2.68618  4.42225  2.77519  2.73123  3.46354  2.40513  3.72494
3.29354  2.67741  2.69355  4.24690  2.90347  2.73739  3.18146  2.89801
2.37887  2.77519  2.98518  4.58477  3.61503
          0.00656  5.42598  6.14832  0.61958  0.77255  0.00000        *
      1   2.78438  5.56200  3.33385  2.44429  4.62749  1.87414  4.04103
4.12723  2.00673  3.46142  3.99031  3.13946  4.24625  2.32448  2.11382
2.83259  3.06020  3.32076  6.00064  4.59592      1 g - - -
          2.68618  4.42225  2.77519  2.73123  3.46354  2.40513  3.72494
3.29354  2.67741  2.69355  4.24690  2.90347  2.73739  3.18146  2.89801
2.37887  2.77519  2.98518  4.58477  3.61503
          0.00656  5.42598  6.14832  0.61958  0.77255  0.48576  0.95510
```

```
      2   2.93566  5.17743  4.47910  4.09814  4.74771  4.15177  5.00079
3.97380  3.43724  2.74980  4.69341  4.32066  0.47397  4.31496  4.09033
3.57907  3.75950  3.43810  6.19176  4.98519      2 P - - -
          2.68618  4.42225  2.77519  2.73123  3.46354  2.40513  3.72494
3.29354  2.67741  2.69355  4.24690  2.90347  2.73739  3.18146  2.89801
2.37887  2.77519  2.98518  4.58477  3.61503
          0.00656  5.42598  6.14832  0.61958  0.77255  0.48576  0.95510
      3   3.09408  5.13358  3.60876  2.51455  3.91175  3.54978  3.04382
2.83291  2.34578  1.72903  4.21682  3.13604  4.35259  2.50453  3.33777
2.94890  2.88030  3.03733  3.45321  3.32651      3 l - - -
          2.68618  4.42225  2.77519  2.73123  3.46354  2.40513  3.72494
3.29354  2.67741  2.69355  4.24690  2.90347  2.73739  3.18146  2.89801
2.37887  2.77519  2.98518  4.58477  3.61503
          0.00656  5.42598  6.14832  0.61958  0.77255  0.48576  0.95510
      4   4.32545  5.56679  6.24864  5.67064  0.58000  5.61525  5.73312
2.60655  5.50469  1.59616  3.37956  5.74788  5.72387  5.38773  5.43315
4.98047  4.53034  3.11585  5.82531  4.14386      4 F - - -
          2.68618  4.42225  2.77519  2.73123  3.46354  2.40513  3.72494
3.29354  2.67741  2.69355  4.24690  2.90347  2.73739  3.18146  2.89801
2.37887  2.77519  2.98518  4.58477  3.61503
          0.00656  5.42598  6.14832  0.61958  0.77255  0.48576  0.95510
      5   2.85920  4.79681  3.45503  2.24596  4.58882  3.90491  4.10537
3.11456  1.96605  3.10232  2.96554  3.23951  4.29538  3.01876  1.88098
2.88468  2.81815  2.46072  5.82832  4.48127      5 r - - -
          2.68618  4.42225  2.77519  2.73123  3.46354  2.40513  3.72494
3.29354  2.67741  2.69355  4.24690  2.90347  2.73739  3.18146  2.89801
2.37887  2.77519  2.98518  4.58477  3.61503
          0.00656  5.42598  6.14832  0.61958  0.77255  0.48576  0.95510
      6   3.32398  4.67552  5.30006  4.69881  2.31166  4.53485  4.88270
1.80736  4.49688  2.51411  3.77358  4.69498  4.88381  4.63187  4.51384
3.85463  2.85553  0.99821  5.35707  2.90638      6 v - - -
          2.68618  4.42225  2.77519  2.73123  3.46354  2.40513  3.72494
3.29354  2.67741  2.69355  4.24690  2.90347  2.73739  3.18146  2.89801
2.37887  2.77519  2.98518  4.58477  3.61503
          0.00656  5.42598  6.14832  0.61958  0.77255  0.48576  0.95510
      7   3.02500  5.49468  3.22004  1.98048  4.80255  3.86670  4.05807
3.67238  2.58536  3.49581  4.54531  3.21159  4.25921  2.80244  2.55168
2.08351  1.85944  2.52925  4.56041  4.03047      7 t - - -
          2.68618  4.42225  2.77519  2.73123  3.46354  2.40513  3.72494
3.29354  2.67741  2.69355  4.24690  2.90347  2.73739  3.18146  2.89801
2.37887  2.77519  2.98518  4.58477  3.61503
          0.00656  5.42598  6.14832  0.61958  0.77255  0.48576  0.95510
      8   2.46836  3.80822  4.28362  4.36929  3.62950  4.29492  4.62161
1.90109  4.18162  2.12891  2.61591  3.99953  3.01166  3.90839  4.23483
2.80325  3.20644  1.58352  5.15507  3.08652      8 v - - -
          2.68618  4.42225  2.77519  2.73123  3.46354  2.40513  3.72494
3.29354  2.67741  2.69355  4.24690  2.90347  2.73739  3.18146  2.89801
2.37887  2.77519  2.98518  4.58477  3.61503
          0.00656  5.42598  6.14832  0.61958  0.77255  0.48576  0.95510
      9   2.71653  4.85361  2.55851  1.58189  4.54931  3.85187  3.37376
3.46057  2.45316  3.11906  4.38701  3.23330  3.87971  2.66575  2.99889
2.43442  2.57114  3.06126  6.00358  4.59765      9 e - - -
          2.68618  4.42225  2.77519  2.73123  3.46354  2.40513  3.72494
3.29354  2.67741  2.69355  4.24690  2.90347  2.73739  3.18146  2.89801
2.37887  2.77519  2.98518  4.58477  3.61503
          0.02484  5.42598  3.90558  0.61958  0.77255  0.48576  0.95510
```

```
     10   3.09217  5.60832  2.08277  1.61674  4.95283  3.38007  3.50783
4.44061  2.70276  3.91336  4.65009  2.63180  4.25149  2.98334  3.13233
1.77138  2.69791  3.99884  6.04027  4.62602     10 e - - -
          2.68618  4.42225  2.77519  2.73123  3.46354  2.40513  3.72494
3.29354  2.67741  2.69355  4.24690  2.90347  2.73739  3.18146  2.89801
2.37887  2.77519  2.98518  4.58477  3.61503
          0.00668  5.40782  6.13017  0.61958  0.77255  0.56057  0.84602
     11   2.71402  3.29249  1.90605  2.10274  4.91652  3.04482  3.35185
4.40161  2.53458  3.49145  3.52669  2.75517  3.35856  2.59436  2.93936
2.66894  3.14819  3.56999  6.00615  4.16245     11 d - - -
          2.68627  4.42261  2.77517  2.73106  3.46377  2.40464  3.72429
3.29353  2.67747  2.69358  4.24725  2.90293  2.73763  3.18175  2.89822
2.37883  2.77555  2.98554  4.58512  3.61478
          0.31240  1.49565  3.11886  0.93239  0.50023  0.44167  1.02991
     12   2.96088  5.57257  1.86118  2.02999  4.91153  3.83734  3.85671
4.39448  2.58978  3.54562  4.29334  3.23147  1.51804  2.95225  3.26661
2.86558  3.30430  3.79594  6.00814  4.59872     17 p - - -
          2.68618  4.42225  2.77519  2.73123  3.46354  2.40513  3.72494
3.29354  2.67741  2.69355  4.24690  2.90347  2.73739  3.18146  2.89801
2.37887  2.77519  2.98518  4.58477  3.61503
          0.02990  5.38382  3.69440  0.61958  0.77255  0.48553  0.95547
     13   3.05738  4.80989  1.75997  2.20616  4.91458  2.01794  3.37942
4.40094  2.58113  3.87465  4.60996  2.95527  3.87537  2.73260  3.14491
2.37106  2.75414  3.96005  6.00169  4.59024     18 d - - -
          2.68618  4.42225  2.77519  2.73123  3.46354  2.40513  3.72494
3.29354  2.67741  2.69355  4.24690  2.90347  2.73739  3.18146  2.89801
2.37887  2.77519  2.98518  4.58477  3.61503
          0.01773  5.38771  4.34234  0.61958  0.77255  0.63598  0.75379
     14   3.02315  3.72850  4.48297  2.19583  2.23316  4.19034  4.48706
2.44128  3.27183  2.03475  3.57214  2.94722  4.56104  4.02491  3.50764
3.21464  2.69570  2.01866  5.21076  3.48507     19 v - - -
          2.68618  4.42225  2.77519  2.73123  3.46354  2.40513  3.72494
3.29354  2.67741  2.69355  4.24690  2.90347  2.73739  3.18146  2.89801
2.37887  2.77519  2.98518  4.58477  3.61503
          0.00689  5.37687  6.09921  0.61958  0.77255  0.50490  0.92525
     15   2.66511  3.68403  3.49054  2.54323  3.00366  3.91231  3.31238
2.98131  2.28135  2.95971  4.32620  3.05449  3.05315  2.97796  2.89678
2.24167  2.58499  2.65421  5.76084  4.09811     20 s - - -
          2.68618  4.42225  2.77519  2.73123  3.46354  2.40513  3.72494
3.29354  2.67741  2.69355  4.24690  2.90347  2.73739  3.18146  2.89801
2.37887  2.77519  2.98518  4.58477  3.61503
          0.00671  5.40404  6.12638  0.61958  0.77255  0.43358  1.04465
     16   2.83063  3.79089  5.09577  3.77984  1.68535  4.31009  3.64204
1.70986  3.90122  2.02968  3.63705  4.46932  4.67388  4.40114  4.27969
3.62095  3.13844  2.30710  3.60221  2.78301     21 f - - -
          2.68620  4.42227  2.77522  2.73125  3.46356  2.40501  3.72497
3.29356  2.67743  2.69357  4.24692  2.90349  2.73742  3.18148  2.89803
2.37889  2.77522  2.98520  4.58479  3.61468
          0.04235  3.23589  6.14832  0.30325  1.34099  0.48576  0.95510
     17   3.01657  4.92653  3.01404  2.06479  4.40491  3.86745  4.05902
2.98038  2.26688  3.27814  4.34201  3.35165  4.25992  2.63550  2.52297
2.35723  2.14589  2.44685  5.94752  4.56113     23 e - - -
          2.68618  4.42225  2.77519  2.73123  3.46354  2.40513  3.72494
3.29354  2.67741  2.69355  4.24690  2.90347  2.73739  3.18146  2.89801
2.37887  2.77519  2.98518  4.58477  3.61503
          0.00656  5.42598  6.14832  0.61958  0.77255  0.48576  0.95510
```

```
     18   1.86275  4.33518  2.30157  2.75141  4.85876  1.99929  3.22505
4.32995  2.60556  3.83121  3.39189  2.74201  4.25158  3.15177  3.28078
2.21671  3.30785  3.91417  5.98078  4.17935      24 a - - -
          2.68618  4.42225  2.77519  2.73123  3.46354  2.40513  3.72494
3.29354  2.67741  2.69355  4.24690  2.90347  2.73739  3.18146  2.89801
2.37887  2.77519  2.98518  4.58477  3.61503
          0.00656  5.42598  6.14832  0.61958  0.77255  0.48576  0.95510
     19   2.63638  4.18869  2.59757  2.30195  4.52156  3.59157  3.72288
4.15604  2.42512  3.85945  4.31201  2.76345  3.78577  3.14152  2.73511
1.79666  2.32410  2.79905  4.72010  4.59431      25 s - - -
          2.68618  4.42225  2.77519  2.73123  3.46354  2.40513  3.72494
3.29354  2.67741  2.69355  4.24690  2.90347  2.73739  3.18146  2.89801
2.37887  2.77519  2.98518  4.58477  3.61503
          0.00656  5.42598  6.14832  0.61958  0.77255  0.48576  0.95510
     20   2.96159  4.49750  2.91591  3.12070  4.85452  3.02883  4.32985
4.29443  2.99415  3.87215  4.65959  3.60283  4.39355  3.47098  3.58834
0.92347  1.95024  3.62111  6.07857  4.73901      26 s - - -
          2.68618  4.42225  2.77519  2.73123  3.46354  2.40513  3.72494
3.29354  2.67741  2.69355  4.24690  2.90347  2.73739  3.18146  2.89801
2.37887  2.77519  2.98518  4.58477  3.61503
          0.00656  5.42598  6.14832  0.61958  0.77255  0.48576  0.95510
     21   1.65091  3.07787  4.80754  4.21058  3.66422  4.27145  3.12412
2.32010  4.05550  2.34448  3.08225  4.33185  1.65383  4.24089  4.16695
3.41612  3.37076  2.62193  5.18332  3.99929      27 a - - -
          2.68618  4.42225  2.77519  2.73123  3.46354  2.40513  3.72494
3.29354  2.67741  2.69355  4.24690  2.90347  2.73739  3.18146  2.89801
2.37887  2.77519  2.98518  4.58477  3.61503
          0.00656  5.42598  6.14832  0.61958  0.77255  0.48576  0.95510
     22   2.83258  5.58587  2.00279  2.29228  4.93131  3.68049  3.71877
4.41753  2.19578  3.42474  4.26144  2.81647  4.24149  2.54866  2.93631
2.42018  1.97893  3.51588  6.01834  4.60717      28 t - - -
          2.68623  4.42231  2.77525  2.73129  3.46359  2.40518  3.72500
3.29359  2.67746  2.69360  4.24695  2.90301  2.73745  3.18152  2.89806
2.37892  2.77525  2.98468  4.58482  3.61509
          0.05267  3.01239  6.14832  0.66771  0.71925  0.48576  0.95510
     23   2.21022  5.58536  2.44739  1.89446  4.39300  2.58672  3.65253
4.41667  1.90368  3.57740  4.33882  3.04968  4.24155  2.83503  2.98889
2.69247  2.99897  3.40475  6.01795  4.60690      31 e - - -
          2.68618  4.42225  2.77519  2.73123  3.46354  2.40513  3.72494
3.29354  2.67741  2.69355  4.24690  2.90347  2.73739  3.18146  2.89801
2.37887  2.77519  2.98518  4.58477  3.61503
          0.00656  5.42598  6.14832  0.61958  0.77255  0.48576  0.95510
     24   1.86547  1.71308  4.92486  4.32782  3.67903  3.19183  4.64147
2.72384  4.15620  2.30395  3.69867  4.39996  2.80198  4.32528  4.23613
3.59550  3.14716  1.78362  5.20371  4.02273      32 c - - -
          2.68618  4.42225  2.77519  2.73123  3.46354  2.40513  3.72494
3.29354  2.67741  2.69355  4.24690  2.90347  2.73739  3.18146  2.89801
2.37887  2.77519  2.98518  4.58477  3.61503
          0.00656  5.42598  6.14832  0.61958  0.77255  0.48576  0.95510
     25   5.24325  6.23372  6.15789  6.03308  2.68700  5.79814  2.85516
4.89029  5.79487  4.10432  5.42502  5.36047  6.05932  5.51438  5.63424
5.19091  5.43921  3.60516  0.36373  2.50967      33 W - - -
          2.68618  4.42225  2.77519  2.73123  3.46354  2.40513  3.72494
3.29354  2.67741  2.69355  4.24690  2.90347  2.73739  3.18146  2.89801
2.37887  2.77519  2.98518  4.58477  3.61503
          0.00656  5.42598  6.14832  0.61958  0.77255  0.48576  0.95510
```

```
     26   2.40593  5.58516  2.67161  2.16469  4.93030  3.65707  4.03515
3.89613  2.20593  3.32971  4.62580  2.61755  4.24158  2.21792  2.57698
2.27843  2.68626  3.34303  6.01780  4.21281      34 e - - -
          2.68618  4.42225  2.77519  2.73123  3.46354  2.40513  3.72494
3.29354  2.67741  2.69355  4.24690  2.90347  2.73739  3.18146  2.89801
2.37887  2.77519  2.98518  4.58477  3.61503
          0.00656  5.42598  6.14832  0.61958  0.77255  0.48576  0.95510
     27   2.36585  5.58070  3.01865  2.07297  4.92390  3.57644  3.57052
3.56302  2.02712  3.29542  2.55050  2.74006  3.53998  2.41672  2.85562
2.74923  3.05882  3.97034  6.01451  4.09920      35 k - - -
          2.68618  4.42225  2.77519  2.73123  3.46354  2.40513  3.72494
3.29354  2.67741  2.69355  4.24690  2.90347  2.73739  3.18146  2.89801
2.37887  2.77519  2.98518  4.58477  3.61503
          0.00656  5.42598  6.14832  0.61958  0.77255  0.48576  0.95510
     28   2.98853  4.63660  5.21321  3.93136  3.76102  4.47444  4.82597
1.42094  4.41424  1.74495  3.76039  4.62391  4.82933  4.56043  4.44445
3.54369  3.50907  1.23237  5.32424  3.67374      36 v - - -
          2.68618  4.42225  2.77519  2.73123  3.46354  2.40513  3.72494
3.29354  2.67741  2.69355  4.24690  2.90347  2.73739  3.18146  2.89801
2.37887  2.77519  2.98518  4.58477  3.61503
          0.00656  5.42598  6.14832  0.61958  0.77255  0.48576  0.95510
     29   3.01979  3.46551  5.04339  4.43224  2.57814  4.30366  4.63397
2.16919  4.23055  1.11978  3.45759  4.02887  4.66796  3.69657  2.88642
3.39335  2.94977  2.56461  5.14622  3.23699      37 l - - -
          2.68618  4.42225  2.77519  2.73123  3.46354  2.40513  3.72494
3.29354  2.67741  2.69355  4.24690  2.90347  2.73739  3.18146  2.89801
2.37887  2.77519  2.98518  4.58477  3.61503
          0.00656  5.42598  6.14832  0.61958  0.77255  0.48576  0.95510
     30   3.07454  4.86042  2.26169  1.72927  4.93414  3.50985  3.19927
4.42091  2.00579  3.40453  4.62826  2.84484  4.24133  2.36333  2.47466
2.71069  3.02475  3.97916  6.01985  4.60822      38 e - - -
          2.68618  4.42225  2.77519  2.73123  3.46354  2.40513  3.72494
3.29354  2.67741  2.69355  4.24690  2.90347  2.73739  3.18146  2.89801
2.37887  2.77519  2.98518  4.58477  3.61503
          0.00656  5.42598  6.14832  0.61958  0.77255  0.48576  0.95510
     31   1.93899  5.57618  2.89458  2.77145  4.91737  3.51952  3.84618
4.03625  1.92177  3.37284  4.61787  3.04730  2.83993  2.83574  1.89633
2.67869  3.23684  3.41512  6.01116  4.60263      39 r - - -
          2.68618  4.42225  2.77519  2.73123  3.46354  2.40513  3.72494
3.29354  2.67741  2.69355  4.24690  2.90347  2.73739  3.18146  2.89801
2.37887  2.77519  2.98518  4.58477  3.61503
          0.00656  5.42598  6.14832  0.61958  0.77255  0.48576  0.95510
     32   3.12924  5.18072  6.05260  5.54594  4.48874  5.47530  6.06595
1.45444  5.44618  1.58851  3.99726  5.64060  5.72516  5.64509  5.54568
4.86950  3.91193  0.87393  6.42919  5.23385      40 v - - -
          2.68618  4.42225  2.77519  2.73123  3.46354  2.40513  3.72494
3.29354  2.67741  2.69355  4.24690  2.90347  2.73739  3.18146  2.89801
2.37887  2.77519  2.98518  4.58477  3.61503
          0.00656  5.42598  6.14832  0.61958  0.77255  0.48576  0.95510
     33   2.24331  4.42156  3.20559  2.36217  4.34417  3.65775  3.60687
4.40827  2.39319  3.52892  4.62172  1.93000  4.24244  1.92907  2.71858
2.52348  3.00733  3.66095  6.01440  4.60462      41 q - - -
          2.68618  4.42225  2.77519  2.73123  3.46354  2.40513  3.72494
3.29354  2.67741  2.69355  4.24690  2.90347  2.73739  3.18146  2.89801
2.37887  2.77519  2.98518  4.58477  3.61503
          0.00656  5.42598  6.14832  0.61958  0.77255  0.48576  0.95510
```

```
     34   2.63487  4.83675  2.53221  1.74833  4.37310  3.84788  3.71421
4.41835  2.03598  3.47590  4.30318  2.40471  4.24136  2.37686  2.68435
2.71540  2.95811  3.68400  6.01865  4.60735     42 e - - -
          2.68641  4.42259  2.77530  2.73008  3.46387  2.40546  3.72501
3.29075  2.67754  2.69378  4.24590  2.90380  2.73773  3.18143  2.89806
2.37910  2.77541  2.98536  4.58510  3.61537
          0.27670  1.70312  2.82010  1.05131  0.42998  0.48576  0.95510
     35   1.92588  3.74907  4.21302  3.63857  3.64419  4.12717  4.40306
2.92987  2.18336  1.77024  2.80576  3.78779  4.24020  3.59342  2.87223
2.65161  3.03851  2.66585  4.41620  4.06866     46 l - - -
          2.68618  4.42225  2.77519  2.73123  3.46354  2.40513  3.72494
3.29354  2.67741  2.69355  4.24690  2.90347  2.73739  3.18146  2.89801
2.37887  2.77519  2.98518  4.58477  3.61503
          0.00696  5.36680  6.08914  0.61958  0.77255  0.70737  0.67912
     36   3.09260  4.61534  3.39367  2.69487  3.89631  3.70963  3.43132
4.24909  2.36120  3.24036  4.53890  3.36413  4.26755  2.44735  1.05388
3.00323  3.20664  3.85928  5.93315  4.56115     47 r - - -
          2.68618  4.42225  2.77519  2.73123  3.46354  2.40513  3.72494
3.29354  2.67741  2.69355  4.24690  2.90347  2.73739  3.18146  2.89801
2.37887  2.77519  2.98518  4.58477  3.61503
          0.00696  5.36680  6.08914  0.61958  0.77255  0.70737  0.67912
     37   2.45157  4.16933  2.98925  2.31690  4.15779  2.76121  3.42994
3.87436  2.03208  3.47715  4.59233  3.04961  3.90091  2.73325  2.16323
2.20186  2.90109  3.94114  5.98485  4.57478     48 k - - -
          2.68618  4.42225  2.77519  2.73123  3.46354  2.40513  3.72494
3.29354  2.67741  2.69355  4.24690  2.90347  2.73739  3.18146  2.89801
2.37887  2.77519  2.98518  4.58477  3.61503
          0.00696  5.36680  6.08914  0.61958  0.77255  0.70737  0.67912
     38   2.39290  4.74709  2.82996  1.98833  4.89587  3.46307  3.30416
3.72844  2.19938  3.53039  3.84069  2.45240  4.21173  2.37649  3.06741
2.34857  2.79445  3.41110  5.98535  4.57510     49 e - - -
          2.68618  4.42225  2.77519  2.73123  3.46354  2.40513  3.72494
3.29354  2.67741  2.69355  4.24690  2.90347  2.73739  3.18146  2.89801
2.37887  2.77519  2.98518  4.58477  3.61503
          0.00696  5.36680  6.08914  0.61958  0.77255  0.70737  0.67912
     39   2.15866  4.42705  2.93590  2.41143  4.87141  3.62896  3.00295
3.89111  2.51235  2.55638  3.71494  2.66980  3.62748  2.34423  2.76313
2.47722  2.77543  3.56100  5.97269  4.56694     50 a - - -
          2.68618  4.42225  2.77519  2.73123  3.46354  2.40513  3.72494
3.29354  2.67741  2.69355  4.24690  2.90347  2.73739  3.18146  2.89801
2.37887  2.77519  2.98518  4.58477  3.61503
          0.00696  5.36680  6.08914  0.61958  0.77255  0.65566  0.73209
     40   2.67828  5.54839  3.05552  2.52967  4.55798  1.75436  3.71599
3.97598  2.20780  3.21455  4.24693  2.74994  3.60861  2.70854  2.33795
2.82740  3.05354  3.31686  5.98376  4.57552     51 g - - -
          2.68618  4.42225  2.77519  2.73123  3.46354  2.40513  3.72494
3.29354  2.67741  2.69355  4.24690  2.90347  2.73739  3.18146  2.89801
2.37887  2.77519  2.98518  4.58477  3.61503
          0.00691  5.37479  6.09713  0.61958  0.77255  0.68087  0.70558
     41   2.71714  4.47852  3.19049  2.51923  3.57780  3.44836  4.17992
2.72633  2.45064  2.00191  2.51883  3.30854  3.90106  3.10205  2.82825
2.72208  2.97544  2.83557  4.73853  4.30590     52 l - - -
          2.68618  4.42225  2.77519  2.73123  3.46354  2.40513  3.72494
3.29354  2.67741  2.69355  4.24690  2.90347  2.73739  3.18146  2.89801
2.37887  2.77519  2.98518  4.58477  3.61503
          0.24984  5.37479  1.53045  0.61958  0.77255  0.38063  1.15020
```

```
      42   2.42116  5.45814  2.93506  2.49343  3.77097  3.56621  3.76097
4.04711  2.06022  3.15840  4.23431  2.55655  2.58887  2.59976  2.78096
2.40688  2.66544  3.43839  5.89469  4.48764      53 k - - -
         2.68678  4.42314  2.77570  2.73151  3.46312  2.40545  3.72479
3.29425  2.67829  2.69165  4.24694  2.90288  2.73542  3.18163  2.89877
2.37858  2.77563  2.98550  4.58566  3.61541
         1.51736  0.76429  1.15505  1.31080  0.31417  0.22919  1.58562
      43   2.62259  3.81066  3.63343  3.39710  3.22886  4.07882  4.38292
2.49322  3.74307  1.24909  3.56109  3.75833  2.94741  3.96021  3.91806
3.20498  2.93648  2.12691  5.05790  3.86672      60 l - - -
         2.68618  4.42225  2.77519  2.73123  3.46354  2.40513  3.72494
3.29354  2.67741  2.69355  4.24690  2.90347  2.73739  3.18146  2.89801
2.37887  2.77519  2.98518  4.58477  3.61503
         0.21281  5.13990  1.68293  0.61958  0.77255  0.89273  0.52685
      44   2.75687  4.52949  3.03238  2.39590  4.72458  3.34824  3.22127
4.20715  1.82953  3.37400  4.42620  2.85140  3.80933  2.04423  2.46212
2.56142  3.11624  3.17226  5.81980  3.91890      61 k - - -
         2.68618  4.42225  2.77519  2.73123  3.46354  2.40513  3.72494
3.29354  2.67741  2.69355  4.24690  2.90347  2.73739  3.18146  2.89801
2.37887  2.77519  2.98518  4.58477  3.61503
         0.00984  5.02250  5.74485  0.61958  0.77255  1.11042  0.39961
      45   2.49995  3.91843  3.55167  2.98746  3.18408  3.90870  4.16047
2.71863  2.97683  1.68938  3.51582  3.26834  2.50511  3.20017  3.23603
2.82517  2.75577  2.74126  5.24511  4.00882      62 l - - -
         2.68618  4.42225  2.77519  2.73123  3.46354  2.40513  3.72494
3.29354  2.67741  2.69355  4.24690  2.90347  2.73739  3.18146  2.89801
2.37887  2.77519  2.98518  4.58477  3.61503
         0.24422  5.08512  1.55828  0.61958  0.77255  1.25346  0.33620
      46   2.82735  4.89976  3.00480  2.75641  1.82495  3.68962  3.46190
3.50248  2.44428  2.56096  3.98060  3.02788  3.06777  3.08521  2.84494
2.59858  2.97059  3.21277  4.71264  4.12027      63 f - - -
         2.68618  4.42225  2.77519  2.73123  3.46354  2.40513  3.72494
3.29354  2.67741  2.69355  4.24690  2.90347  2.73739  3.18146  2.89801
2.37887  2.77519  2.98518  4.58477  3.61503
         0.01154  4.86333  5.58568  0.61958  0.77255  0.72843  0.65907
      47   2.93954  5.17833  3.16120  2.65399  3.37940  3.20493  3.65992
3.68719  2.46749  2.74908  3.53353  3.13400  1.96419  2.87043  2.96787
2.39227  2.75598  2.78299  5.67332  4.32925      64 p - - -
         2.68618  4.42225  2.77519  2.73123  3.46354  2.40513  3.72494
3.29354  2.67741  2.69355  4.24690  2.90347  2.73739  3.18146  2.89801
2.37887  2.77519  2.98518  4.58477  3.61503
         0.00879  5.13387  5.85621  0.61958  0.77255  0.60913  0.78487
      48   2.66572  4.48366  2.74691  2.06290  4.83274  3.12133  3.96826
3.70763  2.29370  3.34061  3.63642  2.71334  3.17495  2.54082  3.08768
2.42394  2.53733  2.87968  5.93295  4.52678      65 e - - -
         2.68618  4.42225  2.77519  2.73123  3.46354  2.40513  3.72494
3.29354  2.67741  2.69355  4.24690  2.90347  2.73739  3.18146  2.89801
2.37887  2.77519  2.98518  4.58477  3.61503
         0.01482  5.28430  4.64137  0.61958  0.77255  0.31204  1.31660
      49   2.59755  5.36864  3.10031  2.69913  3.14207  2.72413  3.41729
3.55644  2.78630  3.32729  3.57694  3.16754  3.76114  2.78829  3.17680
1.84178  2.66255  3.32224  5.85200  2.56284      66 s - - -
         2.68618  4.42225  2.77519  2.73123  3.46354  2.40513  3.72494
3.29354  2.67741  2.69355  4.24690  2.90347  2.73739  3.18146  2.89801
2.37887  2.77519  2.98518  4.58477  3.61503
         0.00666  5.41126  6.13361  0.61958  0.77255  0.50409  0.92648
```

```
     50   3.00036  4.55342  4.20995  4.23547  3.65043  3.74616  4.58959
1.37840  3.52740  1.87265  2.79325  4.34247  3.49834  3.66208  4.17446
3.41624  2.92453  1.83714  5.17072  3.98774     67 i - - -
          2.68618  4.42225  2.77519  2.73123  3.46354  2.40513  3.72494
3.29354  2.67741  2.69355  4.24690  2.90347  2.73739  3.18146  2.89801
2.37887  2.77519  2.98518  4.58477  3.61503
          0.01246  5.41757  4.83572  0.61958  0.77255  0.52126  0.90084
     51   2.74089  3.94576  2.48354  2.76792  4.89711  3.84434  4.03226
4.06867  2.36685  3.86177  4.60251  2.10490  3.79761  3.02901  2.72898
1.65570  2.40226  3.52868  5.99716  4.59094     68 s - - -
          2.68618  4.42225  2.77519  2.73123  3.46354  2.40513  3.72494
3.29354  2.67741  2.69355  4.24690  2.90347  2.73739  3.18146  2.89801
2.37887  2.77519  2.98518  4.58477  3.61503
          0.00665  5.41177  6.13411  0.61958  0.77255  0.50542  0.92446
     52   2.74847  5.32074  3.34230  4.06024  5.77282  0.36087  5.38995
5.26421  4.49138  4.89322  5.67512  4.28460  3.70537  4.64432  4.86673
3.01133  3.84440  4.11315  7.06673  5.83638     69 G - - -
          2.68618  4.42225  2.77519  2.73123  3.46354  2.40513  3.72494
3.29354  2.67741  2.69355  4.24690  2.90347  2.73739  3.18146  2.89801
2.37887  2.77519  2.98518  4.58477  3.61503
          0.00661  5.41757  6.13992  0.61958  0.77255  0.46419  0.99060
     53   2.39345  4.68964  3.35155  1.74312  3.13394  3.95322  3.24779
3.22457  2.93880  2.13678  4.25058  3.51968  2.58426  3.18422  3.26788
2.79597  3.02924  3.04546  5.69620  4.01731     70 e - - -
          2.68618  4.42225  2.77519  2.73123  3.46354  2.40513  3.72494
3.29354  2.67741  2.69355  4.24690  2.90347  2.73739  3.18146  2.89801
2.37887  2.77519  2.98518  4.58477  3.61503
          0.00656  5.42598  6.14832  0.61958  0.77255  0.48576  0.95510
     54   2.84906  4.61726  1.79445  1.66697  4.93115  3.43734  3.76439
4.41733  2.58429  3.89119  4.62637  2.85536  4.24156  2.75838  2.55389
2.54432  3.03206  3.62066  6.01829  3.58011     71 e - - -
          2.68618  4.42225  2.77519  2.73123  3.46354  2.40513  3.72494
3.29354  2.67741  2.69355  4.24690  2.90347  2.73739  3.18146  2.89801
2.37887  2.77519  2.98518  4.58477  3.61503
          0.00656  5.42598  6.14832  0.61958  0.77255  0.48576  0.95510
     55   2.82609  4.53187  5.00029  4.39191  2.32179  4.29826  4.00489
2.69898  4.19918  1.51146  1.50494  4.07471  4.66298  3.90188  3.78083
3.20686  3.37273  2.69701  5.15182  3.25326     72 m - - -
          2.68618  4.42225  2.77519  2.73123  3.46354  2.40513  3.72494
3.29354  2.67741  2.69355  4.24690  2.90347  2.73739  3.18146  2.89801
2.37887  2.77519  2.98518  4.58477  3.61503
          0.00656  5.42598  6.14832  0.61958  0.77255  0.48576  0.95510
     56   3.79191  5.11343  5.58390  5.05608  0.68414  4.90437  4.75132
3.15241  4.85124  1.90226  3.40464  4.94332  5.21982  4.88457  4.83241
3.78360  4.01721  3.31497  5.00442  2.34496     73 F - - -
          2.68618  4.42225  2.77519  2.73123  3.46354  2.40513  3.72494
3.29354  2.67741  2.69355  4.24690  2.90347  2.73739  3.18146  2.89801
2.37887  2.77519  2.98518  4.58477  3.61503
          0.00656  5.42598  6.14832  0.61958  0.77255  0.48576  0.95510
     57   3.18282  5.35179  4.99337  4.98080  5.76648  0.21288  5.92393
5.23752  5.09478  3.53630  5.88289  4.80524  4.96502  5.33379  5.21805
3.65992  4.02278  4.56739  7.14347  5.98743     74 G - - -
          2.68618  4.42225  2.77519  2.73123  3.46354  2.40513  3.72494
3.29354  2.67741  2.69355  4.24690  2.90347  2.73739  3.18146  2.89801
2.37887  2.77519  2.98518  4.58477  3.61503
          0.00656  5.42598  6.14832  0.61958  0.77255  0.48576  0.95510
```

58 4.02064 5.31042 5.98711 5.38175 1.71292 5.27594 5.55246
2.61313 5.19685 0.68343 3.23576 5.44228 5.47081 5.16279 5.15034
4.61909 4.23452 2.53136 5.77106 3.48830 75 L - - -
2.68618 4.42225 2.77519 2.73123 3.46354 2.40513 3.72494
3.29354 2.67741 2.69355 4.24690 2.90347 2.73739 3.18146 2.89801
2.37887 2.77519 2.98518 4.58477 3.61503
0.00656 5.42598 6.14832 0.61958 0.77255 0.48576 0.95510
59 2.80959 5.53130 3.09597 2.79184 4.85357 3.42569 3.74979
4.32374 2.52357 2.33862 4.57809 2.56155 3.93189 2.94447 3.15479
1.80499 1.76081 3.72822 5.97793 4.58119 76 t - - -
2.68618 4.42225 2.77519 2.73123 3.46354 2.40513 3.72494
3.29354 2.67741 2.69355 4.24690 2.90347 2.73739 3.18146 2.89801
2.37887 2.77519 2.98518 4.58477 3.61503
0.00656 5.42598 6.14832 0.61958 0.77255 0.48576 0.95510
60 2.53318 5.55897 2.77550 1.93501 4.19401 3.85333 2.25963
4.02332 2.78082 3.32791 4.60260 2.36252 4.24656 2.94003 3.27169
2.01248 2.91415 3.12950 5.99845 4.59436 77 e - - -
2.68618 4.42225 2.77519 2.73123 3.46354 2.40513 3.72494
3.29354 2.67741 2.69355 4.24690 2.90347 2.73739 3.18146 2.89801
2.37887 2.77519 2.98518 4.58477 3.61503
0.00656 5.42598 6.14832 0.61958 0.77255 0.48576 0.95510
61 2.88722 5.64359 2.71166 2.52164 4.98268 3.72529 3.52088
4.46895 2.72547 3.94646 4.68823 2.52659 1.17660 3.05341 3.33490
2.57002 2.97884 4.03148 6.07796 4.66301 78 p - - -
2.68618 4.42225 2.77519 2.73123 3.46354 2.40513 3.72494
3.29354 2.67741 2.69355 4.24690 2.90347 2.73739 3.18146 2.89801
2.37887 2.77519 2.98518 4.58477 3.61503
0.00656 5.42598 6.14832 0.61958 0.77255 0.48576 0.95510
62 1.62195 5.32474 3.46174 2.74653 3.66942 3.49218 2.99033
3.99758 2.65217 3.00415 4.09817 3.12346 3.48559 2.91956 3.13672
2.51243 2.29178 2.70291 4.79488 4.47505 79 a - - -
2.68618 4.42225 2.77519 2.73123 3.46354 2.40513 3.72494
3.29354 2.67741 2.69355 4.24690 2.90347 2.73739 3.18146 2.89801
2.37887 2.77519 2.98518 4.58477 3.61503
0.00656 5.42598 6.14832 0.61958 0.77255 0.48576 0.95510
63 4.01299 5.22352 6.12609 5.63094 3.96160 5.57343 6.19892
0.95476 5.54576 2.52069 3.68464 5.73939 5.80422 5.74773 5.65434
4.97953 3.56484 0.92330 6.53032 5.33623 80 v - - -
2.68618 4.42225 2.77519 2.73123 3.46354 2.40513 3.72494
3.29354 2.67741 2.69355 4.24690 2.90347 2.73739 3.18146 2.89801
2.37887 2.77519 2.98518 4.58477 3.61503
0.00656 5.42598 6.14832 0.61958 0.77255 0.48576 0.95510
64 2.70161 3.79868 4.26064 3.68574 3.82818 4.16442 3.72685
2.12776 2.87267 2.27773 3.00057 3.61959 4.53899 2.58702 2.67397
3.32669 2.57824 1.78990 5.30952 4.09912 81 v - - -
2.68618 4.42225 2.77519 2.73123 3.46354 2.40513 3.72494
3.29354 2.67741 2.69355 4.24690 2.90347 2.73739 3.18146 2.89801
2.37887 2.77519 2.98518 4.58477 3.61503
0.00656 5.42598 6.14832 0.61958 0.77255 0.48576 0.95510
65 2.88191 5.53305 3.34965 2.67177 3.20841 3.86061 3.30388
3.86041 2.15897 3.40828 4.57973 2.36396 4.25340 2.27716 1.82884
2.94817 3.30927 3.91171 5.97896 2.59345 82 r - - -
2.68618 4.42225 2.77519 2.73123 3.46354 2.40513 3.72494
3.29354 2.67741 2.69355 4.24690 2.90347 2.73739 3.18146 2.89801
2.37887 2.77519 2.98518 4.58477 3.61503
0.00656 5.42598 6.14832 0.61958 0.77255 0.48576 0.95510

66 2.13242 3.85330 5.10803 4.12301 3.32064 4.31740 4.65132
1.89976 4.27916 1.21267 3.20080 4.47871 4.68072 4.41105 4.28871
3.40575 3.37960 2.06603 5.14562 3.46891 83 l - - -
2.68618 4.42225 2.77519 2.73123 3.46354 2.40513 3.72494
3.29354 2.67741 2.69355 4.24690 2.90347 2.73739 3.18146 2.89801
2.37887 2.77519 2.98518 4.58477 3.61503
0.00656 5.42598 6.14832 0.61958 0.77255 0.48576 0.95510
67 2.92168 4.78539 5.43462 4.84057 3.49885 4.69094 5.06043
1.14431 4.64885 1.44075 3.32854 4.85191 5.02403 4.78110 4.67057
4.01706 2.58442 1.99022 5.51864 4.34998 84 i - - -
2.68618 4.42225 2.77519 2.73123 3.46354 2.40513 3.72494
3.29354 2.67741 2.69355 4.24690 2.90347 2.73739 3.18146 2.89801
2.37887 2.77519 2.98518 4.58477 3.61503
0.00656 5.42598 6.14832 0.61958 0.77255 0.48576 0.95510
68 3.38500 5.85079 3.34234 0.75157 5.19795 4.02861 4.26630
4.66756 2.96392 3.51558 4.91770 3.49970 4.47591 1.99214 3.18668
2.97698 3.61884 4.25040 6.26464 4.87638 85 e - - -
2.68618 4.42225 2.77519 2.73123 3.46354 2.40513 3.72494
3.29354 2.67741 2.69355 4.24690 2.90347 2.73739 3.18146 2.89801
2.37887 2.77519 2.98518 4.58477 3.61503
0.00656 5.42598 6.14832 0.61958 0.77255 0.48576 0.95510
69 2.07489 5.58627 3.06883 2.45799 4.92960 3.85105 3.77172
4.41455 2.51923 3.89077 4.38457 2.86888 4.24566 1.88448 3.26859
1.50677 3.04178 3.97602 6.01983 4.60956 86 s - - -
2.68618 4.42225 2.77519 2.73123 3.46354 2.40513 3.72494
3.29354 2.67741 2.69355 4.24690 2.90347 2.73739 3.18146 2.89801
2.37887 2.77519 2.98518 4.58477 3.61503
0.00656 5.42598 6.14832 0.61958 0.77255 0.48576 0.95510
70 3.07795 2.97768 5.01944 4.41191 3.50814 4.31916 4.65092
2.97592 4.21543 0.77811 2.37361 4.44802 4.11489 4.09106 3.99146
3.45270 3.39634 2.79810 5.17521 3.99884 87 l - - -
2.68618 4.42225 2.77519 2.73123 3.46354 2.40513 3.72494
3.29354 2.67741 2.69355 4.24690 2.90347 2.73739 3.18146 2.89801
2.37887 2.77519 2.98518 4.58477 3.61503
0.00656 5.42598 6.14832 0.61958 0.77255 0.48576 0.95510
71 2.89612 5.57904 2.03740 2.33042 4.50424 3.85760 4.05029
4.39559 2.62764 3.88044 4.62255 3.32793 1.32344 2.97037 3.11954
2.70174 3.20026 3.96494 6.01668 3.82014 88 p - - -
2.68618 4.42225 2.77519 2.73123 3.46354 2.40513 3.72494
3.29354 2.67741 2.69355 4.24690 2.90347 2.73739 3.18146 2.89801
2.37887 2.77519 2.98518 4.58477 3.61503
0.00656 5.42598 6.14832 0.61958 0.77255 0.48576 0.95510
72 3.08114 4.94466 3.17782 2.63925 4.88376 1.30134 4.04890
4.35885 2.61782 3.85282 4.59945 2.06792 2.77942 3.15076 2.93956
2.92190 3.16585 3.77583 5.99629 4.26696 89 g - - -
2.68618 4.42225 2.77519 2.73123 3.46354 2.40513 3.72494
3.29354 2.67741 2.69355 4.24690 2.90347 2.73739 3.18146 2.89801
2.37887 2.77519 2.98518 4.58477 3.61503
0.00656 5.42598 6.14832 0.61958 0.77255 0.48576 0.95510
73 1.45632 4.16450 3.82418 3.74433 3.34842 3.90430 4.46057
2.12303 3.32833 2.82728 3.79996 2.74519 4.55213 3.92321 3.65927
2.93806 3.09002 1.87001 5.29047 3.72870 90 a - - -
2.68618 4.42225 2.77519 2.73123 3.46354 2.40513 3.72494
3.29354 2.67741 2.69355 4.24690 2.90347 2.73739 3.18146 2.89801
2.37887 2.77519 2.98518 4.58477 3.61503
0.00656 5.42598 6.14832 0.61958 0.77255 0.48576 0.95510

```
     74   3.07789  5.59122  2.00514  1.69036  4.48181  3.65764  2.39672
4.42397  2.47655  3.89683  4.63178  2.85514  4.24384  2.91879  2.21954
2.74747  3.05154  3.98238  6.02318  4.61140     91 e - - -
          2.68618  4.42225  2.77519  2.73123  3.46354  2.40513  3.72494
3.29354  2.67741  2.69355  4.24690  2.90347  2.73739  3.18146  2.89801
2.37887  2.77519  2.98518  4.58477  3.61503
          0.00656  5.42598  6.14832  0.61958  0.77255  0.48576  0.95510
     75   2.84296  5.57107  3.23756  2.35980  4.91009  3.63281  3.41337
4.12752  1.77144  2.60476  4.17954  2.52795  4.24420  2.55163  2.71514
2.54412  2.57565  3.04673  6.00739  3.95703     92 k - - -
          2.68618  4.42225  2.77519  2.73123  3.46354  2.40513  3.72494
3.29354  2.67741  2.69355  4.24690  2.90347  2.73739  3.18146  2.89801
2.37887  2.77519  2.98518  4.58477  3.61503
          0.00656  5.42598  6.14832  0.61958  0.77255  0.48576  0.95510
     76   3.24476  1.00683  5.14967  4.54080  3.39970  4.41525  4.74841
2.67780  4.34081  1.61558  3.20431  4.55956  4.76927  4.19126  4.36867
3.21677  3.47685  2.75109  5.24082  4.07175     93 c - - -
          2.68618  4.42225  2.77519  2.73123  3.46354  2.40513  3.72494
3.29354  2.67741  2.69355  4.24690  2.90347  2.73739  3.18146  2.89801
2.37887  2.77519  2.98518  4.58477  3.61503
          0.00656  5.42598  6.14832  0.61958  0.77255  0.48576  0.95510
     77   2.48094  5.53890  3.20740  2.44724  3.95202  3.85733  3.37338
3.95874  2.27832  3.17605  3.90618  2.99428  3.78171  2.44460  2.80369
2.32831  2.11262  2.57605  5.98348  4.58464     94 t - - -
          2.68618  4.42225  2.77519  2.73123  3.46354  2.40513  3.72494
3.29354  2.67741  2.69355  4.24690  2.90347  2.73739  3.18146  2.89801
2.37887  2.77519  2.98518  4.58477  3.61503
          0.02656  5.42598  3.82522  0.61958  0.77255  0.48576  0.95510
     78   2.72989  5.58122  2.39797  2.06976  4.92736  2.69255  3.63204
4.41429  2.09770  3.88692  4.62172  1.79108  4.23331  2.85167  2.66882
2.88663  2.95957  3.97246  6.01310  4.60116     95 n - - -
          2.68618  4.42225  2.77519  2.73123  3.46354  2.40513  3.72494
3.29354  2.67741  2.69355  4.24690  2.90347  2.73739  3.18146  2.89801
2.37887  2.77519  2.98518  4.58477  3.61503
          0.00669  5.40610  6.12845  0.61958  0.77255  0.56730  0.83715
     79   5.42324  6.33535  6.19462  6.14636  2.67247  5.86641  3.64374
5.02500  5.90160  4.16033  5.53902  5.38833  6.12318  5.56684  5.70470
5.27819  5.61697  3.71235  4.56195  0.21665     96 Y - - -
          2.68666  4.42286  2.77568  2.73153  3.46394  2.40568  3.72464
3.29321  2.67776  2.69387  4.24641  2.90302  2.73744  3.18129  2.89762
2.37913  2.77552  2.98476  4.56987  3.61528
          0.40947  1.09716  6.12845  1.07134  0.41938  0.43796  1.03664
     80   2.93656  4.54659  3.35199  2.71067  3.78718  3.86130  4.05081
4.31337  1.50874  3.32865  4.57274  2.60256  4.02146  2.50869  1.89134
2.94353  2.84327  3.10112  5.97323  4.57847    100 k - - -
          2.68618  4.42225  2.77519  2.73123  3.46354  2.40513  3.72494
3.29354  2.67741  2.69355  4.24690  2.90347  2.73739  3.18146  2.89801
2.37887  2.77519  2.98518  4.58477  3.61503
          0.00656  5.42598  6.14832  0.61958  0.77255  0.48576  0.95510
     81   2.79851  5.08775  3.32310  2.80580  1.30778  3.63657  3.73949
3.18260  2.55035  2.84027  3.28185  3.28636  2.95177  3.20658  3.50565
3.12690  3.24408  3.20006  5.62895  4.34055    101 f - - -
          2.68618  4.42225  2.77519  2.73123  3.46354  2.40513  3.72494
3.29354  2.67741  2.69355  4.24690  2.90347  2.73739  3.18146  2.89801
2.37887  2.77519  2.98518  4.58477  3.61503
          0.00656  5.42598  6.14832  0.61958  0.77255  0.48576  0.95510
```

```
     82   2.94373  5.58626  2.73455  2.26866  4.93187  3.70880  3.57513
4.41820  1.78331  3.69234  4.23199  2.92275  4.24147  2.67673  1.72818
2.57382  2.96140  3.36571  6.01861  4.11202    102 r - - -
          2.68618  4.42225  2.77519  2.73123  3.46354  2.40513  3.72494
3.29354  2.67741  2.69355  4.24690  2.90347  2.73739  3.18146  2.89801
2.37887  2.77519  2.98518  4.58477  3.61503
          0.01282  5.42598  4.78683  0.61958  0.77255  0.48576  0.95510
     83   2.60083  4.53112  3.43111  2.79475  2.14870  3.81741  3.10724
3.38193  2.95594  3.15161  4.15836  3.21820  3.87739  3.25429  3.01572
2.76098  2.79193  2.59104  5.61432  1.94986    103 y - - -
          2.68618  4.42225  2.77519  2.73123  3.46354  2.40513  3.72494
3.29354  2.67741  2.69355  4.24690  2.90347  2.73739  3.18146  2.89801
2.37887  2.77519  2.98518  4.58477  3.61503
          0.02200  5.41975  4.05499  0.61958  0.77255  0.51219  0.91425
     84   2.71407  3.37311  3.10097  2.49134  3.53813  1.93216  2.41369
4.07538  2.43109  3.57986  4.58156  2.96338  3.86704  2.76218  3.03603
2.36334  3.12804  3.48177  5.97900  4.57781    104 g - - -
          2.68618  4.42225  2.77519  2.73123  3.46354  2.40513  3.72494
3.29354  2.67741  2.69355  4.24690  2.90347  2.73739  3.18146  2.89801
2.37887  2.77519  2.98518  4.58477  3.61503
          0.00452  5.40227        *  0.61958  0.77255  0.00000        *
//
HMMER3/f [3.3 | Nov 2019]
NAME  FYRN
ACC   PF05964.15
DESC  F/Y-rich N-terminus
LENG  51
ALPH  amino
RF    no
MM    no
CONS  yes
CS    no
MAP   yes
DATE  Fri Apr 17 12:05:31 2020
NSEQ  134
EFFN  9.802185
CKSUM 191863786
GA    21.00 21.00
TC    21.00 21.10
NC    20.90 20.80
STATS LOCAL MSV       -8.0706  0.71915
STATS LOCAL VITERBI   -8.4316  0.71915
STATS LOCAL FORWARD   -4.3652  0.71915
HMM          A        C        D        E        F        G        H
I        K        L        M        N        P        Q        R        S
T        V        W        Y
            m->m     m->i     m->d     i->m     i->i     d->m     d->d
  COMPO   3.01589  3.68791  3.25710  2.94455  3.03312  2.83455  3.56501
2.68095  2.72930  2.62332  3.86587  3.24375  3.08804  3.39108  2.74791
2.57887  2.77808  2.66380  4.30580  2.67598
          2.68618  4.42225  2.77519  2.73123  3.46354  2.40513  3.72494
3.29354  2.67741  2.69355  4.24690  2.90347  2.73739  3.18146  2.89801
2.37887  2.77519  2.98518  4.58477  3.61503
          0.00389  5.94709  6.66944  0.61958  0.77255  0.00000        *
      1   2.61595  5.88977  3.19059  2.67748  5.23480  1.05055  3.73967
4.72075  2.84801  3.93390  4.93046  2.96289  3.12884  3.26205  3.07564
2.41637  3.14118  4.28039  6.32247  4.28749      1 g - - -
```

2.68618 4.42225 2.77519 2.73123 3.46354 2.40513 3.72494
3.29354 2.67741 2.69355 4.24690 2.90347 2.73739 3.18146 2.89801
2.37887 2.77519 2.98518 4.58477 3.61503
0.00389 5.94709 6.66944 0.61958 0.77255 0.48576 0.95510
2 2.70984 4.64529 2.57371 2.95584 5.19975 2.60085 4.34609
3.49842 2.52653 3.41011 4.20671 2.66300 4.55113 3.30457 2.99959
1.32128 2.52292 3.83278 6.30438 4.89991 2 s - - -
2.68652 4.42140 2.77530 2.73103 3.46388 2.40547 3.72286
3.29330 2.67753 2.69389 4.24724 2.90381 2.73774 3.18181 2.89694
2.37906 2.77509 2.98553 4.58511 3.61300
0.18085 1.80685 6.66944 0.68726 0.69907 0.48576 0.95510
3 3.07570 3.98280 5.46955 4.85327 2.45727 4.66291 4.99723
2.89339 4.63505 0.88526 2.51537 4.82993 5.02608 4.76326 4.63815
3.42099 3.38724 2.00179 4.19742 4.31094 5 l - - -
2.68618 4.42225 2.77519 2.73123 3.46354 2.40513 3.72494
3.29354 2.67741 2.69355 4.24690 2.90347 2.73739 3.18146 2.89801
2.37887 2.77519 2.98518 4.58477 3.61503
0.00389 5.94709 6.66944 0.61958 0.77255 0.48576 0.95510
4 2.85637 4.79088 3.75526 2.48478 4.54076 4.21081 3.87904
2.81660 2.52239 3.01578 3.79384 3.29698 4.60151 2.57736 2.46506
2.29144 1.68896 2.78243 5.15855 4.79919 6 t - - -
2.68618 4.42225 2.77519 2.73123 3.46354 2.40513 3.72494
3.29354 2.67741 2.69355 4.24690 2.90347 2.73739 3.18146 2.89801
2.37887 2.77519 2.98518 4.58477 3.61503
0.00389 5.94709 6.66944 0.61958 0.77255 0.48576 0.95510
5 4.10889 5.39301 6.15624 5.59530 2.37389 5.46088 5.90182
1.32149 5.43408 1.60766 4.43615 5.62957 5.75971 5.58118 5.47419
4.32953 3.84724 1.09986 6.31636 5.14149 7 v - - -
2.68618 4.42225 2.77519 2.73123 3.46354 2.40513 3.72494
3.29354 2.67741 2.69355 4.24690 2.90347 2.73739 3.18146 2.89801
2.37887 2.77519 2.98518 4.58477 3.61503
0.00389 5.94709 6.66944 0.61958 0.77255 0.48576 0.95510
6 3.40640 5.50033 3.23089 2.70738 4.06657 4.26345 2.24462
2.40128 3.01438 1.65154 4.57954 2.93277 4.65152 3.15493 3.06568
3.28812 2.93502 2.57657 6.02347 2.87030 8 l - - -
2.68618 4.42225 2.77519 2.73123 3.46354 2.40513 3.72494
3.29354 2.67741 2.69355 4.24690 2.90347 2.73739 3.18146 2.89801
2.37887 2.77519 2.98518 4.58477 3.61503
0.00389 5.94709 6.66944 0.61958 0.77255 0.48576 0.95510
7 2.83134 3.69758 2.75422 2.81019 4.61929 4.15296 4.34012
4.72099 2.39000 4.19524 4.93068 1.69654 4.54653 3.05021 2.72168
1.30632 3.50139 4.28062 6.32265 4.91172 9 s - - -
2.68618 4.42225 2.77519 2.73123 3.46354 2.40513 3.72494
3.29354 2.67741 2.69355 4.24690 2.90347 2.73739 3.18146 2.89801
2.37887 2.77519 2.98518 4.58477 3.61503
0.00389 5.94709 6.66944 0.61958 0.77255 0.48576 0.95510
8 3.58770 4.95385 5.56428 4.95003 2.93264 4.76740 5.10270
2.06515 4.73548 0.73683 2.79602 4.93380 4.53386 4.85649 4.73813
3.53009 3.37830 2.32852 5.57684 4.40913 10 l - - -
2.68618 4.42225 2.77519 2.73123 3.46354 2.40513 3.72494
3.29354 2.67741 2.69355 4.24690 2.90347 2.73739 3.18146 2.89801
2.37887 2.77519 2.98518 4.58477 3.61503
0.00389 5.94709 6.66944 0.61958 0.77255 0.48576 0.95510
9 6.19033 7.41070 6.93388 7.01998 7.69655 0.01507 7.69128
7.92621 7.27217 7.15134 8.34201 7.14986 6.73944 7.51820 7.10781
6.45385 6.71733 7.29517 8.14015 7.92186 11 G - - -

2.68618 4.42225 2.77519 2.73123 3.46354 2.40513 3.72494
3.29354 2.67741 2.69355 4.24690 2.90347 2.73739 3.18146 2.89801
2.37887 2.77519 2.98518 4.58477 3.61503
0.00389 5.94709 6.66944 0.61958 0.77255 0.48576 0.95510
10 2.90976 5.88561 3.36793 1.83771 4.41733 4.15335 3.14860
3.27699 1.95149 4.19018 4.57197 3.62146 4.54678 2.11635 2.15601
2.70623 2.54300 3.24498 6.31930 4.31824 12 e - - -
2.68618 4.42225 2.77519 2.73123 3.46354 2.40513 3.72494
3.29354 2.67741 2.69355 4.24690 2.90347 2.73739 3.18146 2.89801
2.37887 2.77519 2.98518 4.58477 3.61503
0.01434 5.94709 4.45469 0.61958 0.77255 0.48576 0.95510
11 4.44796 5.71443 6.46338 5.87741 3.73014 5.77559 6.12838
1.11203 5.71061 1.07182 3.18392 5.95066 5.96857 5.70553 5.68360
5.13215 4.66995 1.66431 6.35069 4.06725 13 l - - -
2.68618 4.42225 2.77519 2.73123 3.46354 2.40513 3.72494
3.29354 2.67741 2.69355 4.24690 2.90347 2.73739 3.18146 2.89801
2.37887 2.77519 2.98518 4.58477 3.61503
0.00393 5.93669 6.65903 0.61958 0.77255 0.44291 1.02768
12 2.81261 4.15114 4.30698 3.65206 3.89011 3.81767 4.29119
2.37645 3.74246 2.05059 3.48007 3.14282 2.27303 3.66236 4.31032
3.08202 2.92291 1.37913 5.60319 4.40197 14 v - - -
2.68618 4.42225 2.77519 2.73123 3.46354 2.40513 3.72494
3.29354 2.67741 2.69355 4.24690 2.90347 2.73739 3.18146 2.89801
2.37887 2.77519 2.98518 4.58477 3.61503
0.01143 5.94709 4.73908 0.61958 0.77255 0.48576 0.95510
13 2.89567 4.71612 3.04980 2.43369 3.64392 3.74589 3.62640
3.75325 2.89633 3.47423 4.46189 3.02996 1.65395 3.51949 3.12260
2.92246 2.23985 3.21149 3.52883 2.81411 15 p - - -
2.68618 4.42225 2.77519 2.73123 3.46354 2.40513 3.72494
3.29354 2.67741 2.69355 4.24690 2.90347 2.73739 3.18146 2.89801
2.37887 2.77519 2.98518 4.58477 3.61503
0.00392 5.93959 6.66193 0.61958 0.77255 0.53870 0.87588
14 2.89026 4.46632 1.71889 2.36748 5.23152 3.19876 2.70956
4.29098 2.33431 2.89732 4.41530 2.91156 2.93845 2.87754 2.85859
2.85016 3.00382 3.44764 6.31818 4.90687 16 d - - -
2.68618 4.42225 2.77519 2.73123 3.46354 2.40513 3.72494
3.29354 2.67741 2.69355 4.24690 2.90347 2.73739 3.18146 2.89801
2.37887 2.77519 2.98518 4.58477 3.61503
0.02220 5.93959 3.94629 0.61958 0.77255 0.53870 0.87588
15 3.36209 5.87471 2.84498 2.54907 3.65975 3.68938 3.19717
4.70718 2.57544 3.16291 4.11726 2.47127 3.64018 2.13633 1.90214
1.88777 3.42492 3.93404 6.30689 4.89543 17 s - - -
2.68626 4.42282 2.77577 2.73152 3.46366 2.40551 3.72521
3.29361 2.67756 2.69252 4.24161 2.90389 2.73786 3.17988 2.89843
2.37873 2.77544 2.98503 4.58534 3.61529
0.93937 1.08165 1.30899 0.50394 0.92671 0.39277 1.12449
16 2.22525 5.69589 2.25480 2.40755 4.35486 3.21192 4.15615
3.99115 2.22619 3.38459 3.84554 3.32468 2.03798 3.05535 2.58334
2.95660 2.69576 3.74093 6.13070 4.72189 21 p - - -
2.68618 4.42225 2.77519 2.73123 3.46354 2.40513 3.72494
3.29354 2.67741 2.69355 4.24690 2.90347 2.73739 3.18146 2.89801
2.37887 2.77519 2.98518 4.58477 3.61503
0.16739 5.64333 1.89322 0.61958 0.77255 0.56236 0.84365
17 2.17776 5.71381 2.76529 2.77586 3.86836 2.38232 3.25228
4.53321 2.18819 3.00691 3.75160 2.47463 3.40116 2.84552 2.73632
2.90076 3.13776 3.60151 4.56535 3.38273 22 a - - -

```
        2.68618  4.42225  2.77519  2.73123  3.46354  2.40513  3.72494
3.29354  2.67741  2.69355  4.24690  2.90347  2.73739  3.18146  2.89801
2.37887  2.77519  2.98518  4.58477  3.61503
        0.00505  5.68730  6.40964  0.61958  0.77255  0.36548  1.18372
     18  5.23773  6.34914  6.49906  6.28276  0.59716  6.00131  4.93364
4.92165  6.05635  3.22430  4.32020  5.69847  6.28515  5.82386  5.90311
2.47390  5.44739  4.80823  2.35667  1.80517     23 F - - -
        2.68618  4.42225  2.77519  2.73123  3.46354  2.40513  3.72494
3.29354  2.67741  2.69355  4.24690  2.90347  2.73739  3.18146  2.89801
2.37887  2.77519  2.98518  4.58477  3.61503
        0.00414  5.88604  6.60839  0.61958  0.77255  0.31330  1.31317
     19  2.94557  3.79957  2.48911  3.13075  4.91166  3.89890  1.05786
3.47913  3.02845  3.54768  4.72129  3.12281  4.60297  3.00799  3.53826
2.96988  3.62523  3.32291  5.09706  2.91853     24 h - - -
        2.68618  4.42225  2.77519  2.73123  3.46354  2.40513  3.72494
3.29354  2.67741  2.69355  4.24690  2.90347  2.73739  3.18146  2.89801
2.37887  2.77519  2.98518  4.58477  3.61503
        0.00389  5.94709  6.66944  0.61958  0.77255  0.48576  0.95510
     20  3.38141  3.88681  2.86514  2.92300  5.17060  4.16266  3.59006
4.13038  2.91033  3.96688  4.89067  1.86645  4.12932  3.31164  3.58387
1.58886  1.58983  3.47437  6.28933  4.34525     25 s - - -
        2.68618  4.42225  2.77519  2.73123  3.46354  2.40513  3.72494
3.29354  2.67741  2.69355  4.24690  2.90347  2.73739  3.18146  2.89801
2.37887  2.77519  2.98518  4.58477  3.61503
        0.00389  5.94709  6.66944  0.61958  0.77255  0.48576  0.95510
     21  2.63781  5.89213  2.88942  1.52427  4.51955  3.46580  4.33885
4.72527  1.97765  3.93640  4.93247  3.33880  2.64107  2.30419  2.88064
2.46590  3.21873  3.63583  6.32408  4.91238     26 e - - -
        2.68618  4.42225  2.77519  2.73123  3.46354  2.40513  3.72494
3.29354  2.67741  2.69355  4.24690  2.90347  2.73739  3.18146  2.89801
2.37887  2.77519  2.98518  4.58477  3.61503
        0.00389  5.94709  6.66944  0.61958  0.77255  0.48576  0.95510
     22  3.07407  4.86354  2.26364  2.56599  4.62072  3.10474  2.90968
4.72387  2.19035  4.19699  4.39189  2.82877  4.54557  2.97956  1.87729
2.87785  2.09954  3.95893  6.32349  3.36059     27 r - - -
        2.68618  4.42225  2.77519  2.73123  3.46354  2.40513  3.72494
3.29354  2.67741  2.69355  4.24690  2.90347  2.73739  3.18146  2.89801
2.37887  2.77519  2.98518  4.58477  3.61503
        0.00389  5.94709  6.66944  0.61958  0.77255  0.48576  0.95510
     23  1.93396  3.31082  4.99263  4.40297  2.54766  4.23584  2.80756
3.28547  3.42716  2.74791  3.88642  3.05450  4.95454  4.48513  3.84803
3.00108  2.83523  2.60971  4.21705  1.56632     28 y - - -
        2.68618  4.42225  2.77519  2.73123  3.46354  2.40513  3.72494
3.29354  2.67741  2.69355  4.24690  2.90347  2.73739  3.18146  2.89801
2.37887  2.77519  2.98518  4.58477  3.61503
        0.00389  5.94709  6.66944  0.61958  0.77255  0.48576  0.95510
     24  3.16407  4.87257  5.45210  4.83814  3.72614  4.66780  5.00218
0.71023  4.62581  1.96002  3.79400  4.02393  4.13916  4.75932  4.63797
3.54188  3.56575  2.28423  5.49840  4.03141     29 i - - -
        2.68618  4.42225  2.77519  2.73123  3.46354  2.40513  3.72494
3.29354  2.67741  2.69355  4.24690  2.90347  2.73739  3.18146  2.89801
2.37887  2.77519  2.98518  4.58477  3.61503
        0.00389  5.94709  6.66944  0.61958  0.77255  0.48576  0.95510
     25  3.71639  3.81282  5.65580  5.06464  1.70420  4.87203  5.04719
2.63880  4.84719  2.49994  3.80187  5.00638  5.22421  4.94683  4.83955
4.18841  3.94697  3.06312  3.04746  0.89586     30 y - - -
```

```
          2.68618  4.42225  2.77519  2.73123  3.46354  2.40513  3.72494
3.29354  2.67741  2.69355  4.24690  2.90347  2.73739  3.18146  2.89801
2.37887  2.77519  2.98518  4.58477  3.61503
          0.00389  5.94709  6.66944  0.61958  0.77255  0.48576  0.95510
     26   3.72086  3.99644  6.00207  6.00380  6.52051  4.43307  6.61162
6.03695  5.95578  5.73107  6.46324  3.98857  0.17315  6.05166  5.93982
3.42282  3.76514  5.07761  7.85973  6.85207      31 P - - -
          2.68618  4.42225  2.77519  2.73123  3.46354  2.40513  3.72494
3.29354  2.67741  2.69355  4.24690  2.90347  2.73739  3.18146  2.89801
2.37887  2.77519  2.98518  4.58477  3.61503
          0.00389  5.94709  6.66944  0.61958  0.77255  0.48576  0.95510
     27   2.39756  4.49968  3.24133  2.88454  4.31190  4.41535  4.66696
1.69448  2.95766  2.56310  4.27588  3.50592  3.28639  3.99294  4.04798
3.36416  3.34293  1.36991  5.75366  4.51710      32 v - - -
          2.68618  4.42225  2.77519  2.73123  3.46354  2.40513  3.72494
3.29354  2.67741  2.69355  4.24690  2.90347  2.73739  3.18146  2.89801
2.37887  2.77519  2.98518  4.58477  3.61503
          0.00389  5.94709  6.66944  0.61958  0.77255  0.48576  0.95510
     28   4.43878  7.18338  2.42096  3.25301  6.44903  0.43541  4.50584
6.02158  3.64915  5.45475  6.31866  2.46633  5.25609  3.49812  4.94182
3.78743  4.73735  5.54340  7.59579  6.00934      33 G - - -
          2.68618  4.42225  2.77519  2.73123  3.46354  2.40513  3.72494
3.29354  2.67741  2.69355  4.24690  2.90347  2.73739  3.18146  2.89801
2.37887  2.77519  2.98518  4.58477  3.61503
          0.00389  5.94709  6.66944  0.61958  0.77255  0.48576  0.95510
     29   6.23572  6.98478  6.85152  6.92250  0.94963  6.50882  5.00015
5.73847  6.68032  4.76768  6.21268  5.97393  6.74358  6.18914  6.38189
5.94634  6.40898  5.72112  5.06057  0.57335      34 Y - - -
          2.68618  4.42225  2.77519  2.73123  3.46354  2.40513  3.72494
3.29354  2.67741  2.69355  4.24690  2.90347  2.73739  3.18146  2.89801
2.37887  2.77519  2.98518  4.58477  3.61503
          0.00389  5.94709  6.66944  0.61958  0.77255  0.48576  0.95510
     30   3.03940  3.16531  3.70587  2.39253  5.02459  4.18876  3.89765
3.63230  1.66074  3.49195  3.64029  3.41868  4.58052  2.84903  2.46889
2.71114  2.01554  2.59345  5.14828  4.34874      35 k - - -
          2.68618  4.42225  2.77519  2.73123  3.46354  2.40513  3.72494
3.29354  2.67741  2.69355  4.24690  2.90347  2.73739  3.18146  2.89801
2.37887  2.77519  2.98518  4.58477  3.61503
          0.00389  5.94709  6.66944  0.61958  0.77255  0.48576  0.95510
     31   1.73330  1.99711  5.30097  4.69856  3.99794  4.63805  4.97757
2.74551  4.51798  3.09193  3.68380  4.75534  5.00974  4.67915  4.58279
1.35017  2.58036  2.30724  5.52323  3.93091      36 s - - -
          2.68618  4.42225  2.77519  2.73123  3.46354  2.40513  3.72494
3.29354  2.67741  2.69355  4.24690  2.90347  2.73739  3.18146  2.89801
2.37887  2.77519  2.98518  4.58477  3.61503
          0.00389  5.94709  6.66944  0.61958  0.77255  0.48576  0.95510
     32   3.38348  4.45294  3.42234  2.12616  4.39430  4.17091  4.14331
3.04852  2.16776  3.75411  3.78535  3.47862  4.56352  3.15734  2.40980
1.99808  1.70813  2.97868  6.26343  4.87371      37 t - - -
          2.68618  4.42225  2.77519  2.73123  3.46354  2.40513  3.72494
3.29354  2.67741  2.69355  4.24690  2.90347  2.73739  3.18146  2.89801
2.37887  2.77519  2.98518  4.58477  3.61503
          0.00389  5.94709  6.66944  0.61958  0.77255  0.48576  0.95510
     33   4.99318  6.88069  6.01894  4.71597  6.60945  5.42493  4.14194
5.77137  2.25490  5.03511  5.96379  4.92353  5.69238  4.14847  0.30235
4.95785  5.01511  2.82511  6.92410  5.94451      38 R - - -
```

```
         2.68618  4.42225  2.77519  2.73123  3.46354  2.40513  3.72494
3.29354  2.67741  2.69355  4.24690  2.90347  2.73739  3.18146  2.89801
2.37887  2.77519  2.98518  4.58477  3.61503
         0.00389  5.94709  6.66944  0.61958  0.77255  0.48576  0.95510
     34  2.98094  5.02103  4.66776  3.22682  3.20013  4.51681  4.32309
2.14943  2.04615  1.65207  3.83150  3.92042  4.27869  3.88414  2.85488
3.27710  2.73802  2.22125  3.76004  3.75005     39 l - - -
         2.68618  4.42225  2.77519  2.73123  3.46354  2.40513  3.72494
3.29354  2.67741  2.69355  4.24690  2.90347  2.73739  3.18146  2.89801
2.37887  2.77519  2.98518  4.58477  3.61503
         0.00389  5.94709  6.66944  0.61958  0.77255  0.48576  0.95510
     35  4.53328  6.54824  6.63004  6.47447  1.25720  4.87388  4.02689
5.16504  6.24376  3.97832  4.31036  5.80985  6.44561  5.95801  6.06333
5.55663  5.70177  5.06119  3.91922  0.52367     40 Y - - -
         2.68618  4.42225  2.77519  2.73123  3.46354  2.40513  3.72494
3.29354  2.67741  2.69355  4.24690  2.90347  2.73739  3.18146  2.89801
2.37887  2.77519  2.98518  4.58477  3.61503
         0.03156  5.94709  3.55962  0.61958  0.77255  0.48576  0.95510
     36  2.37732  5.16379  4.26322  3.69659  2.94133  3.46918  4.10032
3.08433  2.41307  2.95715  3.59560  3.44860  2.99501  3.78545  3.01781
3.26841  2.85663  3.22565  1.58541  2.92208     41 w - - -
         2.68618  4.42268  2.77484  2.73166  3.46397  2.40459  3.72334
3.29397  2.67701  2.69398  4.24732  2.90337  2.73782  3.18189  2.89752
2.37929  2.77529  2.98505  4.58520  3.61439
         0.11372  2.24256  6.64188  1.31616  0.31220  0.38756  1.13542
     37  3.38373  3.76211  3.27666  3.10955  4.50318  3.44179  2.86416
4.58967  2.93617  4.10471  4.86274  3.33745  3.95579  3.46931  3.02387
0.78322  3.12153  4.18644  6.26600  3.89391     46 s - - -
         2.68618  4.42225  2.77519  2.73123  3.46354  2.40513  3.72494
3.29354  2.67741  2.69355  4.24690  2.90347  2.73739  3.18146  2.89801
2.37887  2.77519  2.98518  4.58477  3.61503
         0.00389  5.94709  6.66944  0.61958  0.77255  0.48576  0.95510
     38  2.65588  3.24852  5.43833  4.82383  2.74103  4.05419  4.98787
2.72615  3.57871  2.29678  2.19858  4.81335  2.40703  4.74495  4.62394
3.36526  1.95085  2.10366  5.48527  2.72762     47 t - - -
         2.68618  4.42225  2.77519  2.73123  3.46354  2.40513  3.72494
3.29354  2.67741  2.69355  4.24690  2.90347  2.73739  3.18146  2.89801
2.37887  2.77519  2.98518  4.58477  3.61503
         0.00389  5.94709  6.66944  0.61958  0.77255  0.48576  0.95510
     39  3.02071  5.03095  3.01884  2.78830  3.29904  4.15883  3.80604
3.67466  1.74560  3.08549  4.90516  3.16376  3.57057  2.71529  2.14789
2.54171  2.60551  2.44568  6.30140  4.41449     48 k - - -
         2.68618  4.42225  2.77519  2.73123  3.46354  2.40513  3.72494
3.29354  2.67741  2.69355  4.24690  2.90347  2.73739  3.18146  2.89801
2.37887  2.77519  2.98518  4.58477  3.61503
         0.01109  5.94709  4.77799  0.61958  0.77255  0.48576  0.95510
     40  3.26353  4.27559  1.44453  2.34196  5.23347  3.90797  3.30150
4.72024  2.11957  3.95402  4.92765  2.11261  4.54086  3.17488  2.85835
2.52370  2.95764  4.11218  6.31929  4.06525     49 d - - -
         2.68618  4.42225  2.77519  2.73123  3.46354  2.40513  3.72494
3.29354  2.67741  2.69355  4.24690  2.90347  2.73739  3.18146  2.89801
2.37887  2.77519  2.98518  4.58477  3.61503
         0.00392  5.93992  6.66227  0.61958  0.77255  0.45535  1.00573
     41  2.55183  4.84626  3.16497  2.88989  5.03557  3.22684  3.70189
3.28949  2.78096  2.81543  4.49299  3.53679  1.26292  3.13645  3.47610
2.68101  2.76880  3.48752  6.21668  3.68387     50 p - - -
```

```
          2.68618  4.42225  2.77519  2.73123  3.46354  2.40513  3.72494
3.29354  2.67741  2.69355  4.24690  2.90347  2.73739  3.18146  2.89801
2.37887  2.77519  2.98518  4.58477  3.61503
          0.00389  5.94709  6.66944  0.61958  0.77255  0.48576  0.95510
     42   3.06202  4.60681  2.66341  2.69821  4.57632  2.66547  2.87990
4.71986  2.11608  3.83363  4.06962  2.35666  3.39013  3.30918  2.22262
2.50612  2.50281  4.09976  3.51238  3.96854     51 k - - -
          2.68624  4.42231  2.77525  2.73129  3.46360  2.40475  3.72500
3.29360  2.67747  2.69361  4.24696  2.90353  2.73745  3.18152  2.89807
2.37869  2.77525  2.98524  4.58483  3.61509
          0.07225  2.81658  4.61623  0.16069  1.90753  0.48576  0.95510
     43   2.92362  5.88629  2.20925  2.09795  5.23248  4.14639  3.84807
4.04034  1.66972  3.94618  4.55729  2.95966  4.53989  2.62999  2.08176
2.79718  2.82296  3.12609  6.31830  4.90668     53 k - - -
          2.68618  4.42225  2.77519  2.73123  3.46354  2.40513  3.72494
3.29354  2.67741  2.69355  4.24690  2.90347  2.73739  3.18146  2.89801
2.37887  2.77519  2.98518  4.58477  3.61503
          0.00392  5.93844  6.66078  0.61958  0.77255  0.44954  1.01589
     44   2.84952  4.58819  3.46169  2.81718  4.60210  4.17278  3.83395
3.03430  1.77085  3.01787  3.98745  3.28984  4.28284  3.13313  1.41710
2.84320  2.82482  3.82641  6.25767  3.51763     54 r - - -
          2.68618  4.42225  2.77519  2.73123  3.46354  2.40513  3.72494
3.29354  2.67741  2.69355  4.24690  2.90347  2.73739  3.18146  2.89801
2.37887  2.77519  2.98518  4.58477  3.61503
          0.00389  5.94709  6.66944  0.61958  0.77255  0.48576  0.95510
     45   2.62123  1.51414  5.42898  4.02029  3.95749  4.65401  4.98647
2.39765  4.60483  2.69649  2.51697  4.80930  5.01792  4.01115  4.62070
3.39409  2.24706  1.62720  4.76898  4.31019     55 c - - -
          2.68618  4.42225  2.77519  2.73123  3.46354  2.40513  3.72494
3.29354  2.67741  2.69355  4.24690  2.90347  2.73739  3.18146  2.89801
2.37887  2.77519  2.98518  4.58477  3.61503
          0.00389  5.94709  6.66944  0.61958  0.77255  0.48576  0.95510
     46   3.44911  4.07100  4.12904  2.81549  2.87873  4.45574  4.28376
2.70279  3.07241  2.00696  3.29357  3.29963  4.44837  3.26911  2.05190
2.97350  2.73140  2.28101  2.99334  3.68650     56 l - - -
          2.68618  4.42225  2.77519  2.73123  3.46354  2.40513  3.72494
3.29354  2.67741  2.69355  4.24690  2.90347  2.73739  3.18146  2.89801
2.37887  2.77519  2.98518  4.58477  3.61503
          0.00389  5.94709  6.66944  0.61958  0.77255  0.48576  0.95510
     47   5.91411  4.84462  6.77112  6.74587  2.59280  6.38286  3.70372
4.65297  6.50617  4.66054  6.01985  5.90847  6.62945  6.09787  6.25692
5.78982  6.10123  4.63373  2.97100  0.23752     57 Y - - -
          2.68618  4.42225  2.77519  2.73123  3.46354  2.40513  3.72494
3.29354  2.67741  2.69355  4.24690  2.90347  2.73739  3.18146  2.89801
2.37887  2.77519  2.98518  4.58477  3.61503
          0.00389  5.94709  6.66944  0.61958  0.77255  0.48576  0.95510
     48   3.27651  5.43931  3.75438  2.46301  3.27067  4.28661  3.14832
2.25076  2.45727  2.55151  3.90927  3.70746  4.67349  3.45046  2.50348
3.26950  1.60155  2.78890  5.97430  4.22744     58 t - - -
          2.68618  4.42225  2.77519  2.73123  3.46354  2.40513  3.72494
3.29354  2.67741  2.69355  4.24690  2.90347  2.73739  3.18146  2.89801
2.37887  2.77519  2.98518  4.58477  3.61503
          0.00389  5.94709  6.66944  0.61958  0.77255  0.48576  0.95510
     49   3.29012  0.89741  5.27734  4.67347  3.57862  4.63420  4.95986
2.65219  4.49467  2.93319  2.71215  3.80728  5.00025  3.78316  4.56351
1.98761  3.71753  2.91747  5.50844  4.32815     59 c - - -
```

```
          2.68618  4.42225  2.77519  2.73123  3.46354  2.40513  3.72494
3.29354  2.67741  2.69355  4.24690  2.90347  2.73739  3.18146  2.89801
2.37887  2.77519  2.98518  4.58477  3.61503
          0.00389  5.94709  6.66944  0.61958  0.77255  0.48576  0.95510
     50   3.38571  3.89642  3.68564  1.51696  4.53388  4.17955  4.37231
3.90846  1.95475  3.83295  4.00573  3.66818  4.57176  3.09385  2.32086
2.26689  2.10023  3.41670  5.08973  4.08894     60 e - - -
          2.68618  4.42225  2.77519  2.73123  3.46354  2.40513  3.72494
3.29354  2.67741  2.69355  4.24690  2.90347  2.73739  3.18146  2.89801
2.37887  2.77519  2.98518  4.58477  3.61503
          0.00389  5.94709  6.66944  0.61958  0.77255  0.48576  0.95510
     51   4.62334  5.77855  6.83718  6.44056  5.39111  6.41035  7.53642
0.56518  6.46474  3.72494  5.04193  6.61790  6.60194  6.81837  6.70915
5.91460  3.04503  1.13957  7.73721  6.43829     61 I - - -
          2.68618  4.42225  2.77519  2.73123  3.46354  2.40513  3.72494
3.29354  2.67741  2.69355  4.24690  2.90347  2.73739  3.18146  2.89801
2.37887  2.77519  2.98518  4.58477  3.61503
          0.00262  5.94582        *  0.61958  0.77255  0.00000        *
//
HMMER3/f [3.3 | Nov 2019]
NAME  GAGA_bind
ACC   PF06217.13
DESC  GAGA binding protein-like family
LENG  295
ALPH  amino
RF    no
MM    no
CONS  yes
CS    no
MAP   yes
DATE  Fri Apr 17 13:50:15 2020
NSEQ  30
EFFN  0.999756
CKSUM 3890535106
GA    26.60 26.60
TC    26.60 26.70
NC    25.00 26.50
STATS LOCAL MSV      -11.2438  0.70141
STATS LOCAL VITERBI  -12.3309  0.70141
STATS LOCAL FORWARD   -4.3198  0.70141
HMM          A        C        D        E        F        G        H
I        K        L        M        N        P        Q        R        S
T        V        W        Y
            m->m     m->i     m->d     i->m     i->i     d->m     d->d
  COMPO   2.54226  3.97234  2.93372  2.66944  3.60681  2.84120  3.60876
3.02907  2.48738  2.62430  3.56273  3.00647  3.20159  2.96276  2.78044
2.60945  2.81454  2.84662  4.23376  3.68182
          2.68618  4.42225  2.77519  2.73123  3.46354  2.40513  3.72494
3.29354  2.67741  2.69355  4.24690  2.90347  2.73739  3.18146  2.89801
2.37887  2.77519  2.98518  4.58477  3.61503
          0.06282  4.18974  3.08487  0.61958  0.77255  0.00000        *
      1   3.21265  4.72201  4.36198  3.98565  3.36282  4.04988  4.65657
2.56785  3.73613  1.92292  0.92937  4.22600  4.51971  4.10384  3.90687
3.58661  3.52753  2.60465  5.23532  4.01783      1 m - - -
          2.68618  4.42225  2.77519  2.73123  3.46354  2.40513  3.72494
3.29354  2.67741  2.69355  4.24690  2.90347  2.73739  3.18146  2.89801
2.37887  2.77519  2.98518  4.58477  3.61503
          0.02368  4.15061  4.87295  0.61958  0.77255  0.45745  1.00211
```

```
      2   3.11699  5.69326  0.89259  2.11009  4.99017  3.30280  3.92374
4.54728  3.01736  4.05283  4.93857  2.61079  3.96703  3.10698  3.64777
2.98573  3.42982  4.10768  6.19445  4.65328      2 d - - -
          2.68618  4.42225  2.77519  2.73123  3.46354  2.40513  3.72494
3.29354  2.67741  2.69355  4.24690  2.90347  2.73739  3.18146  2.89801
2.37887  2.77519  2.98518  4.58477  3.61503
          0.02276  4.18974  4.91209  0.61958  0.77255  0.48576  0.95510
      3   2.98196  5.57103  1.27947  1.90262  4.87445  2.77249  3.82245
4.39575  2.81054  3.87675  4.70189  2.52274  3.91511  2.97704  3.40431
2.67589  3.26301  3.95459  6.03449  4.52906      3 d - - -
          2.68618  4.42225  2.77519  2.73123  3.46354  2.40513  3.72494
3.29354  2.67741  2.69355  4.24690  2.90347  2.73739  3.18146  2.89801
2.37887  2.77519  2.98518  4.58477  3.61503
          0.02276  4.18974  4.91209  0.61958  0.77255  0.48576  0.95510
      4   2.62508  5.01895  1.87745  2.35746  4.35541  2.42476  3.69129
3.58265  2.51073  3.17952  4.15519  2.68847  3.83717  2.82743  3.00335
2.55615  2.93892  3.42242  5.55698  4.17373      4 d - - -
          2.68618  4.42225  2.77519  2.73123  3.46354  2.40513  3.72494
3.29354  2.67741  2.69355  4.24690  2.90347  2.73739  3.18146  2.89801
2.37887  2.77519  2.98518  4.58477  3.61503
          0.02276  4.18974  4.91209  0.61958  0.77255  0.48576  0.95510
      5   2.13849  4.60677  2.95350  2.70905  4.33187  1.36058  3.63372
3.78915  2.89199  3.41702  4.25237  2.82299  3.85902  3.20738  3.29675
2.65997  2.94730  3.35234  5.63222  4.32480      5 g - - -
          2.68618  4.42225  2.77519  2.73123  3.46354  2.40513  3.72494
3.29354  2.67741  2.69355  4.24690  2.90347  2.73739  3.18146  2.89801
2.37887  2.77519  2.98518  4.58477  3.61503
          0.04635  4.18974  3.50172  0.61958  0.77255  0.48576  0.95510
      6   2.67408  4.99475  2.81640  2.38252  4.30921  2.71524  2.69038
3.76021  2.42858  3.31114  4.10577  2.75344  3.83552  2.40200  2.88751
2.11414  2.91912  3.38758  5.49792  4.12999      6 s - - -
          2.68618  4.42225  2.77519  2.73123  3.46354  2.40513  3.72494
3.29354  2.67741  2.69355  4.24690  2.90347  2.73739  3.18146  2.89801
2.37887  2.77519  2.98518  4.58477  3.61503
          0.02330  4.16669  4.88903  0.61958  0.77255  0.51394  0.91163
      7   2.65948  4.43154  3.45924  2.87569  3.51586  3.59911  3.31442
2.92550  2.68341  2.01241  2.84063  3.29147  3.97380  3.09534  2.26013
2.84964  2.88544  2.70667  4.94941  3.51659      7 l - - -
          2.68618  4.42225  2.77519  2.73123  3.46354  2.40513  3.72494
3.29354  2.67741  2.69355  4.24690  2.90347  2.73739  3.18146  2.89801
2.37887  2.77519  2.98518  4.58477  3.61503
          0.02330  4.16669  4.88903  0.61958  0.77255  0.51394  0.91163
      8   2.76021  5.26545  2.32535  1.82579  4.57693  2.63383  3.51483
4.06412  2.50941  3.55866  4.33420  2.37007  3.83737  2.70412  3.03120
2.32483  3.00820  3.64176  5.71134  4.27717      8 e - - -
          2.68628  4.42239  2.77534  2.73137  3.46368  2.40485  3.72509
3.29368  2.67755  2.69369  4.24679  2.90281  2.73754  3.18160  2.89732
2.37894  2.77534  2.98532  4.58469  3.61517
          0.33988  1.27076  4.88903  1.00030  0.45850  0.46854  0.98328
      9   2.63520  4.17896  3.86451  3.28849  2.71321  3.69361  2.61362
2.05014  3.19198  2.24240  2.86058  3.57671  4.06199  3.26299  3.44059
2.96678  2.86639  2.42383  4.70804  3.33829     12 i - - -
          2.68618  4.42225  2.77519  2.73123  3.46354  2.40513  3.72494
3.29354  2.67741  2.69355  4.24690  2.90347  2.73739  3.18146  2.89801
2.37887  2.77519  2.98518  4.58477  3.61503
          0.02276  4.18974  4.91209  0.61958  0.77255  0.48576  0.95510
```

```
     10   3.24011  5.32969  3.72077  2.97473  4.84247  3.82256  3.69702
4.16145  1.38708  3.55961  4.43849  3.34648  4.17196  2.82772  1.20401
3.22628  3.38291  3.84609  5.56041  4.43283      13 r - - -
          2.68567  4.42230  2.77507  2.73155  3.46372  2.40512  3.72445
3.29384  2.67733  2.69382  4.24657  2.90369  2.73728  3.18099  2.89826
2.37903  2.77537  2.98543  4.58513  3.61374
          0.32828  1.36695  3.69073  1.88543  0.16460  0.48576  0.95510
     11   2.57501  4.95610  2.90784  2.39347  4.24945  3.15053  3.62960
3.68736  2.34718  3.13903  3.82129  2.09352  3.37278  2.29744  2.84287
2.64027  2.87456  3.32347  5.44018  4.08027      29 n - - -
          2.68618  4.42225  2.77519  2.73123  3.46354  2.40513  3.72494
3.29354  2.67741  2.69355  4.24690  2.90347  2.73739  3.18146  2.89801
2.37887  2.77519  2.98518  4.58477  3.61503
          0.02318  4.17199  4.89433  0.61958  0.77255  0.47235  0.97693
     12   3.91781  5.16875  4.61085  4.40384  3.06619  4.11587  4.34908
3.96967  4.12769  3.30416  4.58623  4.49298  4.66201  4.49800  4.19578
4.11818  4.23697  3.86801  0.43253  3.05368      30 W - - -
          2.68618  4.42225  2.77519  2.73123  3.46354  2.40513  3.72494
3.29354  2.67741  2.69355  4.24690  2.90347  2.73739  3.18146  2.89801
2.37887  2.77519  2.98518  4.58477  3.61503
          0.18541  4.18974  1.87022  0.61958  0.77255  0.48576  0.95510
     13   2.45026  4.51305  2.82930  2.63948  4.41003  1.56420  3.98127
3.88547  2.90584  3.50204  4.32562  2.33282  3.76117  3.19006  3.31979
2.21245  2.87185  3.37933  5.69076  4.37760      31 g - - -
          2.68618  4.42225  2.77519  2.73123  3.46354  2.40513  3.72494
3.29354  2.67741  2.69355  4.24690  2.90347  2.73739  3.18146  2.89801
2.37887  2.77519  2.98518  4.58477  3.61503
          0.34521  4.03106  1.29399  0.61958  0.77255  0.39300  1.12401
     14   2.46813  4.15777  3.63412  3.06568  3.27568  3.38350  3.85840
2.57608  2.97931  2.07929  2.55787  3.40591  3.95182  3.26989  3.27218
2.74941  2.70353  2.30552  4.76632  3.29976      32 l - - -
          2.68618  4.42225  2.77519  2.73123  3.46354  2.40513  3.72494
3.29354  2.67741  2.69355  4.24690  2.90347  2.73739  3.18146  2.89801
2.37887  2.77519  2.98518  4.58477  3.61503
          0.02953  3.93280  4.65514  0.61958  0.77255  0.35615  1.20520
     15   2.62286  4.09647  3.82349  3.49321  2.35134  3.57362  3.99738
2.37160  3.37295  2.24429  2.34268  3.69789  4.08965  3.59031  3.55293
3.01018  2.85481  2.26742  4.65023  2.66745      33 l - - -
          2.68618  4.42225  2.77519  2.73123  3.46354  2.40513  3.72494
3.29354  2.67741  2.69355  4.24690  2.90347  2.73739  3.18146  2.89801
2.37887  2.77519  2.98518  4.58477  3.61503
          0.12686  4.18974  2.26344  0.61958  0.77255  0.48576  0.95510
     16   2.60401  4.42583  3.10426  2.75707  2.81368  3.52335  3.74567
2.95818  2.73923  2.64468  3.53125  3.19731  3.08210  2.97731  3.12659
2.57156  2.83884  2.71792  4.88627  2.45589      34 y - - -
          2.68618  4.42225  2.77519  2.73123  3.46354  2.40513  3.72494
3.29354  2.67741  2.69355  4.24690  2.90347  2.73739  3.18146  2.89801
2.37887  2.77519  2.98518  4.58477  3.61503
          0.02523  4.08811  4.81046  0.61958  0.77255  0.42035  1.06949
     17   2.76654  5.26832  2.41854  1.70297  4.58220  3.36956  3.46889
4.06730  2.48657  3.55821  4.33266  2.73935  3.41318  1.99353  2.99280
2.70880  3.01141  3.64570  5.70549  4.27822      35 e - - -
          2.68618  4.42225  2.77519  2.73123  3.46354  2.40513  3.72494
3.29354  2.67741  2.69355  4.24690  2.90347  2.73739  3.18146  2.89801
2.37887  2.77519  2.98518  4.58477  3.61503
          0.02276  4.18974  4.91209  0.61958  0.77255  0.48576  0.95510
```

```
     18   2.56771  4.71550  3.05821  2.53968  3.96587  3.44034  2.59130
3.38366  2.52227  3.01252  3.84670  3.03634  2.55862  2.88239  2.95651
2.54751  2.41146  3.07622  5.27100  3.68209     36 t - - -
          2.68612  4.42242  2.77530  2.73120  3.46371  2.40529  3.72477
3.29357  2.67723  2.69358  4.24680  2.90363  2.73735  3.18080  2.89810
2.37894  2.77505  2.98523  4.58494  3.61520
          0.57983  1.33539  1.73189  1.17741  0.36828  0.48576  0.95510
     19   2.48872  4.78243  2.83728  2.35202  3.72856  3.39167  3.46294
3.44457  2.40207  3.04508  3.85713  2.92193  3.31736  2.28143  2.86461
2.51042  2.73182  2.90479  5.28590  3.94861     42 q - - -
          2.68618  4.42225  2.77519  2.73123  3.46354  2.40513  3.72494
3.29354  2.67741  2.69355  4.24690  2.90347  2.73739  3.18146  2.89801
2.37887  2.77519  2.98518  4.58477  3.61503
          0.07763  4.00390  2.87434  0.61958  0.77255  0.38169  1.14794
     20   2.59383  4.48957  3.23992  2.60131  3.31000  3.50621  3.52387
3.03297  2.65086  2.61772  3.31110  2.76880  2.58938  2.98092  3.05910
2.73338  2.74368  2.49700  5.04589  3.77666     43 v - - -
          2.68618  4.42225  2.77519  2.73123  3.46354  2.40513  3.72494
3.29354  2.67741  2.69355  4.24690  2.90347  2.73739  3.18146  2.89801
2.37887  2.77519  2.98518  4.58477  3.61503
          0.02371  4.14942  4.87176  0.61958  0.77255  0.48873  0.95037
     21   2.16377  4.92965  3.00806  2.48389  4.26943  3.44505  3.66010
3.68895  1.77013  3.24827  4.05398  2.99107  3.86230  2.69358  2.64715
2.31880  2.85666  3.33377  5.43872  4.11365     44 k - - -
          2.68618  4.42225  2.77519  2.73123  3.46354  2.40513  3.72494
3.29354  2.67741  2.69355  4.24690  2.90347  2.73739  3.18146  2.89801
2.37887  2.77519  2.98518  4.58477  3.61503
          0.02316  4.17286  4.89521  0.61958  0.77255  0.50653  0.92277
     22   2.60298  4.48884  3.26155  2.70032  3.45956  2.49382  3.74866
3.03377  2.65708  2.45424  2.85540  3.04702  3.90239  2.86723  2.78629
2.60597  2.83533  2.78204  5.04718  3.78115     45 l - - -
          2.68618  4.42225  2.77519  2.73123  3.46354  2.40513  3.72494
3.29354  2.67741  2.69355  4.24690  2.90347  2.73739  3.18146  2.89801
2.37887  2.77519  2.98518  4.58477  3.61503
          0.16854  4.17286  1.96830  0.61958  0.77255  0.50653  0.92277
     23   2.50894  4.65501  3.03413  2.50701  3.89914  3.40691  3.01348
3.14407  2.48380  2.93954  3.77766  2.26032  3.82050  2.84368  2.91594
2.56583  2.64876  2.76745  5.21070  3.90164     46 n - - -
          2.68618  4.42225  2.77519  2.73123  3.46354  2.40513  3.72494
3.29354  2.67741  2.69355  4.24690  2.90347  2.73739  3.18146  2.89801
2.37887  2.77519  2.98518  4.58477  3.61503
          0.02673  4.03106  4.75340  0.61958  0.77255  0.65467  0.73316
     24   3.31015  4.68172  4.72499  4.18075  3.06614  4.48613  4.77376
2.22154  3.98020  0.89705  2.22665  4.46694  4.69926  4.17221  4.13006
3.84599  3.53839  2.35501  5.14041  4.03354     47 l - - -
          2.68618  4.42225  2.77519  2.73123  3.46354  2.40513  3.72494
3.29354  2.67741  2.69355  4.24690  2.90347  2.73739  3.18146  2.89801
2.37887  2.77519  2.98518  4.58477  3.61503
          0.02673  4.03106  4.75340  0.61958  0.77255  0.65467  0.73316
     25   2.56301  4.62418  2.75948  2.62059  4.43601  1.42924  4.00317
3.96442  2.93320  3.58011  4.42839  2.04268  3.81134  3.22419  3.33976
2.64917  2.97882  3.47420  5.69939  4.38227     48 g - - -
          2.68618  4.42225  2.77519  2.73123  3.46354  2.40513  3.72494
3.29354  2.67741  2.69355  4.24690  2.90347  2.73739  3.18146  2.89801
2.37887  2.77519  2.98518  4.58477  3.61503
          0.02673  4.03106  4.75340  0.61958  0.77255  0.39300  1.12401
```

```
     26   2.70498  4.55980  3.38428  2.81254  3.72430  3.59351  3.79038
3.03707  1.99119  1.80426  3.41171  3.11773  3.97926  3.03244  2.87825
2.85576  2.81434  2.80717  5.10575  3.85754     49 l - - -
          2.68618  4.42225  2.77519  2.73123  3.46354  2.40513  3.72494
3.29354  2.67741  2.69355  4.24690  2.90347  2.73739  3.18146  2.89801
2.37887  2.77519  2.98518  4.58477  3.61503
          0.02276  4.18974  4.91209  0.61958  0.77255  0.48576  0.95510
     27   2.96042  5.23488  2.95021  2.55951  4.60441  3.54828  3.70939
4.03281  1.89574  3.51338  4.35016  2.87808  3.99436  1.40409  2.50506
2.93232  3.17701  3.67419  5.60589  4.31654     50 q - - -
          2.68618  4.42225  2.77519  2.73123  3.46354  2.40513  3.72494
3.29354  2.67741  2.69355  4.24690  2.90347  2.73739  3.18146  2.89801
2.37887  2.77519  2.98518  4.58477  3.61503
          0.02276  4.18974  4.91209  0.61958  0.77255  0.48576  0.95510
     28   3.19668  4.51857  4.88408  4.33302  3.35900  4.50075  4.90699
1.59328  4.20655  1.14770  3.16285  4.54899  4.75258  4.37625  4.34896
3.85033  3.21571  1.76811  5.32915  4.21239     51 l - - -
          2.68618  4.42225  2.77519  2.73123  3.46354  2.40513  3.72494
3.29354  2.67741  2.69355  4.24690  2.90347  2.73739  3.18146  2.89801
2.37887  2.77519  2.98518  4.58477  3.61503
          0.02276  4.18974  4.91209  0.61958  0.77255  0.48576  0.95510
     29   2.88723  4.43530  4.06197  3.48205  3.29325  3.93497  4.20751
2.41565  3.24279  1.91892  1.49311  3.80084  4.28130  3.60322  3.09554
3.23298  3.12205  2.38430  5.00079  3.81498     52 m - - -
          2.68618  4.42225  2.77519  2.73123  3.46354  2.40513  3.72494
3.29354  2.67741  2.69355  4.24690  2.90347  2.73739  3.18146  2.89801
2.37887  2.77519  2.98518  4.58477  3.61503
          0.02276  4.18974  4.91209  0.61958  0.77255  0.48576  0.95510
     30   2.18845  4.58907  3.16346  2.65393  4.01430  3.35569  3.79687
3.41207  2.55977  3.05635  3.58969  3.01740  3.42509  2.98704  3.06027
1.64498  2.74466  3.07558  5.32885  4.03612     53 s - - -
          2.68618  4.42225  2.77520  2.73124  3.46354  2.40513  3.72495
3.29354  2.67741  2.69351  4.24690  2.90347  2.73735  3.18147  2.89801
2.37887  2.77520  2.98519  4.58477  3.61503
          0.05663  3.04291  4.91209  0.48217  0.96087  0.48576  0.95510
     31   2.60098  4.28684  3.62415  3.07181  3.48687  3.57527  3.94957
2.06057  3.00483  2.37675  3.44907  3.42914  3.69723  3.30794  3.32930
1.97554  2.66170  2.40011  4.96342  3.73851     55 s - - -
          2.68618  4.42225  2.77519  2.73123  3.46354  2.40513  3.72494
3.29354  2.67741  2.69355  4.24690  2.90347  2.73739  3.18146  2.89801
2.37887  2.77519  2.98518  4.58477  3.61503
          0.02276  4.18974  4.91209  0.61958  0.77255  0.48576  0.95510
     32   3.12752  4.45798  4.85580  4.28156  3.01961  4.39510  4.74107
1.88706  4.15443  1.56350  1.95389  4.45831  4.64666  4.27388  4.25515
3.72694  3.35585  1.59468  5.14278  4.03384     56 l - - -
          2.68618  4.42225  2.77519  2.73123  3.46354  2.40513  3.72494
3.29354  2.67741  2.69355  4.24690  2.90347  2.73739  3.18146  2.89801
2.37887  2.77519  2.98518  4.58477  3.61503
          0.04635  4.18974  3.50172  0.61958  0.77255  0.48576  0.95510
     33   1.60603  4.51380  3.18871  2.74494  4.06423  3.28451  3.89064
3.23549  2.75776  3.11560  3.96048  2.69311  2.41541  3.09810  3.16213
2.62564  2.84790  3.08159  5.39509  4.11164     57 a - - -
          2.68602  4.42228  2.77523  2.73127  3.46357  2.40503  3.72498
3.29357  2.67744  2.69358  4.24693  2.90350  2.73726  3.18150  2.89804
2.37890  2.77523  2.98522  4.58480  3.61506
          0.21090  1.70037  4.88903  0.35215  1.21461  0.46854  0.98328
```

```
     34   2.97677  5.56643  1.66600  1.30658  4.83958  3.32740  3.27883
4.37215  2.76753  3.85035  4.67249  2.77946  3.91481  2.95938  3.34240
2.86819  3.25029  3.93668  5.99442  4.49611      60 e - - -
          2.68618  4.42225  2.77519  2.73123  3.46354  2.40513  3.72494
3.29354  2.67741  2.69355  4.24690  2.90347  2.73739  3.18146  2.89801
2.37887  2.77519  2.98518  4.58477  3.61503
          0.09607  4.18974  2.57108  0.61958  0.77255  0.48576  0.95510
     35   3.30388  5.11173  3.82161  3.28987  4.52983  3.73531  4.03265
4.09483  2.37725  3.56623  4.56600  3.62759  4.23139  3.24603  0.66043
3.37997  3.55655  3.80960  5.51635  4.41043      61 R - - -
          2.68618  4.42225  2.77519  2.73123  3.46354  2.40513  3.72494
3.29354  2.67741  2.69355  4.24690  2.90347  2.73739  3.18146  2.89801
2.37887  2.77519  2.98518  4.58477  3.61503
          0.02448  4.11814  4.84049  0.61958  0.77255  0.56886  0.83510
     36   3.03737  5.62868  1.04528  2.06345  4.90297  3.27228  3.83997
4.44199  2.88344  3.93320  4.79572  2.55127  3.91097  2.84776  3.49054
2.91046  3.33264  4.00967  6.09059  4.56368      62 d - - -
          2.68618  4.42225  2.77519  2.73123  3.46354  2.40513  3.72494
3.29354  2.67741  2.69355  4.24690  2.90347  2.73739  3.18146  2.89801
2.37887  2.77519  2.98518  4.58477  3.61503
          0.05237  4.11814  3.35953  0.61958  0.77255  0.56886  0.83510
     37   2.00770  4.65036  2.82614  2.54403  4.07314  3.31741  3.76896
3.46535  2.53465  3.10977  3.94553  2.78963  3.82430  2.95401  3.05469
2.64306  1.91028  3.12394  5.37456  4.06513      63 t - - -
          2.68618  4.42225  2.77519  2.73123  3.46354  2.40513  3.72494
3.29354  2.67741  2.69355  4.24690  2.90347  2.73739  3.18146  2.89801
2.37887  2.77519  2.98518  4.58477  3.61503
          0.34175  4.09093  1.29919  0.61958  0.77255  0.46553  0.98833
     38   2.98080  4.94527  3.15746  2.77691  4.33393  3.46937  3.76424
3.76377  1.02500  3.32665  4.28744  3.20100  3.96890  2.95506  2.43300
3.02206  3.23327  3.47096  5.38446  4.19858      64 k - - -
          2.68618  4.42225  2.77519  2.73123  3.46354  2.40513  3.72494
3.29354  2.67741  2.69355  4.24690  2.90347  2.73739  3.18146  2.89801
2.37887  2.77519  2.98518  4.58477  3.61503
          0.03109  3.88223  4.60458  0.61958  0.77255  0.77243  0.61969
     39   2.63381  4.76832  2.77178  2.42519  4.06627  3.31120  3.36968
3.54361  2.41152  3.13686  3.98347  2.92180  2.23455  2.49995  2.79535
2.55842  2.90285  3.20952  5.32800  3.99184      65 p - - -
          2.68628  4.42236  2.77519  2.73134  3.46342  2.40523  3.72387
3.29345  2.67751  2.69365  4.24700  2.90331  2.73739  3.18088  2.89798
2.37897  2.77530  2.98529  4.58487  3.61514
          0.08709  2.61178  4.60458  1.79082  0.18251  0.34090  1.24177
     40   1.89741  4.24423  4.01221  3.44445  2.92906  3.80481  4.13600
2.25554  3.35222  1.74943  3.25814  3.72813  4.17686  3.60442  3.59728
3.09448  2.72975  2.25229  4.89967  3.70095      81 l - - -
          2.68618  4.42225  2.77519  2.73123  3.46354  2.40513  3.72494
3.29354  2.67741  2.69355  4.24690  2.90347  2.73739  3.18146  2.89801
2.37887  2.77519  2.98518  4.58477  3.61503
          0.02276  4.18974  4.91209  0.61958  0.77255  0.48576  0.95510
     41   2.83066  4.28333  4.25794  3.68326  3.20385  3.95750  3.53712
1.78204  3.53426  1.44080  3.16417  3.91839  4.29517  3.77550  3.72205
3.25737  3.06304  2.08856  4.87837  3.67552      82 l - - -
          2.68618  4.42225  2.77519  2.73123  3.46354  2.40513  3.72494
3.29354  2.67741  2.69355  4.24690  2.90347  2.73739  3.18146  2.89801
2.37887  2.77519  2.98518  4.58477  3.61503
          0.02276  4.18974  4.91209  0.61958  0.77255  0.48576  0.95510
```

```
     42   2.54823  4.99210  2.91986  2.32333  4.28717  3.20315  3.27886
3.73248  2.35715  3.17180  4.05277  2.91386  2.84499  2.26067  2.62339
2.39598  2.86593  3.35702  5.45667  4.08830     83 q - - -
          2.68618  4.42225  2.77519  2.73123  3.46354  2.40513  3.72494
3.29354  2.67741  2.69355  4.24690  2.90347  2.73739  3.18146  2.89801
2.37887  2.77519  2.98518  4.58477  3.61503
          0.02276  4.18974  4.91209  0.61958  0.77255  0.48576  0.95510
     43   2.53268  5.10310  2.76538  1.78294  4.42178  2.86152  3.63517
3.88657  2.32794  3.40241  4.17267  2.55755  3.40133  2.75235  2.79392
2.51229  2.91392  3.48402  5.56592  4.16987     84 e - - -
          2.68618  4.42225  2.77519  2.73123  3.46354  2.40513  3.72494
3.29354  2.67741  2.69355  4.24690  2.90347  2.73739  3.18146  2.89801
2.37887  2.77519  2.98518  4.58477  3.61503
          0.02276  4.18974  4.91209  0.61958  0.77255  0.48576  0.95510
     44   2.56533  4.94516  2.76078  2.41075  4.24618  2.95466  3.63285
3.52532  2.30737  3.23912  4.02914  2.93760  3.82653  2.76125  1.95722
2.51170  2.71324  3.31789  5.43543  4.07999     85 r - - -
          2.68618  4.42225  2.77519  2.73123  3.46354  2.40513  3.72494
3.29354  2.67741  2.69355  4.24690  2.90347  2.73739  3.18146  2.89801
2.37887  2.77519  2.98518  4.58477  3.61503
          0.02276  4.18974  4.91209  0.61958  0.77255  0.48576  0.95510
     45   2.59572  5.12130  2.49936  2.20369  4.43676  3.08823  3.27709
3.90755  2.40890  3.41729  4.18219  2.16097  3.14864  2.74367  2.90721
2.48459  2.75211  3.49846  5.57687  4.17236     86 n - - -
          2.68618  4.42225  2.77519  2.73123  3.46354  2.40513  3.72494
3.29354  2.67741  2.69355  4.24690  2.90347  2.73739  3.18146  2.89801
2.37887  2.77519  2.98518  4.58477  3.61503
          0.18056  4.18974  1.89682  0.61958  0.77255  0.48576  0.95510
     46   2.51344  4.40989  3.27179  2.70798  3.56916  3.49469  3.50912
2.87919  2.66382  2.25565  3.08757  3.02911  3.31972  2.91217  2.93815
2.53924  2.79702  2.59688  4.97385  3.71641     87 l - - -
          2.68618  4.42225  2.77519  2.73123  3.46354  2.40513  3.72494
3.29354  2.67741  2.69355  4.24690  2.90347  2.73739  3.18146  2.89801
2.37887  2.77519  2.98518  4.58477  3.61503
          0.33921  4.03579  1.30935  0.61958  0.77255  0.39508  1.11972
     47   2.47273  4.23662  3.44687  2.80944  2.45044  3.50882  3.79819
2.60103  2.84177  2.45300  3.35778  3.10676  3.57148  3.14709  3.18036
2.77142  2.59398  2.43934  4.83717  3.60129     88 v - - -
          2.68618  4.42225  2.77519  2.73123  3.46354  2.40513  3.72494
3.29354  2.67741  2.69355  4.24690  2.90347  2.73739  3.18146  2.89801
2.37887  2.77519  2.98518  4.58477  3.61503
          0.02944  3.93599  4.65834  0.61958  0.77255  0.73345  0.65441
     48   2.77020  4.31699  3.92202  3.36180  3.25952  3.83676  4.14076
2.16226  3.22444  1.50123  3.15508  3.69621  4.18859  3.22069  3.49109
3.12899  2.88170  2.01778  4.95401  3.76638     89 l - - -
          2.68619  4.42256  2.77551  2.73154  3.46385  2.40532  3.71871
3.29385  2.67772  2.69377  4.24721  2.90347  2.73733  3.18037  2.89832
2.37918  2.77545  2.98529  4.58508  3.61521
          0.41983  1.46968  2.18179  2.01312  0.14338  0.35718  1.20278
     49   1.85494  4.49870  3.26629  2.74669  3.66509  3.47127  2.53761
3.07730  2.65802  2.75214  3.05529  3.18434  3.90616  3.03706  3.02881
2.74898  2.86565  2.82388  5.07338  3.77775    105 a - - -
          2.68618  4.42225  2.77519  2.73123  3.46354  2.40513  3.72494
3.29354  2.67741  2.69355  4.24690  2.90347  2.73739  3.18146  2.89801
2.37887  2.77519  2.98518  4.58477  3.61503
          0.08054  4.10615  2.79837  0.61958  0.77255  0.47496  0.97262
```

```
     50   2.45479  4.22469  3.65271  3.07985  3.32603  3.62537  3.64726
2.24981  2.99899  2.04160  3.01072  3.43491  3.99822  2.71332  3.30162
2.88572  2.83373  2.29226  4.81916  3.60468    106 l - - -
          2.68618  4.42225  2.77519  2.73123  3.46354  2.40513  3.72494
3.29354  2.67741  2.69355  4.24690  2.90347  2.73739  3.18146  2.89801
2.37887  2.77519  2.98518  4.58477  3.61503
          0.02460  4.11322  4.83557  0.61958  0.77255  0.57412  0.82828
     51   2.34226  4.52061  3.19018  2.63906  3.75440  3.25282  3.50561
3.00670  2.61483  2.63609  3.66366  3.10501  3.28545  2.95075  3.03292
2.04704  2.73954  2.74109  5.11954  3.84095    107 s - - -
          2.68618  4.42225  2.77519  2.73123  3.46354  2.40513  3.72494
3.29354  2.67741  2.69355  4.24690  2.90347  2.73739  3.18146  2.89801
2.37887  2.77519  2.98518  4.58477  3.61503
          0.02460  4.11322  4.83557  0.61958  0.77255  0.51078  0.91636
     52   2.61375  5.02172  2.75137  1.78189  4.33031  3.25204  2.61030
3.77763  2.44793  3.32830  4.12362  2.89178  3.83127  2.79567  2.91955
2.40971  2.71647  3.40423  5.51971  4.14478    108 e - - -
          2.68618  4.42225  2.77519  2.73123  3.46354  2.40513  3.72494
3.29354  2.67741  2.69355  4.24690  2.90347  2.73739  3.18146  2.89801
2.37887  2.77519  2.98518  4.58477  3.61503
          0.06403  4.14727  3.07441  0.61958  0.77255  0.53660  0.87884
     53   2.46398  4.87790  2.97833  2.42800  4.16111  3.42191  3.61901
3.58301  1.83991  3.15774  3.54927  2.66160  3.31847  2.75594  2.72906
2.64015  2.69725  3.23857  5.36857  4.03053    109 k - - -
          2.68618  4.42225  2.77519  2.73123  3.46354  2.40513  3.72494
3.29354  2.67741  2.69355  4.24690  2.90347  2.73739  3.18146  2.89801
2.37887  2.77519  2.98518  4.58477  3.61503
          0.02473  4.10797  4.83031  0.61958  0.77255  0.52999  0.88821
     54   2.65504  5.07805  2.75109  2.33152  4.39603  2.97103  3.42416
3.85614  1.82879  3.36728  4.13586  2.62248  3.46053  2.48986  2.80422
2.62805  2.76669  3.45621  5.52397  4.13872    110 k - - -
          2.68618  4.42225  2.77519  2.73123  3.46354  2.40513  3.72494
3.29354  2.67741  2.69355  4.24690  2.90347  2.73739  3.18146  2.89801
2.37887  2.77519  2.98518  4.58477  3.61503
          0.02407  4.13473  4.85707  0.61958  0.77255  0.55073  0.85926
     55   2.12905  4.94408  2.83552  2.14340  4.23615  2.87042  3.05272
3.67488  2.42530  3.23912  4.03395  2.90413  3.59209  2.68386  2.89963
2.41559  2.87718  3.31325  5.44485  4.08042    111 a - - -
          2.68618  4.42225  2.77519  2.73123  3.46354  2.40513  3.72494
3.29354  2.67741  2.69355  4.24690  2.90347  2.73739  3.18146  2.89801
2.37887  2.77519  2.98518  4.58477  3.61503
          0.04904  4.13473  3.44670  0.61958  0.77255  0.55073  0.85926
     56   1.53400  4.55612  3.05177  2.64093  4.09977  3.10172  3.52429
3.51569  2.69949  3.15007  3.98092  2.87295  3.24114  3.03042  3.12152
2.35109  2.83963  3.14332  5.40743  4.11173    112 a - - -
          2.68618  4.42225  2.77519  2.73123  3.46354  2.40513  3.72494
3.29354  2.67741  2.69355  4.24690  2.90347  2.73739  3.18146  2.89801
2.37887  2.77519  2.98518  4.58477  3.61503
          0.02467  4.11035  4.83270  0.61958  0.77255  0.53176  0.88569
     57   2.61045  4.13393  3.89763  3.32073  2.60949  3.67852  3.31484
2.40045  3.22105  2.00742  3.23125  3.58511  4.04765  3.13805  3.45216
2.95521  2.84193  2.16464  4.64820  2.82236    113 l - - -
          2.68618  4.42225  2.77519  2.73123  3.46354  2.40513  3.72494
3.29354  2.67741  2.69355  4.24690  2.90347  2.73739  3.18146  2.89801
2.37887  2.77519  2.98518  4.58477  3.61503
          0.02407  4.13473  4.85707  0.61958  0.77255  0.44718  1.02007
```

```
     58   2.01621  4.73533  3.04676  2.49044  3.95254  3.46352  2.34294
3.36675  2.50297  2.99609  3.83407  3.03392  3.57434  2.79290  2.93026
2.70428  2.87784  2.99441  5.25735  3.73298    114 a - - -
          2.68618  4.42225  2.77519  2.73123  3.46354  2.40513  3.72494
3.29354  2.67741  2.69355  4.24690  2.90347  2.73739  3.18146  2.89801
2.37887  2.77519  2.98518  4.58477  3.61503
          0.02276  4.18974  4.91209  0.61958  0.77255  0.48576  0.95510
     59   2.43238  4.94433  2.89362  1.96467  4.22022  3.41565  3.09914
3.65751  2.41589  3.22363  4.01737  2.92428  2.69375  2.77050  2.88716
2.55974  2.76114  3.30067  5.43019  3.79621    115 e - - -
          2.68618  4.42225  2.77519  2.73123  3.46354  2.40513  3.72494
3.29354  2.67741  2.69355  4.24690  2.90347  2.73739  3.18146  2.89801
2.37887  2.77519  2.98518  4.58477  3.61503
          0.02276  4.18974  4.91209  0.61958  0.77255  0.48576  0.95510
     60   3.16911  5.19441  3.58610  2.96746  4.37699  3.76081  3.11163
4.01787  2.05983  3.46744  4.36930  3.35105  4.15727  2.92206  0.97238
3.18555  3.35322  3.71481  5.41029  4.15214    116 r - - -
          2.68618  4.42225  2.77519  2.73123  3.46354  2.40513  3.72494
3.29354  2.67741  2.69355  4.24690  2.90347  2.73739  3.18146  2.89801
2.37887  2.77519  2.98518  4.58477  3.61503
          0.02276  4.18974  4.91209  0.61958  0.77255  0.48576  0.95510
     61   2.90818  5.42611  1.26789  2.13794  4.73472  2.93413  3.77999
4.23225  2.69375  3.73071  4.54278  2.63442  3.89849  2.92682  2.87022
2.82452  3.17661  3.81119  5.89177  4.42979    117 d - - -
          2.68589  4.42079  2.77582  2.73129  3.46429  2.40438  3.72466
3.29347  2.67816  2.69415  4.24508  2.90356  2.73591  3.18181  2.89876
2.37868  2.77548  2.98512  4.58552  3.61546
          0.39692  1.13863  4.91209  2.39039  0.09606  0.48576  0.95510
     62   2.56131  4.28112  3.60088  2.73669  3.23424  3.42750  3.90364
2.56608  2.97390  2.44066  1.89761  3.41580  4.00827  3.27515  3.29387
2.88785  2.77573  2.49186  4.88594  3.66342    143 m - - -
          2.68618  4.42225  2.77519  2.73123  3.46354  2.40513  3.72494
3.29354  2.67741  2.69355  4.24690  2.90347  2.73739  3.18146  2.89801
2.37887  2.77519  2.98518  4.58477  3.61503
          0.04275  4.18974  3.62315  0.61958  0.77255  0.48576  0.95510
     63   1.85145  5.27773  2.02925  1.94318  4.59301  3.33986  3.73125
4.07124  2.61098  3.58955  4.38827  2.40485  3.86338  2.87050  3.14317
2.75312  3.07328  3.66242  5.76205  4.32605    144 a - - -
          2.68618  4.42225  2.77519  2.73123  3.46354  2.40513  3.72494
3.29354  2.67741  2.69355  4.24690  2.90347  2.73739  3.18146  2.89801
2.37887  2.77519  2.98518  4.58477  3.61503
          0.02322  4.17021  4.89256  0.61958  0.77255  0.50973  0.91794
     64   3.15150  4.50712  4.58589  4.08877  1.44872  4.19160  3.87268
2.30155  3.93834  2.02454  3.39201  4.08617  4.50496  4.02554  4.02467
3.51187  3.37619  2.65867  4.13123  1.90079    145 f - - -
          2.68618  4.42225  2.77519  2.73123  3.46354  2.40513  3.72494
3.29354  2.67741  2.69355  4.24690  2.90347  2.73739  3.18146  2.89801
2.37887  2.77519  2.98518  4.58477  3.61503
          0.04729  4.17021  3.48219  0.61958  0.77255  0.50973  0.91794
     65   2.52569  4.28825  3.51297  2.94598  3.42491  3.57818  3.84292
2.76330  2.88469  2.15465  2.88130  3.33812  3.63829  2.73851  3.22397
2.65741  2.72819  2.15612  4.88072  3.65191    146 l - - -
          2.68618  4.42225  2.77519  2.73123  3.46354  2.40513  3.72494
3.29354  2.67741  2.69355  4.24690  2.90347  2.73739  3.18146  2.89801
2.37887  2.77519  2.98518  4.58477  3.61503
          0.02378  4.14670  4.86904  0.61958  0.77255  0.53725  0.87792
```

```
     66   2.99064  5.20717  3.31300  2.69962  4.60249  3.65197  3.37704
3.99007  1.96180  3.44291  4.28051  3.14935  4.02264  1.79976  1.59684
2.97435  3.17061  3.64973  5.50751  4.28085    147 r - - -
          2.68618  4.42225  2.77519  2.73123  3.46354  2.40513  3.72494
3.29354  2.67741  2.69355  4.24690  2.90347  2.73739  3.18146  2.89801
2.37887  2.77519  2.98518  4.58477  3.61503
          0.15987  4.14670  2.02548  0.61958  0.77255  0.53725  0.87792
     67   2.71784  4.90494  2.96629  2.52659  4.34431  3.19939  3.66971
3.77317  2.25508  3.31509  4.14197  2.32441  3.86555  2.81861  1.70848
2.62695  2.97678  3.40555  5.46137  4.16590    148 r - - -
          2.68618  4.42225  2.77519  2.73123  3.46354  2.40513  3.72494
3.29354  2.67741  2.69355  4.24690  2.90347  2.73739  3.18146  2.89801
2.37887  2.77519  2.98518  4.58477  3.61503
          0.02720  4.01402  4.73637  0.61958  0.77255  0.45262  1.01049
     68   3.08495  5.69877  1.12257  1.62541  4.98083  3.29108  3.87530
4.51974  2.94022  4.00823  4.87879  2.75078  3.93677  3.04850  3.56579
2.94901  3.38289  4.08071  6.15167  4.62186    149 d - - -
          2.68618  4.42225  2.77519  2.73123  3.46354  2.40513  3.72494
3.29354  2.67741  2.69355  4.24690  2.90347  2.73739  3.18146  2.89801
2.37887  2.77519  2.98518  4.58477  3.61503
          0.02378  4.14670  4.86904  0.61958  0.77255  0.49184  0.94546
     69   2.06713  4.70291  3.06585  2.47349  3.95768  3.43761  3.68861
3.35793  2.36490  2.98970  3.45910  2.89110  3.84748  2.86276  2.94911
2.25689  2.47751  3.05216  5.25978  3.94923    150 a - - -
          2.68618  4.42225  2.77519  2.73123  3.46354  2.40513  3.72494
3.29354  2.67741  2.69355  4.24690  2.90347  2.73739  3.18146  2.89801
2.37887  2.77519  2.98518  4.58477  3.61503
          0.02322  4.17021  4.89256  0.61958  0.77255  0.50973  0.91794
     70   1.62305  4.39055  3.80136  3.34731  3.27715  3.52898  4.03323
3.01817  3.18650  2.68800  3.69184  3.58960  4.06871  3.54232  3.44146
2.92410  3.02051  2.79320  1.81943  3.40074    151 a - - -
          2.68618  4.42225  2.77519  2.73123  3.46354  2.40513  3.72494
3.29354  2.67741  2.69355  4.24690  2.90347  2.73739  3.18146  2.89801
2.37887  2.77519  2.98518  4.58477  3.61503
          0.02322  4.17021  4.89256  0.61958  0.77255  0.50973  0.91794
     71   3.21503  4.52632  4.96837  4.39340  2.97604  4.50118  4.83892
1.50521  4.26603  1.36834  2.18641  4.57438  4.71818  4.34936  4.34661
3.84056  3.43937  1.99648  5.17312  4.09260    152 l - - -
          2.68618  4.42225  2.77519  2.73123  3.46354  2.40513  3.72494
3.29354  2.67741  2.69355  4.24690  2.90347  2.73739  3.18146  2.89801
2.37887  2.77519  2.98518  4.58477  3.61503
          0.02322  4.17021  4.89256  0.61958  0.77255  0.50973  0.91794
     72   1.99126  4.76865  2.97394  2.49692  4.11538  3.24333  2.65170
3.54031  2.51990  3.14580  3.96318  2.45799  3.83624  2.87187  2.96802
2.47891  2.87025  3.06914  5.38190  4.05163    153 a - - -
          2.68618  4.42225  2.77519  2.73123  3.46354  2.40513  3.72494
3.29354  2.67741  2.69355  4.24690  2.90347  2.73739  3.18146  2.89801
2.37887  2.77519  2.98518  4.58477  3.61503
          0.04729  4.17021  3.48219  0.61958  0.77255  0.50973  0.91794
     73   2.78488  5.19398  2.61577  1.68663  4.51404  3.37889  3.69158
3.97660  2.46370  3.49191  4.29321  2.86637  2.66867  2.13347  2.92173
2.74248  3.03206  3.58444  5.65346  4.26036    154 e - - -
          2.68620  4.42227  2.77510  2.73125  3.46356  2.40515  3.72496
3.29356  2.67732  2.69357  4.24692  2.90349  2.73742  3.18148  2.89803
2.37889  2.77522  2.98520  4.58479  3.61479
          0.06617  2.87625  4.86904  0.79640  0.59957  0.53725  0.87792
```

```
     74   2.68151  4.95056  3.18370  2.65246  4.37429  3.50973  3.70008
3.77324  2.21223  3.31256  4.14850  3.10985  3.94449  2.63075  1.47481
2.33996  3.04038  3.42683  5.46332  4.20034    158 r - - -
          2.68618  4.42225  2.77519  2.73123  3.46354  2.40513  3.72494
3.29354  2.67741  2.69355  4.24690  2.90347  2.73739  3.18146  2.89801
2.37887  2.77519  2.98518  4.58477  3.61503
          0.02378  4.14670  4.86904  0.61958  0.77255  0.49184  0.94546
     75   2.79154  5.32417  1.96259  2.06477  4.63590  3.35039  3.07075
4.13179  2.52507  3.61411  4.39066  2.16065  3.84760  2.29189  3.04744
2.72283  3.04085  3.70107  5.75722  4.31342    159 d - - -
          2.68649  4.42263  2.77551  2.73127  3.46392  2.40551  3.71969
3.29392  2.67773  2.69386  4.24728  2.90367  2.73722  3.17893  2.89798
2.37911  2.77550  2.98557  4.58515  3.61541
          0.62164  1.55602  1.37848  2.09747  0.13098  0.50973  0.91794
     76   2.98774  4.95258  3.16491  2.78288  4.34299  3.47552  3.76840
3.77330  1.01471  3.33449  4.29484  3.20665  3.97449  2.95876  2.43435
3.02848  3.23962  3.48005  5.39035  4.20555    189 k - - -
          2.68618  4.42225  2.77519  2.73123  3.46354  2.40513  3.72494
3.29354  2.67741  2.69355  4.24690  2.90347  2.73739  3.18146  2.89801
2.37887  2.77519  2.98518  4.58477  3.61503
          0.03085  3.88989  4.61223  0.61958  0.77255  0.76710  0.62429
     77   2.50036  4.13985  3.74825  3.17958  2.74237  3.62271  3.49133
2.46450  3.08942  2.20639  3.21997  3.47944  3.99232  3.35220  3.35278
2.89493  2.81180  2.01925  4.58635  2.86444    190 v - - -
          2.68618  4.42225  2.77519  2.73123  3.46354  2.40513  3.72494
3.29354  2.67741  2.69355  4.24690  2.90347  2.73739  3.18146  2.89801
2.37887  2.77519  2.98518  4.58477  3.61503
          0.03085  3.88989  4.61223  0.61958  0.77255  0.76710  0.62429
     78   3.18916  4.57552  4.55314  4.01089  2.48504  4.34380  4.48848
2.20373  3.87251  1.01333  2.73661  4.27512  4.57440  4.03238  4.03990
3.67899  3.41506  2.30807  4.85228  3.56273    191 l - - -
          2.68618  4.42225  2.77519  2.73123  3.46354  2.40513  3.72494
3.29354  2.67741  2.69355  4.24690  2.90347  2.73739  3.18146  2.89801
2.37887  2.77519  2.98518  4.58477  3.61503
          0.03085  3.88989  4.61223  0.61958  0.77255  0.76710  0.62429
     79   2.75013  5.02038  2.55244  2.18575  4.18545  3.31602  2.35304
3.75986  2.40556  3.31232  4.15553  2.16556  3.81884  2.81001  2.81398
2.73001  3.00104  3.41237  5.42299  4.02579    192 n - - -
          2.68618  4.42225  2.77519  2.73123  3.46354  2.40513  3.72494
3.29354  2.67741  2.69355  4.24690  2.90347  2.73739  3.18146  2.89801
2.37887  2.77519  2.98518  4.58477  3.61503
          0.03085  3.88989  4.61223  0.61958  0.77255  0.76710  0.62429
     80   2.33977  4.27239  3.36929  2.80876  3.43312  3.47796  3.51108
2.65769  2.75141  2.50027  2.93464  3.08947  3.87251  3.07016  3.10894
2.56089  2.68146  2.34782  4.87043  3.63111    193 a - - -
          2.68618  4.42225  2.77519  2.73123  3.46354  2.40513  3.72494
3.29354  2.67741  2.69355  4.24690  2.90347  2.73739  3.18146  2.89801
2.37887  2.77519  2.98518  4.58477  3.61503
          0.03085  3.88989  4.61223  0.61958  0.77255  0.76710  0.62429
     81   2.74799  4.28730  3.91760  3.34631  2.93802  3.81939  4.08249
2.20348  3.22353  1.54141  3.10294  3.67341  4.15972  3.50679  3.06038
3.10192  2.97987  2.13543  4.84847  3.63323    194 l - - -
          2.68618  4.42225  2.77519  2.73123  3.46354  2.40513  3.72494
3.29354  2.67741  2.69355  4.24690  2.90347  2.73739  3.18146  2.89801
2.37887  2.77519  2.98518  4.58477  3.61503
          0.03085  3.88989  4.61223  0.61958  0.77255  0.76710  0.62429
```

```
     82   2.16578  4.23207  3.18862  2.84914  4.13046  2.99442  3.97859
3.50685  2.88778  3.19714  4.03793  3.14050  2.01476  3.21533  3.24339
2.04065  2.40033  3.05282  5.46354  4.21628    195 p - - -
          2.68618  4.42225  2.77519  2.73123  3.46354  2.40513  3.72494
3.29354  2.67741  2.69355  4.24690  2.90347  2.73739  3.18146  2.89801
2.37887  2.77519  2.98518  4.58477  3.61503
          0.03085  3.88989  4.61223  0.61958  0.77255  0.76710  0.62429
     83   2.36060  4.21349  3.57968  3.03401  3.44735  2.89554  3.92282
2.24141  2.96619  2.30378  3.39263  3.38629  3.94734  3.27332  3.29128
2.80434  2.61242  2.09451  4.94444  3.71893    196 v - - -
          2.68618  4.42225  2.77519  2.73123  3.46354  2.40513  3.72494
3.29354  2.67741  2.69355  4.24690  2.90347  2.73739  3.18146  2.89801
2.37887  2.77519  2.98518  4.58477  3.61503
          0.03085  3.88989  4.61223  0.61958  0.77255  0.76710  0.62429
     84   2.52431  4.65775  2.71706  2.44001  4.24460  2.22752  3.74750
3.69576  2.56744  3.29124  4.11912  2.14785  3.74763  2.92666  2.80438
2.43426  2.86063  3.28636  5.49105  4.16097    197 n - - -
          2.68618  4.42225  2.77519  2.73123  3.46354  2.40513  3.72494
3.29354  2.67741  2.69355  4.24690  2.90347  2.73739  3.18146  2.89801
2.37887  2.77519  2.98518  4.58477  3.61503
          0.03085  3.88989  4.61223  0.61958  0.77255  0.76710  0.62429
     85   2.54473  4.62269  2.90100  2.50027  3.97383  3.28018  2.55311
3.44039  2.48644  3.06124  3.90577  2.97161  2.61589  2.88266  2.88124
2.47851  2.67963  3.10280  5.27390  3.94788    198 s - - -
          2.68618  4.42225  2.77519  2.73123  3.46354  2.40513  3.72494
3.29354  2.67741  2.69355  4.24690  2.90347  2.73739  3.18146  2.89801
2.37887  2.77519  2.98518  4.58477  3.61503
          0.03085  3.88989  4.61223  0.61958  0.77255  0.72843  0.65907
     86   2.03831  4.68475  2.62291  2.40175  4.20262  2.87932  3.71743
3.63433  2.58185  3.23538  4.05238  2.37400  2.96645  2.88563  3.04696
2.56569  2.83285  3.24175  5.46284  4.12189    199 a - - -
          2.68621  4.42278  2.77550  2.73169  3.46392  2.40442  3.71923
3.29373  2.67787  2.69397  4.24743  2.90393  2.73694  3.18199  2.89854
2.37923  2.77535  2.98450  4.58530  3.61486
          0.47660  0.99578  4.63800  2.16385  0.12203  0.35074  1.21796
     87   2.48502  4.56076  3.18893  2.64338  3.22346  3.50079  3.73154
3.12696  2.46463  2.79636  3.65752  2.45116  3.89291  2.95880  3.03804
2.54809  2.75472  2.86326  5.09709  2.62036    225 n - - -
          2.68618  4.42225  2.77519  2.73123  3.46354  2.40513  3.72494
3.29354  2.67741  2.69355  4.24690  2.90347  2.73739  3.18146  2.89801
2.37887  2.77519  2.98518  4.58477  3.61503
          0.02276  4.18974  4.91209  0.61958  0.77255  0.48576  0.95510
     88   1.59384  4.55797  3.06192  2.72693  4.28696  1.94425  3.96104
3.71276  2.84266  3.34155  4.16494  2.91351  3.83411  2.91247  3.25822
2.46354  2.88591  3.28239  5.58070  4.28828    226 a - - -
          2.68618  4.42225  2.77519  2.73123  3.46354  2.40513  3.72494
3.29354  2.67741  2.69355  4.24690  2.90347  2.73739  3.18146  2.89801
2.37887  2.77519  2.98518  4.58477  3.61503
          0.02276  4.18974  4.91209  0.61958  0.77255  0.48576  0.95510
     89   2.83524  4.21884  4.53816  3.95193  2.88982  3.75786  4.36249
1.79084  3.80745  1.76111  2.31059  4.09135  4.35384  3.97334  3.91654
3.33952  3.06727  1.81390  4.88770  3.72679    227 l - - -
          2.68618  4.42225  2.77519  2.73123  3.46354  2.40513  3.72494
3.29354  2.67741  2.69355  4.24690  2.90347  2.73739  3.18146  2.89801
2.37887  2.77519  2.98518  4.58477  3.61503
          0.02276  4.18974  4.91209  0.61958  0.77255  0.48576  0.95510
```

```
      90   2.75101  4.17803  4.33453  3.74326  3.13720  3.89167  4.19766
2.06493  3.36109  1.59053  2.19399  3.91571  4.23094  3.78632  3.73385
3.19049  2.98053  2.16818  4.26702  3.61220    228 l - - -
         2.68618  4.42225  2.77519  2.73123  3.46354  2.40513  3.72494
3.29354  2.67741  2.69355  4.24690  2.90347  2.73739  3.18146  2.89801
2.37887  2.77519  2.98518  4.58477  3.61503
         0.02276  4.18974  4.91209  0.61958  0.77255  0.48576  0.95510
      91   2.60248  5.01349  2.67520  1.67619  4.44169  3.34189  3.76425
3.88610  2.59870  3.44969  4.25850  2.91874  2.21006  2.91336  3.07600
2.40391  3.00320  3.49034  5.64626  4.26786    229 e - - -
         2.68618  4.42225  2.77519  2.73123  3.46354  2.40513  3.72494
3.29354  2.67741  2.69355  4.24690  2.90347  2.73739  3.18146  2.89801
2.37887  2.77519  2.98518  4.58477  3.61503
         0.02276  4.18974  4.91209  0.61958  0.77255  0.48576  0.95510
      92   2.62533  5.18115  2.34140  1.99000  4.50421  2.98409  3.64687
3.97822  2.41928  3.47670  4.24508  2.72872  3.83102  2.76335  2.03817
2.67054  2.95287  3.56344  5.62639  4.21844    230 e - - -
         2.68617  4.42230  2.77525  2.73122  3.46345  2.40513  3.72481
3.29359  2.67733  2.69353  4.24695  2.90352  2.73731  3.18141  2.89806
2.37892  2.77525  2.98524  4.58482  3.61491
         0.15987  2.73230  2.49285  1.22202  0.34904  0.48576  0.95510
      93   2.35549  4.85633  1.75897  2.41054  4.37988  3.26837  3.78789
3.79154  2.66599  3.40146  4.21720  2.92332  3.82169  2.94804  3.15380
2.51362  2.13142  3.38921  5.62114  4.25362    241 d - - -
         2.68618  4.42225  2.77519  2.73123  3.46354  2.40513  3.72494
3.29354  2.67741  2.69355  4.24690  2.90347  2.73739  3.18146  2.89801
2.37887  2.77519  2.98518  4.58477  3.61503
         0.02464  4.11144  4.83378  0.61958  0.77255  0.57602  0.82584
      94   2.65451  4.98738  2.70806  2.35411  4.29545  3.37781  3.27006
3.74385  2.41161  3.29384  4.08603  2.07946  3.81467  2.76999  2.71315
2.16932  2.76442  3.37082  5.48299  4.11204    242 n - - -
         2.68618  4.42225  2.77519  2.73123  3.46354  2.40513  3.72494
3.29354  2.67741  2.69355  4.24690  2.90347  2.73739  3.18146  2.89801
2.37887  2.77519  2.98518  4.58477  3.61503
         0.02464  4.11144  4.83378  0.61958  0.77255  0.50070  0.93168
      95   1.55238  4.61132  2.96755  2.48048  4.24125  2.50985  3.88121
3.66349  2.75921  3.28711  4.11026  2.85290  3.82256  3.06868  3.19594
2.26387  2.88007  3.25794  5.52992  4.22207    243 a - - -
         2.68618  4.42225  2.77519  2.73123  3.46354  2.40513  3.72494
3.29354  2.67741  2.69355  4.24690  2.90347  2.73739  3.18146  2.89801
2.37887  2.77519  2.98518  4.58477  3.61503
         0.02365  4.15210  4.87445  0.61958  0.77255  0.45845  1.00038
      96   2.20251  4.25200  3.59008  3.02028  3.27343  3.60438  3.87064
2.37125  2.96016  2.09693  3.35938  3.20064  3.98255  3.00236  3.28229
2.68414  2.66585  2.44360  4.84455  3.47246    244 l - - -
         2.68618  4.42225  2.77519  2.73123  3.46354  2.40513  3.72494
3.29354  2.67741  2.69355  4.24690  2.90347  2.73739  3.18146  2.89801
2.37887  2.77519  2.98518  4.58477  3.61503
         0.02276  4.18974  4.91209  0.61958  0.77255  0.48576  0.95510
      97   2.00041  4.84486  2.95506  2.49073  4.13448  3.43081  2.33048
3.55962  2.46761  3.15802  3.98128  2.86290  3.86683  2.58992  2.89146
2.70363  2.73530  3.22863  5.38486  4.05623    245 a - - -
         2.68618  4.42225  2.77519  2.73123  3.46354  2.40513  3.72494
3.29354  2.67741  2.69355  4.24690  2.90347  2.73739  3.18146  2.89801
2.37887  2.77519  2.98518  4.58477  3.61503
         0.02276  4.18974  4.91209  0.61958  0.77255  0.48576  0.95510
```

```
     98   1.99461  4.54414  3.20375  2.68076  3.89253  3.39285  3.58496
3.28364  2.66354  2.94301  3.79040  3.13018  3.53121  2.91144  3.08009
2.41111  2.01544  2.82656  5.23850  3.95702    246 a - - -
          2.68618  4.42225  2.77519  2.73123  3.46354  2.40513  3.72494
3.29354  2.67741  2.69355  4.24690  2.90347  2.73739  3.18146  2.89801
2.37887  2.77519  2.98518  4.58477  3.61503
          0.02276  4.18974  4.91209  0.61958  0.77255  0.48576  0.95510
     99   3.46323  4.75610  5.11520  4.54384  3.13675  4.75729  5.05172
1.94177  4.39910  0.82379  2.34842  4.79654  4.89074  4.45432  4.48183
4.11335  3.67786  2.35123  5.27358  4.24902    247 l - - -
          2.68618  4.42225  2.77519  2.73123  3.46354  2.40513  3.72494
3.29354  2.67741  2.69355  4.24690  2.90347  2.73739  3.18146  2.89801
2.37887  2.77519  2.98518  4.58477  3.61503
          0.02276  4.18974  4.91209  0.61958  0.77255  0.48576  0.95510
    100   2.79589  5.13924  2.88321  2.24654  4.46250  3.47534  3.41387
3.89949  2.29227  3.40889  4.21083  2.96416  3.54106  1.57717  2.52298
2.77199  3.01958  3.52821  5.55163  4.21343    248 q - - -
          2.68618  4.42225  2.77519  2.73123  3.46354  2.40513  3.72494
3.29354  2.67741  2.69355  4.24690  2.90347  2.73739  3.18146  2.89801
2.37887  2.77519  2.98518  4.58477  3.61503
          0.02276  4.18974  4.91209  0.61958  0.77255  0.48576  0.95510
    101   2.65509  4.14379  4.03593  3.45829  2.93035  3.73852  3.73414
2.37326  3.34008  2.20171  2.09617  3.68059  4.10376  3.56821  3.53790
2.92351  2.88643  2.38492  4.61110  2.39773    249 m - - -
          2.68618  4.42225  2.77519  2.73123  3.46354  2.40513  3.72494
3.29354  2.67741  2.69355  4.24690  2.90347  2.73739  3.18146  2.89801
2.37887  2.77519  2.98518  4.58477  3.61503
          0.25435  4.18974  1.56336  0.61958  0.77255  0.48576  0.95510
    102   2.44543  4.17983  3.75526  3.18278  3.08046  3.65416  3.40767
2.28170  3.08784  2.12476  2.31332  3.50600  4.02062  3.36461  3.36077
2.92533  2.83952  2.35489  4.75422  3.53224    250 l - - -
          2.68618  4.42225  2.77519  2.73123  3.46354  2.40513  3.72494
3.29354  2.67741  2.69355  4.24690  2.90347  2.73739  3.18146  2.89801
2.37887  2.77519  2.98518  4.58477  3.61503
          0.06138  3.96400  3.20539  0.61958  0.77255  0.36670  1.18097
    103   2.89586  5.11764  3.25361  2.64420  4.42849  3.60697  3.19839
3.86749  2.09278  3.15158  4.18884  3.11176  3.98037  1.82197  1.75896
2.89177  3.09071  3.53005  5.45174  4.18037    251 r - - -
          2.68666  4.42039  2.77598  2.73156  3.46381  2.40482  3.72260
3.29351  2.67781  2.69398  4.24426  2.90276  2.73732  3.18132  2.89800
2.37847  2.77542  2.98555  4.58566  3.61580
          0.52416  1.34164  1.92050  2.73892  0.06682  0.51663  0.90765
    104   2.68669  4.99909  2.55785  2.31388  4.28263  3.34895  3.40132
3.72626  2.37070  3.28572  4.09568  2.75302  2.17406  2.53783  2.89155
2.59392  2.93078  3.36780  5.48462  4.11570    283 p - - -
          2.68620  4.42238  2.77533  2.73131  3.46367  2.40526  3.72356
3.29367  2.67754  2.69363  4.24660  2.90360  2.73738  3.18006  2.89814
2.37896  2.77533  2.98532  4.58490  3.61516
          0.46549  1.24766  2.46510  1.05525  0.42787  0.66916  0.71773
    105   2.50027  4.47428  3.16731  2.61275  3.66653  3.44133  3.68328
2.95578  2.48193  2.51104  3.58096  3.07947  3.08003  2.70617  2.96064
2.61074  2.64675  2.53489  5.04023  3.76852    291 k - - -
          2.68618  4.42225  2.77519  2.73123  3.46354  2.40513  3.72494
3.29354  2.67741  2.69355  4.24690  2.90347  2.73739  3.18146  2.89801
2.37887  2.77519  2.98518  4.58477  3.61503
          0.02945  3.93548  4.65783  0.61958  0.77255  0.66409  0.72308
```

```
    106   2.61602  4.98561  2.68701  2.14450  4.27179  3.35339  2.79829
3.72279  2.28851  3.26285  4.04685  2.61914  3.77278  2.53856  2.81886
2.45696  2.84996  3.34669  5.44550  3.64514    292 e - - -
          2.68618  4.42225  2.77519  2.73123  3.46354  2.40513  3.72494
3.29354  2.67741  2.69355  4.24690  2.90347  2.73739  3.18146  2.89801
2.37887  2.77519  2.98518  4.58477  3.61503
          0.02815  3.98017  4.70251  0.61958  0.77255  0.69854  0.68778
    107   2.39750  4.67551  2.92160  2.48017  4.04579  3.31539  2.40470
3.46904  2.49408  3.08416  3.91241  2.88119  2.98995  2.86092  2.92026
2.42651  2.72061  3.12805  5.32404  4.00524    293 a - - -
          2.68618  4.42225  2.77519  2.73123  3.46354  2.40513  3.72494
3.29354  2.67741  2.69355  4.24690  2.90347  2.73739  3.18146  2.89801
2.37887  2.77519  2.98518  4.58477  3.61503
          0.02815  3.98017  4.70251  0.61958  0.77255  0.69854  0.68778
    108   2.52685  4.62397  3.06730  2.44569  3.82996  3.42948  3.63876
3.01329  2.45946  2.73253  3.34361  3.00392  3.33169  2.35686  2.78736
2.53613  2.80011  2.93640  5.15080  3.85204    294 q - - -
          2.68618  4.42225  2.77519  2.73123  3.46354  2.40513  3.72494
3.29354  2.67741  2.69355  4.24690  2.90347  2.73739  3.18146  2.89801
2.37887  2.77519  2.98518  4.58477  3.61503
          0.02815  3.98017  4.70251  0.61958  0.77255  0.66805  0.71889
    109   2.48907  4.10012  3.20487  2.66489  3.68598  3.41692  3.72823
3.05864  2.61994  2.26847  3.60820  2.80432  2.77114  2.86718  3.01675
2.60380  2.73436  2.79047  5.06511  3.79738    295 l - - -
          2.68618  4.42225  2.77519  2.73123  3.46354  2.40513  3.72494
3.29354  2.67741  2.69355  4.24690  2.90347  2.73739  3.18146  2.89801
2.37887  2.77519  2.98518  4.58477  3.61503
          0.02762  3.99872  4.72107  0.61958  0.77255  0.61969  0.77244
    110   2.64436  4.91362  2.87783  2.34797  4.16366  3.25453  3.15093
3.61233  2.34671  3.17947  3.98635  2.70913  3.81420  1.96313  2.77776
2.65107  2.87477  3.15162  5.37238  3.71734    296 q - - -
          2.68618  4.42225  2.77519  2.73123  3.46354  2.40513  3.72494
3.29354  2.67741  2.69355  4.24690  2.90347  2.73739  3.18146  2.89801
2.37887  2.77519  2.98518  4.58477  3.61503
          0.02658  4.03682  4.75916  0.61958  0.77255  0.64944  0.73885
    111   2.57642  4.73063  2.81671  2.24636  3.95638  3.32112  3.44631
3.36573  2.43576  2.62237  3.18546  2.85560  3.44359  2.71517  2.89196
2.57682  2.80804  2.92371  5.24206  3.91766    297 e - - -
          2.68618  4.42225  2.77519  2.73123  3.46354  2.40513  3.72494
3.29354  2.67741  2.69355  4.24690  2.90347  2.73739  3.18146  2.89801
2.37887  2.77519  2.98518  4.58477  3.61503
          0.02658  4.03682  4.75916  0.61958  0.77255  0.64944  0.73885
    112   2.29749  4.57135  3.03010  2.56998  4.05849  2.91651  3.51044
3.47267  2.60205  3.09917  3.92389  2.92694  2.44542  2.93910  3.03564
2.38341  2.37723  3.10985  5.35406  4.05003    298 a - - -
          2.68618  4.42225  2.77519  2.73123  3.46354  2.40513  3.72494
3.29354  2.67741  2.69355  4.24690  2.90347  2.73739  3.18146  2.89801
2.37887  2.77519  2.98518  4.58477  3.61503
          0.02658  4.03682  4.75916  0.61958  0.77255  0.64944  0.73885
    113   2.81986  5.29814  1.99123  1.68362  4.60953  3.27753  3.71382
4.09469  2.62479  3.61189  4.42595  2.75516  2.31140  2.86178  3.16571
2.74796  3.08832  3.68488  5.78134  4.33350    299 e - - -
          2.68618  4.42225  2.77519  2.73123  3.46354  2.40513  3.72494
3.29354  2.67741  2.69355  4.24690  2.90347  2.73739  3.18146  2.89801
2.37887  2.77519  2.98518  4.58477  3.61503
          0.28320  4.03682  1.47414  0.61958  0.77255  0.43223  1.04713
```

```
    114   2.38663  4.60905  2.97488  2.51146  3.99578  3.30328  3.69403
3.39908  2.50896  3.02997  3.86343  2.89497  2.52258  2.51982  2.93020
2.41310  2.80656  2.83438  5.28855  3.98288    300 a - - -
          2.68618  4.42225  2.77519  2.73123  3.46354  2.40513  3.72494
3.29354  2.67741  2.69355  4.24690  2.90347  2.73739  3.18146  2.89801
2.37887  2.77519  2.98518  4.58477  3.61503
          0.18375  3.95156  1.90626  0.61958  0.77255  0.44007  1.03281
    115   2.27051  4.68101  2.67160  2.46181  4.24833  2.96492  3.77593
3.68213  2.64731  3.28479  4.10205  2.60963  2.08068  2.94832  3.10775
2.47139  2.86113  3.27834  5.51220  4.17680    301 p - - -
          2.68618  4.42225  2.77519  2.73123  3.46354  2.40513  3.72494
3.29354  2.67741  2.69355  4.24690  2.90347  2.73739  3.18146  2.89801
2.37887  2.77519  2.98518  4.58477  3.61503
          0.02724  4.01266  4.73500  0.61958  0.77255  0.61219  0.78124
    116   2.29765  4.33325  3.01308  2.27695  3.93442  3.41551  3.63368
3.34064  2.45274  2.96437  3.79068  2.71540  3.34348  2.72527  2.90574
2.49865  2.69834  2.92533  5.22921  3.50938    302 e - - -
          2.68618  4.42225  2.77519  2.73123  3.46354  2.40513  3.72494
3.29354  2.67741  2.69355  4.24690  2.90347  2.73739  3.18146  2.89801
2.37887  2.77519  2.98518  4.58477  3.61503
          0.02629  4.04753  4.76987  0.61958  0.77255  0.63955  0.74978
    117   2.56078  3.92861  3.25760  2.86104  3.29499  3.53714  3.56822
2.78736  2.81505  2.24370  3.19694  3.14995  3.60873  3.01033  3.17092
2.71022  2.79254  2.50164  4.86491  2.71880    303 l - - -
          2.68618  4.42225  2.77519  2.73123  3.46354  2.40513  3.72494
3.29354  2.67741  2.69355  4.24690  2.90347  2.73739  3.18146  2.89801
2.37887  2.77519  2.98518  4.58477  3.61503
          0.02629  4.04753  4.76987  0.61958  0.77255  0.43762  1.03725
    118   2.60438  4.02715  2.85135  2.31240  4.06337  3.23030  3.45290
3.32541  2.43369  3.08151  3.89110  2.84199  2.75464  2.71743  2.89895
2.39332  2.77028  2.94689  5.32109  3.98265    304 e - - -
          2.68618  4.42225  2.77519  2.73123  3.46354  2.40513  3.72494
3.29354  2.67741  2.69355  4.24690  2.90347  2.73739  3.18146  2.89801
2.37887  2.77519  2.98518  4.58477  3.61503
          0.02330  4.16669  4.88903  0.61958  0.77255  0.51394  0.91163
    119   2.52714  4.95478  2.93638  2.31142  4.24343  3.32599  3.62344
3.52430  1.85487  3.23307  4.02469  2.83133  3.66909  2.64954  2.80516
2.64627  2.33855  3.31716  5.42784  4.07440    305 k - - -
          2.68618  4.42225  2.77519  2.73123  3.46354  2.40513  3.72494
3.29354  2.67741  2.69355  4.24690  2.90347  2.73739  3.18146  2.89801
2.37887  2.77519  2.98518  4.58477  3.61503
          0.02330  4.16669  4.88903  0.61958  0.77255  0.46854  0.98328
    120   2.64440  5.08686  2.60424  1.97277  4.40548  3.09528  3.60443
3.87163  2.30704  3.37865  4.13863  2.87701  3.54553  2.62686  2.24091
2.61635  2.87674  3.18063  5.53299  4.13969    306 e - - -
          2.68618  4.42225  2.77519  2.73123  3.46354  2.40513  3.72494
3.29354  2.67741  2.69355  4.24690  2.90347  2.73739  3.18146  2.89801
2.37887  2.77519  2.98518  4.58477  3.61503
          0.02276  4.18974  4.91209  0.61958  0.77255  0.48576  0.95510
    121   2.95843  5.51669  2.07056  1.13632  4.81815  3.32633  3.80747
4.32880  2.76213  3.81914  4.64080  2.60990  3.47713  2.95958  3.33103
2.85866  3.23396  3.89819  5.97642  4.49009    307 e - - -
          2.68618  4.42225  2.77519  2.73123  3.46354  2.40513  3.72494
3.29354  2.67741  2.69355  4.24690  2.90347  2.73739  3.18146  2.89801
2.37887  2.77519  2.98518  4.58477  3.61503
          0.03767  4.18974  3.82494  0.61958  0.77255  0.48576  0.95510
```

```
    122   2.59615  3.42918  3.38228  2.65397  3.52687  3.54985  3.79392
2.83057  2.64153  2.59561  2.62978  3.24690  3.57032  2.94938  3.00071
2.69941  2.55301  2.46217  4.95260  3.70688    308 v - - -
          2.68618  4.42225  2.77519  2.73123  3.46354  2.40513  3.72494
3.29354  2.67741  2.69355  4.24690  2.90347  2.73739  3.18146  2.89801
2.37887  2.77519  2.98518  4.58477  3.61503
          0.02310  4.17517  4.89752  0.61958  0.77255  0.50374  0.92702
    123   2.35072  4.44690  3.29992  2.60728  3.60572  3.41554  2.91261
2.48293  2.70236  2.52752  3.36635  3.18880  3.50213  2.89146  3.09800
2.75918  2.83040  2.54299  5.01074  3.75212    309 a - - -
          2.68618  4.42225  2.77519  2.73123  3.46354  2.40513  3.72494
3.29354  2.67741  2.69355  4.24690  2.90347  2.73739  3.18146  2.89801
2.37887  2.77519  2.98518  4.58477  3.61503
          0.16802  4.17517  1.97120  0.61958  0.77255  0.50374  0.92702
    124   2.44827  4.46703  3.20768  2.64865  3.65229  3.46489  3.71116
2.60265  2.55668  2.70819  3.44549  2.97549  3.12686  2.94958  2.92794
2.42954  2.63203  2.67869  5.03598  3.76674    310 s - - -
          2.68618  4.42225  2.77519  2.73123  3.46354  2.40513  3.72494
3.29354  2.67741  2.69355  4.24690  2.90347  2.73739  3.18146  2.89801
2.37887  2.77519  2.98518  4.58477  3.61503
          0.02666  4.03381  4.75616  0.61958  0.77255  0.65218  0.73587
    125   2.47447  4.67587  3.01835  2.48802  3.98242  3.27543  3.66136
3.39086  2.47283  2.92130  3.83613  2.86596  2.89013  2.72740  2.83040
2.20534  2.56622  2.98238  5.26919  3.95388    311 s - - -
          2.68618  4.42225  2.77519  2.73123  3.46354  2.40513  3.72494
3.29354  2.67741  2.69355  4.24690  2.90347  2.73739  3.18146  2.89801
2.37887  2.77519  2.98518  4.58477  3.61503
          0.02666  4.03381  4.75616  0.61958  0.77255  0.43497  1.04209
    126   2.66978  5.08115  2.22132  2.09501  4.38398  3.18309  3.63433
3.84447  2.37491  3.37226  3.76231  2.86503  2.49823  2.75564  2.91633
2.55212  2.90884  3.45166  5.54704  4.15225    312 e - - -
          2.68618  4.42225  2.77519  2.73123  3.46354  2.40513  3.72494
3.29354  2.67741  2.69355  4.24690  2.90347  2.73739  3.18146  2.89801
2.37887  2.77519  2.98518  4.58477  3.61503
          0.02336  4.16405  4.88639  0.61958  0.77255  0.51708  0.90699
    127   2.09096  4.37363  3.40458  2.84249  3.54621  3.34532  3.81243
2.89664  2.78013  2.01876  3.34766  3.26186  3.46798  3.10891  2.86397
2.78434  2.76948  2.59897  4.97275  3.72987    313 l - - -
          2.68618  4.42225  2.77519  2.73123  3.46354  2.40513  3.72494
3.29354  2.67741  2.69355  4.24690  2.90347  2.73739  3.18146  2.89801
2.37887  2.77519  2.98518  4.58477  3.61503
          0.02336  4.16405  4.88639  0.61958  0.77255  0.51708  0.90699
    128   2.71590  4.12010  4.40636  3.81372  2.53689  3.87565  4.19074
2.05768  3.66128  1.76489  2.80338  3.93885  4.22023  3.83140  3.76627
3.05564  2.94717  1.83316  4.73788  3.32783    314 l - - -
          2.68618  4.42225  2.77519  2.73123  3.46354  2.40513  3.72494
3.29354  2.67741  2.69355  4.24690  2.90347  2.73739  3.18146  2.89801
2.37887  2.77519  2.98518  4.58477  3.61503
          0.02336  4.16405  4.88639  0.61958  0.77255  0.51708  0.90699
    129   2.82993  5.18852  2.56412  1.47644  4.49172  3.37268  3.48326
3.97698  2.57142  3.51361  4.33662  2.88220  2.24933  2.90270  3.03714
2.79252  3.09139  3.59646  5.67736  4.27993    315 e - - -
          2.68618  4.42225  2.77519  2.73123  3.46354  2.40513  3.72494
3.29354  2.67741  2.69355  4.24690  2.90347  2.73739  3.18146  2.89801
2.37887  2.77519  2.98518  4.58477  3.61503
          0.02336  4.16405  4.88639  0.61958  0.77255  0.46667  0.98642
```

```
    130   2.57344  4.84436  2.63085  1.98421  4.09280  3.43505  3.64170
2.96921  2.43520  3.02320  3.76134  2.77262  3.82836  2.78582  2.90236
2.64148  2.66368  2.88993  5.34428  4.00173    316 e - - -
          2.68618  4.42225  2.77519  2.73123  3.46354  2.40513  3.72494
3.29354  2.67741  2.69355  4.24690  2.90347  2.73739  3.18146  2.89801
2.37887  2.77519  2.98518  4.58477  3.61503
          0.02276  4.18974  4.91209  0.61958  0.77255  0.48576  0.95510
    131   2.44779  4.78708  3.00354  2.48116  4.10252  3.40422  2.96951
3.52279  2.48496  3.12450  3.93637  2.55528  3.54537  2.83657  2.94136
1.99696  2.72264  2.94424  5.36255  4.02981    317 s - - -
          2.68618  4.42225  2.77519  2.73123  3.46354  2.40513  3.72494
3.29354  2.67741  2.69355  4.24690  2.90347  2.73739  3.18146  2.89801
2.37887  2.77519  2.98518  4.58477  3.61503
          0.02276  4.18974  4.91209  0.61958  0.77255  0.48576  0.95510
    132   2.36364  3.81469  3.03012  2.64773  3.83924  3.16749  3.75650
3.22891  2.63202  2.88936  3.73779  3.11235  2.60234  2.89019  3.05849
2.41521  2.56348  2.60884  5.19044  3.90573    318 a - - -
          2.68618  4.42225  2.77519  2.73123  3.46354  2.40513  3.72494
3.29354  2.67741  2.69355  4.24690  2.90347  2.73739  3.18146  2.89801
2.37887  2.77519  2.98518  4.58477  3.61503
          0.06329  4.18974  3.07523  0.61958  0.77255  0.48576  0.95510
    133   2.52419  4.23224  3.73649  3.17954  3.41148  3.26306  3.99095
2.18996  3.10594  2.34195  3.37780  3.51065  2.75395  3.39287  3.40339
2.91775  2.86888  1.83374  4.91309  3.69715    319 v - - -
          2.68618  4.42225  2.77519  2.73123  3.46354  2.40513  3.72494
3.29354  2.67741  2.69355  4.24690  2.90347  2.73739  3.18146  2.89801
2.37887  2.77519  2.98518  4.58477  3.61503
          0.02369  4.15014  4.87249  0.61958  0.77255  0.45714  1.00264
    134   2.33424  4.43802  3.31835  2.75647  3.42894  3.41714  3.77636
2.88295  2.62358  2.58591  3.54032  3.20368  2.89826  2.84374  3.11481
2.68016  2.75668  2.20647  5.00933  3.75297    320 v - - -
          2.68618  4.42225  2.77519  2.73123  3.46354  2.40513  3.72494
3.29354  2.67741  2.69355  4.24690  2.90347  2.73739  3.18146  2.89801
2.37887  2.77519  2.98518  4.58477  3.61503
          0.02276  4.18974  4.91209  0.61958  0.77255  0.48576  0.95510
    135   2.47859  4.90456  2.95128  2.22134  4.18464  2.96221  3.62935
3.46202  2.14409  3.18886  3.98154  2.93479  3.25912  2.76123  2.87442
2.26025  2.79400  3.05042  5.40013  4.04411    321 k - - -
          2.68618  4.42225  2.77519  2.73123  3.46354  2.40513  3.72494
3.29354  2.67741  2.69355  4.24690  2.90347  2.73739  3.18146  2.89801
2.37887  2.77519  2.98518  4.58477  3.61503
          0.29025  4.18974  1.44066  0.61958  0.77255  0.48576  0.95510
    136   2.59739  4.90021  2.71354  2.25071  4.18437  3.35020  3.58168
3.61905  2.25409  3.18201  3.97938  2.67539  3.50129  2.71612  2.62466
2.47215  2.36884  3.26199  5.38545  4.02714    322 e - - -
          2.68618  4.42225  2.77519  2.73123  3.46354  2.40513  3.72494
3.29354  2.67741  2.69355  4.24690  2.90347  2.73739  3.18146  2.89801
2.37887  2.77519  2.98518  4.58477  3.61503
          0.02964  3.92914  4.65148  0.61958  0.77255  0.66034  0.72707
    137   2.45949  4.93733  2.60202  2.04936  4.22464  2.84580  3.58905
3.66777  2.27777  3.10484  4.01303  2.84928  3.54504  2.71875  2.85815
2.51352  2.83780  3.17869  5.42241  4.04962    323 e - - -
          2.68621  4.42197  2.77526  2.73124  3.46361  2.40508  3.72501
3.29342  2.67748  2.69348  4.24697  2.90330  2.73730  3.18146  2.89808
2.37891  2.77520  2.98525  4.58484  3.61510
          0.31252  1.34967  4.70203  0.65531  0.73247  0.39083  1.12853
```

```
    138   2.60145  5.01274  2.74497  1.89497  4.32551  2.86732  3.64585
3.77557  2.37855  3.32092  4.10470  2.89309  2.65895  2.77250  2.91835
2.55943  2.77182  3.26438  5.50961  4.12965    327 e - - -
          2.68618  4.42225  2.77519  2.73123  3.46354  2.40513  3.72494
3.29354  2.67741  2.69355  4.24690  2.90347  2.73739  3.18146  2.89801
2.37887  2.77519  2.98518  4.58477  3.61503
          0.02305  4.17722  4.89957  0.61958  0.77255  0.50124  0.93084
    139   2.26574  4.57981  3.17247  2.63143  3.85035  3.43729  3.74596
3.23222  2.60562  2.63216  3.74530  3.10155  2.45364  2.70936  3.02744
2.45942  2.68826  2.79441  5.19607  3.90962    328 a - - -
          2.68618  4.42225  2.77519  2.73123  3.46354  2.40513  3.72494
3.29354  2.67741  2.69355  4.24690  2.90347  2.73739  3.18146  2.89801
2.37887  2.77519  2.98518  4.58477  3.61503
          0.02305  4.17722  4.89957  0.61958  0.77255  0.47620  0.97058
    140   2.14647  4.46785  3.28705  2.73074  3.67877  3.02262  3.77840
2.95489  2.70175  2.63375  3.60728  3.07559  3.19337  3.02797  3.01557
2.62665  2.61141  2.35009  5.07147  3.80804    329 a - - -
          2.68618  4.42225  2.77519  2.73123  3.46354  2.40513  3.72494
3.29354  2.67741  2.69355  4.24690  2.90347  2.73739  3.18146  2.89801
2.37887  2.77519  2.98518  4.58477  3.61503
          0.02276  4.18974  4.91209  0.61958  0.77255  0.48576  0.95510
    141   2.94670  5.14993  3.01251  2.63423  4.62552  3.21307  3.75905
4.04425  1.07813  3.54138  4.38569  2.82313  4.00650  2.90515  2.52304
2.94254  3.19096  3.67296  5.62635  4.35929    330 k - - -
          2.68618  4.42225  2.77519  2.73123  3.46354  2.40513  3.72494
3.29354  2.67741  2.69355  4.24690  2.90347  2.73739  3.18146  2.89801
2.37887  2.77519  2.98518  4.58477  3.61503
          0.02276  4.18974  4.91209  0.61958  0.77255  0.48576  0.95510
    142   2.39159  4.85469  3.01054  2.51313  4.28584  3.39228  3.70910
3.70586  1.65316  3.27853  4.08225  3.00843  2.83746  2.85094  2.83934
2.49479  2.72538  3.33027  5.47671  4.14917    331 k - - -
          2.68618  4.42225  2.77519  2.73123  3.46354  2.40513  3.72494
3.29354  2.67741  2.69355  4.24690  2.90347  2.73739  3.18146  2.89801
2.37887  2.77519  2.98518  4.58477  3.61503
          0.02276  4.18974  4.91209  0.61958  0.77255  0.48576  0.95510
    143   3.09220  5.25758  3.55094  2.83118  4.69386  3.74830  3.42256
4.04462  1.49624  3.21787  4.32640  3.24633  4.09252  2.69831  1.37581
3.07899  3.25102  3.71731  5.51998  4.33844    332 r - - -
          2.68618  4.42225  2.77519  2.73123  3.46354  2.40513  3.72494
3.29354  2.67741  2.69355  4.24690  2.90347  2.73739  3.18146  2.89801
2.37887  2.77519  2.98518  4.58477  3.61503
          0.05689  4.18974  3.21501  0.61958  0.77255  0.48576  0.95510
    144   2.77573  5.06089  3.05273  2.35748  4.39680  3.50705  3.63630
3.80823  2.20769  3.32703  4.13589  3.01097  3.90222  1.82928  2.04914
2.76927  2.83446  3.30045  5.47595  4.16945    333 q - - -
          2.68618  4.42225  2.77519  2.73123  3.46354  2.40513  3.72494
3.29354  2.67741  2.69355  4.24690  2.90347  2.73739  3.18146  2.89801
2.37887  2.77519  2.98518  4.58477  3.61503
          0.02355  4.15639  4.87874  0.61958  0.77255  0.52607  0.89385
    145   2.36353  4.86256  2.97688  2.43183  4.13944  2.71943  3.63450
3.56362  2.33409  3.14595  3.94892  2.88499  3.82269  2.34248  2.62576
2.57654  2.72186  2.93640  5.36675  4.02495    334 k - - -
          2.68618  4.42225  2.77519  2.73123  3.46354  2.40513  3.72494
3.29354  2.67741  2.69355  4.24690  2.90347  2.73739  3.18146  2.89801
2.37887  2.77519  2.98518  4.58477  3.61503
          0.22843  4.15639  1.66837  0.61958  0.77255  0.52607  0.89385
```

```
    146   2.58572  4.70552  2.96918  2.47222  3.98549  2.26158  3.45608
3.40005  2.40910  3.01460  3.53994  2.73489  3.60808  2.81077  2.63718
2.64051  2.83309  3.08376  5.25840  3.94627    335 g - - -
          2.68618  4.42225  2.77519  2.73123  3.46354  2.40513  3.72494
3.29354  2.67741  2.69355  4.24690  2.90347  2.73739  3.18146  2.89801
2.37887  2.77519  2.98518  4.58477  3.61503
          0.02882  3.95679  4.67913  0.61958  0.77255  0.42548  1.05974
    147   2.99390  5.22985  3.34372  2.70406  4.66858  3.31138  3.63828
4.03373  1.49789  3.47110  4.30266  3.15170  4.02255  2.42705  1.61111
2.97288  3.17162  3.68420  5.52826  4.31166    336 k - - -
          2.68616  4.42199  2.77524  2.73128  3.46358  2.40512  3.72499
3.29347  2.67740  2.69359  4.24694  2.90345  2.73744  3.18125  2.89799
2.37883  2.77524  2.98523  4.58481  3.61507
          0.11066  2.53043  3.68350  1.01428  0.45046  0.45527  1.00588
    148   2.57760  4.98845  3.00279  2.44224  4.30080  3.31955  3.62207
3.73019  2.17030  3.26950  4.06117  2.96077  3.84738  2.19572  2.13555
2.52508  2.67889  3.25194  5.44421  4.10501    341 r - - -
          2.68619  4.42226  2.77521  2.73124  3.46355  2.40514  3.72496
3.29355  2.67742  2.69356  4.24691  2.90348  2.73741  3.18130  2.89802
2.37888  2.77521  2.98505  4.58478  3.61504
          0.06440  2.90241  4.89521  0.64333  0.74558  0.50653  0.92277
    149   2.49156  4.58433  3.18260  2.64342  3.85520  3.44508  3.74750
3.23568  2.52758  2.59421  3.75000  3.11012  2.18628  2.95368  2.87594
2.44538  2.71328  2.87508  5.19644  3.91420    344 p - - -
          2.68618  4.42225  2.77519  2.73123  3.46354  2.40513  3.72494
3.29354  2.67741  2.69355  4.24690  2.90347  2.73739  3.18146  2.89801
2.37887  2.77519  2.98518  4.58477  3.61503
          0.02316  4.17286  4.89521  0.61958  0.77255  0.50653  0.92277
    150   2.87508  4.95889  3.29010  2.77440  4.39844  3.55948  3.76923
3.66143  1.11180  3.29184  4.19177  3.20421  4.01349  2.92424  2.48619
2.92350  2.91277  3.11693  5.50510  4.26013    345 k - - -
          2.68618  4.42225  2.77519  2.73123  3.46354  2.40513  3.72494
3.29354  2.67741  2.69355  4.24690  2.90347  2.73739  3.18146  2.89801
2.37887  2.77519  2.98518  4.58477  3.61503
          0.02316  4.17286  4.89521  0.61958  0.77255  0.50653  0.92277
    151   2.62240  4.87537  2.81561  2.11251  4.13072  3.42299  3.63734
3.55571  2.36662  2.96341  3.94611  2.84301  3.82371  2.71422  2.89565
2.20676  2.74724  2.62793  5.36924  4.02097    346 e - - -
          2.68618  4.42225  2.77519  2.73123  3.46354  2.40513  3.72494
3.29354  2.67741  2.69355  4.24690  2.90347  2.73739  3.18146  2.89801
2.37887  2.77519  2.98518  4.58477  3.61503
          0.02316  4.17286  4.89521  0.61958  0.77255  0.50653  0.92277
    152   2.40084  4.79973  2.91497  2.50972  4.17997  3.37443  3.75064
3.60046  2.54930  3.04730  4.03518  2.39840  1.99504  2.76174  2.97885
2.69072  2.92217  3.25172  5.44055  4.11193    347 p - - -
          2.68618  4.42225  2.77519  2.73123  3.46354  2.40513  3.72494
3.29354  2.67741  2.69355  4.24690  2.90347  2.73739  3.18146  2.89801
2.37887  2.77519  2.98518  4.58477  3.61503
          0.02316  4.17286  4.89521  0.61958  0.77255  0.47299  0.97587
    153   3.08029  5.23304  3.44766  2.82077  4.70404  3.70649  3.69136
4.05893  1.05894  3.50343  4.35929  3.23843  4.08851  2.82345  1.95703
2.88258  3.26051  3.72356  5.55021  4.36276    348 k - - -
          2.68618  4.42225  2.77519  2.73123  3.46354  2.40513  3.72494
3.29354  2.67741  2.69355  4.24690  2.90347  2.73739  3.18146  2.89801
2.37887  2.77519  2.98518  4.58477  3.61503
          0.03842  4.18974  3.79223  0.61958  0.77255  0.48576  0.95510
```

154 1.96679 4.44094 3.32257 2.78416 3.80077 3.19350 3.84187
2.97729 2.75451 2.85742 3.54908 3.19907 2.32941 3.08209 3.15282
2.46473 2.72653 2.77803 5.18029 3.91794 349 a - - -
2.68588 4.42241 2.77535 2.73129 3.46370 2.40522 3.72510
3.29362 2.67756 2.69365 4.24664 2.90325 2.73735 3.18144 2.89816
2.37865 2.77509 2.98513 4.58493 3.61519
0.28883 1.41309 4.89678 1.19364 0.36114 0.50463 0.92565
155 2.81054 4.97989 3.23381 2.63268 4.31175 3.56416 3.66562
3.69011 1.32226 3.09627 3.84358 3.10385 3.94973 2.80741 2.38451
2.67755 2.88102 3.36673 5.41478 4.14682 357 k - - -
2.68618 4.42225 2.77519 2.73123 3.46354 2.40513 3.72494
3.29354 2.67741 2.69355 4.24690 2.90347 2.73739 3.18146 2.89801
2.37887 2.77519 2.98518 4.58477 3.61503
0.02312 4.17444 4.89678 0.61958 0.77255 0.50463 0.92565
156 3.18267 5.28405 3.57439 2.91381 4.76626 3.76616 3.70616
4.11310 0.96705 3.53577 4.41457 3.30584 4.13983 2.84127 1.89424
3.17350 3.34539 3.79362 5.55376 4.40114 358 k - - -
2.68618 4.42225 2.77519 2.73123 3.46354 2.40513 3.72494
3.29354 2.67741 2.69355 4.24690 2.90347 2.73739 3.18146 2.89801
2.37887 2.77519 2.98518 4.58477 3.61503
0.02312 4.17444 4.89678 0.61958 0.77255 0.50463 0.92565
157 2.07755 4.46574 3.28598 2.76595 3.92789 2.75511 3.85833
3.31416 2.74824 2.98327 3.31010 3.17606 2.32114 3.07784 3.15553
2.52588 2.42370 2.98363 5.28014 4.00939 359 a - - -
2.68618 4.42225 2.77519 2.73123 3.46354 2.40513 3.72494
3.29354 2.67741 2.69355 4.24690 2.90347 2.73739 3.18146 2.89801
2.37887 2.77519 2.98518 4.58477 3.61503
0.02312 4.17444 4.89678 0.61958 0.77255 0.50463 0.92565
158 2.49887 4.88041 3.00965 2.45194 4.16665 3.43807 3.63803
3.58775 1.79422 3.09073 3.76163 2.96848 3.27243 2.77595 2.70931
2.36422 2.73553 3.24417 5.37851 4.04217 360 k - - -
2.68618 4.42225 2.77519 2.73123 3.46354 2.40513 3.72494
3.29354 2.67741 2.69355 4.24690 2.90347 2.73739 3.18146 2.89801
2.37887 2.77519 2.98518 4.58477 3.61503
0.04944 4.17444 3.41583 0.61958 0.77255 0.47414 0.97397
159 2.97314 5.17428 3.16489 2.67064 4.60687 3.12584 3.69331
3.99298 1.12286 3.46894 4.31226 3.13041 4.01663 2.72042 2.29677
2.96413 3.18019 3.64501 5.55489 4.31727 361 k - - -
2.68618 4.42225 2.77519 2.73123 3.46354 2.40513 3.72494
3.29354 2.67741 2.69355 4.24690 2.90347 2.73739 3.18146 2.89801
2.37887 2.77519 2.98518 4.58477 3.61503
0.24073 4.16443 1.61743 0.61958 0.77255 0.51663 0.90765
160 2.25942 4.45483 3.14072 2.61919 3.58709 2.85696 3.73419
3.22844 2.61310 2.88904 3.73399 2.82906 3.18166 2.94387 3.03369
2.26526 2.68682 2.69910 5.18280 3.90096 362 a - - -
2.68618 4.42225 2.77519 2.73123 3.46354 2.40513 3.72494
3.29354 2.67741 2.69355 4.24690 2.90347 2.73739 3.18146 2.89801
2.37887 2.77519 2.98518 4.58477 3.61503
0.02894 3.95264 4.67499 0.61958 0.77255 0.39576 1.11830
161 2.38757 4.48453 3.26080 2.70417 3.68063 3.49435 3.76020
2.93774 2.67142 2.54682 3.60502 3.16104 2.62705 2.77054 3.07586
2.53244 2.66026 2.48108 5.07014 3.80362 363 a - - -
2.68618 4.42225 2.77519 2.73123 3.46354 2.40513 3.72494
3.29354 2.67741 2.69355 4.24690 2.90347 2.73739 3.18146 2.89801
2.37887 2.77519 2.98518 4.58477 3.61503
0.02330 4.16669 4.88903 0.61958 0.77255 0.51394 0.91163

```
    162   2.12814  4.37355  3.09539  2.52593  4.14223  3.46624  3.65955
3.54880  2.13552  3.13547  3.95388  3.02325  3.86644  2.73179  2.04623
2.53392  2.89756  3.21966  5.35302  4.04440    364 r - - -
          2.68618  4.42225  2.77519  2.73123  3.46354  2.40513  3.72494
3.29354  2.67741  2.69355  4.24690  2.90347  2.73739  3.18146  2.89801
2.37887  2.77519  2.98518  4.58477  3.61503
          0.02330  4.16669  4.88903  0.61958  0.77255  0.51394  0.91163
    163   2.02337  4.64042  3.20003  2.66979  3.99563  3.43825  3.77658
3.26503  1.82249  2.99656  3.86055  3.12820  3.89095  2.96421  2.92490
2.71587  2.70692  2.54294  5.30694  4.02390    365 k - - -
          2.68618  4.42225  2.77519  2.73123  3.46354  2.40513  3.72494
3.29354  2.67741  2.69355  4.24690  2.90347  2.73739  3.18146  2.89801
2.37887  2.77519  2.98518  4.58477  3.61503
          0.02330  4.16669  4.88903  0.61958  0.77255  0.46854  0.98328
    164   2.41553  4.36491  3.44170  2.93071  3.81109  2.96041  3.94640
3.02505  2.89755  2.87036  3.74292  3.28958  1.97115  3.21626  3.26790
2.57761  2.57958  2.25967  5.21195  3.96766    366 p - - -
          2.68618  4.42225  2.77519  2.73123  3.46354  2.40513  3.72494
3.29354  2.67741  2.69355  4.24690  2.90347  2.73739  3.18146  2.89801
2.37887  2.77519  2.98518  4.58477  3.61503
          0.02276  4.18974  4.91209  0.61958  0.77255  0.48576  0.95510
    165   2.66250  4.00815  3.04244  2.47682  4.18179  3.20974  3.64222
3.59961  1.63584  3.05458  3.97973  2.92160  3.62224  2.69135  2.61666
2.61357  2.89075  3.26053  5.38075  4.05382    367 k - - -
          2.68618  4.42225  2.77519  2.73123  3.46354  2.40513  3.72494
3.29354  2.67741  2.69355  4.24690  2.90347  2.73739  3.18146  2.89801
2.37887  2.77519  2.98518  4.58477  3.61503
          0.02276  4.18974  4.91209  0.61958  0.77255  0.48576  0.95510
    166   2.71447  5.19413  2.42430  1.80992  4.52777  3.39927  3.63235
4.00156  1.91859  3.48560  4.24870  2.86780  3.62933  2.74388  2.52812
2.66993  2.94882  3.58081  5.62054  4.21932    368 e - - -
          2.68618  4.42225  2.77519  2.73123  3.46354  2.40513  3.72494
3.29354  2.67741  2.69355  4.24690  2.90347  2.73739  3.18146  2.89801
2.37887  2.77519  2.98518  4.58477  3.61503
          0.02276  4.18974  4.91209  0.61958  0.77255  0.48576  0.95510
    167   2.65123  5.03846  2.29351  1.88576  4.33512  3.08241  3.62909
3.78804  2.40863  3.32604  3.87074  2.78856  3.58183  2.75008  2.89774
2.63360  2.88761  2.82532  5.50803  4.12247    369 e - - -
          2.68618  4.42225  2.77519  2.73123  3.46354  2.40513  3.72494
3.29354  2.67741  2.69355  4.24690  2.90347  2.73739  3.18146  2.89801
2.37887  2.77519  2.98518  4.58477  3.61503
          0.02276  4.18974  4.91209  0.61958  0.77255  0.48576  0.95510
    168   2.62668  4.84713  2.90163  2.47444  4.27951  2.05441  3.73269
3.71403  2.52370  3.29158  4.09573  2.57677  3.83966  2.73654  2.82871
2.19361  2.68725  3.33356  5.50181  4.15853    370 g - - -
          2.68618  4.42225  2.77519  2.73123  3.46354  2.40513  3.72494
3.29354  2.67741  2.69355  4.24690  2.90347  2.73739  3.18146  2.89801
2.37887  2.77519  2.98518  4.58477  3.61503
          0.20556  4.18974  1.76808  0.61958  0.77255  0.48576  0.95510
    169   2.64152  5.16879  2.30131  1.63920  4.48571  3.08195  3.66095
3.95811  2.52260  3.48166  4.27837  2.48991  3.79968  2.79872  3.04322
2.58830  2.71164  3.55563  5.65811  4.23492    371 e - - -
          2.68622  4.42229  2.77503  2.73127  3.46358  2.40511  3.72498
3.29358  2.67744  2.69359  4.24693  2.90350  2.73724  3.18150  2.89804
2.37885  2.77523  2.98514  4.58481  3.61507
          0.32343  1.31848  4.73379  0.22104  1.61790  0.67207  0.71468
```

170 2.42785 4.78922 2.06844 2.39716 4.08654 3.35120 3.63972
3.51188 2.45623 3.11175 3.92551 2.91551 3.58927 2.79302 2.92300
2.36829 2.71675 3.17140 4.47542 4.00194 373 d - - -
2.68618 4.42225 2.77519 2.73123 3.46354 2.40513 3.72494
3.29354 2.67741 2.69355 4.24690 2.90347 2.73739 3.18146 2.89801
2.37887 2.77519 2.98518 4.58477 3.61503
0.02727 4.01145 4.73379 0.61958 0.77255 0.67207 0.71468
171 2.60140 4.35538 3.41948 2.86261 3.51671 3.53572 3.78910
2.82999 2.58293 1.83565 3.46873 3.08425 3.93102 3.08963 2.84375
2.80598 2.68543 2.44137 4.94304 3.70768 374 l - - -
2.68618 4.42225 2.77519 2.73123 3.46354 2.40513 3.72494
3.29354 2.67741 2.69355 4.24690 2.90347 2.73739 3.18146 2.89801
2.37887 2.77519 2.98518 4.58477 3.61503
0.14998 4.01145 2.11059 0.61958 0.77255 0.67207 0.71468
172 2.59019 4.76546 2.45979 2.34769 4.28711 3.17057 3.75111
3.72580 2.63501 3.34621 4.18689 1.71889 3.76080 2.93380 3.08845
2.53058 2.60111 3.32971 5.55125 4.18606 375 n - - -
2.68627 4.42234 2.77529 2.73132 3.46363 2.40517 3.72488
3.29341 2.67719 2.69360 4.24699 2.90348 2.73749 3.18124 2.89779
2.37896 2.77525 2.98496 4.58486 3.61512
0.51068 1.03182 3.13364 0.67504 0.71159 0.47810 0.96747
173 2.06253 4.31406 3.41151 2.85397 3.49606 3.23449 3.80647
2.83712 2.80160 2.48097 2.74600 3.26007 3.90772 2.97010 3.16137
2.68048 2.64402 2.45399 4.93160 3.69302 378 a - - -
2.68618 4.42225 2.77519 2.73123 3.46354 2.40513 3.72494
3.29354 2.67741 2.69355 4.24690 2.90347 2.73739 3.18146 2.89801
2.37887 2.77519 2.98518 4.58477 3.61503
0.04556 4.06302 3.59921 0.61958 0.77255 0.62488 0.76642
174 2.50218 4.33487 3.37067 2.81038 2.88952 3.52050 3.77232
2.69482 2.76576 2.47796 3.43830 3.23283 2.73381 2.94459 3.13430
2.48174 2.80067 2.61238 4.90166 3.50631 379 l - - -
2.68618 4.42225 2.77519 2.73123 3.46354 2.40513 3.72494
3.29354 2.67741 2.69355 4.24690 2.90347 2.73739 3.18146 2.89801
2.37887 2.77519 2.98518 4.58477 3.61503
0.02639 4.04385 4.76619 0.61958 0.77255 0.64297 0.74597
175 2.54035 4.91905 2.80228 2.34839 4.21008 2.85253 3.59457
3.65060 2.17355 3.12288 3.99573 2.56255 3.40885 2.63018 2.83867
2.40180 2.83157 3.14202 5.40726 4.04201 380 k - - -
2.68618 4.42225 2.77519 2.73123 3.46354 2.40513 3.72494
3.29354 2.67741 2.69355 4.24690 2.90347 2.73739 3.18146 2.89801
2.37887 2.77519 2.98518 4.58477 3.61503
0.14090 4.04385 2.17252 0.61958 0.77255 0.64297 0.74597
176 2.58505 4.32744 2.48281 2.21876 4.24203 3.34248 3.56374
3.69026 2.10646 3.23309 4.01425 2.75238 3.26627 2.59349 2.80764
2.50170 2.81956 3.31347 5.41914 4.04329 381 k - - -
2.68618 4.42225 2.77519 2.73123 3.46354 2.40513 3.72494
3.29354 2.67741 2.69355 4.24690 2.90347 2.73739 3.18146 2.89801
2.37887 2.77519 2.98518 4.58477 3.61503
0.06037 3.93249 3.24447 0.61958 0.77255 0.56581 0.83910
177 2.30230 4.62368 3.02307 2.46529 3.91628 2.49452 3.67850
3.31631 2.43843 2.95375 3.78929 3.00129 3.59450 2.86118 2.95412
2.38146 2.70418 2.89342 5.22889 3.73572 382 a - - -
2.68618 4.42225 2.77519 2.73123 3.46354 2.40513 3.72494
3.29354 2.67741 2.69355 4.24690 2.90347 2.73739 3.18146 2.89801
2.37887 2.77519 2.98518 4.58477 3.61503
0.17375 4.01713 1.95551 0.61958 0.77255 0.66710 0.71989

178 2.52532 4.80205 2.72959 2.36475 4.08402 3.34348 2.94191
3.50918 2.36006 3.09601 3.90902 2.88405 2.95909 2.73875 2.59032
2.54417 2.71126 3.17074 5.31796 3.97900 383 k - - -
2.68586 4.42258 2.77476 2.73111 3.46387 2.40502 3.72480
3.29377 2.67738 2.69351 4.24642 2.90345 2.73769 3.18123 2.89829
2.37898 2.77546 2.98537 4.58396 3.61522
0.49443 0.96758 4.59705 1.72755 0.19567 0.36625 1.18198
179 2.56307 4.76416 3.03716 2.48467 4.00873 3.44153 3.65943
3.26186 2.32741 2.96861 3.85403 2.99484 2.65848 2.52086 2.90550
2.40294 2.55613 2.96775 5.28680 3.96437 404 k - - -
2.68618 4.42225 2.77519 2.73123 3.46354 2.40513 3.72494
3.29354 2.67741 2.69355 4.24690 2.90347 2.73739 3.18146 2.89801
2.37887 2.77519 2.98518 4.58477 3.61503
0.02325 4.16893 4.89128 0.61958 0.77255 0.51126 0.91563
180 2.56767 4.82143 3.12326 2.54991 4.09865 3.11062 3.66603
3.49150 2.14315 2.98767 3.92151 3.04299 3.87997 2.82165 1.89489
2.72041 2.77549 2.82462 5.32410 4.02463 405 r - - -
2.68618 4.42225 2.77519 2.73123 3.46354 2.40513 3.72494
3.29354 2.67741 2.69355 4.24690 2.90347 2.73739 3.18146 2.89801
2.37887 2.77519 2.98518 4.58477 3.61503
0.02325 4.16893 4.89128 0.61958 0.77255 0.47014 0.98060
181 2.34687 4.59002 3.16916 2.60840 3.64505 3.49024 3.71406
2.91821 2.52672 2.82749 3.48274 2.95713 3.64474 2.92338 2.54548
2.21029 2.83768 2.68895 5.13506 3.84864 406 s - - -
2.68618 4.42225 2.77519 2.73123 3.46354 2.40513 3.72494
3.29354 2.67741 2.69355 4.24690 2.90347 2.73739 3.18146 2.89801
2.37887 2.77519 2.98518 4.58477 3.61503
0.02276 4.18974 4.91209 0.61958 0.77255 0.48576 0.95510
182 3.18153 5.32309 3.63252 2.89939 4.80007 3.79350 3.39978
4.14046 1.29468 3.54028 4.40197 3.29325 4.13655 2.79885 1.40134
3.16120 3.32721 3.81541 5.54787 4.39328 407 k - - -
2.68618 4.42225 2.77519 2.73123 3.46354 2.40513 3.72494
3.29354 2.67741 2.69355 4.24690 2.90347 2.73739 3.18146 2.89801
2.37887 2.77519 2.98518 4.58477 3.61503
0.02276 4.18974 4.91209 0.61958 0.77255 0.48576 0.95510
183 2.23261 4.64142 3.10901 2.59241 4.05812 2.42075 3.54910
3.47012 2.59808 3.09553 3.91807 2.92476 3.17280 2.93702 3.04081
2.14386 2.75449 2.83684 5.35359 4.04506 408 s - - -
2.68618 4.42225 2.77519 2.73123 3.46354 2.40513 3.72494
3.29354 2.67741 2.69355 4.24690 2.90347 2.73739 3.18146 2.89801
2.37887 2.77519 2.98518 4.58477 3.61503
0.02276 4.18974 4.91209 0.61958 0.77255 0.48576 0.95510
184 2.52490 5.11763 2.21156 1.99006 4.43819 3.15800 3.67374
3.90018 2.48769 3.43042 4.21300 2.86519 2.38024 2.80089 2.99077
2.67669 2.95547 3.28161 5.60757 4.20537 409 e - - -
2.68618 4.42225 2.77519 2.73123 3.46354 2.40513 3.72494
3.29354 2.67741 2.69355 4.24690 2.90347 2.73739 3.18146 2.89801
2.37887 2.77519 2.98518 4.58477 3.61503
0.02276 4.18974 4.91209 0.61958 0.77255 0.48576 0.95510
185 2.84670 4.80796 3.45344 2.80765 3.94563 3.65382 3.70854
3.41425 1.76444 2.79765 3.89286 3.23586 4.01759 2.90709 2.14629
2.93165 3.04825 3.14996 2.36168 3.92161 410 k - - -
2.68618 4.42225 2.77519 2.73123 3.46354 2.40513 3.72494
3.29354 2.67741 2.69355 4.24690 2.90347 2.73739 3.18146 2.89801
2.37887 2.77519 2.98518 4.58477 3.61503
0.02276 4.18974 4.91209 0.61958 0.77255 0.48576 0.95510

186 2.87094 5.25356 2.26589 2.40609 4.60261 3.44900 3.71322
4.05510 1.29477 3.55071 4.36214 2.94078 3.92435 2.84673 2.68214
2.82702 3.10826 3.66656 5.67240 4.31494 411 k - - -
2.68618 4.42225 2.77519 2.73123 3.46354 2.40513 3.72494
3.29354 2.67741 2.69355 4.24690 2.90347 2.73739 3.18146 2.89801
2.37887 2.77519 2.98518 4.58477 3.61503
0.02276 4.18974 4.91209 0.61958 0.77255 0.48576 0.95510
187 2.50155 5.00415 2.69774 2.35813 4.32010 2.91712 3.45373
3.77298 2.36029 3.31265 4.08921 2.47153 3.47605 2.75221 2.89230
2.15908 2.58331 3.38675 5.49595 4.11547 412 s - - -
2.68618 4.42225 2.77519 2.73123 3.46354 2.40513 3.72494
3.29354 2.67741 2.69355 4.24690 2.90347 2.73739 3.18146 2.89801
2.37887 2.77519 2.98518 4.58477 3.61503
0.02276 4.18974 4.91209 0.61958 0.77255 0.48576 0.95510
188 2.41799 4.38639 3.38631 2.76239 3.53277 3.55547 3.45297
2.66158 2.77356 2.60027 3.28364 3.25095 3.93656 2.31812 3.14787
2.79320 2.76850 2.24370 4.27650 3.71223 413 v - - -
2.68618 4.42225 2.77519 2.73123 3.46354 2.40513 3.72494
3.29354 2.67741 2.69355 4.24690 2.90347 2.73739 3.18146 2.89801
2.37887 2.77519 2.98518 4.58477 3.61503
0.02276 4.18974 4.91209 0.61958 0.77255 0.48576 0.95510
189 2.96692 5.56305 1.57096 1.69902 4.86368 2.31039 3.80854
4.38533 2.78027 3.86183 4.67976 2.47097 3.90908 2.95899 3.36792
2.85879 3.24362 3.94215 6.01462 4.51230 414 d - - -
2.68619 4.42226 2.77520 2.73124 3.46355 2.40513 3.72495
3.29355 2.67741 2.69356 4.24647 2.90347 2.73740 3.18147 2.89801
2.37887 2.77517 2.98519 4.58478 3.61504
0.08440 2.79486 3.92146 0.44201 1.02929 0.48576 0.95510
190 2.82705 4.39842 4.65194 4.08016 3.27126 4.26129 4.61304
2.01425 3.95709 1.48021 2.11152 4.28696 4.54750 4.12881 4.10268
3.58294 3.26321 1.65437 5.11282 3.98244 416 l - - -
2.68618 4.42225 2.77519 2.73123 3.46354 2.40513 3.72494
3.29354 2.67741 2.69355 4.24690 2.90347 2.73739 3.18146 2.89801
2.37887 2.77519 2.98518 4.58477 3.61503
0.02305 4.17721 4.89955 0.61958 0.77255 0.47619 0.97060
191 2.69235 4.27830 3.89382 3.34529 3.46447 2.23107 4.13865
2.25311 3.25844 2.39358 3.07724 3.65377 4.14480 3.54764 3.54142
3.03030 2.95694 1.61613 5.02232 3.80766 417 v - - -
2.68618 4.42225 2.77520 2.73124 3.46354 2.40513 3.72495
3.29354 2.67741 2.69355 4.24690 2.90347 2.73740 3.18147 2.89797
2.37883 2.77520 2.98519 4.58477 3.61504
0.05766 3.02267 4.91209 0.47901 0.96600 0.48576 0.95510
192 3.36795 4.65166 5.06750 4.54502 3.32368 4.70704 5.10446
1.19942 4.38478 1.22871 3.12714 4.77049 4.91167 4.54671 4.50652
4.09323 3.60676 1.98088 5.40640 4.27784 419 i - - -
2.68618 4.42225 2.77519 2.73123 3.46354 2.40513 3.72494
3.29354 2.67741 2.69355 4.24690 2.90347 2.73739 3.18146 2.89801
2.37887 2.77519 2.98518 4.58477 3.61503
0.02276 4.18974 4.91209 0.61958 0.77255 0.48576 0.95510
193 2.94132 5.30541 2.31726 2.32016 4.65424 3.34825 3.85120
4.16657 2.55965 3.71021 4.56069 1.16946 3.94112 3.02309 3.21791
2.88679 3.23446 3.76734 5.85273 4.42434 420 n - - -
2.68618 4.42225 2.77519 2.73123 3.46354 2.40513 3.72494
3.29354 2.67741 2.69355 4.24690 2.90347 2.73739 3.18146 2.89801
2.37887 2.77519 2.98518 4.58477 3.61503
0.02276 4.18974 4.91209 0.61958 0.77255 0.48576 0.95510

```
    194   2.70010  4.80156  3.04352  2.58130  4.08508  1.97035  3.73786
3.50566  2.46697  2.65771  3.96434  3.06752  3.90479  2.21574  2.77164
2.75642  2.95304  3.19420  5.35391  4.04520    421 g - - -
          2.68618  4.42225  2.77519  2.73123  3.46354  2.40513  3.72494
3.29354  2.67741  2.69355  4.24690  2.90347  2.73739  3.18146  2.89801
2.37887  2.77519  2.98518  4.58477  3.61503
          0.02276  4.18974  4.91209  0.61958  0.77255  0.48576  0.95510
    195   3.22875  4.50166  5.06498  4.58041  3.70835  4.65476  5.28302
1.08310  4.47781  2.18099  3.49190  4.77261  4.94231  4.73136  4.65764
4.05983  3.49433  1.18847  5.72475  4.52094    422 i - - -
          2.68618  4.42225  2.77519  2.73123  3.46354  2.40513  3.72494
3.29354  2.67741  2.69355  4.24690  2.90347  2.73739  3.18146  2.89801
2.37887  2.77519  2.98518  4.58477  3.61503
          0.02276  4.18974  4.91209  0.61958  0.77255  0.48576  0.95510
    196   2.35346  5.04364  2.26713  2.23713  4.34795  3.39468  3.62081
3.80571  2.39347  3.33557  4.10708  2.54132  3.54330  2.65570  2.88291
2.54180  2.64363  3.09406  5.51094  4.12292    423 e - - -
          2.68618  4.42225  2.77519  2.73123  3.46354  2.40513  3.72494
3.29354  2.67741  2.69355  4.24690  2.90347  2.73739  3.18146  2.89801
2.37887  2.77519  2.98518  4.58477  3.61503
          0.02276  4.18974  4.91209  0.61958  0.77255  0.48576  0.95510
    197   2.92442  4.31718  4.53754  3.95942  1.96145  4.06813  4.24702
2.44596  3.81351  1.53808  2.34963  4.09162  4.37737  3.94854  3.91634
3.37661  3.15012  2.31011  4.67891  2.84396    424 l - - -
          2.68618  4.42225  2.77519  2.73123  3.46354  2.40513  3.72494
3.29354  2.67741  2.69355  4.24690  2.90347  2.73739  3.18146  2.89801
2.37887  2.77519  2.98518  4.58477  3.61503
          0.02276  4.18974  4.91209  0.61958  0.77255  0.48576  0.95510
    198   3.36609  5.50581  0.55193  2.59278  4.93377  3.48351  4.25659
4.60188  3.40745  4.15709  5.15381  3.16092  4.15931  3.50422  3.95815
3.30035  3.71526  4.21066  6.03613  4.76917    425 D - - -
          2.68618  4.42225  2.77519  2.73123  3.46354  2.40513  3.72494
3.29354  2.67741  2.69355  4.24690  2.90347  2.73739  3.18146  2.89801
2.37887  2.77519  2.98518  4.58477  3.61503
          0.02276  4.18974  4.91209  0.61958  0.77255  0.48576  0.95510
    199   2.67104  4.58346  2.99031  2.02708  3.74691  3.54947  3.80040
2.25861  2.69430  2.25760  3.65654  3.16757  3.94864  3.02944  3.10720
2.80424  2.90587  2.72879  5.15950  3.87979    426 e - - -
          2.68618  4.42225  2.77519  2.73123  3.46354  2.40513  3.72494
3.29354  2.67741  2.69355  4.24690  2.90347  2.73739  3.18146  2.89801
2.37887  2.77519  2.98518  4.58477  3.61503
          0.02276  4.18974  4.91209  0.61958  0.77255  0.48576  0.95510
    200   1.99208  4.26856  3.59381  3.24006  4.36599  3.06303  4.28742
3.73215  3.25126  3.44782  4.27620  3.40327  3.79579  3.54837  3.57515
1.20253  2.01141  3.21465  5.71053  4.49732    427 s - - -
          2.68618  4.42225  2.77519  2.73123  3.46354  2.40513  3.72494
3.29354  2.67741  2.69355  4.24690  2.90347  2.73739  3.18146  2.89801
2.37887  2.77519  2.98518  4.58477  3.61503
          0.02276  4.18974  4.91209  0.61958  0.77255  0.48576  0.95510
    201   2.59723  4.63603  3.15621  2.60510  3.91187  2.93068  3.72722
3.18340  2.54847  2.94937  3.79146  2.96369  3.86615  2.91735  2.37947
2.61635  2.08359  2.72198  5.23249  3.93957    428 t - - -
          2.68618  4.42225  2.77519  2.73123  3.46354  2.40513  3.72494
3.29354  2.67741  2.69355  4.24690  2.90347  2.73739  3.18146  2.89801
2.37887  2.77519  2.98518  4.58477  3.61503
          0.02276  4.18974  4.91209  0.61958  0.77255  0.48576  0.95510
```

202 3.27028 4.58787 4.93521 4.37844 3.26847 4.56103 4.91946
1.33935 4.22718 1.62506 1.81541 4.60189 4.78304 4.37870 4.35193
3.91235 3.50185 2.07374 5.28531 4.18292 429 i - - -
2.68618 4.42225 2.77519 2.73123 3.46354 2.40513 3.72494
3.29354 2.67741 2.69355 4.24690 2.90347 2.73739 3.18146 2.89801
2.37887 2.77519 2.98518 4.58477 3.61503
0.02276 4.18974 4.91209 0.61958 0.77255 0.48576 0.95510
203 3.23048 4.92166 3.87360 3.76678 4.71542 3.59185 4.80739
4.35653 3.85409 3.94265 5.01369 4.03612 0.40452 4.23248 4.05300
3.41044 3.69887 3.96295 5.80162 4.84404 430 P - - -
2.68618 4.42225 2.77519 2.73123 3.46354 2.40513 3.72494
3.29354 2.67741 2.69355 4.24690 2.90347 2.73739 3.18146 2.89801
2.37887 2.77519 2.98518 4.58477 3.61503
0.02276 4.18974 4.91209 0.61958 0.77255 0.48576 0.95510
204 2.21351 4.24797 3.76449 3.21167 3.46810 3.61722 4.03325
2.06926 3.14315 2.48199 3.42820 3.53238 3.40126 3.42784 3.44374
2.91330 2.18549 2.05816 4.97035 3.75474 431 v - - -
2.68618 4.42225 2.77519 2.73123 3.46354 2.40513 3.72494
3.29354 2.67741 2.69355 4.24690 2.90347 2.73739 3.18146 2.89801
2.37887 2.77519 2.98518 4.58477 3.61503
0.02276 4.18974 4.91209 0.61958 0.77255 0.48576 0.95510
205 2.86912 4.75274 3.42643 3.19276 4.42174 3.42508 4.29484
3.96722 3.08528 3.56722 4.52293 3.53398 0.77528 3.57346 2.97116
3.00580 3.27899 3.57888 5.65031 4.46769 432 p - - -
2.68618 4.42225 2.77519 2.73123 3.46354 2.40513 3.72494
3.29354 2.67741 2.69355 4.24690 2.90347 2.73739 3.18146 2.89801
2.37887 2.77519 2.98518 4.58477 3.61503
0.02276 4.18974 4.91209 0.61958 0.77255 0.48576 0.95510
206 2.91275 4.43434 4.82086 4.29971 3.66236 4.45153 4.99131
1.61364 4.19340 2.22629 3.26865 4.51460 4.76768 4.45237 4.39332
3.81753 3.37031 0.93838 5.55193 4.35460 433 v - - -
2.68618 4.42225 2.77519 2.73123 3.46354 2.40513 3.72494
3.29354 2.67741 2.69355 4.24690 2.90347 2.73739 3.18146 2.89801
2.37887 2.77519 2.98518 4.58477 3.61503
0.02276 4.18974 4.91209 0.61958 0.77255 0.48576 0.95510
207 3.00446 0.47463 4.57797 4.39845 4.33290 3.54955 4.97748
3.54328 4.23287 3.43754 4.57307 4.30358 4.27949 4.56612 4.29641
3.27500 3.49855 3.26274 5.66619 4.61484 434 C - - -
2.68618 4.42225 2.77519 2.73123 3.46354 2.40513 3.72494
3.29354 2.67741 2.69355 4.24690 2.90347 2.73739 3.18146 2.89801
2.37887 2.77519 2.98518 4.58477 3.61503
0.02276 4.18974 4.91209 0.61958 0.77255 0.48576 0.95510
208 2.34973 4.29567 3.52912 3.25877 4.41749 3.06355 4.33567
3.79862 3.29132 3.52515 4.37283 3.41648 3.81500 3.60754 3.59311
0.96772 2.22264 3.26783 5.76769 4.53956 435 s - - -
2.68618 4.42225 2.77519 2.73123 3.46354 2.40513 3.72494
3.29354 2.67741 2.69355 4.24690 2.90347 2.73739 3.18146 2.89801
2.37887 2.77519 2.98518 4.58477 3.61503
0.02276 4.18974 4.91209 0.61958 0.77255 0.48576 0.95510
209 3.00446 0.47463 4.57797 4.39845 4.33290 3.54955 4.97748
3.54328 4.23287 3.43754 4.57307 4.30358 4.27949 4.56612 4.29641
3.27500 3.49855 3.26274 5.66619 4.61484 436 C - - -
2.68618 4.42225 2.77519 2.73123 3.46354 2.40513 3.72494
3.29354 2.67741 2.69355 4.24690 2.90347 2.73739 3.18146 2.89801
2.37887 2.77519 2.98518 4.58477 3.61503
0.02276 4.18974 4.91209 0.61958 0.77255 0.48576 0.95510

210 2.61901 4.46068 3.74951 3.53189 4.28867 3.28571 4.51986
3.45688 3.48136 3.30855 4.34770 3.67846 4.01883 3.86229 3.71597
2.82506 0.72910 3.11534 5.69655 4.50903 437 t - - -
2.68618 4.42225 2.77519 2.73123 3.46354 2.40513 3.72494
3.29354 2.67741 2.69355 4.24690 2.90347 2.73739 3.18146 2.89801
2.37887 2.77519 2.98518 4.58477 3.61503
0.02276 4.18974 4.91209 0.61958 0.77255 0.48576 0.95510
211 3.10928 4.84088 3.84696 3.79231 4.90541 0.37316 4.88115
4.59416 4.01605 4.20373 5.19050 4.01276 4.24101 4.31570 4.20587
3.29211 3.61492 4.06091 5.90463 5.02212 438 G - - -
2.68618 4.42225 2.77519 2.73123 3.46354 2.40513 3.72494
3.29354 2.67741 2.69355 4.24690 2.90347 2.73739 3.18146 2.89801
2.37887 2.77519 2.98518 4.58477 3.61503
0.02276 4.18974 4.91209 0.61958 0.77255 0.48576 0.95510
212 2.06789 4.41311 3.35785 2.80139 3.59709 3.52391 3.80354
2.76698 2.66508 2.65979 3.54047 2.97265 3.92541 3.08247 3.03074
2.63508 2.64378 2.14081 5.01167 3.76118 439 a - - -
2.68618 4.42225 2.77519 2.73123 3.46354 2.40513 3.72494
3.29354 2.67741 2.69355 4.24690 2.90347 2.73739 3.18146 2.89801
2.37887 2.77519 2.98518 4.58477 3.61503
0.02276 4.18974 4.91209 0.61958 0.77255 0.48576 0.95510
213 2.38667 4.40282 3.38124 2.82565 3.31630 3.55012 3.81054
2.91004 2.78023 2.26579 3.50451 3.25537 2.28700 2.98978 3.15464
2.79623 2.70946 2.67897 4.97924 3.54461 440 l - - -
2.68618 4.42225 2.77519 2.73123 3.46354 2.40513 3.72494
3.29354 2.67741 2.69355 4.24690 2.90347 2.73739 3.18146 2.89801
2.37887 2.77519 2.98518 4.58477 3.61503
0.02276 4.18974 4.91209 0.61958 0.77255 0.48576 0.95510
214 3.01494 5.21373 3.31059 2.72576 4.56886 3.66425 3.48307
4.00120 2.05123 3.45671 4.30366 3.17392 4.04671 1.61091 1.65110
3.00226 3.20059 3.66465 5.51215 4.27131 441 q - - -
2.68618 4.42225 2.77519 2.73123 3.46354 2.40513 3.72494
3.29354 2.67741 2.69355 4.24690 2.90347 2.73739 3.18146 2.89801
2.37887 2.77519 2.98518 4.58477 3.61503
0.02276 4.18974 4.91209 0.61958 0.77255 0.48576 0.95510
215 2.97142 5.05719 2.98305 2.72747 4.40995 3.49417 3.94003
3.97475 2.58443 3.48562 4.42476 3.19914 3.65566 0.98288 2.89022
3.00205 3.27134 3.63823 5.60549 4.30379 442 q - - -
2.68618 4.42225 2.77519 2.73123 3.46354 2.40513 3.72494
3.29354 2.67741 2.69355 4.24690 2.90347 2.73739 3.18146 2.89801
2.37887 2.77519 2.98518 4.58477 3.61503
0.02276 4.18974 4.91209 0.61958 0.77255 0.48576 0.95510
216 2.69024 0.80668 4.20602 3.85631 4.00394 3.39745 4.52723
3.30473 3.53289 3.11714 4.14039 3.88122 4.09161 3.97609 3.19807
2.94045 3.13854 3.01083 5.43376 4.24337 443 c - - -
2.68618 4.42225 2.77519 2.73123 3.46354 2.40513 3.72494
3.29354 2.67741 2.69355 4.24690 2.90347 2.73739 3.18146 2.89801
2.37887 2.77519 2.98518 4.58477 3.61503
0.02276 4.18974 4.91209 0.61958 0.77255 0.48576 0.95510
217 3.73811 5.09692 4.32336 4.12258 2.38102 4.21286 3.81560
3.70761 3.97223 3.07644 4.35081 4.13704 4.68394 4.19889 4.08572
3.83835 4.02896 3.59442 4.03324 0.56668 444 Y - - -
2.68618 4.42225 2.77519 2.73123 3.46354 2.40513 3.72494
3.29354 2.67741 2.69355 4.24690 2.90347 2.73739 3.18146 2.89801
2.37887 2.77519 2.98518 4.58477 3.61503
0.02276 4.18974 4.91209 0.61958 0.77255 0.48576 0.95510

218 3.23378 5.33270 3.71069 2.96490 4.84643 3.81915 3.69371
4.16546 1.28198 3.56158 4.43799 3.33967 4.16761 2.82332 1.30844
3.21819 3.37679 3.84783 5.56194 4.43210 445 k - - -
2.68618 4.42225 2.77519 2.73123 3.46354 2.40513 3.72494
3.29354 2.67741 2.69355 4.24690 2.90347 2.73739 3.18146 2.89801
2.37887 2.77519 2.98518 4.58477 3.61503
0.02276 4.18974 4.91209 0.61958 0.77255 0.48576 0.95510
219 3.91781 5.16875 4.61085 4.40384 3.06619 4.11587 4.34908
3.96967 4.12769 3.30416 4.58623 4.49298 4.66201 4.49800 4.19578
4.11818 4.23697 3.86801 0.43253 3.05368 446 W - - -
2.68618 4.42225 2.77519 2.73123 3.46354 2.40513 3.72494
3.29354 2.67741 2.69355 4.24690 2.90347 2.73739 3.18146 2.89801
2.37887 2.77519 2.98518 4.58477 3.61503
0.02276 4.18974 4.91209 0.61958 0.77255 0.48576 0.95510
220 3.10928 4.84088 3.84696 3.79231 4.90541 0.37316 4.88115
4.59416 4.01605 4.20373 5.19050 4.01276 4.24101 4.31570 4.20587
3.29211 3.61492 4.06091 5.90463 5.02212 447 G - - -
2.68618 4.42225 2.77519 2.73123 3.46354 2.40513 3.72494
3.29354 2.67741 2.69355 4.24690 2.90347 2.73739 3.18146 2.89801
2.37887 2.77519 2.98518 4.58477 3.61503
0.02276 4.18974 4.91209 0.61958 0.77255 0.48576 0.95510
221 1.88361 2.66962 3.41230 2.92681 4.00912 3.23868 3.98862
3.40016 2.91138 3.07751 3.92200 2.05474 3.50803 3.22909 3.29324
2.32042 2.80849 3.02906 5.37033 4.11882 448 a - - -
2.68618 4.42225 2.77519 2.73123 3.46354 2.40513 3.72494
3.29354 2.67741 2.69355 4.24690 2.90347 2.73739 3.18146 2.89801
2.37887 2.77519 2.98518 4.58477 3.61503
0.02276 4.18974 4.91209 0.61958 0.77255 0.48576 0.95510
222 3.10928 4.84088 3.84696 3.79231 4.90541 0.37316 4.88115
4.59416 4.01605 4.20373 5.19050 4.01276 4.24101 4.31570 4.20587
3.29211 3.61492 4.06091 5.90463 5.02212 449 G - - -
2.68618 4.42225 2.77519 2.73123 3.46354 2.40513 3.72494
3.29354 2.67741 2.69355 4.24690 2.90347 2.73739 3.18146 2.89801
2.37887 2.77519 2.98518 4.58477 3.61503
0.02276 4.18974 4.91209 0.61958 0.77255 0.48576 0.95510
223 3.10928 4.84088 3.84696 3.79231 4.90541 0.37316 4.88115
4.59416 4.01605 4.20373 5.19050 4.01276 4.24101 4.31570 4.20587
3.29211 3.61492 4.06091 5.90463 5.02212 450 G - - -
2.68618 4.42225 2.77519 2.73123 3.46354 2.40513 3.72494
3.29354 2.67741 2.69355 4.24690 2.90347 2.73739 3.18146 2.89801
2.37887 2.77519 2.98518 4.58477 3.61503
0.02276 4.18974 4.91209 0.61958 0.77255 0.48576 0.95510
224 3.91781 5.16875 4.61085 4.40384 3.06619 4.11587 4.34908
3.96967 4.12769 3.30416 4.58623 4.49298 4.66201 4.49800 4.19578
4.11818 4.23697 3.86801 0.43253 3.05368 451 W - - -
2.68618 4.42225 2.77519 2.73123 3.46354 2.40513 3.72494
3.29354 2.67741 2.69355 4.24690 2.90347 2.73739 3.18146 2.89801
2.37887 2.77519 2.98518 4.58477 3.61503
0.02276 4.18974 4.91209 0.61958 0.77255 0.48576 0.95510
225 3.18041 5.14900 3.15922 2.93216 4.35873 3.60725 4.08857
4.05223 2.73330 3.52094 4.55241 3.38999 4.17320 0.75945 3.00559
3.22054 3.48305 3.76551 5.57832 4.29970 452 q - - -
2.68618 4.42225 2.77519 2.73123 3.46354 2.40513 3.72494
3.29354 2.67741 2.69355 4.24690 2.90347 2.73739 3.18146 2.89801
2.37887 2.77519 2.98518 4.58477 3.61503
0.02276 4.18974 4.91209 0.61958 0.77255 0.48576 0.95510

```
    226   2.50280  4.42099  3.51846  3.36194  4.41314  3.14677  4.45600
3.98555  3.45818  3.68493  4.59140  3.52517  3.91427  3.78365  3.72501
0.69684  3.00893  3.44828  5.76162  4.50227    453 s - - -
          2.68618  4.42225  2.77519  2.73123  3.46354  2.40513  3.72494
3.29354  2.67741  2.69355  4.24690  2.90347  2.73739  3.18146  2.89801
2.37887  2.77519  2.98518  4.58477  3.61503
          0.02276  4.18974  4.91209  0.61958  0.77255  0.48576  0.95510
    227   1.10966  4.23979  3.64558  3.36731  4.42423  3.03176  4.39989
3.79394  3.40823  3.53033  4.36794  3.46058  3.79830  3.68868  3.69755
1.56475  2.80953  3.24544  5.78424  4.58072    454 a - - -
          2.68618  4.42225  2.77519  2.73123  3.46354  2.40513  3.72494
3.29354  2.67741  2.69355  4.24690  2.90347  2.73739  3.18146  2.89801
2.37887  2.77519  2.98518  4.58477  3.61503
          0.02276  4.18974  4.91209  0.61958  0.77255  0.48576  0.95510
    228   3.00446  0.47463  4.57797  4.39845  4.33290  3.54955  4.97748
3.54328  4.23287  3.43754  4.57307  4.30358  4.27949  4.56612  4.29641
3.27500  3.49855  3.26274  5.66619  4.61484    455 C - - -
          2.68618  4.42225  2.77519  2.73123  3.46354  2.40513  3.72494
3.29354  2.67741  2.69355  4.24690  2.90347  2.73739  3.18146  2.89801
2.37887  2.77519  2.98518  4.58477  3.61503
          0.02276  4.18974  4.91209  0.61958  0.77255  0.48576  0.95510
    229   3.00446  0.47463  4.57797  4.39845  4.33290  3.54955  4.97748
3.54328  4.23287  3.43754  4.57307  4.30358  4.27949  4.56612  4.29641
3.27500  3.49855  3.26274  5.66619  4.61484    456 C - - -
          2.68618  4.42225  2.77519  2.73123  3.46354  2.40513  3.72494
3.29354  2.67741  2.69355  4.24690  2.90347  2.73739  3.18146  2.89801
2.37887  2.77519  2.98518  4.58477  3.61503
          0.02276  4.18974  4.91209  0.61958  0.77255  0.48576  0.95510
    230   2.61901  4.46068  3.74951  3.53189  4.28867  3.28571  4.51986
3.45688  3.48136  3.30855  4.34770  3.67846  4.01883  3.86229  3.71597
2.82506  0.72910  3.11534  5.69655  4.50903    457 t - - -
          2.68618  4.42225  2.77519  2.73123  3.46354  2.40513  3.72494
3.29354  2.67741  2.69355  4.24690  2.90347  2.73739  3.18146  2.89801
2.37887  2.77519  2.98518  4.58477  3.61503
          0.02276  4.18974  4.91209  0.61958  0.77255  0.48576  0.95510
    231   2.61869  4.40531  3.73201  3.29031  3.77988  3.45456  4.21767
2.82883  3.14217  2.63984  3.14332  3.56034  4.04230  3.52832  3.43070
2.85084  1.17759  2.62085  5.33597  4.09335    458 t - - -
          2.68618  4.42225  2.77519  2.73123  3.46354  2.40513  3.72494
3.29354  2.67741  2.69355  4.24690  2.90347  2.73739  3.18146  2.89801
2.37887  2.77519  2.98518  4.58477  3.61503
          0.02276  4.18974  4.91209  0.61958  0.77255  0.48576  0.95510
    232   2.59076  4.26726  3.10278  2.56561  3.94010  3.30748  3.71677
3.34180  2.55188  2.97960  3.81488  2.59060  3.85313  2.77592  2.98968
2.27397  2.02099  3.03535  5.25520  3.68083    459 t - - -
          2.68618  4.42225  2.77519  2.73123  3.46354  2.40513  3.72494
3.29354  2.67741  2.69355  4.24690  2.90347  2.73739  3.18146  2.89801
2.37887  2.77519  2.98518  4.58477  3.61503
          0.02276  4.18974  4.91209  0.61958  0.77255  0.48576  0.95510
    233   3.33970  4.62725  5.08608  4.52151  3.23711  4.67039  5.02530
1.43865  4.39509  1.13908  2.56336  4.73531  4.84965  4.47669  4.48467
4.02507  3.56440  2.03179  5.30761  4.25010    460 l - - -
          2.68618  4.42225  2.77519  2.73123  3.46354  2.40513  3.72494
3.29354  2.67741  2.69355  4.24690  2.90347  2.73739  3.18146  2.89801
2.37887  2.77519  2.98518  4.58477  3.61503
          0.02276  4.18974  4.91209  0.61958  0.77255  0.48576  0.95510
```

234 2.50280 4.42099 3.51846 3.36194 4.41314 3.14677 4.45600
3.98555 3.45818 3.68493 4.59140 3.52517 3.91427 3.78365 3.72501
0.69684 3.00893 3.44828 5.76162 4.50227 461 s - - -
2.68618 4.42225 2.77519 2.73123 3.46354 2.40513 3.72494
3.29354 2.67741 2.69355 4.24690 2.90347 2.73739 3.18146 2.89801
2.37887 2.77519 2.98518 4.58477 3.61503
0.02276 4.18974 4.91209 0.61958 0.77255 0.48576 0.95510
235 2.60090 4.36779 3.42212 2.76774 3.53600 3.54761 3.83087
2.87941 2.81315 2.59690 2.37643 3.28389 3.94684 3.03893 3.17397
2.47381 2.24185 2.44804 4.96904 3.72865 462 t - - -
2.68618 4.42225 2.77519 2.73123 3.46354 2.40513 3.72494
3.29354 2.67741 2.69355 4.24690 2.90347 2.73739 3.18146 2.89801
2.37887 2.77519 2.98518 4.58477 3.61503
0.02276 4.18974 4.91209 0.61958 0.77255 0.48576 0.95510
236 3.62735 5.05746 4.13993 3.84452 2.16403 4.26156 2.92194
3.69994 3.65291 3.05210 4.24677 3.90906 4.64381 3.91211 3.82427
3.67231 3.86312 3.55385 3.82295 0.76288 463 y - - -
2.68618 4.42225 2.77519 2.73123 3.46354 2.40513 3.72494
3.29354 2.67741 2.69355 4.24690 2.90347 2.73739 3.18146 2.89801
2.37887 2.77519 2.98518 4.58477 3.61503
0.02276 4.18974 4.91209 0.61958 0.77255 0.48576 0.95510
237 3.23048 4.92166 3.87360 3.76678 4.71542 3.59185 4.80739
4.35653 3.85409 3.94265 5.01369 4.03612 0.40452 4.23248 4.05300
3.41044 3.69887 3.96295 5.80162 4.84404 464 P - - -
2.68618 4.42225 2.77519 2.73123 3.46354 2.40513 3.72494
3.29354 2.67741 2.69355 4.24690 2.90347 2.73739 3.18146 2.89801
2.37887 2.77519 2.98518 4.58477 3.61503
0.02276 4.18974 4.91209 0.61958 0.77255 0.48576 0.95510
238 3.53202 4.90692 4.69853 4.35150 3.28922 4.38171 4.90937
2.52143 4.11692 0.56391 3.23347 4.59681 4.76983 4.40741 4.23619
4.00827 3.81578 2.63897 5.27097 4.06369 465 L - - -
2.68618 4.42225 2.77519 2.73123 3.46354 2.40513 3.72494
3.29354 2.67741 2.69355 4.24690 2.90347 2.73739 3.18146 2.89801
2.37887 2.77519 2.98518 4.58477 3.61503
0.02276 4.18974 4.91209 0.61958 0.77255 0.48576 0.95510
239 3.23048 4.92166 3.87360 3.76678 4.71542 3.59185 4.80739
4.35653 3.85409 3.94265 5.01369 4.03612 0.40452 4.23248 4.05300
3.41044 3.69887 3.96295 5.80162 4.84404 466 P - - -
2.68618 4.42225 2.77519 2.73123 3.46354 2.40513 3.72494
3.29354 2.67741 2.69355 4.24690 2.90347 2.73739 3.18146 2.89801
2.37887 2.77519 2.98518 4.58477 3.61503
0.02276 4.18974 4.91209 0.61958 0.77255 0.48576 0.95510
240 2.24049 4.39292 3.51967 2.97365 3.52464 3.59945 3.91004
2.80126 2.87414 2.47638 2.02453 3.37156 4.00678 2.65090 3.21059
2.87361 2.90264 2.45201 5.01178 3.78267 467 m - - -
2.68618 4.42225 2.77519 2.73123 3.46354 2.40513 3.72494
3.29354 2.67741 2.69355 4.24690 2.90347 2.73739 3.18146 2.89801
2.37887 2.77519 2.98518 4.58477 3.61503
0.02276 4.18974 4.91209 0.61958 0.77255 0.48576 0.95510
241 2.60508 4.39521 3.39949 2.84296 3.56058 3.54580 3.82458
2.89063 2.79360 2.28113 2.94509 2.73824 3.94436 3.11729 3.16545
2.08800 2.84717 2.42362 4.99263 3.74357 468 s - - -
2.68618 4.42225 2.77519 2.73123 3.46354 2.40513 3.72494
3.29354 2.67741 2.69355 4.24690 2.90347 2.73739 3.18146 2.89801
2.37887 2.77519 2.98518 4.58477 3.61503
0.02276 4.18974 4.91209 0.61958 0.77255 0.48576 0.95510

```
    242   2.58622  4.51622  3.27187  2.73717  3.79590  3.45082  3.80866
 3.13688  2.68278  2.82560  3.47914  3.17955  2.32013  2.88127  3.07713
 2.72117  2.04285  2.66542  5.17506  3.90617    469 t - - -
          2.68618  4.42225  2.77519  2.73123  3.46354  2.40513  3.72494
 3.29354  2.67741  2.69355  4.24690  2.90347  2.73739  3.18146  2.89801
 2.37887  2.77519  2.98518  4.58477  3.61503
          0.02276  4.18974  4.91209  0.61958  0.77255  0.48576  0.95510
    243   2.93572  5.21283  2.87725  2.53681  4.60701  3.50256  3.74380
 4.06023  1.42513  3.55589  4.39248  1.84646  3.98141  2.88712  2.61113
 2.91086  3.17500  3.68556  5.64230  4.33159    470 k - - -
          2.68618  4.42225  2.77519  2.73123  3.46354  2.40513  3.72494
 3.29354  2.67741  2.69355  4.24690  2.90347  2.73739  3.18146  2.89801
 2.37887  2.77519  2.98518  4.58477  3.61503
          0.02276  4.18974  4.91209  0.61958  0.77255  0.48576  0.95510
    244   3.09957  5.26254  3.51952  2.83639  4.73930  3.73485  3.67711
 4.08159  1.38339  3.50908  4.36174  3.25010  3.66783  2.80443  1.40830
 3.08655  3.26687  3.74737  5.54751  4.37169    471 k - - -
          2.68618  4.42225  2.77519  2.73123  3.46354  2.40513  3.72494
 3.29354  2.67741  2.69355  4.24690  2.90347  2.73739  3.18146  2.89801
 2.37887  2.77519  2.98518  4.58477  3.61503
          0.02276  4.18974  4.91209  0.61958  0.77255  0.48576  0.95510
    245   3.19128  5.25508  3.61290  2.94158  4.64344  3.78767  3.38358
 4.05437  1.86949  3.49067  4.37303  3.32380  4.15415  2.84954  1.00390
 3.19198  3.34921  3.74825  5.50260  4.32792    472 r - - -
          2.68618  4.42225  2.77519  2.73123  3.46354  2.40513  3.72494
 3.29354  2.67741  2.69355  4.24690  2.90347  2.73739  3.18146  2.89801
 2.37887  2.77519  2.98518  4.58477  3.61503
          0.02276  4.18974  4.91209  0.61958  0.77255  0.48576  0.95510
    246   2.81296  4.85280  2.98573  2.73874  4.11320  1.51689  1.80113
 3.89082  2.81565  3.47038  4.34709  3.19469  3.98712  3.21379  3.18240
 2.87948  3.15073  3.51036  5.43866  4.05317    473 g - - -
          2.68618  4.42225  2.77519  2.73123  3.46354  2.40513  3.72494
 3.29354  2.67741  2.69355  4.24690  2.90347  2.73739  3.18146  2.89801
 2.37887  2.77519  2.98518  4.58477  3.61503
          0.02276  4.18974  4.91209  0.61958  0.77255  0.48576  0.95510
    247   0.84171  4.23321  3.67671  3.44417  4.41577  3.03474  4.45864
 3.73805  3.48636  3.52290  4.38465  3.50115  3.81371  3.77001  3.74742
 2.13990  2.82752  3.21069  5.79711  4.59622    474 a - - -
          2.68618  4.42225  2.77519  2.73123  3.46354  2.40513  3.72494
 3.29354  2.67741  2.69355  4.24690  2.90347  2.73739  3.18146  2.89801
 2.37887  2.77519  2.98518  4.58477  3.61503
          0.02276  4.18974  4.91209  0.61958  0.77255  0.48576  0.95510
    248   3.40165  5.19430  3.92818  3.38703  4.63184  3.81244  4.10724
 4.20653  2.43575  3.66478  4.66672  3.71701  4.30874  3.32064  0.58608
 3.47692  3.65175  3.91911  5.59114  4.50157    475 R - - -
          2.68618  4.42225  2.77519  2.73123  3.46354  2.40513  3.72494
 3.29354  2.67741  2.69355  4.24690  2.90347  2.73739  3.18146  2.89801
 2.37887  2.77519  2.98518  4.58477  3.61503
          0.02276  4.18974  4.91209  0.61958  0.77255  0.48576  0.95510
    249   3.21441  4.49442  5.01723  4.47997  3.50413  4.61058  5.07457
 1.12846  4.37156  1.90304  2.81720  4.67910  4.85291  4.55512  4.51674
 3.97483  3.45720  1.45524  5.48363  4.33974    476 i - - -
          2.68618  4.42225  2.77519  2.73123  3.46354  2.40513  3.72494
 3.29354  2.67741  2.69355  4.24690  2.90347  2.73739  3.18146  2.89801
 2.37887  2.77519  2.98518  4.58477  3.61503
          0.02276  4.18974  4.91209  0.61958  0.77255  0.48576  0.95510
```

```
    250   1.26014  4.24467  3.57759  3.31118  4.48746  1.63680  4.38617
3.90774  3.40191  3.59552  4.40684  3.42658  3.78330  3.66207  3.70969
2.19861  2.80066  3.31287  5.82450  4.62608    477 a - - -
          2.68618  4.42225  2.77519  2.73123  3.46354  2.40513  3.72494
3.29354  2.67741  2.69355  4.24690  2.90347  2.73739  3.18146  2.89801
2.37887  2.77519  2.98518  4.58477  3.61503
          0.02276  4.18974  4.91209  0.61958  0.77255  0.48576  0.95510
    251   3.10928  4.84088  3.84696  3.79231  4.90541  0.37316  4.88115
4.59416  4.01605  4.20373  5.19050  4.01276  4.24101  4.31570  4.20587
3.29211  3.61492  4.06091  5.90463  5.02212    478 G - - -
          2.68618  4.42225  2.77519  2.73123  3.46354  2.40513  3.72494
3.29354  2.67741  2.69355  4.24690  2.90347  2.73739  3.18146  2.89801
2.37887  2.77519  2.98518  4.58477  3.61503
          0.02276  4.18974  4.91209  0.61958  0.77255  0.48576  0.95510
    252   3.40165  5.19430  3.92818  3.38703  4.63184  3.81244  4.10724
4.20653  2.43575  3.66478  4.66672  3.71701  4.30874  3.32064  0.58608
3.47692  3.65175  3.91911  5.59114  4.50157    479 R - - -
          2.68618  4.42225  2.77519  2.73123  3.46354  2.40513  3.72494
3.29354  2.67741  2.69355  4.24690  2.90347  2.73739  3.18146  2.89801
2.37887  2.77519  2.98518  4.58477  3.61503
          0.02276  4.18974  4.91209  0.61958  0.77255  0.48576  0.95510
    253   3.29546  5.24409  3.49077  3.06762  4.70467  3.73593  3.98007
4.15766  0.67263  3.65883  4.61453  3.47343  4.22315  3.16165  2.54906
3.32341  3.53046  3.85177  5.63637  4.50141    480 K - - -
          2.68618  4.42225  2.77519  2.73123  3.46354  2.40513  3.72494
3.29354  2.67741  2.69355  4.24690  2.90347  2.73739  3.18146  2.89801
2.37887  2.77519  2.98518  4.58477  3.61503
          0.02276  4.18974  4.91209  0.61958  0.77255  0.48576  0.95510
    254   3.25208  4.75649  4.40935  4.03890  3.39116  4.08257  4.70076
2.60074  3.78669  1.94746  0.87734  4.27355  4.55494  4.15293  3.95220
3.62881  3.56898  2.64168  5.26709  4.05214    481 m - - -
          2.68618  4.42225  2.77519  2.73123  3.46354  2.40513  3.72494
3.29354  2.67741  2.69355  4.24690  2.90347  2.73739  3.18146  2.89801
2.37887  2.77519  2.98518  4.58477  3.61503
          0.02276  4.18974  4.91209  0.61958  0.77255  0.48576  0.95510
    255   2.50280  4.42099  3.51846  3.36194  4.41314  3.14677  4.45600
3.98555  3.45818  3.68493  4.59140  3.52517  3.91427  3.78365  3.72501
0.69684  3.00893  3.44828  5.76162  4.50227    482 s - - -
          2.68618  4.42225  2.77519  2.73123  3.46354  2.40513  3.72494
3.29354  2.67741  2.69355  4.24690  2.90347  2.73739  3.18146  2.89801
2.37887  2.77519  2.98518  4.58477  3.61503
          0.02276  4.18974  4.91209  0.61958  0.77255  0.48576  0.95510
    256   2.66279  4.80417  3.03985  2.51743  4.01329  2.21689  3.06888
3.32998  2.44456  2.76854  3.89691  3.02205  3.87291  2.31448  2.86043
2.70855  2.89630  3.14692  5.29230  3.96348    483 g - - -
          2.68618  4.42225  2.77519  2.73123  3.46354  2.40513  3.72494
3.29354  2.67741  2.69355  4.24690  2.90347  2.73739  3.18146  2.89801
2.37887  2.77519  2.98518  4.58477  3.61503
          0.02276  4.18974  4.91209  0.61958  0.77255  0.48576  0.95510
    257   2.43595  4.45721  3.10911  2.89836  4.52504  1.42698  4.17290
3.99997  3.11975  3.62726  4.43842  2.82702  3.81808  3.39624  3.50699
1.67038  2.89416  3.43769  5.81610  4.53868    484 g - - -
          2.68618  4.42225  2.77519  2.73123  3.46354  2.40513  3.72494
3.29354  2.67741  2.69355  4.24690  2.90347  2.73739  3.18146  2.89801
2.37887  2.77519  2.98518  4.58477  3.61503
          0.02276  4.18974  4.91209  0.61958  0.77255  0.48576  0.95510
```

```
    258   1.05956  4.27798  3.94322  3.57228  3.99325  3.30073  4.46224
2.79377  3.50502  2.87223  3.92158  3.67514  3.99420  3.80806  3.76333
2.74120  2.92995  2.01307  5.55016  4.33744    485 a - - -
          2.68618  4.42225  2.77519  2.73123  3.46354  2.40513  3.72494
3.29354  2.67741  2.69355  4.24690  2.90347  2.73739  3.18146  2.89801
2.37887  2.77519  2.98518  4.58477  3.61503
          0.02276  4.18974  4.91209  0.61958  0.77255  0.48576  0.95510
    259   3.69300  5.00457  4.67758  4.46274  0.60725  4.28575  4.06088
3.19052  4.36512  2.49148  3.87941  4.39319  4.74492  4.44753  4.40563
3.93882  3.99821  3.20282  4.23534  2.57061    486 F - - -
          2.68618  4.42225  2.77519  2.73123  3.46354  2.40513  3.72494
3.29354  2.67741  2.69355  4.24690  2.90347  2.73739  3.18146  2.89801
2.37887  2.77519  2.98518  4.58477  3.61503
          0.02276  4.18974  4.91209  0.61958  0.77255  0.48576  0.95510
    260   2.66536  4.92979  3.00669  2.46034  4.23308  3.44981  3.64411
3.40931  1.73486  3.21955  4.02079  2.69551  3.85123  2.77826  2.69073
2.40628  2.50578  3.30453  5.41696  4.08209    487 k - - -
          2.68618  4.42225  2.77519  2.73123  3.46354  2.40513  3.72494
3.29354  2.67741  2.69355  4.24690  2.90347  2.73739  3.18146  2.89801
2.37887  2.77519  2.98518  4.58477  3.61503
          0.02276  4.18974  4.91209  0.61958  0.77255  0.48576  0.95510
    261   3.19918  5.30249  3.60714  2.92891  4.79241  3.78325  3.70861
4.13340  0.96999  3.54918  4.42677  3.31785  4.15123  2.84215  1.84571
3.18800  3.35754  3.81358  5.56258  4.41468    488 k - - -
          2.68618  4.42225  2.77519  2.73123  3.46354  2.40513  3.72494
3.29354  2.67741  2.69355  4.24690  2.90347  2.73739  3.18146  2.89801
2.37887  2.77519  2.98518  4.58477  3.61503
          0.02276  4.18974  4.91209  0.61958  0.77255  0.48576  0.95510
    262   3.20668  4.55287  4.83020  4.32418  3.41724  4.46148  4.94447
1.98976  4.16990  1.30958  3.25202  4.54020  4.76642  4.40018  4.33398
3.84481  3.46720  1.20707  5.39649  4.22057    489 v - - -
          2.68618  4.42225  2.77519  2.73123  3.46354  2.40513  3.72494
3.29354  2.67741  2.69355  4.24690  2.90347  2.73739  3.18146  2.89801
2.37887  2.77519  2.98518  4.58477  3.61503
          0.02276  4.18974  4.91209  0.61958  0.77255  0.48576  0.95510
    263   3.53202  4.90692  4.69853  4.35150  3.28922  4.38171  4.90937
2.52143  4.11692  0.56391  3.23347  4.59681  4.76983  4.40741  4.23619
4.00827  3.81578  2.63897  5.27097  4.06369    490 L - - -
          2.68618  4.42225  2.77519  2.73123  3.46354  2.40513  3.72494
3.29354  2.67741  2.69355  4.24690  2.90347  2.73739  3.18146  2.89801
2.37887  2.77519  2.98518  4.58477  3.61503
          0.02276  4.18974  4.91209  0.61958  0.77255  0.48576  0.95510
    264   2.74831  5.03007  2.63564  1.49996  4.49569  3.33039  3.80309
3.93300  2.64748  3.50792  4.32813  2.92557  3.87934  2.95910  3.11838
1.83852  2.87394  3.53164  5.70257  4.32045    491 e - - -
          2.68618  4.42225  2.77519  2.73123  3.46354  2.40513  3.72494
3.29354  2.67741  2.69355  4.24690  2.90347  2.73739  3.18146  2.89801
2.37887  2.77519  2.98518  4.58477  3.61503
          0.02276  4.18974  4.91209  0.61958  0.77255  0.48576  0.95510
    265   3.22717  5.33182  3.69741  2.95649  4.84407  3.81448  3.69271
4.16481  1.19667  3.56135  4.43633  3.33405  4.16374  2.82197  1.41289
3.21095  3.37144  3.84572  5.56259  4.43004    492 k - - -
          2.68618  4.42225  2.77519  2.73123  3.46354  2.40513  3.72494
3.29354  2.67741  2.69355  4.24690  2.90347  2.73739  3.18146  2.89801
2.37887  2.77519  2.98518  4.58477  3.61503
          0.02276  4.18974  4.91209  0.61958  0.77255  0.48576  0.95510
```

```
    266   3.43876  4.77480  4.95621  4.43016  2.87129  4.64412  4.89171
2.26852  4.27936  0.72733  2.96985  4.66936  4.84535  4.38593  4.39519
4.01579  3.67201  2.25901  5.15229  3.95280    493 l - - -
          2.68618  4.42225  2.77519  2.73123  3.46354  2.40513  3.72494
3.29354  2.67741  2.69355  4.24690  2.90347  2.73739  3.18146  2.89801
2.37887  2.77519  2.98518  4.58477  3.61503
          0.02276  4.18974  4.91209  0.61958  0.77255  0.48576  0.95510
    267   0.88251  4.22046  3.76238  3.46186  4.29564  3.06596  4.42272
3.52944  3.44425  3.36657  4.25202  3.51878  3.82554  3.73765  3.70818
2.25946  2.81741  2.87578  5.70472  4.50173    494 a - - -
          2.68618  4.42225  2.77519  2.73123  3.46354  2.40513  3.72494
3.29354  2.67741  2.69355  4.24690  2.90347  2.73739  3.18146  2.89801
2.37887  2.77519  2.98518  4.58477  3.61503
          0.02276  4.18974  4.91209  0.61958  0.77255  0.48576  0.95510
    268   1.40523  4.26516  3.61080  3.20220  4.28690  1.98037  4.23393
3.68557  3.20352  3.37198  4.19455  3.38599  3.79066  3.49199  3.54371
2.02522  2.59012  2.93384  5.63449  4.41752    495 a - - -
          2.68618  4.42225  2.77519  2.73123  3.46354  2.40513  3.72494
3.29354  2.67741  2.69355  4.24690  2.90347  2.73739  3.18146  2.89801
2.37887  2.77519  2.98518  4.58477  3.61503
          0.02276  4.18974  4.91209  0.61958  0.77255  0.48576  0.95510
    269   2.79362  5.45458  2.20151  1.01667  4.78554  3.33174  3.85319
4.24964  2.81541  3.79731  4.64984  2.82064  3.93601  3.01845  3.36351
2.89703  3.27203  3.84516  5.97389  4.50728    496 e - - -
          2.68618  4.42225  2.77519  2.73123  3.46354  2.40513  3.72494
3.29354  2.67741  2.69355  4.24690  2.90347  2.73739  3.18146  2.89801
2.37887  2.77519  2.98518  4.58477  3.61503
          0.02276  4.18974  4.91209  0.61958  0.77255  0.48576  0.95510
    270   2.86209  4.81966  2.70052  2.95277  4.72923  0.69926  4.36771
4.28800  3.42256  3.91872  4.81403  3.37047  4.04047  3.64203  3.81553
2.96525  3.30850  3.78628  5.89950  4.72398    497 g - - -
          2.68619  4.42226  2.77520  2.73124  3.46355  2.40513  3.72460
3.29355  2.67741  2.69356  4.24690  2.90347  2.73740  3.18147  2.89801
2.37887  2.77520  2.98519  4.58478  3.61504
          0.07094  2.79486  4.91209  0.44201  1.02929  0.48576  0.95510
    271   2.96591  4.80386  3.45629  2.96480  2.89318  3.75893  2.15588
3.40293  2.72486  2.96024  3.91982  3.35743  4.14619  3.05500  2.85834
3.05382  3.18853  3.16724  4.45650  1.63522    499 y - - -
          2.68618  4.42225  2.77519  2.73123  3.46354  2.40513  3.72494
3.29354  2.67741  2.69355  4.24690  2.90347  2.73739  3.18146  2.89801
2.37887  2.77519  2.98518  4.58477  3.61503
          0.02276  4.18974  4.91209  0.61958  0.77255  0.48576  0.95510
    272   2.97945  5.50178  1.39560  2.21231  4.85449  3.30336  3.85827
4.39363  2.86477  3.89330  4.73195  1.68212  3.92388  3.02309  3.45288
2.69233  3.27948  3.94813  6.04179  4.53887    500 d - - -
          2.68618  4.42225  2.77519  2.73123  3.46354  2.40513  3.72494
3.29354  2.67741  2.69355  4.24690  2.90347  2.73739  3.18146  2.89801
2.37887  2.77519  2.98518  4.58477  3.61503
          0.02276  4.18974  4.91209  0.61958  0.77255  0.48576  0.95510
    273   3.50161  4.80940  4.99705  4.49498  2.11524  4.65762  4.64970
2.39952  4.34490  0.79724  2.99195  4.65317  4.84958  4.39371  4.41795
4.03700  3.72444  2.56873  4.84209  3.45635    501 l - - -
          2.68618  4.42225  2.77519  2.73123  3.46354  2.40513  3.72494
3.29354  2.67741  2.69355  4.24690  2.90347  2.73739  3.18146  2.89801
2.37887  2.77519  2.98518  4.58477  3.61503
          0.02276  4.18974  4.91209  0.61958  0.77255  0.48576  0.95510
```

```
     274   2.08711  4.50405  3.18690  2.77999  4.28303  2.96567  3.96632
3.70648  2.83821  3.33832  4.15141  2.39538  3.81280  3.15830  3.25402
1.49150  2.84139  3.04366  5.57280  4.28525    502 s - - -
         2.68618  4.42225  2.77519  2.73123  3.46354  2.40513  3.72494
3.29354  2.67741  2.69355  4.24690  2.90347  2.73739  3.18146  2.89801
2.37887  2.77519  2.98518  4.58477  3.61503
         0.02276  4.18974  4.91209  0.61958  0.77255  0.48576  0.95510
     275   2.60968  4.21433  3.22291  2.66342  3.73561  3.50473  3.74201
2.85559  2.53196  2.66150  3.35391  1.96463  3.89660  2.96730  3.03337
2.62970  2.75082  2.83213  5.10875  3.83257    503 n - - -
         2.68618  4.42225  2.77519  2.73123  3.46354  2.40513  3.72494
3.29354  2.67741  2.69355  4.24690  2.90347  2.73739  3.18146  2.89801
2.37887  2.77519  2.98518  4.58477  3.61503
         0.02276  4.18974  4.91209  0.61958  0.77255  0.48576  0.95510
     276   2.46333  4.63135  3.04251  2.81785  4.30914  3.26987  4.05274
3.73671  2.87337  3.36654  4.27158  3.21208  1.19568  2.95910  3.21316
2.74145  3.02295  3.34313  5.62643  4.33595    504 p - - -
         2.68618  4.42225  2.77519  2.73123  3.46354  2.40513  3.72494
3.29354  2.67741  2.69355  4.24690  2.90347  2.73739  3.18146  2.89801
2.37887  2.77519  2.98518  4.58477  3.61503
         0.02276  4.18974  4.91209  0.61958  0.77255  0.48576  0.95510
     277   3.24938  4.51111  5.08920  4.57395  3.60643  4.69359  5.22281
1.12029  4.47088  1.86378  3.38920  4.77565  4.93632  4.68353  4.63198
4.07769  3.49973  1.31453  5.62425  4.45349    505 i - - -
         2.68618  4.42225  2.77519  2.73123  3.46354  2.40513  3.72494
3.29354  2.67741  2.69355  4.24690  2.90347  2.73739  3.18146  2.89801
2.37887  2.77519  2.98518  4.58477  3.61503
         0.02276  4.18974  4.91209  0.61958  0.77255  0.48576  0.95510
     278   3.36609  5.50581  0.55193  2.59278  4.93377  3.48351  4.25659
4.60188  3.40745  4.15709  5.15381  3.16092  4.15931  3.50422  3.95815
3.30035  3.71526  4.21066  6.03613  4.76917    506 D - - -
         2.68618  4.42225  2.77519  2.73123  3.46354  2.40513  3.72494
3.29354  2.67741  2.69355  4.24690  2.90347  2.73739  3.18146  2.89801
2.37887  2.77519  2.98518  4.58477  3.61503
         0.02276  4.18974  4.91209  0.61958  0.77255  0.48576  0.95510
     279   3.53202  4.90692  4.69853  4.35150  3.28922  4.38171  4.90937
2.52143  4.11692  0.56391  3.23347  4.59681  4.76983  4.40741  4.23619
4.00827  3.81578  2.63897  5.27097  4.06369    507 L - - -
         2.68618  4.42225  2.77519  2.73123  3.46354  2.40513  3.72494
3.29354  2.67741  2.69355  4.24690  2.90347  2.73739  3.18146  2.89801
2.37887  2.77519  2.98518  4.58477  3.61503
         0.02276  4.18974  4.91209  0.61958  0.77255  0.48576  0.95510
     280   3.19276  5.28975  3.57146  2.92351  4.77101  3.77085  3.72026
4.12186  0.93481  3.54655  4.42770  3.31581  4.14930  2.85667  1.94746
3.18460  3.35796  3.80266  5.56410  4.41079    508 k - - -
         2.68618  4.42225  2.77519  2.73123  3.46354  2.40513  3.72494
3.29354  2.67741  2.69355  4.24690  2.90347  2.73739  3.18146  2.89801
2.37887  2.77519  2.98518  4.58477  3.61503
         0.02276  4.18974  4.91209  0.61958  0.77255  0.48576  0.95510
     281   2.68563  5.07497  2.12548  2.24546  4.38501  3.38435  3.65793
3.84203  2.45442  3.37749  4.16011  2.80769  3.39012  2.78408  2.80994
2.56681  2.06144  3.45357  5.55884  4.16783    509 t - - -
         2.68618  4.42225  2.77519  2.73123  3.46354  2.40513  3.72494
3.29354  2.67741  2.69355  4.24690  2.90347  2.73739  3.18146  2.89801
2.37887  2.77519  2.98518  4.58477  3.61503
         0.02276  4.18974  4.91209  0.61958  0.77255  0.48576  0.95510
```

```
    282   3.06779  4.67415  3.87006  3.36630  1.79726  3.94820  1.91031
3.22290  3.27187  2.78698  3.80169  3.40290  4.30768  3.53024  3.35647
3.22702  3.29419  3.02629  4.08569  1.95898    510 f - - -
          2.68618  4.42225  2.77519  2.73123  3.46354  2.40513  3.72494
3.29354  2.67741  2.69355  4.24690  2.90347  2.73739  3.18146  2.89801
2.37887  2.77519  2.98518  4.58477  3.61503
          0.02276  4.18974  4.91209  0.61958  0.77255  0.48576  0.95510
    283   3.91781  5.16875  4.61085  4.40384  3.06619  4.11587  4.34908
3.96967  4.12769  3.30416  4.58623  4.49298  4.66201  4.49800  4.19578
4.11818  4.23697  3.86801  0.43253  3.05368    511 W - - -
          2.68618  4.42225  2.77519  2.73123  3.46354  2.40513  3.72494
3.29354  2.67741  2.69355  4.24690  2.90347  2.73739  3.18146  2.89801
2.37887  2.77519  2.98518  4.58477  3.61503
          0.02276  4.18974  4.91209  0.61958  0.77255  0.48576  0.95510
    284   0.63834  4.38326  3.81701  3.63762  4.40014  3.19674  4.61739
3.57572  3.66304  3.44146  4.44748  3.69667  3.96711  3.97632  3.88254
2.73561  3.03569  3.17302  5.79405  4.63485    512 A - - -
          2.68618  4.42225  2.77519  2.73123  3.46354  2.40513  3.72494
3.29354  2.67741  2.69355  4.24690  2.90347  2.73739  3.18146  2.89801
2.37887  2.77519  2.98518  4.58477  3.61503
          0.02276  4.18974  4.91209  0.61958  0.77255  0.48576  0.95510
    285   3.20815  5.31599  3.64414  2.93690  4.81557  3.79597  3.69927
4.14733  1.02561  3.55401  4.42909  3.32196  4.15462  2.83034  1.70864
3.19412  3.36010  3.82701  5.56233  4.42029    513 k - - -
          2.68618  4.42225  2.77519  2.73123  3.46354  2.40513  3.72494
3.29354  2.67741  2.69355  4.24690  2.90347  2.73739  3.18146  2.89801
2.37887  2.77519  2.98518  4.58477  3.61503
          0.02276  4.18974  4.91209  0.61958  0.77255  0.48576  0.95510
    286   3.40929  5.14819  3.49941  3.30260  3.55988  3.76053  0.61974
4.16296  3.13953  3.61075  4.68806  3.69430  4.33594  3.67638  3.38001
3.48071  3.72844  3.89233  5.01723  3.50419    514 H - - -
          2.68618  4.42225  2.77519  2.73123  3.46354  2.40513  3.72494
3.29354  2.67741  2.69355  4.24690  2.90347  2.73739  3.18146  2.89801
2.37887  2.77519  2.98518  4.58477  3.61503
          0.02276  4.18974  4.91209  0.61958  0.77255  0.48576  0.95510
    287   3.10928  4.84088  3.84696  3.79231  4.90541  0.37316  4.88115
4.59416  4.01605  4.20373  5.19050  4.01276  4.24101  4.31570  4.20587
3.29211  3.61492  4.06091  5.90463  5.02212    515 G - - -
          2.68618  4.42225  2.77519  2.73123  3.46354  2.40513  3.72494
3.29354  2.67741  2.69355  4.24690  2.90347  2.73739  3.18146  2.89801
2.37887  2.77519  2.98518  4.58477  3.61503
          0.02276  4.18974  4.91209  0.61958  0.77255  0.48576  0.95510
    288   2.61901  4.46068  3.74951  3.53189  4.28867  3.28571  4.51986
3.45688  3.48136  3.30855  4.34770  3.67846  4.01883  3.86229  3.71597
2.82506  0.72910  3.11534  5.69655  4.50903    516 t - - -
          2.68618  4.42225  2.77519  2.73123  3.46354  2.40513  3.72494
3.29354  2.67741  2.69355  4.24690  2.90347  2.73739  3.18146  2.89801
2.37887  2.77519  2.98518  4.58477  3.61503
          0.02276  4.18974  4.91209  0.61958  0.77255  0.48576  0.95510
    289   3.02486  5.01057  2.94004  2.86390  4.42850  3.42843  4.23060
4.22159  3.16920  3.83448  4.79714  0.69899  4.10095  3.51411  3.52609
3.08617  3.42333  3.81033  5.69740  4.36195    517 n - - -
          2.68618  4.42225  2.77519  2.73123  3.46354  2.40513  3.72494
3.29354  2.67741  2.69355  4.24690  2.90347  2.73739  3.18146  2.89801
2.37887  2.77519  2.98518  4.58477  3.61503
          0.02276  4.18974  4.91209  0.61958  0.77255  0.48576  0.95510
```

```
    290   3.22717  5.33182  3.69741  2.95649  4.84407  3.81448  3.69271
4.16481  1.19667  3.56135  4.43633  3.33405  4.16374  2.82197  1.41289
3.21095  3.37144  3.84572  5.56259  4.43004    518 k - - -
          2.68618  4.42225  2.77519  2.73123  3.46354  2.40513  3.72494
3.29354  2.67741  2.69355  4.24690  2.90347  2.73739  3.18146  2.89801
2.37887  2.77519  2.98518  4.58477  3.61503
          0.02276  4.18974  4.91209  0.61958  0.77255  0.48576  0.95510
    291   3.91376  5.06812  4.88628  4.63614  1.04470  4.60301  3.55021
3.55755  4.46367  2.84031  4.13618  4.25154  4.89638  4.34403  4.43007
3.98578  4.12974  3.49390  3.66360  1.15761    519 f - - -
          2.68618  4.42225  2.77519  2.73123  3.46354  2.40513  3.72494
3.29354  2.67741  2.69355  4.24690  2.90347  2.73739  3.18146  2.89801
2.37887  2.77519  2.98518  4.58477  3.61503
          0.02276  4.18974  4.91209  0.61958  0.77255  0.48576  0.95510
    292   3.22225  4.49536  5.06014  4.57511  3.72132  4.65007  5.28073
1.22673  4.47435  2.20152  3.50543  4.76694  4.93976  4.73104  4.65659
4.05424  3.48802  1.04365  5.73061  4.52301    520 v - - -
          2.68618  4.42225  2.77519  2.73123  3.46354  2.40513  3.72494
3.29354  2.67741  2.69355  4.24690  2.90347  2.73739  3.18146  2.89801
2.37887  2.77519  2.98518  4.58477  3.61503
          0.02276  4.18974  4.91209  0.61958  0.77255  0.48576  0.95510
    293   2.65063  4.39419  3.82069  3.39327  3.78136  3.51056  4.30696
2.32721  3.26714  2.62108  3.72608  3.64533  4.09932  3.63520  3.54952
2.91420  1.19498  2.43795  5.37227  4.12657    521 t - - -
          2.68618  4.42225  2.77519  2.73123  3.46354  2.40513  3.72494
3.29354  2.67741  2.69355  4.24690  2.90347  2.73739  3.18146  2.89801
2.37887  2.77519  2.98518  4.58477  3.61503
          0.02276  4.18974  4.91209  0.61958  0.77255  0.48576  0.95510
    294   3.28738  4.63375  4.76443  4.40190  3.60929  4.39324  5.08266
0.75312  4.23685  2.14248  3.50777  4.60501  4.80479  4.55976  4.39903
3.92997  3.58520  1.90847  5.55754  4.31595    522 i - - -
          2.68618  4.42225  2.77519  2.73123  3.46354  2.40513  3.72494
3.29354  2.67741  2.69355  4.24690  2.90347  2.73739  3.18146  2.89801
2.37887  2.77519  2.98518  4.58477  3.61503
          0.02276  4.18974  4.91209  0.61958  0.77255  0.48576  0.95510
    295   3.23504  5.33248  3.71292  2.96667  4.84625  3.81992  3.69413
4.16511  1.30050  3.56140  4.43820  3.34087  4.16841  2.82389  1.28832
3.21967  3.37791  3.84785  5.56173  4.43237    523 r - - -
          2.68618  4.42225  2.77519  2.73123  3.46354  2.40513  3.72494
3.29354  2.67741  2.69355  4.24690  2.90347  2.73739  3.18146  2.89801
2.37887  2.77519  2.98518  4.58477  3.61503
          0.01538  4.18236        *  0.61958  0.77255  0.00000        *
//
HMMER3/f [3.3 | Nov 2019]
NAME  GATA
ACC   PF00320.28
DESC  GATA zinc finger
LENG  36
ALPH  amino
RF    no
MM    no
CONS  yes
CS    no
MAP   yes
DATE  Wed Apr 22 09:38:58 2020
NSEQ  56
EFFN  10.298340
CKSUM 142612169
```

```
GA    26.80 26.80
TC    26.80 26.80
NC    26.70 26.70
STATS LOCAL MSV       -7.8431  0.71963
STATS LOCAL VITERBI   -8.2380  0.71963
STATS LOCAL FORWARD   -4.5594  0.71963
HMM          A        C        D        E        F        G        H
I        K        L        M        N        P        Q        R        S
T        V        W        Y
            m->m     m->i     m->d     i->m     i->i     d->m     d->d
  COMPO   2.84077  2.16480  3.48633  2.92117  4.02459  2.69648  3.62136
3.68018  2.58934  2.61085  3.93217  2.78252  3.32210  3.21268  2.58235
2.79796  2.55710  3.45923  3.52875  3.73482
          2.68618  4.42225  2.77519  2.73123  3.46354  2.40513  3.72494
3.29354  2.67741  2.69355  4.24690  2.90347  2.73739  3.18146  2.89801
2.37887  2.77519  2.98518  4.58477  3.61503
          0.00372  5.99268  6.71503  0.61958  0.77255  0.00000        *
      1   6.26615  0.01570  7.21884  7.29927  7.59459  6.11036  7.77235
7.71781  7.40186  7.05871  8.29324  7.33331  6.80896  7.67565  7.18362
6.55182  6.79352  7.20480  8.14945  7.81868      1 C - - -
          2.68618  4.42225  2.77519  2.73123  3.46354  2.40513  3.72494
3.29354  2.67741  2.69355  4.24690  2.90347  2.73739  3.18146  2.89801
2.37887  2.77519  2.98518  4.58477  3.61503
          0.00372  5.99268  6.71503  0.61958  0.77255  0.48576  0.95510
      2   2.12432  5.87424  3.67900  2.07303  3.48843  3.39568  3.49067
4.67224  3.12440  4.17118  4.92007  3.66783  3.67577  2.73425  2.38875
1.91114  2.21353  2.63971  6.31878  4.01348      2 s - - -
          2.68629  4.42236  2.77531  2.73134  3.46365  2.40524  3.72506
3.29365  2.67752  2.69366  4.24305  2.90254  2.73751  3.18158  2.89812
2.37898  2.77531  2.98530  4.58488  3.61514
          0.05627  2.92802  6.71503  0.67782  0.70872  0.48576  0.95510
      3   3.41135  5.91840  3.06975  2.85296  4.23907  4.18461  2.04769
4.74672  2.80599  4.22295  4.95958  1.27558  4.57857  2.24549  3.60069
2.29649  3.26562  4.30817  3.55353  4.94184      5 n - - -
          2.68618  4.42225  2.77519  2.73123  3.46354  2.40513  3.72494
3.29354  2.67741  2.69355  4.24690  2.90347  2.73739  3.18146  2.89801
2.37887  2.77519  2.98518  4.58477  3.61503
          0.00372  5.99268  6.71503  0.61958  0.77255  0.48576  0.95510
      4   6.26615  0.01570  7.21884  7.29927  7.59459  6.11036  7.77235
7.71781  7.40186  7.05871  8.29324  7.33331  6.80896  7.67565  7.18362
6.55182  6.79352  7.20480  8.14945  7.81868      6 C - - -
          2.68618  4.42225  2.77519  2.73123  3.46354  2.40513  3.72494
3.29354  2.67741  2.69355  4.24690  2.90347  2.73739  3.18146  2.89801
2.37887  2.77519  2.98518  4.58477  3.61503
          0.00372  5.99268  6.71503  0.61958  0.77255  0.48576  0.95510
      5   2.90279  5.91631  3.45001  2.97209  3.70492  1.42772  2.51584
4.74519  2.36447  3.85666  4.95730  2.05727  4.57635  2.90064  2.80659
2.87306  3.41002  4.30627  6.34974  3.68539      7 g - - -
          2.68618  4.42225  2.77519  2.73123  3.46354  2.40513  3.72494
3.29354  2.67741  2.69355  4.24690  2.90347  2.73739  3.18146  2.89801
2.37887  2.77519  2.98518  4.58477  3.61503
          0.00372  5.99268  6.71503  0.61958  0.77255  0.48576  0.95510
      6   2.20636  4.17367  2.97669  2.71476  4.97693  3.21572  4.45011
4.40796  3.04759  3.98493  4.77998  3.76060  4.63712  3.58421  3.70426
2.61020  1.07745  2.27494  6.20247  4.84913      8 t - - -
          2.68618  4.42225  2.77519  2.73123  3.46354  2.40513  3.72494
3.29354  2.67741  2.69355  4.24690  2.90347  2.73739  3.18146  2.89801
2.37887  2.77519  2.98518  4.58477  3.61503
```

```
          0.00372  5.99268  6.71503  0.61958  0.77255  0.48576  0.95510
      7   2.97712  5.83271  2.61947  2.99859  4.44190  4.20260  3.97824
2.63314  2.70788  2.47422  4.88315  3.24848  4.59513  3.33353  2.82175
2.25347  1.29365  4.20357  6.28790  4.90012      9 t - - -
          2.68618  4.42225  2.77519  2.73123  3.46354  2.40513  3.72494
3.29354  2.67741  2.69355  4.24690  2.90347  2.73739  3.18146  2.89801
2.37887  2.77519  2.98518  4.58477  3.61503
          0.00372  5.99268  6.71503  0.61958  0.77255  0.48576  0.95510
      8   2.97851  5.92120  2.98489  2.08402  3.07200  3.16759  3.99775
4.75385  1.73933  4.22688  4.96161  2.26324  4.57524  2.64069  3.34864
2.55592  2.20311  4.31234  3.52529  4.94179     10 k - - -
          2.68635  4.42242  2.77537  2.73140  3.46371  2.40479  3.72512
3.29371  2.67699  2.69372  4.24707  2.90364  2.73757  3.18163  2.89818
2.37860  2.77464  2.98535  4.58494  3.61520
          0.12416  2.52818  3.29801  0.68226  0.70416  0.48576  0.95510
      9   3.77098  5.68747  5.98811  6.02101  6.57914  4.47432  6.66286
6.12071  6.02465  5.81924  6.55593  5.37421  5.35905  6.10978  6.00239
1.71636  0.29491  5.14184  7.90748  6.89698     13 T - - -
          2.68623  4.42231  2.77525  2.73129  3.46359  2.40518  3.72500
3.29359  2.67746  2.69360  4.24695  2.90352  2.73745  3.18152  2.89806
2.37831  2.77525  2.98524  4.58482  3.61509
          0.05835  2.89271  6.67863  0.16912  1.86050  0.36025  1.19567
     10   2.53512  3.62096  3.18808  3.53040  3.24782  4.36516  4.59534
3.30936  2.58205  3.06038  4.45344  3.99824  1.10008  3.83420  3.91944
2.28732  2.83532  3.65322  5.91613  4.64552     15 p - - -
          2.68618  4.42225  2.77519  2.73123  3.46354  2.40513  3.72494
3.29354  2.67741  2.69355  4.24690  2.90347  2.73739  3.18146  2.89801
2.37887  2.77519  2.98518  4.58477  3.61503
          0.00372  5.99268  6.71503  0.61958  0.77255  0.48576  0.95510
     11   3.41755  3.04280  2.88002  2.72888  5.07604  4.21513  3.42826
4.52509  3.16951  1.42102  2.54163  3.07701  4.60713  1.78712  3.05250
3.21606  3.34311  4.14659  6.25260  4.87742     16 l - - -
          2.68618  4.42225  2.77519  2.73123  3.46354  2.40513  3.72494
3.29354  2.67741  2.69355  4.24690  2.90347  2.73739  3.18146  2.89801
2.37887  2.77519  2.98518  4.58477  3.61503
          0.00372  5.99268  6.71503  0.61958  0.77255  0.48576  0.95510
     12   5.05093  6.25338  6.94086  6.40684  4.23527  6.34294  6.29589
3.68002  6.18142  2.34358  3.06226  6.45577  6.40325  6.04924  6.09651
5.74978  5.25925  2.87972  0.32603  4.99588     17 W - - -
          2.68618  4.42225  2.77519  2.73123  3.46354  2.40513  3.72494
3.29354  2.67741  2.69355  4.24690  2.90347  2.73739  3.18146  2.89801
2.37887  2.77519  2.98518  4.58477  3.61503
          0.00372  5.99268  6.71503  0.61958  0.77255  0.48576  0.95510
     13   4.38500  6.48748  4.94608  4.14625  5.88354  5.02737  3.02949
3.34617  2.65853  4.69118  5.55611  3.15935  5.35396  4.01127  0.42275
4.37732  4.52567  4.95768  6.70041  3.35505     18 R - - -
          2.68618  4.42225  2.77519  2.73123  3.46354  2.40513  3.72494
3.29354  2.67741  2.69355  4.24690  2.90347  2.73739  3.18146  2.89801
2.37887  2.77519  2.98518  4.58477  3.61503
          0.00372  5.99268  6.71503  0.61958  0.77255  0.48576  0.95510
     14   2.74943  5.92160  3.65673  2.17886  5.26768  3.52568  3.11330
4.75426  2.42435  3.10660  4.96202  2.74725  3.58652  2.96877  1.31341
2.31535  3.40071  4.31279  6.35362  4.94220     19 r - - -
          2.68618  4.42225  2.77519  2.73123  3.46354  2.40513  3.72494
3.29354  2.67741  2.69355  4.24690  2.90347  2.73739  3.18146  2.89801
2.37887  2.77519  2.98518  4.58477  3.61503
          0.00372  5.99268  6.71503  0.61958  0.77255  0.48576  0.95510
```

```
     15   2.41117  5.89618  2.64182  2.46003  5.22691  1.19971  4.39308
4.70213  3.13708  4.19677  4.94399  2.13670  4.59520  3.49548  3.62795
2.76641  3.29317  3.24915  3.64327  4.93906     20 g - - -
          2.68648  4.42255  2.77549  2.73153  3.46383  2.40497  3.72524
3.29384  2.67770  2.69384  4.24719  2.90376  2.73706  3.18176  2.89830
2.37794  2.77491  2.98548  4.58218  3.61398
          0.17973  1.99521  3.55721  0.68642  0.69992  0.48576  0.95510
     16   2.84224  5.88918  3.07015  2.66162  5.22769  2.51364  4.35853
4.70881  2.43846  4.19167  3.53898  3.64025  1.01177  3.45724  2.82640
3.07555  3.62625  3.88046  6.32600  4.91929     23 p - - -
          2.68618  4.42225  2.77519  2.73123  3.46354  2.40513  3.72494
3.29354  2.67741  2.69355  4.24690  2.90347  2.73739  3.18146  2.89801
2.37887  2.77519  2.98518  4.58477  3.61503
          0.00382  5.96500  6.68734  0.61958  0.77255  0.67454  0.71211
     17   2.91544  5.90173  1.98206  2.18873  4.46946  4.16395  3.08770
4.73336  2.94481  3.01629  4.94230  1.94777  3.84320  2.85236  2.53801
2.43246  2.94342  3.10584  6.33422  4.18062     24 n - - -
          2.68618  4.42225  2.77519  2.73123  3.46354  2.40513  3.72494
3.29354  2.67741  2.69355  4.24690  2.90347  2.73739  3.18146  2.89801
2.37887  2.77519  2.98518  4.58477  3.61503
          0.00382  5.96500  6.68734  0.61958  0.77255  0.38358  1.14388
     18   3.45219  5.84051  3.72390  2.70843  5.14042  0.81197  4.43124
3.44092  2.64788  4.12521  2.87695  2.98478  4.63137  3.10204  3.65967
2.85661  3.68361  4.20717  6.30069  4.92167     25 g - - -
          2.68618  4.42225  2.77519  2.73123  3.46354  2.40513  3.72494
3.29354  2.67741  2.69355  4.24690  2.90347  2.73739  3.18146  2.89801
2.37887  2.77519  2.98518  4.58477  3.61503
          0.00372  5.99268  6.71503  0.61958  0.77255  0.48576  0.95510
     19   2.52715  5.92190  3.25816  2.41472  5.26821  3.22767  3.67848
4.75506  2.38634  3.42444  4.96224  2.00200  2.05879  2.39122  3.38995
2.48053  2.45915  4.31320  6.35384  4.94214     26 n - - -
          2.68618  4.42225  2.77519  2.73123  3.46354  2.40513  3.72494
3.29354  2.67741  2.69355  4.24690  2.90347  2.73739  3.18146  2.89801
2.37887  2.77519  2.98518  4.58477  3.61503
          0.00372  5.99268  6.71503  0.61958  0.77255  0.48576  0.95510
     20   2.97226  3.57014  3.22588  3.25923  4.88896  3.47082  4.45753
2.66580  1.86878  2.50784  4.71508  3.20874  2.66509  3.60367  2.13744
2.75125  2.73266  2.94788  6.14502  3.23443     27 k - - -
          2.68618  4.42225  2.77519  2.73123  3.46354  2.40513  3.72494
3.29354  2.67741  2.69355  4.24690  2.90347  2.73739  3.18146  2.89801
2.37887  2.77519  2.98518  4.58477  3.61503
          0.35007  5.99268  1.22804  0.61958  0.77255  0.48576  0.95510
     21   3.20924  3.59235  3.27992  2.48573  3.75794  4.03330  3.12012
4.11475  3.02630  2.60928  3.27006  3.55549  4.42356  2.88021  2.38426
1.78441  1.93969  3.31404  5.94006  4.59687     28 s - - -
          2.68618  4.42225  2.77519  2.73123  3.46354  2.40513  3.72494
3.29354  2.67741  2.69355  4.24690  2.90347  2.73739  3.18146  2.89801
2.37887  2.77519  2.98518  4.58477  3.61503
          0.00525  5.64786  6.37021  0.61958  0.77255  0.12729  2.12424
     22   4.74269  5.98375  6.70526  2.83210  4.39334  6.09519  6.41868
2.89907  5.96777  0.36890  3.13640  6.25818  6.20440  5.89474  5.92691
5.46593  4.95424  2.36663  6.52757  5.53552     29 L - - -
          2.68618  4.42225  2.77519  2.73123  3.46354  2.40513  3.72494
3.29354  2.67741  2.69355  4.24690  2.90347  2.73739  3.18146  2.89801
2.37887  2.77519  2.98518  4.58477  3.61503
          0.00372  5.99268  6.71503  0.61958  0.77255  0.48576  0.95510
```

```
     23   6.26615  0.01570  7.21884  7.29927  7.59459  6.11036  7.77235
7.71781  7.40186  7.05871  8.29324  7.33331  6.80896  7.67565  7.18362
6.55182  6.79352  7.20480  8.14945  7.81868     30 C - - -
          2.68618  4.42225  2.77519  2.73123  3.46354  2.40513  3.72494
3.29354  2.67741  2.69355  4.24690  2.90347  2.73739  3.18146  2.89801
2.37887  2.77519  2.98518  4.58477  3.61503
          0.00372  5.99268  6.71503  0.61958  0.77255  0.48576  0.95510
     24   2.88456  5.93969  3.68363  2.46740  5.27366  4.23722  4.44993
4.74550  2.72910  3.21704  4.99974  0.74077  4.64615  3.55674  3.68510
2.80499  2.85774  4.32778  6.39403  4.99496     31 n - - -
          2.68618  4.42225  2.77519  2.73123  3.46354  2.40513  3.72494
3.29354  2.67741  2.69355  4.24690  2.90347  2.73739  3.18146  2.89801
2.37887  2.77519  2.98518  4.58477  3.61503
          0.00372  5.99268  6.71503  0.61958  0.77255  0.48576  0.95510
     25   0.79453  6.01202  2.81974  3.17973  5.36990  4.24926  4.48547
4.85454  3.24365  4.33734  5.08368  3.06994  2.75538  3.01658  3.31143
2.21103  3.76080  4.41697  6.47269  5.05972     32 a - - -
          2.68618  4.42225  2.77519  2.73123  3.46354  2.40513  3.72494
3.29354  2.67741  2.69355  4.24690  2.90347  2.73739  3.18146  2.89801
2.37887  2.77519  2.98518  4.58477  3.61503
          0.00372  5.99268  6.71503  0.61958  0.77255  0.48576  0.95510
     26   6.26615  0.01570  7.21884  7.29927  7.59459  6.11036  7.77235
7.71781  7.40186  7.05871  8.29324  7.33331  6.80896  7.67565  7.18362
6.55182  6.79352  7.20480  8.14945  7.81868     33 C - - -
          2.68618  4.42225  2.77519  2.73123  3.46354  2.40513  3.72494
3.29354  2.67741  2.69355  4.24690  2.90347  2.73739  3.18146  2.89801
2.37887  2.77519  2.98518  4.58477  3.61503
          0.00372  5.99268  6.71503  0.61958  0.77255  0.48576  0.95510
     27   3.58458  5.16041  4.77550  4.18722  4.27065  0.67923  4.87748
2.94528  3.94372  3.36861  2.81224  3.40206  4.99192  4.31922  2.97594
3.89432  3.81920  3.43466  2.84560  4.53047     34 G - - -
          2.68618  4.42225  2.77519  2.73123  3.46354  2.40513  3.72494
3.29354  2.67741  2.69355  4.24690  2.90347  2.73739  3.18146  2.89801
2.37887  2.77519  2.98518  4.58477  3.61503
          0.00372  5.99268  6.71503  0.61958  0.77255  0.48576  0.95510
     28   3.61888  4.98831  5.55533  3.34707  4.08584  4.80324  5.14504
1.55418  4.74569  0.88729  3.68618  4.95451  5.16050  4.88504  4.76958
3.55768  2.68757  2.31100  5.64162  4.46577     35 l - - -
          2.68618  4.42225  2.77519  2.73123  3.46354  2.40513  3.72494
3.29354  2.67741  2.69355  4.24690  2.90347  2.73739  3.18146  2.89801
2.37887  2.77519  2.98518  4.58477  3.61503
          0.00372  5.99268  6.71503  0.61958  0.77255  0.48576  0.95510
     29   2.42395  5.38371  4.07366  3.51106  2.46378  4.36063  3.13636
3.93765  2.48325  3.61208  3.31306  3.98355  4.74521  3.81629  2.06444
3.02014  3.05621  3.67876  5.93380  1.37715     36 y - - -
          2.68618  4.42225  2.77519  2.73123  3.46354  2.40513  3.72494
3.29354  2.67741  2.69355  4.24690  2.90347  2.73739  3.18146  2.89801
2.37887  2.77519  2.98518  4.58477  3.61503
          0.00372  5.99268  6.71503  0.61958  0.77255  0.48576  0.95510
     30   3.26234  5.02168  4.81029  3.59631  1.76749  4.57505  3.57868
3.26298  4.13232  2.81307  3.58012  2.68507  4.94678  3.03138  4.36132
3.51864  3.46683  3.27866  1.93444  1.66614     37 y - - -
          2.68618  4.42225  2.77519  2.73123  3.46354  2.40513  3.72494
3.29354  2.67741  2.69355  4.24690  2.90347  2.73739  3.18146  2.89801
2.37887  2.77519  2.98518  4.58477  3.61503
          0.00372  5.99268  6.71503  0.61958  0.77255  0.48576  0.95510
```

```
     31   2.32516  6.30812  4.28435  3.64002  5.75637  3.56526  4.67008
5.16130  1.12357  4.57564  5.36682  4.12207  5.01034  2.63459  1.19431
3.90280  3.18448  4.76309  6.62528  5.35206     38 k - - -
          2.68618  4.42225  2.77519  2.73123  3.46354  2.40513  3.72494
3.29354  2.67741  2.69355  4.24690  2.90347  2.73739  3.18146  2.89801
2.37887  2.77519  2.98518  4.58477  3.61503
          0.00372  5.99268  6.71503  0.61958  0.77255  0.48576  0.95510
     32   3.42421  5.74296  3.75886  3.20132  5.01732  4.23067  3.58431
3.78769  1.09006  1.96427  3.59478  3.03340  4.62184  3.21844  2.98467
2.36915  3.11896  3.55340  6.21999  4.85746     39 k - - -
          2.68618  4.42225  2.77519  2.73123  3.46354  2.40513  3.72494
3.29354  2.67741  2.69355  4.24690  2.90347  2.73739  3.18146  2.89801
2.37887  2.77519  2.98518  4.58477  3.61503
          0.00372  5.99268  6.71503  0.61958  0.77255  0.48576  0.95510
     33   3.25316  5.89371  3.66947  2.47735  5.22757  3.00834  2.18389
3.72320  1.74431  3.39710  3.48425  2.75525  4.58124  3.09375  2.62905
3.37757  2.65183  3.82825  6.33314  2.31027     40 k - - -
          2.68618  4.42225  2.77519  2.73123  3.46354  2.40513  3.72494
3.29354  2.67741  2.69355  4.24690  2.90347  2.73739  3.18146  2.89801
2.37887  2.77519  2.98518  4.58477  3.61503
          0.00372  5.99268  6.71503  0.61958  0.77255  0.48576  0.95510
     34   2.59955  5.85030  3.69134  2.40262  3.86980  1.33187  3.13397
3.56925  2.72234  3.45201  3.93853  3.15572  4.59101  3.49533  2.02306
2.94206  3.16987  4.22502  6.30101  4.90856     41 g - - -
          2.68618  4.42225  2.77519  2.73123  3.46354  2.40513  3.72494
3.29354  2.67741  2.69355  4.24690  2.90347  2.73739  3.18146  2.89801
2.37887  2.77519  2.98518  4.58477  3.61503
          0.00372  5.99268  6.71503  0.61958  0.77255  0.48576  0.95510
     35   3.41210  5.86498  3.68423  2.26986  5.18659  3.05206  3.08012
4.08670  2.47623  2.02919  4.91188  3.67216  4.58794  2.41675  2.66465
2.52450  2.21607  2.25783  6.31192  4.91567     42 l - - -
          2.68618  4.42225  2.77519  2.73123  3.46354  2.40513  3.72494
3.29354  2.67741  2.69355  4.24690  2.90347  2.73739  3.18146  2.89801
2.37887  2.77519  2.98518  4.58477  3.61503
          0.04011  5.99268  3.30181  0.61958  0.77255  0.48576  0.95510
     36   2.78136  4.55865  3.37621  2.99951  4.31926  4.19075  3.65818
4.50114  1.86131  1.92099  2.61241  2.96874  2.93392  3.30547  2.18494
3.04836  3.19663  2.97323  6.22849  4.85317     43 k - - -
          2.68618  4.42225  2.77519  2.73123  3.46354  2.40513  3.72494
3.29354  2.67741  2.69355  4.24690  2.90347  2.73739  3.18146  2.89801
2.37887  2.77519  2.98518  4.58477  3.61503
          0.00260  5.95517        *  0.61958  0.77255  0.00000        *
//
HMMER3/f [3.3 | Nov 2019]
NAME  GRAS
ACC   PF03514.15
DESC  GRAS domain family
LENG  374
ALPH  amino
RF    no
MM    no
CONS  yes
CS    yes
MAP   yes
DATE  Sat Apr  4 19:40:56 2020
NSEQ  30
EFFN  2.340088
CKSUM 3600684689
```

```
GA    21.40 21.40
TC    21.50 21.50
NC    21.30 21.20
STATS LOCAL MSV       -10.9609  0.69967
STATS LOCAL VITERBI   -12.2710  0.69967
STATS LOCAL FORWARD    -5.7365  0.69967
HMM          A        C        D        E        F        G        H
I        K        L        M        N        P        Q        R        S
T        V        W        Y
            m->m     m->i     m->d     i->m     i->i     d->m     d->d
  COMPO   2.45708  4.34834  3.07939  2.59467  3.23547  3.09415  3.63579
2.86602  2.74602  2.33464  3.60204  3.08340  3.46184  3.01657  2.86025
2.62603  2.83684  2.64385  4.23673  3.70609
          2.68618  4.42225  2.77519  2.73123  3.46354  2.40513  3.72494
3.29354  2.67741  2.69355  4.24690  2.90347  2.73739  3.18146  2.89801
2.37887  2.77519  2.98518  4.58477  3.61503
          0.01309  4.73812  5.46046  0.61958  0.77255  0.00000        *
      1   2.41464  4.21470  4.53568  3.93536  3.02956  3.94569  4.26634
2.23654  3.76829  1.25218  2.88479  4.02900  3.86897  3.94071  3.85704
2.92538  3.03406  2.45171  4.83153  3.29495      1 l - - H
          2.68618  4.42225  2.77519  2.73123  3.46354  2.40513  3.72494
3.29354  2.67741  2.69355  4.24690  2.90347  2.73739  3.18146  2.89801
2.37887  2.77519  2.98518  4.58477  3.61503
          0.01309  4.73812  5.46046  0.61958  0.77255  0.48576  0.95510
      2   2.78378  4.76618  3.02596  2.46977  3.95218  3.67013  3.88982
2.37292  2.51882  2.58860  3.85358  3.26989  4.05598  3.09962  2.26416
2.88879  2.85661  2.11140  5.30873  4.02270      2 v - - H
          2.68618  4.42225  2.77519  2.73123  3.46354  2.40513  3.72494
3.29354  2.67741  2.69355  4.24690  2.90347  2.73739  3.18146  2.89801
2.37887  2.77519  2.98518  4.58477  3.61503
          0.01309  4.73812  5.46046  0.61958  0.77255  0.48576  0.95510
      3   2.70503  5.28406  2.63680  2.28607  4.62722  3.39601  2.39856
4.11170  2.27641  3.41918  4.32542  2.74937  3.94497  2.31512  2.57650
2.33743  2.83281  3.67372  5.71755  4.30791      3 k - - H
          2.68618  4.42225  2.77519  2.73123  3.46354  2.40513  3.72494
3.29354  2.67741  2.69355  4.24690  2.90347  2.73739  3.18146  2.89801
2.37887  2.77519  2.98518  4.58477  3.61503
          0.01309  4.73812  5.46046  0.61958  0.77255  0.48576  0.95510
      4   2.21628  4.62361  3.07902  2.75895  3.78118  3.71605  3.95494
3.00008  2.90162  1.58081  3.72018  3.38094  4.09994  2.75106  3.29652
2.94868  2.77590  2.53257  5.19054  3.93332      4 l - - H
          2.68618  4.42225  2.77519  2.73123  3.46354  2.40513  3.72494
3.29354  2.67741  2.69355  4.24690  2.90347  2.73739  3.18146  2.89801
2.37887  2.77519  2.98518  4.58477  3.61503
          0.01309  4.73812  5.46046  0.61958  0.77255  0.48576  0.95510
      5   4.02317  5.24000  5.86700  5.32241  3.47303  5.41076  5.75703
2.34789  5.16338  0.44167  3.21812  5.55851  5.44885  5.09468  5.15680
4.83036  4.24056  2.38582  5.74353  4.76613      5 L - - H
          2.68618  4.42225  2.77519  2.73123  3.46354  2.40513  3.72494
3.29354  2.67741  2.69355  4.24690  2.90347  2.73739  3.18146  2.89801
2.37887  2.77519  2.98518  4.58477  3.61503
          0.01309  4.73812  5.46046  0.61958  0.77255  0.48576  0.95510
      6   2.80054  4.18957  4.66448  4.05639  2.59984  3.96086  4.28926
2.10962  3.86363  1.57724  2.70794  3.49355  4.32363  4.01253  3.90715
3.26824  2.55396  2.05842  4.81064  3.18643      6 l - - H
          2.68618  4.42225  2.77519  2.73123  3.46354  2.40513  3.72494
3.29354  2.67741  2.69355  4.24690  2.90347  2.73739  3.18146  2.89801
2.37887  2.77519  2.98518  4.58477  3.61503
```

```
          0.01309  4.73812  5.46046  0.61958  0.77255  0.48576  0.95510
      7   2.19211  5.27060  2.88850  2.17746  4.60863  3.27045  3.19510
4.08966  2.38899  3.57283  4.31326  3.02212  3.49266  2.44868  2.66339
2.28137  2.35091  3.65748  5.70744  4.30073      7 e - - H
          2.68618  4.42225  2.77519  2.73123  3.46354  2.40513  3.72494
3.29354  2.67741  2.69355  4.24690  2.90347  2.73739  3.18146  2.89801
2.37887  2.77519  2.98518  4.58477  3.61503
          0.01309  4.73812  5.46046  0.61958  0.77255  0.48576  0.95510
      8   1.96016  1.32405  4.63089  4.07275  3.17605  4.00939  4.52759
2.33403  3.93320  2.52582  3.53949  4.17230  4.44692  4.13114  4.06631
3.34981  3.18988  2.22110  5.14350  3.95317      8 c - - H
          2.68618  4.42225  2.77519  2.73123  3.46354  2.40513  3.72494
3.29354  2.67741  2.69355  4.24690  2.90347  2.73739  3.18146  2.89801
2.37887  2.77519  2.98518  4.58477  3.61503
          0.01309  4.73812  5.46046  0.61958  0.77255  0.48576  0.95510
      9   0.60856  4.62147  4.44696  4.06477  4.26198  3.67725  4.87285
3.10903  3.94766  2.75382  4.20242  4.10611  4.37472  4.24959  4.17422
3.12021  3.29921  2.58491  5.86866  4.65537      9 A - - H
          2.68618  4.42225  2.77519  2.73123  3.46354  2.40513  3.72494
3.29354  2.67741  2.69355  4.24690  2.90347  2.73739  3.18146  2.89801
2.37887  2.77519  2.98518  4.58477  3.61503
          0.01309  4.73812  5.46046  0.61958  0.77255  0.48576  0.95510
     10   2.57727  5.28692  2.46101  1.88770  4.62975  3.35266  3.74080
4.11442  2.31617  3.25151  4.32849  2.35386  3.94634  2.55834  2.56186
2.74315  2.63787  3.67642  5.72054  4.31040     10 e - - H
          2.68618  4.42225  2.77519  2.73123  3.46354  2.40513  3.72494
3.29354  2.67741  2.69355  4.24690  2.90347  2.73739  3.18146  2.89801
2.37887  2.77519  2.98518  4.58477  3.61503
          0.03415  4.73812  3.69607  0.61958  0.77255  0.48576  0.95510
     11   1.47248  3.42292  3.71074  2.82841  3.08716  3.76769  2.95006
2.94987  3.09000  2.56263  3.56518  3.53059  4.14649  3.38502  3.42791
2.83120  3.00578  2.60140  5.04542  3.51814     11 a - - H
          2.68618  4.42225  2.77519  2.73123  3.46354  2.40513  3.72494
3.29354  2.67741  2.69355  4.24690  2.90347  2.73739  3.18146  2.89801
2.37887  2.77519  2.98518  4.58477  3.61503
          0.01337  4.71733  5.43968  0.61958  0.77255  0.45939  0.99877
     12   3.08695  4.42303  5.01251  4.42761  2.79734  4.33909  4.71432
1.58839  4.25469  1.99311  3.26258  4.47654  4.66764  4.40376  4.30532
3.25144  3.32336  1.19448  5.18776  4.01323     12 v - - H
          2.68618  4.42225  2.77519  2.73123  3.46354  2.40513  3.72494
3.29354  2.67741  2.69355  4.24690  2.90347  2.73739  3.18146  2.89801
2.37887  2.77519  2.98518  4.58477  3.61503
          0.01309  4.73812  5.46046  0.61958  0.77255  0.48576  0.95510
     13   2.03332  5.26316  2.82799  2.10734  4.59361  3.55750  3.75467
4.06942  2.49944  3.56297  4.31049  2.93536  3.95643  2.46757  2.81619
1.98875  2.48248  3.64651  5.70641  4.30358     13 s - - H
          2.68618  4.42225  2.77519  2.73123  3.46354  2.40513  3.72494
3.29354  2.67741  2.69355  4.24690  2.90347  2.73739  3.18146  2.89801
2.37887  2.77519  2.98518  4.58477  3.61503
          0.01309  4.73812  5.46046  0.61958  0.77255  0.48576  0.95510
     14   2.25273  5.26934  2.67806  2.18786  4.60283  3.27911  3.74930
4.08133  2.43329  3.40351  4.31428  2.93076  3.95232  2.32519  2.98377
1.94028  2.53715  3.65400  5.70934  4.30414     14 s - - C
          2.68620  4.42227  2.77522  2.73126  3.46356  2.40501  3.72497
3.29356  2.67743  2.69357  4.24692  2.90326  2.73742  3.18149  2.89803
2.37889  2.77522  2.98521  4.58479  3.61505
          0.07715  3.03743  3.63858  0.65301  0.73497  0.48576  0.95510
```

```
     15   2.63713  5.25283  2.50212  2.23586  3.81003  2.23238  3.73874
4.06167  2.34088  3.55241  4.29740  2.48980  3.94245  2.83988  2.80185
2.24310  3.00434  3.63634  5.69339  3.98520     17 g - - T
          2.68618  4.42225  2.77519  2.73123  3.46354  2.40513  3.72494
3.29354  2.67741  2.69355  4.24690  2.90347  2.73739  3.18146  2.89801
2.37887  2.77519  2.98518  4.58477  3.61503
          0.01339  4.71583  5.43818  0.61958  0.77255  0.45762  1.00182
     16   2.74156  5.47201  1.72709  2.34314  4.80026  3.57738  3.52156
4.29425  2.37608  3.77031  4.52780  1.77408  4.03815  2.97605  3.17776
2.35794  3.18229  3.85566  5.90567  4.47155     18 d - - -
          2.68618  4.42225  2.77519  2.73123  3.46354  2.40513  3.72494
3.29354  2.67741  2.69355  4.24690  2.90347  2.73739  3.18146  2.89801
2.37887  2.77519  2.98518  4.58477  3.61503
          0.01309  4.73812  5.46046  0.61958  0.77255  0.48576  0.95510
     17   2.61430  4.60046  3.51684  2.95261  2.84283  3.72097  3.33519
2.91354  2.75151  1.75289  3.69807  3.01939  3.70650  2.95244  3.11518
2.74318  2.46877  2.88354  5.16835  3.91507     19 l - - H
          2.68618  4.42225  2.77519  2.73123  3.46354  2.40513  3.72494
3.29354  2.67741  2.69355  4.24690  2.90347  2.73739  3.18146  2.89801
2.37887  2.77519  2.98518  4.58477  3.61503
          0.01309  4.73812  5.46046  0.61958  0.77255  0.48576  0.95510
     18   2.29859  4.33605  2.94602  2.12204  4.57330  3.55567  3.74453
4.04747  2.43311  3.38101  3.99472  2.66622  3.94878  2.23884  2.57031
2.22330  2.65756  3.62664  5.68794  4.28750     20 e - - H
          2.68622  4.42229  2.77523  2.73115  3.46358  2.40517  3.72498
3.29358  2.67745  2.69359  4.24694  2.90351  2.73724  3.18150  2.89790
2.37891  2.77523  2.98498  4.58481  3.61507
          0.08055  2.61534  5.46046  0.66208  0.72522  0.48576  0.95510
     19   2.12847  4.10601  3.23704  2.44572  3.90270  3.67742  3.90240
2.98739  2.79346  2.01881  3.27649  2.66428  4.06268  3.12565  2.55075
2.89815  3.01064  2.91885  5.27414  3.99522     23 l - - H
          2.68618  4.42225  2.77519  2.73123  3.46354  2.40513  3.72494
3.29354  2.67741  2.69355  4.24690  2.90347  2.73739  3.18146  2.89801
2.37887  2.77519  2.98518  4.58477  3.61503
          0.01309  4.73812  5.46046  0.61958  0.77255  0.48576  0.95510
     20   0.80103  4.54099  4.29016  3.81359  4.11330  3.68363  4.63710
2.70426  3.73264  3.05419  4.04063  3.96271  4.31021  4.01194  4.00127
3.07981  2.48216  2.47433  5.63995  4.44016     24 a - - H
          2.68618  4.42225  2.77519  2.73123  3.46354  2.40513  3.72494
3.29354  2.67741  2.69355  4.24690  2.90347  2.73739  3.18146  2.89801
2.37887  2.77519  2.98518  4.58477  3.61503
          0.01309  4.73812  5.46046  0.61958  0.77255  0.48576  0.95510
     21   2.77634  5.27597  2.76840  2.28775  4.15692  3.33128  2.94889
4.09906  2.39812  3.38254  4.31793  2.28566  3.94472  2.05013  2.72666
2.46822  2.69734  3.66412  5.71133  4.30318     25 q - - H
          2.68618  4.42225  2.77519  2.73123  3.46354  2.40513  3.72494
3.29354  2.67741  2.69355  4.24690  2.90347  2.73739  3.18146  2.89801
2.37887  2.77519  2.98518  4.58477  3.61503
          0.01309  4.73812  5.46046  0.61958  0.77255  0.48576  0.95510
     22   2.07422  5.20913  2.93597  2.30260  4.52327  2.99197  3.12463
3.98797  2.36246  3.50112  4.25806  2.75605  3.95437  2.38897  2.86063
2.59634  2.87944  2.89661  4.31205  3.78235     26 a - - H
          2.68618  4.42225  2.77519  2.73123  3.46354  2.40513  3.72494
3.29354  2.67741  2.69355  4.24690  2.90347  2.73739  3.18146  2.89801
2.37887  2.77519  2.98518  4.58477  3.61503
          0.01309  4.73812  5.46046  0.61958  0.77255  0.48576  0.95510
```

```
      23   2.32073  3.85023  4.55946  3.95648  3.02337  3.93378  4.25692
 1.81816  3.29624  1.71315  3.00378  4.03116  4.29930  3.61643  3.85691
 3.23642  2.75733  2.31736  4.81174  2.98509      27 l - - H
           2.68618  4.42225  2.77519  2.73123  3.46354  2.40513  3.72494
 3.29354  2.67741  2.69355  4.24690  2.90347  2.73739  3.18146  2.89801
 2.37887  2.77519  2.98518  4.58477  3.61503
           0.01309  4.73812  5.46046  0.61958  0.77255  0.48576  0.95510
      24   3.88869  5.10944  5.80351  5.23427  3.48426  5.29416  5.63479
 2.18851  5.10746  0.59262  2.84954  5.43316  5.35863  5.03371  5.09373
 4.68183  4.10101  1.98678  5.69144  4.71793      28 L - - H
           2.68618  4.42225  2.77519  2.73123  3.46354  2.40513  3.72494
 3.29354  2.67741  2.69355  4.24690  2.90347  2.73739  3.18146  2.89801
 2.37887  2.77519  2.98518  4.58477  3.61503
           0.01309  4.73812  5.46046  0.61958  0.77255  0.48576  0.95510
      25   1.95865  3.99478  3.18103  2.21903  4.21139  3.43154  3.81421
 3.62746  2.11756  2.86652  4.04552  3.00606  3.99968  2.96526  3.07780
 2.32354  2.87588  3.30547  3.59337  4.14380      29 a - - H
           2.68618  4.42225  2.77519  2.73123  3.46354  2.40513  3.72494
 3.29354  2.67741  2.69355  4.24690  2.90347  2.73739  3.18146  2.89801
 2.37887  2.77519  2.98518  4.58477  3.61503
           0.01309  4.73812  5.46046  0.61958  0.77255  0.48576  0.95510
      26   2.78372  5.21234  2.93646  2.11070  4.52699  3.56830  3.57721
 3.18471  2.44058  3.50301  4.26178  3.04932  3.96084  2.08305  1.99210
 2.76191  2.78684  3.01028  5.66218  4.27393      30 r - - H
           2.68618  4.42225  2.77519  2.73123  3.46354  2.40513  3.72494
 3.29354  2.67741  2.69355  4.24690  2.90347  2.73739  3.18146  2.89801
 2.37887  2.77519  2.98518  4.58477  3.61503
           0.01309  4.73812  5.46046  0.61958  0.77255  0.48576  0.95510
      27   3.16221  4.51193  4.98611  4.41210  3.56317  4.40376  4.79571
 1.66505  4.25441  1.04198  3.46602  4.51510  4.72898  4.42099  4.34240
 3.39674  3.04524  1.93536  5.28529  4.12306      31 l - - H
           2.68618  4.42225  2.77519  2.73123  3.46354  2.40513  3.72494
 3.29354  2.67741  2.69355  4.24690  2.90347  2.73739  3.18146  2.89801
 2.37887  2.77519  2.98518  4.58477  3.61503
           0.01309  4.73812  5.46046  0.61958  0.77255  0.48576  0.95510
      28   2.71647  5.26154  3.04010  2.49477  4.59453  2.78359  3.51171
 4.06787  2.38382  3.56093  4.30977  2.18587  3.45250  2.77537  2.35922
 1.87694  3.02446  3.64663  5.70293  4.30464      32 s - - H
           2.68618  4.42225  2.77519  2.73123  3.46354  2.40513  3.72494
 3.29354  2.67741  2.69355  4.24690  2.90347  2.73739  3.18146  2.89801
 2.37887  2.77519  2.98518  4.58477  3.61503
           0.01309  4.73812  5.46046  0.61958  0.77255  0.48576  0.95510
      29   2.59852  5.24219  2.71266  1.96442  4.17694  3.39211  2.68877
 4.03928  2.49362  2.60339  4.28848  2.84011  3.95248  2.14748  2.98313
 2.75116  2.88119  3.62195  5.68691  4.28787      33 e - - H
           2.68618  4.42225  2.77519  2.73123  3.46354  2.40513  3.72494
 3.29354  2.67741  2.69355  4.24690  2.90347  2.73739  3.18146  2.89801
 2.37887  2.77519  2.98518  4.58477  3.61503
           0.01309  4.73812  5.46046  0.61958  0.77255  0.48576  0.95510
      30   2.67564  4.55870  3.57709  3.01107  3.70464  3.73900  3.22938
 2.63192  2.96804  1.69936  2.96077  2.62163  4.11991  3.07425  2.88455
 2.82906  2.77246  2.83748  5.13249  3.88795      34 l - - H
           2.68616  4.42231  2.77525  2.73129  3.46360  2.40519  3.72500
 3.29360  2.67747  2.69299  4.24695  2.90330  2.73745  3.18152  2.89807
 2.37893  2.77525  2.98524  4.58483  3.61509
           0.15520  1.96967  5.46046  0.43825  1.03610  0.48576  0.95510
```

```
     31   1.66462  4.10837  3.38700  2.83281  4.03530  3.62787  3.94234
3.42578  2.81208  3.08421  3.93170  2.93307  3.45842  3.14837  2.79931
2.00483  3.02337  2.44190  5.38357  4.09741      37 a - - -
          2.68618  4.42225  2.77519  2.73123  3.46354  2.40513  3.72494
3.29354  2.67741  2.69355  4.24690  2.90347  2.73739  3.18146  2.89801
2.37887  2.77519  2.98518  4.58477  3.61503
          0.01309  4.73812  5.46046  0.61958  0.77255  0.48576  0.95510
     32   2.38171  4.80102  3.47956  2.98172  4.48619  3.50036  4.14409
3.90996  2.98775  3.52972  4.33597  2.60763  3.41600  3.31945  3.41870
1.08459  2.63595  3.03830  5.76447  4.46675      38 s - - -
          2.68618  4.42225  2.77519  2.73123  3.46354  2.40513  3.72494
3.29354  2.67741  2.69355  4.24690  2.90347  2.73739  3.18146  2.89801
2.37887  2.77519  2.98518  4.58477  3.61503
          0.01309  4.73812  5.46046  0.61958  0.77255  0.48576  0.95510
     33   2.63639  4.87679  3.25265  2.58801  4.09631  3.63389  3.85357
3.16440  2.68271  2.87280  3.96422  2.89003  1.77084  3.02948  2.99737
2.19465  3.01536  2.97679  5.40684  4.09622      39 p - - T
          2.68618  4.42225  2.77519  2.73123  3.46354  2.40513  3.72494
3.29354  2.67741  2.69355  4.24690  2.90347  2.73739  3.18146  2.89801
2.37887  2.77519  2.98518  4.58477  3.61503
          0.01309  4.73812  5.46046  0.61958  0.77255  0.48576  0.95510
     34   2.34491  5.20367  2.28823  2.36429  3.31564  3.28781  3.09978
3.97897  2.50261  3.27854  4.25317  2.92561  3.95530  2.40704  2.81854
2.41411  3.00677  3.32909  5.65666  3.46655      40 d - - T
          2.68618  4.42225  2.77519  2.73123  3.46354  2.40513  3.72494
3.29354  2.67741  2.69355  4.24690  2.90347  2.73739  3.18146  2.89801
2.37887  2.77519  2.98518  4.58477  3.61503
          0.01309  4.73812  5.46046  0.61958  0.77255  0.48576  0.95510
     35   2.54308  5.10139  2.54347  2.69415  4.54481  1.14476  3.99161
3.66191  2.81341  3.56237  4.37007  3.20435  4.08032  3.14347  2.90627
2.90390  3.16353  3.59267  5.77641  4.42392      41 g - - S
          2.68621  4.42229  2.77523  2.73127  3.46357  2.40516  3.72498
3.29357  2.67744  2.69358  4.24693  2.90350  2.73731  3.18150  2.89775
2.37890  2.77503  2.98522  4.58480  3.61507
          0.13737  2.08666  5.46046  0.22737  1.59270  0.48576  0.95510
     36   2.83333  5.34185  1.69939  2.29994  4.67828  2.85029  3.78329
4.16413  2.32876  3.64365  4.38885  2.56376  3.97840  2.88640  2.84474
2.58162  2.44478  3.72864  5.77736  4.36130      43 d - - -
          2.68624  4.42231  2.77526  2.73129  3.46360  2.40519  3.72501
3.29360  2.67729  2.69361  4.24696  2.90307  2.73707  3.18152  2.89807
2.37893  2.77526  2.98524  4.58483  3.61509
          0.05362  3.03743  5.46046  1.18082  0.36677  0.48576  0.95510
     37   1.99008  4.80923  3.49783  3.00619  4.60796  2.77962  4.18214
4.05058  3.01909  3.64968  4.43897  3.04618  1.85870  3.34772  3.45434
1.84272  2.16995  3.57755  5.85979  4.55647      49 s - - H
          2.68618  4.42225  2.77519  2.73123  3.46354  2.40513  3.72494
3.29354  2.67741  2.69355  4.24690  2.90347  2.73739  3.18146  2.89801
2.37887  2.77519  2.98518  4.58477  3.61503
          0.01309  4.73812  5.46046  0.61958  0.77255  0.48576  0.95510
     38   2.69744  4.41218  3.82529  2.70896  3.09900  3.80239  4.07130
2.60303  3.19033  2.27202  2.07313  3.03925  3.77272  3.47159  3.49922
2.92131  2.17273  2.59975  5.00596  3.78813      50 m - - H
          2.68618  4.42225  2.77519  2.73123  3.46354  2.40513  3.72494
3.29354  2.67741  2.69355  4.24690  2.90347  2.73739  3.18146  2.89801
2.37887  2.77519  2.98518  4.58477  3.61503
          0.01309  4.73812  5.46046  0.61958  0.77255  0.48576  0.95510
```

```
     39   2.84273  5.31676  3.05298  1.88352  4.66100  3.22098  3.03991
4.13220  2.47514  3.61181  4.36352  3.06416  3.99286  1.67005  2.41500
2.80693  2.87138  3.70833  5.74001  4.34935     51 q - - H
          2.68618  4.42225  2.77519  2.73123  3.46354  2.40513  3.72494
3.29354  2.67741  2.69355  4.24690  2.90347  2.73739  3.18146  2.89801
2.37887  2.77519  2.98518  4.58477  3.61503
          0.01309  4.73812  5.46046  0.61958  0.77255  0.48576  0.95510
     40   3.83749  5.82452  4.51046  3.55320  5.48153  4.32107  4.05318
4.72132  1.52570  4.02776  4.93654  3.84118  4.63476  3.18088  0.66745
3.80841  3.92055  4.42519  5.94729  4.91251     52 R - - H
          2.68618  4.42225  2.77519  2.73123  3.46354  2.40513  3.72494
3.29354  2.67741  2.69355  4.24690  2.90347  2.73739  3.18146  2.89801
2.37887  2.77519  2.98518  4.58477  3.61503
          0.01309  4.73812  5.46046  0.61958  0.77255  0.48576  0.95510
     41   2.28654  4.61072  5.18875  4.63117  3.69950  4.62212  5.05725
1.77250  4.49380  1.23780  3.55836  4.74220  4.91962  4.65834  4.58615
3.96859  3.54445  1.46067  5.50603  4.34507     53 l - - H
          2.68618  4.42225  2.77519  2.73123  3.46354  2.40513  3.72494
3.29354  2.67741  2.69355  4.24690  2.90347  2.73739  3.18146  2.89801
2.37887  2.77519  2.98518  4.58477  3.61503
          0.01309  4.73812  5.46046  0.61958  0.77255  0.48576  0.95510
     42   1.03884  4.47513  4.12102  3.60932  3.90864  2.83970  4.41839
2.77980  3.54116  2.95106  3.87183  3.82313  4.23336  3.81226  3.82165
3.02708  2.79799  2.06181  5.39011  4.18509     54 a - - H
          2.68618  4.42225  2.77519  2.73123  3.46354  2.40513  3.72494
3.29354  2.67741  2.69355  4.24690  2.90347  2.73739  3.18146  2.89801
2.37887  2.77519  2.98518  4.58477  3.61503
          0.01309  4.73812  5.46046  0.61958  0.77255  0.48576  0.95510
     43   1.66610  4.73580  3.04651  2.80789  2.77071  3.67351  2.95226
3.30112  2.78916  2.96974  3.82771  3.28694  4.06099  3.12154  3.21324
2.35748  2.73657  2.90418  5.28376  3.70771     55 a - - H
          2.68618  4.42225  2.77519  2.73123  3.46354  2.40513  3.72494
3.29354  2.67741  2.69355  4.24690  2.90347  2.73739  3.18146  2.89801
2.37887  2.77519  2.98518  4.58477  3.61503
          0.01309  4.73812  5.46046  0.61958  0.77255  0.48576  0.95510
     44   2.68982  3.82912  4.25613  3.68805  2.60400  3.98080  3.11192
2.86745  3.57425  2.33634  3.52244  3.89992  4.34738  3.45499  3.77557
3.26532  3.14687  2.67394  4.70879  1.28711     56 y - - H
          2.68618  4.42225  2.77519  2.73123  3.46354  2.40513  3.72494
3.29354  2.67741  2.69355  4.24690  2.90347  2.73739  3.18146  2.89801
2.37887  2.77519  2.98518  4.58477  3.61503
          0.01309  4.73812  5.46046  0.61958  0.77255  0.48576  0.95510
     45   2.94939  4.35099  4.64734  4.06287  1.16844  3.43572  4.29294
2.45076  3.89582  2.28024  2.61828  4.14216  4.43534  4.05186  3.98110
3.38889  3.18029  2.55800  4.77102  3.11361     57 f - - H
          2.68618  4.42225  2.77519  2.73123  3.46354  2.40513  3.72494
3.29354  2.67741  2.69355  4.24690  2.90347  2.73739  3.18146  2.89801
2.37887  2.77519  2.98518  4.58477  3.61503
          0.01309  4.73812  5.46046  0.61958  0.77255  0.48576  0.95510
     46   2.18396  4.52381  3.63698  3.07122  3.67157  3.28612  4.01196
2.82838  2.54727  2.12774  3.63302  3.48284  4.13589  3.33277  3.38531
2.89334  1.83270  2.50680  5.11116  3.87357     58 t - - H
          2.68618  4.42225  2.77519  2.73123  3.46354  2.40513  3.72494
3.29354  2.67741  2.69355  4.24690  2.90347  2.73739  3.18146  2.89801
2.37887  2.77519  2.98518  4.58477  3.61503
          0.01309  4.73812  5.46046  0.61958  0.77255  0.48576  0.95510
```

47 2.64219 5.33339 3.03458 1.33361 4.67920 3.59215 3.78060
4.15282 2.29373 3.62921 4.38005 2.73918 3.99624 2.56238 2.47816
2.81242 2.93872 3.72574 5.75538 4.36125 59 e - - H
2.68618 4.42225 2.77519 2.73123 3.46354 2.40513 3.72494
3.29354 2.67741 2.69355 4.24690 2.90347 2.73739 3.18146 2.89801
2.37887 2.77519 2.98518 4.58477 3.61503
0.01309 4.73812 5.46046 0.61958 0.77255 0.48576 0.95510
48 0.50414 4.63479 4.34912 4.21946 5.17083 2.01319 5.17284
4.60281 4.32979 4.32559 5.13191 4.07413 4.24628 4.52699 4.51430
2.92246 3.27592 3.87892 6.49350 5.39324 60 A - - H
2.68618 4.42225 2.77519 2.73123 3.46354 2.40513 3.72494
3.29354 2.67741 2.69355 4.24690 2.90347 2.73739 3.18146 2.89801
2.37887 2.77519 2.98518 4.58477 3.61503
0.01309 4.73812 5.46046 0.61958 0.77255 0.48576 0.95510
49 4.17020 5.38184 5.90159 5.37005 3.40721 5.48607 5.75505
2.64896 5.13963 0.37081 2.79712 5.63127 5.48875 5.07877 5.11767
4.93775 4.37782 2.94919 5.69666 4.71312 61 L - - H
2.68618 4.42225 2.77519 2.73123 3.46354 2.40513 3.72494
3.29354 2.67741 2.69355 4.24690 2.90347 2.73739 3.18146 2.89801
2.37887 2.77519 2.98518 4.58477 3.61503
0.01309 4.73812 5.46046 0.61958 0.77255 0.48576 0.95510
50 1.93363 5.10752 3.10414 2.32897 3.68855 3.28763 3.29591
3.82636 2.54504 3.11766 4.16668 2.92158 3.97248 2.49786 2.79847
2.13982 3.00773 3.07409 5.58277 4.21667 62 a - - H
2.68618 4.42225 2.77519 2.73123 3.46354 2.40513 3.72494
3.29354 2.67741 2.69355 4.24690 2.90347 2.73739 3.18146 2.89801
2.37887 2.77519 2.98518 4.58477 3.61503
0.01309 4.73812 5.46046 0.61958 0.77255 0.48576 0.95510
51 2.10688 5.08170 3.12241 2.56534 4.35192 3.58870 3.37930
3.78685 2.44502 2.80639 4.14443 2.26009 3.98015 2.91488 2.49894
2.22370 2.88050 3.02637 5.56301 4.20463 63 a - - H
2.68618 4.42225 2.77519 2.73123 3.46354 2.40513 3.72494
3.29354 2.67741 2.69355 4.24690 2.90347 2.73739 3.18146 2.89801
2.37887 2.77519 2.98518 4.58477 3.61503
0.01309 4.73812 5.46046 0.61958 0.77255 0.48576 0.95510
52 3.00995 5.04628 3.53682 2.92355 3.57052 3.82103 3.35745
3.66480 2.51780 2.53119 4.12040 3.38229 4.19186 2.75803 1.13539
3.07503 3.21995 3.38706 5.42558 4.11357 64 r - - H
2.68618 4.42225 2.77519 2.73123 3.46354 2.40513 3.72494
3.29354 2.67741 2.69355 4.24690 2.90347 2.73739 3.18146 2.89801
2.37887 2.77519 2.98518 4.58477 3.61503
0.01309 4.73812 5.46046 0.61958 0.77255 0.48576 0.95510
53 2.99742 4.69777 5.36142 4.79439 3.60503 4.75020 5.15440
1.41102 4.64748 1.18713 2.76481 4.88987 5.00263 4.74915 4.69396
4.10106 3.64620 1.75084 5.50527 4.38745 65 l - - H
2.68618 4.42225 2.77519 2.73123 3.46354 2.40513 3.72494
3.29354 2.67741 2.69355 4.24690 2.90347 2.73739 3.18146 2.89801
2.37887 2.77519 2.98518 4.58477 3.61503
0.01309 4.73812 5.46046 0.61958 0.77255 0.48576 0.95510
54 2.06836 4.57805 3.54518 2.80250 3.11132 3.72901 3.59364
2.94383 2.94557 2.30499 3.67693 2.85152 4.11064 2.92293 3.32811
2.63181 2.45124 2.48282 5.14923 3.31809 66 a - - C
2.68618 4.42225 2.77519 2.73123 3.46354 2.40513 3.72494
3.29354 2.67741 2.69355 4.24690 2.90347 2.73739 3.18146 2.89801
2.37887 2.77519 2.98518 4.58477 3.61503
0.03415 4.73812 3.69607 0.61958 0.77255 0.48576 0.95510

55 2.61162 5.26766 2.76023 2.47699 4.60658 2.52614 3.74500
4.08518 2.16491 3.57133 4.31547 2.78595 3.65910 2.84579 2.03885
2.11941 3.01493 3.65619 5.70812 4.30397 67 r - - C
2.68621 4.42229 2.77523 2.73127 3.46357 2.40516 3.72498
3.29357 2.67744 2.69358 4.24693 2.90340 2.73743 3.18116 2.89804
2.37884 2.77515 2.98522 4.58480 3.61484
0.50947 2.06465 1.30078 0.22718 1.59344 0.52943 0.88901
56 2.66829 4.66592 3.21744 2.65729 3.85517 3.54980 3.76936
2.91164 2.47591 2.90173 2.95346 3.14482 3.38125 2.65866 3.06257
1.89499 2.75614 2.96990 5.20584 3.57705 69 s - - H
2.68618 4.42225 2.77519 2.73123 3.46354 2.40513 3.72494
3.29354 2.67741 2.69355 4.24690 2.90347 2.73739 3.18146 2.89801
2.37887 2.77519 2.98518 4.58477 3.61503
0.01831 4.40539 5.12773 0.61958 0.77255 0.96461 0.47986
57 2.65812 3.67567 3.60529 3.03739 3.48599 2.54886 3.92130
2.84500 2.98148 2.45686 3.46017 3.42211 4.03316 2.84441 3.31747
2.63788 2.57583 2.42810 2.66107 3.71310 70 v - - H
2.68618 4.42225 2.77519 2.73123 3.46354 2.40513 3.72494
3.29354 2.67741 2.69355 4.24690 2.90347 2.73739 3.18146 2.89801
2.37887 2.77519 2.98518 4.58477 3.61503
0.01831 4.40539 5.12773 0.61958 0.77255 0.85999 0.55020
58 2.70912 5.09179 2.63076 2.27248 4.39211 3.47531 3.69307
3.84518 2.37966 3.09286 4.15586 2.96763 2.32750 2.81177 2.94590
2.09705 2.60425 3.46025 5.56224 4.18062 71 s - - H
2.68618 4.42225 2.77519 2.73123 3.46354 2.40513 3.72494
3.29354 2.67741 2.69355 4.24690 2.90347 2.73739 3.18146 2.89801
2.37887 2.77519 2.98518 4.58477 3.61503
0.01754 4.44808 5.17042 0.61958 0.77255 0.92167 0.50726
59 2.68532 5.08948 2.97246 2.27902 3.95615 2.49172 3.08485
3.85106 2.41881 3.37712 4.14223 2.95985 3.38060 2.43514 2.67791
2.32266 2.61682 3.45824 5.54785 4.16407 72 e - - C
2.68618 4.42225 2.77519 2.73123 3.46354 2.40513 3.72494
3.29354 2.67741 2.69355 4.24690 2.90347 2.73739 3.18146 2.89801
2.37887 2.77519 2.98518 4.58477 3.61503
0.01754 4.44808 5.17042 0.61958 0.77255 0.92167 0.50726
60 2.65695 3.37704 4.61319 4.02227 3.31147 4.00972 4.36028
1.74350 3.85875 1.65413 2.97827 4.10900 4.36064 4.02999 3.94285
3.32216 2.49940 1.92363 4.89939 3.71959 73 l - - H
2.68618 4.42225 2.77519 2.73123 3.46354 2.40513 3.72494
3.29354 2.67741 2.69355 4.24690 2.90347 2.73739 3.18146 2.89801
2.37887 2.77519 2.98518 4.58477 3.61503
0.01754 4.44808 5.17042 0.61958 0.77255 0.83291 0.57055
61 2.42140 3.79169 3.81676 3.25184 2.83077 3.71336 4.00366
2.80229 3.18027 2.53007 3.43962 3.29013 4.10767 3.45205 3.46831
2.22548 2.94040 2.58962 4.86204 1.88277 74 y - - H
2.68618 4.42225 2.77519 2.73123 3.46354 2.40513 3.72494
3.29354 2.67741 2.69355 4.24690 2.90347 2.73739 3.18146 2.89801
2.37887 2.77519 2.98518 4.58477 3.61503
0.06253 4.48264 3.00968 0.61958 0.77255 0.60854 0.78559
62 2.50030 5.22568 2.63912 2.23849 4.56550 3.49302 3.15471
4.04739 1.92904 3.52773 4.26882 2.68230 3.89096 2.78501 2.59705
2.09271 2.95917 3.61372 5.66004 4.25249 75 k - - H
2.68618 4.42225 2.77519 2.73123 3.46354 2.40513 3.72494
3.29354 2.67741 2.69355 4.24690 2.90347 2.73739 3.18146 2.89801
2.37887 2.77519 2.98518 4.58477 3.61503
0.03591 4.55243 3.69959 0.61958 0.77255 0.79894 0.59748

```
     63   1.97276  4.61913  3.34388  2.78448  3.49985  3.32785  3.85600
2.76856  2.76075  2.87142  3.73435  3.24934  3.23602  3.08985  2.93687
2.24434  2.31248  2.93219  5.19534  3.92302      76 a - - H
          2.68618  4.42225  2.77519  2.73123  3.46354  2.40513  3.72494
3.29354  2.67741  2.69355  4.24690  2.90347  2.73739  3.18146  2.89801
2.37887  2.77519  2.98518  4.58477  3.61503
          0.01610  4.53262  5.25497  0.61958  0.77255  0.58900  0.80941
     64   2.57177  4.16053  4.44142  3.84265  2.57999  3.44854  4.19776
2.21749  3.68145  1.58564  2.77891  3.47016  4.24488  3.86121  3.37288
3.17795  2.97249  2.10604  4.77857  3.59575      77 l - - H
          2.68621  4.42228  2.77522  2.73126  3.46357  2.40516  3.72497
3.29357  2.67744  2.69358  4.24693  2.90342  2.73736  3.18149  2.89804
2.37876  2.77522  2.98521  4.58383  3.61506
          0.13216  2.12846  5.34338  0.25246  1.50009  0.46166  0.99489
     65   2.53576  5.10438  3.08849  2.53172  4.38530  3.56749  3.56173
3.82804  2.45252  3.14229  4.16275  2.55560  2.33858  2.65338  2.63248
2.38678  2.76606  2.82269  5.57744  3.93746      79 p - - H
          2.68618  4.42225  2.77519  2.73123  3.46354  2.40513  3.72494
3.29354  2.67741  2.69355  4.24690  2.90347  2.73739  3.18146  2.89801
2.37887  2.77519  2.98518  4.58477  3.61503
          0.01356  4.70359  5.42594  0.61958  0.77255  0.55683  0.85102
     66   2.33144  3.26024  3.55679  2.85141  3.31852  3.51798  3.97061
2.86324  2.95546  2.77117  3.65469  3.19547  2.32435  3.26740  3.33255
2.37128  2.38651  2.63938  5.12821  3.88235      80 p - - H
          2.68618  4.42225  2.77519  2.73123  3.46354  2.40513  3.72494
3.29354  2.67741  2.69355  4.24690  2.90347  2.73739  3.18146  2.89801
2.37887  2.77519  2.98518  4.58477  3.61503
          0.01356  4.70359  5.42594  0.61958  0.77255  0.55683  0.85102
     67   2.57771  4.94234  3.18064  2.62226  3.61000  3.60180  3.80694
3.58701  2.31053  2.96531  4.01499  3.13888  3.41653  2.62005  2.30520
2.20264  2.50493  2.96233  3.86966  4.11990      81 s - - H
          2.68618  4.42225  2.77519  2.73123  3.46354  2.40513  3.72494
3.29354  2.67741  2.69355  4.24690  2.90347  2.73739  3.18146  2.89801
2.37887  2.77519  2.98518  4.58477  3.61503
          0.01356  4.70359  5.42594  0.61958  0.77255  0.55683  0.85102
     68   2.44065  5.18023  2.86795  2.08759  4.48784  3.55287  3.74458
3.94811  2.49571  2.90358  4.23109  3.03657  2.68128  2.27847  2.87124
2.61127  2.39216  3.55061  4.77480  4.24901      82 e - - H
          2.68618  4.42225  2.77519  2.73123  3.46354  2.40513  3.72494
3.29354  2.67741  2.69355  4.24690  2.90347  2.73739  3.18146  2.89801
2.37887  2.77519  2.98518  4.58477  3.61503
          0.01356  4.70359  5.42594  0.61958  0.77255  0.55683  0.85102
     69   2.36553  4.94284  2.82614  2.62111  3.30135  3.60095  3.80669
3.58814  2.61344  2.72947  3.76990  3.13795  3.06182  2.63397  2.92552
2.30658  2.13951  3.27130  5.45024  4.12026      83 t - - T
          2.68618  4.42225  2.77519  2.73123  3.46354  2.40513  3.72494
3.29354  2.67741  2.69355  4.24690  2.90347  2.73739  3.18146  2.89801
2.37887  2.77519  2.98518  4.58477  3.61503
          0.05723  4.70359  3.06707  0.61958  0.77255  0.55683  0.85102
     70   2.34749  5.21365  2.83256  2.40210  4.53947  3.53088  3.39839
4.01145  2.46701  3.51129  4.26129  2.60508  2.95817  2.82682  2.48503
2.14655  2.31288  3.59431  5.65969  4.26112      84 s - - S
          2.68618  4.42225  2.77519  2.73123  3.46354  2.40513  3.72494
3.29354  2.67741  2.69355  4.24690  2.90347  2.73739  3.18146  2.89801
2.37887  2.77519  2.98518  4.58477  3.61503
          0.20732  4.66052  1.72723  0.61958  0.77255  0.63593  0.75383
```

```
     71   2.61532  5.10222  2.63346  2.12238  4.40882  3.47800  3.67025
3.86835  2.42202  3.39099  4.15359  2.96232  2.95215  2.69930  2.59365
2.29597  2.44049  3.25925  4.25922  4.17259      85 e - - C
          2.68618  4.42225  2.77519  2.73123  3.46354  2.40513  3.72494
3.29354  2.67741  2.69355  4.24690  2.90347  2.73739  3.18146  2.89801
2.37887  2.77519  2.98518  4.58477  3.61503
          0.06416  4.47035  2.98178  0.61958  0.77255  0.89774  0.52339
     72   2.67572  5.10628  2.63453  2.28267  4.41929  3.46159  3.65267
3.88318  2.25989  3.39769  3.40481  2.44993  3.85461  2.54164  2.79705
2.31301  2.55246  3.26326  5.55852  3.72199      86 k - - H
          2.68618  4.42225  2.77519  2.73123  3.46354  2.40513  3.72494
3.29354  2.67741  2.69355  4.24690  2.90347  2.73739  3.18146  2.89801
2.37887  2.77519  2.98518  4.58477  3.61503
          0.01796  4.42415  5.14650  0.61958  0.77255  0.94619  0.49138
     73   2.54595  5.08793  2.52304  2.29818  4.39162  3.46526  3.47081
3.84976  2.41798  2.97424  4.14070  2.28039  3.86118  2.65986  2.90337
2.44408  2.45159  3.16738  5.54644  4.16100      87 n - - C
          2.68637  4.42152  2.77525  2.73142  3.46283  2.40514  3.72475
3.29373  2.67759  2.69374  4.24610  2.90340  2.73758  3.18165  2.89803
2.37837  2.77538  2.98521  4.58496  3.61492
          0.33970  1.26514  5.14650  1.02011  0.44715  0.27813  1.41553
     74   2.58805  4.69927  3.40202  2.50288  3.87119  2.94202  3.68501
3.01925  2.81738  2.40471  3.34438  3.31169  3.79918  2.94394  3.23365
2.02877  2.75002  2.32865  5.25213  3.97861      91 s - - H
          2.68618  4.42225  2.77519  2.73123  3.46354  2.40513  3.72494
3.29354  2.67741  2.69355  4.24690  2.90347  2.73739  3.18146  2.89801
2.37887  2.77519  2.98518  4.58477  3.61503
          0.01309  4.73812  5.46046  0.61958  0.77255  0.48576  0.95510
     75   2.35069  4.88885  2.73994  2.57292  4.10321  3.45844  3.84050
2.62189  2.50084  2.79226  3.96675  3.18609  3.77181  2.87908  3.11968
2.20587  2.55938  2.61445  5.40880  3.81644      92 s - - H
          2.68618  4.42225  2.77519  2.73123  3.46354  2.40513  3.72494
3.29354  2.67741  2.69355  4.24690  2.90347  2.73739  3.18146  2.89801
2.37887  2.77519  2.98518  4.58477  3.61503
          0.01309  4.73812  5.46046  0.61958  0.77255  0.48576  0.95510
     76   2.66917  5.21986  2.18094  1.79457  3.42490  3.56055  3.75172
4.00422  2.36726  3.37148  4.26803  3.03940  3.95408  2.57845  2.89611
2.65138  2.78425  2.92772  5.66938  4.27556      93 e - - H
          2.68618  4.42225  2.77519  2.73123  3.46354  2.40513  3.72494
3.29354  2.67741  2.69355  4.24690  2.90347  2.73739  3.18146  2.89801
2.37887  2.77519  2.98518  4.58477  3.61503
          0.01309  4.73812  5.46046  0.61958  0.77255  0.48576  0.95510
     77   2.50667  4.91027  3.01578  2.19087  3.27418  3.62457  3.23551
3.02896  2.54394  2.70246  3.74835  3.17412  3.75873  2.99946  2.42864
2.61214  2.83332  2.62113  5.42613  4.10651      94 e - - H
          2.68618  4.42225  2.77519  2.73123  3.46354  2.40513  3.72494
3.29354  2.67741  2.69355  4.24690  2.90347  2.73739  3.18146  2.89801
2.37887  2.77519  2.98518  4.58477  3.61503
          0.04898  4.73812  3.24295  0.61958  0.77255  0.48576  0.95510
     78   2.28804  4.29972  4.05913  3.26233  3.19403  3.42370  4.13204
1.79051  3.05511  1.85880  3.41277  3.75301  4.21392  3.63229  3.62067
2.78800  2.68121  2.48308  4.90632  3.70570      95 i - - H
          2.68618  4.42225  2.77519  2.73123  3.46354  2.40513  3.72494
3.29354  2.67741  2.69355  4.24690  2.90347  2.73739  3.18146  2.89801
2.37887  2.77519  2.98518  4.58477  3.61503
          0.01357  4.70270  5.42505  0.61958  0.77255  0.55856  0.84871
```

```
     79   2.21167  4.71376  3.36115  2.79975  3.11942  3.66345  2.80976
2.97610  2.38007  2.61238  3.12495  3.03875  4.04871  3.11077  2.97748
2.24384  2.89740  3.01092  5.26197  3.98270     96 a - - H
          2.68618  4.42225  2.77519  2.73123  3.46354  2.40513  3.72494
3.29354  2.67741  2.69355  4.24690  2.90347  2.73739  3.18146  2.89801
2.37887  2.77519  2.98518  4.58477  3.61503
          0.01357  4.70270  5.42505  0.61958  0.77255  0.55856  0.84871
     80   1.54565  4.39468  3.83180  3.11784  3.15388  3.79177  4.06937
2.73488  3.19620  2.60112  3.50683  3.61149  4.17138  3.47589  3.50051
2.20430  3.00463  2.24566  4.99288  3.21529     97 a - - H
          2.68618  4.42225  2.77519  2.73123  3.46354  2.40513  3.72494
3.29354  2.67741  2.69355  4.24690  2.90347  2.73739  3.18146  2.89801
2.37887  2.77519  2.98518  4.58477  3.61503
          0.04413  4.70270  3.37854  0.61958  0.77255  0.55856  0.84871
     81   2.75493  4.55477  2.91387  2.84921  2.94406  3.70311  3.59860
2.86654  2.91804  2.40110  3.13310  3.38966  4.08478  2.81811  2.64431
2.93808  2.74706  2.49708  5.12562  2.56075     98 l - - H
          2.68643  4.42133  2.77522  2.73105  3.46244  2.40538  3.72520
3.29379  2.67725  2.69359  4.24715  2.90295  2.73765  3.18138  2.89800
2.37882  2.77522  2.98544  4.58502  3.61528
          0.08356  2.86862  3.75544  2.25613  0.11066  0.49901  0.93429
     82   2.77148  3.81047  3.07062  2.40269  4.46923  3.56169  3.39657
3.68830  1.86632  3.11203  4.21822  3.04887  3.95293  2.29894  2.14911
2.65131  2.82102  3.53392  5.62110  3.92859    117 k - - H
          2.68618  4.42225  2.77519  2.73123  3.46354  2.40513  3.72494
3.29354  2.67741  2.69355  4.24690  2.90347  2.73739  3.18146  2.89801
2.37887  2.77519  2.98518  4.58477  3.61503
          0.01373  4.69070  5.41304  0.61958  0.77255  0.52112  0.90104
     83   2.53417  4.22882  4.31497  3.34014  3.05576  3.59406  4.19641
2.28066  3.59159  1.57842  3.34454  3.89982  4.25631  3.79853  3.74584
2.82463  2.35544  2.21766  4.84274  3.10194    118 l - - H
          2.68623  4.42246  2.77541  2.73144  3.46375  2.40534  3.72343
3.29364  2.67731  2.69300  4.24649  2.90359  2.73761  3.18136  2.89822
2.37901  2.77541  2.98518  4.58360  3.61428
          0.27769  1.43509  5.43139  1.02764  0.44293  0.44980  1.01544
     84   3.98552  5.19968  5.43592  5.05398  0.98204  4.96266  4.25032
3.25021  4.86238  1.22672  3.78401  4.79364  5.20139  4.75582  4.80550
4.32463  4.19713  3.33657  4.37199  2.30423    122 f - - H
          2.68618  4.42225  2.77519  2.73123  3.46354  2.40513  3.72494
3.29354  2.67741  2.69355  4.24690  2.90347  2.73739  3.18146  2.89801
2.37887  2.77519  2.98518  4.58477  3.61503
          0.01309  4.73812  5.46046  0.61958  0.77255  0.48576  0.95510
     85   2.79052  4.64391  3.48226  2.92054  2.75573  3.71692  3.68697
3.17740  2.89063  2.73549  3.73997  2.24917  4.10005  2.75095  3.28902
2.33256  3.02209  2.55581  5.17301  2.37174    123 n - - H
          2.68618  4.42225  2.77519  2.73123  3.46354  2.40513  3.72494
3.29354  2.67741  2.69355  4.24690  2.90347  2.73739  3.18146  2.89801
2.37887  2.77519  2.98518  4.58477  3.61503
          0.01309  4.73812  5.46046  0.61958  0.77255  0.48576  0.95510
     86   2.59620  5.41649  2.48903  1.39808  4.75053  2.98066  3.54145
4.24268  2.60380  3.71726  4.46684  2.66206  4.00879  2.31935  3.11238
2.70903  3.12776  3.80250  5.84944  4.42194    124 e - - H
          2.68618  4.42225  2.77519  2.73123  3.46354  2.40513  3.72494
3.29354  2.67741  2.69355  4.24690  2.90347  2.73739  3.18146  2.89801
2.37887  2.77519  2.98518  4.58477  3.61503
          0.01309  4.73812  5.46046  0.61958  0.77255  0.48576  0.95510
```

```
     87   2.40703  3.74426  4.62488  4.02191  3.30882  3.96944  4.30036
2.03087  3.84153  1.97491  2.47253  4.08082  4.33335  4.00284  3.90557
2.73677  2.54620  1.78063  4.84125  3.66093    125 v - - H
          2.68618  4.42225  2.77519  2.73123  3.46354  2.40513  3.72494
3.29354  2.67741  2.69355  4.24690  2.90347  2.73739  3.18146  2.89801
2.37887  2.77519  2.98518  4.58477  3.61503
          0.01309  4.73812  5.46046  0.61958  0.77255  0.48576  0.95510
     88   2.73231  1.98869  4.22040  3.69578  2.64315  3.66492  4.37509
3.07842  3.60304  2.82951  3.73860  3.85603  4.21693  3.84784  3.83409
1.58065  2.07450  2.82630  5.21556  4.00975    126 s - - S
          2.68618  4.42225  2.77519  2.73123  3.46354  2.40513  3.72494
3.29354  2.67741  2.69355  4.24690  2.90347  2.73739  3.18146  2.89801
2.37887  2.77519  2.98518  4.58477  3.61503
          0.01309  4.73812  5.46046  0.61958  0.77255  0.48576  0.95510
     89   4.24998  5.77383  4.94031  4.93600  5.75108  4.41859  5.85863
5.62714  5.08563  5.09807  6.22786  5.13598  0.12068  5.43416  5.15999
4.45838  4.75755  5.15249  6.60716  5.90339    127 P - - S
          2.68618  4.42225  2.77519  2.73123  3.46354  2.40513  3.72494
3.29354  2.67741  2.69355  4.24690  2.90347  2.73739  3.18146  2.89801
2.37887  2.77519  2.98518  4.58477  3.61503
          0.01309  4.73812  5.46046  0.61958  0.77255  0.48576  0.95510
     90   2.80778  3.27595  4.73404  4.12409  2.30754  3.97228  4.28401
1.80716  3.91649  2.21805  3.29767  4.11989  4.33473  4.05066  3.93524
3.28280  2.89963  2.32186  2.98942  2.22850    128 i - - H
          2.68618  4.42225  2.77519  2.73123  3.46354  2.40513  3.72494
3.29354  2.67741  2.69355  4.24690  2.90347  2.73739  3.18146  2.89801
2.37887  2.77519  2.98518  4.58477  3.61503
          0.01309  4.73812  5.46046  0.61958  0.77255  0.48576  0.95510
     91   2.56992  4.22724  4.38560  3.79197  2.42457  3.90871  3.61610
2.25974  3.64890  1.62097  3.34317  3.94288  2.85323  3.84608  3.46662
3.00738  3.01709  2.24389  4.84174  3.41057    129 l - - H
          2.68618  4.42225  2.77519  2.73123  3.46354  2.40513  3.72494
3.29354  2.67741  2.69355  4.24690  2.90347  2.73739  3.18146  2.89801
2.37887  2.77519  2.98518  4.58477  3.61503
          0.01309  4.73812  5.46046  0.61958  0.77255  0.48576  0.95510
     92   2.62668  5.17422  3.11131  2.54900  4.47478  3.59229  3.77324
3.65620  1.49778  3.18613  3.72860  2.89763  3.98233  2.54719  2.35412
2.79039  2.79350  3.34628  5.62970  4.26027    130 k - - H
          2.68618  4.42225  2.77519  2.73123  3.46354  2.40513  3.72494
3.29354  2.67741  2.69355  4.24690  2.90347  2.73739  3.18146  2.89801
2.37887  2.77519  2.98518  4.58477  3.61503
          0.01309  4.73812  5.46046  0.61958  0.77255  0.48576  0.95510
     93   3.33022  4.67130  5.12149  4.54057  1.06860  4.54744  4.82922
2.53034  4.37981  1.68911  3.07264  4.64456  4.82400  4.47567  4.43531
3.87695  3.26436  2.04368  5.20110  4.01060    131 f - - H
          2.68618  4.42225  2.77519  2.73123  3.46354  2.40513  3.72494
3.29354  2.67741  2.69355  4.24690  2.90347  2.73739  3.18146  2.89801
2.37887  2.77519  2.98518  4.58477  3.61503
          0.01309  4.73812  5.46046  0.61958  0.77255  0.48576  0.95510
     94   2.14964  4.17555  3.21379  2.83747  3.93757  2.15900  3.92626
3.06609  2.81694  2.81236  3.84821  3.30719  3.51908  3.14813  3.23838
1.99434  2.87121  2.76483  5.30617  4.02758    132 s - - H
          2.68618  4.42225  2.77519  2.73123  3.46354  2.40513  3.72494
3.29354  2.67741  2.69355  4.24690  2.90347  2.73739  3.18146  2.89801
2.37887  2.77519  2.98518  4.58477  3.61503
          0.01309  4.73812  5.46046  0.61958  0.77255  0.48576  0.95510
```

```
     95   2.99171  4.88448  3.55735  3.02615  2.59273  3.85904  1.75507
3.45879  2.99825  3.07682  3.98621  2.57671  4.24235  3.09253  3.39744
2.85595  3.22215  3.20923  4.84978  1.99825    133 h - - H
          2.68618  4.42225  2.77519  2.73123  3.46354  2.40513  3.72494
3.29354  2.67741  2.69355  4.24690  2.90347  2.73739  3.18146  2.89801
2.37887  2.77519  2.98518  4.58477  3.61503
          0.01309  4.73812  5.46046  0.61958  0.77255  0.48576  0.95510
     96   2.82542  4.26979  4.39880  3.14547  1.65209  3.94902  4.25675
2.49673  3.66878  1.68637  3.05150  3.42445  4.31217  3.86868  3.81175
3.24261  3.05718  2.31929  4.87869  3.69038    134 f - - H
          2.68618  4.42225  2.77519  2.73123  3.46354  2.40513  3.72494
3.29354  2.67741  2.69355  4.24690  2.90347  2.73739  3.18146  2.89801
2.37887  2.77519  2.98518  4.58477  3.61503
          0.01309  4.73812  5.46046  0.61958  0.77255  0.48576  0.95510
     97   2.13525  4.41582  3.91705  3.34986  3.56725  3.81251  4.14237
2.71761  3.27695  2.21374  3.16068  3.68004  4.21223  3.55578  3.57113
2.75643  1.45179  2.64903  5.05144  3.83841    135 t - - H
          2.68618  4.42225  2.77519  2.73123  3.46354  2.40513  3.72494
3.29354  2.67741  2.69355  4.24690  2.90347  2.73739  3.18146  2.89801
2.37887  2.77519  2.98518  4.58477  3.61503
          0.01309  4.73812  5.46046  0.61958  0.77255  0.48576  0.95510
     98   0.73112  4.56039  4.15293  3.74554  4.46624  3.46680  4.66031
3.75339  3.68979  3.06802  4.40739  3.84933  4.18198  3.97955  3.97363
2.64673  2.14489  3.34015  5.87611  4.67977    136 a - - H
          2.68618  4.42225  2.77519  2.73123  3.46354  2.40513  3.72494
3.29354  2.67741  2.69355  4.24690  2.90347  2.73739  3.18146  2.89801
2.37887  2.77519  2.98518  4.58477  3.61503
          0.01309  4.73812  5.46046  0.61958  0.77255  0.48576  0.95510
     99   3.09952  5.15421  3.20698  3.15394  5.02447  3.61038  4.58893
4.59219  3.52121  4.24458  5.13856  0.52537  4.33924  3.83039  3.89354
3.18703  3.02434  4.06887  6.31266  4.95353    137 N - - H
          2.68618  4.42225  2.77519  2.73123  3.46354  2.40513  3.72494
3.29354  2.67741  2.69355  4.24690  2.90347  2.73739  3.18146  2.89801
2.37887  2.77519  2.98518  4.58477  3.61503
          0.01309  4.73812  5.46046  0.61958  0.77255  0.48576  0.95510
    100   2.64626  5.26351  3.06886  2.30489  4.59593  2.77240  3.76077
4.06320  2.32447  3.19057  4.31097  3.05655  3.97430  1.65349  2.40169
2.60830  3.03824  3.64809  5.69861  4.30968    138 q - - H
          2.68618  4.42225  2.77519  2.73123  3.46354  2.40513  3.72494
3.29354  2.67741  2.69355  4.24690  2.90347  2.73739  3.18146  2.89801
2.37887  2.77519  2.98518  4.58477  3.61503
          0.01309  4.73812  5.46046  0.61958  0.77255  0.48576  0.95510
    101   0.96048  4.62247  3.72718  3.20432  4.07640  3.59769  4.20096
3.43039  3.12417  3.13677  3.43306  3.55450  4.13674  3.47002  3.12187
2.77037  2.44996  3.12472  5.46948  4.22944    139 a - - H
          2.68618  4.42225  2.77519  2.73123  3.46354  2.40513  3.72494
3.29354  2.67741  2.69355  4.24690  2.90347  2.73739  3.18146  2.89801
2.37887  2.77519  2.98518  4.58477  3.61503
          0.01309  4.73812  5.46046  0.61958  0.77255  0.48576  0.95510
    102   3.72411  4.92741  5.73415  5.24643  3.89971  5.28051  5.88444
0.62199  5.14857  1.87605  3.64520  5.42875  5.44838  5.29892  5.25972
4.71032  3.97425  1.87130  6.07747  4.94673    140 I - - H
          2.68618  4.42225  2.77519  2.73123  3.46354  2.40513  3.72494
3.29354  2.67741  2.69355  4.24690  2.90347  2.73739  3.18146  2.89801
2.37887  2.77519  2.98518  4.58477  3.61503
          0.01309  4.73812  5.46046  0.61958  0.77255  0.48576  0.95510
```

```
    103   2.55673  4.37733  3.99811  3.42547  3.23313  3.85965  4.12988
2.83248  3.30228  1.09824  3.47694  3.47448  4.23377  3.24457  3.29581
3.13423  3.04596  2.63424  4.95736  3.47400    141 l - - H
          2.68618  4.42225  2.77519  2.73123  3.46354  2.40513  3.72494
3.29354  2.67741  2.69355  4.24690  2.90347  2.73739  3.18146  2.89801
2.37887  2.77519  2.98518  4.58477  3.61503
          0.01309  4.73812  5.46046  0.61958  0.77255  0.48576  0.95510
    104   2.96288  5.49437  2.14406  1.17813  4.82407  3.58677  3.87670
4.31689  2.67056  3.79099  4.55202  3.04742  4.05255  2.60975  2.72732
2.90296  2.92126  3.87925  5.92426  4.49189    142 e - - H
          2.68618  4.42225  2.77519  2.73123  3.46354  2.40513  3.72494
3.29354  2.67741  2.69355  4.24690  2.90347  2.73739  3.18146  2.89801
2.37887  2.77519  2.98518  4.58477  3.61503
          0.01309  4.73812  5.46046  0.61958  0.77255  0.48576  0.95510
    105   1.23752  4.57463  3.62781  2.86962  3.77744  3.72711  4.05375
2.82242  3.03550  2.50218  3.72657  3.48935  4.14500  3.35242  3.40346
2.42803  3.04569  2.75320  5.21072  3.96757    143 a - - H
          2.68618  4.42225  2.77519  2.73123  3.46354  2.40513  3.72494
3.29354  2.67741  2.69355  4.24690  2.90347  2.73739  3.18146  2.89801
2.37887  2.77519  2.98518  4.58477  3.61503
          0.01309  4.73812  5.46046  0.61958  0.77255  0.48576  0.95510
    106   2.43587  4.25036  4.73017  4.12620  1.99530  4.04267  4.37698
2.34978  3.93927  2.01907  2.43227  4.16892  4.39769  4.08737  3.98711
3.35280  2.59290  1.79000  4.89032  3.71668    144 v - - C
          2.68618  4.42225  2.77519  2.73123  3.46354  2.40513  3.72494
3.29354  2.67741  2.69355  4.24690  2.90347  2.73739  3.18146  2.89801
2.37887  2.77519  2.98518  4.58477  3.61503
          0.01309  4.73812  5.46046  0.61958  0.77255  0.48576  0.95510
    107   2.40809  5.28690  2.41248  1.65817  4.62778  3.32934  3.27295
4.11164  2.17643  3.59034  3.95216  2.88875  3.94861  2.76049  2.97591
2.53280  2.91673  3.67538  5.72161  4.31177    145 e - - T
          2.68618  4.42225  2.77519  2.73123  3.46354  2.40513  3.72494
3.29354  2.67741  2.69355  4.24690  2.90347  2.73739  3.18146  2.89801
2.37887  2.77519  2.98518  4.58477  3.61503
          0.01309  4.73812  5.46046  0.61958  0.77255  0.48576  0.95510
    108   2.79499  5.24104  2.92923  2.44134  4.55988  1.72559  3.76335
4.02759  2.24485  3.53442  4.29042  2.62486  3.96476  2.68568  2.90249
2.62505  2.59454  3.61787  5.68881  3.77612    146 g - - T
          2.68618  4.42225  2.77519  2.73123  3.46354  2.40513  3.72494
3.29354  2.67741  2.69355  4.24690  2.90347  2.73739  3.18146  2.89801
2.37887  2.77519  2.98518  4.58477  3.61503
          0.01309  4.73812  5.46046  0.61958  0.77255  0.48576  0.95510
    109   2.54319  3.72783  2.84228  1.76692  3.42229  3.56146  3.43429
3.99087  2.33951  3.50333  4.25992  2.49532  3.95438  2.70789  2.98731
2.38303  3.00715  3.58531  5.66241  3.72216    147 e - - -
          2.68628  4.42235  2.77490  2.73133  3.46364  2.40511  3.72479
3.29364  2.67736  2.69365  4.24700  2.90331  2.73734  3.18156  2.89811
2.37870  2.77529  2.98528  4.58487  3.61513
          0.18400  1.80905  5.46046  0.69382  0.69247  0.48576  0.95510
    110   2.59559  5.28420  2.39510  2.09550  4.62836  2.87480  3.45403
4.11370  2.12554  3.58907  3.80526  2.79845  3.55130  2.62278  2.55504
2.53090  2.79448  3.67436  5.71738  4.30694    151 e - - S
          2.68618  4.42225  2.77519  2.73123  3.46354  2.40513  3.72494
3.29354  2.67741  2.69355  4.24690  2.90347  2.73739  3.18146  2.89801
2.37887  2.77519  2.98518  4.58477  3.61503
          0.01309  4.73812  5.46046  0.61958  0.77255  0.48576  0.95510
```

```
     111   2.45745  4.90951  3.22791  2.58061  3.47637  3.62776  3.56973
3.53455  2.17431  2.95716  2.76740  3.17823  4.01621  3.00211  1.99057
2.64899  2.71894  3.10585  5.42511  4.10698    152 r - - E
           2.68618  4.42225  2.77519  2.73123  3.46354  2.40513  3.72494
3.29354  2.67741  2.69355  4.24690  2.90347  2.73739  3.18146  2.89801
2.37887  2.77519  2.98518  4.58477  3.61503
           0.01309  4.73812  5.46046  0.61958  0.77255  0.48576  0.95510
     112   3.17461  4.50520  5.02950  4.45703  3.63358  4.45438  4.84763
1.65864  3.48992  1.93395  3.04747  4.55900  4.77143  4.48117  4.39338
3.78548  3.41303  1.04522  5.34266  4.16761    153 v - - E
           2.68622  4.42229  2.77508  2.73127  3.46358  2.40517  3.72498
3.29329  2.67745  2.69344  4.24694  2.90351  2.73727  3.18150  2.89805
2.37891  2.77524  2.98522  4.58481  3.61507
           0.08144  2.60419  5.46046  0.66230  0.72497  0.48576  0.95510
     113   4.42075  5.92414  4.44973  4.35690  4.33376  4.51024  0.19260
5.29393  4.21073  4.62641  5.79595  4.70291  5.13069  4.75767  4.38269
4.51207  4.78381  5.00643  5.74348  4.27659    156 H - - E
           2.68618  4.42225  2.77519  2.73123  3.46354  2.40513  3.72494
3.29354  2.67741  2.69355  4.24690  2.90347  2.73739  3.18146  2.89801
2.37887  2.77519  2.98518  4.58477  3.61503
           0.01309  4.73812  5.46046  0.61958  0.77255  0.48576  0.95510
     114   3.64814  4.84038  5.74149  5.30040  4.23158  5.28420  6.11332
0.86844  5.25227  2.62772  3.95047  5.46375  5.50768  5.52395  5.43167
4.74081  3.91499  1.00226  6.40245  5.17109    157 i - - E
           2.68618  4.42225  2.77519  2.73123  3.46354  2.40513  3.72494
3.29354  2.67741  2.69355  4.24690  2.90347  2.73739  3.18146  2.89801
2.37887  2.77519  2.98518  4.58477  3.61503
           0.01309  4.73812  5.46046  0.61958  0.77255  0.48576  0.95510
     115   3.65161  4.84071  5.74152  5.27413  4.13174  5.28742  6.01146
0.85427  5.21807  2.22365  3.85939  5.44090  5.48124  5.44531  5.37240
4.72494  3.90848  1.14107  6.27931  5.08383    158 i - - E
           2.68618  4.42225  2.77519  2.73123  3.46354  2.40513  3.72494
3.29354  2.67741  2.69355  4.24690  2.90347  2.73739  3.18146  2.89801
2.37887  2.77519  2.98518  4.58477  3.61503
           0.01309  4.73812  5.46046  0.61958  0.77255  0.48576  0.95510
     116   4.19541  6.21099  0.21392  3.29677  5.77133  4.13121  5.02229
5.61793  4.30212  5.07223  6.13882  3.87295  4.83602  4.30941  4.87421
4.10846  4.56945  5.18207  6.71811  5.59711    159 D - - E
           2.68618  4.42225  2.77519  2.73123  3.46354  2.40513  3.72494
3.29354  2.67741  2.69355  4.24690  2.90347  2.73739  3.18146  2.89801
2.37887  2.77519  2.98518  4.58477  3.61503
           0.01309  4.73812  5.46046  0.61958  0.77255  0.48576  0.95510
     117   3.57634  4.87226  5.34380  4.80100  1.16793  4.75158  4.68080
2.25237  4.62455  1.25468  3.33936  4.79648  4.98497  4.62032  4.61553
4.09117  3.79350  2.85163  4.89188  3.04651    160 f - - C
           2.68618  4.42225  2.77519  2.73123  3.46354  2.40513  3.72494
3.29354  2.67741  2.69355  4.24690  2.90347  2.73739  3.18146  2.89801
2.37887  2.77519  2.98518  4.58477  3.61503
           0.01309  4.73812  5.46046  0.61958  0.77255  0.48576  0.95510
     118   3.19165  5.77254  1.16936  2.31643  5.08873  2.08622  4.05670
4.60469  2.98534  4.07864  4.87883  3.06672  4.17260  2.73490  3.55397
2.29597  3.46086  4.15909  6.22557  4.73984    161 d - - T
           2.68618  4.42225  2.77519  2.73123  3.46354  2.40513  3.72494
3.29354  2.67741  2.69355  4.24690  2.90347  2.73739  3.18146  2.89801
2.37887  2.77519  2.98518  4.58477  3.61503
           0.01309  4.73812  5.46046  0.61958  0.77255  0.48576  0.95510
```

```
    119   2.39119  4.40193  4.79562  4.21771  3.56280  4.24718  4.62543
1.09698  4.07011  2.10814  3.30190  4.33342  4.59512  4.26100  4.18110
3.08053  3.26926  1.89098  5.18121  3.99503    162 i - - S
          2.68618  4.42225  2.77519  2.73123  3.46354  2.40513  3.72494
3.29354  2.67741  2.69355  4.24690  2.90347  2.73739  3.18146  2.89801
2.37887  2.77519  2.98518  4.58477  3.61503
          0.01309  4.73812  5.46046  0.61958  0.77255  0.48576  0.95510
    120   2.77824  4.98715  3.17497  2.61800  4.23245  2.39018  3.81299
3.65055  2.25247  2.84205  3.20397  2.57759  3.99811  2.96110  3.07278
2.04330  2.62173  3.13264  5.49180  4.15499    163 s - - T
          2.68618  4.42225  2.77519  2.73123  3.46354  2.40513  3.72494
3.29354  2.67741  2.69355  4.24690  2.90347  2.73739  3.18146  2.89801
2.37887  2.77519  2.98518  4.58477  3.61503
          0.01309  4.73812  5.46046  0.61958  0.77255  0.48576  0.95510
    121   2.80878  5.04878  3.17212  2.05740  3.23893  3.62364  2.86006
3.72670  2.60743  3.06994  4.11684  3.14173  4.01456  1.79519  3.07002
2.82853  3.03955  3.39234  5.43778  2.79328    164 q - - T
          2.68618  4.42225  2.77519  2.73123  3.46354  2.40513  3.72494
3.29354  2.67741  2.69355  4.24690  2.90347  2.73739  3.18146  2.89801
2.37887  2.77519  2.98518  4.58477  3.61503
          0.01309  4.73812  5.46046  0.61958  0.77255  0.48576  0.95510
    122   2.70125  2.75311  4.52739  4.26941  4.96689  0.63444  5.09388
4.40571  4.22828  4.11230  4.92984  4.09135  2.44912  4.45842  4.41412
2.91368  3.24188  3.76449  6.31273  5.20168    165 G - - T
          2.68618  4.42225  2.77519  2.73123  3.46354  2.40513  3.72494
3.29354  2.67741  2.69355  4.24690  2.90347  2.73739  3.18146  2.89801
2.37887  2.77519  2.98518  4.58477  3.61503
          0.01309  4.73812  5.46046  0.61958  0.77255  0.48576  0.95510
    123   2.32545  4.25328  4.27936  3.69101  2.85927  2.42622  4.19811
2.57696  3.56626  1.80237  2.52070  3.58353  4.26145  3.78109  3.73643
2.93190  2.75397  2.29815  4.86632  3.67577    166 l - - T
          2.68618  4.42225  2.77519  2.73123  3.46354  2.40513  3.72494
3.29354  2.67741  2.69355  4.24690  2.90347  2.73739  3.18146  2.89801
2.37887  2.77519  2.98518  4.58477  3.61503
          0.01309  4.73812  5.46046  0.61958  0.77255  0.48576  0.95510
    124   3.50884  5.65393  3.30781  3.05777  4.55046  3.95201  3.39681
4.55310  2.74624  3.97863  4.90714  3.53445  4.46846  0.60818  3.01080
3.48263  3.75496  4.21516  5.78666  4.36525    167 Q - - T
          2.68618  4.42225  2.77519  2.73123  3.46354  2.40513  3.72494
3.29354  2.67741  2.69355  4.24690  2.90347  2.73739  3.18146  2.89801
2.37887  2.77519  2.98518  4.58477  3.61503
          0.01309  4.73812  5.46046  0.61958  0.77255  0.48576  0.95510
    125   4.14623  5.35248  5.26009  4.94352  2.43739  4.91712  3.97989
3.11732  4.67723  3.16872  4.41733  4.61694  5.21806  4.66887  4.67222
4.28685  4.36405  3.73930  0.67463  1.88133    168 W - - C
          2.68618  4.42225  2.77519  2.73123  3.46354  2.40513  3.72494
3.29354  2.67741  2.69355  4.24690  2.90347  2.73739  3.18146  2.89801
2.37887  2.77519  2.98518  4.58477  3.61503
          0.01309  4.73812  5.46046  0.61958  0.77255  0.48576  0.95510
    126   2.11980  4.45032  4.04646  3.47883  3.57256  3.91464  4.24489
2.63296  3.39913  2.13966  3.29453  3.79699  1.57660  3.67157  3.67898
3.19476  3.11476  2.24366  5.10590  3.90069    169 p - - H
          2.68618  4.42225  2.77519  2.73123  3.46354  2.40513  3.72494
3.29354  2.67741  2.69355  4.24690  2.90347  2.73739  3.18146  2.89801
2.37887  2.77519  2.98518  4.58477  3.61503
          0.01309  4.73812  5.46046  0.61958  0.77255  0.48576  0.95510
```

```
    127   1.65664  3.63541  3.53917  3.00993  4.38248  2.85555  4.13721
3.79961  2.99562  3.43258  4.24949  2.97260  2.97192  3.32522  3.41992
1.86509  2.08220  3.40180  5.68672  4.40043    170 a - - H
          2.68618  4.42225  2.77519  2.73123  3.46354  2.40513  3.72494
3.29354  2.67741  2.69355  4.24690  2.90347  2.73739  3.18146  2.89801
2.37887  2.77519  2.98518  4.58477  3.61503
          0.01309  4.73812  5.46046  0.61958  0.77255  0.48576  0.95510
    128   4.18760  5.38236  5.88493  5.39207  2.59027  5.44849  5.42620
2.72566  5.23807  0.40097  3.21571  5.54406  5.47340  5.08092  5.17760
4.89677  4.39133  3.03270  5.40233  4.13109    171 L - - H
          2.68618  4.42225  2.77519  2.73123  3.46354  2.40513  3.72494
3.29354  2.67741  2.69355  4.24690  2.90347  2.73739  3.18146  2.89801
2.37887  2.77519  2.98518  4.58477  3.61503
          0.01309  4.73812  5.46046  0.61958  0.77255  0.48576  0.95510
    129   3.82759  5.07094  5.76281  5.16739  2.51209  5.15462  5.43445
1.60882  5.01448  0.99597  1.78824  5.31249  5.24435  4.90858  4.95909
4.52167  4.03250  2.80522  5.51927  4.52909    172 l - - H
          2.68618  4.42225  2.77519  2.73123  3.46354  2.40513  3.72494
3.29354  2.67741  2.69355  4.24690  2.90347  2.73739  3.18146  2.89801
2.37887  2.77519  2.98518  4.58477  3.61503
          0.01309  4.73812  5.46046  0.61958  0.77255  0.48576  0.95510
    130   2.87665  5.35814  2.83009  2.24425  4.71058  3.60927  3.01007
4.18162  2.35708  3.65133  4.40453  3.07761  4.01364  1.44475  2.32307
2.83650  3.10389  3.75449  5.77022  4.38244    173 q - - H
          2.68618  4.42225  2.77519  2.73123  3.46354  2.40513  3.72494
3.29354  2.67741  2.69355  4.24690  2.90347  2.73739  3.18146  2.89801
2.37887  2.77519  2.98518  4.58477  3.61503
          0.01309  4.73812  5.46046  0.61958  0.77255  0.48576  0.95510
    131   1.41910  4.92838  3.22410  2.18100  4.19298  3.61582  3.86415
3.09043  2.67720  3.21986  4.04390  3.19095  4.02568  3.02810  2.96256
2.46216  2.78583  3.28075  5.47952  4.15879    174 a - - H
          2.68618  4.42225  2.77519  2.73123  3.46354  2.40513  3.72494
3.29354  2.67741  2.69355  4.24690  2.90347  2.73739  3.18146  2.89801
2.37887  2.77519  2.98518  4.58477  3.61503
          0.01309  4.73812  5.46046  0.61958  0.77255  0.48576  0.95510
    132   4.02014  5.23041  5.91842  5.32878  2.08640  5.37818  5.59209
2.29424  5.19034  0.58945  2.75773  5.52248  5.38215  5.01517  5.11411
4.76391  4.21452  2.92564  5.57233  4.58330    175 L - - H
          2.68618  4.42225  2.77519  2.73123  3.46354  2.40513  3.72494
3.29354  2.67741  2.69355  4.24690  2.90347  2.73739  3.18146  2.89801
2.37887  2.77519  2.98518  4.58477  3.61503
          0.01309  4.73812  5.46046  0.61958  0.77255  0.48576  0.95510
    133   1.12065  4.71371  3.61980  3.12990  4.42206  3.26449  4.24223
3.83115  3.12973  3.48132  4.30462  2.52778  4.09214  3.44915  3.53659
2.06341  2.79731  2.81102  5.74395  4.47427    176 a - - H
          2.68619  4.42226  2.77521  2.73124  3.46355  2.40500  3.72496
3.29355  2.67742  2.69356  4.24691  2.90348  2.73741  3.18147  2.89802
2.37888  2.77521  2.98519  4.58478  3.61504
          0.05362  3.03743  5.46046  0.38938  1.13157  0.48576  0.95510
    134   2.45192  4.03712  2.81193  2.22133  4.33060  3.58906  3.78775
3.76315  2.27208  2.56063  3.75857  3.09873  3.98030  2.91954  3.03942
1.98013  2.50371  3.41258  5.55063  4.19454    178 s - - C
          2.68618  4.42225  2.77519  2.73123  3.46354  2.40513  3.72494
3.29354  2.67741  2.69355  4.24690  2.90347  2.73739  3.18146  2.89801
2.37887  2.77519  2.98518  4.58477  3.61503
          0.03348  4.73812  3.72280  0.61958  0.77255  0.48576  0.95510
```

```
    135   3.71263  5.70740  4.37908  3.46949  5.25103  4.25094  4.01894
4.53515  1.88092  3.44630  4.80985  3.77602  4.57165  3.15176  0.61643
3.70127  3.81779  4.24760  5.87372  4.79613    179 R - - H
          2.68618  4.42225  2.77519  2.73123  3.46354  2.40513  3.72494
3.29354  2.67741  2.69355  4.24690  2.90347  2.73739  3.18146  2.89801
2.37887  2.77519  2.98518  4.58477  3.61503
          0.03332  4.71800  3.73650  0.61958  0.77255  0.52807  0.89097
    136   2.50572  5.06513  2.82940  2.55064  4.33761  3.57193  3.77373
3.41970  2.54886  2.85460  4.13122  2.66294  2.04366  2.90334  2.79376
2.28671  2.85462  3.41714  5.55064  4.19159    180 p - - T
          2.68632  4.42171  2.77533  2.73109  3.46368  2.40527  3.72508
3.29368  2.67743  2.69369  4.24704  2.90318  2.73732  3.18160  2.89777
2.37857  2.77521  2.98532  4.58491  3.61517
          0.24296  1.55460  5.42066  0.78705  0.60731  0.43809  1.03640
    137   2.64043  5.22074  2.81671  2.18231  4.53866  2.17588  3.75125
3.72909  2.38078  3.51439  4.26881  2.50400  3.95369  2.85718  2.86079
2.29343  3.00828  3.08809  5.67004  3.58222    185 g - - T
          2.68618  4.42225  2.77519  2.73123  3.46354  2.40513  3.72494
3.29354  2.67741  2.69355  4.24690  2.90347  2.73739  3.18146  2.89801
2.37887  2.77519  2.98518  4.58477  3.61503
          0.01309  4.73812  5.46046  0.61958  0.77255  0.48576  0.95510
    138   2.71715  5.28723  2.68291  2.54353  4.66871  1.67548  3.87955
4.13884  2.46457  3.65641  4.42739  2.86257  2.22974  3.00083  3.17314
2.44233  3.13629  3.72071  5.82069  4.42042    186 g - - S
          2.68624  4.42231  2.77526  2.73130  3.46360  2.40519  3.72501
3.29360  2.67731  2.69361  4.24696  2.90333  2.73746  3.18101  2.89807
2.37870  2.77526  2.98525  4.58483  3.61509
          0.04808  3.15369  5.46046  1.26869  0.33017  0.48576  0.95510
    139   2.18196  4.92976  3.25408  2.71443  4.27491  3.25272  3.89613
3.69082  2.57876  3.10543  4.11454  3.21003  1.63909  3.05427  2.99361
2.38349  2.77169  3.35006  5.54434  4.22131    193 p - - -
          2.68618  4.42225  2.77519  2.73123  3.46354  2.40513  3.72494
3.29354  2.67741  2.69355  4.24690  2.90347  2.73739  3.18146  2.89801
2.37887  2.77519  2.98518  4.58477  3.61503
          0.01309  4.73812  5.46046  0.61958  0.77255  0.48576  0.95510
    140   2.47052  4.95088  3.31778  2.93764  4.50416  3.16075  3.47436
3.93163  2.91408  3.54404  4.37676  3.38194  1.00484  3.31980  3.04142
2.96295  3.20690  3.55112  5.76883  4.47285    194 p - - -
          2.68620  4.42227  2.77522  2.73125  3.46356  2.40515  3.72496
3.29356  2.67743  2.69347  4.24692  2.90349  2.73742  3.18129  2.89803
2.37889  2.77511  2.98520  4.58479  3.61505
          0.08425  2.56984  5.46046  0.30703  1.33041  0.48576  0.95510
    141   2.77743  4.92254  3.10330  2.65539  3.43905  3.62177  2.31085
3.26845  2.54696  2.83945  3.70591  2.94867  4.01067  2.85139  2.62138
2.09405  2.87493  2.87151  5.43601  4.11369    196 s - - E
          2.68618  4.42225  2.77519  2.73123  3.46354  2.40513  3.72494
3.29354  2.67741  2.69355  4.24690  2.90347  2.73739  3.18146  2.89801
2.37887  2.77519  2.98518  4.58477  3.61503
          0.01309  4.73812  5.46046  0.61958  0.77255  0.48576  0.95510
    142   3.64686  4.90593  5.59627  5.02119  2.54883  4.99250  5.33444
2.03247  4.87499  0.87244  3.27458  5.14054  5.16012  4.87023  4.87197
4.35500  3.86807  1.62989  5.53217  4.47009    197 l - - E
          2.68618  4.42225  2.77519  2.73123  3.46354  2.40513  3.72494
3.29354  2.67741  2.69355  4.24690  2.90347  2.73739  3.18146  2.89801
2.37887  2.77519  2.98518  4.58477  3.61503
          0.01309  4.73812  5.46046  0.61958  0.77255  0.48576  0.95510
```

143 3.83990 5.82262 4.50829 3.55759 5.47772 4.32127 4.05764
4.72067 1.59370 4.02902 4.93898 3.84493 4.63725 3.18606 0.63858
3.81207 3.92426 4.42504 5.94870 4.91394 198 R - - E
2.68618 4.42225 2.77519 2.73123 3.46354 2.40513 3.72494
3.29354 2.67741 2.69355 4.24690 2.90347 2.73739 3.18146 2.89801
2.37887 2.77519 2.98518 4.58477 3.61503
0.01309 4.73812 5.46046 0.61958 0.77255 0.48576 0.95510
144 3.70260 4.90619 5.73802 5.23374 3.85728 5.25091 5.81768
0.85299 5.14490 1.48277 3.60082 5.40115 5.41129 5.25775 5.23210
4.66664 3.94671 1.68390 6.00944 4.90470 199 i - - E
2.68618 4.42225 2.77519 2.73123 3.46354 2.40513 3.72494
3.29354 2.67741 2.69355 4.24690 2.90347 2.73739 3.18146 2.89801
2.37887 2.77519 2.98518 4.58477 3.61503
0.01309 4.73812 5.46046 0.61958 0.77255 0.48576 0.95510
145 2.74080 4.64798 4.22281 4.07481 4.94363 3.44150 5.03253
4.28675 4.07414 4.08621 4.96200 4.01663 4.25428 4.38078 4.26935
2.66377 0.45645 3.71089 6.32710 5.14264 200 T - - E
2.68618 4.42225 2.77519 2.73123 3.46354 2.40513 3.72494
3.29354 2.67741 2.69355 4.24690 2.90347 2.73739 3.18146 2.89801
2.37887 2.77519 2.98518 4.58477 3.61503
0.01309 4.73812 5.46046 0.61958 0.77255 0.48576 0.95510
146 1.70738 4.54272 4.27486 3.89950 4.59899 0.96501 4.78916
3.87809 3.86732 3.67874 4.53437 3.91463 4.18041 4.12073 4.13091
2.87228 2.67823 2.36841 6.00441 4.82765 201 g - - E
2.68618 4.42225 2.77519 2.73123 3.46354 2.40513 3.72494
3.29354 2.67741 2.69355 4.24690 2.90347 2.73739 3.18146 2.89801
2.37887 2.77519 2.98518 4.58477 3.61503
0.01309 4.73812 5.46046 0.61958 0.77255 0.48576 0.95510
147 3.08446 3.34315 5.05599 4.47338 2.65616 4.35214 4.74219
1.51626 4.29609 2.05231 3.50297 4.50390 4.68349 4.44736 4.33799
3.68285 3.07835 1.27946 5.21266 4.03316 202 v - - E
2.68618 4.42225 2.77519 2.73123 3.46354 2.40513 3.72494
3.29354 2.67741 2.69355 4.24690 2.90347 2.73739 3.18146 2.89801
2.37887 2.77519 2.98518 4.58477 3.61503
0.01309 4.73812 5.46046 0.61958 0.77255 0.48576 0.95510
148 2.33774 5.18237 2.76026 2.30262 4.48591 2.06664 2.91408
3.94161 2.53508 3.47283 4.24149 3.05993 3.63237 2.88912 3.02030
2.36713 3.02185 2.80405 5.64850 4.26547 203 g - - E
2.68618 4.42225 2.77519 2.73123 3.46354 2.40513 3.72494
3.29354 2.67741 2.69355 4.24690 2.90347 2.73739 3.18146 2.89801
2.37887 2.77519 2.98518 4.58477 3.61503
0.01309 4.73812 5.46046 0.61958 0.77255 0.48576 0.95510
149 2.48049 5.23667 2.53279 2.29093 4.04491 3.55686 2.92294
4.03351 2.48922 3.19525 4.28269 2.63059 2.96132 2.84938 2.58273
2.27611 2.91423 3.23846 5.68163 4.00022 204 s - - C
2.68618 4.42225 2.77519 2.73123 3.46354 2.40513 3.72494
3.29354 2.67741 2.69355 4.24690 2.90347 2.73739 3.18146 2.89801
2.37887 2.77519 2.98518 4.58477 3.61503
0.33588 4.73812 1.28542 0.61958 0.77255 0.48576 0.95510
150 2.36143 4.87895 3.05888 2.27871 4.13726 3.49861 3.72708
3.55228 2.52681 2.88077 3.70493 2.77458 2.06694 2.87909 2.98593
2.54664 2.92113 3.22591 5.40038 4.06630 205 p - - E
2.68618 4.42225 2.77519 2.73123 3.46354 2.40513 3.72494
3.29354 2.67741 2.69355 4.24690 2.90347 2.73739 3.18146 2.89801
2.37887 2.77519 2.98518 4.58477 3.61503
0.01803 4.42027 5.14262 0.61958 0.77255 0.95005 0.48893

```
    151   2.49039  5.12878  2.73786  2.12693  4.44828  3.45765  3.65361
3.91662  2.40185  3.42352  4.17808  2.70206  3.85469  2.31777  2.79687
2.23442  2.49726  3.13139  5.57804  4.18247    206 e - - .
          2.68618  4.42225  2.77519  2.73123  3.46354  2.40513  3.72494
3.29354  2.67741  2.69355  4.24690  2.90347  2.73739  3.18146  2.89801
2.37887  2.77519  2.98518  4.58477  3.61503
          0.11493  4.42027  2.33783  0.61958  0.77255  0.95005  0.48893
    152   2.39741  4.89682  3.01592  2.46165  4.15063  3.46685  3.28248
3.57576  2.29726  2.97703  3.96516  2.65513  2.86136  2.80899  2.92155
2.35835  2.62291  3.23800  5.39029  3.44917    207 k - - T
          2.68618  4.42225  2.77519  2.73123  3.46354  2.40513  3.72494
3.29354  2.67741  2.69355  4.24690  2.90347  2.73739  3.18146  2.89801
2.37887  2.77519  2.98518  4.58477  3.61503
          0.01985  4.32520  5.04755  0.61958  0.77255  1.03626  0.43816
    153   2.56633  4.70218  2.53540  2.58621  3.03260  2.41813  3.39109
3.30894  2.58529  2.95456  3.79587  3.08426  3.89945  2.92308  3.02882
2.63743  2.75086  2.60669  5.24184  3.93811    208 g - - T
          2.68619  4.42165  2.77497  2.73143  3.46256  2.40531  3.72524
3.29357  2.67747  2.69369  4.24720  2.90356  2.73726  3.18137  2.89790
2.37885  2.77532  2.98505  4.58507  3.61517
          0.67145  0.72855  5.04755  1.06136  0.42463  0.27424  1.42775
    154   2.77036  4.21146  2.38394  2.35025  4.53616  3.05687  3.74433
3.55866  2.49000  3.51120  4.26463  2.84383  3.08779  2.42768  2.80556
2.03375  3.00156  3.25273  5.66558  3.76820    215 s - - H
          2.68618  4.42225  2.77519  2.73123  3.46354  2.40513  3.72494
3.29354  2.67741  2.69355  4.24690  2.90347  2.73739  3.18146  2.89801
2.37887  2.77519  2.98518  4.58477  3.61503
          0.01328  4.72366  5.44601  0.61958  0.77255  0.46705  0.98578
    155   2.43121  5.18377  3.06709  2.16580  4.12367  3.01001  3.33114
3.67185  2.11234  3.15993  3.72746  2.85548  3.95855  2.66720  2.65691
2.50198  2.78417  2.92811  5.64144  4.25634    216 k - - H
          2.68618  4.42225  2.77519  2.73123  3.46354  2.40513  3.72494
3.29354  2.67741  2.69355  4.24690  2.90347  2.73739  3.18146  2.89801
2.37887  2.77519  2.98518  4.58477  3.61503
          0.01309  4.73812  5.46046  0.61958  0.77255  0.48576  0.95510
    156   2.64079  5.18141  2.41170  1.93755  3.67800  3.12481  3.75853
3.51916  2.51180  3.02806  4.23319  3.05211  3.28094  2.62265  2.77446
2.68145  2.66137  3.05230  5.63968  4.25514    217 e - - H
          2.68618  4.42225  2.77519  2.73123  3.46354  2.40513  3.72494
3.29354  2.67741  2.69355  4.24690  2.90347  2.73739  3.18146  2.89801
2.37887  2.77519  2.98518  4.58477  3.61503
          0.01309  4.73812  5.46046  0.61958  0.77255  0.48576  0.95510
    157   2.36992  5.10515  3.00767  1.93987  4.38227  2.73634  3.77714
3.82288  2.54608  3.19880  3.57259  3.08190  3.50329  2.90159  2.52614
2.52625  2.87686  2.69856  5.58096  3.92389    218 e - - H
          2.68618  4.42225  2.77519  2.73123  3.46354  2.40513  3.72494
3.29354  2.67741  2.69355  4.24690  2.90347  2.73739  3.18146  2.89801
2.37887  2.77519  2.98518  4.58477  3.61503
          0.01309  4.73812  5.46046  0.61958  0.77255  0.48576  0.95510
    158   2.79987  4.22465  4.47516  3.87849  3.31817  3.40912  4.25507
2.48739  3.72167  1.19949  2.98065  3.99827  4.30180  3.90660  3.83146
2.98696  2.82014  2.12126  4.84470  3.26737    219 l - - H
          2.68618  4.42225  2.77519  2.73123  3.46354  2.40513  3.72494
3.29354  2.67741  2.69355  4.24690  2.90347  2.73739  3.18146  2.89801
2.37887  2.77519  2.98518  4.58477  3.61503
          0.01309  4.73812  5.46046  0.61958  0.77255  0.48576  0.95510
```

159 2.67613 5.27000 3.03411 1.87947 4.60818 3.31236 3.55245
3.72654 2.22061 3.57164 4.31276 2.60787 3.94750 2.27553 2.27216
2.67343 3.00939 3.47671 5.70616 3.70245 220 e - - H
2.68618 4.42225 2.77519 2.73123 3.46354 2.40513 3.72494
3.29354 2.67741 2.69355 4.24690 2.90347 2.73739 3.18146 2.89801
2.37887 2.77519 2.98518 4.58477 3.61503
0.01309 4.73812 5.46046 0.61958 0.77255 0.48576 0.95510
160 2.39250 5.16569 2.94712 1.80753 3.39774 3.56975 3.76259
3.48090 2.51882 2.89788 4.21913 2.84457 3.96207 2.31134 2.62506
2.66171 2.90846 3.53071 5.62767 4.24722 221 e - - H
2.68618 4.42225 2.77519 2.73123 3.46354 2.40513 3.72494
3.29354 2.67741 2.69355 4.24690 2.90347 2.73739 3.18146 2.89801
2.37887 2.77519 2.98518 4.58477 3.61503
0.01309 4.73812 5.46046 0.61958 0.77255 0.48576 0.95510
161 2.88594 4.28706 4.61926 4.02454 3.37393 4.05008 4.38066
2.15658 3.86407 2.17734 2.60541 4.12936 4.40578 4.03977 3.96021
3.35511 1.64943 1.72748 4.94061 3.27801 222 t - - H
2.68618 4.42225 2.77519 2.73123 3.46354 2.40513 3.72494
3.29354 2.67741 2.69355 4.24690 2.90347 2.73739 3.18146 2.89801
2.37887 2.77519 2.98518 4.58477 3.61503
0.01309 4.73812 5.46046 0.61958 0.77255 0.48576 0.95510
162 2.38975 5.28656 3.04509 2.39234 4.61204 1.32971 3.87292
4.05324 2.36602 3.58291 4.37564 3.14122 4.07351 2.81740 2.80142
2.92020 3.17319 3.67563 5.75226 4.39254 223 g - - H
2.68618 4.42225 2.77519 2.73123 3.46354 2.40513 3.72494
3.29354 2.67741 2.69355 4.24690 2.90347 2.73739 3.18146 2.89801
2.37887 2.77519 2.98518 4.58477 3.61503
0.01309 4.73812 5.46046 0.61958 0.77255 0.48576 0.95510
163 2.64440 4.59791 2.30469 2.15204 4.60110 3.55238 2.92193
4.08084 2.31813 3.20486 4.30784 2.61826 3.94568 2.65520 2.54300
2.45743 3.00658 3.65074 3.68639 4.29728 224 e - - H
2.68618 4.42225 2.77519 2.73123 3.46354 2.40513 3.72494
3.29354 2.67741 2.69355 4.24690 2.90347 2.73739 3.18146 2.89801
2.37887 2.77519 2.98518 4.58477 3.61503
0.01309 4.73812 5.46046 0.61958 0.77255 0.48576 0.95510
164 3.23474 5.52196 3.60766 2.94190 4.96543 3.91691 3.89404
4.34081 1.83129 3.18339 4.57999 2.36308 4.27396 2.71621 1.12423
3.20587 3.41320 3.97516 5.81050 4.57920 225 r - - H
2.68618 4.42225 2.77519 2.73123 3.46354 2.40513 3.72494
3.29354 2.67741 2.69355 4.24690 2.90347 2.73739 3.18146 2.89801
2.37887 2.77519 2.98518 4.58477 3.61503
0.01309 4.73812 5.46046 0.61958 0.77255 0.48576 0.95510
165 4.17362 5.38106 5.91860 5.37970 3.40366 5.50148 5.76450
2.64750 5.15903 0.37837 2.70519 5.64423 5.49191 5.08184 5.13159
4.94807 4.37822 2.95281 5.69755 4.72609 226 L - - H
2.68618 4.42225 2.77519 2.73123 3.46354 2.40513 3.72494
3.29354 2.67741 2.69355 4.24690 2.90347 2.73739 3.18146 2.89801
2.37887 2.77519 2.98518 4.58477 3.61503
0.01309 4.73812 5.46046 0.61958 0.77255 0.48576 0.95510
166 2.06764 5.01557 3.15527 2.31620 4.26456 3.29949 3.44398
2.88109 2.35322 3.01409 4.08310 3.12167 3.99061 2.94344 2.67723
2.48977 2.36750 3.09167 5.51050 4.16665 227 a - - H
2.68618 4.42225 2.77519 2.73123 3.46354 2.40513 3.72494
3.29354 2.67741 2.69355 4.24690 2.90347 2.73739 3.18146 2.89801
2.37887 2.77519 2.98518 4.58477 3.61503
0.01309 4.73812 5.46046 0.61958 0.77255 0.48576 0.95510

```
    167   2.55036  5.29180  2.54084  2.16327  4.63667  3.01121  3.30901
4.12256  2.13663  3.59669  4.33288  2.82821  3.94611  2.14383  2.43179
2.54636  3.01244  3.68241  5.72406  4.31300    228 k - - H
          2.68618  4.42225  2.77519  2.73123  3.46354  2.40513  3.72494
3.29354  2.67741  2.69355  4.24690  2.90347  2.73739  3.18146  2.89801
2.37887  2.77519  2.98518  4.58477  3.61503
          0.01309  4.73812  5.46046  0.61958  0.77255  0.48576  0.95510
    168   2.98655  4.45913  4.35745  2.73677  1.31967  4.06930  4.24404
2.79559  3.67736  1.79678  3.45168  4.00594  4.42345  3.89164  3.86554
3.36050  3.21681  2.49637  4.81969  3.19414    229 f - - H
          2.68618  4.42225  2.77519  2.73123  3.46354  2.40513  3.72494
3.29354  2.67741  2.69355  4.24690  2.90347  2.73739  3.18146  2.89801
2.37887  2.77519  2.98518  4.58477  3.61503
          0.01309  4.73812  5.46046  0.61958  0.77255  0.48576  0.95510
    169   0.45989  3.25499  4.74533  4.50291  4.93292  2.76806  5.18151
4.29229  4.40557  4.06545  4.88841  4.13299  4.19989  4.58996  4.52537
2.85232  3.18130  3.66212  6.32605  5.22326    230 A - - H
          2.68618  4.42225  2.77519  2.73123  3.46354  2.40513  3.72494
3.29354  2.67741  2.69355  4.24690  2.90347  2.73739  3.18146  2.89801
2.37887  2.77519  2.98518  4.58477  3.61503
          0.01309  4.73812  5.46046  0.61958  0.77255  0.48576  0.95510
    170   2.52398  5.28960  2.23190  1.96448  4.63293  3.15357  3.14920
4.11802  2.22330  3.59394  4.33112  3.01881  3.94692  2.75596  2.40450
2.48333  2.82395  3.67938  5.72283  4.31227    231 e - - H
          2.68618  4.42225  2.77519  2.73123  3.46354  2.40513  3.72494
3.29354  2.67741  2.69355  4.24690  2.90347  2.73739  3.18146  2.89801
2.37887  2.77519  2.98518  4.58477  3.61503
          0.01309  4.73812  5.46046  0.61958  0.77255  0.48576  0.95510
    171   2.40395  5.22674  2.92789  2.09645  4.54766  3.55910  3.74870
4.01675  2.05121  3.01585  3.97713  3.03656  3.95200  2.68311  2.76757
2.12396  2.51117  3.36628  5.67407  4.27845    232 k - - H
          2.68618  4.42225  2.77519  2.73123  3.46354  2.40513  3.72494
3.29354  2.67741  2.69355  4.24690  2.90347  2.73739  3.18146  2.89801
2.37887  2.77519  2.98518  4.58477  3.61503
          0.01309  4.73812  5.46046  0.61958  0.77255  0.48576  0.95510
    172   2.79733  3.86212  4.63799  4.03119  2.62070  3.95441  4.28169
2.06389  3.84288  1.43481  2.59097  3.71171  4.31784  3.99652  3.41681
3.26063  2.72731  2.34285  4.81144  3.63350    233 l - - H
          2.68618  4.42225  2.77519  2.73123  3.46354  2.40513  3.72494
3.29354  2.67741  2.69355  4.24690  2.90347  2.73739  3.18146  2.89801
2.37887  2.77519  2.98518  4.58477  3.61503
          0.01309  4.73812  5.46046  0.61958  0.77255  0.48576  0.95510
    173   2.88884  5.39375  2.25344  2.49002  4.72761  1.79730  3.58507
4.21195  2.30482  3.69240  4.44659  2.13167  4.01327  2.93118  2.68614
2.84170  3.12597  3.78035  5.82723  4.41100    234 g - - T
          2.68618  4.42225  2.77519  2.73123  3.46354  2.40513  3.72494
3.29354  2.67741  2.69355  4.24690  2.90347  2.73739  3.18146  2.89801
2.37887  2.77519  2.98518  4.58477  3.61503
          0.01309  4.73812  5.46046  0.61958  0.77255  0.48576  0.95510
    174   3.29998  4.61202  5.17005  4.60417  3.63666  4.60553  5.00589
1.38512  4.44936  1.57661  3.50017  4.71602  4.89472  3.71663  4.53324
3.94667  3.53742  1.28118  5.44538  4.29200    235 v - - -
          2.68618  4.42225  2.77519  2.73123  3.46354  2.40513  3.72494
3.29354  2.67741  2.69355  4.24690  2.90347  2.73739  3.18146  2.89801
2.37887  2.77519  2.98518  4.58477  3.61503
          0.01309  4.73812  5.46046  0.61958  0.77255  0.48576  0.95510
```

```
    175   2.65177  5.27767  2.79006  2.36379  4.61399  3.27476  3.74947
4.09428  2.49094  3.57951  4.32228  2.63271  2.10199  2.48377  2.61113
2.25951  2.82655  3.66394  5.71610  4.30930    236 p - - -
          2.68618  4.42225  2.77519  2.73123  3.46354  2.40513  3.72494
3.29354  2.67741  2.69355  4.24690  2.90347  2.73739  3.18146  2.89801
2.37887  2.77519  2.98518  4.58477  3.61503
          0.01309  4.73812  5.46046  0.61958  0.77255  0.48576  0.95510
    176   3.73482  5.01644  5.47412  4.92621  0.80959  4.96541  5.00561
2.65419  4.76993  1.65196  2.87605  5.02011  5.14279  4.75921  4.78407
4.32084  3.95170  2.48026  5.18343  3.85178    237 f - - E
          2.68618  4.42225  2.77519  2.73123  3.46354  2.40513  3.72494
3.29354  2.67741  2.69355  4.24690  2.90347  2.73739  3.18146  2.89801
2.37887  2.77519  2.98518  4.58477  3.61503
          0.01309  4.73812  5.46046  0.61958  0.77255  0.48576  0.95510
    177   2.85979  5.37435  2.65785  1.59150  4.71416  3.57099  3.79771
4.20076  2.25424  3.67476  4.42168  3.03576  3.99429  1.88110  2.92566
2.39591  3.09477  3.76322  5.80364  4.38758    238 e - - E
          2.68618  4.42225  2.77519  2.73123  3.46354  2.40513  3.72494
3.29354  2.67741  2.69355  4.24690  2.90347  2.73739  3.18146  2.89801
2.37887  2.77519  2.98518  4.58477  3.61503
          0.01309  4.73812  5.46046  0.61958  0.77255  0.48576  0.95510
    178   3.84396  5.08247  5.30754  4.92686  0.85014  4.80592  4.11230
1.95780  4.73298  2.74533  3.99273  4.64309  5.09563  4.66457  4.69008
4.15832  4.06411  3.25940  4.25739  1.85861    239 f - - E
          2.68618  4.42225  2.77519  2.73123  3.46354  2.40513  3.72494
3.29354  2.67741  2.69355  4.24690  2.90347  2.73739  3.18146  2.89801
2.37887  2.77519  2.98518  4.58477  3.61503
          0.01309  4.73812  5.46046  0.61958  0.77255  0.48576  0.95510
    179   2.77636  4.22212  2.86020  2.23284  4.60343  3.55279  2.58001
3.72397  2.28717  3.56818  4.30954  2.08854  3.94618  2.58799  2.59318
2.51014  2.82061  3.65283  5.70406  4.29852    240 n - - E
          2.68618  4.42225  2.77519  2.73123  3.46354  2.40513  3.72494
3.29354  2.67741  2.69355  4.24690  2.90347  2.73739  3.18146  2.89801
2.37887  2.77519  2.98518  4.58477  3.61503
          0.03894  4.73812  3.52551  0.61958  0.77255  0.48576  0.95510
    180   2.29964  4.30795  4.05510  3.47618  2.67961  2.79479  4.13621
2.41032  3.38373  2.32360  3.42347  3.75213  2.38265  3.63209  3.62278
2.89462  2.68465  2.17253  4.91687  3.71571    241 v - - E
          2.68618  4.42225  2.77519  2.73123  3.46354  2.40513  3.72494
3.29354  2.67741  2.69355  4.24690  2.90347  2.73739  3.18146  2.89801
2.37887  2.77519  2.98518  4.58477  3.61503
          0.37933  4.71261  1.18190  0.61958  0.77255  0.53898  0.87550
    181   3.34443  4.62063  5.19619  4.63930  3.11174  4.70326  5.09265
1.73529  4.51758  1.13766  3.18616  4.80349  4.91193  4.60758  4.59367
4.06201  3.57576  1.46217  5.39548  4.29408    242 l - - E
          2.68619  4.42235  2.77530  2.73124  3.46364  2.40517  3.72505
3.29294  2.67751  2.69365  4.24700  2.90357  2.73736  3.18157  2.89811
2.37887  2.77515  2.98473  4.58487  3.61491
          0.38335  1.73641  1.94998  0.97500  0.47352  0.25762  1.48233
    182   2.48658  4.29684  3.97001  2.94184  3.40974  3.80387  3.15577
2.27033  3.30637  2.20250  3.20715  3.40400  4.17694  3.56290  3.56170
3.07442  2.63544  1.74398  4.89995  3.29198    246 v - - E
          2.68618  4.42225  2.77519  2.73123  3.46354  2.40513  3.72494
3.29354  2.67741  2.69355  4.24690  2.90347  2.73739  3.18146  2.89801
2.37887  2.77519  2.98518  4.58477  3.61503
          0.01444  4.64105  5.36339  0.61958  0.77255  0.66871  0.71819
```

```
    183   2.04341  3.85510  3.53468  2.66631  3.66797  3.42799  3.39345
2.83768  2.93209  2.54671  3.28356  3.18844  3.73376  3.24360  3.30808
2.40856  2.86752  2.15081  5.09588  3.85060    247 a - - E
          2.68618  4.42225  2.77519  2.73123  3.46354  2.40513  3.72494
3.29354  2.67741  2.69355  4.24690  2.90347  2.73739  3.18146  2.89801
2.37887  2.77519  2.98518  4.58477  3.61503
          0.01444  4.64105  5.36339  0.61958  0.77255  0.66871  0.71819
    184   2.56377  5.22020  2.35108  2.28810  4.55109  3.52165  3.71104
4.02711  2.08270  3.05608  4.26483  2.52238  3.91547  2.81228  2.69635
2.26425  2.83702  3.10306  5.66137  4.25916    248 k - - C
          2.68621  4.42228  2.77523  2.73126  3.46357  2.40504  3.72498
3.29357  2.67744  2.69325  4.24693  2.90350  2.73743  3.18149  2.89804
2.37890  2.77523  2.98521  4.58480  3.61506
          0.16270  2.08191  3.67060  0.24179  1.53814  0.47313  0.97563
    185   2.53275  4.58849  2.65023  2.46077  4.57773  3.23177  3.47257
4.05620  2.16852  3.54390  4.28674  2.60310  3.43372  2.82369  2.13201
2.46935  2.44769  3.62814  5.68216  4.27785    250 r - - -
          2.68618  4.42225  2.77519  2.73123  3.46354  2.40513  3.72494
3.29354  2.67741  2.69355  4.24690  2.90347  2.73739  3.18146  2.89801
2.37887  2.77519  2.98518  4.58477  3.61503
          0.01379  4.68669  5.40904  0.61958  0.77255  0.58902  0.80939
    186   2.67457  3.84746  3.94791  3.91582  2.61257  3.23001  4.23018
2.49445  3.74461  1.45165  2.91118  3.99896  4.27407  3.91460  3.82642
3.06494  2.67996  2.29795  3.45608  3.61042    251 l - - G
          2.68618  4.42225  2.77519  2.73123  3.46354  2.40513  3.72494
3.29354  2.67741  2.69355  4.24690  2.90347  2.73739  3.18146  2.89801
2.37887  2.77519  2.98518  4.58477  3.61503
          0.01379  4.68669  5.40904  0.61958  0.77255  0.58902  0.80939
    187   2.51561  4.54886  2.28612  1.90647  4.55826  2.97206  3.25879
4.03237  2.47392  3.08957  4.27661  2.84171  3.93345  2.83303  2.96419
2.21572  2.89945  3.61197  5.67423  4.27345    252 e - - G
          2.68618  4.42225  2.77519  2.73123  3.46354  2.40513  3.72494
3.29354  2.67741  2.69355  4.24690  2.90347  2.73739  3.18146  2.89801
2.37887  2.77519  2.98518  4.58477  3.61503
          0.01379  4.68669  5.40904  0.61958  0.77255  0.52463  0.89593
    188   2.52623  3.21706  2.23926  2.27215  4.04749  3.62786  3.84151
2.93318  2.68107  2.34917  3.51494  2.85992  4.01584  3.02312  3.12739
2.66875  2.87774  3.15446  5.36850  4.06176    253 d - - G
          2.68618  4.42225  2.77519  2.73123  3.46354  2.40513  3.72494
3.29354  2.67741  2.69355  4.24690  2.90347  2.73739  3.18146  2.89801
2.37887  2.77519  2.98518  4.58477  3.61503
          0.01352  4.70635  5.42870  0.61958  0.77255  0.44679  1.02075
    189   2.88082  4.29936  3.68537  3.56998  2.78118  4.02920  4.35021
2.21442  3.79641  1.25471  3.35759  4.08041  4.38546  3.98202  3.91619
3.32968  3.11319  1.87071  4.93559  3.75503    254 l - - S
          2.68622  4.42229  2.77523  2.73127  3.46357  2.40516  3.72498
3.29358  2.67744  2.69340  4.24693  2.90328  2.73724  3.18150  2.89804
2.37890  2.77523  2.98522  4.58481  3.61507
          0.10860  3.03743  2.90135  0.86143  0.54914  0.48576  0.95510
    190   2.77123  5.28065  2.38411  2.16864  4.62391  3.53436  3.72864
4.10944  2.24429  3.58476  4.32264  2.29056  3.93314  2.48939  2.56171
2.30443  2.57273  3.67048  5.71333  4.30191    258 e - - -
          2.68618  4.42225  2.77519  2.73123  3.46354  2.40513  3.72494
3.29354  2.67741  2.69355  4.24690  2.90347  2.73739  3.18146  2.89801
2.37887  2.77519  2.98518  4.58477  3.61503
          0.01380  4.68609  5.40844  0.61958  0.77255  0.49384  0.94232
```

191 2.68464 4.23097 4.32100 3.73010 2.84828 3.53247 4.19982
2.36666 3.59667 1.75051 3.34637 3.90432 2.48884 3.80316 3.26860
3.18177 2.71864 2.07013 4.84472 3.15537 259 l - - H
2.68618 4.42225 2.77519 2.73123 3.46354 2.40513 3.72494
3.29354 2.67741 2.69355 4.24690 2.90347 2.73739 3.18146 2.89801
2.37887 2.77519 2.98518 4.58477 3.61503
0.01339 4.71583 5.43818 0.61958 0.77255 0.45762 1.00182
192 2.77661 5.25916 2.73509 1.85164 3.79955 3.31230 3.74257
4.07036 2.23771 3.55936 4.30299 2.79917 3.94746 2.53175 2.70389
2.25388 3.00779 3.64354 3.17965 4.29496 260 e - - H
2.68618 4.42225 2.77519 2.73123 3.46354 2.40513 3.72494
3.29354 2.67741 2.69355 4.24690 2.90347 2.73739 3.18146 2.89801
2.37887 2.77519 2.98518 4.58477 3.61503
0.01309 4.73812 5.46046 0.61958 0.77255 0.48576 0.95510
193 2.51538 5.17588 2.82464 2.30904 4.47754 3.56766 3.75992
3.63454 2.40195 2.84037 2.71812 2.86412 3.40903 2.26400 2.90617
2.30652 2.89028 3.54297 5.63544 4.25235 261 q - - H
2.68618 4.42225 2.77519 2.73123 3.46354 2.40513 3.72494
3.29354 2.67741 2.69355 4.24690 2.90347 2.73739 3.18146 2.89801
2.37887 2.77519 2.98518 4.58477 3.61503
0.01309 4.73812 5.46046 0.61958 0.77255 0.48576 0.95510
194 4.04224 5.23940 5.94143 5.37208 2.86561 5.44029 5.66840
1.82504 5.24219 0.54655 3.16794 5.57904 5.43597 5.08207 5.18173
4.84128 4.24249 2.78208 5.63198 4.61804 262 L - - C
2.68622 4.42229 2.77524 2.73111 3.46358 2.40505 3.72499
3.29358 2.67745 2.69359 4.24694 2.90351 2.73727 3.18124 2.89805
2.37891 2.77524 2.98523 4.58481 3.61507
0.04871 3.13972 5.46046 1.00991 0.45295 0.48576 0.95510
195 2.46903 5.27703 2.42522 2.23933 4.61806 2.79713 3.17440
4.10112 2.28137 3.58050 3.94934 2.92654 3.94438 2.73498 2.22287
2.37529 3.00726 3.46239 5.71203 4.30359 267 r - - T
2.68618 4.42225 2.77519 2.73123 3.46354 2.40513 3.72494
3.29354 2.67741 2.69355 4.24690 2.90347 2.73739 3.18146 2.89801
2.37887 2.77519 2.98518 4.58477 3.61503
0.01309 4.73812 5.46046 0.61958 0.77255 0.48576 0.95510
196 2.78827 3.28510 4.66306 3.43292 2.80657 3.94851 4.27777
2.09933 3.85849 1.91746 3.29481 4.08116 4.31285 4.00664 3.89841
3.06172 2.56947 1.60265 4.03003 3.62040 268 v - - -
2.68618 4.42225 2.77519 2.73123 3.46354 2.40513 3.72494
3.29354 2.67741 2.69355 4.24690 2.90347 2.73739 3.18146 2.89801
2.37887 2.77519 2.98518 4.58477 3.61503
0.01309 4.73812 5.46046 0.61958 0.77255 0.48576 0.95510
197 2.67912 5.16353 2.46379 2.52000 3.66590 3.56985 3.76267
3.61963 1.91222 3.44817 3.73475 2.83376 3.61525 2.87711 2.40200
2.40610 2.91462 3.04103 5.62595 3.70591 269 k - - -
2.68618 4.42225 2.77519 2.73123 3.46354 2.40513 3.72494
3.29354 2.67741 2.69355 4.24690 2.90347 2.73739 3.18146 2.89801
2.37887 2.77519 2.98518 4.58477 3.61503
0.01309 4.73812 5.46046 0.61958 0.77255 0.48576 0.95510
198 2.55246 5.24654 2.93696 2.19776 4.57377 3.55828 3.74893
4.04680 2.43378 3.54397 4.29275 2.79850 2.13913 2.74127 2.35741
2.66410 2.46865 3.39861 5.69004 4.29071 270 p - - T
2.68636 4.42243 2.77538 2.73064 3.46343 2.40526 3.72513
3.29372 2.67759 2.69355 4.24708 2.90345 2.73748 3.18130 2.89819
2.37838 2.77538 2.98509 4.58495 3.61521
0.27668 1.43779 5.46046 0.87357 0.54035 0.48576 0.95510

```
    199   2.78852  5.25599  2.35453  2.48848  4.58326  1.78186  3.35920
3.80165  2.25847  3.40383  4.30272  2.83847  3.45297  2.85789  2.80976
2.68027  3.02023  3.63732  5.69907  4.29878    274 g - - T
          2.68618  4.42225  2.77519  2.73123  3.46354  2.40513  3.72494
3.29354  2.67741  2.69355  4.24690  2.90347  2.73739  3.18146  2.89801
2.37887  2.77519  2.98518  4.58477  3.61503
          0.01309  4.73812  5.46046  0.61958  0.77255  0.48576  0.95510
    200   4.07586  6.10722  3.27679  0.24975  5.62874  4.12322  4.89501
5.31118  3.93081  4.79767  5.82524  3.85129  4.78870  4.14974  4.37491
4.01078  4.41405  4.92831  6.59487  5.46044    275 E - - S
          2.68618  4.42225  2.77519  2.73123  3.46354  2.40513  3.72494
3.29354  2.67741  2.69355  4.24690  2.90347  2.73739  3.18146  2.89801
2.37887  2.77519  2.98518  4.58477  3.61503
          0.01309  4.73812  5.46046  0.61958  0.77255  0.48576  0.95510
    201   1.70087  3.93301  3.49870  2.93593  3.78923  3.70748  3.95839
2.99015  2.73499  2.85304  3.72819  3.07258  4.09690  3.22528  3.05056
2.60848  2.17989  2.41074  5.19647  3.93940    276 a - - E
          2.68618  4.42225  2.77519  2.73123  3.46354  2.40513  3.72494
3.29354  2.67741  2.69355  4.24690  2.90347  2.73739  3.18146  2.89801
2.37887  2.77519  2.98518  4.58477  3.61503
          0.01309  4.73812  5.46046  0.61958  0.77255  0.48576  0.95510
    202   3.30233  4.60892  5.19405  4.63480  3.69371  4.62501  5.05393
1.72076  4.49484  1.26113  3.55324  4.74401  4.91956  4.65821  4.58432
3.97060  2.41996  1.45146  5.49953  4.33916    277 l - - E
          2.68618  4.42225  2.77519  2.73123  3.46354  2.40513  3.72494
3.29354  2.67741  2.69355  4.24690  2.90347  2.73739  3.18146  2.89801
2.37887  2.77519  2.98518  4.58477  3.61503
          0.01309  4.73812  5.46046  0.61958  0.77255  0.48576  0.95510
    203   1.05700  4.48238  4.44113  3.93357  3.85100  3.55444  4.61967
2.29896  3.83789  2.79036  3.79459  4.08968  4.42407  4.09072  4.05887
3.26431  3.22810  1.68920  5.42098  4.22227    278 a - - E
          2.68618  4.42225  2.77519  2.73123  3.46354  2.40513  3.72494
3.29354  2.67741  2.69355  4.24690  2.90347  2.73739  3.18146  2.89801
2.37887  2.77519  2.98518  4.58477  3.61503
          0.01309  4.73812  5.46046  0.61958  0.77255  0.48576  0.95510
    204   3.59032  4.81006  5.61088  5.13014  3.54981  5.13199  5.76529
1.68320  5.05480  2.51055  3.82468  5.27970  5.36744  5.26900  5.19943
4.54884  3.84859  0.58091  6.08924  4.86158    279 V - - E
          2.68618  4.42225  2.77519  2.73123  3.46354  2.40513  3.72494
3.29354  2.67741  2.69355  4.24690  2.90347  2.73739  3.18146  2.89801
2.37887  2.77519  2.98518  4.58477  3.61503
          0.01309  4.73812  5.46046  0.61958  0.77255  0.48576  0.95510
    205   2.80048  3.87505  3.29280  2.76136  4.12306  3.63177  3.23788
3.55449  2.73625  2.76469  4.02588  1.35294  4.05544  3.09408  3.16781
2.38456  3.05252  3.24810  5.44189  4.12401    280 n - - E
          2.68618  4.42225  2.77519  2.73123  3.46354  2.40513  3.72494
3.29354  2.67741  2.69355  4.24690  2.90347  2.73739  3.18146  2.89801
2.37887  2.77519  2.98518  4.58477  3.61503
          0.01309  4.73812  5.46046  0.61958  0.77255  0.48576  0.95510
    206   2.59133  2.07302  4.57232  3.96946  2.63754  3.93807  4.26141
2.37252  3.79268  2.00133  3.31108  4.03932  4.30406  3.95751  3.86489
2.15391  3.02288  2.44006  3.57951  3.62747    281 l - - E
          2.68618  4.42225  2.77519  2.73123  3.46354  2.40513  3.72494
3.29354  2.67741  2.69355  4.24690  2.90347  2.73739  3.18146  2.89801
2.37887  2.77519  2.98518  4.58477  3.61503
          0.01309  4.73812  5.46046  0.61958  0.77255  0.48576  0.95510
```

```
      207   2.36084  4.34962  3.97313  3.39660  3.46447  3.83521  3.57088
  2.60812  3.31501  2.32903  2.84559  3.70526  3.30644  3.19716  3.58108
  2.58468  3.01544  1.56637  4.95127  3.74455    282 v - - E
            2.68618  4.42225  2.77519  2.73123  3.46354  2.40513  3.72494
  3.29354  2.67741  2.69355  4.24690  2.90347  2.73739  3.18146  2.89801
  2.37887  2.77519  2.98518  4.58477  3.61503
            0.01309  4.73812  5.46046  0.61958  0.77255  0.48576  0.95510
      208   2.64434  4.26724  4.22472  3.63855  2.16782  3.34529  3.06462
  2.49353  3.52206  1.76760  2.86213  3.85504  3.32228  3.29034  3.70974
  3.16736  3.01639  2.52125  4.87701  3.22963    283 l - - S
            2.68618  4.42225  2.77519  2.73123  3.46354  2.40513  3.72494
  3.29354  2.67741  2.69355  4.24690  2.90347  2.73739  3.18146  2.89801
  2.37887  2.77519  2.98518  4.58477  3.61503
            0.06049  4.73812  2.99693  0.61958  0.77255  0.48576  0.95510
      209   2.18818  5.10229  3.08258  2.22966  3.54939  3.56236  3.42439
  3.82725  2.37859  3.37994  3.46242  3.06038  3.95397  2.14385  2.51823
  2.55394  2.99119  3.18683  5.57480  3.82115    284 q - - .
            2.68618  4.42225  2.77519  2.73123  3.46354  2.40513  3.72494
  3.29354  2.67741  2.69355  4.24690  2.90347  2.73739  3.18146  2.89801
  2.37887  2.77519  2.98518  4.58477  3.61503
            0.01372  4.69135  5.41370  0.61958  0.77255  0.58030  0.82037
      210   2.79643  4.39229  4.62973  4.04117  3.36129  4.12872  4.45984
  2.27369  3.88751  1.08766  2.32105  4.18653  4.47192  4.06525  4.00427
  2.75787  3.21208  2.46794  5.01389  3.85454    285 l - - G
            2.68618  4.42225  2.77519  2.73123  3.46354  2.40513  3.72494
  3.29354  2.67741  2.69355  4.24690  2.90347  2.73739  3.18146  2.89801
  2.37887  2.77519  2.98518  4.58477  3.61503
            0.01372  4.69135  5.41370  0.61958  0.77255  0.43088  1.04964
      211   2.79064  4.73934  3.38279  2.69939  3.01703  3.68518  1.61269
  3.30098  2.78554  2.96861  3.29900  3.29775  3.61819  2.87103  2.96045
  2.90750  3.02151  3.03799  5.26946  3.22399    286 h - - G
            2.68618  4.42225  2.77519  2.73123  3.46354  2.40513  3.72494
  3.29354  2.67741  2.69355  4.24690  2.90347  2.73739  3.18146  2.89801
  2.37887  2.77519  2.98518  4.58477  3.61503
            0.01309  4.73812  5.46046  0.61958  0.77255  0.48576  0.95510
      212   2.78133  5.18131  3.07655  2.51929  4.48493  3.20318  2.89138
  3.94084  2.12713  3.46768  4.23376  2.91628  3.72626  2.87397  1.99332
  2.51925  2.52809  3.14999  5.63875  3.31301    287 r - - C
            2.68618  4.42225  2.77519  2.73123  3.46354  2.40513  3.72494
  3.29354  2.67741  2.69355  4.24690  2.90347  2.73739  3.18146  2.89801
  2.37887  2.77519  2.98518  4.58477  3.61503
            0.01309  4.73812  5.46046  0.61958  0.77255  0.48576  0.95510
      213   2.78663  4.20543  4.49589  3.89649  2.47582  3.92570  3.73867
  2.14143  3.73354  1.60780  2.90119  4.00012  4.29187  3.91180  3.12544
  2.87254  2.59923  2.23863  4.82269  3.64004    288 l - - S
            2.68618  4.42225  2.77519  2.73123  3.46354  2.40513  3.72494
  3.29354  2.67741  2.69355  4.24690  2.90347  2.73739  3.18146  2.89801
  2.37887  2.77519  2.98518  4.58477  3.61503
            0.04898  4.73812  3.24295  0.61958  0.77255  0.48576  0.95510
      214   2.76934  4.38951  3.83198  3.25988  3.02962  3.25036  3.67132
  2.86558  2.91844  1.57951  3.49798  3.60992  2.53487  3.47256  3.49516
  2.85317  3.00124  2.51420  4.98219  3.16746    289 l - - S
            2.68568  4.42149  2.77408  2.73189  3.46416  2.40541  3.72531
  3.29502  2.67850  2.69344  4.24086  2.90151  2.73804  3.18165  2.89753
  2.37802  2.77668  2.98560  4.58625  3.61551
            0.28920  2.33131  1.87099  3.57266  0.02848  0.55856  0.84871
```

215 2.14181 5.20292 1.99555 2.29711 4.53085 3.14937 3.35668
4.00678 2.35635 3.50076 4.24930 2.67190 3.89220 2.79474 2.76384
2.61570 2.95999 3.58455 5.64489 3.69935 345 d - - -
2.68618 4.42225 2.77519 2.73123 3.46354 2.40513 3.72494
3.29354 2.67741 2.69355 4.24690 2.90347 2.73739 3.18146 2.89801
2.37887 2.77519 2.98518 4.58477 3.61503
0.01597 4.54069 5.26304 0.61958 0.77255 0.81420 0.58518
216 2.74783 5.23670 2.21879 1.77510 4.56744 3.49096 3.51180
4.04733 2.45677 3.53562 4.28386 2.96091 3.90157 2.40110 2.84814
2.28897 2.98159 3.08990 5.67570 4.26665 346 e - - S
2.68618 4.42225 2.77519 2.73123 3.46354 2.40513 3.72494
3.29354 2.67741 2.69355 4.24690 2.90347 2.73739 3.18146 2.89801
2.37887 2.77519 2.98518 4.58477 3.61503
0.01597 4.54069 5.26304 0.61958 0.77255 0.81420 0.58518
217 2.72641 5.08622 2.51461 2.49014 4.46535 3.16328 3.75839
3.92283 2.53121 3.45706 4.22801 3.02127 2.61000 2.87813 2.85620
1.87870 2.24639 3.51866 5.63277 4.25280 347 s - - S
2.68618 4.42225 2.77519 2.73123 3.46354 2.40513 3.72494
3.29354 2.67741 2.69355 4.24690 2.90347 2.73739 3.18146 2.89801
2.37887 2.77519 2.98518 4.58477 3.61503
0.01597 4.54069 5.26304 0.61958 0.77255 0.60472 0.79016
218 2.33538 4.55713 3.47164 2.77570 3.71642 2.36577 3.41816
3.08511 2.87918 2.21657 3.66060 3.35371 3.31857 3.19783 3.26768
2.90899 2.77734 2.13425 5.13075 3.87759 348 v - - S
2.68618 4.42225 2.77519 2.73123 3.46354 2.40513 3.72494
3.29354 2.67741 2.69355 4.24690 2.90347 2.73739 3.18146 2.89801
2.37887 2.77519 2.98518 4.58477 3.61503
0.01477 4.61810 5.34045 0.61958 0.77255 0.70524 0.68120
219 2.37479 3.95938 2.63888 2.49302 4.42372 3.23005 3.73557
3.87659 2.32863 3.41329 4.18357 2.88920 2.94977 2.85178 2.97755
1.93497 2.50169 3.48817 5.59283 4.21515 349 s - - S
2.68618 4.42225 2.77519 2.73123 3.46354 2.40513 3.72494
3.29354 2.67741 2.69355 4.24690 2.90347 2.73739 3.18146 2.89801
2.37887 2.77519 2.98518 4.58477 3.61503
0.01477 4.61810 5.34045 0.61958 0.77255 0.70524 0.68120
220 2.57739 4.41544 3.31021 3.11176 3.54607 3.73188 3.99178
2.36637 3.05895 1.86216 2.91235 3.26475 4.10996 3.15988 3.03195
2.61194 2.54356 2.49700 5.00273 3.77585 350 l - - C
2.68618 4.42225 2.77519 2.73123 3.46354 2.40513 3.72494
3.29354 2.67741 2.69355 4.24690 2.90347 2.73739 3.18146 2.89801
2.37887 2.77519 2.98518 4.58477 3.61503
0.01477 4.61810 5.34045 0.61958 0.77255 0.70524 0.68120
221 2.44991 5.13933 2.73878 1.98027 4.44272 3.16059 3.71806
3.30258 2.47175 3.23103 4.19147 2.70396 2.96502 2.82989 2.82812
2.50577 2.80195 2.92988 5.59809 4.21383 351 e - - C
2.68618 4.42225 2.77519 2.73123 3.46354 2.40513 3.72494
3.29354 2.67741 2.69355 4.24690 2.90347 2.73739 3.18146 2.89801
2.37887 2.77519 2.98518 4.58477 3.61503
0.01477 4.61810 5.34045 0.61958 0.77255 0.61648 0.77619
222 2.75628 5.22485 2.53986 2.37521 4.55130 3.07772 3.53312
3.11623 2.18151 3.52221 4.27147 2.41518 3.63571 2.82657 2.95996
1.89098 2.98826 3.60565 5.66848 4.26712 352 s - - C
2.68625 4.42232 2.77527 2.73130 3.46361 2.40490 3.72502
3.29361 2.67748 2.69362 4.24697 2.90354 2.73747 3.18153 2.89808
2.37845 2.77527 2.98525 4.58484 3.61510
0.62733 2.00930 1.10291 0.67160 0.71517 0.52878 0.88994

```
    223   2.64682  4.12199  3.04046  2.33477  4.10000  3.16657  3.15066
3.51837  2.46355  2.87464  3.92766  3.00199  2.74618  2.70675  2.40974
2.67783  2.87759  3.19220  5.35594  3.36450    355 e - - H
          2.68618  4.42225  2.77519  2.73123  3.46354  2.40513  3.72494
3.29354  2.67741  2.69355  4.24690  2.90347  2.73739  3.18146  2.89801
2.37887  2.77519  2.98518  4.58477  3.61503
          0.02010  4.31282  5.03516  0.61958  0.77255  0.90459  0.51870
    224   2.70417  4.62596  3.34733  2.77544  3.83084  3.21639  3.81506
2.75882  2.62428  2.57310  3.73726  3.23202  3.98285  3.03897  1.78849
2.83172  2.36073  2.91492  5.19009  3.92575    356 r - - H
          2.68618  4.42225  2.77519  2.73123  3.46354  2.40513  3.72494
3.29354  2.67741  2.69355  4.24690  2.90347  2.73739  3.18146  2.89801
2.37887  2.77519  2.98518  4.58477  3.61503
          0.01884  4.37681  5.09916  0.61958  0.77255  0.83399  0.56972
    225   2.75192  5.16001  1.61585  2.42006  4.52649  3.05398  3.74603
3.99432  2.53639  3.51359  4.28445  2.96381  2.98657  2.86635  2.79272
2.55168  2.56670  3.58109  5.67914  4.28100    357 d - - H
          2.68618  4.42225  2.77519  2.73123  3.46354  2.40513  3.72494
3.29354  2.67741  2.69355  4.24690  2.90347  2.73739  3.18146  2.89801
2.37887  2.77519  2.98518  4.58477  3.61503
          0.01761  4.44388  5.16622  0.61958  0.77255  0.64989  0.73836
    226   2.45254  5.12573  2.66534  2.14024  4.43107  3.22531  3.17814
3.88983  2.45057  2.87898  4.17743  2.99035  3.89838  2.80898  2.67722
2.21467  2.52002  3.18817  5.58325  4.19771    358 e - - H
          2.68618  4.42225  2.77519  2.73123  3.46354  2.40513  3.72494
3.29354  2.67741  2.69355  4.24690  2.90347  2.73739  3.18146  2.89801
2.37887  2.77519  2.98518  4.58477  3.61503
          0.01569  4.55812  5.28047  0.61958  0.77255  0.33330  1.26073
    227   2.65078  4.31773  4.76756  4.17228  1.67923  4.12756  4.47172
2.26598  3.99919  1.91456  3.37300  4.24041  4.47701  4.15693  4.06606
3.44054  2.54025  1.73713  4.99059  3.81456    359 f - - H
          2.68618  4.42225  2.77519  2.73123  3.46354  2.40513  3.72494
3.29354  2.67741  2.69355  4.24690  2.90347  2.73739  3.18146  2.89801
2.37887  2.77519  2.98518  4.58477  3.61503
          0.01309  4.73812  5.46046  0.61958  0.77255  0.48576  0.95510
    228   3.96936  5.18249  5.85249  5.30298  3.50152  5.38466  5.74543
2.21324  5.16447  0.49476  3.24257  5.52842  5.43141  5.09875  5.16134
4.79524  4.18672  2.20139  5.75558  4.77863    360 L - - H
          2.68618  4.42225  2.77519  2.73123  3.46354  2.40513  3.72494
3.29354  2.67741  2.69355  4.24690  2.90347  2.73739  3.18146  2.89801
2.37887  2.77519  2.98518  4.58477  3.61503
          0.01309  4.73812  5.46046  0.61958  0.77255  0.48576  0.95510
    229   2.70123  5.26521  2.91192  2.40315  4.60090  2.74976  3.30807
4.07942  2.14595  3.56586  4.30868  2.26049  3.94875  2.84259  2.23226
2.24549  3.01020  3.65088  4.35143  4.29895    361 k - - H
          2.68618  4.42225  2.77519  2.73123  3.46354  2.40513  3.72494
3.29354  2.67741  2.69355  4.24690  2.90347  2.73739  3.18146  2.89801
2.37887  2.77519  2.98518  4.58477  3.61503
          0.01309  4.73812  5.46046  0.61958  0.77255  0.48576  0.95510
    230   2.06843  3.99294  3.70038  2.74937  2.96410  3.77137  4.02870
2.83185  3.08218  2.00813  3.08390  3.21950  4.14991  3.08845  2.92230
2.76797  2.60694  2.66477  5.06204  3.83234    362 l - - H
          2.68618  4.42225  2.77519  2.73123  3.46354  2.40513  3.72494
3.29354  2.67741  2.69355  4.24690  2.90347  2.73739  3.18146  2.89801
2.37887  2.77519  2.98518  4.58477  3.61503
          0.01309  4.73812  5.46046  0.61958  0.77255  0.48576  0.95510
```

```
    231   2.52829  4.50315  5.12919  4.55322  2.73861  4.47212  4.86680
1.53429  4.38993  1.71730  3.49433  4.61231  4.78377  4.53505  4.44337
3.80748  3.42299  1.36783  5.31511  4.14805    363 v - - H
          2.68618  4.42225  2.77519  2.73123  3.46354  2.40513  3.72494
3.29354  2.67741  2.69355  4.24690  2.90347  2.73739  3.18146  2.89801
2.37887  2.77519  2.98518  4.58477  3.61503
          0.01309  4.73812  5.46046  0.61958  0.77255  0.48576  0.95510
    232   2.87944  5.35386  3.39865  2.77762  4.72072  3.43576  3.84595
4.13010  1.46142  3.61231  4.41179  3.11100  4.15072  2.74436  1.59365
3.02353  3.24261  3.76203  3.13928  4.43208    364 k - - H
          2.68618  4.42225  2.77519  2.73123  3.46354  2.40513  3.72494
3.29354  2.67741  2.69355  4.24690  2.90347  2.73739  3.18146  2.89801
2.37887  2.77519  2.98518  4.58477  3.61503
          0.01309  4.73812  5.46046  0.61958  0.77255  0.48576  0.95510
    233   2.53403  5.25798  2.81399  2.48550  4.58983  2.64193  3.31507
4.06540  2.40467  3.55743  4.30374  3.03114  3.95283  2.56525  2.31713
1.82747  2.84518  3.64184  5.69913  4.29764    365 s - - H
          2.68618  4.42225  2.77519  2.73123  3.46354  2.40513  3.72494
3.29354  2.67741  2.69355  4.24690  2.90347  2.73739  3.18146  2.89801
2.37887  2.77519  2.98518  4.58477  3.61503
          0.01309  4.73812  5.46046  0.61958  0.77255  0.48576  0.95510
    234   3.37477  4.67666  5.27424  4.70239  3.57365  4.68385  5.07263
1.69172  4.55420  0.99026  3.12006  4.80892  4.94689  4.66537  4.61191
4.02748  2.86685  1.82279  5.44995  4.32985    366 l - - C
          2.68618  4.42225  2.77519  2.73123  3.46354  2.40513  3.72494
3.29354  2.67741  2.69355  4.24690  2.90347  2.73739  3.18146  2.89801
2.37887  2.77519  2.98518  4.58477  3.61503
          0.01309  4.73812  5.46046  0.61958  0.77255  0.48576  0.95510
    235   2.66684  5.26905  3.07610  2.54026  4.62227  3.28926  3.78537
4.08715  2.16825  3.58184  4.33941  1.98046  3.99352  2.89216  2.26532
1.86831  3.06428  3.67102  5.72188  4.33817    367 s - - -
          2.68618  4.42225  2.77519  2.73123  3.46354  2.40513  3.72494
3.29354  2.67741  2.69355  4.24690  2.90347  2.73739  3.18146  2.89801
2.37887  2.77519  2.98518  4.58477  3.61503
          0.01309  4.73812  5.46046  0.61958  0.77255  0.48576  0.95510
    236   2.44884  4.65812  3.99827  3.87604  5.09310  3.40060  4.97549
4.54775  4.02802  4.23973  5.05372  3.90928  0.57298  4.28395  4.27351
2.39371  3.25719  3.85520  6.42542  5.24875    368 P - - -
          2.68618  4.42225  2.77519  2.73123  3.46354  2.40513  3.72494
3.29354  2.67741  2.69355  4.24690  2.90347  2.73739  3.18146  2.89801
2.37887  2.77519  2.98518  4.58477  3.61503
          0.01309  4.73812  5.46046  0.61958  0.77255  0.48576  0.95510
    237   2.79448  5.28189  2.63411  2.43050  4.62189  3.39712  3.74934
3.64394  1.68888  3.58195  4.32559  2.48360  3.95888  2.84921  2.08921
2.75984  2.72462  3.67016  5.71468  4.31319    369 k - - S
          2.68618  4.42225  2.77519  2.73123  3.46354  2.40513  3.72494
3.29354  2.67741  2.69355  4.24690  2.90347  2.73739  3.18146  2.89801
2.37887  2.77519  2.98518  4.58477  3.61503
          0.01309  4.73812  5.46046  0.61958  0.77255  0.48576  0.95510
    238   2.86144  4.47988  5.01751  4.45384  3.69181  3.54672  4.85376
1.39252  4.30673  1.90034  3.60903  4.54665  4.76280  4.49638  4.40389
3.76407  3.39126  1.22485  5.36859  4.17884    370 v - - E
          2.68618  4.42225  2.77519  2.73123  3.46354  2.40513  3.72494
3.29354  2.67741  2.69355  4.24690  2.90347  2.73739  3.18146  2.89801
2.37887  2.77519  2.98518  4.58477  3.61503
          0.01309  4.73812  5.46046  0.61958  0.77255  0.48576  0.95510
```

```
     239   3.37462  4.67214  5.31850  4.74075  2.54004  4.67797  5.04920
2.11751  4.58198  1.86085  2.39252  4.82041  4.93653  4.66849  4.61289
4.02143  3.60542  0.98712  5.39919  4.27730    371 v - - E
          2.68618  4.42225  2.77519  2.73123  3.46354  2.40513  3.72494
3.29354  2.67741  2.69355  4.24690  2.90347  2.73739  3.18146  2.89801
2.37887  2.77519  2.98518  4.58477  3.61503
          0.01309  4.73812  5.46046  0.61958  0.77255  0.48576  0.95510
     240   3.11543  4.61192  4.71314  4.22080  3.93712  4.19952  4.91295
2.35989  4.09066  2.49162  3.82557  4.38201  4.68560  4.37431  4.29941
3.58192  1.28989  1.21053  5.64387  4.43605    372 v - - E
          2.68618  4.42225  2.77519  2.73123  3.46354  2.40513  3.72494
3.29354  2.67741  2.69355  4.24690  2.90347  2.73739  3.18146  2.89801
2.37887  2.77519  2.98518  4.58477  3.61503
          0.01309  4.73812  5.46046  0.61958  0.77255  0.48576  0.95510
     241   2.83088  3.16441  4.73771  4.12774  2.61464  3.99978  3.70558
2.47682  3.92710  1.59750  2.76260  4.14287  4.35805  4.06508  3.95519
3.31009  3.06275  1.57732  4.83140  3.65699    373 v - - E
          2.68618  4.42225  2.77519  2.73123  3.46354  2.40513  3.72494
3.29354  2.67741  2.69355  4.24690  2.90347  2.73739  3.18146  2.89801
2.37887  2.77519  2.98518  4.58477  3.61503
          0.01309  4.73812  5.46046  0.61958  0.77255  0.48576  0.95510
     242   2.12510  3.57737  4.23175  3.66315  3.48107  2.95375  4.25090
2.49110  3.55514  2.56590  3.48485  3.86386  4.24950  3.78479  3.75671
2.62935  2.43834  1.48632  4.98460  3.79123    374 v - - E
          2.68618  4.42225  2.77519  2.73123  3.46354  2.40513  3.72494
3.29354  2.67741  2.69355  4.24690  2.90347  2.73739  3.18146  2.89801
2.37887  2.77519  2.98518  4.58477  3.61503
          0.01309  4.73812  5.46046  0.61958  0.77255  0.48576  0.95510
     243   3.10356  5.64223  2.05626  0.92900  4.93456  3.60702  3.43766
3.79406  2.85911  3.93011  4.72020  3.06542  4.13043  3.12047  3.39573
3.02629  3.35953  4.01511  6.07442  4.61633    375 e - - E
          2.68618  4.42225  2.77519  2.73123  3.46354  2.40513  3.72494
3.29354  2.67741  2.69355  4.24690  2.90347  2.73739  3.18146  2.89801
2.37887  2.77519  2.98518  4.58477  3.61503
          0.01309  4.73812  5.46046  0.61958  0.77255  0.48576  0.95510
     244   2.78333  5.24040  3.05263  2.16963  4.56620  3.56346  3.43038
3.52181  2.40607  3.53580  4.28682  2.82884  3.95611  1.71237  2.41528
2.49890  3.01383  3.38411  5.68320  3.85779    376 q - - E
          2.68618  4.42225  2.77519  2.73123  3.46354  2.40513  3.72494
3.29354  2.67741  2.69355  4.24690  2.90347  2.73739  3.18146  2.89801
2.37887  2.77519  2.98518  4.58477  3.61503
          0.01309  4.73812  5.46046  0.61958  0.77255  0.48576  0.95510
     245   3.26989  5.91136  1.75426  1.04925  5.21254  3.20861  4.10094
4.74748  3.07055  4.20366  5.01372  2.31820  4.20480  3.24695  3.66525
2.70705  3.54496  4.28762  6.34346  4.82734    377 e - - S
          2.68621  4.42228  2.77523  2.73126  3.46357  2.40498  3.72498
3.29357  2.67744  2.69346  4.24693  2.90350  2.73743  3.18149  2.89804
2.37890  2.77523  2.98521  4.58398  3.61506
          0.06706  2.80334  5.46046  0.65814  0.72943  0.48576  0.95510
     246   1.86192  3.08586  4.07583  3.50024  3.45515  2.68234  4.16309
2.79806  3.40802  2.31991  2.86324  3.76856  4.22476  3.65630  3.64716
2.38112  2.81247  2.17096  4.94920  3.74845    380 a - - S
          2.68618  4.42225  2.77519  2.73123  3.46354  2.40513  3.72494
3.29354  2.67741  2.69355  4.24690  2.90347  2.73739  3.18146  2.89801
2.37887  2.77519  2.98518  4.58477  3.61503
          0.01309  4.73812  5.46046  0.61958  0.77255  0.48576  0.95510
```

247 2.71070 5.33752 2.01507 2.07415 4.67261 2.84574 3.78870
4.15686 2.55032 3.64044 3.96056 1.97107 3.98135 2.89322 3.05028
2.43918 2.94239 3.72424 5.77761 4.36242 381 n - - -
2.68618 4.42225 2.77519 2.73123 3.46354 2.40513 3.72494
3.29354 2.67741 2.69355 4.24690 2.90347 2.73739 3.18146 2.89801
2.37887 2.77519 2.98518 4.58477 3.61503
0.01309 4.73812 5.46046 0.61958 0.77255 0.48576 0.95510
248 2.78243 3.74031 3.68190 3.11351 3.62446 3.41117 1.99549
2.79596 3.06218 2.19895 3.59424 3.51290 4.14602 3.36382 2.85424
2.76916 2.62498 2.76256 5.07313 2.80510 382 h - - S
2.68612 4.42231 2.77516 2.73129 3.46360 2.40519 3.72501
3.29360 2.67747 2.69349 4.24640 2.90330 2.73746 3.18153 2.89807
2.37882 2.77526 2.98503 4.58483 3.61509
0.27460 1.56523 3.47099 0.15551 1.93779 0.48576 0.95510
249 2.94349 5.24536 2.71603 2.65260 4.84419 2.11955 4.07941
4.32122 2.95750 3.85870 4.65067 1.18565 4.10669 3.22705 3.46349
2.64844 2.69216 3.85940 6.03268 4.63531 384 n - - .
2.68618 4.42225 2.77519 2.73123 3.46354 2.40513 3.72494
3.29354 2.67741 2.69355 4.24690 2.90347 2.73739 3.18146 2.89801
2.37887 2.77519 2.98518 4.58477 3.61503
0.01346 4.71092 5.43326 0.61958 0.77255 0.45193 1.01170
250 2.49015 5.27474 2.43817 2.33877 4.60867 2.57711 3.49300
4.08779 2.42050 3.57561 4.31987 2.85399 3.95411 2.85273 2.84965
1.91170 2.49820 3.65970 5.71444 4.30838 385 s - - S
2.68618 4.42225 2.77519 2.73123 3.46354 2.40513 3.72494
3.29354 2.67741 2.69355 4.24690 2.90347 2.73739 3.18146 2.89801
2.37887 2.77519 2.98518 4.58477 3.61503
0.01309 4.73812 5.46046 0.61958 0.77255 0.48576 0.95510
251 2.21505 5.18883 2.96171 2.17667 4.49535 2.58853 3.23888
3.95493 2.50912 3.25066 3.74974 3.04961 2.99967 2.72077 2.99448
2.25362 2.80877 3.32922 5.64555 4.25920 386 e - - S
2.68624 4.42231 2.77491 2.73113 3.46325 2.40495 3.72501
3.29360 2.67747 2.69361 4.24696 2.90353 2.73746 3.18153 2.89807
2.37893 2.77526 2.98525 4.58483 3.61510
0.04898 3.13380 5.46046 1.27571 0.32744 0.48576 0.95510
252 2.70209 5.13914 2.86789 2.38564 3.47455 2.97999 3.76862
3.66075 2.53037 3.22965 4.19518 2.86836 2.66981 2.73069 2.84605
2.17435 2.38211 3.22214 5.60726 4.23322 393 s - - H
2.68618 4.42225 2.77519 2.73123 3.46354 2.40513 3.72494
3.29354 2.67741 2.69355 4.24690 2.90347 2.73739 3.18146 2.89801
2.37887 2.77519 2.98518 4.58477 3.61503
0.01309 4.73812 5.46046 0.61958 0.77255 0.48576 0.95510
253 4.09335 5.27766 5.48530 5.14219 0.68042 5.01710 4.19351
3.37068 4.93498 1.68098 3.89195 4.80873 5.25555 4.79857 4.85703
4.38789 4.30329 3.45867 3.43924 2.61675 394 F - - H
2.68618 4.42225 2.77519 2.73123 3.46354 2.40513 3.72494
3.29354 2.67741 2.69355 4.24690 2.90347 2.73739 3.18146 2.89801
2.37887 2.77519 2.98518 4.58477 3.61503
0.01309 4.73812 5.46046 0.61958 0.77255 0.48576 0.95510
254 2.48691 4.36132 3.94478 2.89283 3.13657 3.82972 4.10881
2.82718 3.29075 1.52578 2.61645 3.68754 3.47078 3.55695 3.00257
2.91292 3.01572 2.37829 4.96130 3.75272 395 l - - H
2.68618 4.42225 2.77519 2.73123 3.46354 2.40513 3.72494
3.29354 2.67741 2.69355 4.24690 2.90347 2.73739 3.18146 2.89801
2.37887 2.77519 2.98518 4.58477 3.61503
0.01309 4.73812 5.46046 0.61958 0.77255 0.48576 0.95510

255 2.44457 5.28047 2.50520 2.16585 4.62165 2.95191 3.73998
4.10519 2.47711 3.58409 4.32234 2.34331 3.21765 2.49811 2.61691
2.74173 2.55760 3.66906 5.71534 3.79739 396 e - - H
2.68618 4.42225 2.77519 2.73123 3.46354 2.40513 3.72494
3.29354 2.67741 2.69355 4.24690 2.90347 2.73739 3.18146 2.89801
2.37887 2.77519 2.98518 4.58477 3.61503
0.01309 4.73812 5.46046 0.61958 0.77255 0.48576 0.95510
256 3.11403 5.39659 3.33127 2.61081 4.80187 2.55995 3.91657
4.20563 2.41698 3.69033 4.50409 3.30574 4.20283 2.82752 1.03340
3.09911 3.01364 3.83761 5.80059 4.51816 397 r - - H
2.68618 4.42225 2.77519 2.73123 3.46354 2.40513 3.72494
3.29354 2.67741 2.69355 4.24690 2.90347 2.73739 3.18146 2.89801
2.37887 2.77519 2.98518 4.58477 3.61503
0.01309 4.73812 5.46046 0.61958 0.77255 0.48576 0.95510
257 3.62330 4.90833 5.43822 4.89375 1.11454 4.87456 4.99435
2.54838 4.73616 1.47261 3.33823 4.96251 5.08918 4.75432 4.75175
4.23155 3.84904 1.78662 5.20884 3.88451 398 f - - H
2.68618 4.42225 2.77519 2.73123 3.46354 2.40513 3.72494
3.29354 2.67741 2.69355 4.24690 2.90347 2.73739 3.18146 2.89801
2.37887 2.77519 2.98518 4.58477 3.61503
0.01309 4.73812 5.46046 0.61958 0.77255 0.48576 0.95510
258 2.51330 3.76365 3.88058 3.30693 3.50634 3.31659 4.08853
2.31645 2.82340 2.15681 3.07776 3.44577 4.19008 3.51133 2.97628
2.39717 2.50950 2.24759 4.98338 3.77005 399 l - - H
2.68618 4.42225 2.77519 2.73123 3.46354 2.40513 3.72494
3.29354 2.67741 2.69355 4.24690 2.90347 2.73739 3.18146 2.89801
2.37887 2.77519 2.98518 4.58477 3.61503
0.01309 4.73812 5.46046 0.61958 0.77255 0.48576 0.95510
259 2.88954 5.41332 2.56322 1.26551 4.74805 3.57150 3.46721
4.23847 2.43217 3.71309 4.46338 2.46990 4.01014 2.93032 2.89044
2.50322 3.12769 3.79971 5.84463 4.42013 400 e - - H
2.68618 4.42225 2.77519 2.73123 3.46354 2.40513 3.72494
3.29354 2.67741 2.69355 4.24690 2.90347 2.73739 3.18146 2.89801
2.37887 2.77519 2.98518 4.58477 3.61503
0.01309 4.73812 5.46046 0.61958 0.77255 0.48576 0.95510
260 1.47247 3.66219 3.53498 2.98994 4.04154 3.23819 4.05519
3.42617 2.71149 2.86712 3.95630 3.40979 4.08586 3.28884 3.36622
1.81173 2.69977 3.12564 5.41331 4.14761 401 a - - H
2.68618 4.42225 2.77519 2.73123 3.46354 2.40513 3.72494
3.29354 2.67741 2.69355 4.24690 2.90347 2.73739 3.18146 2.89801
2.37887 2.77519 2.98518 4.58477 3.61503
0.01309 4.73812 5.46046 0.61958 0.77255 0.48576 0.95510
261 3.11364 3.52874 5.00114 4.39676 2.97594 4.31076 4.63286
2.40454 4.21099 0.85565 2.93457 4.44697 4.61993 4.30923 4.23476
3.62963 3.34084 2.27225 5.05704 3.54555 402 l - - H
2.68618 4.42225 2.77519 2.73123 3.46354 2.40513 3.72494
3.29354 2.67741 2.69355 4.24690 2.90347 2.73739 3.18146 2.89801
2.37887 2.77519 2.98518 4.58477 3.61503
0.01309 4.73812 5.46046 0.61958 0.77255 0.48576 0.95510
262 2.59956 5.25444 2.65112 2.12690 3.34467 3.55447 2.48479
4.06254 2.37749 3.55387 4.29873 2.68628 3.94797 2.53346 2.62821
2.66863 3.00755 3.63781 5.69516 3.12721 403 e - - H
2.68618 4.42225 2.77519 2.73123 3.46354 2.40513 3.72494
3.29354 2.67741 2.69355 4.24690 2.90347 2.73739 3.18146 2.89801
2.37887 2.77519 2.98518 4.58477 3.61503
0.01309 4.73812 5.46046 0.61958 0.77255 0.48576 0.95510

263 2.81798 3.57863 4.14550 3.56727 2.69996 3.89274 2.74801
2.78720 3.46495 2.52320 3.44371 3.81613 4.26303 3.33493 3.68425
2.96599 2.47336 2.58914 3.77417 1.70956 404 y - - H
2.68618 4.42225 2.77519 2.73123 3.46354 2.40513 3.72494
3.29354 2.67741 2.69355 4.24690 2.90347 2.73739 3.18146 2.89801
2.37887 2.77519 2.98518 4.58477 3.61503
0.01309 4.73812 5.46046 0.61958 0.77255 0.48576 0.95510
264 3.59903 3.98716 4.97707 4.53433 1.88184 4.55489 3.39770
3.42034 4.36845 2.98701 4.05788 4.39854 4.88764 4.40768 4.41596
3.88276 3.82491 2.81426 4.21393 0.76390 405 y - - H
2.68618 4.42225 2.77519 2.73123 3.46354 2.40513 3.72494
3.29354 2.67741 2.69355 4.24690 2.90347 2.73739 3.18146 2.89801
2.37887 2.77519 2.98518 4.58477 3.61503
0.01309 4.73812 5.46046 0.61958 0.77255 0.48576 0.95510
265 2.20378 4.63257 3.48329 2.92044 3.79887 3.27249 3.95026
3.17190 2.88878 2.77283 3.18672 3.37167 4.09315 3.21211 2.97484
1.75289 2.33672 2.92500 3.53922 3.94313 406 s - - H
2.68618 4.42225 2.77519 2.73123 3.46354 2.40513 3.72494
3.29354 2.67741 2.69355 4.24690 2.90347 2.73739 3.18146 2.89801
2.37887 2.77519 2.98518 4.58477 3.61503
0.01309 4.73812 5.46046 0.61958 0.77255 0.48576 0.95510
266 1.44947 4.58489 3.56562 3.00379 3.76232 3.71801 3.99638
2.87994 2.74416 2.82654 3.34555 3.43295 4.11640 3.28246 3.34621
2.29995 2.53947 2.64869 5.18313 3.93446 407 a - - H
2.68618 4.42225 2.77519 2.73123 3.46354 2.40513 3.72494
3.29354 2.67741 2.69355 4.24690 2.90347 2.73739 3.18146 2.89801
2.37887 2.77519 2.98518 4.58477 3.61503
0.01309 4.73812 5.46046 0.61958 0.77255 0.48576 0.95510
267 2.67235 3.49356 4.75862 4.15000 2.97293 4.02312 4.35762
2.13613 3.95093 1.40308 2.56494 4.16668 3.66846 4.08925 3.97985
3.33448 3.08096 1.94266 4.85730 3.68464 408 l - - H
2.68618 4.42225 2.77519 2.73123 3.46354 2.40513 3.72494
3.29354 2.67741 2.69355 4.24690 2.90347 2.73739 3.18146 2.89801
2.37887 2.77519 2.98518 4.58477 3.61503
0.01309 4.73812 5.46046 0.61958 0.77255 0.48576 0.95510
268 4.14901 5.34907 5.62589 5.24834 0.71865 5.18599 4.64796
2.99577 5.06665 1.41816 3.54450 5.10960 5.36599 4.94862 5.01127
4.61310 4.36842 3.19733 4.72941 3.12549 409 f - - H
2.68618 4.42225 2.77519 2.73123 3.46354 2.40513 3.72494
3.29354 2.67741 2.69355 4.24690 2.90347 2.73739 3.18146 2.89801
2.37887 2.77519 2.98518 4.58477 3.61503
0.01309 4.73812 5.46046 0.61958 0.77255 0.48576 0.95510
269 3.60251 6.35579 0.82995 1.29618 5.61854 3.66604 4.32615
5.21943 3.50200 4.65529 5.56638 3.11151 4.35801 3.51158 4.21357
3.42072 3.92045 4.73761 6.78296 5.17473 410 d - - H
2.68618 4.42225 2.77519 2.73123 3.46354 2.40513 3.72494
3.29354 2.67741 2.69355 4.24690 2.90347 2.73739 3.18146 2.89801
2.37887 2.77519 2.98518 4.58477 3.61503
0.01309 4.73812 5.46046 0.61958 0.77255 0.48576 0.95510
270 2.22675 2.79062 4.07135 3.55820 3.98155 3.57583 4.39549
3.31921 3.49012 3.06186 2.98650 3.76479 4.17417 3.76544 3.78295
1.05806 2.76693 3.01992 5.43035 4.22495 411 s - - H
2.68618 4.42225 2.77519 2.73123 3.46354 2.40513 3.72494
3.29354 2.67741 2.69355 4.24690 2.90347 2.73739 3.18146 2.89801
2.37887 2.77519 2.98518 4.58477 3.61503
0.01309 4.73812 5.46046 0.61958 0.77255 0.48576 0.95510

```
    271   2.88834  4.27173  4.74010  4.13425  3.09456  4.05401  4.37422
2.25149  3.94569  1.10758  3.28174  4.17798  4.40392  4.08487  3.98976
3.36333  2.63034  2.18787  3.73710  3.43156    412 l - - H
          2.68624  4.42231  2.77473  2.73080  3.46360  2.40519  3.72501
3.29360  2.67747  2.69361  4.24696  2.90353  2.73746  3.18153  2.89807
2.37893  2.77526  2.98525  4.58483  3.61509
          0.24509  1.54592  5.46046  0.15319  1.95169  0.48576  0.95510
    272   2.36560  5.44084  2.05533  1.52741  4.77169  2.66431  3.41424
4.26271  2.65044  3.74352  4.50056  3.03893  4.02936  2.96620  3.16391
2.42808  3.16360  3.82692  5.88195  4.45211    414 e - - H
          2.68618  4.42225  2.77519  2.73123  3.46354  2.40513  3.72494
3.29354  2.67741  2.69355  4.24690  2.90347  2.73739  3.18146  2.89801
2.37887  2.77519  2.98518  4.58477  3.61503
          0.01309  4.73812  5.46046  0.61958  0.77255  0.48576  0.95510
    273   1.96259  4.96475  3.03100  2.46729  4.21255  2.81490  3.82367
3.62801  2.62781  3.23469  3.73679  3.14985  3.48388  2.97627  3.09014
2.47410  2.11011  2.72658  5.48171  4.14921    415 a - - H
          2.68618  4.42225  2.77519  2.73123  3.46354  2.40513  3.72494
3.29354  2.67741  2.69355  4.24690  2.90347  2.73739  3.18146  2.89801
2.37887  2.77519  2.98518  4.58477  3.61503
          0.01309  4.73812  5.46046  0.61958  0.77255  0.48576  0.95510
    274   2.63341  5.05448  3.13262  2.57553  3.73984  2.75042  3.79105
3.47970  2.18710  3.32448  3.84136  2.37039  3.56490  2.92492  3.04413
2.27511  2.22653  3.25685  5.54247  4.18904    416 k - - H
          2.68615  4.42238  2.77533  2.73119  3.46367  2.40477  3.72508
3.29367  2.67754  2.69368  4.24703  2.90360  2.73736  3.18159  2.89814
2.37852  2.77533  2.98510  4.58490  3.61516
          0.18292  3.13972  2.08850  1.80156  0.18037  0.48576  0.95510
    275   2.43107  4.23533  4.71458  4.11005  2.63605  4.03109  4.35986
2.27644  3.92387  1.42868  2.92456  4.15660  4.37759  4.06258  3.96859
3.34195  3.08777  1.81498  4.85719  3.25973    429 l - - S
          2.68618  4.42225  2.77519  2.73123  3.46354  2.40513  3.72494
3.29354  2.67741  2.69355  4.24690  2.90347  2.73739  3.18146  2.89801
2.37887  2.77519  2.98518  4.58477  3.61503
          0.08648  4.61071  2.61867  0.61958  0.77255  0.71655  0.67028
    276   2.12781  4.72137  3.30038  2.89647  4.44525  2.36718  3.59027
3.87754  2.96531  3.49651  4.30903  3.30570  1.44062  3.28658  3.39014
2.40110  3.02509  3.44213  5.72870  4.43208    430 p - - -
          2.68618  4.42225  2.77519  2.73123  3.46354  2.40513  3.72494
3.29354  2.67741  2.69355  4.24690  2.90347  2.73739  3.18146  2.89801
2.37887  2.77519  2.98518  4.58477  3.61503
          0.01598  4.54022  5.26256  0.61958  0.77255  0.81481  0.58470
    277   2.45023  5.08308  3.02269  2.13237  4.38422  3.21981  3.70808
3.45656  2.46233  3.37325  4.14739  3.00637  2.89233  2.82589  1.97431
2.52636  2.76950  3.45224  5.55580  4.18208    431 r - - T
          2.68618  4.42225  2.77519  2.73123  3.46354  2.40513  3.72494
3.29354  2.67741  2.69355  4.24690  2.90347  2.73739  3.18146  2.89801
2.37887  2.77519  2.98518  4.58477  3.61503
          0.01598  4.54022  5.26256  0.61958  0.77255  0.68529  0.70107
    278   2.47947  5.20247  2.38459  2.11191  4.52606  2.85262  3.71252
3.99781  2.46499  3.08981  4.25330  2.60998  3.39563  2.81854  2.95663
2.71466  2.19811  3.58242  5.65123  4.25117    432 e - - T
          2.68618  4.42225  2.77519  2.73123  3.46354  2.40513  3.72494
3.29354  2.67741  2.69355  4.24690  2.90347  2.73739  3.18146  2.89801
2.37887  2.77519  2.98518  4.58477  3.61503
          0.01525  4.58689  5.30924  0.61958  0.77255  0.45905  0.99935
```

279 2.40330 5.23823 2.48107 2.49847 4.57361 3.54954 3.23511
4.04155 2.56451 3.55664 4.32051 2.31931 3.97450 2.90731 3.05709
1.56606 3.05354 3.43463 5.71828 4.32151 433 s - - -
2.68578 4.42168 2.77530 2.73126 3.46364 2.40508 3.72505
3.29352 2.67751 2.69355 4.24700 2.90322 2.73743 3.18157 2.89811
2.37891 2.77530 2.98529 4.58488 3.61494
0.21241 1.67707 5.41548 0.67719 0.70937 0.43270 1.04628
280 2.77800 5.28544 2.72475 2.05724 4.62906 2.83290 3.73857
4.11403 2.15486 3.07337 4.32667 2.70280 3.17526 2.35464 2.60322
2.45222 3.00924 3.67534 5.71873 4.30856 436 e - - H
2.68618 4.42225 2.77519 2.73123 3.46354 2.40513 3.72494
3.29354 2.67741 2.69355 4.24690 2.90347 2.73739 3.18146 2.89801
2.37887 2.77519 2.98518 4.58477 3.61503
0.01309 4.73812 5.46046 0.61958 0.77255 0.48576 0.95510
281 2.22297 4.47110 2.41874 1.74700 4.48622 3.56650 3.75860
3.94416 2.51175 3.46985 3.73442 2.93231 3.95902 2.45079 2.99653
2.75936 2.78426 3.26734 4.36822 4.25571 437 e - - H
2.68618 4.42225 2.77519 2.73123 3.46354 2.40513 3.72494
3.29354 2.67741 2.69355 4.24690 2.90347 2.73739 3.18146 2.89801
2.37887 2.77519 2.98518 4.58477 3.61503
0.01309 4.73812 5.46046 0.61958 0.77255 0.48576 0.95510
282 2.59515 5.23686 2.58969 2.39110 4.55451 3.37046 3.77575
4.01313 2.38642 3.52537 3.44965 3.06898 3.98458 2.88614 1.42286
2.79481 3.04720 3.61352 5.68219 4.30032 438 r - - H
2.68618 4.42225 2.77519 2.73123 3.46354 2.40513 3.72494
3.29354 2.67741 2.69355 4.24690 2.90347 2.73739 3.18146 2.89801
2.37887 2.77519 2.98518 4.58477 3.61503
0.01309 4.73812 5.46046 0.61958 0.77255 0.48576 0.95510
283 2.46898 4.67282 3.43020 2.86777 3.54834 3.36483 3.50378
2.42200 2.60692 2.45746 3.10581 2.90851 4.07851 2.75123 2.66030
2.80635 2.65015 2.66369 5.22975 3.96170 439 i - - H
2.68618 4.42225 2.77519 2.73123 3.46354 2.40513 3.72494
3.29354 2.67741 2.69355 4.24690 2.90347 2.73739 3.18146 2.89801
2.37887 2.77519 2.98518 4.58477 3.61503
0.01309 4.73812 5.46046 0.61958 0.77255 0.48576 0.95510
284 2.67392 4.54391 3.59349 3.02753 3.14977 3.74276 3.98974
2.85335 1.99248 2.57071 3.22184 2.90494 4.12338 3.29732 3.14847
2.54404 2.50417 2.30372 4.05683 3.87759 440 k - - H
2.68618 4.42225 2.77519 2.73123 3.46354 2.40513 3.72494
3.29354 2.67741 2.69355 4.24690 2.90347 2.73739 3.18146 2.89801
2.37887 2.77519 2.98518 4.58477 3.61503
0.01309 4.73812 5.46046 0.61958 0.77255 0.48576 0.95510
285 2.67660 4.24791 4.64406 3.45230 3.34257 4.01921 4.35066
2.08223 3.87118 1.92766 2.55978 4.11985 4.37782 4.03706 3.94582
3.32568 3.08656 1.39066 4.89445 3.28820 441 v - - H
2.68618 4.42225 2.77519 2.73123 3.46354 2.40513 3.72494
3.29354 2.67741 2.69355 4.24690 2.90347 2.73739 3.18146 2.89801
2.37887 2.77519 2.98518 4.58477 3.61503
0.01309 4.73812 5.46046 0.61958 0.77255 0.48576 0.95510
286 2.93385 5.53509 2.74817 0.71703 5.09430 3.60670 4.20061
4.57266 3.12948 4.11158 4.94370 3.19117 4.22642 3.36520 3.64356
2.38479 3.50316 4.11536 6.27555 4.83813 442 e - - H
2.68618 4.42225 2.77519 2.73123 3.46354 2.40513 3.72494
3.29354 2.67741 2.69355 4.24690 2.90347 2.73739 3.18146 2.89801
2.37887 2.77519 2.98518 4.58477 3.61503
0.01309 4.73812 5.46046 0.61958 0.77255 0.48576 0.95510

```
     287   2.52376  5.30077  3.08031  1.91389  4.64913  3.58917  3.75815
4.12089  2.13400  3.59710  3.88813  2.87320  3.98029  2.17107  1.97342
2.78825  3.04991  3.69408  5.72193  4.33241    443 e - - H
           2.68618  4.42225  2.77519  2.73123  3.46354  2.40513  3.72494
3.29354  2.67741  2.69355  4.24690  2.90347  2.73739  3.18146  2.89801
2.37887  2.77519  2.98518  4.58477  3.61503
           0.01309  4.73812  5.46046  0.61958  0.77255  0.48576  0.95510
     288   2.49888  4.71055  3.39085  2.32059  2.75109  3.26581  3.32881
3.09231  2.62585  2.85135  2.59812  3.30323  4.06697  2.80962  2.86909
2.90406  3.01061  2.40339  5.26146  3.98565    444 e - - H
           2.68618  4.42225  2.77519  2.73123  3.46354  2.40513  3.72494
3.29354  2.67741  2.69355  4.24690  2.90347  2.73739  3.18146  2.89801
2.37887  2.77519  2.98518  4.58477  3.61503
           0.14510  4.73812  2.06902  0.61958  0.77255  0.48576  0.95510
     289   2.78091  2.77490  4.66818  4.06358  2.80124  3.94014  4.22803
2.57305  3.86348  1.52289  3.25619  4.07219  4.30020  3.99941  3.89264
3.24998  3.01261  2.00773  3.23190  2.66826    445 l - - T
           2.68618  4.42225  2.77519  2.73123  3.46354  2.40513  3.72494
3.29354  2.67741  2.69355  4.24690  2.90347  2.73739  3.18146  2.89801
2.37887  2.77519  2.98518  4.58477  3.61503
           0.01493  4.60794  5.33029  0.61958  0.77255  0.36269  1.19008
     290   2.84142  4.57763  5.16173  4.56130  1.78357  4.47280  4.80294
1.89455  4.37990  1.27858  2.74493  4.61754  4.75456  4.45468  4.39270
3.79995  3.47107  2.27094  5.17977  4.06513    446 l - - H
           2.68618  4.42225  2.77519  2.73123  3.46354  2.40513  3.72494
3.29354  2.67741  2.69355  4.24690  2.90347  2.73739  3.18146  2.89801
2.37887  2.77519  2.98518  4.58477  3.61503
           0.01309  4.73812  5.46046  0.61958  0.77255  0.48576  0.95510
     291   2.37827  4.99417  3.16975  2.46817  3.73647  1.73581  3.81226
3.44951  2.60717  3.25826  4.06775  3.13550  3.99790  2.51314  2.88072
2.51710  3.01219  3.17119  5.49726  4.15915    447 g - - H
           2.68618  4.42225  2.77519  2.73123  3.46354  2.40513  3.72494
3.29354  2.67741  2.69355  4.24690  2.90347  2.73739  3.18146  2.89801
2.37887  2.77519  2.98518  4.58477  3.61503
           0.01309  4.73812  5.46046  0.61958  0.77255  0.48576  0.95510
     292   2.83067  5.24136  3.11823  2.20489  4.56590  3.24079  3.77461
4.01974  2.18711  3.52702  4.29287  3.08872  2.63990  2.88427  1.64216
2.80938  2.91414  3.62214  4.24017  4.30575    448 r - - H
           2.68618  4.42225  2.77519  2.73123  3.46354  2.40513  3.72494
3.29354  2.67741  2.69355  4.24690  2.90347  2.73739  3.18146  2.89801
2.37887  2.77519  2.98518  4.58477  3.61503
           0.01309  4.73812  5.46046  0.61958  0.77255  0.48576  0.95510
     293   2.60091  5.28373  2.56737  1.56143  4.62360  3.55467  3.74542
4.10561  2.08119  3.58609  4.32644  3.02385  3.95094  2.46211  2.83673
2.55023  3.01701  3.67172  4.39449  4.31092    449 e - - H
           2.68618  4.42225  2.77519  2.73123  3.46354  2.40513  3.72494
3.29354  2.67741  2.69355  4.24690  2.90347  2.73739  3.18146  2.89801
2.37887  2.77519  2.98518  4.58477  3.61503
           0.01309  4.73812  5.46046  0.61958  0.77255  0.48576  0.95510
     294   2.49055  4.71507  5.38387  4.87312  4.00179  4.88631  5.47426
0.76238  4.77313  2.32143  3.80059  5.01859  5.17371  4.99923  4.92018
4.27095  3.70637  1.63985  5.92211  4.72538    450 i - - H
           2.68618  4.42225  2.77519  2.73123  3.46354  2.40513  3.72494
3.29354  2.67741  2.69355  4.24690  2.90347  2.73739  3.18146  2.89801
2.37887  2.77519  2.98518  4.58477  3.61503
           0.01309  4.73812  5.46046  0.61958  0.77255  0.48576  0.95510
```

```
    295   2.63571  3.38239  3.36649  2.52764  3.91645  3.67447  3.89785
3.30001  2.36112  2.45122  3.36876  3.28476  4.05987  2.82608  2.55900
2.61426  2.79413  2.17537  5.28347  4.00242    451 v - - H
          2.68618  4.42225  2.77519  2.73123  3.46354  2.40513  3.72494
3.29354  2.67741  2.69355  4.24690  2.90347  2.73739  3.18146  2.89801
2.37887  2.77519  2.98518  4.58477  3.61503
          0.01309  4.73812  5.46046  0.61958  0.77255  0.48576  0.95510
    296   2.76480  5.51533  2.03630  2.49003  4.85314  3.27667  3.91351
4.34759  2.20739  3.82855  4.59665  1.37158  4.07222  3.03357  3.25619
2.66697  3.24337  3.91007  5.96920  4.53050    452 n - - H
          2.68618  4.42225  2.77519  2.73123  3.46354  2.40513  3.72494
3.29354  2.67741  2.69355  4.24690  2.90347  2.73739  3.18146  2.89801
2.37887  2.77519  2.98518  4.58477  3.61503
          0.01309  4.73812  5.46046  0.61958  0.77255  0.48576  0.95510
    297   2.36468  4.29756  4.82635  4.22467  3.36458  4.12315  4.46587
1.68644  4.03569  1.74785  3.04715  4.25915  4.47040  4.17787  4.07548
3.43788  3.16323  1.56518  3.96072  3.78983    453 v - - H
          2.68618  4.42225  2.77519  2.73123  3.46354  2.40513  3.72494
3.29354  2.67741  2.69355  4.24690  2.90347  2.73739  3.18146  2.89801
2.37887  2.77519  2.98518  4.58477  3.61503
          0.01309  4.73812  5.46046  0.61958  0.77255  0.48576  0.95510
    298   3.63568  4.83458  5.71028  5.22518  4.03613  5.22903  5.88140
1.27818  5.15462  1.80418  3.77784  5.38082  5.42356  5.34461  5.28189
4.65125  3.88790  0.91265  6.14842  4.98018    454 v - - H
          2.68618  4.42225  2.77519  2.73123  3.46354  2.40513  3.72494
3.29354  2.67741  2.69355  4.24690  2.90347  2.73739  3.18146  2.89801
2.37887  2.77519  2.98518  4.58477  3.61503
          0.01309  4.73812  5.46046  0.61958  0.77255  0.48576  0.95510
    299   1.43642  4.09009  3.47811  2.73774  3.86715  3.11857  3.96759
3.24235  2.89281  2.77182  3.34446  3.36847  4.08680  3.21716  3.29644
2.34623  2.58720  2.98238  5.26007  3.99646    455 a - - C
          2.68618  4.42225  2.77519  2.73123  3.46354  2.40513  3.72494
3.29354  2.67741  2.69355  4.24690  2.90347  2.73739  3.18146  2.89801
2.37887  2.77519  2.98518  4.58477  3.61503
          0.01309  4.73812  5.46046  0.61958  0.77255  0.48576  0.95510
    300   2.42329  1.95245  3.11981  2.76885  3.56329  3.43038  3.58460
3.35123  2.59443  3.01058  3.56592  2.73033  3.67914  3.08777  2.97664
2.87832  2.73929  2.87888  5.31555  4.02598    456 c - - T
          2.68618  4.42225  2.77519  2.73123  3.46354  2.40513  3.72494
3.29354  2.67741  2.69355  4.24690  2.90347  2.73739  3.18146  2.89801
2.37887  2.77519  2.98518  4.58477  3.61503
          0.01309  4.73812  5.46046  0.61958  0.77255  0.48576  0.95510
    301   2.49490  5.19130  2.95205  1.31299  4.49741  3.10005  3.77723
3.55480  2.53332  3.48205  4.25080  3.06273  3.97373  2.89173  2.63391
2.77957  3.02835  3.27285  5.65582  4.27307    457 e - - T
          2.68618  4.42225  2.77519  2.73123  3.46354  2.40513  3.72494
3.29354  2.67741  2.69355  4.24690  2.90347  2.73739  3.18146  2.89801
2.37887  2.77519  2.98518  4.58477  3.61503
          0.01309  4.73812  5.46046  0.61958  0.77255  0.48576  0.95510
    302   2.65545  5.05722  3.10825  2.39624  4.33681  1.42823  3.85145
3.76223  2.53830  3.35117  4.15838  3.14021  3.44133  2.99701  3.11600
2.69941  2.86444  3.42081  5.58005  3.49405    458 g - - C
          2.68618  4.42225  2.77519  2.73123  3.46354  2.40513  3.72494
3.29354  2.67741  2.69355  4.24690  2.90347  2.73739  3.18146  2.89801
2.37887  2.77519  2.98518  4.58477  3.61503
          0.01309  4.73812  5.46046  0.61958  0.77255  0.48576  0.95510
```

```
    303   2.14221  4.98232  2.66034  2.47705  3.08268  3.38030  3.81083
3.25170  2.61003  3.24128  4.05264  3.13685  3.16249  2.95984  2.55088
2.36698  2.61452  3.18687  4.46973  4.14781    459 a - - C
          2.68618  4.42225  2.77519  2.73123  3.46354  2.40513  3.72494
3.29354  2.67741  2.69355  4.24690  2.90347  2.73739  3.18146  2.89801
2.37887  2.77519  2.98518  4.58477  3.61503
          0.03238  4.73812  3.76767  0.61958  0.77255  0.48576  0.95510
    304   2.60706  5.25465  2.38244  1.62446  4.58896  3.31966  3.40363
4.06744  2.39649  3.55528  3.69871  2.84941  3.60048  2.83529  2.65842
2.66525  3.00092  3.63952  4.05345  4.28928    460 e - - C
          2.68618  4.42226  2.77520  2.73124  3.46354  2.40513  3.72495
3.29337  2.67741  2.69355  4.24690  2.90347  2.73740  3.18147  2.89801
2.37887  2.77520  2.98519  4.58477  3.61504
          0.23197  3.51030  1.73082  0.47242  0.97682  0.46148  0.99520
    305   3.26434  5.34532  3.69688  3.08551  4.73490  3.88688  3.37414
4.21917  2.22559  3.67635  4.54650  3.47796  4.29595  3.06832  0.81019
2.78283  3.46584  3.88586  5.68395  4.46384    462 r - - T
          2.68618  4.42225  2.77519  2.73123  3.46354  2.40513  3.72494
3.29354  2.67741  2.69355  4.24690  2.90347  2.73739  3.18146  2.89801
2.37887  2.77519  2.98518  4.58477  3.61503
          0.01579  4.55233  5.27468  0.61958  0.77255  0.71396  0.67276
    306   2.72365  4.80341  2.98667  2.57433  3.19473  3.58389  3.79612
2.74033  2.33246  3.04735  3.88371  3.14970  3.97167  2.79072  2.28018
2.71102  2.46761  2.53515  5.32872  4.02131    463 r - - S
          2.68618  4.42225  2.77519  2.73123  3.46354  2.40513  3.72494
3.29354  2.67741  2.69355  4.24690  2.90347  2.73739  3.18146  2.89801
2.37887  2.77519  2.98518  4.58477  3.61503
          0.01532  4.58221  5.30456  0.61958  0.77255  0.75829  0.63199
    307   2.47649  5.40747  2.75927  1.12734  4.73490  3.13604  3.85243
4.20909  2.51306  3.70865  4.49242  3.01312  4.02081  2.59485  3.13255
2.88938  3.18862  3.79625  5.86060  4.44687    464 e - - S
          2.68618  4.42225  2.77519  2.73123  3.46354  2.40513  3.72494
3.29354  2.67741  2.69355  4.24690  2.90347  2.73739  3.18146  2.89801
2.37887  2.77519  2.98518  4.58477  3.61503
          0.01532  4.58221  5.30456  0.61958  0.77255  0.60081  0.79488
    308   3.05705  5.29290  3.10067  2.48250  4.62921  3.74805  3.87207
3.51038  2.39765  3.55074  4.38174  3.26101  4.15475  3.00842  0.98366
3.05392  3.26954  3.68239  5.69509  4.40969    465 r - - -
          2.68618  4.42225  2.77519  2.73123  3.46354  2.40513  3.72494
3.29354  2.67741  2.69355  4.24690  2.90347  2.73739  3.18146  2.89801
2.37887  2.77519  2.98518  4.58477  3.61503
          0.01449  4.63718  5.35953  0.61958  0.77255  0.45302  1.00980
    309   2.77020  3.81332  3.36911  2.67557  3.89173  3.66875  1.83614
3.27399  2.78387  2.94496  2.94422  3.00490  3.27437  3.11735  2.91835
2.88987  2.80054  2.66921  5.26337  3.68055    466 h - - -
          2.68618  4.42225  2.77519  2.73123  3.46354  2.40513  3.72494
3.29354  2.67741  2.69355  4.24690  2.90347  2.73739  3.18146  2.89801
2.37887  2.77519  2.98518  4.58477  3.61503
          0.01344  4.71225  5.43460  0.61958  0.77255  0.45345  1.00904
    310   2.86119  5.28594  3.06679  1.20807  4.61889  3.28508  3.79894
4.07636  2.29878  3.57850  4.34480  3.08921  4.01293  2.90981  2.53518
2.83416  2.88087  3.32333  5.72440  4.34655    467 e - - -
          2.68618  4.42225  2.77519  2.73123  3.46354  2.40513  3.72494
3.29354  2.67741  2.69355  4.24690  2.90347  2.73739  3.18146  2.89801
2.37887  2.77519  2.98518  4.58477  3.61503
          0.06049  4.73812  2.99693  0.61958  0.77255  0.48576  0.95510
```

311 2.66754 5.13379 3.07067 2.42505 4.42781 2.67923 3.75174
3.87747 2.18393 3.14801 4.19040 3.05105 3.25031 2.86876 2.42930
2.50121 2.11673 3.15283 5.60013 4.22482 468 t - - .
2.68618 4.42225 2.77519 2.73123 3.46354 2.40513 3.72494
3.29354 2.67741 2.69355 4.24690 2.90347 2.73739 3.18146 2.89801
2.37887 2.77519 2.98518 4.58477 3.61503
0.01372 4.69135 5.41370 0.61958 0.77255 0.48986 0.94858
312 2.47943 4.37098 3.89295 3.05605 2.17684 3.42154 4.08703
2.84172 2.62796 1.79053 3.22127 3.65150 4.18692 3.51788 3.53255
2.77430 3.00725 2.63352 4.96825 3.09454 469 l - - H
2.68618 4.42225 2.77519 2.73123 3.46354 2.40513 3.72494
3.29354 2.67741 2.69355 4.24690 2.90347 2.73739 3.18146 2.89801
2.37887 2.77519 2.98518 4.58477 3.61503
0.01335 4.71908 5.44143 0.61958 0.77255 0.52586 0.89415
313 2.45937 5.23400 3.03810 1.87386 4.56005 2.42675 3.74237
4.03154 2.27668 3.53079 3.46572 2.93833 3.39799 2.84583 2.40173
2.58269 3.00476 3.61462 5.67841 4.28092 470 e - - C
2.68618 4.42225 2.77519 2.73123 3.46354 2.40513 3.72494
3.29354 2.67741 2.69355 4.24690 2.90347 2.73739 3.18146 2.89801
2.37887 2.77519 2.98518 4.58477 3.61503
0.01335 4.71908 5.44143 0.61958 0.77255 0.52586 0.89415
314 2.77890 5.23352 3.05004 2.49257 4.55959 3.28584 3.40549
3.38431 1.75892 3.52856 4.28033 3.03541 3.39795 2.18989 2.67694
2.31388 2.70510 3.61416 5.67596 4.28209 471 k - - H
2.68618 4.42225 2.77519 2.73123 3.46354 2.40513 3.72494
3.29354 2.67741 2.69355 4.24690 2.90347 2.73739 3.18146 2.89801
2.37887 2.77519 2.98518 4.58477 3.61503
0.01335 4.71908 5.44143 0.61958 0.77255 0.46148 0.99520
315 5.05712 6.05440 5.55703 5.50682 4.02577 4.85534 5.31184
5.25484 5.30951 4.46680 5.82226 5.54822 5.44445 5.63570 5.25891
5.26087 5.41369 5.15099 0.13078 3.99800 472 W - - H
2.68618 4.42225 2.77519 2.73123 3.46354 2.40513 3.72494
3.29354 2.67741 2.69355 4.24690 2.90347 2.73739 3.18146 2.89801
2.37887 2.77519 2.98518 4.58477 3.61503
0.01309 4.73812 5.46046 0.61958 0.77255 0.48576 0.95510
316 2.79583 5.22669 3.07661 2.26447 4.54656 3.57738 3.48713
3.55291 2.34921 3.51695 4.27570 3.05742 3.57178 2.64618 1.66087
2.24127 3.02518 3.60410 5.67069 3.68069 473 r - - H
2.68618 4.42225 2.77519 2.73123 3.46354 2.40513 3.72494
3.29354 2.67741 2.69355 4.24690 2.90347 2.73739 3.18146 2.89801
2.37887 2.77519 2.98518 4.58477 3.61503
0.01309 4.73812 5.46046 0.61958 0.77255 0.48576 0.95510
317 2.57507 5.26090 2.75726 1.92156 4.59514 3.55348 3.74203
4.07347 2.42267 3.56147 3.66744 2.65611 3.94703 2.32905 2.50555
2.37694 3.00758 2.83064 5.70001 4.29575 474 e - - H
2.68618 4.42225 2.77519 2.73123 3.46354 2.40513 3.72494
3.29354 2.67741 2.69355 4.24690 2.90347 2.73739 3.18146 2.89801
2.37887 2.77519 2.98518 4.58477 3.61503
0.01309 4.73812 5.46046 0.61958 0.77255 0.48576 0.95510
318 2.52468 4.72396 3.49106 2.77968 3.89846 3.73418 3.93448
3.27189 2.60377 2.56355 2.69228 3.36764 4.11381 3.17108 1.49357
2.96898 3.05843 3.01992 4.16992 4.00730 475 r - - H
2.68618 4.42225 2.77519 2.73123 3.46354 2.40513 3.72494
3.29354 2.67741 2.69355 4.24690 2.90347 2.73739 3.18146 2.89801
2.37887 2.77519 2.98518 4.58477 3.61503
0.01309 4.73812 5.46046 0.61958 0.77255 0.48576 0.95510

319 3.11411 4.73684 5.32255 4.72259 2.06601 4.66421 4.97414
1.92428 4.54869 1.38700 1.55302 4.80558 4.89908 4.57891 4.54885
3.99851 3.64217 2.64785 5.27641 4.19415 476 l - - H
2.68618 4.42225 2.77519 2.73123 3.46354 2.40513 3.72494
3.29354 2.67741 2.69355 4.24690 2.90347 2.73739 3.18146 2.89801
2.37887 2.77519 2.98518 4.58477 3.61503
0.01309 4.73812 5.46046 0.61958 0.77255 0.48576 0.95510
320 2.70199 4.21543 2.55716 2.14679 4.53728 2.60564 3.56383
4.00460 2.32072 3.00749 3.60493 3.03857 3.95269 2.77571 2.39616
2.43346 2.88713 3.30959 5.66842 4.27442 477 e - - H
2.68618 4.42225 2.77519 2.73123 3.46354 2.40513 3.72494
3.29354 2.67741 2.69355 4.24690 2.90347 2.73739 3.18146 2.89801
2.37887 2.77519 2.98518 4.58477 3.61503
0.01309 4.73812 5.46046 0.61958 0.77255 0.48576 0.95510
321 2.63640 5.22769 2.72846 2.26320 4.54873 3.55918 3.74898
4.01793 2.49329 2.74407 3.06323 2.52582 3.95226 2.31945 2.31874
2.51750 2.84098 3.60537 5.67489 4.27914 478 e - - C
2.68619 4.42226 2.77520 2.73124 3.46354 2.40505 3.72495
3.29355 2.67741 2.69355 4.24690 2.90347 2.73740 3.18147 2.89801
2.37887 2.77520 2.98519 4.58478 3.61504
0.03840 3.39867 5.46046 0.45123 1.01293 0.48576 0.95510
322 1.42713 3.14641 3.96932 3.39557 3.00971 3.27918 4.12569
2.83554 3.31639 2.43656 3.21781 3.34526 4.20560 3.57927 3.58598
2.60363 3.01778 2.52349 4.97100 3.76367 480 a - - T
2.68618 4.42225 2.77519 2.73123 3.46354 2.40513 3.72494
3.29354 2.67741 2.69355 4.24690 2.90347 2.73739 3.18146 2.89801
2.37887 2.77519 2.98518 4.58477 3.61503
0.01309 4.73812 5.46046 0.61958 0.77255 0.48576 0.95510
323 3.51928 5.48368 3.42464 2.81223 5.47867 0.35391 4.92727
5.07231 3.96220 4.64777 5.56888 3.87563 4.60995 4.20099 4.33954
3.59704 3.97196 4.53290 6.55110 5.42356 481 G - - T
2.68618 4.42225 2.77519 2.73123 3.46354 2.40513 3.72494
3.29354 2.67741 2.69355 4.24690 2.90347 2.73739 3.18146 2.89801
2.37887 2.77519 2.98518 4.58477 3.61503
0.01309 4.73812 5.46046 0.61958 0.77255 0.48576 0.95510
324 3.99689 5.23570 5.54524 5.09476 0.63538 5.10771 4.69590
2.88984 4.90818 1.82283 2.99289 5.04152 5.28282 4.84874 4.89408
4.48951 4.21364 3.06689 4.80602 3.24328 482 F - - E
2.68618 4.42225 2.77519 2.73123 3.46354 2.40513 3.72494
3.29354 2.67741 2.69355 4.24690 2.90347 2.73739 3.18146 2.89801
2.37887 2.77519 2.98518 4.58477 3.61503
0.01309 4.73812 5.46046 0.61958 0.77255 0.48576 0.95510
325 2.70005 5.22717 2.94722 2.12463 3.83001 2.50978 3.74893
4.01735 2.11632 3.52210 4.27442 2.91701 3.95211 2.85368 2.34581
2.46714 2.68692 2.99421 5.67458 4.27884 483 k - - E
2.68618 4.42225 2.77519 2.73123 3.46354 2.40513 3.72494
3.29354 2.67741 2.69355 4.24690 2.90347 2.73739 3.18146 2.89801
2.37887 2.77519 2.98518 4.58477 3.61503
0.01309 4.73812 5.46046 0.61958 0.77255 0.48576 0.95510
326 2.38015 5.02461 3.15742 2.60772 4.31445 3.13154 3.81880
3.74229 2.60600 3.02003 4.12575 2.21167 2.09580 2.82184 3.07485
2.54261 2.77930 3.09167 5.55001 4.20280 484 p - - E
2.68618 4.42225 2.77519 2.73123 3.46354 2.40513 3.72494
3.29354 2.67741 2.69355 4.24690 2.90347 2.73739 3.18146 2.89801
2.37887 2.77519 2.98518 4.58477 3.61503
0.01309 4.73812 5.46046 0.61958 0.77255 0.48576 0.95510

```
    327   2.33022  4.25852  4.32373  3.28881  3.36350  3.91601  4.22483
2.44365  3.36505  2.21777  3.01926  3.91967  4.28354  3.81671  3.76596
3.20612  3.03243  1.30175  4.88011  3.15521    485 v - - E
          2.68618  4.42225  2.77519  2.73123  3.46354  2.40513  3.72494
3.29354  2.67741  2.69355  4.24690  2.90347  2.73739  3.18146  2.89801
2.37887  2.77519  2.98518  4.58477  3.61503
          0.01309  4.73812  5.46046  0.61958  0.77255  0.48576  0.95510
    328   2.52513  5.23871  3.04323  2.38799  4.56328  2.68378  3.19794
4.03428  2.34427  3.53501  4.28576  2.93552  2.18905  2.70255  2.79570
2.46901  2.56188  3.61849  5.68400  4.28668    486 p - - -
          2.68618  4.42225  2.77519  2.73123  3.46354  2.40513  3.72494
3.29354  2.67741  2.69355  4.24690  2.90347  2.73739  3.18146  2.89801
2.37887  2.77519  2.98518  4.58477  3.61503
          0.01309  4.73812  5.46046  0.61958  0.77255  0.48576  0.95510
    329   2.89308  4.27339  4.76082  4.15421  2.78450  4.05993  3.77841
2.07097  3.96213  1.19615  3.08566  4.18905  4.40891  4.09642  3.99943
3.37005  3.12366  2.10603  4.86629  3.18696    487 l - - -
          2.68618  4.42225  2.77519  2.73123  3.46354  2.40513  3.72494
3.29354  2.67741  2.69355  4.24690  2.90347  2.73739  3.18146  2.89801
2.37887  2.77519  2.98518  4.58477  3.61503
          0.01309  4.73812  5.46046  0.61958  0.77255  0.48576  0.95510
    330   2.68138  5.28167  2.81683  2.37285  4.61329  2.34533  3.83272
4.07853  2.45634  3.59582  4.36529  3.07002  4.01171  2.95004  2.93539
1.39811  3.10270  3.67336  5.76020  4.36449    488 s - - T
          2.68618  4.42225  2.77519  2.73123  3.46354  2.40513  3.72494
3.29354  2.67741  2.69355  4.24690  2.90347  2.73739  3.18146  2.89801
2.37887  2.77519  2.98518  4.58477  3.61503
          0.01309  4.73812  5.46046  0.61958  0.77255  0.48576  0.95510
    331   2.68396  5.26334  2.80275  1.99984  3.96239  3.10420  3.74258
4.07674  2.35045  3.56414  4.30689  2.75340  3.27318  2.40110  2.97338
2.09567  3.00853  3.64835  5.70226  3.12803    489 e - - C
          2.68618  4.42225  2.77519  2.73123  3.46354  2.40513  3.72494
3.29354  2.67741  2.69355  4.24690  2.90347  2.73739  3.18146  2.89801
2.37887  2.77519  2.98518  4.58477  3.61503
          0.01309  4.73812  5.46046  0.61958  0.77255  0.48576  0.95510
    332   2.77596  5.16638  2.62149  2.37515  2.80197  3.56949  3.76224
3.68872  2.31397  3.31165  4.21970  2.44478  3.96177  2.68539  2.68912
2.59012  2.62452  3.53156  5.62815  2.86581    490 k - - H
          2.68618  4.42225  2.77519  2.73123  3.46354  2.40513  3.72494
3.29354  2.67741  2.69355  4.24690  2.90347  2.73739  3.18146  2.89801
2.37887  2.77519  2.98518  4.58477  3.61503
          0.01309  4.73812  5.46046  0.61958  0.77255  0.48576  0.95510
    333   1.42312  4.58486  3.55955  2.65503  3.75513  3.72759  3.99897
2.44596  2.97269  2.81804  3.70293  3.15927  3.73435  3.28794  3.35271
2.97365  2.85638  2.41242  5.17899  3.93067    491 a - - H
          2.68618  4.42225  2.77519  2.73123  3.46354  2.40513  3.72494
3.29354  2.67741  2.69355  4.24690  2.90347  2.73739  3.18146  2.89801
2.37887  2.77519  2.98518  4.58477  3.61503
          0.01309  4.73812  5.46046  0.61958  0.77255  0.48576  0.95510
    334   2.15009  4.59254  3.00139  2.58091  3.36359  3.72343  3.96384
3.11352  2.92851  2.38685  2.62260  3.12865  4.10545  3.24497  3.07557
2.85437  2.88394  2.25761  5.16164  3.25602    492 a - - H
          2.68618  4.42225  2.77519  2.73123  3.46354  2.40513  3.72494
3.29354  2.67741  2.69355  4.24690  2.90347  2.73739  3.18146  2.89801
2.37887  2.77519  2.98518  4.58477  3.61503
          0.01309  4.73812  5.46046  0.61958  0.77255  0.48576  0.95510
```

335 2.43337 4.14822 2.70543 2.44138 3.41433 3.59896 3.80013
3.69312 2.10513 2.54964 3.65762 2.98715 3.98934 2.94121 2.92991
2.25968 2.58390 3.35759 5.51333 4.16837 493 k - - H
2.68618 4.42225 2.77519 2.73123 3.46354 2.40513 3.72494
3.29354 2.67741 2.69355 4.24690 2.90347 2.73739 3.18146 2.89801
2.37887 2.77519 2.98518 4.58477 3.61503
0.01309 4.73812 5.46046 0.61958 0.77255 0.48576 0.95510
336 2.92761 5.42597 2.37143 2.40296 4.75462 3.17080 3.86305
4.23960 2.65947 3.72932 4.49231 2.89838 4.03696 1.29229 3.16783
2.63751 2.66684 3.81213 5.87447 4.45093 494 q - - H
2.68618 4.42225 2.77519 2.73123 3.46354 2.40513 3.72494
3.29354 2.67741 2.69355 4.24690 2.90347 2.73739 3.18146 2.89801
2.37887 2.77519 2.98518 4.58477 3.61503
0.01309 4.73812 5.46046 0.61958 0.77255 0.48576 0.95510
337 1.25031 4.39796 3.98217 3.42028 3.58302 3.18853 4.18336
2.48923 3.34504 2.65456 3.13243 3.71972 4.21518 3.61451 3.62469
2.70439 3.04442 2.31311 5.06743 3.85837 495 a - - H
2.68618 4.42225 2.77519 2.73123 3.46354 2.40513 3.72494
3.29354 2.67741 2.69355 4.24690 2.90347 2.73739 3.18146 2.89801
2.37887 2.77519 2.98518 4.58477 3.61503
0.01309 4.73812 5.46046 0.61958 0.77255 0.48576 0.95510
338 2.79789 5.28964 2.86369 2.16438 4.63285 3.34817 3.74927
4.11151 1.65887 3.35610 4.33271 3.03599 3.96062 2.74590 2.14559
2.34825 2.93089 3.67970 5.71959 4.31816 496 k - - H
2.68618 4.42225 2.77519 2.73123 3.46354 2.40513 3.72494
3.29354 2.67741 2.69355 4.24690 2.90347 2.73739 3.18146 2.89801
2.37887 2.77519 2.98518 4.58477 3.61503
0.01309 4.73812 5.46046 0.61958 0.77255 0.48576 0.95510
339 2.37333 3.19315 3.70594 2.87025 3.60607 3.56079 3.65928
2.81686 3.08689 2.05612 2.71657 3.31912 4.15124 3.38302 2.70983
2.52280 2.76467 2.74454 5.05932 3.28530 497 l - - H
2.68618 4.42225 2.77519 2.73123 3.46354 2.40513 3.72494
3.29354 2.67741 2.69355 4.24690 2.90347 2.73739 3.18146 2.89801
2.37887 2.77519 2.98518 4.58477 3.61503
0.01309 4.73812 5.46046 0.61958 0.77255 0.48576 0.95510
340 3.04544 4.49437 4.53045 3.92812 3.41448 4.16278 4.44193
2.32901 3.34326 0.88154 2.99662 4.13968 4.49537 3.96634 3.25261
3.46328 3.27201 2.60740 5.07715 3.92067 498 l - - H
2.68618 4.42225 2.77519 2.73123 3.46354 2.40513 3.72494
3.29354 2.67741 2.69355 4.24690 2.90347 2.73739 3.18146 2.89801
2.37887 2.77519 2.98518 4.58477 3.61503
0.01309 4.73812 5.46046 0.61958 0.77255 0.48576 0.95510
341 2.79458 4.45958 4.68284 4.11879 3.51972 3.40535 4.59293
2.38928 3.97201 0.92721 3.45225 4.26499 4.55214 4.17364 4.10809
3.50197 3.28538 1.94378 5.17731 4.00708 499 l - - H
2.68618 4.42225 2.77519 2.73123 3.46354 2.40513 3.72494
3.29354 2.67741 2.69355 4.24690 2.90347 2.73739 3.18146 2.89801
2.37887 2.77519 2.98518 4.58477 3.61503
0.01309 4.73812 5.46046 0.61958 0.77255 0.48576 0.95510
342 2.42155 5.26400 3.04212 2.22786 4.59930 2.80888 3.48126
4.07590 2.34353 3.56349 4.30791 2.80676 3.95233 2.20291 2.02267
2.61869 3.01369 3.64953 4.53704 4.29973 500 r - - T
2.68618 4.42225 2.77519 2.73123 3.46354 2.40513 3.72494
3.29354 2.67741 2.69355 4.24690 2.90347 2.73739 3.18146 2.89801
2.37887 2.77519 2.98518 4.58477 3.61503
0.01309 4.73812 5.46046 0.61958 0.77255 0.48576 0.95510

343 2.77994 5.14959 2.98053 2.34608 4.44151 2.83608 3.76846
3.89057 2.23564 2.32419 3.00013 2.83522 3.96804 2.78086 2.24691
2.69621 2.87470 3.51140 5.61476 4.24011 501 k - - T
2.68618 4.42225 2.77519 2.73123 3.46354 2.40513 3.72494
3.29354 2.67741 2.69355 4.24690 2.90347 2.73739 3.18146 2.89801
2.37887 2.77519 2.98518 4.58477 3.61503
0.01309 4.73812 5.46046 0.61958 0.77255 0.48576 0.95510
344 2.62088 3.03039 4.00722 3.42942 2.31117 3.84206 2.91290
2.79861 3.34378 2.44190 3.44764 3.21098 4.21520 3.36960 3.19300
2.98879 2.84445 2.31020 4.93837 2.37529 502 v - - S
2.68618 4.42225 2.77519 2.73123 3.46354 2.40513 3.72494
3.29354 2.67741 2.69355 4.24690 2.90347 2.73739 3.18146 2.89801
2.37887 2.77519 2.98518 4.58477 3.61503
0.01309 4.73812 5.46046 0.61958 0.77255 0.48576 0.95510
345 2.41662 5.23836 2.82949 2.48935 4.56193 2.49879 3.33385
4.03328 2.35429 3.53446 4.28511 2.66739 2.85699 2.57494 2.98401
2.46670 3.01091 3.61748 5.68406 2.78756 503 k - - S
2.68628 4.42189 2.77529 2.73133 3.46364 2.40496 3.72434
3.29364 2.67750 2.69365 4.24699 2.90356 2.73706 3.18156 2.89810
2.37887 2.77529 2.98510 4.58487 3.61488
0.59300 1.14605 2.04452 0.11073 2.25551 0.48576 0.95510
346 2.65674 4.85292 2.71021 2.55270 2.99263 2.65584 3.78906
3.47502 2.43690 3.10183 3.35535 2.88463 3.96815 2.95779 2.93469
2.25610 2.96126 2.65033 4.42399 4.05329 505 s - - -
2.68618 4.42225 2.77519 2.73123 3.46354 2.40513 3.72494
3.29354 2.67741 2.69355 4.24690 2.90347 2.73739 3.18146 2.89801
2.37887 2.77519 2.98518 4.58477 3.61503
0.01498 4.60437 5.32671 0.61958 0.77255 0.43995 1.03302
347 2.77636 5.27888 1.95610 2.02039 4.61997 3.37619 3.73513
4.10337 2.21234 3.58189 4.32134 2.61406 3.44705 2.83366 2.37584
2.73912 3.00793 3.66754 4.28534 4.30372 506 d - - T
2.68618 4.42225 2.77519 2.73123 3.46354 2.40513 3.72494
3.29354 2.67741 2.69355 4.24690 2.90347 2.73739 3.18146 2.89801
2.37887 2.77519 2.98518 4.58477 3.61503
0.16701 4.70635 1.93262 0.61958 0.77255 0.55142 0.85833
348 2.98756 5.01752 3.09325 2.59426 4.60380 0.78056 4.27670
4.02708 3.15127 3.17513 4.59108 3.38850 4.19247 3.50369 3.52755
3.07027 3.35953 3.65098 5.90016 4.59403 507 g - - T
2.68622 4.42229 2.77523 2.73127 3.46331 2.40507 3.72498
3.29358 2.67744 2.69359 4.24693 2.90350 2.73743 3.18150 2.89804
2.37890 2.77523 2.98500 4.58372 3.61507
0.09319 2.47842 5.27743 0.66113 0.72622 0.33171 1.26477
349 3.20139 4.66498 4.44246 3.91790 1.57824 4.20801 3.58017
3.15258 3.80556 2.80893 3.23634 3.33771 4.56467 3.98971 3.99147
2.98657 3.43048 2.96649 4.43821 1.28904 510 y - - E
2.68618 4.42225 2.77519 2.73123 3.46354 2.40513 3.72494
3.29354 2.67741 2.69355 4.24690 2.90347 2.73739 3.18146 2.89801
2.37887 2.77519 2.98518 4.58477 3.61503
0.01309 4.73812 5.46046 0.61958 0.77255 0.48576 0.95510
350 2.70114 5.19292 3.07121 2.51390 4.50082 3.56974 2.74939
3.95955 2.20180 3.48109 3.61721 2.42583 3.96184 2.75497 2.25364
2.56608 2.39077 3.04912 5.64752 4.26304 511 k - - E
2.68618 4.42225 2.77519 2.73123 3.46354 2.40513 3.72494
3.29354 2.67741 2.69355 4.24690 2.90347 2.73739 3.18146 2.89801
2.37887 2.77519 2.98518 4.58477 3.61503
0.01309 4.73812 5.46046 0.61958 0.77255 0.48576 0.95510

```
    351   2.91178  4.29066  4.75381  4.15301  3.35664  4.09228  4.42645
1.81279  3.42656  1.66866  3.34541  4.20941  4.44273  4.12322  4.02260
3.40381  3.14448  1.41668  3.82113  3.76531    512 v - - E
          2.68618  4.42225  2.77519  2.73123  3.46354  2.40513  3.72494
3.29354  2.67741  2.69355  4.24690  2.90347  2.73739  3.18146  2.89801
2.37887  2.77519  2.98518  4.58477  3.61503
          0.01309  4.73812  5.46046  0.61958  0.77255  0.48576  0.95510
    352   2.52644  5.29630  2.66829  1.62246  4.63272  3.33605  3.75876
4.11414  2.02309  3.59747  4.34102  3.02703  3.96068  2.34627  2.99478
2.76384  3.03341  3.00221  5.73279  4.32412    513 e - - E
          2.68627  4.42234  2.77515  2.73132  3.46363  2.40522  3.72504
3.29336  2.67732  2.69364  4.24629  2.90356  2.73749  3.18125  2.89754
2.37887  2.77529  2.98527  4.58486  3.61512
          0.11628  2.24884  5.46046  0.96202  0.48146  0.48576  0.95510
    353   2.64512  4.02147  2.77756  1.48267  3.61973  3.56302  3.75517
3.98228  2.22437  3.49778  4.25632  2.92899  3.95661  2.74581  2.99128
2.48217  3.00914  3.36028  5.65950  4.26911    517 e - - E
          2.68618  4.42225  2.77519  2.73123  3.46354  2.40513  3.72494
3.29354  2.67741  2.69355  4.24690  2.90347  2.73739  3.18146  2.89801
2.37887  2.77519  2.98518  4.58477  3.61503
          0.01309  4.73812  5.46046  0.61958  0.77255  0.48576  0.95510
    354   2.68322  5.28165  2.16155  2.02321  4.62276  3.55154  3.42613
4.10610  2.26262  2.94653  4.32362  2.84120  3.45102  2.83895  2.47166
2.32909  3.01105  3.67022  5.71632  4.30744    518 e - - C
          2.68628  4.42235  2.77530  2.73121  3.46364  2.40434  3.72505
3.29364  2.67751  2.69365  4.24700  2.90342  2.73750  3.18157  2.89811
2.37889  2.77530  2.98529  4.58487  3.61513
          0.09338  2.46631  5.46046  1.21605  0.35154  0.48576  0.95510
    355   2.78344  5.29026  2.27376  2.28795  4.63269  3.02135  3.45530
4.11754  2.39664  3.59430  3.94874  2.18646  3.94805  2.45357  2.80485
2.15774  3.01505  3.67957  5.72397  4.31335    525 s - - T
          2.68618  4.42225  2.77519  2.73123  3.46354  2.40513  3.72494
3.29354  2.67741  2.69355  4.24690  2.90347  2.73739  3.18146  2.89801
2.37887  2.77519  2.98518  4.58477  3.61503
          0.01309  4.73812  5.46046  0.61958  0.77255  0.48576  0.95510
    356   2.49850  5.32201  2.73117  2.36083  4.64652  1.67719  3.54649
4.12274  2.57499  3.62108  4.37840  2.31066  3.15702  2.91533  2.91254
2.81287  3.08685  3.70368  5.77057  4.36211    526 g - - T
          2.68618  4.42225  2.77519  2.73123  3.46354  2.40513  3.72494
3.29354  2.67741  2.69355  4.24690  2.90347  2.73739  3.18146  2.89801
2.37887  2.77519  2.98518  4.58477  3.61503
          0.01309  4.73812  5.46046  0.61958  0.77255  0.48576  0.95510
    357   2.32352  2.60411  3.39589  2.34645  3.92993  2.69192  3.92562
3.31347  2.81787  2.98380  3.84199  3.30832  4.06566  3.14915  3.23822
2.10477  2.60022  3.04420  3.64755  4.02229    527 s - - C
          2.68618  4.42225  2.77519  2.73123  3.46354  2.40513  3.72494
3.29354  2.67741  2.69355  4.24690  2.90347  2.73739  3.18146  2.89801
2.37887  2.77519  2.98518  4.58477  3.61503
          0.01309  4.73812  5.46046  0.61958  0.77255  0.48576  0.95510
    358   2.23026  4.26734  4.51484  3.92016  2.69278  3.07643  4.30387
2.63665  3.76628  1.28214  2.77680  4.04408  4.34286  3.94686  3.87687
3.28116  3.07684  2.30696  4.88464  3.70608    528 l - - C
          2.68618  4.42225  2.77519  2.73123  3.46354  2.40513  3.72494
3.29354  2.67741  2.69355  4.24690  2.90347  2.73739  3.18146  2.89801
2.37887  2.77519  2.98518  4.58477  3.61503
          0.01309  4.73812  5.46046  0.61958  0.77255  0.48576  0.95510
```

```
     359   2.58784  3.51044  4.61332  4.00695  2.62911  3.93914  3.84342
2.33415  3.39734  1.92658  3.01707  4.05587  4.30420  3.97749  3.54625
3.24436  2.63937  1.67459  4.80148  3.11332    529 v - - E
         2.68622  4.42229  2.77524  2.73127  3.46323  2.40506  3.72499
3.29358  2.67745  2.69359  4.24694  2.90351  2.73744  3.18151  2.89805
2.37884  2.77498  2.98523  4.58481  3.61507
         0.16322  1.92177  5.46046  0.20280  1.69520  0.48576  0.95510
     360   3.47476  4.82131  5.14952  4.56370  2.71742  4.67826  4.93549
2.10481  4.40444  0.71383  3.20287  4.73439  4.90835  3.14462  4.47559
4.00465  3.69238  2.76139  5.27870  4.15820    531 l - - E
         2.68618  4.42225  2.77519  2.73123  3.46354  2.40513  3.72494
3.29354  2.67741  2.69355  4.24690  2.90347  2.73739  3.18146  2.89801
2.37887  2.77519  2.98518  4.58477  3.61503
         0.01309  4.73812  5.46046  0.61958  0.77255  0.48576  0.95510
     361   2.51079  2.00058  3.81824  3.27207  3.83308  1.63248  4.17686
3.19057  3.19477  2.90799  3.26929  3.60476  4.15436  3.50911  2.90436
2.97051  2.78944  2.92849  5.26934  4.04475    532 g - - E
         2.68618  4.42225  2.77519  2.73123  3.46354  2.40513  3.72494
3.29354  2.67741  2.69355  4.24690  2.90347  2.73739  3.18146  2.89801
2.37887  2.77519  2.98518  4.58477  3.61503
         0.01309  4.73812  5.46046  0.61958  0.77255  0.48576  0.95510
     362   5.05712  6.05440  5.55703  5.50682  4.02577  4.85534  5.31184
5.25484  5.30951  4.46680  5.82226  5.54822  5.44445  5.63570  5.25891
5.26087  5.41369  5.15099  0.13078  3.99800    533 W - - E
         2.68618  4.42225  2.77519  2.73123  3.46354  2.40513  3.72494
3.29354  2.67741  2.69355  4.24690  2.90347  2.73739  3.18146  2.89801
2.37887  2.77519  2.98518  4.58477  3.61503
         0.01309  4.73812  5.46046  0.61958  0.77255  0.48576  0.95510
     363   2.81139  5.25799  3.08767  2.44818  4.59008  3.58698  2.70032
4.05449  1.84221  3.27246  3.31244  2.91171  3.97730  1.93012  2.70776
2.63685  3.03910  3.64232  5.69107  4.30668    534 k - - T
         2.68618  4.42225  2.77519  2.73123  3.46354  2.40513  3.72494
3.29354  2.67741  2.69355  4.24690  2.90347  2.73739  3.18146  2.89801
2.37887  2.77519  2.98518  4.58477  3.61503
         0.01309  4.73812  5.46046  0.61958  0.77255  0.48576  0.95510
     364   2.83208  5.33654  1.89761  2.29262  4.67172  2.27407  3.57791
4.15643  2.54083  3.63798  4.38408  2.54523  3.97824  2.88675  2.63432
2.64179  2.57792  3.72271  5.77328  4.35833    535 d - - T
         2.68618  4.42225  2.77519  2.73123  3.46354  2.40513  3.72494
3.29354  2.67741  2.69355  4.24690  2.90347  2.73739  3.18146  2.89801
2.37887  2.77519  2.98518  4.58477  3.61503
         0.01309  4.73812  5.46046  0.61958  0.77255  0.48576  0.95510
     365   2.99080  5.36555  3.31217  2.51600  4.73848  3.73353  3.35841
4.16484  2.07119  2.87792  4.41721  3.21367  4.10916  2.23864  1.39898
2.96491  2.98154  3.77525  5.73839  4.42221    536 r - - E
         2.68618  4.42225  2.77519  2.73123  3.46354  2.40513  3.72494
3.29354  2.67741  2.69355  4.24690  2.90347  2.73739  3.18146  2.89801
2.37887  2.77519  2.98518  4.58477  3.61503
         0.01309  4.73812  5.46046  0.61958  0.77255  0.48576  0.95510
     366   2.24856  4.44488  3.06105  2.34579  4.24228  3.60278  3.81329
3.47381  2.60955  3.26022  4.06908  3.13580  1.88374  2.96008  2.89108
2.41635  2.87090  3.03735  5.49900  4.16053    537 p - - E
         2.68618  4.42225  2.77519  2.73123  3.46354  2.40513  3.72494
3.29354  2.67741  2.69355  4.24690  2.90347  2.73739  3.18146  2.89801
2.37887  2.77519  2.98518  4.58477  3.61503
         0.01309  4.73812  5.46046  0.61958  0.77255  0.48576  0.95510
```

```
    367   3.87783  5.10569  5.76698  5.22403  3.54312  5.28461  5.67992
2.28374  5.08458  0.57478  3.29346  5.42826  5.38082  5.06902  5.10488
4.68871  4.10341  1.83033  5.75639  4.74653    538 L - - E
          2.68618  4.42225  2.77519  2.73123  3.46354  2.40513  3.72494
3.29354  2.67741  2.69355  4.24690  2.90347  2.73739  3.18146  2.89801
2.37887  2.77519  2.98518  4.58477  3.61503
          0.01309  4.73812  5.46046  0.61958  0.77255  0.48576  0.95510
    368   2.80306  4.18968  4.67754  4.06948  2.61461  3.96599  4.29513
2.10841  3.87501  2.09341  3.08914  4.09800  4.32881  4.02295  3.91562
2.96547  2.76603  1.65833  4.81346  2.29837    539 v - - E
          2.68618  4.42225  2.77519  2.73123  3.46354  2.40513  3.72494
3.29354  2.67741  2.69355  4.24690  2.90347  2.73739  3.18146  2.89801
2.37887  2.77519  2.98518  4.58477  3.61503
          0.01309  4.73812  5.46046  0.61958  0.77255  0.48576  0.95510
    369   1.94373  4.40354  3.86466  3.29487  2.91772  3.44991  4.09588
2.89588  3.22863  2.62491  3.52887  3.63588  4.18384  3.50601  3.52917
2.01730  2.11979  2.40970  3.85334  3.79902    540 a - - E
          2.68618  4.42225  2.77519  2.73123  3.46354  2.40513  3.72494
3.29354  2.67741  2.69355  4.24690  2.90347  2.73739  3.18146  2.89801
2.37887  2.77519  2.98518  4.58477  3.61503
          0.01309  4.73812  5.46046  0.61958  0.77255  0.48576  0.95510
    370   1.86161  3.57152  4.45808  3.86529  3.15885  3.94115  4.26979
2.37490  3.71681  2.24543  3.36156  3.99735  4.31102  3.90796  3.83875
2.86174  2.33134  1.66196  4.87402  3.68848    541 v - - E
          2.68618  4.42225  2.77519  2.73123  3.46354  2.40513  3.72494
3.29354  2.67741  2.69355  4.24690  2.90347  2.73739  3.18146  2.89801
2.37887  2.77519  2.98518  4.58477  3.61503
          0.01309  4.73812  5.46046  0.61958  0.77255  0.48576  0.95510
    371   2.62502  3.12303  4.56113  4.24750  4.87509  3.36985  5.03325
4.25712  4.15666  4.00189  4.82203  4.04059  4.18077  4.39050  4.34751
0.55071  2.46155  3.64425  6.25747  5.11897    542 S - - E
          2.68618  4.42225  2.77519  2.73123  3.46354  2.40513  3.72494
3.29354  2.67741  2.69355  4.24690  2.90347  2.73739  3.18146  2.89801
2.37887  2.77519  2.98518  4.58477  3.61503
          0.01309  4.73812  5.46046  0.61958  0.77255  0.48576  0.95510
    372   1.18388  3.55650  4.14617  3.58467  3.20201  3.79655  4.25112
2.83774  3.49145  2.43792  3.54600  3.81960  4.24158  3.73850  3.72714
2.47026  3.05201  2.34873  5.04853  3.84811    543 a - - E
          2.68618  4.42225  2.77519  2.73123  3.46354  2.40513  3.72494
3.29354  2.67741  2.69355  4.24690  2.90347  2.73739  3.18146  2.89801
2.37887  2.77519  2.98518  4.58477  3.61503
          0.01309  4.73812  5.46046  0.61958  0.77255  0.48576  0.95510
    373   5.05712  6.05440  5.55703  5.50682  4.02577  4.85534  5.31184
5.25484  5.30951  4.46680  5.82226  5.54822  5.44445  5.63570  5.25891
5.26087  5.41369  5.15099  0.13078  3.99800    544 W - - E
          2.68618  4.42225  2.77519  2.73123  3.46354  2.40513  3.72494
3.29354  2.67741  2.69355  4.24690  2.90347  2.73739  3.18146  2.89801
2.37887  2.77519  2.98518  4.58477  3.61503
          0.01309  4.73812  5.46046  0.61958  0.77255  0.48576  0.95510
    374   2.78506  5.31995  3.33208  2.61396  4.68063  3.73871  3.83532
4.09665  1.59636  3.58994  4.38323  3.23121  4.11699  2.95193  1.47568
2.97486  2.65922  3.29201  5.71314  4.40504    545 r - - E
          2.68618  4.42225  2.77519  2.73123  3.46354  2.40513  3.72494
3.29354  2.67741  2.69355  4.24690  2.90347  2.73739  3.18146  2.89801
2.37887  2.77519  2.98518  4.58477  3.61503
          0.00883  4.73386        *  0.61958  0.77255  0.00000        *
//
HMMER3/f [3.3 | Nov 2019]
```

```
NAME  Helicase_C
ACC   PF00271.32
DESC  Helicase conserved C-terminal domain
LENG  111
ALPH  amino
RF    no
MM    no
CONS  yes
CS    yes
MAP   yes
DATE  Mon Apr  6 12:55:19 2020
NSEQ  421
EFFN  6.774055
CKSUM 1130081330
GA    23.50 23.50
TC    23.50 23.50
NC    23.40 23.40
STATS LOCAL MSV       -9.5035  0.71514
STATS LOCAL VITERBI  -10.5919  0.71514
STATS LOCAL FORWARD   -3.9356  0.71514
HMM          A        C        D        E        F        G        H
I        K        L        M        N        P        Q        R        S
T        V        W        Y
            m->m     m->i     m->d     i->m     i->i     d->m     d->d
  COMPO   2.59820  4.31883  2.96753  2.75483  3.29280  2.83301  3.66326
2.65062  2.68853  2.43241  3.78770  3.03169  3.52715  3.04201  2.69186
2.67312  2.81143  2.64695  5.08886  3.60349
          2.68618  4.42225  2.77519  2.73123  3.46354  2.40513  3.72494
3.29354  2.67741  2.69355  4.24690  2.90347  2.73739  3.18146  2.89801
2.37887  2.77519  2.98518  4.58477  3.61503
          0.00706  5.61208  5.68920  0.61958  0.77255  0.00000        *
      1   2.43169  4.82918  1.93560  1.97461  4.69174  2.97103  3.96728
3.96405  2.48315  3.45263  4.31632  2.71763  3.58991  2.91879  2.89537
2.47122  3.03175  3.56641  4.88250  3.78602      1 d - - H
          2.68618  4.42225  2.77519  2.73123  3.46354  2.40513  3.72494
3.29354  2.67741  2.69355  4.24690  2.90347  2.73739  3.18146  2.89801
2.37887  2.77519  2.98518  4.58477  3.61503
          0.00545  5.61048  6.33282  0.61958  0.77255  0.49409  0.94193
      2   2.59592  4.94649  3.60800  2.99214  3.51476  3.74492  3.83660
3.48454  1.16273  2.64189  4.23471  3.43795  3.96631  3.07930  2.55814
3.00041  3.10551  3.02065  5.87310  4.11083      2 k - - H
          2.68618  4.42225  2.77519  2.73123  3.46354  2.40513  3.72494
3.29354  2.67741  2.69355  4.24690  2.90347  2.73739  3.18146  2.89801
2.37887  2.77519  2.98518  4.58477  3.61503
          0.00545  5.61048  6.33282  0.61958  0.77255  0.49409  0.94193
      3   2.78196  4.44246  3.10964  3.56711  2.97722  3.99084  4.39771
2.02330  3.29831  1.75362  3.35040  3.44949  2.43573  3.87852  3.43684
3.27176  3.14878  2.40975  4.00777  3.40045      3 l - - H
          2.68618  4.42225  2.77519  2.73123  3.46354  2.40513  3.72494
3.29354  2.67741  2.69355  4.24690  2.90347  2.73739  3.18146  2.89801
2.37887  2.77519  2.98518  4.58477  3.61503
          0.00545  5.61048  6.33282  0.61958  0.77255  0.48059  0.96343
      4   2.26783  5.35316  2.59429  2.05508  4.57956  3.46235  3.73274
3.90461  2.40480  2.32845  4.34880  2.70002  3.90599  2.78680  2.65672
2.41187  3.07242  3.35700  5.78759  4.64354      4 e - - H
          2.68618  4.42225  2.77519  2.73123  3.46354  2.40513  3.72494
3.29354  2.67741  2.69355  4.24690  2.90347  2.73739  3.18146  2.89801
2.37887  2.77519  2.98518  4.58477  3.61503
```

```
          0.00544  5.61208  6.33443  0.61958  0.77255  0.48576  0.95510
      5   2.24645  3.87610  3.20630  2.98997  3.31166  3.98100  2.63896
2.33382  2.67373  2.65301  3.55238  3.48444  3.38886  3.14411  2.81686
3.01375  2.86982  2.87661  4.87111  2.91871      5 a - - H
          2.68618  4.42225  2.77519  2.73123  3.46354  2.40513  3.72494
3.29354  2.67741  2.69355  4.24690  2.90347  2.73739  3.18146  2.89801
2.37887  2.77519  2.98518  4.58477  3.61503
          0.00544  5.61208  6.33443  0.61958  0.77255  0.48576  0.95510
      6   2.99654  3.16058  5.23089  4.61492  2.54242  4.26862  4.76278
1.76124  4.39780  1.16473  3.40184  4.44845  4.50563  4.17508  4.40287
3.42195  3.12678  2.20850  4.93526  4.02893      6 l - - H
          2.68618  4.42225  2.77519  2.73123  3.46354  2.40513  3.72494
3.29354  2.67741  2.69355  4.24690  2.90347  2.73739  3.18146  2.89801
2.37887  2.77519  2.98518  4.58477  3.61503
          0.00544  5.61208  6.33443  0.61958  0.77255  0.48576  0.95510
      7   2.86526  3.22129  3.28515  3.05442  3.68526  3.61471  3.97000
2.34206  2.78186  1.86124  3.59898  3.75520  3.67654  3.43164  3.21565
2.97032  2.81125  2.52442  5.15033  2.42723      7 l - - H
          2.68618  4.42225  2.77519  2.73123  3.46354  2.40513  3.72494
3.29354  2.67741  2.69355  4.24690  2.90347  2.73739  3.18146  2.89801
2.37887  2.77519  2.98518  4.58477  3.61503
          0.01348  5.61208  4.63199  0.61958  0.77255  0.48576  0.95510
      8   1.96351  4.41840  2.60443  1.94347  4.38142  3.43450  3.75358
4.29247  2.32307  3.85422  4.72195  2.51558  3.95155  2.65072  2.70121
2.76441  3.11568  3.42824  5.90142  3.21363      8 e - - H
          2.68618  4.42225  2.77519  2.73123  3.46354  2.40513  3.72494
3.29354  2.67741  2.69355  4.24690  2.90347  2.73739  3.18146  2.89801
2.37887  2.77519  2.98518  4.58477  3.61503
          0.00902  5.60409  5.24091  0.61958  0.77255  0.50140  0.93060
      9   2.73489  4.07558  4.30693  2.68066  2.78492  3.85373  3.92249
2.13601  3.66007  1.29161  3.45210  3.60376  4.69413  3.59290  3.63585
3.10344  3.08922  2.72116  5.17709  3.47666      9 l - - H
          2.68616  4.42218  2.77525  2.73128  3.46362  2.40512  3.72494
3.29343  2.67735  2.69351  4.24698  2.90349  2.73745  3.18145  2.89804
2.37892  2.77523  2.98499  4.58474  3.61500
          0.23221  2.12177  2.43726  0.12138  2.16888  0.48760  0.95218
     10   2.86495  4.24129  4.81564  4.15510  2.92642  4.26302  4.37330
1.97486  4.31924  0.97876  3.30158  4.35033  4.64879  4.38256  4.10964
3.46964  3.21392  2.31268  5.19825  3.42229     11 l - - H
          2.68657  4.42063  2.77513  2.73132  3.46321  2.40505  3.72521
3.29259  2.67718  2.69379  4.24489  2.90392  2.73752  3.18161  2.89842
2.37903  2.77470  2.98549  4.58544  3.61484
          1.14646  0.98611  1.17372  1.14588  0.38264  0.77983  0.61338
     11   2.83407  4.19341  2.75178  2.17835  4.65588  3.63224  3.45203
2.99368  1.63631  3.38449  4.41463  2.95559  3.78533  2.77356  3.00382
2.58353  2.57397  3.61713  5.13313  3.94408     23 k - - H
          2.68618  4.42225  2.77519  2.73123  3.46354  2.40513  3.72494
3.29354  2.67741  2.69355  4.24690  2.90347  2.73739  3.18146  2.89801
2.37887  2.77519  2.98518  4.58477  3.61503
          0.08260  5.16875  2.60932  0.61958  0.77255  0.78378  0.61005
     12   2.61740  4.56338  2.95585  2.28647  3.21755  3.45295  3.52523
3.64928  2.58891  1.85068  3.66989  2.86420  4.18123  2.90685  2.87427
2.51668  2.86136  3.35857  4.91927  4.22474     24 l - - H
          2.68618  4.42225  2.77519  2.73123  3.46354  2.40513  3.72494
3.29354  2.67741  2.69355  4.24690  2.90347  2.73739  3.18146  2.89801
2.37887  2.77519  2.98518  4.58477  3.61503
          0.00837  5.29667  5.70672  0.61958  0.77255  1.06424  0.42311
```

```
     13   2.76396  5.17443  3.17329  1.96262  4.31305  3.40440  3.58872
3.49041  2.21681  3.08764  4.19656  2.76089  3.84247  2.85587  2.29005
2.50144  2.72826  2.80576  5.67792  3.91383     25 e - - H
          2.68618  4.42225  2.77519  2.73123  3.46354  2.40513  3.72494
3.29354  2.67741  2.69355  4.24690  2.90347  2.73739  3.18146  2.89801
2.37887  2.77519  2.98518  4.58477  3.61503
          0.00707  5.35097  6.07332  0.61958  0.77255  0.63133  0.75904
     14   2.50598  5.59721  3.10357  2.09415  4.59486  3.17527  3.25798
3.89048  2.15906  2.63294  4.14292  2.73710  4.08322  2.74689  2.72557
2.56817  3.05349  2.51808  6.03416  4.23077     26 e - - T
          2.68618  4.42225  2.77519  2.73123  3.46354  2.40513  3.72494
3.29354  2.67741  2.69355  4.24690  2.90347  2.73739  3.18146  2.89801
2.37887  2.77519  2.98518  4.58477  3.61503
          0.00621  5.48015  6.20250  0.61958  0.77255  0.65658  0.73110
     15   2.71636  5.41672  2.75850  2.31077  3.82343  3.19360  3.65435
2.76854  2.36973  3.37526  4.18378  2.88435  3.04693  2.84129  2.48713
2.53047  3.05727  2.57904  5.87333  3.80694     27 e - - T
          2.68618  4.42225  2.77519  2.73123  3.46354  2.40513  3.72494
3.29354  2.67741  2.69355  4.24690  2.90347  2.73739  3.18146  2.89801
2.37887  2.77519  2.98518  4.58477  3.61503
          0.00590  5.53079  6.25314  0.61958  0.77255  0.65712  0.73052
     16   2.89382  5.19244  2.74654  2.55333  4.35217  1.86517  3.66135
3.91730  2.49112  3.81898  4.45575  2.66956  3.25339  2.36066  2.30488
2.75787  2.91361  3.55136  5.52221  4.20348     28 g - - T
          2.68618  4.42225  2.77519  2.73123  3.46354  2.40513  3.72494
3.29354  2.67741  2.69355  4.24690  2.90347  2.73739  3.18146  2.89801
2.37887  2.77519  2.98518  4.58477  3.61503
          0.00577  5.55421  6.27655  0.61958  0.77255  0.71508  0.67168
     17   2.92368  5.41571  2.77734  2.16101  4.30511  1.89479  3.24901
4.27957  2.03393  3.79238  4.40999  2.94800  3.41898  2.94510  2.69724
2.67872  3.00792  3.70349  5.66538  3.47201     29 g - - S
          2.68618  4.42225  2.77519  2.73123  3.46354  2.40513  3.72494
3.29354  2.67741  2.69355  4.24690  2.90347  2.73739  3.18146  2.89801
2.37887  2.77519  2.98518  4.58477  3.61503
          0.00575  5.55766  6.28000  0.61958  0.77255  0.70657  0.67991
     18   2.80237  4.39759  2.86992  2.63347  5.00192  3.64645  4.08168
4.48780  1.44847  3.13920  4.42429  2.95508  3.87085  2.18094  2.23734
2.75921  2.83160  3.86690  6.08972  4.67885     30 k - - E
          2.68618  4.42225  2.77519  2.73123  3.46354  2.40513  3.72494
3.29354  2.67741  2.69355  4.24690  2.90347  2.73739  3.18146  2.89801
2.37887  2.77519  2.98518  4.58477  3.61503
          0.00573  5.56050  6.28285  0.61958  0.77255  0.42107  1.06811
     19   2.00777  3.74726  4.61460  4.46010  3.52636  3.90746  4.72785
1.64798  4.27660  2.74093  3.23555  4.26371  4.46562  4.43541  4.29579
2.50967  1.94480  1.86521  5.26809  4.08846     31 i - - E
          2.68617  4.42229  2.77523  2.73127  3.46353  2.40514  3.72498
3.29330  2.67740  2.69354  4.24694  2.90348  2.73743  3.18150  2.89804
2.37886  2.77510  2.98522  4.58481  3.61499
          0.03224  3.50876  6.32502  0.66148  0.72585  0.45711  1.00269
     20   3.16211  4.63416  4.77909  4.27404  3.32859  4.42776  4.55785
1.51493  4.38923  1.17936  3.09569  4.43210  4.79145  4.52118  4.18382
3.66570  3.42492  2.05312  5.25598  3.11448     34 l - - E
          2.68618  4.42225  2.77519  2.73123  3.46354  2.40513  3.72494
3.29354  2.67741  2.69355  4.24690  2.90347  2.73739  3.18146  2.89801
2.37887  2.77519  2.98518  4.58477  3.61503
          0.00544  5.61208  6.33443  0.61958  0.77255  0.48576  0.95510
```

```
     21   3.03251  4.02613  5.02414  4.33337  3.53808  4.24396  4.75982
1.14171  4.23965  2.20870  3.51821  3.88308  4.79060  4.30468  4.39592
3.73761  3.35718  1.39784  5.06413  3.80446     35 i - - E
          2.68618  4.42225  2.77519  2.73123  3.46354  2.40513  3.72494
3.29354  2.67741  2.69355  4.24690  2.90347  2.73739  3.18146  2.89801
2.37887  2.77519  2.98518  4.58477  3.61503
          0.00544  5.61208  6.33443  0.61958  0.77255  0.48576  0.95510
     22   3.23408  4.46743  5.03492  3.93938  0.82114  4.42407  4.75532
2.81259  4.18410  2.38429  3.56164  4.35052  4.78806  4.50495  4.38757
3.70773  3.28501  2.35673  4.78082  2.86464     36 f - - E
          2.68618  4.42225  2.77519  2.73123  3.46354  2.40513  3.72494
3.29354  2.67741  2.69355  4.24690  2.90347  2.73739  3.18146  2.89801
2.37887  2.77519  2.98518  4.58477  3.61503
          0.00544  5.61208  6.33443  0.61958  0.77255  0.48576  0.95510
     23   2.40611  2.40153  4.72311  3.93358  3.40033  3.97112  4.14424
2.31014  3.97175  2.23383  3.57684  3.46415  4.75487  4.18229  4.11615
2.19522  2.51987  1.74441  5.29348  3.63068     37 v - - E
          2.68618  4.42225  2.77519  2.73123  3.46354  2.40513  3.72494
3.29354  2.67741  2.69355  4.24690  2.90347  2.73739  3.18146  2.89801
2.37887  2.77519  2.98518  4.58477  3.61503
          0.00544  5.61208  6.33443  0.61958  0.77255  0.48576  0.95510
     24   2.81152  5.68096  3.12027  2.42026  3.63629  3.69096  3.68455
4.18377  2.56026  3.48719  4.40371  1.72568  2.65240  2.31117  2.65375
2.75534  2.70506  3.86824  5.08205  4.13144     38 n - - S
          2.68618  4.42225  2.77519  2.73123  3.46354  2.40513  3.72494
3.29354  2.67741  2.69355  4.24690  2.90347  2.73739  3.18146  2.89801
2.37887  2.77519  2.98518  4.58477  3.61503
          0.00544  5.61208  6.33443  0.61958  0.77255  0.48576  0.95510
     25   2.67466  5.38197  3.46376  2.86453  3.22561  2.74549  3.97505
3.75810  2.76565  2.78115  3.78650  3.13782  4.41405  3.13796  2.69722
2.04643  1.86071  3.59971  4.57829  2.88404     39 t - - S
          2.68618  4.42225  2.77519  2.73123  3.46354  2.40513  3.72494
3.29354  2.67741  2.69355  4.24690  2.90347  2.73739  3.18146  2.89801
2.37887  2.77519  2.98518  4.58477  3.61503
          0.00544  5.61208  6.33443  0.61958  0.77255  0.48576  0.95510
     26   2.94423  4.17920  3.75890  3.03392  3.85722  3.88303  4.29968
1.58001  2.28020  3.03046  3.99005  3.58269  4.53917  2.99869  2.30831
3.20075  2.76937  2.40352  3.82928  3.61299     40 i - - H
          2.68618  4.42225  2.77519  2.73123  3.46354  2.40513  3.72494
3.29354  2.67741  2.69355  4.24690  2.90347  2.73739  3.18146  2.89801
2.37887  2.77519  2.98518  4.58477  3.61503
          0.00544  5.61208  6.33443  0.61958  0.77255  0.48576  0.95510
     27   2.58544  5.48933  1.66096  2.21088  4.65566  3.82770  3.85880
3.92605  2.02916  3.93931  4.41790  2.78841  4.28214  2.93040  2.55048
2.54723  2.91176  3.49605  6.11949  4.27222     41 d - - H
          2.68608  4.42207  2.77518  2.73068  3.46196  2.40451  3.72500
3.29302  2.67801  2.69387  4.24248  2.90405  2.73801  3.18169  2.89817
2.37934  2.77550  2.98520  4.58495  3.61546
          0.40857  1.14997  3.97634  1.10114  0.40420  0.48576  0.95510
     28   2.71100  5.24507  2.66314  2.23427  4.13726  3.55965  3.63153
3.93852  2.39443  3.01484  3.07571  3.16418  4.20517  2.91787  1.93244
2.39213  2.59566  3.44077  5.82519  4.03863     48 r - - H
          2.68618  4.42225  2.77519  2.73123  3.46354  2.40513  3.72494
3.29354  2.67741  2.69355  4.24690  2.90347  2.73739  3.18146  2.89801
2.37887  2.77519  2.98518  4.58477  3.61503
          0.00969  5.59496  5.12903  0.61958  0.77255  0.53046  0.88754
```

```
      29   2.16002  2.73905  5.18166  4.36622  3.02394  4.33926  4.56932
2.14801  4.12867  1.46357  3.29479  4.39888  4.77736  3.48659  4.37819
2.91988  3.17839  1.91984  5.24784  4.07121      49 l - - H
         2.68625  4.42180  2.77507  2.73093  3.46373  2.40517  3.72482
3.29333  2.67729  2.69352  4.24709  2.90339  2.73759  3.18150  2.89806
2.37897  2.77526  2.98537  4.58425  3.61522
         0.35598  3.40059  1.32367  1.76311  0.18815  0.52048  0.90198
      30   2.74776  5.35166  1.86739  1.79801  4.78522  3.48881  3.66505
4.35183  2.17776  3.82578  4.47474  2.89829  4.10751  2.69692  2.99582
2.68193  3.08832  3.72939  5.95294  2.85461      59 e - - H
         2.68618  4.42225  2.77519  2.73123  3.46354  2.40513  3.72494
3.29354  2.67741  2.69355  4.24690  2.90347  2.73739  3.18146  2.89801
2.37887  2.77519  2.98518  4.58477  3.61503
         0.32139  5.29644  1.30989  0.61958  0.77255  1.03755  0.43745
      31   2.70745  5.11476  2.79267  2.46503  3.79942  3.47792  3.76136
3.19577  1.83202  2.74030  3.40934  3.07940  3.07449  2.80908  2.86509
2.68179  2.92994  3.11084  5.04860  3.74585      60 k - - H
         2.68582  4.42240  2.77539  2.73073  3.46397  2.40548  3.72560
3.29329  2.67761  2.69161  4.24684  2.90405  2.73789  3.18175  2.89807
2.37928  2.77505  2.98471  4.58532  3.61572
         1.02271  0.45048  5.78975  1.15238  0.37963  1.43205  0.27288
      32   2.35038  3.71450  3.53887  2.75973  3.03554  3.58207  3.98084
2.69925  2.88024  2.14264  3.36759  3.54761  3.75337  3.45859  3.45196
2.80307  2.39938  2.13922  4.89201  3.64306      77 v - - H
         2.68618  4.42225  2.77519  2.73123  3.46354  2.40513  3.72494
3.29354  2.67741  2.69355  4.24690  2.90347  2.73739  3.18146  2.89801
2.37887  2.77519  2.98518  4.58477  3.61503
         0.00897  5.11441  5.83675  0.61958  0.77255  0.13538  2.06659
      33   2.62640  5.36201  2.68085  2.01192  4.88285  3.08641  3.76830
4.32111  1.81932  3.79376  4.51269  2.95209  3.96333  2.79214  2.31759
2.43712  2.79631  3.12951  5.86583  4.66195      78 k - - H
         2.68618  4.42225  2.77519  2.73123  3.46354  2.40513  3.72494
3.29354  2.67741  2.69355  4.24690  2.90347  2.73739  3.18146  2.89801
2.37887  2.77519  2.98518  4.58477  3.61503
         0.00544  5.61208  6.33443  0.61958  0.77255  0.48576  0.95510
      34   2.96226  4.66195  3.96711  3.26498  2.72875  4.19370  4.00220
2.30096  2.98121  1.58978  3.28998  3.93914  4.56509  3.53736  3.36216
2.82114  3.24120  2.50341  4.07172  2.30452      79 l - - H
         2.68618  4.42225  2.77519  2.73123  3.46354  2.40513  3.72494
3.29354  2.67741  2.69355  4.24690  2.90347  2.73739  3.18146  2.89801
2.37887  2.77519  2.98518  4.58477  3.61503
         0.00544  5.61208  6.33443  0.61958  0.77255  0.48576  0.95510
      35   2.99602  4.30747  5.22423  4.37180  1.86940  4.42793  4.49001
2.14700  4.30725  1.11612  3.11413  4.59106  4.79160  4.46214  4.26708
3.12749  3.33237  2.50578  5.16664  3.57435      80 l - - H
         2.68634  4.42264  2.77554  2.73085  3.46382  2.40543  3.72507
3.29340  2.67692  2.69350  4.24674  2.90324  2.73792  3.18143  2.89799
2.37878  2.77548  2.98532  4.58249  3.61426
         0.58500  1.85215  1.25179  1.50865  0.25001  0.48576  0.95510
      36   2.74452  4.15054  3.23132  2.48827  3.73995  3.40380  3.73931
3.45854  2.16901  1.65839  3.97460  3.06192  3.45611  2.83804  2.74819
2.90204  2.89050  3.56724  5.76288  3.97046      96 l - - H
         2.68618  4.42225  2.77519  2.73123  3.46354  2.40513  3.72494
3.29354  2.67741  2.69355  4.24690  2.90347  2.73739  3.18146  2.89801
2.37887  2.77519  2.98518  4.58477  3.61503
         0.03971  5.27770  3.38660  0.61958  0.77255  1.36030  0.29650
```

37 2.80167 5.10803 2.95513 1.58754 3.86108 3.34027 3.60648
3.64038 2.17972 2.90524 4.39277 2.83879 3.90356 2.64123 2.74062
2.60797 2.89417 3.68806 5.24458 4.34970 97 e - - H
2.68618 4.42225 2.77519 2.73123 3.46354 2.40513 3.72494
3.29354 2.67741 2.69355 4.24690 2.90347 2.73739 3.18146 2.89801
2.37887 2.77519 2.98518 4.58477 3.61503
0.01469 5.25156 4.67261 0.61958 0.77255 0.31477 1.30919
38 2.72030 4.87233 3.02227 2.48799 4.85045 3.81130 3.73884
3.85659 1.81287 2.90065 4.25093 2.61851 3.70537 2.33263 2.28681
2.59756 2.76395 3.56714 5.41871 3.85692 98 k - - H
2.68618 4.42225 2.77519 2.73123 3.46354 2.40513 3.72494
3.29354 2.67741 2.69355 4.24690 2.90347 2.73739 3.18146 2.89801
2.37887 2.77519 2.98518 4.58477 3.61503
0.00582 5.54550 6.26784 0.61958 0.77255 0.59611 0.80062
39 2.75243 5.64066 3.09718 2.26808 2.28067 3.10288 3.71893
3.55837 2.24404 3.07057 4.38139 2.42359 3.81588 2.54644 2.75846
2.55226 3.20772 3.76053 5.30501 3.90139 99 k - - T
2.68611 4.42292 2.77541 2.73102 3.46296 2.40484 3.72525
3.29249 2.67749 2.69293 4.24685 2.90350 2.73681 3.18175 2.89798
2.37938 2.77599 2.98578 4.58440 3.61543
0.61367 0.97462 2.50959 1.19109 0.36225 0.41065 1.08832
40 2.52915 5.12635 2.91063 2.35029 4.50224 1.58140 3.68948
4.10165 2.24226 3.50165 4.55473 2.71063 3.85020 2.81542 2.97869
2.51161 3.26850 3.64969 6.06969 4.31661 113 g - - T
2.68619 4.42231 2.77513 2.73125 3.46360 2.40519 3.72488
3.29360 2.67740 2.69346 4.24696 2.90347 2.73738 3.18145 2.89796
2.37888 2.77526 2.98507 4.58483 3.61509
0.04975 3.06618 6.25013 0.70156 0.68480 0.31592 1.30607
41 2.84429 4.56836 4.30769 3.82842 2.23849 3.79095 3.81080
1.56587 3.53629 2.14270 3.50294 4.39219 4.42698 4.00439 3.10377
2.93811 3.19013 2.28018 4.54634 2.73339 118 i - - S
2.68618 4.42225 2.77519 2.73123 3.46354 2.40513 3.72494
3.29354 2.67741 2.69355 4.24690 2.90347 2.73739 3.18146 2.89801
2.37887 2.77519 2.98518 4.58477 3.61503
0.00544 5.61208 6.33443 0.61958 0.77255 0.48576 0.95510
42 2.92307 5.10223 3.12140 2.51061 4.40450 3.30205 3.70290
3.75161 1.83929 3.46877 3.90936 2.49678 3.28402 2.55924 2.08858
2.47808 2.73145 3.70062 6.11794 4.55255 119 k - - C
2.68618 4.42225 2.77519 2.73123 3.46354 2.40513 3.72494
3.29354 2.67741 2.69355 4.24690 2.90347 2.73739 3.18146 2.89801
2.37887 2.77519 2.98518 4.58477 3.61503
0.00544 5.61208 6.33443 0.61958 0.77255 0.48576 0.95510
43 2.00383 3.28083 5.18638 4.48538 2.63315 4.02695 4.32505
1.94594 4.29780 2.34228 3.71030 4.41381 4.28092 4.40691 4.38657
2.35393 2.80117 1.87205 5.25904 2.72633 120 v - - E
2.68618 4.42225 2.77519 2.73123 3.46354 2.40513 3.72494
3.29354 2.67741 2.69355 4.24690 2.90347 2.73739 3.18146 2.89801
2.37887 2.77519 2.98518 4.58477 3.61503
0.04744 5.61208 3.15402 0.61958 0.77255 0.48576 0.95510
44 1.71301 2.83845 3.31288 3.09832 3.48433 3.09600 4.04546
2.59561 3.16151 2.19726 3.81181 3.54680 4.21699 3.46831 3.29734
2.79498 2.80239 2.61992 5.53207 3.65803 121 a - - E
2.68618 4.42225 2.77519 2.73123 3.46354 2.40513 3.72494
3.29354 2.67741 2.69355 4.24690 2.90347 2.73739 3.18146 2.89801
2.37887 2.77519 2.98518 4.58477 3.61503
0.00568 5.57032 6.29267 0.61958 0.77255 0.67864 0.70787

```
     45   2.53512  3.94170  4.19541  3.32952  3.45600  3.92995  3.80337
2.31101  2.79309  2.42938  3.61780  3.86110  2.76170  3.52231  2.61783
2.83040  2.64690  2.05762  5.07935  2.97450    122 v - - E
          2.68618  4.42225  2.77519  2.73123  3.46354  2.40513  3.72494
3.29354  2.67741  2.69355  4.24690  2.90347  2.73739  3.18146  2.89801
2.37887  2.77519  2.98518  4.58477  3.61503
          0.00568  5.57032  6.29267  0.61958  0.77255  0.67864  0.70787
     46   2.99731  3.92318  5.18818  4.57330  2.99345  3.40955  3.63936
1.79797  4.17766  1.20028  2.96591  4.09061  4.76368  4.49215  3.93938
3.67147  3.43827  2.23210  5.22888  3.09623    123 l - - E
          2.68618  4.42225  2.77519  2.73123  3.46354  2.40513  3.72494
3.29354  2.67741  2.69355  4.24690  2.90347  2.73739  3.18146  2.89801
2.37887  2.77519  2.98518  4.58477  3.61503
          0.00568  5.57032  6.29267  0.61958  0.77255  0.38167  1.14797
     47   3.04570  5.62249  2.15123  2.75902  4.25675  3.96282  1.50703
3.92492  2.82674  3.72892  4.55467  2.10604  4.35563  3.18499  3.22495
2.97998  2.86850  3.69979  6.07164  2.98399    124 h - - S
          2.68618  4.42225  2.77519  2.73123  3.46354  2.40513  3.72494
3.29354  2.67741  2.69355  4.24690  2.90347  2.73739  3.18146  2.89801
2.37887  2.77519  2.98518  4.58477  3.61503
          0.00544  5.61208  6.33443  0.61958  0.77255  0.48576  0.95510
     48   2.49497  4.90554  3.67679  3.03564  4.23850  1.00990  4.16844
3.74460  3.12062  3.52074  4.29888  3.66311  4.46736  3.49650  3.57723
1.86602  3.30635  3.55011  5.80906  4.33097    125 g - - T
          2.68620  4.42227  2.77518  2.73123  3.46356  2.40515  3.72497
3.29356  2.67740  2.69357  4.24692  2.90349  2.73742  3.18144  2.89800
2.37878  2.77513  2.98521  4.58479  3.61505
          0.11744  3.28837  2.61056  0.28040  1.40847  0.48576  0.95510
     49   2.81378  5.49279  2.42716  1.83457  4.89748  2.79437  3.78317
4.48046  2.41510  3.57112  4.62338  2.88242  4.24210  2.58798  2.33495
2.03348  2.90738  4.03867  6.07936  4.58898    127 e - - T
          2.68607  4.42245  2.77516  2.73124  3.46363  2.40496  3.72515
3.29374  2.67743  2.69375  4.24710  2.90340  2.73748  3.18132  2.89800
2.37882  2.77513  2.98508  4.58497  3.61511
          0.05242  3.09757  5.13170  1.64934  0.21341  0.35233  1.21418
     50   3.11720  4.41731  4.57245  4.28870  3.69430  4.04592  4.59465
2.43379  3.31476  1.26829  1.87840  4.37630  4.74268  3.80004  3.19086
3.45000  2.32846  2.73910  5.29605  3.67635    137 l - - S
          2.68618  4.42225  2.77519  2.73123  3.46354  2.40513  3.72494
3.29354  2.67741  2.69355  4.24690  2.90347  2.73739  3.18146  2.89801
2.37887  2.77519  2.98518  4.58477  3.61503
          0.00635  5.60393  5.93275  0.61958  0.77255  0.50940  0.91843
     51   3.06215  5.67906  3.02729  2.45766  4.64883  3.16968  3.90850
4.47017  2.40120  3.41760  3.96158  2.76397  1.84718  3.01146  3.14561
2.01447  2.17204  4.01292  6.11258  4.65001    138 p - - T
          2.68618  4.42225  2.77519  2.73123  3.46354  2.40513  3.72494
3.29354  2.67741  2.69355  4.24690  2.90347  2.73739  3.18146  2.89801
2.37887  2.77519  2.98518  4.58477  3.61503
          0.00548  5.60520  6.32755  0.61958  0.77255  0.48296  0.95960
     52   2.12649  5.24143  3.32658  2.73915  4.44478  3.49043  3.69910
3.98362  2.05663  3.47230  4.57317  3.03983  3.12914  1.60062  3.14799
2.60680  2.79932  3.55222  6.10435  4.00408    139 q - - H
          2.68618  4.42225  2.77519  2.73123  3.46354  2.40513  3.72494
3.29354  2.67741  2.69355  4.24690  2.90347  2.73739  3.18146  2.89801
2.37887  2.77519  2.98518  4.58477  3.61503
          0.00545  5.60979  6.33213  0.61958  0.77255  0.47841  0.96697
```

```
     53   2.62088  5.68782  2.53959  2.05694  4.23413  3.40785  3.91788
3.76876  1.94352  3.79226  4.55196  1.92770  3.71419  2.88902  2.78608
2.58516  3.06206  3.57441  6.12005  4.53601    140 n - - H
          2.68618  4.42225  2.77519  2.73123  3.46354  2.40513  3.72494
3.29354  2.67741  2.69355  4.24690  2.90347  2.73739  3.18146  2.89801
2.37887  2.77519  2.98518  4.58477  3.61503
          0.00544  5.61208  6.33443  0.61958  0.77255  0.48576  0.95510
     54   2.70496  5.68496  2.50184  1.63985  4.93823  3.52513  3.66305
2.97019  2.68506  3.45805  4.41969  2.43359  4.34295  2.27029  3.15635
2.35096  3.02984  3.63218  6.11795  4.64254    141 e - - H
          2.68618  4.42225  2.77519  2.73123  3.46354  2.40513  3.72494
3.29354  2.67741  2.69355  4.24690  2.90347  2.73739  3.18146  2.89801
2.37887  2.77519  2.98518  4.58477  3.61503
          0.00952  5.61208  5.14664  0.61958  0.77255  0.48576  0.95510
     55   3.21034  5.46059  3.48485  2.89077  4.99331  3.94358  3.93926
4.08388  2.34082  3.68113  4.45745  3.12017  3.51040  2.17664  1.01993
3.14342  3.38644  3.28534  6.09942  4.71033    142 r - - H
          2.68618  4.42225  2.77519  2.73123  3.46354  2.40513  3.72494
3.29354  2.67741  2.69355  4.24690  2.90347  2.73739  3.18146  2.89801
2.37887  2.77519  2.98518  4.58477  3.61503
          0.00546  5.60803  6.33038  0.61958  0.77255  0.50658  0.92269
     56   2.89610  5.51535  2.44295  2.00157  4.54955  3.63793  4.11219
2.76802  2.40770  2.12838  4.34274  3.03886  4.17407  2.61478  2.47029
2.88210  2.94950  3.26417  5.78426  4.61431    143 e - - H
          2.68613  4.42254  2.77542  2.73048  3.46299  2.40538  3.72523
3.29383  2.67704  2.69384  4.24560  2.90334  2.73768  3.18058  2.89788
2.37915  2.77548  2.98540  4.58506  3.61532
          0.04952  3.06768  6.33038  1.89008  0.16377  0.47296  0.97592
     57   2.47593  4.66590  2.85448  1.84766  4.83840  3.55207  2.28089
3.88697  2.03225  3.34645  4.24021  3.23528  4.30493  2.66117  2.67885
2.76925  2.92577  3.67348  5.15951  4.37816    156 e - - H
          2.68618  4.42225  2.77519  2.73123  3.46354  2.40513  3.72494
3.29354  2.67741  2.69355  4.24690  2.90347  2.73739  3.18146  2.89801
2.37887  2.77519  2.98518  4.58477  3.61503
          0.00544  5.61208  6.33443  0.61958  0.77255  0.48576  0.95510
     58   2.37076  4.46172  4.40370  3.78316  3.86596  4.03281  4.44841
1.50579  3.61438  2.36335  3.55889  3.70430  4.69414  3.43488  3.72121
2.64693  2.26880  1.87296  5.36498  4.16680    157 i - - H
          2.68618  4.42225  2.77519  2.73123  3.46354  2.40513  3.72494
3.29354  2.67741  2.69355  4.24690  2.90347  2.73739  3.18146  2.89801
2.37887  2.77519  2.98518  4.58477  3.61503
          0.00544  5.61208  6.33443  0.61958  0.77255  0.48576  0.95510
     59   2.95153  4.52063  5.18493  3.04249  2.43968  4.42304  4.49495
1.78123  4.01738  1.28389  2.57811  4.57346  4.56709  4.12095  4.38607
3.64219  3.36040  2.34021  5.25929  3.81336    158 l - - H
          2.68618  4.42225  2.77519  2.73123  3.46354  2.40513  3.72494
3.29354  2.67741  2.69355  4.24690  2.90347  2.73739  3.18146  2.89801
2.37887  2.77519  2.98518  4.58477  3.61503
          0.00544  5.61208  6.33443  0.61958  0.77255  0.48576  0.95510
     60   2.48997  5.68864  2.48955  1.72269  4.85686  3.81433  3.63582
4.46864  2.26541  3.64964  4.35827  2.57965  4.34224  2.68606  2.43211
2.38930  2.64186  3.90283  5.86069  4.49480    159 e - - H
          2.68618  4.42225  2.77519  2.73123  3.46354  2.40513  3.72494
3.29354  2.67741  2.69355  4.24690  2.90347  2.73739  3.18146  2.89801
2.37887  2.77519  2.98518  4.58477  3.61503
          0.00544  5.61208  6.33443  0.61958  0.77255  0.48576  0.95510
```

```
     61   2.51762  4.70972  2.58639  2.38892  5.03293  3.47376  3.90878
4.19735  2.06818  3.30386  4.58097  3.07825  3.65414  1.93610  2.18159
2.46445  3.12392  3.76618  5.59071  4.39532    160 q - - H
          2.68618  4.42225  2.77519  2.73123  3.46354  2.40513  3.72494
3.29354  2.67741  2.69355  4.24690  2.90347  2.73739  3.18146  2.89801
2.37887  2.77519  2.98518  4.58477  3.61503
          0.00544  5.61208  6.33443  0.61958  0.77255  0.48576  0.95510
     62   2.92554  4.55407  5.07113  4.46555  0.75732  3.84700  4.47064
2.92341  4.07932  2.42304  3.72861  4.35786  3.63805  4.24463  4.34247
3.62488  3.30849  2.81414  4.39704  3.47166    161 f - - H
          2.68619  4.42228  2.77520  2.73126  3.46356  2.40515  3.72497
3.29357  2.67743  2.69357  4.24678  2.90337  2.73742  3.18149  2.89803
2.37883  2.77519  2.98507  4.58479  3.61506
          0.17152  3.84988  1.99266  0.65437  0.73348  0.48576  0.95510
     63   2.68288  5.60127  3.30020  2.73842  4.81393  3.80631  3.89646
4.22672  1.92649  3.79475  4.64271  1.59803  3.20754  2.92161  1.88501
2.85431  3.17599  3.92975  5.84216  4.34058    164 n - - H
          2.68591  4.42253  2.77517  2.73121  3.46370  2.40541  3.72522
3.29270  2.67766  2.69379  4.24721  2.90281  2.73748  3.18094  2.89825
2.37865  2.77531  2.98547  4.58512  3.61528
          0.26003  1.49796  5.22406  0.85769  0.55189  0.27248  1.43334
     64   1.95752  5.39040  2.67885  2.37652  4.40316  3.60424  3.74840
4.41044  1.95417  3.71257  4.35735  2.53800  3.25715  2.57119  2.77743
2.36184  2.91990  3.96064  5.90605  4.37948    170 k - - T
          2.68619  4.42230  2.77513  2.73118  3.46359  2.40515  3.72499
3.29359  2.67739  2.69360  4.24694  2.90338  2.73738  3.18151  2.89803
2.37883  2.77516  2.98523  4.58482  3.61508
          0.09456  3.29875  2.93178  0.65216  0.73589  0.49669  0.93788
     65   2.93930  5.13738  2.91844  2.57452  4.66444  1.12921  3.30161
4.39376  2.51473  3.83305  4.63636  2.78582  3.63568  3.04763  3.11475
2.70905  3.19011  4.03903  6.08359  4.67398    173 g - - T
          2.68618  4.42225  2.77519  2.73123  3.46354  2.40513  3.72494
3.29354  2.67741  2.69355  4.24690  2.90347  2.73739  3.18146  2.89801
2.37887  2.77519  2.98518  4.58477  3.61503
          0.00663  5.55538  5.89835  0.61958  0.77255  0.40584  1.09787
     66   2.86086  4.26848  2.51757  1.99981  5.02539  3.57006  3.54178
4.38948  2.03564  2.55515  4.72116  2.94878  3.85952  2.96264  2.72867
2.30836  2.44951  3.48744  6.11324  4.55980    174 e - - S
          2.68623  4.42275  2.77520  2.73174  3.46404  2.40563  3.72416
3.29320  2.67700  2.69359  4.24740  2.90283  2.73742  3.18047  2.89851
2.37866  2.77520  2.98501  4.58527  3.61553
          0.08252  3.00941  3.51044  2.36906  0.09824  0.45713  1.00266
     67   2.89846  3.47069  3.86785  3.16299  2.43666  3.65704  3.59468
2.66287  2.88329  2.56944  3.79994  3.22610  4.32682  3.14274  2.74080
2.39472  2.66355  2.45611  5.55100  2.60289    191 s - - S
          2.68618  4.42225  2.77519  2.73123  3.46354  2.40513  3.72494
3.29354  2.67741  2.69355  4.24690  2.90347  2.73739  3.18146  2.89801
2.37887  2.77519  2.98518  4.58477  3.61503
          0.00669  5.58386  5.83962  0.61958  0.77255  0.60429  0.79068
     68   2.99624  5.25507  1.77243  2.76065  3.88987  3.74167  3.74758
3.19211  2.04540  3.43790  4.60406  2.41016  3.17741  2.92270  2.58334
2.79126  2.66528  3.72826  5.22918  4.58333    192 d - - S
          2.68625  4.42129  2.77533  2.73136  3.46368  2.40526  3.72395
3.29338  2.67724  2.69360  4.24688  2.90351  2.73755  3.18138  2.89807
2.37898  2.77533  2.98469  4.58495  3.61509
          0.28280  1.40852  6.30716  0.06643  2.74461  0.41182  1.08603
```

```
     69   3.06896  3.92587  5.24891  4.63363  2.54154  4.27504  4.78119
1.54911  4.41659  2.10988  3.41487  4.54999  4.80812  4.54599  4.42105
3.75775  3.26723  1.35498  5.27068  2.61397    194 v - - E
          2.68618  4.42225  2.77519  2.73123  3.46354  2.40513  3.72494
3.29354  2.67741  2.69355  4.24690  2.90347  2.73739  3.18146  2.89801
2.37887  2.77519  2.98518  4.58477  3.61503
          0.00544  5.61208  6.33443  0.61958  0.77255  0.48576  0.95510
     70   4.08908  4.39876  6.24076  5.66848  3.82301  5.57998  5.96608
1.74706  5.51547  0.59206  3.31120  5.75408  5.76707  5.52532  5.50276
4.94551  4.43731  2.05530  6.18279  5.12223    195 L - - E
          2.68620  4.42229  2.77523  2.73127  3.46338  2.40507  3.72498
3.29342  2.67744  2.69348  4.24693  2.90350  2.73743  3.18150  2.89804
2.37890  2.77520  2.98512  4.58480  3.61499
          0.03152  3.53165  6.33443  0.63510  0.75477  0.48576  0.95510
     71   3.14977  4.38774  5.31845  4.70848  3.22625  4.37894  4.87239
1.24859  4.49707  1.70063  3.61182  4.69300  4.88601  4.63007  4.50638
3.61210  3.44739  1.41084  5.35919  4.00091    198 i - - E
          2.68618  4.42225  2.77519  2.73123  3.46354  2.40513  3.72494
3.29354  2.67741  2.69355  4.24690  2.90347  2.73739  3.18146  2.89801
2.37887  2.77519  2.98518  4.58477  3.61503
          0.00544  5.61208  6.33443  0.61958  0.77255  0.48576  0.95510
     72   1.11138  3.23730  3.64606  3.27378  4.06584  2.86915  4.27078
3.83325  3.25579  3.49481  4.34565  3.25977  4.50618  3.56010  3.68292
1.90371  2.62425  3.27555  5.79983  4.51532    199 a - - E
          2.68618  4.42225  2.77519  2.73123  3.46354  2.40513  3.72494
3.29354  2.67741  2.69355  4.24690  2.90347  2.73739  3.18146  2.89801
2.37887  2.77519  2.98518  4.58477  3.61503
          0.00544  5.61208  6.33443  0.61958  0.77255  0.48576  0.95510
     73   3.13500  4.62048  4.52660  3.95504  3.97104  4.29635  4.64809
2.95166  3.86606  2.74001  3.92508  4.23221  4.49313  4.02928  4.11728
1.85591  0.85480  2.87890  5.46265  4.18502    200 t - - S
          2.68618  4.42225  2.77519  2.73123  3.46354  2.40513  3.72494
3.29354  2.67741  2.69355  4.24690  2.90347  2.73739  3.18146  2.89801
2.37887  2.77519  2.98518  4.58477  3.61503
          0.00544  5.61208  6.33443  0.61958  0.77255  0.48576  0.95510
     74   2.82715  5.49406  1.50328  2.78154  5.03154  3.87333  4.13636
4.51756  2.39616  3.82676  4.47989  2.11125  4.04789  2.96158  2.73689
2.12819  2.72959  3.99278  6.11903  4.27097    201 d - - G
          2.68618  4.42225  2.77519  2.73123  3.46354  2.40513  3.72494
3.29354  2.67741  2.69355  4.24690  2.90347  2.73739  3.18146  2.89801
2.37887  2.77519  2.98518  4.58477  3.61503
          0.00544  5.61208  6.33443  0.61958  0.77255  0.48576  0.95510
     75   2.06400  4.25149  2.82009  2.91587  3.62231  4.36593  4.67458
1.88514  4.07124  2.38478  3.46249  4.37878  4.73430  4.27769  4.22469
3.25050  2.96301  1.42634  5.31981  3.98218    202 v - - G
          2.68666  4.42342  2.77561  2.73096  3.46471  2.40474  3.72224
3.29471  2.67654  2.69472  4.24155  2.90378  2.73712  3.18263  2.89748
2.37751  2.77561  2.98635  4.58594  3.61620
          0.07073  2.73200  5.74421  2.95672  0.05339  0.48576  0.95510
     76   1.43279  4.22822  5.09133  4.48446  3.43622  2.36642  4.73675
2.19324  4.29678  1.82713  3.45910  2.77019  4.77503  4.33229  4.34997
3.61560  3.42003  2.34978  4.91042  4.04129    228 a - - G
          2.68618  4.42225  2.77519  2.73123  3.46354  2.40513  3.72494
3.29354  2.67741  2.69355  4.24690  2.90347  2.73739  3.18146  2.89801
2.37887  2.77519  2.98518  4.58477  3.61503
          0.00545  5.61066  6.33300  0.61958  0.77255  0.48117  0.96250
```

```
     77   1.99287  5.70437  3.17995  1.54722  5.04661  2.36693  4.16003
4.53113  2.86607  4.00946  4.74871  3.24925  4.36271  3.16773  3.23278
1.65960  3.14514  4.00178  6.14063  4.72969    229 e - - T
          2.68618  4.42225  2.77519  2.73123  3.46354  2.40513  3.72494
3.29354  2.67741  2.69355  4.24690  2.90347  2.73739  3.18146  2.89801
2.37887  2.77519  2.98518  4.58477  3.61503
          0.00544  5.61208  6.33443  0.61958  0.77255  0.48576  0.95510
     78   2.92853  4.96368  3.48684  1.84749  4.63612  3.97730  4.17068
4.07235  2.81557  2.75812  3.73367  3.04159  4.36937  2.47778  1.71497
2.78746  2.50231  2.81064  6.02373  4.35576    230 r - - S
          2.68563  4.42439  2.77635  2.72868  3.46372  2.40591  3.72708
3.29568  2.67068  2.69478  4.24904  2.90115  2.73859  3.18213  2.89793
2.38035  2.77635  2.98490  4.58691  3.61717
          0.08973  2.47635  6.33443  3.34275  0.03598  0.48576  0.95510
     79   3.23138  4.94629  5.75359  5.77183  6.26111  0.30334  6.34379
5.79222  5.74088  5.48431  6.20164  5.03588  4.99874  5.79095  5.69951
1.93448  4.00095  4.78666  7.59411  6.60436    266 G - - S
          2.68618  4.42225  2.77519  2.73123  3.46354  2.40513  3.72494
3.29354  2.67741  2.69355  4.24690  2.90347  2.73739  3.18146  2.89801
2.37887  2.77519  2.98518  4.58477  3.61503
          0.00544  5.61208  6.33443  0.61958  0.77255  0.48576  0.95510
     80   3.51218  4.86098  5.49300  4.88745  3.48801  4.72193  5.06918
1.08408  4.68357  1.37825  3.52862  4.88652  5.05826  4.80054  4.12233
4.04247  3.47878  1.78090  5.52177  4.07587    267 i - - S
          2.68618  4.42225  2.77519  2.73123  3.46354  2.40513  3.72494
3.29354  2.67741  2.69355  4.24690  2.90347  2.73739  3.18146  2.89801
2.37887  2.77519  2.98518  4.58477  3.61503
          0.00544  5.61208  6.33443  0.61958  0.77255  0.48576  0.95510
     81   3.72164  6.26198  0.81792  3.08308  5.61037  4.00741  4.21245
5.12019  3.52755  4.17404  5.39518  1.84619  4.73595  3.75798  4.08333
3.20456  2.02618  4.67461  6.74986  5.27973    268 d - - T
          2.68618  4.42225  2.77519  2.73123  3.46354  2.40513  3.72494
3.29354  2.67741  2.69355  4.24690  2.90347  2.73739  3.18146  2.89801
2.37887  2.77519  2.98518  4.58477  3.61503
          0.00544  5.61208  6.33443  0.61958  0.77255  0.48576  0.95510
     82   3.31047  4.61375  5.32553  4.71341  1.89789  4.53259  4.87206
1.38793  4.28971  1.55356  3.58210  4.69812  4.88796  4.53030  4.50663
3.81667  3.40610  1.88181  5.35182  4.00894    269 i - - S
          2.68618  4.42225  2.77519  2.73123  3.46354  2.40513  3.72494
3.29354  2.67741  2.69355  4.24690  2.90347  2.73739  3.18146  2.89801
2.37887  2.77519  2.98518  4.58477  3.61503
          0.03435  5.61208  3.50278  0.61958  0.77255  0.48576  0.95510
     83   2.89493  5.08949  2.78929  2.65828  4.85658  3.37828  3.78769
3.99333  2.09008  3.87419  4.70575  3.04582  1.65263  2.31543  3.26543
2.70790  2.64628  3.57245  6.09859  3.62646    270 p - - T
          2.68618  4.42225  2.77520  2.73124  3.46354  2.40513  3.72495
3.29354  2.67741  2.69351  4.24690  2.90347  2.73740  3.18141  2.89801
2.37887  2.77520  2.98519  4.58477  3.61503
          0.03136  4.50502  3.92111  0.49272  0.94407  0.40850  1.09258
     84   2.58603  3.04112  2.23312  2.44660  4.57984  2.28631  3.80716
4.45271  2.04635  3.82820  4.66422  2.46379  4.33266  2.83227  2.78843
2.61422  3.01765  3.89132  6.10929  4.30058    272 k - - T
          2.68618  4.42225  2.77519  2.73123  3.46354  2.40513  3.72494
3.29354  2.67741  2.69355  4.24690  2.90347  2.73739  3.18146  2.89801
2.37887  2.77519  2.98518  4.58477  3.61503
          0.00554  5.59445  6.31679  0.61958  0.77255  0.43495  1.04212
```

```
     85   1.86556  2.93106  4.66381  4.54380  3.68680  4.06109  4.75328
2.15185  4.21465  2.28932  3.76500  4.56176  4.78690  4.48901  3.78918
3.48190  3.40143  1.11856  5.26836  4.09067    273 v - - E
          2.68618  4.42225  2.77519  2.73123  3.46354  2.40513  3.72494
3.29354  2.67741  2.69355  4.24690  2.90347  2.73739  3.18146  2.89801
2.37887  2.77519  2.98518  4.58477  3.61503
          0.00544  5.61208  6.33443  0.61958  0.77255  0.48576  0.95510
     86   2.98576  4.99576  2.18459  2.65850  5.03369  3.78374  2.72936
4.21006  2.38750  3.91262  4.55866  1.86295  4.09906  2.56491  2.64157
2.41930  2.54112  3.52277  6.12008  4.70866    274 n - - E
          2.68618  4.42225  2.77519  2.73123  3.46354  2.40513  3.72494
3.29354  2.67741  2.69355  4.24690  2.90347  2.73739  3.18146  2.89801
2.37887  2.77519  2.98518  4.58477  3.61503
          0.00544  5.61208  6.33443  0.61958  0.77255  0.48576  0.95510
     87   2.90105  3.90241  4.09328  4.06941  3.13426  4.27017  2.32581
2.97378  3.85487  1.87358  3.41155  2.77781  4.71662  3.91593  3.11055
3.55720  2.03146  2.12491  4.67038  2.93913    275 l - - E
          2.68618  4.42225  2.77519  2.73123  3.46354  2.40513  3.72494
3.29354  2.67741  2.69355  4.24690  2.90347  2.73739  3.18146  2.89801
2.37887  2.77519  2.98518  4.58477  3.61503
          0.00544  5.61208  6.33443  0.61958  0.77255  0.48576  0.95510
     88   3.29336  3.95530  5.54019  4.70437  4.04866  4.44217  5.17631
2.09132  4.75980  2.33736  2.92100  4.95614  5.13602  4.90890  4.78755
4.12039  3.61503  0.64134  5.65403  4.47043    276 V - - E
          2.68618  4.42225  2.77519  2.73123  3.46354  2.40513  3.72494
3.29354  2.67741  2.69355  4.24690  2.90347  2.73739  3.18146  2.89801
2.37887  2.77519  2.98518  4.58477  3.61503
          0.00544  5.61208  6.33443  0.61958  0.77255  0.48576  0.95510
     89   3.03134  4.51374  5.23026  4.61530  3.04418  4.14393  3.47281
0.94419  4.40045  2.41715  3.32514  4.59901  4.79966  4.53162  4.40872
3.74809  3.49808  1.69088  5.02735  3.98211    277 i - - E
          2.68621  4.42232  2.77510  2.73126  3.46349  2.40512  3.72501
3.29361  2.67743  2.69358  4.24696  2.90348  2.73746  3.18153  2.89800
2.37878  2.77526  2.98509  4.58484  3.61484
          0.02812  3.65157  6.33443  1.09095  0.40932  0.48576  0.95510
     90   3.20230  4.51362  2.44451  3.06309  3.13581  4.08462  2.99240
2.16265  3.09957  2.69740  3.35588  1.70154  4.47097  2.99419  2.88370
3.23569  3.35224  3.01993  5.74239  4.14404    282 n - - E
          2.68618  4.42225  2.77519  2.73123  3.46354  2.40513  3.72494
3.29354  2.67741  2.69355  4.24690  2.90347  2.73739  3.18146  2.89801
2.37887  2.77519  2.98518  4.58477  3.61503
          0.00544  5.61208  6.33443  0.61958  0.77255  0.48576  0.95510
     91   3.01267  3.54638  4.71776  3.59859  1.59367  4.00010  4.24968
2.56108  4.33200  2.18023  3.01898  4.30479  4.39275  4.37287  4.27321
2.89896  2.93602  2.68969  4.71779  1.83067    283 f - - S
          2.68618  4.42225  2.77519  2.73123  3.46354  2.40513  3.72494
3.29354  2.67741  2.69355  4.24690  2.90347  2.73739  3.18146  2.89801
2.37887  2.77519  2.98518  4.58477  3.61503
          0.00544  5.61208  6.33443  0.61958  0.77255  0.48576  0.95510
     92   3.12484  4.40358  1.24257  2.40173  4.88863  2.44555  3.15717
4.40535  2.85594  3.82311  4.65360  2.79454  4.18478  3.08149  3.03612
2.50349  3.06955  4.07412  6.11746  4.70715    284 d - - S
          2.68618  4.42225  2.77519  2.73123  3.46354  2.40513  3.72494
3.29354  2.67741  2.69355  4.24690  2.90347  2.73739  3.18146  2.89801
2.37887  2.77519  2.98518  4.58477  3.61503
          0.00544  5.61208  6.33443  0.61958  0.77255  0.48576  0.95510
```

```
     93   2.26083  3.97262  4.96528  4.28502  2.50201  3.74536  4.52474
2.67056  3.18681  1.68812  2.31973  4.07727  2.49956  4.11101  3.71027
3.13722  3.12668  2.63456  5.28906  3.16786    285 l - - C
          2.68618  4.42225  2.77519  2.73123  3.46354  2.40513  3.72494
3.29354  2.67741  2.69355  4.24690  2.90347  2.73739  3.18146  2.89801
2.37887  2.77519  2.98518  4.58477  3.61503
          0.00544  5.61208  6.33443  0.61958  0.77255  0.48576  0.95510
     94   2.65456  3.84936  2.47579  2.72574  3.42645  3.80977  3.81618
3.77758  2.72194  3.62319  4.22935  3.43406  1.39533  3.17273  3.35746
2.86145  2.96055  2.83711  4.12883  4.43631    286 p - - S
          2.68663  4.42261  2.77519  2.73130  3.46318  2.40559  3.72541
3.29361  2.67652  2.69367  4.24594  2.90346  2.73769  3.18166  2.89731
2.37899  2.77537  2.98469  4.58505  3.61478
          0.23294  1.61521  4.71624  1.35884  0.29700  0.48576  0.95510
     95   2.96730  5.44017  2.83460  2.69254  2.94441  3.04918  3.90783
4.21995  2.40884  3.22676  4.50223  3.20266  3.45842  2.35585  2.22043
2.68980  2.70337  3.94977  2.54810  3.36992    294 r - - S
          2.68618  4.42225  2.77519  2.73123  3.46354  2.40513  3.72494
3.29354  2.67741  2.69355  4.24690  2.90347  2.73739  3.18146  2.89801
2.37887  2.77519  2.98518  4.58477  3.61503
          0.00548  5.60488  6.32723  0.61958  0.77255  0.50104  0.93115
     96   3.08113  5.48166  2.28550  2.36224  4.89151  2.63923  3.93029
4.35111  2.80478  3.65623  4.72352  1.71028  4.16832  3.15304  3.21034
1.76865  2.68930  4.00632  6.11572  4.70510    295 n - - S
          2.68630  4.42230  2.77534  2.73125  3.46338  2.40515  3.72492
3.29356  2.67743  2.69337  4.24557  2.90358  2.73750  3.18159  2.89764
2.37890  2.77524  2.98529  4.58480  3.61512
          0.14068  2.08328  5.00436  0.76846  0.62311  0.47119  0.97885
     97   2.88480  4.65921  4.25557  3.26497  3.72177  4.11427  4.24313
2.46233  3.51254  2.33884  3.28179  3.96443  2.11696  3.91075  3.43854
2.90730  2.43022  1.71681  5.17131  2.83262    300 v - - H
          2.68639  4.42147  2.77561  2.73176  3.46372  2.40606  3.72595
3.29248  2.67736  2.69311  4.24681  2.90393  2.73752  3.18148  2.89817
2.37824  2.77463  2.98480  4.58310  3.61422
          0.15199  1.97171  6.32952  2.27797  0.10813  0.47606  0.97082
     98   2.10893  4.62379  2.52256  2.38490  4.59562  2.99016  3.76129
3.13511  2.72263  3.51076  4.34290  3.30219  4.14567  2.86794  2.79699
2.25488  1.96693  3.48079  6.09300  4.38144    319 t - - H
          2.68618  4.42225  2.77519  2.73123  3.46354  2.40513  3.72494
3.29354  2.67741  2.69355  4.24690  2.90347  2.73739  3.18146  2.89801
2.37887  2.77519  2.98518  4.58477  3.61503
          0.00545  5.61138  6.33373  0.61958  0.77255  0.48349  0.95875
     99   2.76254  5.67316  2.57910  2.40506  4.60916  2.66939  3.73765
3.11563  2.69081  3.11470  4.02371  2.79372  4.34525  2.28034  2.90524
1.87833  2.84403  3.35134  5.81288  4.64584    320 s - - H
          2.68618  4.42225  2.77519  2.73123  3.46354  2.40513  3.72494
3.29354  2.67741  2.69355  4.24690  2.90347  2.73739  3.18146  2.89801
2.37887  2.77519  2.98518  4.58477  3.61503
          0.00544  5.61208  6.33443  0.61958  0.77255  0.48576  0.95510
    100   2.52312  3.99661  3.09321  3.16092  2.88951  4.36178  4.39844
2.61282  4.05765  1.97958  3.25728  4.29742  4.73041  4.02531  3.61827
3.55924  3.32541  2.69023  5.32115  1.34135    321 y - - H
          2.68618  4.42225  2.77519  2.73123  3.46354  2.40513  3.72494
3.29354  2.67741  2.69355  4.24690  2.90347  2.73739  3.18146  2.89801
2.37887  2.77519  2.98518  4.58477  3.61503
          0.00544  5.61208  6.33443  0.61958  0.77255  0.48576  0.95510
```

```
    101   2.85601  4.91652  3.35013  2.79305  4.28449  4.04796  3.01926
1.49539  2.59101  2.52441  3.62071  3.10076  4.50413  3.20822  3.24819
3.23303  3.16050  2.28218  4.29520  3.91588    322 i - - H
          2.68618  4.42225  2.77519  2.73123  3.46354  2.40513  3.72494
3.29354  2.67741  2.69355  4.24690  2.90347  2.73739  3.18146  2.89801
2.37887  2.77519  2.98518  4.58477  3.61503
          0.00544  5.61208  6.33443  0.61958  0.77255  0.48576  0.95510
    102   4.11650  6.24883  4.09000  3.74301  5.00247  4.62083  1.26864
5.14190  3.19010  4.35716  5.47069  4.05843  5.09621  0.75557  3.42256
4.09533  4.34197  4.26847  6.26100  4.80555    323 q - - H
          2.68618  4.42225  2.77519  2.73123  3.46354  2.40513  3.72494
3.29354  2.67741  2.69355  4.24690  2.90347  2.73739  3.18146  2.89801
2.37887  2.77519  2.98518  4.58477  3.61503
          0.00544  5.61208  6.33443  0.61958  0.77255  0.48576  0.95510
    103   2.28916  4.70428  3.92998  3.13659  4.16906  4.04406  4.38615
2.96309  3.33483  2.63849  3.19814  3.81507  4.53166  3.21344  1.11874
2.54059  3.13748  3.14279  5.61716  4.36049    324 r - - H
          2.68618  4.42225  2.77519  2.73123  3.46354  2.40513  3.72494
3.29354  2.67741  2.69355  4.24690  2.90347  2.73739  3.18146  2.89801
2.37887  2.77519  2.98518  4.58477  3.61503
          0.00544  5.61208  6.33443  0.61958  0.77255  0.48576  0.95510
    104   2.07400  4.32894  4.26201  3.36022  3.76303  3.99222  4.51601
1.66879  2.71904  2.64605  3.14965  3.95637  4.62480  3.55380  2.29099
2.46569  3.27721  2.39204  5.45963  4.24076    325 i - - H
          2.68618  4.42225  2.77519  2.73123  3.46354  2.40513  3.72494
3.29354  2.67741  2.69355  4.24690  2.90347  2.73739  3.18146  2.89801
2.37887  2.77519  2.98518  4.58477  3.61503
          0.00544  5.61208  6.33443  0.61958  0.77255  0.48576  0.95510
    105   3.09089  4.07075  2.75119  3.59562  5.19883  0.46495  4.91206
4.62378  3.82357  4.25869  4.70748  3.86884  4.73707  4.11344  4.24902
3.23970  3.77173  3.86712  6.50394  5.21824    326 G - - T
          2.68618  4.42225  2.77519  2.73123  3.46354  2.40513  3.72494
3.29354  2.67741  2.69355  4.24690  2.90347  2.73739  3.18146  2.89801
2.37887  2.77519  2.98518  4.58477  3.61503
          0.00544  5.61208  6.33443  0.61958  0.77255  0.48576  0.95510
    106   5.00363  6.74679  5.75805  4.82458  6.53314  5.28260  5.18897
5.92744  3.09310  5.17622  6.14200  5.03459  5.67850  4.34513  0.13878
5.01205  5.11535  5.62462  6.94336  6.01599    327 R - - T
          2.68618  4.42225  2.77519  2.73123  3.46354  2.40513  3.72494
3.29354  2.67741  2.69355  4.24690  2.90347  2.73739  3.18146  2.89801
2.37887  2.77519  2.98518  4.58477  3.61503
          0.01498  5.61208  4.49079  0.61958  0.77255  0.48576  0.95510
    107   1.46741  3.71350  4.85194  4.26150  3.80714  3.65874  4.69607
2.56404  4.11995  2.75376  3.82349  4.23063  4.31821  4.19059  4.25620
3.20107  1.42497  2.05573  5.32209  4.13581    328 t - - S
          2.68618  4.42225  2.77519  2.73123  3.46354  2.40513  3.72494
3.29354  2.67741  2.69355  4.24690  2.90347  2.73739  3.18146  2.89801
2.37887  2.77519  2.98518  4.58477  3.61503
          0.04874  5.60260  3.12614  0.61958  0.77255  0.45692  1.00301
    108   1.88399  4.79247  4.15854  3.78264  3.83432  1.17931  2.95829
2.78165  3.70715  2.77592  3.66356  3.97223  4.62740  3.89462  3.78280
3.41972  3.35651  2.83854  4.25089  3.38614    329 g - - S
          2.68618  4.42225  2.77519  2.73123  3.46354  2.40513  3.72494
3.29354  2.67741  2.69355  4.24690  2.90347  2.73739  3.18146  2.89801
2.37887  2.77519  2.98518  4.58477  3.61503
          0.00871  5.56949  5.32599  0.61958  0.77255  0.38010  1.15137
```

```
    109   4.46706  5.60453  5.37110  4.38313  6.15860  4.95588  4.46090
5.44918  2.80452  4.75771  5.67530  4.38743  5.33349  3.92926  0.23083
3.98693  4.61196  5.12043  6.65537  5.62573    330 R - - S
          2.68618  4.42225  2.77519  2.73123  3.46354  2.40513  3.72494
3.29354  2.67741  2.69355  4.24690  2.90347  2.73739  3.18146  2.89801
2.37887  2.77519  2.98518  4.58477  3.61503
          0.00546  5.60919  6.33154  0.61958  0.77255  0.50066  0.93173
    110   1.58979  4.43518  3.90403  3.64227  3.07749  2.47651  3.95231
2.27658  3.22290  2.55871  3.43330  3.46443  3.75888  3.84643  3.49441
3.08440  3.11148  2.32229  4.65965  3.43540    331 a - - T
          2.68618  4.42225  2.77519  2.73123  3.46354  2.40513  3.72494
3.29354  2.67741  2.69355  4.24690  2.90347  2.73739  3.18146  2.89801
2.37887  2.77519  2.98518  4.58477  3.61503
          0.01080  5.60919  4.95098  0.61958  0.77255  0.50066  0.93173
    111   2.88366  4.68525  3.13786  2.60453  4.73054  1.44044  3.80873
4.32735  2.41855  3.70080  4.46156  2.41165  3.50664  3.01996  2.71267
2.82169  3.27694  3.81742  6.10480  2.90205    332 g - - T
          2.68618  4.42225  2.77519  2.73123  3.46354  2.40513  3.72494
3.29354  2.67741  2.69355  4.24690  2.90347  2.73739  3.18146  2.89801
2.37887  2.77519  2.98518  4.58477  3.61503
          0.00370  5.60209        *  0.61958  0.77255  0.00000        *
//
HMMER3/f [3.3 | Nov 2019]
NAME  HSF_DNA-bind
ACC   PF00447.18
DESC  HSF-type DNA-binding
LENG  99
ALPH  amino
RF    no
MM    no
CONS  yes
CS    yes
MAP   yes
DATE  Tue Apr 21 21:24:00 2020
NSEQ  371
EFFN  2.459709
CKSUM 4237207462
GA    21.80 21.80
TC    21.80 21.80
NC    21.70 21.70
STATS LOCAL MSV       -9.6468  0.71780
STATS LOCAL VITERBI  -10.7247  0.71780
STATS LOCAL FORWARD   -3.6686  0.71780
HMM          A        C        D        E        F        G        H
I        K        L        M        N        P        Q        R        S
T        V        W        Y
            m->m     m->i     m->d     i->m     i->i     d->m     d->d
  COMPO   2.74270  4.58734  3.02343  2.65414  2.76703  3.13225  3.47441
2.96949  2.49777  2.51673  3.70768  2.96357  3.50303  2.94103  2.83196
2.61183  2.90690  2.81914  4.24074  3.36451
          2.68618  4.42225  2.77519  2.73123  3.46354  2.40513  3.72494
3.29354  2.67741  2.69355  4.24690  2.90347  2.73739  3.18146  2.89801
2.37887  2.77519  2.98518  4.58477  3.61503
          0.01261  4.77510  5.49744  0.61958  0.77255  0.00000        *
      1   4.64247  5.78651  5.51449  5.44979  0.23321  4.96089  4.61332
4.17679  5.36837  3.35935  4.79570  5.17065  5.44464  5.30781  5.26713
4.81092  4.96526  4.21769  4.72354  3.03279      1 F - - H
```

```
         2.68618  4.42225  2.77519  2.73123  3.46354  2.40513  3.72494
3.29354  2.67741  2.69355  4.24690  2.90347  2.73739  3.18146  2.89801
2.37887  2.77519  2.98518  4.58477  3.61503
         0.01261  4.77510  5.49744  0.61958  0.77255  0.48576  0.95510
      2  2.82921  4.27859  4.57716  3.98152  3.36152  4.01736  4.24626
1.77276  3.81941  1.59277  3.36242  4.09097  2.48041  3.99940  3.91970
3.31150  3.06427  1.94896  4.91302  3.51351      2 l - - H
         2.68618  4.42225  2.77519  2.73123  3.46354  2.40513  3.72494
3.29354  2.67741  2.69355  4.24690  2.90347  2.73739  3.18146  2.89801
2.37887  2.77519  2.98518  4.58477  3.61503
         0.01261  4.77510  5.49744  0.61958  0.77255  0.48576  0.95510
      3  2.59505  3.92999  3.03475  2.31786  4.35983  3.54783  2.68743
3.35845  2.26533  3.00360  3.71863  2.91605  3.78330  2.64381  2.68357
2.39060  2.65952  3.28520  5.53267  4.02798      3 k - - H
         2.68618  4.42225  2.77519  2.73123  3.46354  2.40513  3.72494
3.29354  2.67741  2.69355  4.24690  2.90347  2.73739  3.18146  2.89801
2.37887  2.77519  2.98518  4.58477  3.61503
         0.01261  4.77510  5.49744  0.61958  0.77255  0.48576  0.95510
      4  2.87290  5.31022  3.08161  2.57936  4.61432  3.49288  3.27478
3.94617  1.15076  3.57984  4.35851  2.92636  4.02527  2.61294  2.73758
2.78283  3.03657  3.61775  5.72785  4.35733      4 k - - H
         2.68618  4.42225  2.77519  2.73123  3.46354  2.40513  3.72494
3.29354  2.67741  2.69355  4.24690  2.90347  2.73739  3.18146  2.89801
2.37887  2.77519  2.98518  4.58477  3.61503
         0.01261  4.77510  5.49744  0.61958  0.77255  0.48576  0.95510
      5  3.41429  4.78774  5.05115  4.47609  3.45389  4.61363  4.96703
2.51581  4.33149  0.73604  3.11631  4.67096  4.88861  4.47106  4.44885
3.94419  2.20840  2.43152  5.39973  4.30292      5 l - - H
         2.68618  4.42225  2.77519  2.73123  3.46354  2.40513  3.72494
3.29354  2.67741  2.69355  4.24690  2.90347  2.73739  3.18146  2.89801
2.37887  2.77519  2.98518  4.58477  3.61503
         0.01261  4.77510  5.49744  0.61958  0.77255  0.48576  0.95510
      6  3.05538  4.43844  4.56069  3.94720  1.99912  4.11286  2.95368
2.90494  3.67940  2.63313  3.41345  4.09999  4.49774  4.02938  3.89839
3.35167  3.27850  2.74591  2.48762  1.44050      6 y - - H
         2.68618  4.42225  2.77519  2.73123  3.46354  2.40513  3.72494
3.29354  2.67741  2.69355  4.24690  2.90347  2.73739  3.18146  2.89801
2.37887  2.77519  2.98518  4.58477  3.61503
         0.01261  4.77510  5.49744  0.61958  0.77255  0.48576  0.95510
      7  2.51800  5.10478  2.35043  2.19304  3.72367  3.51300  3.10150
3.95309  2.19051  3.50669  4.10357  2.79205  3.95775  2.66624  2.54181
2.43766  2.67890  3.60739  5.72025  4.20884      7 k - - H
         2.68618  4.42225  2.77519  2.73123  3.46354  2.40513  3.72494
3.29354  2.67741  2.69355  4.24690  2.90347  2.73739  3.18146  2.89801
2.37887  2.77519  2.98518  4.58477  3.61503
         0.01261  4.77510  5.49744  0.61958  0.77255  0.48576  0.95510
      8  2.85762  4.29990  4.93090  4.15103  3.15584  4.23333  4.57309
1.68139  4.14292  1.64471  1.73026  4.37040  4.56493  4.26924  4.17720
3.55067  3.26394  2.13464  5.03996  3.80630      8 l - - H
         2.68618  4.42216  2.77521  2.73121  3.46355  2.40514  3.72496
3.29346  2.67742  2.69353  4.24668  2.90348  2.73741  3.18148  2.89802
2.37885  2.77521  2.98520  4.58478  3.61504
         0.03618  3.45981  5.49744  0.66614  0.72091  0.48576  0.95510
      9  2.62312  4.20040  4.69295  4.09950  3.40288  4.02235  4.43092
2.20417  3.93247  1.34681  3.34537  3.90582  4.44378  4.10679  4.01691
3.37060  2.90757  1.54284  4.98191  3.79900     12 l - - H
```

```
          2.68627  4.42231  2.77471  2.73126  3.46376  2.40525  3.72499
3.29368  2.67749  2.69363  4.24711  2.90320  2.73716  3.18163  2.89810
2.37867  2.77525  2.98543  4.58502  3.61504
          0.20178  1.82585  3.83298  1.44097  0.27010  0.48576  0.95510
     10   2.60673  4.45457  2.49992  1.75931  4.48998  3.25200  3.47396
4.10171  2.46888  3.54576  4.28953  2.34197  3.95202  2.64301  2.92180
2.30133  2.99118  3.62840  5.71662  4.19243     18 e - - C
          2.68632  4.42154  2.77524  2.73125  3.46349  2.40520  3.72479
3.29341  2.67741  2.69339  4.24687  2.90324  2.73743  3.18130  2.89812
2.37900  2.77524  2.98513  4.58491  3.61517
          0.25088  2.57139  1.92792  1.53258  0.24332  0.46227  0.99386
     11   2.76891  5.26965  1.70941  2.28785  4.60343  3.49364  3.58219
4.03195  2.45920  3.57009  4.27335  2.36920  3.66343  2.59541  2.94789
2.43052  2.92421  3.46715  5.70715  4.29606     29 d - - T
          2.68618  4.42225  2.77519  2.73123  3.46354  2.40513  3.72494
3.29354  2.67741  2.69355  4.24690  2.90347  2.73739  3.18146  2.89801
2.37887  2.77519  2.98518  4.58477  3.61503
          0.32295  4.62567  1.32353  0.61958  0.77255  0.71453  0.67221
     12   2.64150  5.03895  2.75217  2.11046  4.38587  3.37434  3.59534
3.65392  2.31924  3.32269  4.13257  2.83470  2.24050  2.64476  2.86426
2.49805  2.83903  3.43286  5.53595  4.09478     30 e - - G
          2.68611  4.42190  2.77535  2.73134  3.46372  2.40503  3.72512
3.29366  2.67734  2.69357  4.24708  2.90357  2.73715  3.18126  2.89785
2.37890  2.77532  2.98536  4.58495  3.61473
          0.15259  2.27028  3.26391  1.78907  0.18286  1.06932  0.42044
     13   2.37822  4.97725  2.59060  2.17152  4.40693  3.11341  3.60961
3.79411  2.30890  3.22086  4.08742  2.54047  3.82117  2.53770  2.80272
2.37585  2.83955  3.29243  5.53808  4.12139     47 e - - G
          2.68618  4.42225  2.77519  2.73123  3.46354  2.40513  3.72494
3.29354  2.67741  2.69355  4.24690  2.90347  2.73739  3.18146  2.89801
2.37887  2.77519  2.98518  4.58477  3.61503
          0.02018  4.30897  5.03132  0.61958  0.77255  1.02199  0.44610
     14   2.60554  4.37756  3.30739  2.59895  3.22041  3.54865  3.69117
2.85090  2.73012  2.19597  3.48681  2.96509  3.97022  3.11338  3.18276
2.69076  2.38162  2.70200  5.01966  2.92126     48 l - - G
          2.68618  4.42225  2.77519  2.73123  3.46354  2.40513  3.72494
3.29354  2.67741  2.69355  4.24690  2.90347  2.73739  3.18146  2.89801
2.37887  2.77519  2.98518  4.58477  3.61503
          0.02087  4.34141  4.87475  0.61958  0.77255  0.53609  0.87956
     15   2.50452  4.81391  2.32031  2.30859  4.56741  3.10209  3.50228
4.00129  2.30940  3.51432  4.26951  2.63745  3.20389  2.46290  2.68401
2.34404  2.91367  3.57571  5.66320  3.99653     49 e - - T
          2.68618  4.42225  2.77519  2.73123  3.46354  2.40513  3.72494
3.29354  2.67741  2.69355  4.24690  2.90347  2.73739  3.18146  2.89801
2.37887  2.77519  2.98518  4.58477  3.61503
          0.02082  4.60041  4.55052  0.61958  0.77255  0.34325  1.23600
     16   2.50185  4.30640  2.37843  2.24966  4.62830  3.02055  2.91576
4.11171  2.32941  3.57332  4.23101  2.73173  3.51510  2.60147  2.55873
2.54456  2.83042  3.66391  5.72172  3.70537     50 e - - T
          2.68618  4.42225  2.77519  2.73123  3.46354  2.40513  3.72494
3.29354  2.67741  2.69355  4.24690  2.90347  2.73739  3.18146  2.89801
2.37887  2.77519  2.98518  4.58477  3.61503
          0.01274  4.76556  5.48790  0.61958  0.77255  0.49464  0.94108
     17   2.74152  4.07276  4.42886  3.99733  2.67122  3.76654  4.25950
1.79757  3.85840  1.69139  3.22575  3.96862  4.32210  4.00922  3.90342
2.94075  2.92624  1.99267  4.81126  3.00378     51 l - - T
```

2.68627 4.42224 2.77519 2.73121 3.46363 2.40519 3.72475
3.29354 2.67746 2.69357 4.24699 2.90348 2.73754 3.18142 2.89799
2.37867 2.77522 2.98530 4.58379 3.61496
0.20135 1.72455 5.49131 1.05545 0.42776 0.47730 0.96878
18 3.17584 4.41912 5.46627 4.95289 4.01024 4.80963 5.50380
0.90593 4.85251 2.48007 3.76987 5.06827 5.20456 5.07786 4.98027
4.27030 3.62518 1.17507 5.93993 4.72738 60 i - - S
2.68618 4.42225 2.77519 2.73123 3.46354 2.40513 3.72494
3.29354 2.67741 2.69355 4.24690 2.90347 2.73739 3.18146 2.89801
2.37887 2.77519 2.98518 4.58477 3.61503
0.01388 4.77510 5.23193 0.61958 0.77255 0.48576 0.95510
19 2.68459 3.87681 3.19549 2.63225 4.00225 3.21470 3.39605
3.57111 2.55803 3.21080 4.01111 3.08441 4.01908 2.65317 2.42611
1.95372 2.66177 3.20462 3.27173 3.52050 61 s - - E
2.68618 4.42225 2.77519 2.73123 3.46354 2.40513 3.72494
3.29354 2.67741 2.69355 4.24690 2.90347 2.73739 3.18146 2.89801
2.37887 2.77519 2.98518 4.58477 3.61503
0.01263 4.77385 5.49620 0.61958 0.77255 0.48402 0.95790
20 4.03653 5.36332 5.07985 4.77927 2.41465 4.01046 4.08786
3.93660 4.53013 3.30776 4.53192 4.58236 5.14714 4.63584 4.58379
4.18044 4.30468 3.82072 0.52215 2.51121 62 W - - E
2.68619 4.42226 2.77521 2.73124 3.46355 2.40514 3.72496
3.29355 2.67742 2.69356 4.24691 2.90348 2.73741 3.18147 2.89802
2.37888 2.77521 2.98497 4.58478 3.61499
0.08652 3.03812 3.35371 0.38300 1.14512 0.48576 0.95510
21 2.61406 4.90777 2.64870 2.50224 4.36907 2.83336 3.72369
3.77932 2.53785 3.17031 3.94011 2.45378 3.76282 2.88147 3.00004
1.91232 2.61205 2.94445 5.60045 4.17829 64 s - - S
2.68618 4.42225 2.77519 2.73123 3.46354 2.40513 3.72494
3.29354 2.67741 2.69355 4.24690 2.90347 2.73739 3.18146 2.89801
2.37887 2.77519 2.98518 4.58477 3.61503
0.01531 4.74375 5.03849 0.61958 0.77255 0.55294 0.85627
22 2.56258 5.28096 2.43849 2.04153 4.62304 3.41393 3.61337
4.02791 2.26924 3.40054 4.28865 2.89874 2.53550 2.71796 2.54592
2.47489 2.87038 3.66161 5.71534 4.25813 65 e - - S
2.68620 4.42227 2.77506 2.73121 3.46354 2.40514 3.72493
3.29354 2.67739 2.69357 4.24687 2.90348 2.73741 3.18148 2.89802
2.37888 2.77520 2.98518 4.58479 3.61505
0.07149 2.73704 5.46384 0.33572 1.25466 0.44336 1.02687
23 2.48167 4.79387 2.19101 2.21313 4.63460 3.29317 3.31510
4.10038 2.33840 3.56566 4.33405 2.32435 3.82757 2.66899 2.89393
2.46639 2.58187 3.68158 5.72702 4.12995 67 d - - S
2.68621 4.42228 2.77515 2.73115 3.46356 2.40515 3.72493
3.29356 2.67743 2.69358 4.24693 2.90335 2.73736 3.18145 2.89804
2.37890 2.77521 2.98520 4.58480 3.61501
0.07375 3.06919 3.70367 0.80214 0.59487 0.48576 0.95510
24 2.75956 4.78357 2.82372 2.49287 4.38192 1.45624 3.78757
3.97396 2.33045 3.48765 4.26921 2.81419 3.98353 2.80274 2.92482
2.64124 3.03199 3.58165 5.67265 4.28840 72 g - - S
2.68620 4.42143 2.77522 2.73125 3.46356 2.40504 3.72497
3.29356 2.67743 2.69354 4.24692 2.90349 2.73742 3.18149 2.89799
2.37889 2.77522 2.98521 4.58479 3.61505
0.05807 3.07894 4.56540 0.65244 0.73558 0.47210 0.97734
25 2.70386 5.11638 2.29152 2.29580 4.62475 3.29209 3.64227
3.79900 2.16584 3.25654 4.14744 2.39462 3.73599 2.65686 2.76971
2.47442 2.45804 3.63809 5.71977 4.31164 75 k - - S

```
          2.68633  4.42240  2.77497  2.73103  3.46369  2.40476  3.72509
3.29306  2.67739  2.69370  4.24705  2.90362  2.73737  3.18161  2.89795
2.37902  2.77534  2.98533  4.58492  3.61518
          0.04938  3.12261  5.48733  1.89042  0.16371  0.47770  0.96813
     26   2.21860  3.69712  3.34426  2.78859  3.89802  3.06120  3.77878
3.15747  2.48342  2.97842  3.62134  3.18405  4.07017  3.12374  3.15549
1.84850  2.54470  2.73805  5.30396  3.67556     89 s - - E
          2.68618  4.42225  2.77519  2.73123  3.46354  2.40513  3.72494
3.29354  2.67741  2.69355  4.24690  2.90347  2.73739  3.18146  2.89801
2.37887  2.77519  2.98518  4.58477  3.61503
          0.01263  4.77349  5.49583  0.61958  0.77255  0.48938  0.94934
     27   3.18903  4.53732  5.04783  4.45600  1.14046  4.38785  4.65347
2.12639  4.27480  1.99155  3.22685  4.50485  4.69331  4.37432  4.21342
3.70943  3.41686  1.94130  4.71897  3.82921     90 f - - E
          2.68618  4.42225  2.77519  2.73123  3.46354  2.40513  3.72494
3.29354  2.67741  2.69355  4.24690  2.90347  2.73739  3.18146  2.89801
2.37887  2.77519  2.98518  4.58477  3.61503
          0.01263  4.77349  5.49583  0.61958  0.77255  0.48351  0.95872
     28   2.47903  3.51763  4.05595  3.71316  3.17874  3.76150  3.82301
1.99248  3.54095  2.20551  3.25411  3.86607  4.29201  3.57450  3.67252
2.87600  2.97169  1.58528  4.76972  3.35598     91 v - - E
          2.68618  4.42225  2.77519  2.73123  3.46354  2.40513  3.72494
3.29354  2.67741  2.69355  4.24690  2.90347  2.73739  3.18146  2.89801
2.37887  2.77519  2.98518  4.58477  3.61503
          0.01261  4.77510  5.49744  0.61958  0.77255  0.48576  0.95510
     29   3.50944  4.49584  5.55154  5.02715  3.36044  4.98007  5.50423
1.11130  4.91642  1.95053  3.66279  5.13158  5.22336  5.07816  5.00163
4.36182  3.75525  1.12085  5.85253  4.68500     92 i - - E
          2.68618  4.42225  2.77519  2.73123  3.46354  2.40513  3.72494
3.29354  2.67741  2.69355  4.24690  2.90347  2.73739  3.18146  2.89801
2.37887  2.77519  2.98518  4.58477  3.61503
          0.13420  4.77510  2.14429  0.61958  0.77255  0.48576  0.95510
     30   2.60004  4.46361  3.28703  2.74472  3.53297  3.64745  3.25569
2.86356  2.16989  2.53970  3.59794  3.15643  3.64488  2.90000  2.78343
2.68344  2.84430  2.88630  3.23592  3.37952     93 k - - S
          2.68618  4.42225  2.77519  2.73123  3.46354  2.40513  3.72494
3.29354  2.67741  2.69355  4.24690  2.90347  2.73739  3.18146  2.89801
2.37887  2.77519  2.98518  4.58477  3.61503
          0.01601  4.65513  5.05612  0.61958  0.77255  0.45657  1.00363
     31   2.76782  5.22412  1.89614  2.36060  4.61825  3.52952  3.55269
4.10064  2.36026  3.54338  4.17211  2.28209  2.80143  2.68812  2.75978
2.64286  3.00430  3.45654  5.71405  4.23362     94 d - - -
          2.68618  4.42227  2.77519  2.73114  3.46356  2.40515  3.72497
3.29356  2.67743  2.69354  4.24692  2.90349  2.73733  3.18148  2.89803
2.37889  2.77522  2.98510  4.58479  3.61505
          0.04729  3.51229  4.11281  0.84528  0.56114  0.44120  1.03075
     32   2.61080  4.90691  3.08585  2.27589  4.13426  3.18670  3.50772
3.76907  2.37167  3.35750  3.65840  2.57625  2.44467  2.61301  2.85769
2.58048  2.75377  3.16453  5.63442  3.58666     99 e - - H
          2.68618  4.42225  2.77519  2.73123  3.46354  2.40513  3.72494
3.29354  2.67741  2.69355  4.24690  2.90347  2.73739  3.18146  2.89801
2.37887  2.77519  2.98518  4.58477  3.61503
          0.01276  4.76334  5.48568  0.61958  0.77255  0.51176  0.91490
     33   2.52165  5.28883  2.53979  1.91417  4.63118  3.43680  3.05829
3.59636  2.14827  3.58355  4.33023  2.53199  3.81778  2.60656  2.93506
2.51077  2.92324  3.45290  5.72310  4.13088    100 e - - H
```

2.68613 4.42229 2.77523 2.73108 3.46358 2.40513 3.72492
3.29358 2.67744 2.69357 4.24693 2.90350 2.73731 3.18148 2.89802
2.37889 2.77520 2.98522 4.58481 3.61507
0.14602 2.07536 4.57084 0.22179 1.61485 0.46985 0.98109
34 2.46570 5.18502 2.87672 1.64943 4.38313 3.49865 3.65888
3.84315 2.23961 2.83326 4.10985 2.93959 3.96181 2.67255 2.68720
2.61475 2.90684 3.36036 5.51486 4.29161 102 e - - H
2.68618 4.42225 2.77519 2.73123 3.46354 2.40513 3.72494
3.29354 2.67741 2.69355 4.24690 2.90347 2.73739 3.18146 2.89801
2.37887 2.77519 2.98518 4.58477 3.61503
0.01269 4.76895 5.49129 0.61958 0.77255 0.49948 0.93356
35 3.28515 4.53597 5.09214 4.51473 0.94242 4.53591 4.72887
2.62181 4.34454 1.61111 3.00638 4.60870 4.81619 4.43849 4.27783
3.81707 3.58262 2.67684 5.08091 3.80780 103 f - - H
2.68618 4.42225 2.77519 2.73123 3.46354 2.40513 3.72494
3.29354 2.67741 2.69355 4.24690 2.90347 2.73739 3.18146 2.89801
2.37887 2.77519 2.98518 4.58477 3.61503
0.01269 4.76895 5.49129 0.61958 0.77255 0.49948 0.93356
36 2.05703 4.61451 3.07168 2.14871 4.30318 3.43835 3.73106
3.63865 2.48043 3.22017 3.83106 3.09201 3.90627 2.36786 2.94442
2.23455 2.57539 3.10706 5.59396 4.22768 104 a - - H
2.68618 4.42225 2.77519 2.73123 3.46354 2.40513 3.72494
3.29354 2.67741 2.69355 4.24690 2.90347 2.73739 3.18146 2.89801
2.37887 2.77519 2.98518 4.58477 3.61503
0.01499 4.76895 5.05332 0.61958 0.77255 0.49948 0.93356
37 2.52389 5.12820 2.81814 2.21142 4.62450 3.50132 3.67540
4.01698 2.01195 3.48811 4.28302 2.63744 3.90625 2.66028 2.35673
2.34364 2.55402 3.31009 5.71989 4.31203 105 k - - H
2.68618 4.42225 2.77519 2.73123 3.46354 2.40513 3.72494
3.29354 2.67741 2.69355 4.24690 2.90347 2.73739 3.18146 2.89801
2.37887 2.77519 2.98518 4.58477 3.61503
0.08505 4.76668 2.61696 0.61958 0.77255 0.50446 0.92591
38 2.65780 5.18687 2.76976 2.00588 4.45010 3.45548 3.50281
3.49210 1.93567 3.16459 4.17636 2.83230 3.93249 2.62830 2.75736
2.48985 2.68469 3.29604 5.67820 4.15857 106 k - - H
2.68619 4.42226 2.77521 2.73124 3.46355 2.40511 3.72496
3.29355 2.67739 2.69350 4.24691 2.90348 2.73738 3.18147 2.89802
2.37888 2.77521 2.98515 4.58478 3.61504
0.04115 3.76318 4.06853 0.74438 0.64441 0.40411 1.10133
39 2.61305 3.67448 4.67661 4.06904 3.10319 3.88737 4.08474
1.93889 3.87616 2.13069 3.23649 4.05481 4.33494 4.02664 3.91985
3.25377 2.84340 1.32413 4.82501 3.47368 110 v - - T
2.68618 4.42225 2.77519 2.73123 3.46354 2.40513 3.72494
3.29354 2.67741 2.69355 4.24690 2.90347 2.73739 3.18146 2.89801
2.37887 2.77519 2.98518 4.58477 3.61503
0.01279 4.76105 5.48340 0.61958 0.77255 0.51668 0.90757
40 3.22478 4.19702 5.26171 4.67177 3.34429 4.68098 5.01284
2.34485 4.51453 0.65496 2.60735 4.79568 4.92376 4.58000 4.55504
4.01527 3.54570 2.55708 5.35051 4.27067 111 L - - H
2.68618 4.42225 2.77519 2.73123 3.46354 2.40513 3.72494
3.29354 2.67741 2.69355 4.24690 2.90347 2.73739 3.18146 2.89801
2.37887 2.77519 2.98518 4.58477 3.61503
0.04500 4.76105 3.33967 0.61958 0.77255 0.50848 0.91983
41 2.56123 4.81165 2.99709 2.38245 4.19944 2.99400 3.71045
3.93604 2.29921 3.41542 4.22845 2.97936 1.88618 2.65749 2.85802
2.62879 2.75890 3.49717 5.37498 4.19178 112 p - - H

```
          2.68620  4.42216  2.77515  2.73112  3.46363  2.40510  3.72497
3.29361  2.67743  2.69357  4.24697  2.90356  2.73731  3.18152  2.89775
2.37892  2.77523  2.98523  4.58484  3.61514
          0.14624  2.26036  3.45018  1.33110  0.30678  0.47026  0.98041
     42   2.72761  5.02405  2.96895  2.41807  4.59567  3.24958  3.60215
4.03731  1.96699  3.17082  4.17365  2.66007  3.79843  2.16846  2.24317
2.66382  2.72015  3.53941  5.69935  4.14733    122 k - - H
          2.68623  4.42146  2.77525  2.73123  3.46359  2.40503  3.72500
3.29359  2.67746  2.69360  4.24695  2.90352  2.73729  3.18152  2.89794
2.37892  2.77525  2.98524  4.58482  3.61470
          0.05713  2.97058  5.45872  1.04865  0.43142  0.47596  0.97098
     43   2.72672  4.25763  4.24380  3.65723  2.64309  3.76479  3.02004
2.38220  3.51851  2.22987  3.29786  3.53877  4.21124  3.62873  3.42181
3.17278  2.82914  2.37127  4.73830  1.80485    127 y - - H
          2.68618  4.42225  2.77519  2.73123  3.46354  2.40513  3.72494
3.29354  2.67741  2.69355  4.24690  2.90347  2.73739  3.18146  2.89801
2.37887  2.77519  2.98518  4.58477  3.61503
          0.01276  4.76334  5.48568  0.61958  0.77255  0.46985  0.98109
     44   3.18282  4.42928  4.82664  4.28909  0.84824  4.26744  4.16486
2.86552  4.11311  2.56893  3.64270  4.26700  4.61574  4.22105  4.16910
3.27362  3.41244  2.49969  3.95093  2.73633    128 f - - S
          2.68618  4.42225  2.77519  2.73123  3.46354  2.40513  3.72494
3.29354  2.67741  2.69355  4.24690  2.90347  2.73739  3.18146  2.89801
2.37887  2.77519  2.98518  4.58477  3.61503
          0.01261  4.77510  5.49744  0.61958  0.77255  0.48576  0.95510
     45   2.77358  5.32030  3.03776  2.36335  4.66938  3.26612  3.64036
4.14204  1.38218  3.61699  4.36415  2.98808  3.81057  2.66599  2.18933
2.73088  3.01010  3.68365  5.74094  4.30477    129 k - - T
          2.68618  4.42225  2.77519  2.73123  3.46354  2.40513  3.72494
3.29354  2.67741  2.69355  4.24690  2.90347  2.73739  3.18146  2.89801
2.37887  2.77519  2.98518  4.58477  3.61503
          0.01511  4.77510  5.02685  0.61958  0.77255  0.48576  0.95510
     46   2.69891  4.56334  3.39349  2.83196  3.83544  3.62740  1.58443
2.93012  2.78140  2.97061  3.69417  3.26544  4.01377  2.91481  3.22568
2.71610  2.34389  3.03727  5.19036  3.94916    130 h - - T
          2.68618  4.42225  2.77520  2.73123  3.46354  2.40513  3.72495
3.29354  2.67741  2.69355  4.24690  2.90347  2.73740  3.18143  2.89797
2.37887  2.77520  2.98519  4.58477  3.61503
          0.02988  3.94633  4.59444  0.52917  0.88939  0.48232  0.96063
     47   2.73161  4.76041  2.84937  2.50959  4.46224  2.67585  3.72947
3.86477  2.38618  3.38821  4.19080  2.31636  3.86982  2.80813  2.85802
1.93510  2.44882  3.33688  5.66607  4.27661    132 s - - -
          2.68618  4.42225  2.77519  2.73123  3.46354  2.40513  3.72494
3.29354  2.67741  2.69355  4.24690  2.90347  2.73739  3.18146  2.89801
2.37887  2.77519  2.98518  4.58477  3.61503
          0.01269  4.76909  5.49144  0.61958  0.77255  0.47748  0.96849
     48   2.75115  4.65426  3.02618  2.44315  4.48194  3.51490  3.58408
4.04363  2.23110  3.52317  4.29747  1.50801  3.67328  2.64905  2.91988
2.42510  2.98813  3.62940  5.69566  4.25335    133 n - - -
          2.68618  4.42225  2.77519  2.73123  3.46354  2.40513  3.72494
3.29354  2.67741  2.69355  4.24690  2.90347  2.73739  3.18146  2.89801
2.37887  2.77519  2.98518  4.58477  3.61503
          0.01261  4.77510  5.49744  0.61958  0.77255  0.48576  0.95510
     49   2.78814  4.18861  4.70326  4.04014  1.74730  3.90031  4.29381
2.23148  3.89152  2.17145  2.94962  3.86338  4.16248  3.97055  3.92220
3.25707  3.01593  2.01940  3.90770  2.54086    134 f - - H
```

```
          2.68618  4.42225  2.77519  2.73123  3.46354  2.40513  3.72494
3.29354  2.67741  2.69355  4.24690  2.90347  2.73739  3.18146  2.89801
2.37887  2.77519  2.98518  4.58477  3.61503
          0.01261  4.77510  5.49744  0.61958  0.77255  0.48576  0.95510
     50   1.99882  4.89753  3.05751  2.45895  4.47752  3.07121  3.41215
3.98339  2.32321  3.36926  4.22525  2.98323  3.76322  2.65145  2.87590
1.92213  2.57673  3.58326  5.66591  4.27742    135 s - - H
          2.68618  4.42225  2.77519  2.73123  3.46354  2.40513  3.72494
3.29354  2.67741  2.69355  4.24690  2.90347  2.73739  3.18146  2.89801
2.37887  2.77519  2.98518  4.58477  3.61503
          0.01261  4.77510  5.49744  0.61958  0.77255  0.48576  0.95510
     51   2.65849  4.33553  3.62356  3.08829  4.44449  3.17876  4.23986
3.85612  3.11074  3.50052  3.95764  2.97337  4.11064  3.44118  3.51714
0.85613  2.78943  3.44650  5.75842  4.48531    136 s - - H
          2.68618  4.42225  2.77519  2.73123  3.46354  2.40513  3.72494
3.29354  2.67741  2.69355  4.24690  2.90347  2.73739  3.18146  2.89801
2.37887  2.77519  2.98518  4.58477  3.61503
          0.01261  4.77510  5.49744  0.61958  0.77255  0.48576  0.95510
     52   4.19335  5.40451  5.55240  5.23984  0.48884  5.12359  4.40823
3.15517  5.02359  2.19831  3.79151  4.97220  5.37445  4.93042  4.97821
4.53446  4.43439  3.25648  4.50432  2.82775    137 F - - H
          2.68618  4.42225  2.77519  2.73123  3.46354  2.40513  3.72494
3.29354  2.67741  2.69355  4.24690  2.90347  2.73739  3.18146  2.89801
2.37887  2.77519  2.98518  4.58477  3.61503
          0.01261  4.77510  5.49744  0.61958  0.77255  0.48576  0.95510
     53   2.63785  4.21985  4.08713  3.64101  3.11959  3.89626  4.11800
2.24176  3.08611  2.23934  3.26782  3.84558  4.26627  2.95798  3.67610
3.13464  2.72494  1.45191  4.89693  3.50248    138 v - - H
          2.68618  4.42225  2.77519  2.73123  3.46354  2.40513  3.72494
3.29354  2.67741  2.69355  4.24690  2.90347  2.73739  3.18146  2.89801
2.37887  2.77519  2.98518  4.58477  3.61503
          0.01261  4.77510  5.49744  0.61958  0.77255  0.48576  0.95510
     54   3.05231  4.76465  3.63516  3.01905  4.67772  3.71911  3.88998
4.04707  2.26944  3.57259  4.17746  3.45847  4.28318  3.04032  0.82063
3.10986  3.37590  3.72651  5.70948  4.48326    139 r - - H
          2.68622  4.42229  2.77524  2.73127  3.46338  2.40517  3.72499
3.29341  2.67745  2.69359  4.24694  2.90351  2.73734  3.18136  2.89805
2.37884  2.77504  2.98523  4.58481  3.61507
          0.03192  3.60018  5.49744  1.20303  0.35708  0.48576  0.95510
     55   2.86705  5.20024  3.01815  2.48111  4.48803  3.63138  3.79163
3.92745  2.55027  3.06343  3.94415  3.02753  3.98272  1.16987  2.98901
2.77193  3.09722  3.56185  5.66108  4.30130    147 q - - H
          2.68619  4.42226  2.77521  2.73124  3.46343  2.40514  3.72496
3.29355  2.67742  2.69356  4.24691  2.90348  2.73741  3.18147  2.89802
2.37888  2.77517  2.98512  4.58453  3.61504
          0.03238  3.58391  5.49744  0.64156  0.74754  0.48576  0.95510
     56   4.07631  4.98511  5.92025  5.33771  2.89706  5.42509  5.63183
2.67094  5.19745  0.43518  2.57543  5.55364  5.42542  5.03940  5.14064
4.81530  4.27197  2.97069  5.61000  4.61067    150 L - - H
          2.68618  4.42225  2.77519  2.73123  3.46354  2.40513  3.72494
3.29354  2.67741  2.69355  4.24690  2.90347  2.73739  3.18146  2.89801
2.37887  2.77519  2.98518  4.58477  3.61503
          0.01261  4.77510  5.49744  0.61958  0.77255  0.48576  0.95510
     57   2.77193  5.26869  3.11339  2.58986  4.58402  3.63620  2.90552
4.03478  2.43994  3.55122  4.32723  1.31198  4.03757  2.87348  2.64789
2.66477  3.10931  3.60870  5.70769  4.14076    151 n - - H
```

2.68618 4.42225 2.77519 2.73123 3.46354 2.40513 3.72494
3.29354 2.67741 2.69355 4.24690 2.90347 2.73739 3.18146 2.89801
2.37887 2.77519 2.98518 4.58477 3.61503
0.01261 4.77510 5.49744 0.61958 0.77255 0.48576 0.95510
58 2.76525 4.33480 4.00983 3.13246 3.12470 3.76222 4.01276
2.43863 2.90409 1.95226 2.06528 3.43158 4.23911 3.62624 3.51173
2.98822 2.63693 2.48196 4.88981 2.99911 152 l - - H
2.68618 4.42225 2.77519 2.73123 3.46354 2.40513 3.72494
3.29354 2.67741 2.69355 4.24690 2.90347 2.73739 3.18146 2.89801
2.37887 2.77519 2.98518 4.58477 3.61503
0.01501 4.77510 5.04252 0.61958 0.77255 0.48576 0.95510
59 4.87375 5.77334 5.65787 5.60368 1.96056 5.32011 3.47384
4.47628 5.37451 3.61442 4.98553 4.83060 5.56748 5.01018 5.16652
4.72480 5.05980 4.41855 3.22310 0.40895 153 Y - - T
2.68638 4.42180 2.77545 2.73137 3.46264 2.40541 3.72475
3.29336 2.67750 2.69324 4.24680 2.90315 2.73764 3.18147 2.89796
2.37882 2.77538 2.98504 4.58483 3.61498
0.07637 2.66764 5.49508 2.29685 0.10600 0.48246 0.96040
60 2.93879 5.28104 2.51417 2.50046 4.56424 1.20217 3.71362
4.01130 2.72254 3.56860 3.54086 2.95080 4.07599 3.02953 3.20855
2.81076 3.15119 3.64430 5.77102 4.38911 175 g - - T
2.68618 4.42225 2.77519 2.73123 3.46354 2.40513 3.72494
3.29354 2.67741 2.69355 4.24690 2.90347 2.73739 3.18146 2.89801
2.37887 2.77519 2.98518 4.58477 3.61503
0.01261 4.77510 5.49744 0.61958 0.77255 0.48576 0.95510
61 4.25090 5.42072 5.45244 5.17273 0.42768 5.04860 4.09323
3.49866 4.94016 2.85874 4.24311 4.77248 5.32522 4.82524 4.87756
4.42996 4.21076 3.69708 3.81351 2.47019 176 F - - E
2.68626 4.42233 2.77495 2.73116 3.46362 2.40521 3.72503
3.29307 2.67749 2.69363 4.24698 2.90355 2.73748 3.18155 2.89791
2.37883 2.77526 2.98527 4.58386 3.61511
0.05517 3.13295 4.59691 1.42803 0.27415 0.48576 0.95510
62 2.76605 5.23755 3.06872 2.38048 4.46938 3.57570 2.20154
3.74241 2.12590 3.39078 4.19858 2.99322 3.92520 2.65952 2.19590
2.39771 2.87271 3.37356 5.68386 4.21708 185 k - - E
2.68618 4.42225 2.77519 2.73123 3.46354 2.40513 3.72494
3.29354 2.67741 2.69355 4.24690 2.90347 2.73739 3.18146 2.89801
2.37887 2.77519 2.98518 4.58477 3.61503
0.01269 4.76909 5.49144 0.61958 0.77255 0.49915 0.93406
63 3.29337 5.50030 3.75117 3.04281 4.92461 3.98205 3.85902
3.93434 0.85298 3.73065 3.73322 3.43270 4.32988 3.03338 1.90005
3.21974 3.46491 3.94517 5.79422 4.58720 186 k - - E
2.68638 4.42217 2.77512 2.73117 3.46376 2.40518 3.72493
3.29353 2.67709 2.69372 4.24660 2.90294 2.73766 3.18165 2.89783
2.37883 2.77525 2.98518 4.58495 3.61535
0.39143 1.51943 2.25306 1.76426 0.18791 0.49915 0.93406
64 2.63222 4.26379 3.16965 3.15500 3.42076 3.72745 3.89609
2.25280 2.98483 2.15936 3.33046 3.52563 4.01697 3.20896 3.30478
2.86251 2.75754 1.79726 4.97597 3.58666 194 v - - -
2.68618 4.42225 2.77519 2.73123 3.46354 2.40513 3.72494
3.29354 2.67741 2.69355 4.24690 2.90347 2.73739 3.18146 2.89801
2.37887 2.77519 2.98518 4.58477 3.61503
0.04415 4.66267 3.38891 0.61958 0.77255 0.69219 0.69410
65 2.61228 4.09519 2.71260 2.31917 4.31299 3.50335 3.33388
3.71660 2.16082 3.37205 4.15854 2.61890 3.28050 2.63803 2.64025
2.32783 2.73449 3.21214 5.47560 4.11107 195 k - - -

```
          2.68618  4.42225  2.77519  2.73123  3.46354  2.40513  3.72494
3.29354  2.67741  2.69355  4.24690  2.90347  2.73739  3.18146  2.89801
2.37887  2.77519  2.98518  4.58477  3.61503
          0.30004  4.63568  1.38824  0.61958  0.77255  0.47611  0.97074
     66   2.59760  5.12187  2.44852  2.33576  4.45062  3.04353  3.07353
3.69451  2.34497  3.16099  4.20318  2.68615  3.48248  2.59958  2.51606
2.48998  2.81209  3.31538  5.60113  4.14597    196 e - - -
          2.68618  4.42225  2.77519  2.73123  3.46354  2.40513  3.72494
3.29354  2.67741  2.69355  4.24690  2.90347  2.73739  3.18146  2.89801
2.37887  2.77519  2.98518  4.58477  3.61503
          0.05880  4.47454  3.08555  0.61958  0.77255  0.94162  0.49429
     67   2.54032  4.72211  2.66715  2.50779  3.99212  3.17167  3.47281
3.27394  2.47608  2.56222  3.88785  2.83925  3.32048  2.82826  2.90262
2.52053  2.66933  2.71935  4.89151  3.98214    197 k - - S
          2.68618  4.42225  2.77519  2.73123  3.46354  2.40513  3.72494
3.29354  2.67741  2.69355  4.24690  2.90347  2.73739  3.18146  2.89801
2.37887  2.77519  2.98518  4.58477  3.61503
          0.03775  4.43526  3.68101  0.61958  0.77255  0.98581  0.46703
     68   2.62560  4.78021  2.61360  2.34448  3.75278  3.18372  3.28161
3.82104  2.31237  3.18137  4.12170  2.59052  3.28704  2.50403  2.74824
2.39145  2.78181  3.36180  5.55414  3.97361    198 k - - S
          2.68618  4.42225  2.77519  2.73123  3.46354  2.40513  3.72494
3.29354  2.67741  2.69355  4.24690  2.90347  2.73739  3.18146  2.89801
2.37887  2.77519  2.98518  4.58477  3.61503
          0.10629  4.41562  2.42193  0.61958  0.77255  1.00453  0.45605
     69   2.56743  5.05123  2.72893  2.21390  4.02796  3.26676  3.21155
3.44540  2.34082  3.10233  4.10327  2.63268  3.42528  2.58477  2.76379
2.34314  2.84166  3.26245  5.34296  4.12619    199 e - - B
          2.68563  4.42306  2.77533  2.73156  3.46426  2.40507  3.72502
3.29401  2.67825  2.69390  4.24682  2.90101  2.73756  3.18180  2.89874
2.37792  2.77491  2.98549  4.58616  3.61597
          0.85701  0.58074  4.12987  2.57782  0.07898  1.07922  0.41530
     70   2.46485  4.56101  2.85550  2.34562  4.16109  3.01373  3.60443
3.49192  2.23819  3.08449  3.94948  2.73713  3.46143  2.70305  2.74201
2.30402  2.70233  3.18811  5.45366  4.06401    247 k - - T
          2.68618  4.42225  2.77519  2.73123  3.46354  2.40513  3.72494
3.29354  2.67741  2.69355  4.24690  2.90347  2.73739  3.18146  2.89801
2.37887  2.77519  2.98518  4.58477  3.61503
          0.01997  4.31937  5.04172  0.61958  0.77255  0.89946  0.52220
     71   2.62797  4.19901  2.55359  2.23598  4.38633  2.87646  3.57561
3.83035  2.18271  3.31067  3.83761  2.71657  3.59943  2.59532  2.75295
2.45288  2.71990  3.34878  5.49546  4.14386    248 k - - T
          2.68618  4.42225  2.77519  2.73123  3.46354  2.40513  3.72494
3.29354  2.67741  2.69355  4.24690  2.90347  2.73739  3.18146  2.89801
2.37887  2.77519  2.98518  4.58477  3.61503
          0.01832  4.40449  5.12684  0.61958  0.77255  0.96239  0.48124
     72   2.60215  4.83029  2.58843  2.32802  4.11354  2.96150  3.66445
3.61563  2.25276  2.99336  3.72178  2.78503  3.56964  2.53908  2.83795
2.37631  2.78474  3.18522  5.19433  4.11559    249 k - - B
          2.68618  4.42225  2.77519  2.73123  3.46354  2.40513  3.72494
3.29354  2.67741  2.69355  4.24690  2.90347  2.73739  3.18146  2.89801
2.37887  2.77519  2.98518  4.58477  3.61503
          0.01794  4.42558  5.14793  0.61958  0.77255  0.92352  0.50603
     73   2.60062  4.95610  2.67343  2.31512  4.22317  3.21081  3.25337
3.37131  2.23755  3.12496  3.26061  2.80565  3.16725  2.61458  2.76650
2.55745  2.84801  3.35659  5.44670  3.87852    250 k - - -
```

2.68618 4.42225 2.77519 2.73123 3.46354 2.40513 3.72494
3.29354 2.67741 2.69355 4.24690 2.90347 2.73739 3.18146 2.89801
2.37887 2.77519 2.98518 4.58477 3.61503
0.01743 4.45394 5.17629 0.61958 0.77255 0.89477 0.52544
74 2.50601 4.56255 2.76927 2.32734 4.01776 3.05394 3.39482
3.44580 2.17319 3.13510 4.04468 2.82148 3.63065 2.73876 2.82626
2.44592 2.64785 3.04299 5.51210 3.76311 251 k - - -
2.68618 4.42225 2.77519 2.73123 3.46354 2.40513 3.72494
3.29354 2.67741 2.69355 4.24690 2.90347 2.73739 3.18146 2.89801
2.37887 2.77519 2.98518 4.58477 3.61503
0.21662 4.48188 1.69582 0.61958 0.77255 0.73409 0.65382
75 2.38345 5.04926 2.79728 2.18016 4.24028 2.95082 3.64994
3.69003 2.33150 3.16457 3.99101 2.84554 2.98329 2.73524 2.82007
2.35452 2.78650 3.17106 5.51214 4.13235 252 e - - T
2.68618 4.42225 2.77519 2.73123 3.46354 2.40513 3.72494
3.29354 2.67741 2.69355 4.24690 2.90347 2.73739 3.18146 2.89801
2.37887 2.77519 2.98518 4.58477 3.61503
0.01874 4.38209 5.10444 0.61958 0.77255 0.81228 0.58671
76 2.56238 5.06293 2.47265 2.24493 4.51396 3.33508 3.18260
3.81881 2.14283 3.35975 4.10658 2.63673 3.33126 2.60209 2.77560
2.49284 2.67522 3.39968 5.61693 4.17552 253 k - - T
2.68618 4.42225 2.77519 2.73123 3.46354 2.40513 3.72494
3.29354 2.67741 2.69355 4.24690 2.90347 2.73739 3.18146 2.89801
2.37887 2.77519 2.98518 4.58477 3.61503
0.01701 4.47825 5.20060 0.61958 0.77255 0.91921 0.50888
77 2.52981 4.38534 3.37348 2.76538 3.55697 3.42153 3.57894
2.52355 2.80504 2.41366 3.26023 3.30839 3.75355 3.10229 2.65609
2.52181 2.83396 2.36979 4.83922 3.28820 254 v - - S
2.68618 4.42225 2.77519 2.73123 3.46354 2.40513 3.72494
3.29354 2.67741 2.69355 4.24690 2.90347 2.73739 3.18146 2.89801
2.37887 2.77519 2.98518 4.58477 3.61503
0.01687 4.48675 5.20909 0.61958 0.77255 0.88284 0.53376
78 2.68103 4.51932 3.15612 2.62705 3.68835 3.51835 3.29797
2.88395 2.76351 2.74647 3.45961 3.25858 3.95482 3.03716 3.12403
2.47287 2.87080 2.81582 2.05805 3.84348 255 w - - E
2.68623 4.42110 2.77528 2.73131 3.46323 2.40520 3.72499
3.29361 2.67746 2.69362 4.24698 2.90352 2.73727 3.18153 2.89806
2.37884 2.77510 2.98522 4.58458 3.61492
0.20554 1.71243 5.22770 0.65160 0.73649 0.55574 0.85249
79 2.70649 4.62010 3.08814 1.54599 4.07877 3.32056 3.64387
3.48476 2.45723 3.26484 4.03866 3.03266 3.94624 2.76755 2.80942
2.46033 2.76160 3.10169 5.52778 3.68066 258 e - - E
2.68618 4.42225 2.77519 2.73123 3.46354 2.40513 3.72494
3.29354 2.67741 2.69355 4.24690 2.90347 2.73739 3.18146 2.89801
2.37887 2.77519 2.98518 4.58477 3.61503
0.03806 4.64810 3.58403 0.61958 0.77255 0.38731 1.13594
80 4.71815 5.67021 5.61508 5.53017 0.62135 5.24234 3.81424
4.33827 4.89769 3.53183 4.86742 4.79070 5.49712 4.96064 5.10242
4.64041 4.91062 4.26710 2.88556 1.40395 259 F - - E
2.68618 4.42225 2.77519 2.73123 3.46354 2.40513 3.72494
3.29354 2.67741 2.69355 4.24690 2.90347 2.73739 3.18146 2.89801
2.37887 2.77519 2.98518 4.58477 3.61503
0.01301 4.74424 5.46659 0.61958 0.77255 0.51589 0.90874
81 2.35855 4.67719 3.16939 2.53033 3.67973 3.46014 3.33824
3.51438 2.27686 3.22944 3.81690 3.11520 3.96665 2.62323 2.40451
2.30377 2.74171 2.89451 5.34581 3.27670 260 k - - E

```
        2.68618  4.42225  2.77519  2.73123  3.46354  2.40513  3.72494
3.29354  2.67741  2.69355  4.24690  2.90347  2.73739  3.18146  2.89801
2.37887  2.77519  2.98518  4.58477  3.61503
        0.01288  4.75453  5.47688  0.61958  0.77255  0.53058  0.88737
     82  2.94284  4.97614  3.01480  2.64278  4.51152  3.47194  1.29408
3.98615  2.68145  3.54419  4.35223  2.12896  4.09410  2.98011  3.10558
2.85201  3.16382  3.62442  5.71948  4.26752    261 h - - E
        2.68622  4.42234  2.77528  2.73132  3.46363  2.40515  3.72486
3.29363  2.67749  2.69359  4.24696  2.90347  2.73719  3.18154  2.89810
2.37847  2.77522  2.98527  4.58486  3.61512
        0.16642  1.90294  5.47688  0.67491  0.71172  0.53058  0.88737
     83  2.73675  4.90885  2.61780  2.04781  4.61083  2.65799  3.68368
3.70429  2.24056  3.57566  4.28924  2.87875  2.28812  2.73819  2.89971
2.62422  2.90741  3.62465  5.71135  4.30519    264 e - - S
        2.68618  4.42225  2.77519  2.73123  3.46354  2.40513  3.72494
3.29354  2.67741  2.69355  4.24690  2.90347  2.73739  3.18146  2.89801
2.37887  2.77519  2.98518  4.58477  3.61503
        0.01288  4.75453  5.47688  0.61958  0.77255  0.53058  0.88737
     84  2.73392  4.17113  3.04244  2.65711  3.27060  3.00589  3.39955
3.35505  2.43730  2.71616  3.41148  2.43692  4.03328  2.98116  2.97385
2.39767  2.91949  3.10486  4.82035  2.90645    265 s - - S
        2.68618  4.42225  2.77519  2.73123  3.46354  2.40513  3.72494
3.29354  2.67741  2.69355  4.24690  2.90347  2.73739  3.18146  2.89801
2.37887  2.77519  2.98518  4.58477  3.61503
        0.01288  4.75453  5.47688  0.61958  0.77255  0.53058  0.88737
     85  3.85075  5.18198  5.45294  5.01060  0.62819  4.99772  4.48965
2.86670  4.81875  1.86989  3.58603  4.89276  5.21294  4.77047  4.80509
4.36124  4.15095  3.10463  4.44782  3.01281    266 F - - -
        2.68618  4.42225  2.77519  2.73123  3.46354  2.40513  3.72494
3.29354  2.67741  2.69355  4.24690  2.90347  2.73739  3.18146  2.89801
2.37887  2.77519  2.98518  4.58477  3.61503
        0.01288  4.75453  5.47688  0.61958  0.77255  0.53058  0.88737
     86  2.72515  4.61889  3.09788  2.55990  4.19954  3.20176  3.30324
3.57015  2.07654  2.64200  4.09994  3.03053  3.98177  2.21464  2.37235
2.68082  2.81559  3.06146  5.57445  3.91238    267 k - - -
        2.68618  4.42225  2.77519  2.73123  3.46354  2.40513  3.72494
3.29354  2.67741  2.69355  4.24690  2.90347  2.73739  3.18146  2.89801
2.37887  2.77519  2.98518  4.58477  3.61503
        0.01288  4.75453  5.47688  0.61958  0.77255  0.45872  0.99992
     87  2.62405  5.19533  3.18382  2.58028  4.55470  3.65298  3.35214
4.02673  1.66933  3.49672  4.11702  3.13333  3.78969  2.74177  1.59485
2.81844  3.09339  3.66016  5.71078  4.35231    268 r - - X
        2.68618  4.42225  2.77519  2.73123  3.46354  2.40513  3.72494
3.29354  2.67741  2.69355  4.24690  2.90347  2.73739  3.18146  2.89801
2.37887  2.77519  2.98518  4.58477  3.61503
        0.01261  4.77510  5.49744  0.61958  0.77255  0.48576  0.95510
     88  2.78884  4.62889  2.28103  2.27208  4.56697  1.63107  3.56963
4.06909  2.54060  3.57444  4.33093  2.60465  3.98731  2.88253  3.04158
2.74989  3.02492  3.65673  5.43505  4.32856    269 g - - X
        2.68618  4.42225  2.77519  2.73123  3.46354  2.40513  3.72494
3.29354  2.67741  2.69355  4.24690  2.90347  2.73739  3.18146  2.89801
2.37887  2.77519  2.98518  4.58477  3.61503
        0.01261  4.77510  5.49744  0.61958  0.77255  0.48576  0.95510
     89  2.61864  4.61492  2.35622  2.42517  4.49454  3.21629  3.23748
4.12079  2.09752  3.54409  4.28254  2.70833  3.44686  2.37265  2.39519
2.52714  2.95852  3.61495  5.72760  4.08126    270 k - - X
```

```
          2.68623  4.42174  2.77524  2.73128  3.46337  2.40517  3.72499
3.29359  2.67745  2.69340  4.24647  2.90351  2.73742  3.18151  2.89793
2.37891  2.77513  2.98523  4.58482  3.61508
          0.15500  3.43635  2.19460  1.19241  0.36167  0.48576  0.95510
     90   2.72854  5.09246  3.00281  2.18591  4.27896  3.19513  3.66919
3.59545  2.08100  3.25104  4.11160  3.01164  2.27026  2.75690  2.65629
2.58958  2.84368  3.26482  5.61560  4.04350    278 k - - X
          2.68626  4.42233  2.77518  2.73132  3.46345  2.40518  3.72503
3.29343  2.67739  2.69344  4.24648  2.90355  2.73737  3.18136  2.89796
2.37880  2.77528  2.98527  4.58485  3.61511
          0.06203  2.92330  5.05247  1.38170  0.28922  0.37091  1.17153
     91   2.46201  4.95464  2.14698  2.07394  4.44341  3.35410  2.75322
4.00199  2.30157  3.47637  4.32646  2.81879  3.95704  2.55127  2.93343
2.58136  2.84150  3.62921  5.01746  4.19547    288 e - - X
          2.68618  4.42225  2.77519  2.73123  3.46354  2.40513  3.72494
3.29354  2.67741  2.69355  4.24690  2.90347  2.73739  3.18146  2.89801
2.37887  2.77519  2.98518  4.58477  3.61503
          0.01263  4.77349  5.49583  0.61958  0.77255  0.48351  0.95872
     92   2.62920  4.57429  2.97625  2.83927  3.67171  3.24879  3.93106
3.14826  2.81418  1.58408  3.55513  3.17539  4.09494  2.97353  3.15081
2.39021  2.91878  2.81398  4.86903  3.81690    289 l - - X
          2.68619  4.42221  2.77512  2.73118  3.46359  2.40513  3.72500
3.29356  2.67746  2.69358  4.24695  2.90348  2.73743  3.18145  2.89806
2.37886  2.77509  2.98512  4.58482  3.61508
          0.09080  2.49246  5.49744  0.70363  0.68277  0.48576  0.95510
     93   3.16490  4.21835  5.08180  4.49032  3.37446  4.50526  4.83886
2.37467  4.32920  0.68948  2.84126  4.60781  4.78870  4.33807  4.39072
3.76491  3.46003  2.44736  5.25218  4.14060    293 L - - X
          2.68618  4.42225  2.77519  2.73123  3.46354  2.40513  3.72494
3.29354  2.67741  2.69355  4.24690  2.90347  2.73739  3.18146  2.89801
2.37887  2.77519  2.98518  4.58477  3.61503
          0.01261  4.77510  5.49744  0.61958  0.77255  0.48576  0.95510
     94   2.62392  3.34572  2.71477  2.36036  3.56833  3.43945  3.25022
3.56350  2.12195  3.14112  4.14133  2.92761  3.48497  2.80073  2.73701
2.49087  2.85315  3.02694  4.97823  3.92426    294 k - - X
          2.68618  4.42225  2.77519  2.73123  3.46354  2.40513  3.72494
3.29354  2.67741  2.69355  4.24690  2.90347  2.73739  3.18146  2.89801
2.37887  2.77519  2.98518  4.58477  3.61503
          0.01261  4.77510  5.49744  0.61958  0.77255  0.48576  0.95510
     95   2.75089  5.11076  2.92237  2.32212  4.08978  3.51847  3.44764
3.99534  2.04393  3.00886  3.67403  2.37185  3.96228  2.46358  2.62939
2.41638  2.75506  3.61914  5.57151  3.71575    295 k - - X
          2.68618  4.42225  2.77519  2.73123  3.46354  2.40513  3.72494
3.29354  2.67741  2.69355  4.24690  2.90347  2.73739  3.18146  2.89801
2.37887  2.77519  2.98518  4.58477  3.61503
          0.01261  4.77510  5.49744  0.61958  0.77255  0.48576  0.95510
     96   3.31186  4.31658  5.25694  4.69208  3.49212  4.63960  5.05694
0.83685  4.42293  2.03441  2.85392  4.77440  4.92984  4.68951  4.60880
3.98431  3.43303  1.87883  5.48094  4.32264    296 i - - X
          2.68618  4.42225  2.77519  2.73123  3.46354  2.40513  3.72494
3.29354  2.67741  2.69355  4.24690  2.90347  2.73739  3.18146  2.89801
2.37887  2.77519  2.98518  4.58477  3.61503
          0.01261  4.77510  5.49744  0.61958  0.77255  0.48576  0.95510
     97   2.80426  4.96093  3.08816  2.43721  4.54269  3.59083  3.49093
3.97202  1.47286  3.54829  4.28414  3.02570  3.93549  2.30845  2.38971
2.73575  2.76952  3.34139  5.37822  4.31086    297 k - - X
```

```
          2.68618  4.42225  2.77519  2.73123  3.46354  2.40513  3.72494
3.29354  2.67741  2.69355  4.24690  2.90347  2.73739  3.18146  2.89801
2.37887  2.77519  2.98518  4.58477  3.61503
          0.01261  4.77510  5.49744  0.61958  0.77255  0.48576  0.95510
     98   2.94019  5.13198  3.33937  2.67671  4.54825  3.68452  3.85568
3.75574  2.33662  3.45549  4.20921  3.24527  3.51832  2.86356  1.09907
2.94409  2.83441  3.61074  5.65798  4.35121    298 r - - X
          2.68618  4.42225  2.77519  2.73123  3.46354  2.40513  3.72494
3.29354  2.67741  2.69355  4.24690  2.90347  2.73739  3.18146  2.89801
2.37887  2.77519  2.98518  4.58477  3.61503
          0.01261  4.77510  5.49744  0.61958  0.77255  0.48576  0.95510
     99   2.64804  5.09433  3.07285  2.47684  4.61188  3.24379  3.72361
4.08165  1.68473  3.40943  4.28225  2.82101  3.85877  2.73327  1.83626
2.64899  2.99276  3.57934  5.71090  4.31944    299 k - - X
          2.68618  4.42225  2.77519  2.73123  3.46354  2.40513  3.72494
3.29354  2.67741  2.69355  4.24690  2.90347  2.73739  3.18146  2.89801
2.37887  2.77519  2.98518  4.58477  3.61503
          0.00851  4.77099        *  0.61958  0.77255  0.00000        *
//
HMMER3/f [3.3 | Nov 2019]
NAME  IQ
ACC   PF00612.28
DESC  IQ calmodulin-binding motif
LENG  21
ALPH  amino
RF    no
MM    no
CONS  yes
CS    yes
MAP   yes
DATE  Wed Apr 22 17:23:20 2020
NSEQ  245
EFFN  245.000000
CKSUM 1020059171
GA    20.30 11.10
TC    20.30 11.10
NC    20.20 11.00
STATS LOCAL MSV       -6.8777  0.74075
STATS LOCAL VITERBI   -6.8796  0.74075
STATS LOCAL FORWARD   -3.8537  0.74075
HMM          A        C        D        E        F        G        H
I        K        L        M        N        P        Q        R        S
T        V        W        Y
            m->m     m->i     m->d     i->m     i->i     d->m     d->d
  COMPO   2.17490  4.03729  4.42889  3.74545  3.31791  3.30675  3.66516
2.47384  2.44972  2.54488  3.74549  3.94083  5.83063  2.57560  1.90027
3.04939  3.18801  2.85543  3.77915  3.10076
          2.68618  4.42225  2.77519  2.73123  3.46354  2.40513  3.72494
3.29354  2.67741  2.69355  4.24690  2.90347  2.73739  3.18146  2.89801
2.37887  2.77519  2.98518  4.58477  3.61503
          0.02226  9.08738  3.82124  0.61958  0.77255  0.00000        *
      1   3.40768  8.59478  3.44826  2.22110  4.72253  3.27308  2.63016
3.15438  2.64119  2.59480  3.57664  3.26023  4.68315  2.97385  1.76138
3.25205  2.09442  3.08597  9.02746  4.35825      1 r - - H
          2.68618  4.42225  2.77519  2.73123  3.46354  2.40513  3.72494
3.29354  2.67741  2.69355  4.24690  2.90347  2.73739  3.18146  2.89801
2.37887  2.77519  2.98518  4.58477  3.61503
          0.00017  9.06529  9.78764  0.61958  0.77255  0.21419  1.64607
```

```
      2   2.45737  4.42048  2.52982  2.53144  7.96220  3.98656  3.79012
4.44308  1.54410  4.07551  3.77117  3.18284  4.05108  2.87295  1.73255
2.52429  4.06143  3.59501  4.51300  4.78967      2 k - - H
          2.68618  4.42225  2.77519  2.73123  3.46354  2.40513  3.72494
3.29354  2.67741  2.69355  4.24690  2.90347  2.73739  3.18146  2.89801
2.37887  2.77519  2.98518  4.58477  3.61503
          0.00017  9.08431  9.80665  0.61958  0.77255  0.29284  1.37098
      3   0.72047  3.39068  4.87511  4.29401  4.11053  3.53002  5.08801
2.87840  3.24411  3.25824  7.44523  3.80053  7.35317  4.89322  3.71983
1.70799  4.85215  4.36106  5.00991  4.69394      3 a - - H
          2.68618  4.42225  2.77519  2.73123  3.46354  2.40513  3.72494
3.29354  2.67741  2.69355  4.24690  2.90347  2.73739  3.18146  2.89801
2.37887  2.77519  2.98518  4.58477  3.61503
          0.00017  9.08738  9.80973  0.61958  0.77255  0.48576  0.95510
      4   0.70957  4.27015  8.51912  7.91600  3.88180  7.73352  8.09330
1.54836  7.70959  4.41837  3.37622  7.90216  8.08738  7.84676  7.72173
3.66440  2.91531  2.03371  8.57493  7.39537      4 a - - H
          2.68618  4.42225  2.77519  2.73123  3.46354  2.40513  3.72494
3.29354  2.67741  2.69355  4.24690  2.90347  2.73739  3.18146  2.89801
2.37887  2.77519  2.98518  4.58477  3.61503
          0.00017  9.08738  9.80973  0.61958  0.77255  0.48576  0.95510
      5   3.37539  4.11803  8.29115  7.67490  4.91943  7.48558  7.81977
1.07836  3.52113  2.21102  3.23796  4.55093  4.77092  4.70520  4.92344
3.48011  1.75156  1.70769  8.30990  7.13423      5 i - - H
          2.68618  4.42225  2.77519  2.73123  3.46354  2.40513  3.72494
3.29354  2.67741  2.69355  4.24690  2.90347  2.73739  3.18146  2.89801
2.37887  2.77519  2.98518  4.58477  3.61503
          0.00017  9.08738  9.80973  0.61958  0.77255  0.48576  0.95510
      6   3.31307  3.64171  7.84677  4.77646  4.17946  3.87403  4.50232
2.05141  1.44651  2.20368  4.02901  3.46532  7.77828  3.83897  2.73206
3.80559  2.25051  2.09759  4.87730  4.34038      6 k - - H
          2.68618  4.42225  2.77519  2.73123  3.46354  2.40513  3.72494
3.29354  2.67741  2.69355  4.24690  2.90347  2.73739  3.18146  2.89801
2.37887  2.77519  2.98518  4.58477  3.61503
          0.00017  9.08738  9.80973  0.61958  0.77255  0.48576  0.95510
      7   4.36932  4.44905  9.59784  9.08572  2.79051  9.02343  9.58301
0.59618  8.98750  1.40549  3.30516  9.19687  9.21773  9.10717  9.05104
8.42608  3.76647  2.86627  9.82611  8.69853      7 I - - H
          2.68618  4.42225  2.77519  2.73123  3.46354  2.40513  3.72494
3.29354  2.67741  2.69355  4.24690  2.90347  2.73739  3.18146  2.89801
2.37887  2.77519  2.98518  4.58477  3.61503
          0.00017  9.08738  9.80973  0.61958  0.77255  0.48576  0.95510
      8   4.60313  8.77615  9.35112  9.22832  9.73551  4.48514  9.61407
4.65487  8.46385  8.88579  9.61923  8.47176  8.44540  0.07012  4.38101
4.39393  7.45462  4.59601 10.96884 10.01298      8 Q - - H
          2.68618  4.42225  2.77519  2.73123  3.46354  2.40513  3.72494
3.29354  2.67741  2.69355  4.24690  2.90347  2.73739  3.18146  2.89801
2.37887  2.77519  2.98518  4.58477  3.61503
          0.00017  9.08738  9.80973  0.61958  0.77255  0.48576  0.95510
      9   1.22579  3.42675  6.41120  5.85431  7.84675  5.09676  7.10159
7.30671  1.96774  4.84605  4.81474  4.08259  7.30372  4.67962  1.72250
1.40452  3.25086  6.90921  4.94594  4.83389      9 a - - H
          2.68618  4.42225  2.77519  2.73123  3.46354  2.40513  3.72494
3.29354  2.67741  2.69355  4.24690  2.90347  2.73739  3.18146  2.89801
2.37887  2.77519  2.98518  4.58477  3.61503
          0.00017  9.08738  9.80973  0.61958  0.77255  0.48576  0.95510
```

```
     10   1.98329  4.08375  4.78821  4.75635  2.49507  3.21201  2.52472
2.97866  3.88489  2.66237  3.80972  3.41044  7.60422  2.92759  2.59868
3.47792  3.37694  2.35044  2.63452  2.67520      10 a - - H
          2.68618  4.42225  2.77519  2.73123  3.46354  2.40513  3.72494
3.29354  2.67741  2.69355  4.24690  2.90347  2.73739  3.18146  2.89801
2.37887  2.77519  2.98518  4.58477  3.61503
          0.00017  9.08738  9.80973  0.61958  0.77255  0.48576  0.95510
     11   2.82416  2.44791  8.29464  7.67815  2.06301  4.54283  7.82043
1.97173  7.45939  2.94099  3.97120  7.65368  7.84963  5.20778  7.46190
3.72999  3.98526  2.26938  1.45263  2.14508      11 w - - H
          2.68618  4.42225  2.77519  2.73123  3.46354  2.40513  3.72494
3.29354  2.67741  2.69355  4.24690  2.90347  2.73739  3.18146  2.89801
2.37887  2.77519  2.98518  4.58477  3.61503
          0.00017  9.08738  9.80973  0.61958  0.77255  0.48576  0.95510
     12   8.17136 10.00617  9.45241  7.87784  9.78265  8.55151  8.08472
8.88809  2.44943  3.51549  4.11624  8.04135  8.79620  7.20842  0.14618
8.12474  8.15362  8.65552  9.99940  9.06150      12 R - - H
          2.68618  4.42225  2.77519  2.73123  3.46354  2.40513  3.72494
3.29354  2.67741  2.69355  4.24690  2.90347  2.73739  3.18146  2.89801
2.37887  2.77519  2.98518  4.58477  3.61503
          0.00017  9.08738  9.80973  0.61958  0.77255  0.48576  0.95510
     13   2.14823  4.43570  6.81008  6.24821  7.28648  0.68522  4.76209
4.54106  3.41993  4.50506  2.53019  3.91367  7.48056  4.94394  2.42302
2.76640  3.44210  4.69316  8.66614  7.39196      13 G - - H
          2.68618  4.42225  2.77519  2.73123  3.46354  2.40513  3.72494
3.29354  2.67741  2.69355  4.24690  2.90347  2.73739  3.18146  2.89801
2.37887  2.77519  2.98518  4.58477  3.61503
          0.00017  9.08738  9.80973  0.61958  0.77255  0.48576  0.95510
     14   4.52150  4.29354  7.72653  4.04529  2.01940  4.69356  2.06766
4.47903  3.22360  3.36469  3.14794  3.21984  7.75420  3.42063  3.67549
4.30173  3.85255  3.30625  2.37582  1.21166      14 y - - H
          2.68618  4.42225  2.77519  2.73123  3.46354  2.40513  3.72494
3.29354  2.67741  2.69355  4.24690  2.90347  2.73739  3.18146  2.89801
2.37887  2.77519  2.98518  4.58477  3.61503
          0.00017  9.08738  9.80973  0.61958  0.77255  0.48576  0.95510
     15   4.17500  7.76559  5.32409  7.15448  3.89338  4.71949  4.26697
2.84559  2.22790  0.78717  3.07965  3.80793  7.75728  3.05272  2.62664
4.14617  3.98950  2.57692  4.20024  7.18433      15 l - - H
          2.68618  4.42225  2.77519  2.73123  3.46354  2.40513  3.72494
3.29354  2.67741  2.69355  4.24690  2.90347  2.73739  3.18146  2.89801
2.37887  2.77519  2.98518  4.58477  3.61503
          0.00017  9.08738  9.80973  0.61958  0.77255  0.48576  0.95510
     16   0.98531  3.56937  3.94822  3.87174  4.20358  4.74005  4.72746
3.51238  4.47936  2.59242  3.86731  4.63866  7.60663  2.72495  4.64306
2.90269  2.49041  1.99440  5.29317  3.69992      16 a - - H
          2.68618  4.42225  2.77519  2.73123  3.46354  2.40513  3.72494
3.29354  2.67741  2.69355  4.24690  2.90347  2.73739  3.18146  2.89801
2.37887  2.77519  2.98518  4.58477  3.61503
          0.00017  9.08738  9.80973  0.61958  0.77255  0.48576  0.95510
     17   4.53803  8.19670  6.70379  4.89694  5.12042  4.86672  3.97285
5.01387  2.57855  4.51968  3.72616  6.63610  7.43732  3.25133  0.28646
4.59534  4.54272  4.49863  8.73233  4.99566      17 R - - H
          2.68618  4.42225  2.77519  2.73123  3.46354  2.40513  3.72494
3.29354  2.67741  2.69355  4.24690  2.90347  2.73739  3.18146  2.89801
2.37887  2.77519  2.98518  4.58477  3.61503
          0.00017  9.08738  9.80973  0.61958  0.77255  0.48576  0.95510
```

```
     18   3.62148  3.56082  4.37631  4.04610  7.95763  6.88022  4.18705
4.64089  1.07386  3.13491  4.46725  3.45901  7.27366  2.51657  1.37466
2.82428  3.46381  3.51238  9.04718  5.20825     18 k - - H
          2.68618  4.42225  2.77519  2.73123  3.46354  2.40513  3.72494
3.29354  2.67741  2.69355  4.24690  2.90347  2.73739  3.18146  2.89801
2.37887  2.77519  2.98518  4.58477  3.61503
          0.00017  9.08738  9.80973  0.61958  0.77255  0.48576  0.95510
     19   2.45518  5.07438  3.19240  2.11137  7.96485  3.97005  2.66886
3.88940  2.11547  3.05081  7.65903  2.81167  7.27226  2.46538  1.87389
3.06602  3.63857  3.59931  4.05029  2.96777     19 r - - H
          2.68618  4.42225  2.77519  2.73123  3.46354  2.40513  3.72494
3.29354  2.67741  2.69355  4.24690  2.90347  2.73739  3.18146  2.89801
2.37887  2.77519  2.98518  4.58477  3.61503
          0.00017  9.08738  9.80973  0.61958  0.77255  0.48576  0.95510
     20   3.12330  4.02963  5.01441  3.59288  1.43264  4.69006  3.20286
3.25037  7.45707  2.09940  3.91154  7.65220  5.04168  4.64817  7.46069
4.00320  3.79298  2.84592  4.68156  1.17624     20 y - - H
          2.68618  4.42225  2.77519  2.73123  3.46354  2.40513  3.72494
3.29354  2.67741  2.69355  4.24690  2.90347  2.73739  3.18146  2.89801
2.37887  2.77519  2.98518  4.58477  3.61503
          0.00960  9.08738  4.66252  0.61958  0.77255  0.48576  0.95510
     21   3.95265  4.32328  3.39516  2.80865  5.23220  3.68185  3.15887
3.23339  1.39731  2.52277  5.19195  3.57730  4.35938  2.48114  1.55248
3.84468  3.66168  3.33449  9.03887  4.67763     21 k - - H
          2.68618  4.42225  2.77519  2.73123  3.46354  2.40513  3.72494
3.29354  2.67741  2.69355  4.24690  2.90347  2.73739  3.18146  2.89801
2.37887  2.77519  2.98518  4.58477  3.61503
          0.00011  9.07789        *  0.61958  0.77255  0.00000        *
//
HMMER3/f [3.3 | Nov 2019]
NAME  JmjC
ACC   PF02373.23
DESC  JmjC domain, hydroxylase
LENG  114
ALPH  amino
RF    no
MM    no
CONS  yes
CS    yes
MAP   yes
DATE  Tue Apr 21 05:31:54 2020
NSEQ  30
EFFN  4.295654
CKSUM 1364841153
GA    21.30 21.30
TC    21.30 21.30
NC    21.20 21.20
STATS LOCAL MSV       -9.5772  0.71455
STATS LOCAL VITERBI  -10.1706  0.71455
STATS LOCAL FORWARD   -3.8654  0.71455
HMM          A        C        D        E        F        G        H
I        K        L        M        N        P        Q        R        S
T        V        W        Y
            m->m     m->i     m->d     i->m     i->i     d->m     d->d
  COMPO   2.67006  4.01114  3.09865  2.69496  3.08263  2.92458  3.32189
2.87733  2.74083  2.61159  3.66413  2.98699  3.24816  2.98782  3.14660
2.63338  2.81979  2.62634  3.84551  3.30567
```

2.68618 4.42225 2.77519 2.73123 3.46354 2.40513 3.72494
3.29354 2.67741 2.69355 4.24690 2.90347 2.73739 3.18146 2.89801
2.37887 2.77519 2.98518 4.58477 3.61503
0.00808 5.21799 5.94034 0.61958 0.77255 0.00000 *
1 3.00220 4.74133 3.85094 3.28372 2.87749 3.97360 3.31782
3.24407 2.71841 2.52043 3.22818 3.69723 4.35337 2.23761 2.88664
3.21596 2.82936 3.01664 2.21933 2.41299 1 w - - E
2.68618 4.42225 2.77519 2.73123 3.46354 2.40513 3.72494
3.29354 2.67741 2.69355 4.24690 2.90347 2.73739 3.18146 2.89801
2.37887 2.77519 2.98518 4.58477 3.61503
0.00808 5.21799 5.94034 0.61958 0.77255 0.48576 0.95510
2 2.67167 3.28112 2.76502 4.34119 2.79442 4.22119 4.55402
2.38862 4.14307 1.31589 3.09987 4.36065 4.58273 4.28708 4.17663
3.53074 3.28550 1.80534 5.06522 3.88849 2 l - - E
2.68618 4.42225 2.77519 2.73123 3.46354 2.40513 3.72494
3.29354 2.67741 2.69355 4.24690 2.90347 2.73739 3.18146 2.89801
2.37887 2.77519 2.98518 4.58477 3.61503
0.00808 5.21799 5.94034 0.61958 0.77255 0.48576 0.95510
3 3.03284 4.53660 4.39911 3.81979 2.99124 3.15551 4.39739
2.99231 3.71426 2.25926 3.65284 3.32311 4.48765 3.94743 3.92363
2.31922 3.26605 2.38454 5.10808 1.28807 3 y - - E
2.68618 4.42225 2.77519 2.73123 3.46354 2.40513 3.72494
3.29354 2.67741 2.69355 4.24690 2.90347 2.73739 3.18146 2.89801
2.37887 2.77519 2.98518 4.58477 3.61503
0.00808 5.21799 5.94034 0.61958 0.77255 0.48576 0.95510
4 2.31595 3.60464 3.39223 4.34995 2.64125 4.19957 4.53264
1.93350 4.14303 1.86270 2.27115 4.35081 4.56285 4.28097 4.16371
3.50985 2.94220 1.90613 5.03579 3.85901 4 l - - E
2.68618 4.42225 2.77519 2.73123 3.46354 2.40513 3.72494
3.29354 2.67741 2.69355 4.24690 2.90347 2.73739 3.18146 2.89801
2.37887 2.77519 2.98518 4.58477 3.61503
0.00808 5.21799 5.94034 0.61958 0.77255 0.48576 0.95510
5 2.58975 3.19131 4.12786 3.65771 5.00939 0.82038 4.74627
4.44152 2.25904 4.07320 4.87505 3.94165 4.45112 3.95125 3.98447
2.14445 3.45710 3.93276 6.29730 5.04704 5 g - - E
2.68618 4.42225 2.77519 2.73123 3.46354 2.40513 3.72494
3.29354 2.67741 2.69355 4.24690 2.90347 2.73739 3.18146 2.89801
2.37887 2.77519 2.98518 4.58477 3.61503
0.00808 5.21799 5.94034 0.61958 0.77255 0.48576 0.95510
6 2.97726 5.32146 2.81575 2.74148 4.60364 3.77880 3.97429
4.04743 2.47136 3.29583 1.97805 2.77425 2.48620 2.57965 3.21895
2.25765 3.20853 2.66760 5.79381 4.42447 6 m - - -
2.68618 4.42225 2.77519 2.73123 3.46354 2.40513 3.72494
3.29354 2.67741 2.69355 4.24690 2.90347 2.73739 3.18146 2.89801
2.37887 2.77519 2.98518 4.58477 3.61503
0.00808 5.21799 5.94034 0.61958 0.77255 0.48576 0.95510
7 2.73938 2.81081 3.67339 2.67596 4.02216 3.91741 3.33797
3.03573 2.47651 2.75718 3.29785 2.50118 2.47462 3.40640 3.48609
2.87767 3.22490 2.80935 2.83662 3.49592 7 p - - T
2.68618 4.42225 2.77519 2.73123 3.46354 2.40513 3.72494
3.29354 2.67741 2.69355 4.24690 2.90347 2.73739 3.18146 2.89801
2.37887 2.77519 2.98518 4.58477 3.61503
0.00808 5.21799 5.94034 0.61958 0.77255 0.48576 0.95510
8 2.98002 5.32031 2.83347 2.74531 2.34525 2.32264 3.97692
4.04451 1.95113 2.29322 4.37879 2.95946 4.17354 3.09947 2.70261
2.58243 3.21123 3.67631 5.79304 4.42490 8 k - - T

```
          2.68618  4.42225  2.77519  2.73123  3.46354  2.40513  3.72494
3.29354  2.67741  2.69355  4.24690  2.90347  2.73739  3.18146  2.89801
2.37887  2.77519  2.98518  4.58477  3.61503
          0.00808  5.21799  5.94034  0.61958  0.77255  0.48576  0.95510
      9   2.39571  2.57634  5.14758  4.85430  5.32653  3.75707  5.54373
4.74643  4.75480  4.46494  5.26981  4.50810  4.58460  4.93570  4.89753
0.87026  1.28451  4.08853  6.71259  5.60512      9 s - - -
          2.68618  4.42225  2.77519  2.73123  3.46354  2.40513  3.72494
3.29354  2.67741  2.69355  4.24690  2.90347  2.73739  3.18146  2.89801
2.37887  2.77519  2.98518  4.58477  3.61503
          0.00808  5.21799  5.94034  0.61958  0.77255  0.48576  0.95510
     10   3.00168  3.54866  3.90597  3.33772  3.84050  3.03842  4.24378
2.53568  3.28876  2.91641  3.81039  3.73595  4.36679  2.90990  2.53037
2.43180  1.76188  2.97826  3.25050  2.66667     10 t - - E
          2.68618  4.42225  2.77519  2.73123  3.46354  2.40513  3.72494
3.29354  2.67741  2.69355  4.24690  2.90347  2.73739  3.18146  2.89801
2.37887  2.77519  2.98518  4.58477  3.61503
          0.00808  5.21799  5.94034  0.61958  0.77255  0.48576  0.95510
     11   2.99046  5.13529  2.84358  2.86446  1.94237  3.83075  4.04089
3.76694  2.51305  2.91331  4.21160  3.37698  4.22173  2.61239  3.31206
2.73887  1.60321  3.45917  5.64846  4.32581     11 t - - E
          2.68618  4.42225  2.77519  2.73123  3.46354  2.40513  3.72494
3.29354  2.67741  2.69355  4.24690  2.90347  2.73739  3.18146  2.89801
2.37887  2.77519  2.98518  4.58477  3.61503
          0.00808  5.21799  5.94034  0.61958  0.77255  0.48576  0.95510
     12   2.40849  2.58386  2.49516  2.79666  3.69074  2.43548  4.00832
3.94234  2.79281  3.52405  4.32187  3.32125  2.07394  3.14510  2.78695
2.60060  2.93095  3.59616  5.74599  4.39628     12 p - - E
          2.68618  4.42225  2.77519  2.73123  3.46354  2.40513  3.72494
3.29354  2.67741  2.69355  4.24690  2.90347  2.73739  3.18146  2.89801
2.37887  2.77519  2.98518  4.58477  3.61503
          0.00808  5.21799  5.94034  0.61958  0.77255  0.48576  0.95510
     13   2.53454  4.41767  4.90617  4.29768  2.49671  2.63958  4.51168
2.41532  4.10132  2.34611  3.53413  4.32179  4.55342  4.24777  4.13962
3.49705  3.26216  2.31882  1.45635  3.27022     13 w - - E
          2.68618  4.42225  2.77519  2.73123  3.46354  2.40513  3.72494
3.29354  2.67741  2.69355  4.24690  2.90347  2.73739  3.18146  2.89801
2.37887  2.77519  2.98518  4.58477  3.61503
          0.00808  5.21799  5.94034  0.61958  0.77255  0.48576  0.95510
     14   3.29337  5.49842  3.68412  3.08153  4.81073  4.04946  0.91520
3.13716  2.44195  3.74572  4.56896  3.56349  4.42477  2.80284  2.57795
2.86324  3.49913  3.87936  5.90337  4.62620     14 h - - E
          2.68618  4.42225  2.77519  2.73123  3.46354  2.40513  3.72494
3.29354  2.67741  2.69355  4.24690  2.90347  2.73739  3.18146  2.89801
2.37887  2.77519  2.98518  4.58477  3.61503
          0.00808  5.21799  5.94034  0.61958  0.77255  0.48576  0.95510
     15   2.51627  4.52457  4.35559  3.02075  3.34346  4.09425  4.38807
1.94729  3.67180  2.01682  2.96203  4.02732  4.46551  3.09803  2.82557
3.00067  2.36403  2.06737  5.13200  3.93434     15 i - - -
          2.68618  4.42225  2.77519  2.73123  3.46354  2.40513  3.72494
3.29354  2.67741  2.69355  4.24690  2.90347  2.73739  3.18146  2.89801
2.37887  2.77519  2.98518  4.58477  3.61503
          0.00808  5.21799  5.94034  0.61958  0.77255  0.48576  0.95510
     16   3.97276  6.77380  1.12409  0.93166  6.03654  2.31133  4.69083
5.64528  3.88903  5.06564  5.97828  3.45982  4.71965  3.87592  4.62719
3.78310  4.29473  5.14701  7.20770  5.56599     16 e - - -
```

```
          2.68618  4.42225  2.77519  2.73123  3.46354  2.40513  3.72494
3.29354  2.67741  2.69355  4.24690  2.90347  2.73739  3.18146  2.89801
2.37887  2.77519  2.98518  4.58477  3.61503
          0.00808  5.21799  5.94034  0.61958  0.77255  0.48576  0.95510
     17   2.97286  5.47133  1.88177  2.50679  4.81120  3.74796  3.93596
4.29358  2.25299  3.77439  3.30626  2.42774  3.15903  2.74549  3.16487
2.18945  2.73551  2.93001  5.90710  4.49919      17 d - - G
          2.68618  4.42225  2.77519  2.73123  3.46354  2.40513  3.72494
3.29354  2.67741  2.69355  4.24690  2.90347  2.73739  3.18146  2.89801
2.37887  2.77519  2.98518  4.58477  3.61503
          0.00808  5.21799  5.94034  0.61958  0.77255  0.48576  0.95510
     18   2.97900  3.45072  3.35407  2.32331  3.66074  3.11405  2.73729
3.89730  2.79142  2.85018  2.69248  2.41292  3.34668  2.18638  3.26041
2.75581  3.21029  3.56141  5.71672  3.45952      18 q - - G
          2.68618  4.42225  2.77519  2.73123  3.46354  2.40513  3.72494
3.29354  2.67741  2.69355  4.24690  2.90347  2.73739  3.18146  2.89801
2.37887  2.77519  2.98518  4.58477  3.61503
          0.00808  5.21799  5.94034  0.61958  0.77255  0.48576  0.95510
     19   2.49776  4.42403  2.29747  2.42373  4.79694  2.19579  3.04519
4.27654  2.39260  3.30390  4.50403  2.31871  4.14257  3.03663  2.88900
2.64824  3.20329  3.84671  4.03446  3.57257      19 g - - G
          2.68618  4.42225  2.77519  2.73123  3.46354  2.40513  3.72494
3.29354  2.67741  2.69355  4.24690  2.90347  2.73739  3.18146  2.89801
2.37887  2.77519  2.98518  4.58477  3.61503
          0.00808  5.21799  5.94034  0.61958  0.77255  0.48576  0.95510
     20   2.60756  4.60344  4.12701  3.55299  2.85353  3.10326  4.32118
3.07518  3.10955  1.45818  3.22821  3.88511  3.28454  3.75220  3.76817
2.21376  2.32432  2.86679  5.20214  3.99040      20 l - - -
          2.68618  4.42225  2.77519  2.73123  3.46354  2.40513  3.72494
3.29354  2.67741  2.69355  4.24690  2.90347  2.73739  3.18146  2.89801
2.37887  2.77519  2.98518  4.58477  3.61503
          0.00808  5.21799  5.94034  0.61958  0.77255  0.48576  0.95510
     21   2.73918  3.00573  4.26575  3.68712  2.78281  2.86209  2.59265
3.01448  3.59793  2.27411  3.66607  3.97303  3.50560  3.84986  3.84454
2.14051  3.24353  2.81247  5.15658  2.00937      21 y - - E
          2.68618  4.42225  2.77519  2.73123  3.46354  2.40513  3.72494
3.29354  2.67741  2.69355  4.24690  2.90347  2.73739  3.18146  2.89801
2.37887  2.77519  2.98518  4.58477  3.61503
          0.23606  5.21799  1.58549  0.61958  0.77255  0.48576  0.95510
     22   2.37062  4.84833  3.93918  3.55673  4.69727  2.80210  4.64326
4.15955  3.59984  3.82196  4.65272  3.82435  4.32132  3.89579  3.96382
0.65394  3.32505  3.69765  6.05416  3.23352      22 S - - E
          2.68626  4.42233  2.77528  2.73131  3.46362  2.40521  3.72503
3.29362  2.67749  2.69322  4.24698  2.90304  2.73748  3.18087  2.89809
2.37895  2.77528  2.98527  4.58485  3.61511
          0.18032  2.58485  2.41249  0.95311  0.48701  0.25506  1.49106
     23   1.68766  4.73030  5.38231  4.80728  3.85267  4.70871  5.11221
1.42889  4.64113  2.07702  2.34172  4.85625  5.02420  4.78999  4.69097
4.04547  3.65435  1.63431  5.56377  4.39324      26 i - - E
          2.68618  4.42225  2.77519  2.73123  3.46354  2.40513  3.72494
3.29354  2.67741  2.69355  4.24690  2.90347  2.73739  3.18146  2.89801
2.37887  2.77519  2.98518  4.58477  3.61503
          0.00868  5.14666  5.86901  0.61958  0.77255  0.37099  1.17135
     24   2.99992  3.75427  3.23198  3.25867  3.20624  3.96527  3.37035
3.26119  3.21818  2.57575  3.85611  1.53706  4.34566  2.97763  3.58554
3.20553  3.23161  2.27261  2.61831  4.08661      27 n - - E
```

```
          2.68618  4.42225  2.77519  2.73123  3.46354  2.40513  3.72494
3.29354  2.67741  2.69355  4.24690  2.90347  2.73739  3.18146  2.89801
2.37887  2.77519  2.98518  4.58477  3.61503
          0.00808  5.21799  5.94034  0.61958  0.77255  0.48576  0.95510
     25   3.08546  4.50913  4.73341  4.14507  3.03198  4.20937  4.45499
2.35947  3.99056  2.69542  3.62713  3.06665  4.57449  4.16873  4.10047
2.29634  3.31728  2.44243  2.52270  1.41105     28 y - - E
          2.68618  4.42225  2.77519  2.73123  3.46354  2.40513  3.72494
3.29354  2.67741  2.69355  4.24690  2.90347  2.73739  3.18146  2.89801
2.37887  2.77519  2.98518  4.58477  3.61503
          0.00808  5.21799  5.94034  0.61958  0.77255  0.48576  0.95510
     26   2.74478  5.23168  2.81351  2.79163  4.48466  3.79934  2.22695
3.51257  2.78712  1.92003  4.29803  2.36068  4.19001  2.80280  3.25706
2.57530  2.68211  3.05950  5.72392  4.37662     29 l - - E
          2.68618  4.42225  2.77519  2.73123  3.46354  2.40513  3.72494
3.29354  2.67741  2.69355  4.24690  2.90347  2.73739  3.18146  2.89801
2.37887  2.77519  2.98518  4.58477  3.61503
          0.00808  5.21799  5.94034  0.61958  0.77255  0.48576  0.95510
     27   2.99721  4.92573  2.90535  3.04741  4.09853  3.89884  1.53867
2.25729  3.02191  2.38340  4.01666  2.74497  4.28440  3.35543  3.06543
3.12301  3.22868  2.76277  5.47610  4.20051     30 h - - E
          2.68618  4.42225  2.77519  2.73123  3.46354  2.40513  3.72494
3.29354  2.67741  2.69355  4.24690  2.90347  2.73739  3.18146  2.89801
2.37887  2.77519  2.98518  4.58477  3.61503
          0.00808  5.21799  5.94034  0.61958  0.77255  0.48576  0.95510
     28   3.02018  4.41831  4.84012  3.70261  1.95525  2.35620  4.49885
2.41073  4.04991  2.11749  3.02626  4.28702  4.53829  3.10178  4.10879
3.47797  2.73817  2.29605  3.76928  2.55059     31 f - - E
          2.68618  4.42225  2.77519  2.73123  3.46354  2.40513  3.72494
3.29354  2.67741  2.69355  4.24690  2.90347  2.73739  3.18146  2.89801
2.37887  2.77519  2.98518  4.58477  3.61503
          0.00808  5.21799  5.94034  0.61958  0.77255  0.48576  0.95510
     29   2.60755  3.77315  2.74093  2.69948  4.72823  1.52897  3.95250
4.19344  2.41921  3.70526  4.46173  3.24194  2.74446  2.81121  2.53311
2.95349  3.20764  3.30407  5.86363  4.47195     32 g - - E
          2.68618  4.42225  2.77519  2.73123  3.46354  2.40513  3.72494
3.29354  2.67741  2.69355  4.24690  2.90347  2.73739  3.18146  2.89801
2.37887  2.77519  2.98518  4.58477  3.61503
          0.00808  5.21799  5.94034  0.61958  0.77255  0.48576  0.95510
     30   1.47885  5.54729  2.69328  2.24431  4.89502  1.79012  4.03442
4.37535  2.80242  3.86744  4.62080  3.26922  4.21767  3.14280  2.74752
2.65759  3.31146  3.94653  6.01108  4.60019     33 a - - S
          2.68618  4.42225  2.77519  2.73123  3.46354  2.40513  3.72494
3.29354  2.67741  2.69355  4.24690  2.90347  2.73739  3.18146  2.89801
2.37887  2.77519  2.98518  4.58477  3.61503
          0.00808  5.21799  5.94034  0.61958  0.77255  0.48576  0.95510
     31   2.83141  5.46011  2.40446  2.40223  3.38277  3.21712  2.65467
4.27505  2.39286  3.29131  4.50327  3.22063  2.17913  2.72083  2.98259
2.62396  2.77474  3.22575  5.89850  4.49333     34 p - - -
          2.68618  4.42225  2.77519  2.73123  3.46354  2.40513  3.72494
3.29354  2.67741  2.69355  4.24690  2.90347  2.73739  3.18146  2.89801
2.37887  2.77519  2.98518  4.58477  3.61503
          0.00808  5.21799  5.94034  0.61958  0.77255  0.48576  0.95510
     32   2.57474  2.63855  2.46108  2.74909  4.70123  3.79418  3.97924
4.15152  1.27187  3.08711  4.45011  3.28424  4.18662  3.09338  2.59611
2.99379  3.23721  3.76439  5.84947  4.47608     35 k - - E
```

```
          2.68618  4.42225  2.77519  2.73123  3.46354  2.40513  3.72494
3.29354  2.67741  2.69355  4.24690  2.90347  2.73739  3.18146  2.89801
2.37887  2.77519  2.98518  4.58477  3.61503
          0.05546  5.21799  3.02559  0.61958  0.77255  0.48576  0.95510
     33   2.98170  3.21744  4.04959  2.88015  3.72136  4.00745  4.27836
2.35367  3.01466  2.65870  3.70822  3.82575  4.38322  2.62245  2.90104
3.07218  2.21904  1.86823  3.34995  3.36977     36 v - - E
          2.68618  4.42225  2.77519  2.73123  3.46354  2.40513  3.72494
3.29354  2.67741  2.69355  4.24690  2.90347  2.73739  3.18146  2.89801
2.37887  2.77519  2.98518  4.58477  3.61503
          0.00847  5.17101  5.89335  0.61958  0.77255  0.63500  0.75489
     34   3.88785  5.71238  3.97528  2.70265  4.47869  4.40462  4.84680
4.54580  3.77867  4.11733  5.09023  4.21876  2.90408  4.24193  4.06301
3.98753  4.19441  4.27192  0.44168  4.47020     37 W - - E
          2.68618  4.42225  2.77519  2.73123  3.46354  2.40513  3.72494
3.29354  2.67741  2.69355  4.24690  2.90347  2.73739  3.18146  2.89801
2.37887  2.77519  2.98518  4.58477  3.61503
          0.00847  5.17101  5.89335  0.61958  0.77255  0.63500  0.75489
     35   3.25793  3.06581  4.86076  4.29576  1.93936  4.35443  4.40756
3.10498  2.50392  2.24756  3.77422  4.35996  4.71220  4.28710  4.21913
3.65908  3.48840  2.92645  4.83157  1.19923     38 y - - E
          2.68618  4.42225  2.77519  2.73123  3.46354  2.40513  3.72494
3.29354  2.67741  2.69355  4.24690  2.90347  2.73739  3.18146  2.89801
2.37887  2.77519  2.98518  4.58477  3.61503
          0.00847  5.17101  5.89335  0.61958  0.77255  0.40262  1.10434
     36   2.20623  3.47233  4.89548  4.28725  2.96650  2.30015  4.51609
1.85086  4.09319  2.01184  3.53487  4.31678  4.55014  4.24253  4.13530
2.49800  3.25841  1.99691  5.03898  3.86066     39 i - - E
          2.68618  4.42225  2.77519  2.73123  3.46354  2.40513  3.72494
3.29354  2.67741  2.69355  4.24690  2.90347  2.73739  3.18146  2.89801
2.37887  2.77519  2.98518  4.58477  3.61503
          0.00808  5.21799  5.94034  0.61958  0.77255  0.48576  0.95510
     37   3.12910  4.49822  5.04521  4.44130  2.44283  4.31819  4.66232
1.37128  4.24525  2.62682  3.11151  4.46124  4.67369  4.39113  3.17503
3.63219  2.73756  1.41990  5.16477  3.98733     40 i - - E
          2.68618  4.42225  2.77519  2.73123  3.46354  2.40513  3.72494
3.29354  2.67741  2.69355  4.24690  2.90347  2.73739  3.18146  2.89801
2.37887  2.77519  2.98518  4.58477  3.61503
          0.00808  5.21799  5.94034  0.61958  0.77255  0.48576  0.95510
     38   2.78694  5.51684  2.78131  2.13903  4.83102  3.44178  3.49299
4.30201  2.79742  3.81043  4.57242  3.26733  1.19969  3.13694  3.29426
2.75950  3.30050  3.89230  5.96829  3.37499     41 p - - -
          2.68618  4.42225  2.77519  2.73123  3.46354  2.40513  3.72494
3.29354  2.67741  2.69355  4.24690  2.90347  2.73739  3.18146  2.89801
2.37887  2.77519  2.98518  4.58477  3.61503
          0.00808  5.21799  5.94034  0.61958  0.77255  0.48576  0.95510
     39   2.69808  5.23958  3.34369  2.19626  3.37519  2.60930  3.99703
3.92245  2.54024  2.95540  3.62995  3.31294  2.66040  2.93180  3.25273
2.27225  2.36633  3.58114  5.72994  2.97278     42 e - - G
          2.68618  4.42225  2.77519  2.73123  3.46354  2.40513  3.72494
3.29354  2.67741  2.69355  4.24690  2.90347  2.73739  3.18146  2.89801
2.37887  2.77519  2.98518  4.58477  3.61503
          0.00808  5.21799  5.94034  0.61958  0.77255  0.48576  0.95510
     40   2.41008  4.01919  2.59872  1.70091  4.74119  3.28716  3.29300
3.31131  2.45160  2.64092  4.46884  3.09013  4.14977  2.80057  3.18036
2.43042  3.20434  3.21111  5.86959  4.47444     43 e - - G
```

```
          2.68618  4.42225  2.77519  2.73123  3.46354  2.40513  3.72494
3.29354  2.67741  2.69355  4.24690  2.90347  2.73739  3.18146  2.89801
2.37887  2.77519  2.98518  4.58477  3.61503
          0.00808  5.21799  5.94034  0.61958  0.77255  0.48576  0.95510
     41   2.50277  4.45721  2.36397  2.30834  4.81759  3.43381  2.46117
4.30144  2.15427  3.77950  4.51697  2.94892  4.14022  2.87422  2.75130
2.93566  2.70055  3.86455  5.90997  2.39111     44 k - - G
          2.68618  4.42225  2.77519  2.73123  3.46354  2.40513  3.72494
3.29354  2.67741  2.69355  4.24690  2.90347  2.73739  3.18146  2.89801
2.37887  2.77519  2.98518  4.58477  3.61503
          0.00808  5.21799  5.94034  0.61958  0.77255  0.48576  0.95510
     42   2.08105  4.31327  3.27013  2.33722  4.67579  2.77251  3.95894
4.13210  1.97019  3.11847  4.42668  3.25350  2.74666  3.07148  2.74694
2.47187  2.93934  3.74129  3.25251  4.45100     45 k - - H
          2.68618  4.42225  2.77519  2.73123  3.46354  2.40513  3.72494
3.29354  2.67741  2.69355  4.24690  2.90347  2.73739  3.18146  2.89801
2.37887  2.77519  2.98518  4.58477  3.61503
          0.00808  5.21799  5.94034  0.61958  0.77255  0.48576  0.95510
     43   2.62116  5.47931  2.39245  2.04288  4.04121  2.80724  3.12812
4.30821  2.40891  2.93431  4.52030  2.87767  3.38903  2.70837  2.66599
2.59893  2.89691  3.13819  5.91275  4.50255     46 e - - H
          2.68618  4.42225  2.77519  2.73123  3.46354  2.40513  3.72494
3.29354  2.67741  2.69355  4.24690  2.90347  2.73739  3.18146  2.89801
2.37887  2.77519  2.98518  4.58477  3.61503
          0.00808  5.21799  5.94034  0.61958  0.77255  0.48576  0.95510
     44   2.58506  5.48071  3.22385  2.10653  4.82482  3.52458  3.12752
4.30951  1.80183  3.78521  4.52178  3.21571  4.14107  2.51987  2.39839
2.09705  2.65539  3.46000  5.91363  4.50411     47 k - - H
          2.68618  4.42225  2.77519  2.73123  3.46354  2.40513  3.72494
3.29354  2.67741  2.69355  4.24690  2.90347  2.73739  3.18146  2.89801
2.37887  2.77519  2.98518  4.58477  3.61503
          0.00808  5.21799  5.94034  0.61958  0.77255  0.48576  0.95510
     45   2.26444  4.43981  4.74098  3.16411  1.74487  4.16319  4.48250
2.39523  3.97639  1.57943  3.13482  4.24078  4.52901  4.15289  3.09142
3.46313  3.25497  2.68780  5.05644  3.17871     48 l - - H
          2.68618  4.42225  2.77519  2.73123  3.46354  2.40513  3.72494
3.29354  2.67741  2.69355  4.24690  2.90347  2.73739  3.18146  2.89801
2.37887  2.77519  2.98518  4.58477  3.61503
          0.00808  5.21799  5.94034  0.61958  0.77255  0.48576  0.95510
     46   2.66745  5.37371  3.27227  1.30912  4.67355  3.77046  3.96356
3.52667  2.30694  2.55240  4.42690  2.36454  4.16353  3.07716  3.20045
2.76395  3.21075  3.73980  5.83414  3.84747     49 e - - H
          2.68609  4.42248  2.77542  2.73146  3.46292  2.40535  3.72407
3.29377  2.67704  2.69288  4.24712  2.90369  2.73762  3.18110  2.89775
2.37909  2.77542  2.98541  4.58499  3.61526
          0.24494  1.68802  3.43071  0.86605  0.54578  0.48576  0.95510
     47   2.56249  5.44202  2.46985  2.37390  3.81030  2.70935  3.92455
2.80216  1.75463  3.74251  4.48565  2.73753  4.12951  3.02458  2.63003
2.62774  3.18950  3.36118  5.88147  4.47738     53 k - - H
          2.68618  4.42225  2.77519  2.73123  3.46354  2.40513  3.72494
3.29354  2.67741  2.69355  4.24690  2.90347  2.73739  3.18146  2.89801
2.37887  2.77519  2.98518  4.58477  3.61503
          0.00833  5.18781  5.91015  0.61958  0.77255  0.58494  0.81450
     48   2.38408  4.48178  3.42011  3.85067  2.20206  4.09668  4.39769
2.34243  3.06189  2.02949  3.14109  4.06805  4.46654  3.09111  3.92320
2.82654  3.23037  1.79267  5.09240  3.89941     54 v - - H
```

2.68618 4.42225 2.77519 2.73123 3.46354 2.40513 3.72494
3.29354 2.67741 2.69355 4.24690 2.90347 2.73739 3.18146 2.89801
2.37887 2.77519 2.98518 4.58477 3.61503
0.00833 5.18781 5.91015 0.61958 0.77255 0.58494 0.81450
49 2.46902 3.25840 4.84947 3.18850 3.49163 4.15997 4.48718
2.40615 4.05239 1.71151 2.25726 3.15299 4.52486 4.20575 4.10230
3.46600 2.64530 2.20853 3.29033 3.13732 55 l - - H
2.68618 4.42225 2.77519 2.73123 3.46354 2.40513 3.72494
3.29354 2.67741 2.69355 4.24690 2.90347 2.73739 3.18146 2.89801
2.37887 2.77519 2.98518 4.58477 3.61503
0.00833 5.18781 5.91015 0.61958 0.77255 0.58494 0.81450
50 2.63436 5.46622 2.65162 2.51013 4.81037 2.75456 3.31998
4.29566 2.19155 2.88387 4.50714 2.44612 4.12533 2.71974 2.37183
2.19075 3.18897 3.85637 5.89944 3.41011 56 s - - H
2.68618 4.42225 2.77519 2.73123 3.46354 2.40513 3.72494
3.29354 2.67741 2.69355 4.24690 2.90347 2.73739 3.18146 2.89801
2.37887 2.77519 2.98518 4.58477 3.61503
0.00833 5.18781 5.91015 0.61958 0.77255 0.42839 1.05428
51 2.73953 5.48409 2.05612 2.06153 4.82927 3.48251 3.93347
4.31538 2.01597 3.78940 4.52479 3.21326 2.51921 2.41964 2.92408
2.70881 2.87039 3.28902 5.91665 4.50551 57 k - - H
2.68618 4.42225 2.77519 2.73123 3.46354 2.40513 3.72494
3.29354 2.67741 2.69355 4.24690 2.90347 2.73739 3.18146 2.89801
2.37887 2.77519 2.98518 4.58477 3.61503
0.00808 5.21799 5.94034 0.61958 0.77255 0.48576 0.95510
52 2.60677 5.39975 2.84399 2.37884 3.51556 3.76122 2.53110
3.43405 2.37586 3.04497 3.38288 3.00192 4.15383 2.42304 2.48783
2.65749 2.89953 3.04904 5.85323 3.96447 58 k - - H
2.68626 4.42282 2.77512 2.73041 3.46256 2.40519 3.72551
3.29371 2.67798 2.69305 4.24747 2.90376 2.73648 3.18167 2.89858
2.37872 2.77576 2.98575 4.58534 3.61437
0.80322 1.09532 1.52471 1.17872 0.36770 0.48576 0.95510
53 2.86946 5.13509 2.73084 2.28131 3.41978 3.20917 3.88680
3.82014 2.67051 2.38441 3.49385 2.21824 4.07776 2.43130 3.14192
2.62620 3.10075 3.47740 3.56956 3.52378 65 n - - H
2.68618 4.42225 2.77519 2.73123 3.46354 2.40513 3.72494
3.29354 2.67741 2.69355 4.24690 2.90347 2.73739 3.18146 2.89801
2.37887 2.77519 2.98518 4.58477 3.61503
0.24366 4.97585 1.56377 0.61958 0.77255 0.49139 0.94617
54 2.46469 4.95269 3.32589 2.76681 2.86715 2.42424 3.14639
3.00648 2.54926 3.19864 4.03167 2.98624 3.31898 3.09892 2.88872
2.23399 2.88660 3.04072 5.47525 3.43426 66 s - - H
2.68618 4.42225 2.77519 2.73123 3.46354 2.40513 3.72494
3.29354 2.67741 2.69355 4.24690 2.90347 2.73739 3.18146 2.89801
2.37887 2.77519 2.98518 4.58477 3.61503
0.15881 4.93541 1.96858 0.61958 0.77255 0.75386 0.63591
55 2.83481 5.29979 2.80264 2.23552 4.62615 2.49108 3.80483
3.62776 2.23213 3.06966 4.34542 2.74913 3.38555 2.39687 2.62019
2.80607 3.06597 2.93501 3.65959 4.34449 67 k - - C
2.68618 4.42225 2.77519 2.73123 3.46354 2.40513 3.72494
3.29354 2.67741 2.69355 4.24690 2.90347 2.73739 3.18146 2.89801
2.37887 2.77519 2.98518 4.58477 3.61503
0.01115 4.89746 5.61980 0.61958 0.77255 0.91767 0.50991
56 2.71971 5.35364 3.11598 2.01556 4.69153 2.95052 3.05671
3.15664 2.19672 3.65574 4.39624 2.38466 4.03020 2.67319 2.54193
2.40218 3.09171 3.16403 5.79052 4.38421 68 e - - C

2.68618 4.42225 2.77519 2.73123 3.46354 2.40513 3.72494
3.29354 2.67741 2.69355 4.24690 2.90347 2.73739 3.18146 2.89801
2.37887 2.77519 2.98518 4.58477 3.61503
0.01046 4.96151 5.68386 0.61958 0.77255 1.06982 0.42018
57 2.50883 2.38406 2.68222 2.60256 4.57751 3.65600 3.01658
4.03478 2.35917 3.56079 4.32610 3.14255 4.05028 1.85584 3.08436
2.85226 2.49749 3.64184 5.73142 4.34777 69 q - - S
2.68618 4.42225 2.77519 2.73123 3.46354 2.40513 3.72494
3.29354 2.67741 2.69355 4.24690 2.90347 2.73739 3.18146 2.89801
2.37887 2.77519 2.98518 4.58477 3.61503
0.01046 4.96151 5.68386 0.61958 0.77255 1.06982 0.42018
58 2.87213 5.31830 2.96364 2.58560 3.55634 3.65080 3.84191
4.10645 2.29818 3.61229 4.36678 2.81620 1.66254 2.69689 2.63653
2.25398 3.10327 3.69599 5.76537 4.37165 70 p - - T
2.68618 4.42225 2.77519 2.73123 3.46354 2.40513 3.72494
3.29354 2.67741 2.69355 4.24690 2.90347 2.73739 3.18146 2.89801
2.37887 2.77519 2.98518 4.58477 3.61503
0.01046 4.96151 5.68386 0.61958 0.77255 0.79827 0.59804
59 2.23111 5.39226 1.86848 2.25251 4.72082 2.62408 3.87888
2.69002 2.43664 3.69083 4.43917 2.69965 4.07888 2.98232 3.12125
2.59424 3.14793 3.77436 5.83466 4.42999 71 d - - T
2.68618 4.42225 2.77519 2.73123 3.46354 2.40513 3.72494
3.29354 2.67741 2.69355 4.24690 2.90347 2.73739 3.18146 2.89801
2.37887 2.77519 2.98518 4.58477 3.61503
0.05721 5.02987 3.01468 0.61958 0.77255 0.95661 0.48482
60 2.88442 4.73712 3.57914 2.51188 2.34714 2.93397 2.30077
3.26918 2.70573 2.01128 3.83280 3.47029 4.19583 3.30941 3.38423
3.04262 2.74330 3.02373 5.29785 3.36096 72 l - - G
2.68590 4.42240 2.77407 2.73138 3.46369 2.40528 3.72509
3.29369 2.67756 2.69370 4.24705 2.90361 2.73754 3.18119 2.89815
2.37876 2.77534 2.98533 4.58492 3.61518
0.47582 0.98005 5.70524 0.07760 2.59470 0.25079 1.50591
61 3.01446 4.50190 4.43875 3.07931 3.29755 4.11040 3.58641
2.33377 3.73937 1.29618 3.09214 4.07614 4.48043 2.62017 3.93232
3.01370 2.82285 2.24287 5.11199 3.91838 74 l - - C
2.68618 4.42225 2.77519 2.73123 3.46354 2.40513 3.72494
3.29354 2.67741 2.69355 4.24690 2.90347 2.73739 3.18146 2.89801
2.37887 2.77519 2.98518 4.58477 3.61503
0.00808 5.21799 5.94034 0.61958 0.77255 0.48576 0.95510
62 2.97935 5.21159 3.07176 2.80346 3.93631 2.92664 2.67901
3.08928 2.59289 2.45428 3.32553 2.81626 3.38379 3.14908 2.33992
2.62198 2.69422 3.54834 5.70793 3.04668 75 r - - C
2.68618 4.42225 2.77519 2.73123 3.46354 2.40513 3.72494
3.29354 2.67741 2.69355 4.24690 2.90347 2.73739 3.18146 2.89801
2.37887 2.77519 2.98518 4.58477 3.61503
0.00808 5.21799 5.94034 0.61958 0.77255 0.48576 0.95510
63 2.99122 4.91461 3.61096 2.42422 2.97343 3.89674 2.23718
2.99049 3.02662 2.67250 4.00582 2.44971 4.28147 2.59604 3.44366
2.75219 3.22275 2.91891 2.87052 3.17766 76 h - - T
2.68618 4.42225 2.77519 2.73123 3.46354 2.40513 3.72494
3.29354 2.67741 2.69355 4.24690 2.90347 2.73739 3.18146 2.89801
2.37887 2.77519 2.98518 4.58477 3.61503
0.00808 5.21799 5.94034 0.61958 0.77255 0.48576 0.95510
64 2.99244 4.89148 3.63750 2.60744 2.67591 3.90577 4.13553
3.43669 2.02629 2.04868 3.58411 3.54131 3.56928 2.49156 3.46081
2.79786 3.22396 3.18301 3.73032 2.53657 77 k - - T

```
          2.68618  4.42225  2.77519  2.73123  3.46354  2.40513  3.72494
3.29354  2.67741  2.69355  4.24690  2.90347  2.73739  3.18146  2.89801
2.37887  2.77519  2.98518  4.58477  3.61503
          0.00808  5.21799  5.94034  0.61958  0.77255  0.48576  0.95510
     65   2.50043  4.90627  3.18831  3.05763  4.07709  3.89946  4.12782
2.97253  2.78540  2.36890  2.77179  2.35754  3.55069  3.36312  3.44960
2.25420  2.37109  2.38106  5.45991  4.18744     78 s - - T
          2.68618  4.42225  2.77519  2.73123  3.46354  2.40513  3.72494
3.29354  2.67741  2.69355  4.24690  2.90347  2.73739  3.18146  2.89801
2.37887  2.77519  2.98518  4.58477  3.61503
          0.00808  5.21799  5.94034  0.61958  0.77255  0.48576  0.95510
     66   2.97905  5.22081  2.52475  2.79771  3.33714  3.38245  4.00289
2.93711  2.53130  3.48441  3.59102  2.79779  4.19203  2.86546  2.73840
2.60942  1.80458  2.84041  5.71519  3.62543     79 t - - C
          2.68618  4.42225  2.77519  2.73123  3.46354  2.40513  3.72494
3.29354  2.67741  2.69355  4.24690  2.90347  2.73739  3.18146  2.89801
2.37887  2.77519  2.98518  4.58477  3.61503
          0.00808  5.21799  5.94034  0.61958  0.77255  0.48576  0.95510
     67   2.82376  4.93600  3.16873  2.59914  3.13042  3.38075  4.11342
2.32804  2.67267  2.18560  2.61142  3.50332  4.27359  2.64166  2.87433
2.49086  3.22139  2.86386  5.48462  4.20571     80 l - - E
          2.68618  4.42225  2.77519  2.73123  3.46354  2.40513  3.72494
3.29354  2.67741  2.69355  4.24690  2.90347  2.73739  3.18146  2.89801
2.37887  2.77519  2.98518  4.58477  3.61503
          0.00808  5.21799  5.94034  0.61958  0.77255  0.48576  0.95510
     68   2.65944  4.41907  3.52531  4.22937  2.81958  4.17239  4.49786
1.78682  3.08119  2.20146  2.63399  4.28451  4.53760  4.20507  4.10673
2.85969  2.93631  2.20147  3.43671  2.48969     81 i - - E
          2.68618  4.42225  2.77519  2.73123  3.46354  2.40513  3.72494
3.29354  2.67741  2.69355  4.24690  2.90347  2.73739  3.18146  2.89801
2.37887  2.77519  2.98518  4.58477  3.61503
          0.00808  5.21799  5.94034  0.61958  0.77255  0.48576  0.95510
     69   2.97468  5.39993  2.37844  2.70200  4.71094  3.76191  3.13216
4.17358  2.70245  2.72411  4.44982  2.77496  2.77799  3.06165  2.70166
1.76901  2.91389  3.77220  2.96870  4.46418     82 s - - -
          2.68618  4.42225  2.77519  2.73123  3.46354  2.40513  3.72494
3.29354  2.67741  2.69355  4.24690  2.90347  2.73739  3.18146  2.89801
2.37887  2.77519  2.98518  4.58477  3.61503
          0.00808  5.21799  5.94034  0.61958  0.77255  0.48576  0.95510
     70   3.46944  5.71937  3.81122  2.81057  5.11735  4.16274  4.18371
4.49736  2.22686  3.12014  4.78814  3.65857  0.98046  3.30746  1.87194
3.45463  3.65866  4.14778  6.05351  4.80918     83 p - - H
          2.68618  4.42225  2.77519  2.73123  3.46354  2.40513  3.72494
3.29354  2.67741  2.69355  4.24690  2.90347  2.73739  3.18146  2.89801
2.37887  2.77519  2.98518  4.58477  3.61503
          0.00808  5.21799  5.94034  0.61958  0.77255  0.48576  0.95510
     71   2.97374  3.64261  2.82855  2.17450  3.59493  3.76134  3.58275
2.65073  1.93454  3.68990  4.44900  2.87996  4.15394  2.91309  3.04941
2.24974  2.51343  3.77146  5.85285  3.38642     84 k - - H
          2.68618  4.42225  2.77519  2.73123  3.46354  2.40513  3.72494
3.29354  2.67741  2.69355  4.24690  2.90347  2.73739  3.18146  2.89801
2.37887  2.77519  2.98518  4.58477  3.61503
          0.00808  5.21799  5.94034  0.61958  0.77255  0.48576  0.95510
     72   2.98541  5.04337  2.70800  2.41073  3.60447  3.85184  4.06653
3.06648  2.91227  2.36763  3.48071  3.42492  4.23928  2.24355  2.55440
2.72702  2.80319  2.31843  5.57300  3.30885     85 q - - H
```

```
         2.68652  4.42259  2.77495  2.73096  3.46388  2.40525  3.72529
3.29388  2.67715  2.69271  4.24588  2.90364  2.73683  3.18147  2.89835
2.37921  2.77554  2.98553  4.58511  3.61446
         0.45042  1.02163  5.94034  0.63661  0.75307  0.48576  0.95510
     73  3.04630  4.61696  4.21412  3.64117  2.92751  4.08627  4.36065
3.07609  2.89745  0.89878  3.71850  3.95038  4.46189  3.02835  3.76034
2.98868  2.93816  2.87616  5.21123  4.00343     88 l - - H
         2.68618  4.42225  2.77519  2.73123  3.46354  2.40513  3.72494
3.29354  2.67741  2.69355  4.24690  2.90347  2.73739  3.18146  2.89801
2.37887  2.77519  2.98518  4.58477  3.61503
         0.05546  5.21799  3.02559  0.61958  0.77255  0.48576  0.95510
     74  2.96252  3.73634  3.07118  2.25260  4.22465  3.82835  4.04284
3.08152  2.37027  2.52268  3.75594  3.40069  4.21583  2.70362  2.37010
2.77576  3.19394  2.36374  3.43073  3.27544     89 e - - H
         2.68618  4.42225  2.77519  2.73123  3.46354  2.40513  3.72494
3.29354  2.67741  2.69355  4.24690  2.90347  2.73739  3.18146  2.89801
2.37887  2.77519  2.98518  4.58477  3.61503
         0.00847  5.17101  5.89335  0.61958  0.77255  0.63500  0.75489
     75  2.75080  5.45345  3.20162  1.96447  4.79506  2.98880  3.91328
4.27857  1.95805  3.75722  4.49509  2.54125  3.13723  2.32685  3.14143
2.51126  3.18224  3.34565  5.88817  2.82765     90 k - - H
         2.68618  4.42225  2.77519  2.73123  3.46354  2.40513  3.72494
3.29354  2.67741  2.69355  4.24690  2.90347  2.73739  3.18146  2.89801
2.37887  2.77519  2.98518  4.58477  3.61503
         0.00847  5.17101  5.89335  0.61958  0.77255  0.63500  0.75489
     76  2.11825  5.44235  3.20646  2.09226  4.77942  2.63259  2.60482
4.25975  2.44324  3.07478  4.48516  2.21335  3.51885  3.01438  2.96620
2.59595  3.18207  3.82869  5.87981  3.98016     91 e - - T
         2.68618  4.42225  2.77519  2.73123  3.46354  2.40513  3.72494
3.29354  2.67741  2.69355  4.24690  2.90347  2.73739  3.18146  2.89801
2.37887  2.77519  2.98518  4.58477  3.61503
         0.11984  5.17101  2.23249  0.61958  0.77255  0.63500  0.75489
     77  2.96194  5.47073  2.93651  2.42741  4.81159  1.50294  3.90974
4.29660  2.36396  3.77253  4.51571  2.24939  3.07780  2.73676  2.74438
2.92009  3.19521  3.85975  5.90215  4.48962     92 g - - T
         2.68618  4.42225  2.77519  2.73123  3.46354  2.40513  3.72494
3.29354  2.67741  2.69355  4.24690  2.90347  2.73739  3.18146  2.89801
2.37887  2.77519  2.98518  4.58477  3.61503
         0.00947  5.06063  5.78298  0.61958  0.77255  0.44827  1.01814
     78  3.12268  4.49259  5.02100  4.42408  2.49466  4.29081  3.36987
1.29096  4.22273  2.62298  3.59405  4.42074  4.64730  4.35510  4.24786
3.60425  3.35507  1.39889  5.00734  3.18415     93 i - - -
         2.68618  4.42225  2.77519  2.73123  3.46354  2.40513  3.72494
3.29354  2.67741  2.69355  4.24690  2.90347  2.73739  3.18146  2.89801
2.37887  2.77519  2.98518  4.58477  3.61503
         0.00847  5.17101  5.89335  0.61958  0.77255  0.63500  0.75489
     79  2.96743  5.44319  3.03123  2.36001  4.77801  3.74254  3.92666
4.25272  2.17635  3.15115  4.48768  3.21516  1.58308  2.40354  2.50178
2.93557  3.19759  3.16751  5.87951  4.48074     94 p - - -
         2.68618  4.42225  2.77519  2.73123  3.46354  2.40513  3.72494
3.29354  2.67741  2.69355  4.24690  2.90347  2.73739  3.18146  2.89801
2.37887  2.77519  2.98518  4.58477  3.61503
         0.00847  5.17101  5.89335  0.61958  0.77255  0.63500  0.75489
     80  2.99548  3.12704  4.85065  4.24275  2.91700  3.14997  4.48094
2.09671  4.05123  2.57846  3.50635  4.27886  2.62947  4.20289  4.09788
3.06229  2.76095  1.45967  5.00830  2.76499     95 v - - -
```

```
          2.68618  4.42225  2.77519  2.73123  3.46354  2.40513  3.72494
3.29354  2.67741  2.69355  4.24690  2.90347  2.73739  3.18146  2.89801
2.37887  2.77519  2.98518  4.58477  3.61503
          0.00847  5.17101  5.89335  0.61958  0.77255  0.63500  0.75489
     81   2.71241  3.59177  3.50912  3.25392  3.85696  3.94640  3.15222
2.65511  2.79789  2.42788  3.81905  2.85498  4.32639  3.12354  3.57396
3.00402  2.88859  2.99231  2.85338  1.85130     96 y - - E
          2.68618  4.42225  2.77519  2.73123  3.46354  2.40513  3.72494
3.29354  2.67741  2.69355  4.24690  2.90347  2.73739  3.18146  2.89801
2.37887  2.77519  2.98518  4.58477  3.61503
          0.00847  5.17101  5.89335  0.61958  0.77255  0.63500  0.75489
     82   2.58418  5.29783  3.27750  2.26985  3.97412  3.75730  3.95216
3.29824  2.08493  3.57562  4.35587  3.25525  4.14892  3.07415  1.94180
2.50860  2.10616  2.94598  5.77025  4.40149     97 r - - E
          2.68618  4.42225  2.77519  2.73123  3.46354  2.40513  3.72494
3.29354  2.67741  2.69355  4.24690  2.90347  2.73739  3.18146  2.89801
2.37887  2.77519  2.98518  4.58477  3.61503
          0.00847  5.17101  5.89335  0.61958  0.77255  0.40262  1.10434
     83   2.63611  2.91852  5.02772  4.41539  1.77923  4.24097  4.57944
1.78224  4.20355  2.15154  3.09572  4.40278  4.60149  4.33607  4.21427
3.55403  2.43404  2.08379  5.07052  3.89461     98 f - - E
          2.68618  4.42225  2.77519  2.73123  3.46354  2.40513  3.72494
3.29354  2.67741  2.69355  4.24690  2.90347  2.73739  3.18146  2.89801
2.37887  2.77519  2.98518  4.58477  3.61503
          0.00808  5.21799  5.94034  0.61958  0.77255  0.48576  0.95510
     84   2.99343  4.86494  3.37041  2.45977  3.28143  3.91593  3.69013
3.10771  2.60808  2.34141  3.95943  2.99108  4.29950  3.40254  3.48268
3.14352  2.45626  1.47168  5.42528  4.16169     99 v - - E
          2.68618  4.42225  2.77519  2.73123  3.46354  2.40513  3.72494
3.29354  2.67741  2.69355  4.24690  2.90347  2.73739  3.18146  2.89801
2.37887  2.77519  2.98518  4.58477  3.61503
          0.00808  5.21799  5.94034  0.61958  0.77255  0.48576  0.95510
     85   3.33910  5.31341  3.81506  3.28568  4.27735  4.13011  3.62025
3.85100  2.94816  2.82361  4.36930  3.73483  4.53249  0.71172  3.25446
3.42529  3.56636  2.88545  5.65935  4.30297    100 q - - E
          2.68618  4.42225  2.77519  2.73123  3.46354  2.40513  3.72494
3.29354  2.67741  2.69355  4.24690  2.90347  2.73739  3.18146  2.89801
2.37887  2.77519  2.98518  4.58477  3.61503
          0.00808  5.21799  5.94034  0.61958  0.77255  0.48576  0.95510
     86   2.97417  4.54030  3.23176  2.32429  3.14422  3.09633  2.70494
4.27817  1.84373  3.76371  4.50560  2.42171  4.14420  2.74782  2.08991
2.94068  3.20518  3.84850  5.90008  3.52176    101 k - - -
          2.68618  4.42225  2.77519  2.73123  3.46354  2.40513  3.72494
3.29354  2.67741  2.69355  4.24690  2.90347  2.73739  3.18146  2.89801
2.37887  2.77519  2.98518  4.58477  3.61503
          0.00808  5.21799  5.94034  0.61958  0.77255  0.48576  0.95510
     87   1.97771  5.24091  3.36219  2.66752  4.51365  3.81285  4.03880
3.93743  2.82717  2.53178  4.33195  3.34842  1.68053  2.31557  3.29096
2.39476  3.24332  3.59799  5.75612  4.41277    102 p - - T
          2.68618  4.42225  2.77519  2.73123  3.46354  2.40513  3.72494
3.29354  2.67741  2.69355  4.24690  2.90347  2.73739  3.18146  2.89801
2.37887  2.77519  2.98518  4.58477  3.61503
          0.00808  5.21799  5.94034  0.61958  0.77255  0.48576  0.95510
     88   3.77843  6.40442  2.19000  2.86144  5.74397  0.70758  3.48993
5.32019  3.67834  4.78059  5.64219  2.00009  4.64267  3.76853  4.32021
3.64471  4.08973  4.84571  6.92040  5.36223    103 g - - T
```

2.68618 4.42225 2.77519 2.73123 3.46354 2.40513 3.72494
3.29354 2.67741 2.69355 4.24690 2.90347 2.73739 3.18146 2.89801
2.37887 2.77519 2.98518 4.58477 3.61503
0.00808 5.21799 5.94034 0.61958 0.77255 0.48576 0.95510
89 3.77440 6.49911 1.64022 0.82079 5.77248 3.97831 4.54534
5.33788 3.60509 4.77433 5.62227 3.09065 4.61955 1.98003 4.24980
3.62244 4.06661 4.86219 6.91280 5.34245 104 e - - -
2.68618 4.42225 2.77519 2.73123 3.46354 2.40513 3.72494
3.29354 2.67741 2.69355 4.24690 2.90347 2.73739 3.18146 2.89801
2.37887 2.77519 2.98518 4.58477 3.61503
0.00808 5.21799 5.94034 0.61958 0.77255 0.48576 0.95510
90 2.37176 4.41591 4.95331 4.34150 1.53893 4.20103 4.53212
2.58368 4.13695 2.08183 2.80437 4.34827 4.56364 4.27569 4.16118
3.51072 2.14722 2.33156 5.03692 2.91331 105 f - - E
2.68618 4.42225 2.77519 2.73123 3.46354 2.40513 3.72494
3.29354 2.67741 2.69355 4.24690 2.90347 2.73739 3.18146 2.89801
2.37887 2.77519 2.98518 4.58477 3.61503
0.00808 5.21799 5.94034 0.61958 0.77255 0.48576 0.95510
91 3.98381 5.17938 6.09047 5.59463 4.27452 5.56587 6.17742
1.61823 5.51698 2.00493 2.71509 5.73301 5.74175 5.65374 5.60482
4.98415 4.23081 0.68330 6.39414 5.26287 106 V - - E
2.68618 4.42225 2.77519 2.73123 3.46354 2.40513 3.72494
3.29354 2.67741 2.69355 4.24690 2.90347 2.73739 3.18146 2.89801
2.37887 2.77519 2.98518 4.58477 3.61503
0.00808 5.21799 5.94034 0.61958 0.77255 0.48576 0.95510
92 3.25785 4.61295 5.17758 4.59218 1.42906 4.41392 4.56684
1.42922 4.37956 2.43997 3.66307 4.53832 4.76196 4.47724 4.37625
3.73131 3.48826 2.18963 3.31328 2.76604 107 f - - E
2.68618 4.42225 2.77519 2.73123 3.46354 2.40513 3.72494
3.29354 2.67741 2.69355 4.24690 2.90347 2.73739 3.18146 2.89801
2.37887 2.77519 2.98518 4.58477 3.61503
0.00808 5.21799 5.94034 0.61958 0.77255 0.48576 0.95510
93 3.58317 3.46555 5.59867 5.06845 4.21324 4.93907 5.50273
1.40095 4.93624 2.95031 4.08005 5.12699 5.27856 5.13638 5.02936
4.30946 1.34734 1.29344 5.97542 4.77237 108 v - - E
2.68618 4.42225 2.77519 2.73123 3.46354 2.40513 3.72494
3.29354 2.67741 2.69355 4.24690 2.90347 2.73739 3.18146 2.89801
2.37887 2.77519 2.98518 4.58477 3.61503
0.00808 5.21799 5.94034 0.61958 0.77255 0.48576 0.95510
94 3.07101 4.87631 3.79629 3.29679 1.51385 3.37126 4.27790
3.45532 3.29851 3.14676 4.03480 2.69436 1.61565 3.60248 3.67552
2.80466 3.32721 3.20852 5.39205 3.54937 109 f - - -
2.68618 4.42225 2.77519 2.73123 3.46354 2.40513 3.72494
3.29354 2.67741 2.69355 4.24690 2.90347 2.73739 3.18146 2.89801
2.37887 2.77519 2.98518 4.58477 3.61503
0.00808 5.21799 5.94034 0.61958 0.77255 0.48576 0.95510
95 1.83680 4.96418 4.21394 3.73489 4.84430 2.73121 4.77564
3.36629 3.71507 3.92185 4.74551 3.98699 1.02298 4.01583 4.08357
2.05582 2.92256 3.80030 6.18850 4.95298 110 p - - T
2.68618 4.42225 2.77519 2.73123 3.46354 2.40513 3.72494
3.29354 2.67741 2.69355 4.24690 2.90347 2.73739 3.18146 2.89801
2.37887 2.77519 2.98518 4.58477 3.61503
0.00808 5.21799 5.94034 0.61958 0.77255 0.48576 0.95510
96 2.99387 5.41125 2.73558 2.71540 3.75449 1.30650 3.96845
4.18074 2.32620 3.70012 4.46306 2.86735 4.17004 3.07937 2.57375
2.97376 3.22526 3.78283 5.86341 3.19101 111 g - - T

2.68618 4.42225 2.77519 2.73123 3.46354 2.40513 3.72494
3.29354 2.67741 2.69355 4.24690 2.90347 2.73739 3.18146 2.89801
2.37887 2.77519 2.98518 4.58477 3.61503
0.00808 5.21799 5.94034 0.61958 0.77255 0.48576 0.95510
97 1.95108 2.39354 4.34347 3.81177 4.10432 2.49835 4.60608
3.46797 3.74218 3.19291 4.08535 4.04007 4.46697 4.00831 4.02464
1.97897 1.99204 3.19901 2.14814 4.36107 112 a - - -
2.68618 4.42225 2.77519 2.73123 3.46354 2.40513 3.72494
3.29354 2.67741 2.69355 4.24690 2.90347 2.73739 3.18146 2.89801
2.37887 2.77519 2.98518 4.58477 3.61503
0.00808 5.21799 5.94034 0.61958 0.77255 0.48576 0.95510
98 3.05960 4.44392 4.93459 4.33046 2.13075 4.21540 3.36127
2.49885 4.13247 2.62932 3.14271 4.34325 2.70813 4.27316 4.16651
3.52407 3.29153 2.07921 3.24914 1.61116 113 y - - E
2.68618 4.42225 2.77519 2.73123 3.46354 2.40513 3.72494
3.29354 2.67741 2.69355 4.24690 2.90347 2.73739 3.18146 2.89801
2.37887 2.77519 2.98518 4.58477 3.61503
0.00808 5.21799 5.94034 0.61958 0.77255 0.48576 0.95510
99 4.49522 5.84382 5.20784 4.89988 2.87644 5.15771 0.54328
4.47146 4.62009 3.81525 5.03511 4.81805 5.51947 4.83355 4.73753
4.54841 4.73326 3.16103 4.54195 1.72012 114 H - - E
2.68618 4.42225 2.77519 2.73123 3.46354 2.40513 3.72494
3.29354 2.67741 2.69355 4.24690 2.90347 2.73739 3.18146 2.89801
2.37887 2.77519 2.98518 4.58477 3.61503
0.00808 5.21799 5.94034 0.61958 0.77255 0.48576 0.95510
100 1.94645 4.85371 3.69067 3.12788 4.01864 3.92061 4.16177
3.39174 3.09776 3.08081 3.38172 3.58143 4.30689 1.92154 3.49732
1.91461 2.83074 2.63222 2.87009 4.16016 115 s - - E
2.68618 4.42225 2.77519 2.73123 3.46354 2.40513 3.72494
3.29354 2.67741 2.69355 4.24690 2.90347 2.73739 3.18146 2.89801
2.37887 2.77519 2.98518 4.58477 3.61503
0.00808 5.21799 5.94034 0.61958 0.77255 0.48576 0.95510
101 2.35533 4.79930 4.24071 3.74679 4.21788 1.61947 4.62078
3.49938 3.03411 3.28594 4.18242 4.01291 4.48689 3.98118 3.98630
3.28455 2.74047 1.13409 5.66913 4.45664 116 v - - E
2.68618 4.42225 2.77519 2.73123 3.46354 2.40513 3.72494
3.29354 2.67741 2.69355 4.24690 2.90347 2.73739 3.18146 2.89801
2.37887 2.77519 2.98518 4.58477 3.61503
0.00808 5.21799 5.94034 0.61958 0.77255 0.48576 0.95510
102 3.02608 4.46407 4.64214 4.04776 1.59127 4.15137 3.27253
2.74295 3.89807 1.77775 3.57829 4.19042 4.51837 2.61799 3.07517
3.44621 3.25803 2.16256 3.78970 3.87191 117 f - - E
2.68618 4.42225 2.77519 2.73123 3.46354 2.40513 3.72494
3.29354 2.67741 2.69355 4.24690 2.90347 2.73739 3.18146 2.89801
2.37887 2.77519 2.98518 4.58477 3.61503
0.00808 5.21799 5.94034 0.61958 0.77255 0.48576 0.95510
103 2.57954 3.01283 2.64399 2.93635 4.80468 3.78166 4.18329
4.25595 2.97484 3.81256 4.59013 1.11424 4.26578 3.31512 3.44792
2.30136 2.76518 3.83257 6.00066 4.64247 118 n - - E
2.68618 4.42225 2.77519 2.73123 3.46354 2.40513 3.72494
3.29354 2.67741 2.69355 4.24690 2.90347 2.73739 3.18146 2.89801
2.37887 2.77519 2.98518 4.58477 3.61503
0.00808 5.21799 5.94034 0.61958 0.77255 0.48576 0.95510
104 3.00881 2.44881 4.18530 3.30391 3.39333 4.05811 2.73211
2.68361 3.53017 1.85972 3.21429 3.00601 3.31682 3.51377 2.99931
3.32517 2.50114 2.65410 5.18072 3.13466 119 l - - S

2.68618 4.42225 2.77519 2.73123 3.46354 2.40513 3.72494
3.29354 2.67741 2.69355 4.24690 2.90347 2.73739 3.18146 2.89801
2.37887 2.77519 2.98518 4.58477 3.61503
0.00808 5.21799 5.94034 0.61958 0.77255 0.48576 0.95510
105 3.01034 5.31199 2.54257 2.78114 4.59300 1.21400 4.02072
4.02963 2.44778 3.59757 4.38923 3.31261 4.20493 3.15222 3.27893
2.75895 2.92294 3.02871 5.80634 3.33164 120 g - - S
2.68618 4.42225 2.77519 2.73123 3.46354 2.40513 3.72494
3.29354 2.67741 2.69355 4.24690 2.90347 2.73739 3.18146 2.89801
2.37887 2.77519 2.98518 4.58477 3.61503
0.00808 5.21799 5.94034 0.61958 0.77255 0.48576 0.95510
106 3.01132 4.69693 2.34447 3.37707 1.88793 4.00436 4.25600
3.18779 3.32609 2.61814 3.80225 3.76506 3.18972 3.62003 3.66428
2.39818 2.60125 2.96803 3.51727 2.38112 121 f - - S
2.68618 4.42225 2.77519 2.73123 3.46354 2.40513 3.72494
3.29354 2.67741 2.69355 4.24690 2.90347 2.73739 3.18146 2.89801
2.37887 2.77519 2.98518 4.58477 3.61503
0.00808 5.21799 5.94034 0.61958 0.77255 0.48576 0.95510
107 2.27932 1.73274 4.26355 3.79357 4.95288 2.73389 4.83794
4.37823 3.77530 4.03363 4.84583 1.30675 4.46322 4.07288 4.14133
2.64319 2.05525 3.88032 6.28347 5.05127 122 n - - E
2.68618 4.42225 2.77519 2.73123 3.46354 2.40513 3.72494
3.29354 2.67741 2.69355 4.24690 2.90347 2.73739 3.18146 2.89801
2.37887 2.77519 2.98518 4.58477 3.61503
0.00808 5.21799 5.94034 0.61958 0.77255 0.48576 0.95510
108 3.04597 2.67422 4.98940 4.37752 2.38360 3.78679 4.55414
1.44244 4.16903 1.76733 3.53842 3.25064 4.58075 4.30533 4.18642
3.52994 3.27863 2.16077 5.05277 3.87577 123 i - - E
2.68618 4.42225 2.77519 2.73123 3.46354 2.40513 3.72494
3.29354 2.67741 2.69355 4.24690 2.90347 2.73739 3.18146 2.89801
2.37887 2.77519 2.98518 4.58477 3.61503
0.00808 5.21799 5.94034 0.61958 0.77255 0.48576 0.95510
109 1.69190 5.35614 3.30094 2.75629 4.68662 2.93323 3.99873
4.13886 2.18235 2.81072 4.44743 2.24629 4.18354 3.11604 3.23490
1.91494 3.23061 3.74881 5.85508 4.47943 124 a - - E
2.68618 4.42225 2.77519 2.73123 3.46354 2.40513 3.72494
3.29354 2.67741 2.69355 4.24690 2.90347 2.73739 3.18146 2.89801
2.37887 2.77519 2.98518 4.58477 3.61503
0.00808 5.21799 5.94034 0.61958 0.77255 0.48576 0.95510
110 3.02894 4.67628 4.02857 1.48416 3.06608 4.04253 4.30969
2.11180 3.39864 2.87149 3.78307 3.83081 4.41881 3.68919 3.06951
3.29821 2.69621 1.86785 5.27999 4.05756 125 e - - E
2.68618 4.42225 2.77519 2.73123 3.46354 2.40513 3.72494
3.29354 2.67741 2.69355 4.24690 2.90347 2.73739 3.18146 2.89801
2.37887 2.77519 2.98518 4.58477 3.61503
0.00808 5.21799 5.94034 0.61958 0.77255 0.48576 0.95510
111 1.53347 5.41592 3.28405 2.73315 4.76427 3.77302 3.98097
4.22875 2.23463 3.73850 4.49584 2.63400 4.17587 3.08901 2.78844
1.85515 2.47559 3.81699 5.89308 4.50614 126 a - - E
2.68618 4.42225 2.77519 2.73123 3.46354 2.40513 3.72494
3.29354 2.67741 2.69355 4.24690 2.90347 2.73739 3.18146 2.89801
2.37887 2.77519 2.98518 4.58477 3.61503
0.00808 5.21799 5.94034 0.61958 0.77255 0.48576 0.95510
112 2.73911 4.59148 4.15490 2.93136 3.06528 3.17666 3.00250
2.80097 3.50447 2.79320 2.95494 3.90295 4.42557 2.93721 3.78343
2.90093 2.54445 1.63966 2.81402 3.98095 127 v - - E

```
          2.68618  4.42225  2.77519  2.73123  3.46354  2.40513  3.72494
3.29354  2.67741  2.69355  4.24690  2.90347  2.73739  3.18146  2.89801
2.37887  2.77519  2.98518  4.58477  3.61503
          0.00808  5.21799  5.94034  0.61958  0.77255  0.48576  0.95510
    113   2.58837  5.54777  2.37073  2.51789  4.87646  3.78261  2.82409
4.35724  2.77574  3.84716  4.59855  1.22356  3.36670  3.11782  3.27488
3.01831  3.29380  3.19167  5.98956  4.57724    128 n - - E
          2.68618  4.42225  2.77519  2.73123  3.46354  2.40513  3.72494
3.29354  2.67741  2.69355  4.24690  2.90347  2.73739  3.18146  2.89801
2.37887  2.77519  2.98518  4.58477  3.61503
          0.00808  5.21799  5.94034  0.61958  0.77255  0.48576  0.95510
    114   3.75904  5.06027  5.54392  5.03735  0.80802  4.88069  4.66778
2.69608  4.83545  2.70388  3.91516  4.90498  5.18381  4.85198  4.81065
4.21731  3.98559  1.97564  2.31845  3.33718    129 f - - E
          2.68618  4.42225  2.77519  2.73123  3.46354  2.40513  3.72494
3.29354  2.67741  2.69355  4.24690  2.90347  2.73739  3.18146  2.89801
2.37887  2.77519  2.98518  4.58477  3.61503
          0.00545  5.21536        *  0.61958  0.77255  0.00000        *
//
HMMER3/f [3.3 | Nov 2019]
NAME  JmjN
ACC   PF02375.18
DESC  jmjN domain
LENG  34
ALPH  amino
RF    no
MM    no
CONS  yes
CS    yes
MAP   yes
DATE  Tue Apr 21 07:53:16 2020
NSEQ  289
EFFN  10.993607
CKSUM 4117382378
GA    26.00 26.00
TC    26.00 26.10
NC    25.90 25.80
STATS LOCAL MSV       -6.9655  0.71971
STATS LOCAL VITERBI   -7.4909  0.71971
STATS LOCAL FORWARD   -4.2021  0.71971
HMM          A        C        D        E        F        G        H
I        K        L        M        N        P        Q        R        S
T        V        W        Y
            m->m     m->i     m->d     i->m     i->i     d->m     d->d
  COMPO   2.87346  4.31569  3.27451  2.47051  2.70053  2.85187  4.19174
2.42577  2.56624  3.03061  3.61489  3.56458  2.29943  3.47836  3.19281
2.99539  3.08527  2.68083  5.00863  2.88533
          2.68618  4.42225  2.77519  2.73123  3.46354  2.40513  3.72494
3.29354  2.67741  2.69355  4.24690  2.90347  2.73739  3.18146  2.89801
2.37887  2.77519  2.98518  4.58477  3.61503
          0.07341  6.05326  2.68191  0.61958  0.77255  0.00000        *
      1   4.93963  6.64099  6.36876  6.35270  6.31162  5.42299  7.01346
5.95894  6.23755  5.21439  2.39872  6.22387  0.15075  6.59370  6.26240
5.17588  5.48470  5.70246  7.70583  6.69641      1 P - - -
          2.68618  4.42225  2.77519  2.73123  3.46354  2.40513  3.72494
3.29354  2.67741  2.69355  4.24690  2.90347  2.73739  3.18146  2.89801
2.37887  2.77519  2.98518  4.58477  3.61503
          0.00375  5.98359  6.70594  0.61958  0.77255  0.34072  1.24221
```

```
      2   3.54892  4.50755  5.65314  3.62154  4.00584  4.93493  5.30275
1.99540  4.86988  3.26933  4.24081  5.08243  5.29488  5.02374  4.91272
3.82166  1.53557  0.84768  5.81183  4.62992       2 v - - E
         2.68618  4.42225  2.77519  2.73123  3.46354  2.40513  3.72494
3.29354  2.67741  2.69355  4.24690  2.90347  2.73739  3.18146  2.89801
2.37887  2.77519  2.98518  4.58477  3.61503
         0.00352  6.04568  6.76802  0.61958  0.77255  0.54502  0.86710
      3   4.95016  6.16270  6.49694  6.14305  0.48734  5.89951  4.35305
4.07263  5.92149  2.00760  5.12140  5.74754  6.18976  5.79394  5.81651
5.25421  5.16732  2.91511  5.28578  2.15550       3 F - - E
         2.68618  4.42225  2.77519  2.73123  3.46354  2.40513  3.72494
3.29354  2.67741  2.69355  4.24690  2.90347  2.73739  3.18146  2.89801
2.37887  2.77519  2.98518  4.58477  3.61503
         0.00352  6.04568  6.76802  0.61958  0.77255  0.52580  0.89424
      4   3.34292  4.68144  3.72936  2.36816  4.17207  4.23561  2.60237
4.49463  2.25880  4.01423  4.57269  3.38660  4.62829  3.53287  1.64801
3.13108  2.88330  4.25963  6.33630  1.56565       4 y - - -
         2.68618  4.42225  2.77519  2.73123  3.46354  2.40513  3.72494
3.29354  2.67741  2.69355  4.24690  2.90347  2.73739  3.18146  2.89801
2.37887  2.77519  2.98518  4.58477  3.61503
         0.00352  6.04716  6.76951  0.61958  0.77255  0.45691  1.00304
      5   3.28043  5.76788  6.01964  6.03333  6.53642  4.56858  6.69612
4.51177  5.97188  5.63951  6.48890  5.44237  0.13882  6.12963  5.97558
4.08149  3.97966  5.05371  7.93096  6.89069       5 P - - -
         2.68618  4.42225  2.77519  2.73123  3.46354  2.40513  3.72494
3.29354  2.67741  2.69355  4.24690  2.90347  2.73739  3.18146  2.89801
2.37887  2.77519  2.98518  4.58477  3.61503
         0.00350  6.05326  6.77560  0.61958  0.77255  0.48576  0.95510
      6   3.70226  6.30628  3.20443  3.22460  5.64366  4.43329  4.75502
5.12673  3.46526  4.62251  5.39706  3.29614  4.91483  3.73265  3.70851
1.63582  0.66357  4.70279  6.77355  5.34911       6 T - - -
         2.68618  4.42225  2.77519  2.73123  3.46354  2.40513  3.72494
3.29354  2.67741  2.69355  4.24690  2.90347  2.73739  3.18146  2.89801
2.37887  2.77519  2.98518  4.58477  3.61503
         0.00350  6.05326  6.77560  0.61958  0.77255  0.48576  0.95510
      7   2.63297  5.68834  3.22826  1.66692  4.23370  4.29529  4.33936
3.32199  2.64813  2.69099  1.91237  3.65670  3.45478  3.39656  3.37190
2.81864  3.14380  3.45928  3.17338  3.46199       7 e - - H
         2.68618  4.42225  2.77519  2.73123  3.46354  2.40513  3.72494
3.29354  2.67741  2.69355  4.24690  2.90347  2.73739  3.18146  2.89801
2.37887  2.77519  2.98518  4.58477  3.61503
         0.00350  6.05326  6.77560  0.61958  0.77255  0.48576  0.95510
      8   2.91179  5.96299  2.45299  0.83157  5.30922  4.08325  3.70566
4.79603  2.58980  4.26874  4.50069  3.51065  4.61614  2.68832  3.20118
2.99458  3.10360  4.20494  6.39493  4.98323       8 e - - H
         2.68618  4.42225  2.77519  2.73123  3.46354  2.40513  3.72494
3.29354  2.67741  2.69355  4.24690  2.90347  2.73739  3.18146  2.89801
2.37887  2.77519  2.98518  4.58477  3.61503
         0.00350  6.05326  6.77560  0.61958  0.77255  0.48576  0.95510
      9   4.77939  7.65381  2.01301  0.40842  6.87688  4.76104  5.45786
6.50663  4.70221  5.91045  6.85566  4.19224  5.47862  2.17721  5.47675
4.56737  4.34797  5.99869  8.05682  6.37280       9 E - - H
         2.68618  4.42225  2.77519  2.73123  3.46354  2.40513  3.72494
3.29354  2.67741  2.69355  4.24690  2.90347  2.73739  3.18146  2.89801
2.37887  2.77519  2.98518  4.58477  3.61503
         0.00350  6.05326  6.77560  0.61958  0.77255  0.48576  0.95510
```

```
      10   5.86576  6.82049  6.89372  6.80333  0.21513  6.48009  5.17499
 4.57817  6.57279  3.77967  3.25405  6.06012  6.69489  6.18723  6.33826
 5.88549  6.05358  5.22924  2.95328  3.29266      10 F - - H
           2.68621  4.42228  2.77522  2.73126  3.46356  2.40515  3.72497
 3.29283  2.67743  2.69357  4.24692  2.90349  2.73742  3.18149  2.89803
 2.37889  2.77522  2.98521  4.58479  3.61506
           0.02697  3.67034  6.77560  0.27047  1.43979  0.48576  0.95510
      11   2.21801  5.38396  3.41424  2.09647  5.30755  4.04297  3.45011
 4.61915  1.59858  3.83470  3.98658  2.71782  4.61537  2.32242  2.46784
 2.40309  3.37006  3.49080  6.39356  4.75108      12 k - - C
           2.68638  4.42245  2.77540  2.73143  3.46374  2.40458  3.72515
 3.29374  2.67761  2.69269  4.24710  2.90252  2.73760  3.18166  2.89821
 2.37907  2.77540  2.98538  4.58497  3.61523
           0.09153  2.44963  6.77560  0.69897  0.68735  0.48576  0.95510
      12   3.42527  5.39824  0.80142  2.85094  5.47892  2.79033  4.07357
 4.97088  3.20539  4.44335  5.18856  1.82435  4.74315  3.49767  3.72792
 3.13380  3.85037  4.52876  6.57284  4.86832      16 d - - -
           2.68618  4.42225  2.77519  2.73123  3.46354  2.40513  3.72494
 3.29354  2.67741  2.69355  4.24690  2.90347  2.73739  3.18146  2.89801
 2.37887  2.77519  2.98518  4.58477  3.61503
           0.00350  6.05326  6.77560  0.61958  0.77255  0.48576  0.95510
      13   3.46957  5.17216  5.33801  4.79164  1.25276  4.65605  5.23830
 3.73815  4.62170  3.48380  3.91327  4.86488  0.86138  4.84105  4.18640
 3.77836  3.16107  3.35716  5.89740  4.70488      17 p - - H
           2.68618  4.42225  2.77519  2.73123  3.46354  2.40513  3.72494
 3.29354  2.67741  2.69355  4.24690  2.90347  2.73739  3.18146  2.89801
 2.37887  2.77519  2.98518  4.58477  3.61503
           0.00350  6.05326  6.77560  0.61958  0.77255  0.48576  0.95510
      14   3.17734  5.10121  4.47197  3.41126  3.23357  4.09632  4.87085
 1.88567  3.27801  1.54529  2.51347  3.05244  3.72625  3.57239  4.03927
 2.80956  3.25376  3.12840  5.70257  2.32897      18 l - - H
           2.68618  4.42225  2.77519  2.73123  3.46354  2.40513  3.72494
 3.29354  2.67741  2.69355  4.24690  2.90347  2.73739  3.18146  2.89801
 2.37887  2.77519  2.98518  4.58477  3.61503
           0.00350  6.05326  6.77560  0.61958  0.77255  0.48576  0.95510
      15   2.00378  5.96179  3.10564  2.10326  5.30800  2.16513  3.76684
 4.30098  1.72690  3.74628  5.00215  3.16847  4.61531  2.85950  2.65558
 2.67934  3.09064  3.70115  6.39378  4.48799      19 k - - H
           2.68618  4.42225  2.77519  2.73123  3.46354  2.40513  3.72494
 3.29354  2.67741  2.69355  4.24690  2.90347  2.73739  3.18146  2.89801
 2.37887  2.77519  2.98518  4.58477  3.61503
           0.00350  6.05326  6.77560  0.61958  0.77255  0.48576  0.95510
      16   6.32144  7.07473  6.95464  7.02012  1.68958  6.61507  5.09679
 5.78594  6.77396  3.63964  6.25987  6.07103  6.84185  6.28191  6.47613
 6.04179  6.49228  5.78303  5.15681  0.28562      20 Y - - H
           2.68618  4.42225  2.77519  2.73123  3.46354  2.40513  3.72494
 3.29354  2.67741  2.69355  4.24690  2.90347  2.73739  3.18146  2.89801
 2.37887  2.77519  2.98518  4.58477  3.61503
           0.00350  6.05326  6.77560  0.61958  0.77255  0.48576  0.95510
      17   3.47481  4.62636  5.58519  4.97012  3.23971  4.78136  5.11730
 0.93680  4.75328  1.69132  2.46692  3.92316  5.14147  4.88000  4.75641
 4.09439  3.83762  2.11469  4.36374  4.42808      21 i - - H
           2.68618  4.42225  2.77519  2.73123  3.46354  2.40513  3.72494
 3.29354  2.67741  2.69355  4.24690  2.90347  2.73739  3.18146  2.89801
 2.37887  2.77519  2.98518  4.58477  3.61503
           0.00350  6.05326  6.77560  0.61958  0.77255  0.48576  0.95510
```

18 2.21317 4.52090 2.82350 1.61202 5.02482 3.93816 4.15065
4.60303 2.08859 4.00952 4.60658 2.68506 4.61530 2.81103 2.77761
1.85730 3.40789 3.96962 6.39380 4.98215 22 e - - H
2.68618 4.42225 2.77519 2.73123 3.46354 2.40513 3.72494
3.29354 2.67741 2.69355 4.24690 2.90347 2.73739 3.18146 2.89801
2.37887 2.77519 2.98518 4.58477 3.61503
0.00350 6.05326 6.77560 0.61958 0.77255 0.48576 0.95510
19 2.86379 5.93160 3.60649 2.65549 4.22057 4.22875 3.85082
4.52751 1.51185 3.92769 4.07278 3.55681 4.62193 2.79027 2.31653
1.71129 3.38373 3.67201 3.40097 2.94450 23 k - - H
2.68618 4.42225 2.77519 2.73123 3.46354 2.40513 3.72494
3.29354 2.67741 2.69355 4.24690 2.90347 2.73739 3.18146 2.89801
2.37887 2.77519 2.98518 4.58477 3.61503
0.00854 6.05326 5.09130 0.61958 0.77255 0.48576 0.95510
20 3.36738 4.95252 4.93007 3.88941 3.86422 4.39212 5.04011
0.91517 4.02284 2.33865 2.53410 4.51690 3.23096 4.35811 4.16528
3.87737 3.18742 2.03311 5.57231 3.84818 24 i - - H
2.68618 4.42225 2.77519 2.73123 3.46354 2.40513 3.72494
3.29354 2.67741 2.69355 4.24690 2.90347 2.73739 3.18146 2.89801
2.37887 2.77519 2.98518 4.58477 3.61503
0.00352 6.04824 6.77058 0.61958 0.77255 0.52546 0.89474
21 2.18759 4.94624 2.64904 1.58281 5.30445 3.94784 3.43389
4.79119 2.66429 4.11747 4.38275 2.81454 4.17459 3.35891 1.75872
2.47185 3.21057 3.66295 6.39033 4.97872 25 e - - H
2.68618 4.42225 2.77519 2.73123 3.46354 2.40513 3.72494
3.29354 2.67741 2.69355 4.24690 2.90347 2.73739 3.18146 2.89801
2.37887 2.77519 2.98518 4.58477 3.61503
0.00352 6.04824 6.77058 0.61958 0.77255 0.52546 0.89474
22 2.55584 4.76111 2.54363 2.59184 4.23914 3.84136 3.61882
2.97799 2.03272 3.88151 4.99051 3.44335 1.93665 2.76092 3.10076
2.10628 3.58175 3.27059 6.38357 4.21458 26 p - - H
2.68604 4.42281 2.77518 2.73156 3.46410 2.40445 3.72432
3.29390 2.67711 2.69373 4.24746 2.90376 2.73725 3.18016 2.89754
2.37923 2.77575 2.98558 4.58533 3.61520
0.23243 1.57868 6.77058 0.83585 0.56829 0.46172 0.99479
23 3.18075 4.94107 3.72061 2.05340 4.11499 4.27909 3.56703
2.81036 2.57979 2.01001 4.58153 2.67338 4.07302 2.41607 3.02029
3.30693 3.53229 3.18208 3.89489 2.22243 31 l - - H
2.68618 4.42225 2.77519 2.73123 3.46354 2.40513 3.72494
3.29354 2.67741 2.69355 4.24690 2.90347 2.73739 3.18146 2.89801
2.37887 2.77519 2.98518 4.58477 3.61503
0.00350 6.05326 6.77560 0.61958 0.77255 0.48576 0.95510
24 1.39093 4.20597 4.89271 4.33294 4.25882 0.90411 4.96392
3.61437 4.23771 3.04780 4.26123 4.32166 4.99329 4.47727 4.46301
3.15945 3.63094 2.90588 5.70849 4.08996 32 g - - H
2.68618 4.42225 2.77519 2.73123 3.46354 2.40513 3.72494
3.29354 2.67741 2.69355 4.24690 2.90347 2.73739 3.18146 2.89801
2.37887 2.77519 2.98518 4.58477 3.61503
0.00350 6.05326 6.77560 0.61958 0.77255 0.48576 0.95510
25 1.95877 4.80900 3.51035 1.56737 5.28896 3.51522 3.78225
3.80106 2.15085 3.51607 2.58177 3.43873 4.61815 3.08210 2.50335
2.79134 3.19334 4.18913 6.38417 3.82527 33 e - - C
2.68630 4.42246 2.77527 2.73144 3.46375 2.40534 3.71953
3.29375 2.67762 2.69317 4.24643 2.90356 2.73761 3.18153 2.89822
2.37908 2.77541 2.98540 4.58498 3.61524
0.16268 1.90384 6.77560 0.24861 1.51359 0.48576 0.95510

26 2.83504 5.96216 3.08773 2.24948 5.30855 4.05013 3.60165
4.79544 1.32001 3.88564 4.74968 2.85970 2.99246 2.43395 2.10045
3.11267 3.44028 3.88586 6.39406 4.06686 36 k - - C
2.68618 4.42225 2.77519 2.73123 3.46354 2.40513 3.72494
3.29354 2.67741 2.69355 4.24690 2.90347 2.73739 3.18146 2.89801
2.37887 2.77519 2.98518 4.58477 3.61503
0.00350 6.05326 6.77560 0.61958 0.77255 0.48576 0.95510
27 2.19566 3.90904 5.08937 4.49885 2.25610 3.71235 3.12459
3.15686 4.36700 3.20376 4.12530 4.26103 5.03486 4.57464 4.11498
2.02238 3.30121 3.26194 5.01615 1.18795 37 y - - C
2.68618 4.42225 2.77519 2.73123 3.46354 2.40513 3.72494
3.29354 2.67741 2.69355 4.24690 2.90347 2.73739 3.18146 2.89801
2.37887 2.77519 2.98518 4.58477 3.61503
0.00350 6.05326 6.77560 0.61958 0.77255 0.48576 0.95510
28 6.37227 7.55268 7.08715 7.17437 7.83374 0.01286 7.82652
8.08151 7.41994 7.28898 8.49308 7.31330 6.87371 7.66968 7.24332
6.64184 6.89506 7.46305 8.25762 8.06289 38 G - - S
2.68618 4.42225 2.77519 2.73123 3.46354 2.40513 3.72494
3.29354 2.67741 2.69355 4.24690 2.90347 2.73739 3.18146 2.89801
2.37887 2.77519 2.98518 4.58477 3.61503
0.00350 6.05326 6.77560 0.61958 0.77255 0.48576 0.95510
29 3.16441 5.83945 6.74330 6.23564 4.88167 6.18292 6.75534
0.55553 6.14386 1.94115 2.36871 6.35701 6.36477 6.25734 6.20940
5.59176 4.88000 2.25057 6.97457 5.85525 39 I - - E
2.68618 4.42225 2.77519 2.73123 3.46354 2.40513 3.72494
3.29354 2.67741 2.69355 4.24690 2.90347 2.73739 3.18146 2.89801
2.37887 2.77519 2.98518 4.58477 3.61503
0.00350 6.05326 6.77560 0.61958 0.77255 0.48576 0.95510
30 1.87447 1.32887 5.80214 5.20620 3.63317 4.43562 5.40633
2.17349 5.00725 2.81812 4.28593 5.20013 5.38217 5.14974 5.02662
4.35546 4.04054 1.30254 5.88650 4.70513 40 v - - E
2.68618 4.42225 2.77519 2.73123 3.46354 2.40513 3.72494
3.29354 2.67741 2.69355 4.24690 2.90347 2.73739 3.18146 2.89801
2.37887 2.77519 2.98518 4.58477 3.61503
0.00350 6.05326 6.77560 0.61958 0.77255 0.48576 0.95510
31 4.76915 4.57427 5.47935 4.48257 6.46653 5.30763 4.59963
5.69702 0.31747 4.36343 5.89254 3.86573 5.60224 3.93229 2.04411
4.73302 4.85389 4.79884 6.93962 5.88441 41 K - - E
2.68618 4.42225 2.77519 2.73123 3.46354 2.40513 3.72494
3.29354 2.67741 2.69355 4.24690 2.90347 2.73739 3.18146 2.89801
2.37887 2.77519 2.98518 4.58477 3.61503
0.01730 6.05326 4.21275 0.61958 0.77255 0.48576 0.95510
32 4.77364 5.90779 6.98793 6.58834 5.39054 6.62992 7.72895
0.60798 6.62736 2.57402 5.03632 6.80483 6.75167 6.94056 6.86682
6.14767 5.03838 1.08776 7.80449 6.55424 42 I - - E
2.68618 4.42225 2.77519 2.73123 3.46354 2.40513 3.72494
3.29354 2.67741 2.69355 4.24690 2.90347 2.73739 3.18146 2.89801
2.37887 2.77519 2.98518 4.58477 3.61503
0.01162 6.03950 4.69130 0.61958 0.77255 0.59054 0.80749
33 3.51798 4.45331 5.51581 4.90624 4.10378 4.78913 5.12579
1.09293 3.54830 3.00739 4.11847 4.92735 5.15060 4.50991 3.75593
3.89462 3.69044 0.97016 5.64289 4.46420 43 v - - -
2.68618 4.42225 2.77519 2.73123 3.46354 2.40513 3.72494
3.29354 2.67741 2.69355 4.24690 2.90347 2.73739 3.18146 2.89801
2.37887 2.77519 2.98518 4.58477 3.61503
0.12276 6.03145 2.17928 0.61958 0.77255 0.64652 0.74206

```
     34   6.42407  7.48418  6.95676  7.03764  7.63864  6.09398  7.68037
7.93392  7.24509  7.11571  8.37132  7.23914  0.01505  7.53871  7.07970
6.70522  6.92364  7.40954  8.09072  7.84720     44 P - - -
          2.68618  4.42225  2.77519  2.73123  3.46354  2.40513  3.72494
3.29354  2.67741  2.69355  4.24690  2.90347  2.73739  3.18146  2.89801
2.37887  2.77519  2.98518  4.58477  3.61503
          0.00271  5.91140        *  0.61958  0.77255  0.00000        *
//
HMMER3/f [3.3 | Nov 2019]
NAME  K-box
ACC   PF01486.18
DESC  K-box region
LENG  93
ALPH  amino
RF    no
MM    no
CONS  yes
CS    no
MAP   yes
DATE  Thu Apr 16 06:08:25 2020
NSEQ  168
EFFN  3.863159
CKSUM 2092162593
GA    8.65 8.65
STATS LOCAL MSV       -9.2423  0.71836
STATS LOCAL VITERBI  -10.1089  0.71836
STATS LOCAL FORWARD   -4.0118  0.71836
HMM          A        C        D        E        F        G        H
I        K        L        M        N        P        Q        R        S
T        V        W        Y
            m->m     m->i     m->d     i->m     i->i     d->m     d->d
  COMPO   2.76831  4.69257  3.00826  2.24994  4.00779  3.25376  3.56579
3.03026  2.36673  2.17393  3.56701  2.96675  4.03029  2.65009  2.65946
2.64782  2.96813  3.04796  5.18684  3.96184
          2.68618  4.42225  2.77519  2.73123  3.46354  2.40513  3.72494
3.29354  2.67741  2.69355  4.24690  2.90347  2.73739  3.18146  2.89801
2.37887  2.77519  2.98518  4.58477  3.61503
          0.28741  5.12993  1.41109  0.61958  0.77255  0.00000        *
      1   2.32253  5.10728  2.81216  2.33469  4.49963  3.22289  3.40136
3.94779  2.16640  3.51481  4.12768  2.97504  3.62906  2.55282  2.46976
2.44628  2.34624  3.31445  5.74726  4.34098      1 k - - -
          2.68618  4.42225  2.77519  2.73123  3.46354  2.40513  3.72494
3.29354  2.67741  2.69355  4.24690  2.90347  2.73739  3.18146  2.89801
2.37887  2.77519  2.98518  4.58477  3.61503
          0.01165  4.85417  5.57652  0.61958  0.77255  0.88356  0.53326
      2   2.46289  4.53168  2.90395  2.43796  3.89265  3.67340  3.87790
3.17584  2.60368  2.94538  3.87494  2.88655  3.17859  2.86018  3.11341
2.40779  2.03137  2.70077  5.53644  4.20327      2 t - - -
          2.68618  4.42225  2.77519  2.73123  3.46354  2.40513  3.72494
3.29354  2.67741  2.69355  4.24690  2.90347  2.73739  3.18146  2.89801
2.37887  2.77519  2.98518  4.58477  3.61503
          0.01111  4.90143  5.62377  0.61958  0.77255  0.34122  1.24099
      3   2.61475  5.36169  2.68261  2.28045  4.67908  3.24640  3.83965
3.40402  2.47156  2.83052  3.22222  2.73642  3.75491  2.79319  3.03462
1.92053  2.69753  3.29957  5.81131  4.27310      3 s - - -
          2.68618  4.42225  2.77519  2.73123  3.46354  2.40513  3.72494
3.29354  2.67741  2.69355  4.24690  2.90347  2.73739  3.18146  2.89801
2.37887  2.77519  2.98518  4.58477  3.61503
```

```
          0.00910  5.10023  5.82258  0.61958  0.77255  0.43344  1.04490
      4   2.77817  4.87658  2.61631  1.76794  4.78474  2.62555  2.95643
4.03155  2.45230  3.60344  4.48119  2.95292  3.34244  2.34550  2.99303
2.55887  2.85837  3.66934  5.10473  4.46290      4 e - - -
          2.68618  4.42225  2.77519  2.73123  3.46354  2.40513  3.72494
3.29354  2.67741  2.69355  4.24690  2.90347  2.73739  3.18146  2.89801
2.37887  2.77519  2.98518  4.58477  3.61503
          0.00883  5.12993  5.85228  0.61958  0.77255  0.48576  0.95510
      5   2.57581  5.42560  2.64019  2.17337  4.76366  3.70804  2.96154
3.17395  2.41929  3.45865  4.46810  2.95980  3.93639  2.14322  3.08054
2.27627  2.55444  3.10649  5.86249  4.32461      5 q - - -
          2.68618  4.42225  2.77519  2.73123  3.46354  2.40513  3.72494
3.29354  2.67741  2.69355  4.24690  2.90347  2.73739  3.18146  2.89801
2.37887  2.77519  2.98518  4.58477  3.61503
          0.00883  5.12993  5.85228  0.61958  0.77255  0.48576  0.95510
      6   2.75134  5.16128  2.18177  2.58157  4.09522  3.70783  2.95534
3.86036  2.27905  3.41486  4.25255  2.33784  3.68994  2.86236  2.67173
2.28170  2.50002  3.69924  5.86323  3.98573      6 d - - -
          2.68589  4.42257  2.77499  2.73140  3.46386  2.40531  3.72489
3.29336  2.67772  2.69308  4.24721  2.90373  2.73771  3.18178  2.89832
2.37880  2.77537  2.98515  4.57783  3.61527
          0.26667  1.73155  2.86352  1.28694  0.32312  0.48576  0.95510
      7   2.30921  5.05817  2.53546  2.14347  4.73144  3.62532  2.86353
3.41849  2.26041  3.53174  3.76216  3.03623  3.98249  2.25312  3.10208
2.43265  2.90114  3.18329  5.83392  4.42862     11 e - - -
          2.68619  4.42226  2.77520  2.73124  3.46355  2.40513  3.72495
3.29355  2.67741  2.69356  4.24690  2.90347  2.73740  3.18140  2.89792
2.37887  2.77520  2.98519  4.58478  3.61504
          0.07561  3.67893  3.04559  0.44195  1.02942  0.39711  1.11553
      8   2.56190  5.37048  2.92027  2.22910  4.69369  3.62326  2.90233
3.74504  2.55478  3.10010  4.12374  2.73057  3.78495  2.31524  2.80956
2.34858  2.98605  2.98176  5.13901  2.92051     13 e - - -
          2.68618  4.42225  2.77519  2.73123  3.46354  2.40513  3.72494
3.29354  2.67741  2.69355  4.24690  2.90347  2.73739  3.18146  2.89801
2.37887  2.77519  2.98518  4.58477  3.61503
          0.10029  5.08690  2.41635  0.61958  0.77255  0.41390  1.08194
      9   2.86031  4.11425  2.70289  1.69680  4.74156  3.40909  3.51093
4.22572  2.24055  3.37160  4.21227  2.99820  3.73747  1.77830  3.01372
2.85783  3.06961  3.73693  5.83313  4.42376     14 e - - -
          2.68618  4.42225  2.77519  2.73123  3.46354  2.40513  3.72494
3.29354  2.67741  2.69355  4.24690  2.90347  2.73739  3.18146  2.89801
2.37887  2.77519  2.98518  4.58477  3.61503
          0.18600  5.04370  1.81233  0.61958  0.77255  0.72106  0.66600
     10   2.72238  4.87511  2.97765  2.19118  3.09911  3.61223  3.46654
3.84032  2.29372  3.33416  4.06066  2.33768  4.00480  2.50321  2.70719
2.49043  3.02073  3.37129  5.70101  2.94250     15 e - - -
          2.68618  4.42225  2.77519  2.73123  3.46354  2.40513  3.72494
3.29354  2.67741  2.69355  4.24690  2.90347  2.73739  3.18146  2.89801
2.37887  2.77519  2.98518  4.58477  3.61503
          0.01147  4.86918  5.59153  0.61958  0.77255  0.27033  1.44024
     11   2.89612  4.01890  4.51328  3.42479  2.83847  3.98492  4.43622
2.50124  3.87252  1.86635  2.40797  3.83323  4.24532  4.12441  3.44236
2.89415  3.06375  2.47785  2.05537  3.02233     16 l - - -
          2.68618  4.42225  2.77519  2.73123  3.46354  2.40513  3.72494
3.29354  2.67741  2.69355  4.24690  2.90347  2.73739  3.18146  2.89801
2.37887  2.77519  2.98518  4.58477  3.61503
          0.00890  5.12159  5.84394  0.61958  0.77255  0.46965  0.98143
```

```
     12   2.93393  3.60825  3.23022  2.49800  4.45042  3.65232  3.82648
3.98129  1.98916  3.09212  4.13251  3.05018  4.00325  1.94394  2.59380
2.31684  2.96644  3.37926  5.79172  2.97736     17 q - - -
          2.68618  4.42225  2.77519  2.73123  3.46354  2.40513  3.72494
3.29354  2.67741  2.69355  4.24690  2.90347  2.73739  3.18146  2.89801
2.37887  2.77519  2.98518  4.58477  3.61503
          0.00883  5.12993  5.85228  0.61958  0.77255  0.48576  0.95510
     13   2.43503  4.82512  2.94965  2.19956  4.56868  3.56155  3.31102
4.11532  2.48578  3.26604  3.97609  2.39905  4.09992  2.00003  2.26335
2.61779  3.05284  3.76354  5.86862  4.10440     18 q - - -
          2.68618  4.42225  2.77519  2.73123  3.46354  2.40513  3.72494
3.29354  2.67741  2.69355  4.24690  2.90347  2.73739  3.18146  2.89801
2.37887  2.77519  2.98518  4.58477  3.61503
          0.00883  5.12993  5.85228  0.61958  0.77255  0.48576  0.95510
     14   3.32928  5.90636  2.61521  0.70354  5.22243  3.83044  3.77609
4.73136  2.99331  4.19775  4.97709  2.59478  4.35206  3.22791  3.47007
3.24546  3.16700  4.28561  6.33282  4.86798     19 e - - -
          2.68618  4.42225  2.77519  2.73123  3.46354  2.40513  3.72494
3.29354  2.67741  2.69355  4.24690  2.90347  2.73739  3.18146  2.89801
2.37887  2.77519  2.98518  4.58477  3.61503
          0.00883  5.12993  5.85228  0.61958  0.77255  0.48576  0.95510
     15   1.93958  4.37691  4.42497  4.15258  3.33707  4.02453  3.79818
2.05388  3.76258  1.90403  3.04599  4.22211  4.48640  4.14016  4.04700
2.92949  2.96746  1.90784  4.99573  2.61975     20 l - - -
          2.68618  4.42225  2.77519  2.73123  3.46354  2.40513  3.72494
3.29354  2.67741  2.69355  4.24690  2.90347  2.73739  3.18146  2.89801
2.37887  2.77519  2.98518  4.58477  3.61503
          0.00883  5.12993  5.85228  0.61958  0.77255  0.48576  0.95510
     16   2.28467  5.37179  2.59480  2.29716  4.38994  3.52107  3.90847
3.25417  2.32309  2.77041  3.94387  2.99403  3.85294  3.01495  2.76649
2.13559  2.37786  3.62724  5.82218  4.26871     21 s - - -
          2.68618  4.42225  2.77519  2.73123  3.46354  2.40513  3.72494
3.29354  2.67741  2.69355  4.24690  2.90347  2.73739  3.18146  2.89801
2.37887  2.77519  2.98518  4.58477  3.61503
          0.00883  5.12993  5.85228  0.61958  0.77255  0.48576  0.95510
     17   2.76900  5.10303  2.94448  2.59079  3.32948  3.49544  3.63897
3.72860  1.56611  3.46620  3.69645  2.97490  4.11785  3.02996  2.10586
2.55230  2.88320  3.42481  5.06678  4.41087     22 k - - -
          2.68618  4.42225  2.77519  2.73123  3.46354  2.40513  3.72494
3.29354  2.67741  2.69355  4.24690  2.90347  2.73739  3.18146  2.89801
2.37887  2.77519  2.98518  4.58477  3.61503
          0.00883  5.12993  5.85228  0.61958  0.77255  0.48576  0.95510
     18   4.00987  5.25448  5.97803  5.39652  3.72512  5.36763  5.70876
2.03653  5.24860  0.56628  2.54801  5.52950  5.49425  5.19628  5.21991
4.73745  3.58237  2.41417  5.84190  4.83589     23 L - - -
          2.68618  4.42225  2.77519  2.73123  3.46354  2.40513  3.72494
3.29354  2.67741  2.69355  4.24690  2.90347  2.73739  3.18146  2.89801
2.37887  2.77519  2.98518  4.58477  3.61503
          0.00883  5.12993  5.85228  0.61958  0.77255  0.48576  0.95510
     19   2.67718  5.37748  3.22535  2.66718  4.69504  3.26668  3.86279
4.05044  1.38408  3.24018  3.99336  3.13812  4.12027  2.91913  2.06375
2.46187  2.87646  3.62570  5.82538  4.43664     24 k - - -
          2.68618  4.42225  2.77519  2.73123  3.46354  2.40513  3.72494
3.29354  2.67741  2.69355  4.24690  2.90347  2.73739  3.18146  2.89801
2.37887  2.77519  2.98518  4.58477  3.61503
          0.00883  5.12993  5.85228  0.61958  0.77255  0.48576  0.95510
```

20 2.29508 5.44341 2.44328 2.38944 4.78900 3.54698 3.42743
4.27529 1.90107 3.74888 4.26020 2.86911 4.09862 2.05061 2.70830
2.45136 3.00155 3.61460 5.87575 4.46454 25 k - - -
2.68618 4.42225 2.77519 2.73123 3.46354 2.40513 3.72494
3.29354 2.67741 2.69355 4.24690 2.90347 2.73739 3.18146 2.89801
2.37887 2.77519 2.98518 4.58477 3.61503
0.00883 5.12993 5.85228 0.61958 0.77255 0.48576 0.95510
21 2.93332 5.43010 3.06502 1.81956 4.76989 3.70891 3.37010
3.34575 1.86664 3.73281 3.81511 3.12880 4.10216 2.03576 2.54660
2.89819 2.88202 3.33416 5.86565 4.45866 26 e - - -
2.68618 4.42225 2.77519 2.73123 3.46354 2.40513 3.72494
3.29354 2.67741 2.69355 4.24690 2.90347 2.73739 3.18146 2.89801
2.37887 2.77519 2.98518 4.58477 3.61503
0.00883 5.12993 5.85228 0.61958 0.77255 0.48576 0.95510
22 2.90120 3.68135 4.80769 4.20093 3.21628 4.12789 4.26026
1.54139 3.89508 1.82838 3.38428 2.41118 4.49287 4.16898 3.76166
3.43342 3.03539 2.17321 4.98846 2.86831 27 i - - -
2.68618 4.42225 2.77519 2.73123 3.46354 2.40513 3.72494
3.29354 2.67741 2.69355 4.24690 2.90347 2.73739 3.18146 2.89801
2.37887 2.77519 2.98518 4.58477 3.61503
0.00883 5.12993 5.85228 0.61958 0.77255 0.48576 0.95510
23 2.66734 5.44342 2.17428 1.45273 4.78845 3.60307 3.30272
4.19253 2.55536 3.74866 4.23979 2.66051 4.09947 2.67177 2.69472
2.71839 3.09197 3.67434 5.87605 4.35935 28 e - - -
2.68618 4.42225 2.77519 2.73123 3.46354 2.40513 3.72494
3.29354 2.67741 2.69355 4.24690 2.90347 2.73739 3.18146 2.89801
2.37887 2.77519 2.98518 4.58477 3.61503
0.00883 5.12993 5.85228 0.61958 0.77255 0.48576 0.95510
24 2.63002 4.87135 2.89015 2.48625 4.36485 3.67961 3.68686
3.83034 2.24536 2.52077 3.93526 2.31749 4.11656 2.84653 2.55028
2.30412 2.97466 2.72833 5.79924 3.87545 29 k - - -
2.68618 4.42225 2.77519 2.73123 3.46354 2.40513 3.72494
3.29354 2.67741 2.69355 4.24690 2.90347 2.73739 3.18146 2.89801
2.37887 2.77519 2.98518 4.58477 3.61503
0.00883 5.12993 5.85228 0.61958 0.77255 0.48576 0.95510
25 3.09351 4.49210 4.86072 4.26024 3.40771 4.25035 4.57512
2.42598 3.83920 0.82743 2.88120 4.34589 4.60209 4.24630 4.16228
2.69039 3.18412 2.61552 5.10830 3.93996 30 l - - -
2.68618 4.42225 2.77519 2.73123 3.46354 2.40513 3.72494
3.29354 2.67741 2.69355 4.24690 2.90347 2.73739 3.18146 2.89801
2.37887 2.77519 2.98518 4.58477 3.61503
0.00883 5.12993 5.85228 0.61958 0.77255 0.48576 0.95510
26 2.95872 5.44074 3.16141 1.70979 4.78009 3.72940 3.83147
4.25596 2.59587 3.66363 4.32503 3.11035 4.12317 1.42105 2.44983
2.88442 3.08734 3.36604 5.87296 4.47415 31 q - - -
2.68618 4.42225 2.77519 2.73123 3.46354 2.40513 3.72494
3.29354 2.67741 2.69355 4.24690 2.90347 2.73739 3.18146 2.89801
2.37887 2.77519 2.98518 4.58477 3.61503
0.00883 5.12993 5.85228 0.61958 0.77255 0.48576 0.95510
27 2.17223 5.42783 2.81949 2.35606 4.57897 3.63779 3.43220
3.27547 2.16876 3.21123 4.26164 2.76603 4.10098 2.53259 2.04873
2.80211 2.80457 3.56251 5.86414 4.45688 32 r - - -
2.68618 4.42225 2.77519 2.73123 3.46354 2.40513 3.72494
3.29354 2.67741 2.69355 4.24690 2.90347 2.73739 3.18146 2.89801
2.37887 2.77519 2.98518 4.58477 3.61503
0.00883 5.12993 5.85228 0.61958 0.77255 0.48576 0.95510

```
     28   2.78320  4.55275  3.12074  2.08194  4.59460  3.20542  3.39106
4.03607  2.49494  3.52379  4.46457  2.64012  4.10203  2.45379  3.02720
1.82658  2.28434  3.80760  5.85954  3.76557      33 s - - -
          2.68618  4.42225  2.77519  2.73123  3.46354  2.40513  3.72494
3.29354  2.67741  2.69355  4.24690  2.90347  2.73739  3.18146  2.89801
2.37887  2.77519  2.98518  4.58477  3.61503
          0.00883  5.12993  5.85228  0.61958  0.77255  0.48576  0.95510
     29   2.93940  5.14569  3.26416  2.77868  4.38538  3.76957  2.89825
2.61898  2.20235  2.31053  3.60342  2.53857  4.15938  2.04308  3.14908
2.82037  3.05689  3.00088  5.64706  4.31075      34 q - - -
          2.68618  4.42225  2.77519  2.73123  3.46354  2.40513  3.72494
3.29354  2.67741  2.69355  4.24690  2.90347  2.73739  3.18146  2.89801
2.37887  2.77519  2.98518  4.58477  3.61503
          0.00883  5.12993  5.85228  0.61958  0.77255  0.48576  0.95510
     30   3.70626  5.89321  4.15966  3.28552  5.40711  4.04736  4.20114
4.72526  2.17245  3.50888  4.95756  3.71908  4.66460  2.61828  0.61680
3.38853  3.85095  4.38693  6.11153  4.95761      35 R - - -
          2.68618  4.42225  2.77519  2.73123  3.46354  2.40513  3.72494
3.29354  2.67741  2.69355  4.24690  2.90347  2.73739  3.18146  2.89801
2.37887  2.77519  2.98518  4.58477  3.61503
          0.00883  5.12993  5.85228  0.61958  0.77255  0.48576  0.95510
     31   2.93912  4.91323  3.20668  2.64964  3.93666  3.58172  1.95566
4.20376  1.96322  3.70021  4.44906  2.57172  4.11155  2.00114  2.74910
2.85852  3.10035  3.78505  5.84513  3.57329      36 h - - -
          2.68618  4.42225  2.77519  2.73123  3.46354  2.40513  3.72494
3.29354  2.67741  2.69355  4.24690  2.90347  2.73739  3.18146  2.89801
2.37887  2.77519  2.98518  4.58477  3.61503
          0.00883  5.12993  5.85228  0.61958  0.77255  0.48576  0.95510
     32   2.98433  4.17213  4.75983  4.15639  2.93973  4.13232  4.45434
2.54437  3.82984  1.10393  2.47055  4.23033  4.49644  3.56613  2.76566
3.43533  3.21625  2.63532  5.00584  3.09505      37 l - - -
          2.68618  4.42225  2.77519  2.73123  3.46354  2.40513  3.72494
3.29354  2.67741  2.69355  4.24690  2.90347  2.73739  3.18146  2.89801
2.37887  2.77519  2.98518  4.58477  3.61503
          0.00883  5.12993  5.85228  0.61958  0.77255  0.48576  0.95510
     33   2.85117  4.44985  4.41311  3.82653  3.55384  4.06747  3.89946
2.64071  2.98349  1.38999  2.03662  3.91230  4.43722  3.50741  3.72552
3.26012  2.51882  2.46393  5.06051  3.41423      38 l - - -
          2.68618  4.42225  2.77519  2.73123  3.46354  2.40513  3.72494
3.29354  2.67741  2.69355  4.24690  2.90347  2.73739  3.18146  2.89801
2.37887  2.77519  2.98518  4.58477  3.61503
          0.00883  5.12993  5.85228  0.61958  0.77255  0.48576  0.95510
     34   3.16256  5.02057  4.72776  4.55867  4.94650  0.35411  5.39675
4.18474  4.49793  3.26500  5.06439  4.46872  4.67602  4.80789  4.66778
3.38768  3.68837  3.14018  6.46520  5.20693      39 G - - -
          2.68624  4.42231  2.77490  2.73130  3.46326  2.40495  3.72501
3.29360  2.67747  2.69361  4.24696  2.90353  2.73746  3.18153  2.89807
2.37881  2.77526  2.98525  4.58483  3.61509
          0.07045  2.94410  4.17493  0.92716  0.50365  0.48576  0.95510
     35   3.22188  5.78046  2.16673  0.88057  5.10503  3.79480  4.12160
4.60779  2.66004  4.07577  4.84139  3.13054  4.28424  2.57685  3.47130
3.15098  3.46972  4.16318  6.20764  3.80355      43 e - - -
          2.68620  4.42227  2.77521  2.73125  3.46355  2.40504  3.72496
3.29356  2.67742  2.69343  4.24691  2.90348  2.73741  3.18148  2.89802
2.37888  2.77521  2.98520  4.58479  3.61505
          0.02867  3.67491  5.83970  0.64720  0.74131  0.46191  0.99446
```

36 2.88743 4.07415 1.53559 2.11130 4.79295 2.66583 3.74234
4.27844 2.29120 3.75414 4.49118 2.85822 4.10546 2.84437 3.00069
2.56430 3.09599 3.83941 5.88296 4.47180 46 d - - -
2.68618 4.42225 2.77519 2.73123 3.46354 2.40513 3.72494
3.29354 2.67741 2.69355 4.24690 2.90347 2.73739 3.18146 2.89801
2.37887 2.77519 2.98518 4.58477 3.61503
0.00883 5.12993 5.85228 0.61958 0.77255 0.48576 0.95510
37 2.94527 4.43392 4.26467 4.22261 3.49771 3.81848 4.51582
2.26259 4.04516 0.83590 3.11404 4.29390 4.54965 4.20542 4.11443
3.49651 3.26743 2.55293 5.05345 3.32272 47 l - - -
2.68611 4.42227 2.77521 2.73125 3.46355 2.40514 3.72496
3.29344 2.67742 2.69356 4.24691 2.90341 2.73741 3.18148 2.89802
2.37888 2.77516 2.98520 4.58478 3.61505
0.07121 3.16643 3.62770 0.34295 1.23676 0.48576 0.95510
38 2.44404 5.43367 2.29050 2.03022 4.77647 2.32132 3.80974
4.13722 2.62477 3.73803 4.47525 2.44322 3.81826 2.81016 3.11791
2.12275 3.01079 3.73675 5.86755 4.19578 49 e - - -
2.68618 4.42225 2.77519 2.73123 3.46354 2.40513 3.72494
3.29354 2.67741 2.69355 4.24690 2.90347 2.73739 3.18146 2.89801
2.37887 2.77519 2.98518 4.58477 3.61503
0.00904 5.10594 5.82829 0.61958 0.77255 0.55912 0.84796
39 2.77819 5.05065 2.70639 2.47003 4.74980 2.58058 2.90738
4.23012 2.47666 3.55321 4.25574 3.02851 2.93956 2.92673 3.11551
1.60507 2.78899 3.75053 5.85038 4.44432 50 s - - -
2.68618 4.42225 2.77519 2.73123 3.46354 2.40513 3.72494
3.29354 2.67741 2.69355 4.24690 2.90347 2.73739 3.18146 2.89801
2.37887 2.77519 2.98518 4.58477 3.61503
0.00904 5.10594 5.82829 0.61958 0.77255 0.55912 0.84796
40 2.91657 2.33553 4.94142 4.33310 3.33919 4.21477 4.54855
2.69697 3.99518 1.08201 2.19714 4.35580 4.57022 4.27290 4.16696
3.36755 3.27706 2.52318 5.04723 3.87745 51 l - - -
2.68618 4.42225 2.77520 2.73124 3.46354 2.40513 3.72495
3.29354 2.67741 2.69355 4.24690 2.90347 2.73740 3.18147 2.89801
2.37881 2.77520 2.98519 4.58477 3.61503
0.02135 4.00755 5.82829 0.48966 0.94891 0.44248 1.02846
41 2.41095 5.42479 2.93916 2.60249 4.75837 2.87942 3.68502
4.23635 2.62117 3.72593 4.47057 2.45251 3.91304 2.87924 3.08451
1.49842 2.31968 3.80969 5.86601 4.46144 53 s - - -
2.68618 4.42225 2.77519 2.73123 3.46354 2.40513 3.72494
3.29354 2.67741 2.69355 4.24690 2.90347 2.73739 3.18146 2.89801
2.37887 2.77519 2.98518 4.58477 3.61503
0.00883 5.12993 5.85228 0.61958 0.77255 0.48576 0.95510
42 2.78710 4.35401 4.90215 4.28924 2.66984 3.97678 3.96814
2.11553 4.08191 1.46071 3.00774 4.29005 3.23794 4.21979 4.10258
3.19358 2.90242 1.86166 4.61435 3.40696 54 l - - -
2.68618 4.42225 2.77519 2.73123 3.46354 2.40513 3.72494
3.29354 2.67741 2.69355 4.24690 2.90347 2.73739 3.18146 2.89801
2.37887 2.77519 2.98518 4.58477 3.61503
0.00883 5.12993 5.85228 0.61958 0.77255 0.48576 0.95510
43 2.89561 5.44753 2.63978 1.85960 4.79365 3.41295 3.35155
4.17940 1.54863 3.75306 4.48807 2.90764 3.43745 2.52657 2.71056
2.84431 3.04959 3.83876 5.87930 4.46781 55 k - - -
2.68618 4.42225 2.77519 2.73123 3.46354 2.40513 3.72494
3.29354 2.67741 2.69355 4.24690 2.90347 2.73739 3.18146 2.89801
2.37887 2.77519 2.98518 4.58477 3.61503
0.00883 5.12993 5.85228 0.61958 0.77255 0.48576 0.95510

```
     44   3.33634  5.92355  1.71305  0.89581  5.23848  3.59212  3.94414
4.75176  3.09162  4.21679  4.99580  3.14016  4.35481  2.96643  3.64151
2.92221  3.59324  3.86851  6.35239  4.88034      56 e - - -
          2.68618  4.42225  2.77519  2.73123  3.46354  2.40513  3.72494
3.29354  2.67741  2.69355  4.24690  2.90347  2.73739  3.18146  2.89801
2.37887  2.77519  2.98518  4.58477  3.61503
          0.00883  5.12993  5.85228  0.61958  0.77255  0.48576  0.95510
     45   4.64430  5.77097  6.57107  5.99735  3.54491  6.13061  6.32230
2.86005  5.86907  0.25631  3.19766  6.31060  5.93384  5.54845  5.72864
5.59354  4.82409  3.11901  6.04807  5.15715      57 L - - -
          2.68618  4.42225  2.77519  2.73123  3.46354  2.40513  3.72494
3.29354  2.67741  2.69355  4.24690  2.90347  2.73739  3.18146  2.89801
2.37887  2.77519  2.98518  4.58477  3.61503
          0.00883  5.12993  5.85228  0.61958  0.77255  0.48576  0.95510
     46   2.93172  4.89257  3.01779  2.11512  4.77470  3.47742  2.69962
3.65937  2.15945  3.29411  3.76352  2.86032  4.10022  1.81456  2.51085
2.77524  3.16286  3.70081  5.86822  4.45963      58 q - - -
          2.68618  4.42225  2.77519  2.73123  3.46354  2.40513  3.72494
3.29354  2.67741  2.69355  4.24690  2.90347  2.73739  3.18146  2.89801
2.37887  2.77519  2.98518  4.58477  3.61503
          0.00883  5.12993  5.85228  0.61958  0.77255  0.48576  0.95510
     47   2.44123  5.43993  2.79490  2.03838  4.57439  2.93179  3.47323
3.97807  2.49709  3.47986  4.21673  2.63072  3.98564  1.98409  3.04803
2.20111  2.92411  3.83009  5.87314  4.46272      59 q - - -
          2.68618  4.42225  2.77519  2.73123  3.46354  2.40513  3.72494
3.29354  2.67741  2.69355  4.24690  2.90347  2.73739  3.18146  2.89801
2.37887  2.77519  2.98518  4.58477  3.61503
          0.00883  5.12993  5.85228  0.61958  0.77255  0.48576  0.95510
     48   4.51998  5.66097  6.49583  5.92892  3.51887  6.02442  6.28663
2.18324  5.82224  0.34297  3.43508  6.20616  5.88839  5.54329  5.70407
5.47165  4.71087  2.86828  6.06774  5.17126      60 L - - -
          2.68618  4.42225  2.77519  2.73123  3.46354  2.40513  3.72494
3.29354  2.67741  2.69355  4.24690  2.90347  2.73739  3.18146  2.89801
2.37887  2.77519  2.98518  4.58477  3.61503
          0.00883  5.12993  5.85228  0.61958  0.77255  0.48576  0.95510
     49   3.20982  5.70184  2.85515  0.68939  5.01805  3.61519  3.99873
4.50104  2.94066  3.99688  4.41223  3.27624  4.29001  2.95717  3.44852
3.15364  3.16598  3.94083  6.14828  4.72181      61 E - - -
          2.68618  4.42225  2.77519  2.73123  3.46354  2.40513  3.72494
3.29354  2.67741  2.69355  4.24690  2.90347  2.73739  3.18146  2.89801
2.37887  2.77519  2.98518  4.58477  3.61503
          0.00883  5.12993  5.85228  0.61958  0.77255  0.48576  0.95510
     50   2.90047  5.44131  2.88077  2.25244  4.78610  2.84573  3.12148
3.76737  2.26767  3.34627  4.13115  2.41073  4.09872  2.08643  2.39230
2.49999  3.10015  3.58974  5.87415  4.46340      62 q - - -
          2.68618  4.42225  2.77519  2.73123  3.46354  2.40513  3.72494
3.29354  2.67741  2.69355  4.24690  2.90347  2.73739  3.18146  2.89801
2.37887  2.77519  2.98518  4.58477  3.61503
          0.00883  5.12993  5.85228  0.61958  0.77255  0.48576  0.95510
     51   2.67602  5.43812  2.93467  2.39375  4.78120  3.58005  3.75393
4.26553  2.33648  3.59566  4.32450  2.65375  4.10041  1.40507  2.66144
2.85820  2.66755  3.82771  5.87182  4.02966      63 q - - -
          2.68618  4.42225  2.77519  2.73123  3.46354  2.40513  3.72494
3.29354  2.67741  2.69355  4.24690  2.90347  2.73739  3.18146  2.89801
2.37887  2.77519  2.98518  4.58477  3.61503
          0.00883  5.12993  5.85228  0.61958  0.77255  0.48576  0.95510
```

```
     52   4.37462  5.54523  6.36875  5.79290  3.72336  5.83958  6.14304
2.00813  5.67554  0.49146  2.53659  6.01993  5.78527  5.46039  5.58058
5.25738  4.57245  2.48247  6.02466  5.12102     64 L - - -
          2.68618  4.42225  2.77519  2.73123  3.46354  2.40513  3.72494
3.29354  2.67741  2.69355  4.24690  2.90347  2.73739  3.18146  2.89801
2.37887  2.77519  2.98518  4.58477  3.61503
          0.00883  5.12993  5.85228  0.61958  0.77255  0.48576  0.95510
     53   2.92680  5.49695  2.19576  1.09830  4.83620  3.60459  3.93795
4.32241  2.46339  3.71576  4.54151  2.96562  4.13674  2.89283  3.08919
2.94441  3.13958  3.51797  5.93042  4.35240     65 e - - -
          2.68618  4.42225  2.77519  2.73123  3.46354  2.40513  3.72494
3.29354  2.67741  2.69355  4.24690  2.90347  2.73739  3.18146  2.89801
2.37887  2.77519  2.98518  4.58477  3.61503
          0.00883  5.12993  5.85228  0.61958  0.77255  0.48576  0.95510
     54   2.39798  4.74603  3.23884  2.43844  4.39654  3.38446  3.22663
3.19133  2.11192  2.95750  3.29211  2.80596  3.95587  2.60064  2.81313
2.34359  2.68769  3.45155  5.77806  3.72520     66 k - - -
          2.68618  4.42225  2.77519  2.73123  3.46354  2.40513  3.72494
3.29354  2.67741  2.69355  4.24690  2.90347  2.73739  3.18146  2.89801
2.37887  2.77519  2.98518  4.58477  3.61503
          0.00883  5.12993  5.85228  0.61958  0.77255  0.48576  0.95510
     55   1.87104  4.90708  3.87570  3.35537  4.47804  1.92965  4.42613
3.87875  3.33232  3.54265  4.38347  3.72349  4.32773  3.65361  3.65519
1.24434  2.66435  3.51876  3.51252  4.57054     67 s - - -
          2.68618  4.42225  2.77519  2.73123  3.46354  2.40513  3.72494
3.29354  2.67741  2.69355  4.24690  2.90347  2.73739  3.18146  2.89801
2.37887  2.77519  2.98518  4.58477  3.61503
          0.00883  5.12993  5.85228  0.61958  0.77255  0.48576  0.95510
     56   3.02914  4.64119  5.22308  4.61299  3.36376  4.48171  4.80764
2.05805  4.41398  0.81069  2.90505  4.63552  4.79806  4.16236  4.42030
3.80048  3.51513  2.21123  4.64383  4.09517     68 l - - -
          2.68618  4.42225  2.77519  2.73123  3.46354  2.40513  3.72494
3.29354  2.67741  2.69355  4.24690  2.90347  2.73739  3.18146  2.89801
2.37887  2.77519  2.98518  4.58477  3.61503
          0.00883  5.12993  5.85228  0.61958  0.77255  0.48576  0.95510
     57   2.67114  5.08784  3.04977  2.49830  4.76583  3.11324  3.13741
3.92413  1.91894  3.50408  4.30357  2.62339  4.10109  2.96502  2.20569
2.20239  2.57369  3.60483  5.86361  3.86770     69 k - - -
          2.68618  4.42225  2.77519  2.73123  3.46354  2.40513  3.72494
3.29354  2.67741  2.69355  4.24690  2.90347  2.73739  3.18146  2.89801
2.37887  2.77519  2.98518  4.58477  3.61503
          0.00883  5.12993  5.85228  0.61958  0.77255  0.48576  0.95510
     58   2.83313  4.77083  3.13156  2.55577  4.75619  3.19425  3.19593
3.71797  2.02628  3.29722  4.10683  2.57922  4.10230  2.46650  2.13753
2.18783  2.89118  3.38207  5.85856  4.45326     70 k - - -
          2.68618  4.42225  2.77519  2.73123  3.46354  2.40513  3.72494
3.29354  2.67741  2.69355  4.24690  2.90347  2.73739  3.18146  2.89801
2.37887  2.77519  2.98518  4.58477  3.61503
          0.00883  5.12993  5.85228  0.61958  0.77255  0.48576  0.95510
     59   3.13046  4.89679  5.66341  5.14963  4.27912  5.08220  5.67105
0.85305  5.04069  2.93213  3.83548  5.24407  5.38275  5.26497  5.15953
4.24527  3.06362  1.14830  6.12222  4.90739     71 i - - -
          2.68618  4.42225  2.77519  2.73123  3.46354  2.40513  3.72494
3.29354  2.67741  2.69355  4.24690  2.90347  2.73739  3.18146  2.89801
2.37887  2.77519  2.98518  4.58477  3.61503
          0.00883  5.12993  5.85228  0.61958  0.77255  0.48576  0.95510
```

```
     60   4.00779  6.03356  4.64400  3.72567  5.57908  4.54326  3.06709
4.87685  2.24605  3.63766  5.11121  3.76037  4.85113  3.41240  0.47625
3.98217  4.10069  4.57977  6.15996  5.07939     72 R - - -
          2.68618  4.42225  2.77519  2.73123  3.46354  2.40513  3.72494
3.29354  2.67741  2.69355  4.24690  2.90347  2.73739  3.18146  2.89801
2.37887  2.77519  2.98518  4.58477  3.61503
          0.00883  5.12993  5.85228  0.61958  0.77255  0.48576  0.95510
     61   2.37505  5.16197  2.81537  2.13637  4.77187  2.94884  3.40202
4.25464  2.32245  3.53015  3.82627  2.98127  4.10037  2.62595  3.09019
1.74060  2.89238  3.34183  5.86678  4.45859     73 s - - -
          2.68618  4.42225  2.77519  2.73123  3.46354  2.40513  3.72494
3.29354  2.67741  2.69355  4.24690  2.90347  2.73739  3.18146  2.89801
2.37887  2.77519  2.98518  4.58477  3.61503
          0.00883  5.12993  5.85228  0.61958  0.77255  0.48576  0.95510
     62   2.80738  5.41527  3.27050  2.55007  4.74404  3.76180  3.45826
3.84521  1.87090  3.33861  4.07550  3.20154  4.15101  2.86659  1.36686
2.96020  2.64414  3.79791  5.84887  4.47082     74 r - - -
          2.68618  4.42225  2.77519  2.73123  3.46354  2.40513  3.72494
3.29354  2.67741  2.69355  4.24690  2.90347  2.73739  3.18146  2.89801
2.37887  2.77519  2.98518  4.58477  3.61503
          0.00883  5.12993  5.85228  0.61958  0.77255  0.48576  0.95510
     63   3.69893  6.02640  4.48275  3.62695  5.62323  4.48240  4.26128
4.88718  0.46378  3.74076  5.09952  3.97235  4.80009  2.76393  2.37563
3.89507  4.03765  4.57039  6.17671  5.08941     75 K - - -
          2.68601  4.42057  2.77535  2.73138  3.46369  2.40528  3.72418
3.29369  2.67756  2.69338  4.24551  2.90362  2.73755  3.18109  2.89816
2.37878  2.77535  2.98534  4.58492  3.61518
          0.05727  2.94145  5.85228  1.58415  0.22957  0.48576  0.95510
     64   2.38188  5.40778  2.50465  2.18098  3.85845  3.46001  3.32178
4.21432  2.48369  3.44923  2.96491  2.41249  4.10443  2.77425  3.00256
2.80499  2.32988  3.71936  5.84920  3.34552     83 e - - -
          2.68638  4.42245  2.77539  2.73143  3.46374  2.40533  3.72410
3.29306  2.67761  2.69300  4.24710  2.90321  2.73760  3.18166  2.89775
2.37852  2.77539  2.98538  4.58497  3.61523
          0.06431  2.82319  5.85228  1.73278  0.19455  0.48576  0.95510
     65   2.94191  5.04907  2.86164  2.03102  4.79767  3.71245  2.71003
4.28262  2.21241  3.66013  4.49215  2.91440  4.10655  1.52963  2.63269
2.87644  3.17264  3.84273  5.88157  4.47256     92 q - - -
          2.68618  4.42225  2.77519  2.73123  3.46354  2.40513  3.72494
3.29354  2.67741  2.69355  4.24690  2.90347  2.73739  3.18146  2.89801
2.37887  2.77519  2.98518  4.58477  3.61503
          0.00883  5.12993  5.85228  0.61958  0.77255  0.48576  0.95510
     66   2.65770  4.28319  4.82289  4.21518  3.16198  4.12996  3.82186
2.12285  3.03249  1.41947  3.16245  4.25405  4.49477  4.06899  4.07321
3.30624  3.12358  1.70774  4.98644  3.42139     93 l - - -
          2.68618  4.42225  2.77519  2.73123  3.46354  2.40513  3.72494
3.29354  2.67741  2.69355  4.24690  2.90347  2.73739  3.18146  2.89801
2.37887  2.77519  2.98518  4.58477  3.61503
          0.00883  5.12993  5.85228  0.61958  0.77255  0.48576  0.95510
     67   2.87978  4.35573  4.91043  4.29723  2.58132  4.14274  4.30886
2.04756  4.08867  1.40292  1.99024  3.75985  3.76083  4.22515  4.10723
3.45281  3.20872  2.55865  4.05819  3.43240     94 l - - -
          2.68618  4.42225  2.77519  2.73123  3.46354  2.40513  3.72494
3.29354  2.67741  2.69355  4.24690  2.90347  2.73739  3.18146  2.89801
2.37887  2.77519  2.98518  4.58477  3.61503
          0.05241  5.12993  3.09797  0.61958  0.77255  0.48576  0.95510
```

68 2.55917 4.62572 3.61413 2.60728 3.10365 3.17883 3.44979
2.70503 2.57621 2.15233 2.97926 3.29047 4.23813 3.11606 3.31837
2.31497 2.64612 2.92895 5.35395 3.73717 95 l - - -
2.68618 4.42225 2.77519 2.73123 3.46354 2.40513 3.72494
3.29354 2.67741 2.69355 4.24690 2.90347 2.73739 3.18146 2.89801
2.37887 2.77519 2.98518 4.58477 3.61503
0.00922 5.08675 5.80909 0.61958 0.77255 0.61303 0.78024
69 2.55308 5.43209 2.06974 1.69680 4.77730 3.32697 3.87847
4.26401 2.44552 3.73743 4.47301 2.59151 3.59671 2.39581 2.96711
2.76360 2.79476 3.73961 5.86438 4.45229 96 e - - -
2.68618 4.42225 2.77519 2.73123 3.46354 2.40513 3.72494
3.29354 2.67741 2.69355 4.24690 2.90347 2.73739 3.18146 2.89801
2.37887 2.77519 2.98518 4.58477 3.61503
0.00922 5.08675 5.80909 0.61958 0.77255 0.61303 0.78024
70 2.95326 5.45446 3.14107 1.58559 4.80263 3.71396 3.50025
4.28352 2.22258 3.75577 4.49693 3.18170 4.11062 1.62985 2.79212
2.58647 2.99343 3.84733 5.87983 4.47696 97 e - - -
2.68618 4.42225 2.77519 2.73123 3.46354 2.40513 3.72494
3.29354 2.67741 2.69355 4.24690 2.90347 2.73739 3.18146 2.89801
2.37887 2.77519 2.98518 4.58477 3.61503
0.00922 5.08675 5.80909 0.61958 0.77255 0.61303 0.78024
71 3.09202 4.51291 5.04574 4.45133 3.62060 4.36167 4.72039
1.02712 4.26963 1.81371 2.84221 4.49513 4.70866 4.42272 4.32021
3.24227 2.66601 2.23722 5.22211 4.04640 98 i - - -
2.68618 4.42225 2.77519 2.73123 3.46354 2.40513 3.72494
3.29354 2.67741 2.69355 4.24690 2.90347 2.73739 3.18146 2.89801
2.37887 2.77519 2.98518 4.58477 3.61503
0.00922 5.08675 5.80909 0.61958 0.77255 0.41369 1.08237
72 2.60171 3.95593 2.36029 1.57473 4.65002 3.32569 3.69230
4.09830 2.48189 3.45836 4.47720 2.95658 4.09965 2.61370 2.93792
2.18546 3.07738 3.77581 5.87011 4.36297 99 e - - -
2.68618 4.42225 2.77519 2.73123 3.46354 2.40513 3.72494
3.29354 2.67741 2.69355 4.24690 2.90347 2.73739 3.18146 2.89801
2.37887 2.77519 2.98518 4.58477 3.61503
0.00883 5.12993 5.85228 0.61958 0.77255 0.48576 0.95510
73 2.81905 5.42737 2.11908 1.91174 4.60218 3.54818 3.77981
4.24778 2.33300 3.29914 3.45975 2.86286 4.10103 2.41570 3.00915
2.78942 2.52012 3.76383 5.22499 3.12561 100 e - - -
2.68618 4.42225 2.77519 2.73123 3.46354 2.40513 3.72494
3.29354 2.67741 2.69355 4.24690 2.90347 2.73739 3.18146 2.89801
2.37887 2.77519 2.98518 4.58477 3.61503
0.00883 5.12993 5.85228 0.61958 0.77255 0.48576 0.95510
74 2.93085 3.53400 4.71046 4.10939 3.25801 4.11510 3.81073
2.82313 3.65713 0.94731 2.76086 4.19993 4.48114 3.92449 4.02797
2.87689 3.10060 2.60069 5.00340 3.41962 101 l - - -
2.68618 4.42225 2.77519 2.73123 3.46354 2.40513 3.72494
3.29354 2.67741 2.69355 4.24690 2.90347 2.73739 3.18146 2.89801
2.37887 2.77519 2.98518 4.58477 3.61503
0.00883 5.12993 5.85228 0.61958 0.77255 0.48576 0.95510
75 2.99488 5.49835 3.22570 2.72012 4.85839 3.78528 3.58264
4.32558 1.51895 3.79193 4.54183 2.84822 4.17289 2.05332 1.73668
2.92044 3.21108 3.67037 5.90579 4.10765 102 k - - -
2.68618 4.42225 2.77519 2.73123 3.46354 2.40513 3.72494
3.29354 2.67741 2.69355 4.24690 2.90347 2.73739 3.18146 2.89801
2.37887 2.77519 2.98518 4.58477 3.61503
0.00883 5.12993 5.85228 0.61958 0.77255 0.48576 0.95510

```
      76   2.71662  5.44205  3.10479  2.09245  4.78702  3.48757  3.57557
4.27261  1.72100  3.45096  4.48281  2.70647  3.61661  2.70639  1.94241
2.74755  2.96120  3.74559  5.56433  4.46416    103 k - - -
         2.68618  4.42225  2.77519  2.73123  3.46354  2.40513  3.72494
3.29354  2.67741  2.69355  4.24690  2.90347  2.73739  3.18146  2.89801
2.37887  2.77519  2.98518  4.58477  3.61503
         0.00883  5.12993  5.85228  0.61958  0.77255  0.48576  0.95510
      77   2.99338  5.44997  3.26799  2.45121  4.79231  3.76416  3.92877
3.67305  1.16963  3.74242  4.01116  2.82675  4.15316  2.74967  2.19020
2.96282  3.13246  3.42918  5.87359  4.49148    104 k - - -
         2.68609  4.42233  2.77528  2.73132  3.46362  2.40512  3.72503
3.29275  2.67749  2.69363  4.24698  2.90355  2.73748  3.18155  2.89809
2.37873  2.77528  2.98527  4.58485  3.61467
         0.05386  3.00455  5.85228  1.15453  0.37864  0.48576  0.95510
      78   2.80595  5.38310  2.72848  1.15966  4.42341  3.05333  3.57514
3.55171  2.62476  3.50596  4.43074  3.10658  4.11127  2.86410  3.08825
2.90999  3.16668  2.98318  5.83140  4.43623    110 e - - -
         2.68618  4.42225  2.77519  2.73123  3.46354  2.40513  3.72494
3.29354  2.67741  2.69355  4.24690  2.90347  2.73739  3.18146  2.89801
2.37887  2.77519  2.98518  4.58477  3.61503
         0.00883  5.12993  5.85228  0.61958  0.77255  0.48576  0.95510
      79   2.81187  5.42434  3.15273  2.42168  4.55602  3.20094  3.25754
3.74560  1.90579  3.01903  3.75741  2.82393  4.10171  2.43777  2.03358
2.43897  3.02415  3.67653  5.86153  4.45525    111 k - - -
         2.68618  4.42225  2.77519  2.73123  3.46354  2.40513  3.72494
3.29354  2.67741  2.69355  4.24690  2.90347  2.73739  3.18146  2.89801
2.37887  2.77519  2.98518  4.58477  3.61503
         0.00883  5.12993  5.85228  0.61958  0.77255  0.48576  0.95510
      80   2.63035  5.37297  3.00798  2.28233  4.58282  3.35417  3.56395
2.78994  2.47915  2.92252  3.06204  2.66605  4.11068  2.22328  2.88928
2.24505  2.95063  3.56560  5.82307  4.32087    112 q - - -
         2.68618  4.42225  2.77519  2.73123  3.46354  2.40513  3.72494
3.29354  2.67741  2.69355  4.24690  2.90347  2.73739  3.18146  2.89801
2.37887  2.77519  2.98518  4.58477  3.61503
         0.00883  5.12993  5.85228  0.61958  0.77255  0.48576  0.95510
      81   2.76311  4.40442  4.61453  3.84548  3.45791  4.10135  4.41615
2.48140  3.86638  0.95629  3.37474  3.58214  4.46847  3.82941  3.47412
3.17162  3.17478  2.27397  4.61078  3.83548    113 l - - -
         2.68618  4.42225  2.77519  2.73123  3.46354  2.40513  3.72494
3.29354  2.67741  2.69355  4.24690  2.90347  2.73739  3.18146  2.89801
2.37887  2.77519  2.98518  4.58477  3.61503
         0.00883  5.12993  5.85228  0.61958  0.77255  0.48576  0.95510
      82   2.75797  5.00008  3.16983  1.95583  4.52839  3.10100  2.63565
4.01635  2.38107  2.69474  3.85554  3.13310  4.11261  2.09225  2.86431
2.83140  3.13986  2.87382  5.81532  4.03676    114 e - - -
         2.68618  4.42225  2.77519  2.73123  3.46354  2.40513  3.72494
3.29354  2.67741  2.69355  4.24690  2.90347  2.73739  3.18146  2.89801
2.37887  2.77519  2.98518  4.58477  3.61503
         0.00883  5.12993  5.85228  0.61958  0.77255  0.48576  0.95510
      83   2.54844  5.44517  2.41870  1.59200  4.79069  3.70605  3.78303
4.27691  1.95764  3.43334  4.16943  2.65574  4.10001  2.45632  2.87741
2.84915  3.12682  3.83607  5.87738  4.46623    115 e - - -
         2.68618  4.42225  2.77519  2.73123  3.46354  2.40513  3.72494
3.29354  2.67741  2.69355  4.24690  2.90347  2.73739  3.18146  2.89801
2.37887  2.77519  2.98518  4.58477  3.61503
         0.00883  5.12993  5.85228  0.61958  0.77255  0.48576  0.95510
```

84 2.39124 5.38219 2.76282 1.34341 4.70228 3.71645 3.72680
3.03858 2.62177 3.43347 3.75246 3.14510 4.10940 2.41496 3.03231
2.82931 3.05599 3.57335 5.83008 4.43473 116 e - - -
2.68618 4.42225 2.77519 2.73123 3.46354 2.40513 3.72494
3.29354 2.67741 2.69355 4.24690 2.90347 2.73739 3.18146 2.89801
2.37887 2.77519 2.98518 4.58477 3.61503
0.00883 5.12993 5.85228 0.61958 0.77255 0.48576 0.95510
85 3.04637 4.88507 3.33592 2.78884 4.69422 3.81588 3.14167
4.14014 2.67213 3.66998 4.45531 0.97818 4.21145 2.90351 2.67426
3.01014 3.05633 3.76670 5.83342 4.24534 117 n - - -
2.68618 4.42225 2.77519 2.73123 3.46354 2.40513 3.72494
3.29354 2.67741 2.69355 4.24690 2.90347 2.73739 3.18146 2.89801
2.37887 2.77519 2.98518 4.58477 3.61503
0.00883 5.12993 5.85228 0.61958 0.77255 0.48576 0.95510
86 2.57296 5.42583 2.99228 2.20400 4.76400 3.26969 3.89576
3.58418 1.91411 3.51515 3.46224 2.53768 4.10129 2.75568 2.52086
2.23851 2.99940 3.36684 5.86266 3.80728 118 k - - -
2.68618 4.42225 2.77519 2.73123 3.46354 2.40513 3.72494
3.29354 2.67741 2.69355 4.24690 2.90347 2.73739 3.18146 2.89801
2.37887 2.77519 2.98518 4.58477 3.61503
0.00883 5.12993 5.85228 0.61958 0.77255 0.48576 0.95510
87 2.78269 4.59365 3.13753 2.29646 3.55005 3.37042 3.44353
3.26142 2.50687 2.59983 3.07815 2.58172 4.13948 2.42420 2.69126
2.73736 2.82092 3.29651 5.71268 3.31749 119 e - - -
2.68618 4.42225 2.77519 2.73123 3.46354 2.40513 3.72494
3.29354 2.67741 2.69355 4.24690 2.90347 2.73739 3.18146 2.89801
2.37887 2.77519 2.98518 4.58477 3.61503
0.00883 5.12993 5.85228 0.61958 0.77255 0.48576 0.95510
88 4.64756 5.77401 6.57110 6.00575 3.68769 6.13533 6.35219
2.66310 5.84601 0.25794 3.23759 6.32246 5.94395 5.55958 5.71669
5.60999 4.83077 3.24641 6.07253 5.19087 120 L - - -
2.68618 4.42225 2.77519 2.73123 3.46354 2.40513 3.72494
3.29354 2.67741 2.69355 4.24690 2.90347 2.73739 3.18146 2.89801
2.37887 2.77519 2.98518 4.58477 3.61503
0.00883 5.12993 5.85228 0.61958 0.77255 0.48576 0.95510
89 2.80006 3.66396 3.22784 2.54680 4.64143 3.64688 3.41917
3.89182 2.13133 2.87153 4.23462 3.05599 4.11786 2.48333 1.69958
2.62801 2.82036 3.58500 5.79668 3.69745 121 r - - -
2.68618 4.42225 2.77519 2.73123 3.46354 2.40513 3.72494
3.29354 2.67741 2.69355 4.24690 2.90347 2.73739 3.18146 2.89801
2.37887 2.77519 2.98518 4.58477 3.61503
0.01364 5.12993 4.87597 0.61958 0.77255 0.48576 0.95510
90 2.70758 5.42632 2.75821 2.03147 3.71781 3.62982 3.19796
3.50448 2.11777 3.08600 4.35667 3.01598 4.09872 2.39264 2.17034
2.70100 3.03418 3.64772 5.86248 3.98629 122 e - - -
2.68618 4.42225 2.77519 2.73123 3.46354 2.40513 3.72494
3.29354 2.67741 2.69355 4.24690 2.90347 2.73739 3.18146 2.89801
2.37887 2.77519 2.98518 4.58477 3.61503
0.01529 5.12517 4.68573 0.61958 0.77255 0.50092 0.93134
91 2.93914 5.41701 3.06114 2.22897 4.75236 3.71582 3.89938
3.87140 1.36165 3.52356 3.25421 3.14453 4.10819 2.36419 2.33055
2.90714 3.16931 3.72049 5.04067 4.45382 123 k - - -
2.68618 4.42225 2.77519 2.73123 3.46354 2.40513 3.72494
3.29354 2.67741 2.69355 4.24690 2.90347 2.73739 3.18146 2.89801
2.37887 2.77519 2.98518 4.58477 3.61503
0.05943 5.11881 2.96208 0.61958 0.77255 0.52067 0.90170

```
     92   2.94333  2.92737  4.91103  4.29541  2.55866  4.11314  4.26311
1.87880  4.07936  1.54520  2.95546  4.27688  4.47666  3.99937  4.08604
3.29273  3.12338  1.96107  4.40668  3.33924    124 l - - -
          2.68618  4.42225  2.77519  2.73123  3.46354  2.40513  3.72494
3.29354  2.67741  2.69355  4.24690  2.90347  2.73739  3.18146  2.89801
2.37887  2.77519  2.98518  4.58477  3.61503
          0.20891  5.06877  1.70244  0.61958  0.77255  0.66014  0.72728
     93   2.12688  5.33167  2.54298  1.87088  4.67483  3.46156  3.17201
4.01766  2.08222  3.63610  4.27891  2.89051  3.99275  2.41711  3.01480
2.67727  2.99808  3.52769  5.76540  4.23534    125 e - - -
          2.68618  4.42225  2.77519  2.73123  3.46354  2.40513  3.72494
3.29354  2.67741  2.69355  4.24690  2.90347  2.73739  3.18146  2.89801
2.37887  2.77519  2.98518  4.58477  3.61503
          0.00772  4.86759        *  0.61958  0.77255  0.00000        *
//
HMMER3/f [3.3 | Nov 2019]
NAME  KNOX1
ACC   PF03790.14
DESC  KNOX1 domain
LENG  43
ALPH  amino
RF    no
MM    no
CONS  yes
CS    no
MAP   yes
DATE  Thu Apr 16 21:49:29 2020
NSEQ  38
EFFN  5.292725
CKSUM 4135660479
GA    20.50 20.50
TC    21.30 20.70
NC    20.20 20.40
STATS LOCAL MSV       -7.8852  0.71939
STATS LOCAL VITERBI   -7.8520  0.71939
STATS LOCAL FORWARD   -4.4171  0.71939
HMM          A        C        D        E        F        G        H
I        K        L        M        N        P        Q        R        S
T        V        W        Y
            m->m     m->i     m->d     i->m     i->i     d->m     d->d
  COMPO   2.19413  3.72714  3.17896  2.78214  4.01838  3.30704  3.09125
2.64085  2.66258  2.23444  3.72572  3.51694  2.92099  3.01361  3.12703
2.77729  3.24313  2.64907  5.77387  3.29480
          2.68618  4.42225  2.77519  2.73123  3.46354  2.40513  3.72494
3.29354  2.67741  2.69355  4.24690  2.90347  2.73739  3.18146  2.89801
2.37887  2.77519  2.98518  4.58477  3.61503
          0.15264  5.39558  1.98759  0.61958  0.77255  0.00000        *
      1   1.74276  5.23131  3.11963  2.66236  4.47939  3.81920  4.02073
3.21742  2.58097  2.49527  4.29900  3.34118  4.20948  2.94595  2.59400
2.57651  2.06855  3.05865  5.72684  4.38349      1 a - - -
          2.68618  4.42225  2.77519  2.73123  3.46354  2.40513  3.72494
3.29354  2.67741  2.69355  4.24690  2.90347  2.73739  3.18146  2.89801
2.37887  2.77519  2.98518  4.58477  3.61503
          0.00782  5.25076  5.97310  0.61958  0.77255  0.40557  1.09841
      2   3.35037  3.87622  5.33320  4.73044  3.74484  4.56709  4.91954
1.09358  4.52901  2.06734  1.83968  4.73103  4.90424  4.65100  4.54007
3.88876  3.58341  2.11221  5.37563  3.04255      2 i - - -
```

```
          2.68618  4.42225  2.77519  2.73123  3.46354  2.40513  3.72494
3.29354  2.67741  2.69355  4.24690  2.90347  2.73739  3.18146  2.89801
2.37887  2.77519  2.98518  4.58477  3.61503
          0.00699  5.36235  6.08469  0.61958  0.77255  0.41316  1.08339
      3   4.30798  6.29223  5.09347  4.04351  5.92388  4.81815  4.51279
5.14655  0.38269  4.45735  3.18867  4.32204  5.11232  3.63872  2.18315
4.28179  4.38206  4.86241  6.40485  5.36919      3 K - - -
          2.68618  4.42225  2.77519  2.73123  3.46354  2.40513  3.72494
3.29354  2.67741  2.69355  4.24690  2.90347  2.73739  3.18146  2.89801
2.37887  2.77519  2.98518  4.58477  3.61503
          0.00676  5.39558  6.11792  0.61958  0.77255  0.48576  0.95510
      4   0.79688  5.33075  3.14857  3.13787  4.88452  2.77130  4.36803
3.77718  3.18144  3.90649  4.69797  3.03654  4.40978  3.51484  3.64478
2.39211  3.13360  3.91114  6.11596  4.77730      4 a - - -
          2.68618  4.42225  2.77519  2.73123  3.46354  2.40513  3.72494
3.29354  2.67741  2.69355  4.24690  2.90347  2.73739  3.18146  2.89801
2.37887  2.77519  2.98518  4.58477  3.61503
          0.00676  5.39558  6.11792  0.61958  0.77255  0.48576  0.95510
      5   2.50973  5.57288  2.55393  2.15123  4.91782  3.83496  4.02273
3.53864  1.42683  3.87787  4.61377  3.30245  4.22915  2.42592  3.03834
2.13793  3.29401  3.96349  6.00533  4.59465      5 k - - -
          2.68618  4.42225  2.77519  2.73123  3.46354  2.40513  3.72494
3.29354  2.67741  2.69355  4.24690  2.90347  2.73739  3.18146  2.89801
2.37887  2.77519  2.98518  4.58477  3.61503
          0.00676  5.39558  6.11792  0.61958  0.77255  0.48576  0.95510
      6   4.40930  5.56615  6.49670  6.00899  4.25269  6.04436  6.61654
0.34375  5.92206  2.23651  3.17245  6.22313  6.08393  5.92732  5.95957
5.50991  4.64657  2.61610  6.56721  5.53421      6 I - - -
          2.68618  4.42225  2.77519  2.73123  3.46354  2.40513  3.72494
3.29354  2.67741  2.69355  4.24690  2.90347  2.73739  3.18146  2.89801
2.37887  2.77519  2.98518  4.58477  3.61503
          0.00676  5.39558  6.11792  0.61958  0.77255  0.48576  0.95510
      7   1.89038  4.59405  5.14414  4.54020  3.70587  4.41162  4.75794
1.91624  4.34415  2.13625  2.24182  4.55710  4.76939  4.48964  4.37710
2.62024  3.45817  1.49835  5.26185  4.08370      7 v - - -
          2.68618  4.42225  2.77519  2.73123  3.46354  2.40513  3.72494
3.29354  2.67741  2.69355  4.24690  2.90347  2.73739  3.18146  2.89801
2.37887  2.77519  2.98518  4.58477  3.61503
          0.00676  5.39558  6.11792  0.61958  0.77255  0.48576  0.95510
      8   1.80135  3.81827  3.01554  2.84091  4.73518  2.91713  4.07703
4.18394  2.83995  3.72843  4.50292  2.56168  4.26389  3.19614  3.04640
1.59674  2.48059  3.27881  5.91477  4.54209      8 s - - -
          2.68618  4.42225  2.77519  2.73123  3.46354  2.40513  3.72494
3.29354  2.67741  2.69355  4.24690  2.90347  2.73739  3.18146  2.89801
2.37887  2.77519  2.98518  4.58477  3.61503
          0.00676  5.39558  6.11792  0.61958  0.77255  0.48576  0.95510
      9   5.73022  6.88968  5.74893  5.75200  5.53193  5.47132  0.04946
6.75120  5.69854  5.93751  7.20663  6.05425  6.11414  6.19198  5.76280
5.87944  6.13462  6.42917  6.80471  5.48963      9 H - - -
          2.68618  4.42225  2.77519  2.73123  3.46354  2.40513  3.72494
3.29354  2.67741  2.69355  4.24690  2.90347  2.73739  3.18146  2.89801
2.37887  2.77519  2.98518  4.58477  3.61503
          0.00676  5.39558  6.11792  0.61958  0.77255  0.48576  0.95510
     10   2.04957  5.18748  4.19444  3.83444  4.69869  4.06243  3.53039
4.18932  3.75544  3.06726  4.75286  4.13455  0.61084  4.14665  4.06200
3.47555  3.69057  3.84174  6.10793  4.81860     10 P - - -
```

```
          2.68618  4.42225  2.77519  2.73123  3.46354  2.40513  3.72494
3.29354  2.67741  2.69355  4.24690  2.90347  2.73739  3.18146  2.89801
2.37887  2.77519  2.98518  4.58477  3.61503
          0.00676  5.39558  6.11792  0.61958  0.77255  0.48576  0.95510
     11   3.12859  5.02950  3.77415  3.20293  4.19337  4.03296  1.90108
3.57837  3.10502  1.55546  3.25657  3.66419  4.41460  2.00380  2.77284
3.05877  3.35712  3.32479  5.56986  3.69302     11 l - - -
          2.68618  4.42225  2.77519  2.73123  3.46354  2.40513  3.72494
3.29354  2.67741  2.69355  4.24690  2.90347  2.73739  3.18146  2.89801
2.37887  2.77519  2.98518  4.58477  3.61503
          0.00676  5.39558  6.11792  0.61958  0.77255  0.48576  0.95510
     12   4.45158  2.75689  5.85766  5.56396  2.29936  5.27462  4.52830
4.31125  5.35747  3.77526  4.93048  5.13630  5.64578  5.24619  5.28519
3.39951  4.71205  4.16384  4.65996  0.40572     12 Y - - -
          2.68618  4.42225  2.77519  2.73123  3.46354  2.40513  3.72494
3.29354  2.67741  2.69355  4.24690  2.90347  2.73739  3.18146  2.89801
2.37887  2.77519  2.98518  4.58477  3.61503
          0.00676  5.39558  6.11792  0.61958  0.77255  0.48576  0.95510
     13   3.20192  5.65541  3.29511  1.80600  4.98571  3.91379  4.15022
4.45838  2.50881  3.95828  4.72163  3.39322  1.39622  3.26284  3.38270
1.80306  3.43917  4.04402  6.10599  3.86656     13 p - - -
          2.68618  4.42225  2.77519  2.73123  3.46354  2.40513  3.72494
3.29354  2.67741  2.69355  4.24690  2.90347  2.73739  3.18146  2.89801
2.37887  2.77519  2.98518  4.58477  3.61503
          0.00676  5.39558  6.11792  0.61958  0.77255  0.48576  0.95510
     14   2.53054  5.57466  2.25329  2.43898  4.92074  3.83332  4.02113
4.40750  2.30192  3.88031  4.61517  2.48573  4.22748  1.97887  2.50780
2.08188  2.79874  3.96586  6.00660  4.59487     14 q - - -
          2.68618  4.42225  2.77519  2.73123  3.46354  2.40513  3.72494
3.29354  2.67741  2.69355  4.24690  2.90347  2.73739  3.18146  2.89801
2.37887  2.77519  2.98518  4.58477  3.61503
          0.00676  5.39558  6.11792  0.61958  0.77255  0.48576  0.95510
     15   3.18141  5.77718  6.04114  5.74560  4.15153  5.28917  6.23554
3.35222  5.55946  0.21944  3.93172  5.78230  5.72948  5.61306  5.55544
4.83258  4.69308  3.56847  6.36510  5.35487     15 L - - -
          2.68618  4.42225  2.77519  2.73123  3.46354  2.40513  3.72494
3.29354  2.67741  2.69355  4.24690  2.90347  2.73739  3.18146  2.89801
2.37887  2.77519  2.98518  4.58477  3.61503
          0.00676  5.39558  6.11792  0.61958  0.77255  0.48576  0.95510
     16   3.86318  5.18583  5.70195  5.14624  3.95297  5.11754  5.50864
2.65586  4.96491  0.46582  3.79700  3.51294  5.39138  5.08353  5.01885
4.47219  4.10158  2.11136  5.86563  4.74715     16 L - - -
          2.68618  4.42225  2.77519  2.73123  3.46354  2.40513  3.72494
3.29354  2.67741  2.69355  4.24690  2.90347  2.73739  3.18146  2.89801
2.37887  2.77519  2.98518  4.58477  3.61503
          0.00676  5.39558  6.11792  0.61958  0.77255  0.48576  0.95510
     17   1.71102  5.54777  3.31767  2.45724  4.88333  2.90926  3.60451
4.36247  2.76396  3.26740  4.59114  3.05912  4.23093  2.42308  2.68227
1.80410  2.73782  3.93334  5.98627  4.58134     17 a - - -
          2.68618  4.42225  2.77519  2.73123  3.46354  2.40513  3.72494
3.29354  2.67741  2.69355  4.24690  2.90347  2.73739  3.18146  2.89801
2.37887  2.77519  2.98518  4.58477  3.61503
          0.00676  5.39558  6.11792  0.61958  0.77255  0.48576  0.95510
     18   0.52816  5.07569  5.27065  4.99364  5.53778  3.91647  5.71545
4.92629  4.91274  4.67040  5.47396  4.66300  4.74682  5.09610  5.06255
1.64875  2.66709  3.20547  6.91822  5.81218     18 A - - -
```

```
          2.68618  4.42225  2.77519  2.73123  3.46354  2.40513  3.72494
3.29354  2.67741  2.69355  4.24690  2.90347  2.73739  3.18146  2.89801
2.37887  2.77519  2.98518  4.58477  3.61503
          0.04061  5.39558  3.34503  0.61958  0.77255  0.48576  0.95510
     19   5.20041  6.18059  6.11828  6.00477  2.27397  5.75403  1.45120
4.81776  5.77898  2.05527  5.34837  5.31425  6.01109  5.47192  5.60366
5.14803  5.39488  4.75195  4.53059  0.78007     19 y - - -
          2.68618  4.42225  2.77519  2.73123  3.46354  2.40513  3.72494
3.29354  2.67741  2.69355  4.24690  2.90347  2.73739  3.18146  2.89801
2.37887  2.77519  2.98518  4.58477  3.61503
          0.00699  5.36196  6.08431  0.61958  0.77255  0.41246  1.08476
     20   4.24623  5.44247  6.32292  5.79269  4.16084  5.76530  6.24683
1.39282  5.69078  1.19839  2.22269  5.94153  5.87580  5.68807  5.70054
5.17453  4.47730  1.35408  6.34085  5.30667     20 l - - -
          2.68618  4.42225  2.77519  2.73123  3.46354  2.40513  3.72494
3.29354  2.67741  2.69355  4.24690  2.90347  2.73739  3.18146  2.89801
2.37887  2.77519  2.98518  4.58477  3.61503
          0.00676  5.39558  6.11792  0.61958  0.77255  0.48576  0.95510
     21   2.09598  5.86281  1.62065  1.95004  5.19535  2.79174  4.22968
4.69590  3.03699  4.16391  4.91913  1.76750  4.40086  2.98752  3.55828
2.84125  3.55533  4.24908  6.29400  4.85176     21 d - - -
          2.68618  4.42225  2.77519  2.73123  3.46354  2.40513  3.72494
3.29354  2.67741  2.69355  4.24690  2.90347  2.73739  3.18146  2.89801
2.37887  2.77519  2.98518  4.58477  3.61503
          0.00676  5.39558  6.11792  0.61958  0.77255  0.48576  0.95510
     22   3.93487  0.40570  6.05763  5.78489  4.80830  4.86483  6.22597
1.90371  5.58193  3.41712  4.73321  5.54623  5.51298  5.80958  5.61924
4.42139  4.35910  2.96767  6.68088  5.46227     22 C - - -
          2.68618  4.42225  2.77519  2.73123  3.46354  2.40513  3.72494
3.29354  2.67741  2.69355  4.24690  2.90347  2.73739  3.18146  2.89801
2.37887  2.77519  2.98518  4.58477  3.61503
          0.00676  5.39558  6.11792  0.61958  0.77255  0.48576  0.95510
     23   3.22655  5.48240  3.60178  3.01761  4.76358  4.01599  3.36189
4.18752  2.61779  1.81029  4.54684  3.18644  4.39751  1.24420  2.49128
3.23674  3.44570  3.84509  5.92267  3.30965     23 q - - -
          2.68618  4.42225  2.77519  2.73123  3.46354  2.40513  3.72494
3.29354  2.67741  2.69355  4.24690  2.90347  2.73739  3.18146  2.89801
2.37887  2.77519  2.98518  4.58477  3.61503
          0.00676  5.39558  6.11792  0.61958  0.77255  0.48576  0.95510
     24   4.48982  6.42704  5.38849  4.17819  6.18612  4.92521  4.53630
5.33283  0.54002  4.57818  5.49895  4.41116  5.19611  2.94122  1.42705
4.44267  4.51695  5.05396  6.46226  5.47938     24 K - - -
          2.68548  4.42261  2.77556  2.73159  3.46390  2.40549  3.72531
3.29390  2.67777  2.69177  4.24726  2.90383  2.73776  3.18008  2.89837
2.37923  2.77556  2.98411  4.58513  3.61539
          0.13337  2.09839  6.11792  1.49191  0.25482  0.48576  0.95510
     25   3.83548  5.11172  5.69732  3.33956  4.50460  5.25438  5.84181
1.84031  5.12002  3.14283  4.32066  5.36777  5.56492  5.37920  5.28110
4.63870  3.22302  0.48148  6.35539  5.13462     30 V - - -
          2.68618  4.42225  2.77519  2.73123  3.46354  2.40513  3.72494
3.29354  2.67741  2.69355  4.24690  2.90347  2.73739  3.18146  2.89801
2.37887  2.77519  2.98518  4.58477  3.61503
          0.00676  5.39558  6.11792  0.61958  0.77255  0.48576  0.95510
     26   1.60830  5.28820  4.40093  4.30265  5.65004  0.51961  3.20356
5.21573  4.53942  4.85882  5.66369  4.42246  4.80088  4.78553  4.81658
3.50934  3.86658  4.50361  6.97150  5.75350     31 G - - -
```

2.68618 4.42225 2.77519 2.73123 3.46354 2.40513 3.72494
3.29354 2.67741 2.69355 4.24690 2.90347 2.73739 3.18146 2.89801
2.37887 2.77519 2.98518 4.58477 3.61503
0.00676 5.39558 6.11792 0.61958 0.77255 0.48576 0.95510
27 0.53051 5.04567 5.20311 4.87377 5.01079 4.04332 5.54930
2.91490 4.76400 3.98251 4.94845 4.67744 4.81063 4.98452 4.91568
3.50638 1.71177 3.60192 6.52584 5.37368 32 A - - -
2.68618 4.42225 2.77519 2.73123 3.46354 2.40513 3.72494
3.29354 2.67741 2.69355 4.24690 2.90347 2.73739 3.18146 2.89801
2.37887 2.77519 2.98518 4.58477 3.61503
0.00676 5.39558 6.11792 0.61958 0.77255 0.48576 0.95510
28 3.29929 5.18265 4.86540 4.71599 5.33546 4.01587 5.60168
3.08378 4.70182 4.46577 5.38765 4.61413 0.34795 4.98921 4.87462
2.64956 3.83428 4.15998 6.77830 5.57071 33 P - - -
2.68618 4.42225 2.77519 2.73123 3.46354 2.40513 3.72494
3.29354 2.67741 2.69355 4.24690 2.90347 2.73739 3.18146 2.89801
2.37887 2.77519 2.98518 4.58477 3.61503
0.00676 5.39558 6.11792 0.61958 0.77255 0.48576 0.95510
29 2.49066 4.67556 4.37655 3.80248 3.80349 4.17608 4.49061
2.50563 3.71230 2.88445 3.41735 4.08705 1.12483 3.40724 3.96213
2.89167 3.36162 2.08868 5.29693 4.09384 34 p - - -
2.68618 4.42225 2.77519 2.73123 3.46354 2.40513 3.72494
3.29354 2.67741 2.69355 4.24690 2.90347 2.73739 3.18146 2.89801
2.37887 2.77519 2.98518 4.58477 3.61503
0.00676 5.39558 6.11792 0.61958 0.77255 0.48576 0.95510
30 3.23485 6.17689 1.12476 1.06664 5.49097 3.43381 4.46853
5.00270 3.38693 4.47901 3.37030 3.49131 4.58960 3.60692 3.95181
3.50657 3.86535 4.55898 6.62606 5.14446 35 e - - -
2.68618 4.42225 2.77519 2.73123 3.46354 2.40513 3.72494
3.29354 2.67741 2.69355 4.24690 2.90347 2.73739 3.18146 2.89801
2.37887 2.77519 2.98518 4.58477 3.61503
0.00676 5.39558 6.11792 0.61958 0.77255 0.48576 0.95510
31 3.12571 4.62820 4.49853 3.91200 3.73319 4.21166 3.57378
2.27582 3.78268 2.35039 3.06981 4.15458 4.58143 2.22712 2.43382
3.49296 3.35719 1.30359 5.23635 4.04102 36 v - - -
2.68618 4.42225 2.77519 2.73123 3.46354 2.40513 3.72494
3.29354 2.67741 2.69355 4.24690 2.90347 2.73739 3.18146 2.89801
2.37887 2.77519 2.98518 4.58477 3.61503
0.00676 5.39558 6.11792 0.61958 0.77255 0.48576 0.95510
32 1.55523 4.65964 5.12443 4.53603 3.80600 4.44878 4.83659
2.60433 4.36150 1.69189 3.79728 4.58791 4.82193 4.52762 4.42915
3.31910 3.09687 1.25380 5.37178 4.18982 37 v - - -
2.68618 4.42225 2.77519 2.73123 3.46354 2.40513 3.72494
3.29354 2.67741 2.69355 4.24690 2.90347 2.73739 3.18146 2.89801
2.37887 2.77519 2.98518 4.58477 3.61503
0.00676 5.39558 6.11792 0.61958 0.77255 0.48576 0.95510
33 1.60998 3.64430 2.78201 2.37709 4.84328 3.28860 4.03463
4.31419 2.77892 3.81613 4.56680 2.75803 2.32365 3.13858 3.26791
2.23337 3.03704 3.89874 5.96625 3.58521 38 a - - -
2.68618 4.42225 2.77519 2.73123 3.46354 2.40513 3.72494
3.29354 2.67741 2.69355 4.24690 2.90347 2.73739 3.18146 2.89801
2.37887 2.77519 2.98518 4.58477 3.61503
0.00676 5.39558 6.11792 0.61958 0.77255 0.48576 0.95510
34 3.20954 5.29491 3.70101 3.11259 4.52684 4.04344 4.19931
2.90528 1.86882 2.13330 3.45425 3.59958 4.42250 2.79824 1.35866
2.94769 3.43040 3.62417 5.78477 4.49822 39 r - - -

```
          2.68618  4.42225  2.77519  2.73123  3.46354  2.40513  3.72494
3.29354  2.67741  2.69355  4.24690  2.90347  2.73739  3.18146  2.89801
2.37887  2.77519  2.98518  4.58477  3.61503
          0.00676  5.39558  6.11792  0.61958  0.77255  0.48576  0.95510
     35   4.81143  5.93838  6.80320  6.21169  3.10998  6.30430  6.51494
1.58695  6.10236  0.49422  2.73650  6.50314  6.11250  5.74522  5.93249
5.75088  4.98718  3.48871  6.24650  5.39758     40 L - - -
          2.68618  4.42225  2.77519  2.73123  3.46354  2.40513  3.72494
3.29354  2.67741  2.69355  4.24690  2.90347  2.73739  3.18146  2.89801
2.37887  2.77519  2.98518  4.58477  3.61503
          0.00676  5.39558  6.11792  0.61958  0.77255  0.48576  0.95510
     36   2.80300  5.94029  1.20674  1.72529  5.29559  3.96882  4.32634
4.79815  3.16992  4.27302  5.04147  3.44094  4.47174  3.44835  3.70247
1.83627  2.64842  4.34940  6.41046  4.96023     41 d - - -
          2.68618  4.42225  2.77519  2.73123  3.46354  2.40513  3.72494
3.29354  2.67741  2.69355  4.24690  2.90347  2.73739  3.18146  2.89801
2.37887  2.77519  2.98518  4.58477  3.61503
          0.00676  5.39558  6.11792  0.61958  0.77255  0.48576  0.95510
     37   1.35550  5.62142  3.31627  1.21531  5.02045  3.41679  4.20132
4.49383  2.65064  4.00035  4.76310  3.42576  4.35923  3.31700  3.46651
2.94199  3.01259  4.06543  6.15330  4.75347     42 e - - -
          2.68618  4.42225  2.77519  2.73123  3.46354  2.40513  3.72494
3.29354  2.67741  2.69355  4.24690  2.90347  2.73739  3.18146  2.89801
2.37887  2.77519  2.98518  4.58477  3.61503
          0.00676  5.39558  6.11792  0.61958  0.77255  0.48576  0.95510
     38   2.03470  4.59266  4.69904  4.10958  3.70117  4.26289  4.57861
1.61615  3.97508  2.17463  3.71123  4.28108  4.63147  2.08676  4.12696
3.13742  2.97106  1.91412  5.22037  4.03173     43 i - - -
          2.68618  4.42225  2.77519  2.73123  3.46354  2.40513  3.72494
3.29354  2.67741  2.69355  4.24690  2.90347  2.73739  3.18146  2.89801
2.37887  2.77519  2.98518  4.58477  3.61503
          0.00676  5.39558  6.11792  0.61958  0.77255  0.48576  0.95510
     39   1.84086  3.53397  3.81748  3.25346  4.07100  2.23314  4.26634
3.13025  2.88090  2.01888  4.01381  3.69927  3.73871  3.05081  2.68388
2.85280  3.08143  2.57546  5.48360  4.22890     44 a - - -
          2.68618  4.42225  2.77519  2.73123  3.46354  2.40513  3.72494
3.29354  2.67741  2.69355  4.24690  2.90347  2.73739  3.18146  2.89801
2.37887  2.77519  2.98518  4.58477  3.61503
          0.00676  5.39558  6.11792  0.61958  0.77255  0.48576  0.95510
     40   2.01946  5.57036  3.07902  2.54239  4.91461  3.30978  3.61153
4.39964  2.57826  3.87500  4.61145  3.30313  4.22921  1.57170  2.06165
2.52793  2.97016  3.96060  6.00323  4.59323     45 q - - -
          2.68618  4.42225  2.77519  2.73123  3.46354  2.40513  3.72494
3.29354  2.67741  2.69355  4.24690  2.90347  2.73739  3.18146  2.89801
2.37887  2.77519  2.98518  4.58477  3.61503
          0.13337  5.39558  2.11760  0.61958  0.77255  0.48576  0.95510
     41   2.79949  5.52651  3.05199  1.25825  4.87199  3.41180  3.31735
4.35881  2.18378  3.83155  4.56775  2.89527  4.17497  2.14375  2.99178
2.82589  3.24436  3.91751  5.95796  4.54569     46 e - - -
          2.68618  4.42225  2.77519  2.73123  3.46354  2.40513  3.72494
3.29354  2.67741  2.69355  4.24690  2.90347  2.73739  3.18146  2.89801
2.37887  2.77519  2.98518  4.58477  3.61503
          0.02193  5.26988  4.10166  0.61958  0.77255  0.88767  0.53037
     42   3.01692  3.33183  3.78575  3.02237  2.33981  3.96665  4.20923
3.32870  3.18438  2.05114  3.91122  2.42270  4.34826  3.15069  2.74741
1.81789  3.24858  2.85357  5.38360  4.13515     47 s - - -
```

```
          2.68618  4.42225  2.77519  2.73123  3.46354  2.40513  3.72494
3.29354  2.67741  2.69355  4.24690  2.90347  2.73739  3.18146  2.89801
2.37887  2.77519  2.98518  4.58477  3.61503
          0.16406  5.25573  1.92353  0.61958  0.77255  0.92180  0.50717
     43   3.00987  5.53604  2.05319  1.62916  4.87649  3.72264  2.28508
4.36765  2.71324  3.83902  4.58193  2.86148  4.14621  2.19431  3.00634
2.62771  3.24571  3.92498  5.96682  4.54470     48 e - - -
          2.68618  4.42225  2.77519  2.73123  3.46354  2.40513  3.72494
3.29354  2.67741  2.69355  4.24690  2.90347  2.73739  3.18146  2.89801
2.37887  2.77519  2.98518  4.58477  3.61503
          0.00613  5.09780        *  0.61958  0.77255  0.00000        *
//
HMMER3/f [3.3 | Nov 2019]
NAME  KNOX2
ACC   PF03791.14
DESC  KNOX2 domain
LENG  48
ALPH  amino
RF    no
MM    no
CONS  yes
CS    no
MAP   yes
DATE  Thu Apr 16 21:50:22 2020
NSEQ  35
EFFN  3.208618
CKSUM 246647427
GA    20.80 20.80
TC    21.20 21.40
NC    20.60 20.70
STATS LOCAL MSV       -8.0371  0.71923
STATS LOCAL VITERBI   -8.4053  0.71923
STATS LOCAL FORWARD   -4.6607  0.71923
HMM          A        C        D        E        F        G        H
I        K        L        M        N        P        Q        R        S
T        V        W        Y
            m->m     m->i     m->d     i->m     i->i     d->m     d->d
  COMPO   2.61589  3.85658  2.74935  2.27309  2.84708  3.62048  3.73472
3.14489  2.82127  2.10933  3.30569  3.33343  3.56251  2.86423  3.06462
2.87723  3.01083  2.95705  5.34771  3.23079
          2.68618  4.42225  2.77519  2.73123  3.46354  2.40513  3.72494
3.29354  2.67741  2.69355  4.24690  2.90347  2.73739  3.18146  2.89801
2.37887  2.77519  2.98518  4.58477  3.61503
          0.10388  4.97991  2.38818  0.61958  0.77255  0.00000        *
      1   2.72424  3.48656  2.87575  2.13659  4.51034  3.62678  2.79123
3.96275  2.57778  3.49874  3.43596  3.11675  3.61935  2.33391  3.06056
1.97012  3.06315  2.90060  5.67845  4.29958      1 s - - -
          2.68618  4.42225  2.77519  2.73123  3.46354  2.40513  3.72494
3.29354  2.67741  2.69355  4.24690  2.90347  2.73739  3.18146  2.89801
2.37887  2.77519  2.98518  4.58477  3.61503
          0.01127  4.88729  5.60964  0.61958  0.77255  0.46003  0.99768
      2   2.53931  4.48833  3.28936  3.38180  3.33496  3.90390  3.22997
2.16172  3.31467  2.38509  3.38532  3.72709  2.33398  3.29457  3.61327
3.16397  2.62140  1.97493  5.08408  3.86924      2 v - - -
          2.68618  4.42225  2.77519  2.73123  3.46354  2.40513  3.72494
3.29354  2.67741  2.69355  4.24690  2.90347  2.73739  3.18146  2.89801
2.37887  2.77519  2.98518  4.58477  3.61503
          0.01055  4.95273  5.67507  0.61958  0.77255  0.44352  1.02659
```

```
      3   2.51188  3.70515  3.42737  2.72072  4.07745  1.67652  2.74038
3.46662  2.84979  2.91849  3.97387  3.35567  4.14700  3.18756  3.11280
2.34475  3.11136  2.68339  5.42675  4.13610      3 g - - -
          2.68618  4.42225  2.77519  2.73123  3.46354  2.40513  3.72494
3.29354  2.67741  2.69355  4.24690  2.90347  2.73739  3.18146  2.89801
2.37887  2.77519  2.98518  4.58477  3.61503
          0.01027  4.97991  5.70225  0.61958  0.77255  0.48576  0.95510
      4   1.93634  5.29967  2.15254  2.18405  4.61288  3.65500  3.84578
4.07696  2.43891  3.12758  3.53822  3.13594  3.73087  2.95344  2.85613
2.54327  2.63587  3.11326  5.75194  4.36107      4 a - - -
          2.68618  4.42225  2.77519  2.73123  3.46354  2.40513  3.72494
3.29354  2.67741  2.69355  4.24690  2.90347  2.73739  3.18146  2.89801
2.37887  2.77519  2.98518  4.58477  3.61503
          0.01027  4.97991  5.70225  0.61958  0.77255  0.48576  0.95510
      5   3.34791  5.94679  0.90217  2.49379  5.26000  3.30495  4.19644
4.77823  3.10162  4.24018  5.03785  2.18034  4.32265  3.33458  2.37930
3.24832  3.61290  4.32857  6.37632  4.89207      5 d - - -
          2.68618  4.42225  2.77519  2.73123  3.46354  2.40513  3.72494
3.29354  2.67741  2.69355  4.24690  2.90347  2.73739  3.18146  2.89801
2.37887  2.77519  2.98518  4.58477  3.61503
          0.01027  4.97991  5.70225  0.61958  0.77255  0.48576  0.95510
      6   2.94533  5.53136  3.34756  2.93425  4.97843  3.88623  4.11281
4.39609  2.09503  3.89089  4.70811  3.43142  1.01367  2.28172  2.99646
3.23847  3.49189  4.01259  6.01194  4.71014      6 p - - -
          2.68618  4.42225  2.77519  2.73123  3.46354  2.40513  3.72494
3.29354  2.67741  2.69355  4.24690  2.90347  2.73739  3.18146  2.89801
2.37887  2.77519  2.98518  4.58477  3.61503
          0.01027  4.97991  5.70225  0.61958  0.77255  0.48576  0.95510
      7   3.61368  6.27393  2.47654  0.52394  5.51536  3.82271  2.97015
5.10892  3.41752  4.56030  5.41963  3.27006  4.46231  3.55198  4.02404
3.47031  3.90551  4.65376  6.67565  5.12895      7 E - - -
          2.68618  4.42225  2.77519  2.73123  3.46354  2.40513  3.72494
3.29354  2.67741  2.69355  4.24690  2.90347  2.73739  3.18146  2.89801
2.37887  2.77519  2.98518  4.58477  3.61503
          0.01027  4.97991  5.70225  0.61958  0.77255  0.48576  0.95510
      8   4.65159  5.84612  5.94763  5.68955  3.97246  5.38137  6.06973
3.29624  5.50279  0.18621  3.82118  5.90786  5.70462  5.56685  5.46040
5.28896  4.92264  3.58815  6.10176  5.05276      8 L - - -
          2.68618  4.42225  2.77519  2.73123  3.46354  2.40513  3.72494
3.29354  2.67741  2.69355  4.24690  2.90347  2.73739  3.18146  2.89801
2.37887  2.77519  2.98518  4.58477  3.61503
          0.01027  4.97991  5.70225  0.61958  0.77255  0.48576  0.95510
      9   4.60304  6.52241  0.13921  3.67921  6.15238  4.46546  5.40701
6.08324  4.73574  5.48643  6.58716  4.25584  5.17461  4.71693  5.29637
4.51669  4.98530  5.63188  7.01704  5.99118      9 D - - -
          2.68618  4.42225  2.77519  2.73123  3.46354  2.40513  3.72494
3.29354  2.67741  2.69355  4.24690  2.90347  2.73739  3.18146  2.89801
2.37887  2.77519  2.98518  4.58477  3.61503
          0.01027  4.97991  5.70225  0.61958  0.77255  0.48576  0.95510
     10   2.95125  5.47396  2.41168  1.82505  4.81575  3.66796  2.96852
4.30529  2.54797  3.77635  4.51957  2.70294  4.08888  1.54356  2.92592
2.90455  3.18625  3.86364  5.90320  4.48407     10 q - - -
          2.68618  4.42225  2.77519  2.73123  3.46354  2.40513  3.72494
3.29354  2.67741  2.69355  4.24690  2.90347  2.73739  3.18146  2.89801
2.37887  2.77519  2.98518  4.58477  3.61503
          0.01027  4.97991  5.70225  0.61958  0.77255  0.48576  0.95510
```

```
     11   4.98929  6.07484  5.80065  5.77768  0.16685  5.20081  4.85423
4.56571  5.70933  3.71150  5.15952  5.45501  5.69530  5.61373  5.56800
5.12971  5.31757  4.61012  4.95495  3.26218     11 F - - -
          2.68618  4.42225  2.77519  2.73123  3.46354  2.40513  3.72494
3.29354  2.67741  2.69355  4.24690  2.90347  2.73739  3.18146  2.89801
2.37887  2.77519  2.98518  4.58477  3.61503
          0.01027  4.97991  5.70225  0.61958  0.77255  0.48576  0.95510
     12   4.26523  5.50437  5.99374  5.47415  3.59342  5.52677  5.83341
2.83245  5.21233  1.45490  0.59616  5.69543  5.60155  5.20928  5.20438
4.98200  4.48602  3.10271  5.83821  4.81090     12 M - - -
          2.68618  4.42225  2.77519  2.73123  3.46354  2.40513  3.72494
3.29354  2.67741  2.69355  4.24690  2.90347  2.73739  3.18146  2.89801
2.37887  2.77519  2.98518  4.58477  3.61503
          0.01027  4.97991  5.70225  0.61958  0.77255  0.48576  0.95510
     13   2.25141  5.13883  3.26424  1.23068  4.50953  3.29617  3.98388
3.93623  2.77883  3.52118  4.31761  3.26629  4.12041  3.12340  3.24867
2.57522  2.76825  2.58185  5.73833  4.38889     13 e - - -
          2.68618  4.42225  2.77519  2.73123  3.46354  2.40513  3.72494
3.29354  2.67741  2.69355  4.24690  2.90347  2.73739  3.18146  2.89801
2.37887  2.77519  2.98518  4.58477  3.61503
          0.01027  4.97991  5.70225  0.61958  0.77255  0.48576  0.95510
     14   1.65743  5.10316  3.26917  2.71682  4.37105  3.69789  2.83772
3.45983  2.70714  3.38656  4.19267  3.23620  4.09723  2.38385  3.17030
2.41071  2.06173  3.45720  5.61925  4.27884     14 a - - -
          2.68618  4.42225  2.77519  2.73123  3.46354  2.40513  3.72494
3.29354  2.67741  2.69355  4.24690  2.90347  2.73739  3.18146  2.89801
2.37887  2.77519  2.98518  4.58477  3.61503
          0.01027  4.97991  5.70225  0.61958  0.77255  0.48576  0.95510
     15   5.11010  5.99753  5.83737  5.82250  2.11561  5.50555  4.13432
4.65022  5.59846  3.75402  5.16506  5.04395  5.76998  5.23145  5.38125
4.94727  5.30924  4.62644  4.20449  0.29265     15 Y - - -
          2.68618  4.42225  2.77519  2.73123  3.46354  2.40513  3.72494
3.29354  2.67741  2.69355  4.24690  2.90347  2.73739  3.18146  2.89801
2.37887  2.77519  2.98518  4.58477  3.61503
          0.01027  4.97991  5.70225  0.61958  0.77255  0.48576  0.95510
     16   2.93594  1.32246  4.70473  4.10511  3.39726  4.08409  3.55682
2.58724  3.92579  2.23050  3.44929  4.17838  4.44830  4.09550  4.00233
3.38876  3.16877  1.89872  4.93540  3.05387     16 c - - -
          2.68618  4.42225  2.77519  2.73123  3.46354  2.40513  3.72494
3.29354  2.67741  2.69355  4.24690  2.90347  2.73739  3.18146  2.89801
2.37887  2.77519  2.98518  4.58477  3.61503
          0.01027  4.97991  5.70225  0.61958  0.77255  0.48576  0.95510
     17   2.39680  5.34937  2.14701  2.27907  4.68228  3.64615  3.08903
4.15974  2.45744  2.45057  4.39326  2.65784  4.03944  2.54684  2.71825
2.83600  3.09920  3.14449  5.78935  3.80130     17 d - - -
          2.68618  4.42225  2.77519  2.73123  3.46354  2.40513  3.72494
3.29354  2.67741  2.69355  4.24690  2.90347  2.73739  3.18146  2.89801
2.37887  2.77519  2.98518  4.58477  3.61503
          0.01027  4.97991  5.70225  0.61958  0.77255  0.48576  0.95510
     18   3.76506  5.00414  5.78641  5.23612  3.80993  5.18962  5.62801
1.84308  5.10655  1.57323  1.67015  5.34986  5.37578  5.15860  5.13219
4.56698  3.99760  1.17317  5.85677  4.77827     18 v - - -
          2.68618  4.42225  2.77519  2.73123  3.46354  2.40513  3.72494
3.29354  2.67741  2.69355  4.24690  2.90347  2.73739  3.18146  2.89801
2.37887  2.77519  2.98518  4.58477  3.61503
          0.01027  4.97991  5.70225  0.61958  0.77255  0.48576  0.95510
```

19 4.48395 5.63980 6.33366 5.77749 3.56222 5.88823 6.12022
2.83570 5.59159 0.33072 2.43338 6.06976 5.76673 5.37143 5.49116
5.35528 4.67226 3.20053 5.91478 5.01158 19 L - - -
2.68618 4.42225 2.77519 2.73123 3.46354 2.40513 3.72494
3.29354 2.67741 2.69355 4.24690 2.90347 2.73739 3.18146 2.89801
2.37887 2.77519 2.98518 4.58477 3.61503
0.01027 4.97991 5.70225 0.61958 0.77255 0.48576 0.95510
20 2.65731 2.60131 3.76915 2.88851 3.73779 3.86600 3.43661
2.78161 2.61092 2.65128 3.70509 3.17634 4.24529 3.45607 3.50660
2.67453 2.89448 1.59587 5.18408 3.95054 20 v - - -
2.68618 4.42225 2.77519 2.73123 3.46354 2.40513 3.72494
3.29354 2.67741 2.69355 4.24690 2.90347 2.73739 3.18146 2.89801
2.37887 2.77519 2.98518 4.58477 3.61503
0.01027 4.97991 5.70225 0.61958 0.77255 0.48576 0.95510
21 2.74806 5.46427 3.27506 2.31619 4.83512 3.76376 3.90397
4.28955 1.49575 3.75411 4.51576 3.23825 4.15368 3.00788 1.96014
1.87907 3.23981 3.87255 5.86233 4.50362 21 k - - -
2.68618 4.42225 2.77519 2.73123 3.46354 2.40513 3.72494
3.29354 2.67741 2.69355 4.24690 2.90347 2.73739 3.18146 2.89801
2.37887 2.77519 2.98518 4.58477 3.61503
0.01027 4.97991 5.70225 0.61958 0.77255 0.48576 0.95510
22 5.16354 6.01023 5.88819 5.89429 1.16733 5.54477 4.11628
4.68557 5.66224 3.77434 5.18779 5.05068 5.79162 5.24561 5.41373
4.97260 5.35061 4.66177 4.18277 0.58324 22 Y - - -
2.68618 4.42225 2.77519 2.73123 3.46354 2.40513 3.72494
3.29354 2.67741 2.69355 4.24690 2.90347 2.73739 3.18146 2.89801
2.37887 2.77519 2.98518 4.58477 3.61503
0.01027 4.97991 5.70225 0.61958 0.77255 0.48576 0.95510
23 3.38827 5.72191 3.65338 2.12888 5.18906 3.43865 4.04054
4.57118 1.00703 3.96891 4.78009 3.51476 4.41199 3.15254 1.68399
3.34625 3.57003 4.18770 5.99978 4.75829 23 k - - -
2.68618 4.42225 2.77519 2.73123 3.46354 2.40513 3.72494
3.29354 2.67741 2.69355 4.24690 2.90347 2.73739 3.18146 2.89801
2.37887 2.77519 2.98518 4.58477 3.61503
0.01027 4.97991 5.70225 0.61958 0.77255 0.48576 0.95510
24 2.89704 5.37655 2.60616 1.26491 4.70611 3.65586 3.85905
3.54501 2.60744 3.34747 4.42330 3.12793 4.05927 2.32810 3.09995
2.46606 2.96701 3.75901 5.81789 4.41271 24 e - - -
2.68618 4.42225 2.77519 2.73123 3.46354 2.40513 3.72494
3.29354 2.67741 2.69355 4.24690 2.90347 2.73739 3.18146 2.89801
2.37887 2.77519 2.98518 4.58477 3.61503
0.01027 4.97991 5.70225 0.61958 0.77255 0.48576 0.95510
25 3.52064 6.19462 2.07818 0.83787 5.48216 3.79342 4.31632
5.02990 3.31703 4.47657 5.30684 3.23624 4.40860 1.79553 3.92134
3.38712 3.80292 4.56589 6.61240 5.07658 25 e - - -
2.68618 4.42225 2.77519 2.73123 3.46354 2.40513 3.72494
3.29354 2.67741 2.69355 4.24690 2.90347 2.73739 3.18146 2.89801
2.37887 2.77519 2.98518 4.58477 3.61503
0.01027 4.97991 5.70225 0.61958 0.77255 0.48576 0.95510
26 4.49380 5.64355 6.25011 5.76132 2.47672 5.79166 5.68143
2.92221 5.61636 0.33691 3.37372 5.89939 5.74086 5.35592 5.49510
5.26365 4.68612 3.27784 5.58732 4.31239 26 L - - -
2.68618 4.42225 2.77519 2.73123 3.46354 2.40513 3.72494
3.29354 2.67741 2.69355 4.24690 2.90347 2.73739 3.18146 2.89801
2.37887 2.77519 2.98518 4.58477 3.61503
0.01027 4.97991 5.70225 0.61958 0.77255 0.48576 0.95510

```
     27   2.44815  5.21162  3.22169  2.66481  4.50826  3.68508  3.88648
3.94819  2.63943  3.05464  4.28577  3.19453  4.08132  2.02137  2.43645
2.11034  1.91720  3.58002  5.69661  4.33465     27 t - - -
          2.68618  4.42225  2.77519  2.73123  3.46354  2.40513  3.72494
3.29354  2.67741  2.69355  4.24690  2.90347  2.73739  3.18146  2.89801
2.37887  2.77519  2.98518  4.58477  3.61503
          0.01027  4.97991  5.70225  0.61958  0.77255  0.48576  0.95510
     28   3.38855  5.71119  3.74725  3.07584  5.18489  4.06034  3.08401
4.56054  1.87397  3.95309  4.76388  2.66689  4.41545  2.03106  1.10807
3.34954  3.56407  4.17981  5.97780  4.74731     28 r - - -
          2.68618  4.42225  2.77519  2.73123  3.46354  2.40513  3.72494
3.29354  2.67741  2.69355  4.24690  2.90347  2.73739  3.18146  2.89801
2.37887  2.77519  2.98518  4.58477  3.61503
          0.01027  4.97991  5.70225  0.61958  0.77255  0.48576  0.95510
     29   2.96121  4.88339  3.68237  3.21837  4.25252  3.77225  2.05031
3.65718  3.14866  3.34334  4.21883  3.61940  1.16521  3.52796  3.51085
3.10518  2.87036  2.59127  5.63123  4.34909     29 p - - -
          2.68618  4.42225  2.77519  2.73123  3.46354  2.40513  3.72494
3.29354  2.67741  2.69355  4.24690  2.90347  2.73739  3.18146  2.89801
2.37887  2.77519  2.98518  4.58477  3.61503
          0.01027  4.97991  5.70225  0.61958  0.77255  0.48576  0.95510
     30   3.67752  4.93961  5.68456  5.11895  1.66693  5.03920  5.42097
1.64071  4.96833  1.49072  3.53673  5.19963  5.25398  5.01643  4.97818
4.40075  3.90757  1.45679  5.68466  4.57242     30 v - - -
          2.68618  4.42225  2.77519  2.73123  3.46354  2.40513  3.72494
3.29354  2.67741  2.69355  4.24690  2.90347  2.73739  3.18146  2.89801
2.37887  2.77519  2.98518  4.58477  3.61503
          0.01027  4.97991  5.70225  0.61958  0.77255  0.48576  0.95510
     31   2.87529  3.73368  2.23273  1.97537  4.72984  3.64529  3.59328
4.21516  2.32306  3.68931  4.42585  2.67387  4.04024  2.07247  2.22841
2.83724  3.10642  3.77549  5.81649  4.40636     31 e - - -
          2.68534  4.42256  2.77551  2.73154  3.46385  2.40544  3.72202
3.29327  2.67772  2.69386  4.24470  2.90378  2.73771  3.18177  2.89832
2.37918  2.77551  2.98354  4.58508  3.61534
          0.22732  1.60944  5.70225  1.29339  0.32067  0.48576  0.95510
     32   3.82193  6.15639  3.02212  0.38524  5.77049  2.56692  4.73765
5.37564  3.80825  4.85033  5.75752  3.60596  4.68794  3.94050  4.36393
3.73783  4.17537  4.88311  6.84129  5.47023     36 E - - -
          2.68618  4.42225  2.77519  2.73123  3.46354  2.40513  3.72494
3.29354  2.67741  2.69355  4.24690  2.90347  2.73739  3.18146  2.89801
2.37887  2.77519  2.98518  4.58477  3.61503
          0.01027  4.97991  5.70225  0.61958  0.77255  0.48576  0.95510
     33   0.18380  5.15806  4.99517  4.96359  5.56266  3.93778  5.77642
4.92866  5.01937  4.72202  5.68294  4.71940  4.76863  5.24283  5.09089
3.53481  3.88412  4.32818  6.81237  5.82929     37 A - - -
          2.68618  4.42225  2.77519  2.73123  3.46354  2.40513  3.72494
3.29354  2.67741  2.69355  4.24690  2.90347  2.73739  3.18146  2.89801
2.37887  2.77519  2.98518  4.58477  3.61503
          0.01027  4.97991  5.70225  0.61958  0.77255  0.48576  0.95510
     34   2.38580  4.69248  2.89907  2.85772  3.84426  3.82668  4.06772
2.58154  2.72606  2.91072  2.35057  3.50749  4.20878  3.35002  3.42028
2.86134  2.08061  2.14393  5.26244  4.01100     38 t - - -
          2.68618  4.42225  2.77519  2.73123  3.46354  2.40513  3.72494
3.29354  2.67741  2.69355  4.24690  2.90347  2.73739  3.18146  2.89801
2.37887  2.77519  2.98518  4.58477  3.61503
          0.01027  4.97991  5.70225  0.61958  0.77255  0.48576  0.95510
```

```
     35   2.46934  5.25305  2.91116  1.79724  4.54838  3.66502  3.85940
4.00051  2.61781  3.07997  2.77121  3.15528  4.05796  2.67448  3.10056
2.31017  2.14954  3.61672  5.71721  4.33849     39 e - - -
          2.68618  4.42225  2.77519  2.73123  3.46354  2.40513  3.72494
3.29354  2.67741  2.69355  4.24690  2.90347  2.73739  3.18146  2.89801
2.37887  2.77519  2.98518  4.58477  3.61503
          0.01027  4.97991  5.70225  0.61958  0.77255  0.48576  0.95510
     36   1.65365  5.03627  4.99655  4.72647  0.64813  4.20301  4.77534
3.61091  4.64751  3.17381  4.37554  4.59349  4.84758  4.78052  4.73592
3.67705  3.82667  3.44570  5.12903  3.53810     40 F - - -
          2.68618  4.42225  2.77519  2.73123  3.46354  2.40513  3.72494
3.29354  2.67741  2.69355  4.24690  2.90347  2.73739  3.18146  2.89801
2.37887  2.77519  2.98518  4.58477  3.61503
          0.01027  4.97991  5.70225  0.61958  0.77255  0.48576  0.95510
     37   3.46397  1.64731  5.40009  4.79634  2.25851  4.69084  5.00213
2.46736  4.60889  1.09662  2.91518  4.84505  4.95345  4.65280  4.60132
4.02133  3.68669  2.76380  5.33431  4.22518     41 l - - -
          2.68618  4.42225  2.77519  2.73123  3.46354  2.40513  3.72494
3.29354  2.67741  2.69355  4.24690  2.90347  2.73739  3.18146  2.89801
2.37887  2.77519  2.98518  4.58477  3.61503
          0.01027  4.97991  5.70225  0.61958  0.77255  0.48576  0.95510
     38   2.88857  5.36712  3.14998  2.49822  4.70576  3.19692  3.84296
4.18057  1.98196  3.66552  4.41111  2.39289  4.05489  2.60906  1.85047
2.57613  2.79970  3.75467  3.20917  4.40245     42 r - - -
          2.68618  4.42225  2.77519  2.73123  3.46354  2.40513  3.72494
3.29354  2.67741  2.69355  4.24690  2.90347  2.73739  3.18146  2.89801
2.37887  2.77519  2.98518  4.58477  3.61503
          0.01027  4.97991  5.70225  0.61958  0.77255  0.48576  0.95510
     39   2.87111  5.38093  2.52743  1.85307  4.72547  2.99477  3.83117
4.21116  2.16749  3.34633  4.42196  2.67518  4.03721  2.92793  2.22779
2.44976  2.62219  3.77133  5.81368  4.40283     43 e - - -
          2.68618  4.42225  2.77519  2.73123  3.46354  2.40513  3.72494
3.29354  2.67741  2.69355  4.24690  2.90347  2.73739  3.18146  2.89801
2.37887  2.77519  2.98518  4.58477  3.61503
          0.01027  4.97991  5.70225  0.61958  0.77255  0.48576  0.95510
     40   2.73118  4.36314  4.50760  3.91538  3.46619  4.04791  4.36134
1.32359  2.87847  2.30561  2.51347  4.07507  4.41379  3.36837  3.91230
3.34202  2.91813  2.08301  4.98931  3.80246     44 i - - -
          2.68618  4.42225  2.77519  2.73123  3.46354  2.40513  3.72494
3.29354  2.67741  2.69355  4.24690  2.90347  2.73739  3.18146  2.89801
2.37887  2.77519  2.98518  4.58477  3.61503
          0.01027  4.97991  5.70225  0.61958  0.77255  0.48576  0.95510
     41   3.11090  5.56052  3.09424  0.86893  4.89894  3.76632  3.60542
4.36064  2.57098  3.84377  4.62175  3.23734  4.20730  2.95353  2.94078
3.06594  2.64502  3.95210  5.97380  4.59216     45 e - - -
          2.68618  4.42225  2.77519  2.73123  3.46354  2.40513  3.72494
3.29354  2.67741  2.69355  4.24690  2.90347  2.73739  3.18146  2.89801
2.37887  2.77519  2.98518  4.58477  3.61503
          0.01027  4.97991  5.70225  0.61958  0.77255  0.48576  0.95510
     42   2.26212  4.34144  2.65064  2.47040  4.60780  3.65584  3.36733
4.07087  2.59638  3.58707  3.43949  3.13764  4.04869  2.02538  3.08287
2.02377  2.36662  3.66866  5.74936  4.35951     46 s - - -
          2.68618  4.42225  2.77519  2.73123  3.46354  2.40513  3.72494
3.29354  2.67741  2.69355  4.24690  2.90347  2.73739  3.18146  2.89801
2.37887  2.77519  2.98518  4.58477  3.61503
          0.01027  4.97991  5.70225  0.61958  0.77255  0.48576  0.95510
```

```
     43   3.21451  5.68946  2.94769  2.43572  5.04923  3.76278  4.10948
4.53319  2.89692  4.01763  4.80699  2.67011  4.26919  0.97976  3.37050
2.06518  3.46930  4.10748  6.15429  4.73631     47 q - - -
          2.68618  4.42225  2.77519  2.73123  3.46354  2.40513  3.72494
3.29354  2.67741  2.69355  4.24690  2.90347  2.73739  3.18146  2.89801
2.37887  2.77519  2.98518  4.58477  3.61503
          0.01027  4.97991  5.70225  0.61958  0.77255  0.48576  0.95510
     44   4.38515  5.54353  6.33115  5.74177  2.88298  5.82490  6.03707
2.22214  5.62079  0.40495  2.90581  5.99684  5.70927  5.33958  5.49321
5.24633  4.56827  3.13669  5.86487  4.96302     48 L - - -
          2.68618  4.42225  2.77519  2.73123  3.46354  2.40513  3.72494
3.29354  2.67741  2.69355  4.24690  2.90347  2.73739  3.18146  2.89801
2.37887  2.77519  2.98518  4.58477  3.61503
          0.01027  4.97991  5.70225  0.61958  0.77255  0.48576  0.95510
     45   2.75822  5.37420  2.37124  2.56456  4.71535  2.92048  2.92330
3.70985  2.47158  3.67783  4.41621  2.20579  4.03881  2.13252  3.06354
2.20232  2.87092  3.76280  5.80914  4.39995     49 q - - -
          2.68618  4.42225  2.77519  2.73123  3.46354  2.40513  3.72494
3.29354  2.67741  2.69355  4.24690  2.90347  2.73739  3.18146  2.89801
2.37887  2.77519  2.98518  4.58477  3.61503
          0.01027  4.97991  5.70225  0.61958  0.77255  0.48576  0.95510
     46   2.10704  5.03635  3.15329  2.74215  4.26771  3.71286  3.92280
3.67816  2.64038  2.83008  3.57339  2.49114  4.10373  3.08161  3.19285
1.61551  2.64346  3.03286  5.54989  4.22351     50 s - - -
          2.68618  4.42225  2.77519  2.73123  3.46354  2.40513  3.72494
3.29354  2.67741  2.69355  4.24690  2.90347  2.73739  3.18146  2.89801
2.37887  2.77519  2.98518  4.58477  3.61503
          0.04289  4.97991  3.34941  0.61958  0.77255  0.48576  0.95510
     47   4.22798  5.41017  6.17725  5.60377  2.56944  5.64317  5.85822
1.62096  5.47443  0.58823  3.30124  5.80188  5.61193  5.26907  5.38179
5.05087  4.42446  2.95915  5.78212  4.77838     51 L - - -
          2.68618  4.42225  2.77519  2.73123  3.46354  2.40513  3.72494
3.29354  2.67741  2.69355  4.24690  2.90347  2.73739  3.18146  2.89801
2.37887  2.77519  2.98518  4.58477  3.61503
          0.15072  4.94763  2.01882  0.61958  0.77255  0.56991  0.83373
     48   2.24992  1.41845  4.70048  4.35546  4.64844  3.45457  5.02946
4.00455  4.24927  3.76184  4.61248  4.12149  4.24352  4.45169  4.40711
2.31543  1.12520  3.50737  6.07686  4.93799     52 t - - -
          2.68618  4.42225  2.77519  2.73123  3.46354  2.40513  3.72494
3.29354  2.67741  2.69355  4.24690  2.90347  2.73739  3.18146  2.89801
2.37887  2.77519  2.98518  4.58477  3.61503
          0.00822  4.80513        *  0.61958  0.77255  0.00000        *
//
HMMER3/f [3.3 | Nov 2019]
NAME  LIM
ACC   PF00412.23
DESC  LIM domain
LENG  58
ALPH  amino
RF    no
MM    no
CONS  yes
CS    yes
MAP   yes
DATE  Wed Apr 22 10:18:45 2020
NSEQ  34
EFFN  2.784912
CKSUM 230097389
```

```
GA    22.90 22.90
TC    22.90 22.90
NC    22.80 22.80
STATS LOCAL MSV       -8.7031  0.71899
STATS LOCAL VITERBI   -9.7713  0.71899
STATS LOCAL FORWARD   -3.9524  0.71899
HMM          A        C        D        E        F        G        H
I        K        L        M        N        P        Q        R        S
T        V        W        Y
            m->m     m->i     m->d     i->m     i->i     d->m     d->d
  COMPO   2.71344  2.31549  2.98157  2.64799  3.19476  3.20067  3.16011
3.13137  2.59234  2.67456  3.89008  3.13882  3.69730  3.04065  2.97301
2.81732  2.96639  2.97822  4.50580  3.18952
          2.68618  4.42225  2.77519  2.73123  3.46354  2.40513  3.72494
3.29354  2.67741  2.69355  4.24690  2.90347  2.73739  3.18146  2.89801
2.37887  2.77519  2.98518  4.58477  3.61503
          0.01147  4.86922  5.59157  0.61958  0.77255  0.00000        *
      1   4.19043  0.11254  5.68945  5.68136  5.65831  4.50964  6.15917
5.15133  5.60067  4.91657  6.08970  5.50793  5.25528  5.89809  5.52010
4.45489  4.72824  4.78100  6.69656  5.90809      1 C - - -
          2.68618  4.42225  2.77519  2.73123  3.46354  2.40513  3.72494
3.29354  2.67741  2.69355  4.24690  2.90347  2.73739  3.18146  2.89801
2.37887  2.77519  2.98518  4.58477  3.61503
          0.01147  4.86922  5.59157  0.61958  0.77255  0.48576  0.95510
      2   1.69013  5.16339  3.14936  2.41640  4.44350  2.92688  3.22693
3.88575  2.59325  3.23567  4.22262  3.12874  3.50542  2.69183  3.07164
2.20314  2.92415  3.51881  5.63789  3.26783      2 a - - T
          2.68618  4.42225  2.77519  2.73123  3.46354  2.40513  3.72494
3.29354  2.67741  2.69355  4.24690  2.90347  2.73739  3.18146  2.89801
2.37887  2.77519  2.98518  4.58477  3.61503
          0.01147  4.86922  5.59157  0.61958  0.77255  0.48576  0.95510
      3   2.36607  3.73839  3.18608  2.62784  4.38300  1.95896  3.84142
3.81429  2.27991  3.19816  4.18176  3.15581  4.03559  2.81525  2.26129
2.44901  3.06358  3.46480  5.60143  3.86690      3 g - - T
          2.68618  4.42225  2.77519  2.73123  3.46354  2.40513  3.72494
3.29354  2.67741  2.69355  4.24690  2.90347  2.73739  3.18146  2.89801
2.37887  2.77519  2.98518  4.58477  3.61503
          0.01147  4.86922  5.59157  0.61958  0.77255  0.48576  0.95510
      4   4.19043  0.11254  5.68945  5.68136  5.65831  4.50964  6.15917
5.15133  5.60067  4.91657  6.08970  5.50793  5.25528  5.89809  5.52010
4.45489  4.72824  4.78100  6.69656  5.90809      4 C - - T
          2.68618  4.42225  2.77519  2.73123  3.46354  2.40513  3.72494
3.29354  2.67741  2.69355  4.24690  2.90347  2.73739  3.18146  2.89801
2.37887  2.77519  2.98518  4.58477  3.61503
          0.01147  4.86922  5.59157  0.61958  0.77255  0.48576  0.95510
      5   2.53344  5.32394  2.55682  2.52257  4.66315  2.54223  3.11565
4.14510  2.14241  3.40533  3.98786  1.96048  3.99491  2.59548  3.01872
2.65365  3.05817  3.71148  5.75996  4.35235      5 n - - -
          2.68618  4.42225  2.77519  2.73123  3.46354  2.40513  3.72494
3.29354  2.67741  2.69355  4.24690  2.90347  2.73739  3.18146  2.89801
2.37887  2.77519  2.98518  4.58477  3.61503
          0.01147  4.86922  5.59157  0.61958  0.77255  0.48576  0.95510
      6   2.58728  5.33583  2.81890  2.08908  4.68059  3.01184  3.06591
3.80760  1.76833  3.63998  4.37701  3.06956  3.99536  2.47913  2.27370
2.79200  3.06016  3.72640  5.76771  4.35896      6 k - - -
          2.68618  4.42225  2.77519  2.73123  3.46354  2.40513  3.72494
3.29354  2.67741  2.69355  4.24690  2.90347  2.73739  3.18146  2.89801
2.37887  2.77519  2.98518  4.58477  3.61503
```

```
        0.01147  4.86922  5.59157  0.61958  0.77255  0.48576  0.95510
      7   2.64142  5.07088  3.20104  2.48500  3.81554  2.61045  3.84939
3.39894  2.30840  2.50870  4.13781  3.16828  2.76064  2.70506  2.79502
2.64263  2.61630  3.40967  5.56466  3.29683      7 k - - B
        2.68618  4.42225  2.77519  2.73123  3.46354  2.40513  3.72494
3.29354  2.67741  2.69355  4.24690  2.90347  2.73739  3.18146  2.89801
2.37887  2.77519  2.98518  4.58477  3.61503
        0.01147  4.86922  5.59157  0.61958  0.77255  0.48576  0.95510
      8   3.45570  4.72315  5.44737  4.91046  3.13910  4.86599  5.35781
0.81933  4.78608  2.42240  3.77594  5.00626  5.14654  4.96707  4.87835
4.23116  2.94896  1.49535  5.77720  4.59028      8 i - - E
        2.68618  4.42225  2.77519  2.73123  3.46354  2.40513  3.72494
3.29354  2.67741  2.69355  4.24690  2.90347  2.73739  3.18146  2.89801
2.37887  2.77519  2.98518  4.58477  3.61503
        0.01147  4.86922  5.59157  0.61958  0.77255  0.48576  0.95510
      9   2.43878  4.23716  3.07467  2.55265  3.66724  3.69203  3.90573
2.66680  2.63442  2.65162  3.97960  2.87360  3.74055  2.57875  2.85963
2.58292  2.67643  2.82596  5.42609  2.96159      9 a - - -
        2.68635  4.42242  2.77493  2.73091  3.46371  2.40512  3.72512
3.29371  2.67758  2.69372  4.24707  2.90364  2.73690  3.18098  2.89818
2.37875  2.77537  2.98536  4.58494  3.61520
        0.23689  2.06096  2.48178  1.23410  0.34404  0.48576  0.95510
     10   2.28469  5.28270  2.21071  2.24823  3.80924  2.88089  3.45113
4.09749  2.41132  3.58387  4.32609  3.04128  2.82891  2.85924  2.52482
2.45635  3.02598  3.66817  5.72115  4.31577     14 d - - T
        2.68618  4.42225  2.77519  2.73123  3.46354  2.40513  3.72494
3.29354  2.67741  2.69355  4.24690  2.90347  2.73739  3.18146  2.89801
2.37887  2.77519  2.98518  4.58477  3.61503
        0.01248  4.78599  5.50834  0.61958  0.77255  0.57583  0.82608
     11   2.34368  5.27209  2.84370  2.42346  4.60044  3.15411  3.77105
3.36279  2.33408  3.57017  4.31721  2.67585  3.97531  2.63038  2.12974
2.57167  2.33313  3.65372  5.71485  3.80949     15 r - - T
        2.68618  4.42225  2.77519  2.73123  3.46354  2.40513  3.72494
3.29354  2.67741  2.69355  4.24690  2.90347  2.73739  3.18146  2.89801
2.37887  2.77519  2.98518  4.58477  3.61503
        0.01216  4.81119  5.53354  0.61958  0.77255  0.41200  1.08568
     12   2.82498  4.03986  2.74436  1.81877  3.26157  3.33003  3.39781
3.61870  2.25742  3.45570  4.23372  3.12186  3.58781  2.60216  3.06465
2.82080  3.05621  2.86952  5.64731  2.95435     16 e - - T
        2.68618  4.42225  2.77519  2.73123  3.46354  2.40513  3.72494
3.29354  2.67741  2.69355  4.24690  2.90347  2.73739  3.18146  2.89801
2.37887  2.77519  2.98518  4.58477  3.61503
        0.01147  4.86922  5.59157  0.61958  0.77255  0.48576  0.95510
     13   2.54477  4.37769  4.10555  2.74705  3.28823  3.90704  4.19474
2.40132  3.01491  1.88047  3.20664  3.80843  4.27948  3.68577  3.04878
3.18025  2.52771  1.98933  4.98224  3.00354     17 l - - E
        2.68618  4.42225  2.77519  2.73123  3.46354  2.40513  3.72494
3.29354  2.67741  2.69355  4.24690  2.90347  2.73739  3.18146  2.89801
2.37887  2.77519  2.98518  4.58477  3.61503
        0.01147  4.86922  5.59157  0.61958  0.77255  0.48576  0.95510
     14   2.71691  3.23995  4.35229  3.13204  3.18464  3.95281  4.25907
2.46702  3.11730  2.08978  3.22098  3.95208  4.32165  3.84821  3.80101
2.79581  2.10134  1.71495  4.92012  3.73017     18 v - - E
        2.68618  4.42225  2.77519  2.73123  3.46354  2.40513  3.72494
3.29354  2.67741  2.69355  4.24690  2.90347  2.73739  3.18146  2.89801
2.37887  2.77519  2.98518  4.58477  3.61503
        0.33223  4.86922  1.29099  0.61958  0.77255  0.48576  0.95510
```

```
     15   2.71512  4.33572  3.77793  3.20386  3.45696  3.73883  4.00699
2.33530  2.93140  2.14791  2.52859  3.55402  4.11424  3.15780  2.33595
2.99827  2.56176  2.46544  4.92972  3.38997     19 l - - E
          2.68618  4.42225  2.77519  2.73123  3.46354  2.40513  3.72494
3.29354  2.67741  2.69355  4.24690  2.90347  2.73739  3.18146  2.89801
2.37887  2.77519  2.98518  4.58477  3.61503
          0.17184  4.55277  1.91498  0.61958  0.77255  0.62728  0.76367
     16   2.49067  5.21895  3.07236  2.50428  4.56125  3.55715  3.71319
4.01665  1.88814  3.50678  4.26921  2.71697  3.94568  2.23067  1.98538
2.63259  3.01471  3.26211  5.63994  4.27187     20 k - - E
          2.68618  4.42225  2.77519  2.73123  3.46354  2.40513  3.72494
3.29354  2.67741  2.69355  4.24690  2.90347  2.73739  3.18146  2.89801
2.37887  2.77519  2.98518  4.58477  3.61503
          0.01560  4.56425  5.28660  0.61958  0.77255  0.26546  1.45608
     17   1.33777  3.77055  4.29895  3.72074  2.66793  3.01431  4.27625
2.81327  3.60625  2.56618  3.23211  3.92410  4.31055  3.83042  3.79609
2.73208  3.08513  2.09339  4.98179  3.78920     21 a - - E
          2.68618  4.42225  2.77519  2.73123  3.46354  2.40513  3.72494
3.29354  2.67741  2.69355  4.24690  2.90347  2.73739  3.18146  2.89801
2.37887  2.77519  2.98518  4.58477  3.61503
          0.01147  4.86922  5.59157  0.61958  0.77255  0.48576  0.95510
     18   2.72224  4.99329  3.05328  2.56881  4.22171  3.35422  3.87340
3.63287  2.44024  1.99359  3.12172  2.77427  4.05643  2.90084  2.54440
2.68249  3.05904  2.97629  5.50281  2.88582     22 l - - T
          2.68618  4.42225  2.77519  2.73123  3.46354  2.40513  3.72494
3.29354  2.67741  2.69355  4.24690  2.90347  2.73739  3.18146  2.89801
2.37887  2.77519  2.98518  4.58477  3.61503
          0.01147  4.86922  5.59157  0.61958  0.77255  0.48576  0.95510
     19   3.36443  6.01085  1.64197  1.82352  5.31284  1.68235  4.19268
4.85017  3.17015  4.30500  5.11741  1.81813  4.29326  3.33997  3.76933
2.98310  3.64131  4.38841  6.44480  4.92451     23 d - - T
          2.68618  4.42225  2.77519  2.73123  3.46354  2.40513  3.72494
3.29354  2.67741  2.69355  4.24690  2.90347  2.73739  3.18146  2.89801
2.37887  2.77519  2.98518  4.58477  3.61503
          0.01147  4.86922  5.59157  0.61958  0.77255  0.48576  0.95510
     20   2.70759  4.53015  2.89496  2.35273  4.12725  3.60243  2.88840
4.11974  1.51906  3.15323  4.35148  2.79073  3.99559  2.74688  2.56894
2.69657  2.93282  3.69253  5.74696  4.34328     24 k - - E
          2.68618  4.42225  2.77519  2.73123  3.46354  2.40513  3.72494
3.29354  2.67741  2.69355  4.24690  2.90347  2.73739  3.18146  2.89801
2.37887  2.77519  2.98518  4.58477  3.61503
          0.01147  4.86922  5.59157  0.61958  0.77255  0.48576  0.95510
     21   2.61373  3.82690  2.86195  2.73842  4.13822  3.68418  3.60288
3.53956  2.50807  2.50259  4.00587  2.92229  3.39119  3.07070  2.63246
2.53461  2.82093  2.28676  5.44928  2.92285     25 v - - E
          2.68618  4.42225  2.77519  2.73123  3.46354  2.40513  3.72494
3.29354  2.67741  2.69355  4.24690  2.90347  2.73739  3.18146  2.89801
2.37887  2.77519  2.98518  4.58477  3.61503
          0.01147  4.86922  5.59157  0.61958  0.77255  0.48576  0.95510
     22   5.03037  5.88619  5.79643  5.78748  1.70001  5.44074  4.00607
4.58730  5.54971  3.69859  5.08988  4.93994  5.68174  5.13257  5.30125
4.85322  5.21280  4.54392  1.05558  1.07571     26 w - - E
          2.68618  4.42225  2.77519  2.73123  3.46354  2.40513  3.72494
3.29354  2.67741  2.69355  4.24690  2.90347  2.73739  3.18146  2.89801
2.37887  2.77519  2.98518  4.58477  3.61503
          0.01147  4.86922  5.59157  0.61958  0.77255  0.48576  0.95510
```

```
     23   4.68725  6.12316  4.70864  4.63942  4.55977  4.70631  0.14513
5.59389  4.50556  4.89499  6.08768  4.97444  5.33446  5.04815  4.65800
4.78716  5.06101  5.30012  5.94943  4.50403     27 H - - E
          2.68618  4.42225  2.77519  2.73123  3.46354  2.40513  3.72494
3.29354  2.67741  2.69355  4.24690  2.90347  2.73739  3.18146  2.89801
2.37887  2.77519  2.98518  4.58477  3.61503
          0.01147  4.86922  5.59157  0.61958  0.77255  0.48576  0.95510
     24   2.59792  4.80123  3.40584  2.18882  3.98335  3.72219  3.94455
2.53761  2.71619  2.44068  3.59114  3.32734  2.33157  2.99607  2.81063
2.81136  3.06260  2.55030  5.34580  4.06197     28 e - - T
          2.68618  4.42225  2.77519  2.73123  3.46354  2.40513  3.72494
3.29354  2.67741  2.69355  4.24690  2.90347  2.73739  3.18146  2.89801
2.37887  2.77519  2.98518  4.58477  3.61503
          0.01147  4.86922  5.59157  0.61958  0.77255  0.48576  0.95510
     25   2.32048  4.34397  2.65271  1.74856  4.67099  3.59871  3.53540
4.15493  2.35294  3.63297  4.37061  2.71215  3.49556  2.74855  2.87769
2.08554  3.05651  3.71802  5.76347  4.35414     29 e - - T
          2.68618  4.42225  2.77519  2.73123  3.46354  2.40513  3.72494
3.29354  2.67741  2.69355  4.24690  2.90347  2.73739  3.18146  2.89801
2.37887  2.77519  2.98518  4.58477  3.61503
          0.01147  4.86922  5.59157  0.61958  0.77255  0.48576  0.95510
     26   3.46336  0.47510  4.73311  4.44544  4.18494  4.10334  2.19249
4.28460  4.11458  3.93343  4.93583  4.50659  4.78191  4.58588  4.26952
3.68313  3.90225  3.95463  5.67272  4.23194     30 C - - T
          2.68618  4.42225  2.77519  2.73123  3.46354  2.40513  3.72494
3.29354  2.67741  2.69355  4.24690  2.90347  2.73739  3.18146  2.89801
2.37887  2.77519  2.98518  4.58477  3.61503
          0.01147  4.86922  5.59157  0.61958  0.77255  0.48576  0.95510
     27   4.11767  5.34111  5.76039  5.31282  0.67356  5.26902  4.91539
2.91768  5.13984  1.52046  3.50648  5.25180  5.42373  5.02991  5.09328
4.66402  4.33698  2.86335  4.99833  3.46446     31 F - - S
          2.68618  4.42225  2.77519  2.73123  3.46354  2.40513  3.72494
3.29354  2.67741  2.69355  4.24690  2.90347  2.73739  3.18146  2.89801
2.37887  2.77519  2.98518  4.58477  3.61503
          0.01147  4.86922  5.59157  0.61958  0.77255  0.48576  0.95510
     28   2.71924  3.43718  3.53375  2.97022  3.55796  3.76312  3.99876
2.67461  2.48716  2.75351  3.50341  3.14590  4.14607  2.52507  2.40831
2.99287  2.15866  2.43975  5.24588  3.98678     32 t - - B
          2.68618  4.42225  2.77519  2.73123  3.46354  2.40513  3.72494
3.29354  2.67741  2.69355  4.24690  2.90347  2.73739  3.18146  2.89801
2.37887  2.77519  2.98518  4.58477  3.61503
          0.01147  4.86922  5.59157  0.61958  0.77255  0.48576  0.95510
     29   4.19043  0.11254  5.68945  5.68136  5.65831  4.50964  6.15917
5.15133  5.60067  4.91657  6.08970  5.50793  5.25528  5.89809  5.52010
4.45489  4.72824  4.78100  6.69656  5.90809     33 C - - -
          2.68618  4.42225  2.77519  2.73123  3.46354  2.40513  3.72494
3.29354  2.67741  2.69355  4.24690  2.90347  2.73739  3.18146  2.89801
2.37887  2.77519  2.98518  4.58477  3.61503
          0.01147  4.86922  5.59157  0.61958  0.77255  0.48576  0.95510
     30   2.05340  3.28590  3.20679  2.69218  3.18108  3.74111  3.96991
3.11823  2.87878  2.79498  3.27256  3.37146  4.12556  2.63682  3.12820
1.98037  2.81442  3.03482  5.29634  3.66761     34 s - - T
          2.68618  4.42225  2.77519  2.73123  3.46354  2.40513  3.72494
3.29354  2.67741  2.69355  4.24690  2.90347  2.73739  3.18146  2.89801
2.37887  2.77519  2.98518  4.58477  3.61503
          0.01147  4.86922  5.59157  0.61958  0.77255  0.48576  0.95510
```

```
     31   2.58697  4.32742  2.28351  2.30371  3.99921  3.63138  3.59363
3.42445  2.37562  3.18805  3.81987  2.93251  4.02281  2.70323  3.07530
2.51069  2.51668  2.46811  5.62566  4.26136      35 d - - T
          2.68618  4.42225  2.77519  2.73123  3.46354  2.40513  3.72494
3.29354  2.67741  2.69355  4.24690  2.90347  2.73739  3.18146  2.89801
2.37887  2.77519  2.98518  4.58477  3.61503
          0.01147  4.86922  5.59157  0.61958  0.77255  0.48576  0.95510
     32   4.19043  0.11254  5.68945  5.68136  5.65831  4.50964  6.15917
5.15133  5.60067  4.91657  6.08970  5.50793  5.25528  5.89809  5.52010
4.45489  4.72824  4.78100  6.69656  5.90809      36 C - - T
          2.68618  4.42225  2.77519  2.73123  3.46354  2.40513  3.72494
3.29354  2.67741  2.69355  4.24690  2.90347  2.73739  3.18146  2.89801
2.37887  2.77519  2.98518  4.58477  3.61503
          0.01147  4.86922  5.59157  0.61958  0.77255  0.48576  0.95510
     33   2.82480  5.32612  2.78075  2.37714  3.74317  2.36070  2.97778
4.15055  2.19605  3.41870  4.36787  2.83037  3.99293  2.05633  2.64048
2.58377  3.05594  3.71474  5.76090  4.35244      37 q - - -
          2.68618  4.42225  2.77519  2.73123  3.46354  2.40513  3.72494
3.29354  2.67741  2.69355  4.24690  2.90347  2.73739  3.18146  2.89801
2.37887  2.77519  2.98518  4.58477  3.61503
          0.01147  4.86922  5.59157  0.61958  0.77255  0.48576  0.95510
     34   2.60779  3.98858  2.81566  2.19467  4.65253  3.60171  3.46768
4.13237  1.63578  3.39668  4.35829  3.07243  3.99490  2.49332  2.44614
2.79130  2.67602  3.47036  5.75253  4.34724      38 k - - S
          2.68618  4.42225  2.77519  2.73123  3.46354  2.40513  3.72494
3.29354  2.67741  2.69355  4.24690  2.90347  2.73739  3.18146  2.89801
2.37887  2.77519  2.98518  4.58477  3.61503
          0.01147  4.86922  5.59157  0.61958  0.77255  0.48576  0.95510
     35   2.72511  5.18215  3.14020  2.32487  3.60711  3.62469  3.28681
3.55376  2.58206  2.87901  4.23873  2.43586  2.36474  2.44177  2.91625
2.46467  2.82839  3.28046  5.65158  4.27907      39 e - - B
          2.68618  4.42225  2.77519  2.73123  3.46354  2.40513  3.72494
3.29354  2.67741  2.69355  4.24690  2.90347  2.73739  3.18146  2.89801
2.37887  2.77519  2.98518  4.58477  3.61503
          0.01147  4.86922  5.59157  0.61958  0.77255  0.48576  0.95510
     36   4.14913  5.34459  6.08624  5.49612  2.23147  5.53357  5.76107
2.01725  5.36009  0.56717  2.93293  5.69494  5.51220  5.15595  5.26507
4.92853  4.34199  2.99839  5.70235  4.74204      40 L - - -
          2.68618  4.42225  2.77519  2.73123  3.46354  2.40513  3.72494
3.29354  2.67741  2.69355  4.24690  2.90347  2.73739  3.18146  2.89801
2.37887  2.77519  2.98518  4.58477  3.61503
          0.01147  4.86922  5.59157  0.61958  0.77255  0.48576  0.95510
     37   2.48889  4.28771  2.80357  2.26707  4.59272  2.68850  3.79739
3.75516  2.17007  3.18203  4.32007  2.60753  3.63177  2.90240  2.91668
2.49741  2.35520  3.14003  5.72074  4.32544      41 k - - C
          2.68618  4.42225  2.77519  2.73123  3.46354  2.40513  3.72494
3.29354  2.67741  2.69355  4.24690  2.90347  2.73739  3.18146  2.89801
2.37887  2.77519  2.98518  4.58477  3.61503
          0.01147  4.86922  5.59157  0.61958  0.77255  0.48576  0.95510
     38   2.58085  5.32437  2.51503  2.22861  4.66528  3.15867  3.50573
4.14834  2.31438  3.62788  3.92652  2.81663  2.50279  2.77240  2.87992
2.37005  2.46222  3.41210  5.75954  4.35108      42 e - - T
          2.68618  4.42225  2.77519  2.73123  3.46354  2.40513  3.72494
3.29354  2.67741  2.69355  4.24690  2.90347  2.73739  3.18146  2.89801
2.37887  2.77519  2.98518  4.58477  3.61503
          0.01147  4.86922  5.59157  0.61958  0.77255  0.48576  0.95510
```

```
     39   2.82440  5.25534  2.81431  2.38919  4.56854  2.05057  3.54175
3.63883  2.10348  3.54686  3.47913  3.09196  4.00364  2.78193  2.49934
2.70566  2.58275  3.38566  4.55482  4.31681      43 g - - T
          2.68618  4.42225  2.77519  2.73123  3.46354  2.40513  3.72494
3.29354  2.67741  2.69355  4.24690  2.90347  2.73739  3.18146  2.89801
2.37887  2.77519  2.98518  4.58477  3.61503
          0.11052  4.86922  2.33350  0.61958  0.77255  0.48576  0.95510
     40   2.78956  5.13168  2.32369  2.33186  4.41299  2.57120  3.78711
3.02812  2.55378  3.17823  4.18992  2.96597  3.98318  2.90940  2.89794
2.31775  2.65034  3.48762  5.60462  2.80950      44 s - - .
          2.68637  4.42244  2.77506  2.73089  3.46287  2.40532  3.72514
3.29373  2.67760  2.69344  4.24709  2.90366  2.73727  3.18134  2.89805
2.37892  2.77492  2.98538  4.58496  3.61492
          0.66553  0.87738  2.65739  0.40509  1.09936  0.37492  1.16265
     41   2.63705  2.89519  4.45035  3.85508  2.12705  3.94386  3.30177
2.69104  3.70614  2.18818  3.36659  3.99103  3.02118  3.89719  3.82897
3.23945  2.48887  2.27763  4.86436  2.47439      49 f - - C
          2.68618  4.42225  2.77519  2.73123  3.46354  2.40513  3.72494
3.29354  2.67741  2.69355  4.24690  2.90347  2.73739  3.18146  2.89801
2.37887  2.77519  2.98518  4.58477  3.61503
          0.01221  4.80749  5.52984  0.61958  0.77255  0.40817  1.09322
     42   2.50390  4.36676  4.13909  3.55855  2.03000  3.58878  3.54335
2.82422  3.25388  2.34945  3.19859  3.82867  4.28562  3.70838  3.32539
3.01687  2.74499  2.53446  4.97203  1.94069      50 y - - E
          2.68618  4.42225  2.77519  2.73123  3.46354  2.40513  3.72494
3.29354  2.67741  2.69355  4.24690  2.90347  2.73739  3.18146  2.89801
2.37887  2.77519  2.98518  4.58477  3.61503
          0.01147  4.86922  5.59157  0.61958  0.77255  0.48576  0.95510
     43   2.83374  4.63459  3.59489  1.93665  3.30233  3.78151  4.02332
2.59564  2.99563  2.13639  3.73327  2.89862  4.16321  2.68486  3.37908
2.63762  3.06539  2.61746  3.70968  3.60589      51 e - - E
          2.68618  4.42225  2.77519  2.73123  3.46354  2.40513  3.72494
3.29354  2.67741  2.69355  4.24690  2.90347  2.73739  3.18146  2.89801
2.37887  2.77519  2.98518  4.58477  3.61503
          0.01147  4.86922  5.59157  0.61958  0.77255  0.48576  0.95510
     44   2.83452  4.72737  3.48655  2.92333  3.53346  3.74937  3.05433
2.59902  1.90363  2.66267  3.30075  3.38756  4.13318  3.06553  2.36705
2.97524  2.75164  2.40792  5.28374  4.01626      52 k - - E
          2.68618  4.42225  2.77519  2.73123  3.46354  2.40513  3.72494
3.29354  2.67741  2.69355  4.24690  2.90347  2.73739  3.18146  2.89801
2.37887  2.77519  2.98518  4.58477  3.61503
          0.01147  4.86922  5.59157  0.61958  0.77255  0.48576  0.95510
     45   2.85682  5.81991  1.35925  1.53422  5.13293  3.67757  4.08305
4.65130  2.44441  4.11184  4.89586  2.16827  4.21745  3.21425  3.53247
3.12403  3.47742  4.19816  6.24597  4.76482      53 d - - T
          2.68618  4.42225  2.77519  2.73123  3.46354  2.40513  3.72494
3.29354  2.67741  2.69355  4.24690  2.90347  2.73739  3.18146  2.89801
2.37887  2.77519  2.98518  4.58477  3.61503
          0.01147  4.86922  5.59157  0.61958  0.77255  0.48576  0.95510
     46   2.41437  5.36261  2.51658  2.29149  4.69963  1.79189  3.45593
4.18228  1.96884  3.66407  4.40818  3.07962  4.01843  2.91979  2.92784
2.68179  3.09660  3.74948  5.79847  4.38796      54 g - - T
          2.68611  4.42233  2.77511  2.73132  3.46362  2.40512  3.72503
3.29362  2.67719  2.69363  4.24698  2.90318  2.73748  3.18155  2.89809
2.37879  2.77528  2.98527  4.58485  3.61511
          0.27164  1.55177  3.64983  0.23781  1.55283  0.48576  0.95510
```

47 2.82702 5.32245 2.90400 2.19347 4.66474 3.59962 3.78360
4.14521 1.70071 2.90513 4.36479 2.92393 3.99290 2.43908 2.30689
2.68366 2.57109 3.71181 5.75459 4.35036 57 k - - E
2.68629 4.42236 2.77530 2.73092 3.46365 2.40523 3.72505
3.29292 2.67751 2.69366 4.24700 2.90357 2.73750 3.18157 2.89811
2.37839 2.77530 2.98529 4.58488 3.61514
0.09236 2.47213 5.56905 1.16947 0.37183 0.45363 1.00873
48 2.72244 4.27716 4.49984 3.90355 2.97053 3.97853 3.73669
1.94007 3.40407 1.43083 3.17333 4.03223 2.70429 3.93914 3.86767
3.27515 2.94398 2.40893 4.89352 3.70928 62 l - - E
2.68618 4.42225 2.77519 2.73123 3.46354 2.40513 3.72494
3.29354 2.67741 2.69355 4.24690 2.90347 2.73739 3.18146 2.89801
2.37887 2.77519 2.98518 4.58477 3.61503
0.01147 4.86922 5.59157 0.61958 0.77255 0.48576 0.95510
49 3.42062 4.75708 5.13822 4.59821 2.84643 4.55821 4.50293
2.85092 4.42027 1.43648 3.56034 4.58670 4.86255 4.49360 4.45282
3.88463 3.64731 1.95287 4.79109 1.23916 63 y - - E
2.68618 4.42225 2.77519 2.73123 3.46354 2.40513 3.72494
3.29354 2.67741 2.69355 4.24690 2.90347 2.73739 3.18146 2.89801
2.37887 2.77519 2.98518 4.58477 3.61503
0.01147 4.86922 5.59157 0.61958 0.77255 0.48576 0.95510
50 4.19043 0.11254 5.68945 5.68136 5.65831 4.50964 6.15917
5.15133 5.60067 4.91657 6.08970 5.50793 5.25528 5.89809 5.52010
4.45489 4.72824 4.78100 6.69656 5.90809 64 C - - E
2.68618 4.42225 2.77519 2.73123 3.46354 2.40513 3.72494
3.29354 2.67741 2.69355 4.24690 2.90347 2.73739 3.18146 2.89801
2.37887 2.77519 2.98518 4.58477 3.61503
0.01147 4.86922 5.59157 0.61958 0.77255 0.48576 0.95510
51 2.84153 5.34273 3.09100 2.10499 4.68960 2.92258 3.51237
4.17116 1.73680 3.32885 4.38435 2.84616 4.00491 2.31599 2.37225
2.49300 3.07130 3.73465 5.77094 4.36691 65 k - - H
2.68618 4.42225 2.77519 2.73123 3.46354 2.40513 3.72494
3.29354 2.67741 2.69355 4.24690 2.90347 2.73739 3.18146 2.89801
2.37887 2.77519 2.98518 4.58477 3.61503
0.01147 4.86922 5.59157 0.61958 0.77255 0.48576 0.95510
52 2.53452 5.32347 2.85640 2.43794 4.66407 3.39108 2.61320
4.14688 2.23717 3.62683 4.36532 2.71608 3.10117 2.56173 2.53772
2.41553 2.80175 3.48759 5.75881 3.17909 66 k - - H
2.68619 4.42226 2.77520 2.73124 3.46355 2.40514 3.72495
3.29355 2.67742 2.69356 4.24691 2.90348 2.73723 3.18147 2.89802
2.37888 2.77520 2.98519 4.58478 3.61504
0.06134 3.25540 3.86672 0.40458 1.10039 0.48576 0.95510
53 3.09688 1.91617 1.22572 2.79394 4.51221 3.71877 2.47176
4.09440 3.04208 3.68999 4.53254 3.31752 4.24334 3.33361 3.51647
3.11460 3.38052 3.74100 5.82250 4.42063 68 d - - H
2.68618 4.42225 2.77519 2.73123 3.46354 2.40513 3.72494
3.29354 2.67741 2.69355 4.24690 2.90347 2.73739 3.18146 2.89801
2.37887 2.77519 2.98518 4.58477 3.61503
0.01168 4.85188 5.57422 0.61958 0.77255 0.46056 0.99677
54 3.73067 5.09872 4.90389 4.45460 1.75693 4.64384 2.57631
3.15162 4.32436 3.16994 4.24231 4.41671 4.97717 4.40858 4.44164
2.81489 3.95444 3.46219 4.29750 0.88367 69 y - - H
2.68618 4.42225 2.77519 2.73123 3.46354 2.40513 3.72494
3.29354 2.67741 2.69355 4.24690 2.90347 2.73739 3.18146 2.89801
2.37887 2.77519 2.98518 4.58477 3.61503
0.05491 4.86922 3.08453 0.61958 0.77255 0.48576 0.95510

```
     55   2.41991  3.41168  4.04589  2.82756  2.44984  3.49754  3.28951
2.47283  3.38275  2.29758  3.48423  3.40409  4.25270  2.96151  3.63813
2.83163  3.05224  2.50028  3.72703  2.80838     70 l - - H
          2.68618  4.42225  2.77519  2.73123  3.46354  2.40513  3.72494
3.29354  2.67741  2.69355  4.24690  2.90347  2.73739  3.18146  2.89801
2.37887  2.77519  2.98518  4.58477  3.61503
          0.01198  4.82629  5.54864  0.61958  0.77255  0.48974  0.94878
     56   2.53716  5.32230  2.28617  2.34467  4.66506  3.35986  3.77906
3.79977  1.99285  3.62648  3.86763  2.82146  3.98531  2.48862  2.17803
2.67192  2.79308  3.71171  5.75624  4.34690     71 k - - H
          2.68618  4.42225  2.77519  2.73123  3.46354  2.40513  3.72494
3.29354  2.67741  2.69355  4.24690  2.90347  2.73739  3.18146  2.89801
2.37887  2.77519  2.98518  4.58477  3.61503
          0.01168  4.85182  5.57416  0.61958  0.77255  0.52757  0.89168
     57   2.31233  4.38178  2.74026  2.42465  3.92380  3.62979  3.82754
3.81879  2.23922  2.51846  3.37792  2.72046  4.02090  2.95759  2.33573
2.56552  2.87103  3.46527  4.38059  4.24140     72 k - - H
          2.68618  4.42225  2.77519  2.73123  3.46354  2.40513  3.72494
3.29354  2.67741  2.69355  4.24690  2.90347  2.73739  3.18146  2.89801
2.37887  2.77519  2.98518  4.58477  3.61503
          0.01168  4.85182  5.57416  0.61958  0.77255  0.52757  0.89168
     58   2.82242  4.89820  3.30863  2.49783  2.11990  3.68396  2.81463
3.50216  2.44230  3.14173  3.54696  3.25111  3.21763  3.07823  2.60212
2.76982  2.74919  2.99830  5.42435  3.21461     73 f - - C
          2.68618  4.42225  2.77519  2.73123  3.46354  2.40513  3.72494
3.29354  2.67741  2.69355  4.24690  2.90347  2.73739  3.18146  2.89801
2.37887  2.77519  2.98518  4.58477  3.61503
          0.00787  4.84801        *  0.61958  0.77255  0.00000        *
//
HMMER3/f [3.3 | Nov 2019]
NAME  MBF1
ACC   PF08523.11
DESC  Multiprotein bridging factor 1
LENG  75
ALPH  amino
RF    no
MM    no
CONS  yes
CS    no
MAP   yes
DATE  Sat Apr 18 03:02:58 2020
NSEQ  111
EFFN  4.258026
CKSUM 2616371805
GA    22.20 22.20
TC    22.80 22.60
NC    21.40 22.10
STATS LOCAL MSV       -8.9010  0.71864
STATS LOCAL VITERBI   -9.6300  0.71864
STATS LOCAL FORWARD   -3.7567  0.71864
HMM          A        C        D        E        F        G        H
I        K        L        M        N        P        Q        R        S
T        V        W        Y
            m->m     m->i     m->d     i->m     i->i     d->m     d->d
  COMPO   2.34869  5.15692  2.73455  2.53133  4.15629  2.84696  3.74845
3.15442  2.26411  2.97363  4.14199  2.84371  3.53675  2.90408  2.66394
2.68926  2.65138  2.73503  4.31429  4.20689
```

```
          2.68618  4.42225  2.77519  2.73123  3.46354  2.40513  3.72494
3.29354  2.67741  2.69355  4.24690  2.90347  2.73739  3.18146  2.89801
2.37887  2.77519  2.98518  4.58477  3.61503
          0.38868  5.21063  1.15015  0.61958  0.77255  0.00000        *
      1   2.86866  3.80616  2.36804  2.60588  4.61799  3.60771  3.88915
4.07973  2.66281  3.60979  4.38148  3.13974  4.04859  1.56593  3.14593
1.75877  3.12231  3.67248  5.77989  4.39513      1 q - - -
          2.68618  4.42225  2.77519  2.73123  3.46354  2.40513  3.72494
3.29354  2.67741  2.69355  4.24690  2.90347  2.73739  3.18146  2.89801
2.37887  2.77519  2.98518  4.58477  3.61503
          0.01189  4.83384  5.55619  0.61958  0.77255  0.36265  1.19016
      2   2.90659  6.49732  0.52392  2.17637  5.76241  3.62779  4.03623
5.33078  3.63401  4.77509  5.64388  3.36801  4.58869  3.44739  4.30320
3.61100  4.07257  4.85970  6.92432  5.33937      2 D - - -
          2.68618  4.42225  2.77519  2.73123  3.46354  2.40513  3.72494
3.29354  2.67741  2.69355  4.24690  2.90347  2.73739  3.18146  2.89801
2.37887  2.77519  2.98518  4.58477  3.61503
          0.00878  5.13554  5.85789  0.61958  0.77255  0.42463  1.06135
      3   4.32151  5.79225  5.42818  5.15677  3.52995  4.91015  4.90209
4.45166  4.69614  3.23687  5.04301  5.08617  3.71989  5.09745  4.77960
4.51926  4.67651  4.34572  0.28094  3.51636      3 W - - -
          2.68618  4.42225  2.77519  2.73123  3.46354  2.40513  3.72494
3.29354  2.67741  2.69355  4.24690  2.90347  2.73739  3.18146  2.89801
2.37887  2.77519  2.98518  4.58477  3.61503
          0.00826  5.19665  5.91899  0.61958  0.77255  0.53297  0.88397
      4   3.03887  5.81934  1.06042  1.73347  5.14556  3.83587  4.16338
4.64918  2.57925  4.11737  4.88102  2.53461  4.32440  3.28138  3.52251
3.07398  3.27324  3.91213  6.25001  4.79911      4 d - - -
          2.68618  4.42225  2.77519  2.73123  3.46354  2.40513  3.72494
3.29354  2.67741  2.69355  4.24690  2.90347  2.73739  3.18146  2.89801
2.37887  2.77519  2.98518  4.58477  3.61503
          0.00826  5.19665  5.91899  0.61958  0.77255  0.53297  0.88397
      5   2.91910  5.45447  2.77687  2.29310  4.78776  3.74364  3.84835
4.26598  2.56793  3.75486  4.49891  3.04212  2.29765  2.92207  3.16895
1.75599  2.05899  3.17995  5.89433  4.48963      5 s - - -
          2.68614  4.42227  2.77514  2.73121  3.46356  2.40515  3.72497
3.29356  2.67731  2.69357  4.24692  2.90349  2.73737  3.18148  2.89803
2.37889  2.77522  2.98520  4.58479  3.61505
          0.05595  2.96186  5.91899  0.29555  1.36307  0.45734  1.00231
      6   2.51599  5.02384  3.50723  2.80373  4.22243  3.86041  4.07724
3.39415  2.24668  3.26427  4.10916  3.44259  3.60023  2.72305  3.36095
2.97127  2.73734  1.26649  5.55818  4.26133      7 v - - -
          2.68618  4.42225  2.77519  2.73123  3.46354  2.40513  3.72494
3.29354  2.67741  2.69355  4.24690  2.90347  2.73739  3.18146  2.89801
2.37887  2.77519  2.98518  4.58477  3.61503
          0.00814  5.21063  5.93298  0.61958  0.77255  0.48576  0.95510
      7   3.01421  4.52329  4.36565  3.35354  3.63247  3.59117  4.39334
2.57302  3.67819  2.42629  3.40409  4.03434  4.23322  3.91791  3.50047
3.37710  1.25165  1.74303  5.13388  3.93659      8 t - - -
          2.68625  4.42232  2.77527  2.73130  3.46307  2.40520  3.72502
3.29353  2.67748  2.69362  4.24697  2.90354  2.73747  3.18153  2.89808
2.37894  2.77527  2.98497  4.58113  3.61510
          0.33760  1.91610  1.97086  0.14131  2.02662  0.48576  0.95510
      8   2.91706  4.86620  3.51160  2.94997  4.04406  3.41308  3.62002
2.68629  1.90877  3.09675  3.68389  2.86488  4.19951  3.26053  2.93716
2.90093  2.82263  1.75162  5.41400  3.31583     10 v - - -
```

```
          2.68618  4.42225  2.77519  2.73123  3.46354  2.40513  3.72494
3.29354  2.67741  2.69355  4.24690  2.90347  2.73739  3.18146  2.89801
2.37887  2.77519  2.98518  4.58477  3.61503
          0.00944  5.06365  5.78600  0.61958  0.77255  0.30208  1.34429
      9   3.15182  4.53627  4.96876  4.36900  3.27454  4.32762  4.66173
0.96964  3.06030  1.71272  3.44889  4.43660  4.67956  4.34923  4.25336
3.26349  3.38456  2.47581  5.18746  4.01269      11 i - - -
          2.68618  4.42225  2.77519  2.73123  3.46354  2.40513  3.72494
3.29354  2.67741  2.69355  4.24690  2.90347  2.73739  3.18146  2.89801
2.37887  2.77519  2.98518  4.58477  3.61503
          0.00814  5.21063  5.93298  0.61958  0.77255  0.48576  0.95510
     10   3.13504  5.54272  3.41307  2.84552  4.89864  1.36847  2.98354
4.34432  2.40329  3.82804  4.59881  3.36865  4.27595  2.93258  1.80283
2.84803  2.85854  3.94329  5.95175  4.60147      12 g - - -
          2.68618  4.42225  2.77519  2.73123  3.46354  2.40513  3.72494
3.29354  2.67741  2.69355  4.24690  2.90347  2.73739  3.18146  2.89801
2.37887  2.77519  2.98518  4.58477  3.61503
          0.00814  5.21063  5.93298  0.61958  0.77255  0.48576  0.95510
     11   2.83420  5.47523  3.07950  2.48931  3.67027  3.74393  3.64522
4.12494  1.44028  3.77951  4.51647  2.65471  3.87806  2.71383  2.49414
2.03501  3.20083  3.86490  5.90880  4.49943      13 k - - -
          2.68618  4.42225  2.77519  2.73123  3.46354  2.40513  3.72494
3.29354  2.67741  2.69355  4.24690  2.90347  2.73739  3.18146  2.89801
2.37887  2.77519  2.98518  4.58477  3.61503
          0.00814  5.21063  5.93298  0.61958  0.77255  0.48576  0.95510
     12   2.83662  5.48674  3.24220  2.68498  4.83260  2.81729  3.72868
4.31296  1.43939  3.78890  4.52918  2.58510  3.62802  2.93112  1.97545
2.72867  2.80252  3.87824  5.91616  4.51335      14 k - - -
          2.68618  4.42225  2.77519  2.73123  3.46354  2.40513  3.72494
3.29354  2.67741  2.69355  4.24690  2.90347  2.73739  3.18146  2.89801
2.37887  2.77519  2.98518  4.58477  3.61503
          0.00814  5.21063  5.93298  0.61958  0.77255  0.48576  0.95510
     13   1.85037  4.51458  3.28691  2.73023  4.18200  3.05164  3.62340
3.91900  2.13621  3.61048  4.38686  3.06220  2.97120  2.69622  2.75511
2.78903  2.63894  2.45773  5.79959  4.42681      15 a - - -
          2.68618  4.42225  2.77519  2.73123  3.46354  2.40513  3.72494
3.29354  2.67741  2.69355  4.24690  2.90347  2.73739  3.18146  2.89801
2.37887  2.77519  2.98518  4.58477  3.61503
          0.01280  5.21063  4.92550  0.61958  0.77255  0.48576  0.95510
     14   2.91528  5.45595  3.22576  2.56050  4.79199  3.61000  3.36824
4.27122  2.44865  3.75711  4.09045  2.99224  2.13913  2.45045  1.77392
2.55859  2.79191  3.60903  5.89383  3.82191      16 r - - -
          2.68636  4.42255  2.77543  2.73153  3.46384  2.40348  3.72525
3.29366  2.67771  2.69385  4.24720  2.90377  2.73723  3.18158  2.89800
2.37880  2.77537  2.98526  4.58507  3.61533
          1.16222  1.19341  0.95705  0.68832  0.69800  0.47594  0.97102
     15   2.11540  5.02527  3.16725  2.61929  4.31987  1.75993  3.82496
3.74599  2.44298  3.33034  4.13146  3.13781  3.99988  2.70902  2.89446
2.63385  2.66130  3.11615  5.55321  4.20964      20 g - - -
          2.68618  4.42225  2.77519  2.73123  3.46354  2.40513  3.72494
3.29354  2.67741  2.69355  4.24690  2.90347  2.73739  3.18146  2.89801
2.37887  2.77519  2.98518  4.58477  3.61503
          0.07521  4.73323  2.75430  0.61958  0.77255  1.32791  0.30792
     16   2.03643  5.05513  3.10946  2.55896  4.37322  2.32697  3.78299
3.81242  2.55248  3.37430  4.16002  3.02989  2.69434  2.72003  2.51723
2.50496  2.93172  3.16214  5.57576  4.21596      21 a - - -
```

2.68618 4.42225 2.77519 2.73123 3.46354 2.40513 3.72494
3.29354 2.67741 2.69355 4.24690 2.90347 2.73739 3.18146 2.89801
2.37887 2.77519 2.98518 4.58477 3.61503
0.01399 4.67202 5.39436 0.61958 0.77255 1.38168 0.28922
17 2.08174 5.09954 3.08278 2.52795 4.40033 2.37807 3.75904
3.84512 2.28692 3.39409 4.17281 3.06051 2.93581 2.83005 2.52403
2.64478 2.58041 3.24652 5.58456 4.21609 22 a - - -
2.68616 4.42240 2.77531 2.73114 3.46369 2.40528 3.72510
3.29369 2.67756 2.69367 4.24705 2.90355 2.73633 3.18113 2.89816
2.37900 2.77531 2.98527 4.58492 3.61475
1.24853 0.73579 1.45267 0.16970 1.85738 0.30499 1.33611
18 2.87545 5.35581 3.29744 2.72107 4.69283 3.75251 3.90230
4.13293 1.69959 3.64082 4.41746 2.81491 4.13998 3.01570 1.54340
2.88721 2.45951 2.96416 5.78630 4.43759 25 r - - -
2.68618 4.42225 2.77519 2.73123 3.46354 2.40513 3.72494
3.29354 2.67741 2.69355 4.24690 2.90347 2.73739 3.18146 2.89801
2.37887 2.77519 2.98518 4.58477 3.61503
0.03971 4.97704 3.44081 0.61958 0.77255 1.03106 0.44103
19 1.81285 5.32531 3.11812 1.82242 4.65373 2.97551 3.82794
4.12742 2.44279 3.48933 4.37230 2.98695 3.02025 2.75055 3.06032
2.44701 2.88328 3.52974 5.77006 4.36995 26 a - - -
2.68618 4.42225 2.77519 2.73123 3.46354 2.40513 3.72494
3.29354 2.67741 2.69355 4.24690 2.90347 2.73739 3.18146 2.89801
2.37887 2.77519 2.98518 4.58477 3.61503
0.01060 4.94793 5.67027 0.61958 0.77255 1.07588 0.41703
20 2.25412 5.25529 3.15313 2.59710 4.55700 3.09978 3.64961
3.28739 2.31875 3.54191 4.30835 2.83753 4.04183 2.63122 2.87420
2.29252 1.80694 3.62257 5.71535 4.33313 27 t - - -
2.68619 4.42226 2.77520 2.73124 3.46354 2.40513 3.72495
3.29355 2.67741 2.69355 4.24690 2.90347 2.73740 3.18129 2.89801
2.37887 2.77520 2.98519 4.58477 3.61504
0.03036 3.63212 5.67027 0.45512 1.00614 0.28279 1.40110
21 2.36133 5.28416 2.77663 2.52972 4.55791 2.98634 3.96872
3.68745 2.73995 3.55850 3.94070 3.27502 3.79813 2.57752 2.90026
2.76123 2.50810 1.66045 5.76216 4.39892 29 v - - -
2.68618 4.42225 2.77519 2.73123 3.46354 2.40513 3.72494
3.29354 2.67741 2.69355 4.24690 2.90347 2.73739 3.18146 2.89801
2.37887 2.77519 2.98518 4.58477 3.61503
0.00828 5.19335 5.91569 0.61958 0.77255 0.54370 0.86892
22 1.74192 4.57013 4.16442 3.58852 3.68581 3.71292 4.32311
2.54782 2.99202 2.05514 3.18290 3.59922 3.68771 3.77465 3.40771
2.75587 2.67916 1.92930 5.17042 3.96226 30 a - - -
2.68618 4.42225 2.77519 2.73123 3.46354 2.40513 3.72494
3.29354 2.67741 2.69355 4.24690 2.90347 2.73739 3.18146 2.89801
2.37887 2.77519 2.98518 4.58477 3.61503
0.00828 5.19335 5.91569 0.61958 0.77255 0.54370 0.86892
23 2.83775 5.48336 3.30483 2.73807 4.82824 3.53536 3.96354
4.29263 1.45540 3.77718 4.53124 3.13386 4.18863 2.72627 1.61179
2.75907 2.79532 3.28533 5.90768 4.52724 31 k - - -
2.68618 4.42225 2.77519 2.73123 3.46354 2.40513 3.72494
3.29354 2.67741 2.69355 4.24690 2.90347 2.73739 3.18146 2.89801
2.37887 2.77519 2.98518 4.58477 3.61503
0.00828 5.19335 5.91569 0.61958 0.77255 0.45117 1.01303
24 2.89043 5.47375 2.40229 2.66800 4.81470 2.28746 3.93643
4.29756 2.16260 3.77766 4.51671 2.87477 4.14141 2.90186 3.03178
1.83358 2.18663 3.59904 5.90985 4.50136 32 s - - -

```
          2.68618  4.42225  2.77519  2.73123  3.46354  2.40513  3.72494
3.29354  2.67741  2.69355  4.24690  2.90347  2.73739  3.18146  2.89801
2.37887  2.77519  2.98518  4.58477  3.61503
          0.03502  5.21063  3.54209  0.61958  0.77255  0.48576  0.95510
     25   2.55374  5.46896  2.96699  1.95258  4.81490  3.33767  3.71371
4.02785  2.04510  3.77460  4.50942  2.49169  2.77971  2.12622  2.78327
2.69555  3.05759  3.86007  5.90109  4.48960     33 e - - -
          2.68618  4.42225  2.77519  2.73123  3.46354  2.40513  3.72494
3.29354  2.67741  2.69355  4.24690  2.90347  2.73739  3.18146  2.89801
2.37887  2.77519  2.98518  4.58477  3.61503
          0.00836  5.18398  5.90632  0.61958  0.77255  0.43464  1.04270
     26   1.89848  5.46699  3.23087  2.60364  4.80626  3.24615  3.93962
4.28563  2.04003  3.76940  4.51137  3.12166  4.14613  2.27793  2.90819
1.71073  2.89448  3.85499  5.90402  4.49980     34 s - - -
          2.68618  4.42225  2.77519  2.73123  3.46354  2.40513  3.72494
3.29354  2.67741  2.69355  4.24690  2.90347  2.73739  3.18146  2.89801
2.37887  2.77519  2.98518  4.58477  3.61503
          0.00814  5.21063  5.93298  0.61958  0.77255  0.48576  0.95510
     27   1.31843  5.39993  2.60811  2.19158  4.71169  3.58324  3.95105
3.94940  2.70105  3.33376  4.45009  3.10669  4.15225  2.63615  3.18775
2.75147  2.94869  3.02094  5.85360  4.46360     35 a - - -
          2.68618  4.42225  2.77519  2.73123  3.46354  2.40513  3.72494
3.29354  2.67741  2.69355  4.24690  2.90347  2.73739  3.18146  2.89801
2.37887  2.77519  2.98518  4.58477  3.61503
          0.00814  5.21063  5.93298  0.61958  0.77255  0.48576  0.95510
     28   2.62294  4.45329  4.98790  4.37991  3.54572  4.25263  4.58920
1.77996  4.18025  1.31020  3.40618  4.39590  4.61169  4.32316  3.58599
3.56399  3.12770  1.67760  5.09438  3.57593     36 l - - -
          2.68618  4.42225  2.77519  2.73123  3.46354  2.40513  3.72494
3.29354  2.67741  2.69355  4.24690  2.90347  2.73739  3.18146  2.89801
2.37887  2.77519  2.98518  4.58477  3.61503
          0.00814  5.21063  5.93298  0.61958  0.77255  0.48576  0.95510
     29   2.87492  5.63968  2.96465  2.64841  4.95397  3.84813  3.65021
4.42230  2.92316  3.80342  4.71504  0.78778  4.30882  3.25802  3.18354
3.15941  3.07332  4.01948  6.09696  4.69271     37 n - - -
          2.68618  4.42225  2.77519  2.73123  3.46354  2.40513  3.72494
3.29354  2.67741  2.69355  4.24690  2.90347  2.73739  3.18146  2.89801
2.37887  2.77519  2.98518  4.58477  3.61503
          0.00814  5.21063  5.93298  0.61958  0.77255  0.48576  0.95510
     30   1.15595  5.45129  3.00902  2.36243  4.78186  3.64929  3.77796
3.90529  2.54758  3.74993  4.49633  3.22607  4.14736  2.59898  2.71048
2.64079  3.12771  3.83423  5.89227  4.49083     38 a - - -
          2.68618  4.42225  2.77519  2.73123  3.46354  2.40513  3.72494
3.29354  2.67741  2.69355  4.24690  2.90347  2.73739  3.18146  2.89801
2.37887  2.77519  2.98518  4.58477  3.61503
          0.00814  5.21063  5.93298  0.61958  0.77255  0.48576  0.95510
     31   0.30172  4.95971  4.89235  4.72351  5.29511  3.80591  5.53875
4.48694  4.70809  4.39719  5.27304  3.75403  4.63123  4.93128  4.85633
3.29783  3.62202  2.97033  6.72179  5.57222     39 A - - -
          2.68618  4.42225  2.77519  2.73123  3.46354  2.40513  3.72494
3.29354  2.67741  2.69355  4.24690  2.90347  2.73739  3.18146  2.89801
2.37887  2.77519  2.98518  4.58477  3.61503
          0.00814  5.21063  5.93298  0.61958  0.77255  0.48576  0.95510
     32   2.50414  5.18698  3.40772  2.84546  4.13708  3.82828  4.02417
3.47220  2.50027  2.77432  3.31760  3.36116  4.21651  1.89720  1.52186
2.97813  3.22768  3.51916  5.68608  4.35753     40 r - - -
```

```
          2.68618  4.42225  2.77519  2.73123  3.46354  2.40513  3.72494
3.29354  2.67741  2.69355  4.24690  2.90347  2.73739  3.18146  2.89801
2.37887  2.77519  2.98518  4.58477  3.61503
          0.00814  5.21063  5.93298  0.61958  0.77255  0.48576  0.95510
     33   4.15818  6.14387  4.89910  3.87977  5.76366  4.66729  4.36150
4.99281  2.26892  3.18578  5.21759  4.16413  4.96106  2.70134  0.45389
4.13068  4.23042  4.70733  6.25216  5.21282     41 R - - -
          2.68618  4.42225  2.77519  2.73123  3.46354  2.40513  3.72494
3.29354  2.67741  2.69355  4.24690  2.90347  2.73739  3.18146  2.89801
2.37887  2.77519  2.98518  4.58477  3.61503
          0.00814  5.21063  5.93298  0.61958  0.77255  0.48576  0.95510
     34   2.03045  5.38899  3.25873  2.57568  4.69734  3.76017  3.80372
4.15796  2.53059  3.28116  4.00939  3.16534  4.15266  2.32119  2.76317
1.60999  2.59226  3.33700  5.84420  4.45694     42 s - - -
          2.68618  4.42225  2.77519  2.73123  3.46354  2.40513  3.72494
3.29354  2.67741  2.69355  4.24690  2.90347  2.73739  3.18146  2.89801
2.37887  2.77519  2.98518  4.58477  3.61503
          0.00814  5.21063  5.93298  0.61958  0.77255  0.48576  0.95510
     35   4.18575  6.13375  3.89610  2.88433  6.23716  0.19911  5.54778
5.94444  4.68364  5.44550  6.39979  4.42651  5.18494  4.83972  5.08743
4.24331  4.65531  5.33029  7.19140  6.14476     43 G - - -
          2.68618  4.42225  2.77519  2.73123  3.46354  2.40513  3.72494
3.29354  2.67741  2.69355  4.24690  2.90347  2.73739  3.18146  2.89801
2.37887  2.77519  2.98518  4.58477  3.61503
          0.00814  5.21063  5.93298  0.61958  0.77255  0.48576  0.95510
     36   1.59276  5.15827  3.25533  2.46344  4.39074  2.68605  4.01890
3.23852  2.82503  2.68525  3.53175  2.82810  4.20307  2.85262  3.28645
2.60726  2.80822  2.93940  5.66497  4.33443     44 a - - -
          2.68618  4.42225  2.77519  2.73123  3.46354  2.40513  3.72494
3.29354  2.67741  2.69355  4.24690  2.90347  2.73739  3.18146  2.89801
2.37887  2.77519  2.98518  4.58477  3.61503
          0.02957  5.21063  3.74330  0.61958  0.77255  0.48576  0.95510
     37   2.17967  5.30682  2.78162  2.18299  4.58881  3.50463  3.96012
2.77812  2.72599  3.10825  4.36470  2.83249  3.11812  2.51103  3.20531
2.84917  3.04373  2.14243  5.77926  4.40991     45 v - - -
          2.68618  4.42225  2.77519  2.73123  3.46354  2.40513  3.72494
3.29354  2.67741  2.69355  4.24690  2.90347  2.73739  3.18146  2.89801
2.37887  2.77519  2.98518  4.58477  3.61503
          0.05063  5.18938  3.12830  0.61958  0.77255  0.44400  1.02573
     38   3.88253  5.07520  6.00859  5.54017  4.48727  5.51360  6.24605
1.01536  5.48415  2.26650  4.22347  5.67798  5.73182  5.73274  5.63558
4.94374  3.26333  0.91689  6.56594  5.34412     46 v - - -
          2.68621  4.42229  2.77523  2.73127  3.46357  2.40516  3.72498
3.29358  2.67744  2.69331  4.24693  2.90350  2.73714  3.18150  2.89804
2.37890  2.77523  2.98522  4.58480  3.61507
          0.06991  3.13429  3.73020  0.66027  0.72714  0.41176  1.08613
     39   2.34201  5.46412  2.29767  1.86050  4.80539  2.62748  3.92445
3.92652  2.66160  3.65653  4.50622  3.10411  4.12980  2.57164  3.15410
2.01574  2.71976  3.73705  5.89933  4.49033     49 e - - -
          2.68618  4.42225  2.77519  2.73123  3.46354  2.40513  3.72494
3.29354  2.67741  2.69355  4.24690  2.90347  2.73739  3.18146  2.89801
2.37887  2.77519  2.98518  4.58477  3.61503
          0.00831  5.19005  5.91239  0.61958  0.77255  0.44518  1.02361
     40   2.90525  4.78345  4.32747  3.82904  4.26434  3.50400  4.67846
3.39504  3.77321  3.34635  4.23377  3.60164  4.47750  4.04896  4.06829
2.61329  0.77873  2.09969  5.71319  4.50616     50 t - - -
```

```
          2.68618  4.42225  2.77519  2.73123  3.46354  2.40513  3.72494
3.29354  2.67741  2.69355  4.24690  2.90347  2.73739  3.18146  2.89801
2.37887  2.77519  2.98518  4.58477  3.61503
          0.00814  5.21063  5.93298  0.61958  0.77255  0.48576  0.95510
     41   2.97037  5.43727  2.17031  1.49220  4.76427  3.75040  3.93992
3.40200  2.68225  3.73483  3.81881  3.06306  4.14379  2.55389  3.11586
2.42884  2.81210  2.82597  5.88091  4.48099     51 e - - -
          2.68618  4.42225  2.77519  2.73123  3.46354  2.40513  3.72494
3.29354  2.67741  2.69355  4.24690  2.90347  2.73739  3.18146  2.89801
2.37887  2.77519  2.98518  4.58477  3.61503
          0.00814  5.21063  5.93298  0.61958  0.77255  0.48576  0.95510
     42   2.86773  5.46332  3.39304  2.81607  4.39075  3.85524  4.00461
3.90593  0.97786  3.65537  4.51728  3.34138  4.24065  2.85897  2.19038
2.95083  2.96218  3.72050  5.89037  4.53669     52 k - - -
          2.68618  4.42225  2.77519  2.73123  3.46354  2.40513  3.72494
3.29354  2.67741  2.69355  4.24690  2.90347  2.73739  3.18146  2.89801
2.37887  2.77519  2.98518  4.58477  3.61503
          0.00814  5.21063  5.93298  0.61958  0.77255  0.48576  0.95510
     43   4.34445  6.26651  5.06177  4.04845  5.99656  4.75936  4.46875
5.21741  0.32239  4.48385  5.41034  4.30051  5.07432  3.59866  2.23673
4.31098  4.40700  4.92869  6.36163  5.36776     53 K - - -
          2.68618  4.42225  2.77519  2.73123  3.46354  2.40513  3.72494
3.29354  2.67741  2.69355  4.24690  2.90347  2.73739  3.18146  2.89801
2.37887  2.77519  2.98518  4.58477  3.61503
          0.24834  5.21063  1.53970  0.61958  0.77255  0.48576  0.95510
     44   2.75291  4.39400  4.25201  3.08759  1.85548  3.36082  4.26917
2.74752  3.56445  2.59091  3.26060  3.91520  4.34476  3.13992  3.77691
2.92252  2.30263  2.27600  3.62813  2.64338     54 f - - -
          2.68618  4.42225  2.77519  2.73123  3.46354  2.40513  3.72494
3.29354  2.67741  2.69355  4.24690  2.90347  2.73739  3.18146  2.89801
2.37887  2.77519  2.98518  4.58477  3.61503
          0.02830  4.97263  3.86417  0.61958  0.77255  0.25037  1.50740
     45   2.04249  5.04122  2.97526  2.90844  2.89257  2.65667  3.38830
3.64166  2.89543  3.00670  3.63068  2.70519  4.22640  3.23690  3.14509
2.73565  2.81972  3.18143  5.56932  2.22756     55 a - - -
          2.68618  4.42225  2.77519  2.73123  3.46354  2.40513  3.72494
3.29354  2.67741  2.69355  4.24690  2.90347  2.73739  3.18146  2.89801
2.37887  2.77519  2.98518  4.58477  3.61503
          0.02196  5.19665  4.12388  0.61958  0.77255  0.45734  1.00231
     46   1.33791  4.84947  3.73951  3.18324  3.68259  1.72293  4.21322
3.27197  3.15384  3.03934  4.00680  3.61892  4.31565  3.19697  3.54941
2.68985  2.89813  3.04492  5.47310  4.21533     56 a - - -
          2.68618  4.42225  2.77519  2.73123  3.46354  2.40513  3.72494
3.29354  2.67741  2.69355  4.24690  2.90347  2.73739  3.18146  2.89801
2.37887  2.77519  2.98518  4.58477  3.61503
          0.04198  5.19724  3.33590  0.61958  0.77255  0.53104  0.88671
     47   2.47598  5.26990  3.33987  2.79613  4.64538  1.40100  4.02441
4.09022  2.79159  3.64447  4.42453  3.16605  4.18121  2.74779  2.88504
2.38996  2.18990  3.53907  5.83669  4.47492     57 g - - -
          2.68618  4.42225  2.77519  2.73123  3.46354  2.40513  3.72494
3.29354  2.67741  2.69355  4.24690  2.90347  2.73739  3.18146  2.89801
2.37887  2.77519  2.98518  4.58477  3.61503
          0.01319  5.16379  4.90816  0.61958  0.77255  0.51932  0.90369
     48   2.29092  5.30557  3.27904  2.63246  3.96576  2.17764  3.63804
3.72487  2.49291  3.28292  4.36316  3.11924  4.15214  2.67369  3.20009
2.04073  2.10660  3.66238  5.77736  3.98141     58 s - - -
```

```
         2.68618  4.42225  2.77519  2.73123  3.46354  2.40513  3.72494
3.29354  2.67741  2.69355  4.24690  2.90347  2.73739  3.18146  2.89801
2.37887  2.77519  2.98518  4.58477  3.61503
         0.00838  5.18144  5.90379  0.61958  0.77255  0.43040  1.05054
     49  3.26772  5.75370  3.24985  3.25098  5.74705  3.44433  4.85476
5.27888  3.46850  4.81430  5.67609  0.37135  4.69176  4.06037  4.24695
3.59169  3.99978  4.69030  6.92798  5.53462     59 N - - -
         2.68618  4.42225  2.77519  2.73123  3.46354  2.40513  3.72494
3.29354  2.67741  2.69355  4.24690  2.90347  2.73739  3.18146  2.89801
2.37887  2.77519  2.98518  4.58477  3.61503
         0.00814  5.21063  5.93298  0.61958  0.77255  0.48576  0.95510
     50  2.28809  5.43634  3.24396  2.68713  4.76331  3.03139  3.70388
4.10712  1.33013  3.62713  4.48267  3.23118  4.14834  2.79952  2.72712
2.44175  2.61603  3.46918  5.87969  4.48257     60 k - - -
         2.68618  4.42225  2.77519  2.73123  3.46354  2.40513  3.72494
3.29354  2.67741  2.69355  4.24690  2.90347  2.73739  3.18146  2.89801
2.37887  2.77519  2.98518  4.58477  3.61503
         0.33917  5.21063  1.26522  0.61958  0.77255  0.48576  0.95510
     51  1.87705  5.18264  3.15072  2.59456  4.47177  2.63112  3.82855
3.31418  2.21041  3.46585  4.24364  3.12962  3.62372  2.65002  2.97894
2.09655  2.76016  3.45069  5.65663  4.28569     61 a - - -
         2.68618  4.42225  2.77519  2.73123  3.46354  2.40513  3.72494
3.29354  2.67741  2.69355  4.24690  2.90347  2.73739  3.18146  2.89801
2.37887  2.77519  2.98518  4.58477  3.61503
         0.04613  4.88278  3.28330  0.61958  0.77255  1.16532  0.37371
     52  2.36406  5.24266  3.04899  2.54660  4.55467  2.72077  2.32402
4.01686  2.33186  3.37320  4.29395  3.08821  3.86442  2.77555  2.95138
2.06792  2.44933  3.61579  5.69732  4.30858     62 s - - -
         2.68618  4.42225  2.77519  2.73123  3.46354  2.40513  3.72494
3.29354  2.67741  2.69355  4.24690  2.90347  2.73739  3.18146  2.89801
2.37887  2.77519  2.98518  4.58477  3.61503
         0.05070  4.84837  3.17983  0.61958  0.77255  0.92534  0.50483
     53  2.24787  5.33062  2.94242  2.53134  4.67018  2.97210  3.40010
4.15207  1.75374  3.63343  4.37289  2.69888  3.78770  2.42956  2.83211
2.35353  3.06479  3.19656  5.76653  4.35920     63 k - - -
         2.68618  4.42225  2.77519  2.73123  3.46354  2.40513  3.72494
3.29354  2.67741  2.69355  4.24690  2.90347  2.73739  3.18146  2.89801
2.37887  2.77519  2.98518  4.58477  3.61503
         0.01121  4.89235  5.61469  0.61958  0.77255  1.02202  0.44608
     54  2.02499  5.20343  3.09499  2.55945  4.50012  2.18429  3.84868
3.94733  2.52285  3.49374  4.06312  2.89160  2.63584  2.96840  3.09269
2.28686  2.70928  3.24738  5.68254  4.31007     64 a - - -
         2.68618  4.42225  2.77519  2.73123  3.46354  2.40513  3.72494
3.29354  2.67741  2.69355  4.24690  2.90347  2.73739  3.18146  2.89801
2.37887  2.77519  2.98518  4.58477  3.61503
         0.23705  4.92579  1.59067  0.61958  0.77255  0.32973  1.26983
     55  2.38835  5.27588  3.17268  2.61631  4.12398  2.96724  3.40060
4.03326  2.43274  3.06415  4.32836  2.89715  2.85032  2.11452  3.10019
2.48995  2.23558  3.49304  5.73577  3.93480     65 q - - -
         2.68602  4.42262  2.77557  2.73161  3.46391  2.40489  3.72245
3.29385  2.67772  2.69379  4.24597  2.90379  2.73772  3.18159  2.89810
2.37867  2.77521  2.98411  4.58514  3.61540
         0.78099  0.61846  5.72176  0.54169  0.87171  0.30057  1.34859
     56  2.32419  5.42574  2.15120  1.76767  4.75113  3.30400  3.93379
3.55923  2.67666  3.26291  3.81585  2.84768  3.08236  3.03681  3.16597
2.79472  2.40758  3.54883  5.87045  4.47168     71 e - - -
```

```
          2.68618  4.42225  2.77519  2.73123  3.46354  2.40513  3.72494
3.29354  2.67741  2.69355  4.24690  2.90347  2.73739  3.18146  2.89801
2.37887  2.77519  2.98518  4.58477  3.61503
          0.00825  5.19707  5.91941  0.61958  0.77255  0.53159  0.88594
     57   2.59456  5.34264  2.88266  2.71433  4.63618  1.79900  2.34833
3.48743  2.71357  3.62570  3.99927  3.05563  3.50876  2.86152  3.19590
2.51724  2.60759  3.38261  5.80754  3.94858     72 g - - -
          2.68618  4.42225  2.77519  2.73123  3.46354  2.40513  3.72494
3.29354  2.67741  2.69355  4.24690  2.90347  2.73739  3.18146  2.89801
2.37887  2.77519  2.98518  4.58477  3.61503
          0.00825  5.19707  5.91941  0.61958  0.77255  0.45814  1.00092
     58   2.39557  5.33719  3.28581  2.61174  4.62632  3.56574  3.96531
3.60857  2.05907  2.96097  3.99240  3.26548  3.61415  1.41171  3.04802
2.85437  3.20467  3.17111  5.80468  4.43091     73 q - - -
          2.68618  4.42225  2.77519  2.73123  3.46354  2.40513  3.72494
3.29354  2.67741  2.69355  4.24690  2.90347  2.73739  3.18146  2.89801
2.37887  2.77519  2.98518  4.58477  3.61503
          0.00814  5.21063  5.93298  0.61958  0.77255  0.48576  0.95510
     59   2.98630  5.48034  3.05908  2.68137  4.19685  3.75760  2.89203
4.30230  2.18247  3.78184  4.52308  1.68559  3.72693  3.04103  1.73981
2.67712  3.21665  3.86958  5.91254  4.50874     74 n - - -
          2.68618  4.42225  2.77519  2.73123  3.46354  2.40513  3.72494
3.29354  2.67741  2.69355  4.24690  2.90347  2.73739  3.18146  2.89801
2.37887  2.77519  2.98518  4.58477  3.61503
          0.00814  5.21063  5.93298  0.61958  0.77255  0.48576  0.95510
     60   2.05192  4.49247  4.02629  3.86955  3.59654  4.11030  4.12666
1.97195  3.42065  1.68058  2.92064  3.53800  4.48010  3.97534  3.93870
3.39500  2.00022  2.57684  5.10339  3.91070     75 l - - -
          2.68618  4.42225  2.77519  2.73123  3.46354  2.40513  3.72494
3.29354  2.67741  2.69355  4.24690  2.90347  2.73739  3.18146  2.89801
2.37887  2.77519  2.98518  4.58477  3.61503
          0.00814  5.21063  5.93298  0.61958  0.77255  0.48576  0.95510
     61   1.40426  5.28578  3.36549  2.80812  4.58947  3.30339  4.01701
4.02255  2.19522  3.26876  4.37998  3.33095  4.20421  3.14752  2.68759
2.83505  1.90461  3.66268  5.79311  4.44164     76 a - - -
          2.68618  4.42225  2.77519  2.73123  3.46354  2.40513  3.72494
3.29354  2.67741  2.69355  4.24690  2.90347  2.73739  3.18146  2.89801
2.37887  2.77519  2.98518  4.58477  3.61503
          0.00814  5.21063  5.93298  0.61958  0.77255  0.48576  0.95510
     62   3.72780  5.87544  4.26213  3.48395  5.36244  4.37505  4.25808
4.68262  0.57254  4.09806  3.44490  3.87966  4.71159  3.38243  2.03341
3.42751  3.87939  3.99803  6.12926  4.97302     77 K - - -
          2.68618  4.42225  2.77519  2.73123  3.46354  2.40513  3.72494
3.29354  2.67741  2.69355  4.24690  2.90347  2.73739  3.18146  2.89801
2.37887  2.77519  2.98518  4.58477  3.61503
          0.00814  5.21063  5.93298  0.61958  0.77255  0.48576  0.95510
     63   4.20638  5.38028  6.28621  5.77399  4.05471  5.78588  6.30803
1.74070  5.69245  0.68844  3.77579  5.96314  5.84932  5.67050  5.70949
5.22101  4.43859  1.54629  6.31647  5.30398     78 L - - -
          2.68618  4.42225  2.77519  2.73123  3.46354  2.40513  3.72494
3.29354  2.67741  2.69355  4.24690  2.90347  2.73739  3.18146  2.89801
2.37887  2.77519  2.98518  4.58477  3.61503
          0.00814  5.21063  5.93298  0.61958  0.77255  0.48576  0.95510
     64   3.71065  6.84164  0.42218  2.00417  6.11036  4.02068  4.72661
5.73011  3.97425  5.15040  6.09278  3.46219  4.73939  3.92020  4.74758
3.38492  4.35710  5.22375  7.30148  5.62833     79 D - - -
```

2.68618 4.42225 2.77519 2.73123 3.46354 2.40513 3.72494
3.29354 2.67741 2.69355 4.24690 2.90347 2.73739 3.18146 2.89801
2.37887 2.77519 2.98518 4.58477 3.61503
0.00814 5.21063 5.93298 0.61958 0.77255 0.48576 0.95510
65 2.75918 5.58371 2.69565 1.97302 4.92612 3.78541 4.00649
4.41321 2.75299 3.88526 4.63010 2.70753 4.20503 2.70046 1.20770
2.87840 3.30162 3.97393 6.01221 4.59671 80 r - - -
2.68618 4.42225 2.77519 2.73123 3.46354 2.40513 3.72494
3.29354 2.67741 2.69355 4.24690 2.90347 2.73739 3.18146 2.89801
2.37887 2.77519 2.98518 4.58477 3.61503
0.00814 5.21063 5.93298 0.61958 0.77255 0.48576 0.95510
66 2.58409 5.52180 2.07701 1.25651 4.85810 3.65385 3.98752
4.33991 2.74392 3.82641 4.57353 3.23982 4.18222 3.09133 3.24121
2.14749 3.01352 3.68308 5.96521 4.11544 81 e - - -
2.68618 4.42225 2.77519 2.73123 3.46354 2.40513 3.72494
3.29354 2.67741 2.69355 4.24690 2.90347 2.73739 3.18146 2.89801
2.37887 2.77519 2.98518 4.58477 3.61503
0.00814 5.21063 5.93298 0.61958 0.77255 0.48576 0.95510
67 3.05712 5.64464 1.80161 2.54615 4.96667 3.81269 3.86322
4.45021 2.88267 3.59331 4.70471 1.88484 4.26407 3.20177 3.39207
2.99948 1.46026 4.02552 6.08993 4.66795 82 t - - -
2.68618 4.42225 2.77519 2.73123 3.46354 2.40513 3.72494
3.29354 2.67741 2.69355 4.24690 2.90347 2.73739 3.18146 2.89801
2.37887 2.77519 2.98518 4.58477 3.61503
0.00814 5.21063 5.93298 0.61958 0.77255 0.48576 0.95510
68 3.15732 6.53839 1.43709 0.75765 5.83436 2.75987 4.58923
5.40709 3.69405 4.84651 5.71102 3.42746 4.64327 3.75931 4.36640
3.66017 4.12201 4.92420 6.99100 5.40356 83 e - - -
2.68618 4.42225 2.77519 2.73123 3.46354 2.40513 3.72494
3.29354 2.67741 2.69355 4.24690 2.90347 2.73739 3.18146 2.89801
2.37887 2.77519 2.98518 4.58477 3.61503
0.00814 5.21063 5.93298 0.61958 0.77255 0.48576 0.95510
69 2.97800 5.27726 2.87132 1.90803 4.54437 3.78623 3.75440
2.25302 2.70476 3.54864 4.33961 2.72654 2.67635 3.11617 3.23806
2.88362 2.78099 2.14255 5.75965 4.40092 84 e - - -
2.68618 4.42225 2.77519 2.73123 3.46354 2.40513 3.72494
3.29354 2.67741 2.69355 4.24690 2.90347 2.73739 3.18146 2.89801
2.37887 2.77519 2.98518 4.58477 3.61503
0.00814 5.21063 5.93298 0.61958 0.77255 0.48576 0.95510
70 1.82454 4.40511 4.91667 4.30606 2.35509 3.69996 4.51283
2.34004 3.39657 1.63950 3.16935 4.32267 4.54669 4.24969 4.13789
3.49108 3.25236 1.88970 5.02655 3.56615 85 l - - -
2.68618 4.42225 2.77519 2.73123 3.46354 2.40513 3.72494
3.29354 2.67741 2.69355 4.24690 2.90347 2.73739 3.18146 2.89801
2.37887 2.77519 2.98518 4.58477 3.61503
0.00814 5.21063 5.93298 0.61958 0.77255 0.48576 0.95510
71 2.35011 5.49054 3.26388 2.62322 4.83503 3.63129 2.52564
4.30986 1.62096 3.78900 4.53429 3.24831 2.00559 2.72828 2.78597
2.91876 3.16590 3.88152 5.91765 4.52228 86 k - - -
2.68618 4.42225 2.77519 2.73123 3.46354 2.40513 3.72494
3.29354 2.67741 2.69355 4.24690 2.90347 2.73739 3.18146 2.89801
2.37887 2.77519 2.98518 4.58477 3.61503
0.01987 5.21063 4.25345 0.61958 0.77255 0.48576 0.95510
72 3.02270 4.54473 4.33155 3.75138 3.65281 4.09932 1.91262
2.41868 3.64990 2.34652 3.51951 4.01883 1.68186 3.89834 3.88004
3.37696 2.82430 2.20514 5.15723 3.95757 87 p - - -

```
          2.68618  4.42225  2.77519  2.73123  3.46354  2.40513  3.72494
3.29354  2.67741  2.69355  4.24690  2.90347  2.73739  3.18146  2.89801
2.37887  2.77519  2.98518  4.58477  3.61503
          0.00824  5.19900  5.92135  0.61958  0.77255  0.52523  0.89507
     73   2.74372  5.50296  2.41908  1.78091  4.84758  3.60399  3.94323
4.33527  1.67338  3.80801  4.54475  2.80860  2.56695  2.73316  3.17920
2.77483  3.07513  3.89355  5.93487  4.52078     88 k - - -
          2.68618  4.42225  2.77519  2.73123  3.46354  2.40513  3.72494
3.29354  2.67741  2.69355  4.24690  2.90347  2.73739  3.18146  2.89801
2.37887  2.77519  2.98518  4.58477  3.61503
          0.03643  5.19900  3.49824  0.61958  0.77255  0.52523  0.89507
     74   3.36874  5.83716  4.04207  3.32643  5.33302  4.26034  4.19373
4.67964  1.18931  4.08176  4.91399  3.75464  4.61048  3.19367  1.50023
3.57611  1.66634  4.32304  6.10666  4.91553     89 k - - -
          2.68618  4.42225  2.77519  2.73123  3.46354  2.40513  3.72494
3.29354  2.67741  2.69355  4.24690  2.90347  2.73739  3.18146  2.89801
2.37887  2.77519  2.98518  4.58477  3.61503
          0.07305  5.17104  2.73697  0.61958  0.77255  0.55685  0.85099
     75   3.95587  5.11983  6.11452  5.67878  4.52513  5.68602  6.55744
1.46415  5.65923  2.50671  4.21930  5.85607  5.85775  5.92808  5.84512
5.15632  4.21758  0.53486  6.76759  5.54009     90 V - - -
          2.68618  4.42225  2.77519  2.73123  3.46354  2.40513  3.72494
3.29354  2.67741  2.69355  4.24690  2.90347  2.73739  3.18146  2.89801
2.37887  2.77519  2.98518  4.58477  3.61503
          0.00602  5.11527        *  0.61958  0.77255  0.00000        *
//
HMMER3/f [3.3 | Nov 2019]
NAME  Med26
ACC   PF08711.12
DESC  TFIIS helical bundle-like domain
LENG  53
ALPH  amino
RF    no
MM    no
CONS  yes
CS    yes
MAP   yes
DATE  Sat Apr 18 04:04:18 2020
NSEQ  430
EFFN  25.244522
CKSUM 1880163765
GA    20.60 20.60
TC    20.60 20.60
NC    20.50 20.50
STATS LOCAL MSV       -7.7255  0.71910
STATS LOCAL VITERBI   -8.5700  0.71910
STATS LOCAL FORWARD   -4.1512  0.71910
HMM          A        C        D        E        F        G        H
I        K        L        M        N        P        Q        R        S
T        V        W        Y
            m->m     m->i     m->d     i->m     i->i     d->m     d->d
  COMPO   2.63290  4.53319  3.14513  2.72748  4.06400  3.11324  3.83864
2.63694  2.26942  2.15205  3.97901  3.12662  3.55108  3.23093  2.70000
2.71706  2.74170  2.64928  3.86878  4.23528
          2.68618  4.42225  2.77519  2.73123  3.46354  2.40513  3.72494
3.29354  2.67741  2.69355  4.24690  2.90347  2.73739  3.18146  2.89801
2.37887  2.77519  2.98518  4.58477  3.61503
          0.00158  6.84388  7.56622  0.61958  0.77255  0.00000        *
```

```
      1   2.67043  5.53692  2.48360  2.17391  5.47554  2.03419  2.66454
3.63640  1.61016  3.50386  4.46177  3.40458  4.21818  2.84097  3.42767
2.93380  2.99361  4.56267  6.98564  5.57394      1 k - - H
          2.68618  4.42225  2.77519  2.73123  3.46354  2.40513  3.72494
3.29354  2.67741  2.69355  4.24690  2.90347  2.73739  3.18146  2.89801
2.37887  2.77519  2.98518  4.58477  3.61503
          0.00158  6.84388  7.56622  0.61958  0.77255  0.48576  0.95510
      2   2.44120  2.89830  5.68808  4.54339  4.43262  3.94680  3.89061
1.14822  5.33955  1.70284  2.80034  5.05353  5.35243  4.79965  5.34561
3.60343  3.33078  2.12884  6.19809  3.95875      2 i - - H
          2.68618  4.42225  2.77519  2.73123  3.46354  2.40513  3.72494
3.29354  2.67741  2.69355  4.24690  2.90347  2.73739  3.18146  2.89801
2.37887  2.77519  2.98518  4.58477  3.61503
          0.00158  6.84388  7.56622  0.61958  0.77255  0.48576  0.95510
      3   6.36755  7.41788  8.38336  7.80164  4.13515  8.01967  8.18789
1.62834  7.74021  0.35581  3.28077  8.24722  7.60691  7.22081  7.49835
7.55630  6.52513  3.21731  7.68069  6.91973      3 L - - H
          2.68618  4.42225  2.77519  2.73123  3.46354  2.40513  3.72494
3.29354  2.67741  2.69355  4.24690  2.90347  2.73739  3.18146  2.89801
2.37887  2.77519  2.98518  4.58477  3.61503
          0.00158  6.84388  7.56622  0.61958  0.77255  0.48576  0.95510
      4   1.76073  4.07408  2.86148  2.38543  4.46788  3.54916  3.40969
4.51948  2.11054  3.14391  4.56448  2.37795  4.76572  2.85500  2.46500
2.54809  3.05114  4.59071  5.67558  3.91006      4 a - - H
          2.68618  4.42225  2.77519  2.73123  3.46354  2.40513  3.72494
3.29354  2.67741  2.69355  4.24690  2.90347  2.73739  3.18146  2.89801
2.37887  2.77519  2.98518  4.58477  3.61503
          0.00158  6.84388  7.56622  0.61958  0.77255  0.48576  0.95510
      5   2.70164  4.79518  2.54327  2.30890  4.03484  4.25917  4.37790
3.37498  2.32367  2.84304  3.99949  2.79718  4.85590  1.89269  2.45231
3.28135  2.42684  3.28992  6.97851  3.73099      5 q - - H
          2.68629  4.42236  2.77531  2.73134  3.46365  2.40524  3.72506
3.29365  2.67699  2.69366  4.24701  2.90358  2.73710  3.18158  2.89764
2.37898  2.77531  2.98530  4.58488  3.61418
          0.02582  3.69003  7.56622  0.60918  0.78482  0.48576  0.95510
      6   5.40992  7.16167  7.98453  7.38397  2.30691  7.42037  7.60433
2.76034  7.25094  0.29394  3.24465  7.61111  7.30506  6.93841  7.09212
6.83716  5.33791  4.85343  7.43146  3.58991      8 L - - S
          2.68618  4.42225  2.77519  2.73123  3.46354  2.40513  3.72494
3.29354  2.67741  2.69355  4.24690  2.90347  2.73739  3.18146  2.89801
2.37887  2.77519  2.98518  4.58477  3.61503
          0.00158  6.84388  7.56622  0.61958  0.77255  0.48576  0.95510
      7   3.18028  6.55372  2.40898  1.39095  4.66696  4.02156  4.01509
3.58188  2.14717  4.19338  5.59405  3.10441  2.10451  3.04901  2.71335
2.62620  3.78176  4.24573  4.69631  4.56895      9 e - - -
          2.68622  4.42229  2.77524  2.73112  3.46318  2.40517  3.72499
3.29358  2.67745  2.69359  4.24694  2.90332  2.73744  3.18150  2.89805
2.37891  2.77524  2.98522  4.58481  3.61472
          0.03221  4.05638  4.24162  0.20699  1.67681  0.48576  0.95510
      8   2.92198  5.82950  2.56791  3.11432  5.88223  3.43488  4.67289
2.48871  1.23277  3.18030  4.69497  2.96307  3.12506  3.46333  2.59828
2.92851  3.06283  3.15954  6.97109  4.87779     11 k - - -
          2.68618  4.42225  2.77519  2.73123  3.46354  2.40513  3.72494
3.29354  2.67741  2.69355  4.24690  2.90347  2.73739  3.18146  2.89801
2.37887  2.77519  2.98518  4.58477  3.61503
          0.00650  6.82991  5.22213  0.61958  0.77255  0.70663  0.67985
```

```
       9   3.45534  4.26014  3.01669  3.28500  2.92632  3.91965  5.26185
 2.63770  3.30873  1.25934  3.16421  4.15847  4.61931  4.17198  4.17826
 3.28183  1.94524  2.41421  4.40652  4.09181      12 l - - -
           2.68698  4.41819  2.77356  2.72970  3.46411  2.40426  3.72541
 3.29455  2.67785  2.69291  4.24791  2.90448  2.73841  3.18154  2.89902
 2.37861  2.77558  2.98570  4.58578  3.61459
           0.69119  2.65368  0.84717  1.68495  0.20512  0.64273  0.74624
      10   3.28266  4.91291  2.35819  2.87309  4.29557  4.08913  3.41511
 4.47688  2.52716  3.89793  4.77051  2.52133  1.40066  3.02904  2.41211
 2.90931  3.44123  2.87431  5.49785  4.72496      20 p - - .
           2.68618  4.42225  2.77519  2.73123  3.46354  2.40513  3.72494
 3.29354  2.67741  2.69355  4.24690  2.90347  2.73739  3.18146  2.89801
 2.37887  2.77519  2.98518  4.58477  3.61503
           0.19436  6.27476  1.74435  0.61958  0.77255  0.68312  0.70328
      11   3.49690  5.28923  5.27707  4.05757  3.96232  4.67148  5.00957
 1.48734  3.85528  2.48867  2.26954  4.27326  2.94907  4.57584  3.36502
 3.55141  1.74371  2.04320  3.88914  4.72418      21 i - - .
           2.68656  4.42185  2.77569  2.73181  3.46411  2.40570  3.72552
 3.29353  2.67788  2.69381  4.24654  2.90404  2.73797  3.18204  2.89764
 2.37944  2.77577  2.97728  4.58534  3.61560
           0.39431  1.12364  7.18147  0.02232  3.81356  0.86558  0.54612
      12   3.25743  6.32280  1.37648  3.40359  5.64405  4.52390  4.84347
 3.66488  3.40611  4.48188  5.11093  2.67634  2.54084  3.94800  3.82090
 2.72487  1.28177  3.42457  6.77001  4.60651      23 t - - .
           2.68618  4.42225  2.77519  2.73123  3.46354  2.40513  3.72494
 3.29354  2.67741  2.69355  4.24690  2.90347  2.73739  3.18146  2.89801
 2.37887  2.77519  2.98518  4.58477  3.61503
           0.00197  6.62586  7.34820  0.61958  0.77255  1.67254  0.20797
      13   2.83803  4.99137  5.63651  5.04193  3.86438  4.86270  5.45729
 2.18417  2.52834  2.20329  3.58923  4.00176  4.11638  4.41101  2.37225
 3.21104  1.48857  2.09054  6.07084  3.53004      24 t - - .
           2.68618  4.42225  2.77519  2.73123  3.46354  2.40513  3.72494
 3.29354  2.67741  2.69355  4.24690  2.90347  2.73739  3.18146  2.89801
 2.37887  2.77519  2.98518  4.58477  3.61503
           0.00194  6.64165  7.36400  0.61958  0.77255  0.09459  2.40516
      14   3.52907  6.55409  2.00218  1.17189  5.90056  4.48550  4.67224
 4.86890  2.38637  4.85997  5.59438  2.27760  4.23341  3.20000  2.63862
 2.60075  2.78848  3.90018  6.98593  5.57414      25 e - - H
           2.68682  4.42329  2.77475  2.72652  3.46149  2.40558  3.72560
 3.29441  2.67704  2.69405  4.24794  2.90426  2.73827  3.18183  2.89763
 2.37910  2.77601  2.98565  4.58581  3.61607
           0.51157  0.91647  7.56622  0.01253  4.38600  0.48576  0.95510
      15   2.40317  4.65516  3.39103  3.04485  5.33779  4.38407  2.28993
 2.76088  2.63324  2.35595  3.19523  3.87400  3.97271  2.38524  3.95590
 3.36687  1.81988  2.59476  6.88545  5.05947      27 t - - H
           2.68618  4.42225  2.77519  2.73123  3.46354  2.40513  3.72494
 3.29354  2.67741  2.69355  4.24690  2.90347  2.73739  3.18146  2.89801
 2.37887  2.77519  2.98518  4.58477  3.61503
           0.00158  6.84388  7.56622  0.61958  0.77255  0.48576  0.95510
      16   4.35594  5.71917  5.34681  5.72130  4.48341  5.53945  5.87557
 2.40587  5.50758  0.33433  3.80277  5.70654  5.88904  4.81442  5.50919
 4.03096  4.20023  2.70239  6.34325  5.17847      28 L - - H
           2.68657  4.42264  2.77559  2.73162  3.46393  2.40552  3.72491
 3.29357  2.67780  2.69027  4.24647  2.90386  2.73779  3.18172  2.89746
 2.37870  2.77559  2.98558  4.58516  3.61542
           0.14414  2.02498  6.10189  0.16759  1.86886  0.48576  0.95510
```

```
     17   3.11150  6.53969  4.29320  2.64333  5.88015  4.40090  5.00306
3.83012  1.53825  3.60417  4.91611  3.91772  5.20893  1.94481  1.49038
2.96721  2.77117  2.74871  6.97512  5.56702      31 r - - H
          2.68618  4.42225  2.77519  2.73123  3.46354  2.40513  3.72494
3.29354  2.67741  2.69355  4.24690  2.90347  2.73739  3.18146  2.89801
2.37887  2.77519  2.98518  4.58477  3.61503
          0.00159  6.84215  7.56450  0.61958  0.77255  0.46732  0.98531
     18   2.99818  4.02624  3.12236  2.10517  5.89996  3.51636  4.35385
4.61020  2.37114  4.85947  3.66997  3.25422  4.30216  1.76945  3.10367
1.59656  2.46342  4.10890  5.48083  4.73820      32 s - - H
          2.68618  4.42225  2.77519  2.73123  3.46354  2.40513  3.72494
3.29354  2.67741  2.69355  4.24690  2.90347  2.73739  3.18146  2.89801
2.37887  2.77519  2.98518  4.58477  3.61503
          0.00158  6.84388  7.56622  0.61958  0.77255  0.48576  0.95510
     19   3.83522  4.28203  4.52373  3.33962  5.61051  4.60579  5.17402
4.80188  3.80922  4.44036  5.43616  3.67048  4.53068  4.32117  4.43555
1.15374  0.77693  4.69264  6.86472  5.18501      33 t - - H
          2.68621  4.42074  2.77523  2.73127  3.46357  2.40516  3.72498
3.29358  2.67744  2.69358  4.24693  2.90350  2.73715  3.18150  2.89804
2.37890  2.77523  2.98522  4.58480  3.61507
          0.01591  4.18235  7.56622  0.22583  1.59878  0.48576  0.95510
     20   3.87919  6.55332  3.02794  2.88171  3.96428  1.12760  4.80609
5.38613  1.70879  3.32604  4.74594  3.01389  4.97767  3.08146  2.04585
3.44740  4.16246  4.71907  6.98537  5.57380      35 g - - S
          2.68618  4.42225  2.77519  2.73123  3.46354  2.40513  3.72494
3.29354  2.67741  2.69355  4.24690  2.90347  2.73739  3.18146  2.89801
2.37887  2.77519  2.98518  4.58477  3.61503
          0.00617  6.84388  5.28151  0.61958  0.77255  0.48576  0.95510
     21   3.47419  5.00858  7.07275  6.54164  5.56931  6.42245  6.94637
0.60320  6.40899  1.40584  4.94284  6.59389  6.71385  6.60086  6.48230
5.13991  4.38378  2.07067  7.36802  6.16445      36 I - - H
          2.68618  4.42225  2.77519  2.73123  3.46354  2.40513  3.72494
3.29354  2.67741  2.69355  4.24690  2.90347  2.73739  3.18146  2.89801
2.37887  2.77519  2.98518  4.58477  3.61503
          0.00159  6.83930  7.56165  0.61958  0.77255  0.43976  1.03336
     22   2.73960  5.89435  4.93827  3.24750  4.73026  0.41991  4.01095
3.98463  3.92642  4.01858  4.99508  4.79692  3.78104  4.64288  4.47729
4.32890  3.97402  3.32738  4.84884  4.44418      37 G - - H
          2.68618  4.42225  2.77519  2.73123  3.46354  2.40513  3.72494
3.29354  2.67741  2.69355  4.24690  2.90347  2.73739  3.18146  2.89801
2.37887  2.77519  2.98518  4.58477  3.61503
          0.00158  6.84388  7.56622  0.61958  0.77255  0.48576  0.95510
     23   3.59920  5.12981  3.60495  3.15994  5.84985  4.60944  4.01875
2.66845  0.87676  3.67724  3.86352  3.25931  4.75222  3.41862  2.24129
3.48300  4.01480  2.39903  6.96057  4.84263      38 k - - H
          2.68618  4.42225  2.77519  2.73123  3.46354  2.40513  3.72494
3.29354  2.67741  2.69355  4.24690  2.90347  2.73739  3.18146  2.89801
2.37887  2.77519  2.98518  4.58477  3.61503
          0.00158  6.84388  7.56622  0.61958  0.77255  0.48576  0.95510
     24   1.88371  5.40437  4.83420  4.33969  3.27767  5.25675  4.62205
2.45229  4.43403  3.03389  4.50110  4.61888  2.61853  4.63711  2.00168
3.06562  1.95193  1.74256  6.29142  3.94149      39 v - - H
          2.68618  4.42225  2.77519  2.73123  3.46354  2.40513  3.72494
3.29354  2.67741  2.69355  4.24690  2.90347  2.73739  3.18146  2.89801
2.37887  2.77519  2.98518  4.58477  3.61503
          0.00158  6.84388  7.56622  0.61958  0.77255  0.48576  0.95510
```

```
     25   5.05499  6.66836  7.74492  7.33754  6.03588  7.38677  8.43215
1.66100  7.37265  2.32553  3.28733  7.56144  7.48594  7.63250  7.58588
6.90326  5.79773  0.42259  8.45378  7.25717     40 V - - H
          2.68618  4.42225  2.77519  2.73123  3.46354  2.40513  3.72494
3.29354  2.67741  2.69355  4.24690  2.90347  2.73739  3.18146  2.89801
2.37887  2.77519  2.98518  4.58477  3.61503
          0.00158  6.84388  7.56622  0.61958  0.77255  0.48576  0.95510
     26   2.94282  4.83069  4.22724  3.23041  5.48122  2.87514  4.14046
4.90218  2.23168  3.48689  2.47817  1.11212  5.21739  3.52521  2.61189
3.51569  2.87629  3.51103  6.95584  3.67325     41 n - - H
          2.68618  4.42225  2.77519  2.73123  3.46354  2.40513  3.72494
3.29354  2.67741  2.69355  4.24690  2.90347  2.73739  3.18146  2.89801
2.37887  2.77519  2.98518  4.58477  3.61503
          0.00158  6.84388  7.56622  0.61958  0.77255  0.48576  0.95510
     27   1.66564  3.30396  3.78474  3.25921  2.17600  3.00170  4.68180
5.36704  1.83884  3.51463  4.31014  3.13504  4.52849  2.91908  2.59487
2.77231  3.43038  4.57581  6.97760  3.10815     42 a - - H
          2.68622  4.42229  2.77523  2.73127  3.46320  2.40517  3.72498
3.29312  2.67745  2.69359  4.24694  2.90351  2.73743  3.18150  2.89805
2.37891  2.77523  2.98522  4.58481  3.61464
          0.01723  4.10020  7.56622  0.21344  1.64924  0.48576  0.95510
     28   3.71743  5.26924  6.24780  5.63225  3.27912  5.44316  5.77786
1.29307  5.41515  0.80742  3.81850  5.61096  5.80093  4.77211  5.41662
4.75641  4.31994  2.67311  6.25764  2.80218     44 l - - H
          2.68618  4.42225  2.77519  2.73123  3.46354  2.40513  3.72494
3.29354  2.67741  2.69355  4.24690  2.90347  2.73739  3.18146  2.89801
2.37887  2.77519  2.98518  4.58477  3.61503
          0.23252  6.84388  1.57793  0.61958  0.77255  0.48576  0.95510
     29   2.25949  3.93389  3.61528  3.27424  3.80296  4.02593  4.27582
3.94657  2.46137  2.74971  4.05630  3.89885  5.06136  3.27170  1.42639
2.01058  2.67199  4.30707  5.03201  3.13317     45 r - - H
          2.68642  4.42451  2.77500  2.73143  3.46580  2.40488  3.72152
3.29407  2.67579  2.69272  4.24822  2.90256  2.73690  3.18245  2.89607
2.38054  2.77655  2.98665  4.58703  3.61240
          0.40185  1.32734  2.72210  1.40704  0.28086  2.02684  0.14128
     30   3.59182  5.36813  3.04598  2.84803  5.66383  3.06869  4.35993
4.81622  0.90904  3.88036  5.35778  2.86852  3.86307  3.17183  2.05588
2.81244  3.33290  4.70880  5.78531  4.87184     54 k - - H
          2.68630  4.41449  2.77341  2.73023  3.46448  2.40618  3.72582
3.29414  2.67757  2.69155  4.24828  2.90244  2.73820  3.18188  2.89939
2.37916  2.77657  2.98598  4.58615  3.61282
          0.38678  2.06216  1.64207  1.69364  0.20316  0.07130  2.67623
     31   3.53997  4.07060  3.51872  2.52225  4.61343  4.07517  1.75728
4.20868  2.63761  3.76361  5.18566  2.57522  5.08357  3.67058  1.86737
2.05821  2.65661  4.52195  6.85793  2.85989     63 h - - -
          2.68618  4.42225  2.77519  2.73123  3.46354  2.40513  3.72494
3.29354  2.67741  2.69355  4.24690  2.90347  2.73739  3.18146  2.89801
2.37887  2.77519  2.98518  4.58477  3.61503
          0.15820  6.68933  1.93048  0.61958  0.77255  1.69770  0.20224
     32   3.20183  3.62856  2.48434  2.81461  5.33677  3.76486  3.83253
4.73504  1.22004  4.42866  5.34965  3.19121  2.09077  3.45029  3.47122
2.38956  2.74872  3.51008  6.74131  5.32969     64 k - - T
          2.68618  4.42225  2.77519  2.73123  3.46354  2.40513  3.72494
3.29354  2.67741  2.69355  4.24690  2.90347  2.73739  3.18146  2.89801
2.37887  2.77519  2.98518  4.58477  3.61503
          0.09638  6.53598  2.40319  0.61958  0.77255  1.14676  0.38224
```

```
     33   3.05016  5.08685  3.16953  2.19716  4.96512  3.55532  1.63123
5.14464  2.19522  4.02649  4.98431  2.77940  2.64661  3.34705  2.83610
2.31374  2.66067  4.70270  6.74325  4.24092      65 h - - T
          2.68618  4.42225  2.77519  2.73123  3.46354  2.40513  3.72494
3.29354  2.67741  2.69355  4.24690  2.90347  2.73739  3.18146  2.89801
2.37887  2.77519  2.98518  4.58477  3.61503
          0.28927  6.53808  1.38732  0.61958  0.77255  2.00287  0.14496
     34   3.24820  1.79548  2.59103  1.40529  3.91058  3.65780  4.56443
3.91209  2.50876  4.38474  5.12737  3.44471  2.70786  3.44098  3.79432
3.21485  2.76297  3.35176  6.52297  5.11844      66 e - - S
          2.68720  4.41711  2.77283  2.73117  3.46456  2.40455  3.72597
3.29257  2.67741  2.69353  4.24792  2.90314  2.73727  3.18249  2.89903
2.37975  2.77503  2.98474  4.58579  3.61605
          0.11708  2.21125  6.98946  1.80594  0.17951  0.04708  3.07938
     35   2.00290  5.06547  2.30432  2.48941  5.89682  3.88330  3.01303
3.76676  3.36940  3.96464  5.26379  1.89095  4.74253  3.64041  3.84134
2.06435  1.77998  4.43671  6.98359  5.09235      74 t - - -
          2.68618  4.42225  2.77519  2.73123  3.46354  2.40513  3.72494
3.29354  2.67741  2.69355  4.24690  2.90347  2.73739  3.18146  2.89801
2.37887  2.77519  2.98518  4.58477  3.61503
          0.01835  6.84273  4.06802  0.61958  0.77255  0.47330  0.97536
     36   1.93025  6.53953  2.57256  2.15308  3.39370  3.72902  4.47546
4.32787  2.29522  4.42036  3.96909  3.19334  2.08712  3.03357  2.90002
2.39078  3.26331  3.11712  6.97162  5.56007      75 a - - H
          2.68618  4.42225  2.77519  2.73123  3.46354  2.40513  3.72494
3.29354  2.67741  2.69355  4.24690  2.90347  2.73739  3.18146  2.89801
2.37887  2.77519  2.98518  4.58477  3.61503
          0.00161  6.82716  7.54951  0.61958  0.77255  0.74459  0.64422
     37   2.82710  6.54029  2.91333  1.76405  5.25434  3.54567  3.70490
4.32346  1.99439  4.02862  4.60113  3.40194  3.75883  2.21776  1.88573
2.41028  3.39759  3.52548  6.97217  4.95450      76 e - - H
          2.68618  4.42225  2.77519  2.73123  3.46354  2.40513  3.72494
3.29354  2.67741  2.69355  4.24690  2.90347  2.73739  3.18146  2.89801
2.37887  2.77519  2.98518  4.58477  3.61503
          0.00161  6.82716  7.54951  0.61958  0.77255  0.35244  1.21393
     38   3.61430  5.58542  6.19014  5.57454  3.74409  5.38600  4.55435
1.32120  5.35717  1.61344  4.70433  2.20303  5.74902  5.48635  5.36138
4.69818  3.91670  1.27355  6.21074  4.45938      77 v - - H
          2.68618  4.42225  2.77519  2.73123  3.46354  2.40513  3.72494
3.29354  2.67741  2.69355  4.24690  2.90347  2.73739  3.18146  2.89801
2.37887  2.77519  2.98518  4.58477  3.61503
          0.00158  6.84388  7.56622  0.61958  0.77255  0.48576  0.95510
     39   2.47401  5.92590  4.28884  2.04031  5.89713  3.15333  3.16867
3.74615  1.48249  4.85715  5.59231  3.79962  5.20750  2.82594  1.89284
2.28098  4.01560  2.96322  5.07333  5.57309      78 k - - H
          2.68618  4.42225  2.77519  2.73123  3.46354  2.40513  3.72494
3.29354  2.67741  2.69355  4.24690  2.90347  2.73739  3.18146  2.89801
2.37887  2.77519  2.98518  4.58477  3.61503
          0.00352  6.84388  6.01070  0.61958  0.77255  0.48576  0.95510
     40   2.81475  5.55971  2.33514  2.44141  4.94497  4.06923  4.43769
4.59381  2.09710  3.11584  5.59249  3.34380  4.44876  2.11402  2.30387
2.46814  1.81998  4.18700  6.98409  5.32693      79 t - - H
          2.68618  4.42225  2.77519  2.73123  3.46354  2.40513  3.72494
3.29354  2.67741  2.69355  4.24690  2.90347  2.73739  3.18146  2.89801
2.37887  2.77519  2.98518  4.58477  3.61503
          0.00159  6.84194  7.56429  0.61958  0.77255  0.46513  0.98900
```

```
     41   3.16934  6.04939  4.58378  3.00517  4.64550  4.80300  4.66199
3.00503  2.25453  0.97946  3.23530  4.37657  4.55403  3.66331  1.96268
3.69275  3.50084  3.13712  6.59768  4.58374     80 l - - H
          2.68800  4.40145  2.77656  2.73796  3.47027  2.40815  3.71787
3.30027  2.67443  2.69042  4.25363  2.90410  2.73817  3.16427  2.89265
2.38199  2.77656  2.97874  4.59150  3.62176
          0.16237  1.90144  7.56622  2.82451  0.06117  0.48576  0.95510
     42   0.42552  2.92582  6.16057  5.55779  4.73797  4.96389  5.75348
3.72623  5.35332  3.06166  4.55799  5.54905  5.75735  5.49359  4.32531
2.79836  4.35712  2.57986  6.26660  5.09023    101 A - - H
          2.68618  4.42225  2.77519  2.73123  3.46354  2.40513  3.72494
3.29354  2.67741  2.69355  4.24690  2.90347  2.73739  3.18146  2.89801
2.37887  2.77519  2.98518  4.58477  3.61503
          0.00158  6.84388  7.56622  0.61958  0.77255  0.48576  0.95510
     43   1.98399  6.55392  3.27886  2.52383  4.93007  3.36366  4.21949
4.01505  1.38866  4.35284  5.31565  3.02960  3.82958  3.08312  2.37826
2.27639  2.71340  4.66453  6.98580  4.30060    102 k - - H
          2.68618  4.42225  2.77519  2.73123  3.46354  2.40513  3.72494
3.29354  2.67741  2.69355  4.24690  2.90347  2.73739  3.18146  2.89801
2.37887  2.77519  2.98518  4.58477  3.61503
          0.00158  6.84388  7.56622  0.61958  0.77255  0.48576  0.95510
     44   2.57177  4.77925  2.18525  2.08284  5.90022  3.54751  4.19650
4.40551  1.93096  4.11857  5.36117  2.65942  5.20693  2.58780  2.66356
1.82022  3.24145  4.16312  4.80790  4.79962    103 s - - H
          2.68618  4.42225  2.77519  2.73123  3.46354  2.40513  3.72494
3.29354  2.67741  2.69355  4.24690  2.90347  2.73739  3.18146  2.89801
2.37887  2.77519  2.98518  4.58477  3.61503
          0.00158  6.84388  7.56622  0.61958  0.77255  0.48576  0.95510
     45   4.58371  6.73567  7.55623  6.98889  5.25338  6.93536  5.14818
1.88767  6.85128  0.36928  4.04063  7.11686  7.04188  6.78600  6.81213
6.32157  5.73558  2.27667  7.40353  6.39924    104 L - - H
          2.68618  4.42225  2.77519  2.73123  3.46354  2.40513  3.72494
3.29354  2.67741  2.69355  4.24690  2.90347  2.73739  3.18146  2.89801
2.37887  2.77519  2.98518  4.58477  3.61503
          0.00158  6.84388  7.56622  0.61958  0.77255  0.48576  0.95510
     46   3.95230  3.98680  6.17955  4.28104  3.55077  5.37327  5.35833
1.44993  2.88403  2.00338  3.73661  5.54004  5.73685  5.47372  5.03658
4.53599  3.63936  0.99874  6.19741  3.38533    105 v - - H
          2.68618  4.42225  2.77519  2.73123  3.46354  2.40513  3.72494
3.29354  2.67741  2.69355  4.24690  2.90347  2.73739  3.18146  2.89801
2.37887  2.77519  2.98518  4.58477  3.61503
          0.00158  6.84388  7.56622  0.61958  0.77255  0.48576  0.95510
     47   2.76808  5.10074  2.21198  1.50236  5.90048  3.41863  3.60716
4.77248  1.89981  4.43911  5.59432  2.26206  5.00128  3.25289  2.98947
2.34804  3.14549  4.22379  6.98587  4.73084    106 e - - H
          2.68618  4.42225  2.77519  2.73123  3.46354  2.40513  3.72494
3.29354  2.67741  2.69355  4.24690  2.90347  2.73739  3.18146  2.89801
2.37887  2.77519  2.98518  4.58477  3.61503
          0.00158  6.84388  7.56622  0.61958  0.77255  0.48576  0.95510
     48   3.34019  5.19718  3.28264  2.17279  4.82810  3.82207  4.44097
4.63983  1.26606  4.26565  5.23077  2.91190  5.20694  3.07184  1.47308
2.68466  3.38342  4.80087  6.98573  4.79642    107 k - - H
          2.68627  4.42234  2.77452  2.73133  3.46363  2.40522  3.72504
3.29363  2.67750  2.69364  4.24699  2.90356  2.73749  3.18156  2.89810
2.37896  2.77529  2.98528  4.58486  3.61335
          0.02014  3.94149  7.56622  0.67564  0.71097  0.48576  0.95510
```

```
     49   7.94677  8.61826  8.15679  8.24374  8.05171  5.05889  8.59704
9.06772  8.40042  8.12328  9.47603  8.48752  7.86525  8.68930  8.16196
8.28446  8.37757  8.69187  0.01071  8.10637    110 W - - H
          2.68618  4.42225  2.77519  2.73123  3.46354  2.40513  3.72494
3.29354  2.67741  2.69355  4.24690  2.90347  2.73739  3.18146  2.89801
2.37887  2.77519  2.98518  4.58477  3.61503
          0.00158  6.84388  7.56622  0.61958  0.77255  0.48576  0.95510
     50   2.66942  6.54515  4.29219  3.02470  4.97027  3.28705  4.27308
4.25830  1.13406  4.35339  3.72044  3.66245  5.20910  3.33262  1.84323
2.03563  3.13865  3.48725  6.97946  5.25441    111 k - - H
          2.68625  4.42232  2.77526  2.73130  3.46361  2.40508  3.72501
3.29361  2.67734  2.69362  4.24696  2.90353  2.73746  3.18153  2.89719
2.37893  2.77526  2.98525  4.58484  3.61510
          0.11734  3.60083  2.48391  0.14720  1.98867  0.48576  0.95510
     51   2.07563  6.48375  2.95738  2.99111  5.83016  3.34014  3.41849
4.84693  1.51525  3.41473  4.69278  3.07805  4.38112  2.99096  1.68094
2.67675  3.10368  4.43818  6.91563  4.76240    113 k - - H
          2.68587  4.42238  2.77532  2.73136  3.46367  2.40508  3.72507
3.29326  2.67725  2.69368  4.24702  2.90359  2.73752  3.18159  2.89760
2.37878  2.77532  2.98531  4.58490  3.61516
          0.02901  3.57450  7.47969  0.67926  0.70723  0.37529  1.16183
     52   1.75706  5.19856  3.23407  3.42950  3.79344  4.66369  4.17463
2.02702  3.25104  2.06485  2.84963  3.35394  2.23447  3.26699  3.62014
2.74762  3.62010  3.12287  6.69548  5.38002    116 a - - H
          2.68618  4.42225  2.77519  2.73123  3.46354  2.40513  3.72494
3.29354  2.67741  2.69355  4.24690  2.90347  2.73739  3.18146  2.89801
2.37887  2.77519  2.98518  4.58477  3.61503
          0.00162  6.82173  7.54407  0.61958  0.77255  0.75804  0.63221
     53   2.94229  4.56552  6.16364  5.54724  3.58325  4.97062  5.06891
1.49057  5.32875  2.07568  3.50624  5.52337  5.71972  5.45716  2.86380
3.42213  4.18402  1.00304  6.17997  3.96556    117 v - - H
          2.68618  4.42225  2.77519  2.73123  3.46354  2.40513  3.72494
3.29354  2.67741  2.69355  4.24690  2.90347  2.73739  3.18146  2.89801
2.37887  2.77519  2.98518  4.58477  3.61503
          0.00109  6.82328        *  0.61958  0.77255  0.00000        *
//
HMMER3/f [3.3 | Nov 2019]
NAME  Med31
ACC   PF05669.13
DESC  SOH1
LENG  95
ALPH  amino
RF    no
MM    no
CONS  yes
CS    yes
MAP   yes
DATE  Fri Apr 17 10:25:45 2020
NSEQ  72
EFFN  1.226074
CKSUM 510158446
GA    25.00 25.00
TC    25.00 25.00
NC    24.50 24.80
STATS LOCAL MSV       -9.4984  0.71829
STATS LOCAL VITERBI  -10.4061  0.71829
STATS LOCAL FORWARD   -4.1832  0.71829
```

```
HMM          A        C        D        E        F        G        H
I        K        L        M        N        P        Q        R        S
T        V        W        Y
             m->m     m->i     m->d     i->m     i->i     d->m     d->d
  COMPO   2.63013  4.41947  3.15369  2.58822  2.88695  3.44050  3.62476
2.98392  2.69816  2.20631  3.68378  3.07044  3.41721  2.94246  2.96214
2.76430  2.95250  2.82201  4.33221  2.83136
          2.68618  4.42225  2.77519  2.73123  3.46354  2.40513  3.72494
3.29354  2.67741  2.69355  4.24690  2.90347  2.73739  3.18146  2.89801
2.37887  2.77519  2.98518  4.58477  3.61503
          0.06819  4.30604  2.94836  0.61958  0.77255  0.00000        *
      1   2.63978  5.10279  2.83029  2.23512  4.43047  3.41543  3.38268
3.90216  2.10968  3.39891  4.14895  2.50677  3.36309  2.45621  2.55368
2.42627  2.87052  3.44602  5.54414  3.62518      1 k - - C
          2.68618  4.42225  2.77519  2.73123  3.46354  2.40513  3.72494
3.29354  2.67741  2.69355  4.24690  2.90347  2.73739  3.18146  2.89801
2.37887  2.77519  2.98518  4.58477  3.61503
          0.02122  4.25908  4.98143  0.61958  0.77255  0.44850  1.01773
      2   2.46383  4.70292  3.13367  2.57393  3.90686  3.50255  3.71624
3.02439  2.36905  2.66419  3.59568  2.88089  2.96516  2.77416  2.89457
2.61237  2.37516  2.89202  5.23131  3.92743      2 k - - H
          2.68618  4.42225  2.77519  2.73123  3.46354  2.40513  3.72494
3.29354  2.67741  2.69355  4.24690  2.90347  2.73739  3.18146  2.89801
2.37887  2.77519  2.98518  4.58477  3.61503
          0.02024  4.30604  5.02839  0.61958  0.77255  0.48576  0.95510
      3   3.04986  5.20293  3.65762  3.03250  4.72898  3.77365  3.81102
4.07366  2.01075  3.55056  4.43941  3.39756  4.19277  2.95910  0.89249
3.11022  3.37445  3.75088  5.60483  4.44504      3 r - - H
          2.68618  4.42225  2.77519  2.73123  3.46354  2.40513  3.72494
3.29354  2.67741  2.69355  4.24690  2.90347  2.73739  3.18146  2.89801
2.37887  2.77519  2.98518  4.58477  3.61503
          0.02024  4.30604  5.02839  0.61958  0.77255  0.48576  0.95510
      4   3.95589  5.11714  5.03602  4.77522  0.64721  4.66756  3.70385
3.52044  4.56708  2.75905  4.08130  4.38851  4.96850  4.46273  4.51865
4.08147  4.17778  3.49861  2.91652  2.08903      4 F - - H
          2.68618  4.42225  2.77519  2.73123  3.46354  2.40513  3.72494
3.29354  2.67741  2.69355  4.24690  2.90347  2.73739  3.18146  2.89801
2.37887  2.77519  2.98518  4.58477  3.61503
          0.02024  4.30604  5.02839  0.61958  0.77255  0.48576  0.95510
      5   2.68114  5.02293  2.92022  1.69479  4.31482  3.44850  3.59276
3.66380  2.41892  3.27320  4.09208  2.94868  3.85685  2.42419  2.88466
2.57588  2.37688  3.26095  5.49659  4.12906      5 e - - H
          2.68618  4.42225  2.77519  2.73123  3.46354  2.40513  3.72494
3.29354  2.67741  2.69355  4.24690  2.90347  2.73739  3.18146  2.89801
2.37887  2.77519  2.98518  4.58477  3.61503
          0.02024  4.30604  5.02839  0.61958  0.77255  0.48576  0.95510
      6   2.54731  3.86323  3.94098  3.25479  3.25755  3.70349  3.99460
2.26664  3.10797  1.72338  3.06217  3.62429  4.07503  3.35209  3.19910
2.92421  2.73598  2.21534  4.75549  3.44742      6 l - - H
          2.68618  4.42225  2.77519  2.73123  3.46354  2.40513  3.72494
3.29354  2.67741  2.69355  4.24690  2.90347  2.73739  3.18146  2.89801
2.37887  2.77519  2.98518  4.58477  3.61503
          0.02024  4.30604  5.02839  0.61958  0.77255  0.48576  0.95510
      7   3.16261  5.73588  1.99799  0.85984  5.03451  3.39597  3.96609
4.56903  3.00015  4.06323  4.92987  2.86560  4.03158  3.13779  3.59940
3.03739  3.45768  4.13564  6.19495  4.69103      7 e - - H
```

2.68618 4.42225 2.77519 2.73123 3.46354 2.40513 3.72494
3.29354 2.67741 2.69355 4.24690 2.90347 2.73739 3.18146 2.89801
2.37887 2.77519 2.98518 4.58477 3.61503
0.02024 4.30604 5.02839 0.61958 0.77255 0.48576 0.95510
8 3.25854 3.89227 4.83602 4.35395 3.33324 4.28992 4.86298
2.41155 4.12497 0.67452 3.22857 4.50735 4.69046 4.35556 4.24977
3.73428 3.55435 2.44140 5.28592 4.10499 8 L - - H
2.68618 4.42225 2.77519 2.73123 3.46354 2.40513 3.72494
3.29354 2.67741 2.69355 4.24690 2.90347 2.73739 3.18146 2.89801
2.37887 2.77519 2.98518 4.58477 3.61503
0.02024 4.30604 5.02839 0.61958 0.77255 0.48576 0.95510
9 3.21268 5.60995 2.37104 0.72353 4.95675 3.45973 4.04819
4.47801 3.03478 4.01958 4.91895 2.98833 4.09307 3.23508 3.56220
3.11935 3.51548 4.07894 6.10659 4.69191 9 e - - H
2.68618 4.42225 2.77519 2.73123 3.46354 2.40513 3.72494
3.29354 2.67741 2.69355 4.24690 2.90347 2.73739 3.18146 2.89801
2.37887 2.77519 2.98518 4.58477 3.61503
0.02024 4.30604 5.02839 0.61958 0.77255 0.48576 0.95510
10 3.86995 5.14876 4.84261 4.65798 0.50288 4.41565 4.15102
3.36536 4.56086 2.64205 4.04057 4.54039 4.87822 4.61239 4.57185
4.10282 4.17821 3.38479 4.30688 2.63420 10 F - - H
2.68618 4.42225 2.77519 2.73123 3.46354 2.40513 3.72494
3.29354 2.67741 2.69355 4.24690 2.90347 2.73739 3.18146 2.89801
2.37887 2.77519 2.98518 4.58477 3.61503
0.02024 4.30604 5.02839 0.61958 0.77255 0.48576 0.95510
11 3.28237 4.56691 5.07012 4.56910 3.70590 4.68451 5.25149
1.74842 4.44649 2.00277 3.50808 4.77405 4.96725 4.70317 4.62863
4.07701 3.54382 0.79605 5.70519 4.49691 11 v - - H
2.68618 4.42225 2.77519 2.73123 3.46354 2.40513 3.72494
3.29354 2.67741 2.69355 4.24690 2.90347 2.73739 3.18146 2.89801
2.37887 2.77519 2.98518 4.58477 3.61503
0.02024 4.30604 5.02839 0.61958 0.77255 0.48576 0.95510
12 2.70379 4.88431 3.05742 2.54152 4.14683 3.49059 3.71628
3.57511 2.45596 2.76048 3.86793 2.85924 3.90535 1.67675 2.86842
2.33622 2.94279 3.24961 5.39262 4.06365 12 q - - H
2.68618 4.42225 2.77519 2.73123 3.46354 2.40513 3.72494
3.29354 2.67741 2.69355 4.24690 2.90347 2.73739 3.18146 2.89801
2.37887 2.77519 2.98518 4.58477 3.61503
0.02024 4.30604 5.02839 0.61958 0.77255 0.48576 0.95510
13 2.04053 2.80179 3.73570 3.18765 3.54682 3.51695 4.02640
2.84968 3.11806 2.39301 3.23727 3.49181 3.99952 3.40546 3.42491
1.82493 2.86871 2.61001 5.01338 3.79685 13 s - - H
2.68618 4.42225 2.77519 2.73123 3.46354 2.40513 3.72494
3.29354 2.67741 2.69355 4.24690 2.90347 2.73739 3.18146 2.89801
2.37887 2.77519 2.98518 4.58477 3.61503
0.02024 4.30604 5.02839 0.61958 0.77255 0.48576 0.95510
14 3.62060 4.92538 5.15931 4.64936 3.09070 4.84352 5.10017
2.24223 4.44816 0.60026 3.00757 4.89310 5.00730 4.55551 4.53826
4.24555 3.85198 2.48822 5.28925 4.12563 14 L - - H
2.68618 4.42225 2.77519 2.73123 3.46354 2.40513 3.72494
3.29354 2.67741 2.69355 4.24690 2.90347 2.73739 3.18146 2.89801
2.37887 2.77519 2.98518 4.58477 3.61503
0.02024 4.30604 5.02839 0.61958 0.77255 0.48576 0.95510
15 0.94346 3.08303 4.05845 3.67054 4.32434 2.75231 4.51715
3.71061 3.60987 3.43253 4.26799 3.63129 3.86077 3.85826 3.85726
2.01791 2.83002 3.19809 5.71881 4.55115 15 a - - C

```
          2.68618  4.42225  2.77519  2.73123  3.46354  2.40513  3.72494
3.29354  2.67741  2.69355  4.24690  2.90347  2.73739  3.18146  2.89801
2.37887  2.77519  2.98518  4.58477  3.61503
          0.02024  4.30604  5.02839  0.61958  0.77255  0.48576  0.95510
     16   2.84432  5.05250  2.70360  2.52845  4.44117  3.37946  3.58183
4.07849  2.78723  3.63646  4.48098  1.13482  3.97506  3.12315  3.21696
2.40808  3.17255  3.66148  5.71558  4.29805     16 n - - S
          2.68618  4.42225  2.77519  2.73123  3.46354  2.40513  3.72494
3.29354  2.67741  2.69355  4.24690  2.90347  2.73739  3.18146  2.89801
2.37887  2.77519  2.98518  4.58477  3.61503
          0.02024  4.30604  5.02839  0.61958  0.77255  0.48576  0.95510
     17   2.73164  4.52051  3.72091  3.29836  3.76924  3.56452  4.24788
2.47549  3.17490  2.61265  3.76881  3.60354  1.21380  3.56118  3.47436
2.95658  3.08739  2.63607  5.35209  4.06550     17 p - - H
          2.68618  4.42225  2.77519  2.73123  3.46354  2.40513  3.72494
3.29354  2.67741  2.69355  4.24690  2.90347  2.73739  3.18146  2.89801
2.37887  2.77519  2.98518  4.58477  3.61503
          0.02024  4.30604  5.02839  0.61958  0.77255  0.48576  0.95510
     18   2.47685  4.72961  2.87641  2.53352  3.27508  3.49559  2.99839
3.30192  2.54082  2.94882  3.80904  2.85982  3.73453  2.51945  2.89471
2.70540  2.84283  3.01991  4.32466  2.82816     18 a - - H
          2.68618  4.42225  2.77519  2.73123  3.46354  2.40513  3.72494
3.29354  2.67741  2.69355  4.24690  2.90347  2.73739  3.18146  2.89801
2.37887  2.77519  2.98518  4.58477  3.61503
          0.02024  4.30604  5.02839  0.61958  0.77255  0.48576  0.95510
     19   3.93892  5.18847  4.71948  4.48192  2.07596  4.55573  3.68500
3.68982  4.31241  2.99701  4.30428  4.26674  4.91598  4.34554  4.36164
3.99701  4.18712  3.61357  3.82720  0.57538     19 Y - - H
          2.68618  4.42225  2.77519  2.73123  3.46354  2.40513  3.72494
3.29354  2.67741  2.69355  4.24690  2.90347  2.73739  3.18146  2.89801
2.37887  2.77519  2.98518  4.58477  3.61503
          0.02024  4.30604  5.02839  0.61958  0.77255  0.48576  0.95510
     20   3.49751  4.77109  5.23881  4.69004  3.29622  4.84283  5.21344
1.79598  4.55086  0.79738  3.06686  4.91911  4.99836  4.62618  4.63685
4.21861  3.72563  2.01301  5.43393  4.38119     20 l - - H
          2.68618  4.42225  2.77519  2.73123  3.46354  2.40513  3.72494
3.29354  2.67741  2.69355  4.24690  2.90347  2.73739  3.18146  2.89801
2.37887  2.77519  2.98518  4.58477  3.61503
          0.02024  4.30604  5.02839  0.61958  0.77255  0.48576  0.95510
     21   2.57940  5.03785  2.94484  2.32559  4.33706  3.44131  2.91717
3.78933  2.33228  3.21684  3.87145  2.32076  3.83449  2.51488  2.77185
2.19678  2.86169  3.40390  5.49702  3.98080     21 s - - H
          2.68618  4.42225  2.77519  2.73123  3.46354  2.40513  3.72494
3.29354  2.67741  2.69355  4.24690  2.90347  2.73739  3.18146  2.89801
2.37887  2.77519  2.98518  4.58477  3.61503
          0.02024  4.30604  5.02839  0.61958  0.77255  0.48576  0.95510
     22   2.63585  4.47611  3.39655  2.72910  2.67886  3.59926  2.43879
3.00171  2.79678  2.68926  3.57527  3.06528  3.98059  2.91906  3.18055
2.67632  2.89503  2.76349  4.17579  2.73509     22 h - - H
          2.68618  4.42225  2.77519  2.73123  3.46354  2.40513  3.72494
3.29354  2.67741  2.69355  4.24690  2.90347  2.73739  3.18146  2.89801
2.37887  2.77519  2.98518  4.58477  3.61503
          0.02024  4.30604  5.02839  0.61958  0.77255  0.48576  0.95510
     23   3.58777  4.94259  4.97435  4.54401  3.21480  4.66526  5.04501
2.37085  4.27960  0.57261  3.09622  4.77692  4.93880  4.50169  4.38637
4.13922  3.84886  2.55641  5.31193  4.12014     23 L - - T
```

```
          2.68610  4.42228  2.77522  2.73126  3.46357  2.40515  3.72497
3.29357  2.67743  2.69358  4.24692  2.90349  2.73742  3.18149  2.89790
2.37882  2.77511  2.98521  4.58480  3.61506
          0.05665  3.02571  5.02839  0.93162  0.50073  0.48576  0.95510
     24   1.49698  4.54108  3.26524  2.70394  3.59930  3.42695  3.68367
3.11561  2.71750  2.79703  3.66691  3.19956  3.94317  2.96059  3.11604
2.74900  2.78163  2.85734  5.11731  3.41499      28 a - - T
          2.68618  4.42225  2.77519  2.73123  3.46354  2.40513  3.72494
3.29354  2.67741  2.69355  4.24690  2.90347  2.73739  3.18146  2.89801
2.37887  2.77519  2.98518  4.58477  3.61503
          0.02024  4.30604  5.02839  0.61958  0.77255  0.48576  0.95510
     25   2.58214  4.86008  2.97162  2.27832  4.10348  3.46829  3.62483
3.38910  2.43951  2.99296  3.92964  2.93520  3.85898  2.21320  2.88882
2.43400  2.70992  2.53870  5.35877  3.87345      29 q - - S
          2.68618  4.42225  2.77519  2.73123  3.46354  2.40513  3.72494
3.29354  2.67741  2.69355  4.24690  2.90347  2.73739  3.18146  2.89801
2.37887  2.77519  2.98518  4.58477  3.61503
          0.02024  4.30604  5.02839  0.61958  0.77255  0.48576  0.95510
     26   2.49338  5.09088  2.92114  2.12466  4.40985  3.37700  3.39711
3.87539  2.27861  3.26981  4.13937  2.53488  3.82526  2.39108  2.55868
2.54809  2.55307  3.46761  5.53776  3.92435      30 e - - S
          2.68618  4.42225  2.77519  2.73123  3.46354  2.40513  3.72494
3.29354  2.67741  2.69355  4.24690  2.90347  2.73739  3.18146  2.89801
2.37887  2.77519  2.98518  4.58477  3.61503
          0.15776  4.30604  2.02149  0.61958  0.77255  0.48576  0.95510
     27   2.71003  5.17145  2.40030  2.25595  4.48808  2.25999  3.64341
3.96016  2.06638  3.46277  4.23368  2.61720  3.82694  2.66440  2.81037
2.59277  2.94930  3.54956  5.61643  4.20968      31 k - - S
          2.68621  4.42228  2.77522  2.73126  3.46357  2.40504  3.72497
3.29357  2.67733  2.69347  4.24693  2.90350  2.73742  3.18149  2.89804
2.37886  2.77517  2.98521  4.58480  3.61506
          0.08376  2.61923  4.89382  0.83945  0.56554  0.39572  1.11839
     28   2.95354  4.39178  4.30232  3.76748  2.25033  3.98860  3.48274
2.63138  3.63914  2.14923  3.48882  3.80099  4.34070  3.80986  3.79742
3.28632  3.18226  2.52898  4.24539  1.40270      35 y - - S
          2.68651  4.42281  2.77409  2.73158  3.46410  2.40530  3.72534
3.29352  2.67723  2.69335  4.24746  2.90333  2.73737  3.18160  2.89818
2.37814  2.77532  2.98566  4.58533  3.61560
          0.15776  1.97044  5.02839  2.66402  0.07221  0.48576  0.95510
     29   2.65659  4.29430  3.20553  3.10153  2.35946  3.66140  3.92013
2.75966  3.04882  1.74202  3.39336  3.46171  3.28385  3.33091  3.36070
2.81274  2.89073  2.55277  4.83770  3.49151      63 l - - S
          2.68618  4.42225  2.77519  2.73123  3.46354  2.40513  3.72494
3.29354  2.67741  2.69355  4.24690  2.90347  2.73739  3.18146  2.89801
2.37887  2.77519  2.98518  4.58477  3.61503
          0.02024  4.30604  5.02839  0.61958  0.77255  0.48576  0.95510
     30   2.60060  5.19381  2.25188  1.98792  4.52632  3.33511  3.64124
4.00976  2.19000  3.49323  4.24189  2.74551  3.83542  2.50222  2.82235
2.31983  2.83329  3.57848  5.63022  4.21597      64 e - - S
          2.68618  4.42225  2.77519  2.73123  3.46354  2.40513  3.72494
3.29354  2.67741  2.69355  4.24690  2.90347  2.73739  3.18146  2.89801
2.37887  2.77519  2.98518  4.58477  3.61503
          0.02024  4.30604  5.02839  0.61958  0.77255  0.48576  0.95510
     31   2.38412  4.91647  2.04757  2.24912  4.47575  3.41942  3.64364
3.95012  2.30663  3.44972  4.20586  2.60936  3.83664  2.57113  2.87878
2.29777  2.92061  3.53289  5.59965  4.19441      65 d - - S
```

```
         2.68618  4.42225  2.77519  2.73123  3.46354  2.40513  3.72494
3.29354  2.67741  2.69355  4.24690  2.90347  2.73739  3.18146  2.89801
2.37887  2.77519  2.98518  4.58477  3.61503
         0.02024  4.30604  5.02839  0.61958  0.77255  0.48576  0.95510
     32  2.67525  5.13200  2.73553  1.96464  4.45285  3.42569  3.63533
3.92335  2.25147  3.42572  4.18231  2.90092  2.55020  2.45291  2.83235
2.45022  2.79690  3.51021  5.57688  4.02407     66 e - - T
         2.68618  4.42225  2.77519  2.73123  3.46354  2.40513  3.72494
3.29354  2.67741  2.69355  4.24690  2.90347  2.73739  3.18146  2.89801
2.37887  2.77519  2.98518  4.58477  3.61503
         0.02024  4.30604  5.02839  0.61958  0.77255  0.48576  0.95510
     33  1.94206  4.74479  2.85079  2.28798  4.27608  3.44249  3.64054
3.72048  2.38805  3.27115  4.05077  2.74482  3.50990  2.68730  2.65944
2.43828  2.71332  3.31007  5.46410  3.98921     67 a - - H
         2.68618  4.42225  2.77519  2.73123  3.46354  2.40513  3.72494
3.29354  2.67741  2.69355  4.24690  2.90347  2.73739  3.18146  2.89801
2.37887  2.77519  2.98518  4.58477  3.61503
         0.02024  4.30604  5.02839  0.61958  0.77255  0.48576  0.95510
     34  3.54627  4.87810  4.88347  4.45913  0.85798  4.54234  4.18883
2.72178  4.27887  1.94674  3.03630  4.45341  4.83539  4.35496  4.35513
3.92309  3.79085  2.79854  4.37688  2.76253     68 f - - H
         2.68618  4.42225  2.77519  2.73123  3.46354  2.40513  3.72494
3.29354  2.67741  2.69355  4.24690  2.90347  2.73739  3.18146  2.89801
2.37887  2.77519  2.98518  4.58477  3.61503
         0.02024  4.30604  5.02839  0.61958  0.77255  0.48576  0.95510
     35  2.19363  4.06206  3.81303  3.23636  3.32611  3.68793  3.96864
2.04789  3.02786  2.13166  3.26442  3.55073  4.06142  3.02557  3.20406
2.95609  2.87017  2.14763  4.81527  3.60775     69 i - - H
         2.68618  4.42225  2.77519  2.73123  3.46354  2.40513  3.72494
3.29354  2.67741  2.69355  4.24690  2.90347  2.73739  3.18146  2.89801
2.37887  2.77519  2.98518  4.58477  3.61503
         0.02024  4.30604  5.02839  0.61958  0.77255  0.48576  0.95510
     36  2.01687  5.04318  2.94681  2.40647  4.34203  3.41027  3.46988
3.78396  2.34806  3.27465  4.10739  2.15296  3.86444  2.55251  2.57794
2.65176  2.92500  3.41126  5.50295  4.14087     70 a - - H
         2.68618  4.42225  2.77519  2.73123  3.46354  2.40513  3.72494
3.29354  2.67741  2.69355  4.24690  2.90347  2.73739  3.18146  2.89801
2.37887  2.77519  2.98518  4.58477  3.61503
         0.02024  4.30604  5.02839  0.61958  0.77255  0.48576  0.95510
     37  4.04149  5.20527  4.90866  4.67466  1.82481  4.67855  3.63462
3.70915  4.50395  2.98044  4.29495  4.32370  4.98809  4.41890  4.49335
4.08031  4.26550  3.64595  3.75102  0.61714     71 Y - - H
         2.68618  4.42225  2.77519  2.73123  3.46354  2.40513  3.72494
3.29354  2.67741  2.69355  4.24690  2.90347  2.73739  3.18146  2.89801
2.37887  2.77519  2.98518  4.58477  3.61503
         0.02024  4.30604  5.02839  0.61958  0.77255  0.48576  0.95510
     38  3.59999  4.92447  5.08824  4.60719  3.20237  4.77243  5.10588
2.24162  4.36209  0.59676  3.04431  4.85070  4.98233  4.53490  4.46228
4.20409  3.84383  2.48273  5.33543  4.18725     72 L - - H
         2.68618  4.42225  2.77519  2.73123  3.46354  2.40513  3.72494
3.29354  2.67741  2.69355  4.24690  2.90347  2.73739  3.18146  2.89801
2.37887  2.77519  2.98518  4.58477  3.61503
         0.02024  4.30604  5.02839  0.61958  0.77255  0.48576  0.95510
     39  2.20598  5.13992  2.74048  2.17944  4.47080  3.43273  3.59399
3.94218  1.89960  3.43496  4.18751  2.77806  3.83384  2.53440  2.58863
2.59294  2.86303  3.52358  5.57641  4.18039     73 k - - H
```

2.68618 4.42225 2.77519 2.73123 3.46354 2.40513 3.72494
3.29354 2.67741 2.69355 4.24690 2.90347 2.73739 3.18146 2.89801
2.37887 2.77519 2.98518 4.58477 3.61503
0.02024 4.30604 5.02839 0.61958 0.77255 0.48576 0.95510
40 3.85356 5.20101 4.36995 4.14975 2.21972 4.41250 3.42826
3.86581 3.96076 3.17124 4.42139 4.11457 4.81481 4.16786 4.07961
3.88602 4.10643 3.74084 3.88357 0.58063 74 Y - - G
2.68618 4.42225 2.77519 2.73123 3.46354 2.40513 3.72494
3.29354 2.67741 2.69355 4.24690 2.90347 2.73739 3.18146 2.89801
2.37887 2.77519 2.98518 4.58477 3.61503
0.02024 4.30604 5.02839 0.61958 0.77255 0.48576 0.95510
41 3.59608 4.94523 5.01207 4.55344 3.19855 4.70373 5.05387
2.39962 4.27352 0.57457 2.98513 4.79362 4.94977 4.49076 4.37946
4.15614 3.84888 2.56456 5.31281 4.14329 75 L - - G
2.68621 4.42228 2.77523 2.73126 3.46334 2.40516 3.72498
[truncated: 441,631 more chars]
