# Supplementary material for: Enhanced sensitivity of TAPscan v4 enables comprehensive analysis of streptophyte transcription factor evolution
Source: Plant J. 2024 Dec 12;121(1):e17184. doi: 10.1111/tpj.17184 (PMC11712027; doi:10.1111/tpj.17184)
Supplement: Supplementary file 11 — File S5. Phylogenies of the LBD and HDZ families to support the definition of subfamilies. [file TPJ-121-0-s006.pdf]

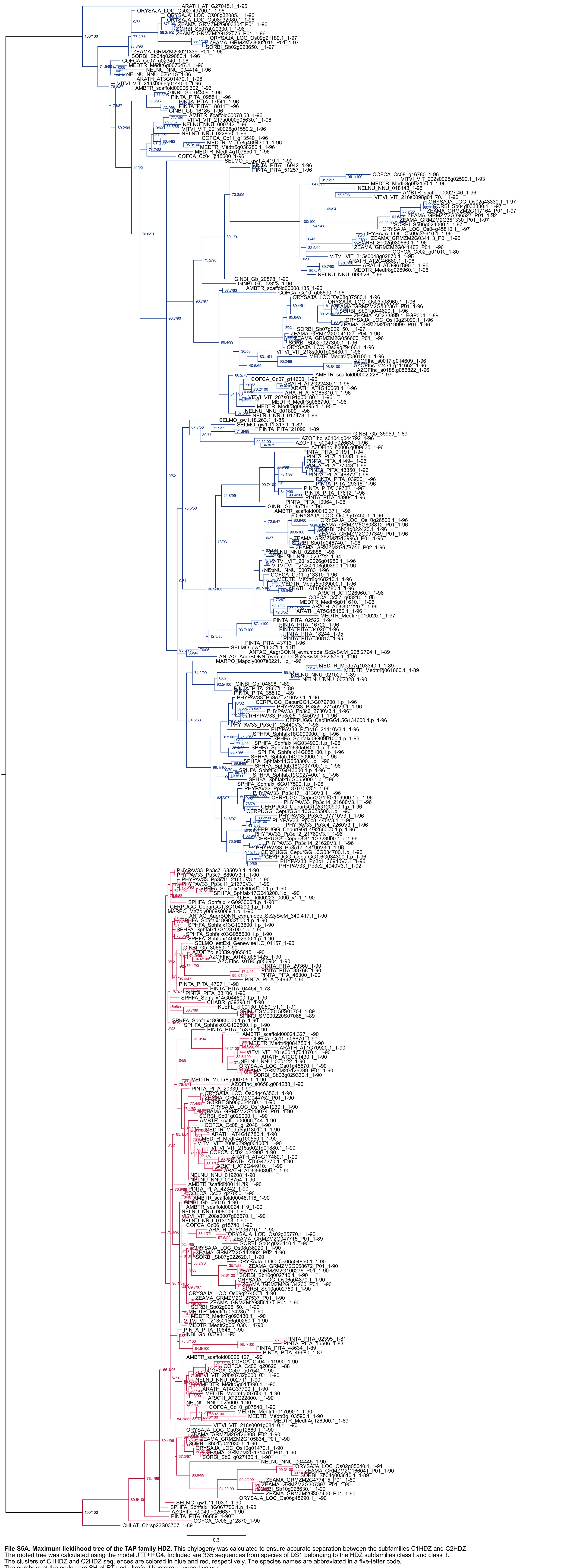

File S5A. Maximum lieklihood tree of the TAP family HDZ. This phylogeny was calculated to ensure accurate separation between the subfamilies C1HDZ and C2HDZ. The rooted tree was calculated using the model JTT+I+G4. Included are 335 sequences from species of DS1 belonging to the HDZ subfamilies class I and class II. The clusters of C1HDZ and C2HDZ sequences are colored in blue and red, respectively. The species names are abbreviated in a five-letter code. The numbers at the nodes are SH-aLRT and ultrafast bootstrap support values.

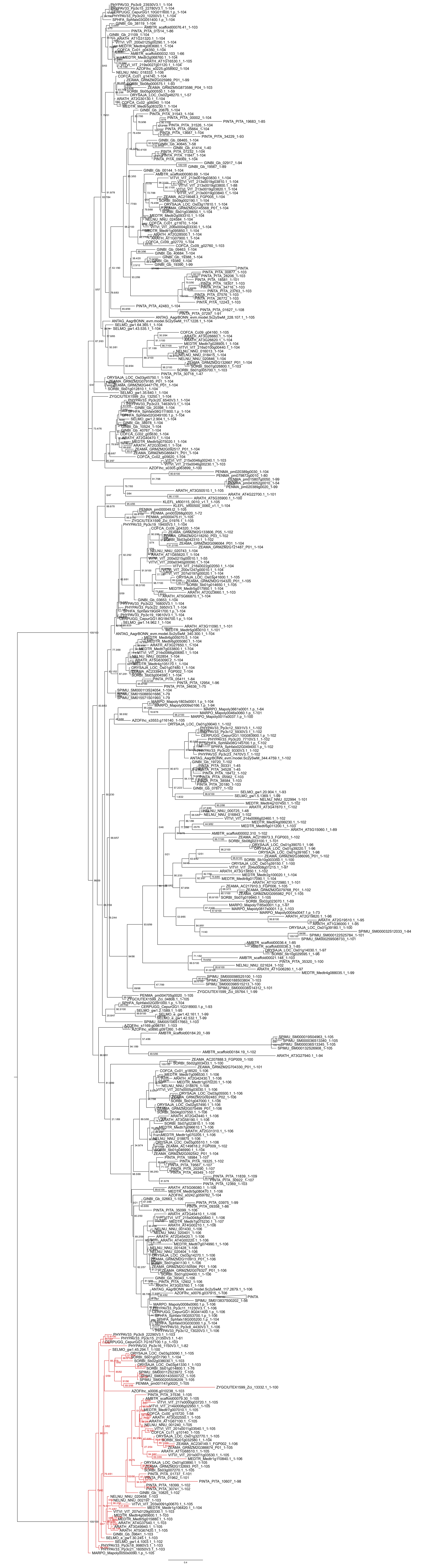

File S5B. Maximum lieklihood tree of the TAP subfamilies LOB1 and LOB2. This phylogeny was calculated to ensure accurate separation between the subfamilies LOB1 and LOB2. The rooted tree was calculated using the model JTT+R5. Included are 465 sequences from species of DS1 belonging to the LBD subfamilies LOB1 and LOB2. The cluster of LOB2 is colored in red. The species names are abbreviated in a five-letter code. Clusters in which only sequences belonging to *Pinus taeda* are present have been grouped for clarity. The numbers at the nodes are SH-aLRT and ultrafast bootstrap support values.
